# Supplementary material for: Long-read detection of transposable element mobilization in the soma of hypomethylated Arabidopsis thaliana individuals
Source: Genome Biol. 2025 Jul 30;26:231. doi: 10.1186/s13059-025-03691-7 (PMC12312487; doi:10.1186/s13059-025-03691-7)
Supplement: Supplementary file 2 — Additional file 2. Visual inspection of somatic insertion and excision events, available at https://github.com/aerilli/Somatic-transposition_met1/tree/551df407370c6528225f404ba62a073dced14b08/Supplementary-Files/Visual_inspection. [file 13059_2025_3691_MOESM2_ESM.gz › Split_Supplementary-File4/File3_CIGAR_Excisions.pdf]

CIGAR_Excisions

Only deletions that match 99% with a TE

Merged elements

```
"SatCEN" = satellite or centromeric rearrangements - not to be trusted
```

met1_01

> Chr2 6734994 6775798 ATMSAT1 met1_01

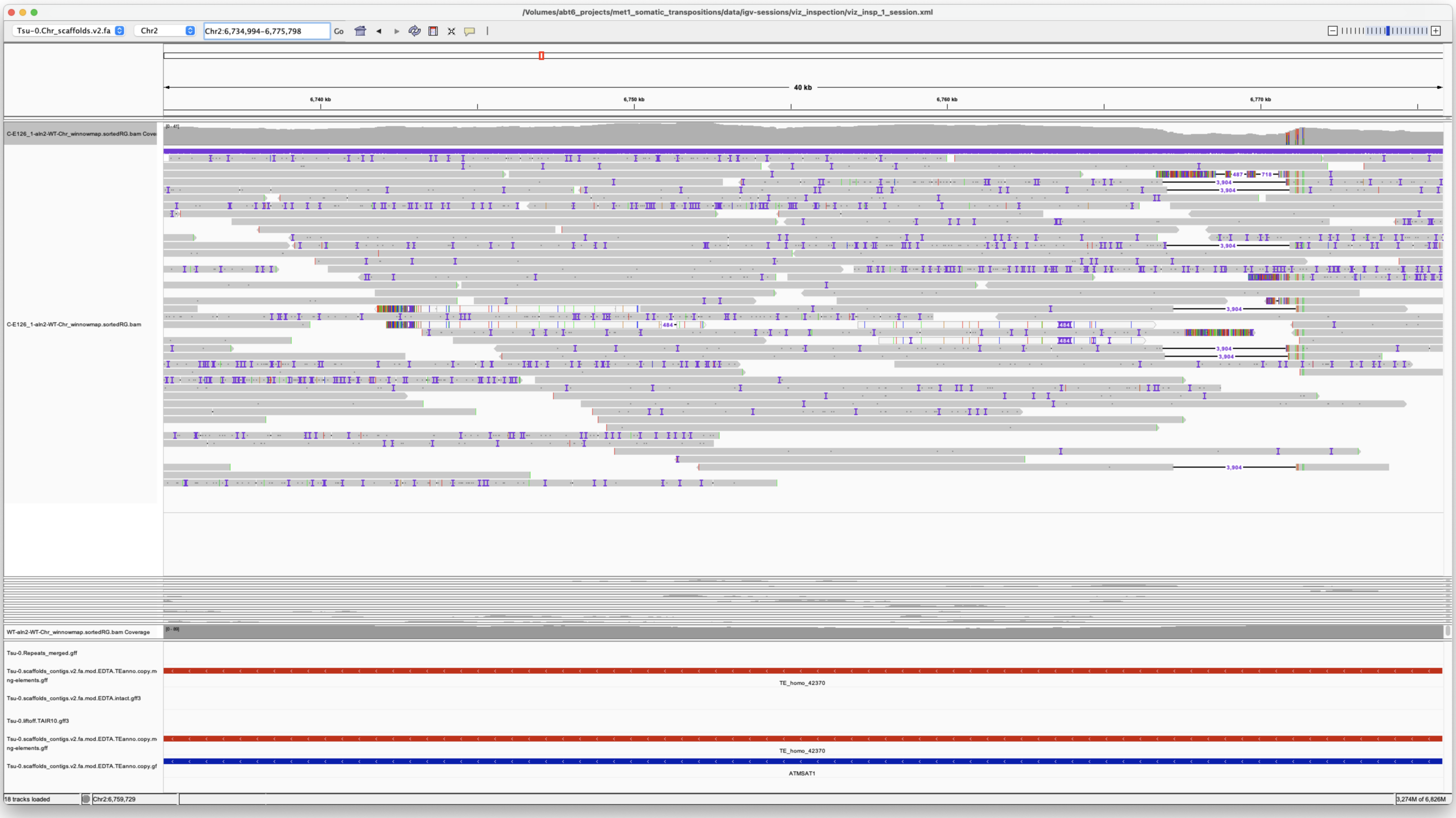

SatCEN

> Chr2 8591002 8595324 ATENSPM1 met1_01

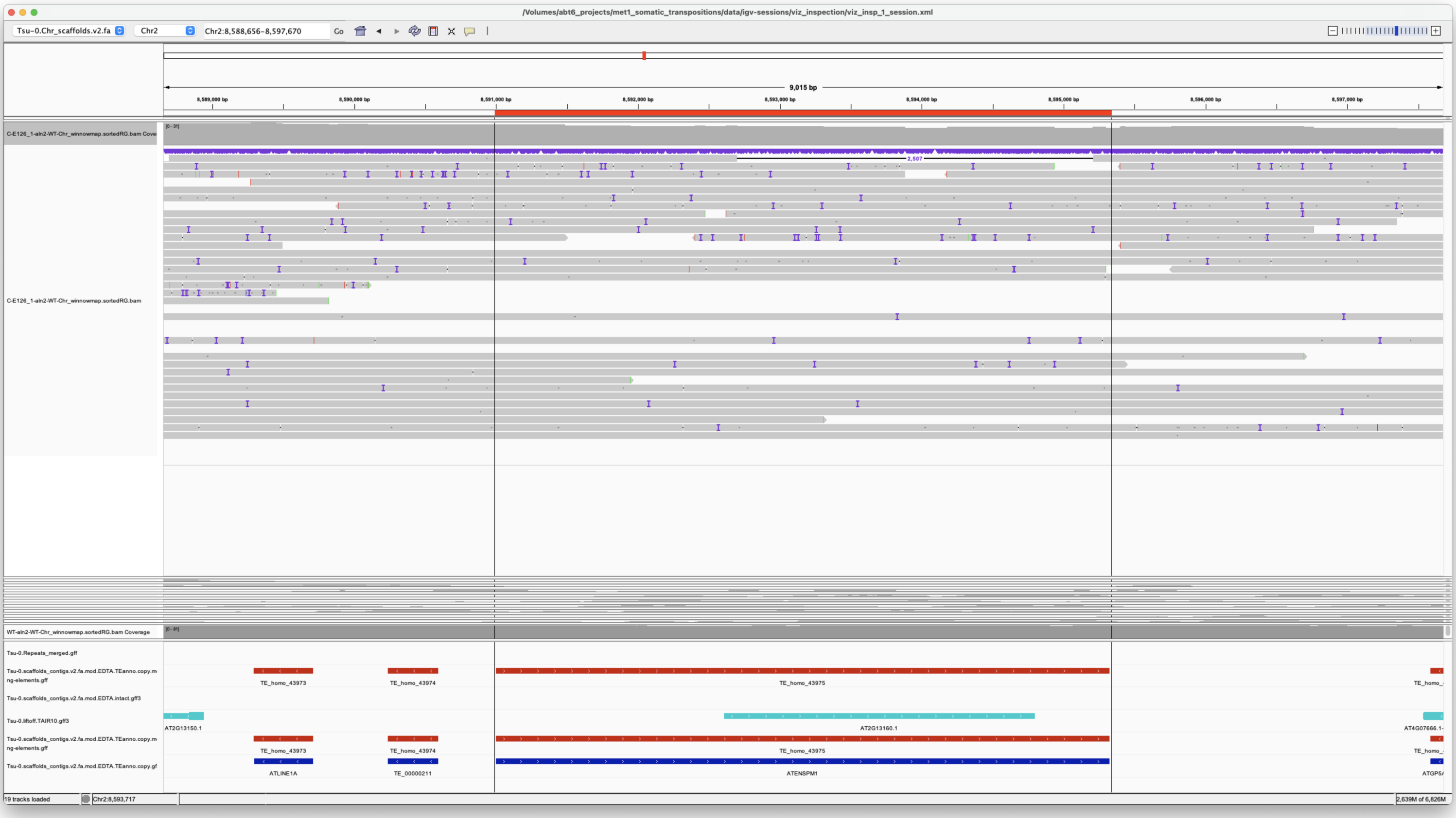

internal deletion not associated to insertions

```
There is also the possibility this is misannotation
```

Rearrangement

> Chr5 19152829 19160826 VANDAL21 met1_01

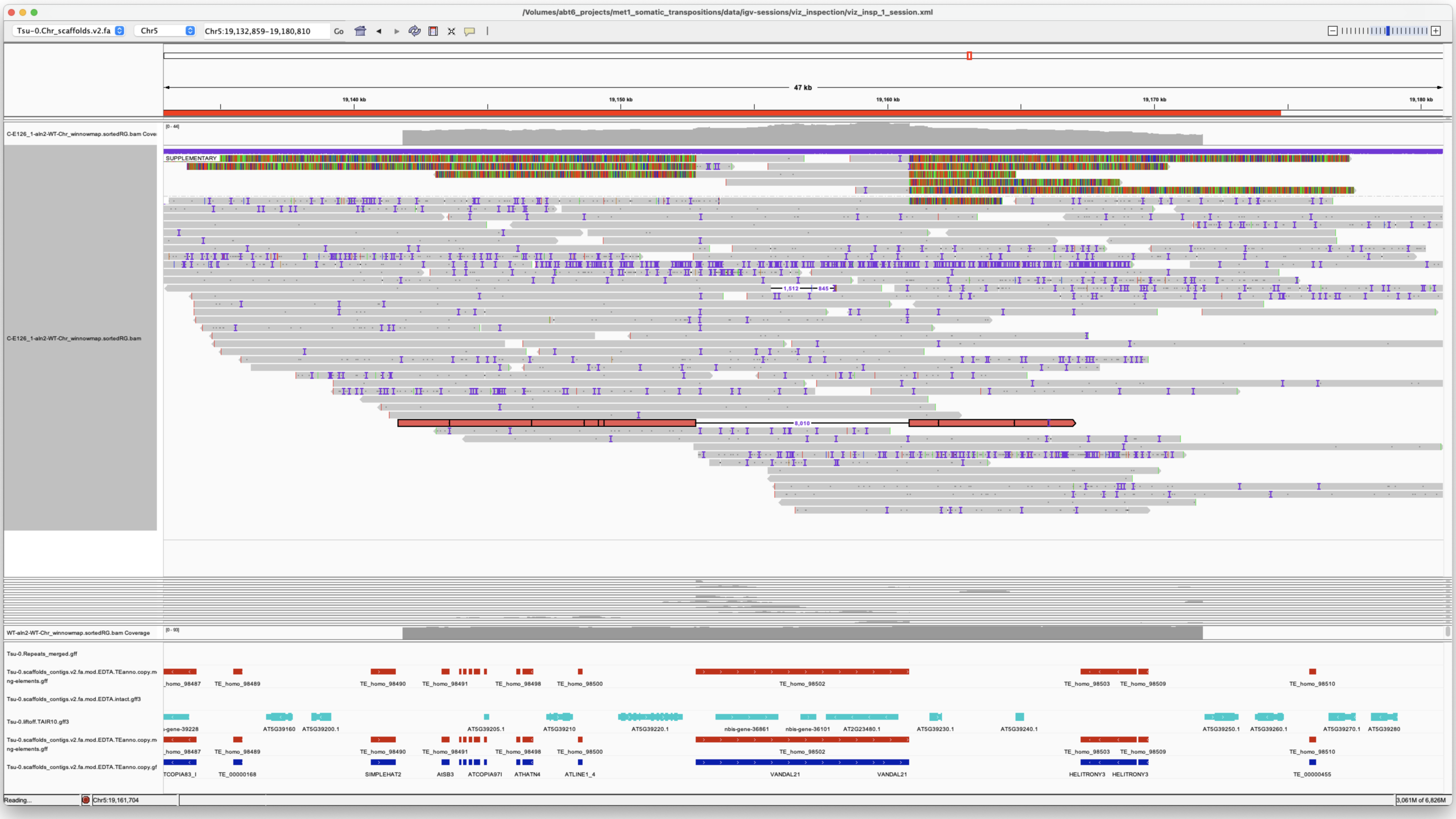

Associated with insertion

Confirmed

---

met1_02

---

met1_03

> Chr5 875414 876434 PAC met1_03

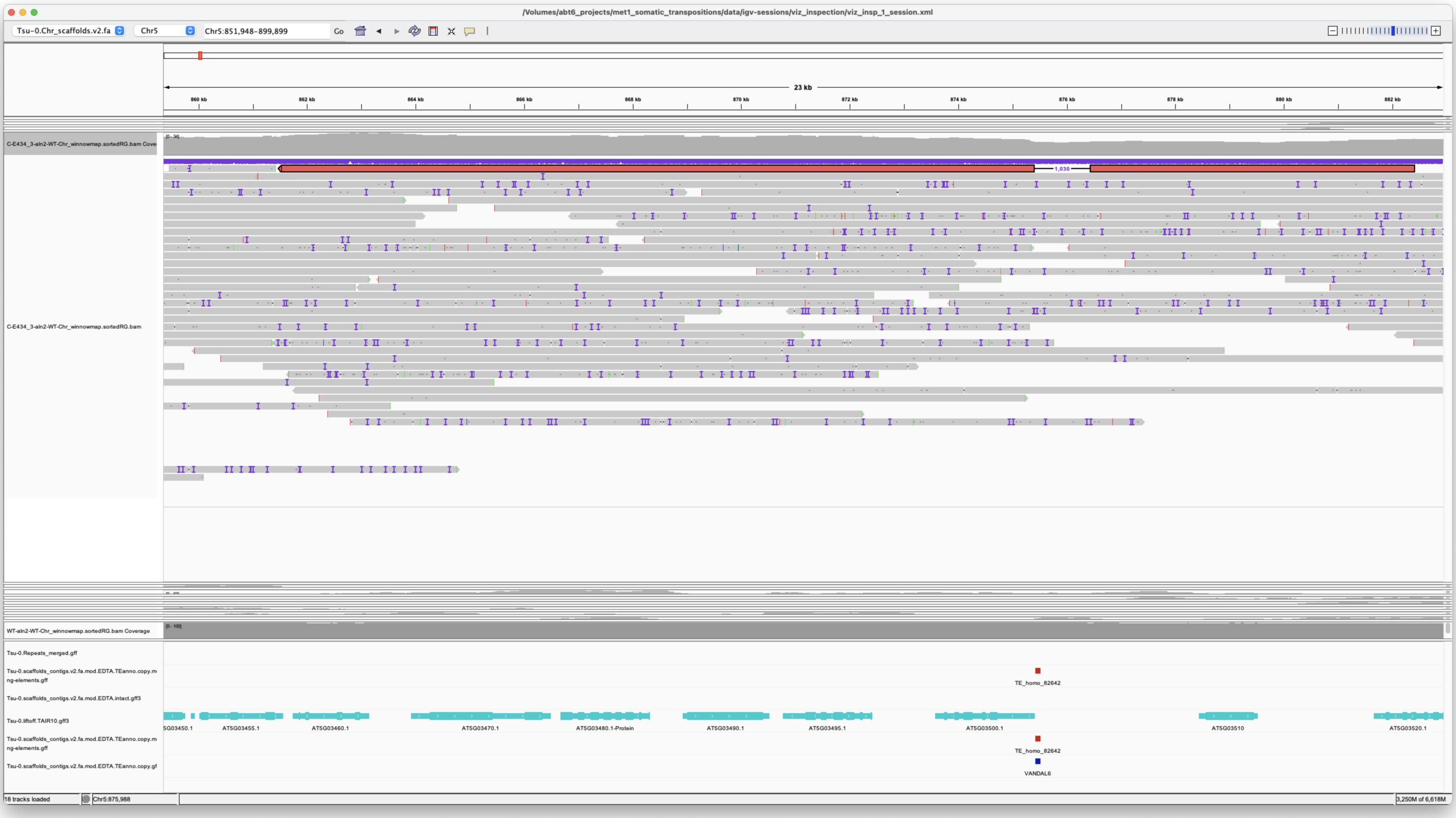

Annotation of putative Pack-Type missing from bottom tracks

Confirmed

---

met1_04

Chr2 6734994 6775798 ATMSAT1 met1_04

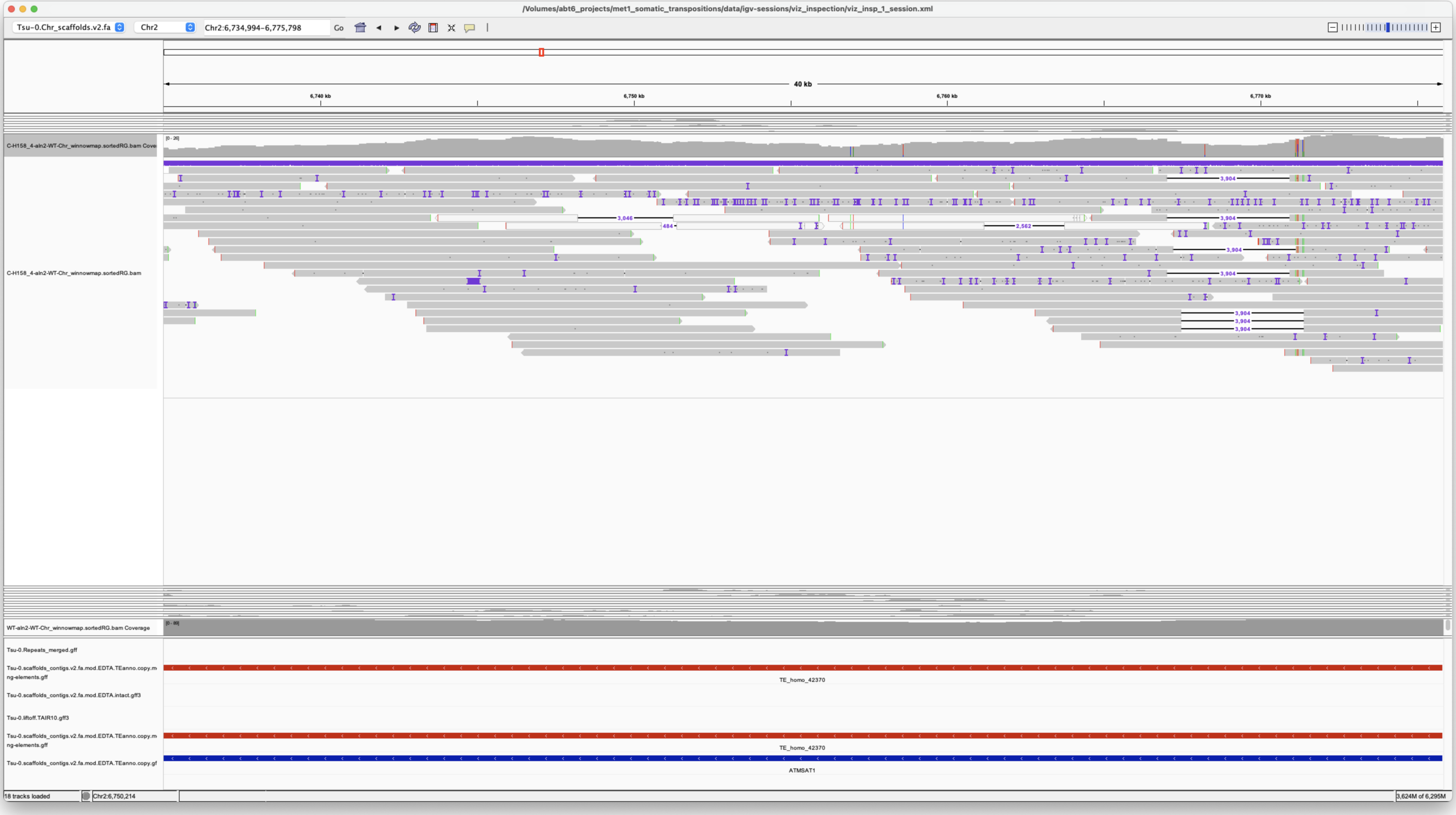

SatCEN

Chr5 19152829 19160826 VANDAL21 met1_04

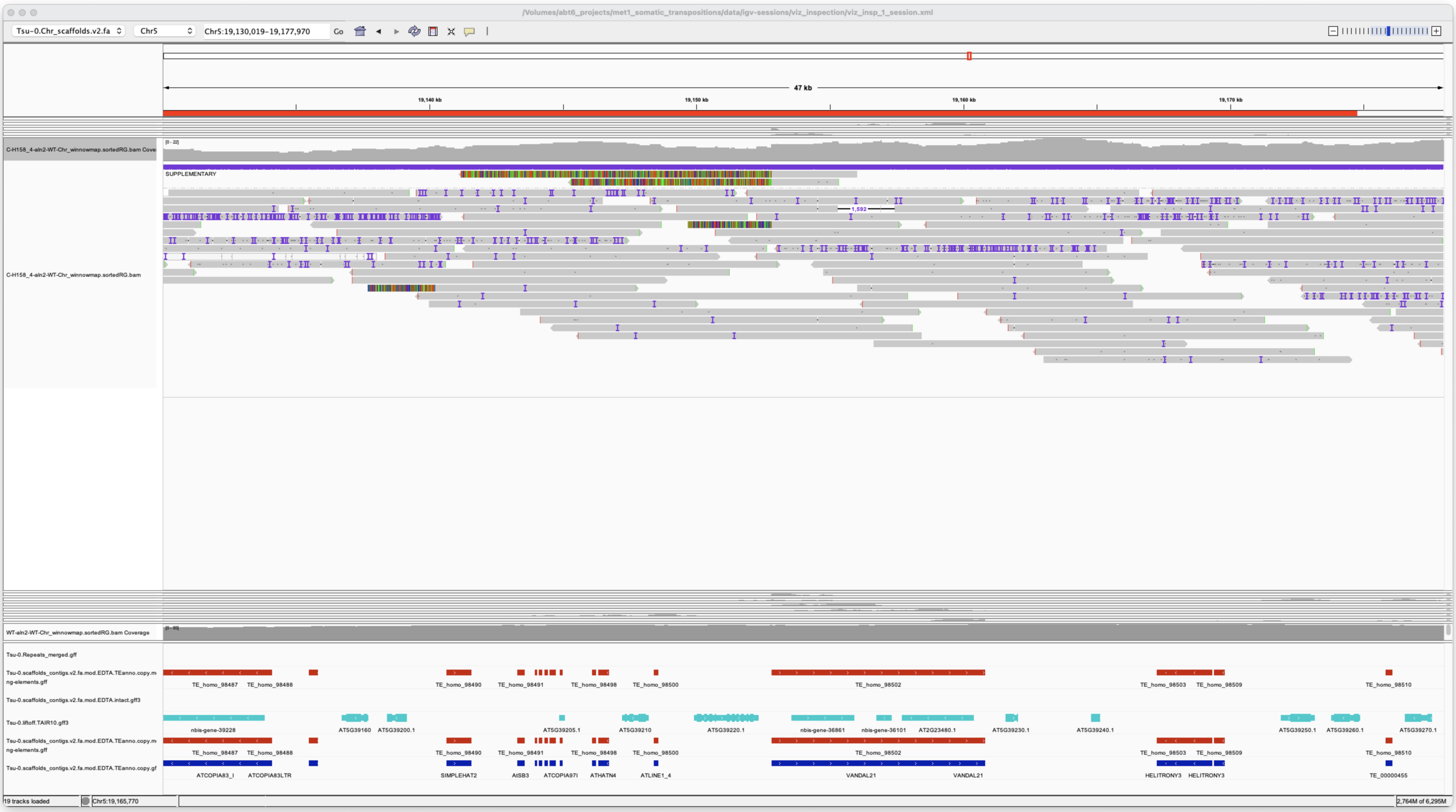

Internal deletion

met1_05

Chr2 6734994 6775798 ATMSAT1 met1_05

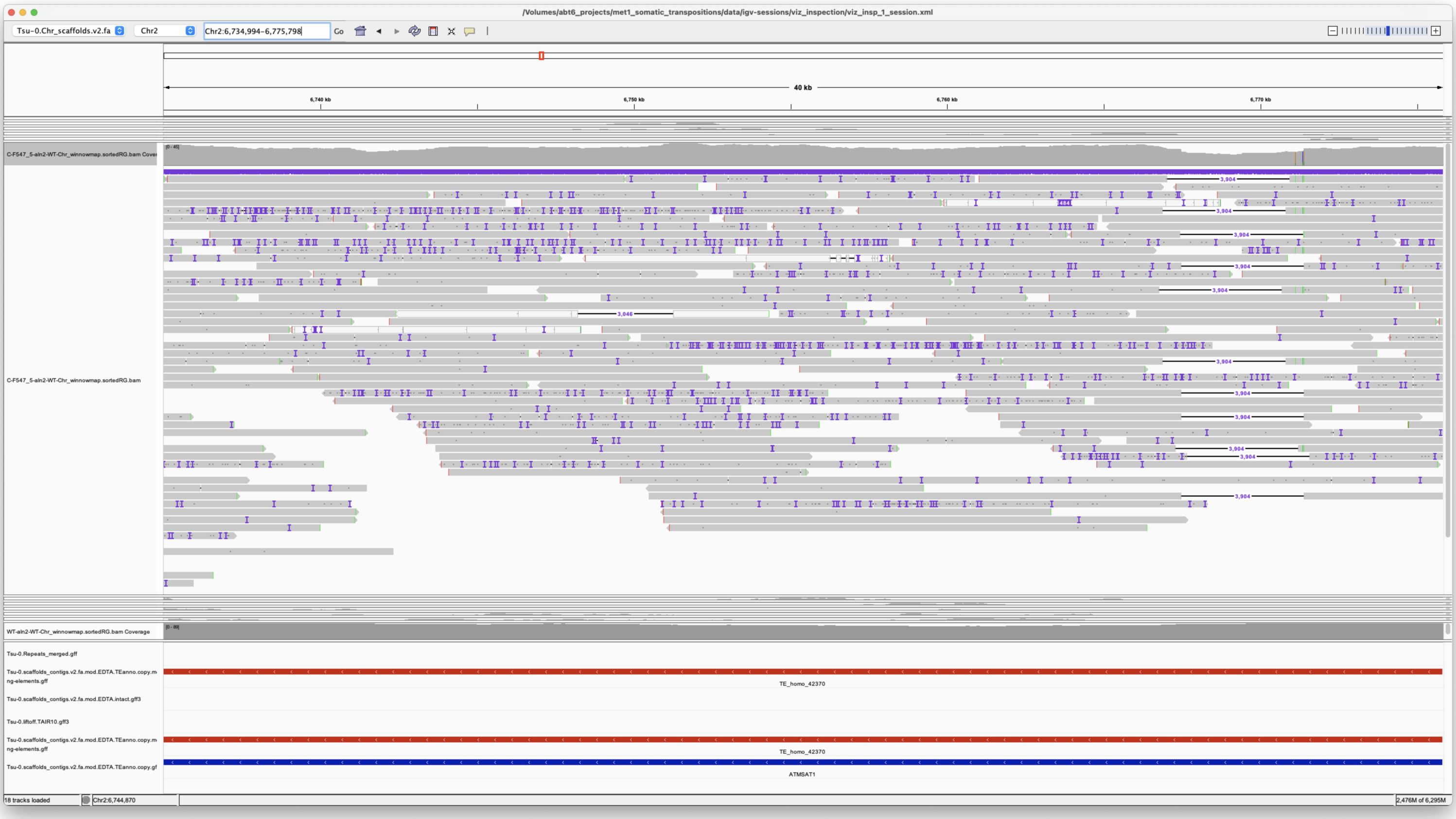

SatCEN

| Chr5 19152829 19160826 VANDAL21 met1_05

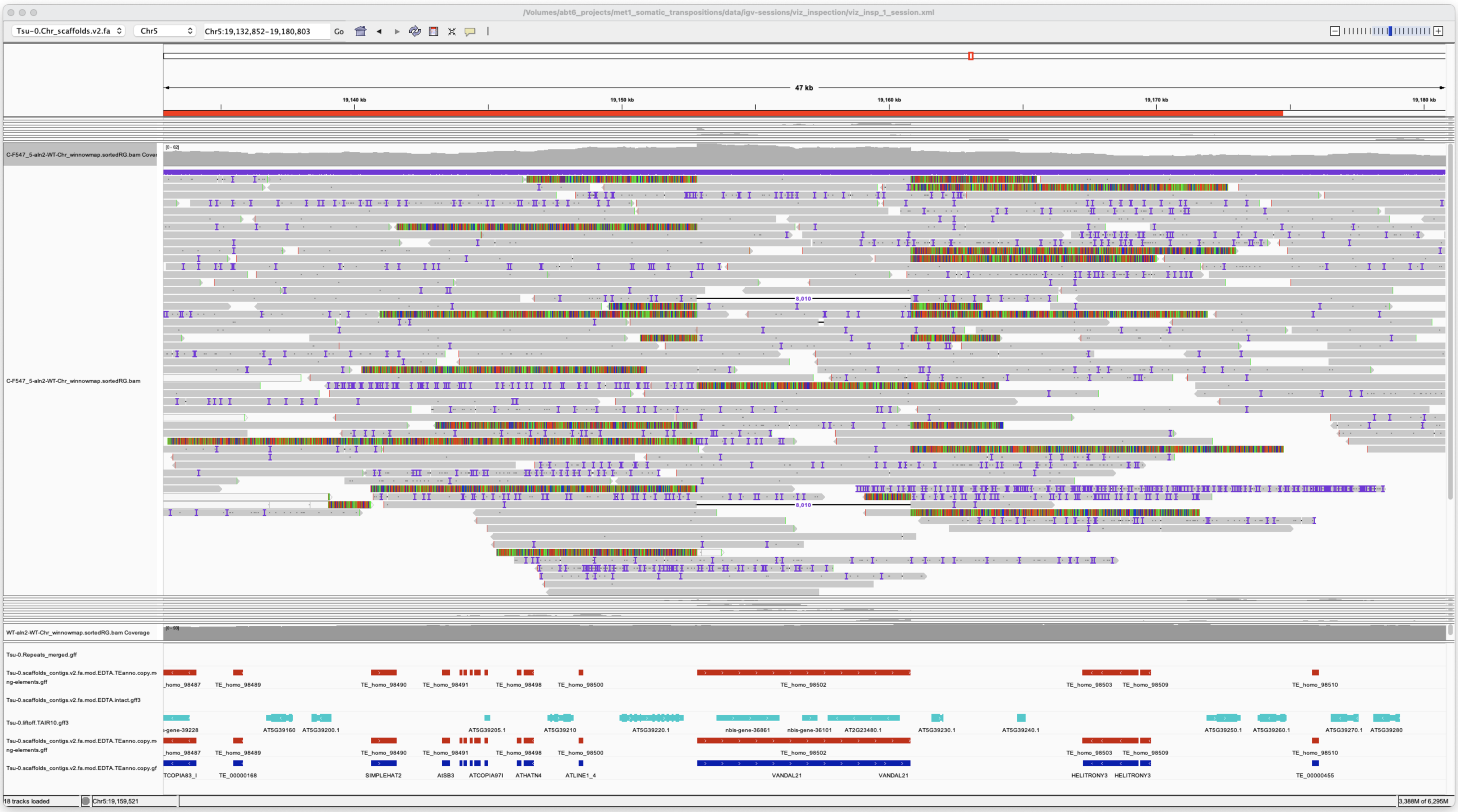

2x

Confirmed

---

met1_06

| Chr2 6734994 6775798 ATMSAT1 met1_06

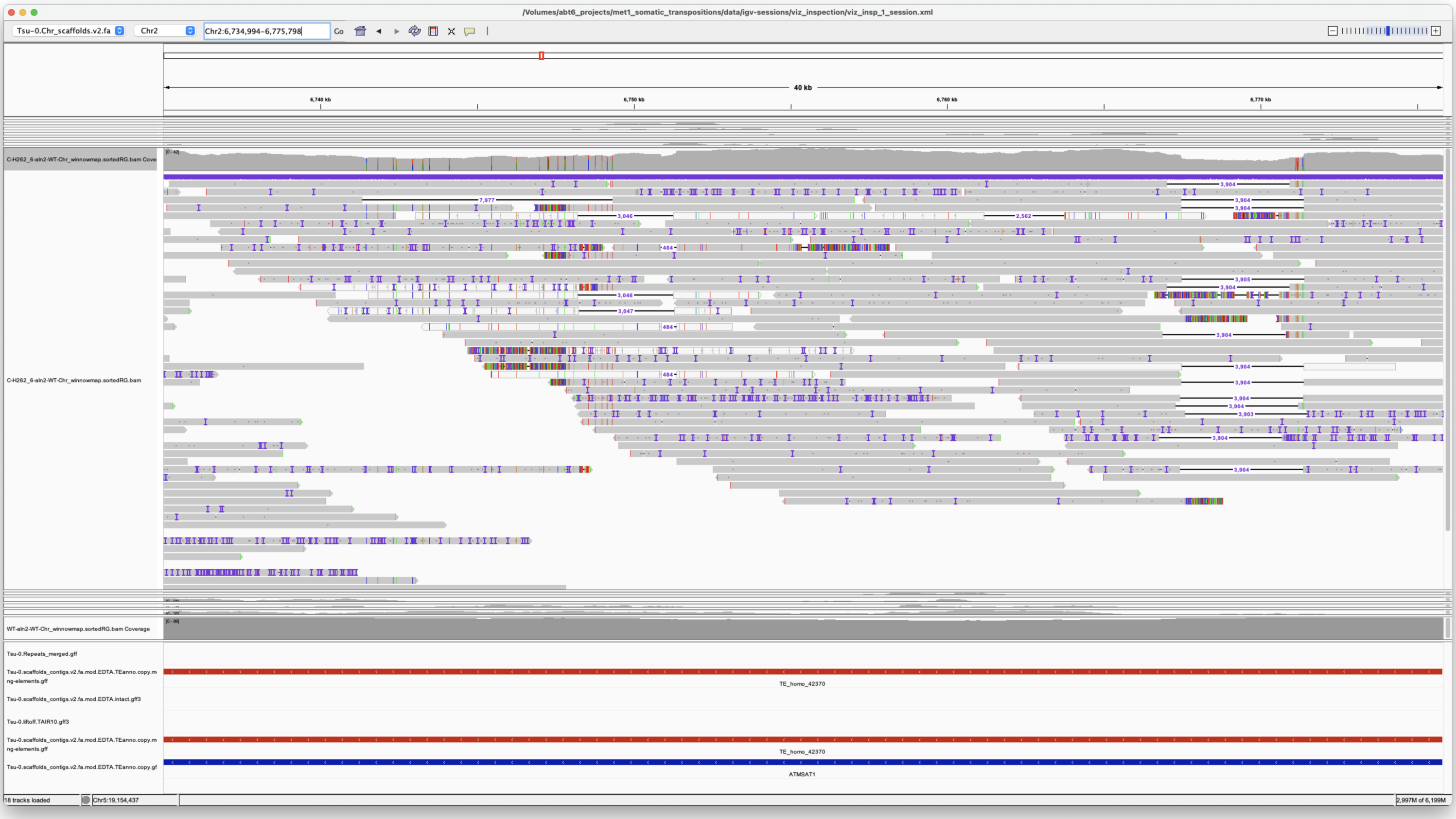

SatCEN

Chr3 16344522 16352497 VANDAL6 met1_06

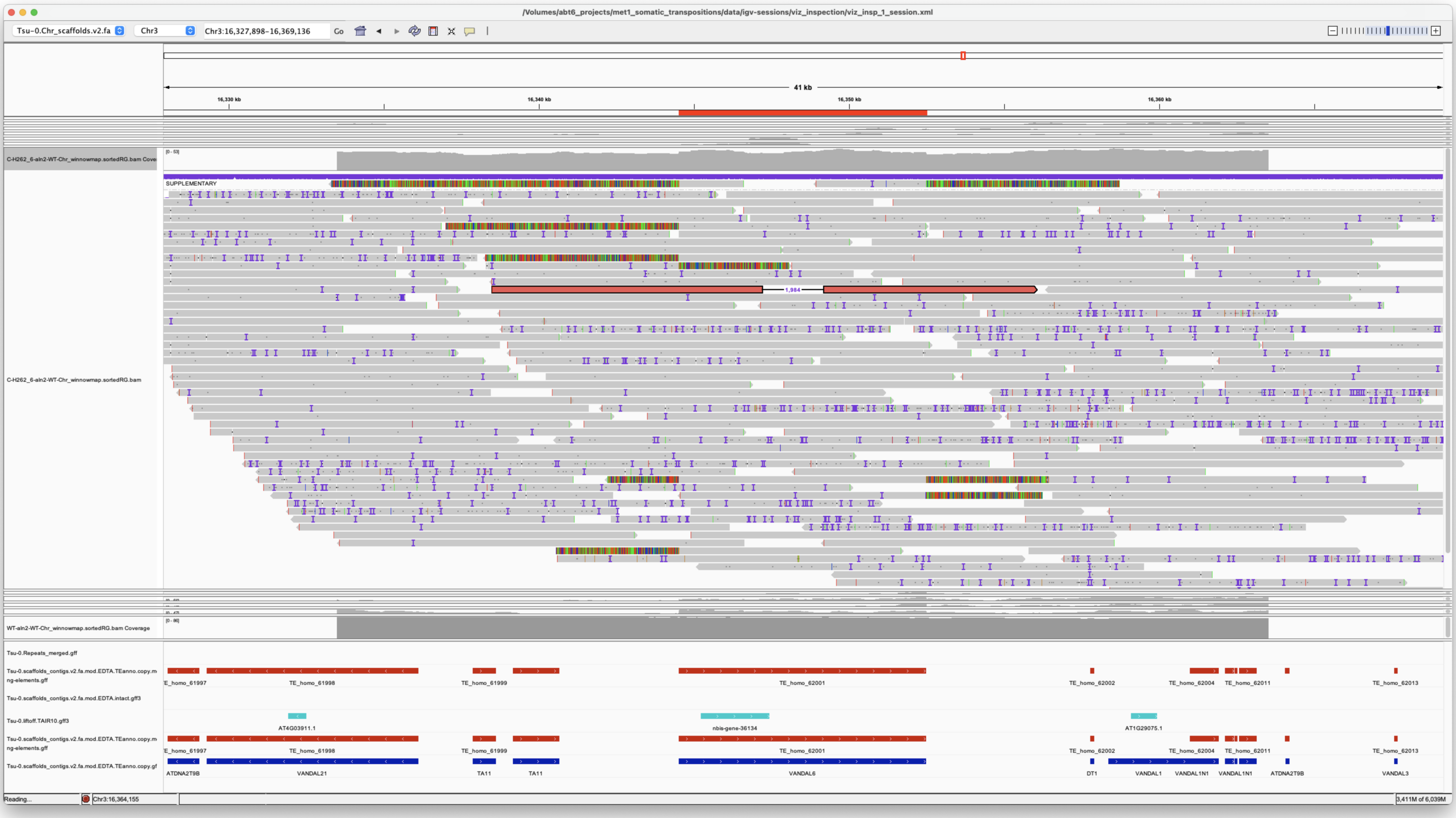

internal deletion

Rearrangement

Chr5 19152829 19160826 VANDAL21 met1_06

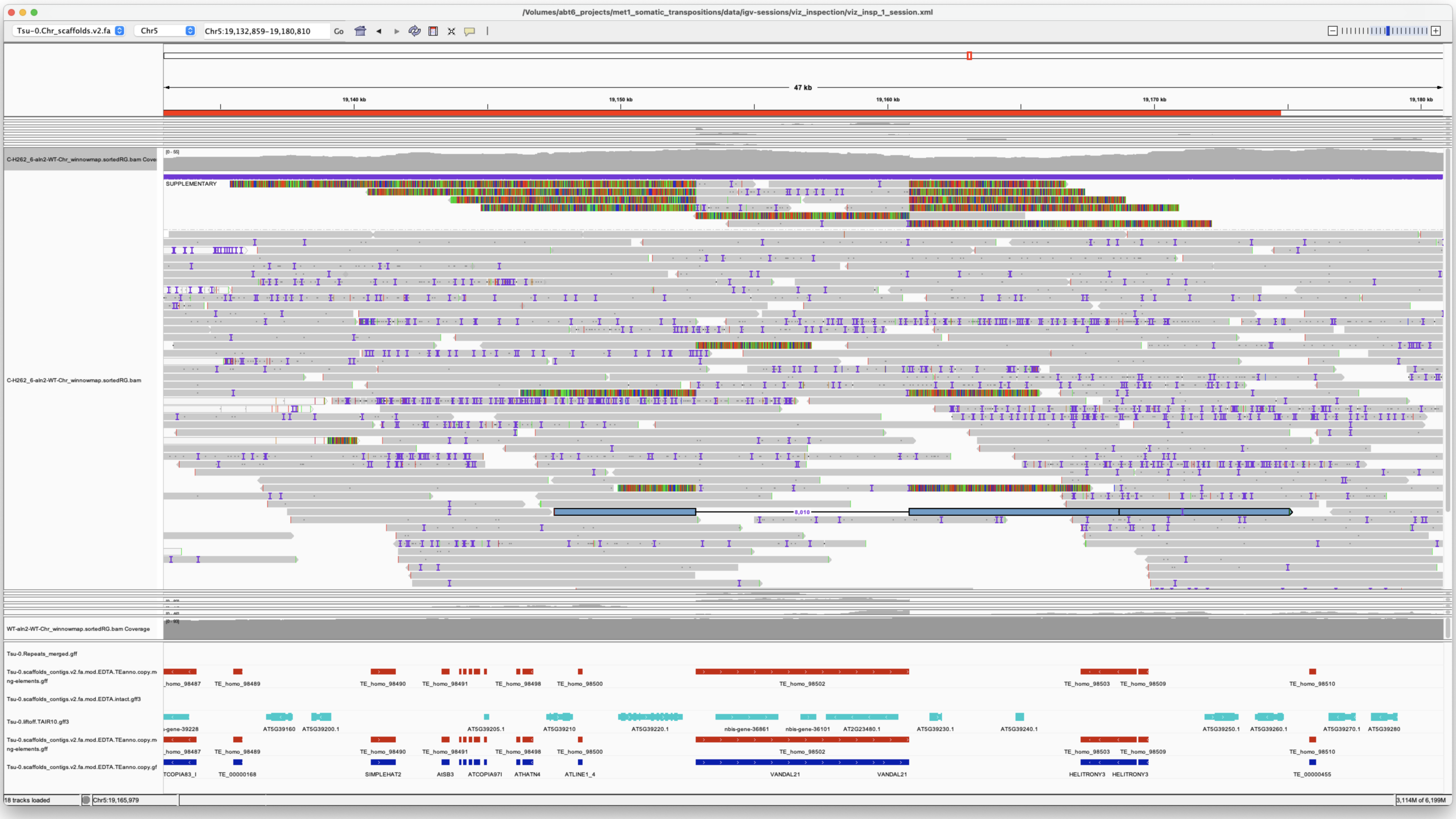

Confirmed

---

met1_07

> Chr5 19152829 19160826 VANDAL21 met1_07

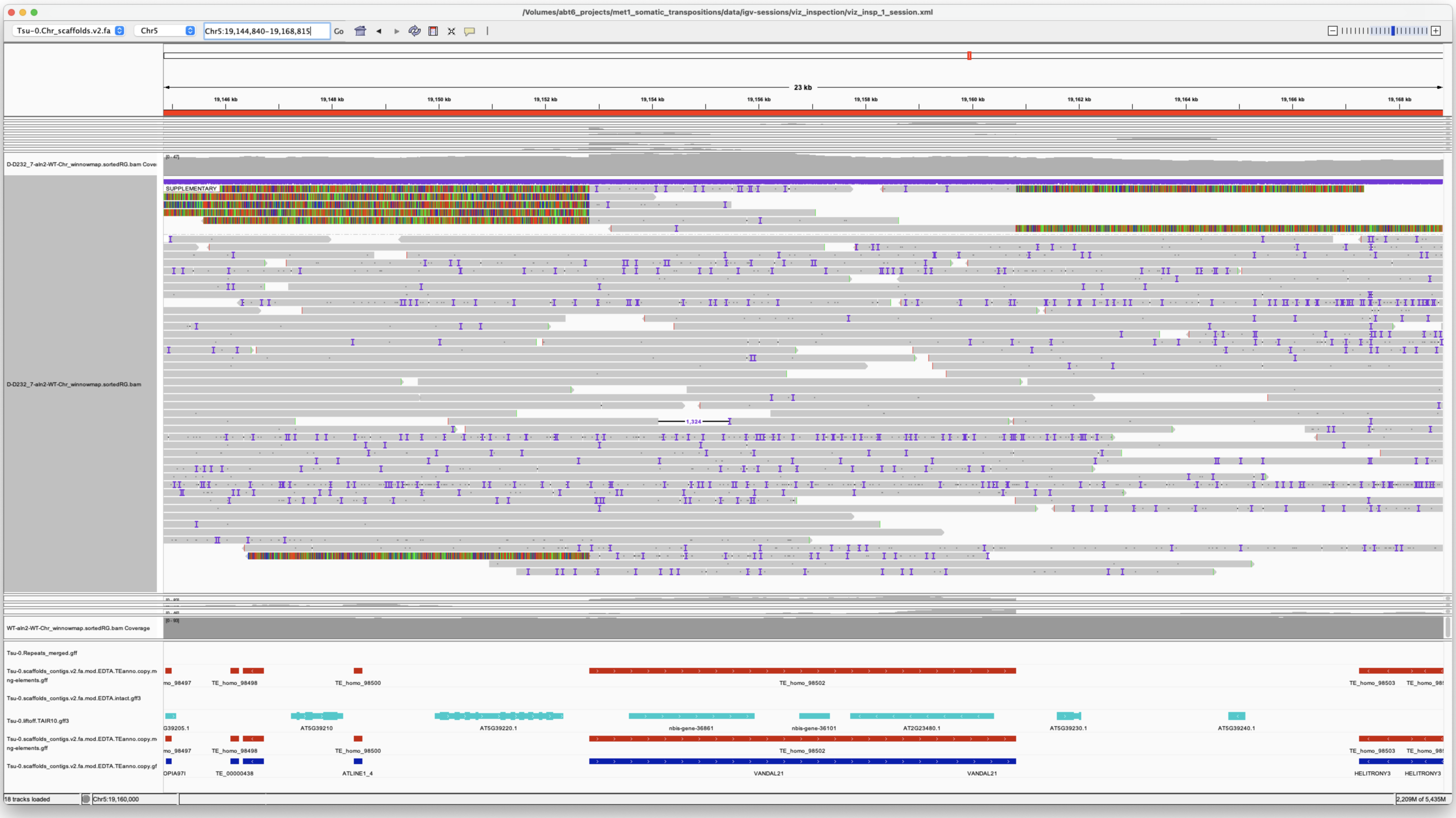

Internal deletion

Rearrangement

> Chr5 19872565 19877095 VANDAL21 met1_07

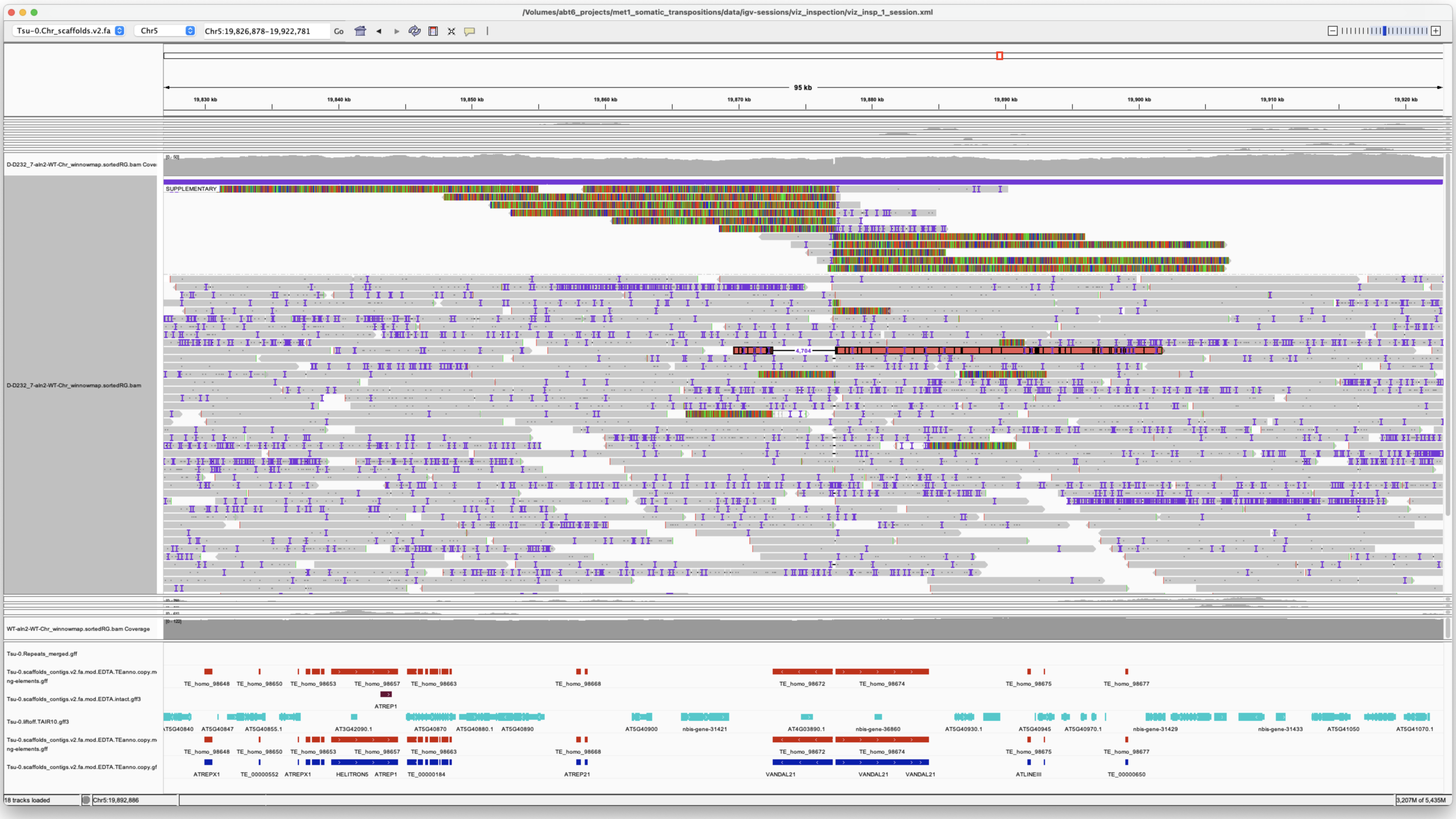

Hypermutable region, probable alternative transposition involved

Excluded

---

met1_08

> Chr2 6734994 6775798 ATMSAT1 met1_08

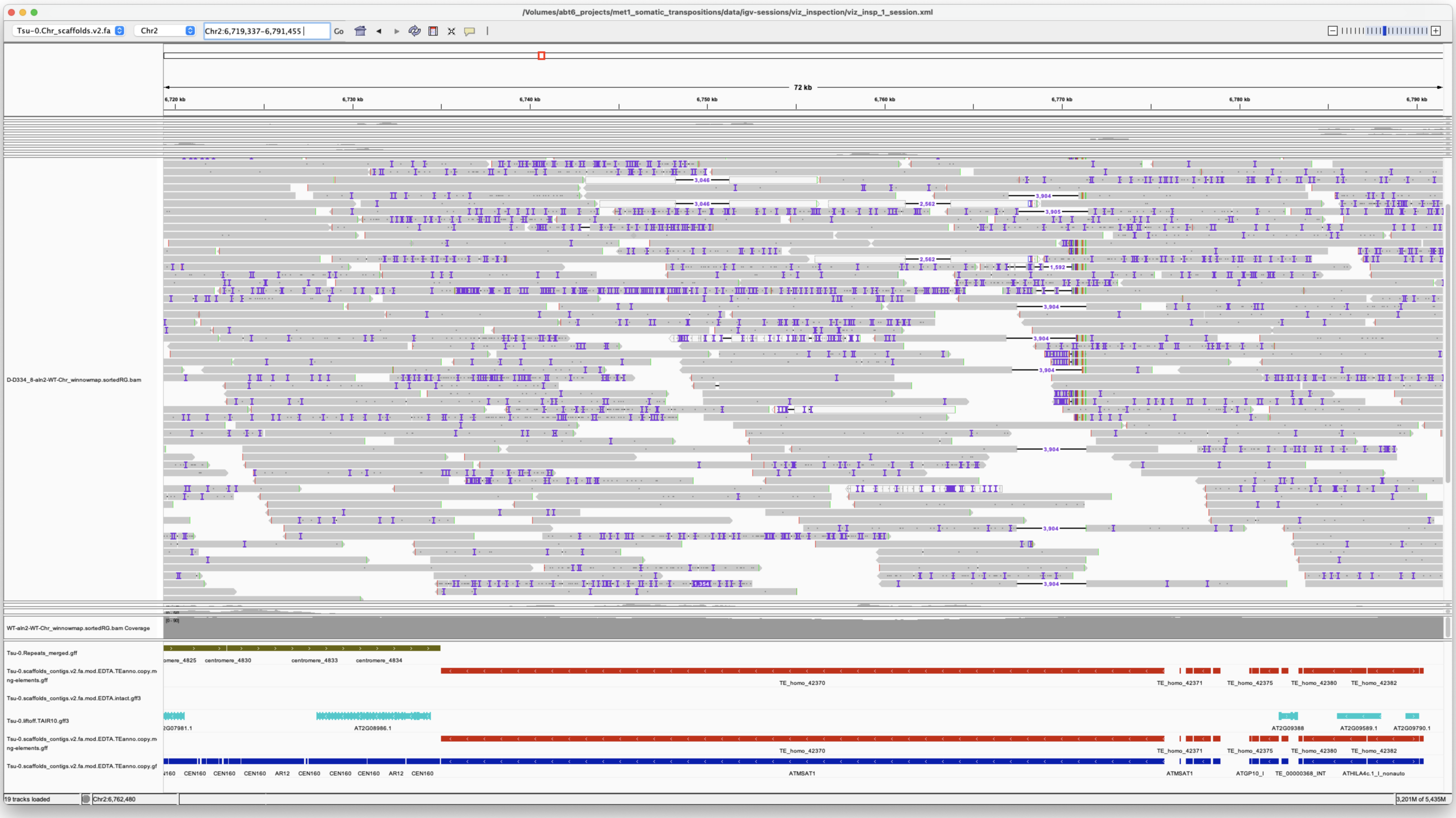

SatCEN

> Chr5 19152829 19160826 VANDAL21 met1_08

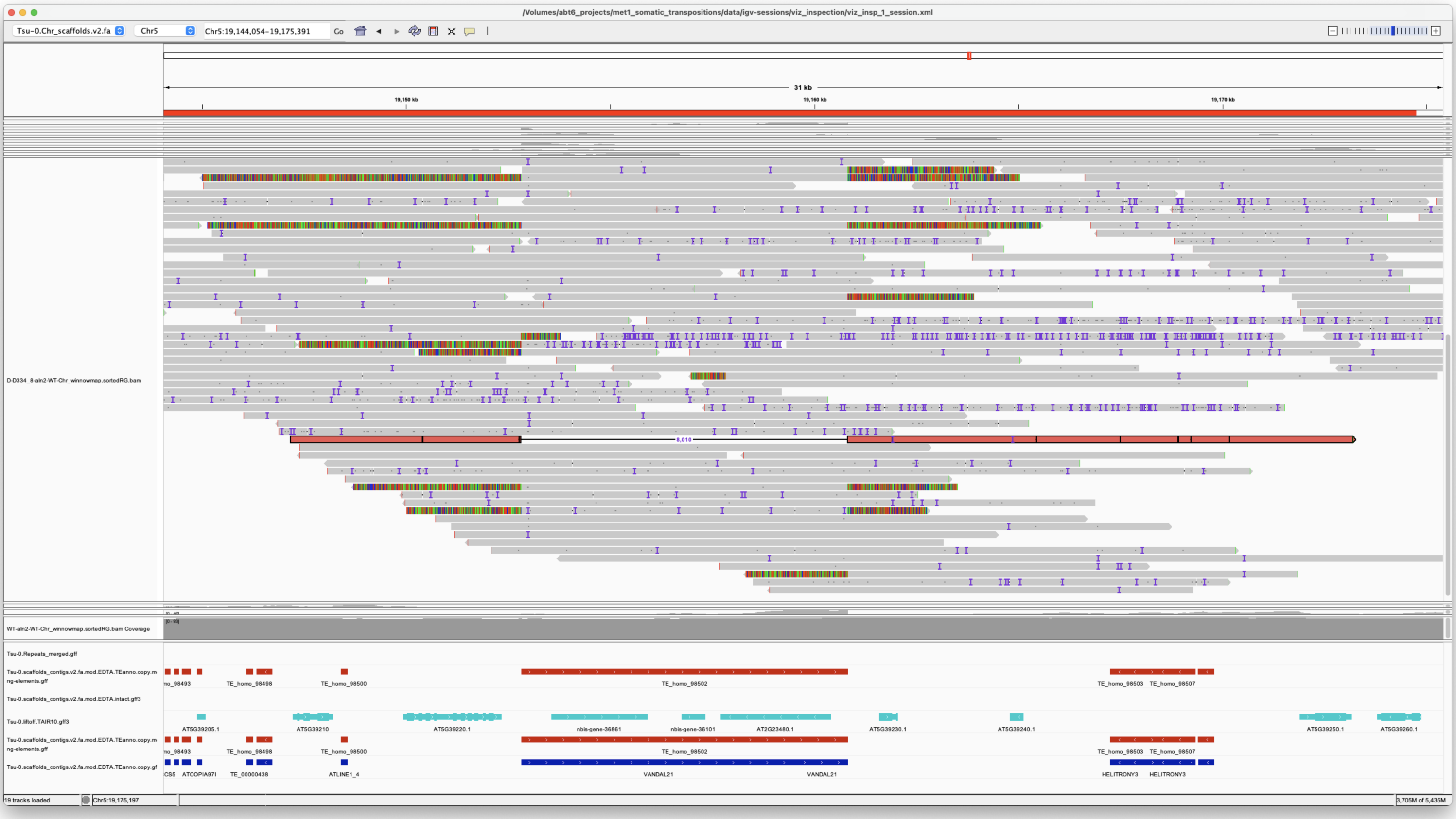

Confirmed

met1_09

> Chr2 6734994 6775798 ATMSAT1 met1_09

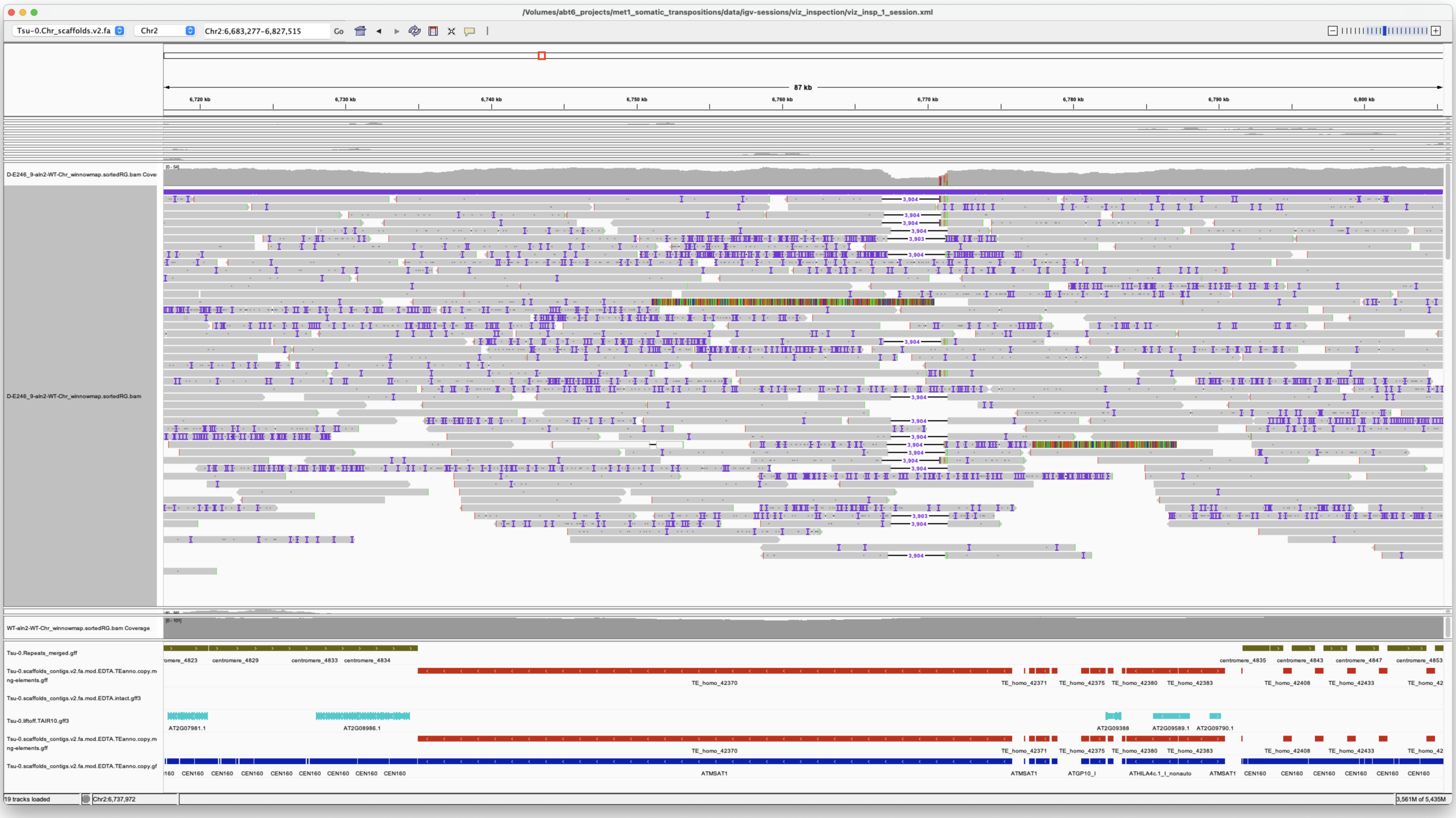

SatCEN

> Chr3 19893498 19897077 ATLINE1_12 met1_09

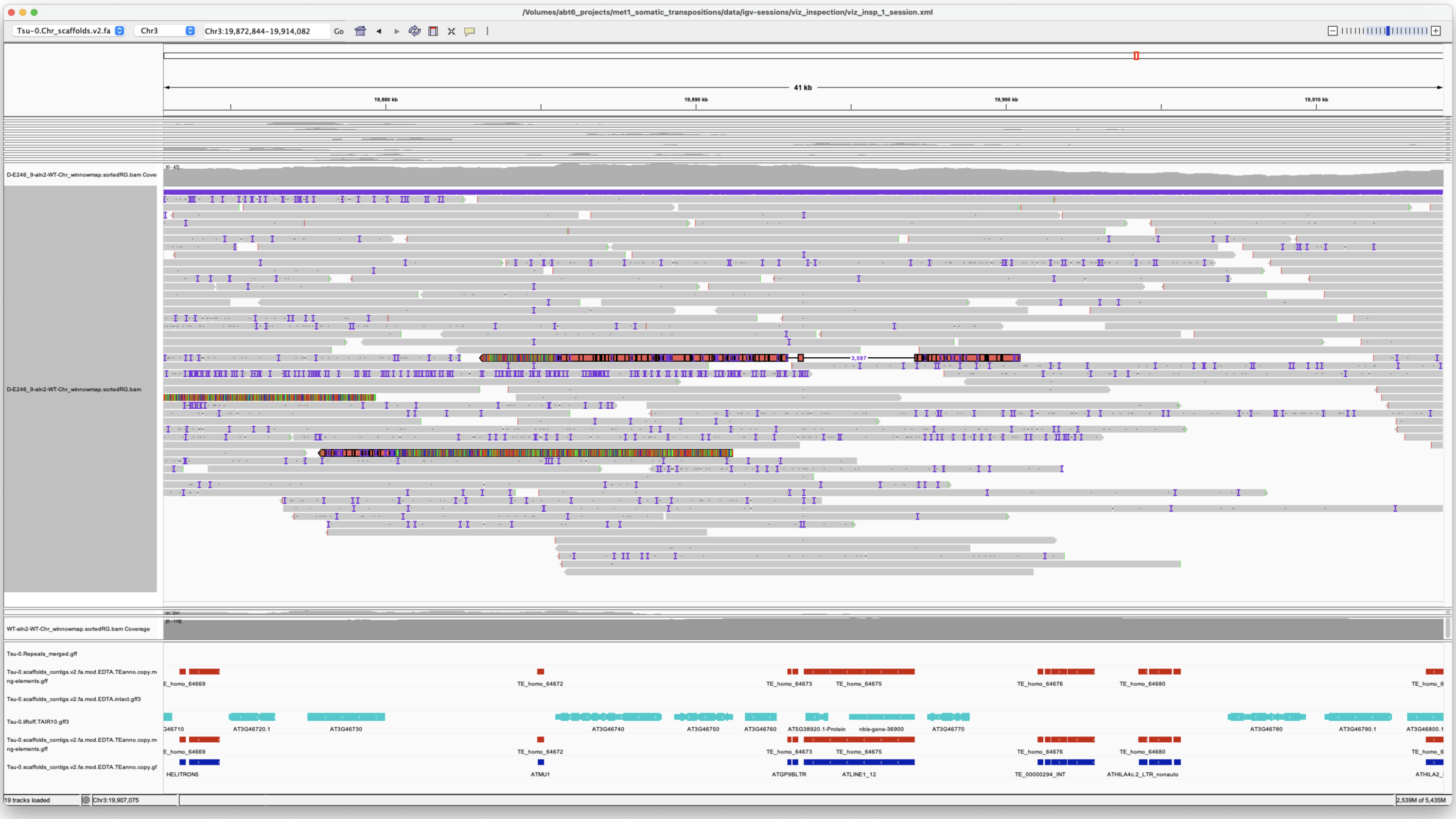

Weird, interesting Rearrangement

but confirmed that the deletion is overlapping perfectly with the annotated TE, which is a LINE and shouldnt be excided but..
probably something weird going on here OR ARTIFACT!!!
There is also a supplementary alignment on the left, THEREFORE:

Not Confirmed

> Chr5 4335532 4336888 ATDNAI27T9A met1_09

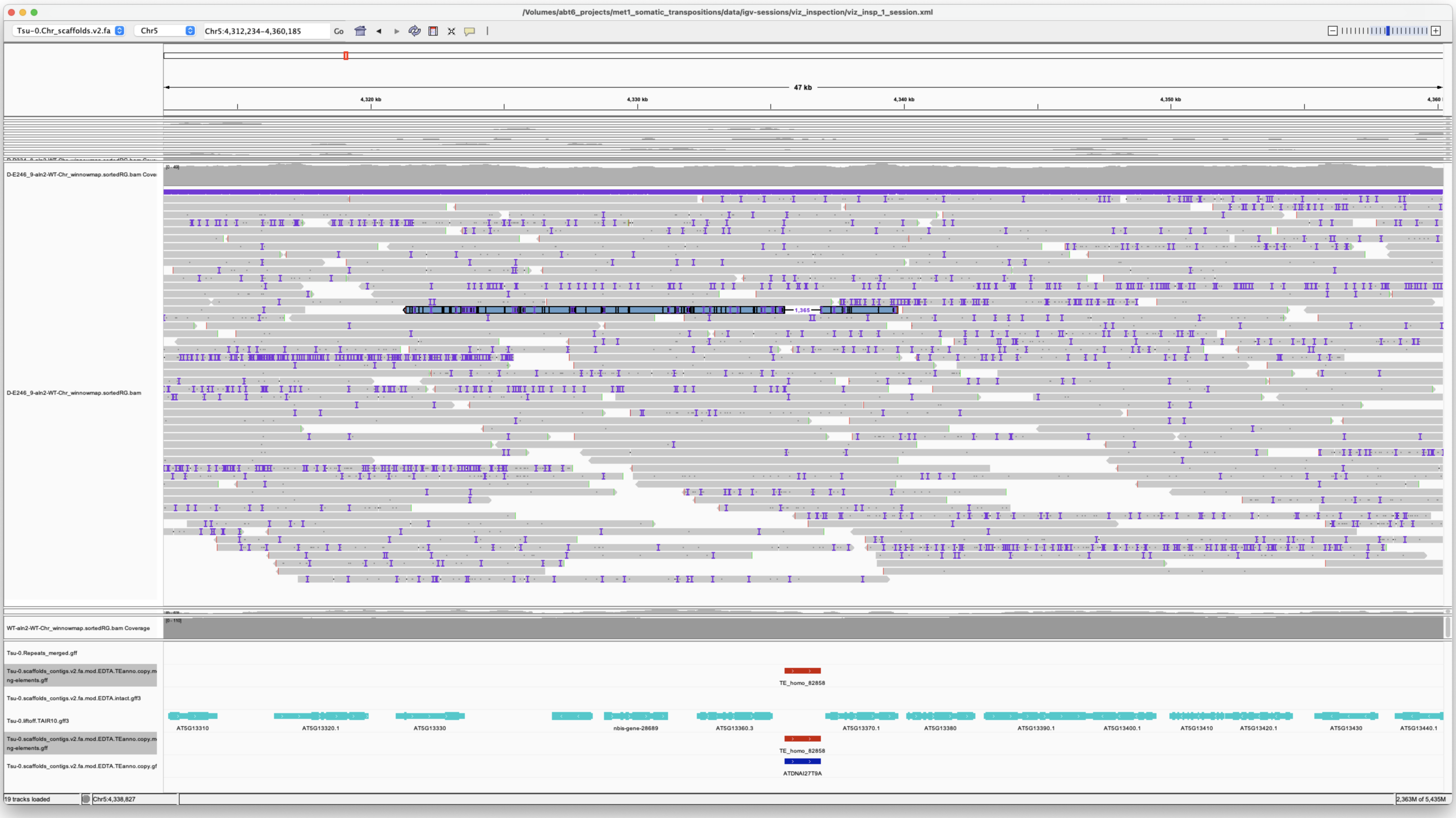

Seems real because it coinciding with the underlying annotation BUT there are no evidences of this element to insert in any met1 individual plant

> Chr5 8256939 8265133 CAC2A met1_09

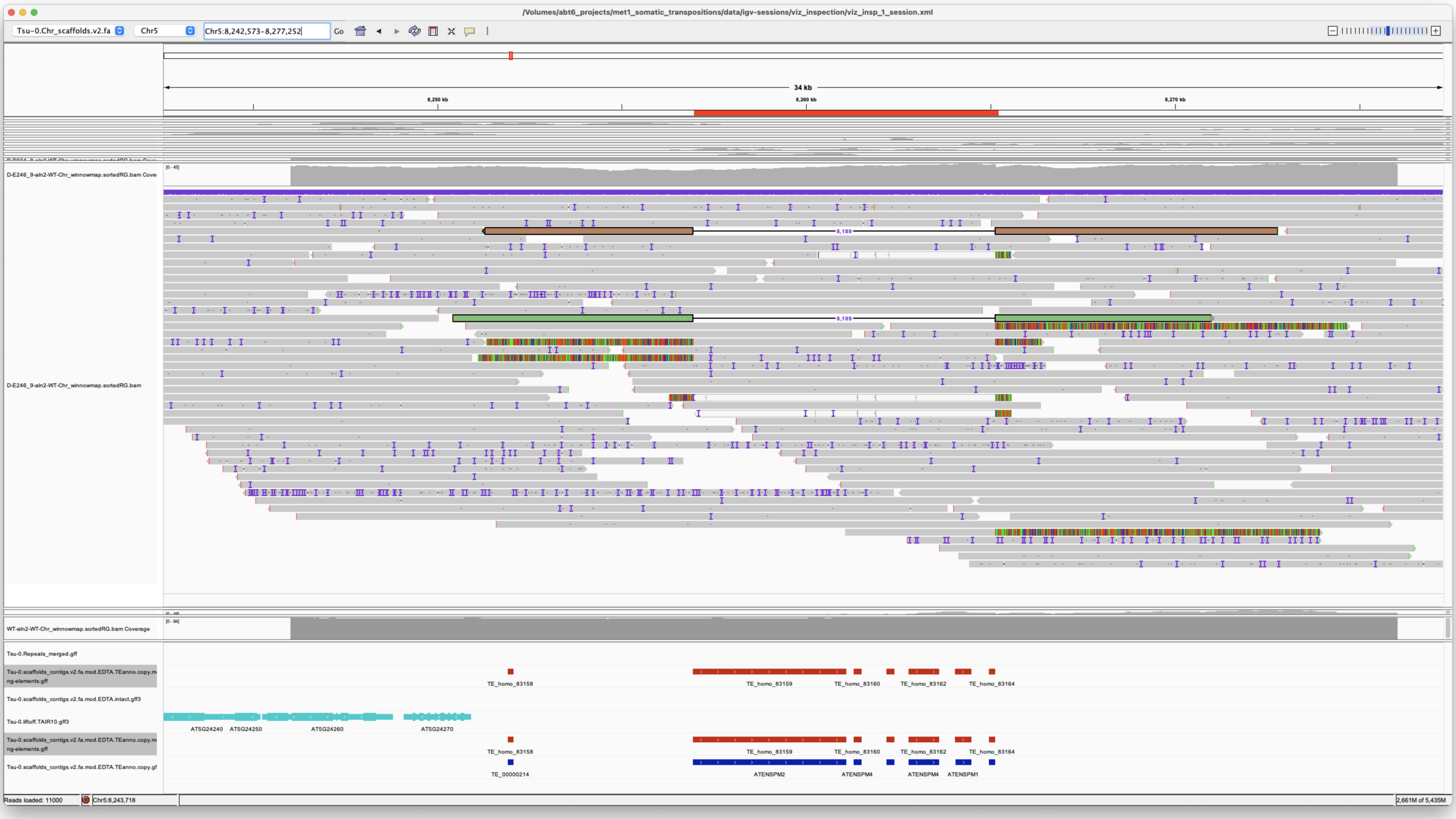

2x coverage

Confirmed

met1_10

Chr2 6734994 6775798 ATMSAT1 met1_10

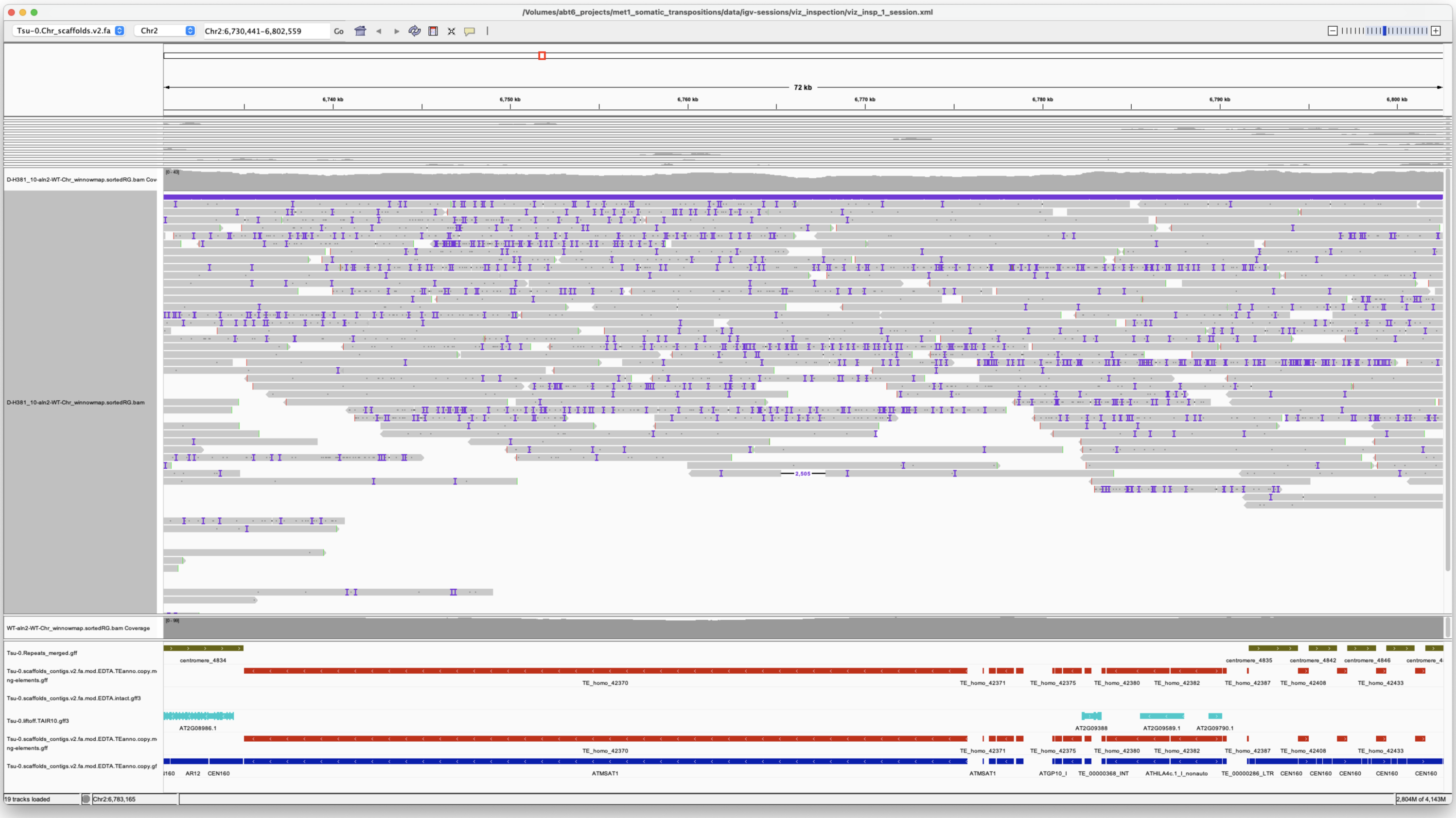

SATELLITE

Chr5 19872565 19877095 VANDAL21 met1_10

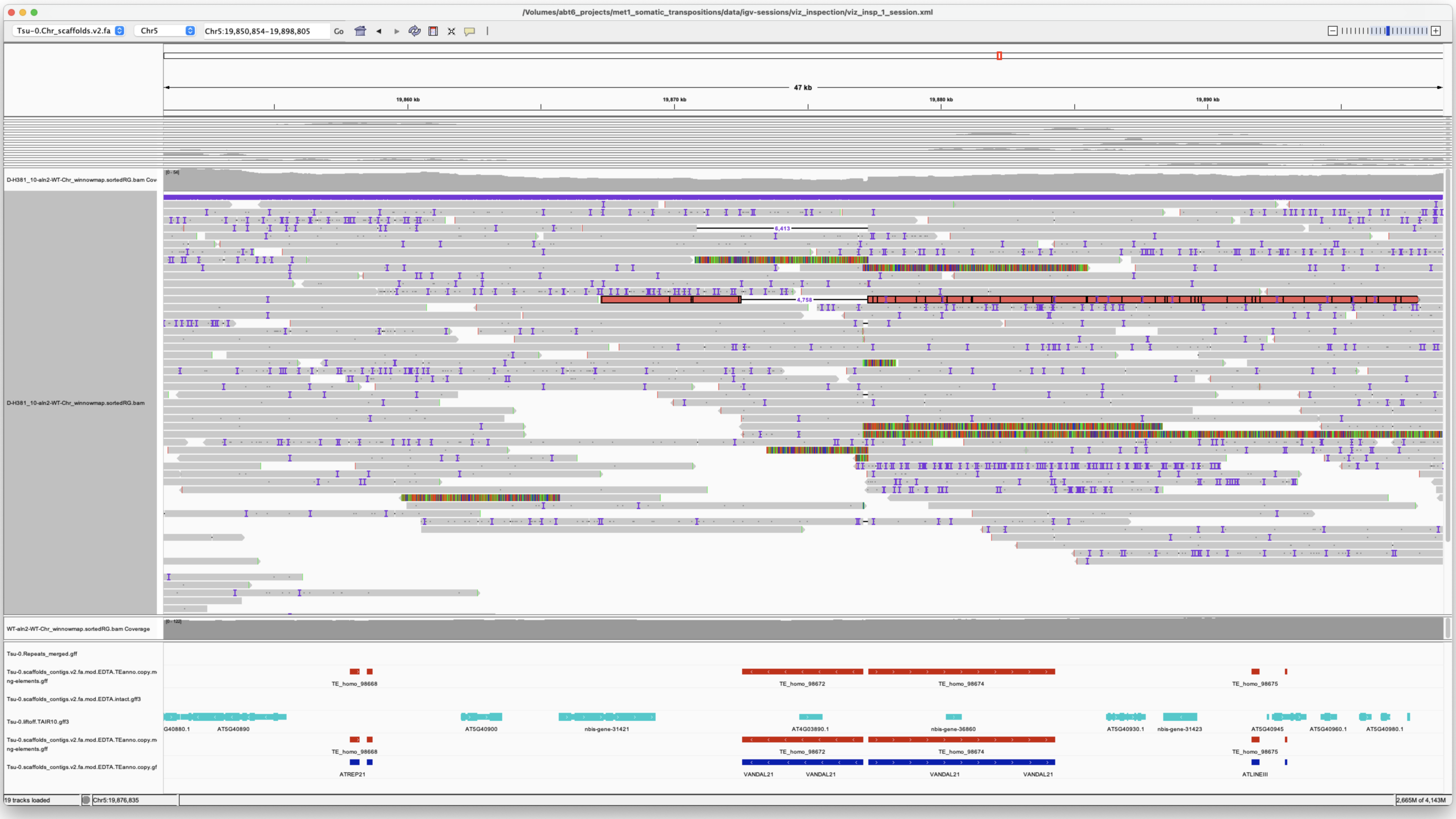

~200bp to the right of the element are also cut.

In this locus there are several rearrangements! this could be easily one of them

Not Confirmed

WT
