# Supplementary material for: Long-read detection of transposable element mobilization in the soma of hypomethylated Arabidopsis thaliana individuals
Source: Genome Biol. 2025 Jul 30;26:231. doi: 10.1186/s13059-025-03691-7 (PMC12312487; doi:10.1186/s13059-025-03691-7)
Supplement: Supplementary file 2 — Additional file 2. Visual inspection of somatic insertion and excision events, available at https://github.com/aerilli/Somatic-transposition_met1/tree/551df407370c6528225f404ba62a073dced14b08/Supplementary-Files/Visual_inspection. [file 13059_2025_3691_MOESM2_ESM.gz › Split_Supplementary-File4/File1_SupplementaryALN_Insertions/File1_SupplementaryALN_Insertions-137-204.pdf]

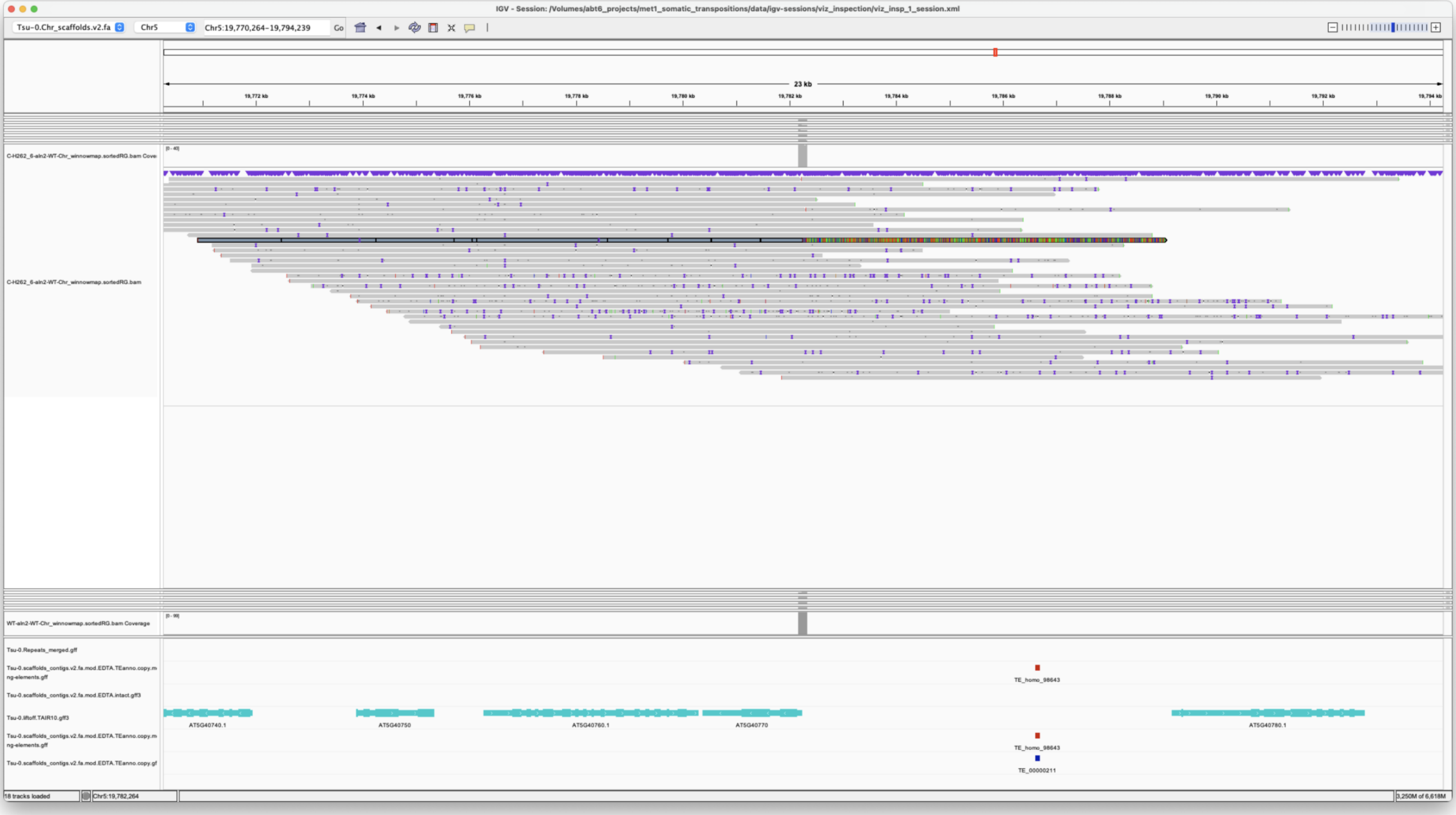

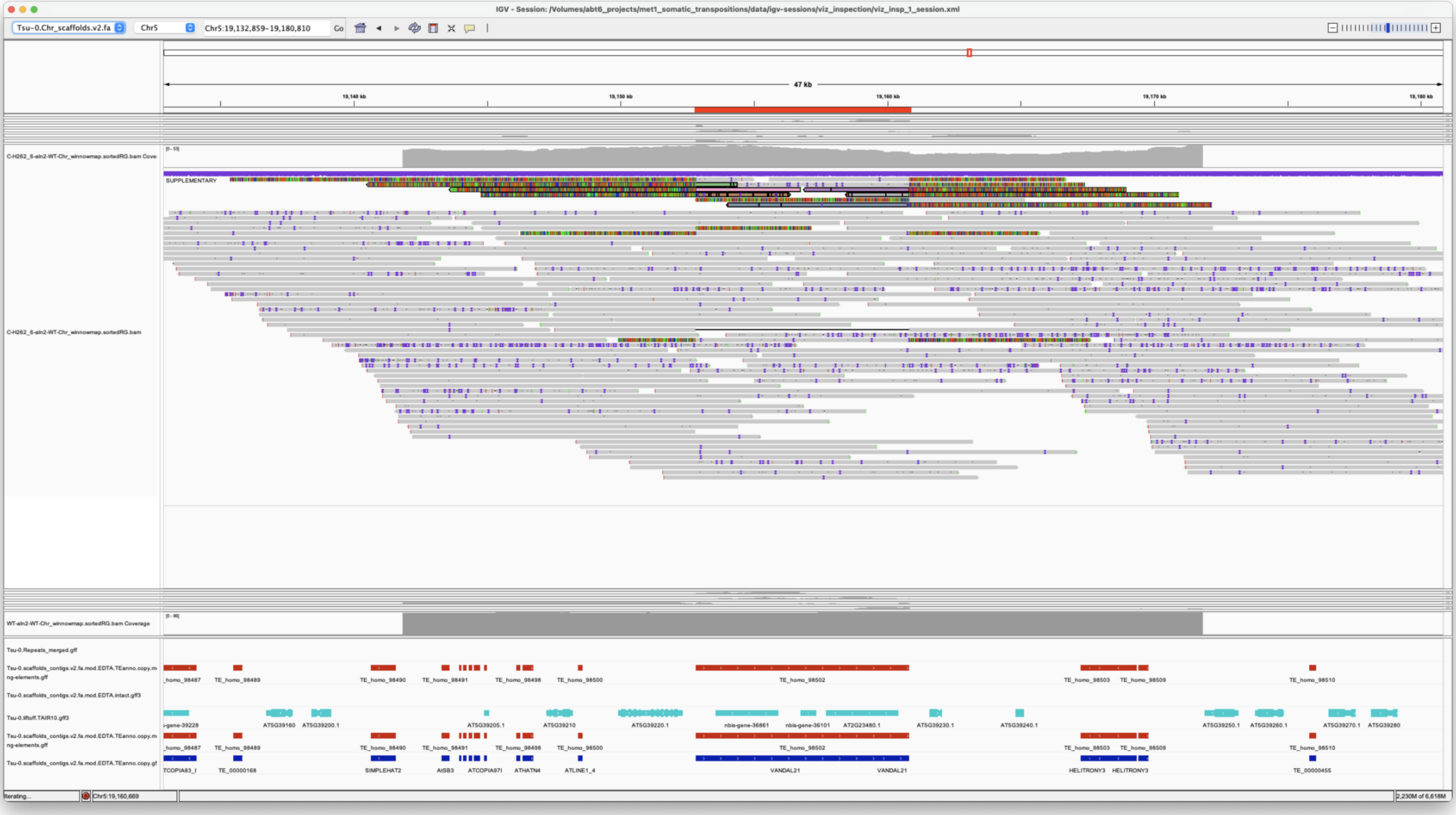

Partial

Confirmed

Chr5 19975545 19975545 + 1 Chr5;19872565;19877095;VANDAL21 m64079_240212_113350/1247781/ccs met1_06

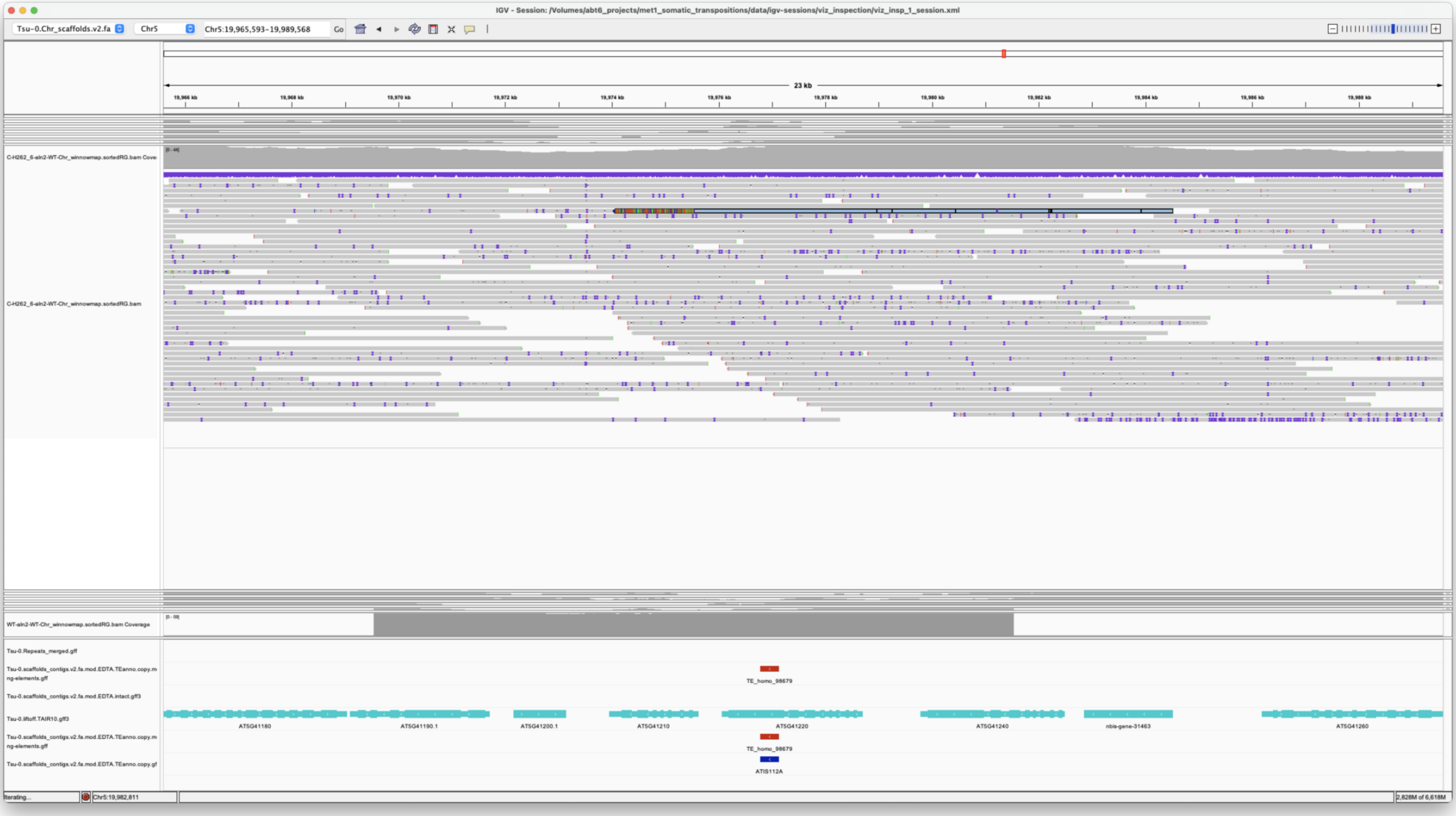

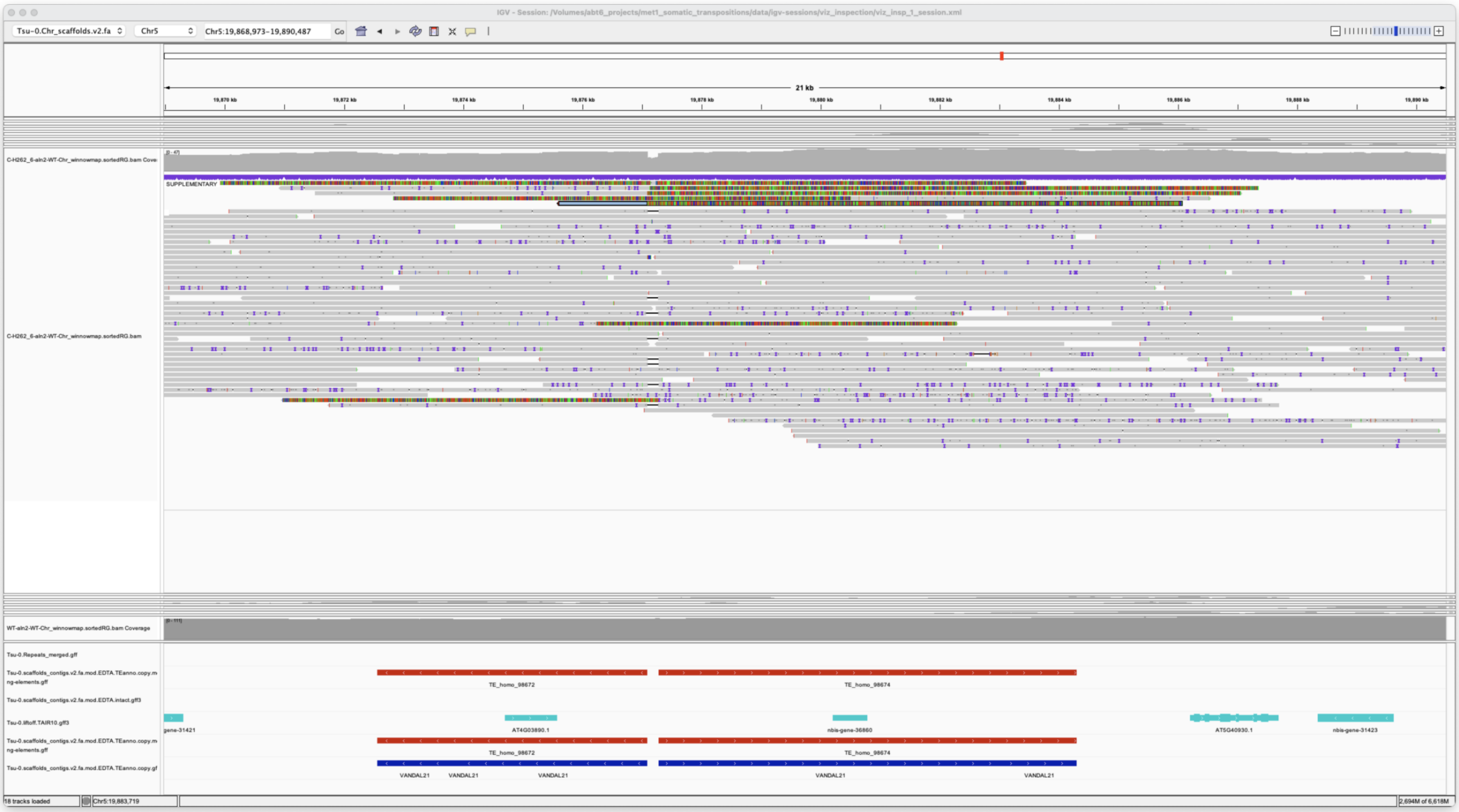

I don't trust this to be an insertion!!

Hypermutable region

Rearrangement

unsupported

> Chr5 22178360 22178360 - 1 Chr5;19152829;19160826;VANDAL21 m64079_221220_112036/156108083/ccs met1_06

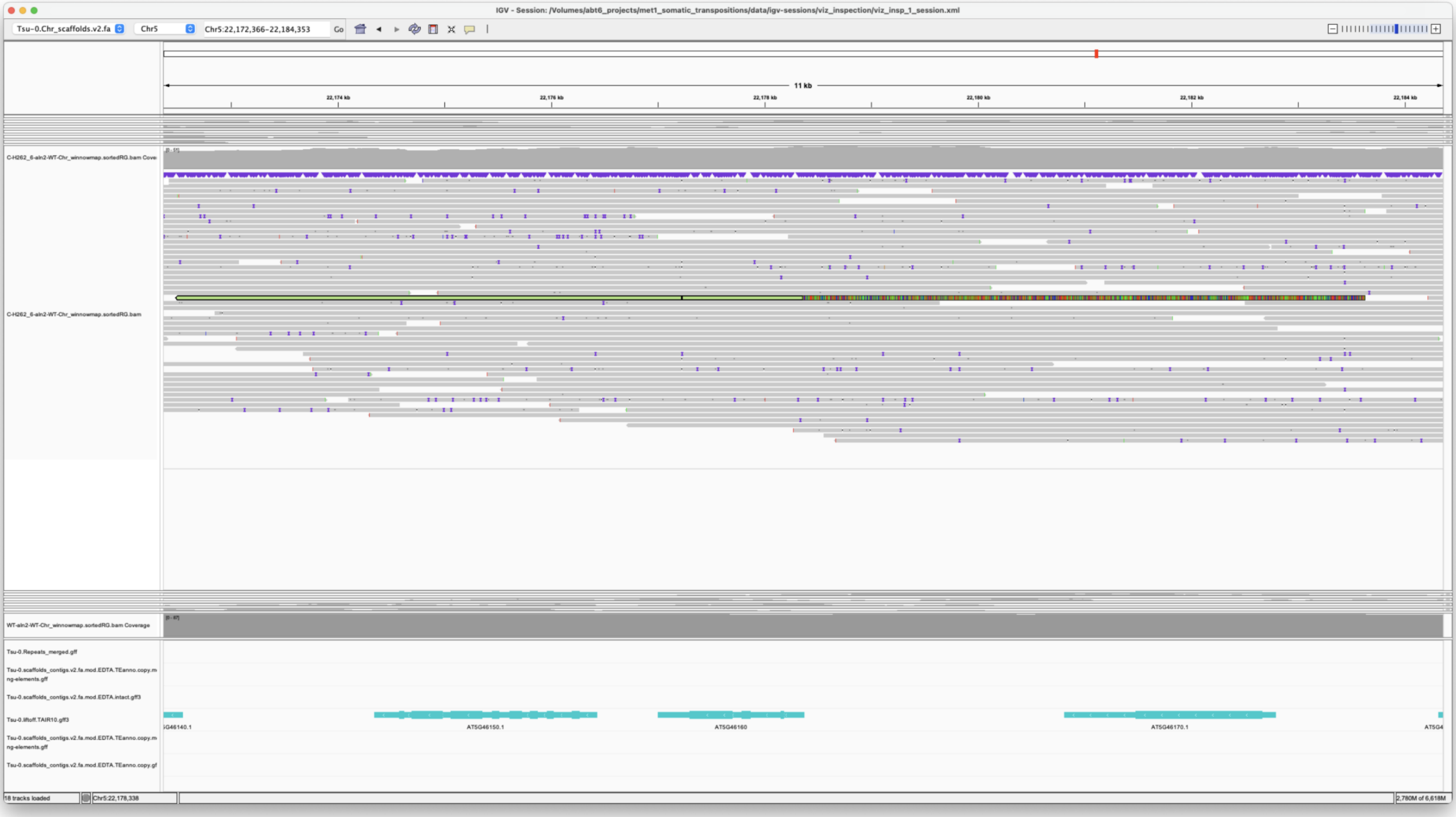

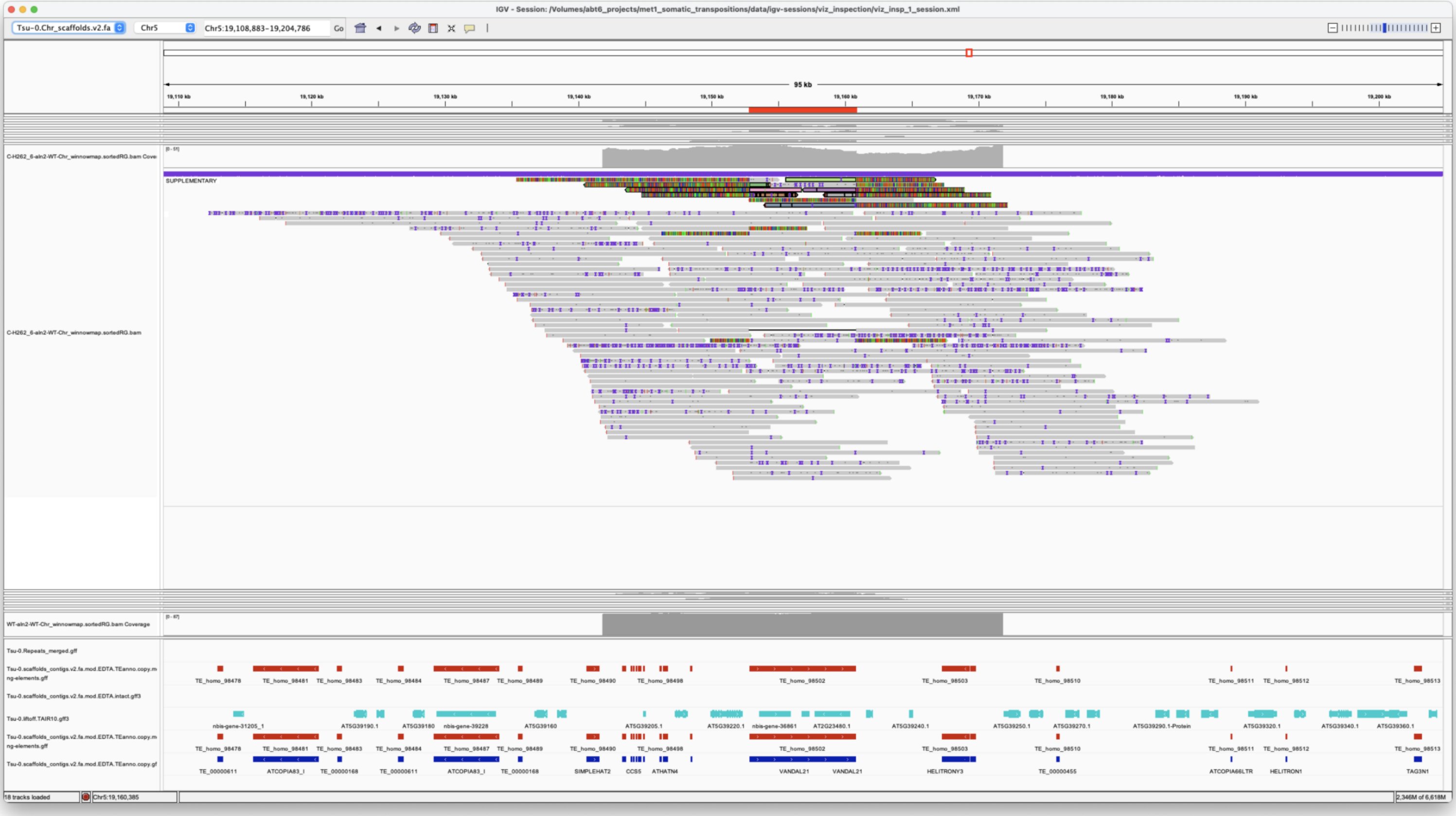

Partial

Confirmed

Chr5 23899981 23899981 + 1 Chr3;16344522;16352497;VANDAL6 m64079_221220_112036/49219298/ccs met1_06

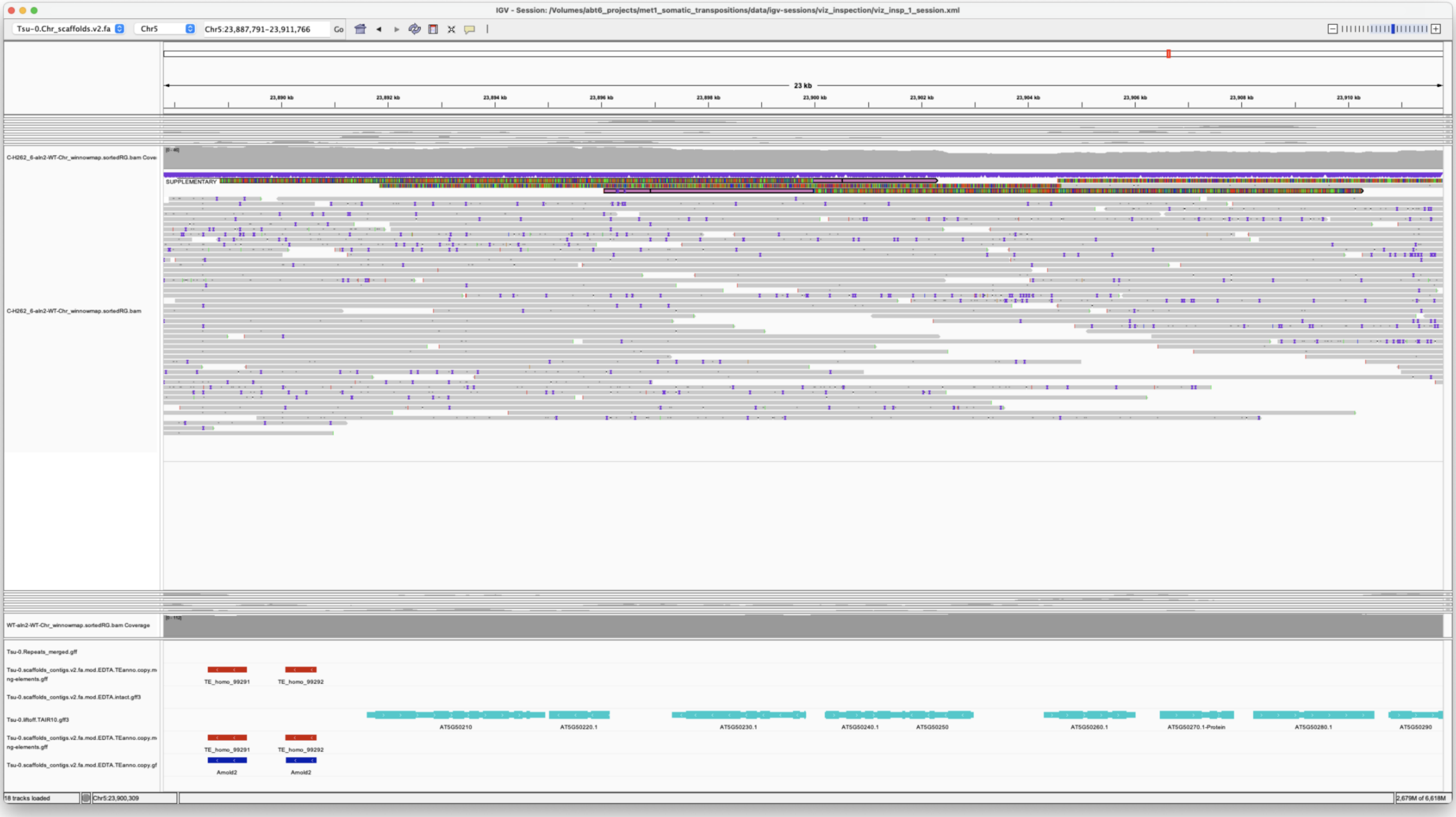

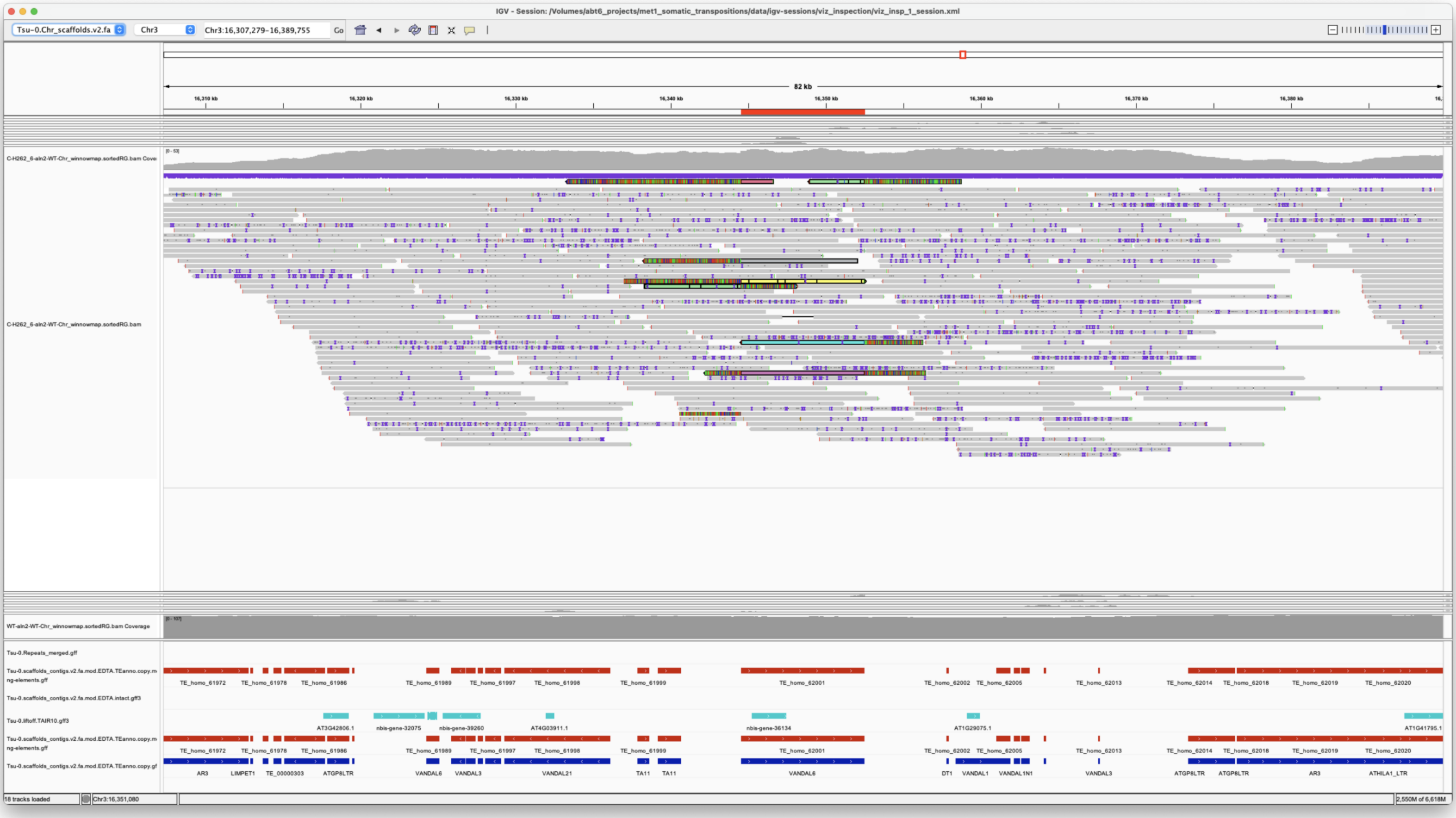

Central
TSD

Confirmed

Chr5 24523721 24523721 + 1 Chr1;11941106;11946436;ATCOPIA93_Evade m64079_240212_113350/107283444/ccs met1_06

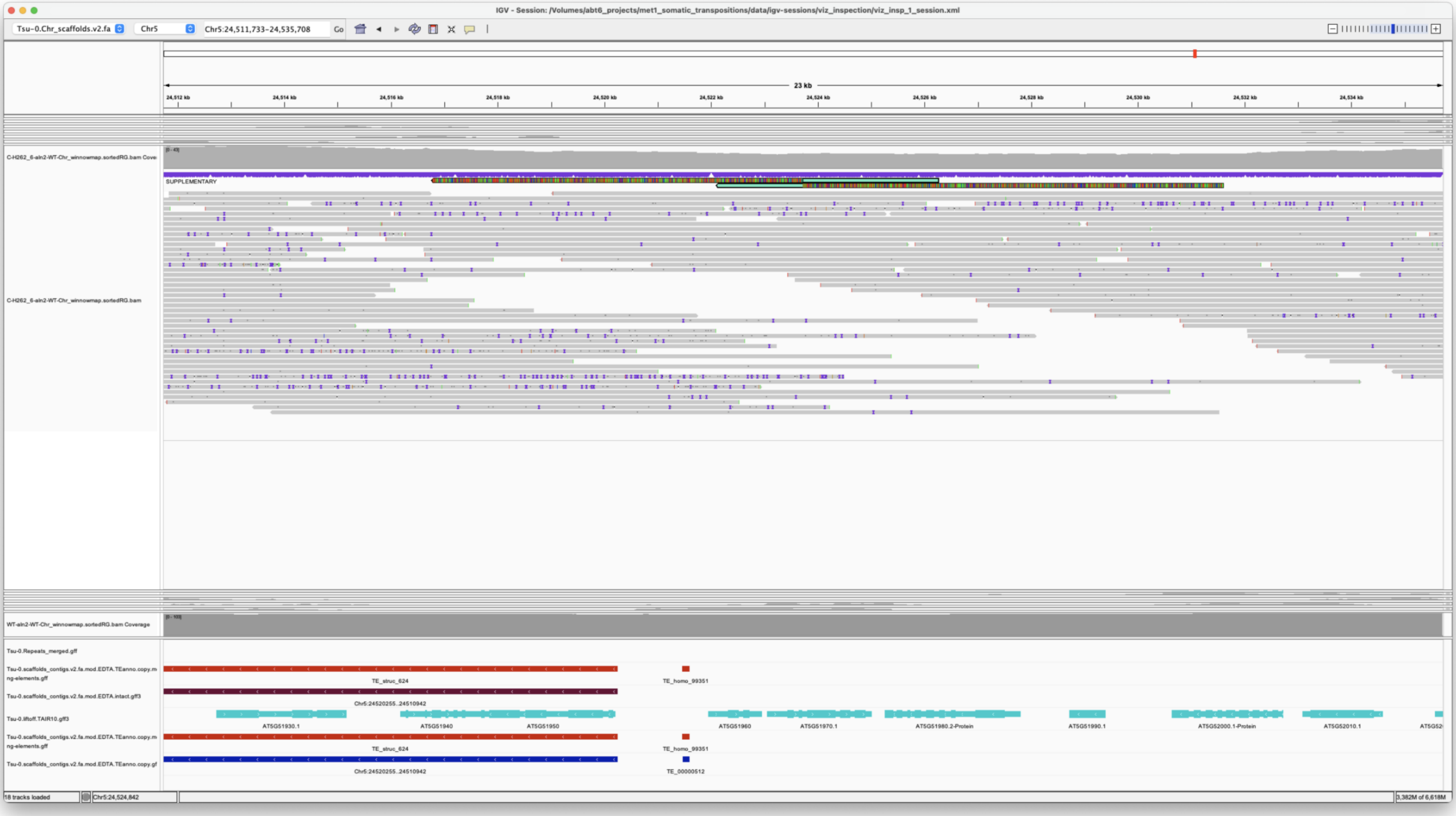

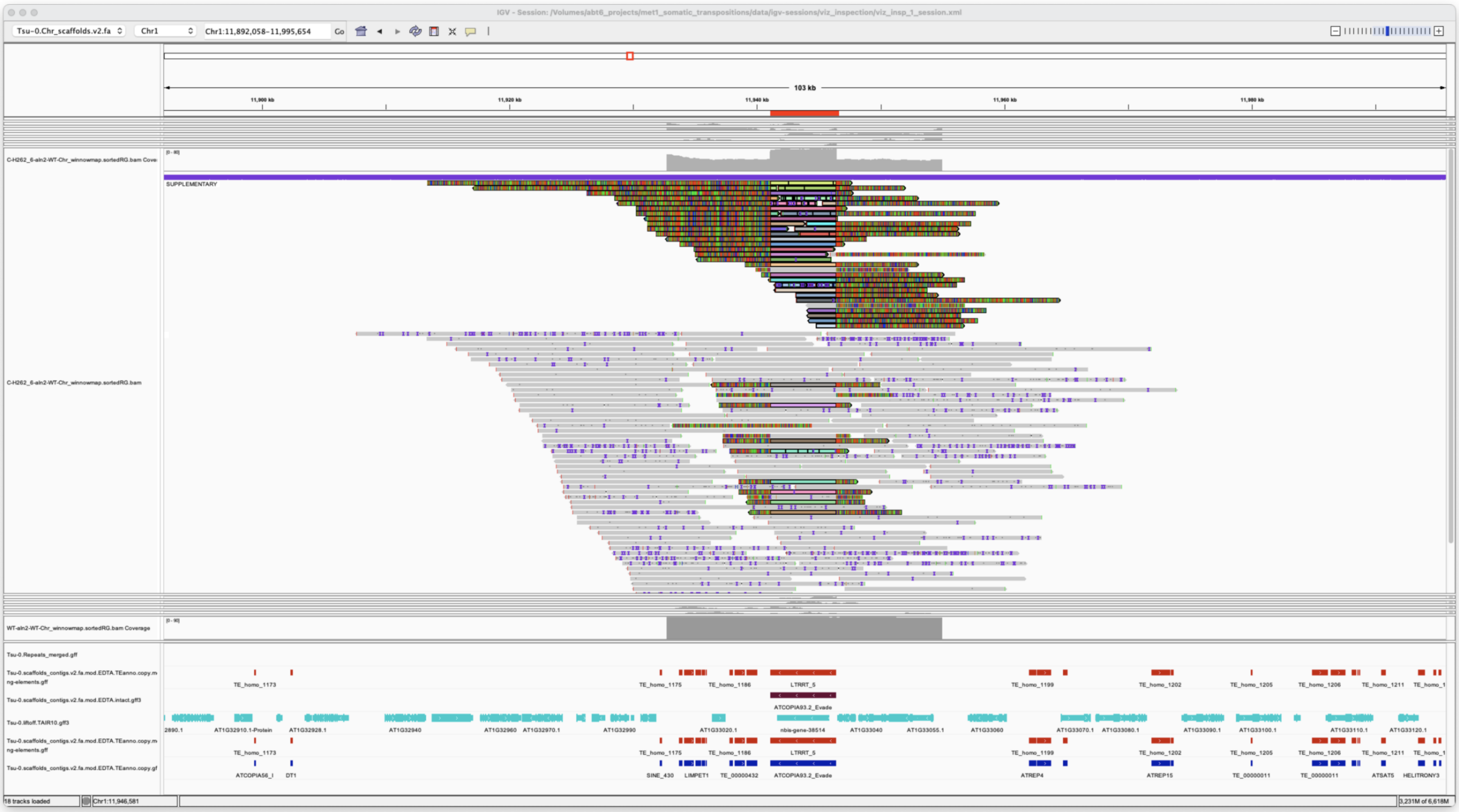

Central
TSD

Confirmed

Chr5 28568264 28568264 + 1 Chr3;20158137;20166150;VANDAL6 m64079_240212_113350/167511582/ccs met1_06

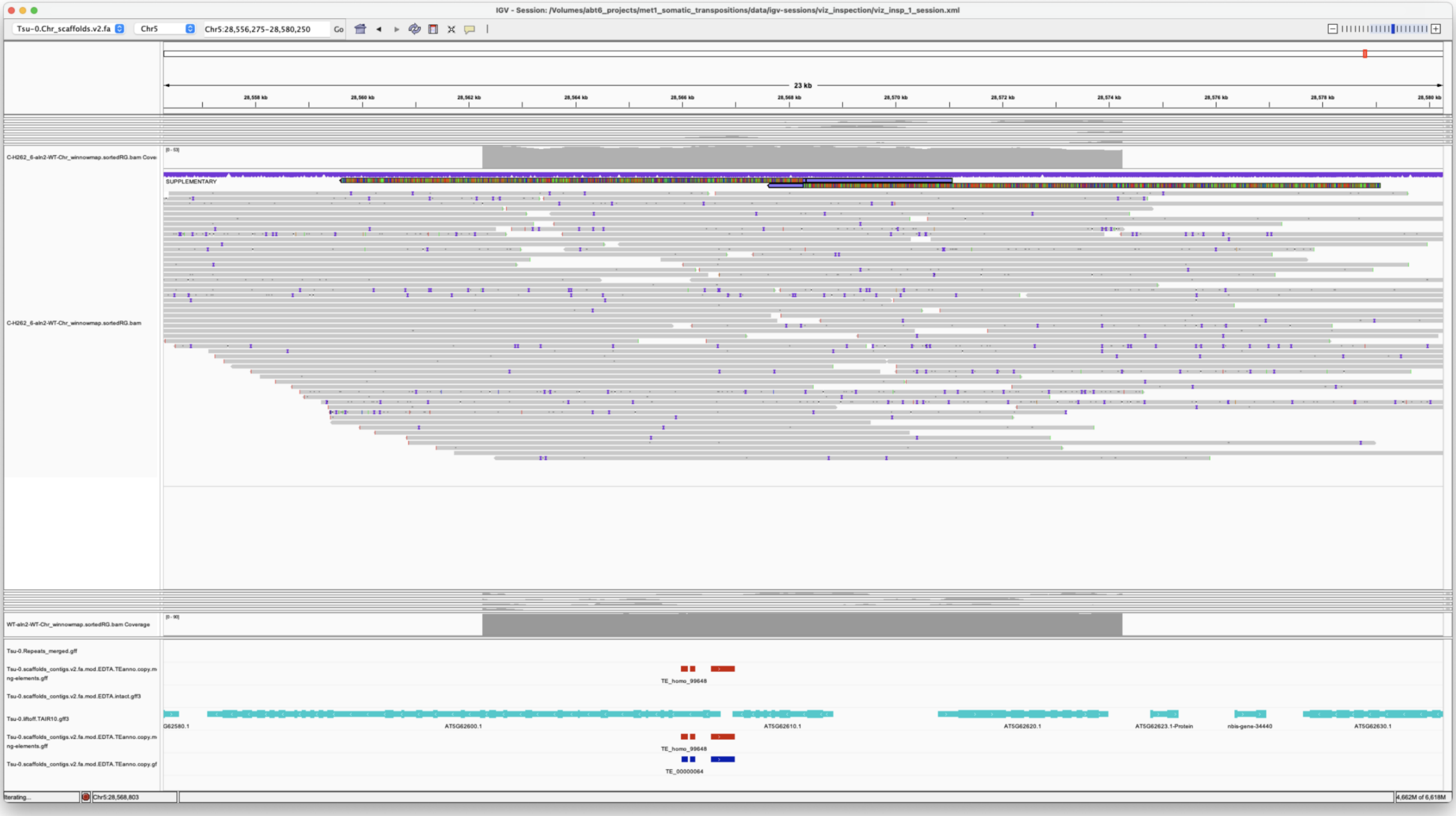

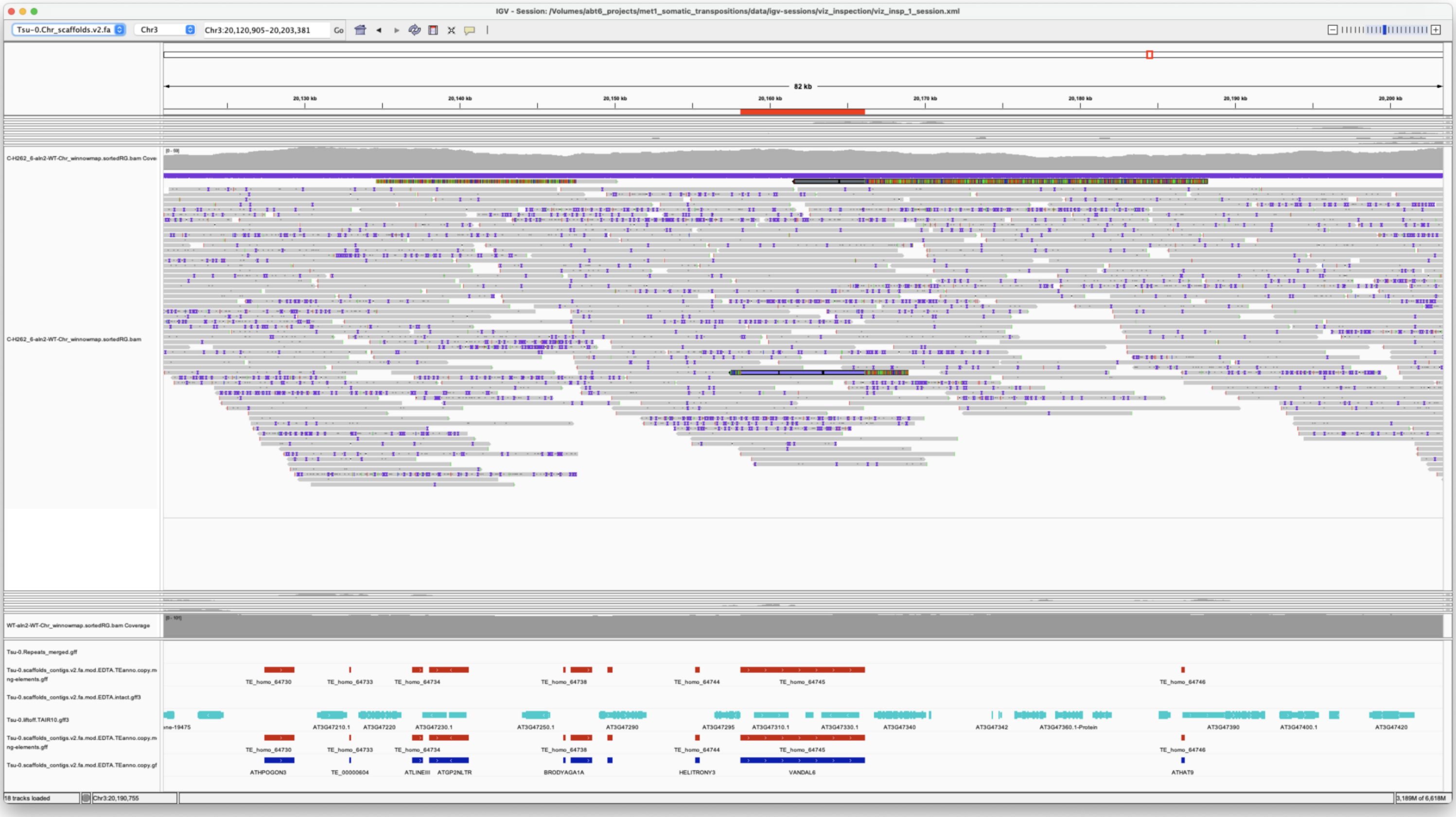

Central
TSD

Confirmed

---

met1_07

| Chr1 2444901 2444901 - 1 Chr5;19152829;19160826;VANDAL21 m64079_221220_112036/136511916/ccs met1_07

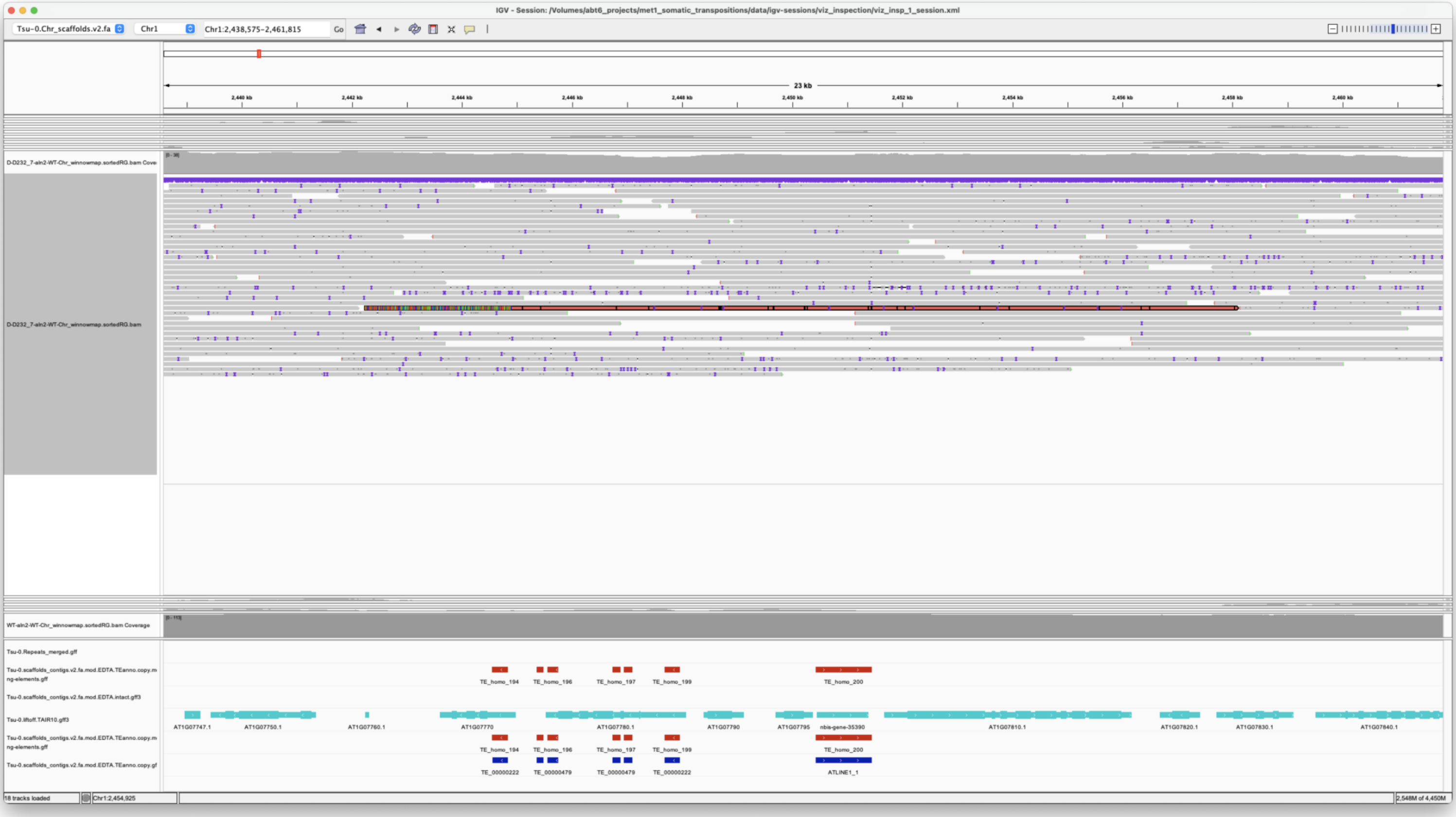

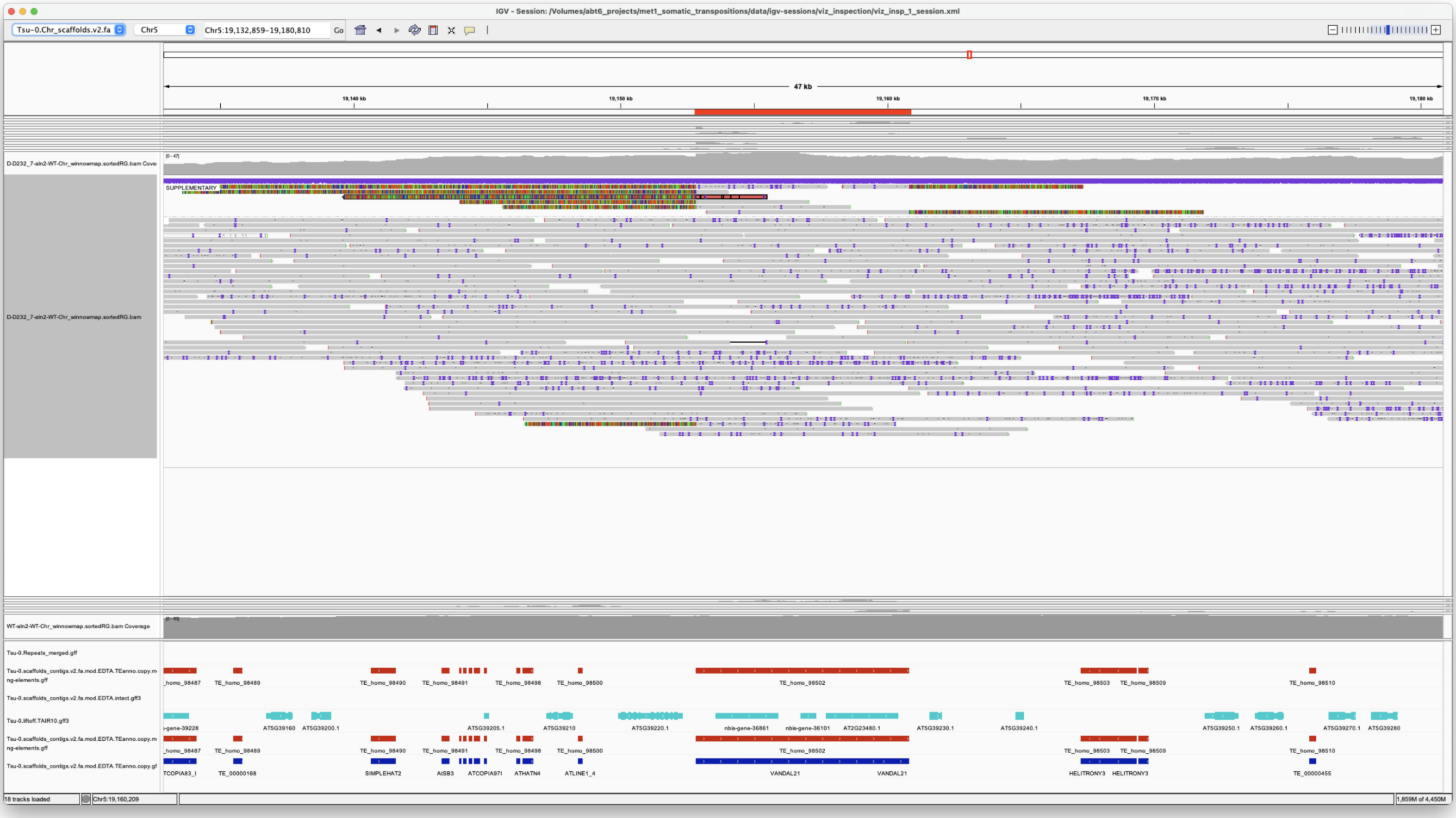

Partial

Confirmed

Chr1 5462985 5462985 + 1 Chr1;11941106;11946436;ATCOPIA93_Evade m64079_240212_113350/159187683/ccs met1_07

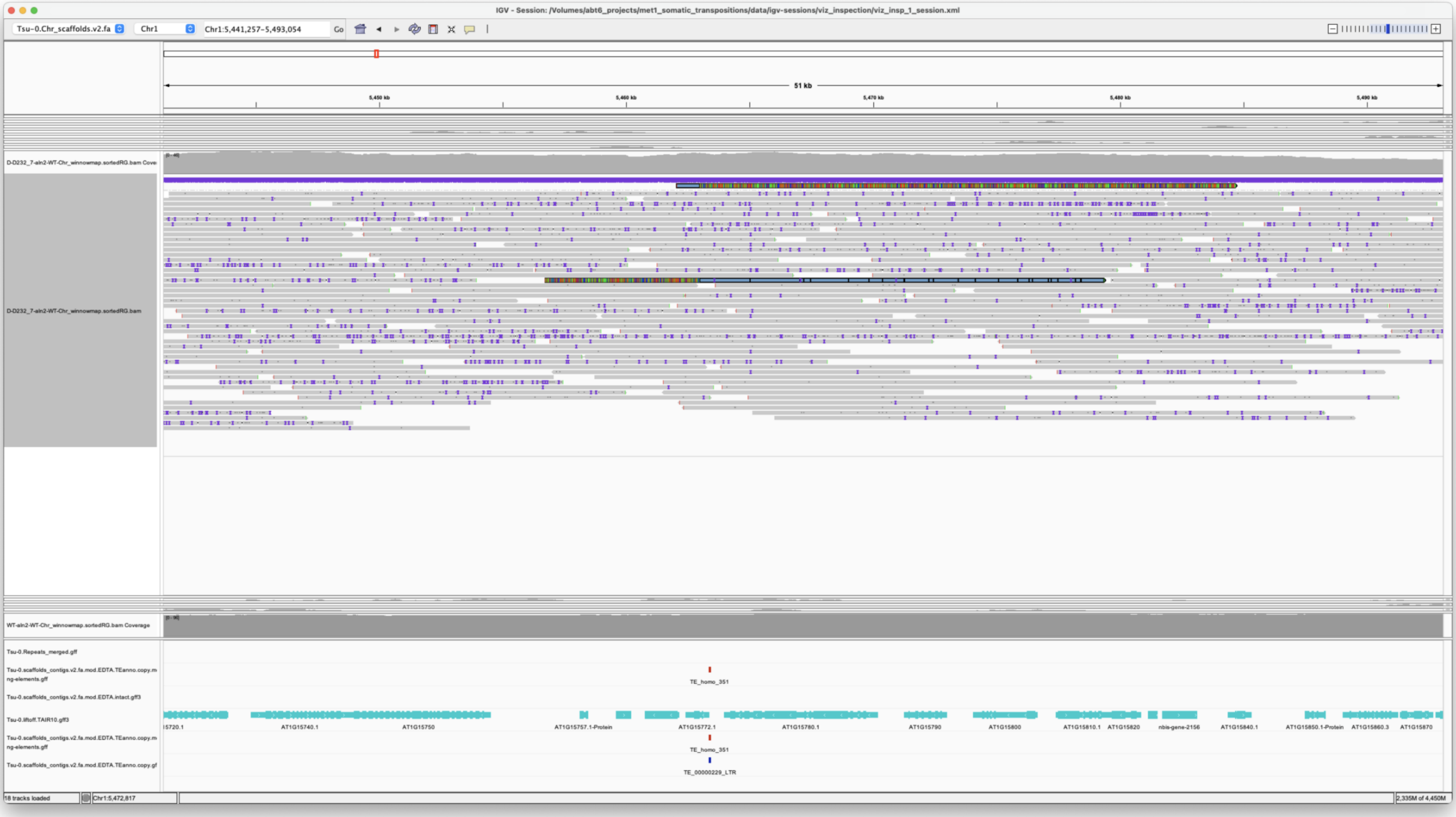

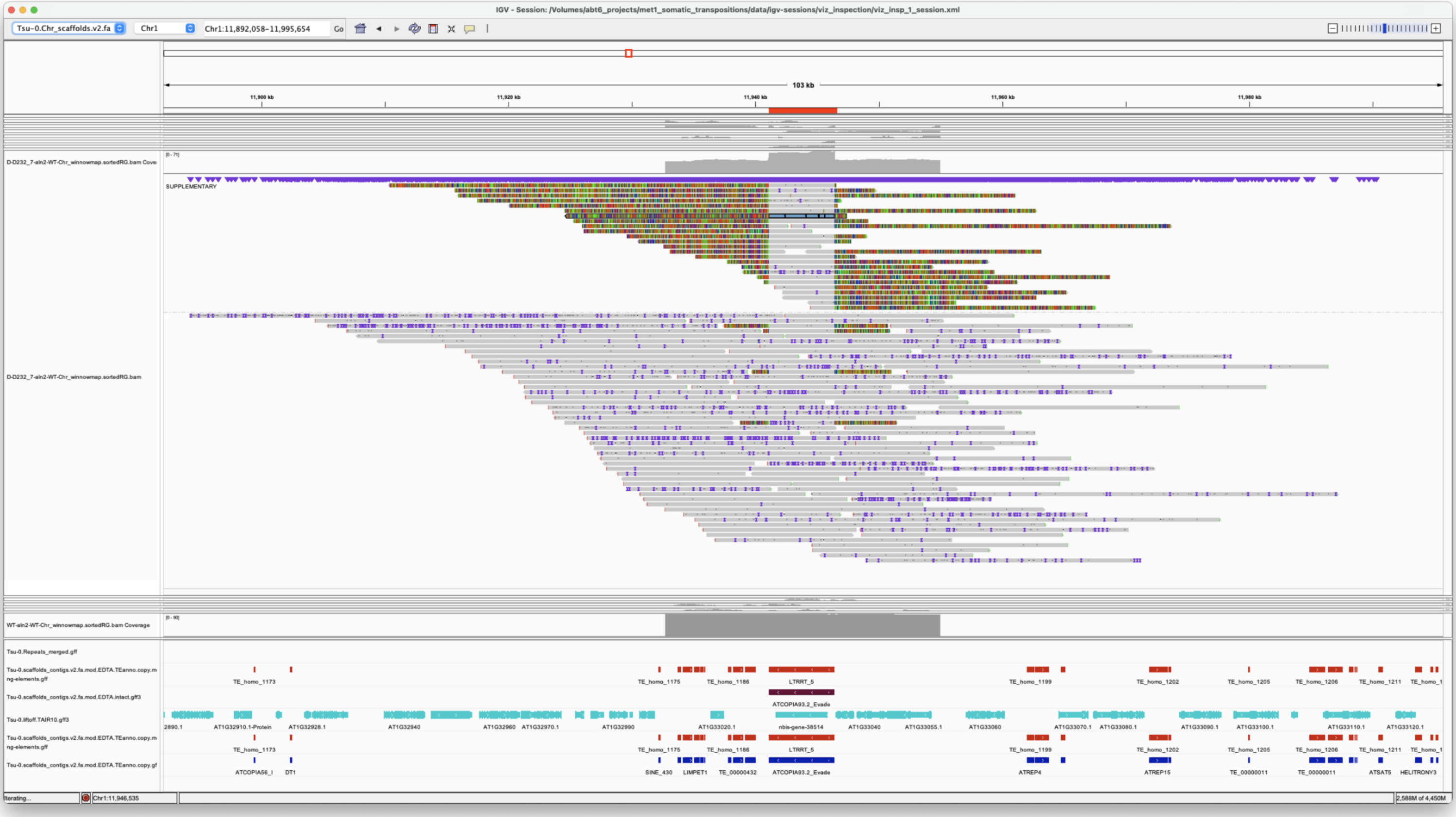

Central
TSD

Confirmed

Chr1 19126631 19126631 - 1 Chr5;19152829;19160826;VANDAL21 m64079_240212_113350/124257824/ccs met1_07

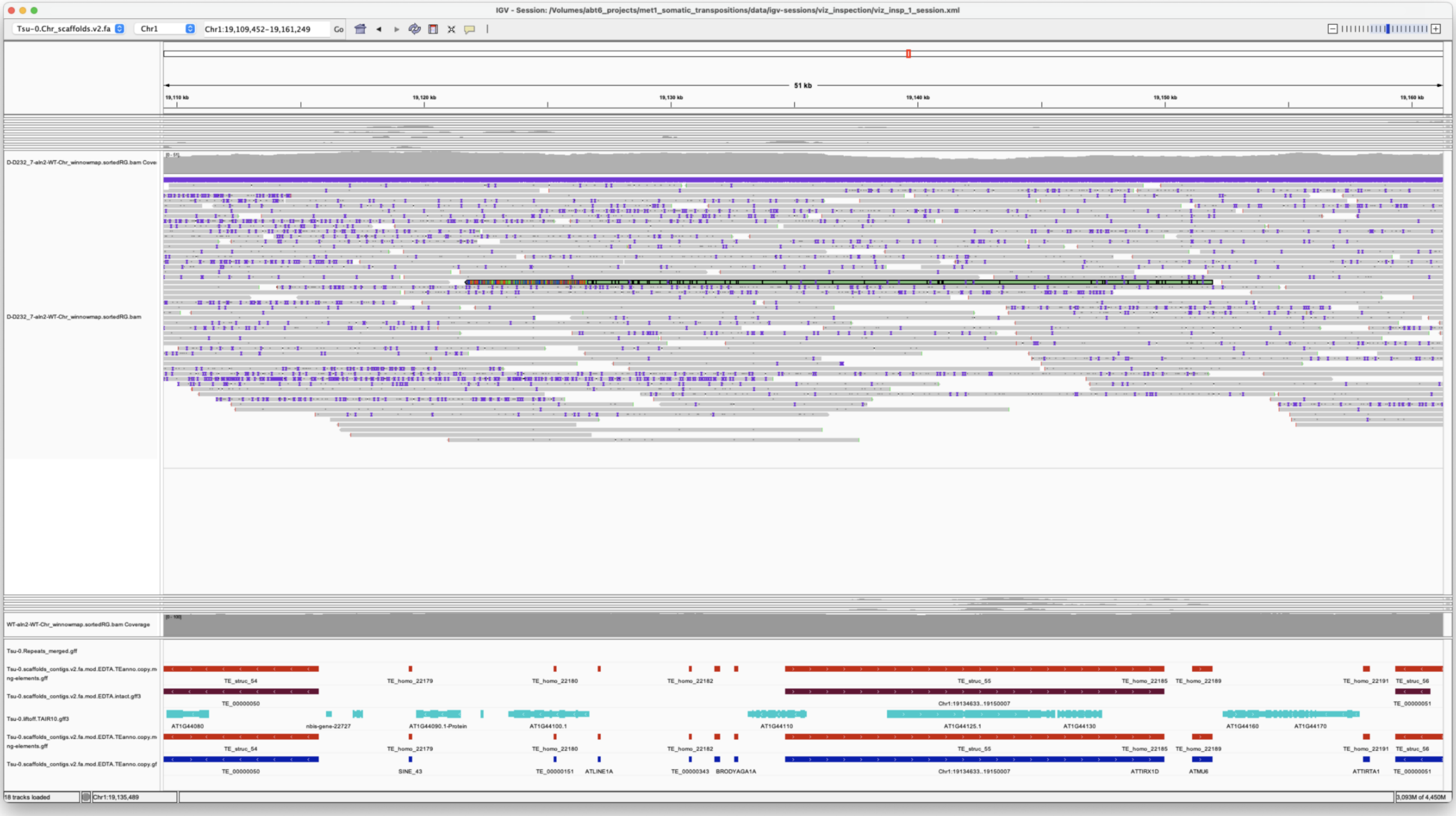

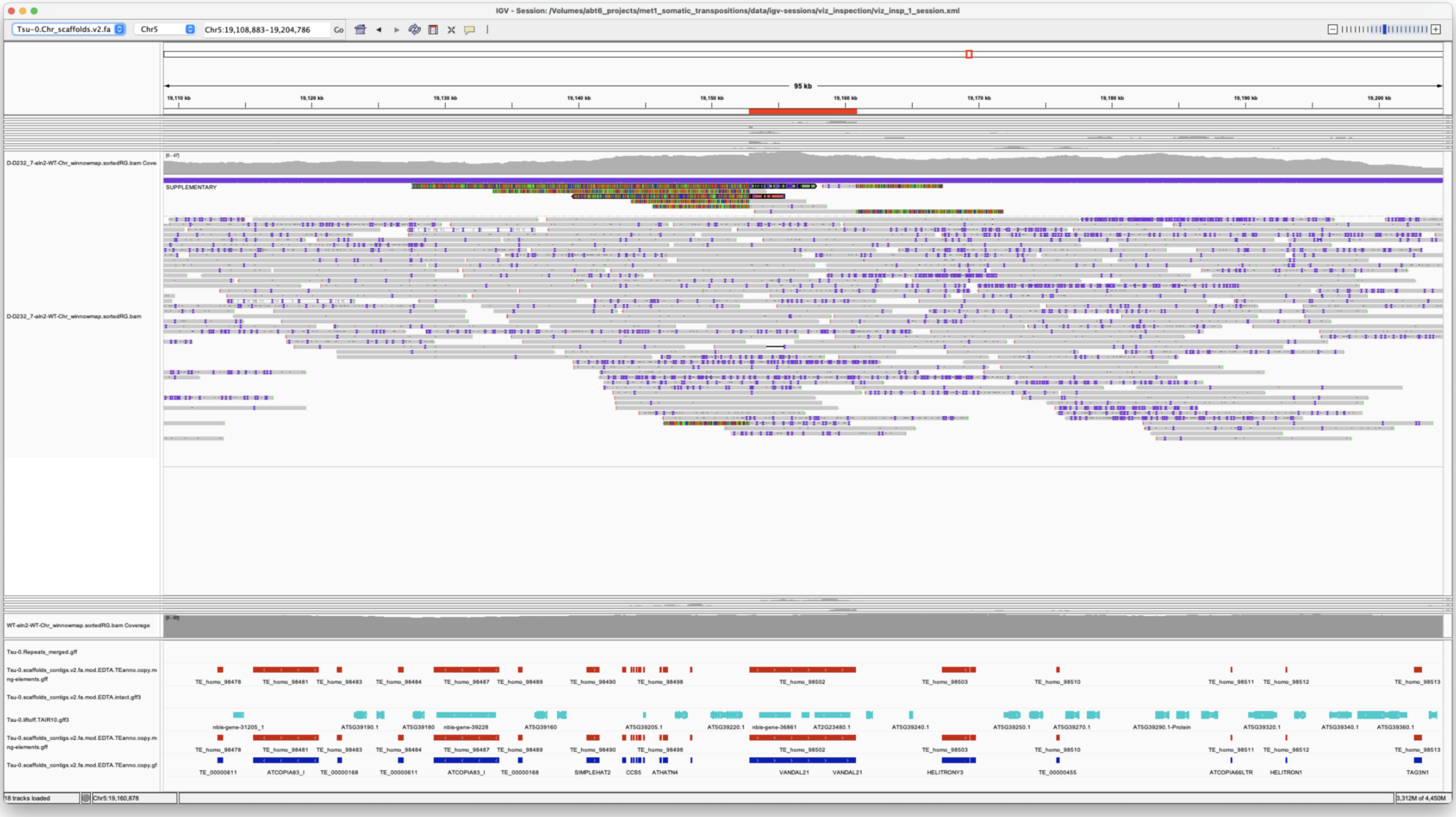

Partial

Confirmed

Chr1 24311719 24311719 + 1 Chr5;19152829;19160826;VANDAL21 m64079_240212_113350/139854094/ccs met1_07

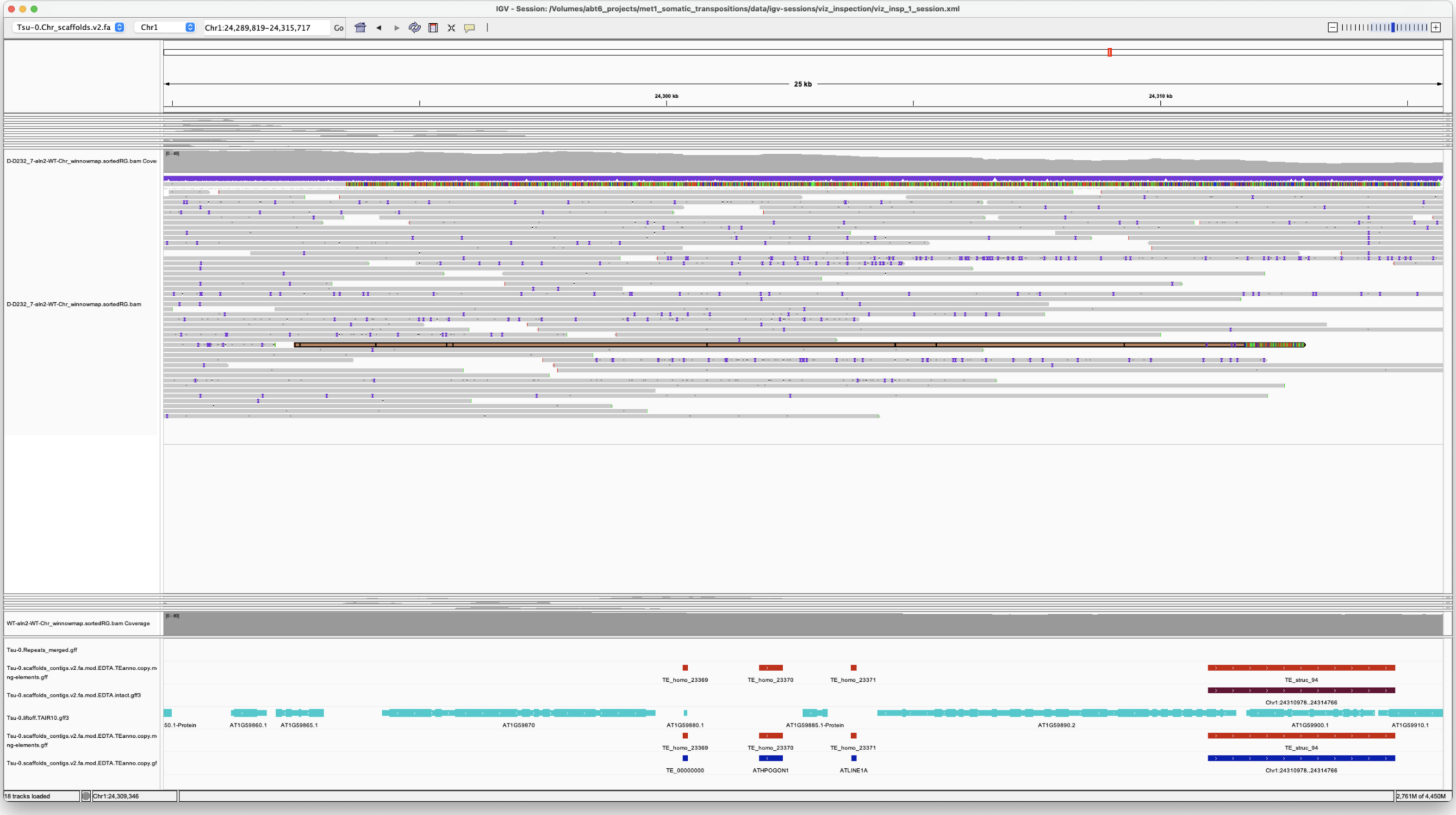

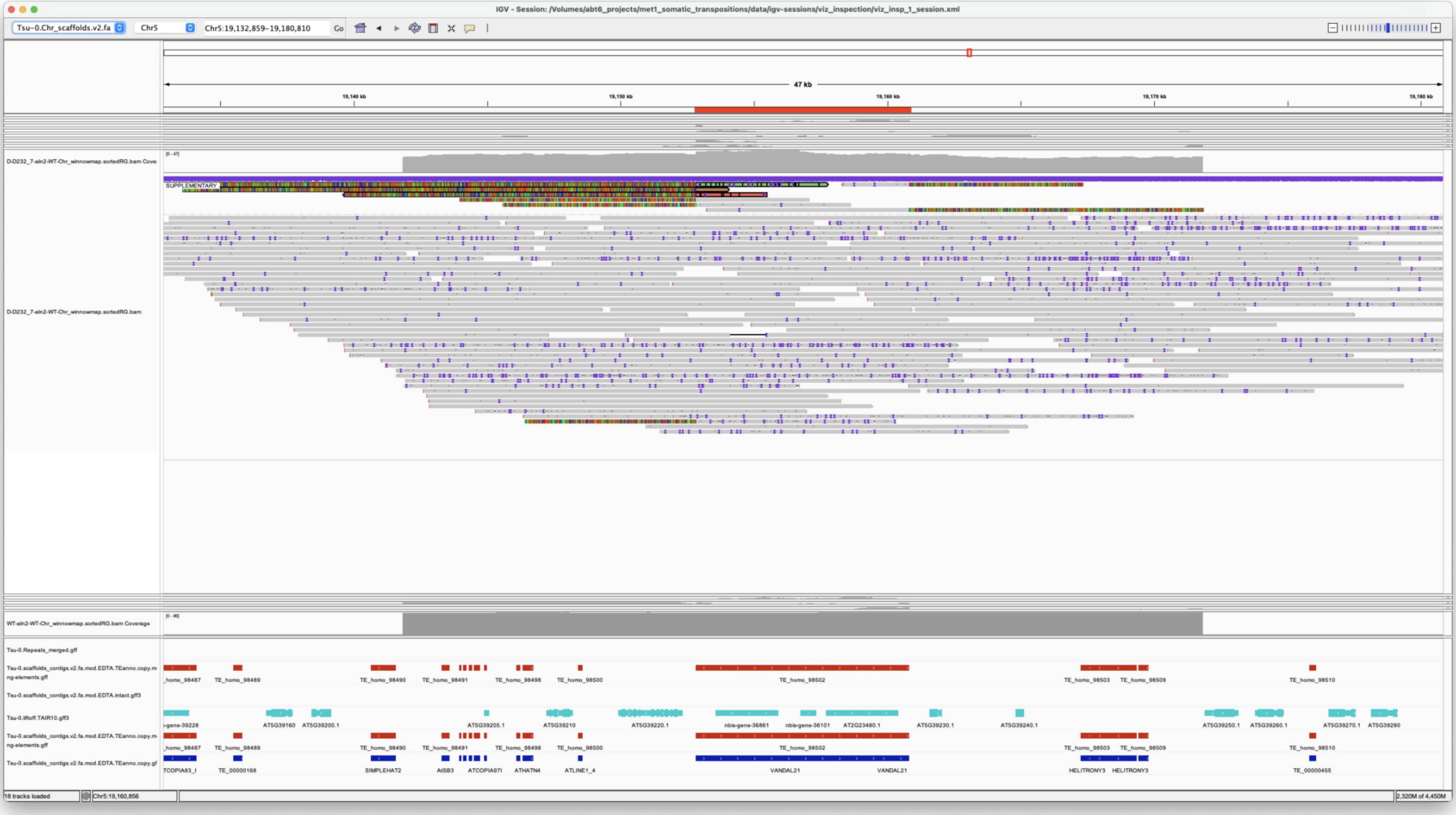

Partial

Confirmed

Chr1 26654694 26654694 - 1 Chr1;11941106;11946436;ATCOPIA93_Evade m64079_221220_112036/12847280/ccs met1_07

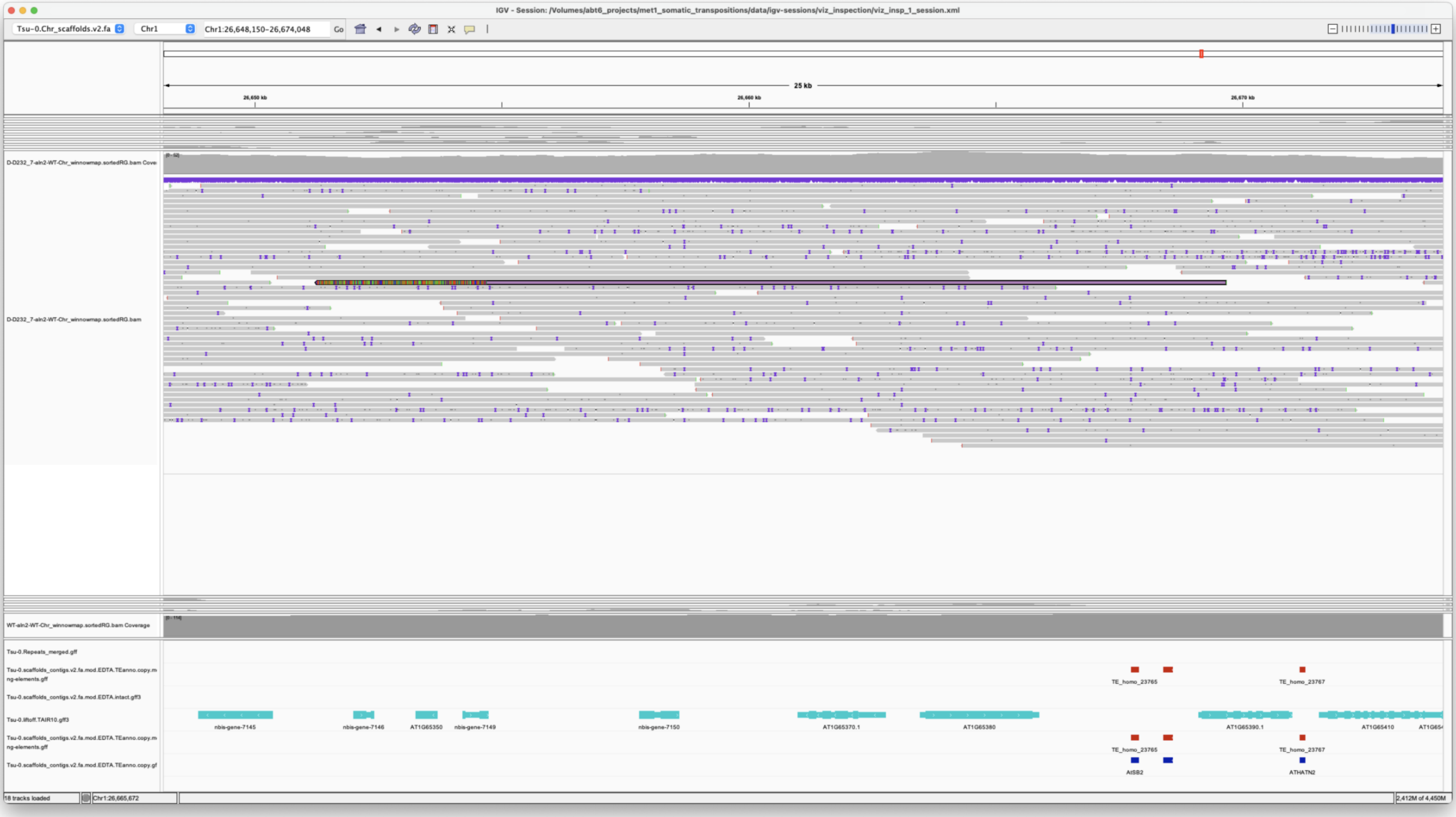

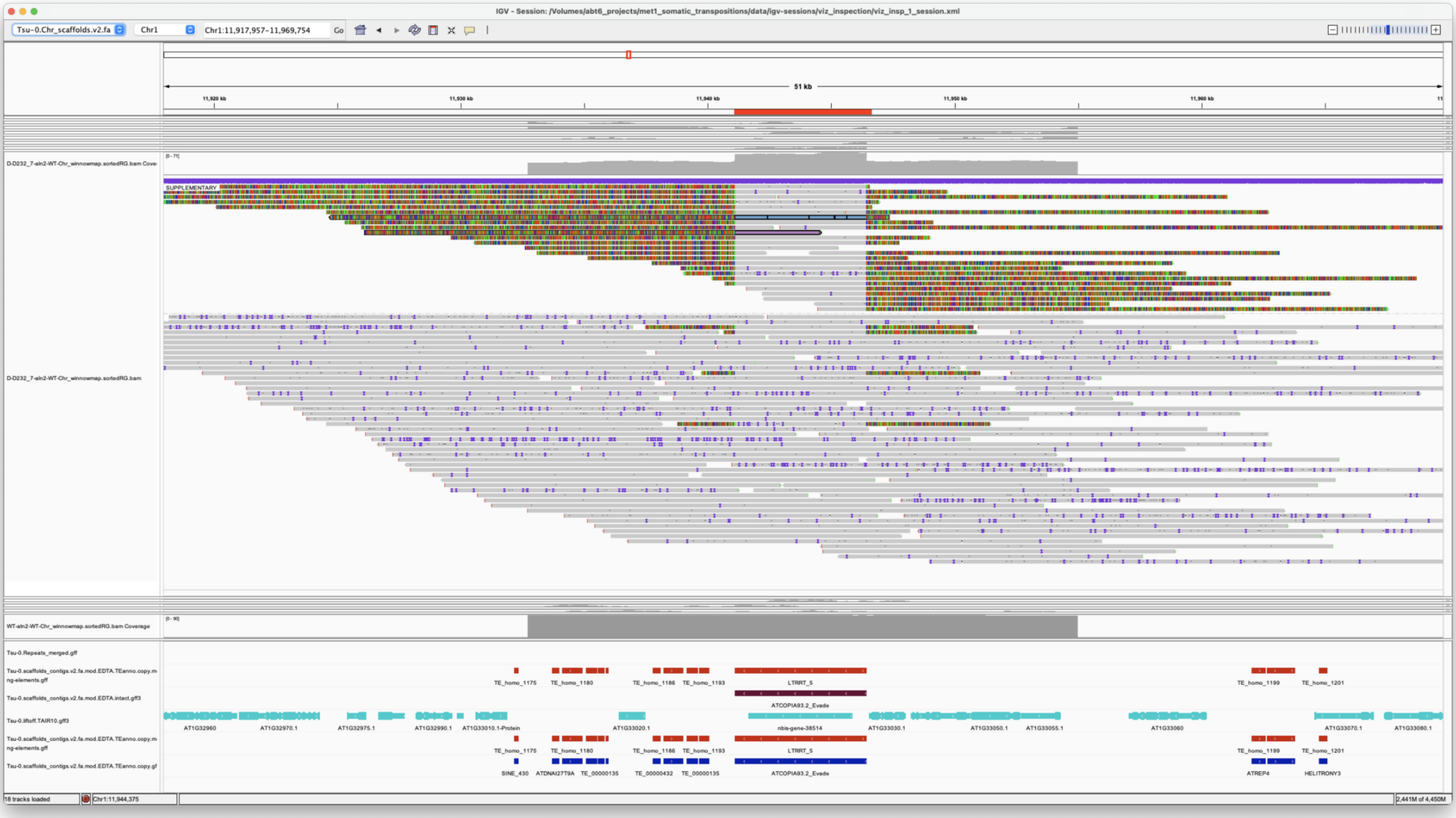

Partial

Confirmed

Chr1 30489568 30489568 + 1 Chr1;11941106;11946436;ATCOPIA93_Evade m64079_240212_113350/176161312/ccs met1_07

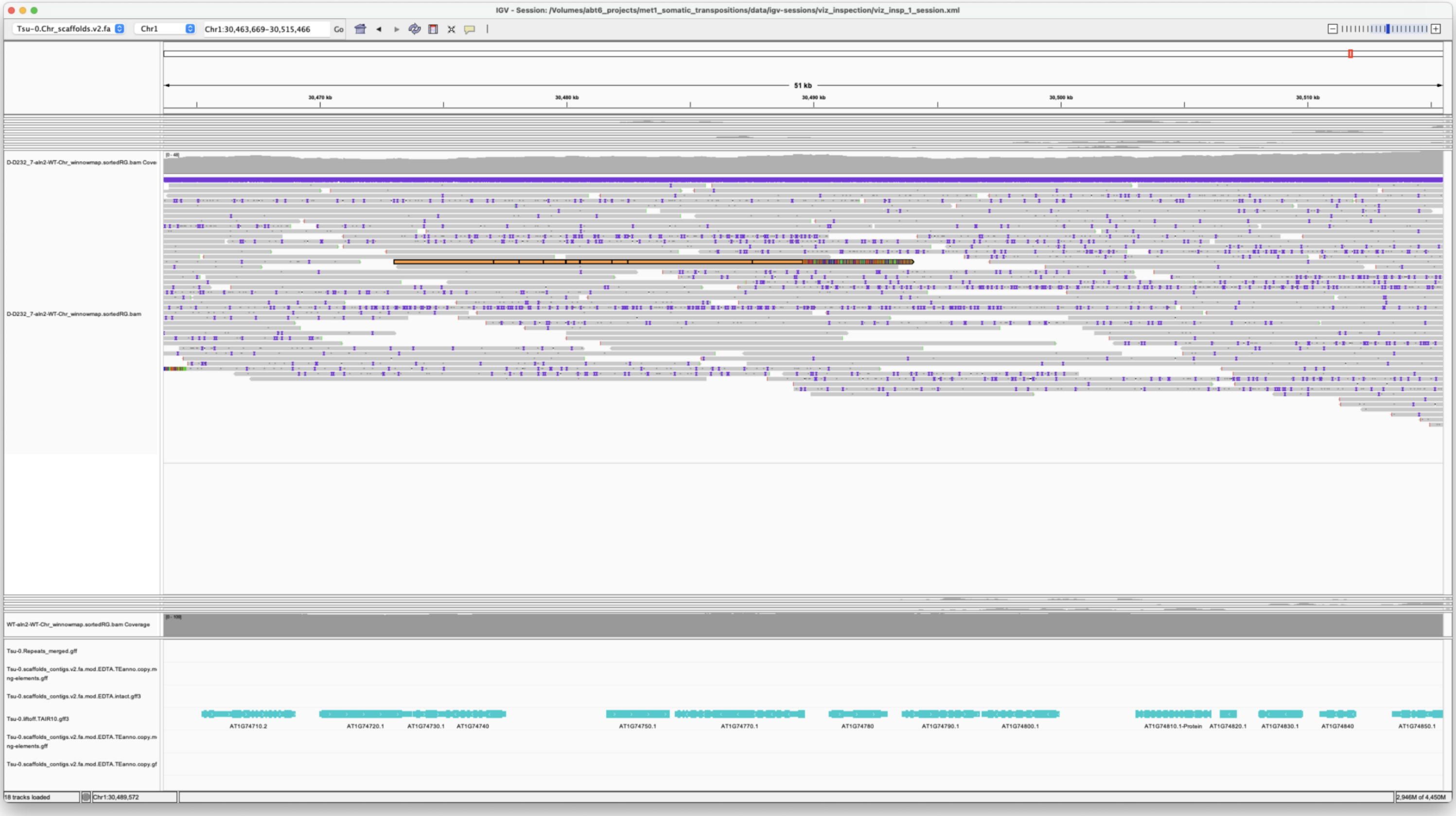

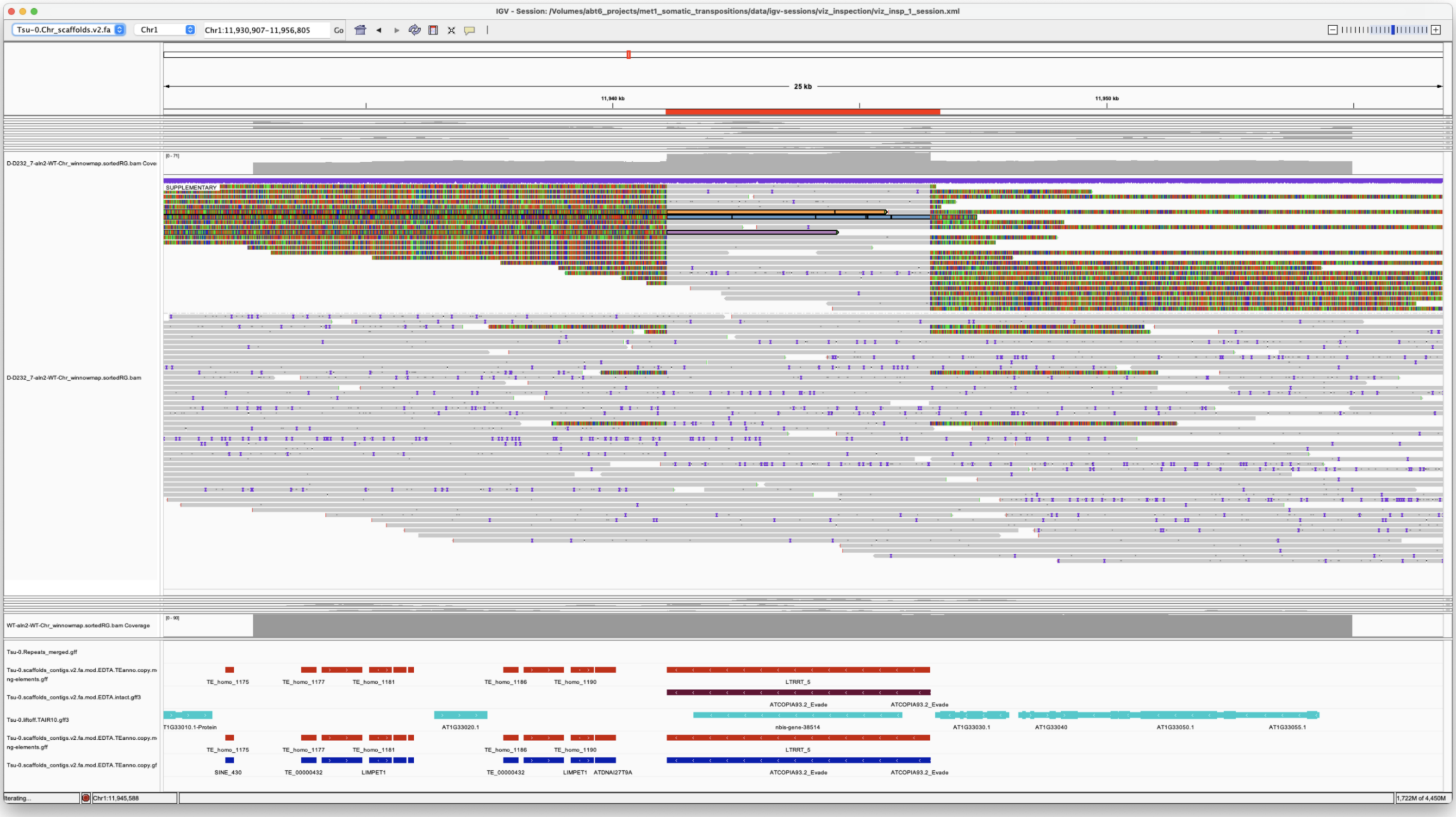

Partial

Confirmed

Chr1 32043486 32043486 + 1 Chr1;11941106;11946436;ATCOPIA93_Evade m64079_221220_112036/5572078/ccs met1_07

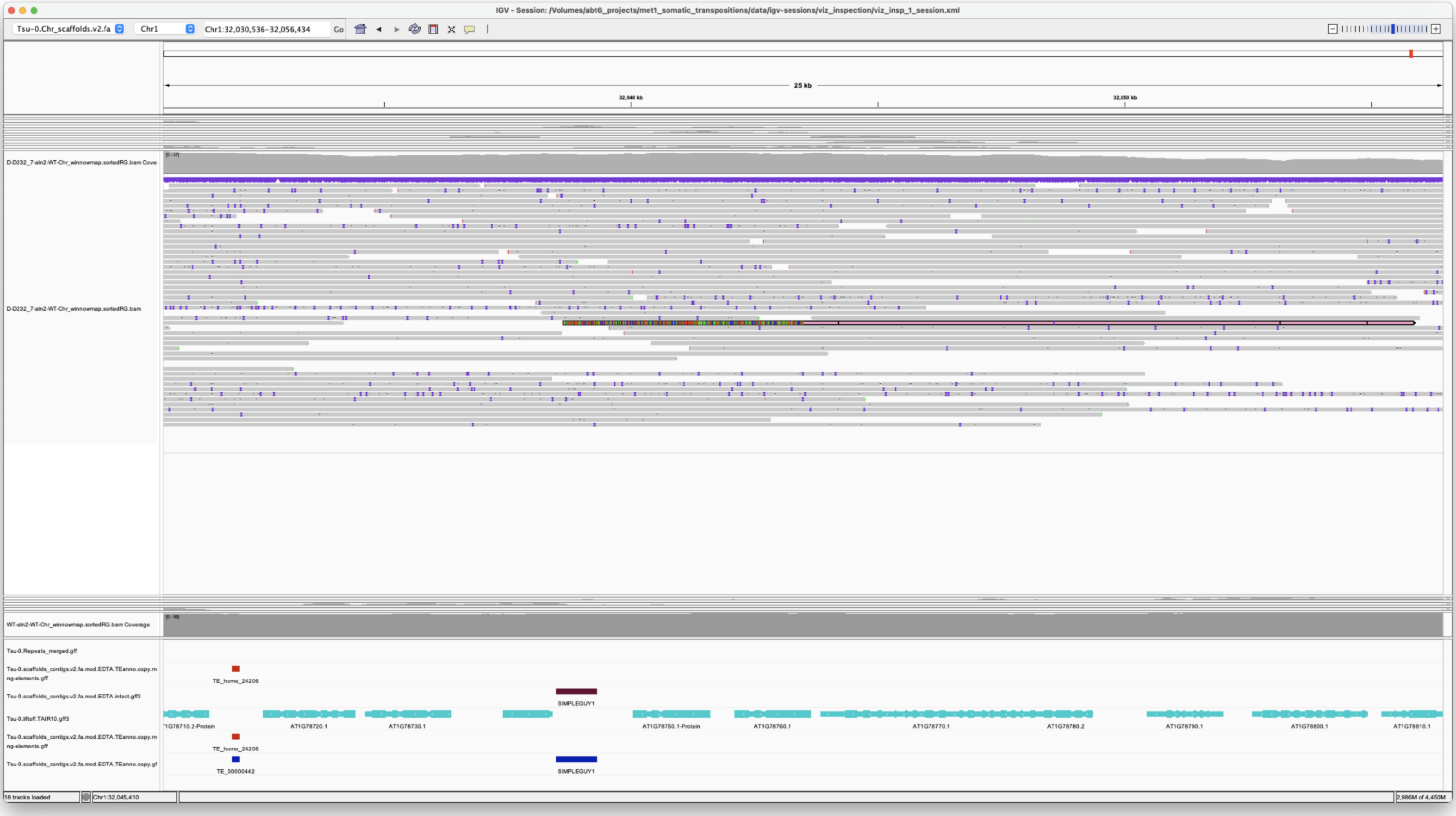

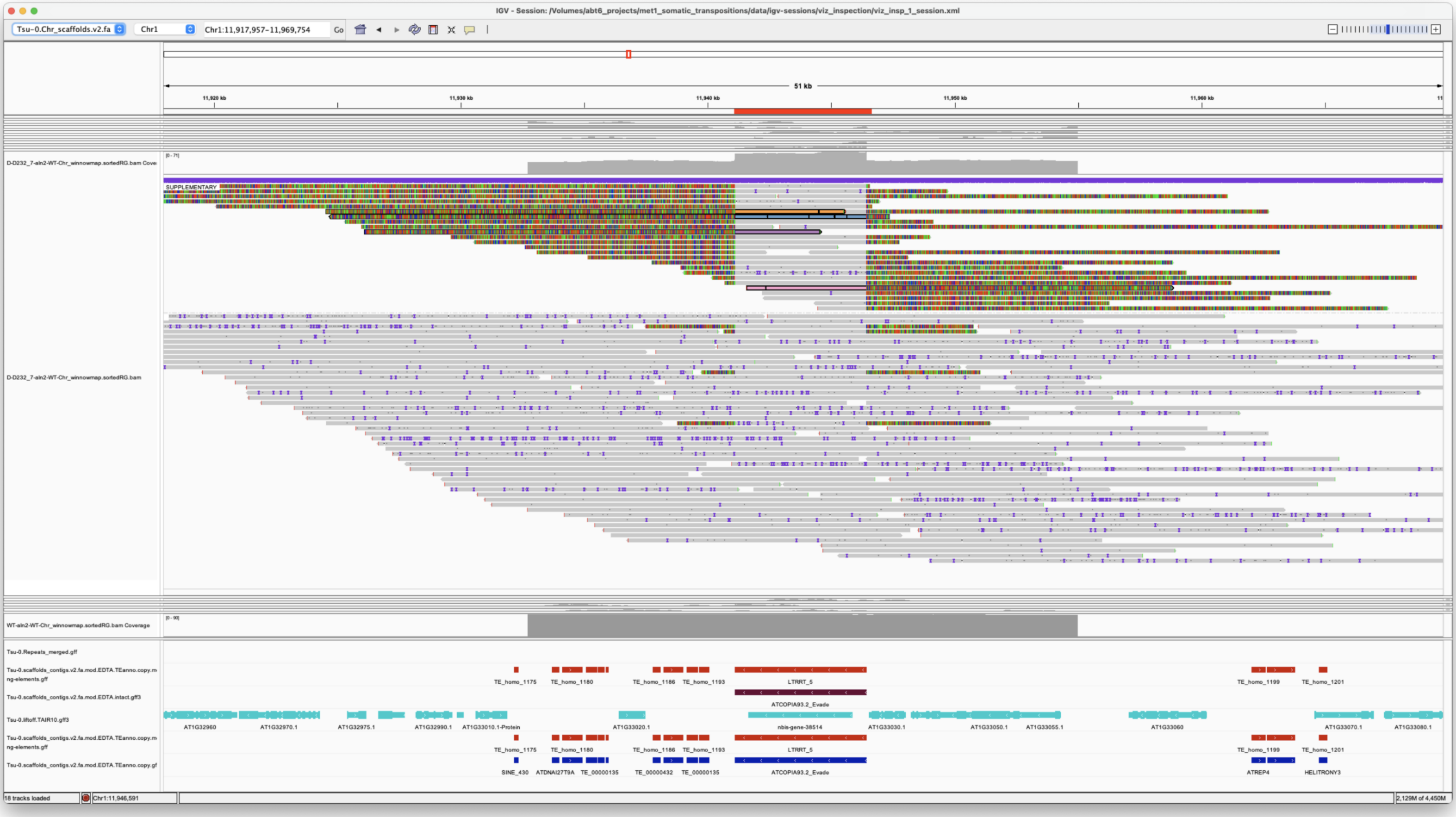

Partial

Confirmed

Chr2 6811365 6811365 - 1 Chr5;19152829;19160826;VANDAL21 m64079_221220_112036/126749221/ccs met1_07

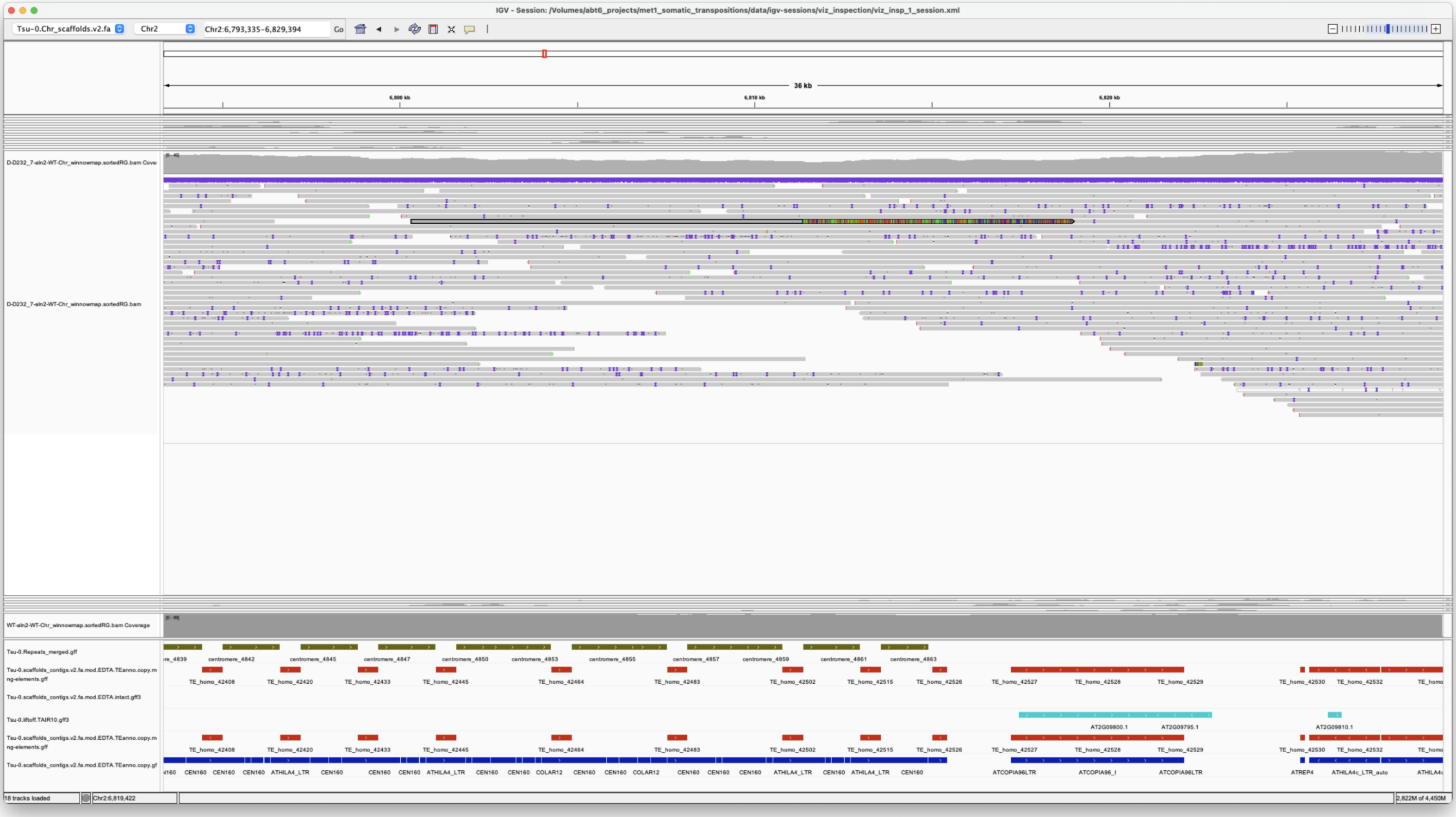

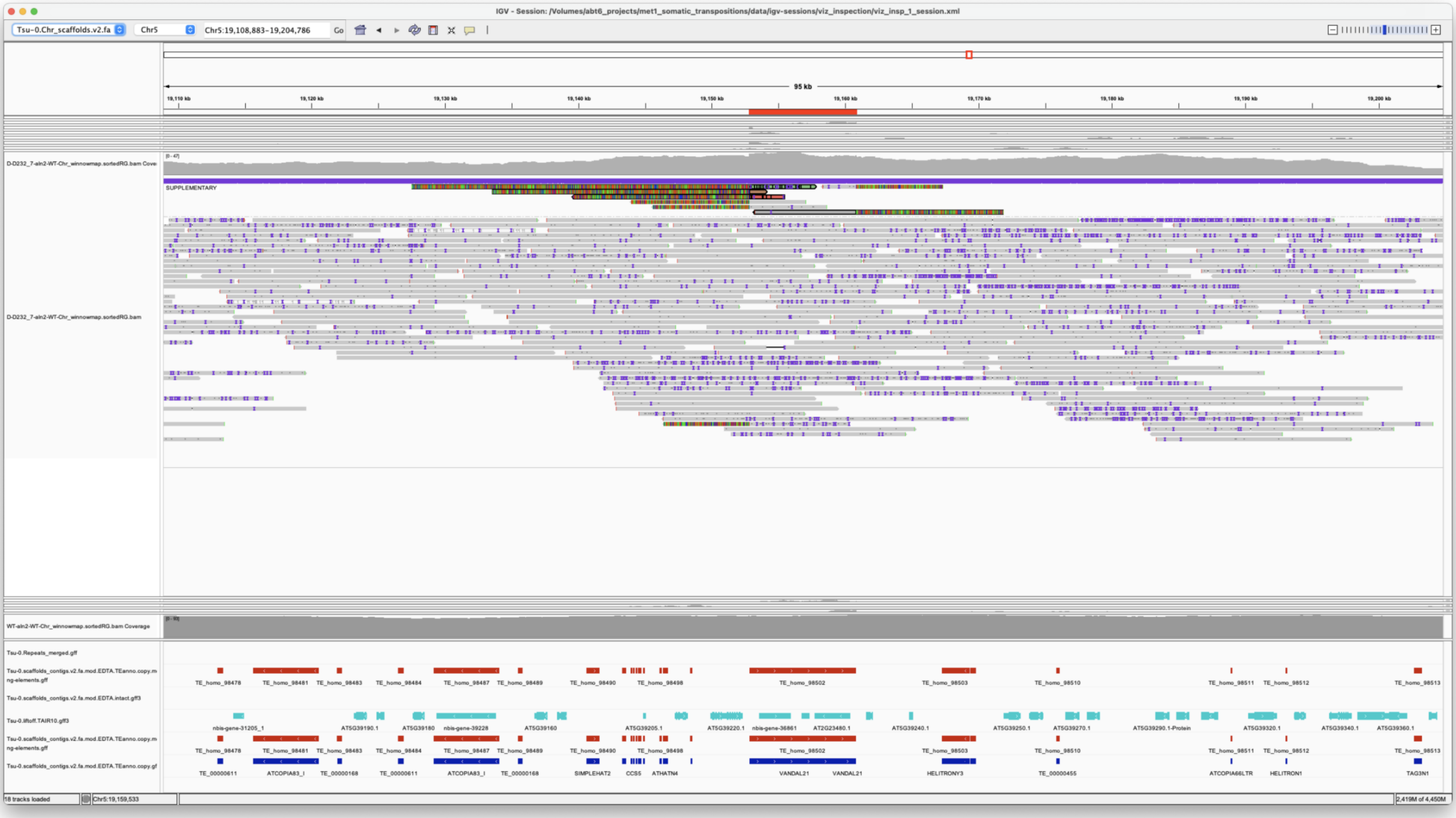

Centromeric Athila

Partial

Confirmed

Chr2 10220481 10220481 + 1 Chr5;19152829;19160826;VANDAL21 m64079_240212_113350/9570703/ccs met1_07

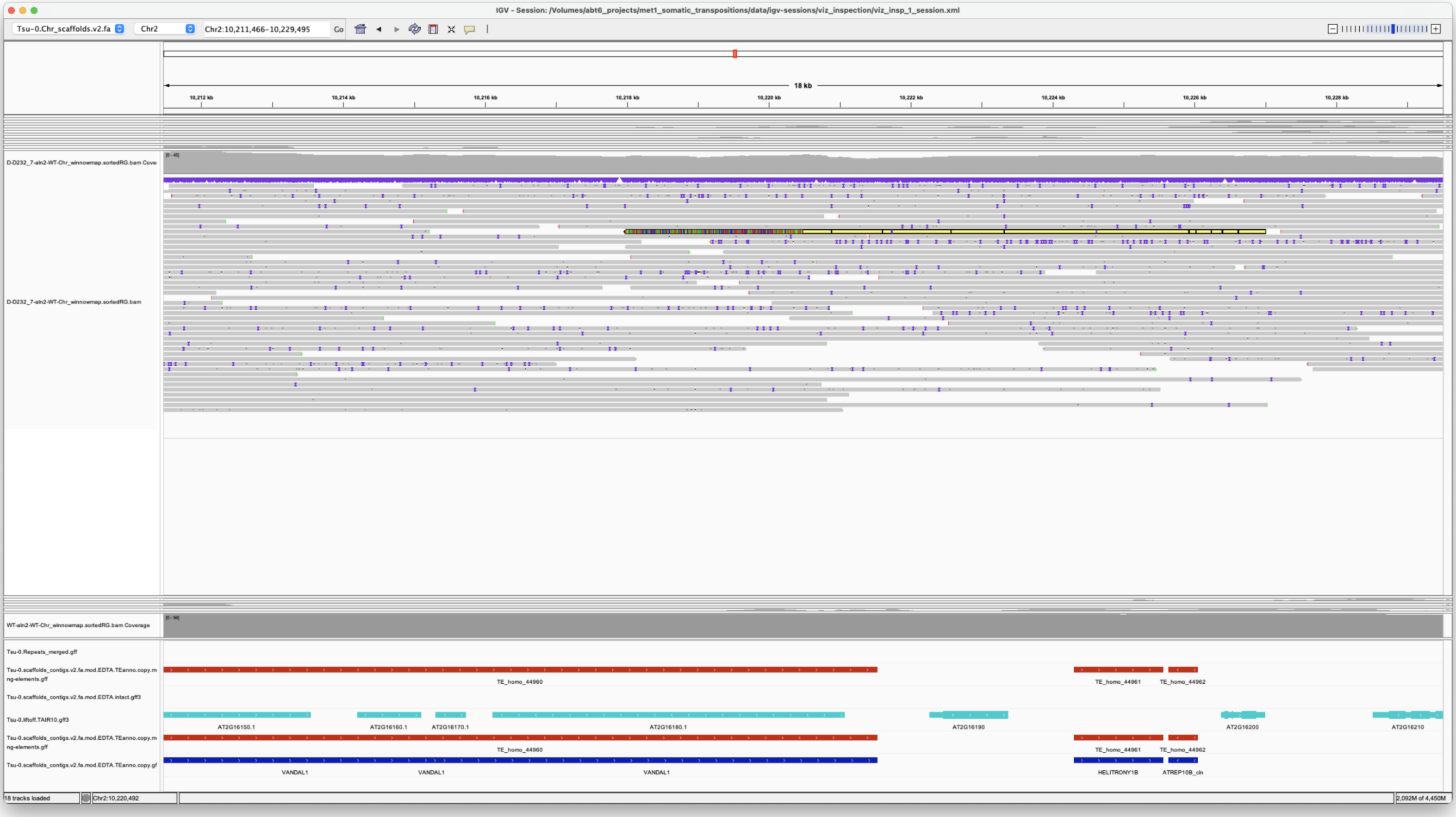

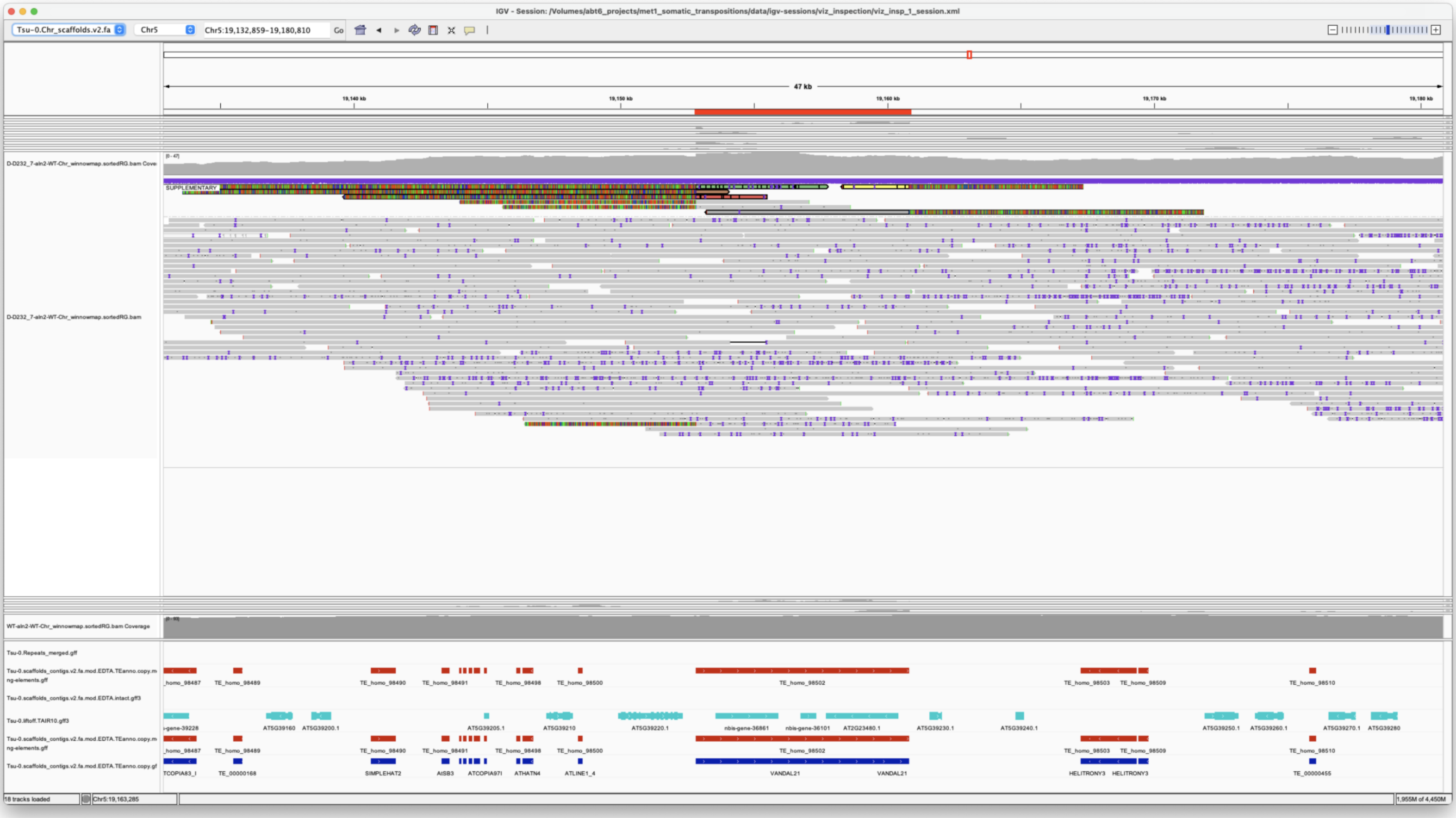

Partial

Confirmed

Chr2 13193892 13193892 + 1 Chr1;11941106;11946436;ATCOPIA93_Evade m64079_221220_112036/97584457/ccs met1_07

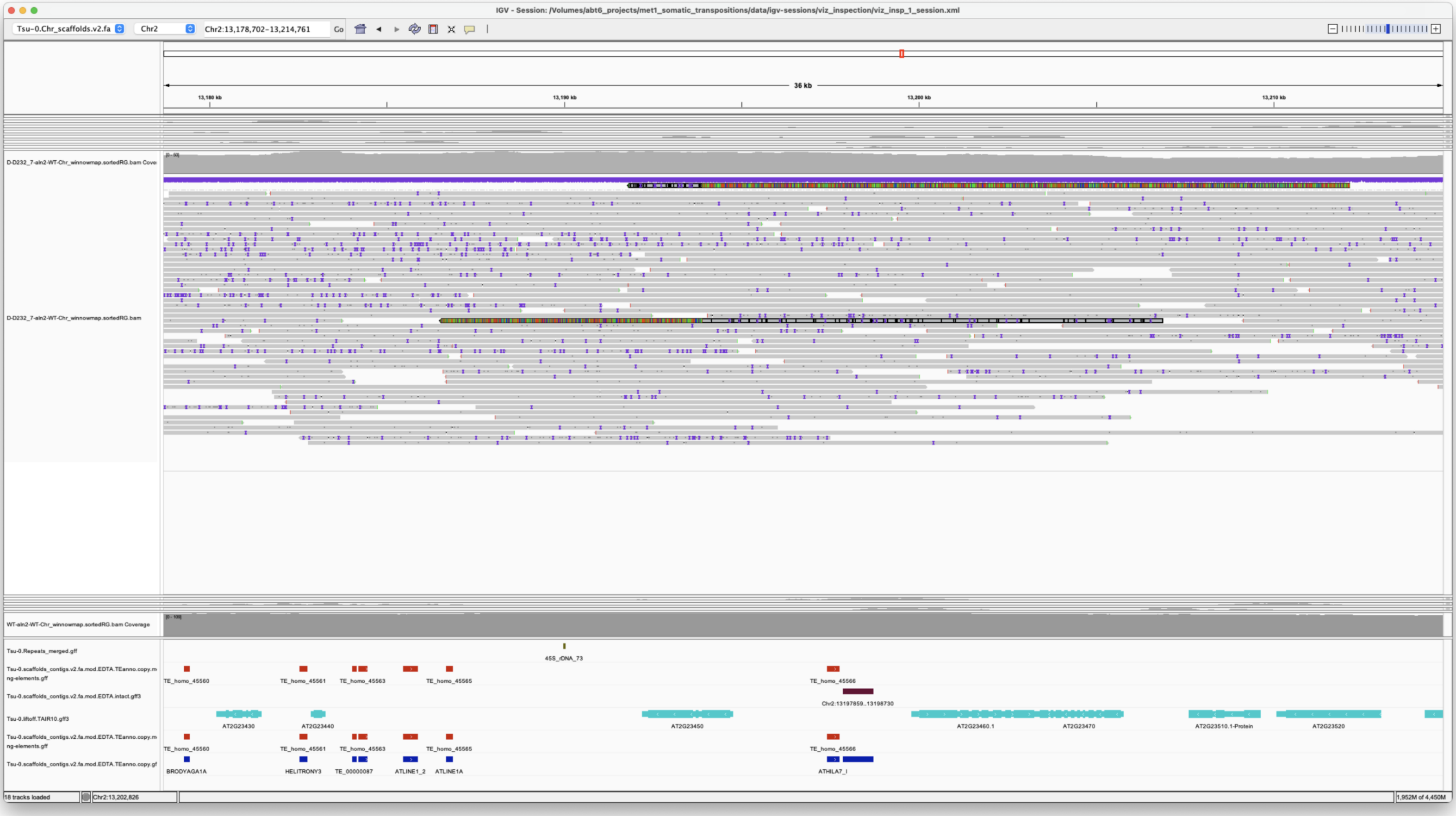

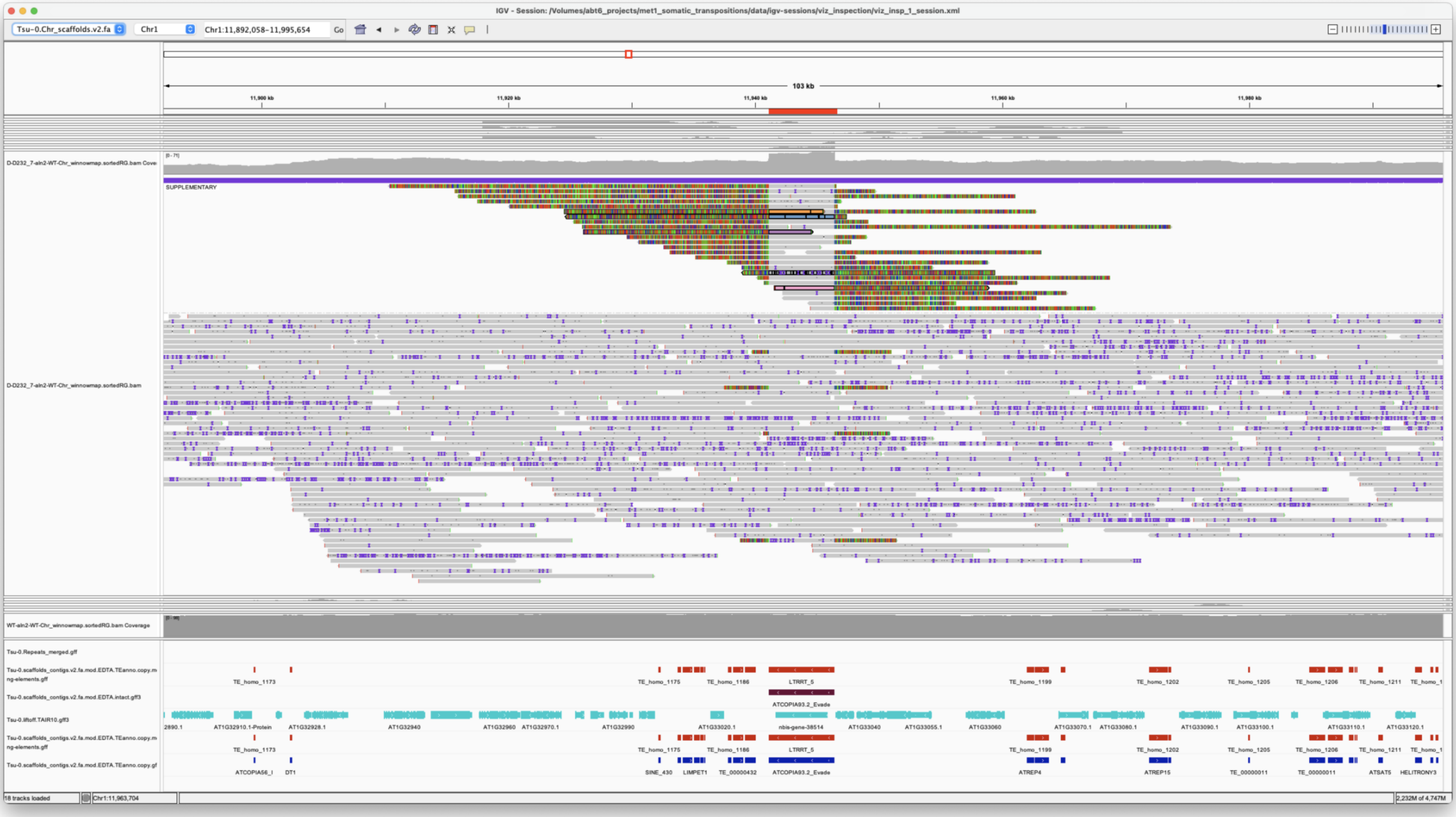

Central
TSD

Confirmed

Chr2 17563335 17563335 + 1 Chr5;21419693;21425022;ATCOPIA93_Evade m64079_221220_112036/82577985/ccs met1_07

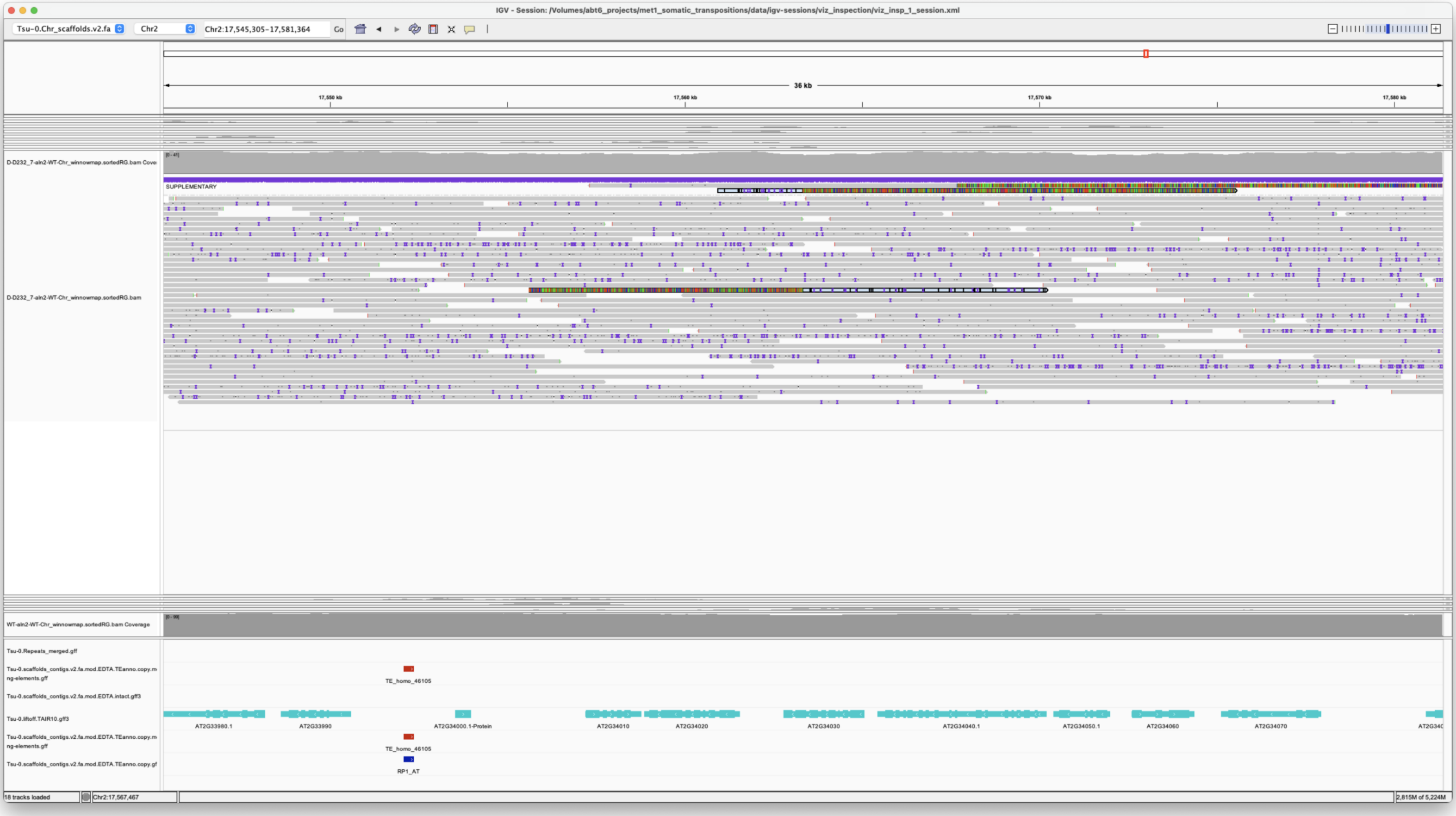

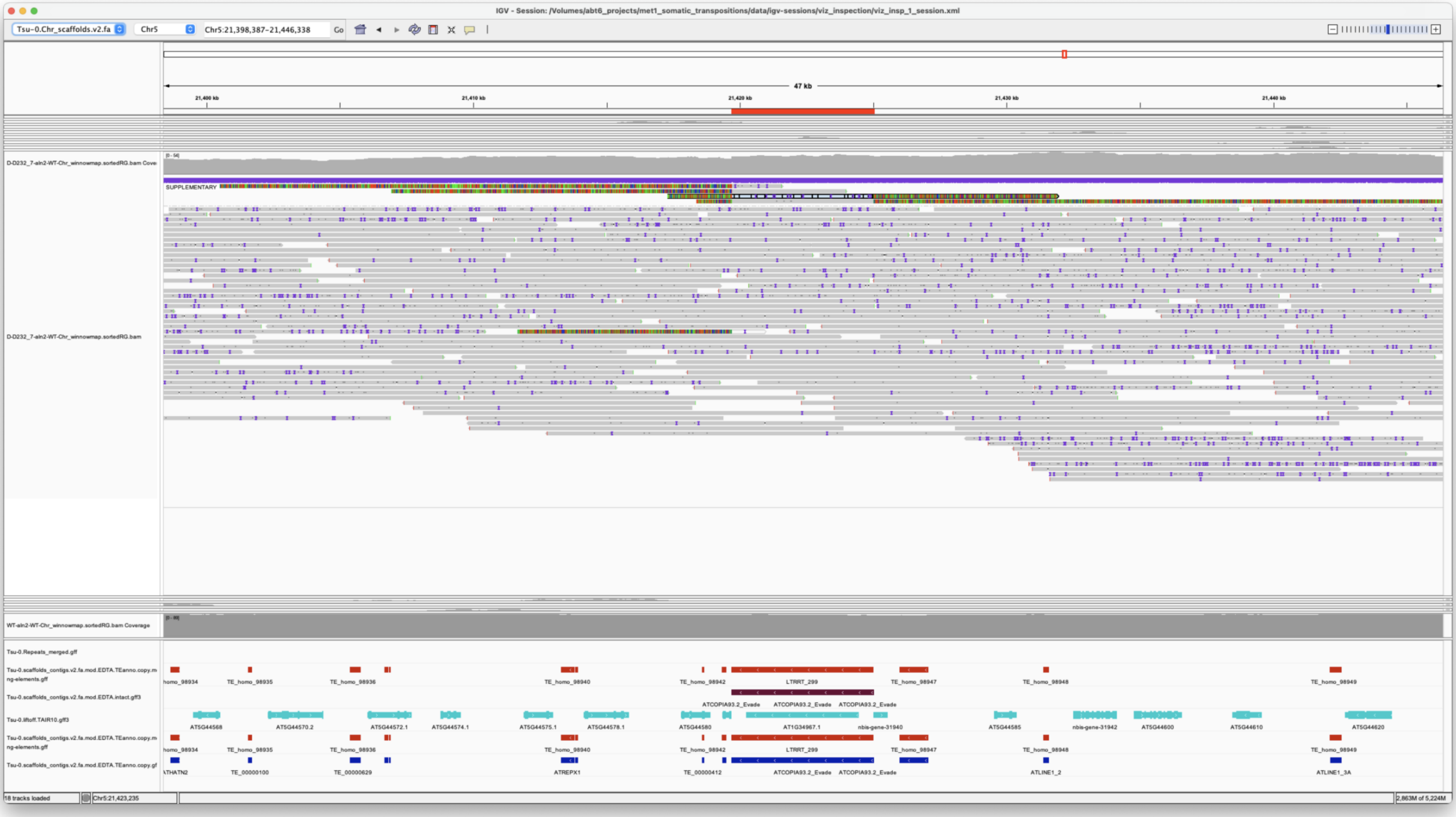

Confirmed

Chr3 2942237 2942239 +,+,+,+,+,+ 6
Chr1;11941106;11946436;ATCOPIA93_Evade,Chr1;11941106;11946436;ATCOPIA93_Evade,Chr1;11941106;11946436;ATCOPIA93_Evade,Chr1;11941106;11946436;ATCOPIA93_Evade,Chr1;11941106;11946436;ATCOPIA93_Evade,Chr1;11941106;11946436;ATCOPIA93_Evade
m64079_221220_112036/119472554/ccs,m64079_221220_112036/142740019/ccs,m64079_221220_112036/158008007/ccs,m64079_240212_113350/35980318/ccs,m64079_240212_113350/3802510/ccs,m64079_240212_113350/9242397/ccs met1_07

Chr3 2942243 2942245 +,+,+,+,+,+ 6
Chr1;11941106;11946436;ATCOPIA93_Evade,Chr1;11941106;11946436;ATCOPIA93_Evade,Chr1;11941106;11946436;ATCOPIA93_Evade,Chr1;11941106;11946436;ATCOPIA93_Evade,Chr1;11941106;11946436;ATCOPIA93_Evade,Chr1;11941106;11946436;ATCOPIA93_Evade
m64079_221220_112036/156566758/ccs,m64079_221220_112036/35457398/ccs,m64079_221220_112036/68487307/ccs,m64079_240212_113350/15991896/ccs,m64079_240212_113350/49021176/ccs,m64079_240212_113350/70844841/ccs met1_07

m64079_221220_112036/119472554/ccs

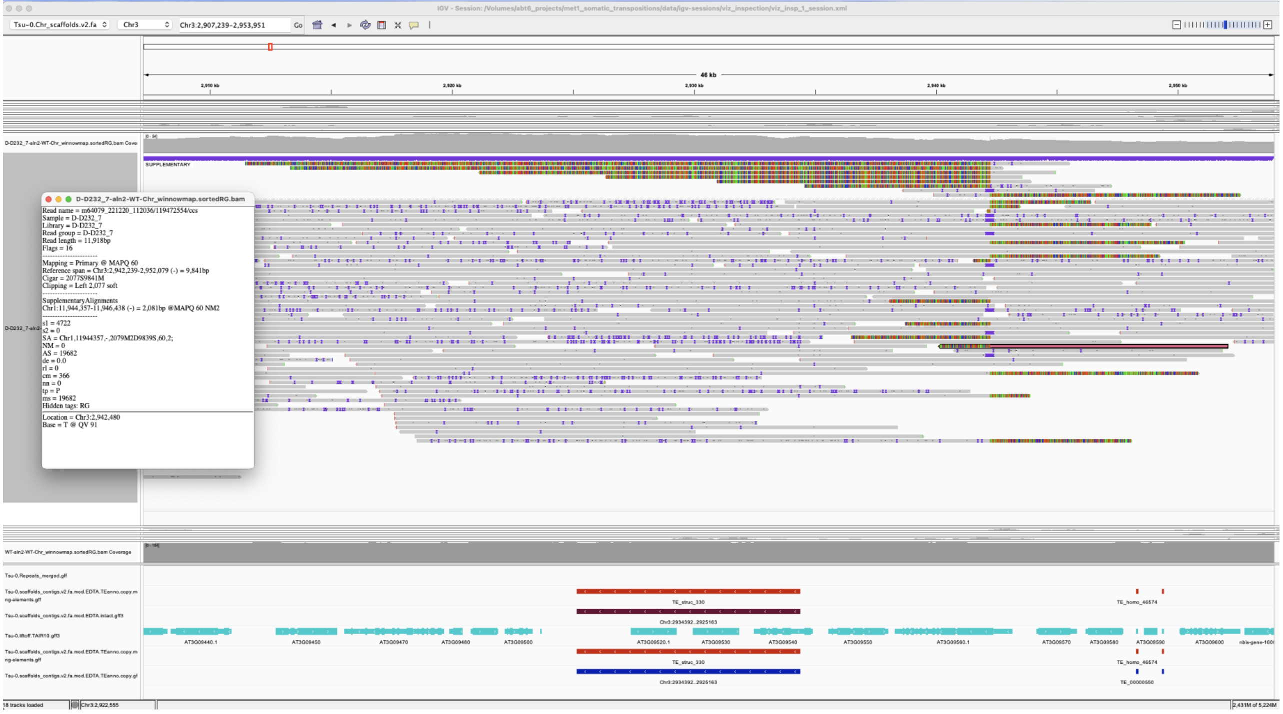

m64079_221220_112036/142740019/ccs

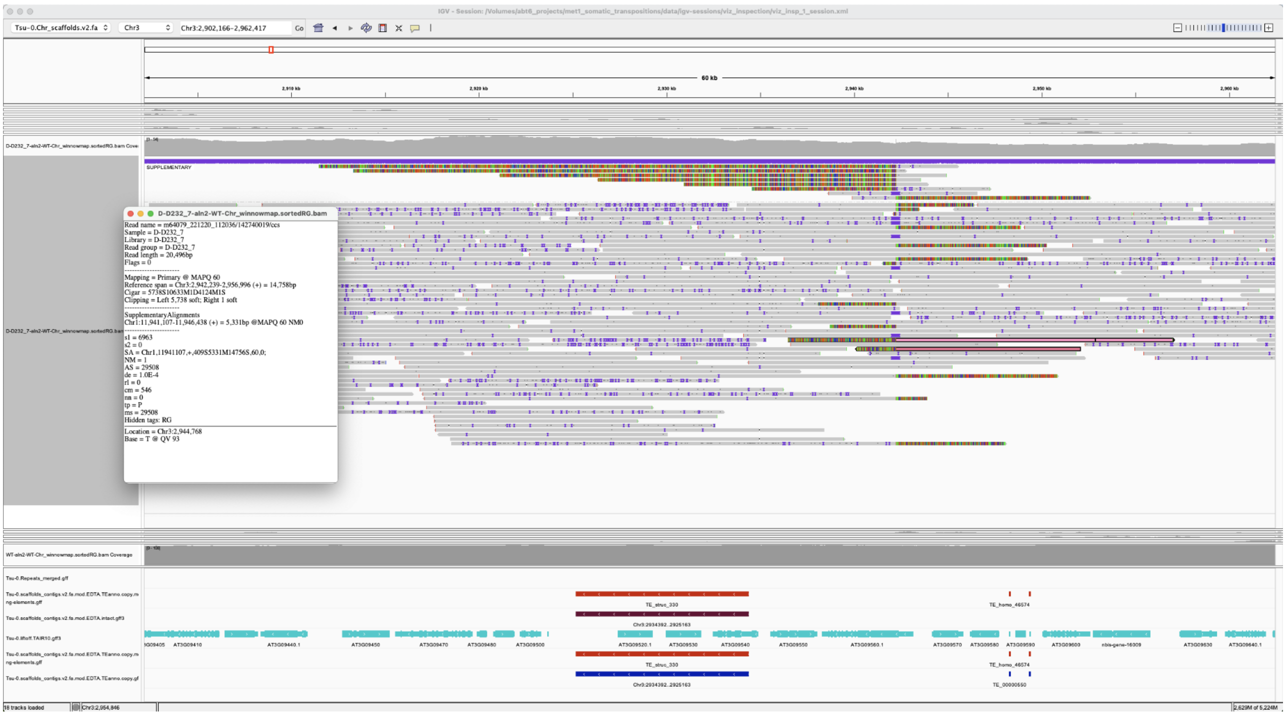

m64079_221220_112036/158008007/ccs

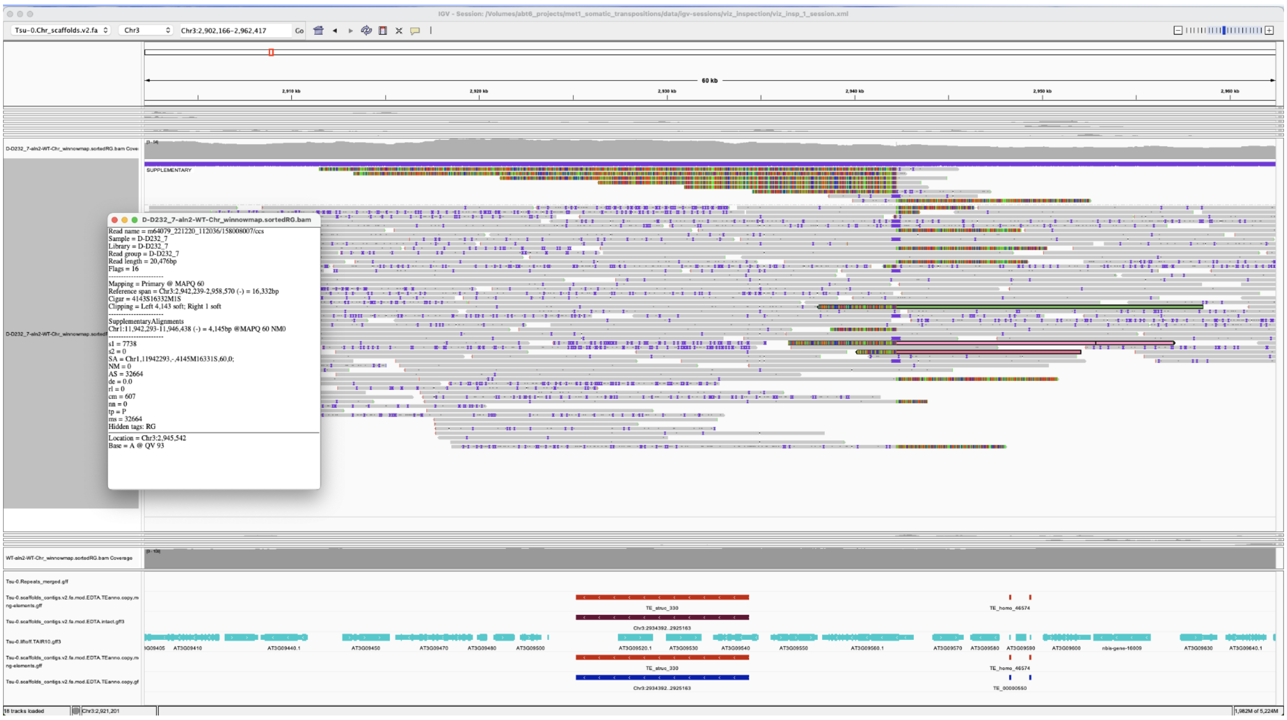

m64079_240212_113350/35980318/ccs

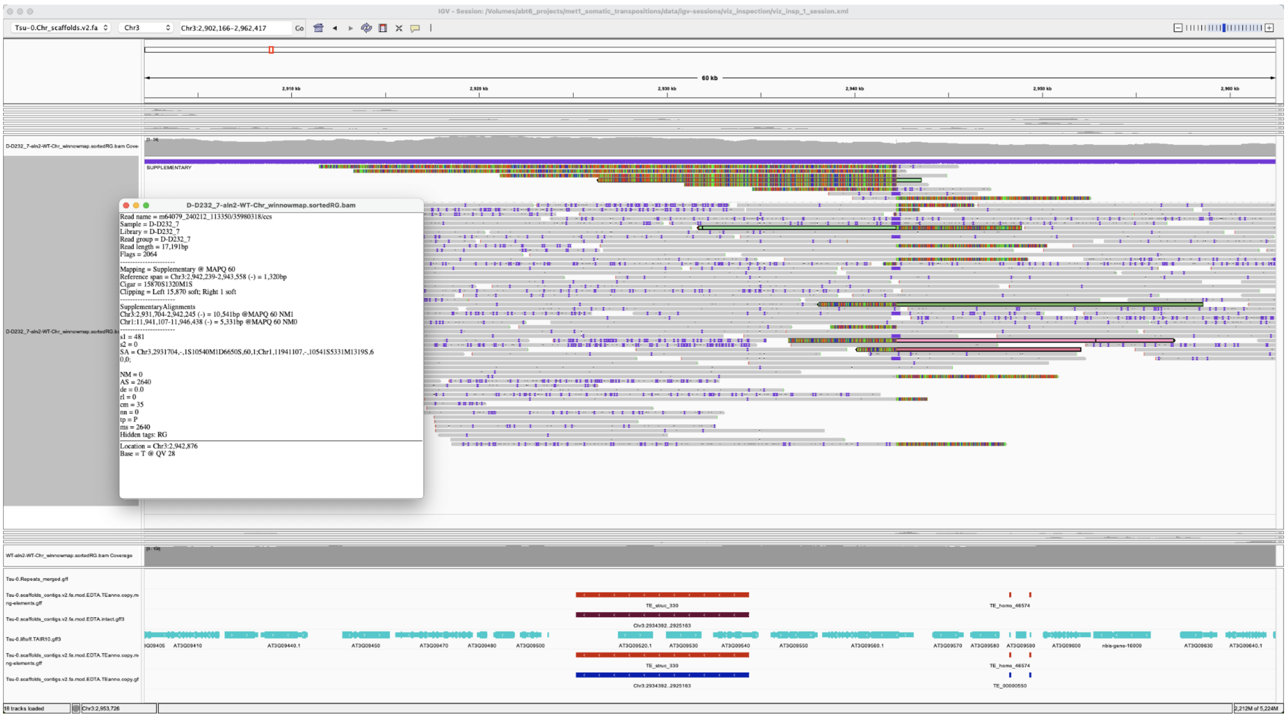

m64079_240212_113350/3802510/ccs

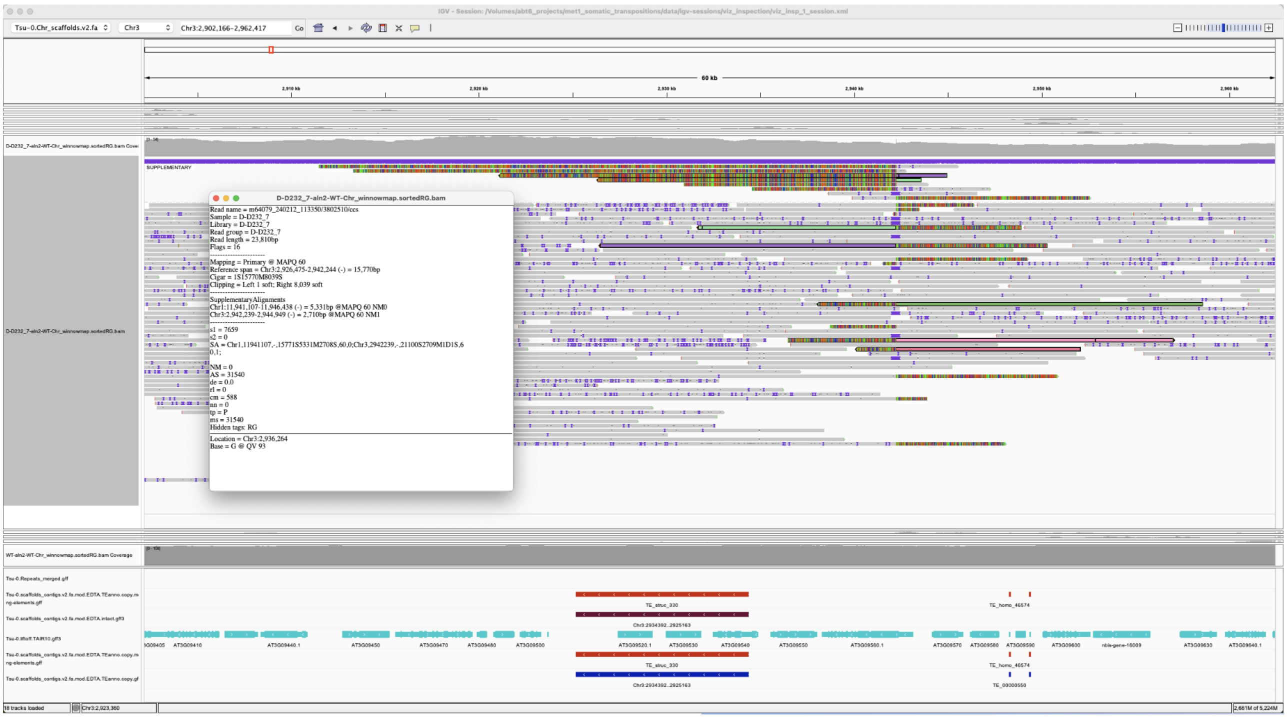

m64079_240212_113350/9242397/ccs

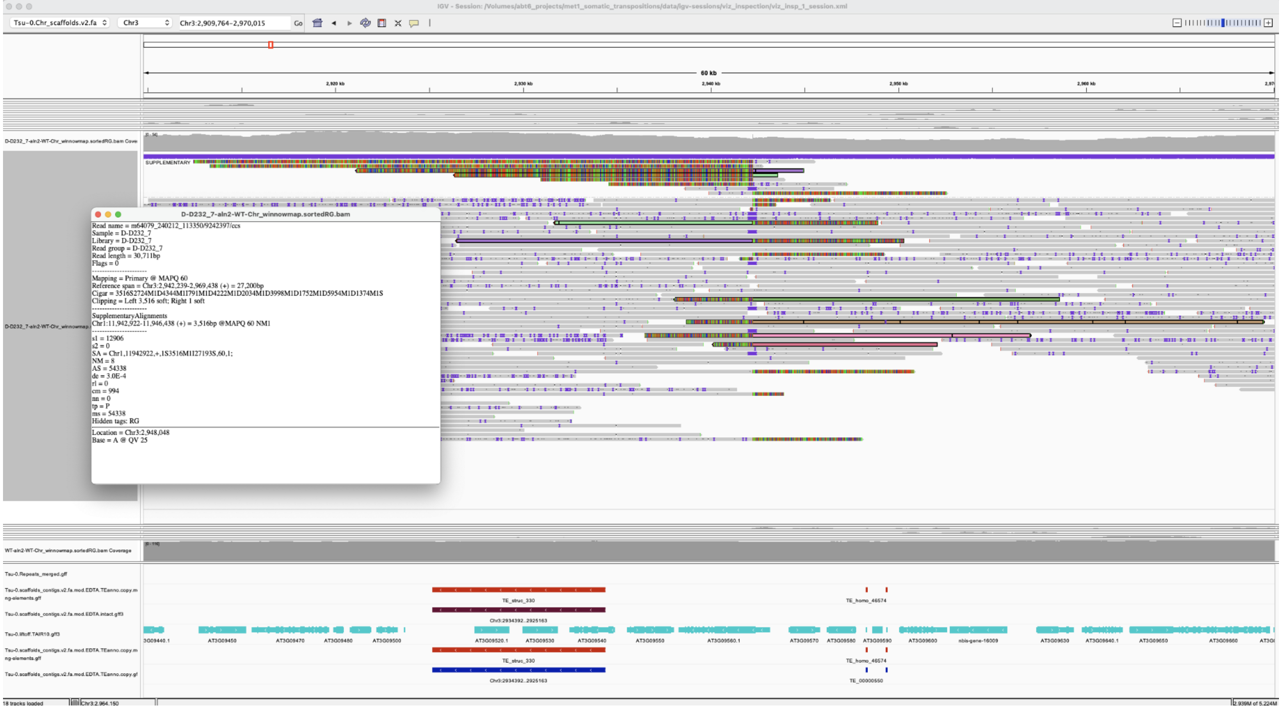

m64079_221220_112036/156566758/ccs

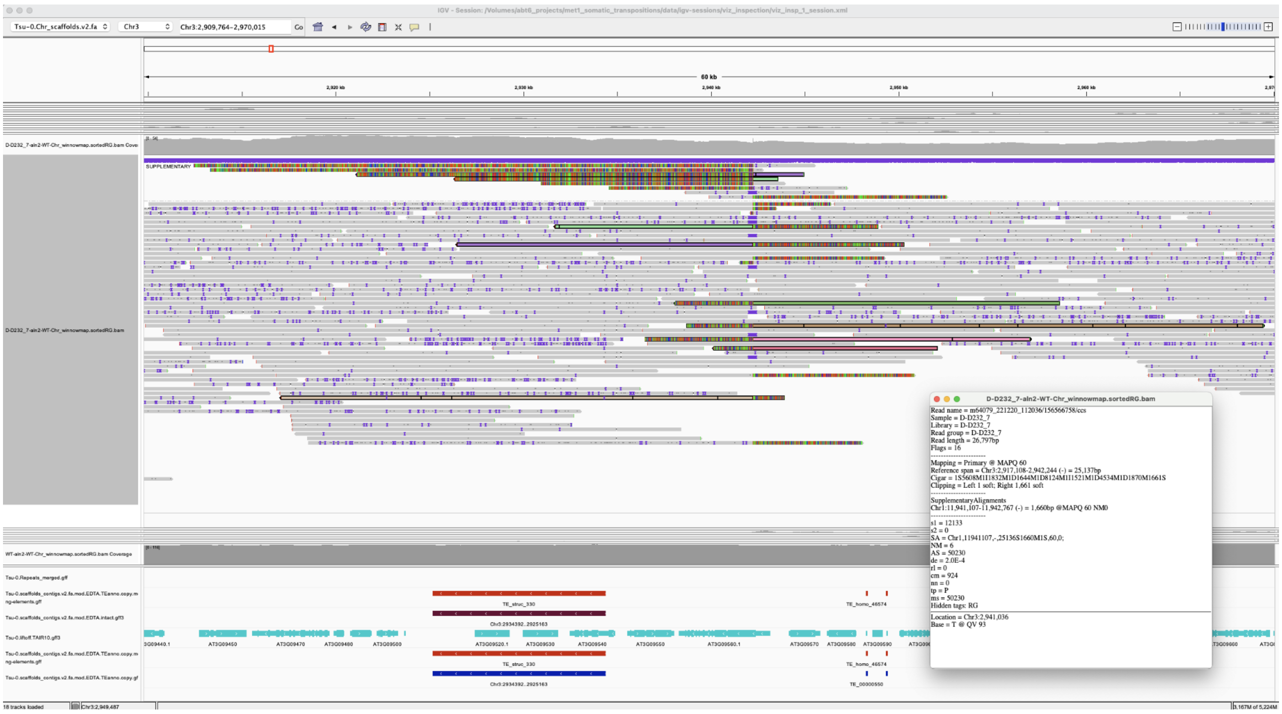

m64079_221220_112036/35457398/ccs

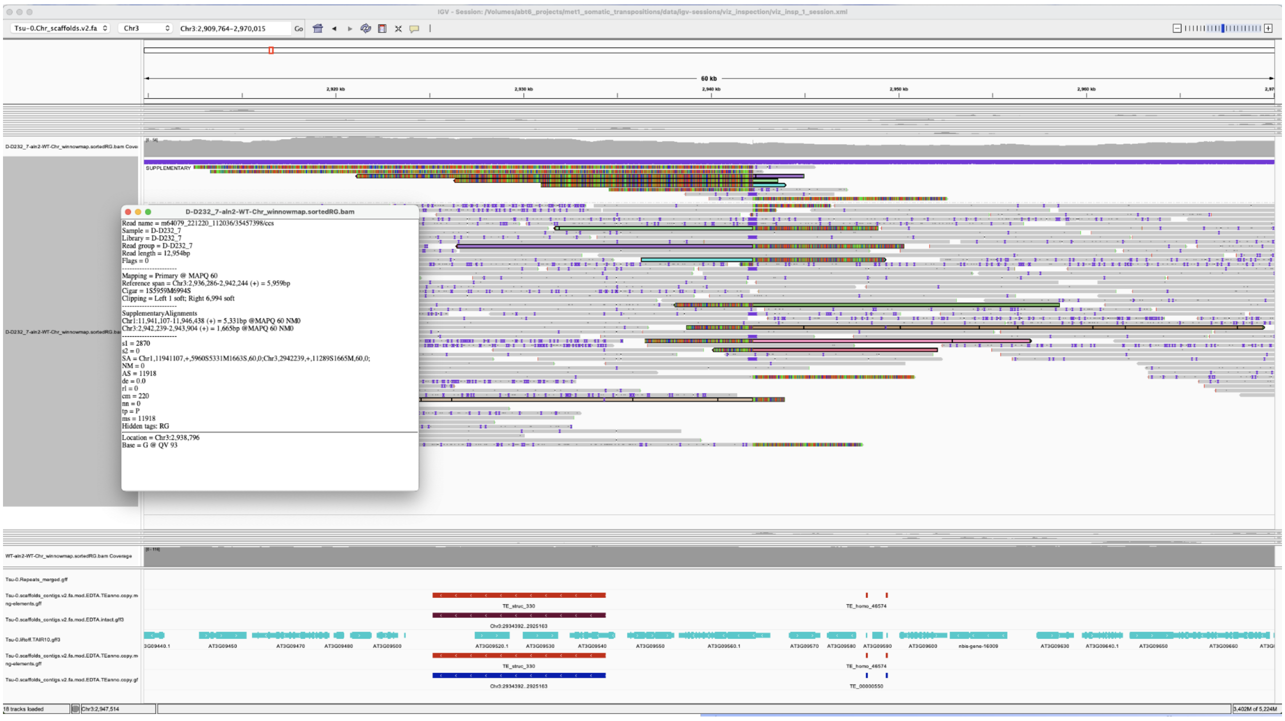

m64079_221220_112036/68487307/ccs

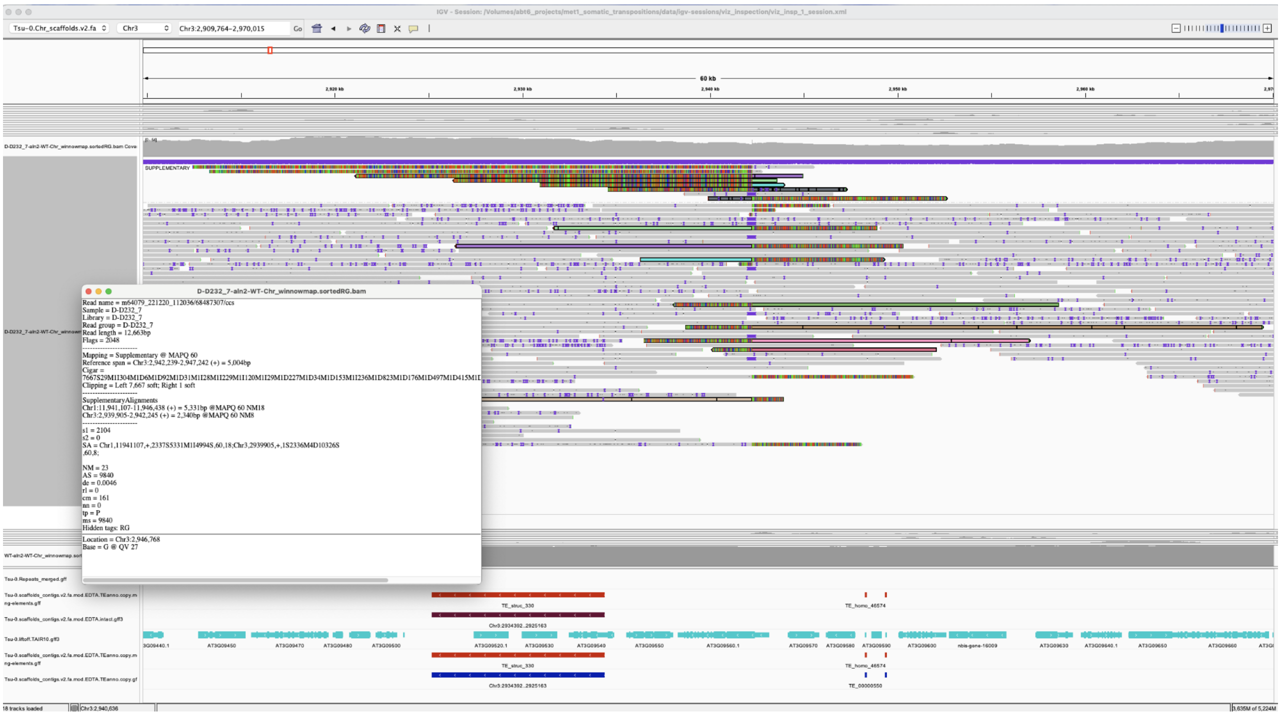

m64079_240212_113350/15991896/ccs

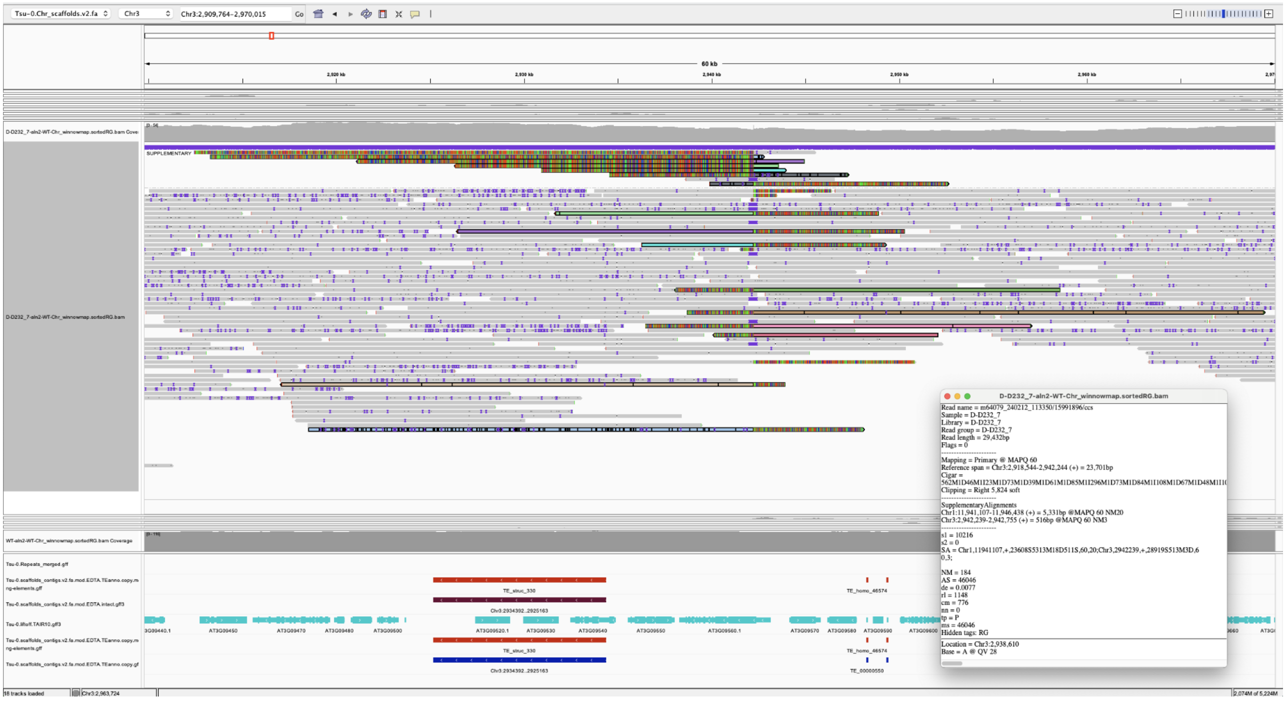

m64079_240212_113350/49021176/ccs

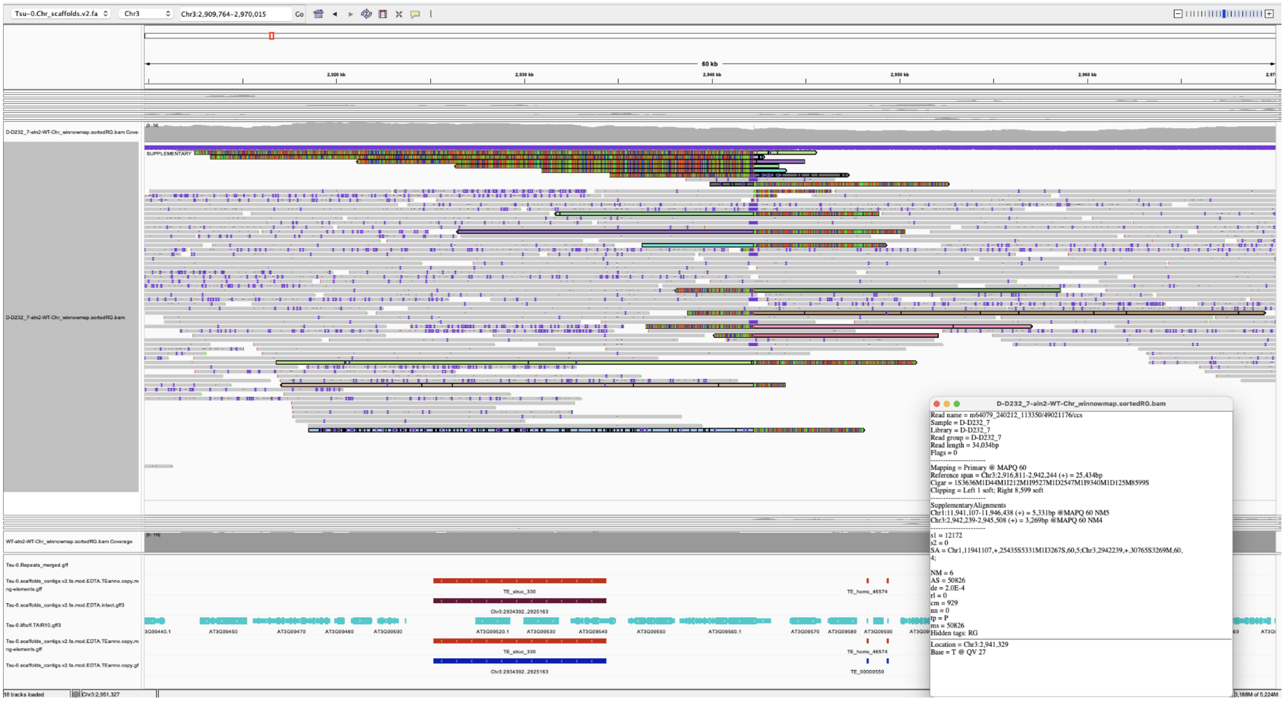

m64079_240212_113350/70844841/ccs

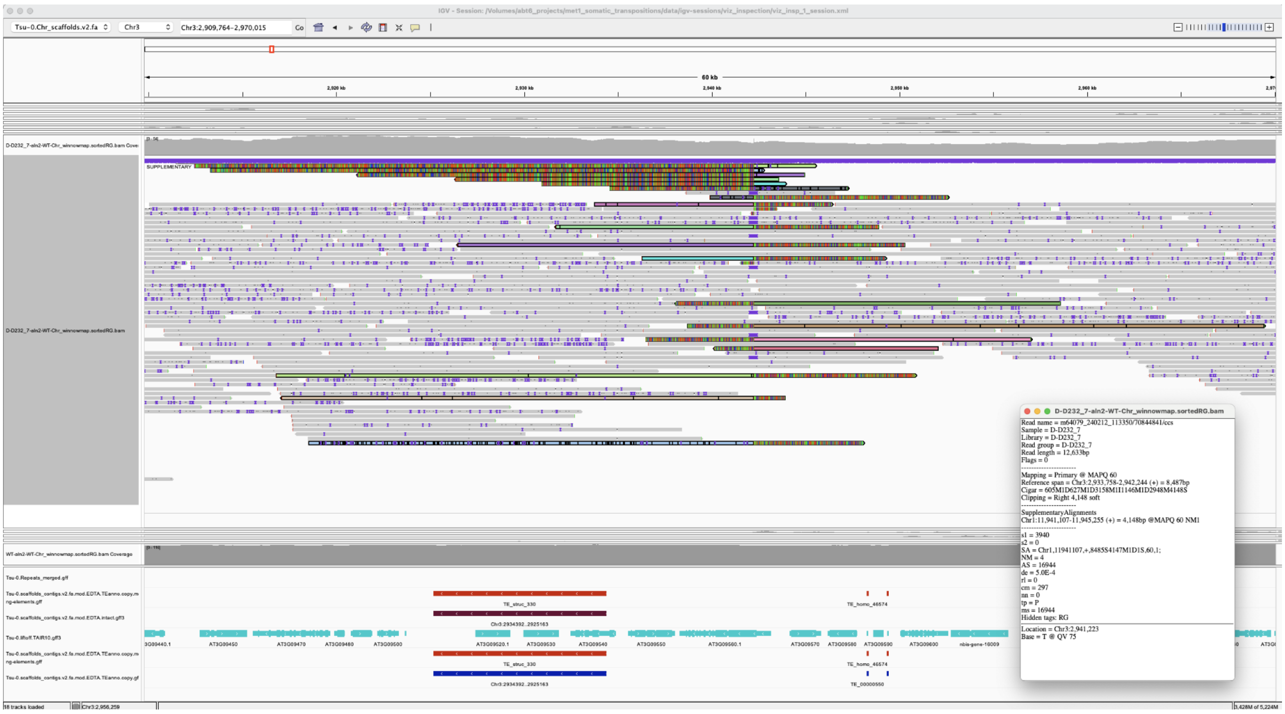

Additional 11 reads

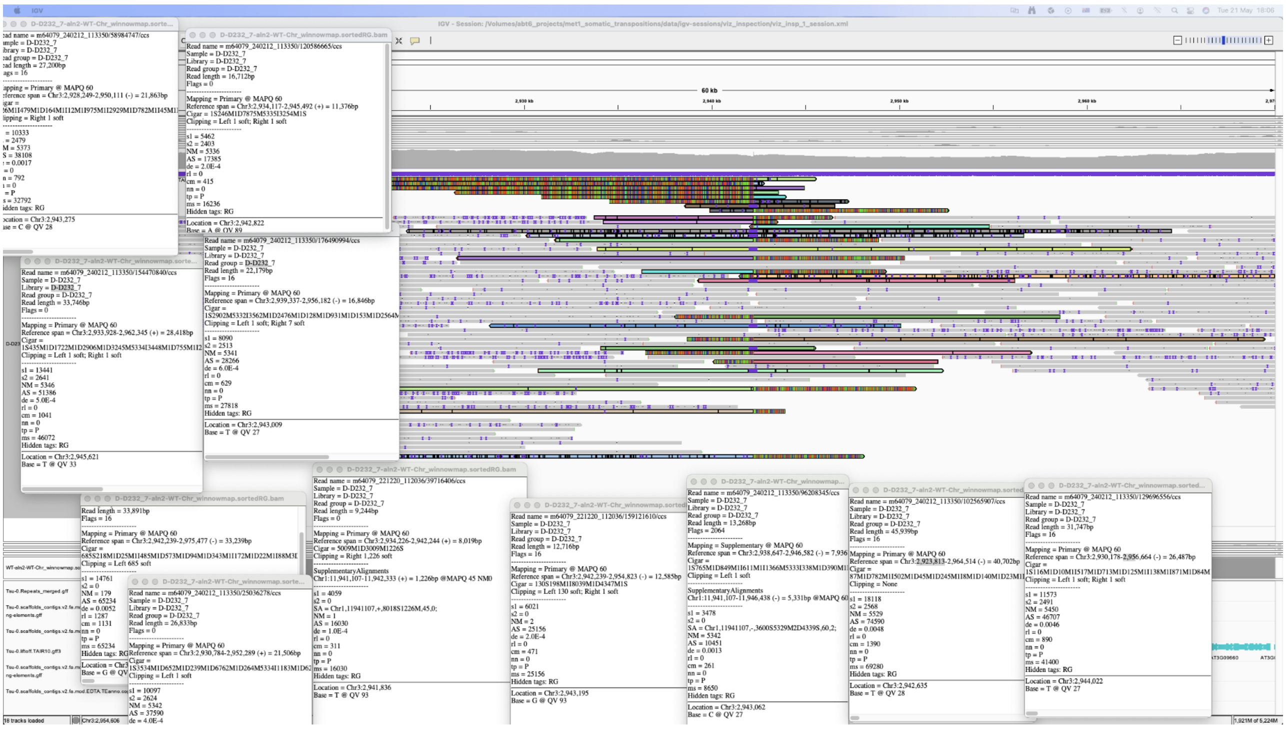

Mixed

Confirmed

Chr3 5310087 5310087 - 1 Chr1;11941106;11946436;ATCOPIA93_Evade m64079_221220_112036/157614992/ccs met1_07

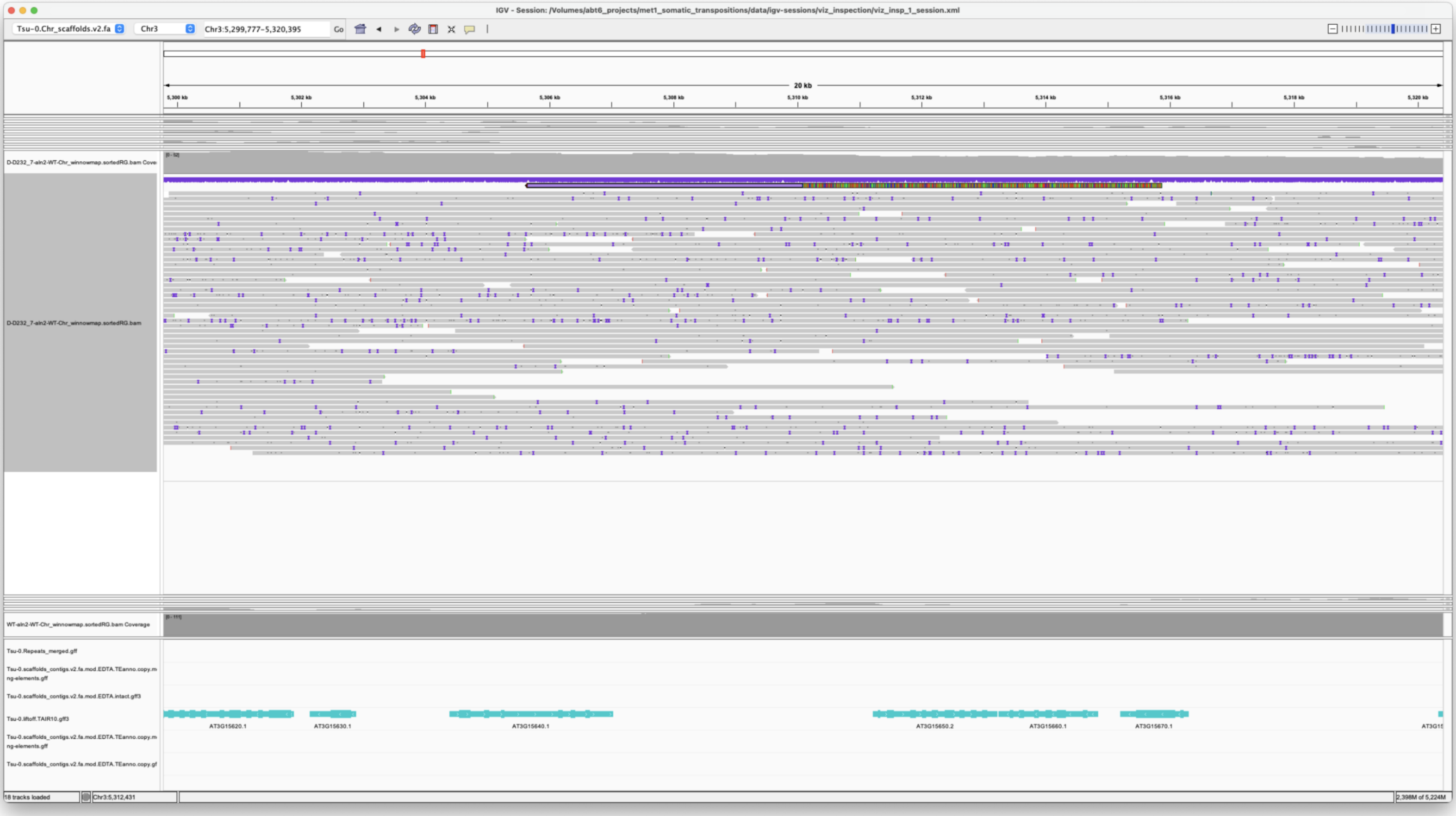

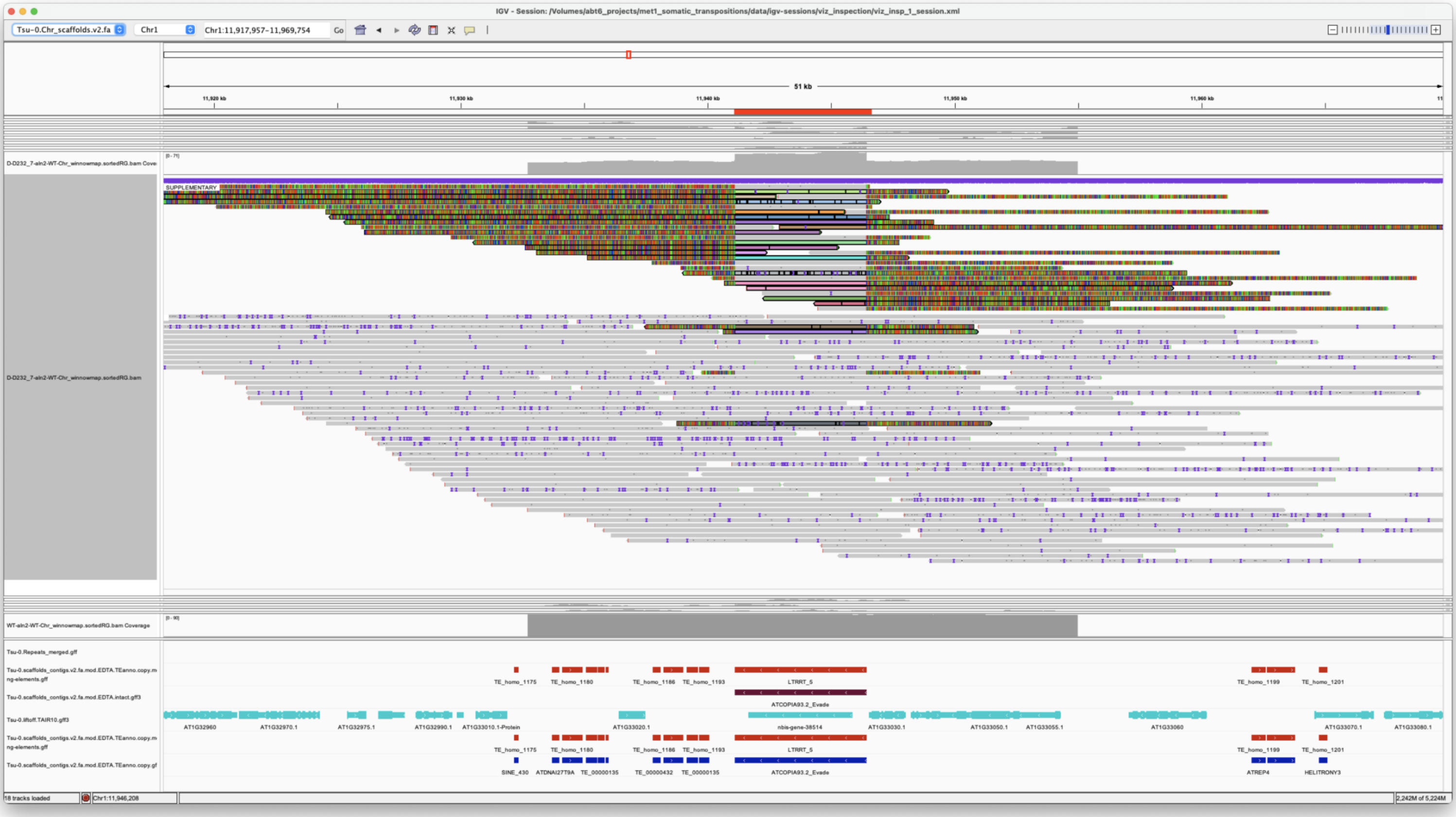

Partial

Confirmed

> Chr3 8404326 8404326 + 1 Chr1;11941106;11946436;ATCOPIA93_Evade m64079_221220_112036/121963960/ccs met1_07

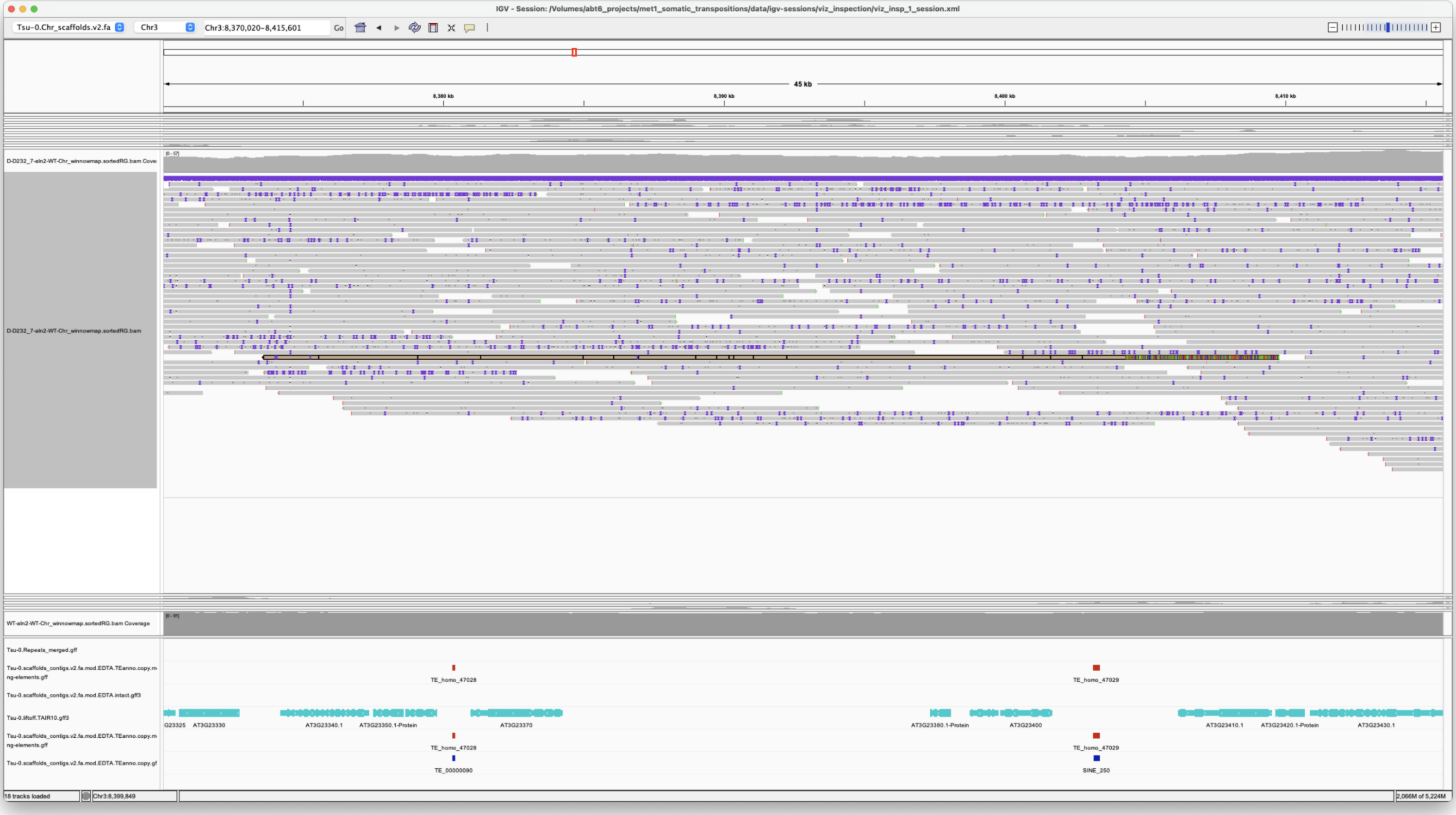

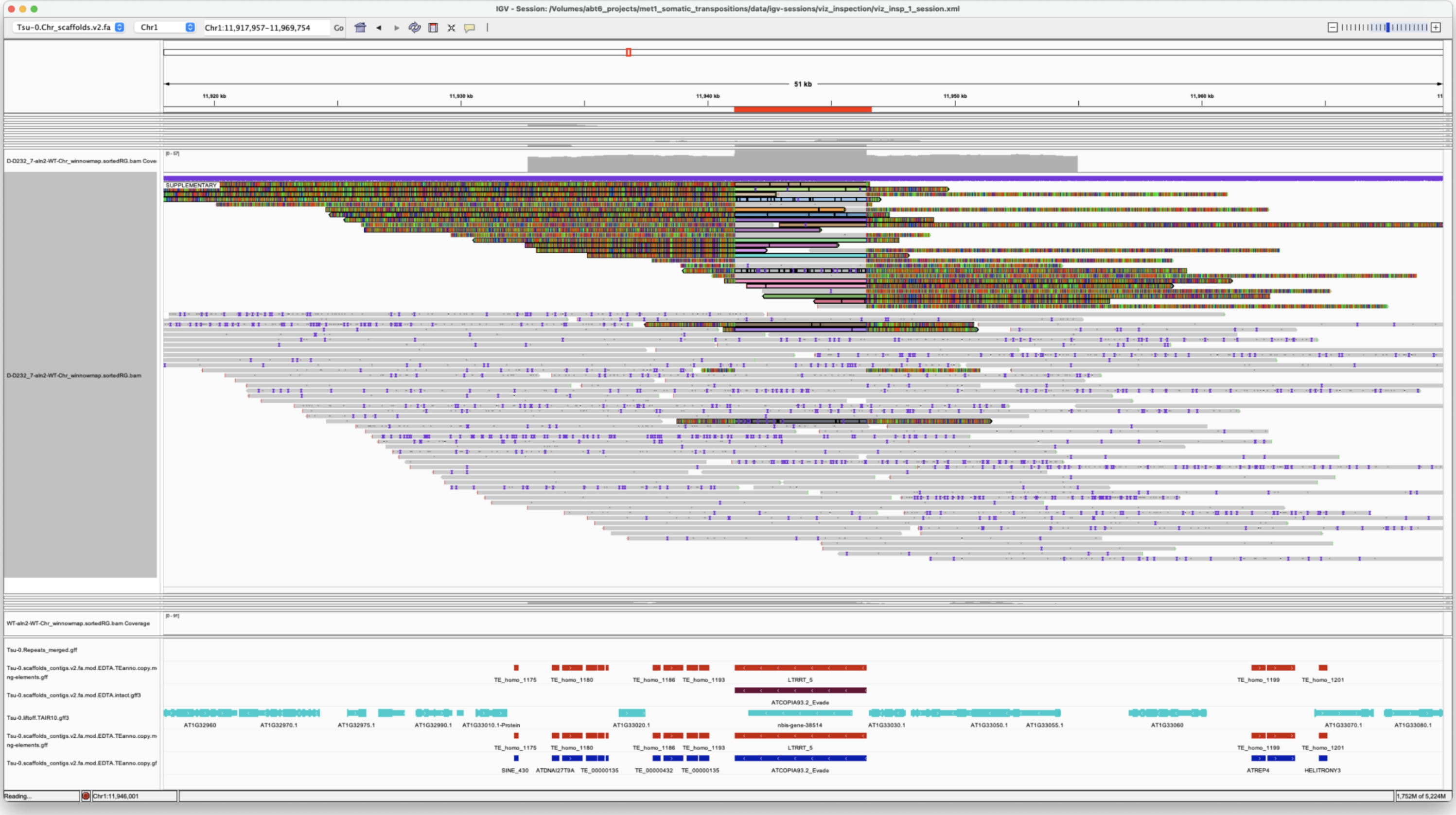

Partial

Confirmed

Chr3 9895841 9895841 + 1 Chr5;21419693;21425022;ATCOPIA93_Evade m64079_221220_112036/47319481/ccs met1_07

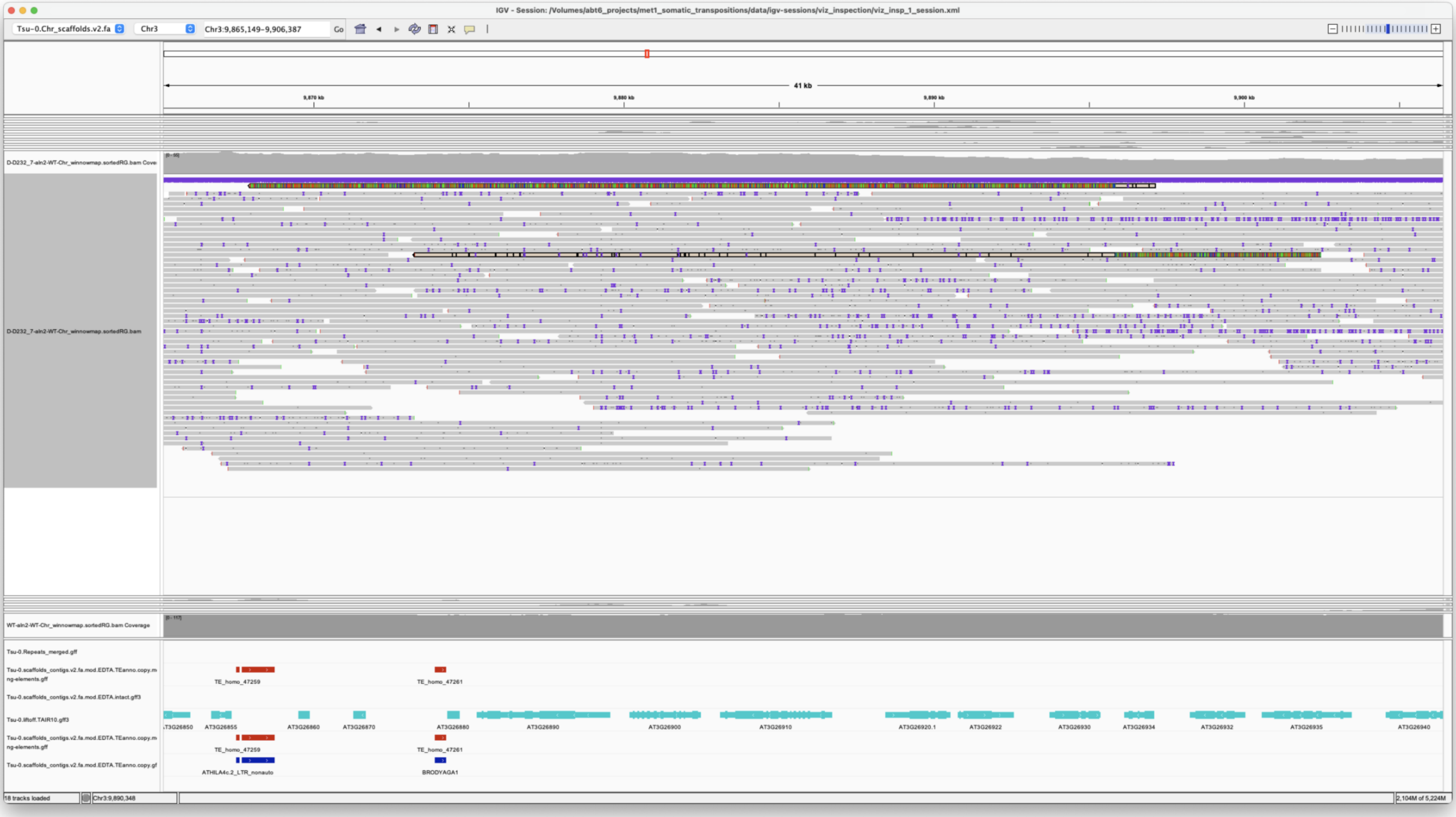

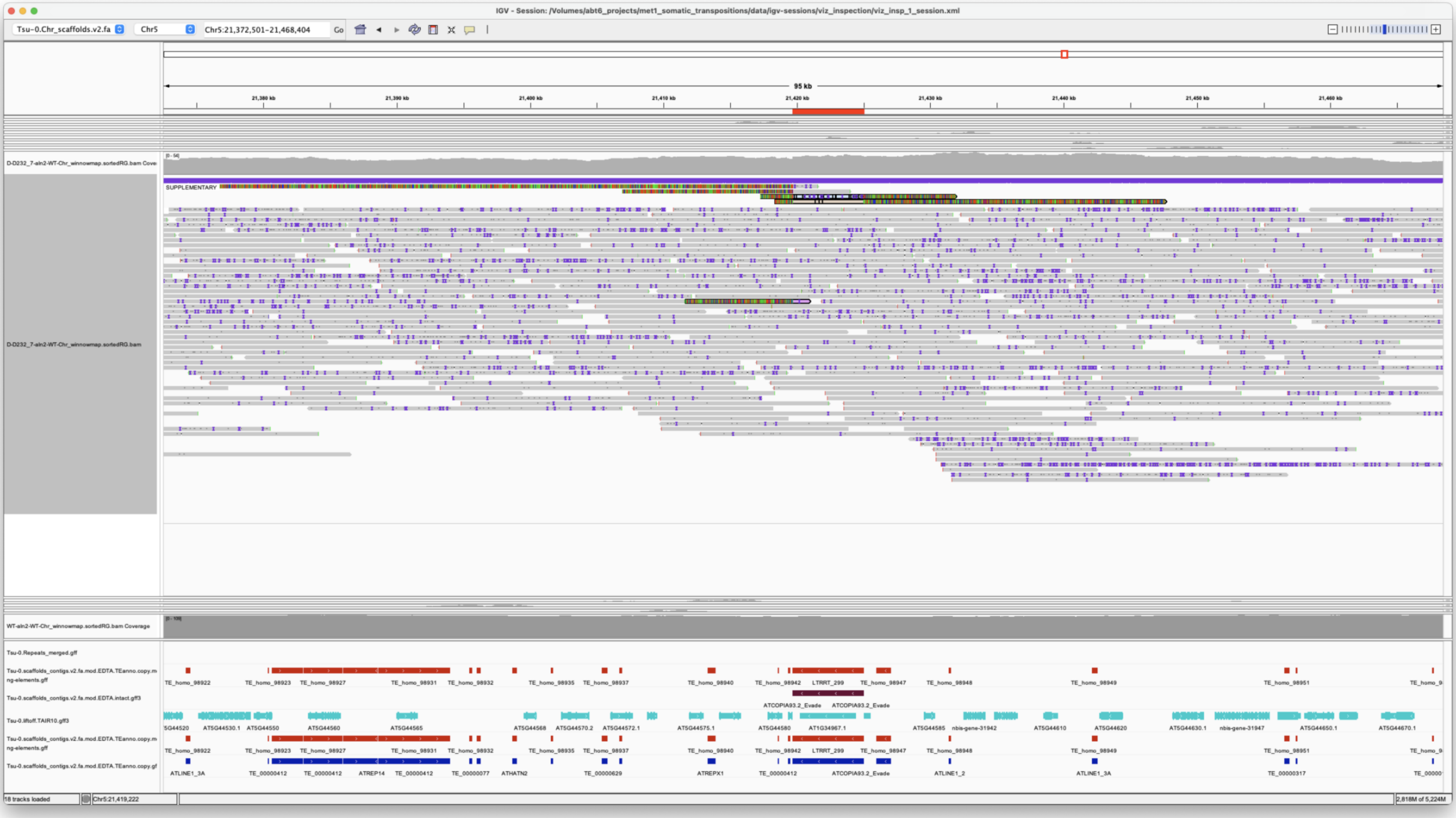

Central
TSD

Confirmed

> Chr3 11549049 11549049 - 1 Chr5;19152829;19160826;VANDAL21 m64079_221220_112036/45615434/ccs met1_07

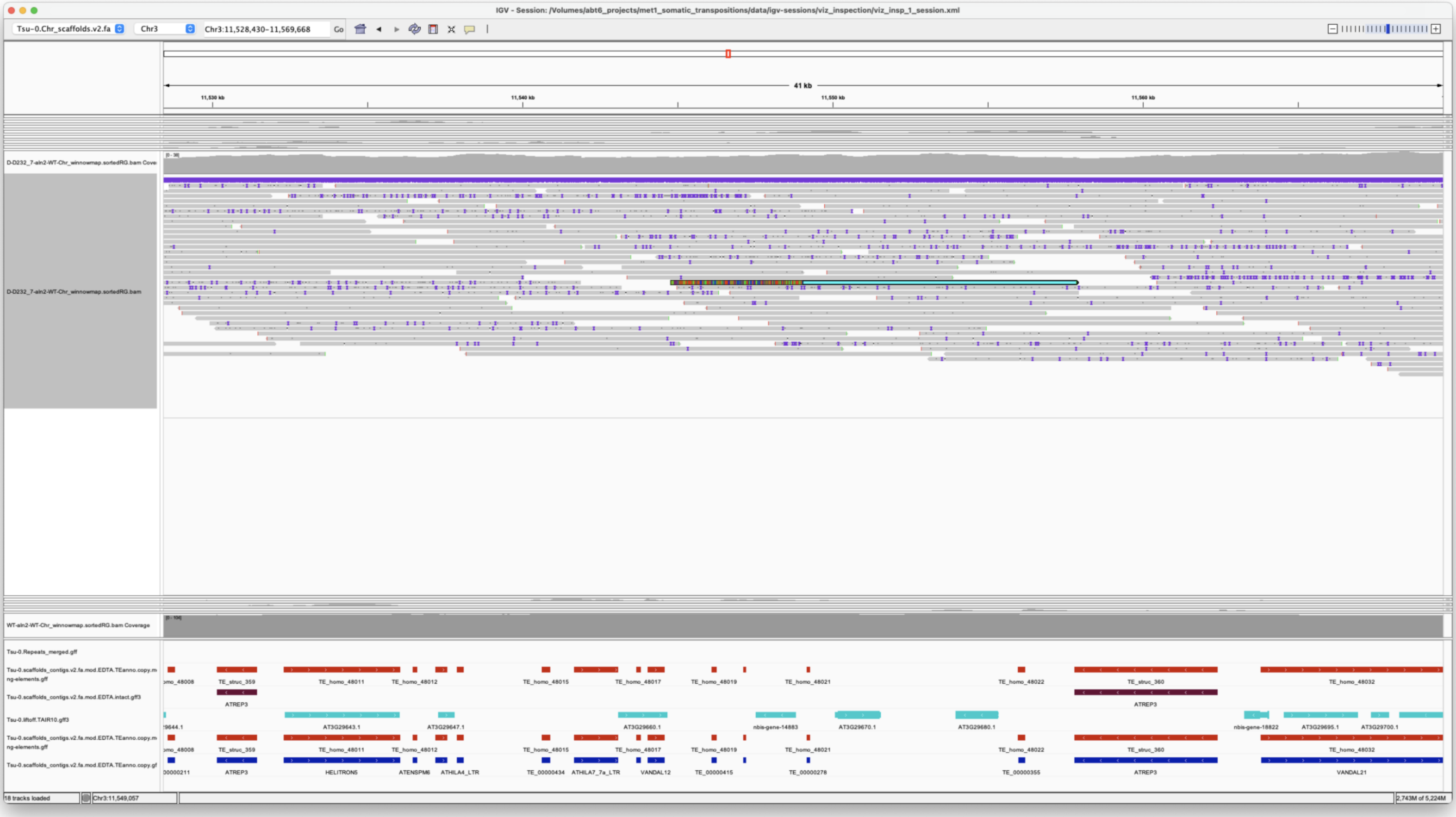

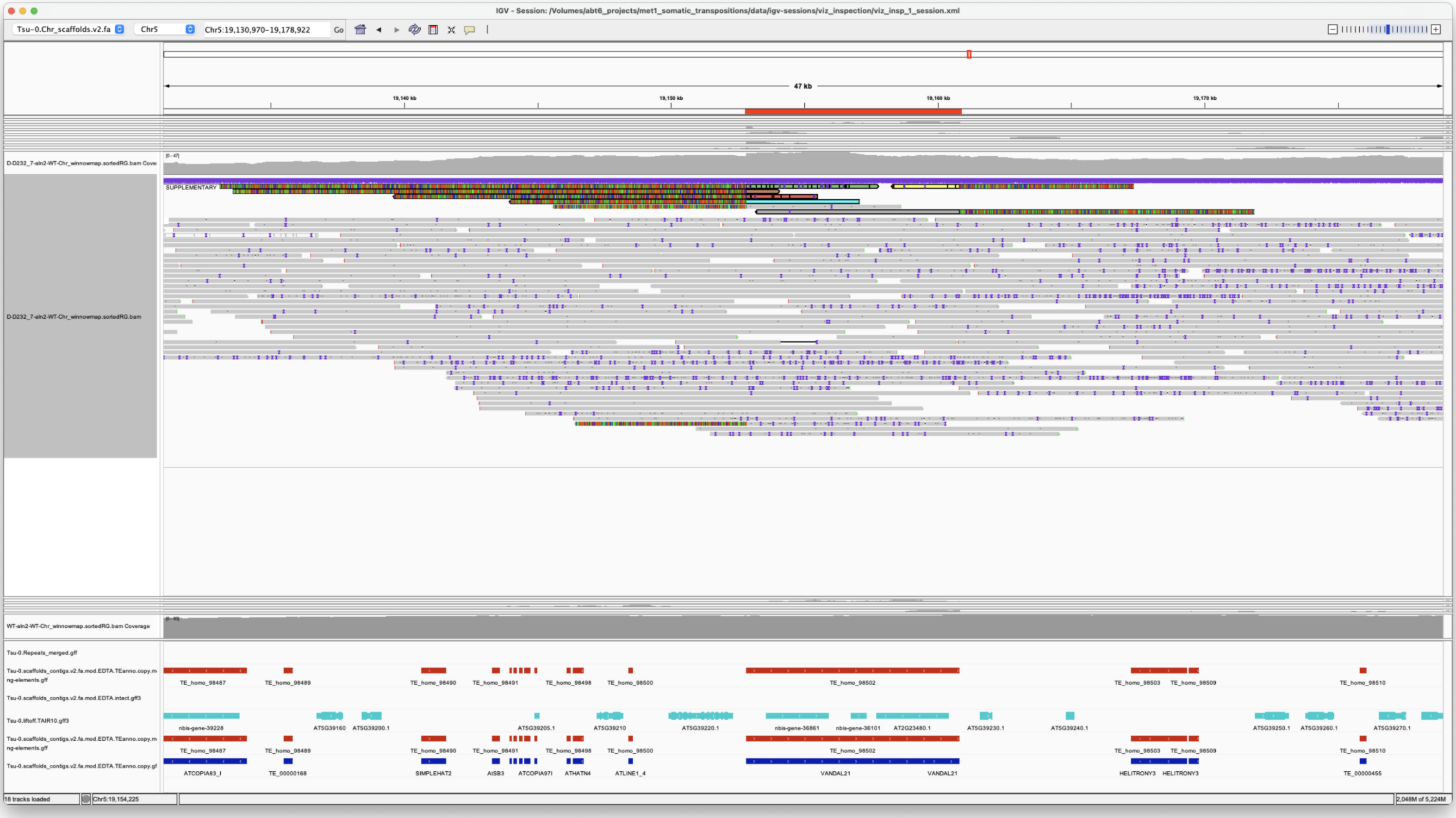

Partial

Confirmed

Chr3 12111670 12111670 + 1 Chr1;11941106;11946436;ATCOPIA93_Evade m64079_240212_113350/39977805/ccs met1_07

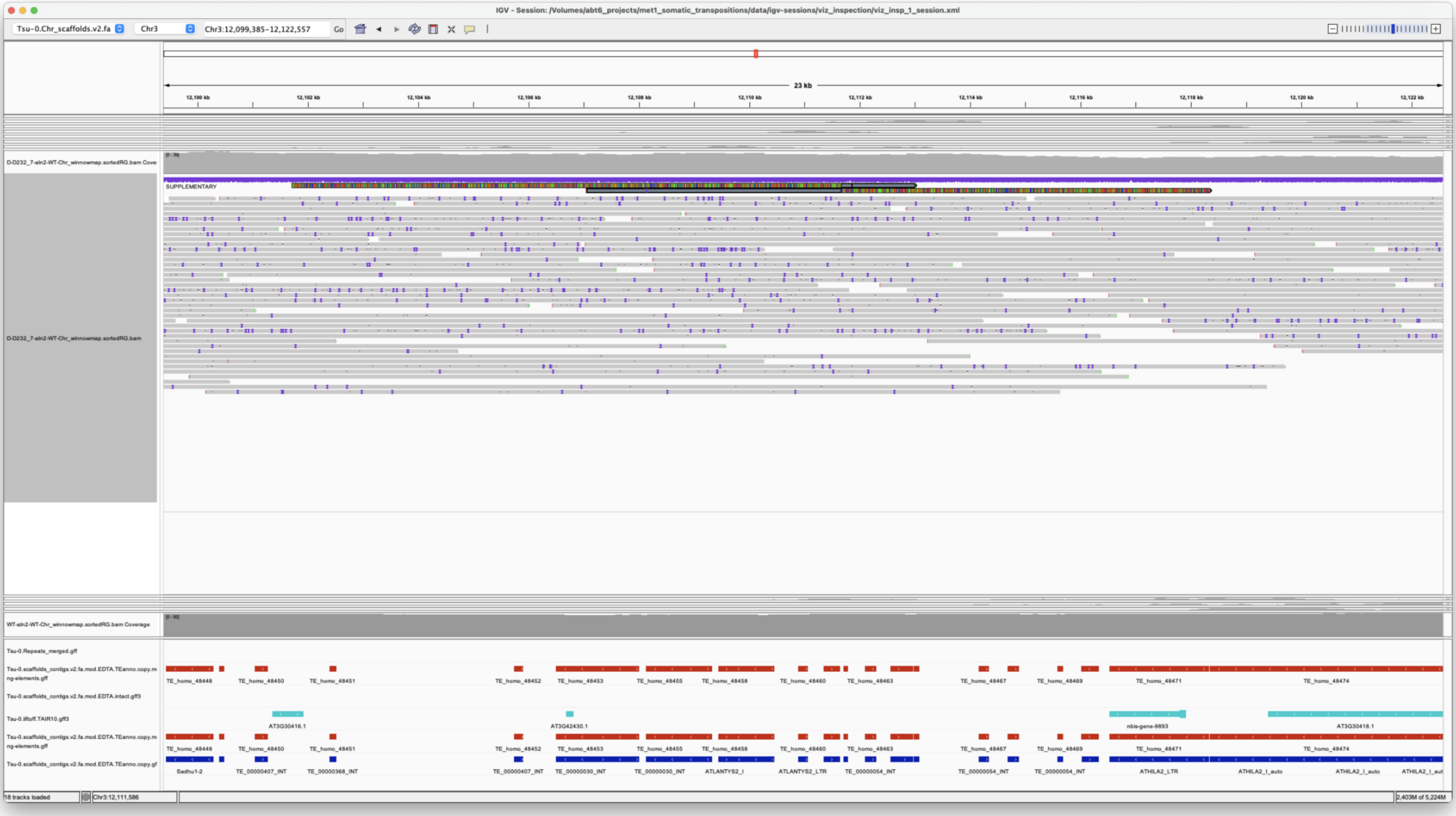

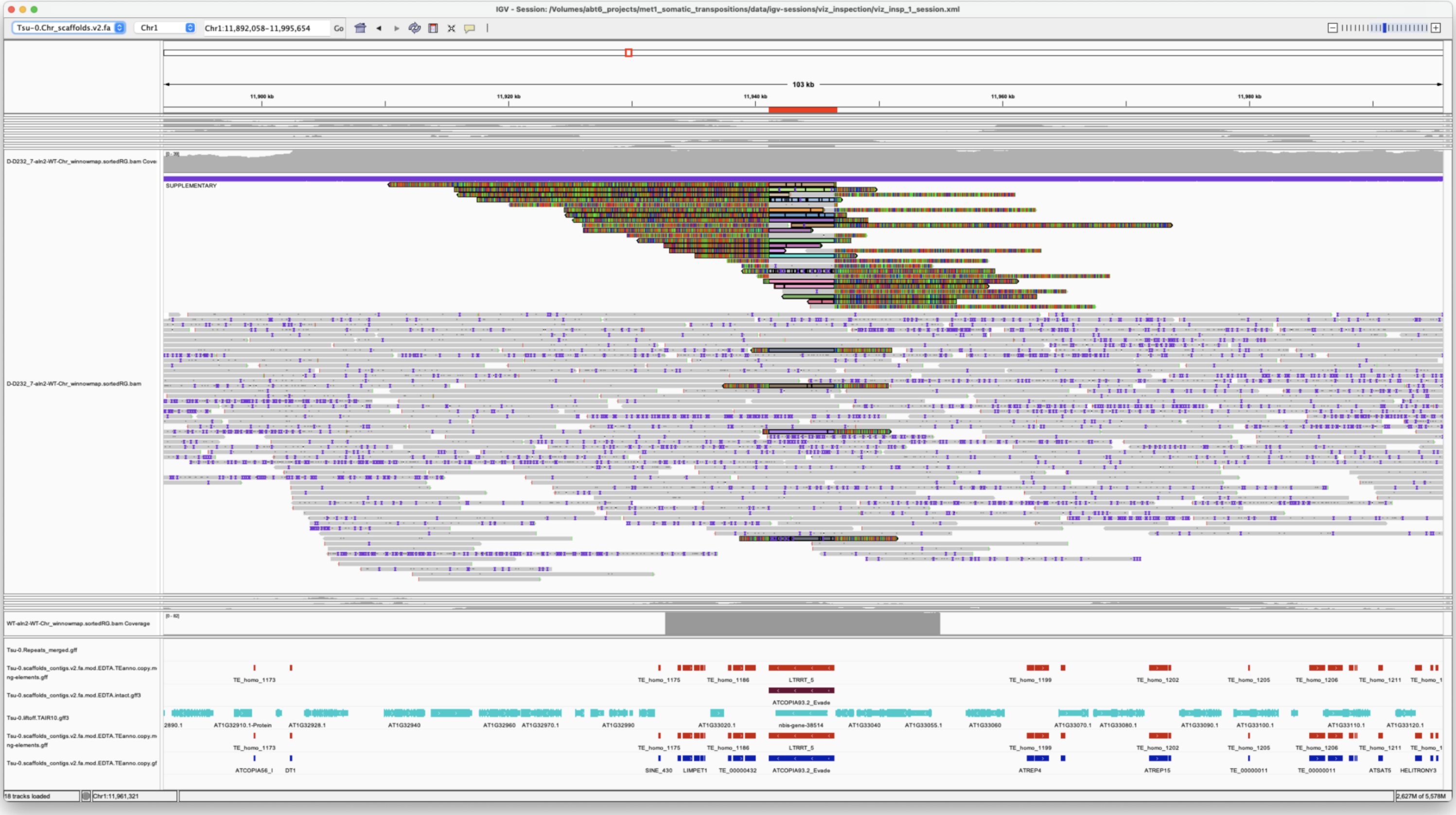

Central
TSD

Confirmed

Chr3 12810809 12810809 + 1 Chr1;11941106;11946436;ATCOPIA93_Evade m64079_240212_113350/63308391/ccs met1_07

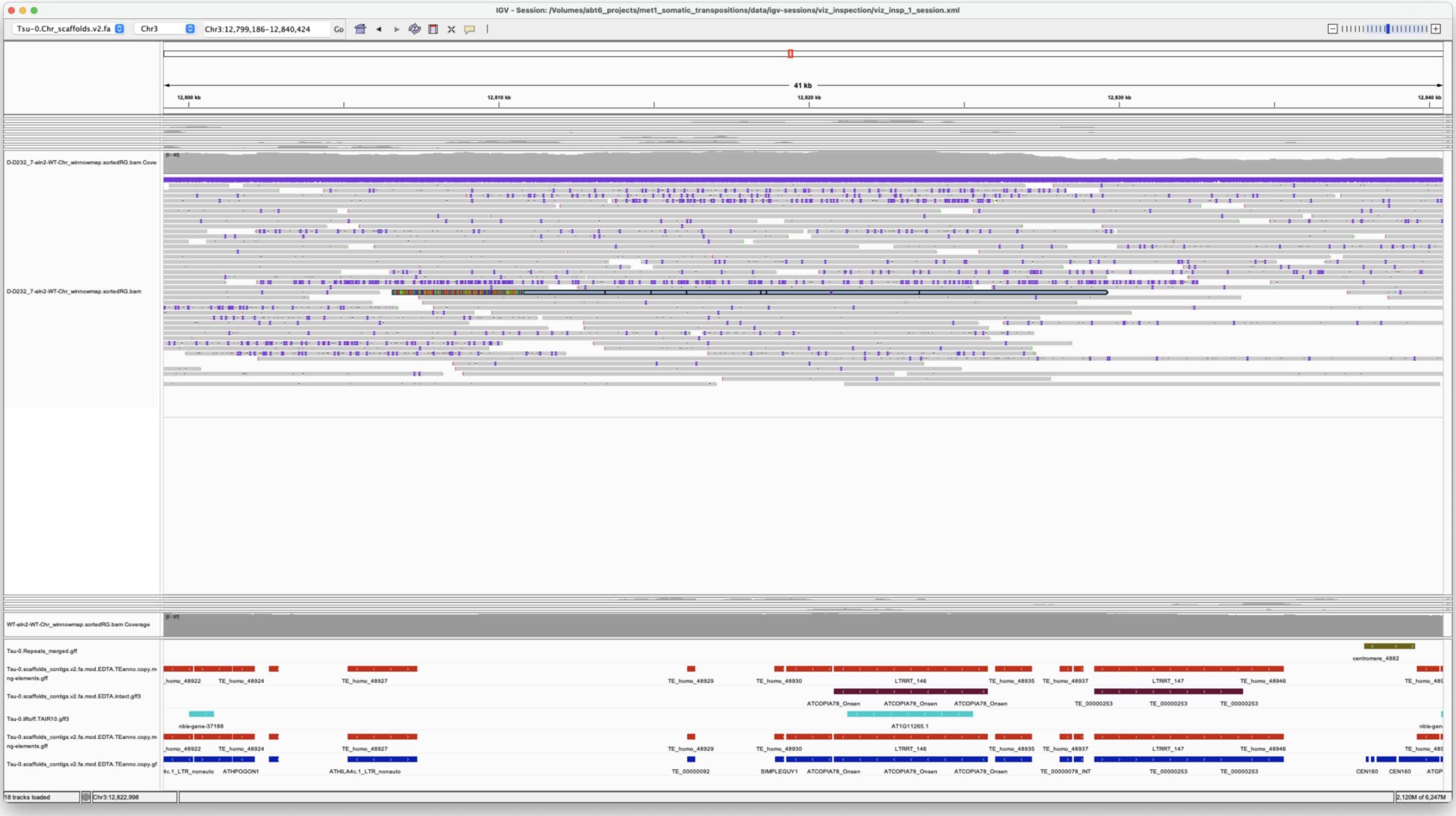

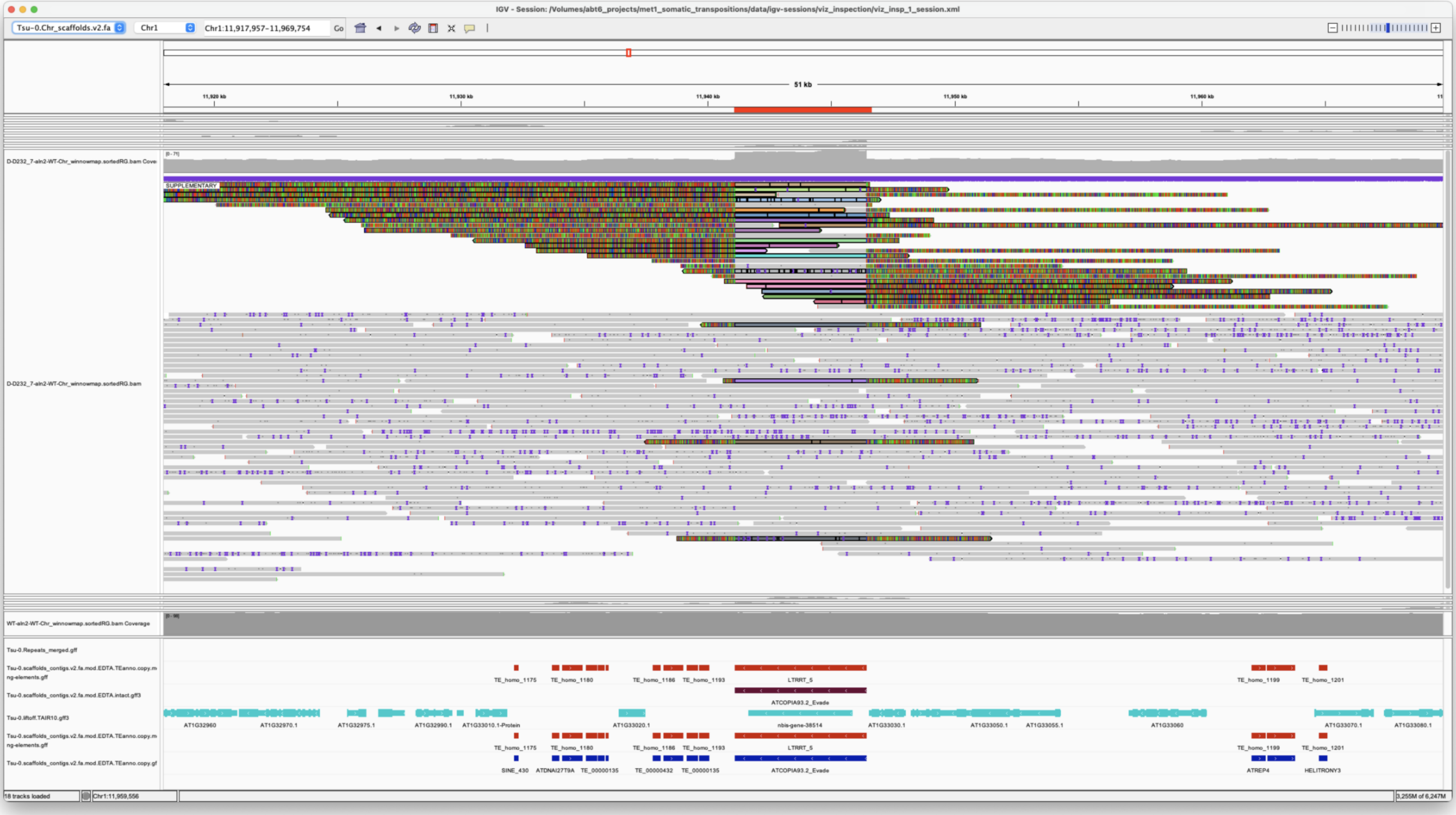

Partial

Confirmed

Chr3 18181483 18181483 - 1 Chr1;11941106;11946436;ATCOPIA93_Evade m64079_240212_113350/75302644/ccs met1_07

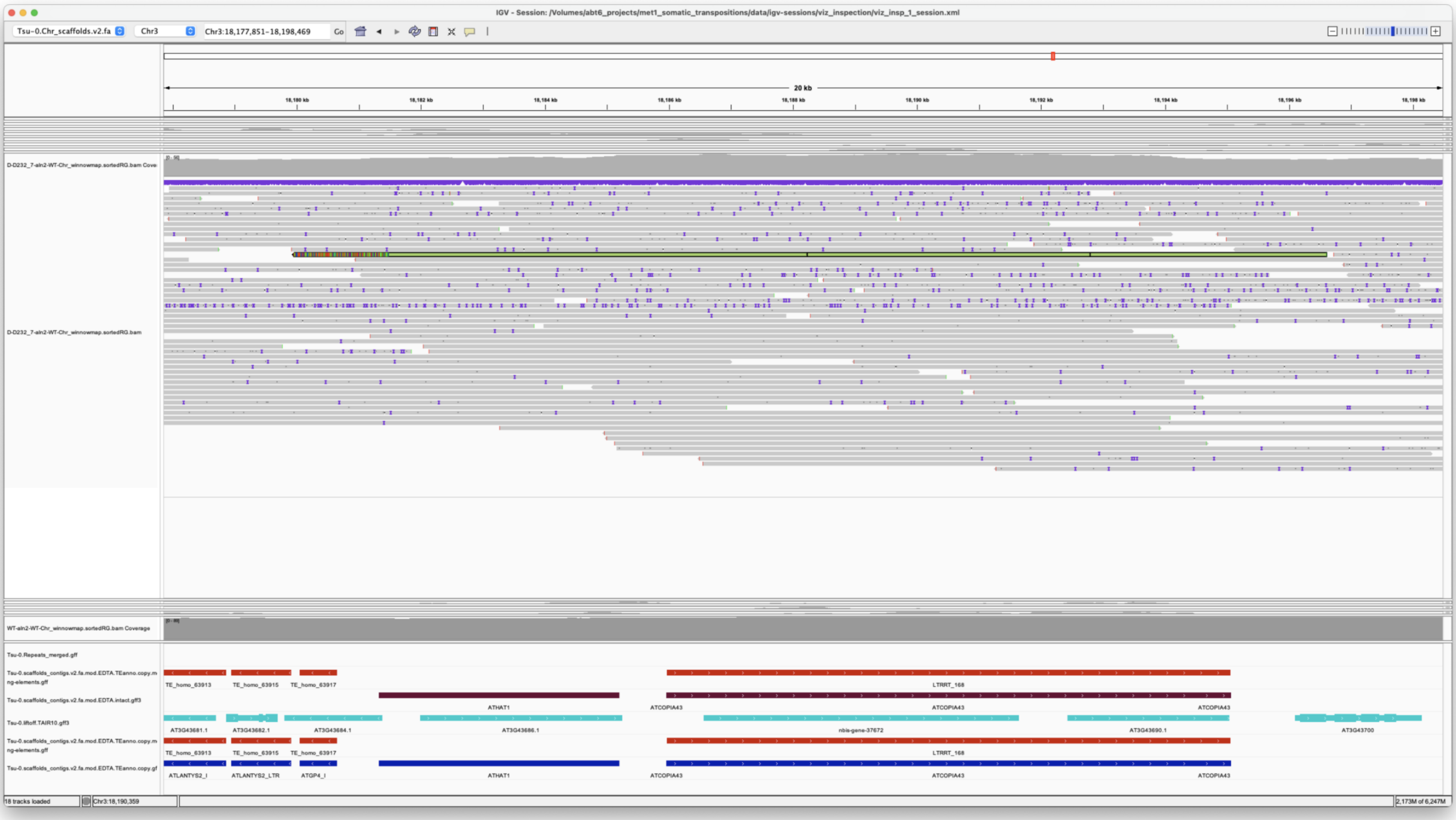

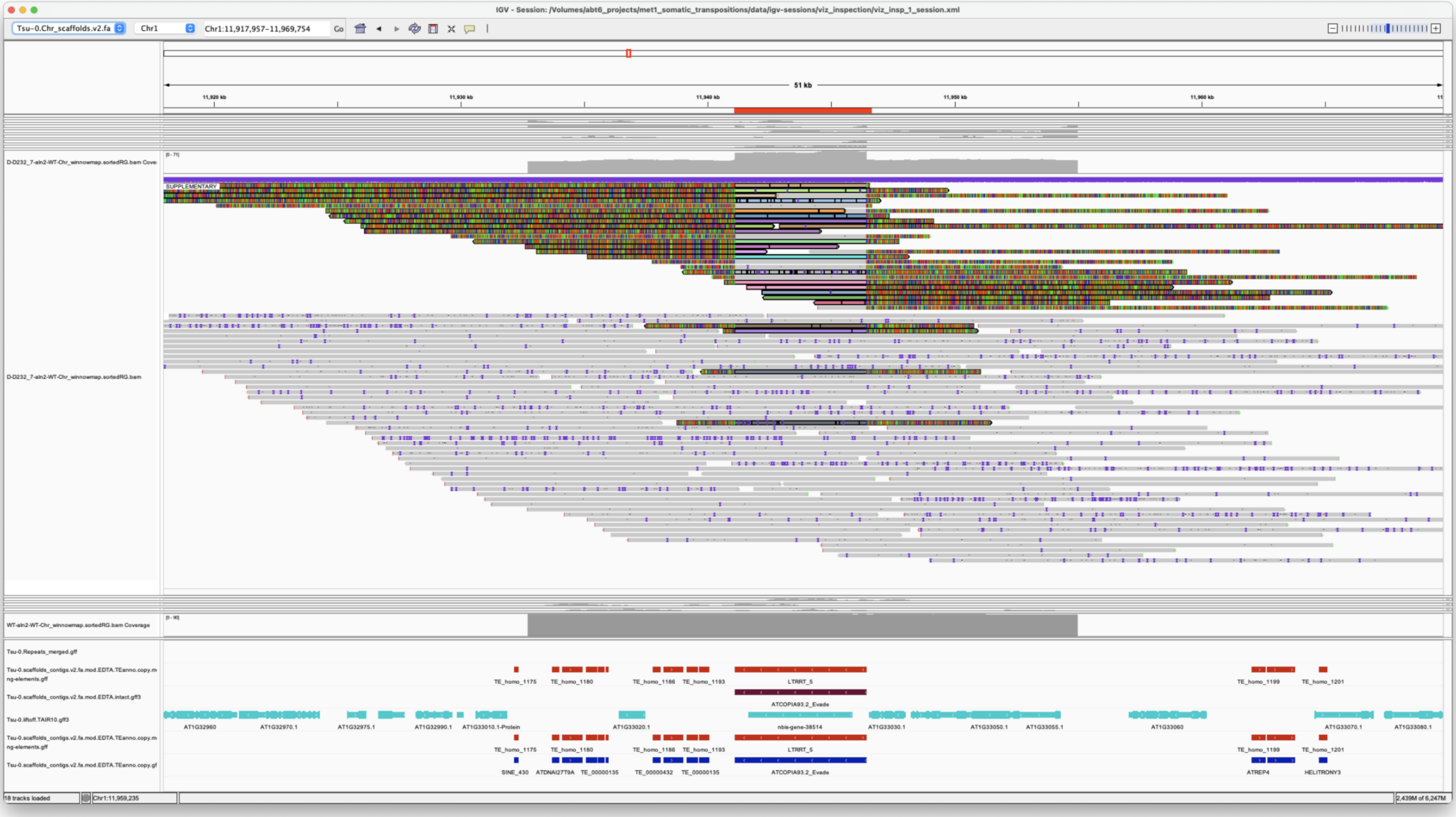

Partial

Confirmed

Chr3 20194685 20194685 + 1 Chr1;11941106;11946436;ATCOPIA93_Evade m64079_221220_112036/40372065/ccs met1_07

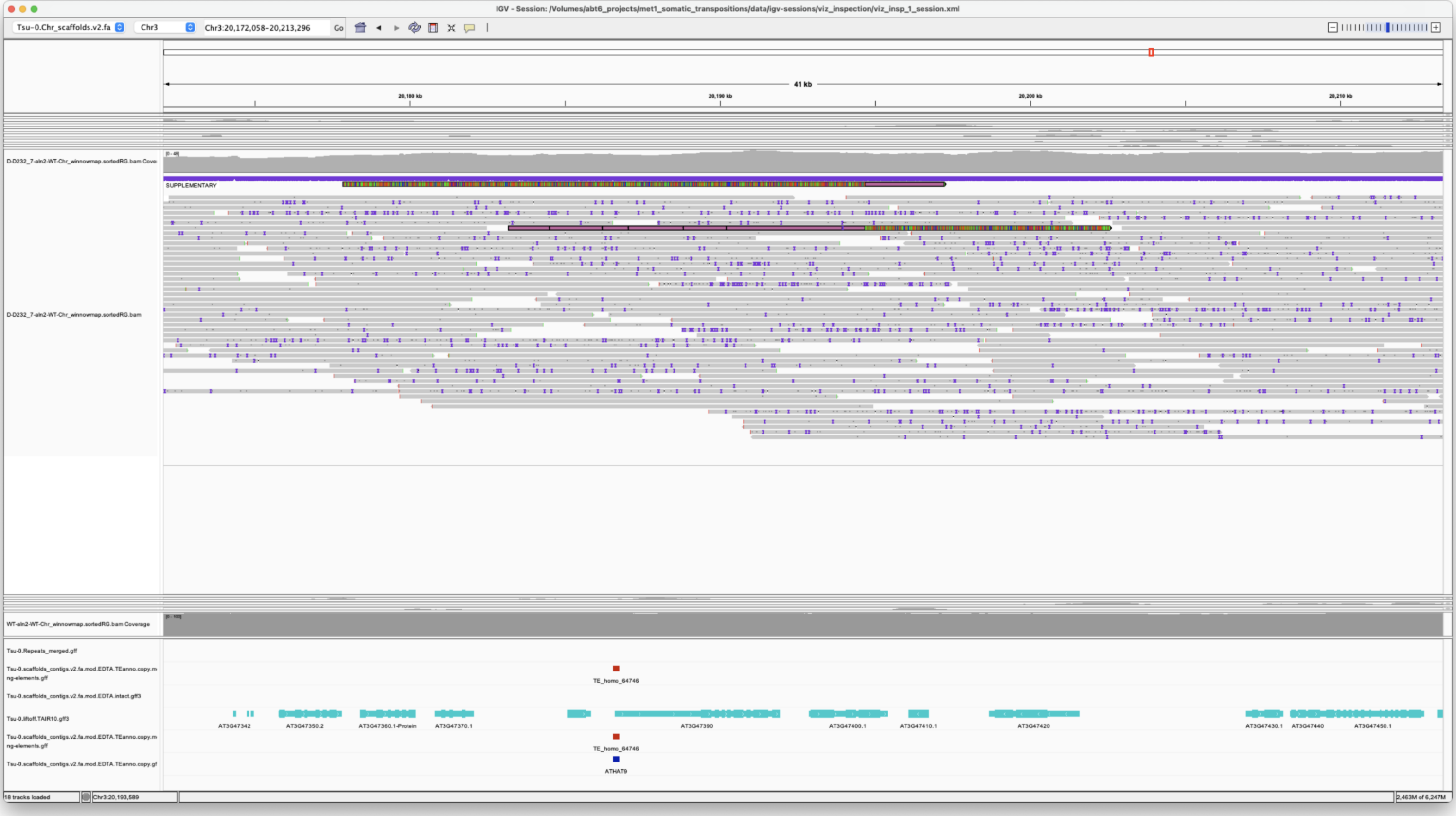

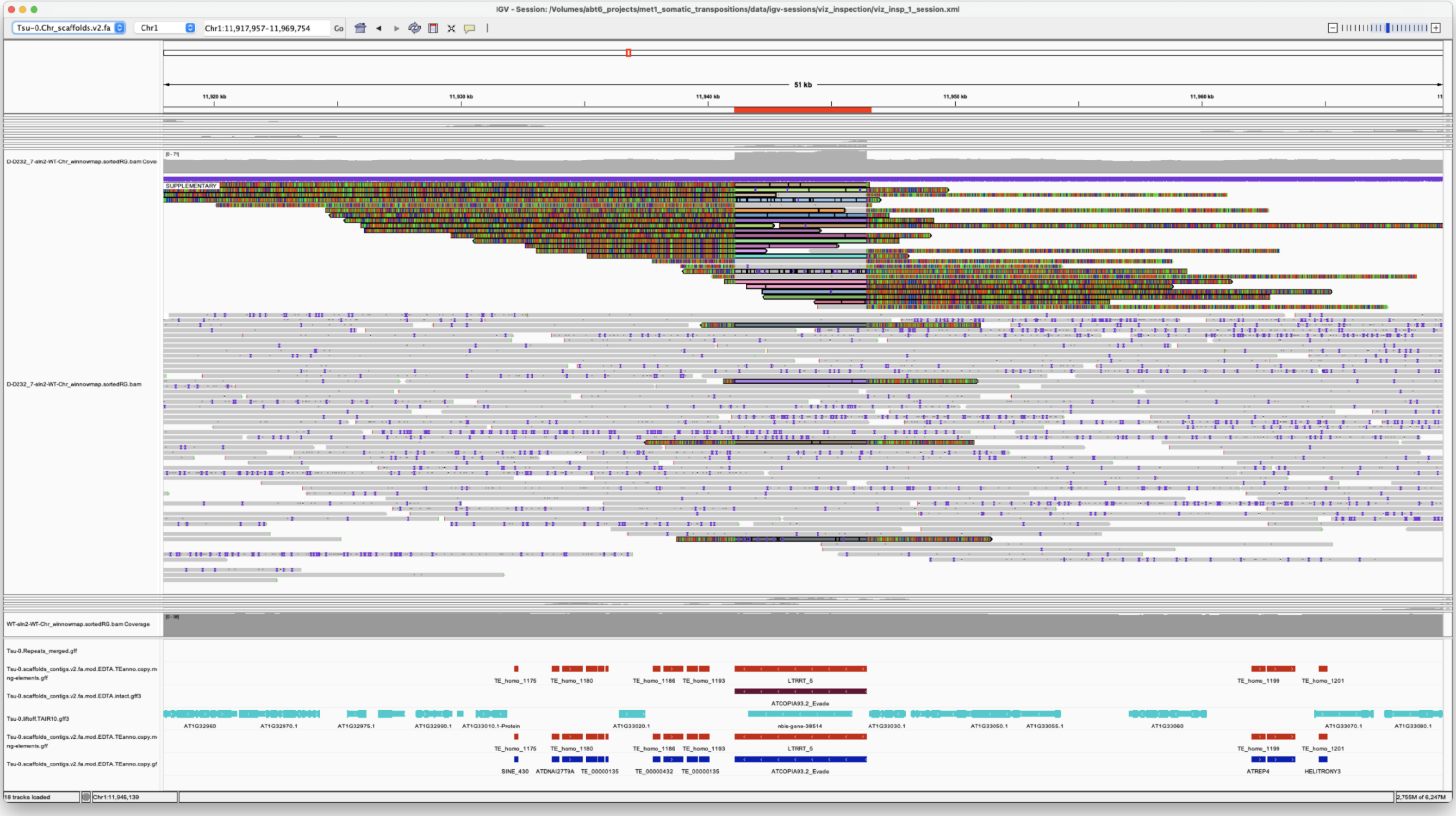

Central
TSD

Confirmed

Chr3 21532333 21532333 + 1 Chr1;11941106;11946436;ATCOPIA93_Evade m64079_221220_112036/49348919/ccs met1_07

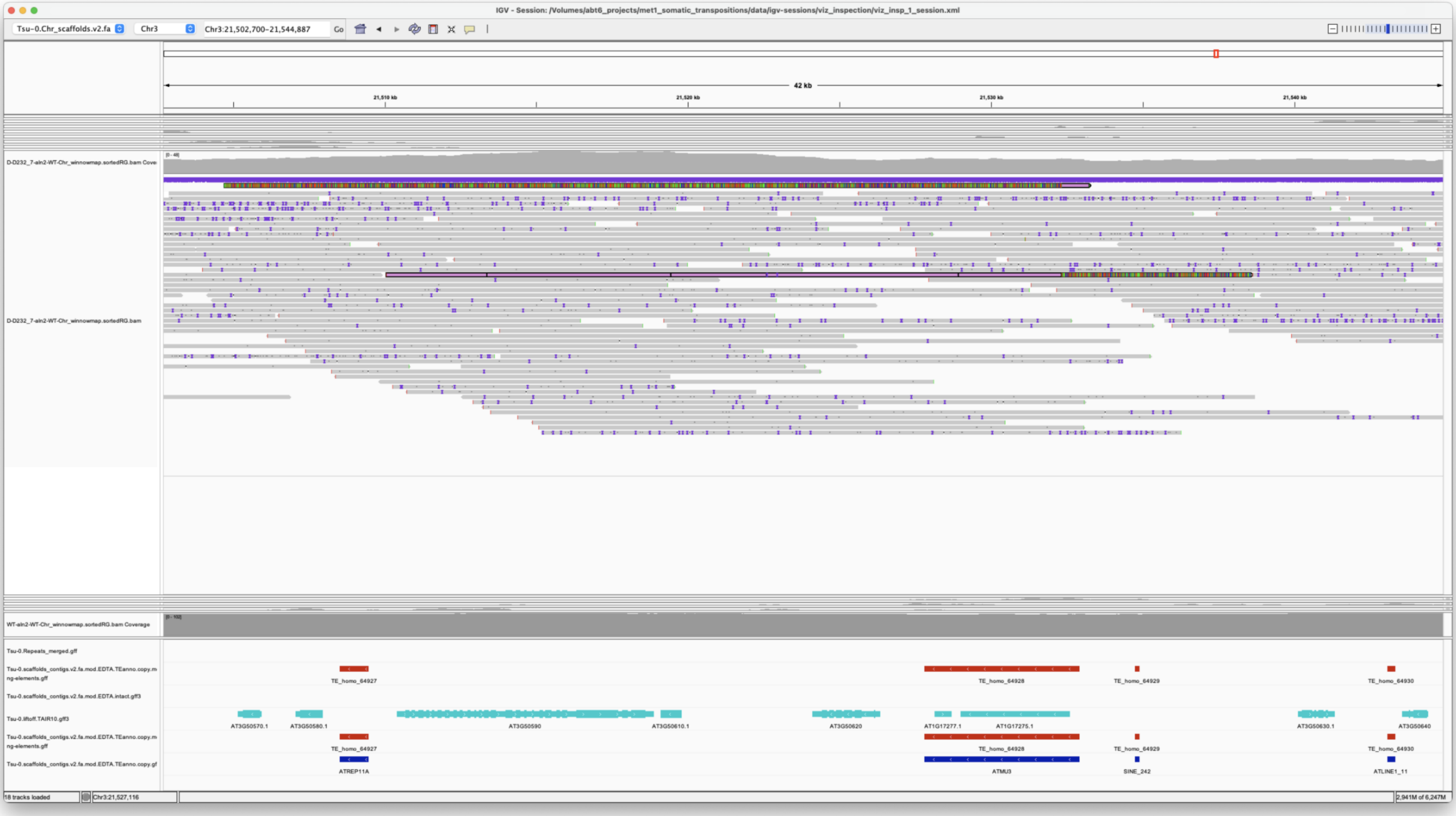

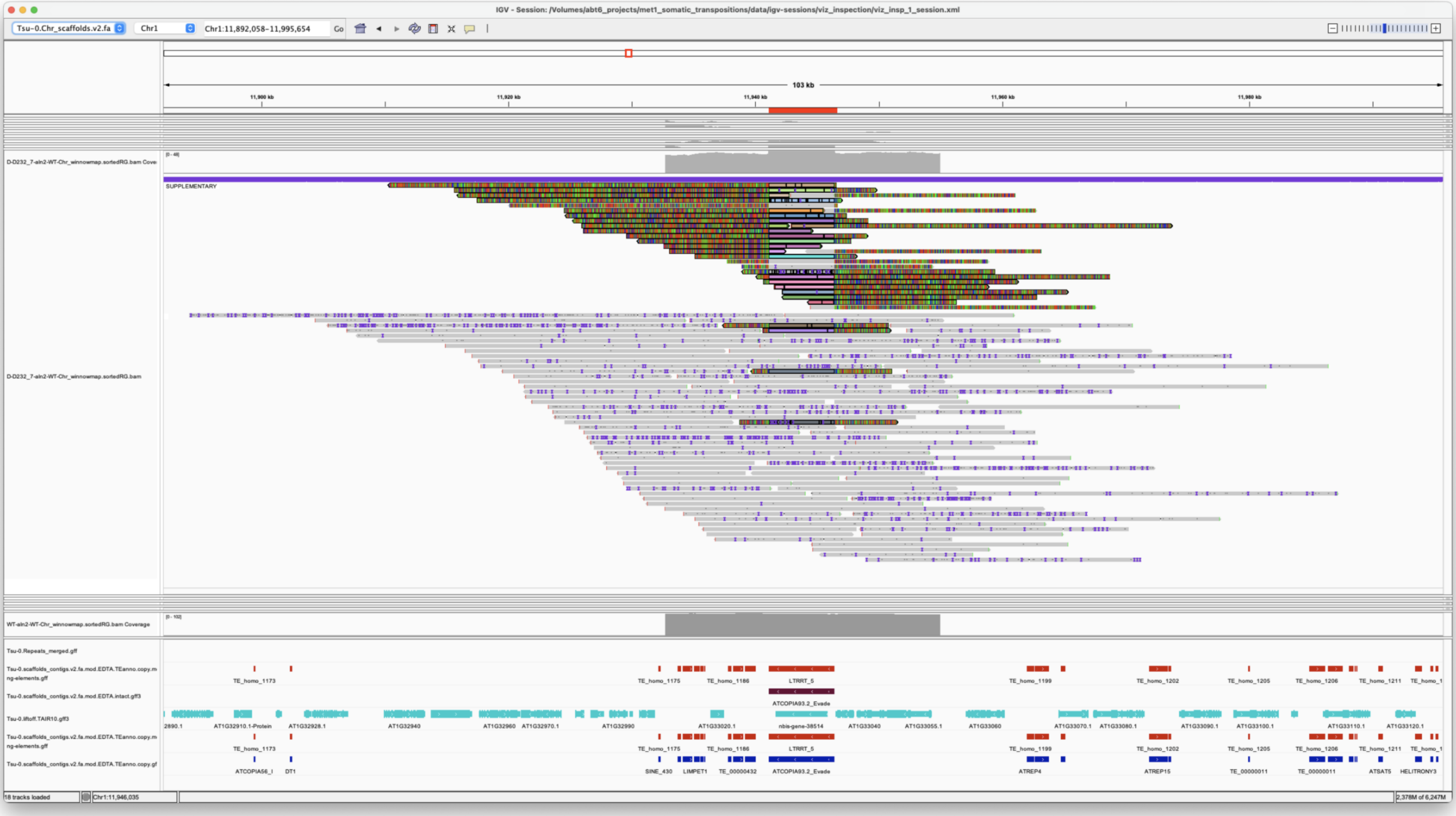

Central
TSD

Confirmed

Chr3 26057985 26057985 - 1 Chr3;20158137;20166150;VANDAL6 m64079_240212_113350/163318297/ccs met1_07

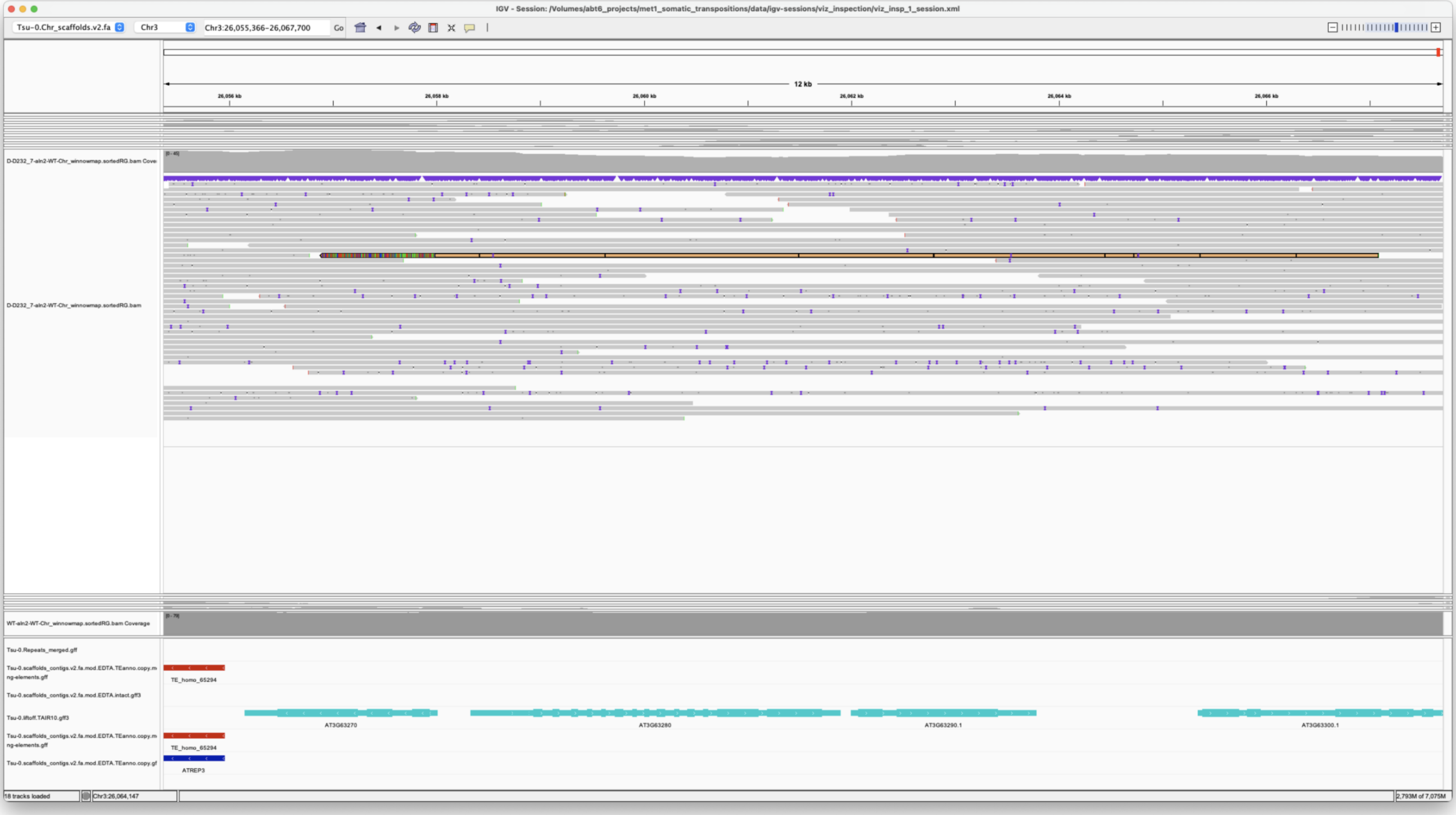

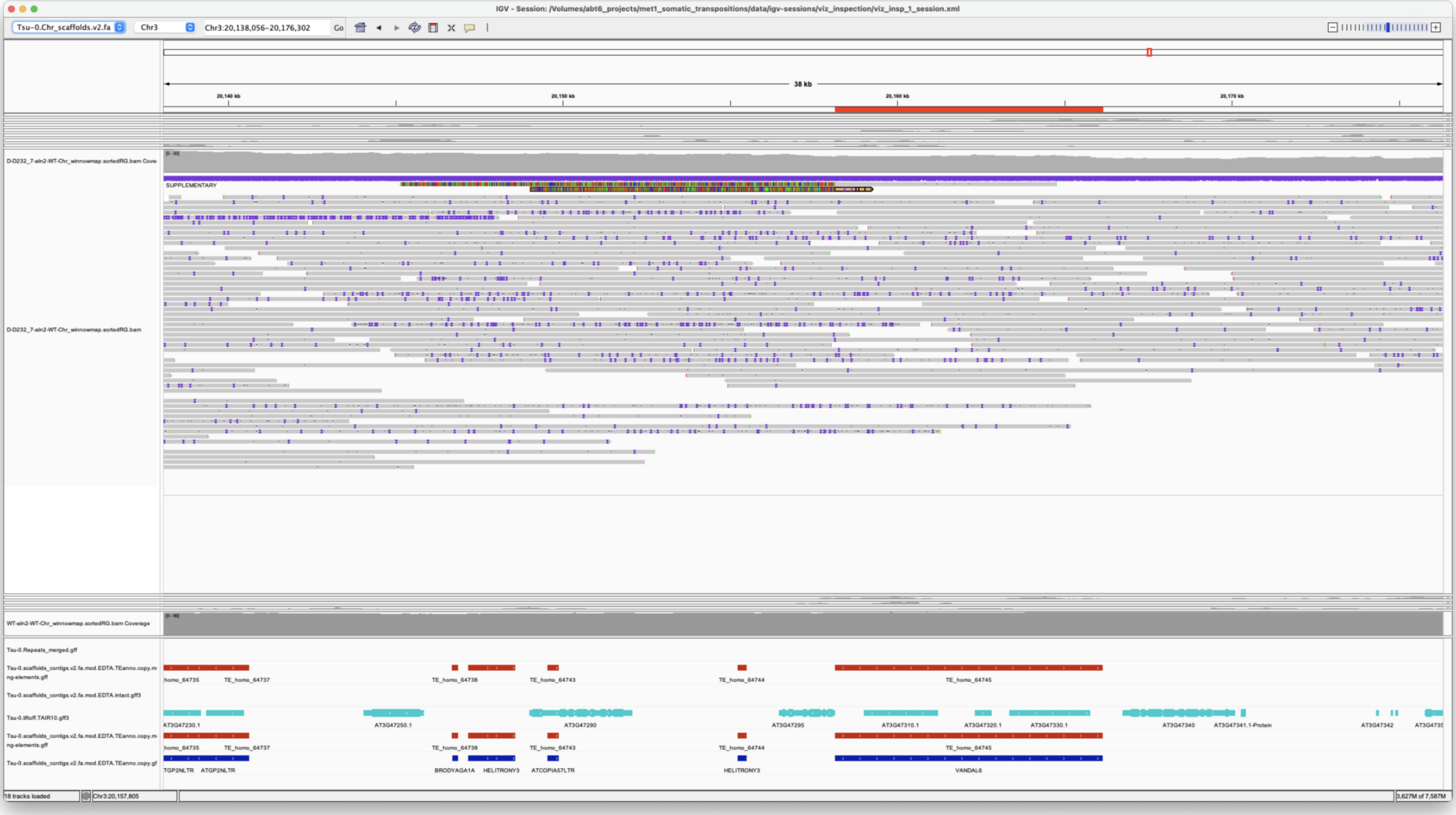

Partial

Confirmed

Chr4 9036234 9036236 +,+ 2 Chr4;9027642;9031534;VANDAL8,Chr4;9027642;9031534;VANDAL8 m64079_221220_112036/150536265/ccs,m64079_240212_113350/27067180/ccs met1_07

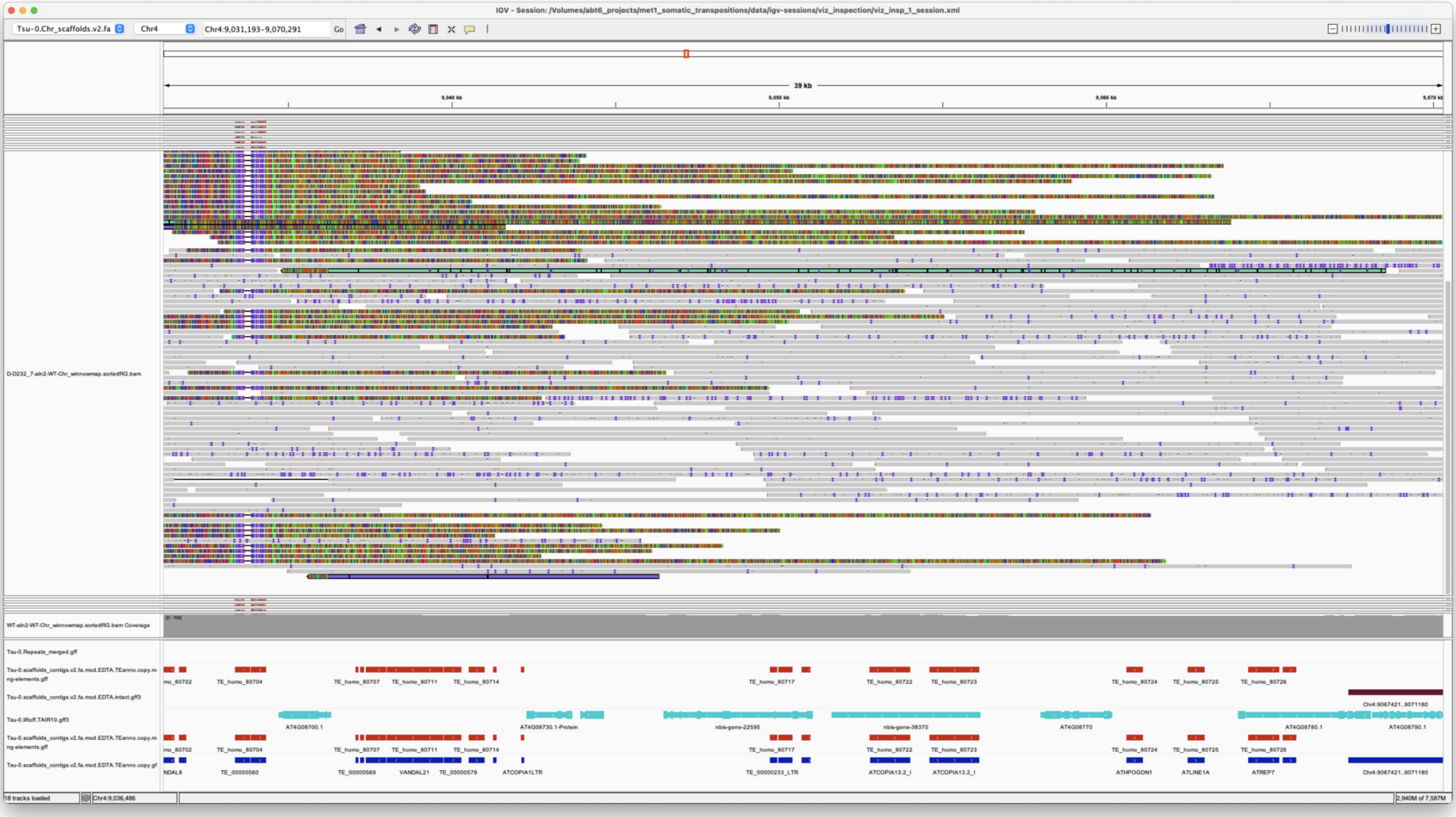

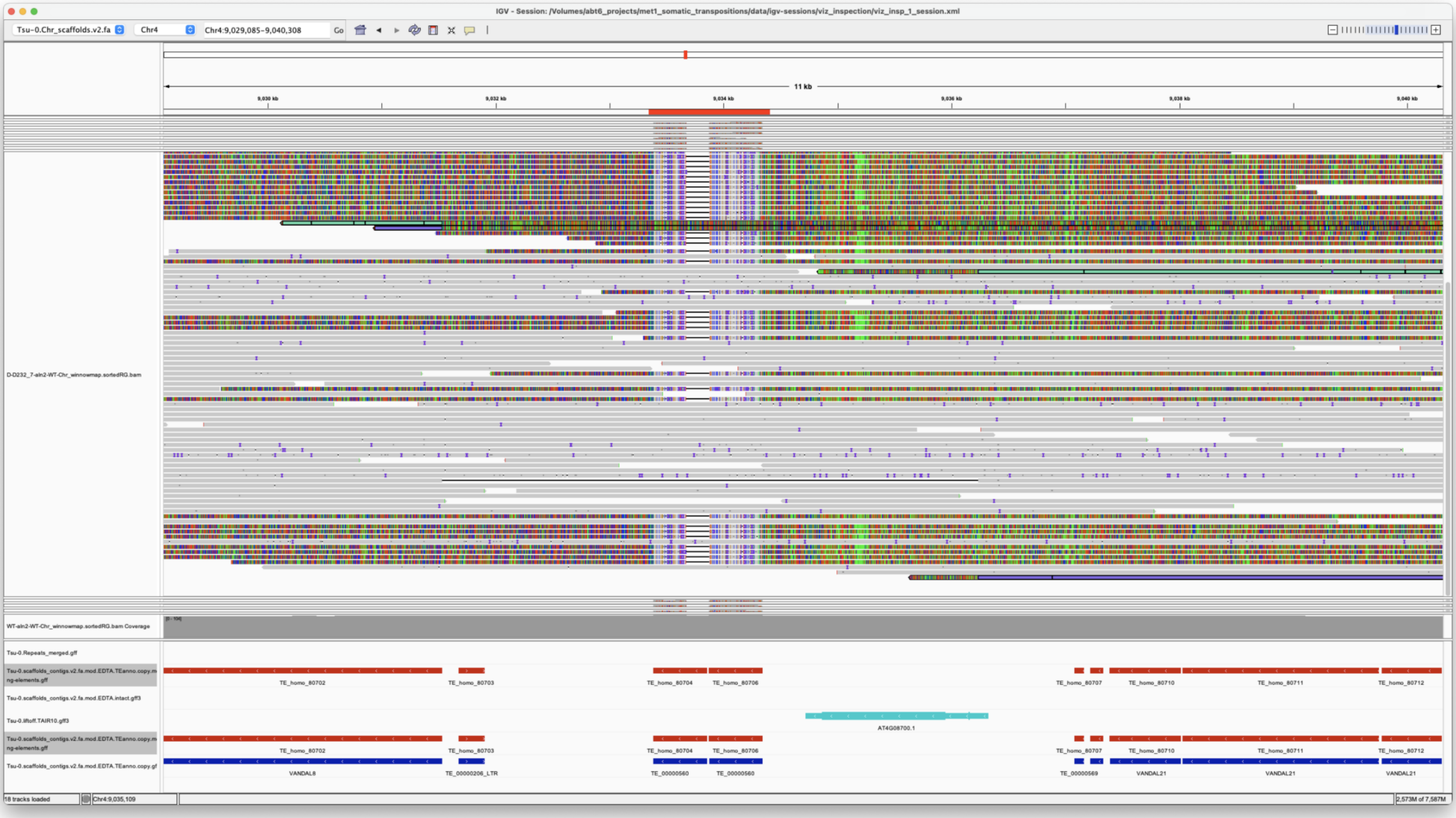

Probably a deletion because of VANDAL cut but cannot be sure

Hypermutable

Rearrangement

unsupported

Chr4 13794306 13794306 + 1 Chr1;11941106;11946436;ATCOPIA93_Evade m64079_221220_112036/168427757/ccs met1_07

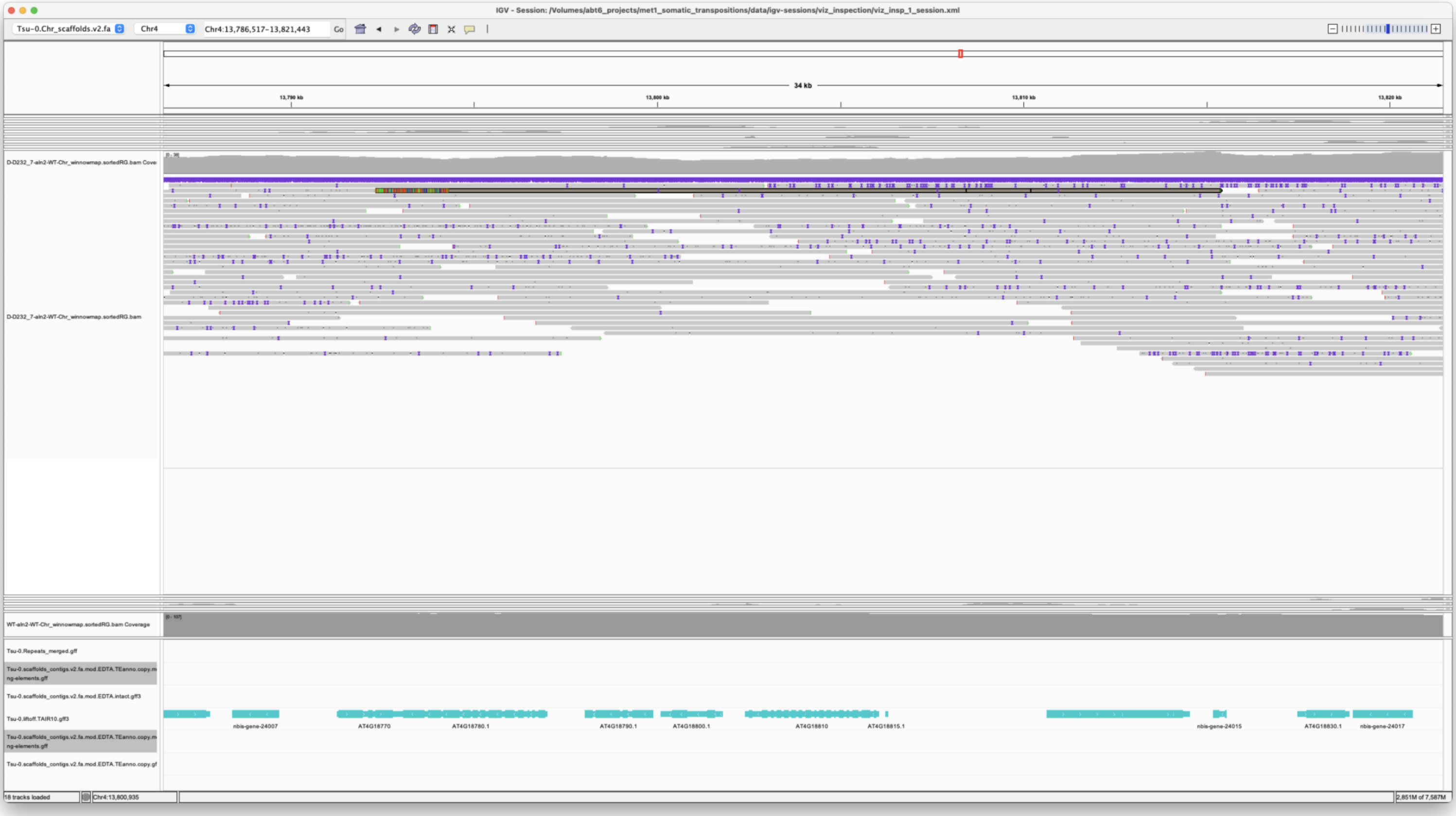

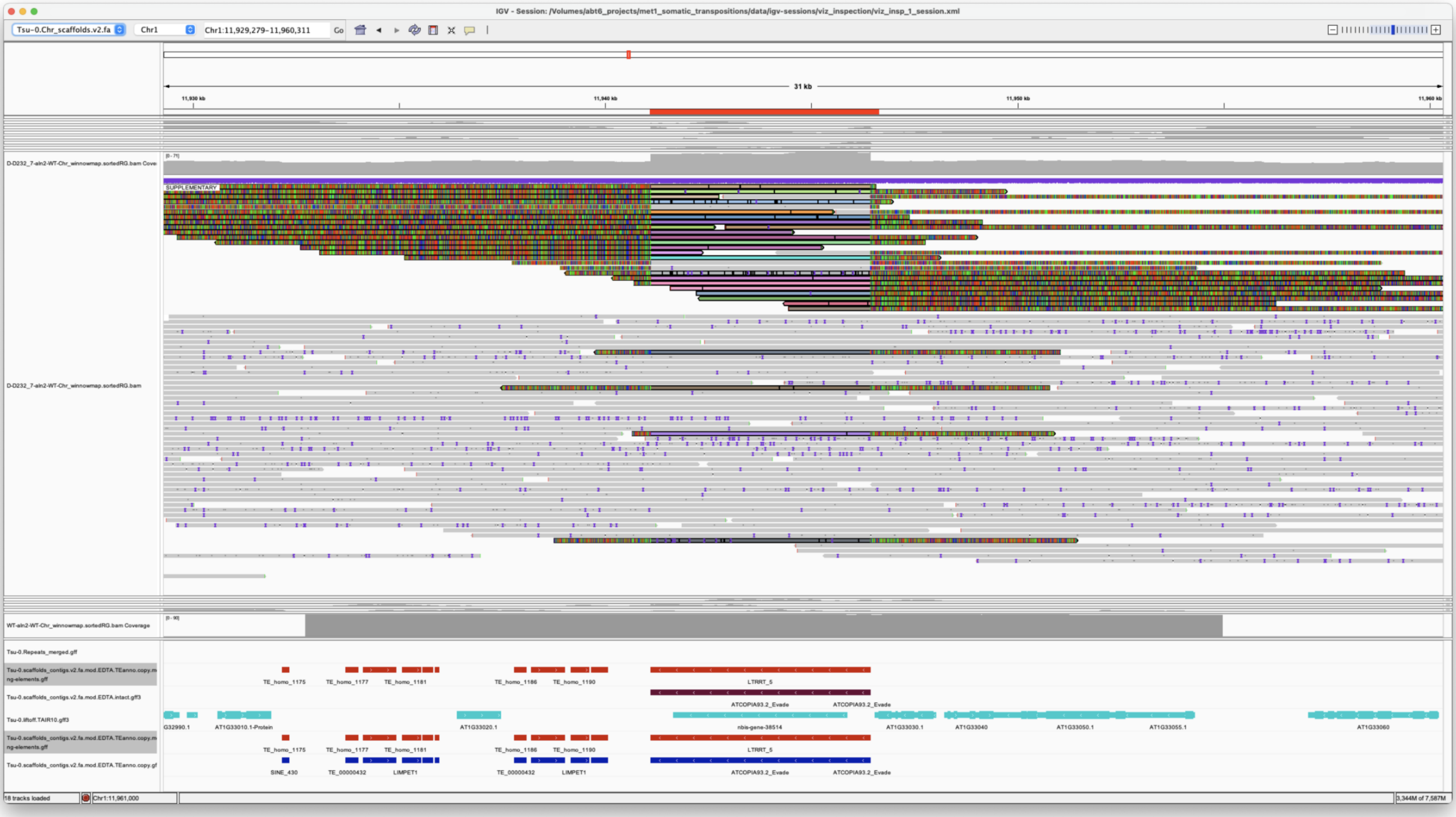

Partial

Confirmed

Chr4 15590879 15590879 - 1 Chr1;11941106;11946436;ATCOPIA93_Evade m64079_221220_112036/112592716/ccs met1_07

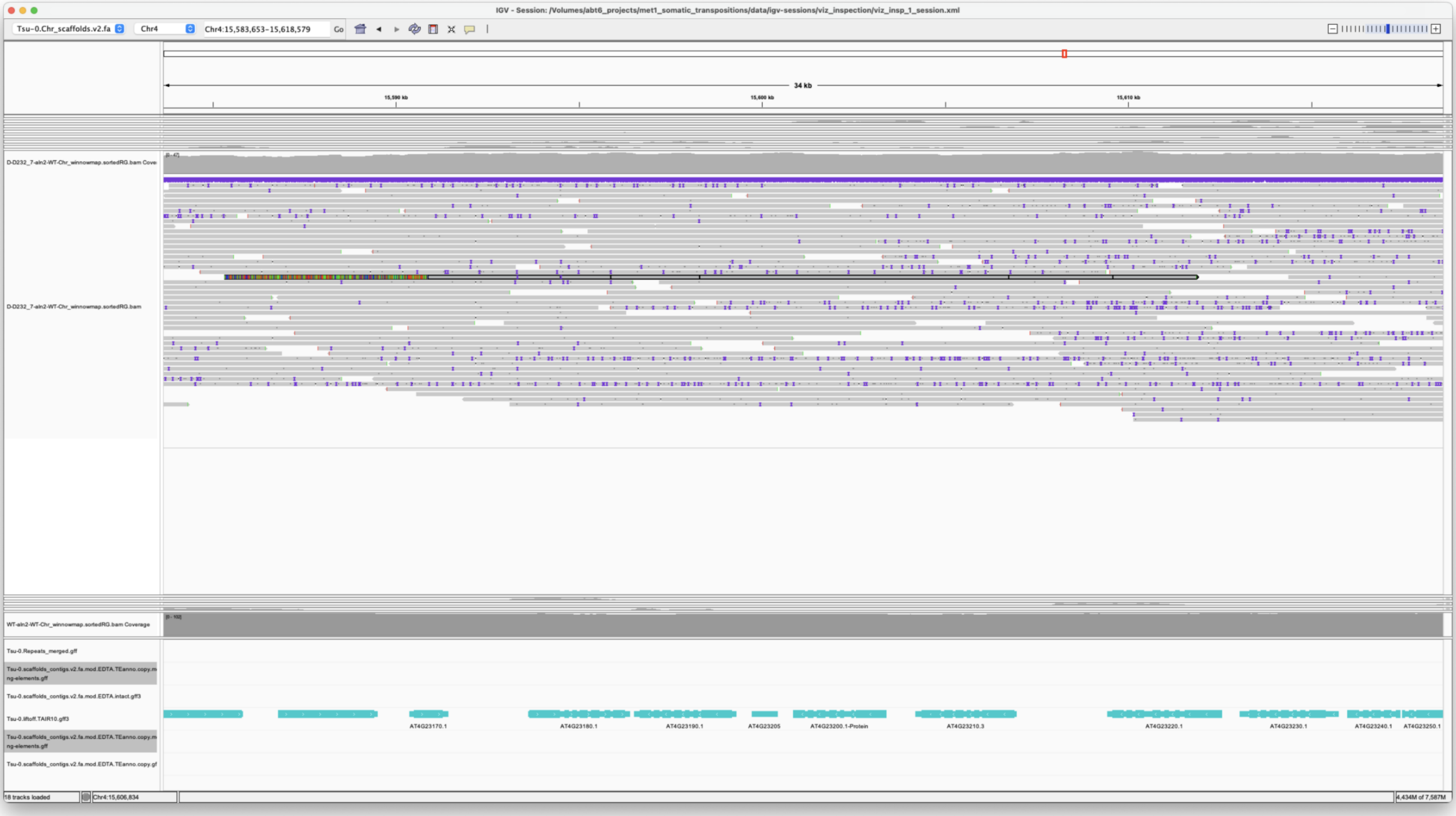

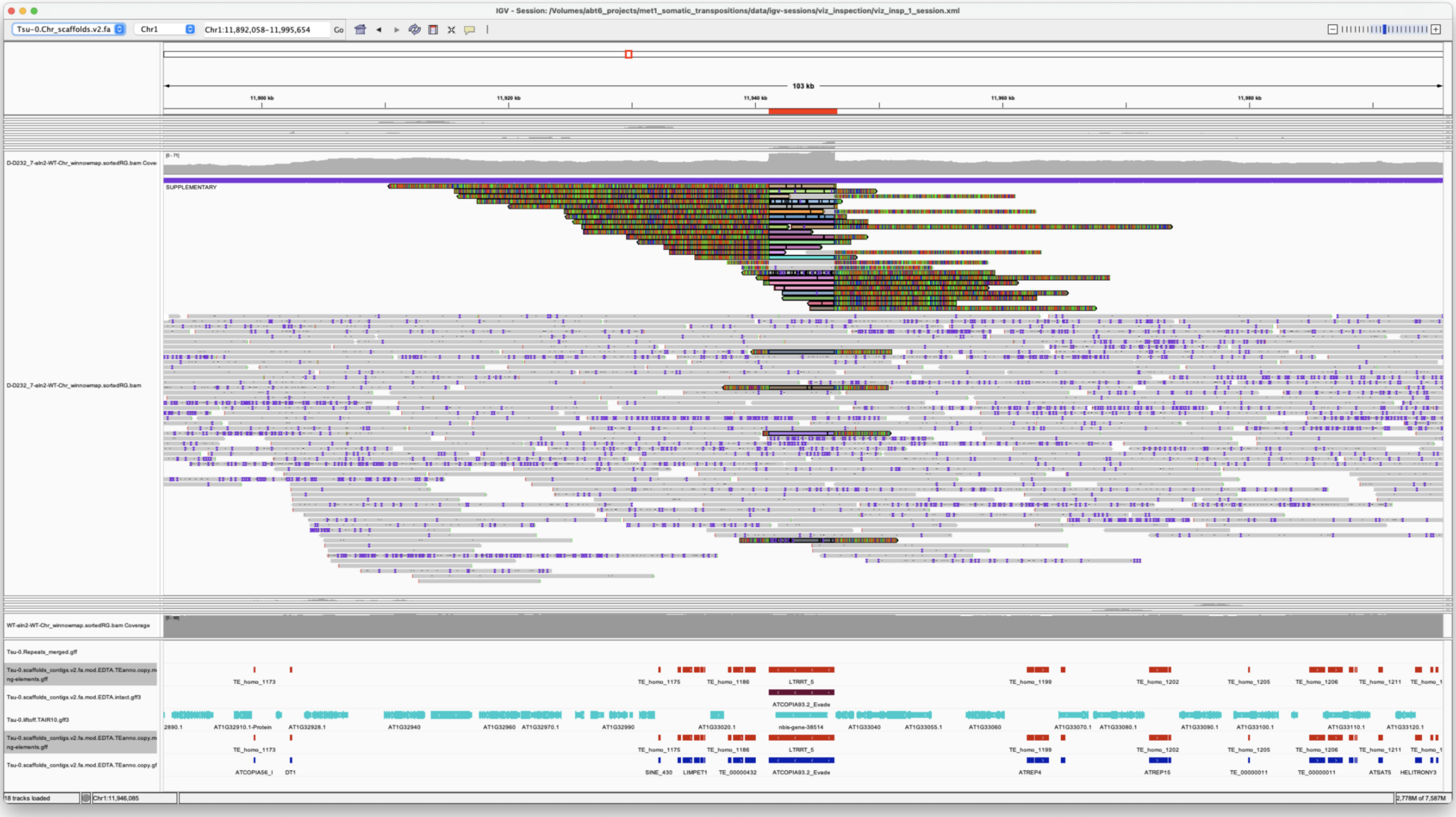

Partial

Confirmed

Chr4 17642094 17642094 + 1 Chr1;11941106;11946436;ATCOPIA93_Evade m64079_240212_113350/157417977/ccs met1_07

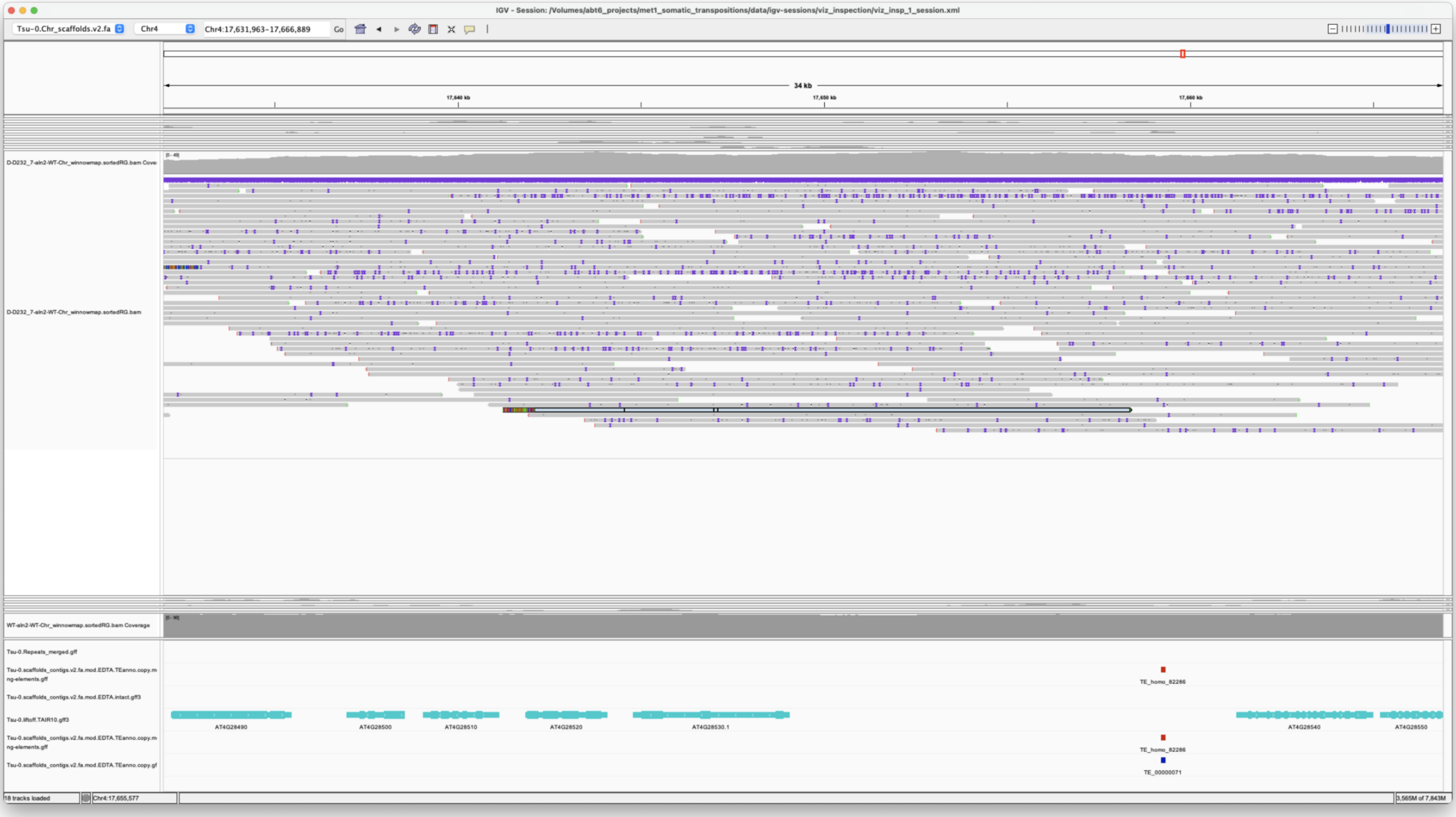

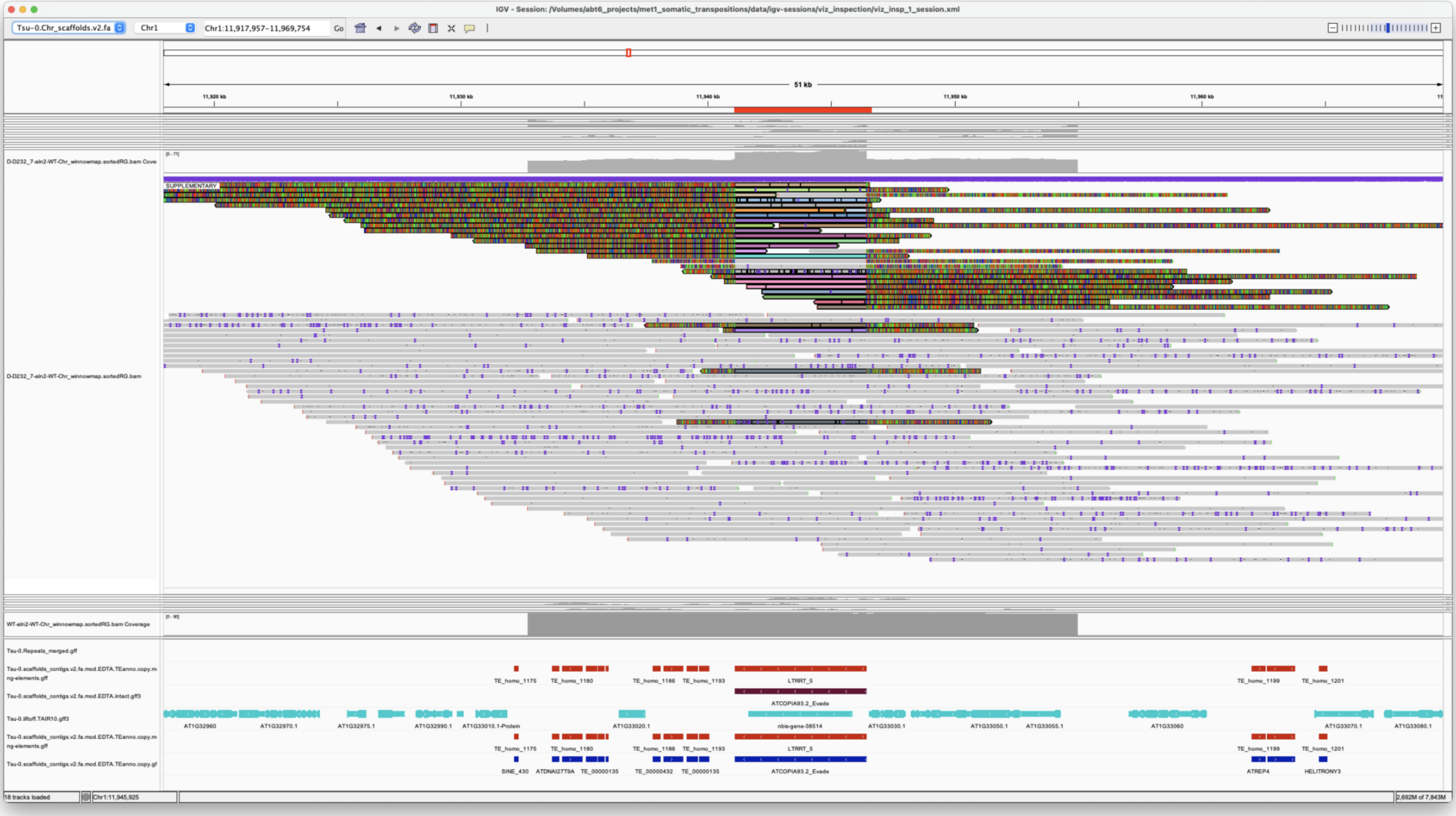

Partial

Confirmed

Chr4 18186288 18186288 + 1 Chr5;21419693;21425022;ATCOPIA93_Evade m64079_240212_113350/135727227/ccs met1_07

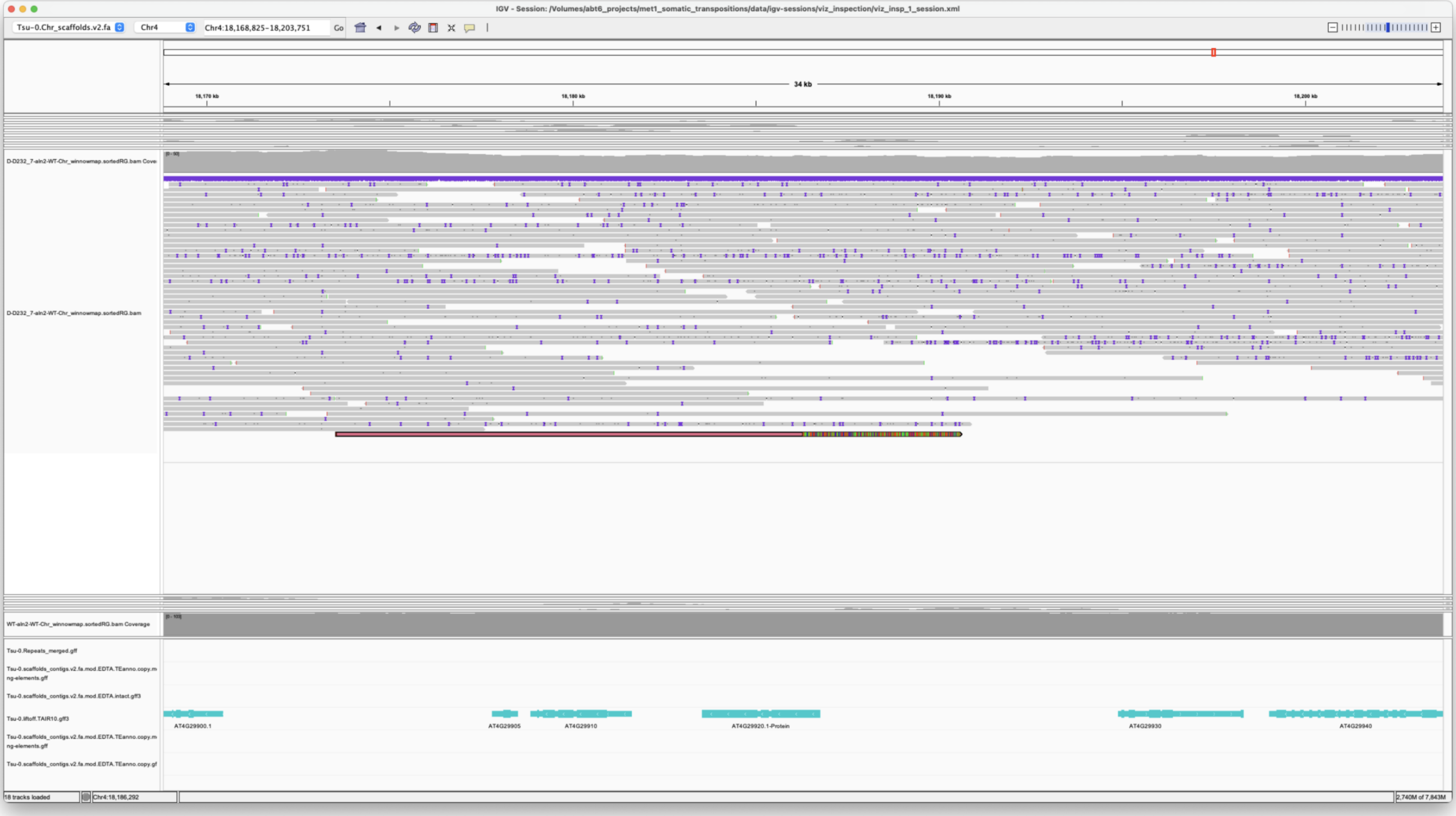

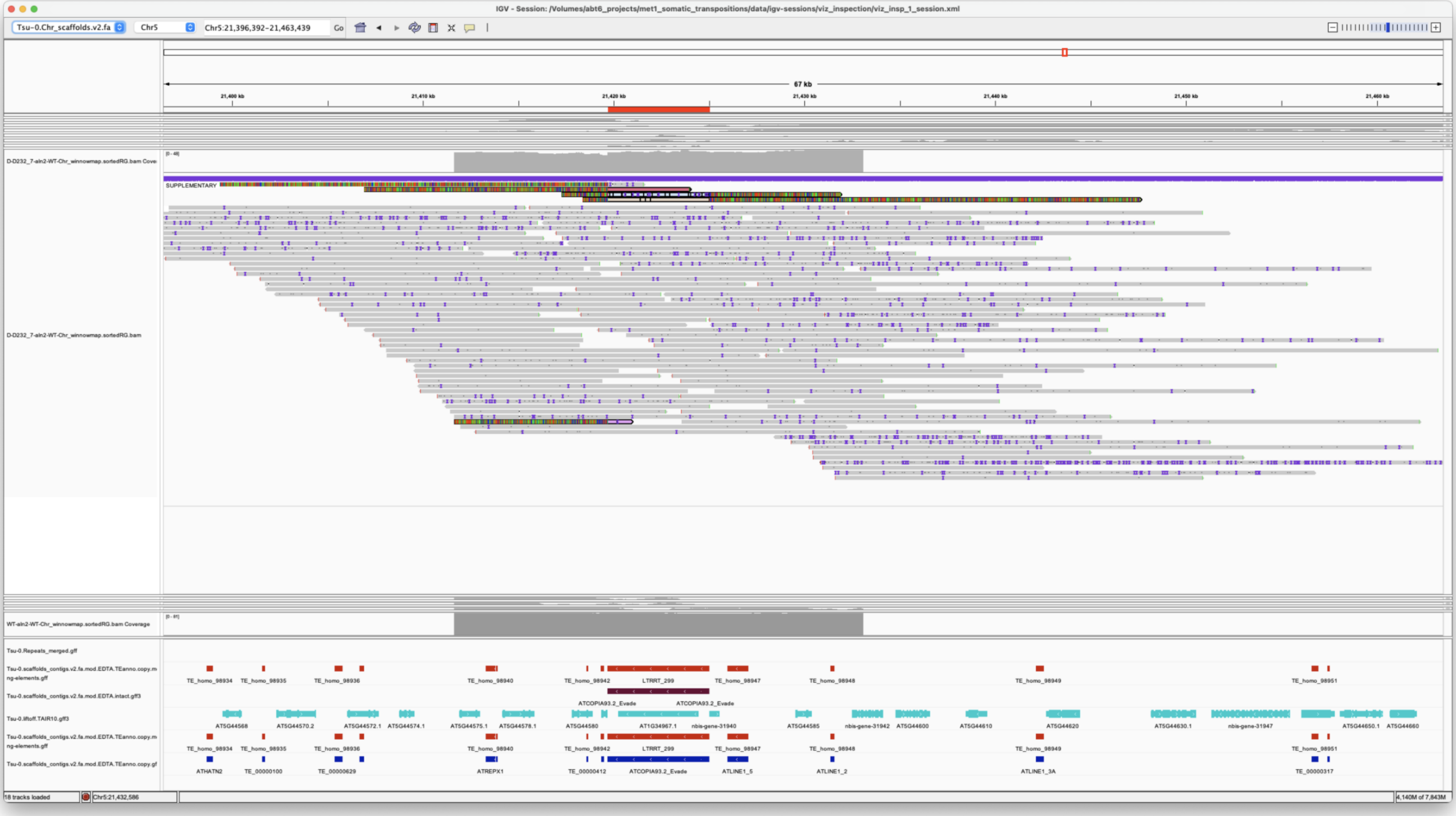

Partial

Confirmed

Chr4 21727886 21727886 - 1 Chr3;16344522;16352497;VANDAL6 m64079_221220_112036/139265760/ccs met1_07

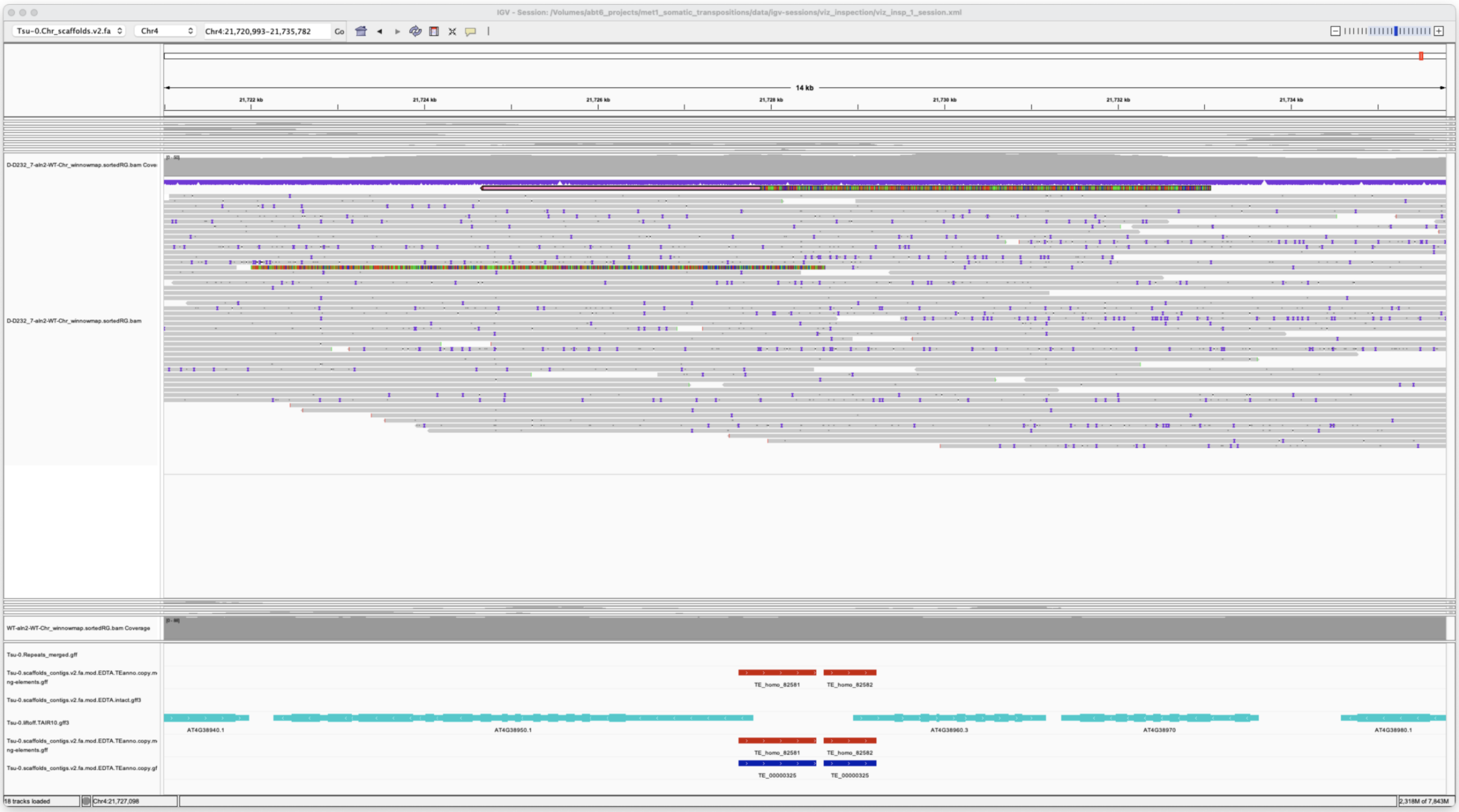

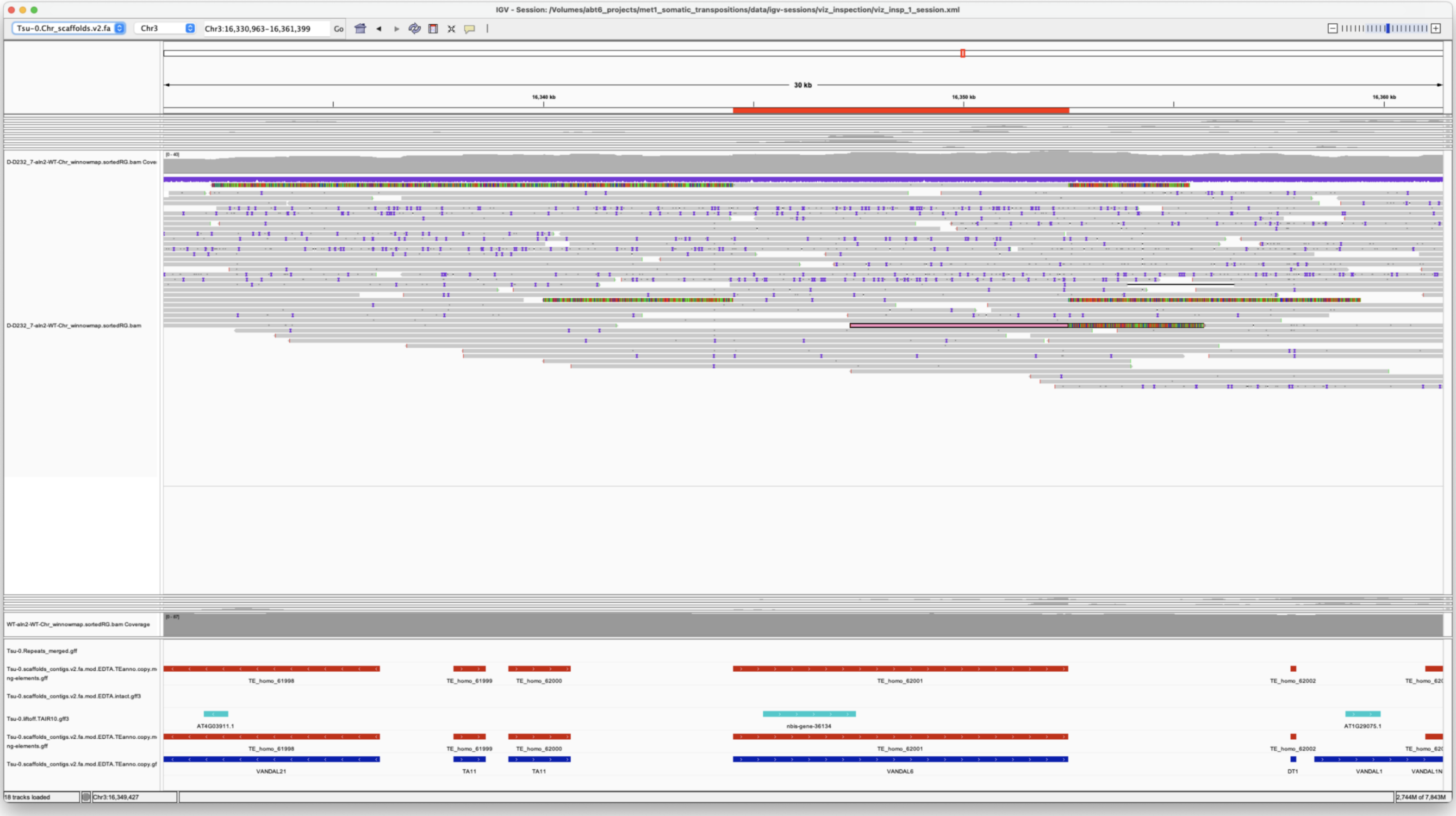

Independent transposition of a different starting VANDAL6 nearby

Partial

Confirmed

Chr4 21728624 21728624 - 1 Chr3;20158137;20166150;VANDAL6 m64079_240212_113350/55181977/ccs met1_07

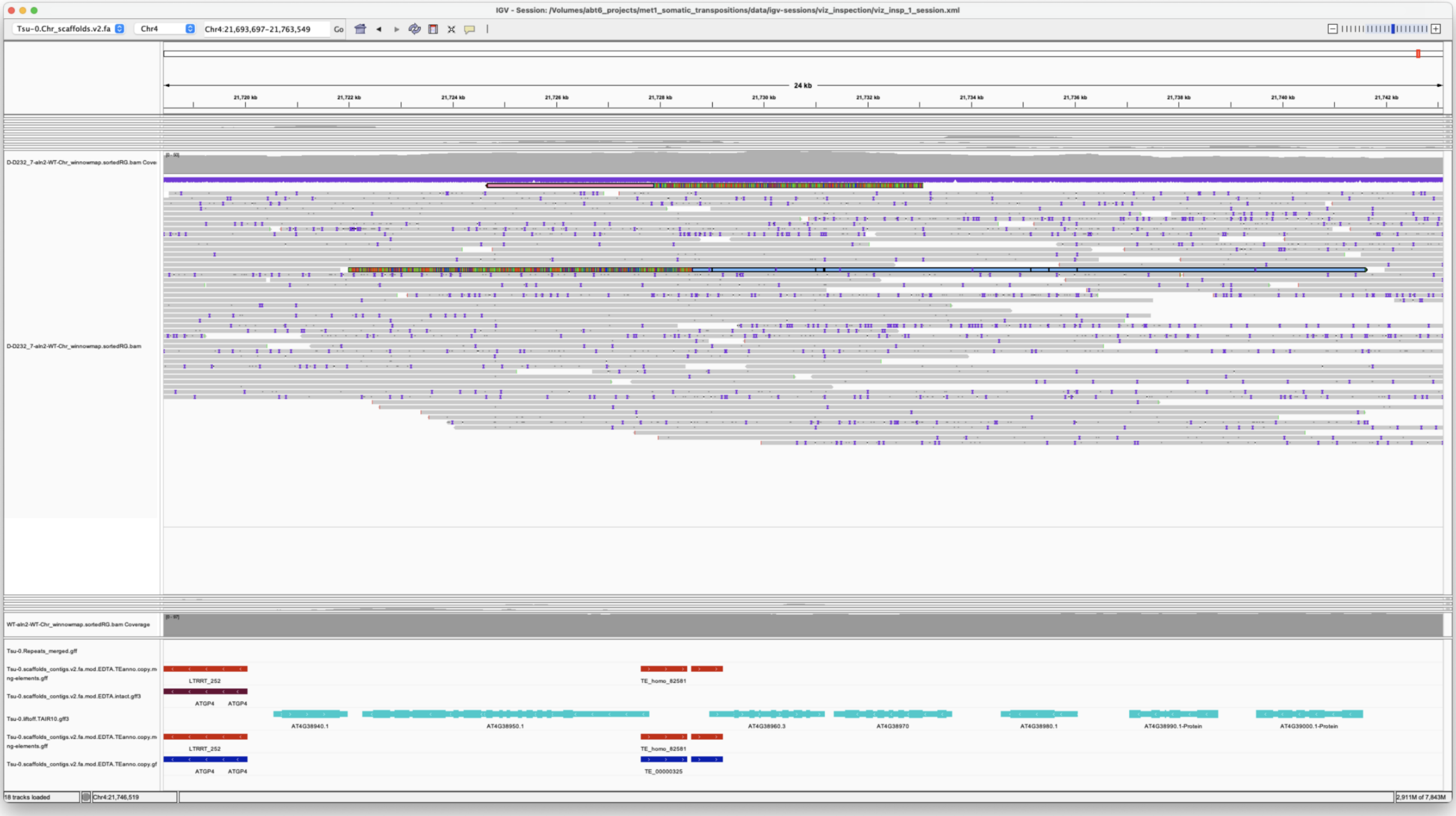

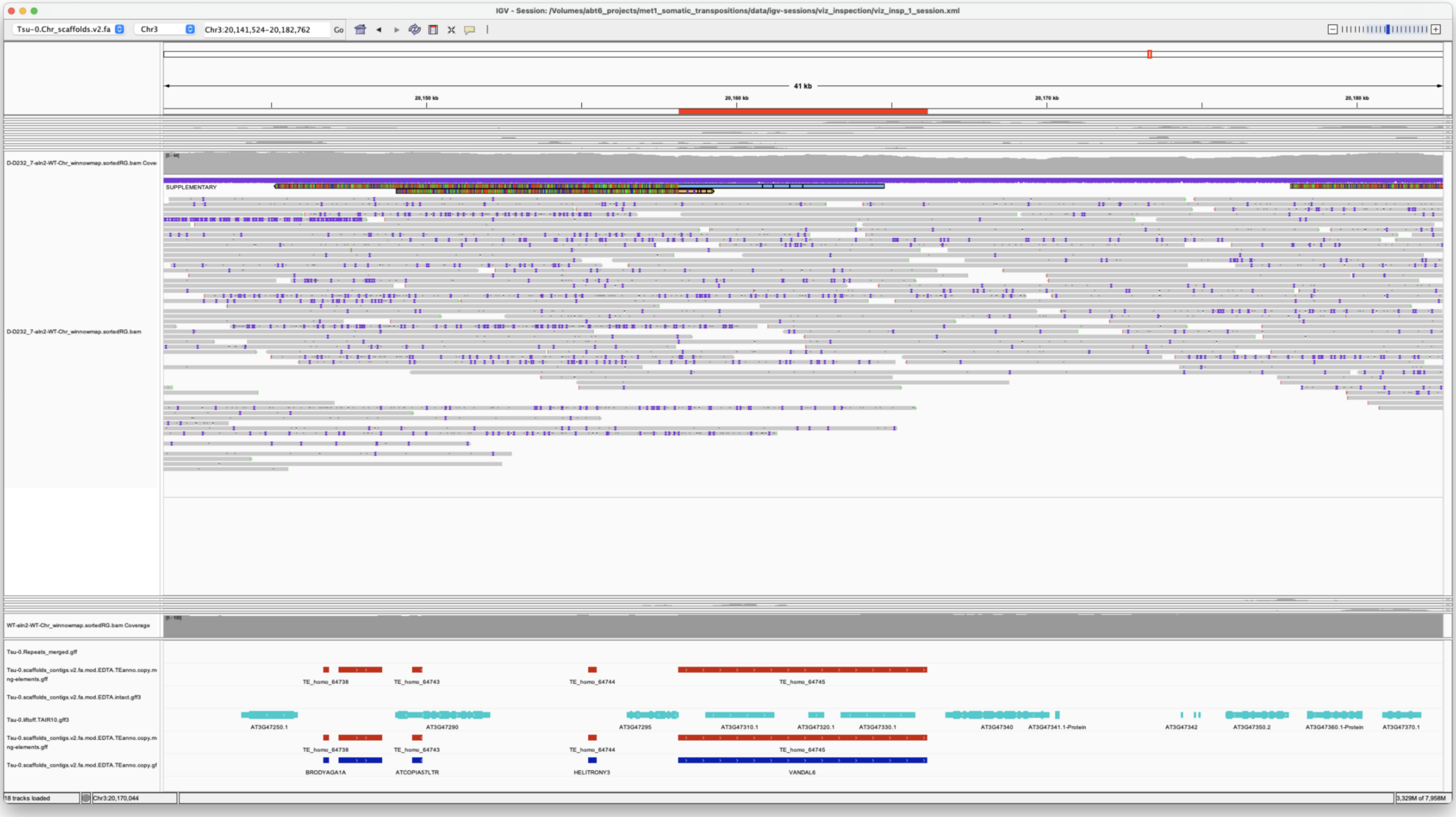

Independent transposition of a different starting VANDAL6 nearby

Partial

Confirmed

Chr5 1032011 1032011 - 1 Chr5;19152829;19160826;VANDAL21 m64079_240212_113350/38930783/ccs met1_07

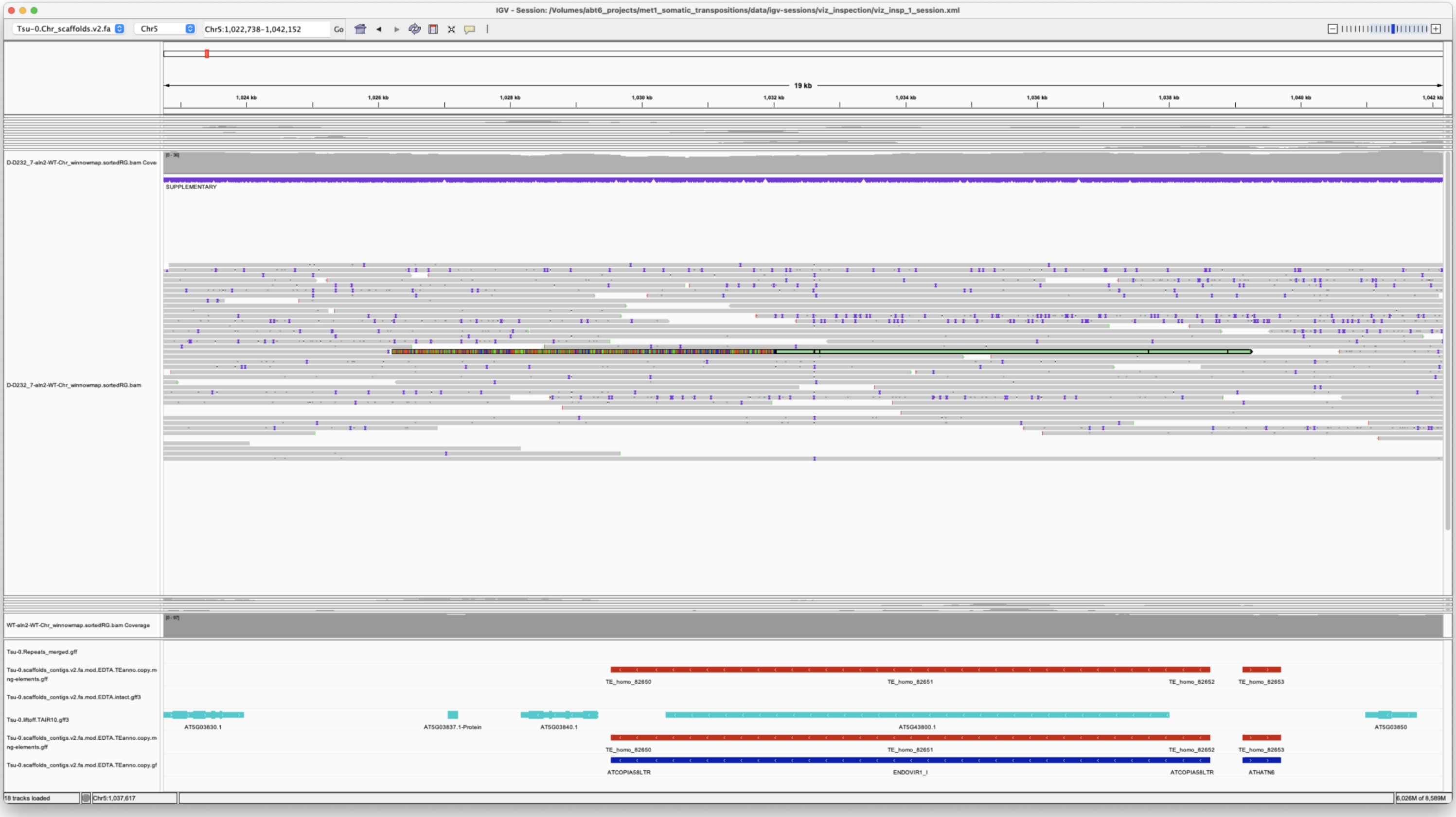

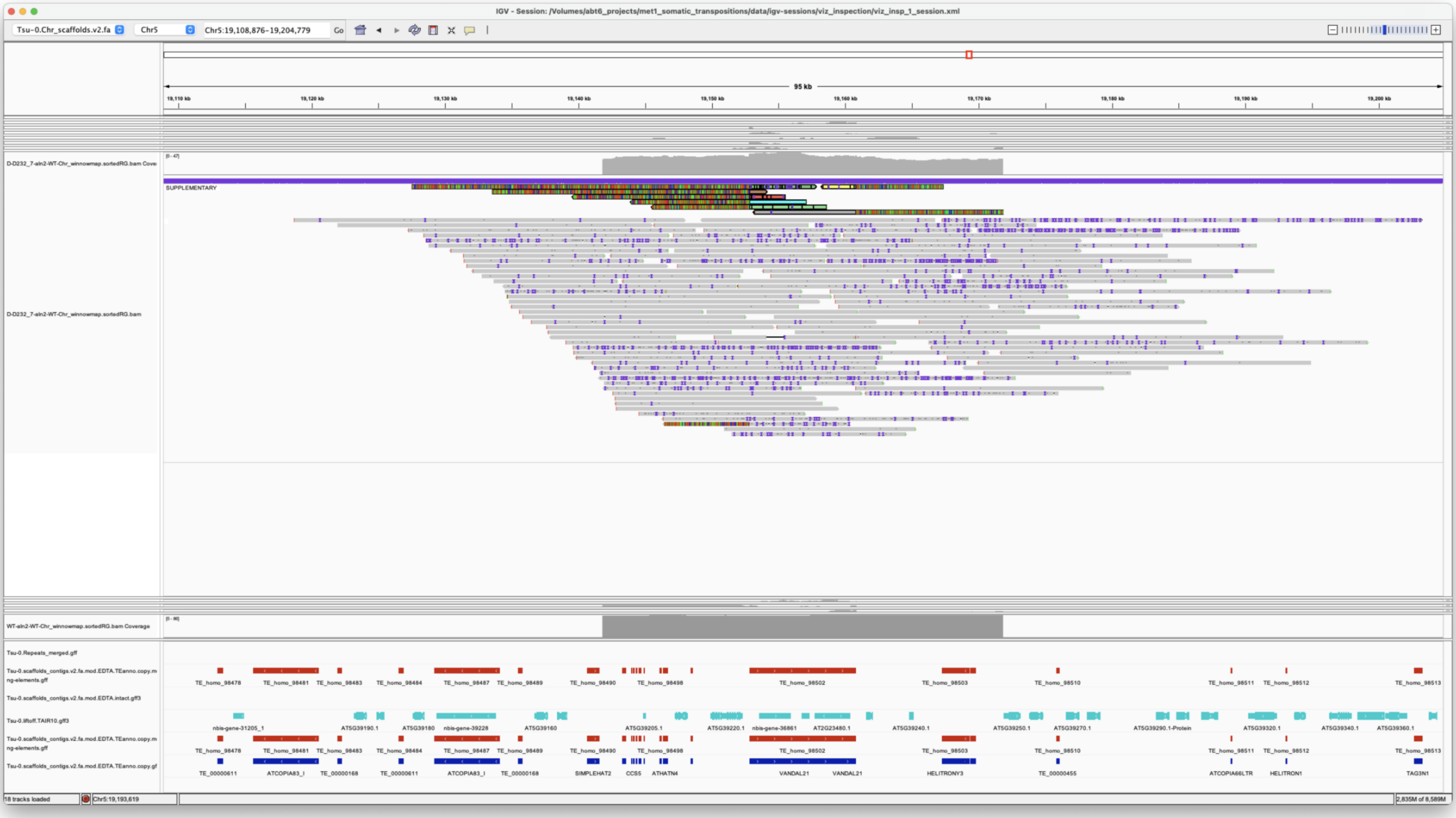

Partial

Confirmed

Chr5 2789752 2789752 + 1 Chr1;11941106;11946436;ATCOPIA93_Evade m64079_221220_112036/18680142/ccs met1_07

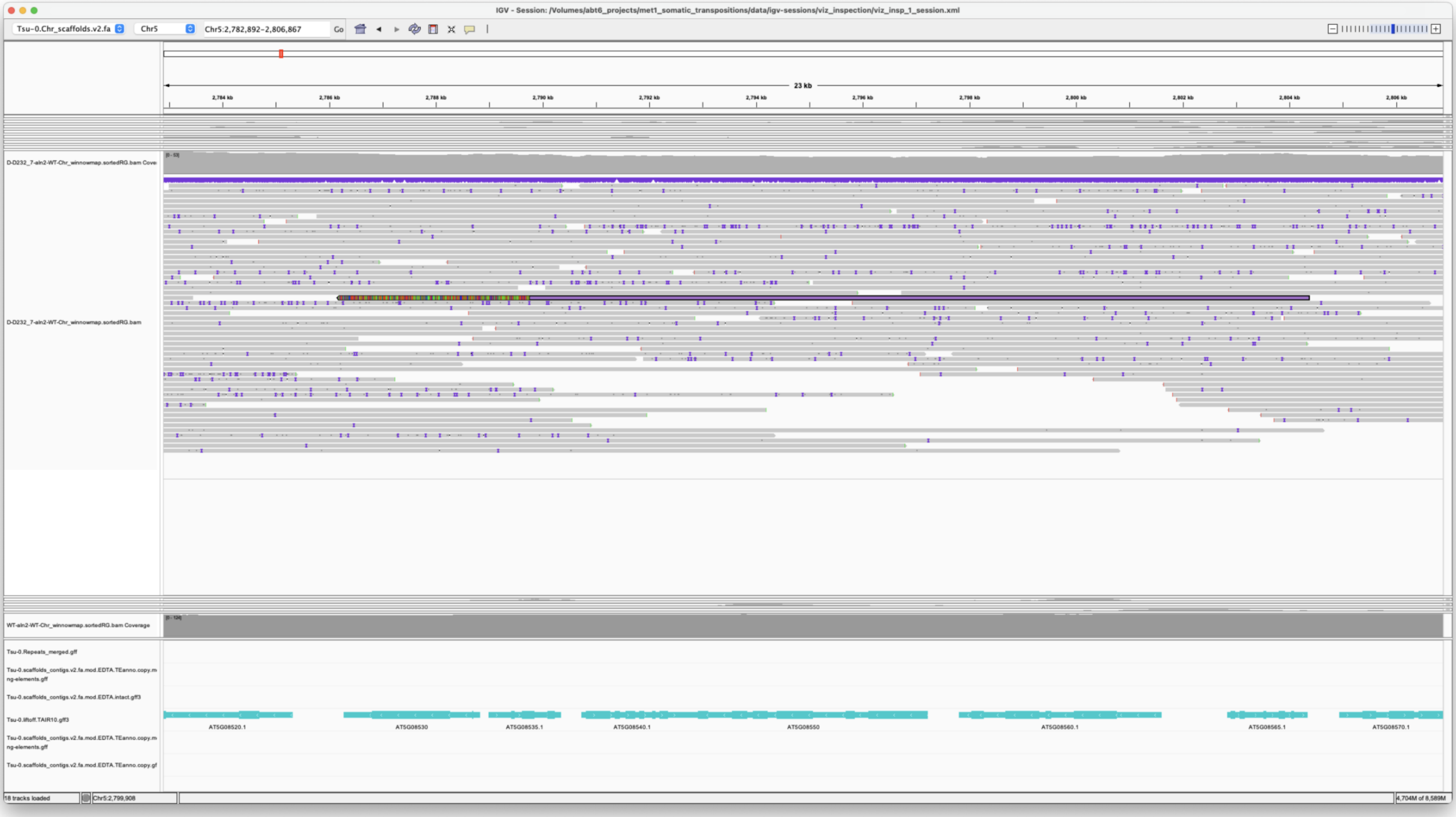

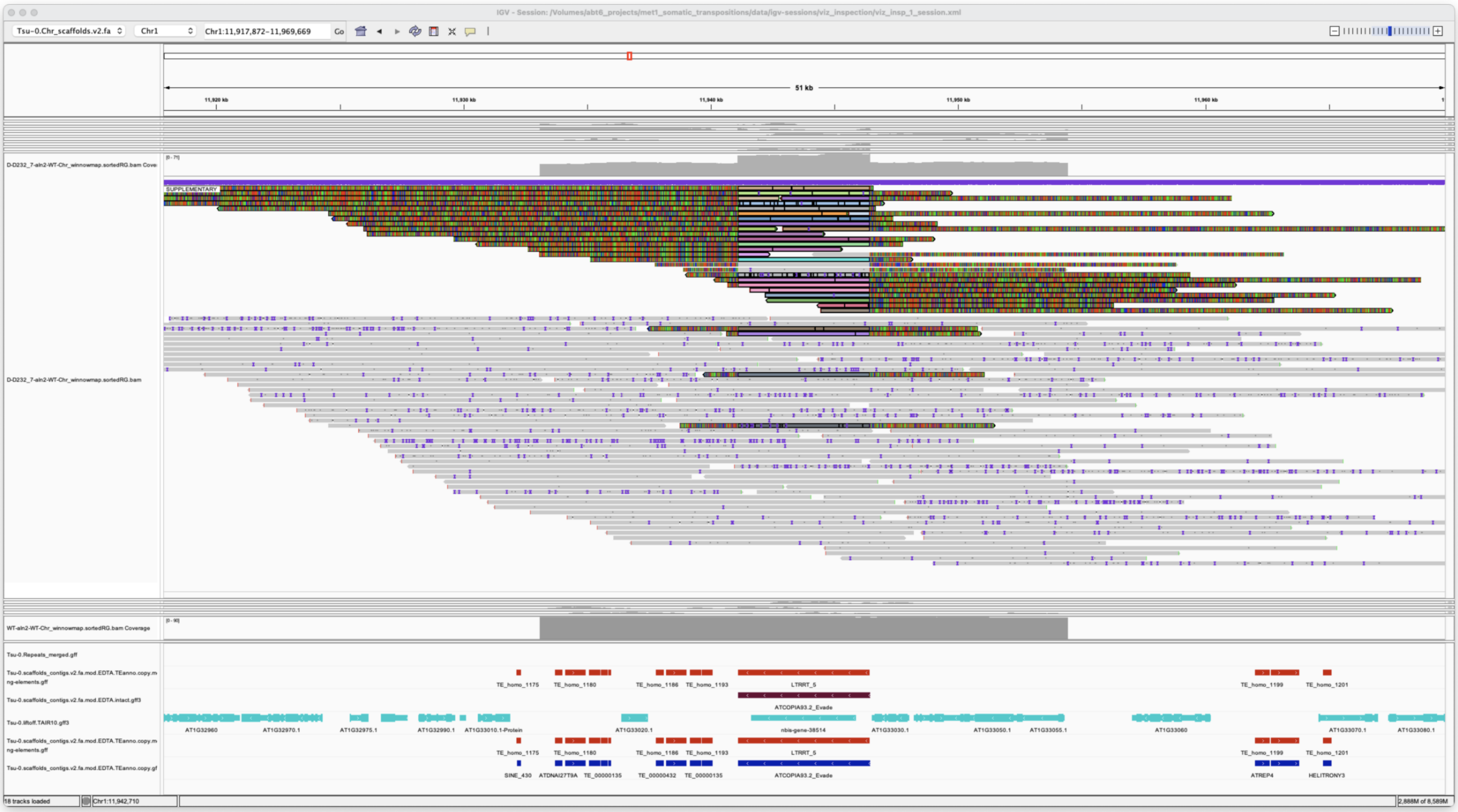

Partial

Confirmed

Chr5 10009344 10009344 + 1 Chr5;19152829;19160826;VANDAL21 m64079_240212_113350/69863891/ccs met1_07

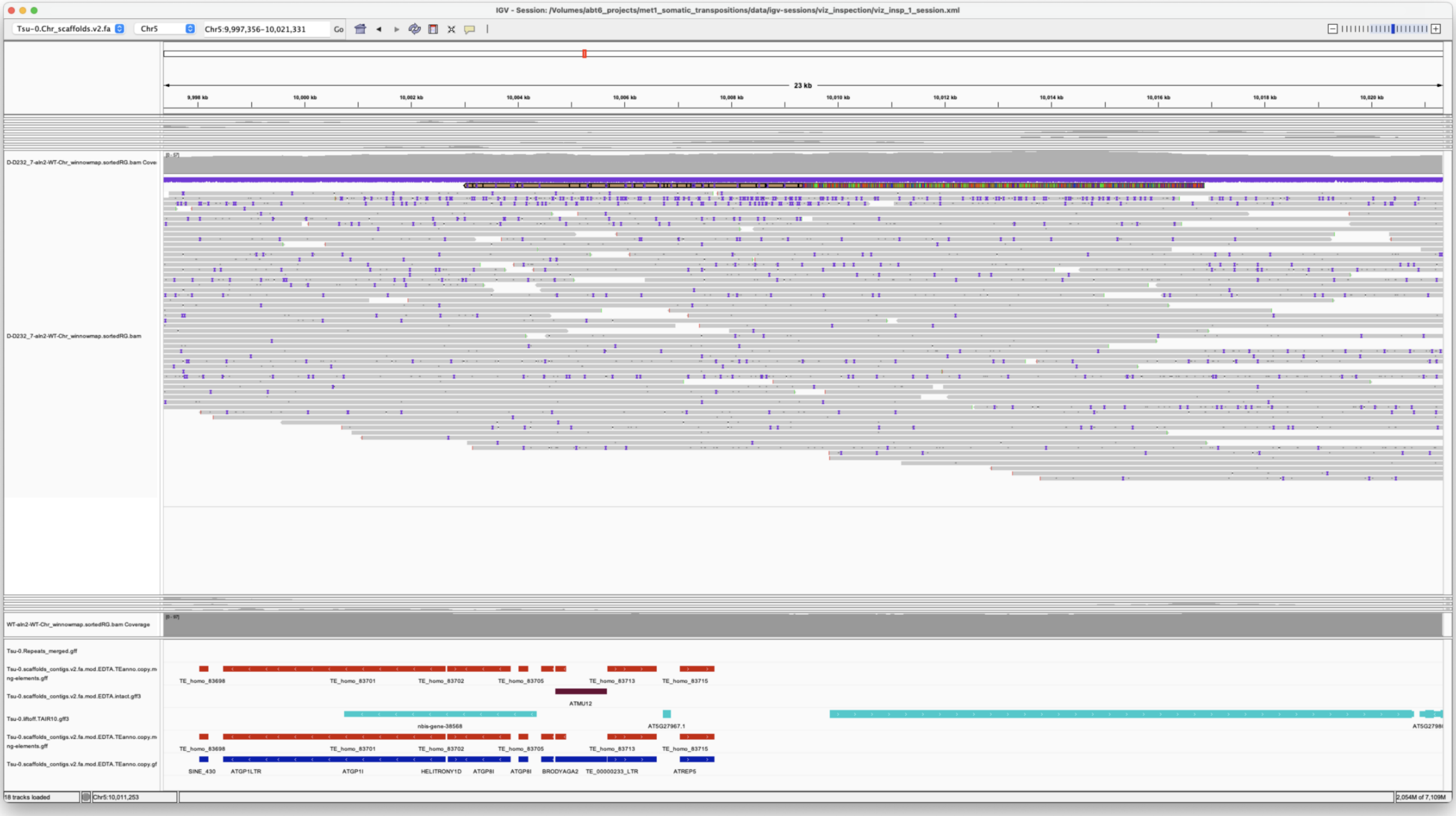

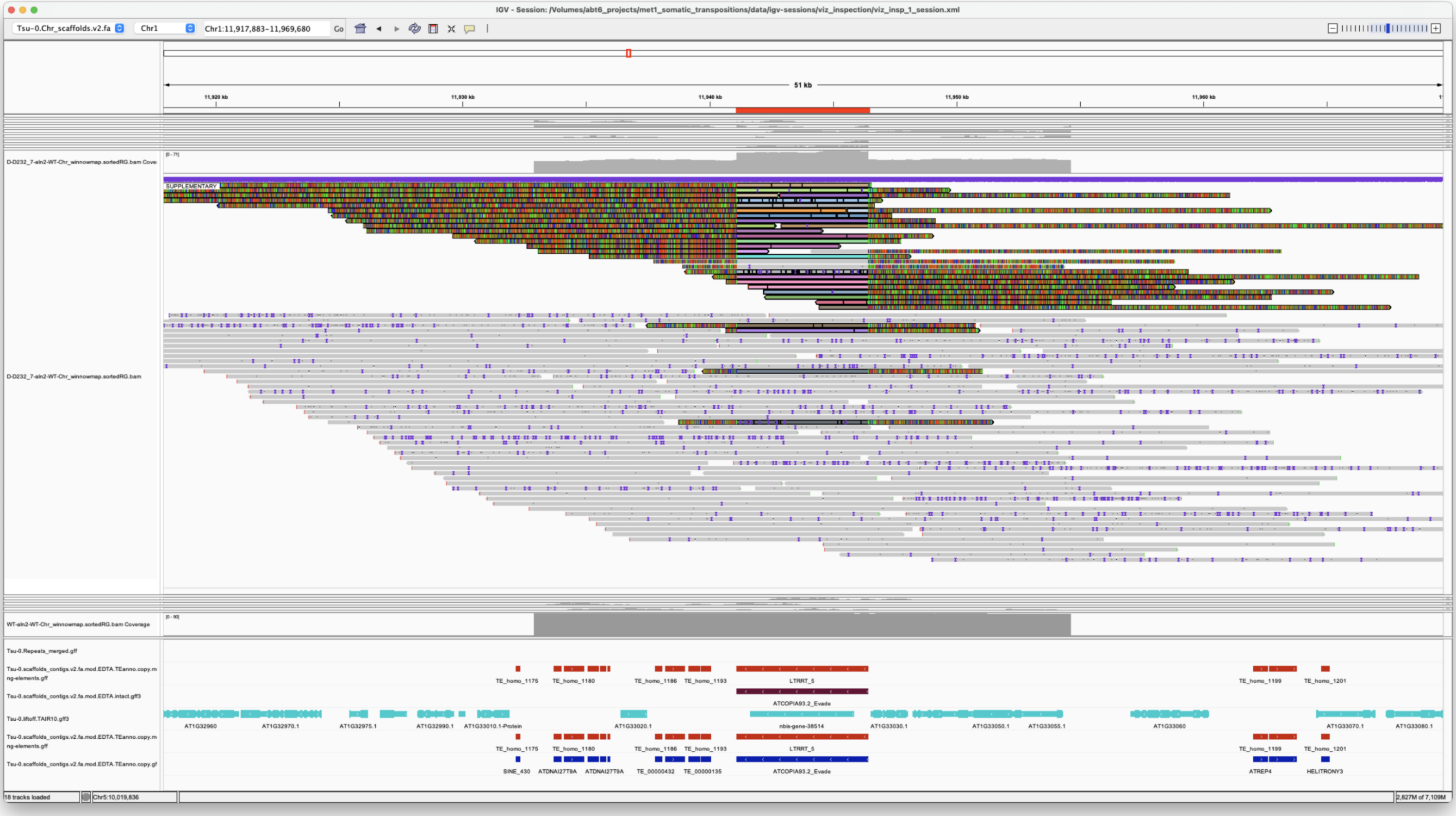

Partial

Confirmed

Chr5 10049542 10049542 + 1 Chr1;11941106;11946436;ATCOPIA93_Evade m64079_221220_112036/49875104/ccs met1_07

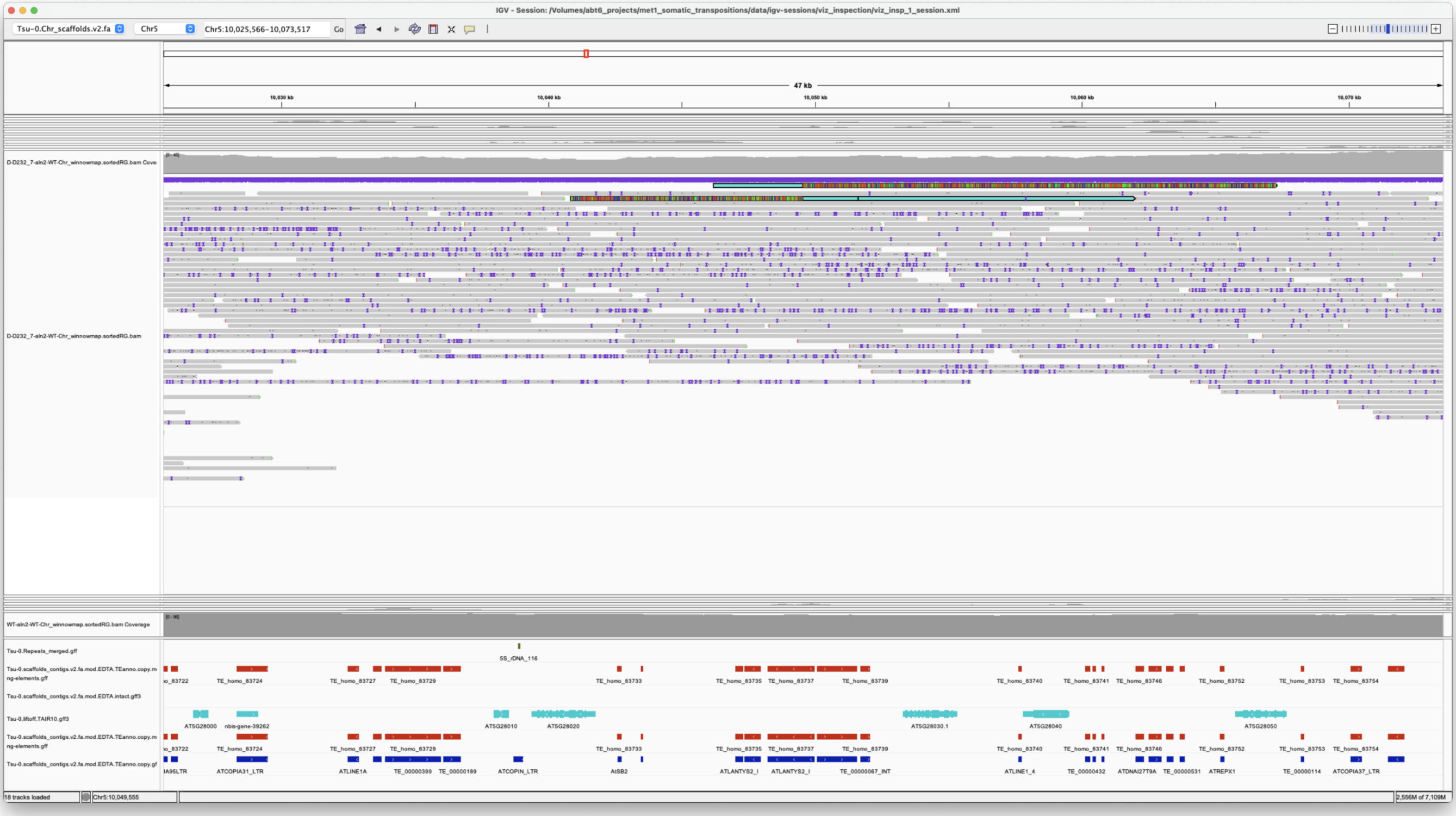

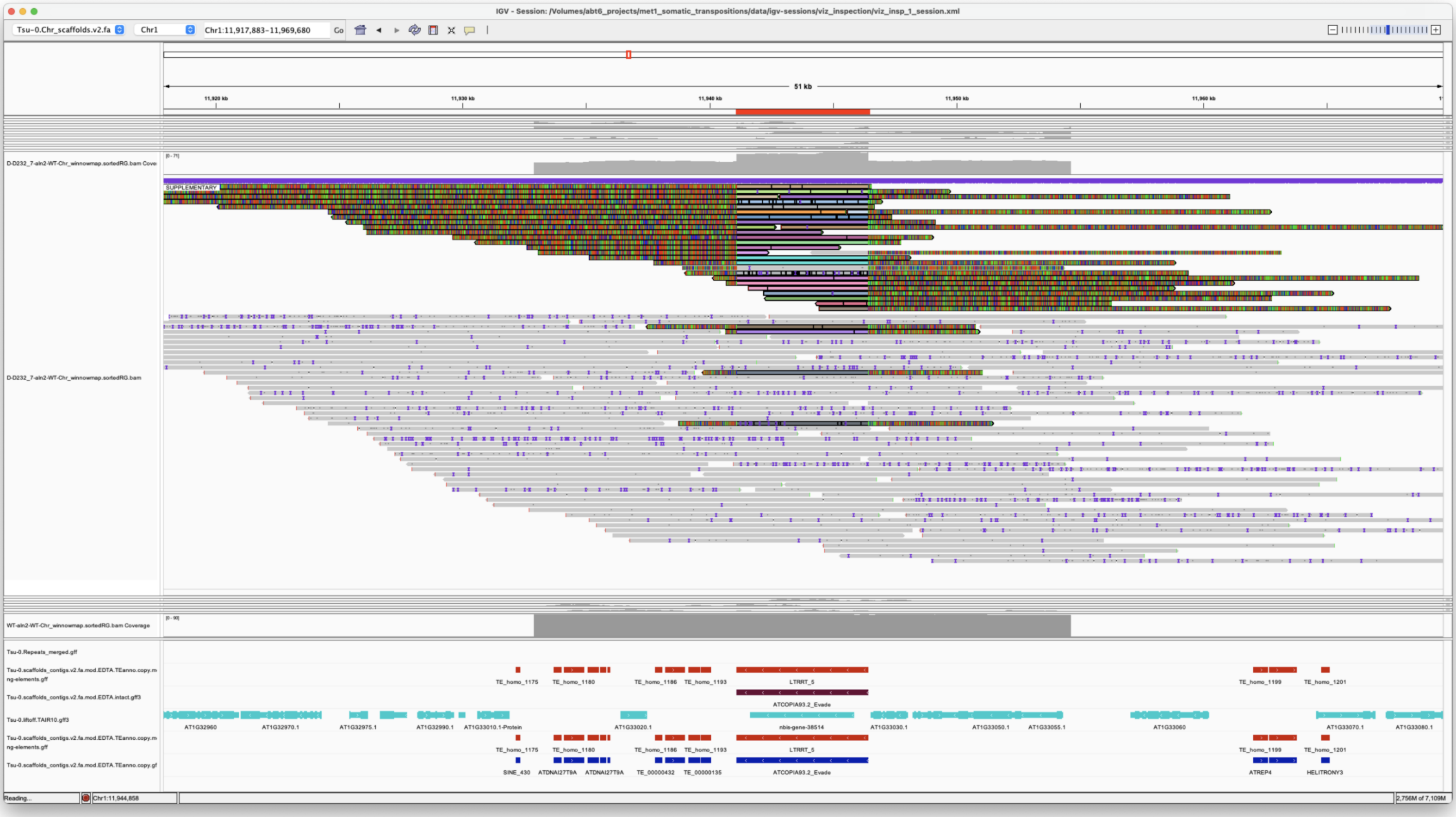

Central
TSD

Confirmed

Chr5 13503017 13503017 - 1 Chr1;11941106;11946436;ATCOPIA93_Evade m64079_221220_112036/106431763/ccs met1_07

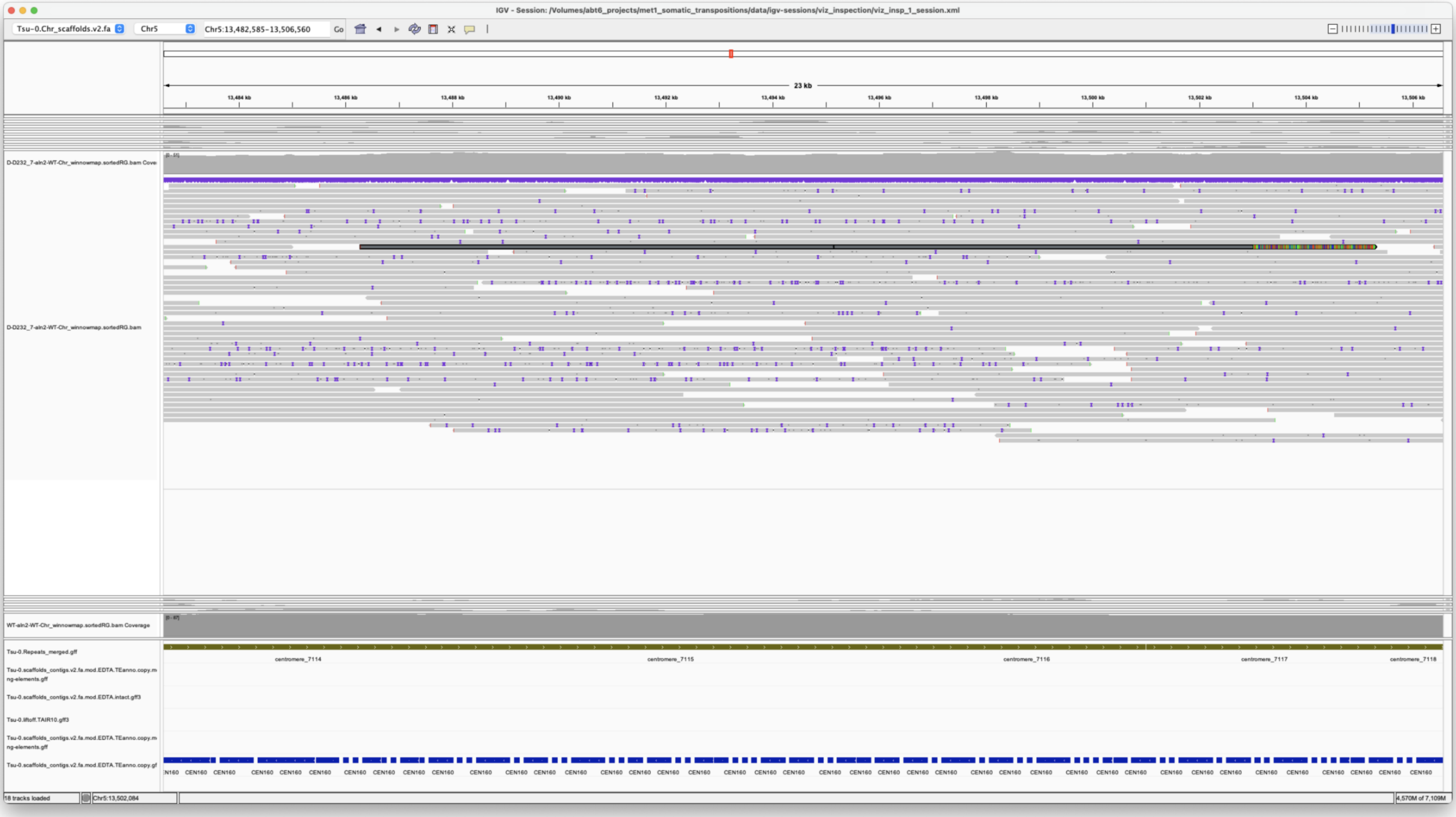

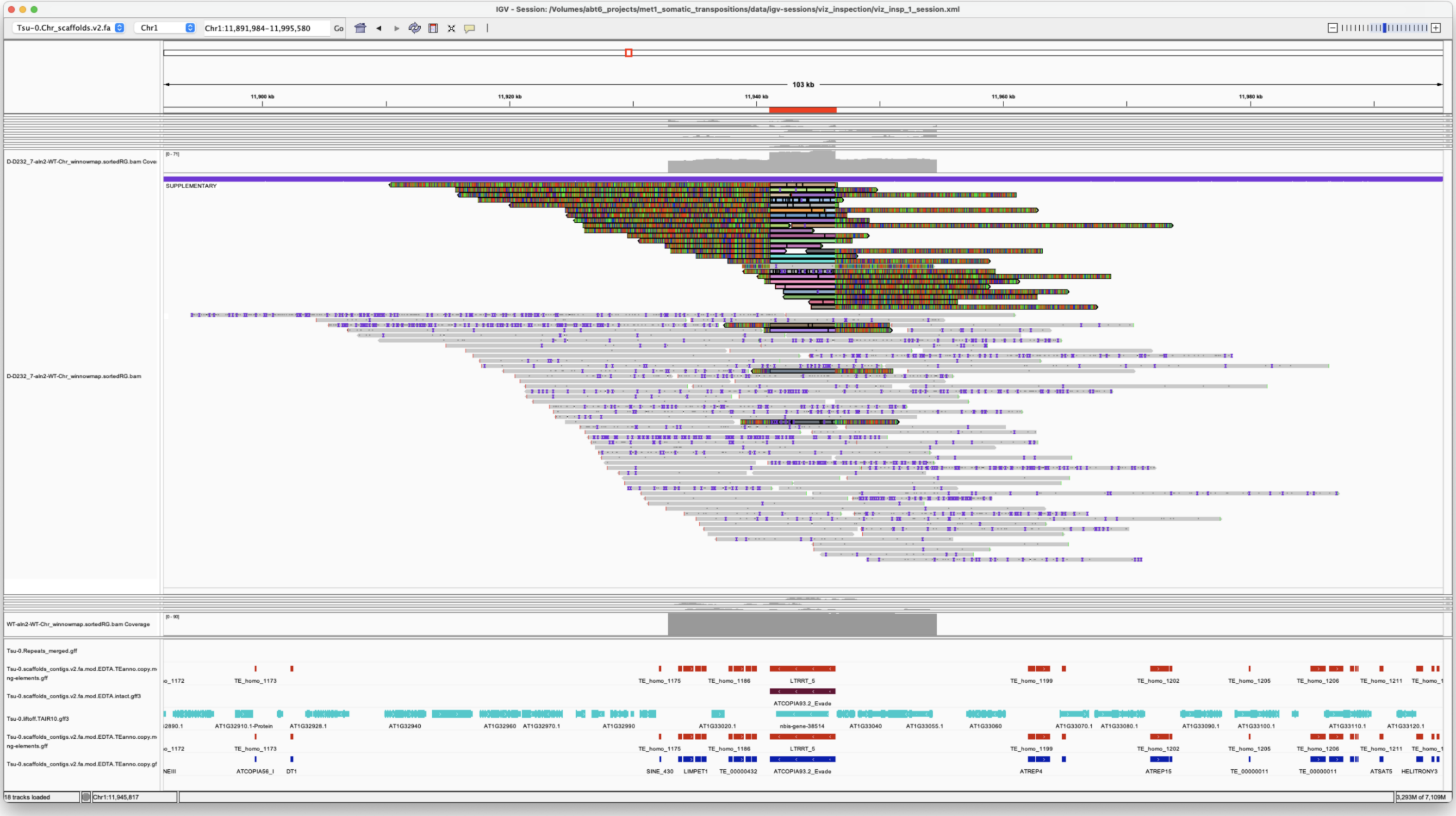

Centromeric

Partial

Confirmed

> Chr5 15617123 15617123 + 1 Chr5;19877283;19884298;VANDAL21 m64079_221220_112036/152896694/ccs met1_07

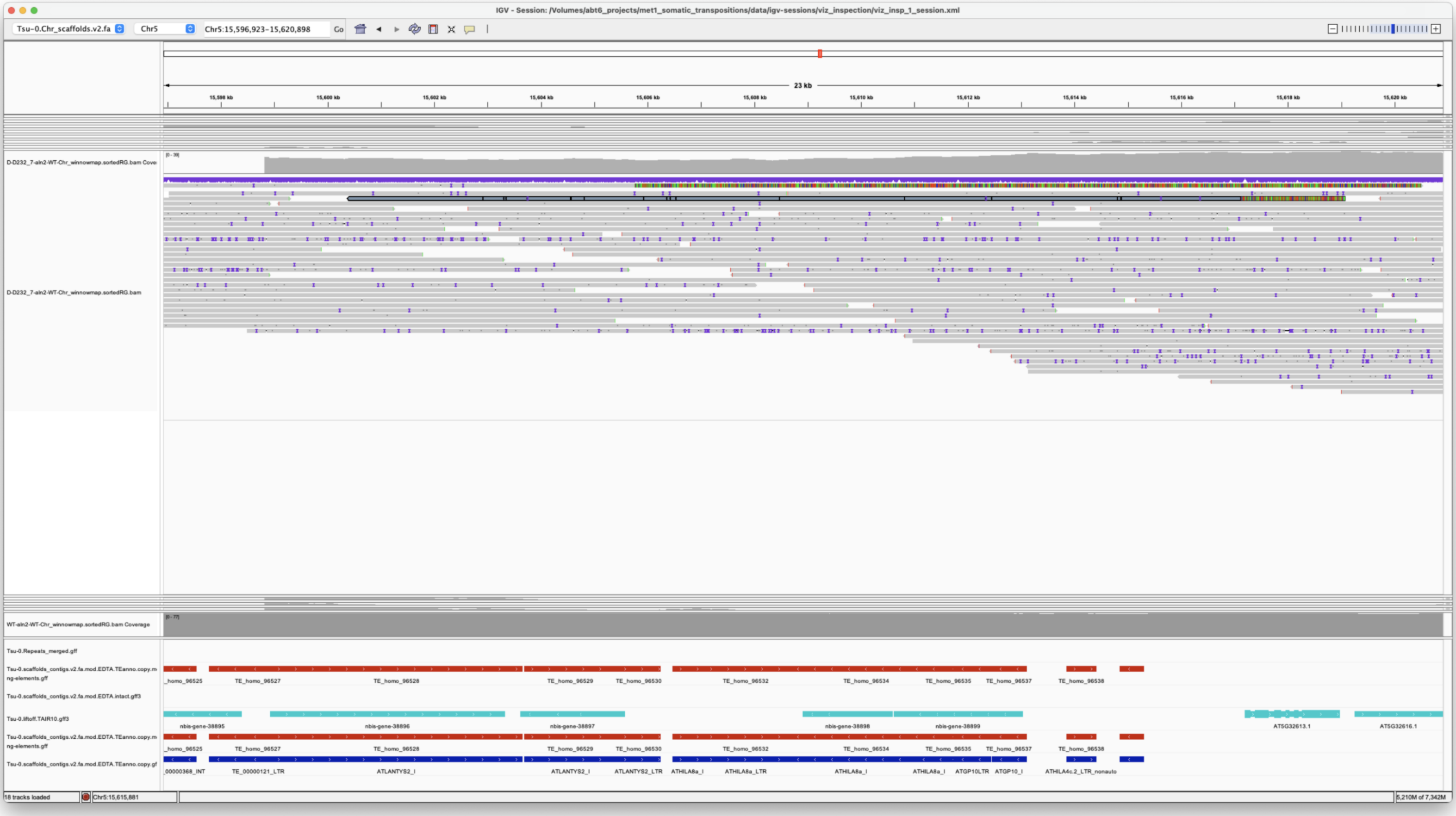

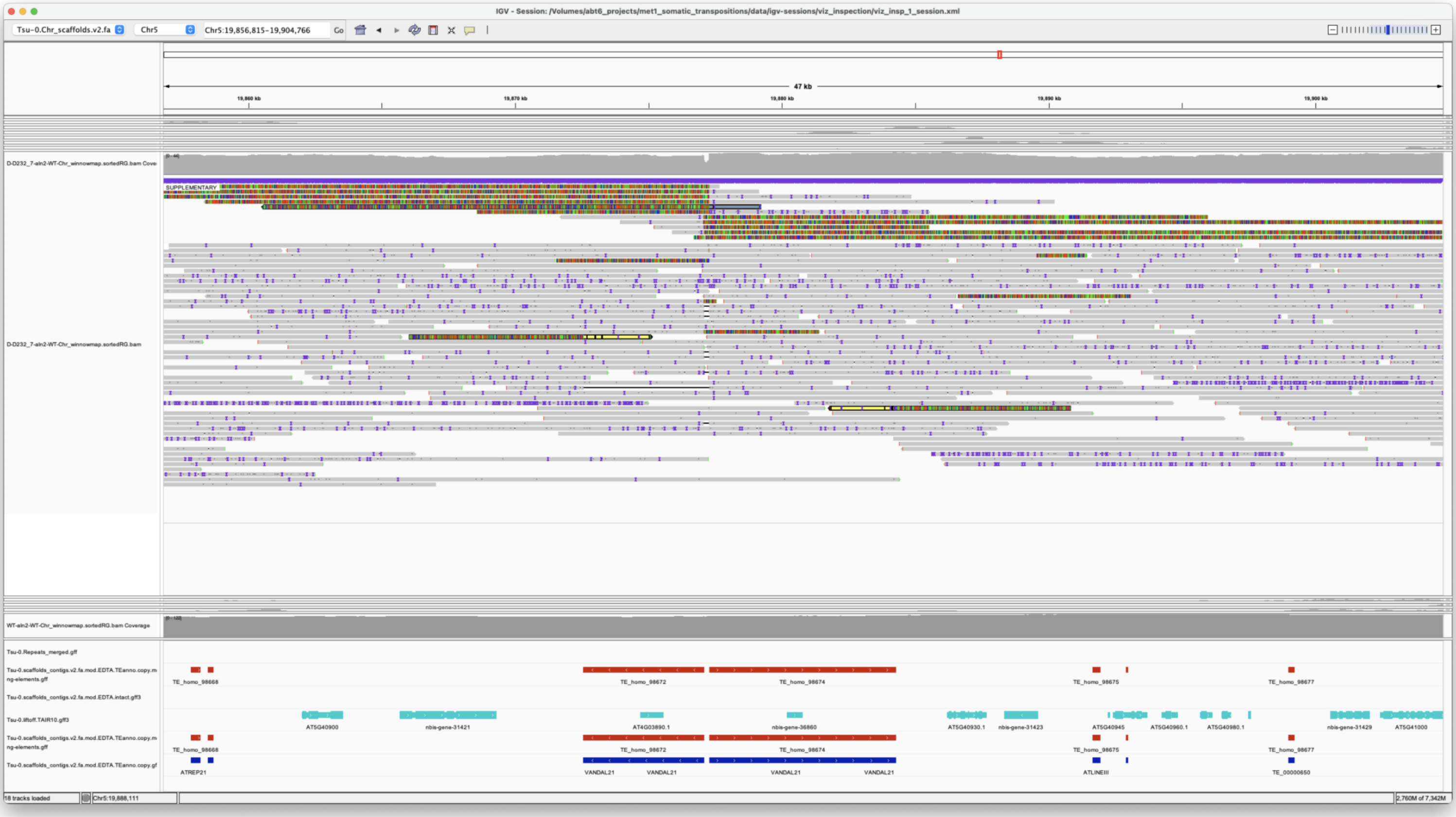

Hypermutable region

Rearrangement

unsupported

> Chr5 19405545 19405545 - 1 Chr5;19872565;19877095;VANDAL21 m64079_240212_113350/62325150/ccs met1_07
>
> Chr5 19889563 19889563 - 1 Chr5;19872565;19877095;VANDAL21 m64079_240212_113350/5374539/ccs met1_07
>
> Chr5 22409994 22409994 + 1 Chr5;19877283;19884298;VANDAL21 m64079_240212_113350/151914089/ccs met1_07

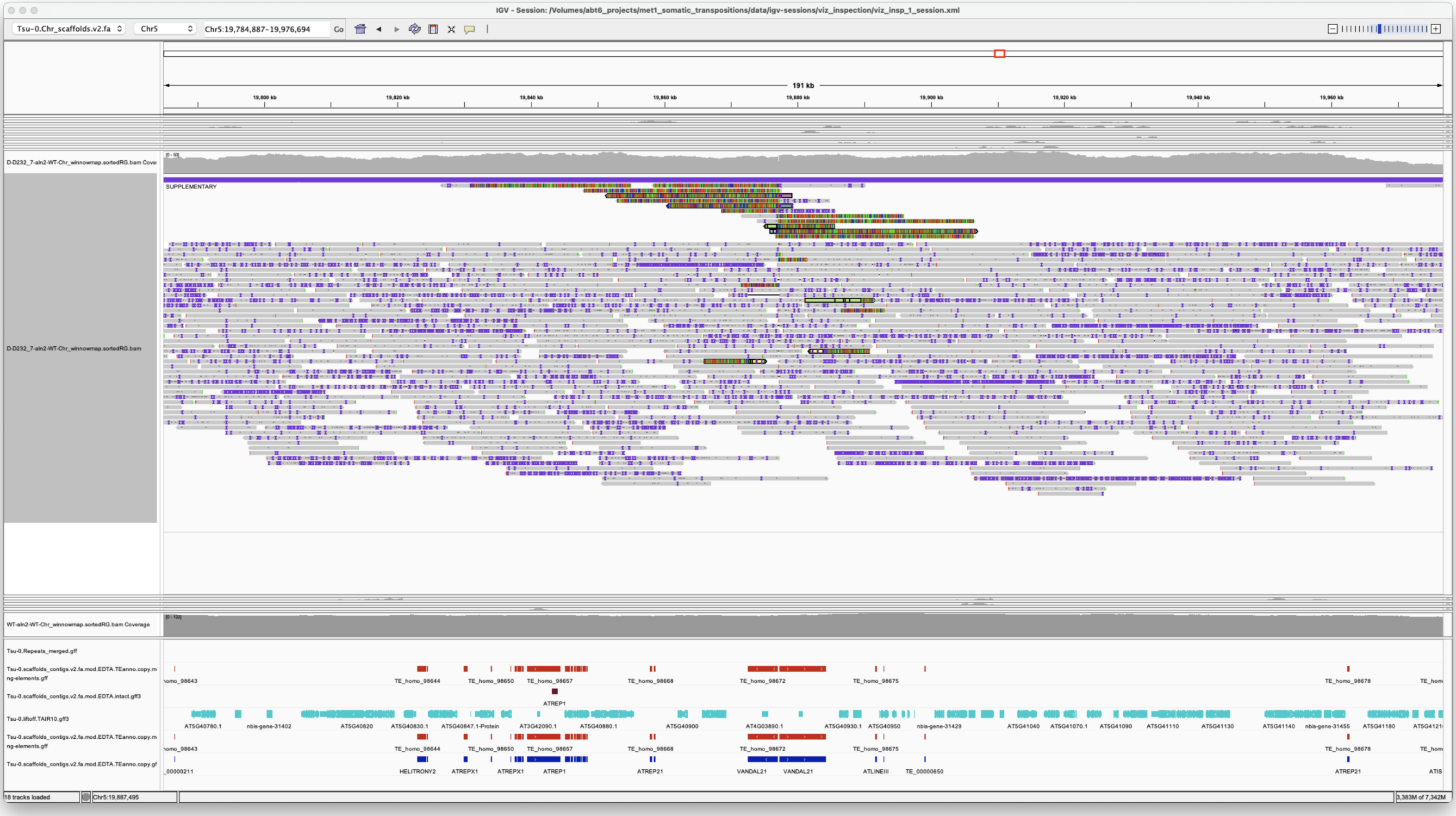

Hypermutable region

OTHER REARRANGEMENTS

unsupported

Chr5 28025720 28025720 + 1 Chr3;16344522;16352497;VANDAL6 m64079_240212_113350/158138475/ccs met1_07

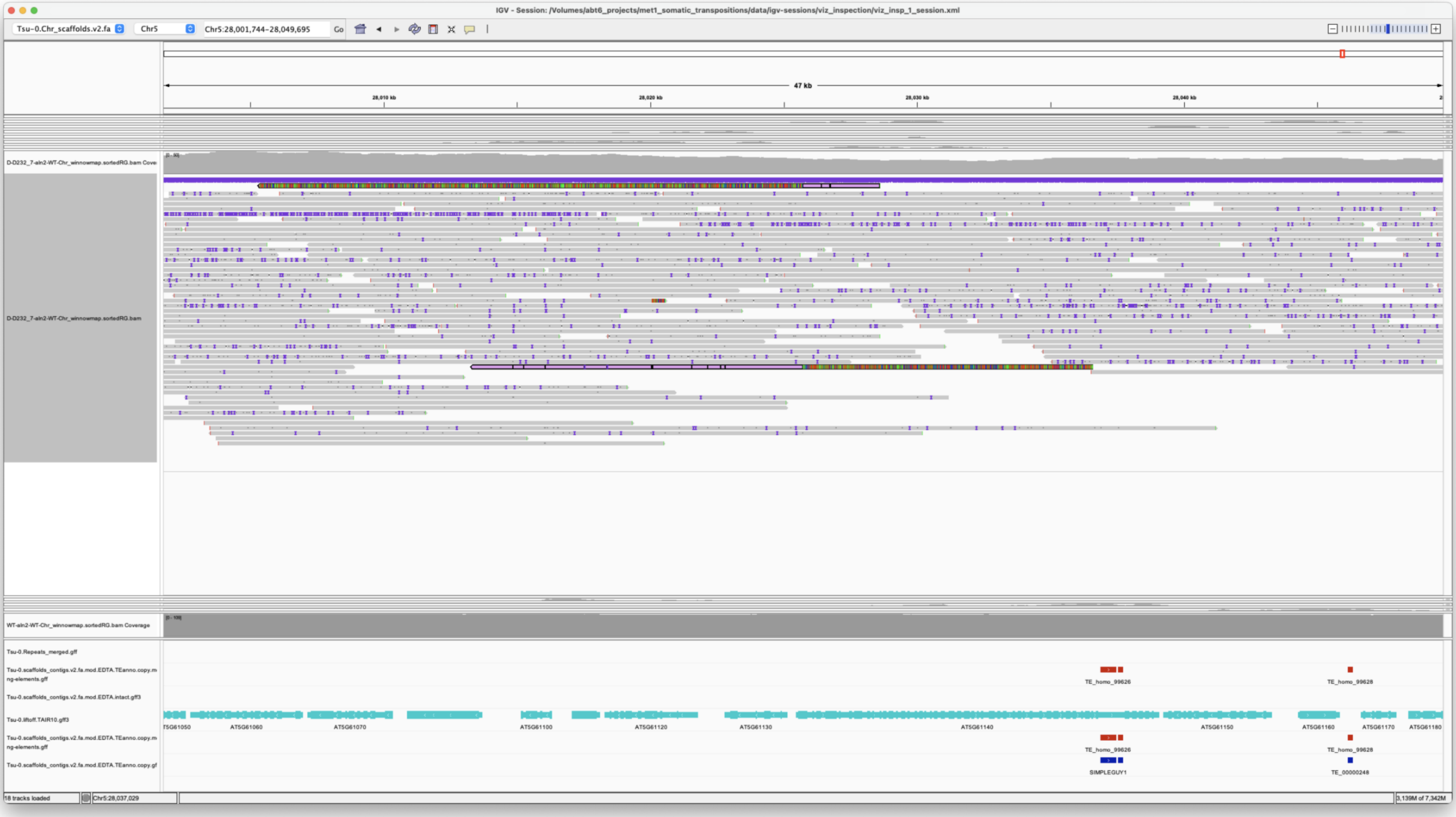

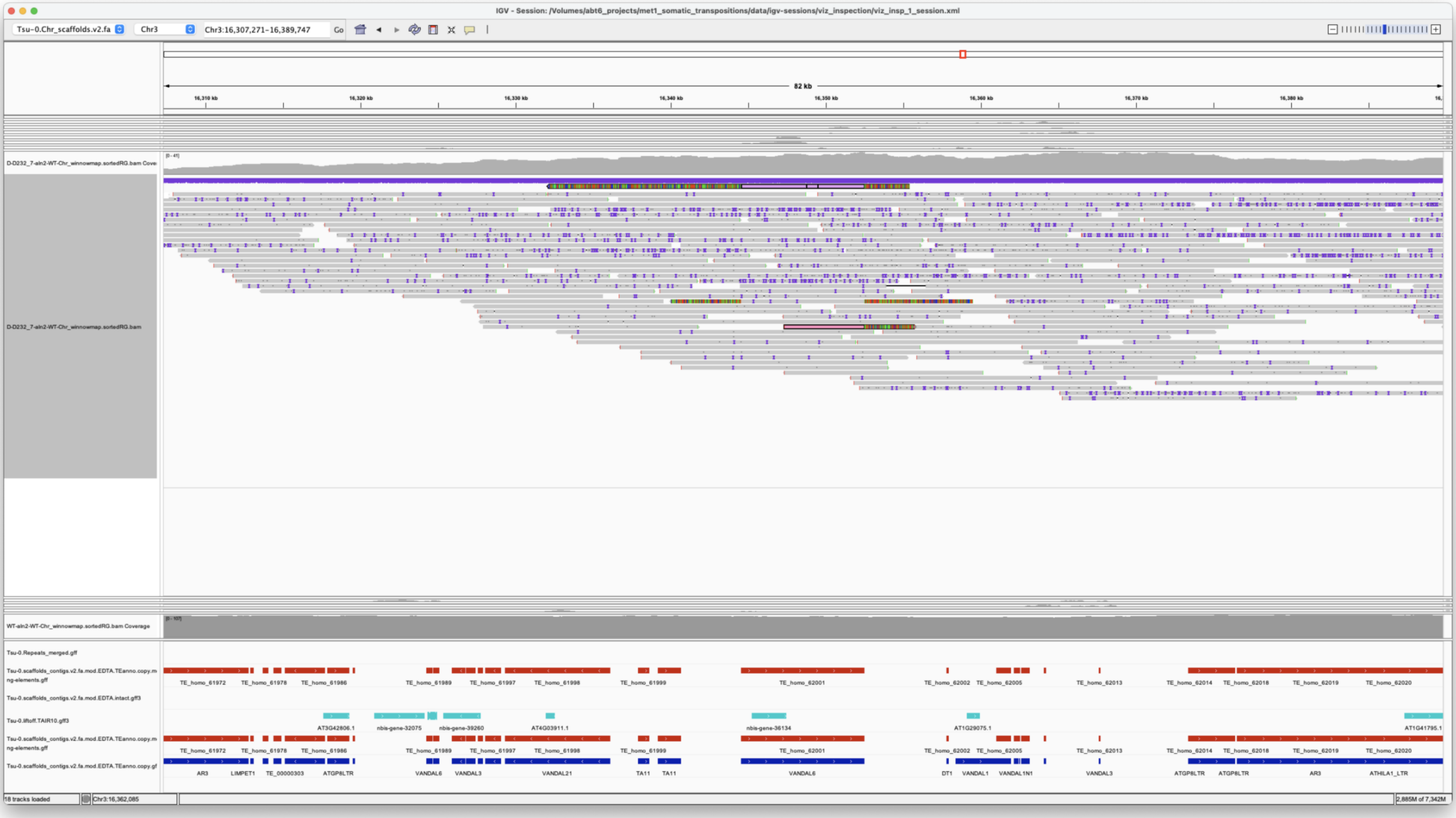

Central
TSD

Confirmed

---

met1_08

Chr1 2001748 2001748 + 1 Chr5;19152829;19160826;VANDAL21 m64079_240212_113350/12256956/ccs met1_08

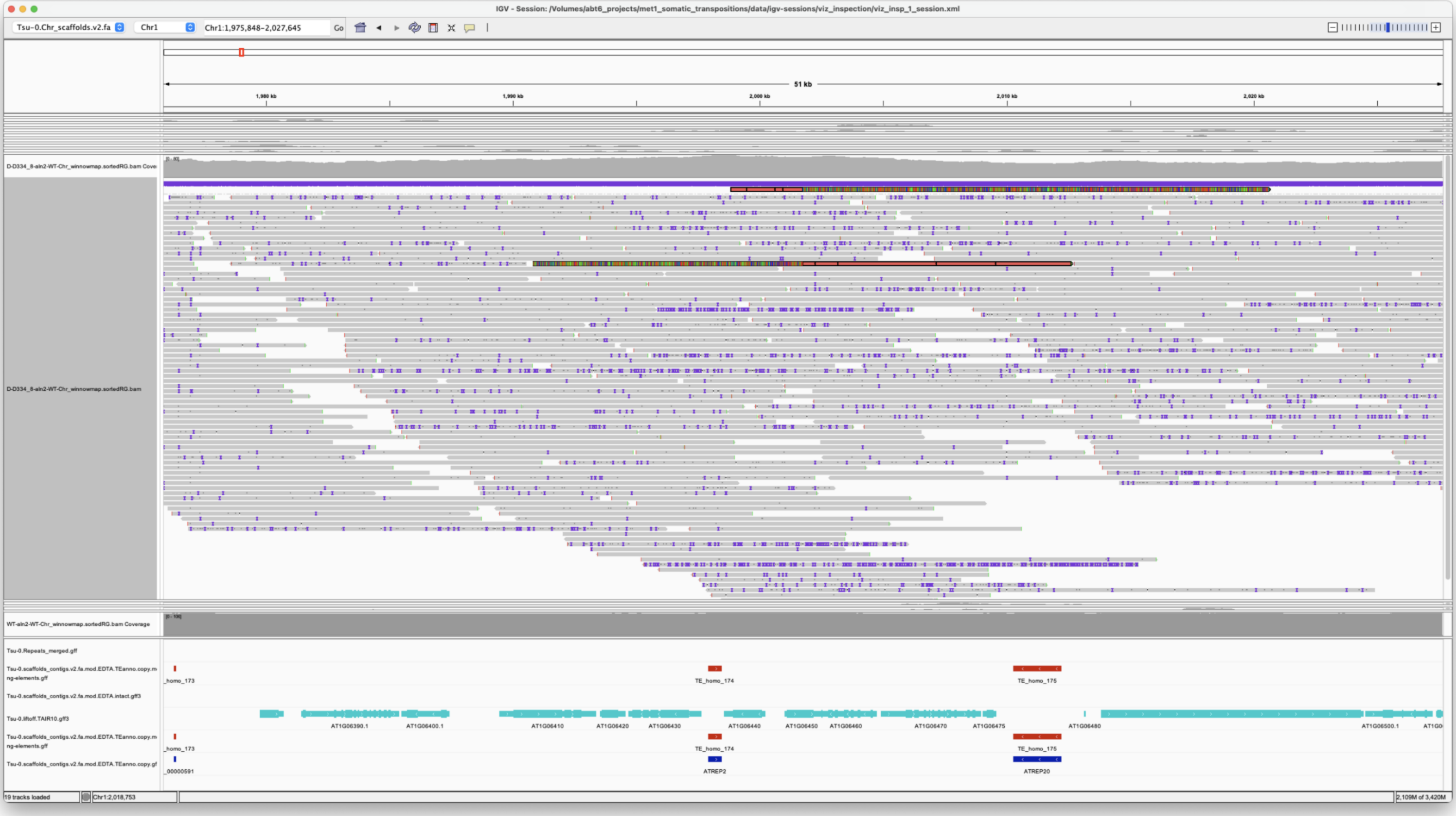

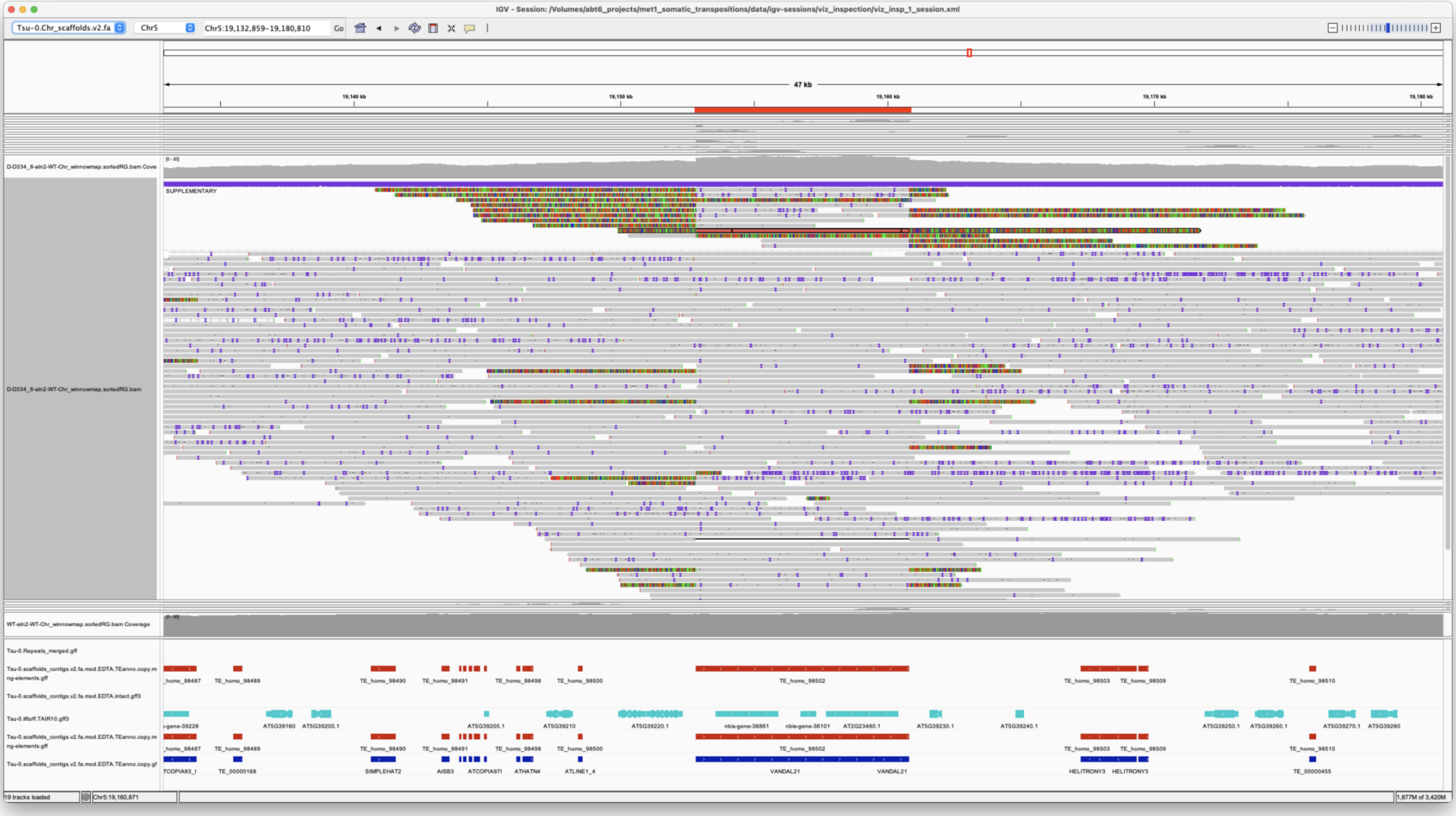

Central
TSD

Confirmed

Chr1 2152817 2152817 - 1 Chr3;20158137;20166150;VANDAL6 m64079_221220_112036/29884628/ccs met1_08

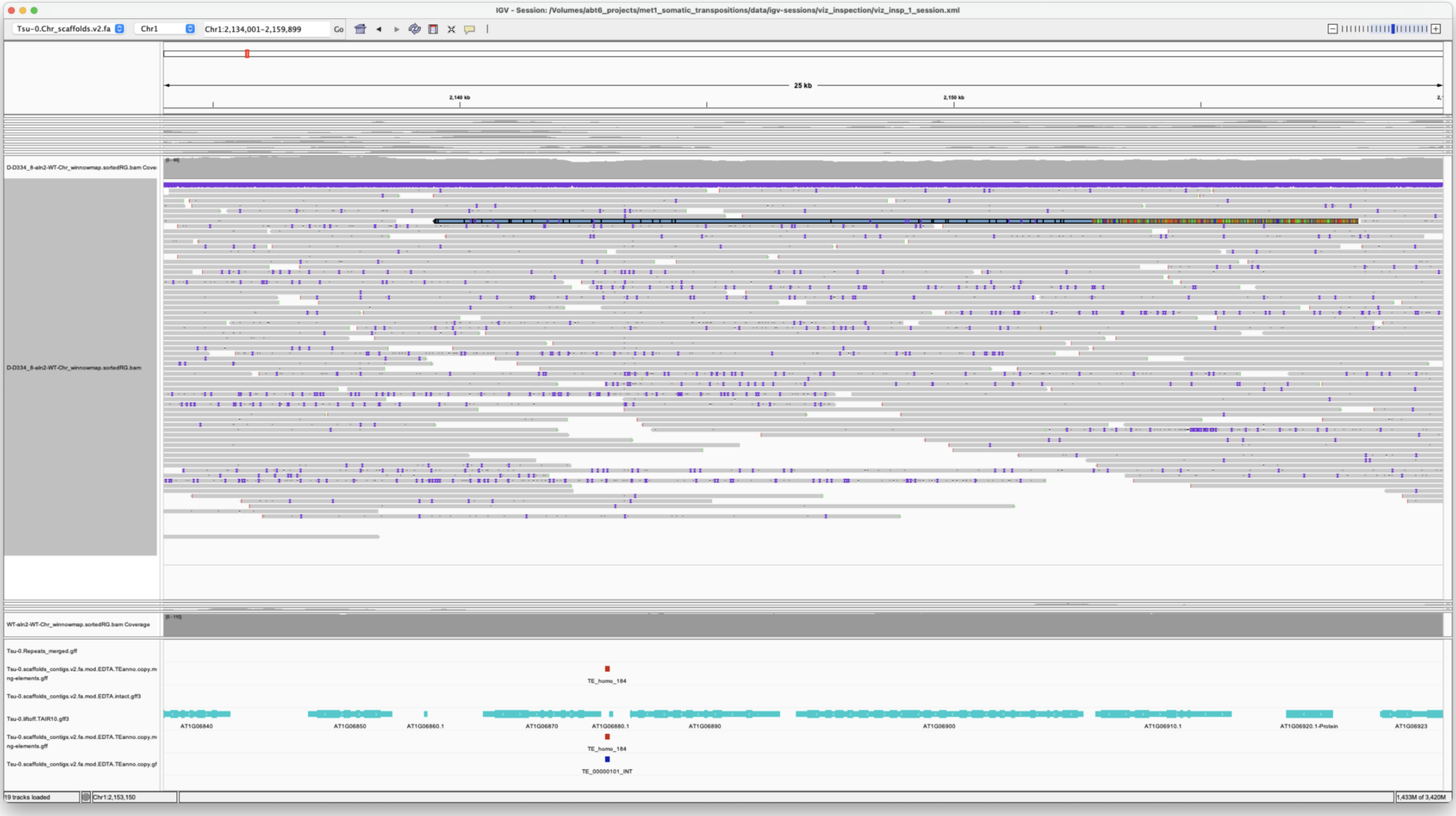

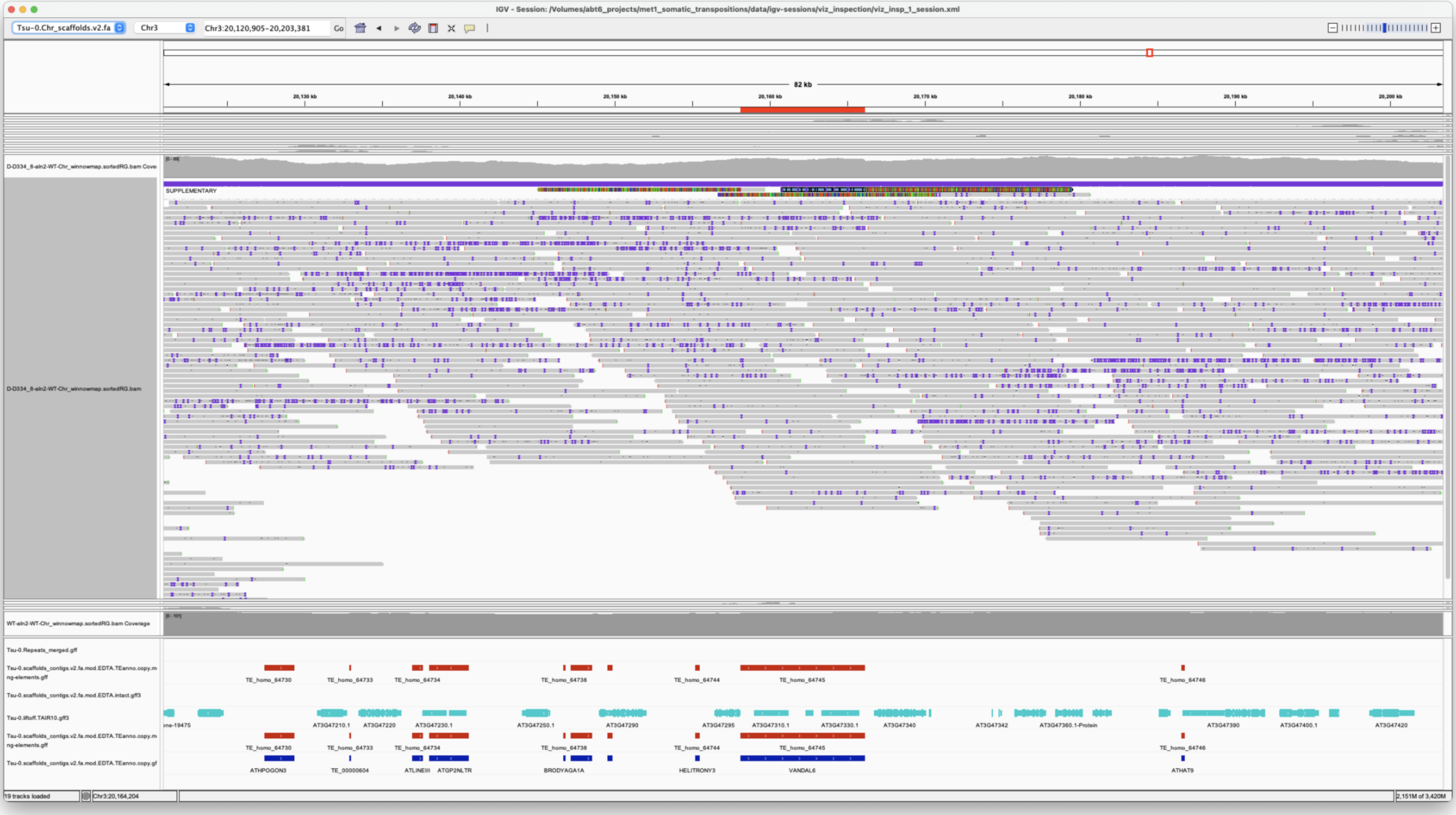

Partial

Confirmed

Chr1 3318828 3318828 + 1 Chr3;16344522;16352497;VANDAL6 m64079_240212_113350/53741303/ccs met1_08

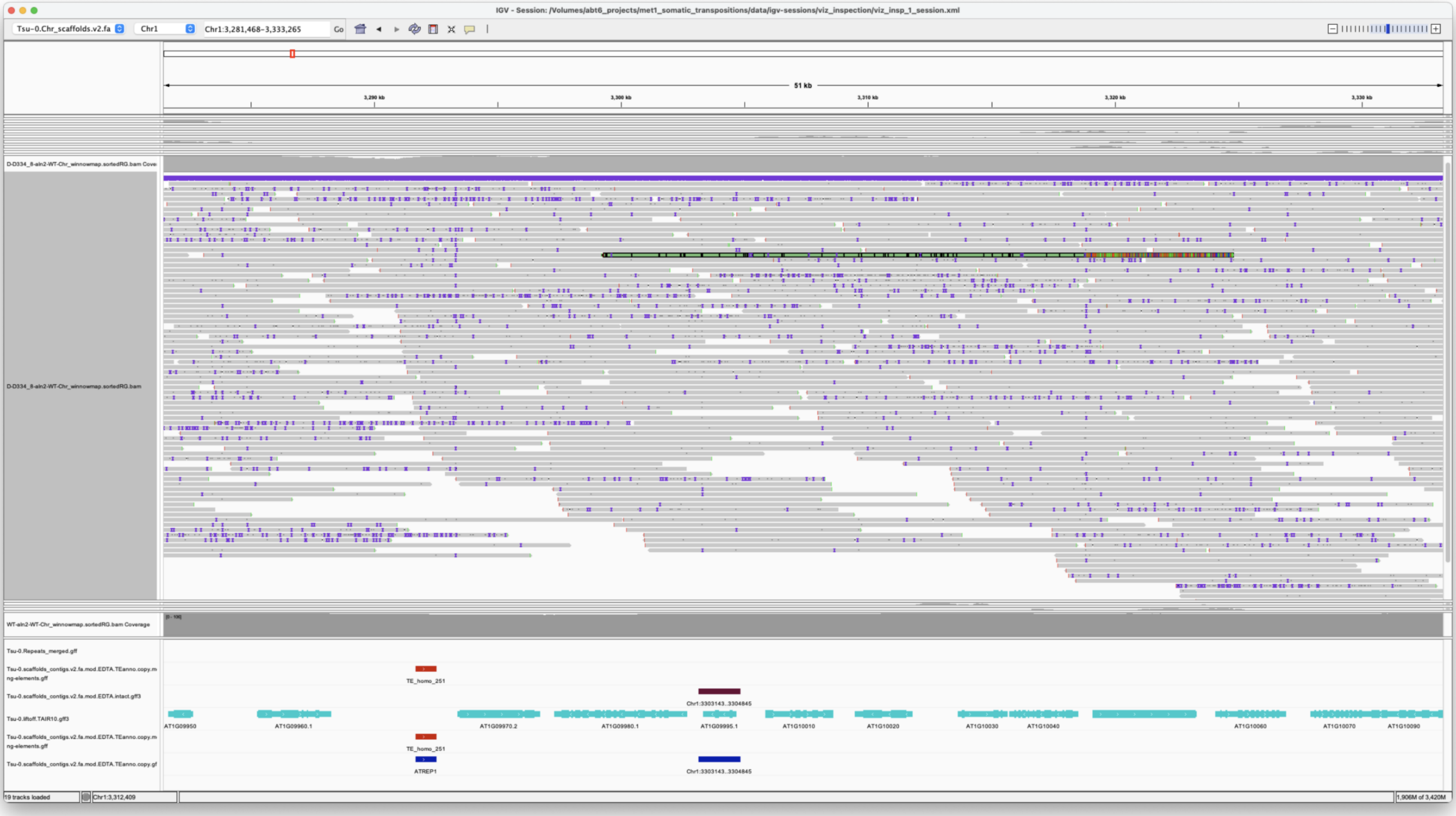

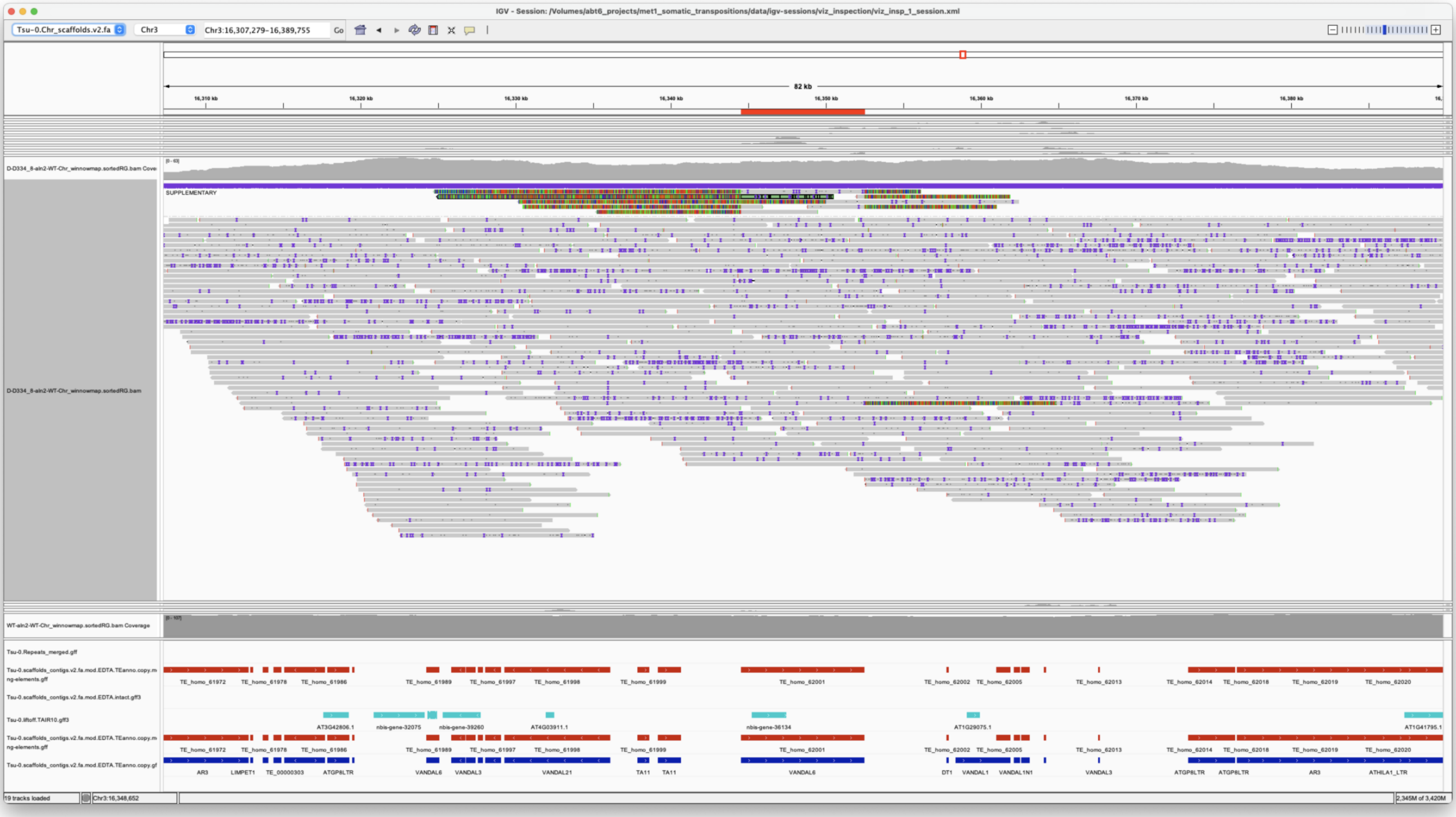

Partial

Confirmed

Chr1 4429093 4429093 + 1 Chr3;20158137;20166150;VANDAL6 m64079_221220_112036/133432715/ccs met1_08

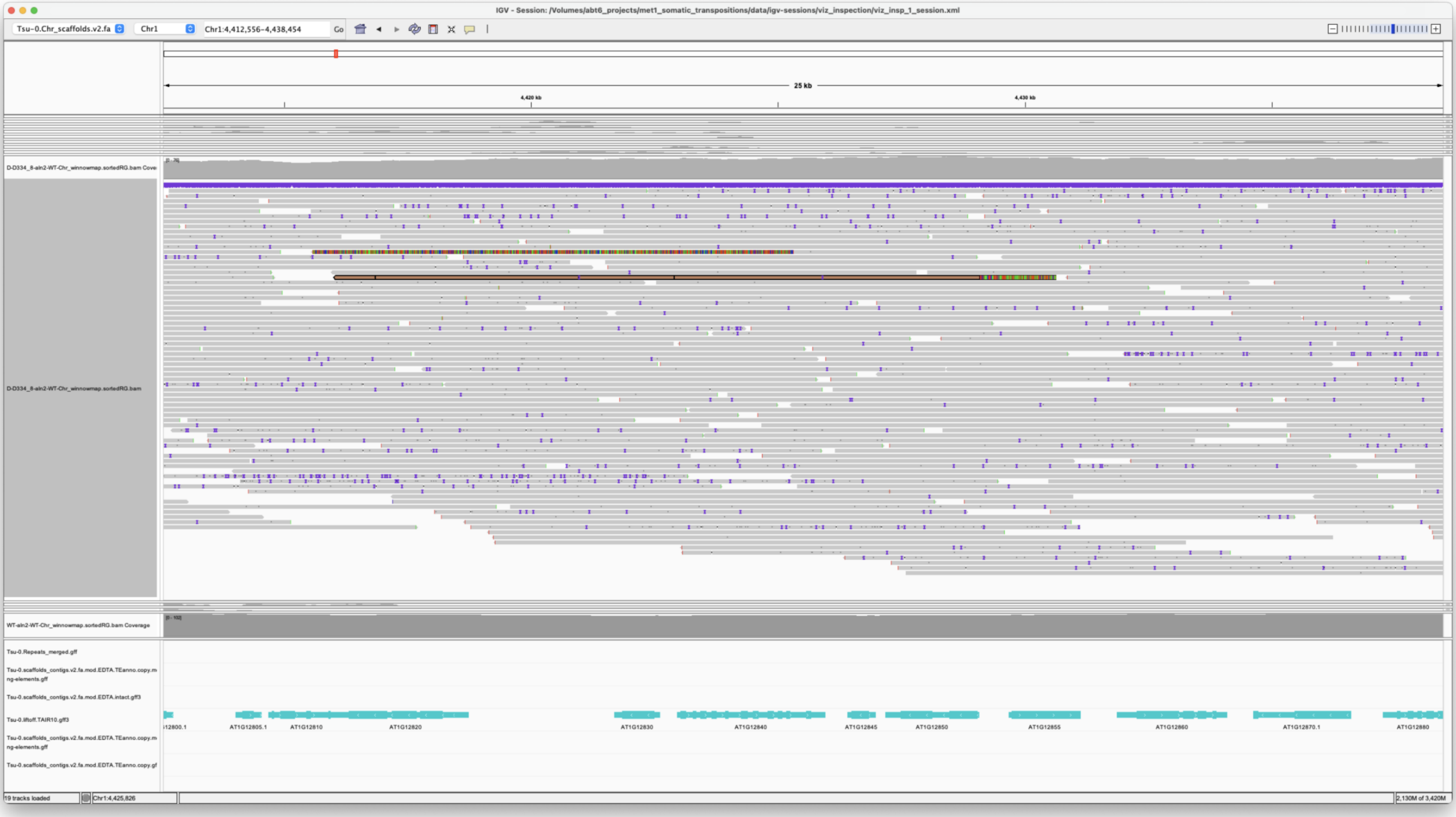

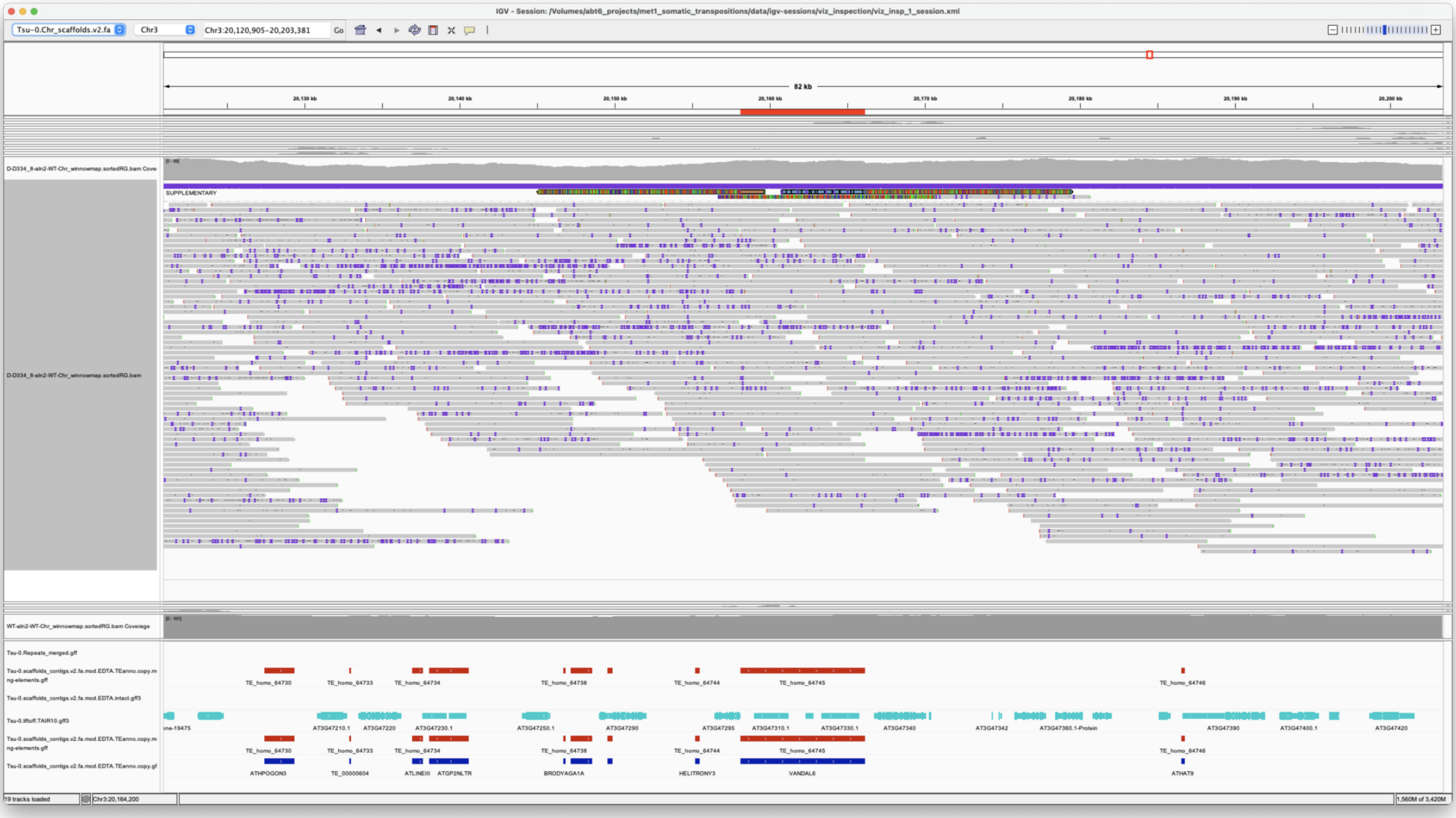

Partial

Confirmed

Chr1 9120942 9120942 + 1 Chr5;19152829;19160826;VANDAL21 m64079_240212_113350/159122047/ccs met1_08

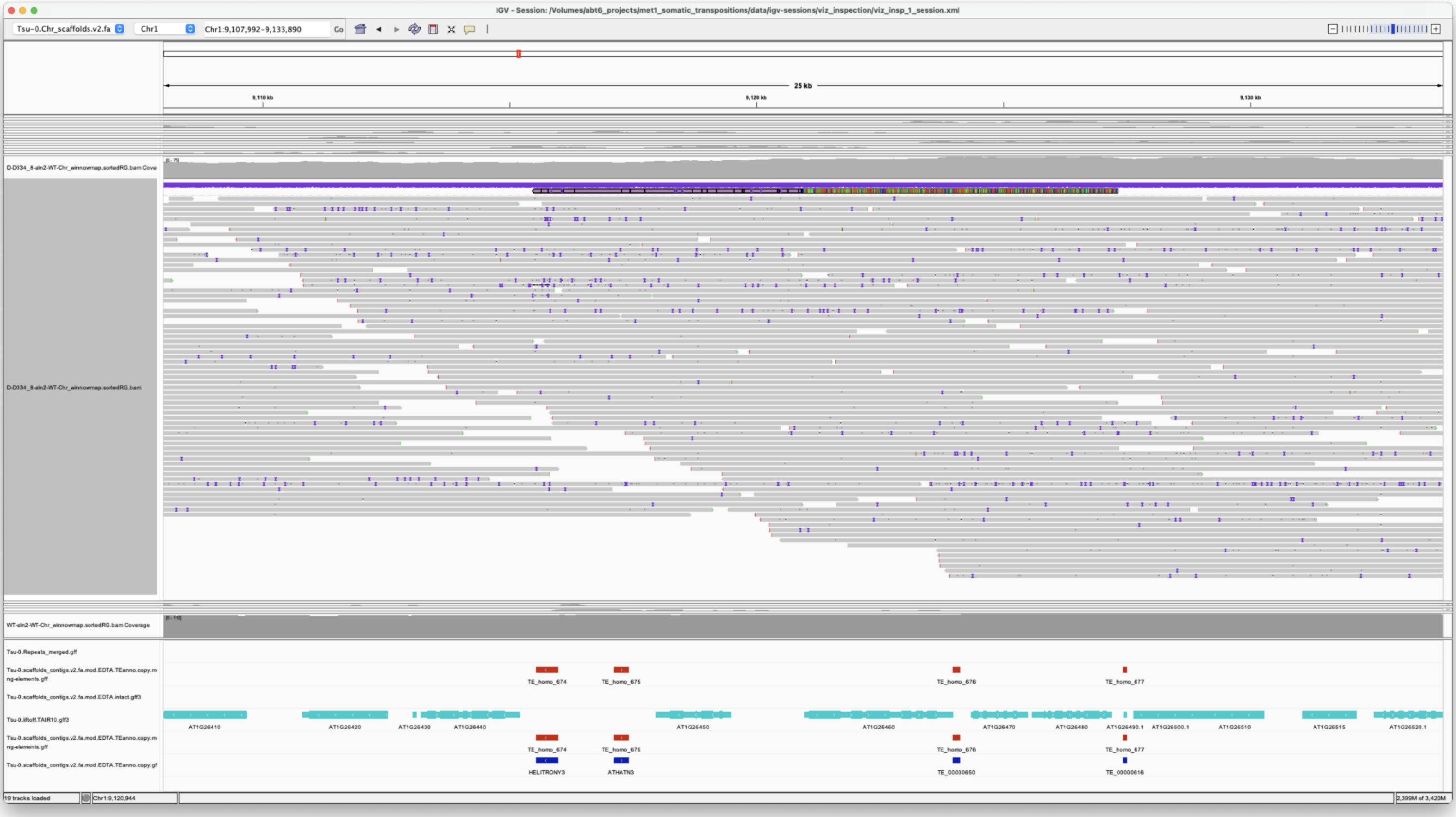

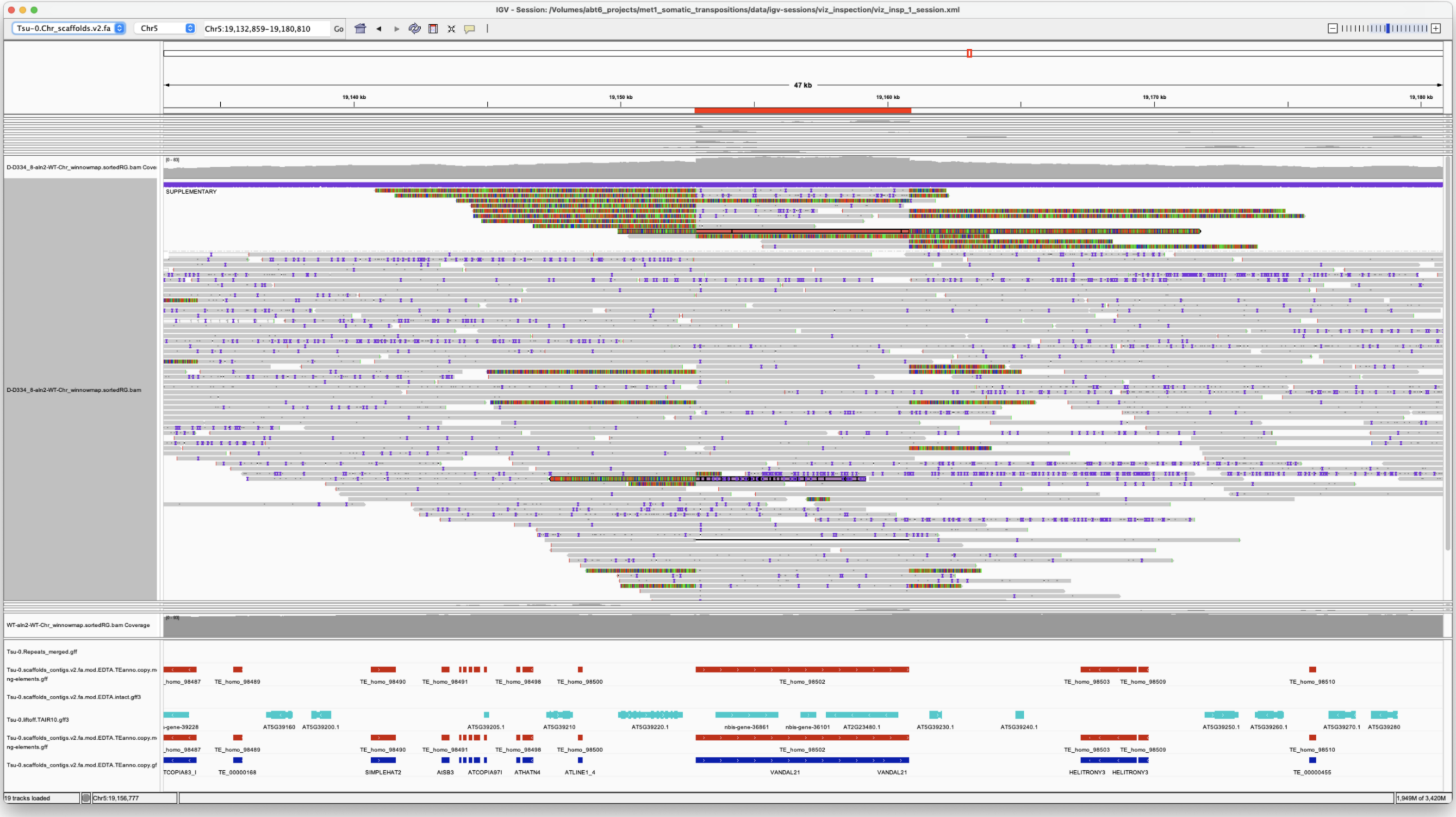

Partial

Confirmed

Chr1 10375779 10375779 + 1 Chr5;19152829;19160826;VANDAL21 m64079_240212_113350/170723688/ccs met1_08

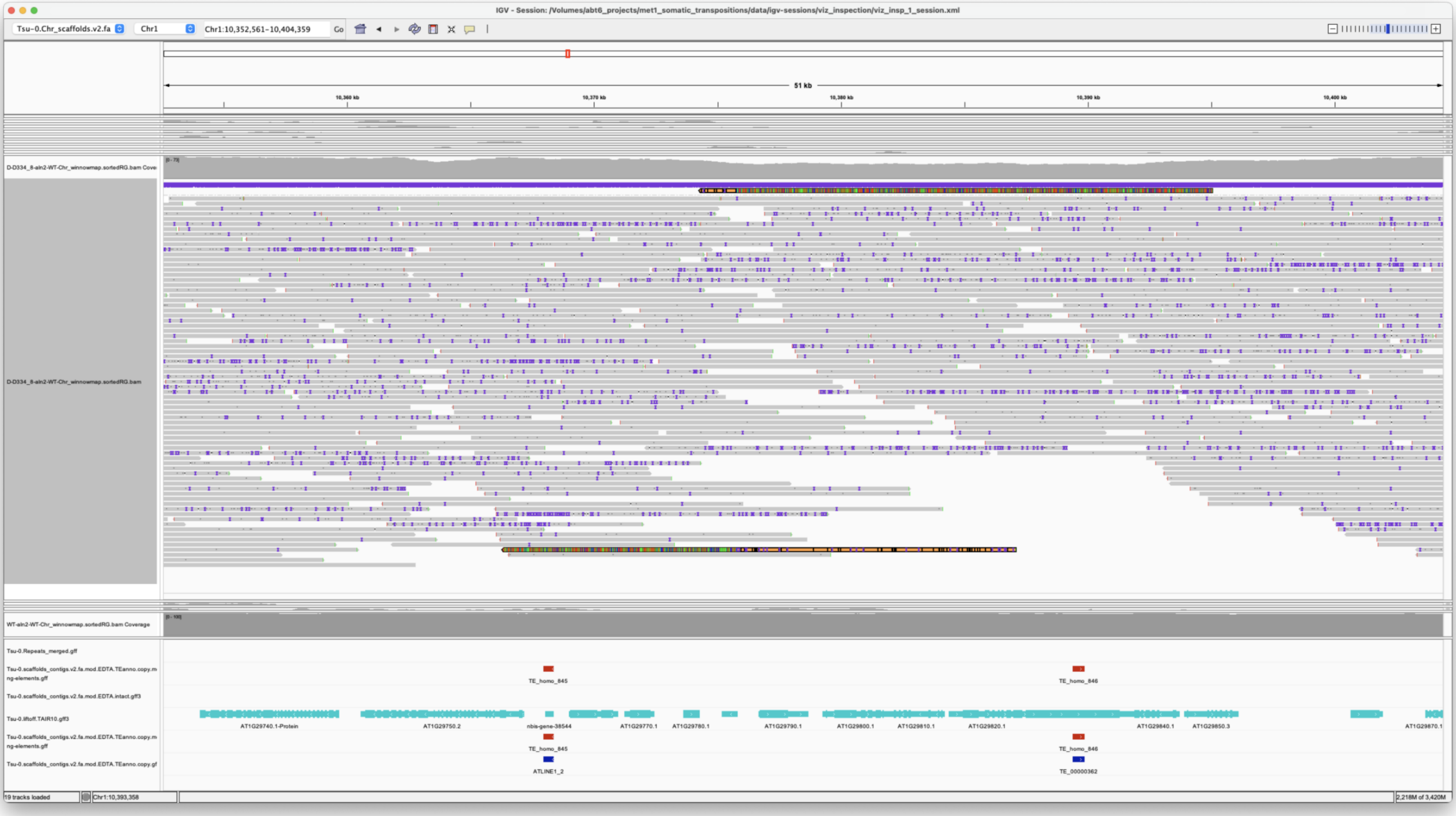

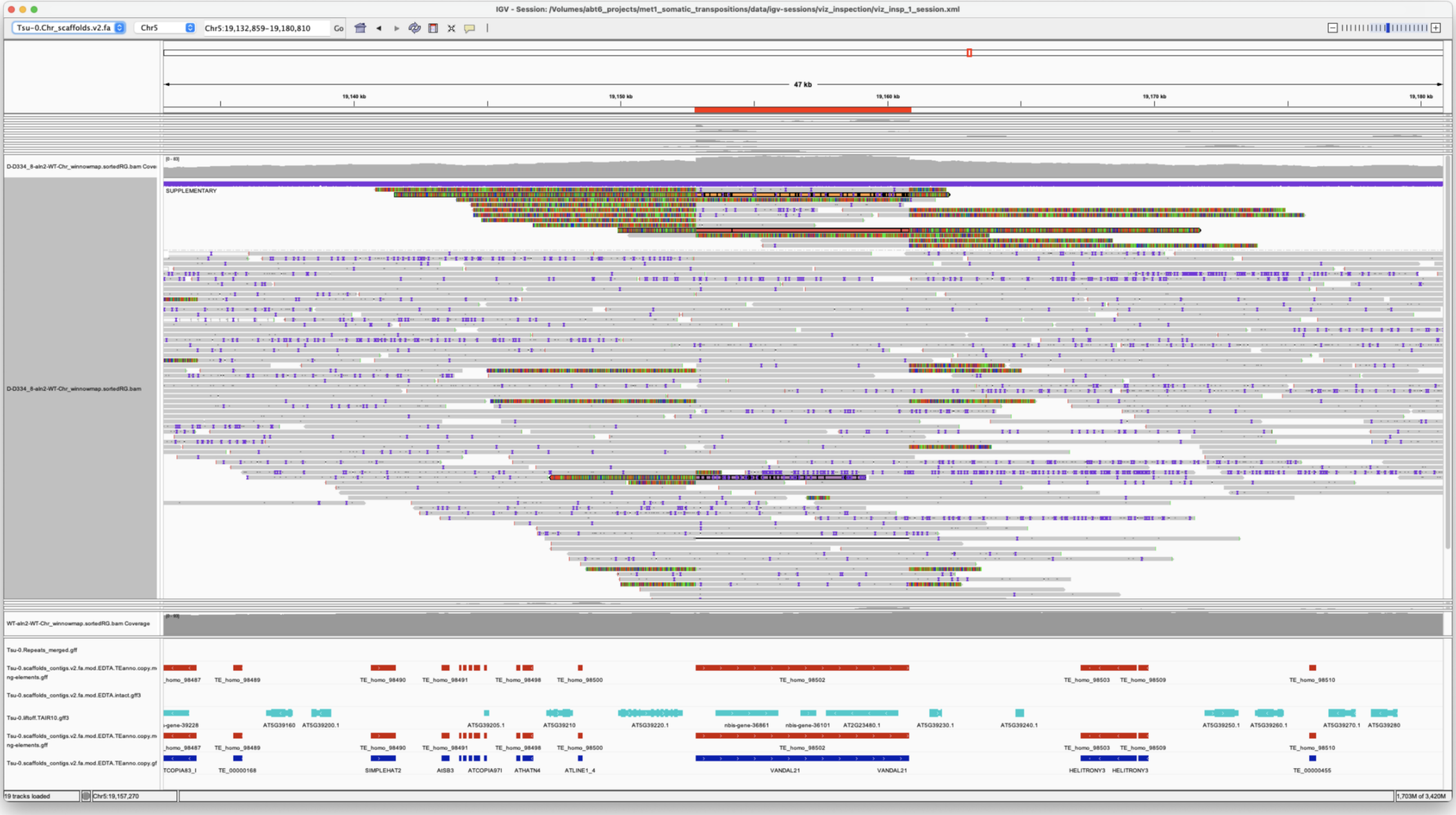

Central
TSD

Confirmed

Chr1 21143922 21143922 + 1 Chr5;19152829;19160826;VANDAL21 m64079_221220_112036/11667630/ccs met1_08

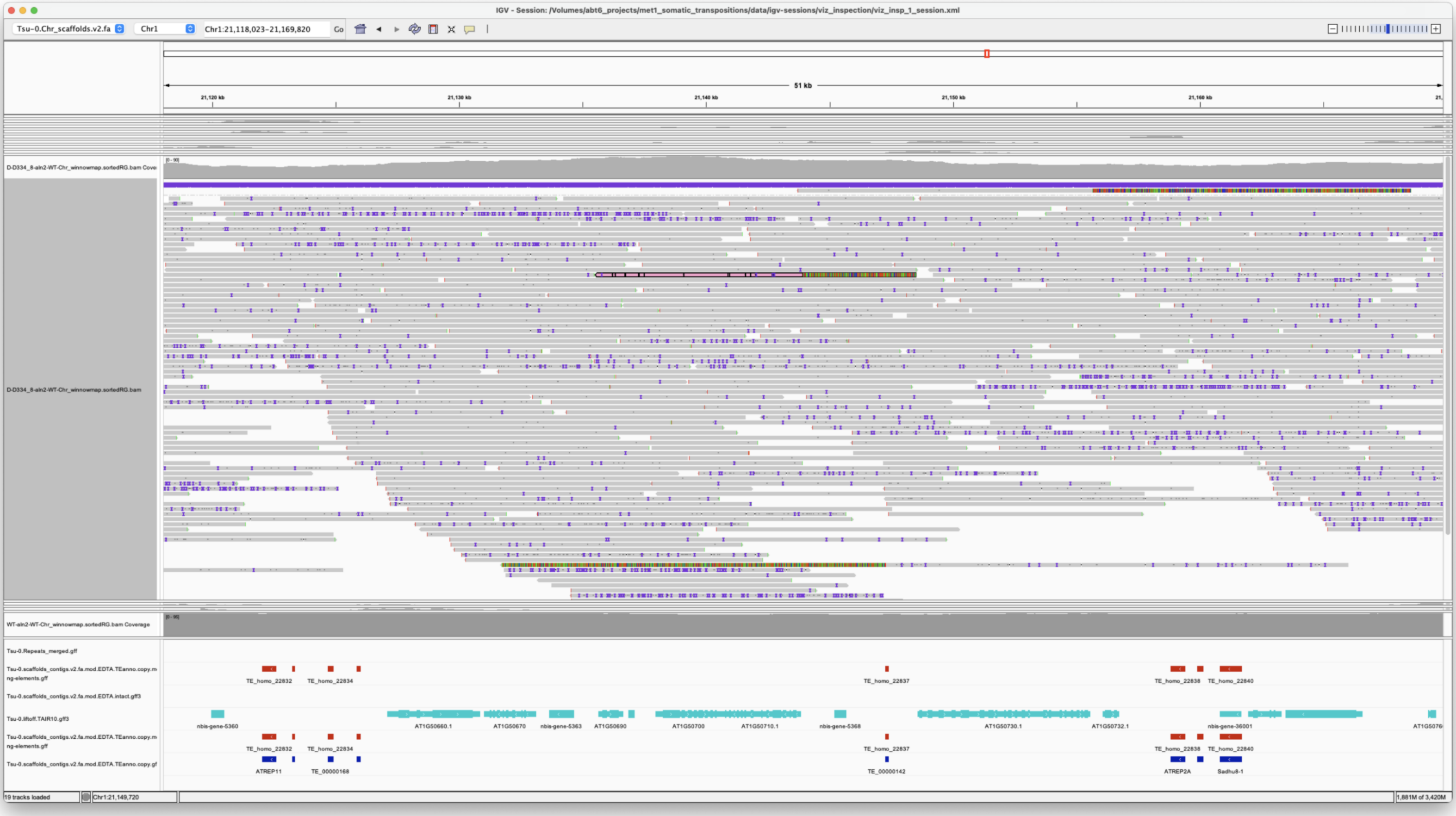

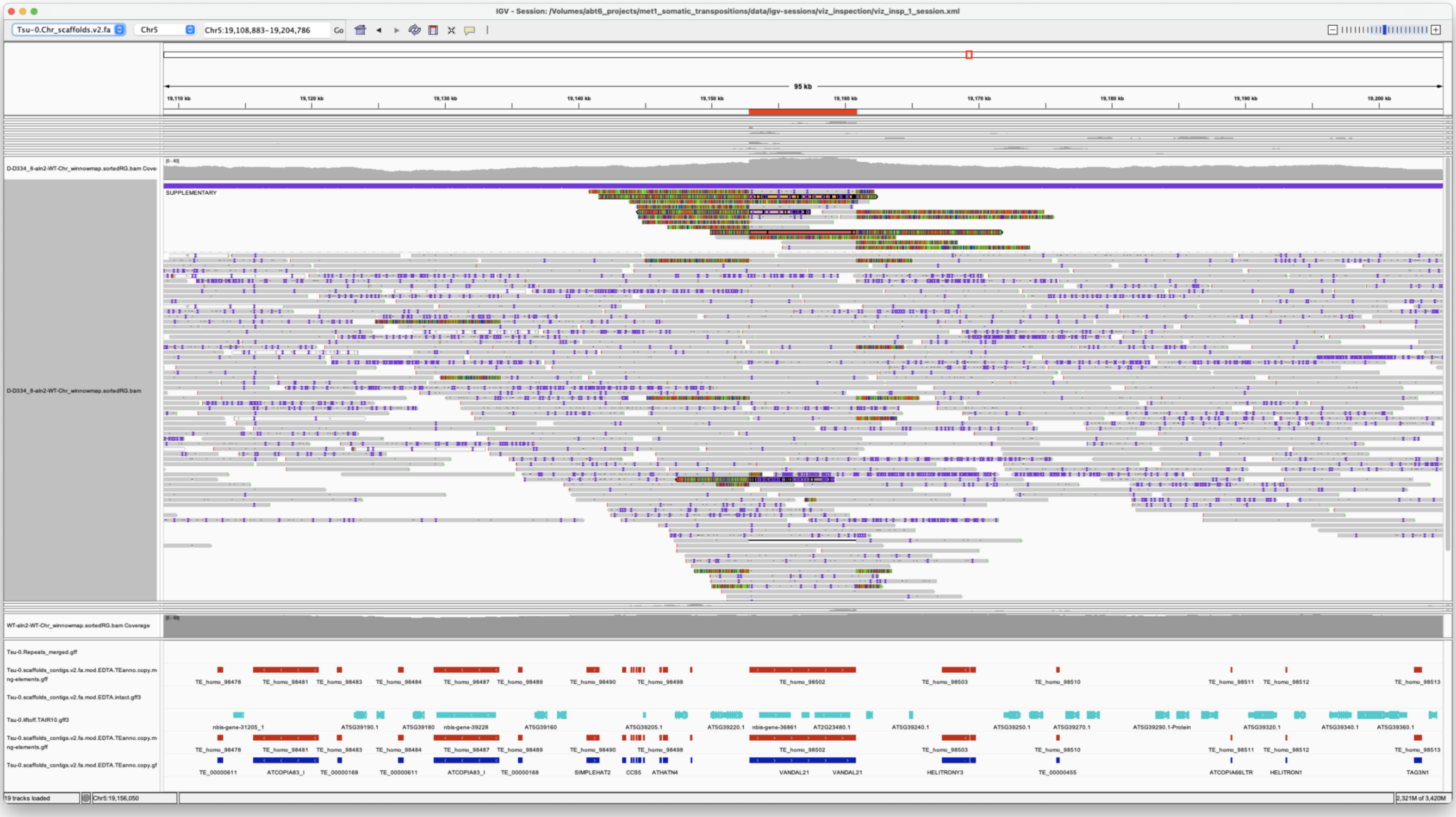

Partial

Confirmed

Chr1 25126037 25126037 + 1 Chr5;19152829;19160826;VANDAL21 m64079_221220_112036/17236260/ccs met1_08

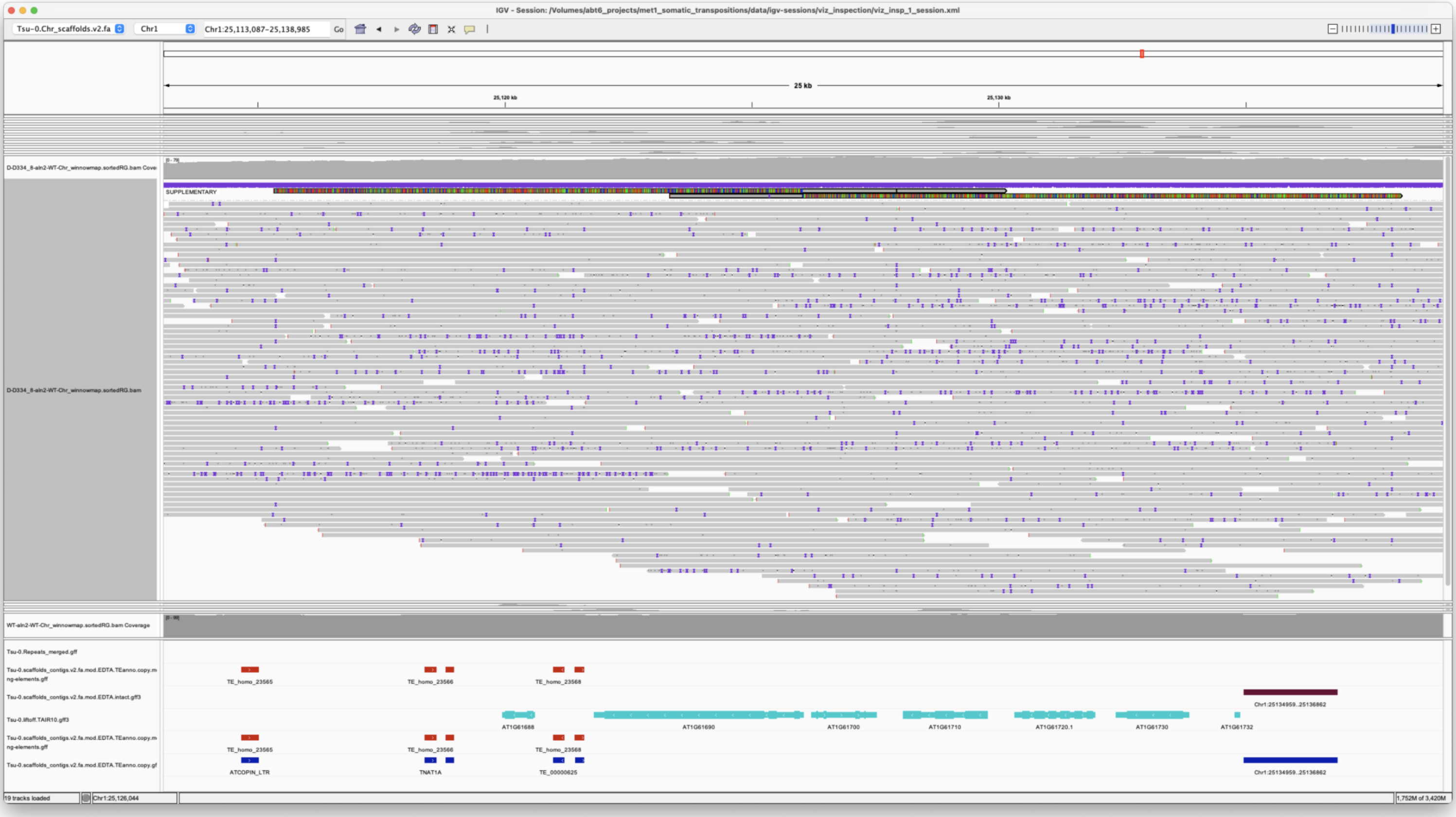

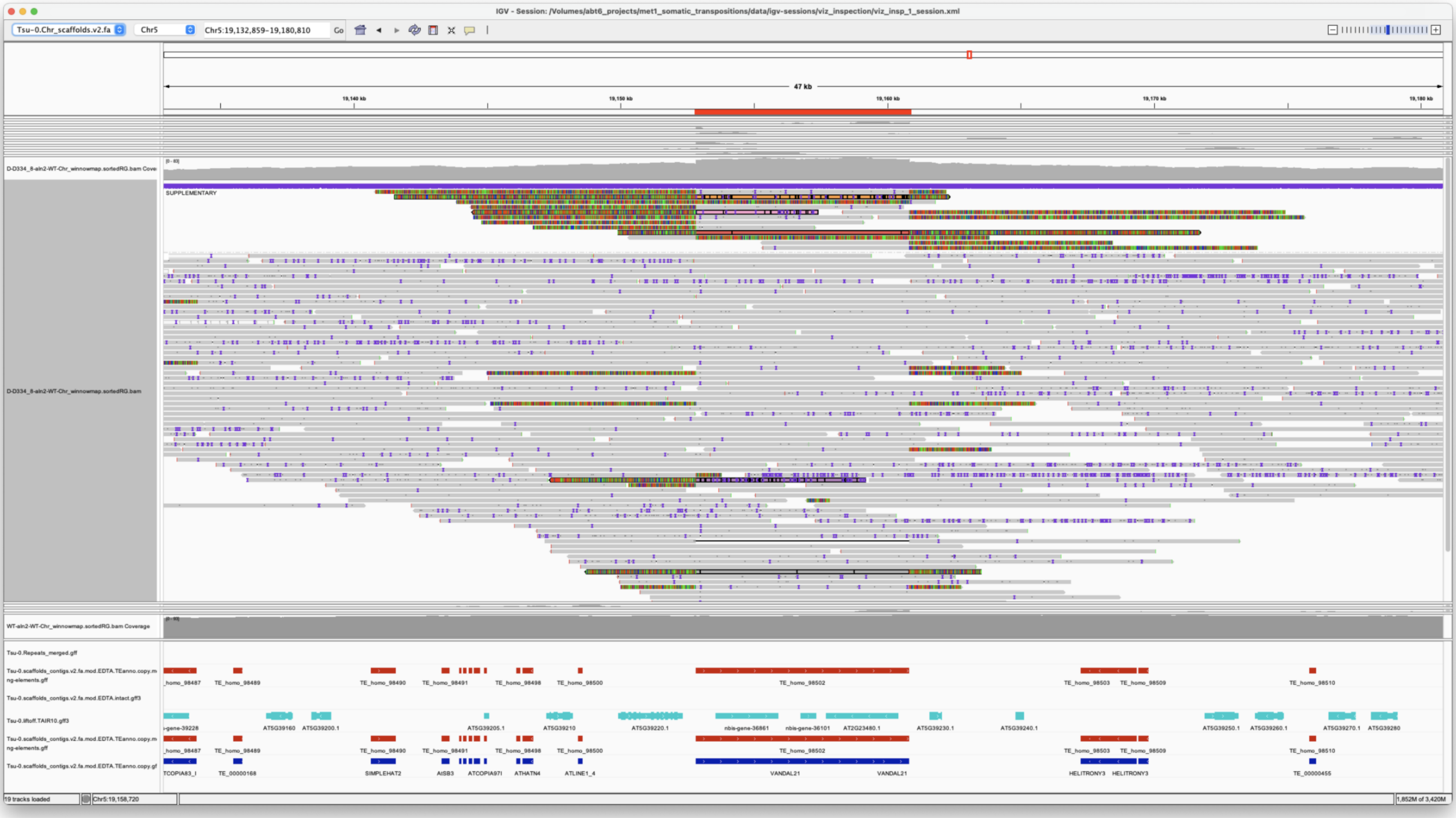

Central
TSD

Confirmed

Chr2 2848324 2848324 + 1 Chr5;19152829;19160826;VANDAL21 m64079_240212_113350/52888370/ccs met1_08

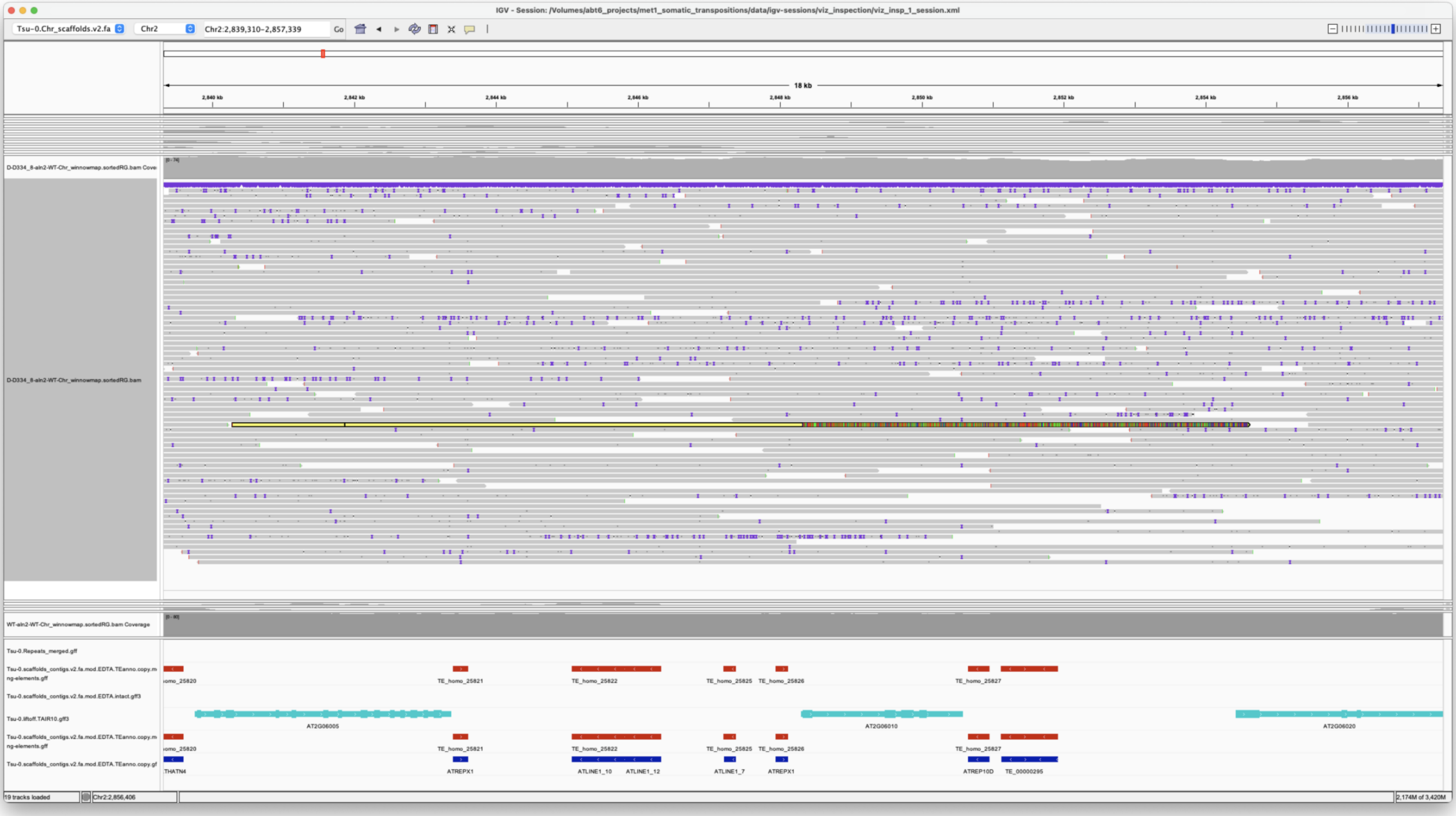

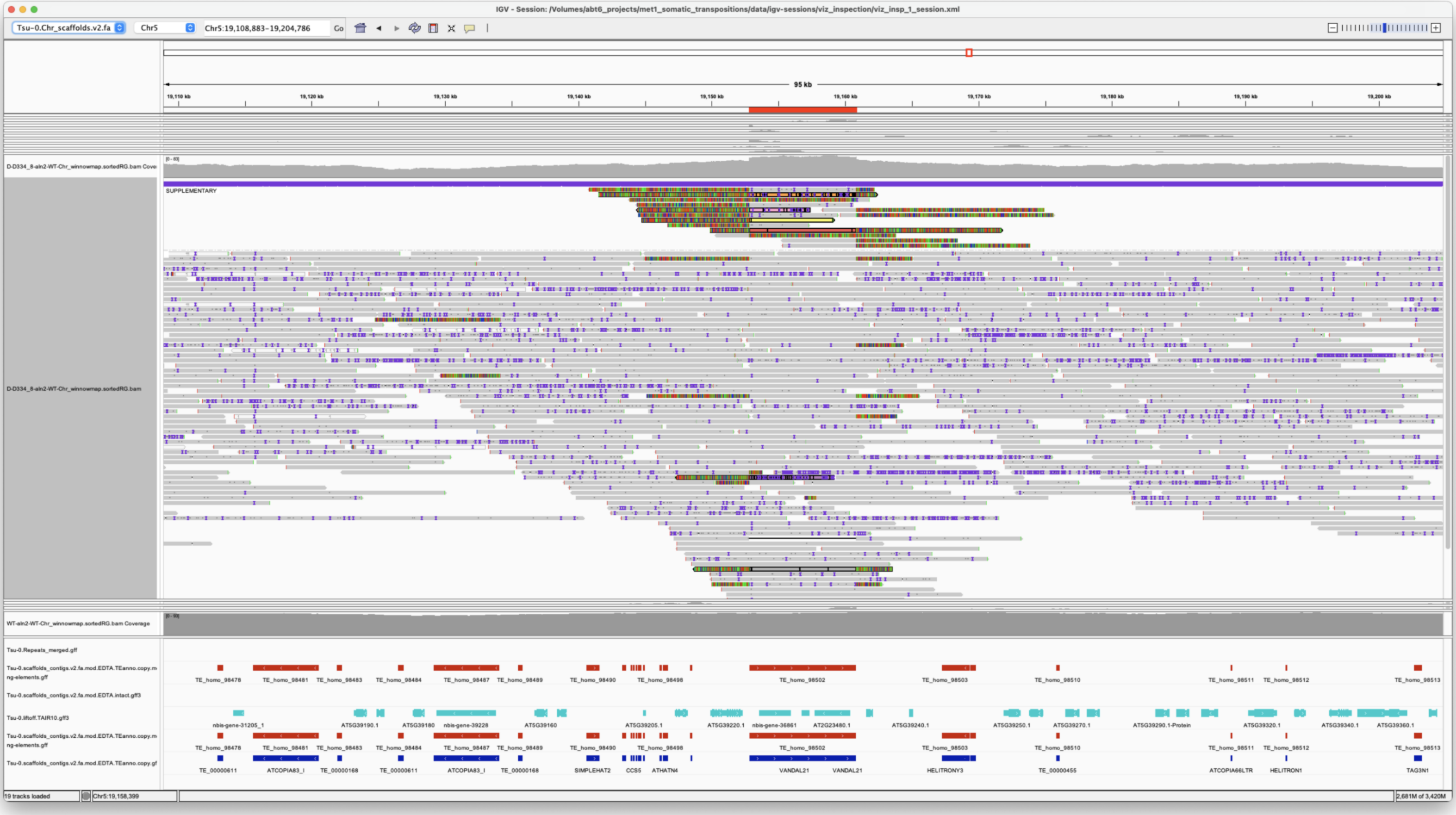

Partial

Confirmed

Chr2 7979469 7979469 - 1 Chr5;19152829;19160826;VANDAL21 m64079_221220_112036/105777959/ccs met1_08

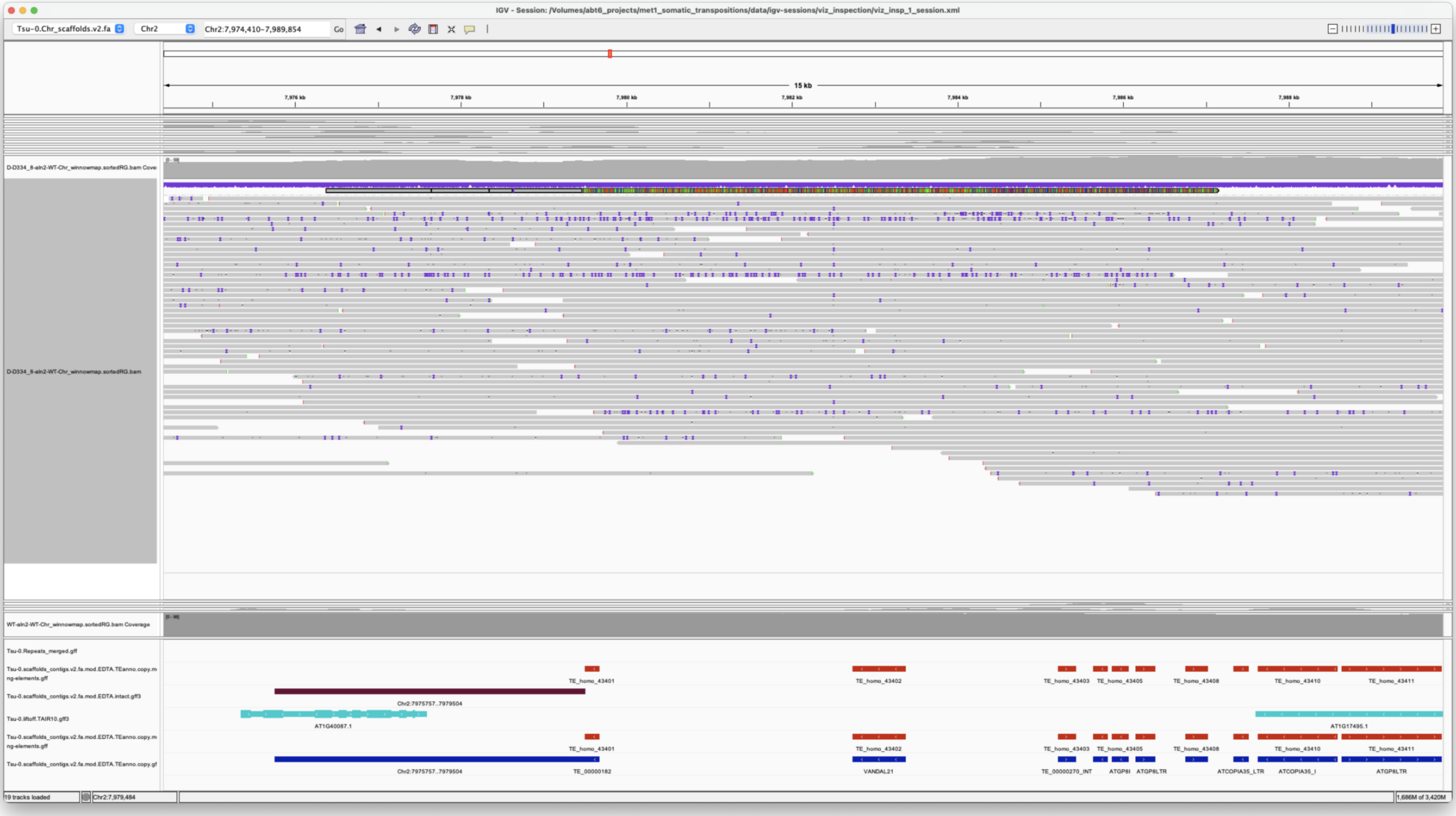

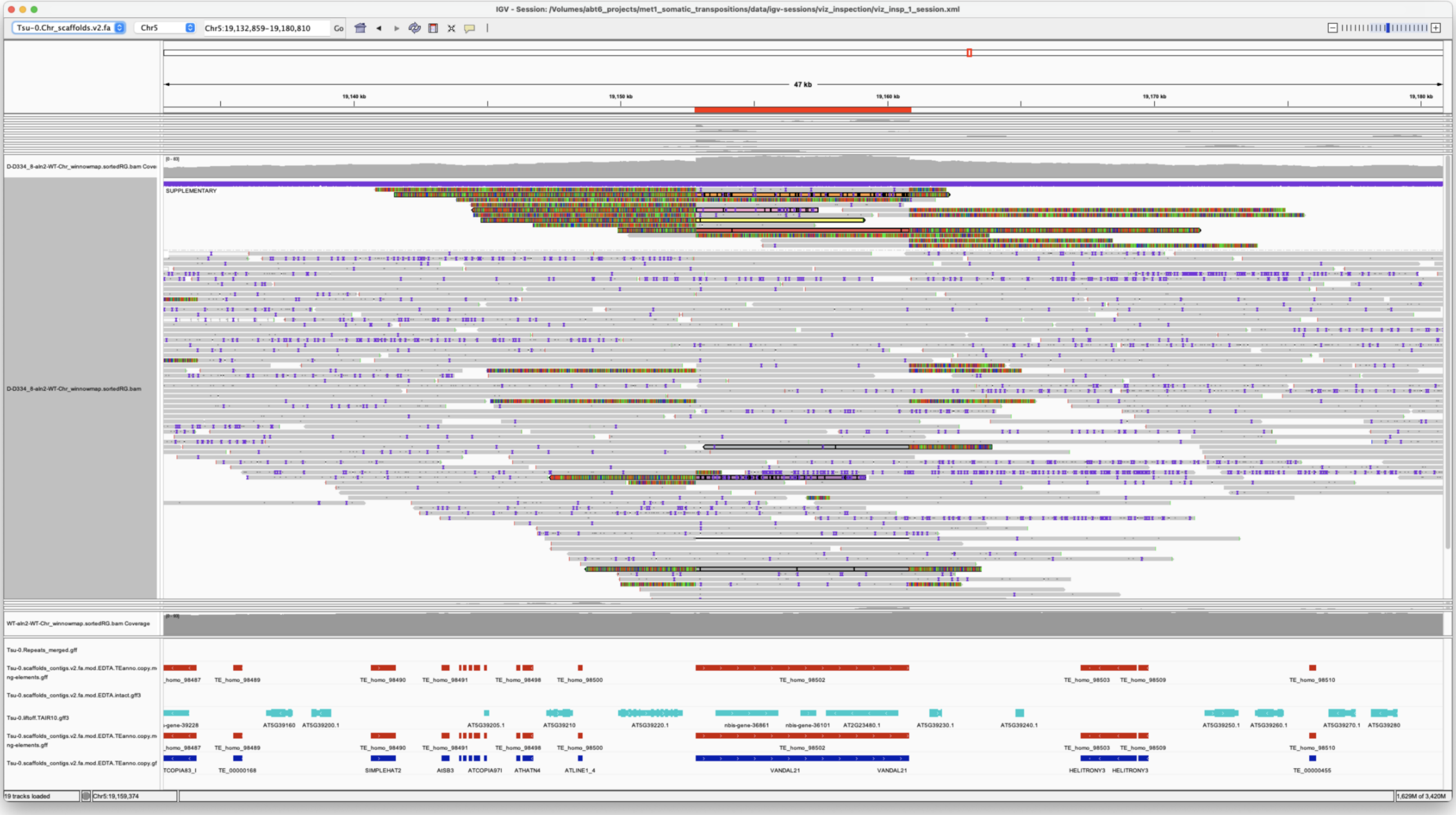

Partial

Confirmed

Chr2 8079759 8079759 + 1 Chr2;8337982;8342127;ATENSPM2 m64079_221220_112036/179110753/ccs met1_08

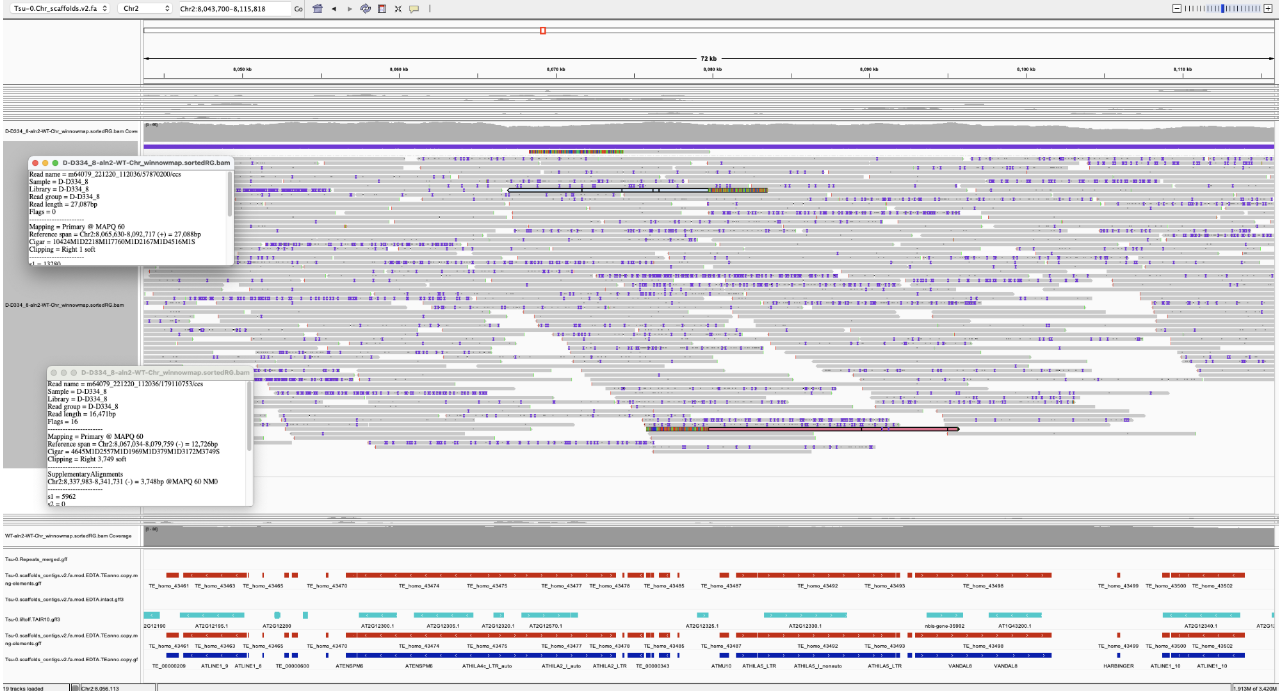

Another clipped read without supplementary aln that is a ATENSPM2

> m64079_221220_112036/60426770/ccs
> EXCLUDED BECAUSE OF LOW QUALITY

Map of Hits

SVG viewer is required to view graphical representation of the map as Scalable Vector Graphics (SVG plot).

/tmp/censor.114754.tmp/data.ori (SVG Plot; Alignments; Masked)

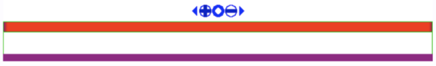

Name	From	To	Name	From	To	Class	Dir	Sim	Pos/Mm:Ts	Score
/tmp/censor.114754.tmp/data.ori	4	3749	ATENSPM2	1	3863	DNA/EnSpm/CACTA	c	0.8236	1.8266	18266

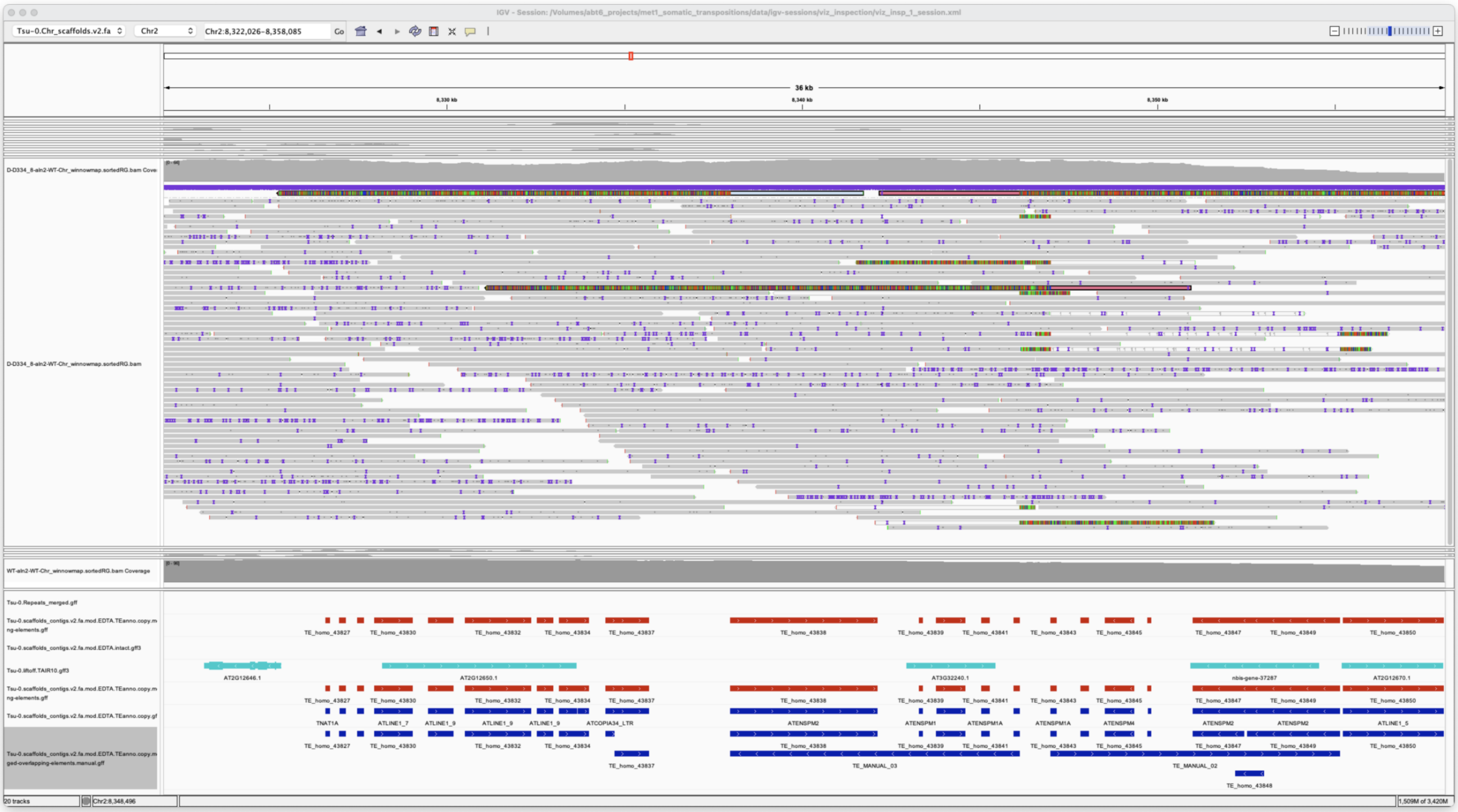

Partial

Confirmed

> Chr2 8761120 8761120 + 1 Chr5;21419693;21425022;ATCOPIA93_Evade m64079_221220_112036/73860238/ccs met1_08

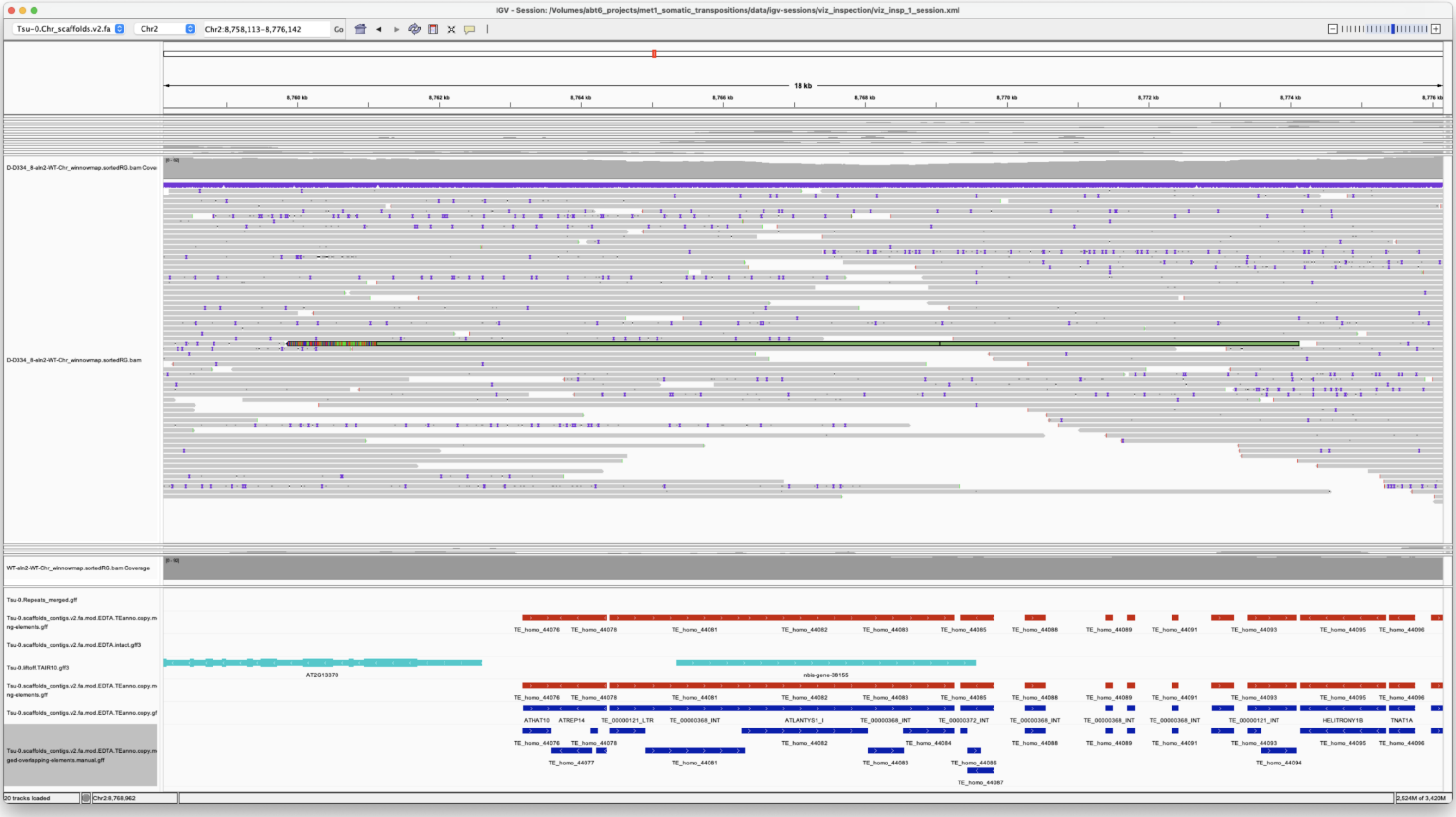

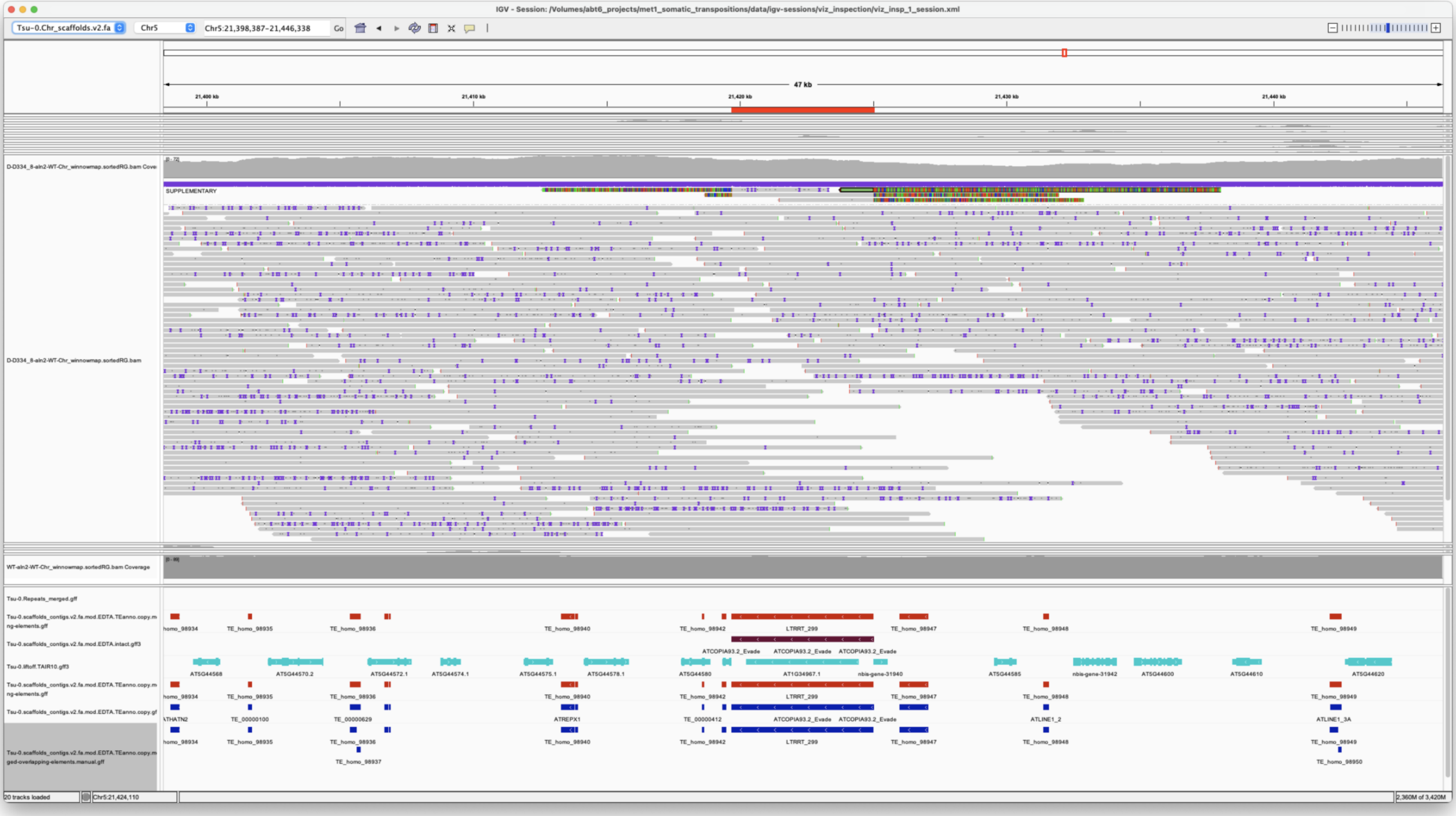

Partial

Confirmed

Chr2 12376563 12376563 - 1 Chr5;19152829;19160826;VANDAL21 m64079_221220_112036/141035914/ccs met1_08

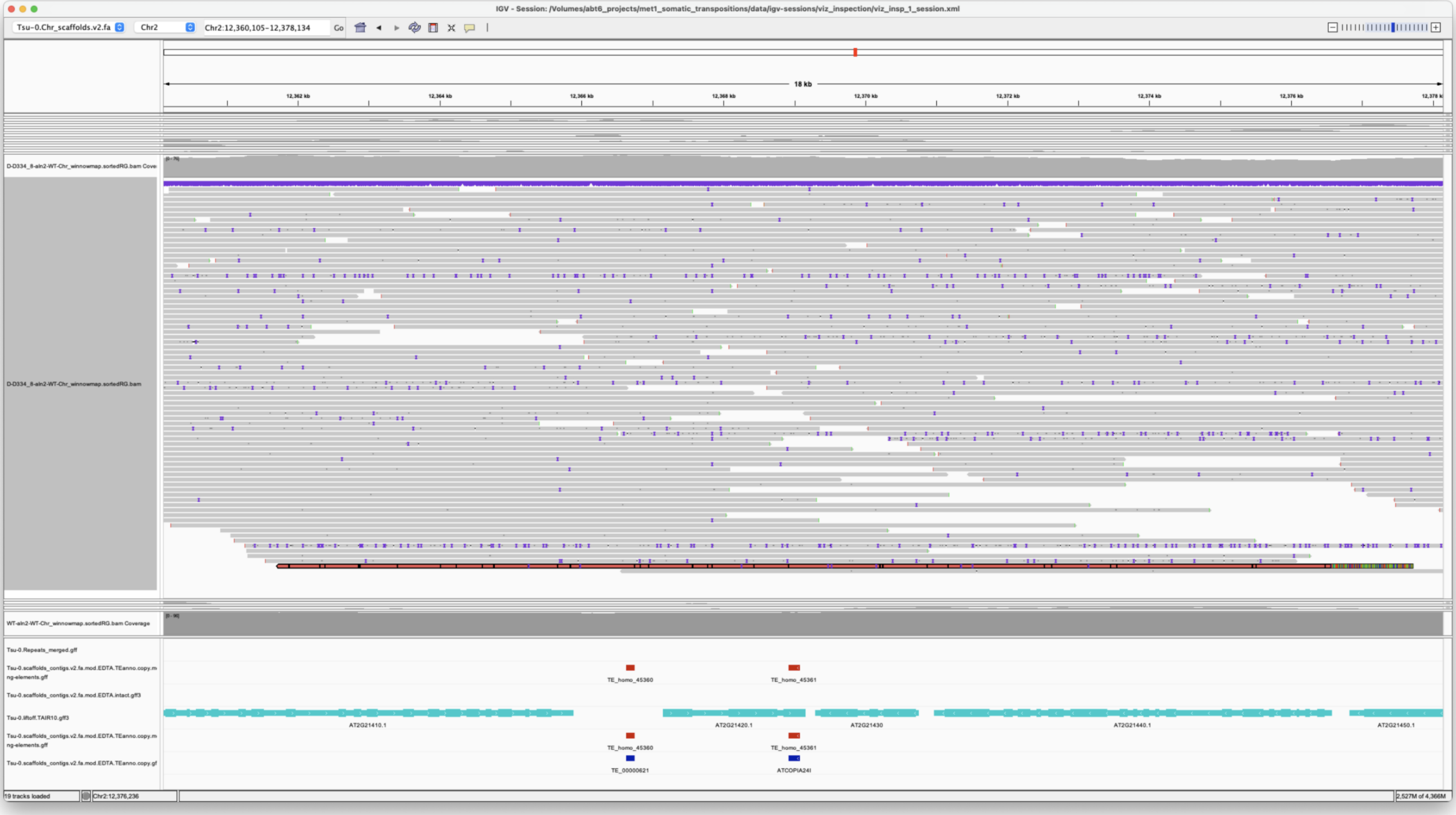

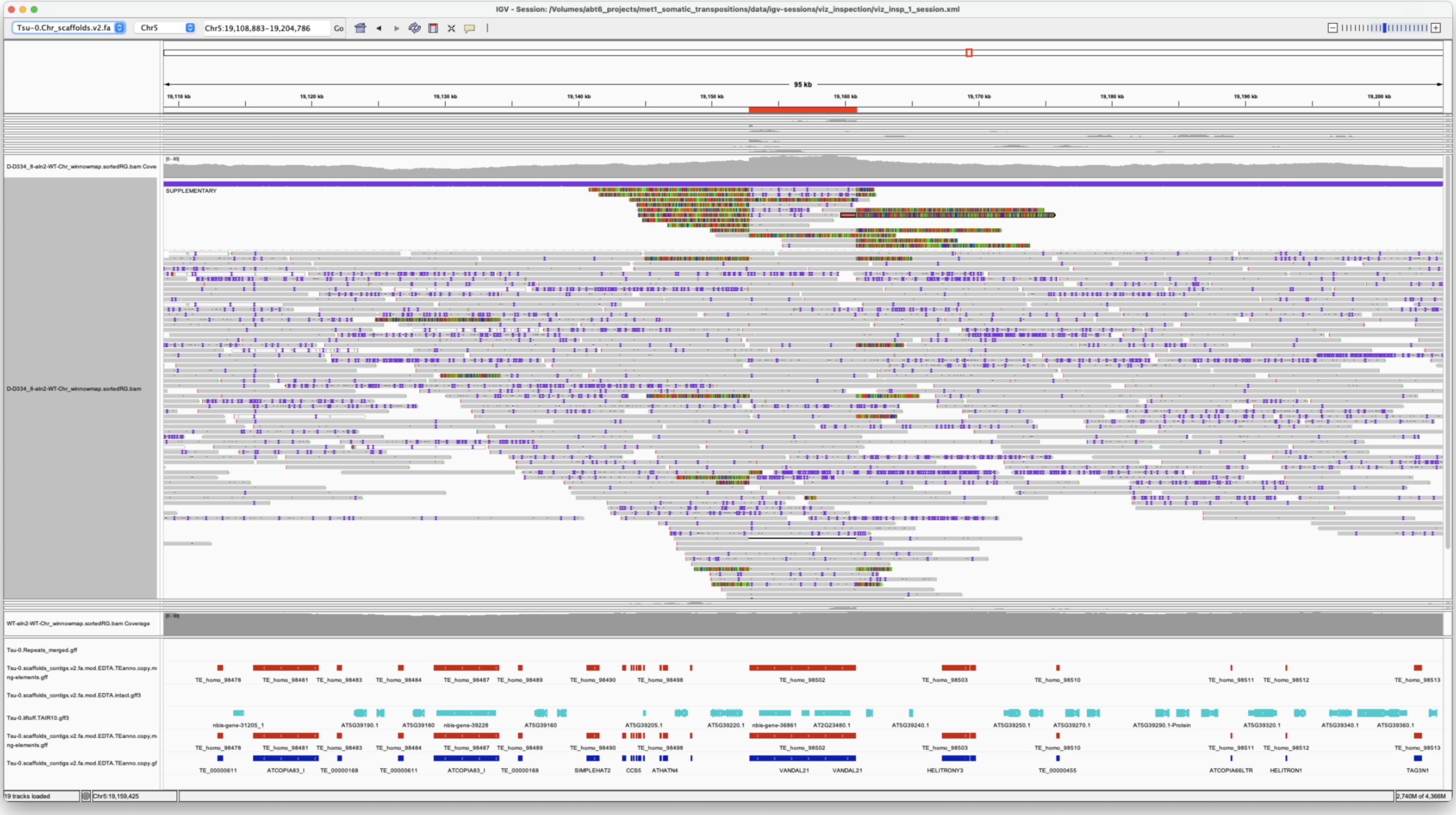

Partial

Confirmed

Chr2 22853432 22853432 - 1 Chr3;16344522;16352497;VANDAL6 m64079_221220_112036/159779033/ccs met1_08

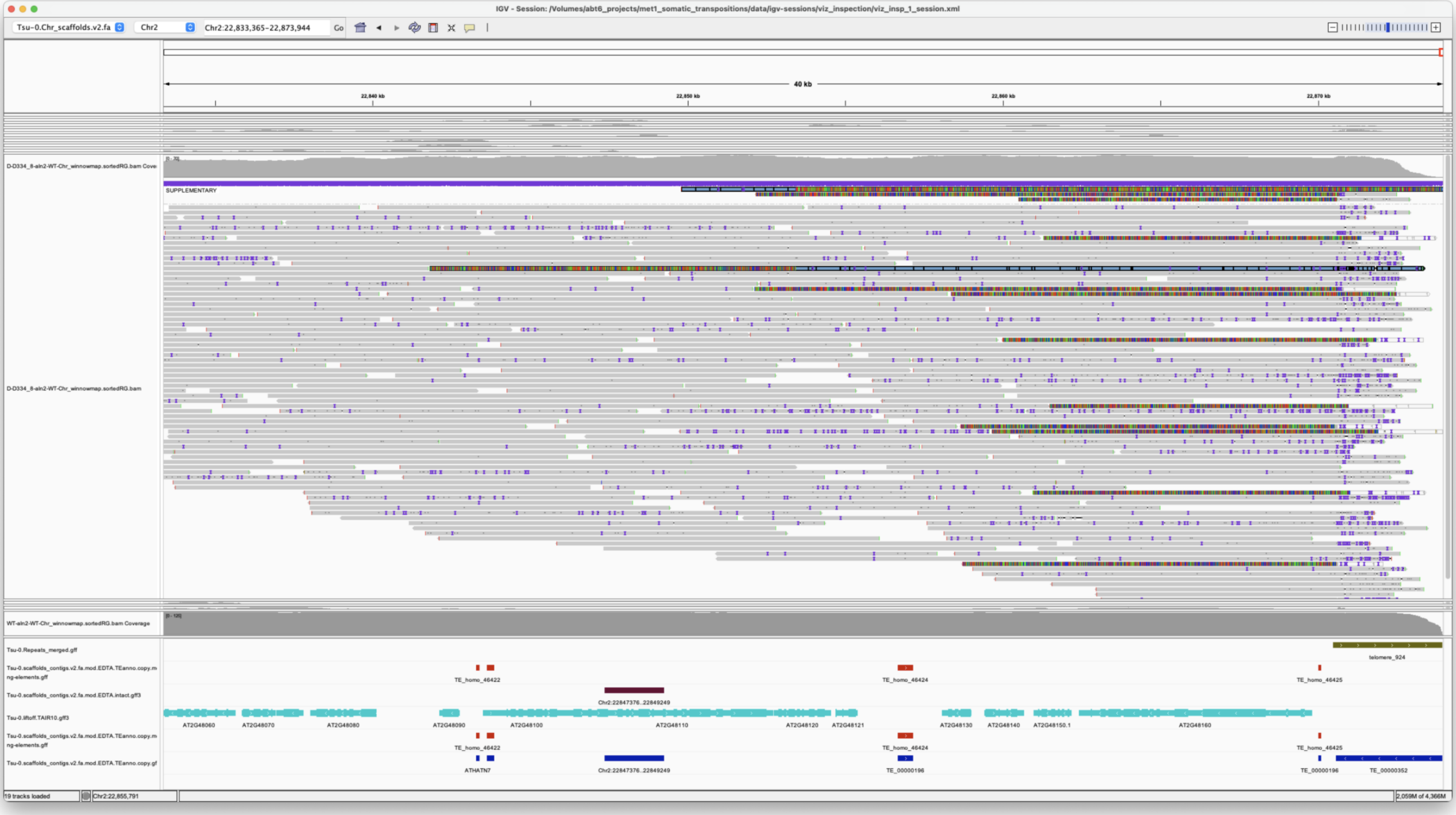

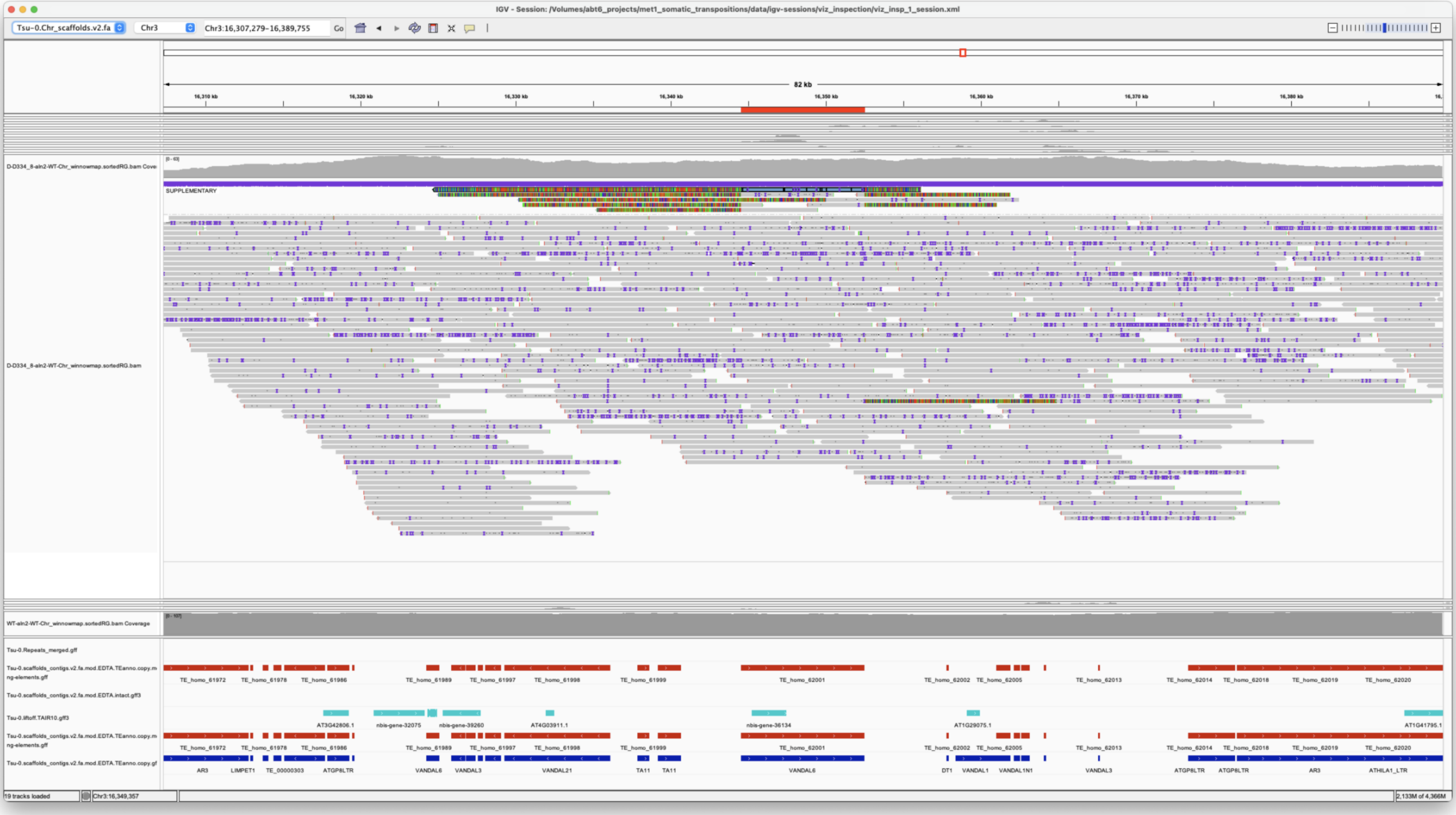

TELOMERIC

Confirmed

Chr3 2672825 2672825 + 1 Chr1;11941106;11946436;ATCOPIA93_Evade m64079_240212_113350/58524204/ccs met1_08

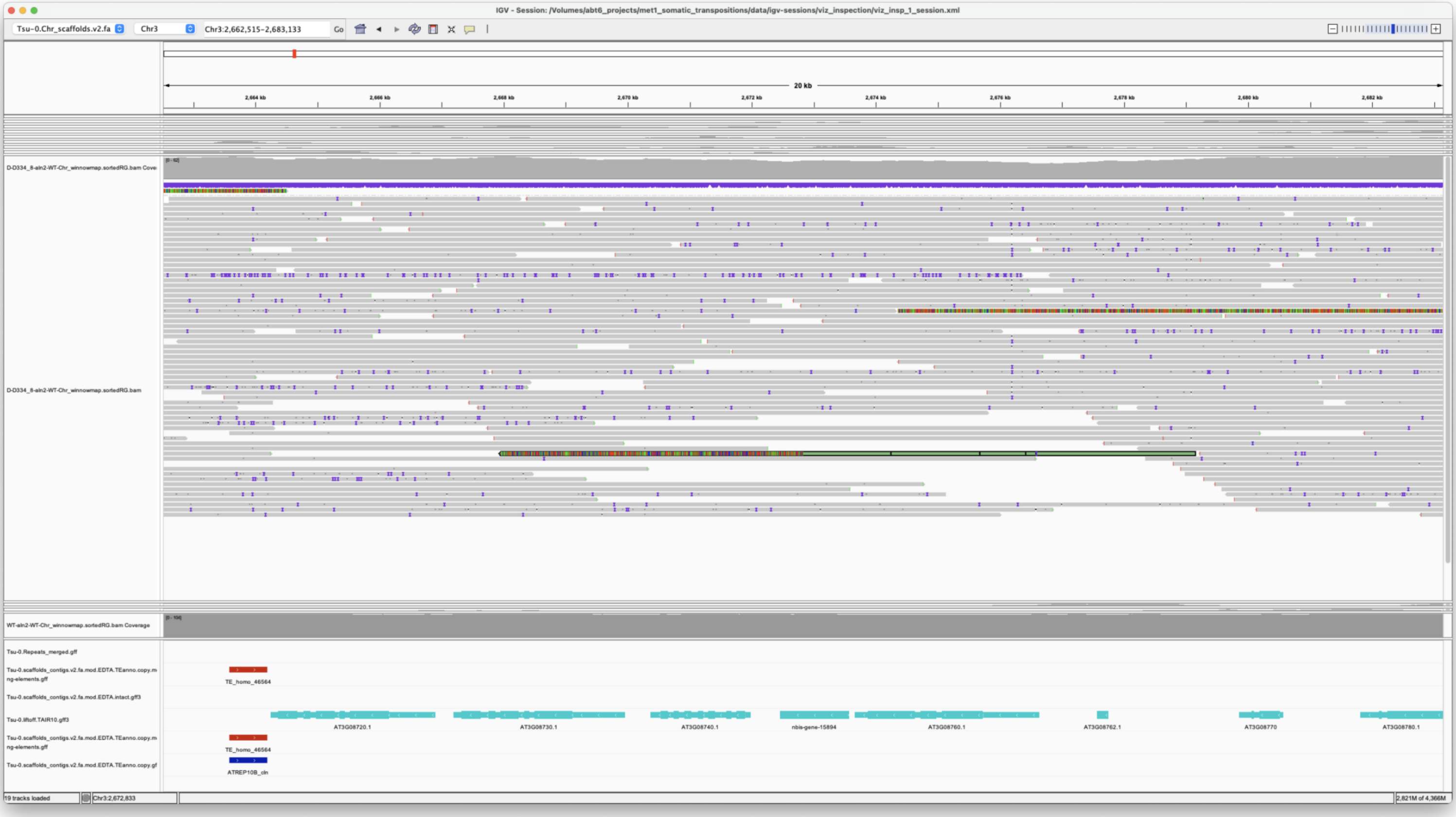

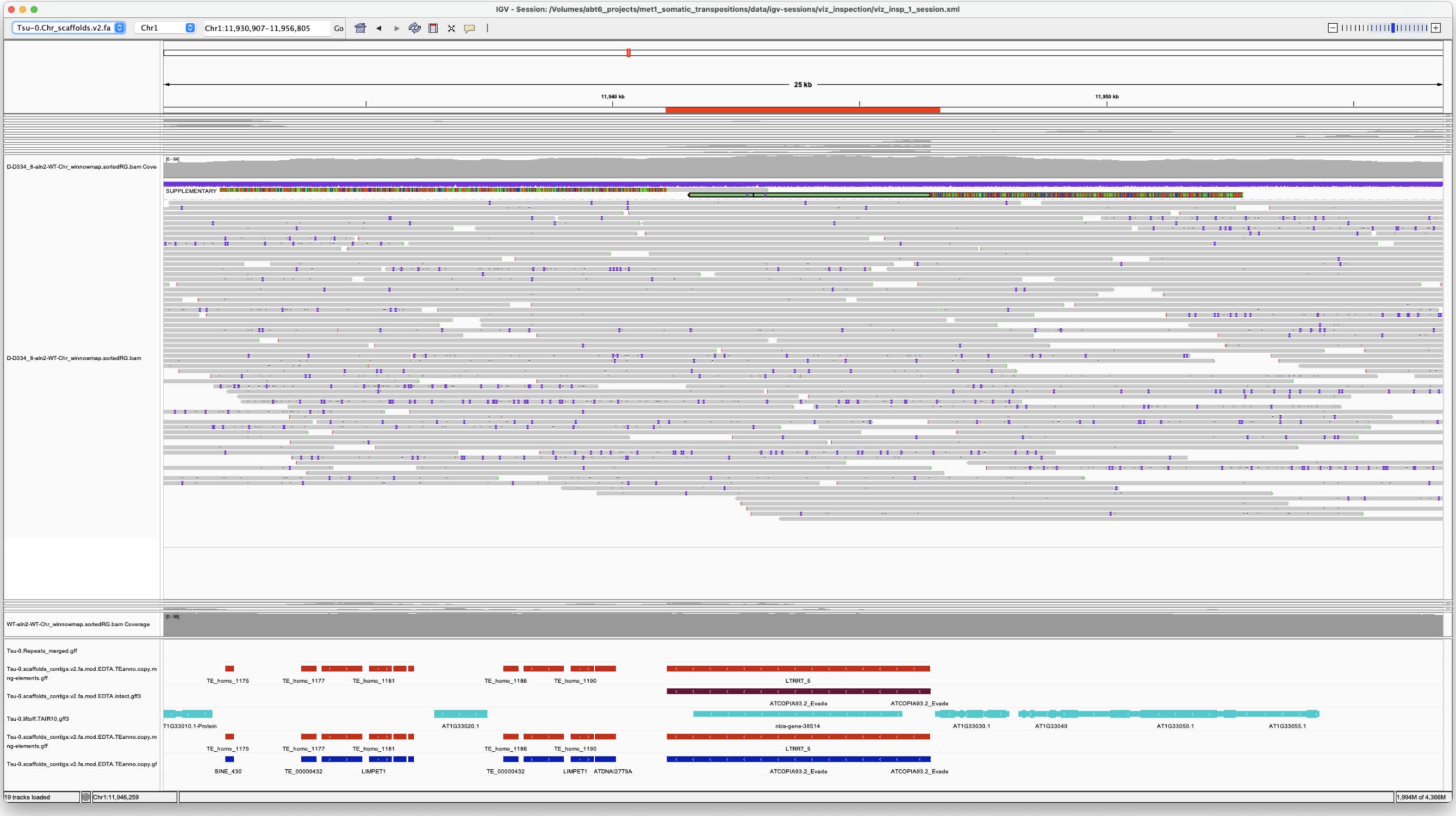

Partial

Confirmed

Chr3 8845271 8845271 - 1 Chr3;16344522;16352497;VANDAL6 m64079_240212_113350/113248392/ccs met1_08

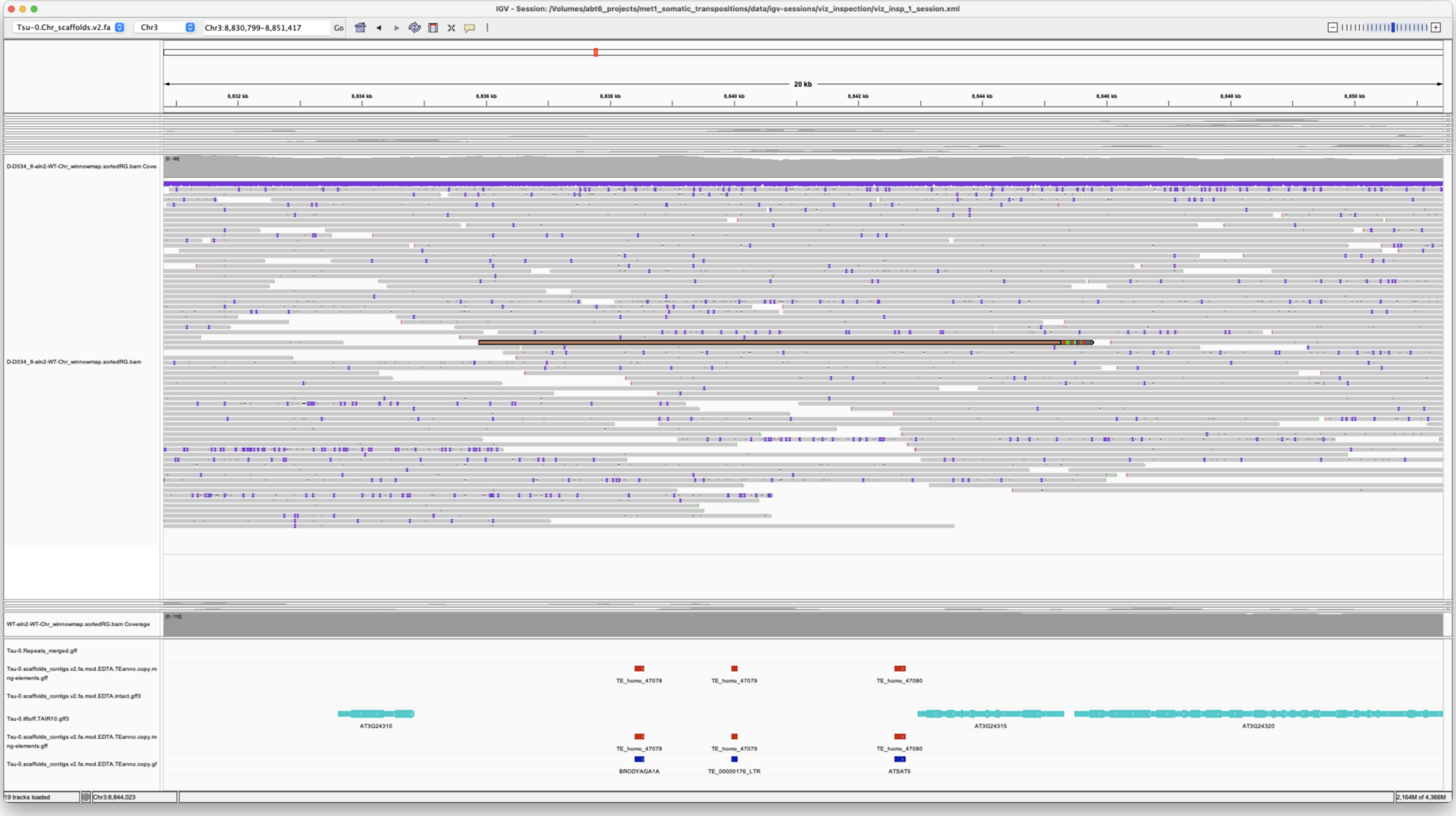

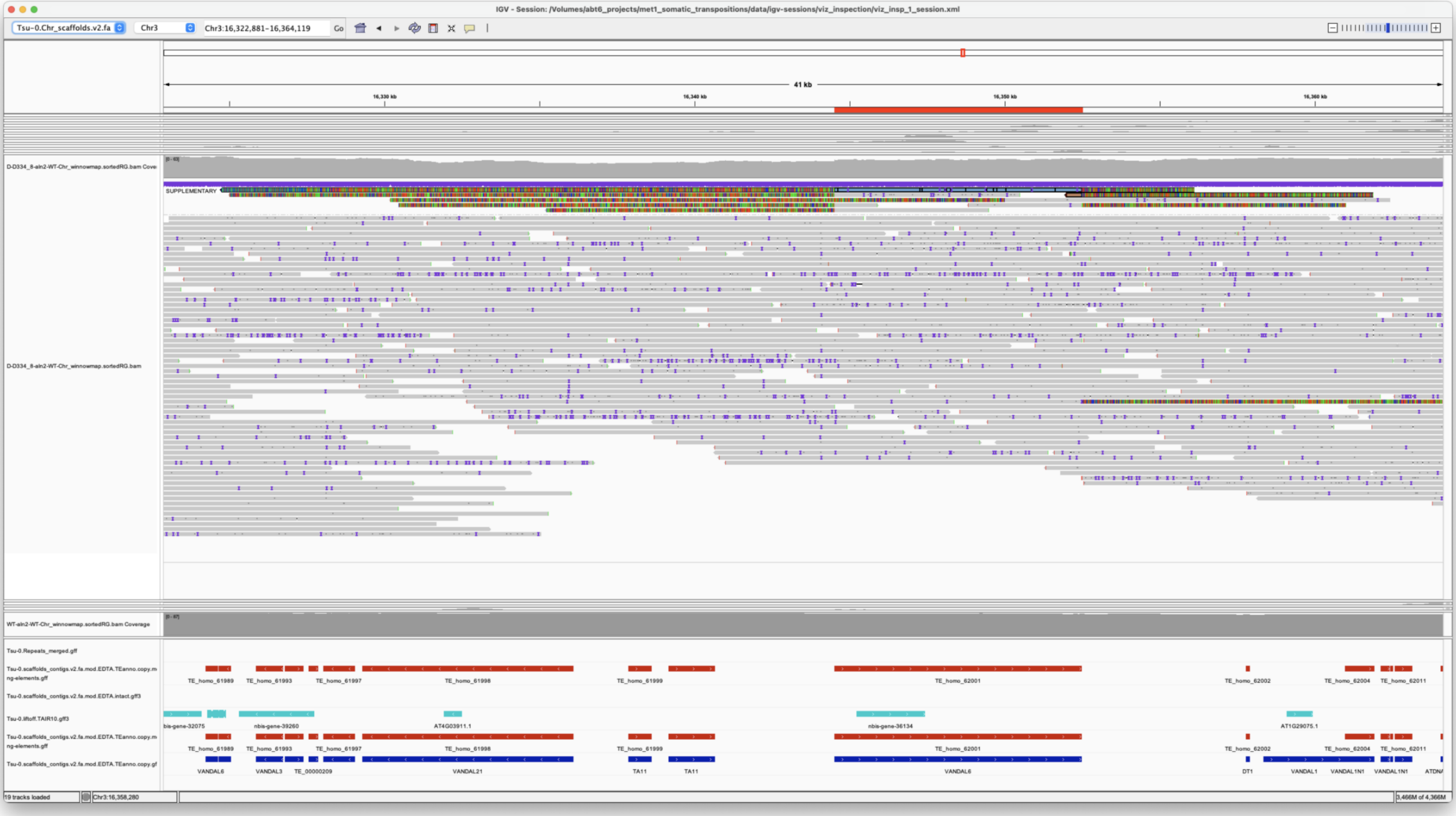

Partial

Confirmed

Chr3 12260272 12260272 + 1 Chr5;19152829;19160826;VANDAL21 m64079_221220_112036/59902159/ccs met1_08

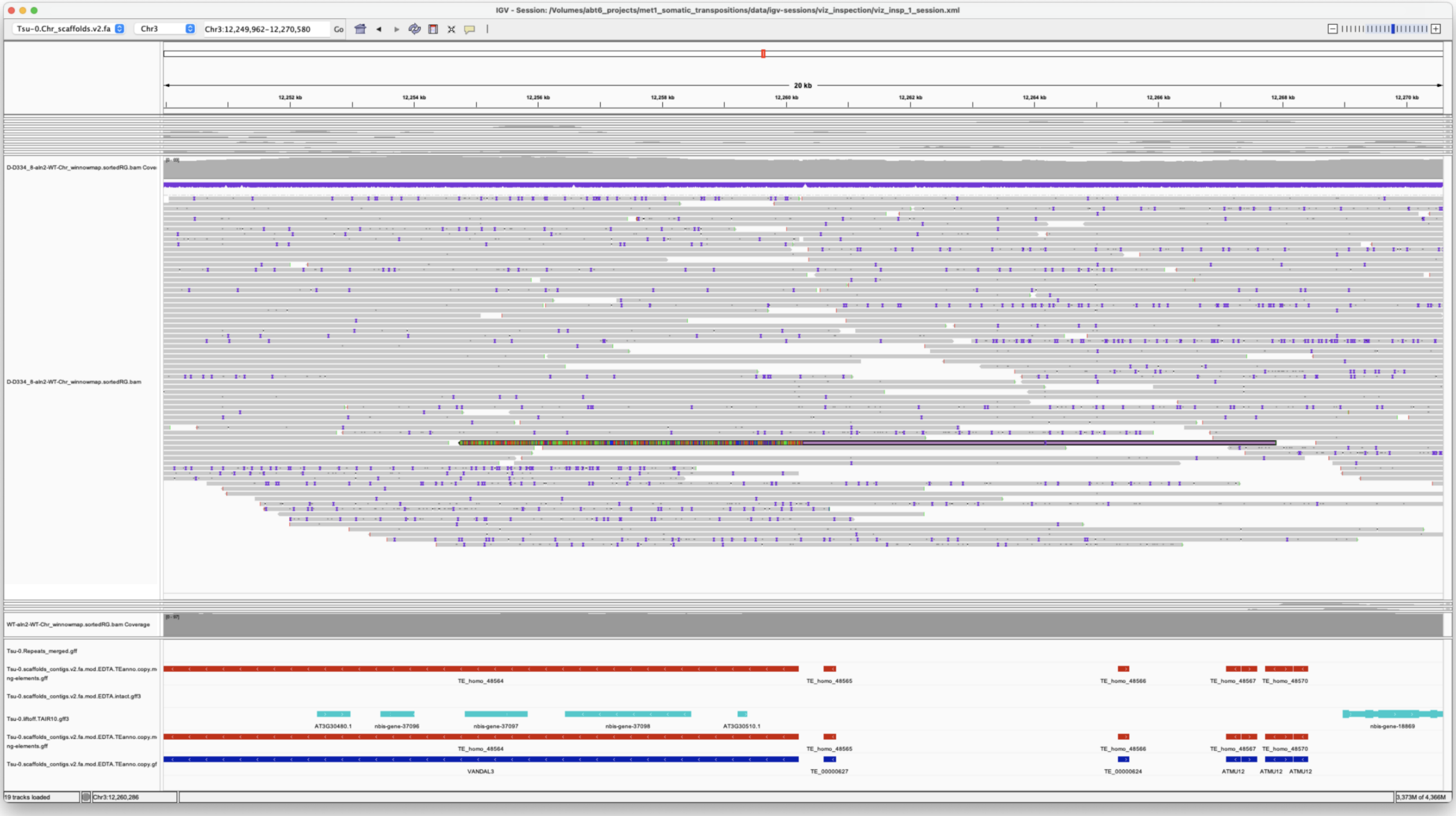

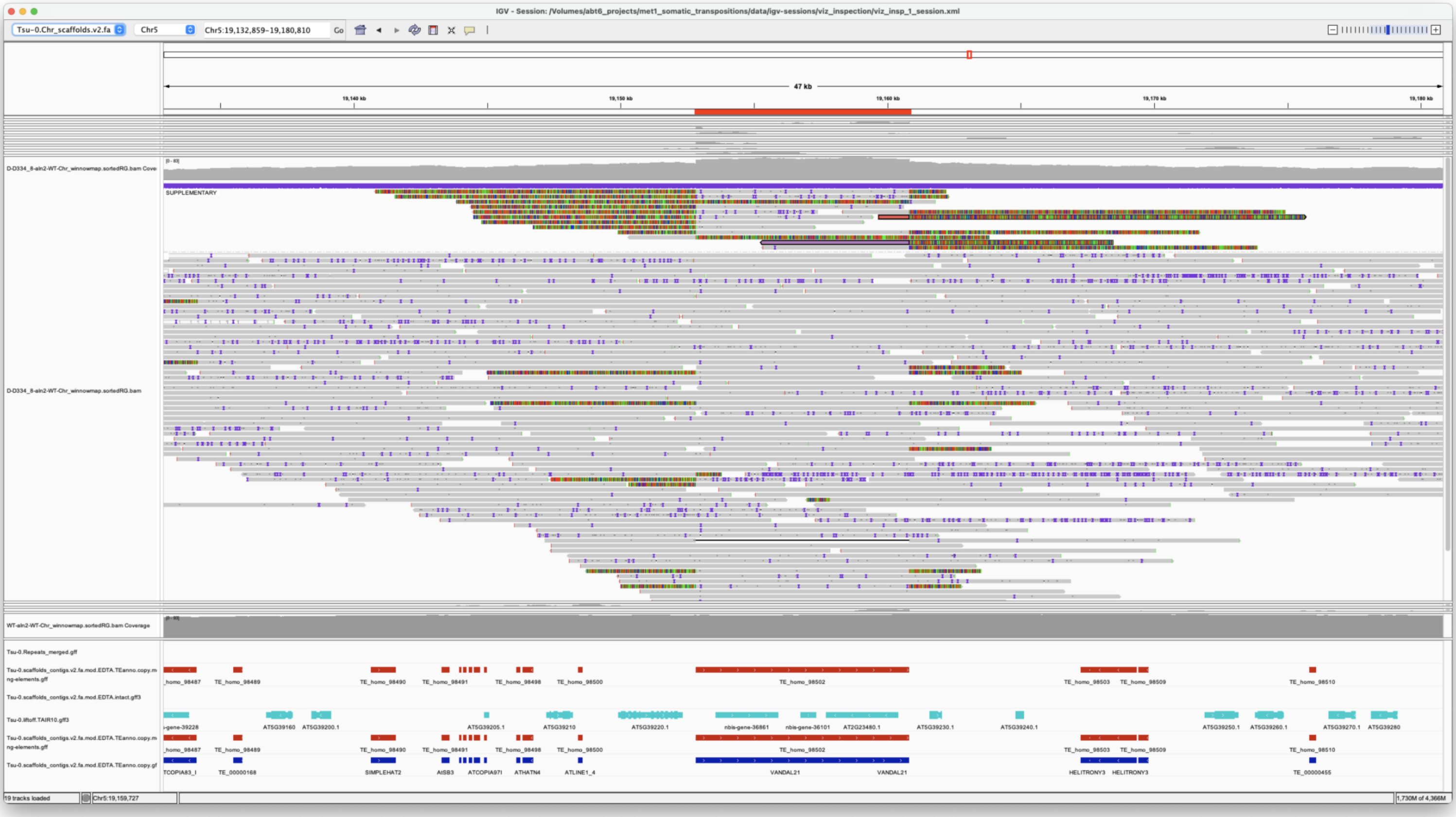

Partial

Confirmed

Chr3 18060064 18060064 + 1 Chr5;19152829;19160826;VANDAL21 m64079_221220_112036/11011008/ccs met1_08

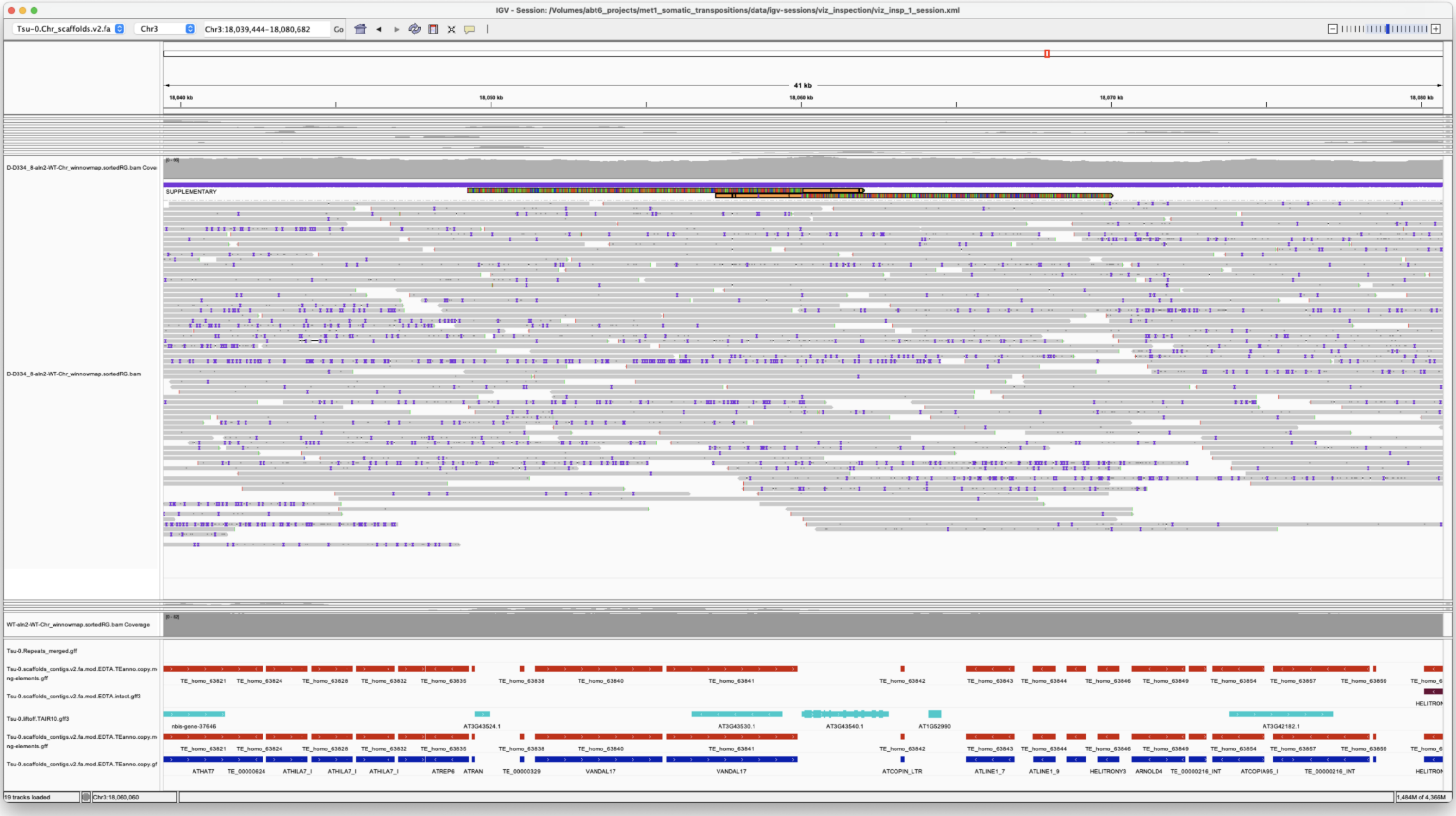

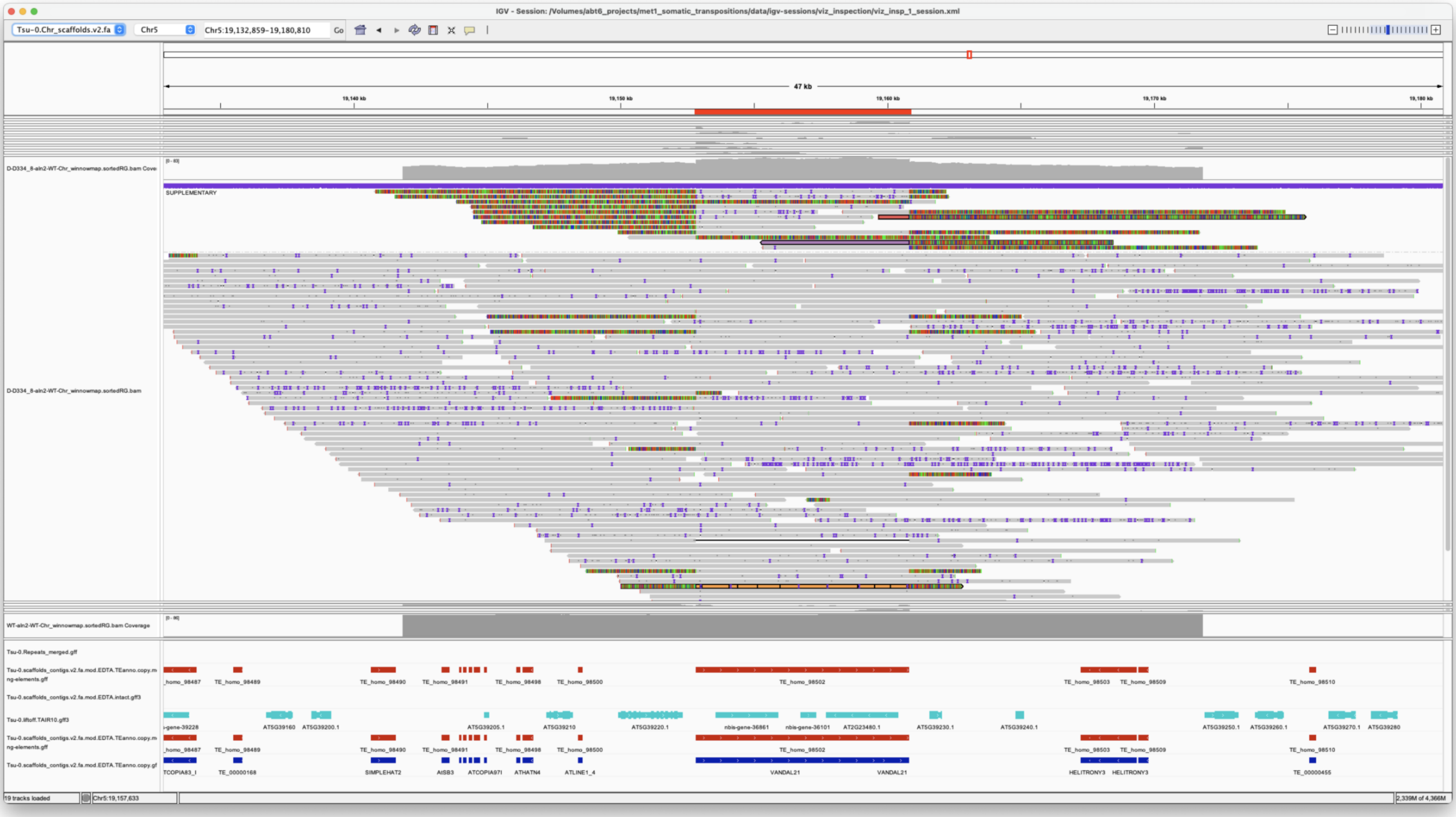

Central
TSD

Confirmed

Chr3 18437634 18437634 - 1 Chr5;21419693;21425022;ATCOPIA93_Evade m64079_221220_112036/157289134/ccs met1_08

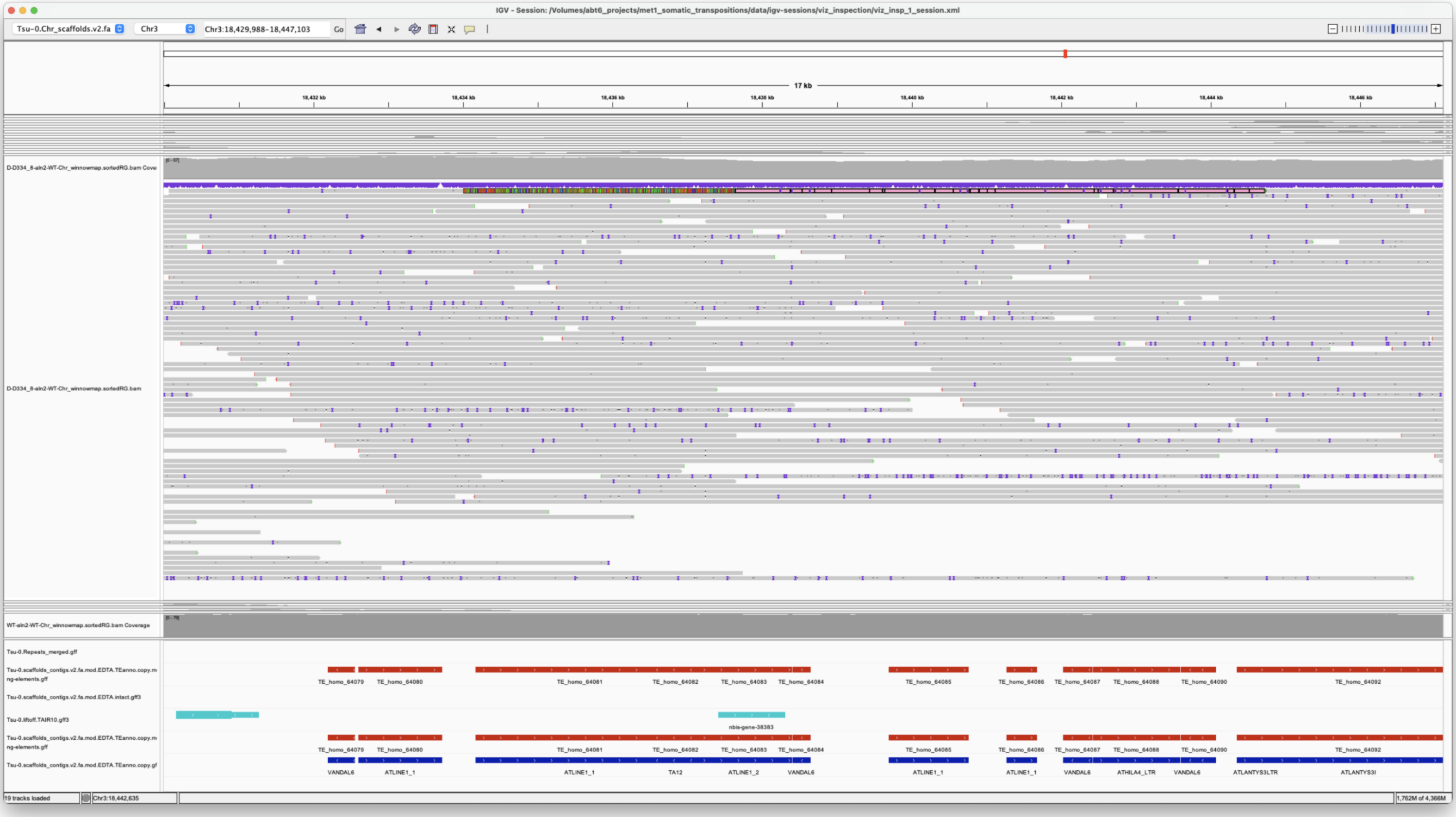

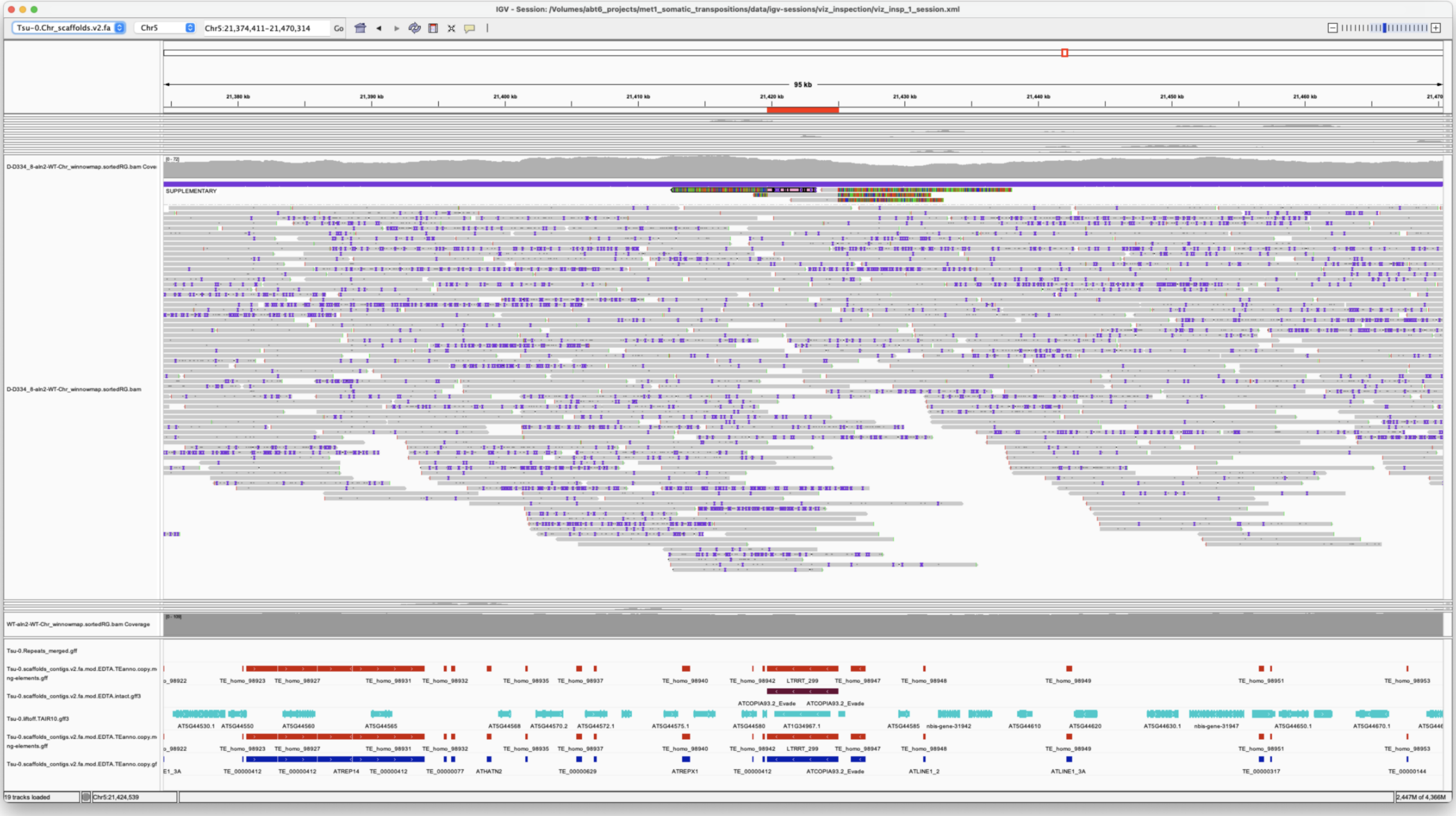

Partial

Confirmed

Chr3 19960509 19960509 - 1 Chr5;19152829;19160826;VANDAL21 m64079_240212_113350/112002216/ccs met1_08

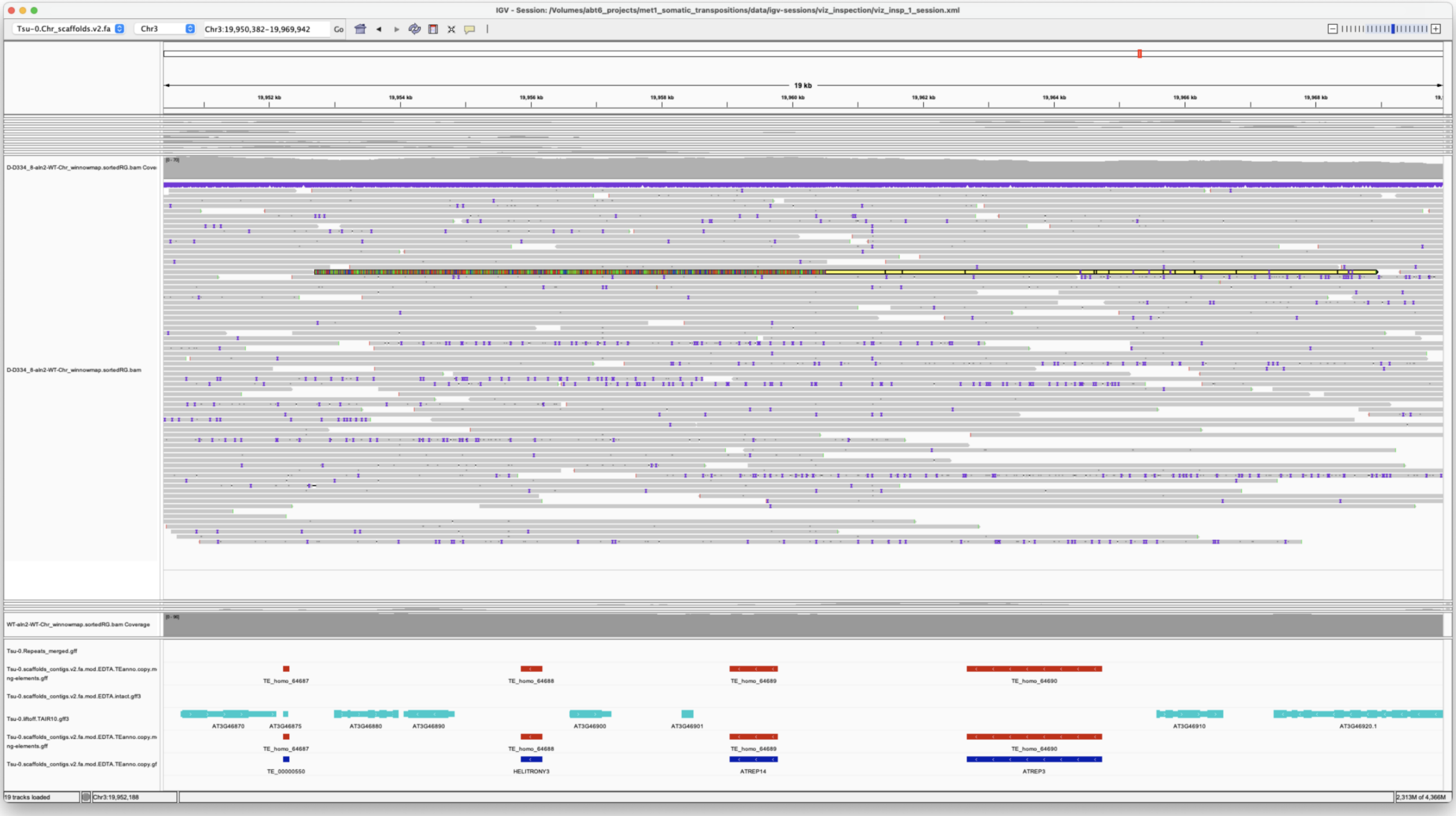

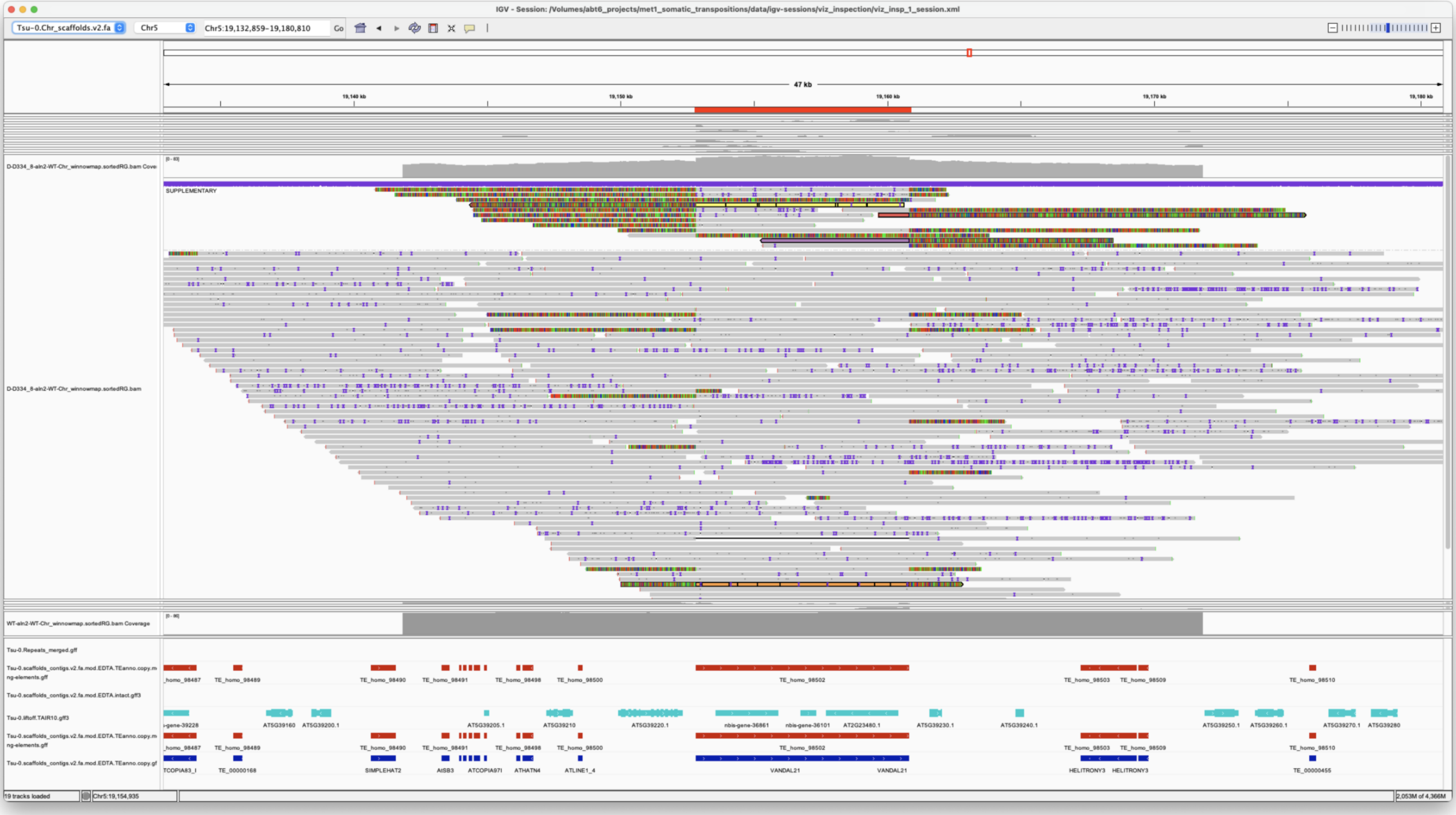

Partial

Confirmed

Chr3 23535154 23535154 + 1 Chr5;19152829;19160826;VANDAL21 m64079_240212_113350/106496594/ccs met1_08

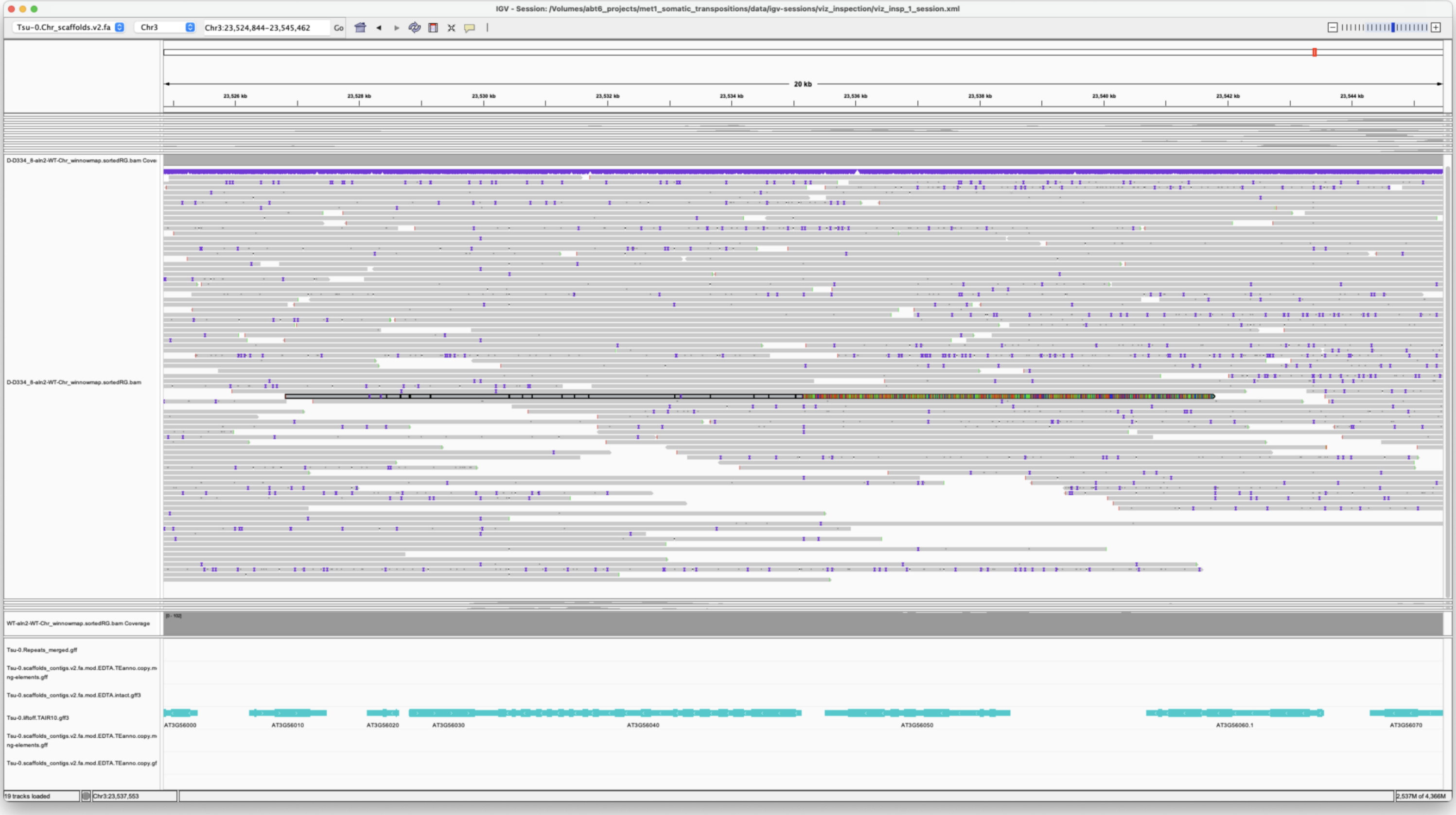

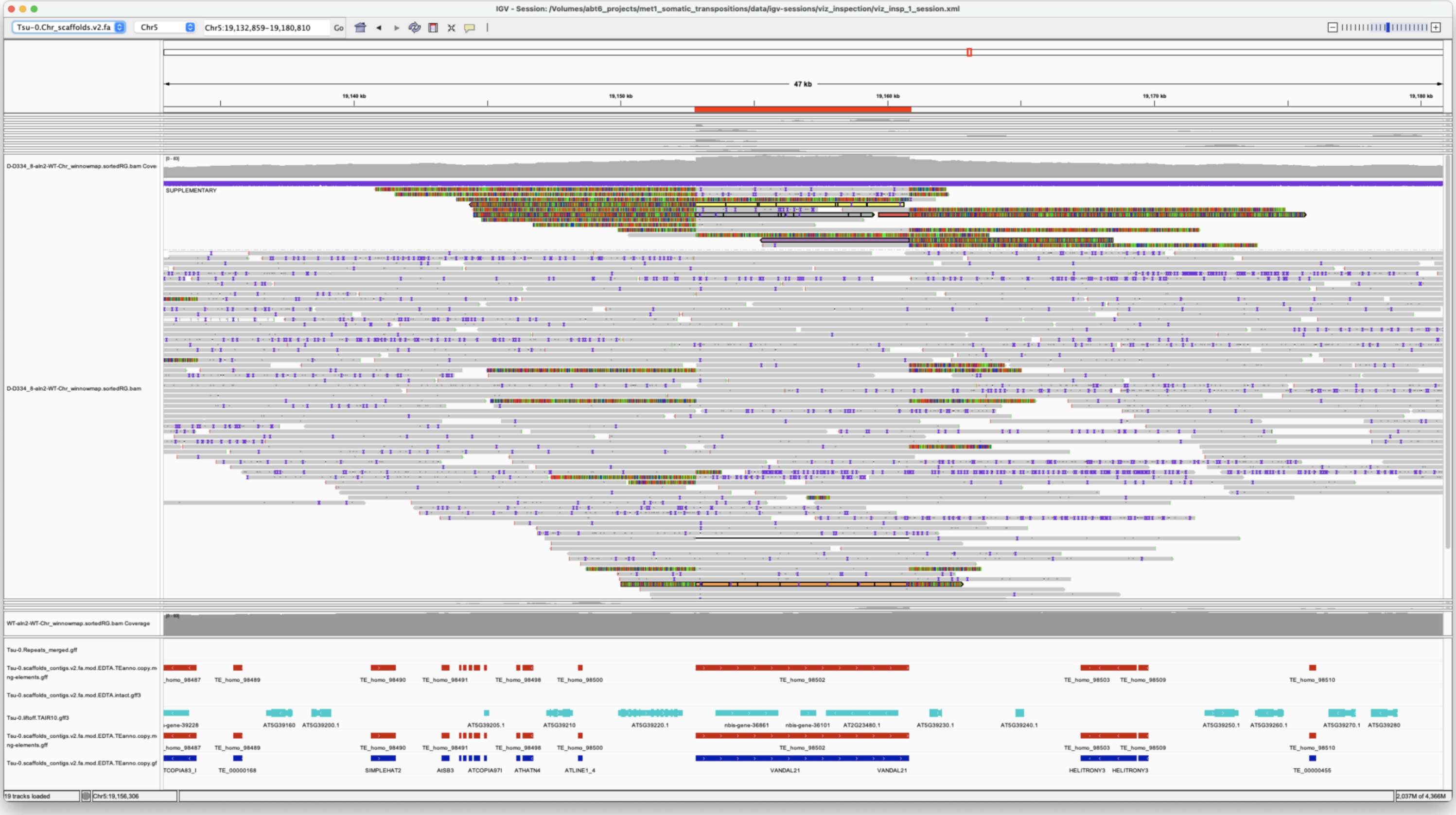

Partial

Confirmed

> Chr4 4894262 4894262 + 1 Chr5;19152829;19160826;VANDAL21 m64079_240212_113350/33884554/ccs met1_08
