# Supplementary material for: Long-read detection of transposable element mobilization in the soma of hypomethylated Arabidopsis thaliana individuals
Source: Genome Biol. 2025 Jul 30;26:231. doi: 10.1186/s13059-025-03691-7 (PMC12312487; doi:10.1186/s13059-025-03691-7)
Supplement: Supplementary file 2 — Additional file 2. Visual inspection of somatic insertion and excision events, available at https://github.com/aerilli/Somatic-transposition_met1/tree/551df407370c6528225f404ba62a073dced14b08/Supplementary-Files/Visual_inspection. [file 13059_2025_3691_MOESM2_ESM.gz › Split_Supplementary-File4/File1_SupplementaryALN_Insertions/File1_SupplementaryALN_Insertions-205-270.pdf]

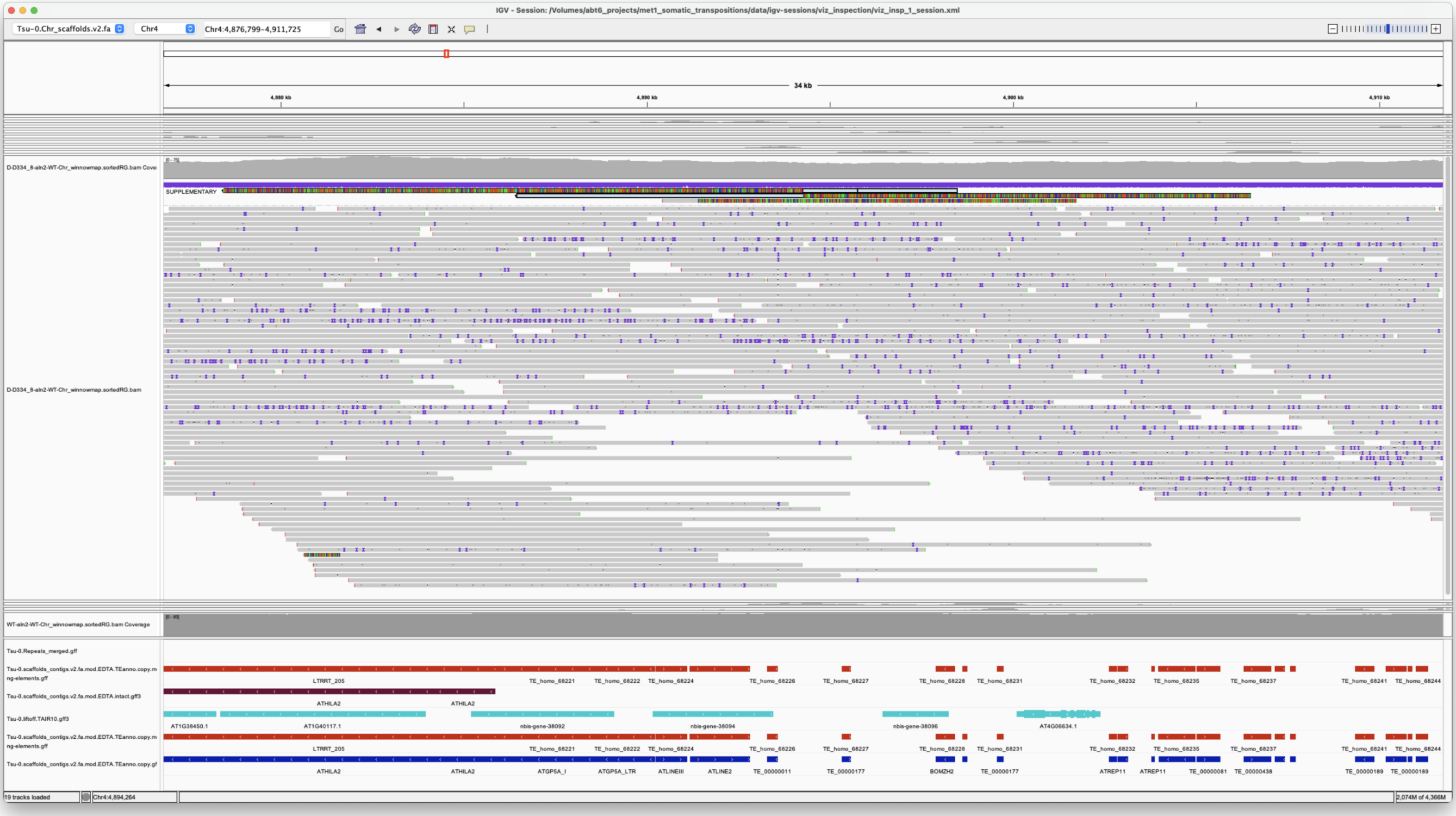

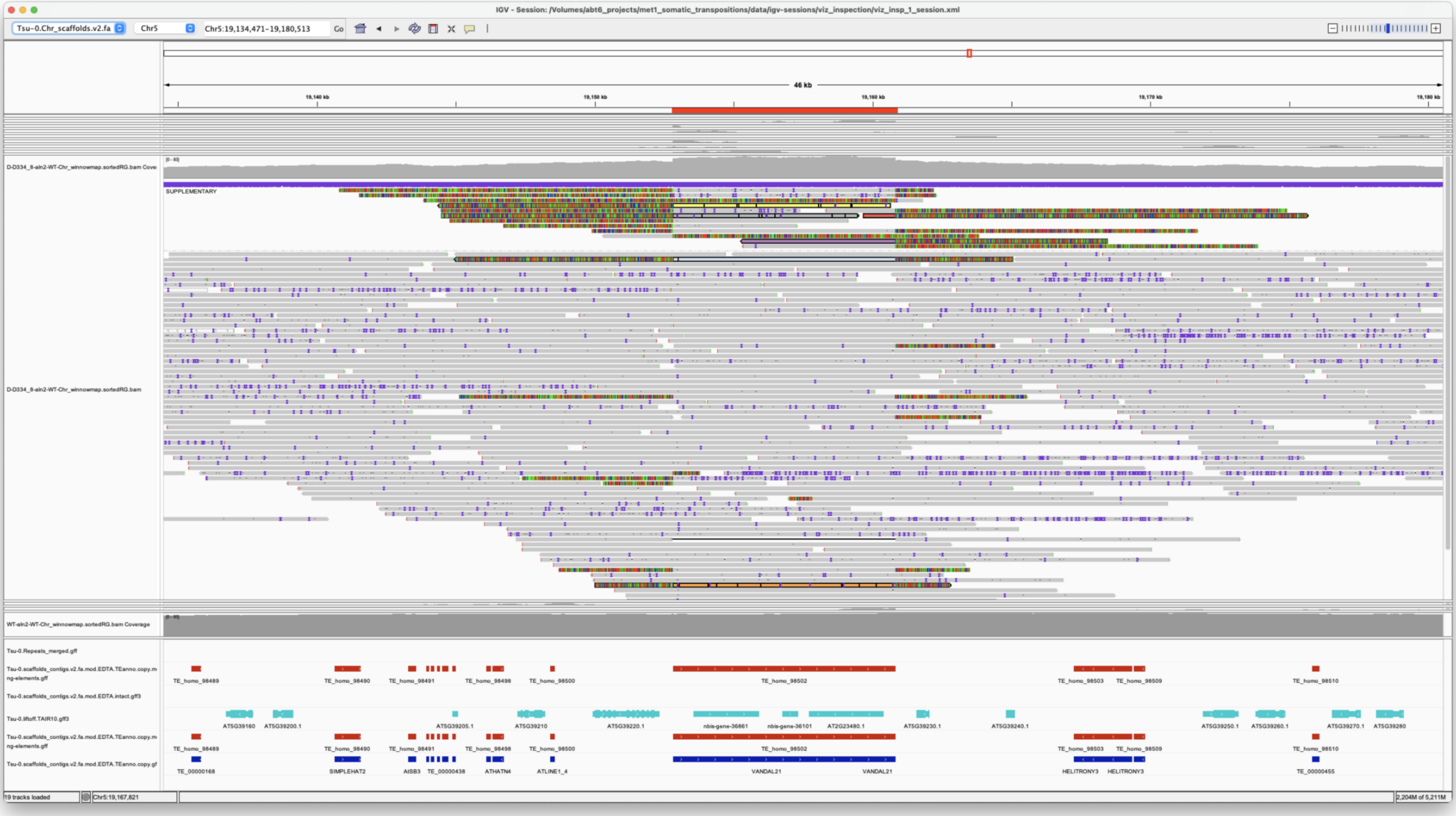

Central
TSD

Confirmed

Chr4 11843272 11843272 - 1 Chr3;16344522;16352497;VANDAL6 m64079_221220_112036/54855010/ccs met1_08

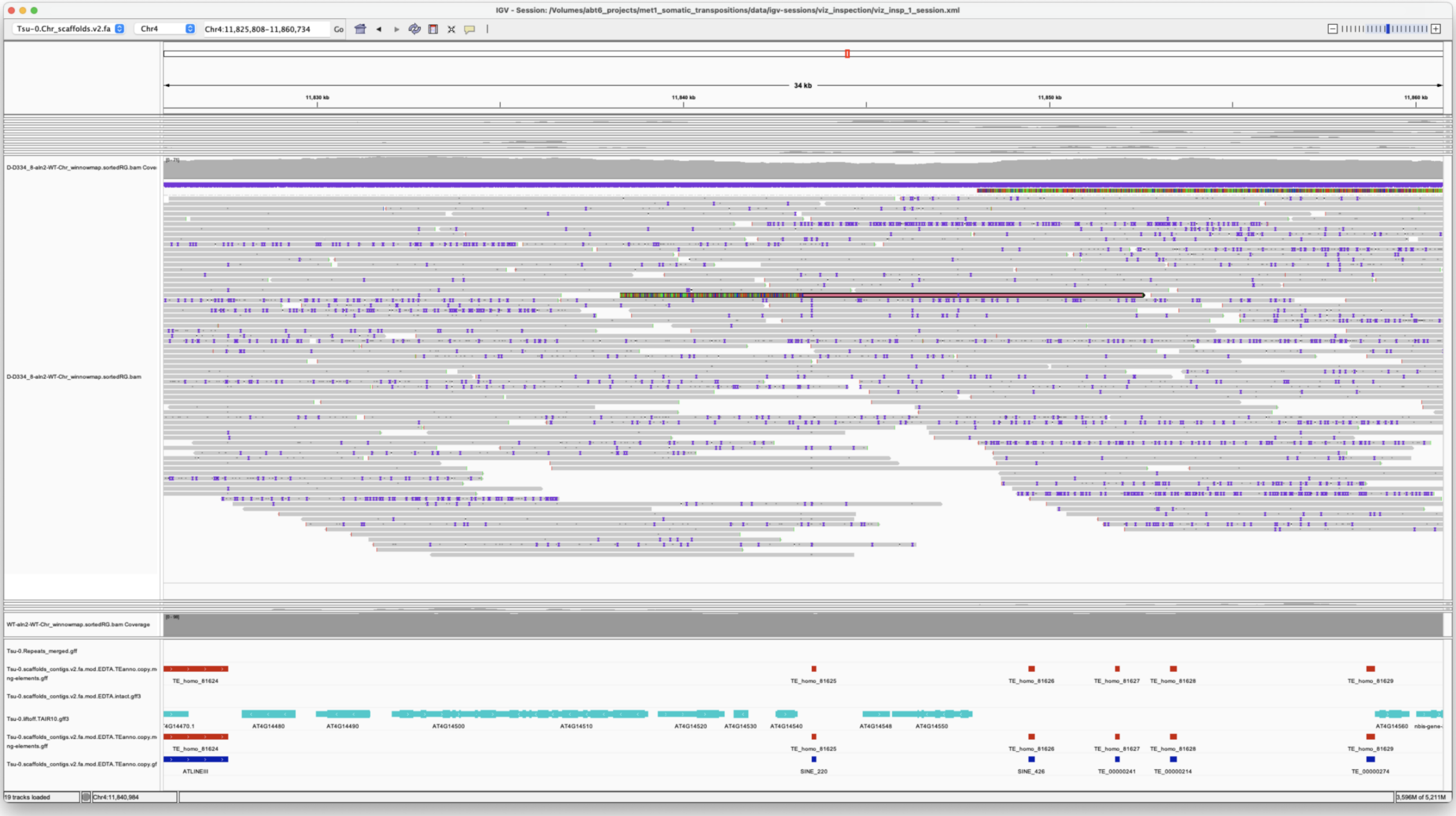

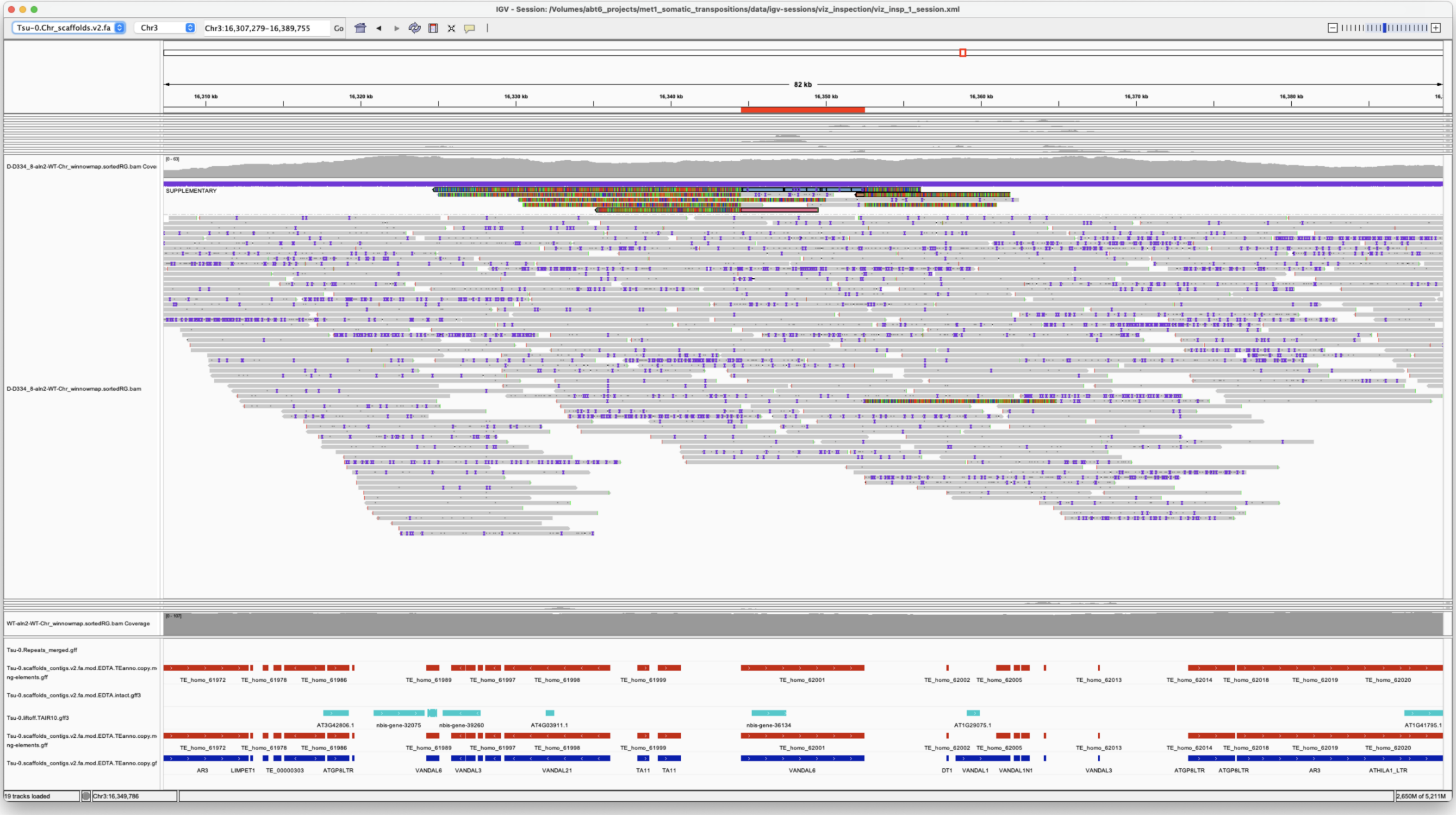

Partial

Confirmed

Chr4 19779417 19779417 + 1 Chr5;21419693;21425022;ATCOPIA93_Evade m64079_221220_112036/39651024/ccs met1_08

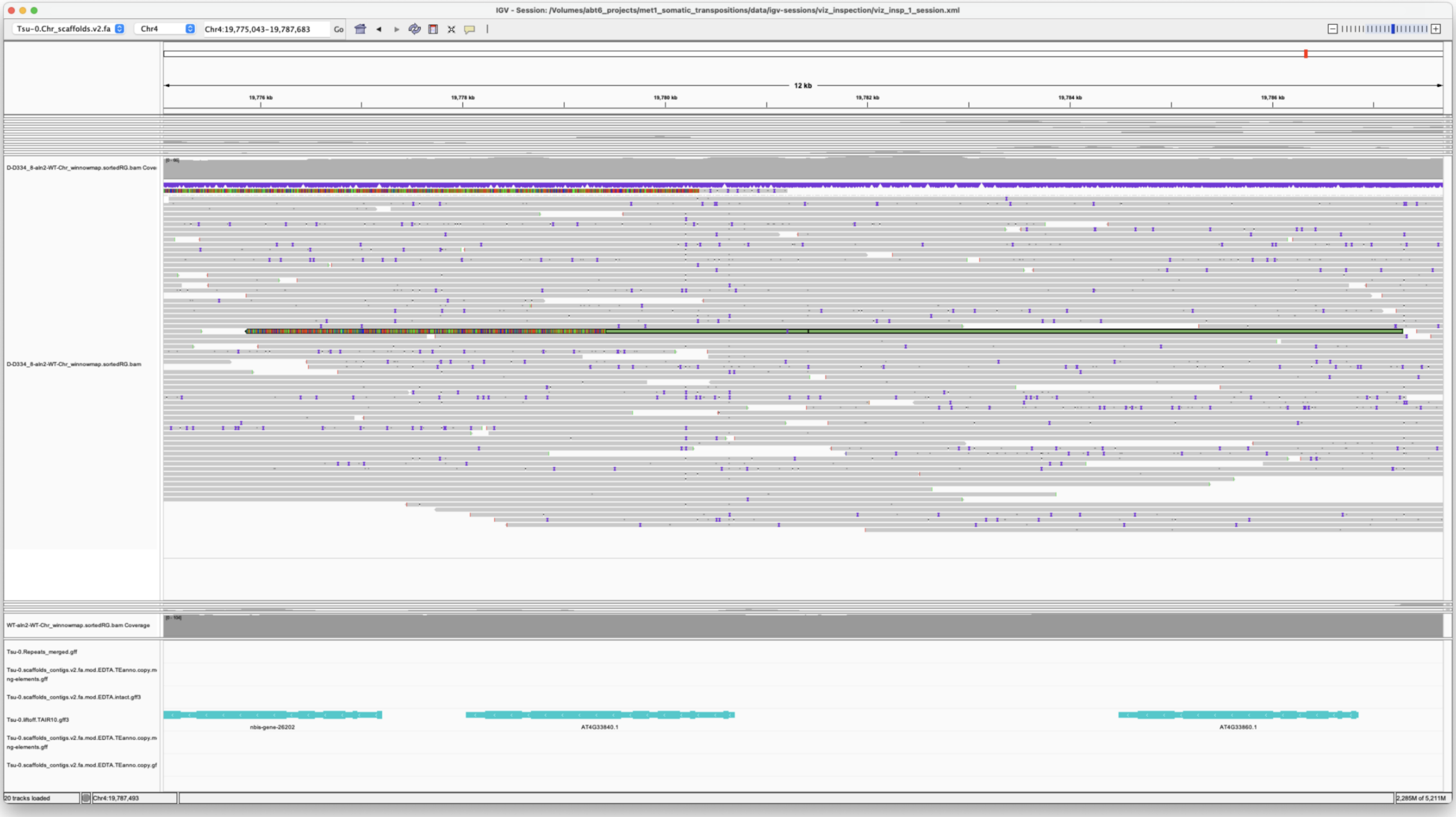

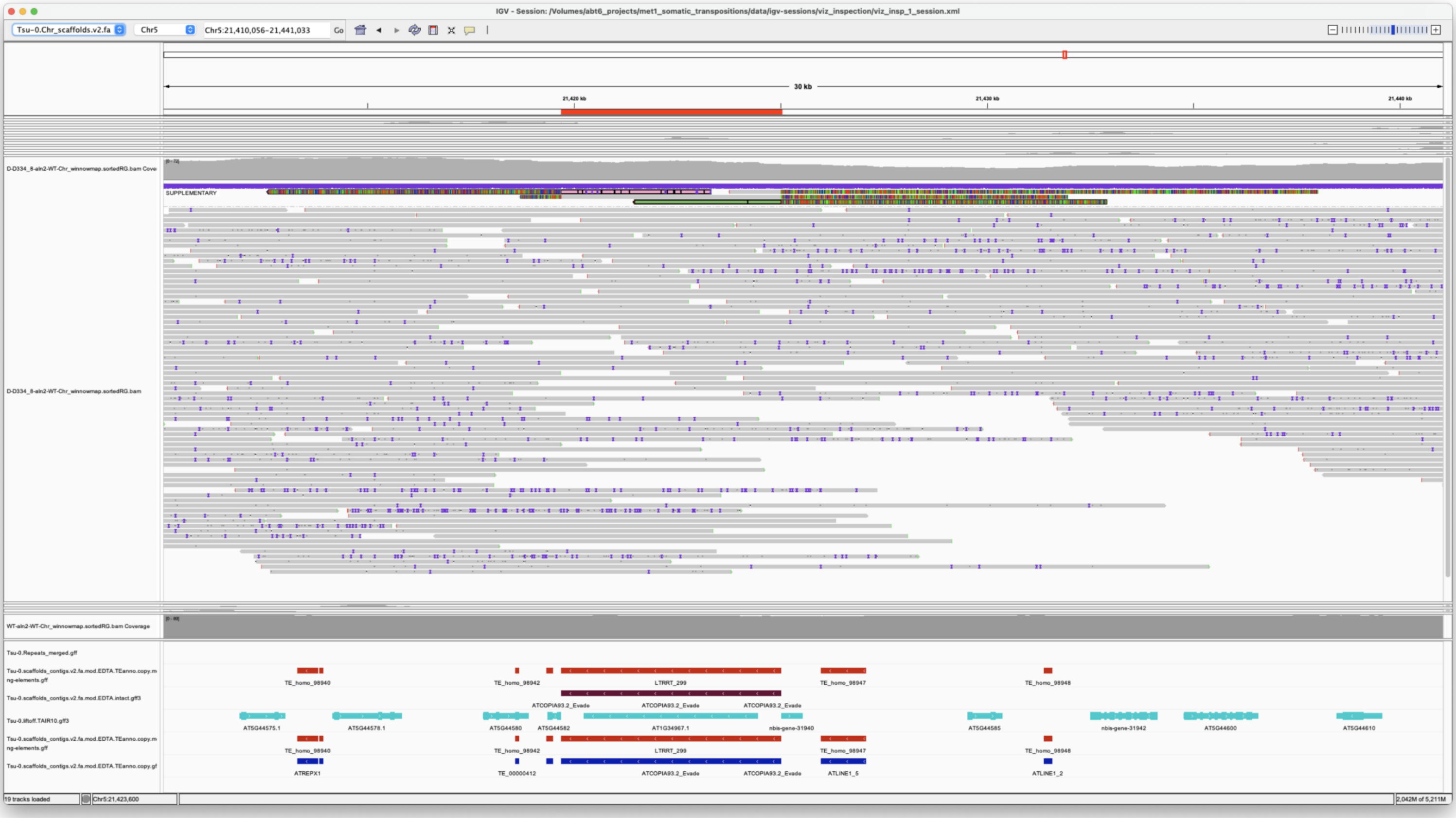

Partial

Confirmed

Chr5 5558299 5558299 + 1 Chr3;16344522;16352497;VANDAL6 m64079_240212_113350/108857957/ccs met1_08

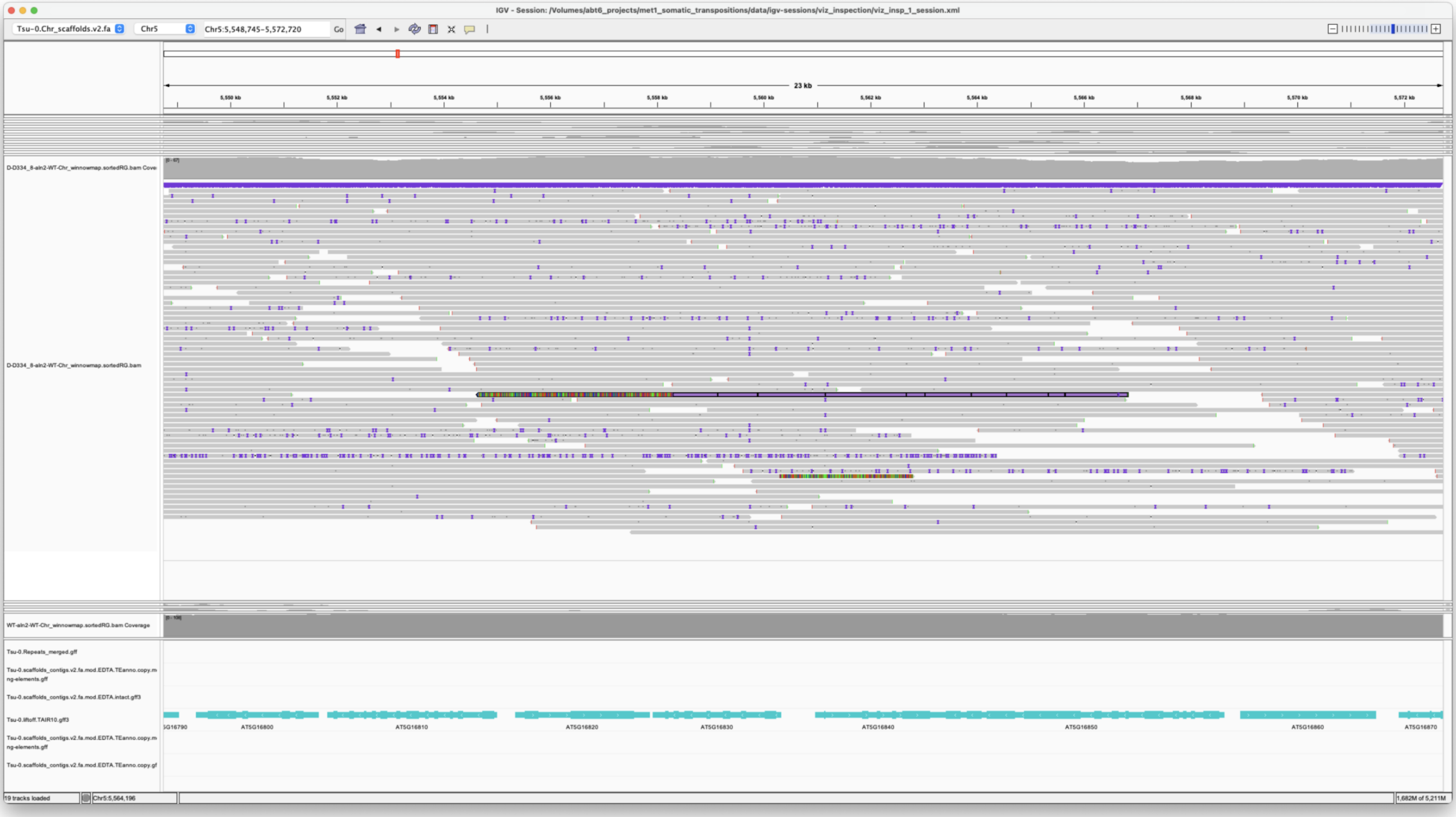

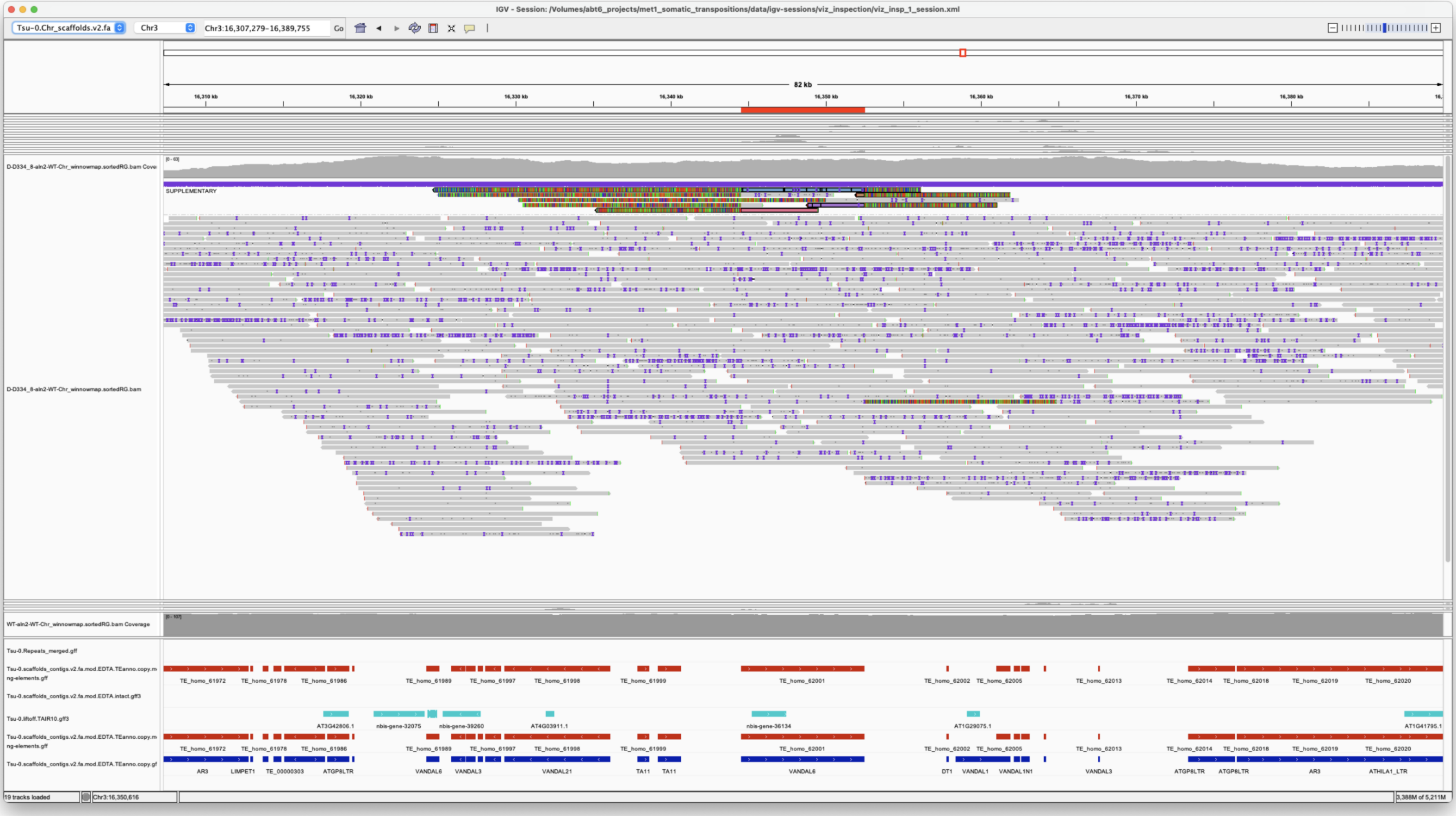

Partial

Confirmed

Chr5 5560304 5560304 - 1 Chr5;19152829;19160826;VANDAL21 m64079_221220_112036/152700670/ccs met1_08

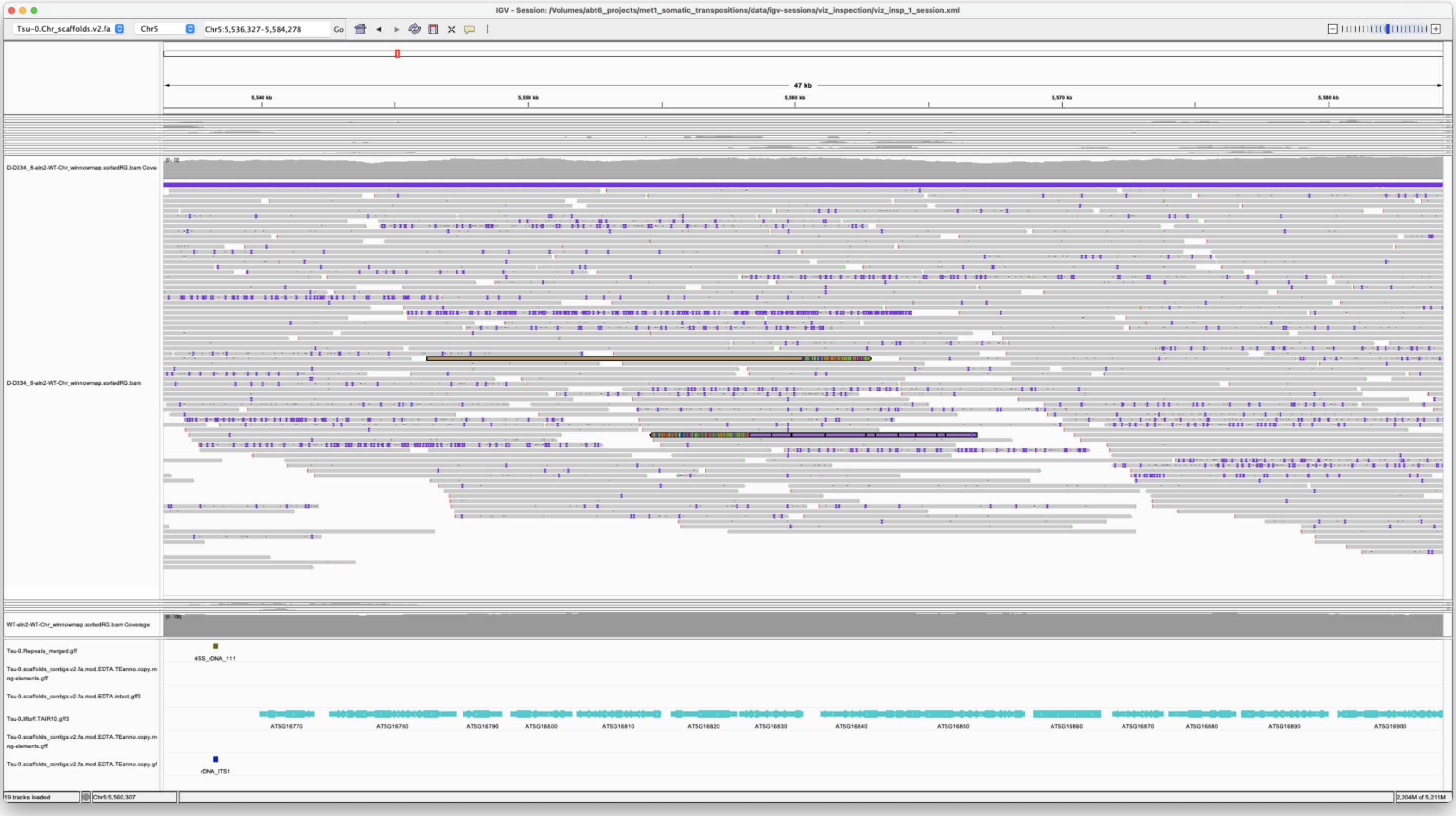

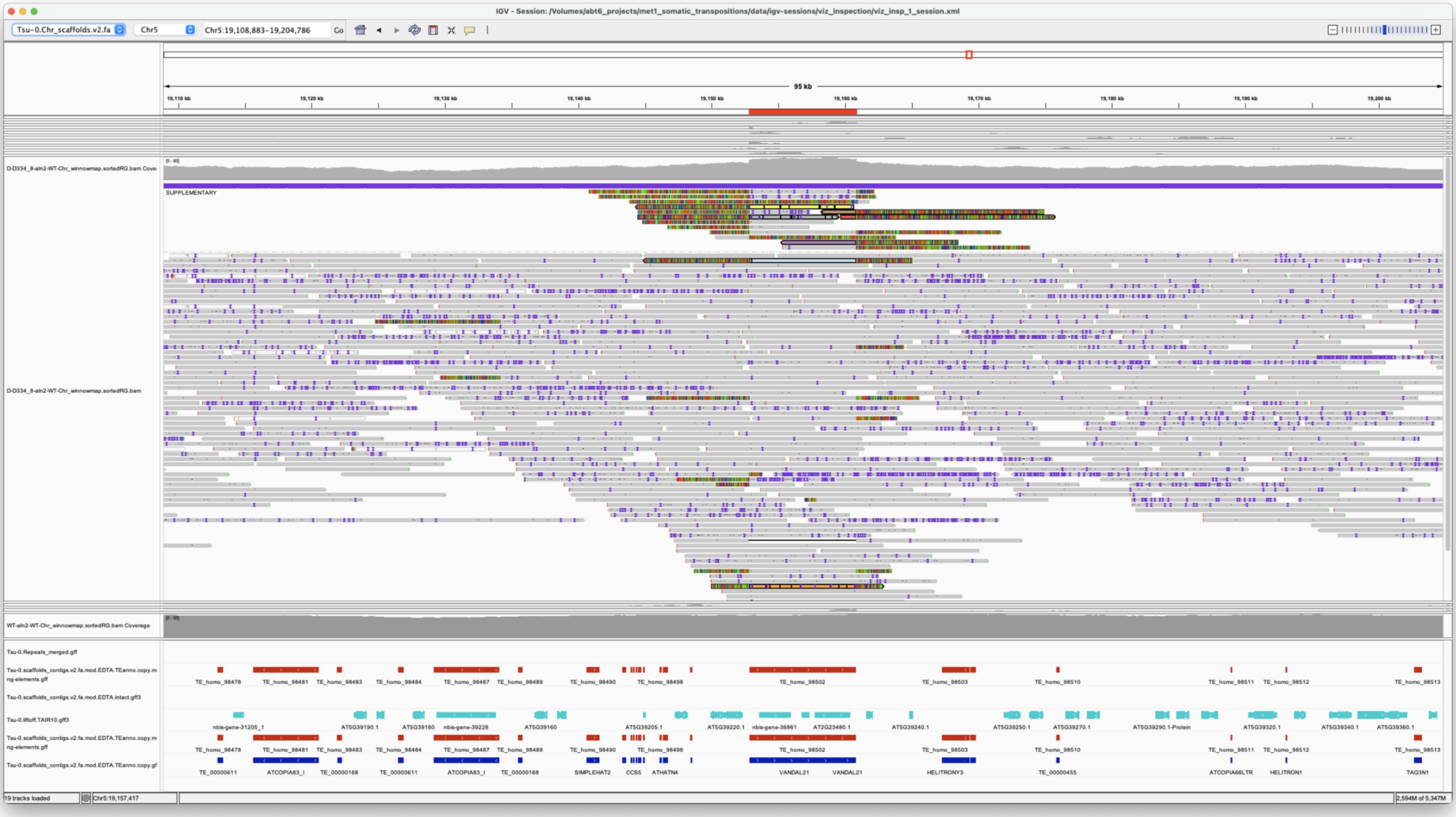

Fell Very close to a insertion site of a VANDAL6

Partial

Confirmed

Chr5 8850660 8850660 - 1 Chr5;8256939;8265133;CAC2A m64079_221220_112036/106891773/ccs met1_08

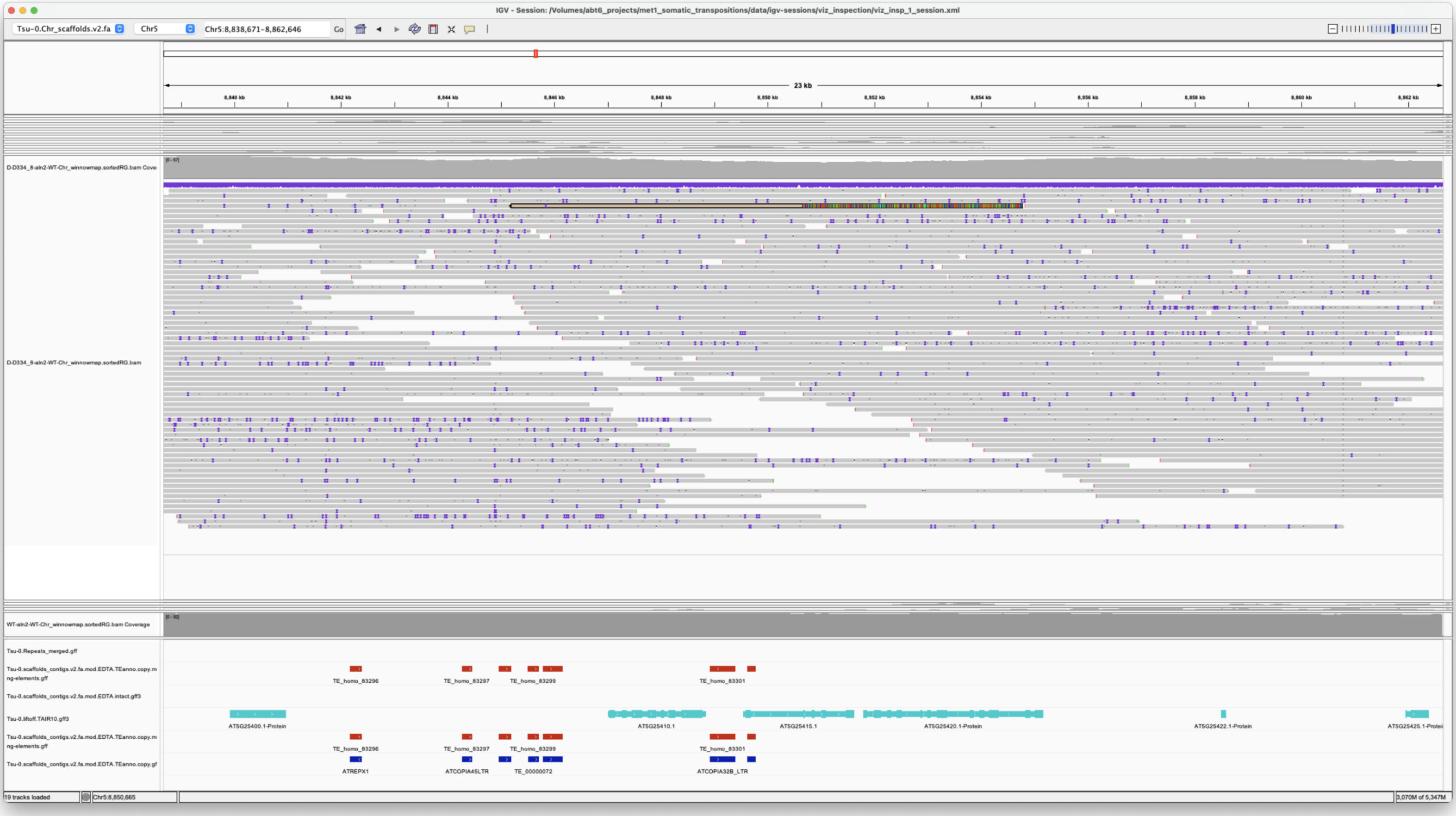

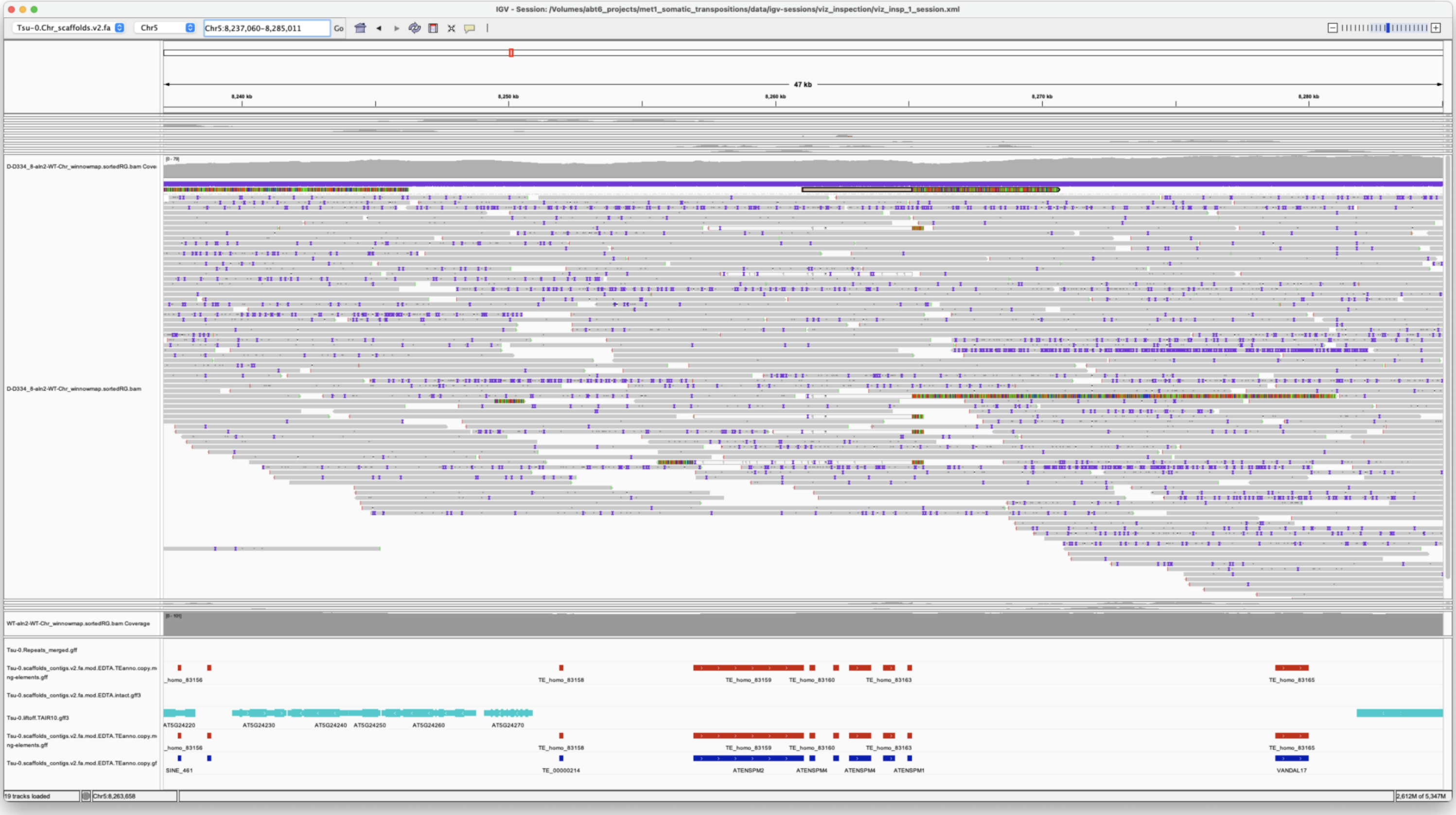

More SA reads mapping, probably because these elements are very much similar

Partial

Confirmed

Chr5 10159502 10159502 + 1 Chr5;19152829;19160826;VANDAL21 m64079_221220_112036/164495591/ccs met1_08

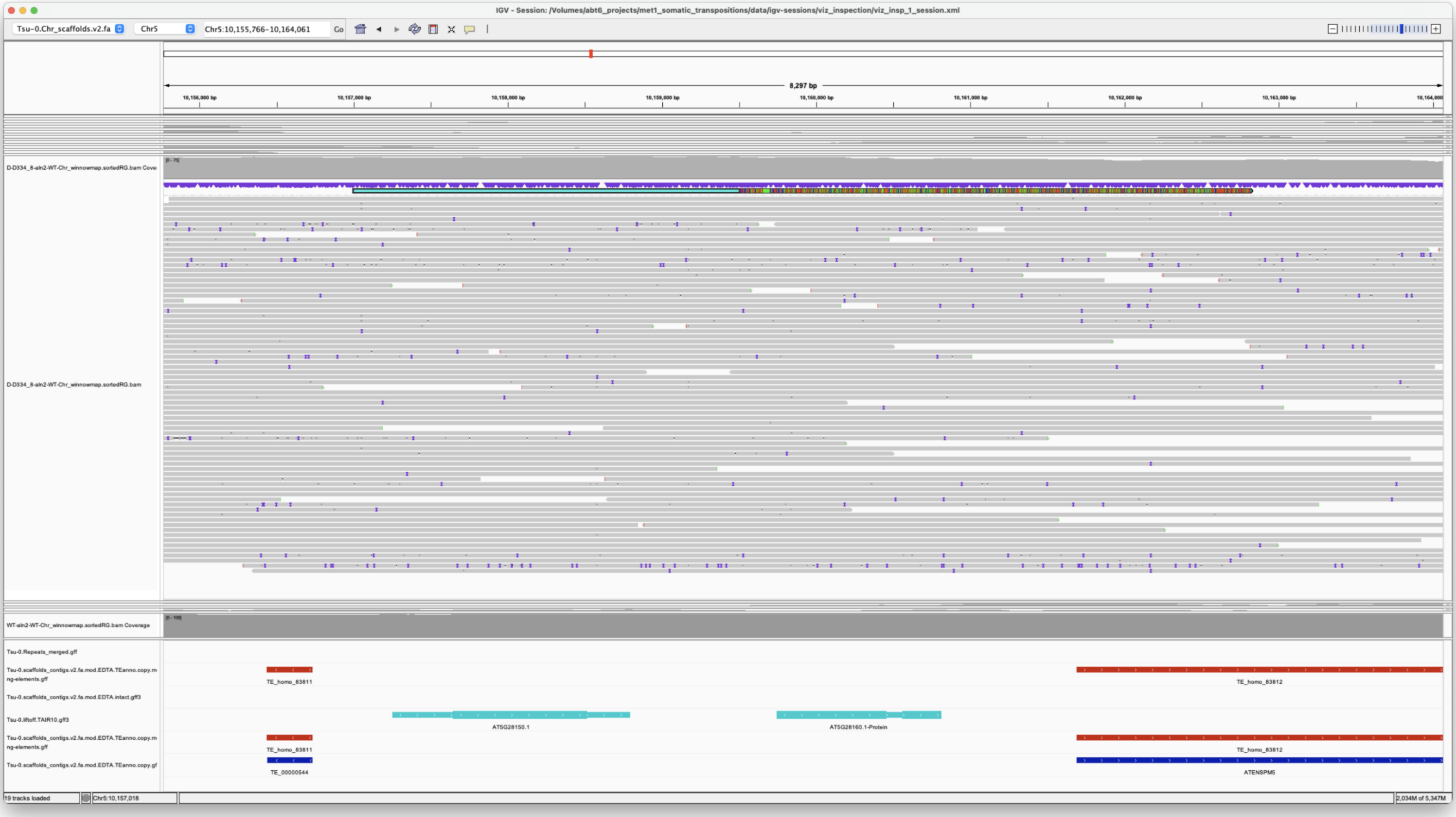

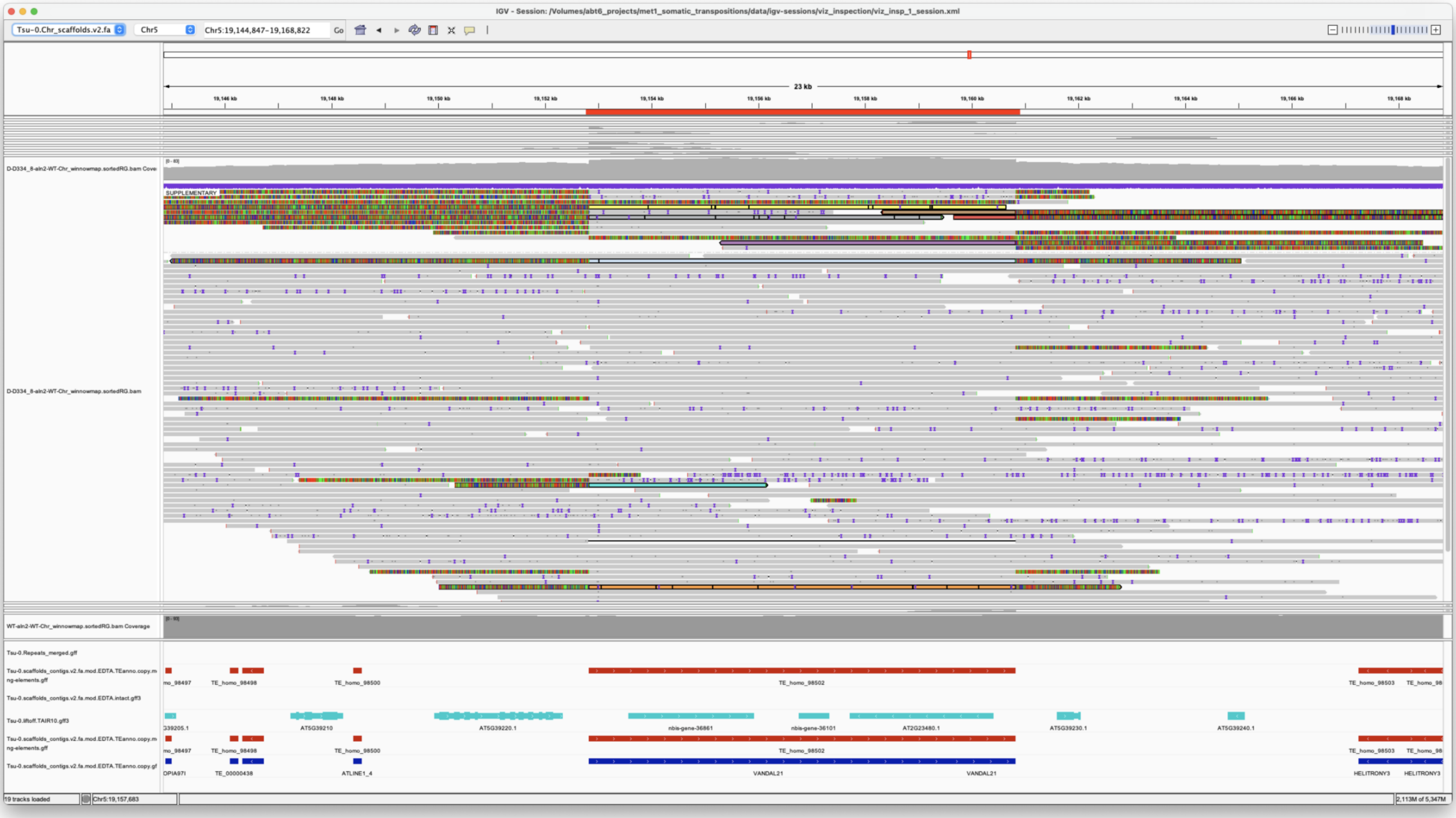

Partial

Confirmed

Chr5 13090588 13090588 + 1 Chr5;19152829;19160826;VANDAL21 m64079_221220_112036/125632727/ccs met1_08

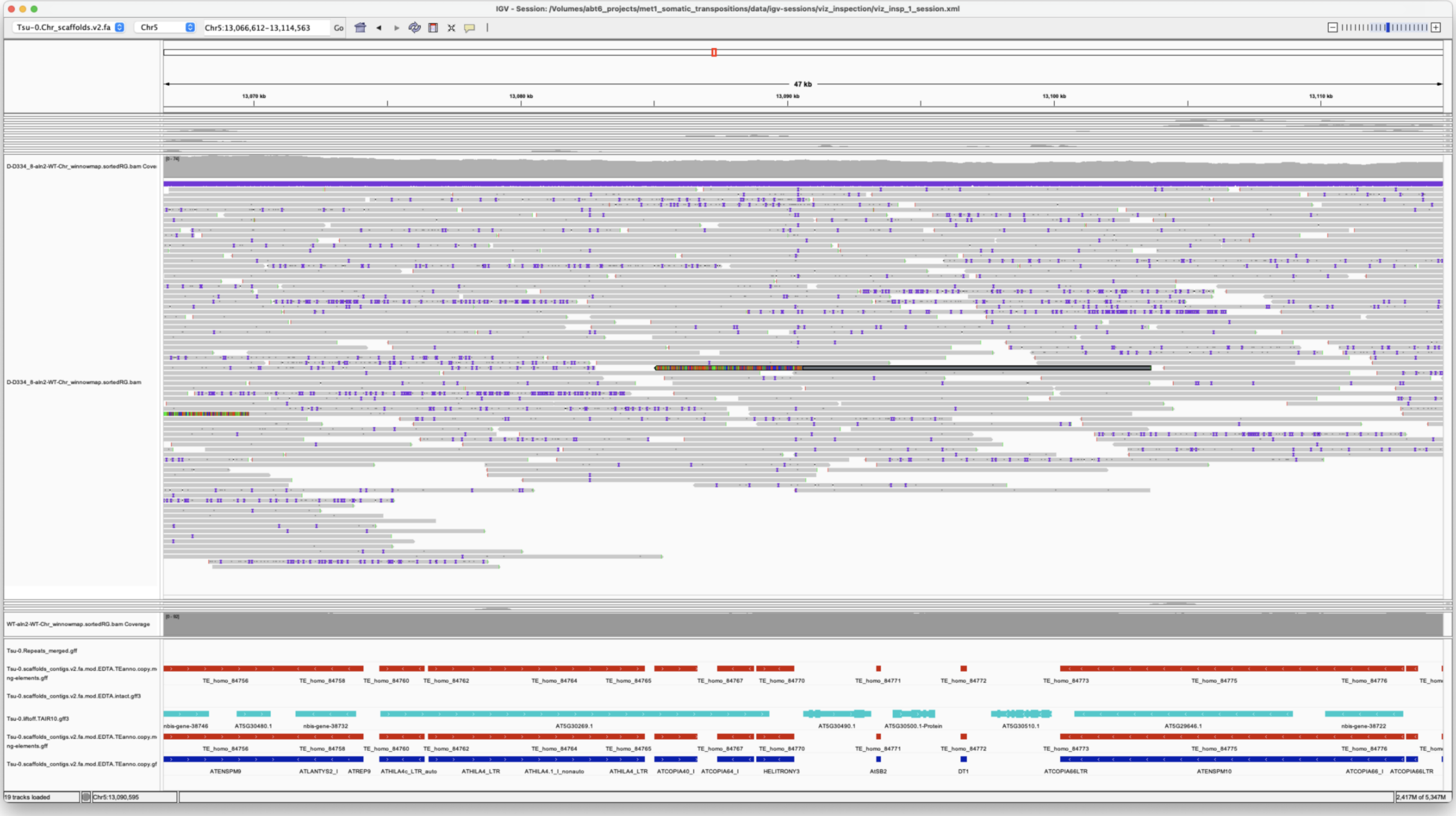

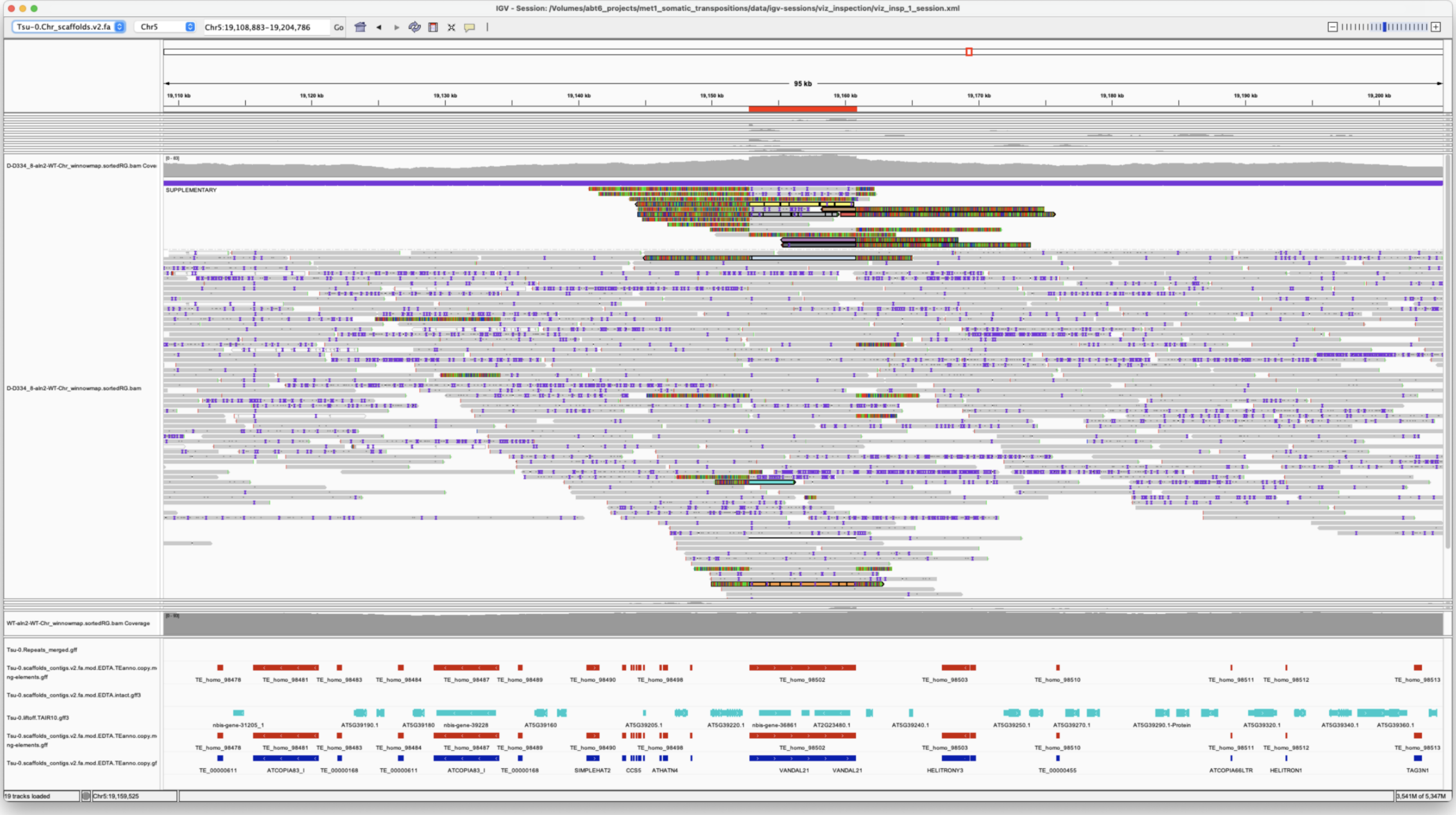

Partial

Confirmed

Chr5 13244910 13244910 + 1 Chr5;19152829;19160826;VANDAL21 m64079_240212_113350/56623671/ccs met1_08

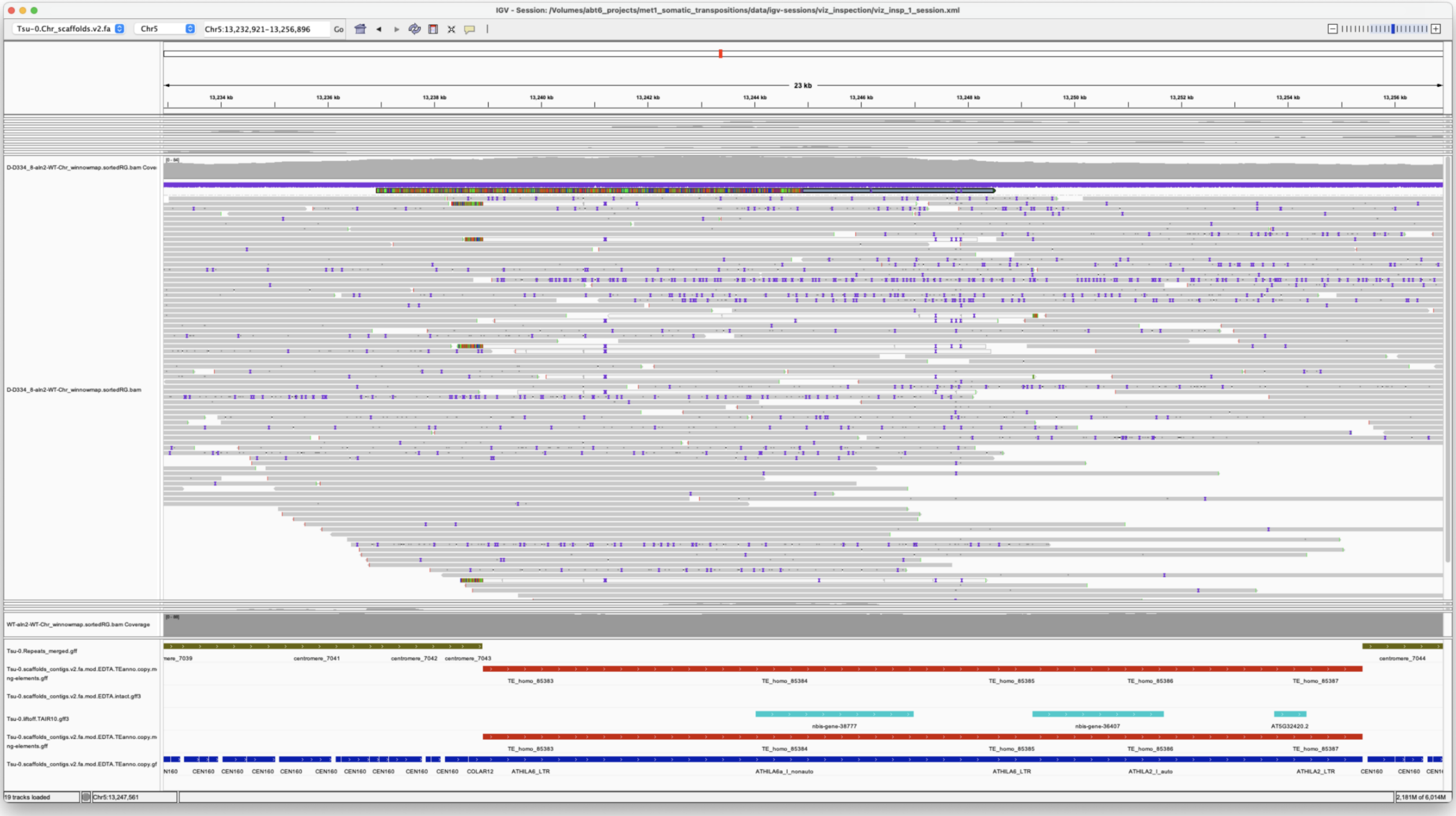

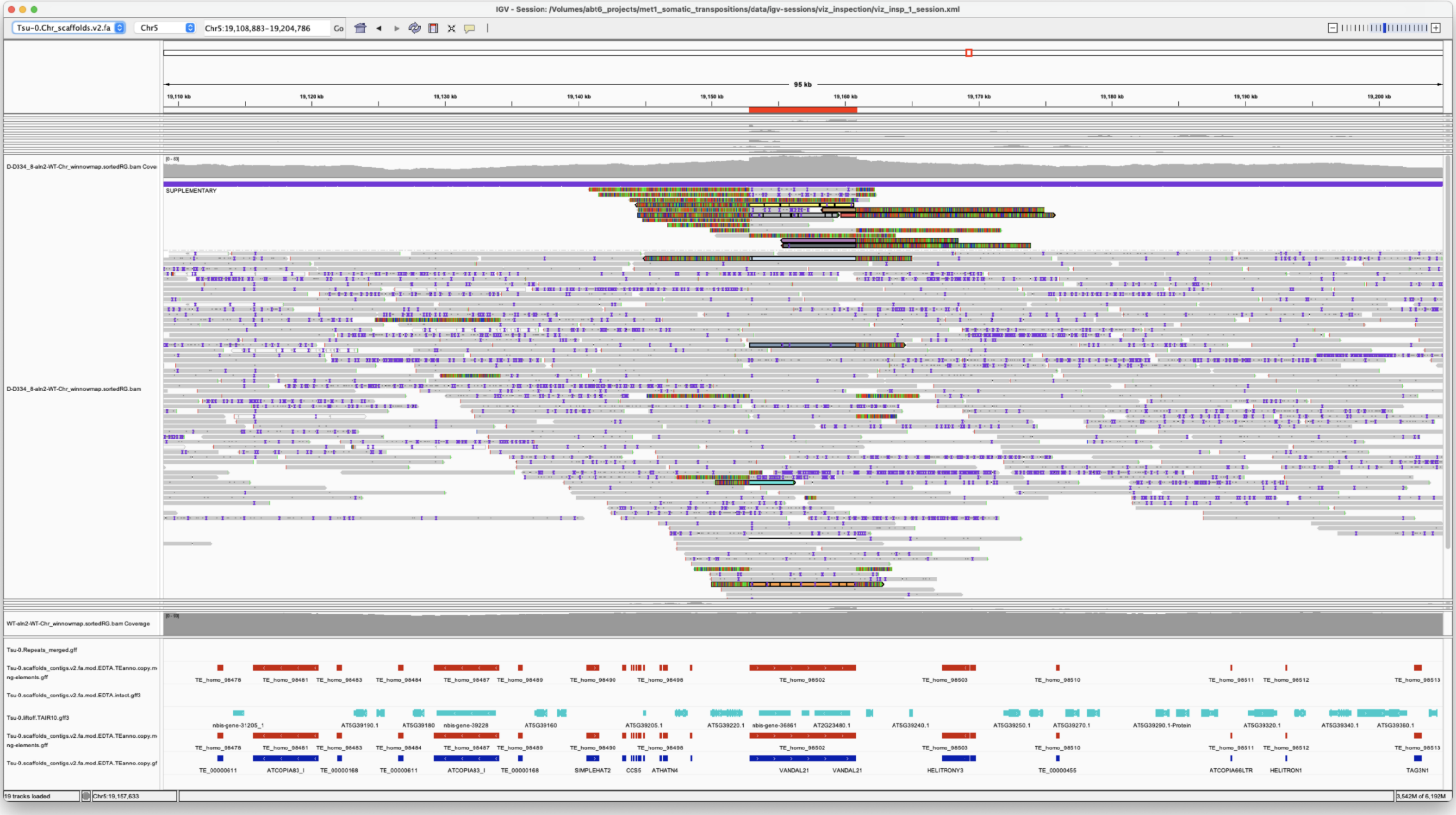

Centromeric Athila

Partial

Confirmed

| Chr5 19134139 19134141 -,- 2 Chr5;19152829;19160826;VANDAL21,Chr5;19152829;19160826;VANDAL21 m64079_221220_112036/94963270/ccs,m64079_240212_113350/128123395/ccs met1_08

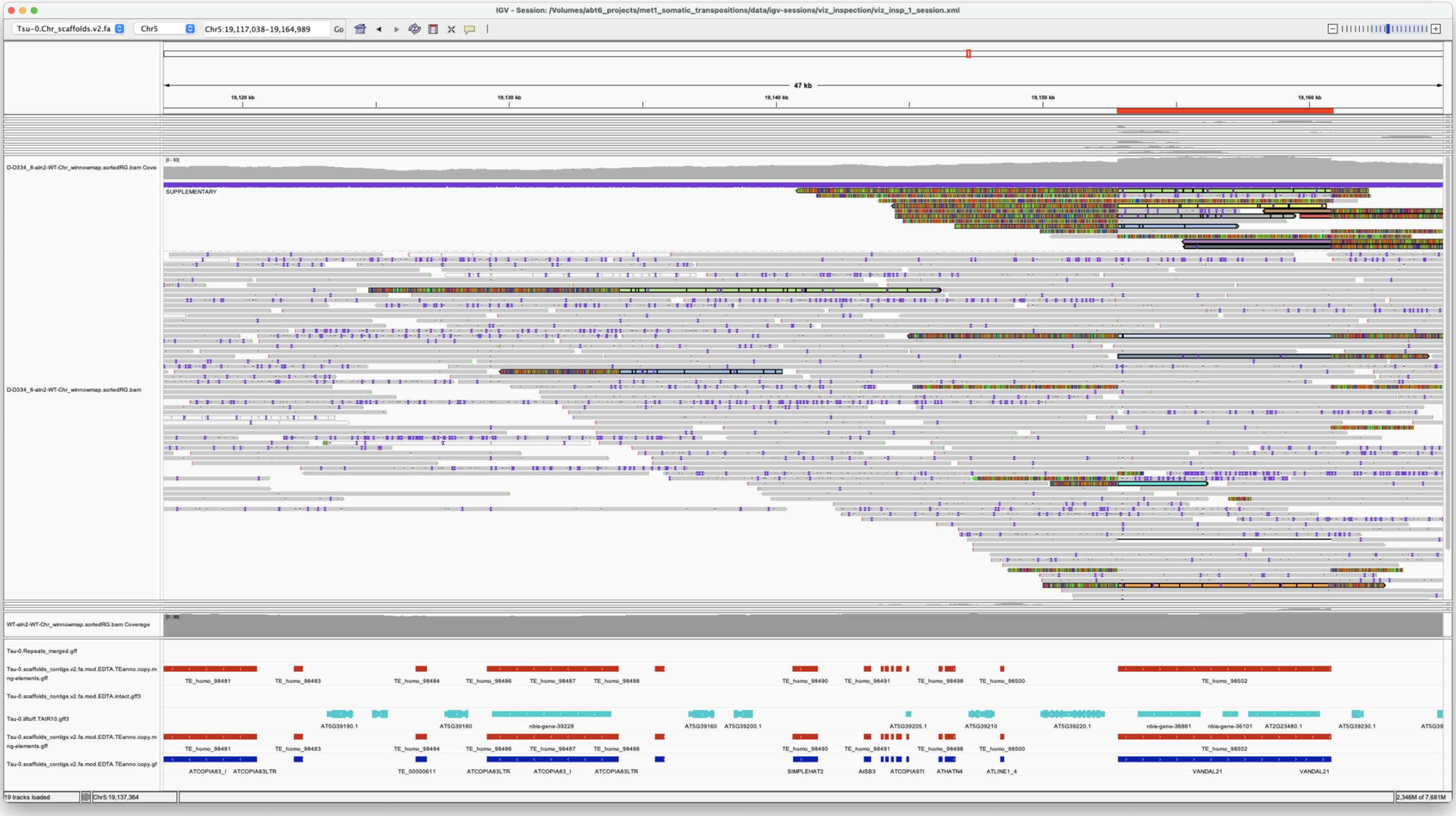

Why the green read mapping to the TE has a right clipped part that is longer than the right clipped when the same read maps to the neighbouring lest side?

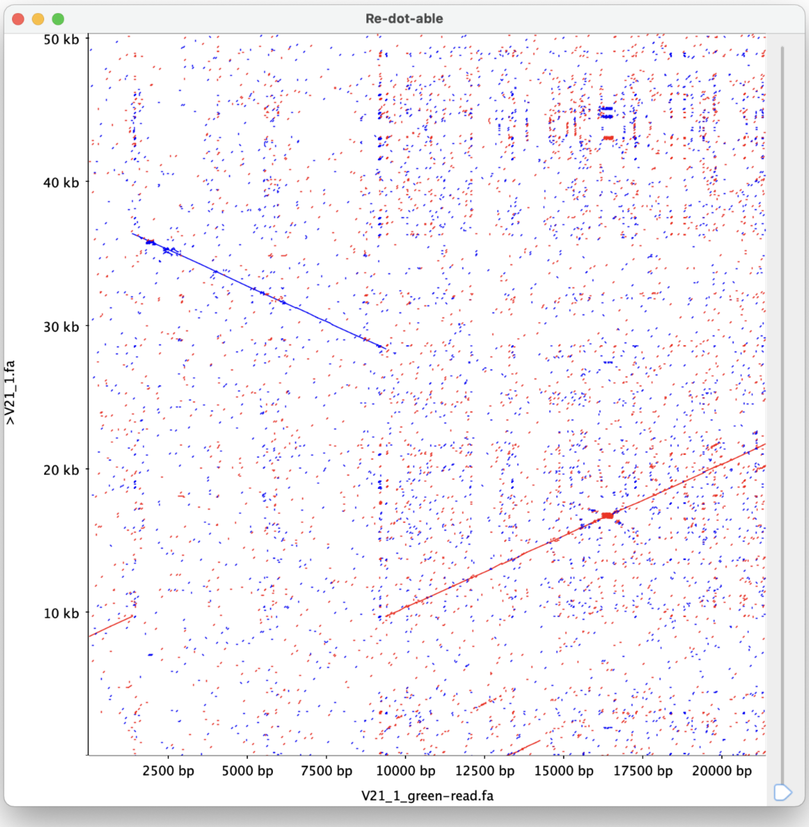

but looks good enough from this that it is an insertion with central configuration

Partial

Confirmed

Chr5 25821316 25821316 - 1 Chr3;16344522;16352497;VANDAL6 m64079_221220_112036/8848491/ccs met1_08

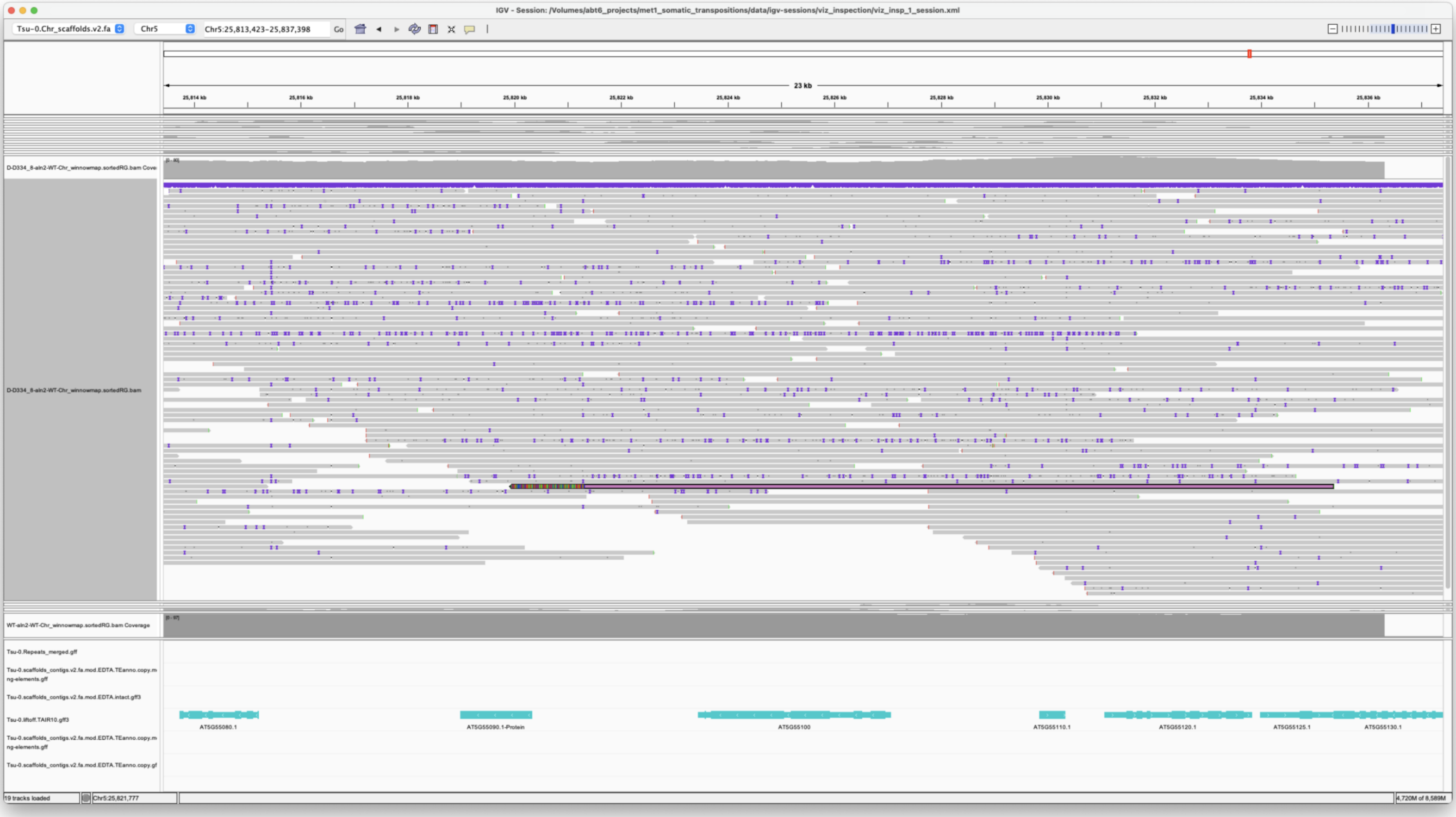

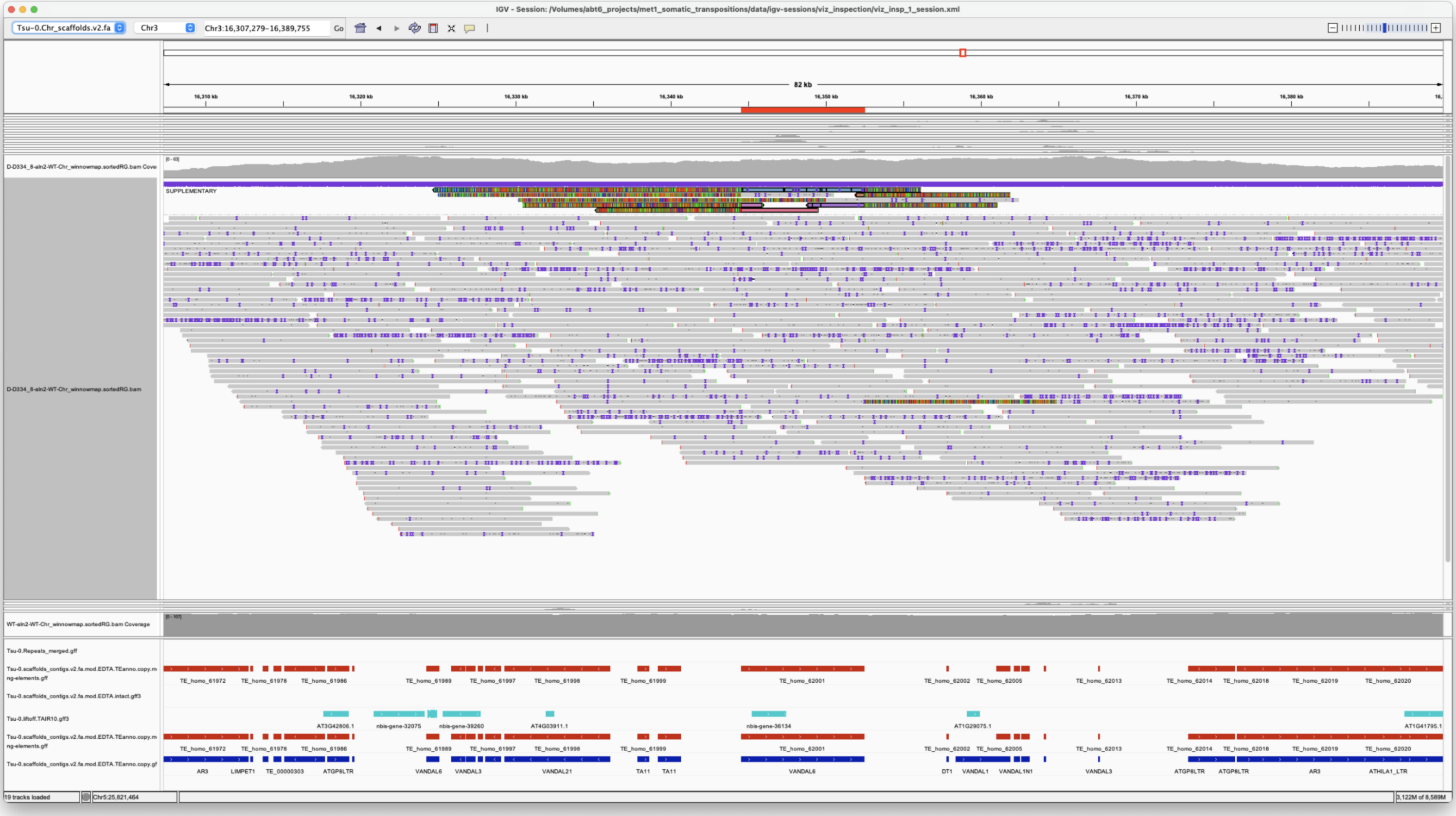

Partial

Confirmed

Chr5 27480891 27480891 - 1 Chr1;11941106;11946436;ATCOPIA93_Evade m64079_240212_113350/101845376/ccs met1_08

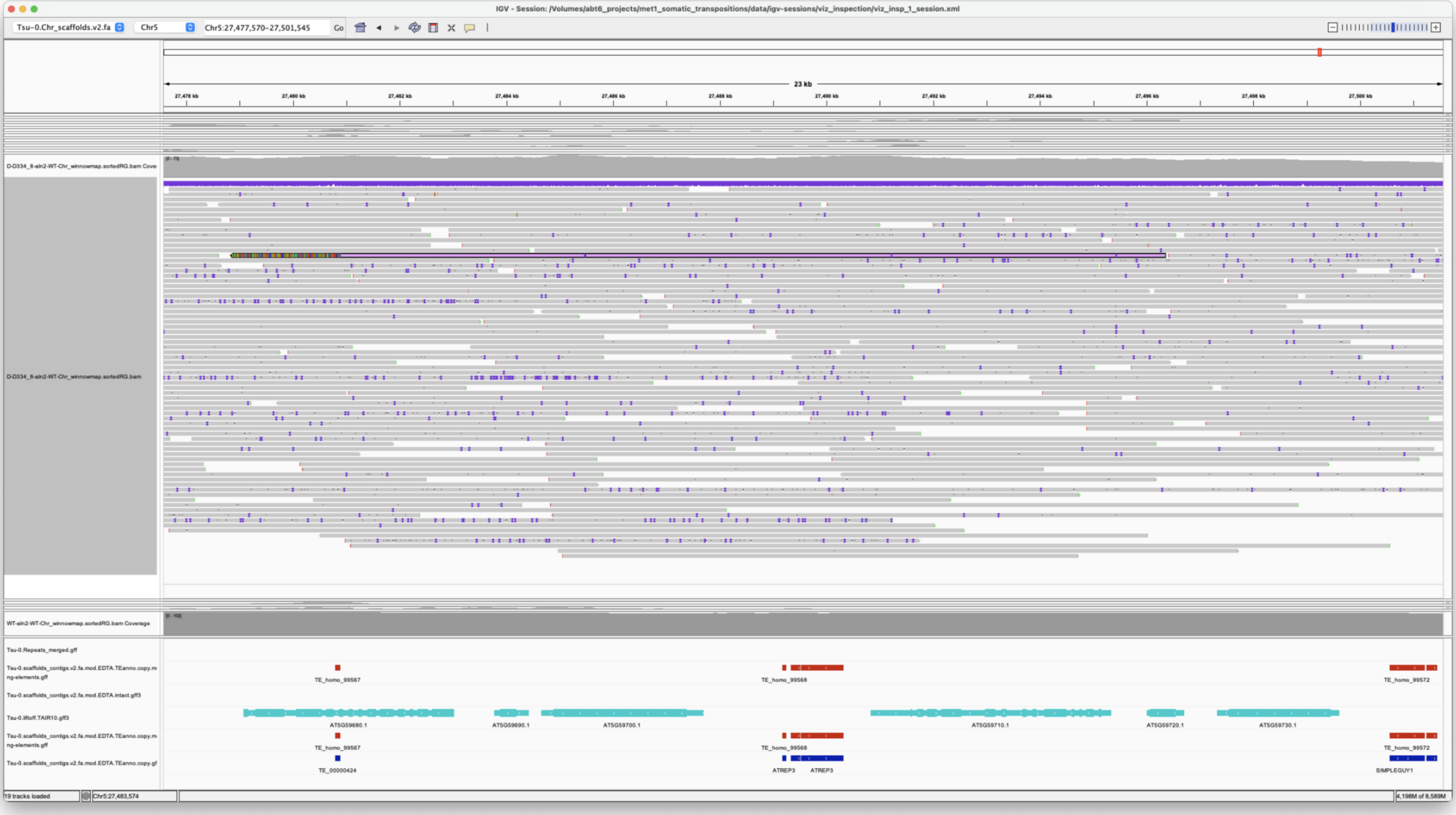

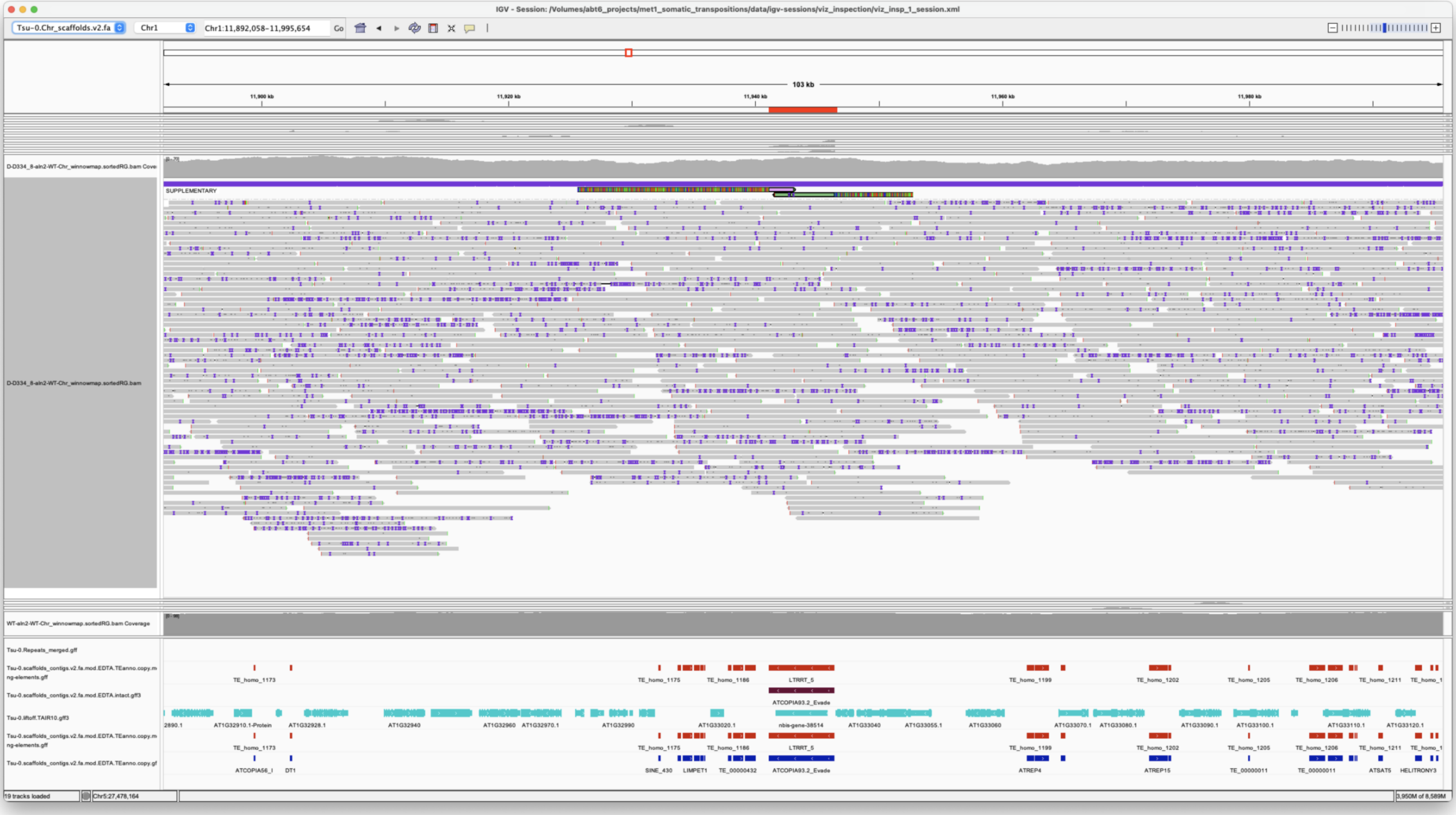

Partial

Confirmed

---

met1_09

Chr1 3725934 3725934 + 1 Chr3;16344522;16352497;VANDAL6 m64079_221220_112036/109446837/ccs met1_09

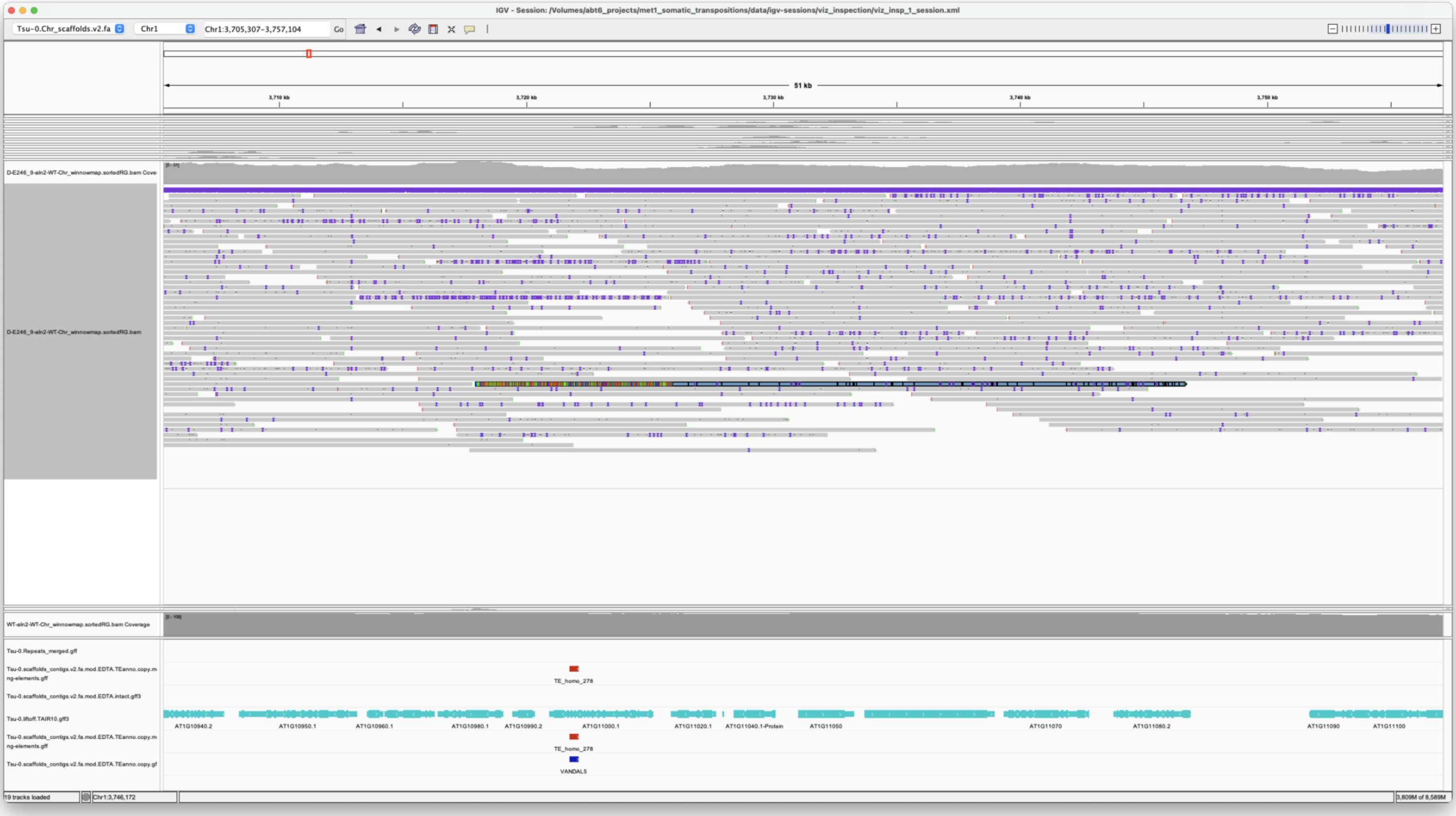

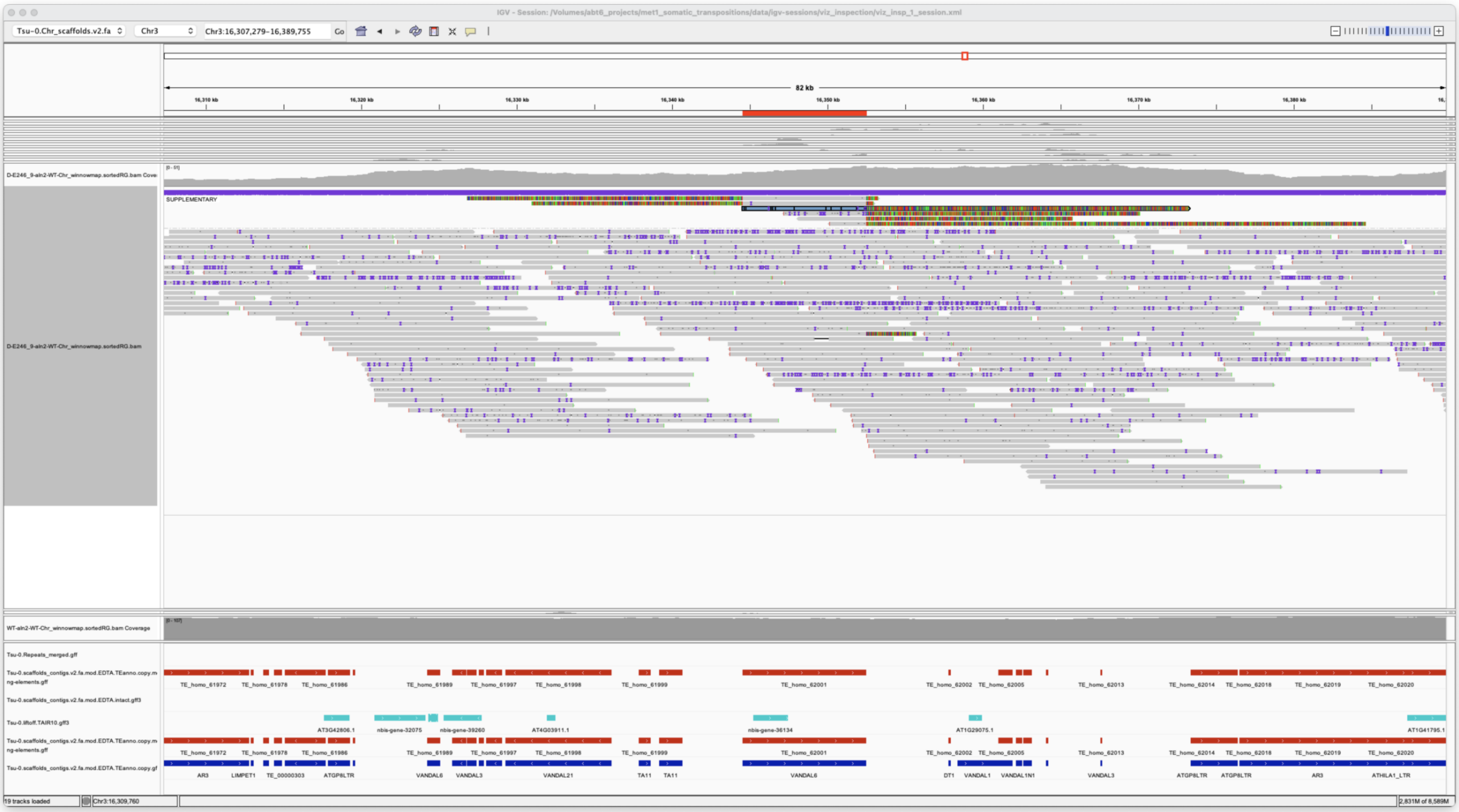

Partial

Confirmed

Chr1 10545157 10545157 + 1 Chr3;16344522;16352497;VANDAL6 m64079_221220_112036/175244627/ccs met1_09

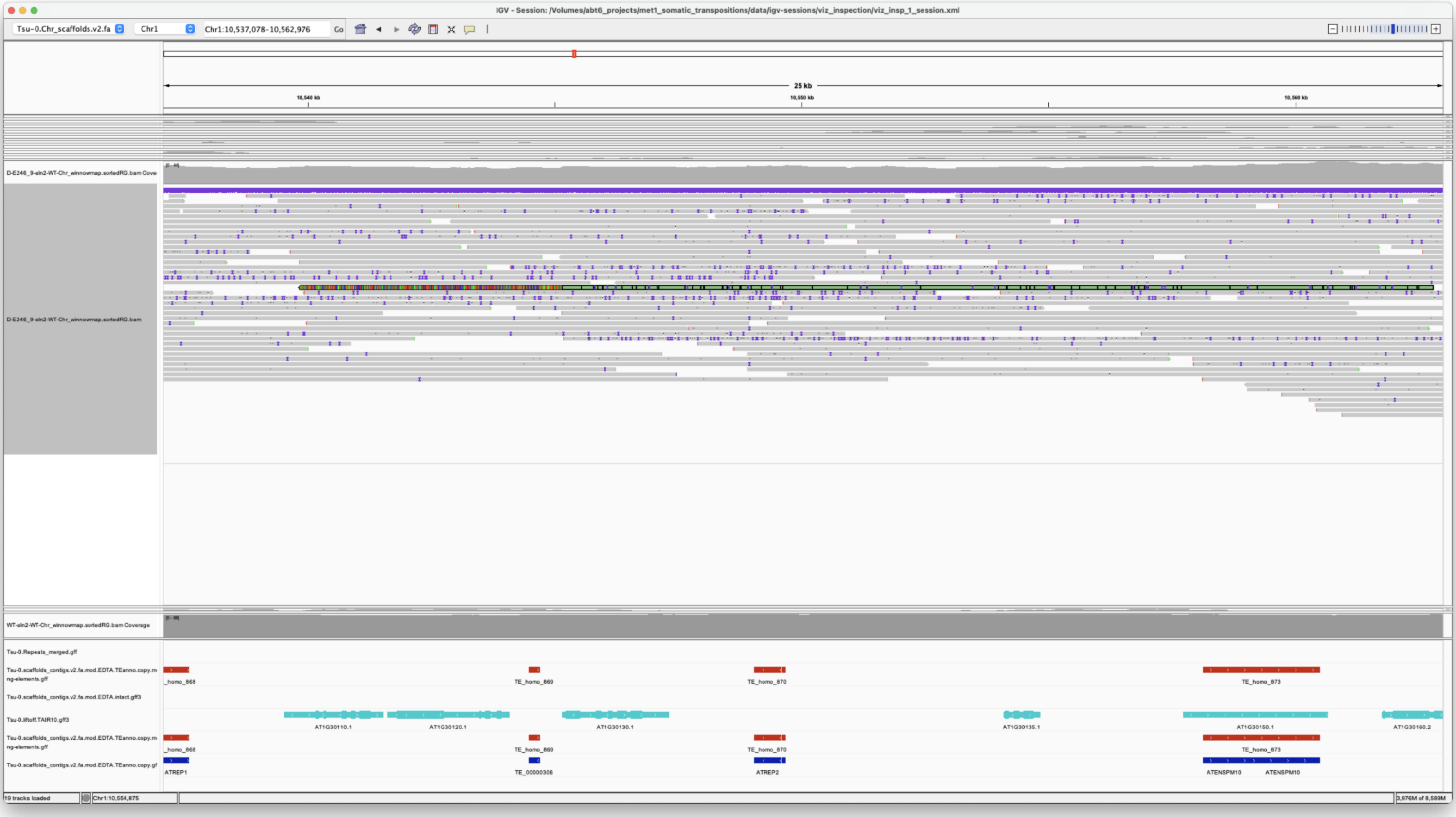

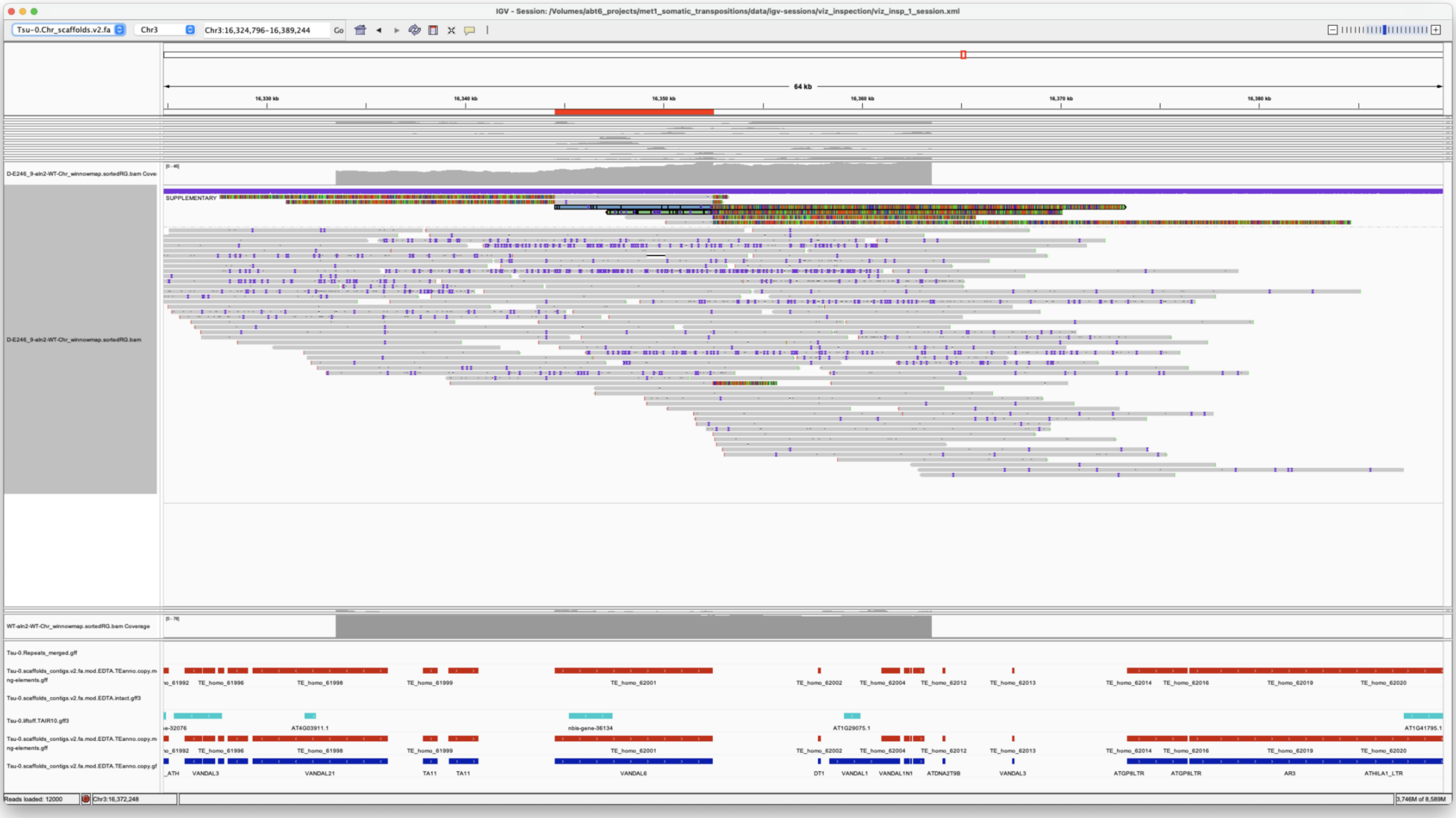

Partial

Confirmed

Chr1 21148623 21148623 + 1 Chr5;19152829;19160826;VANDAL21 m64079_221220_112036/172689386/ccs met1_09

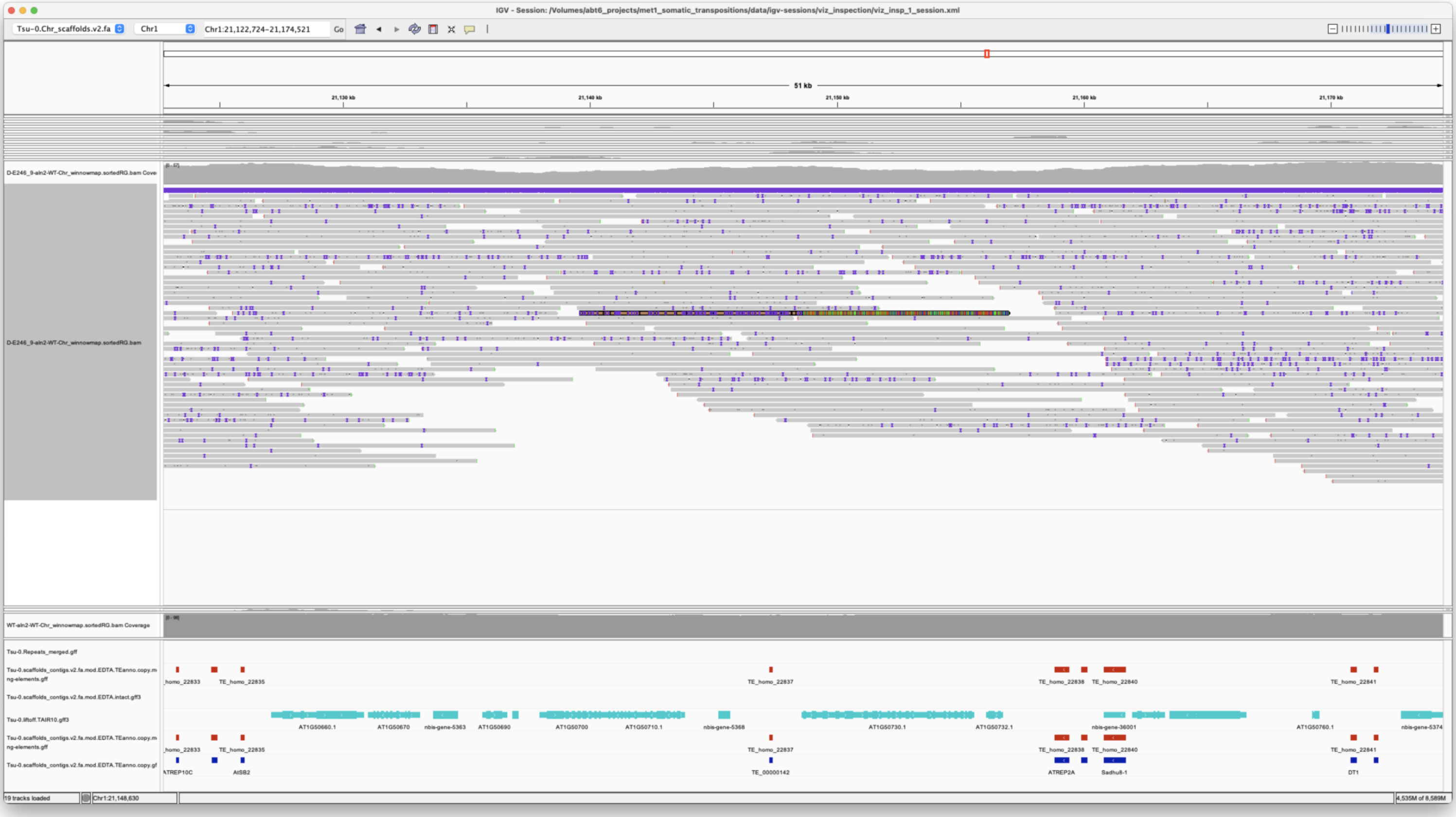

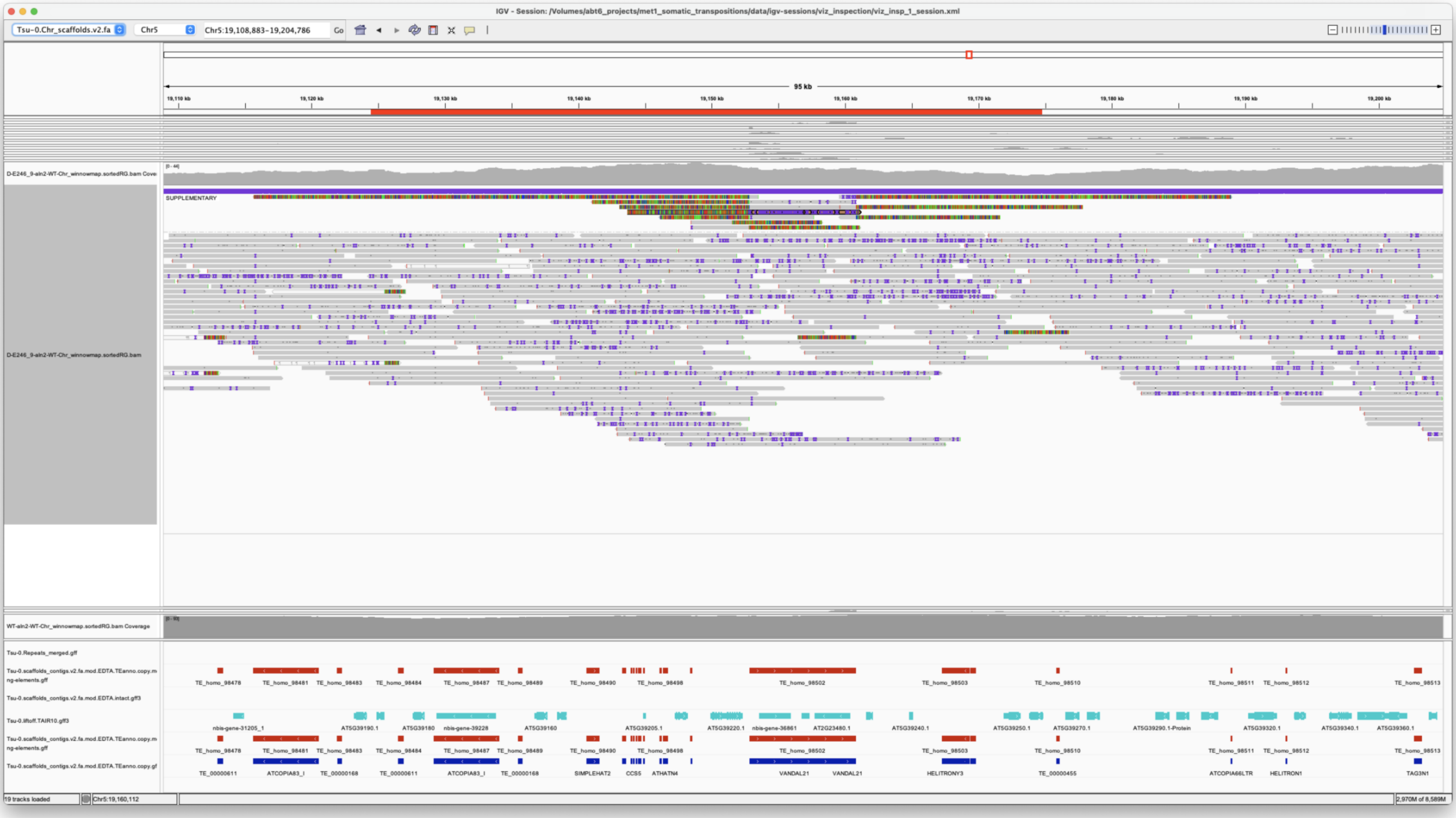

Partial

Confirmed

Chr1 29220751 29220751 + 1 Chr3;16344522;16352497;VANDAL6 m64079_221220_112036/106039110/ccs met1_09

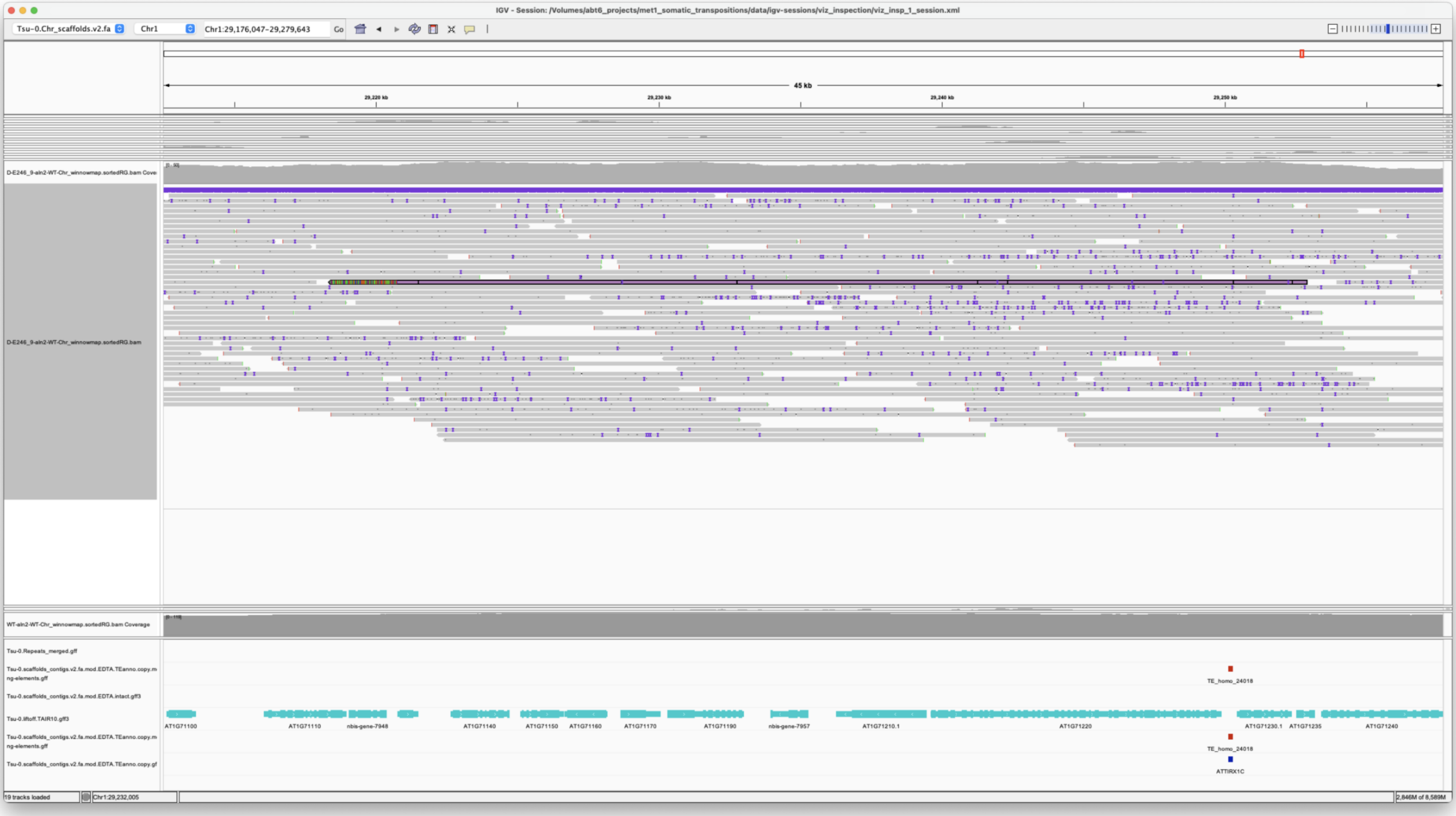

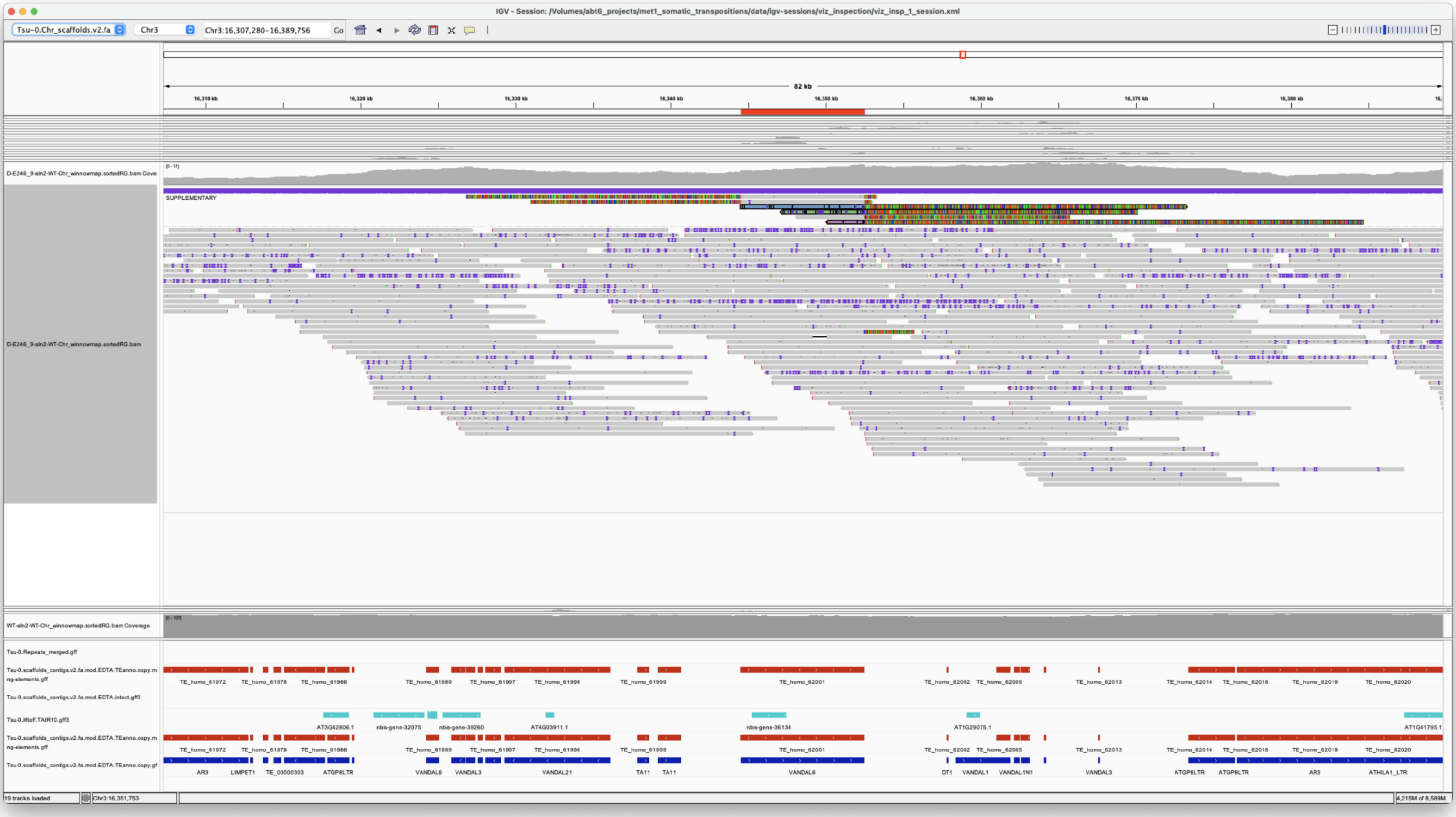

Partial

Confirmed

Chr1 32259560 32259560 - 1 Chr5;19152829;19160826;VANDAL21 m64079_240212_113350/121832980/ccs met1_09

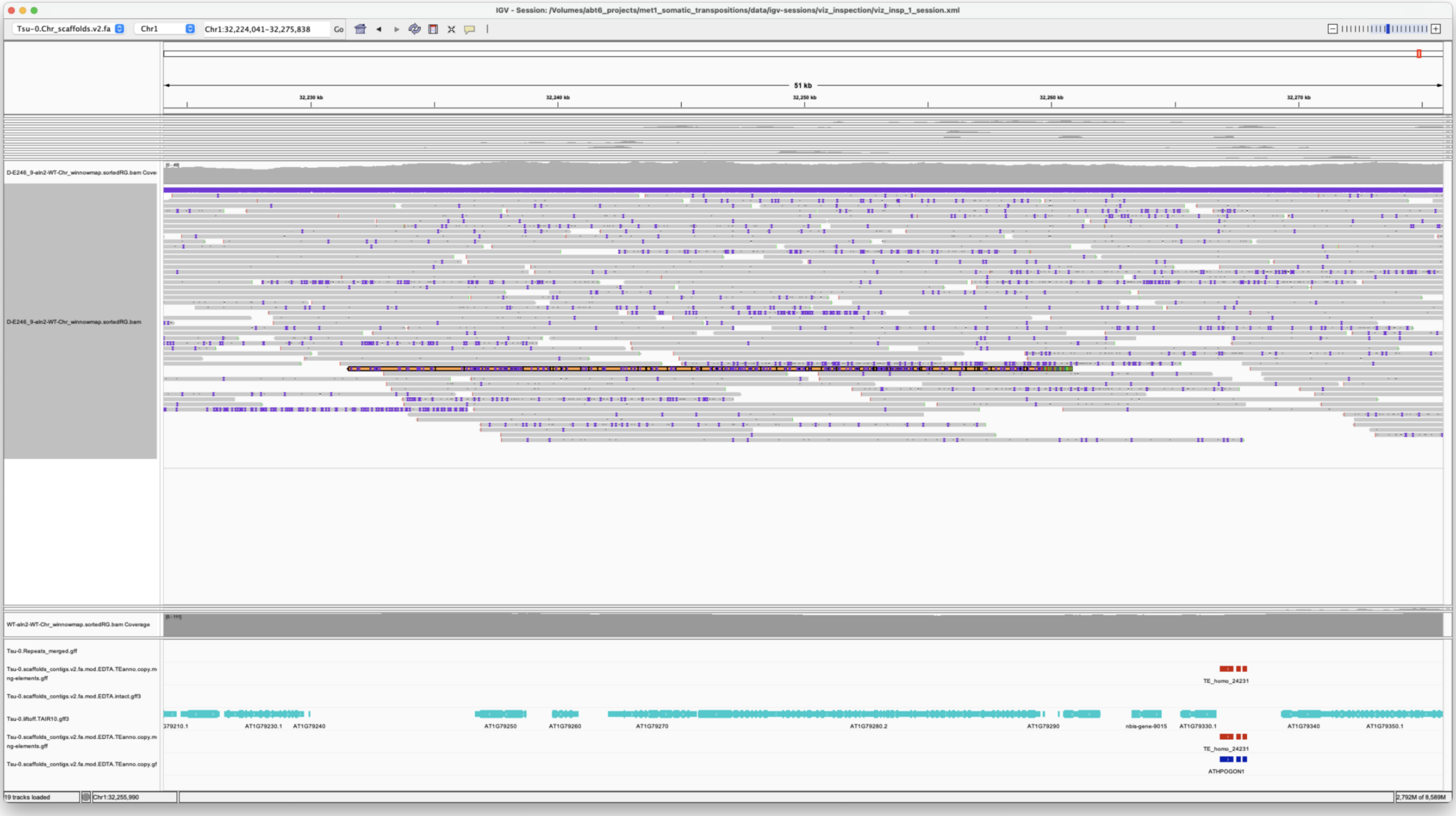

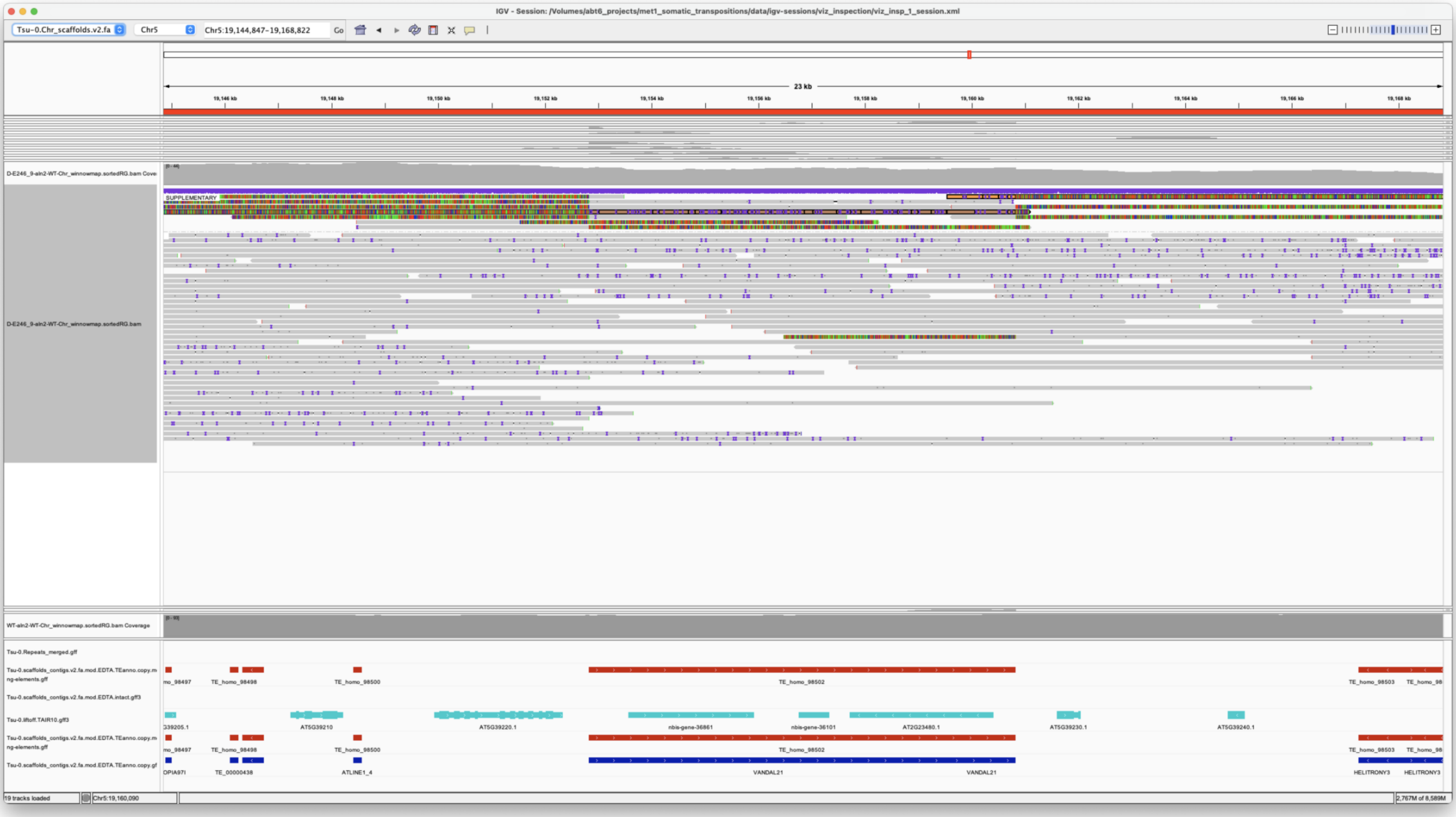

Partial

Confirmed

Chr2 10820496 10820496 + 1 Chr5;19152829;19160826;VANDAL21 m64079_221220_112036/58329751/ccs met1_09

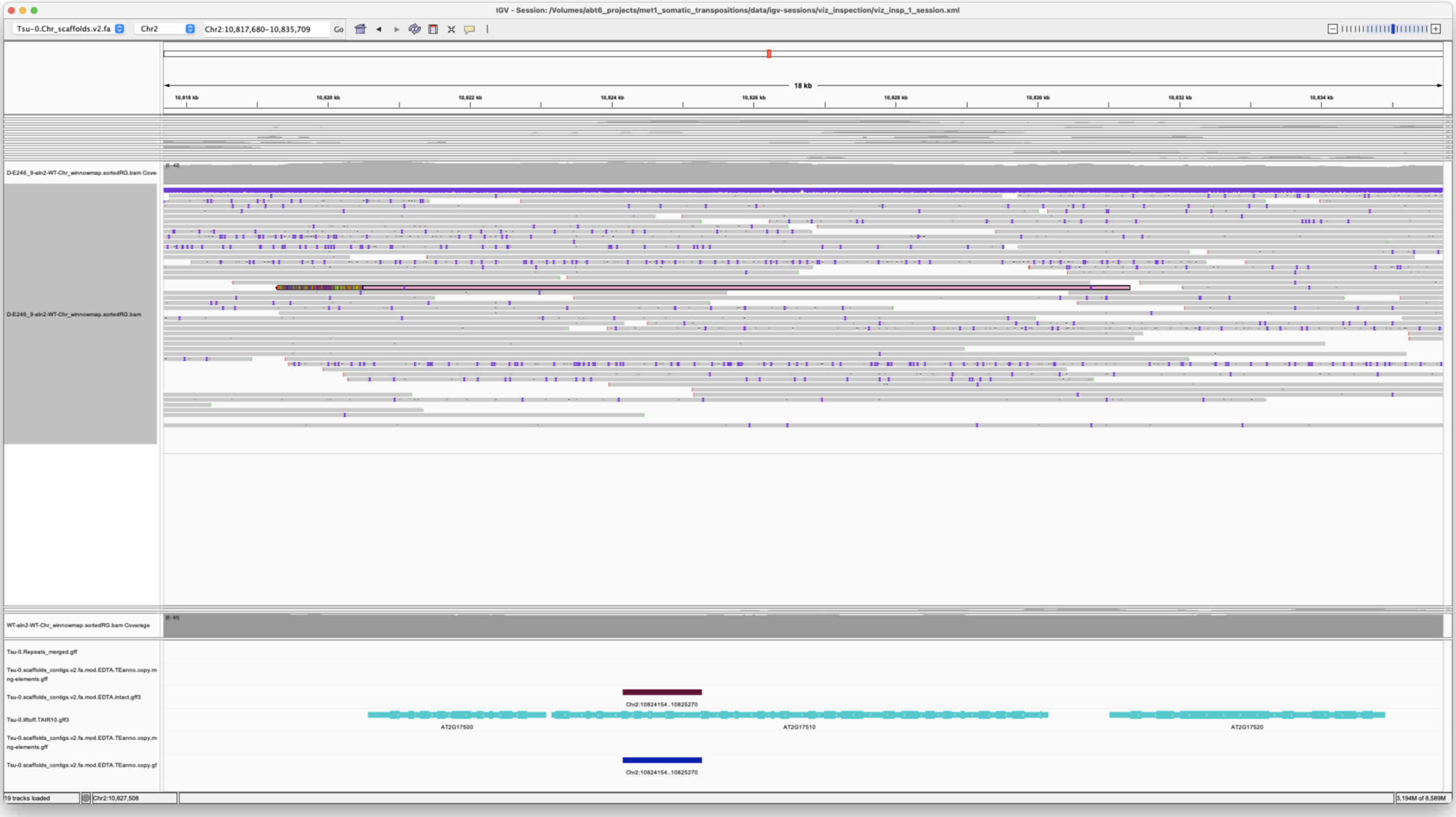

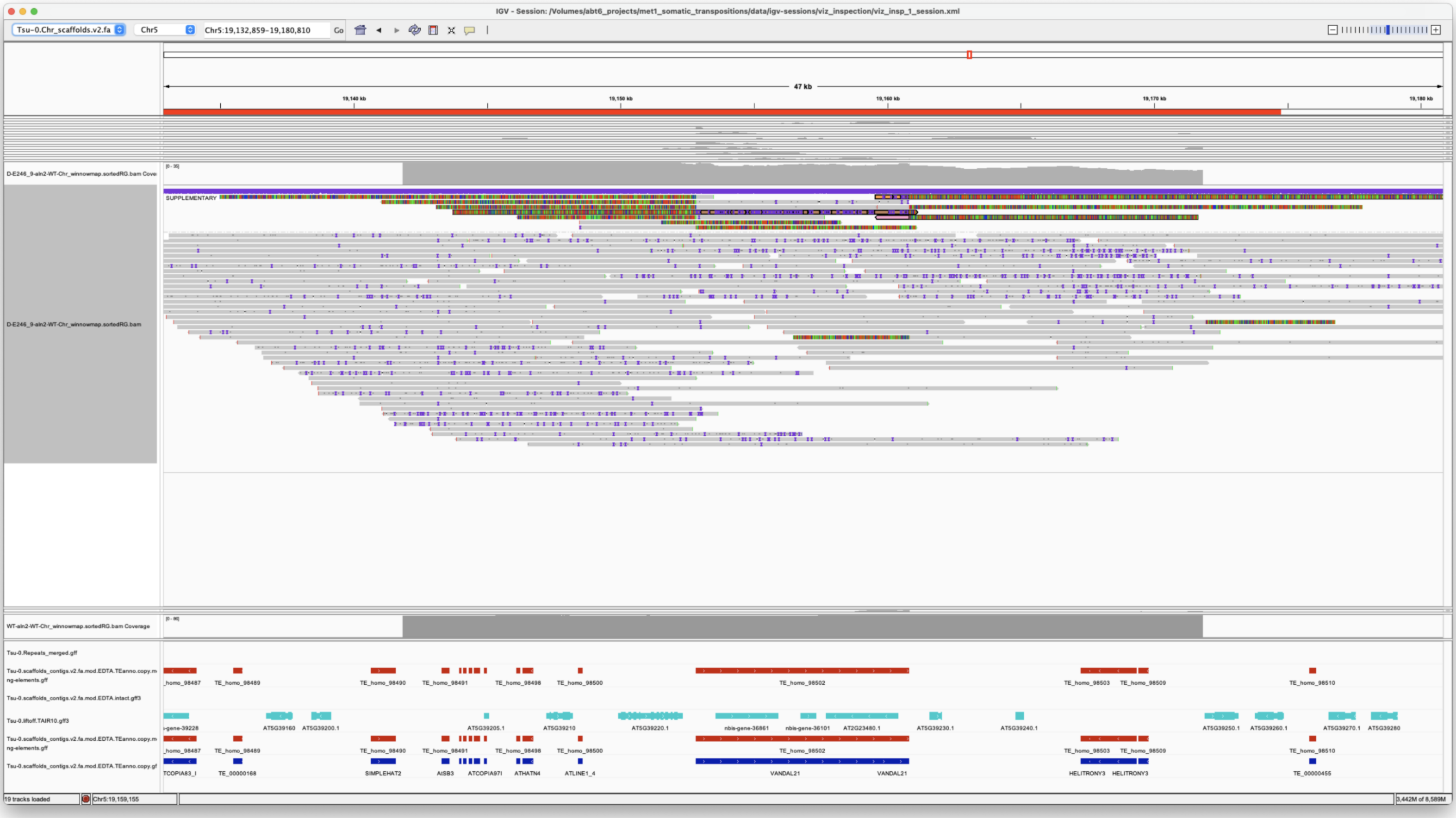

Partial

Confirmed

Chr3 5890020 5890020 + 1 Chr3;16344522;16352497;VANDAL6 m64079_221220_112036/111280480/ccs met1_09

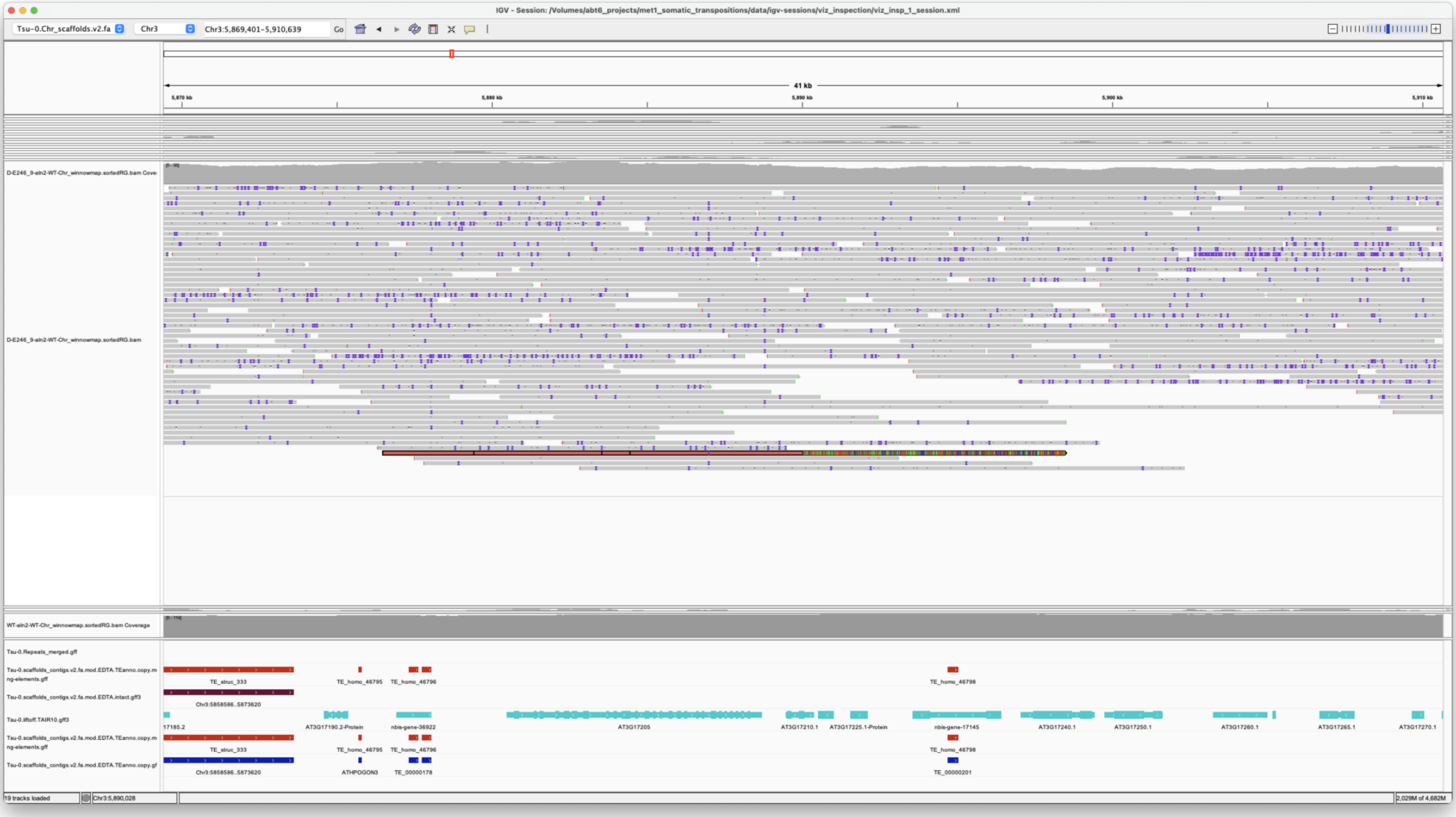

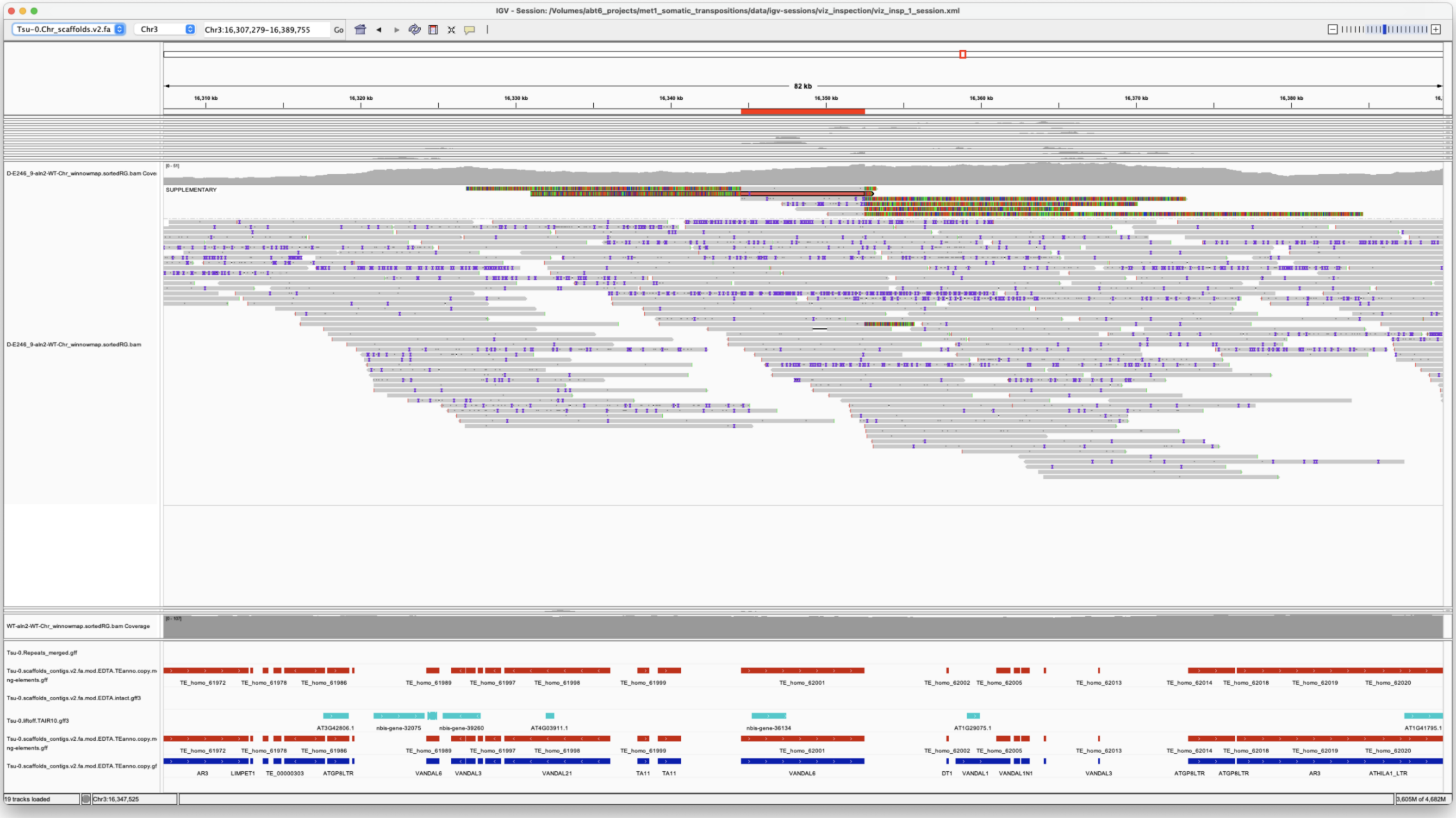

Partial

Confirmed

> Chr3 13397987 13397987 + 1 Chr5;19152829;19160826;VANDAL21 m64079_221220_112036/31457762/ccs met1_09

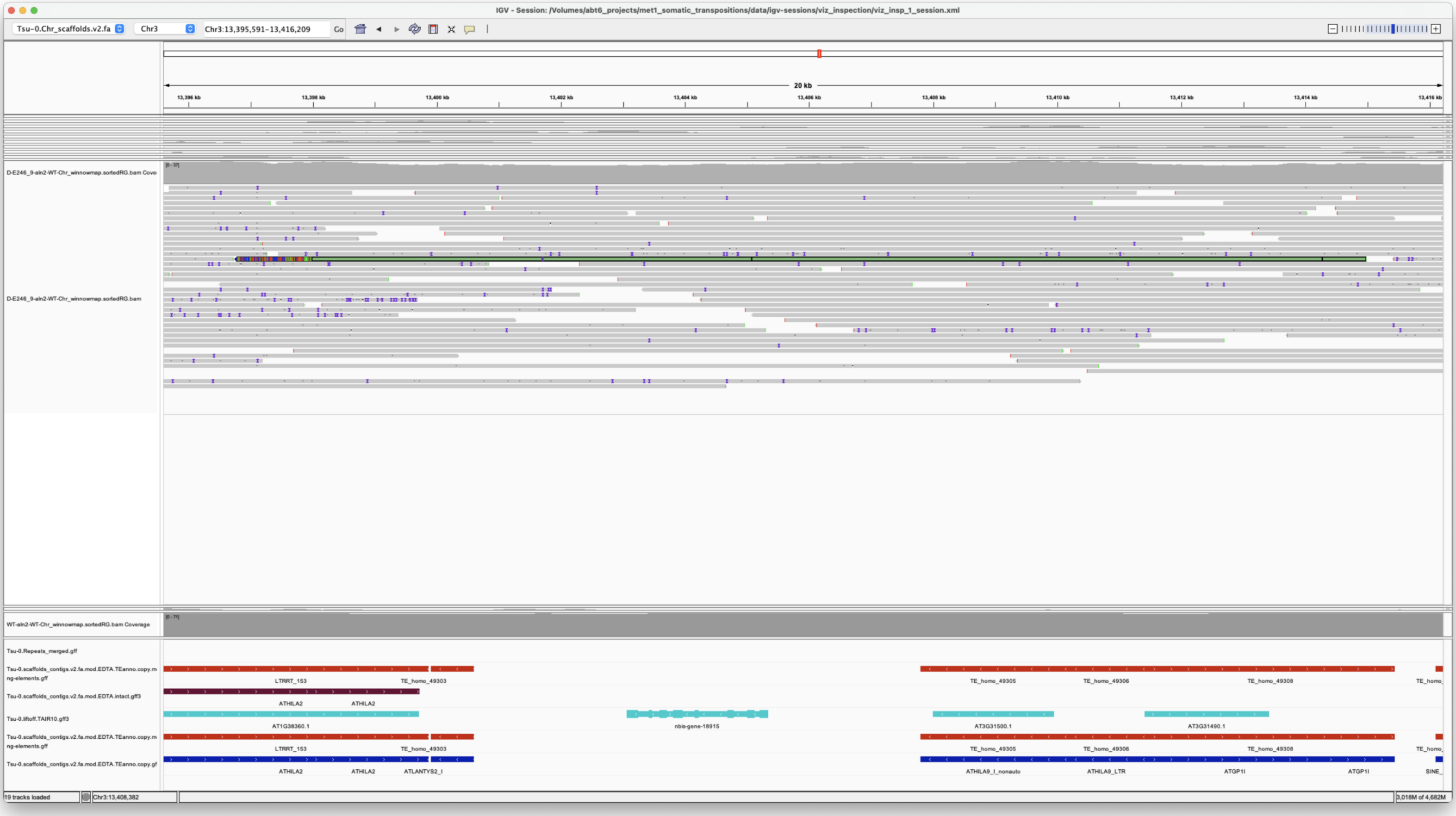

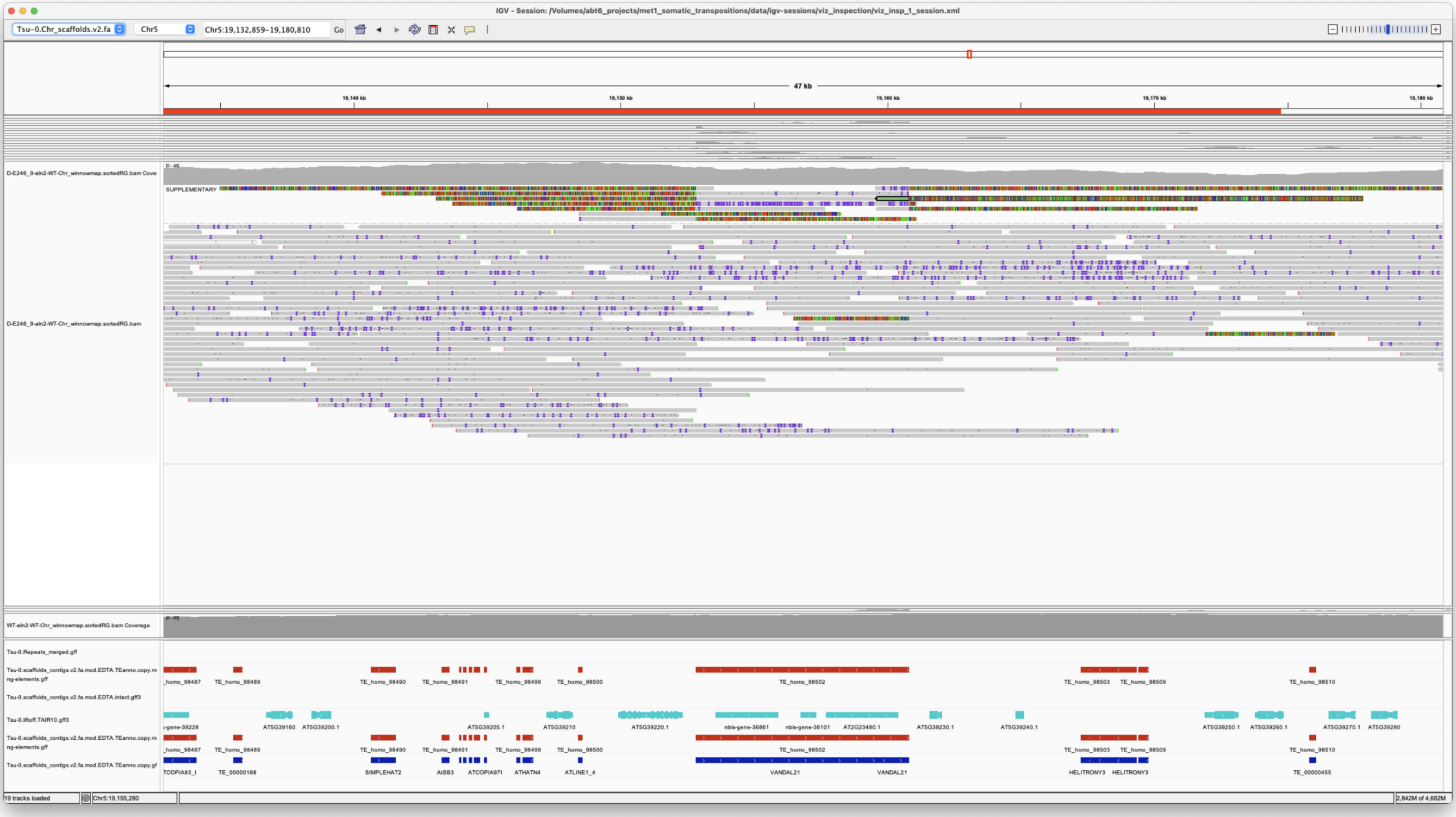

Partial

Confirmed

Chr3 20266688 20266688 + 1 Chr5;19152829;19160826;VANDAL21 m64079_221220_112036/116129982/ccs met1_09

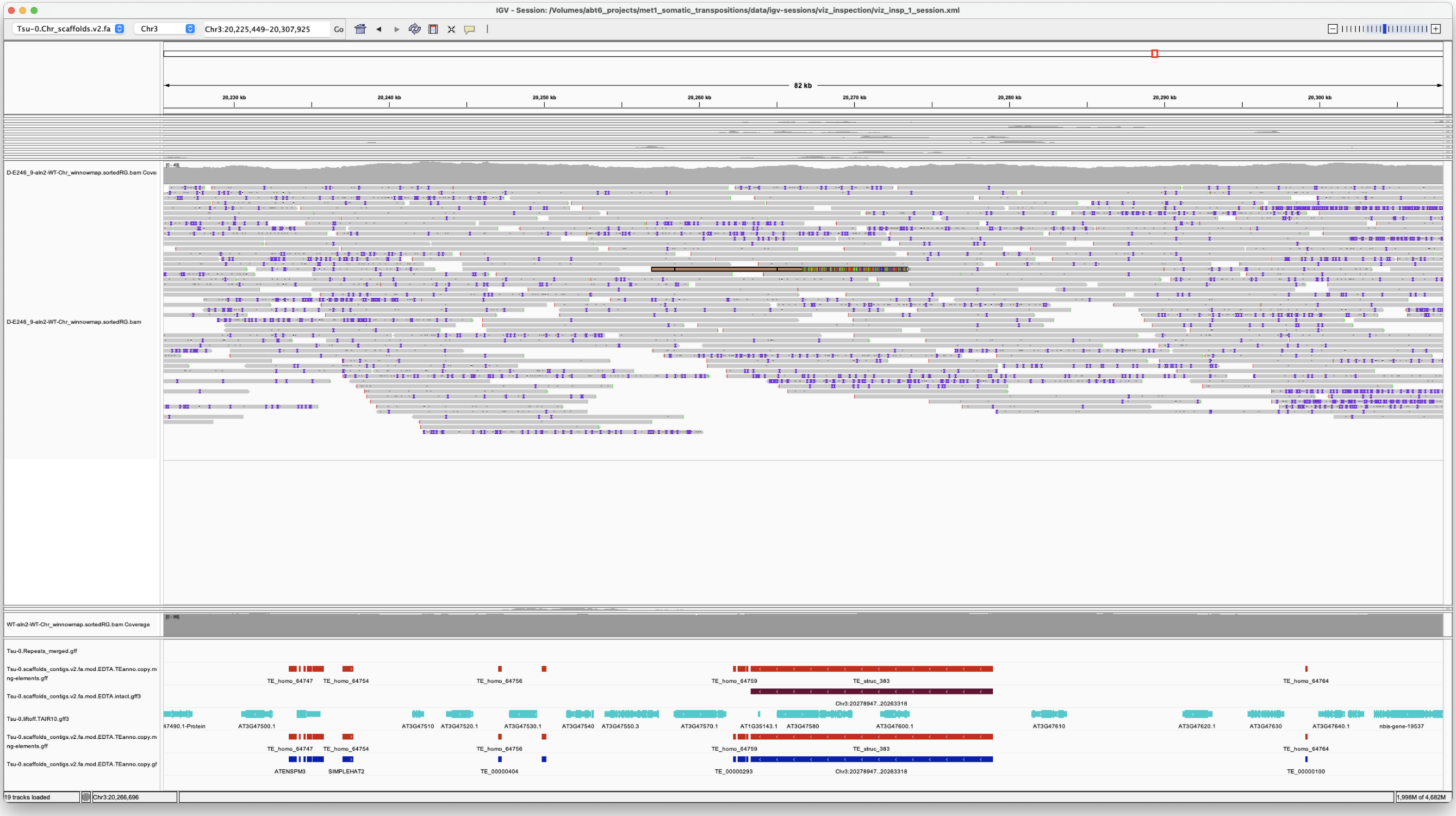

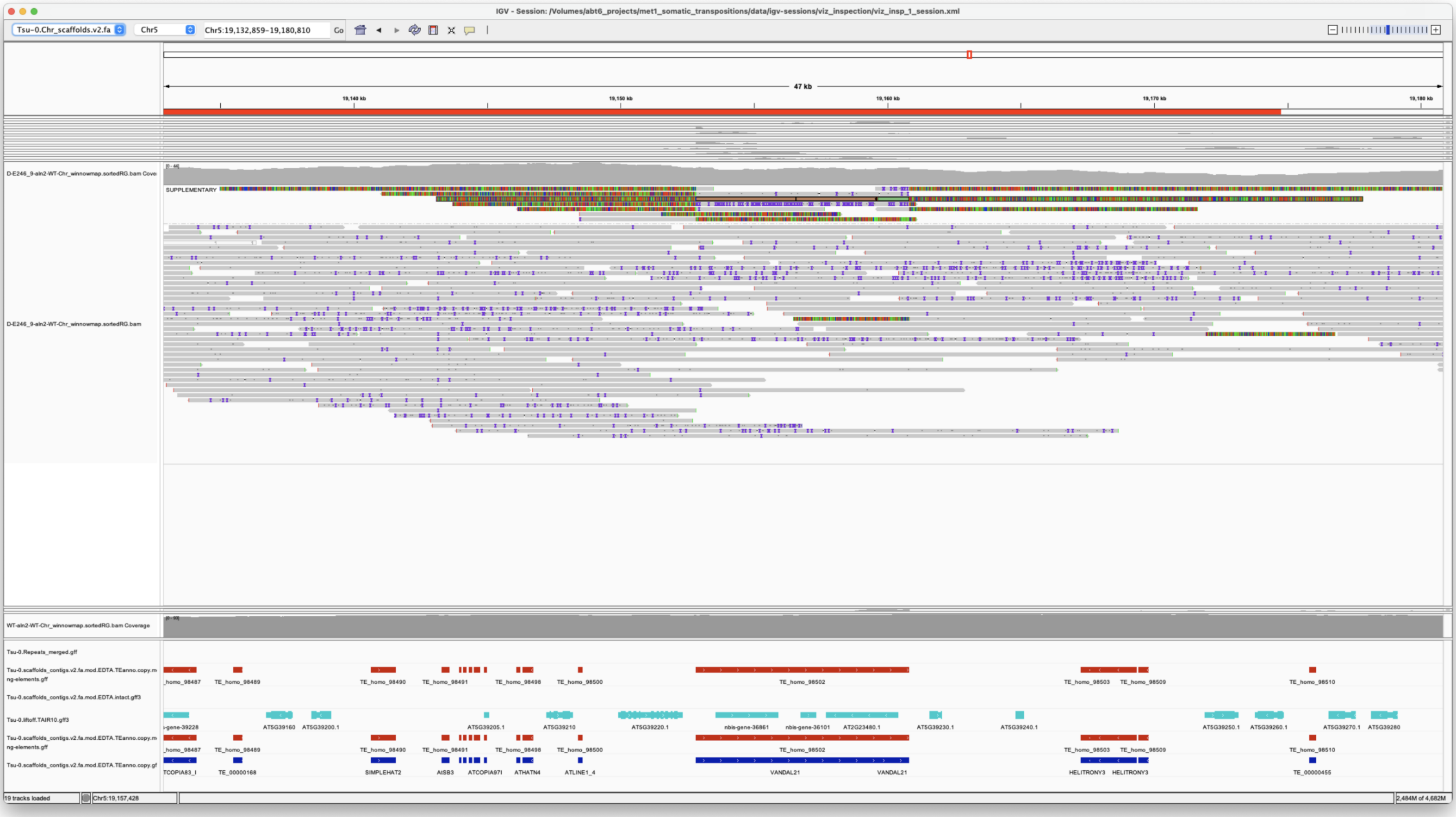

Partial

Confirmed

> Chr4 15434774 15434776 +,- 2 Chr5;8256939;8265133;CAC2A,Chr5;8256939;8265133;CAC2A m64079_221220_112036/41485182/ccs,m64079_240212_113350/99025652/ccs met1_09

> Chr4 15434778 15434778 - 1 Chr5;8256939;8265133;CAC2A m64079_221220_112036/23725887/ccs met1_09

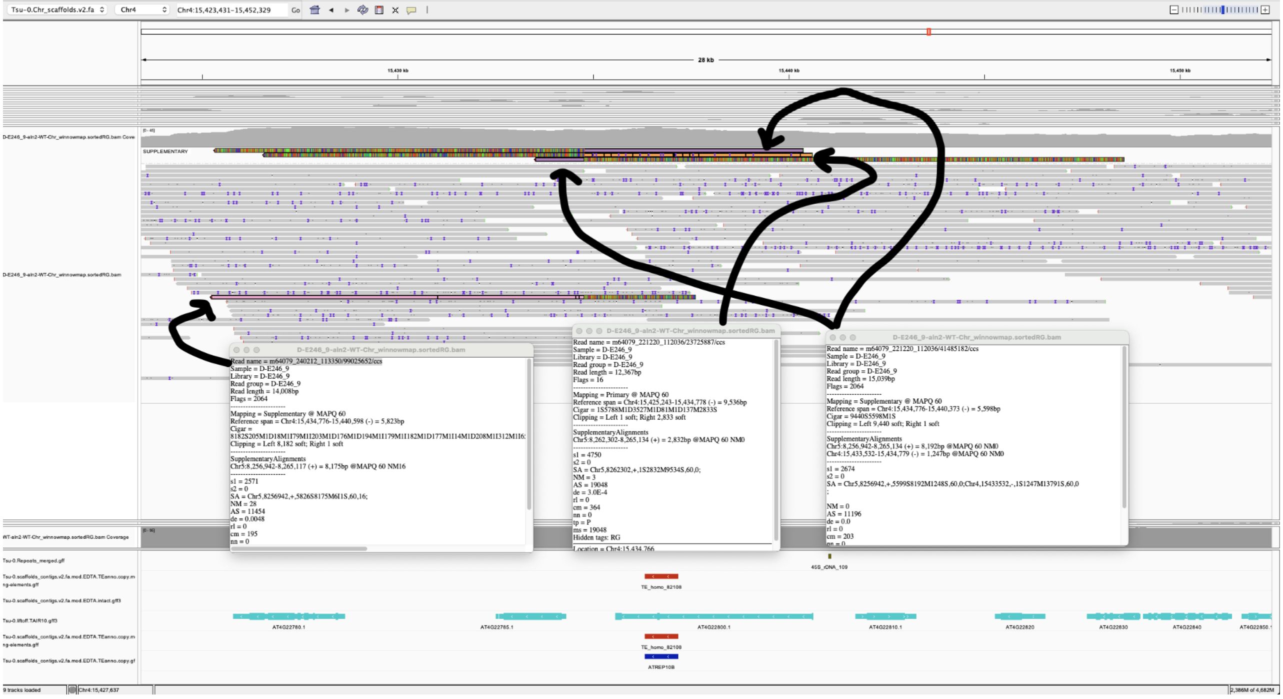

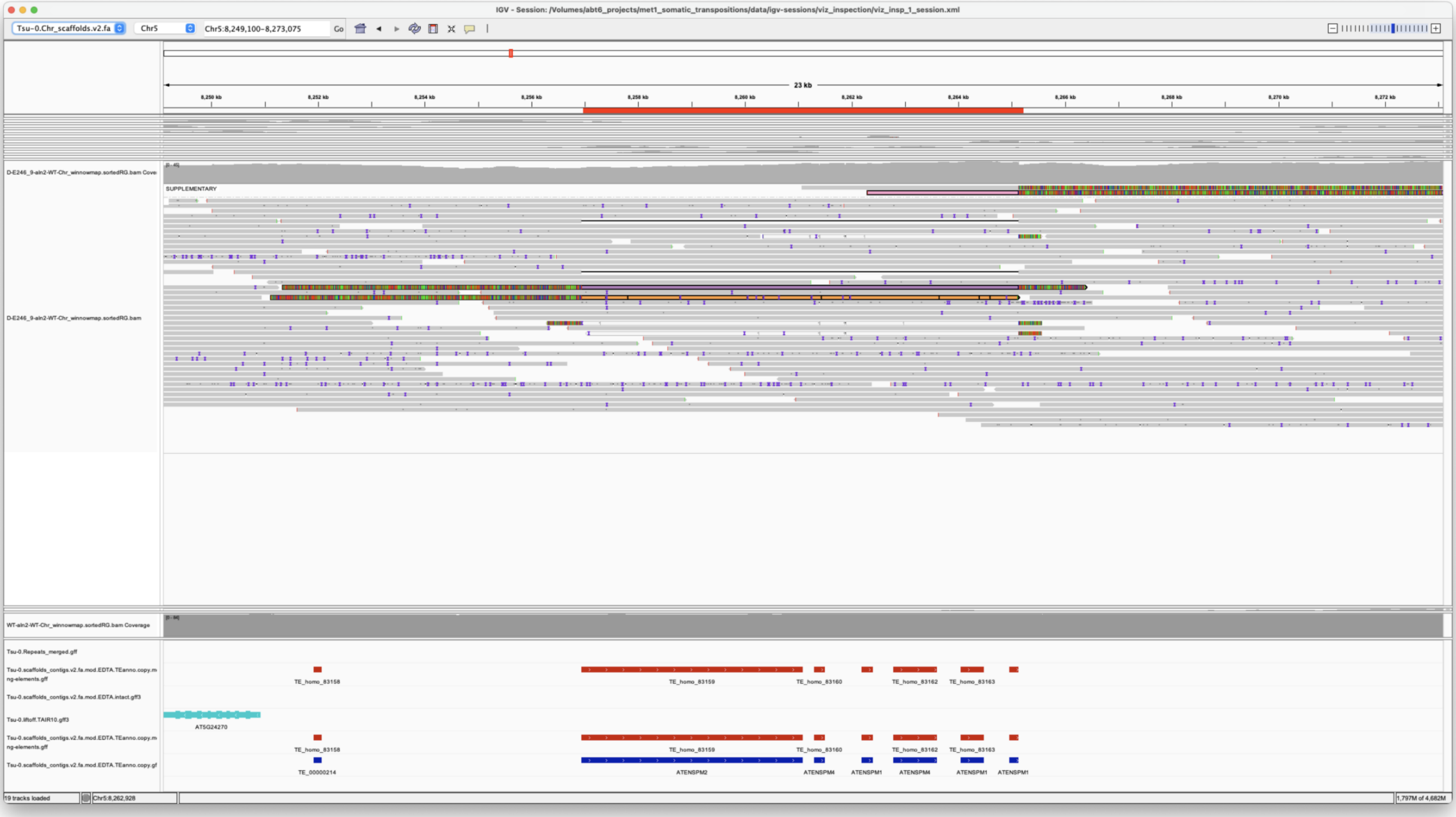

(-) insertion

Corrected, merged

Mixed

Confirmed

Chr4 18769338 18769338 + 1 Chr3;16344522;16352497;VANDAL6 m64079_221220_112036/45547713/ccs met1_09

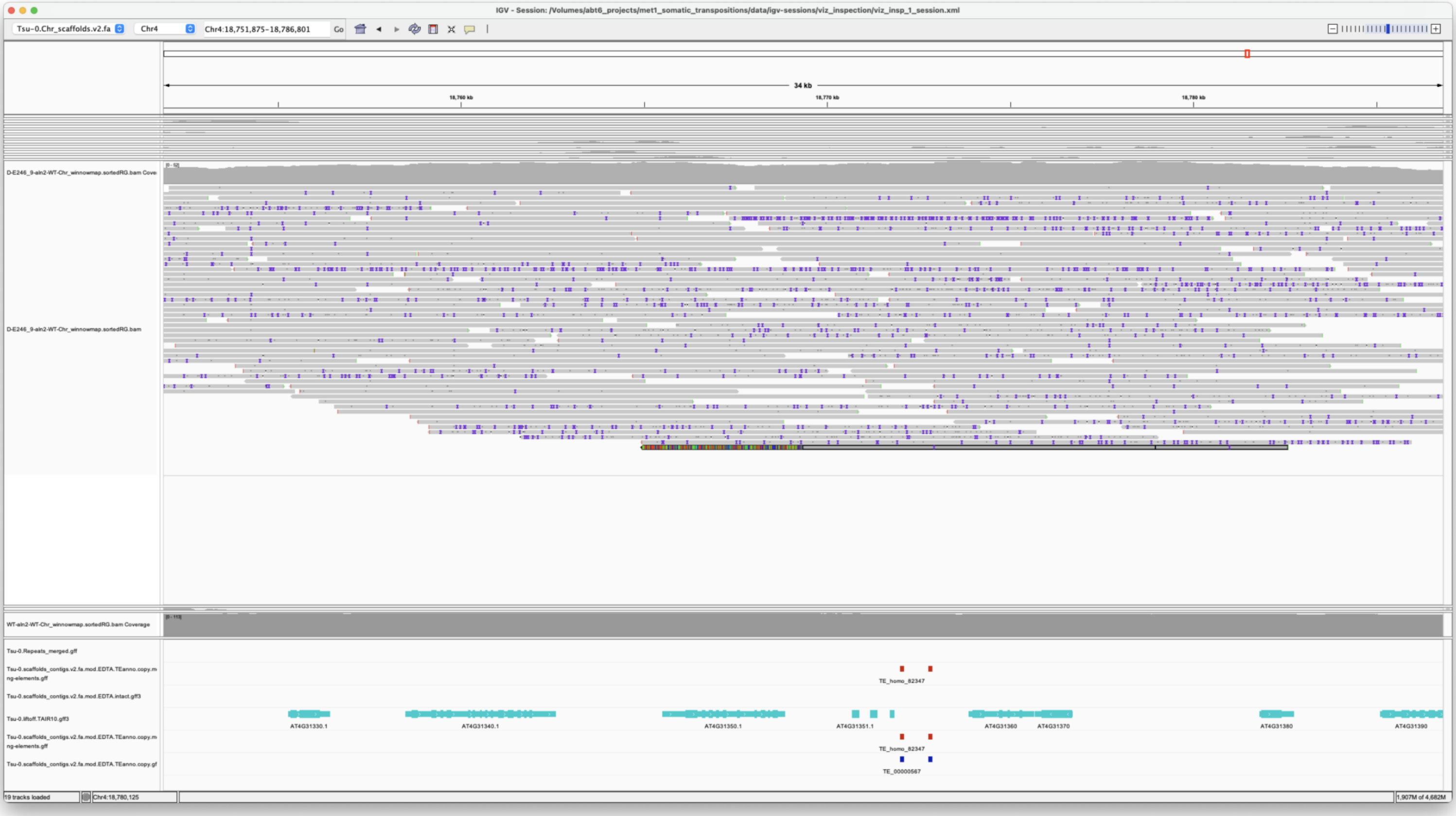

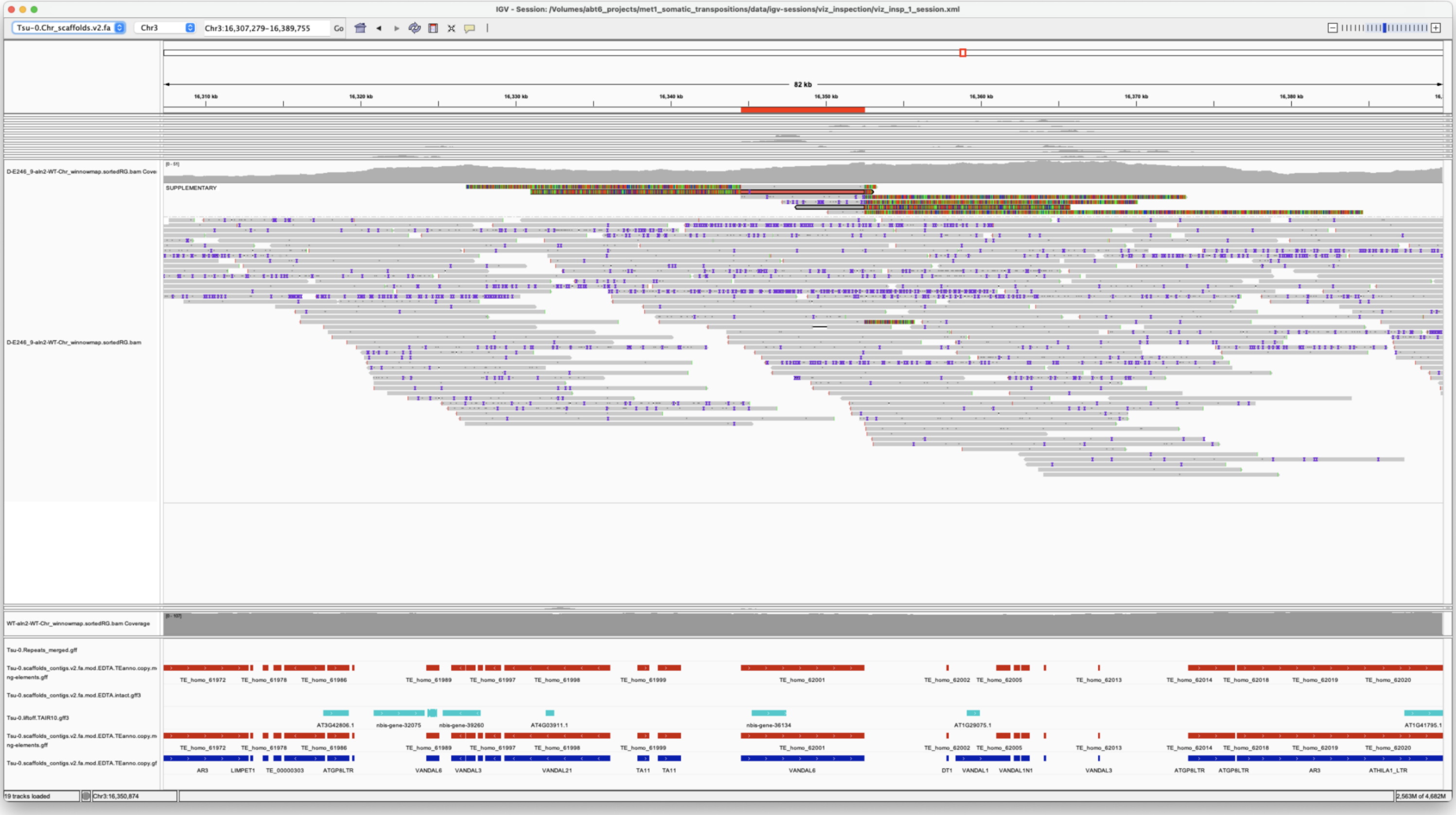

Partial

Confirmed

Chr4 19088936 19088936 + 1 Chr5;19152829;19160826;VANDAL21 m64079_240212_113350/175768560/ccs met1_09

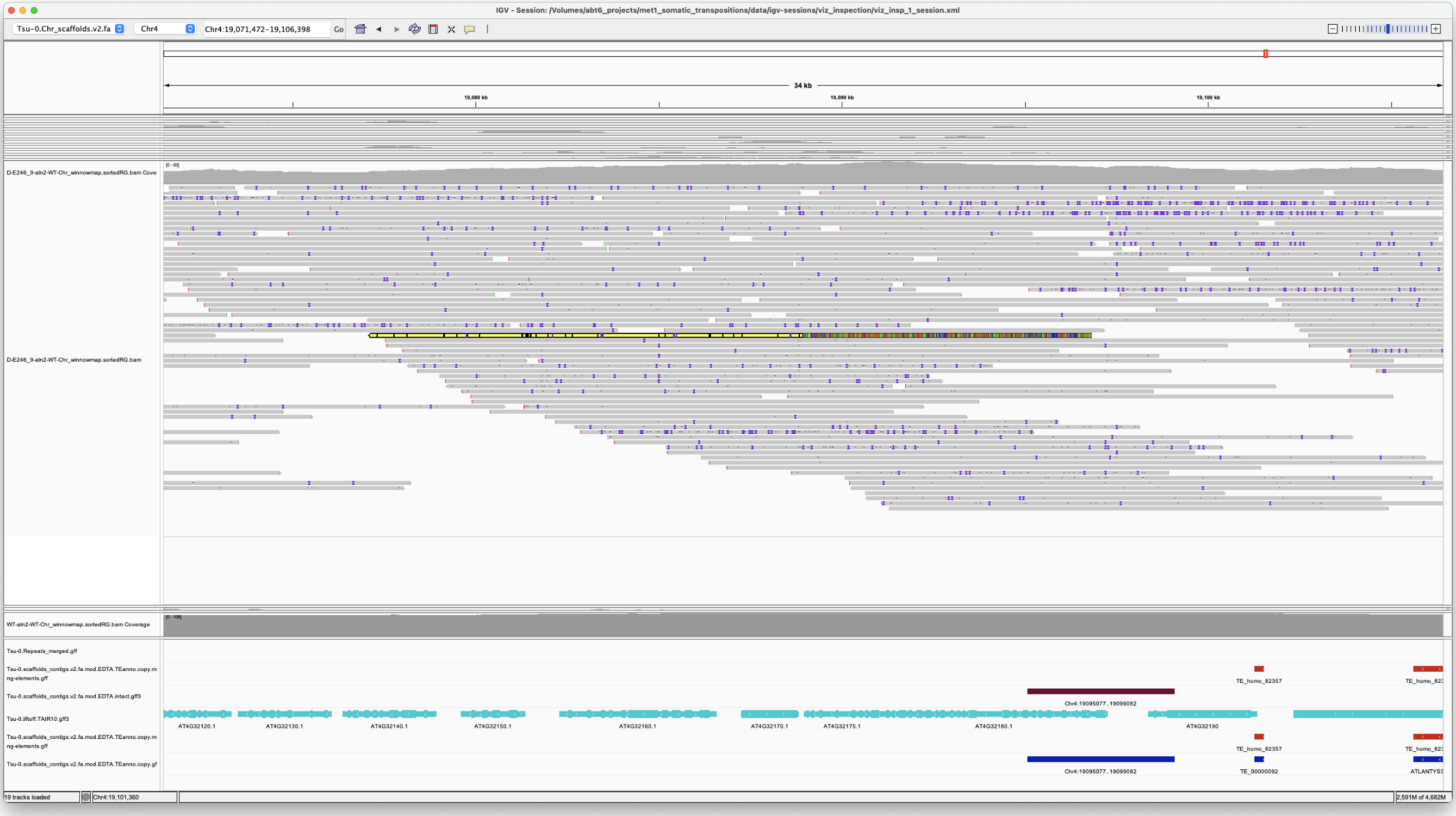

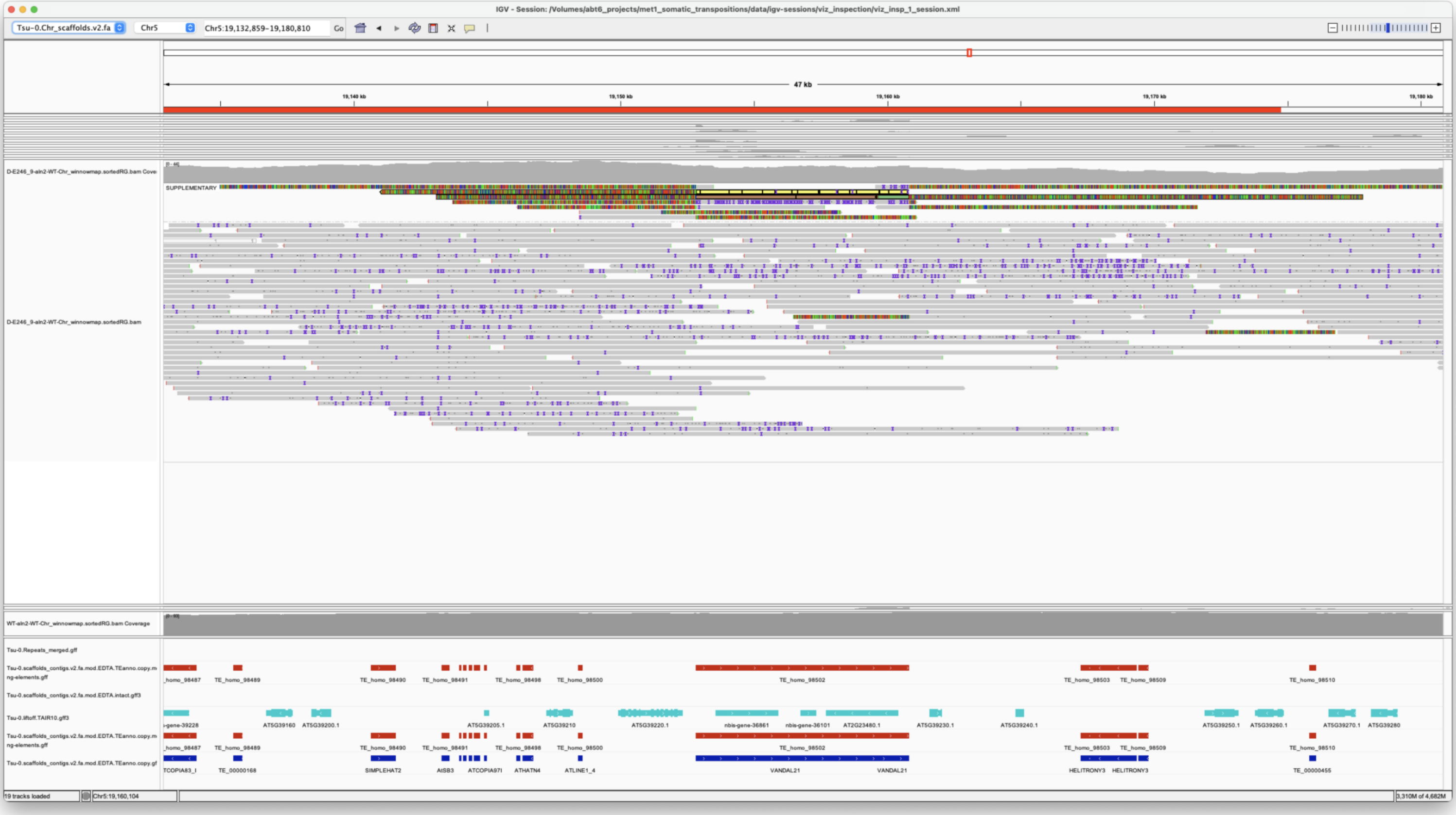

Partial

Confirmed

Chr5 6475054 6475054 + 1 Chr3;16344522;16352497;VANDAL6 m64079_221220_112036/60819523/ccs met1_09

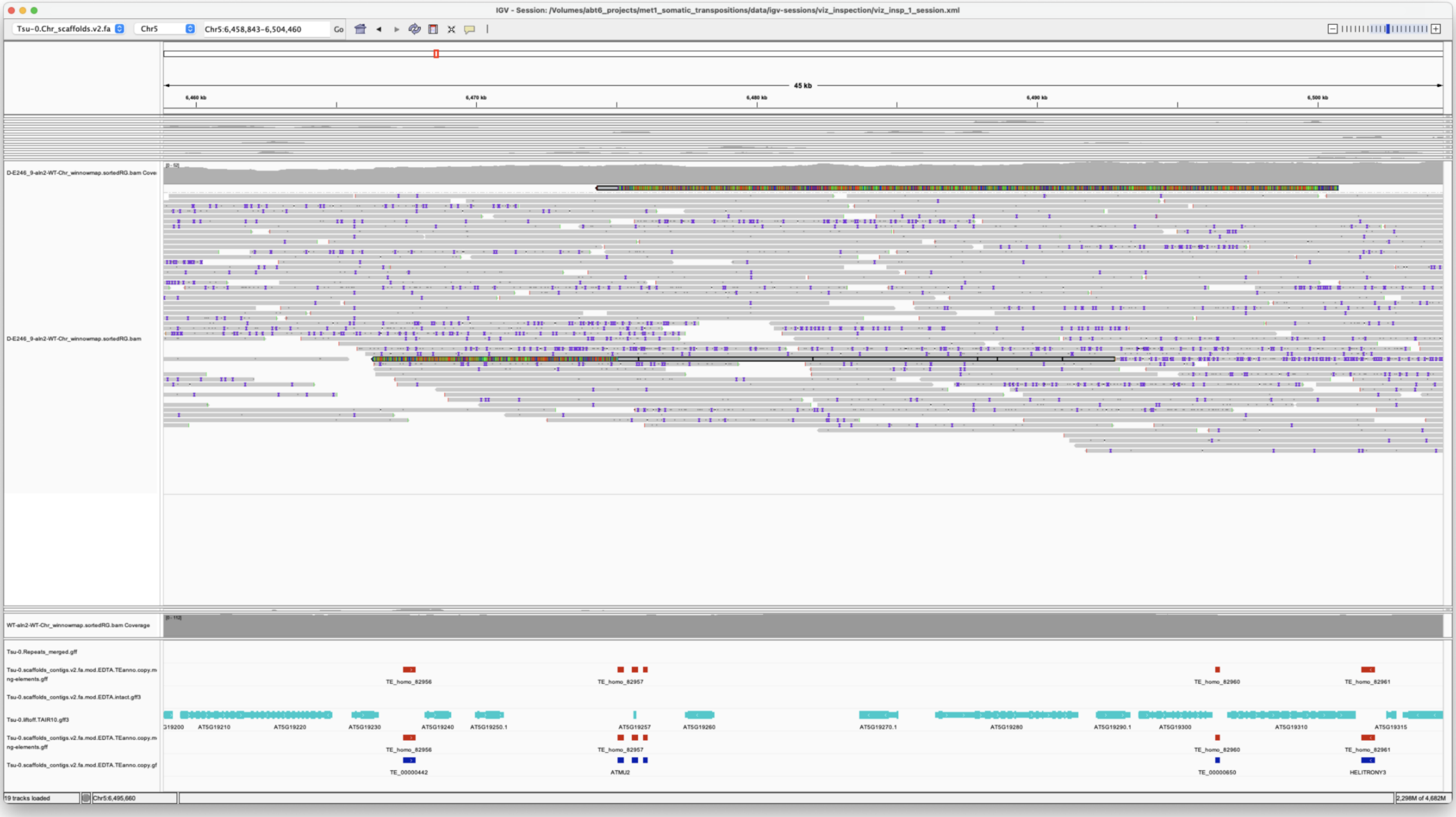

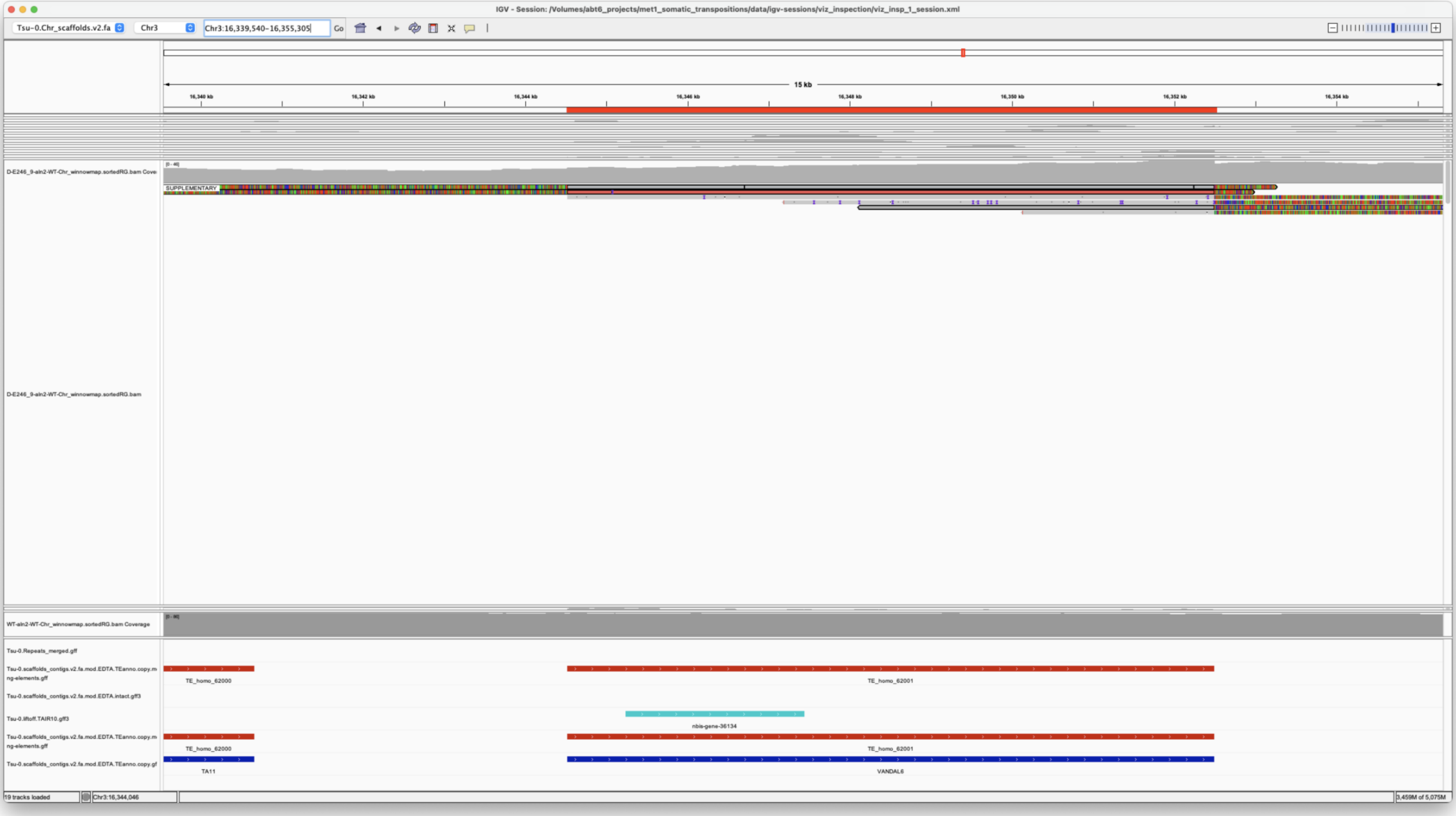

Central
TSD

Confirmed

Chr5 8096312 8096312 + 1 Chr5;8256939;8265133;CAC2A m64079_221220_112036/151978242/ccs met1_09

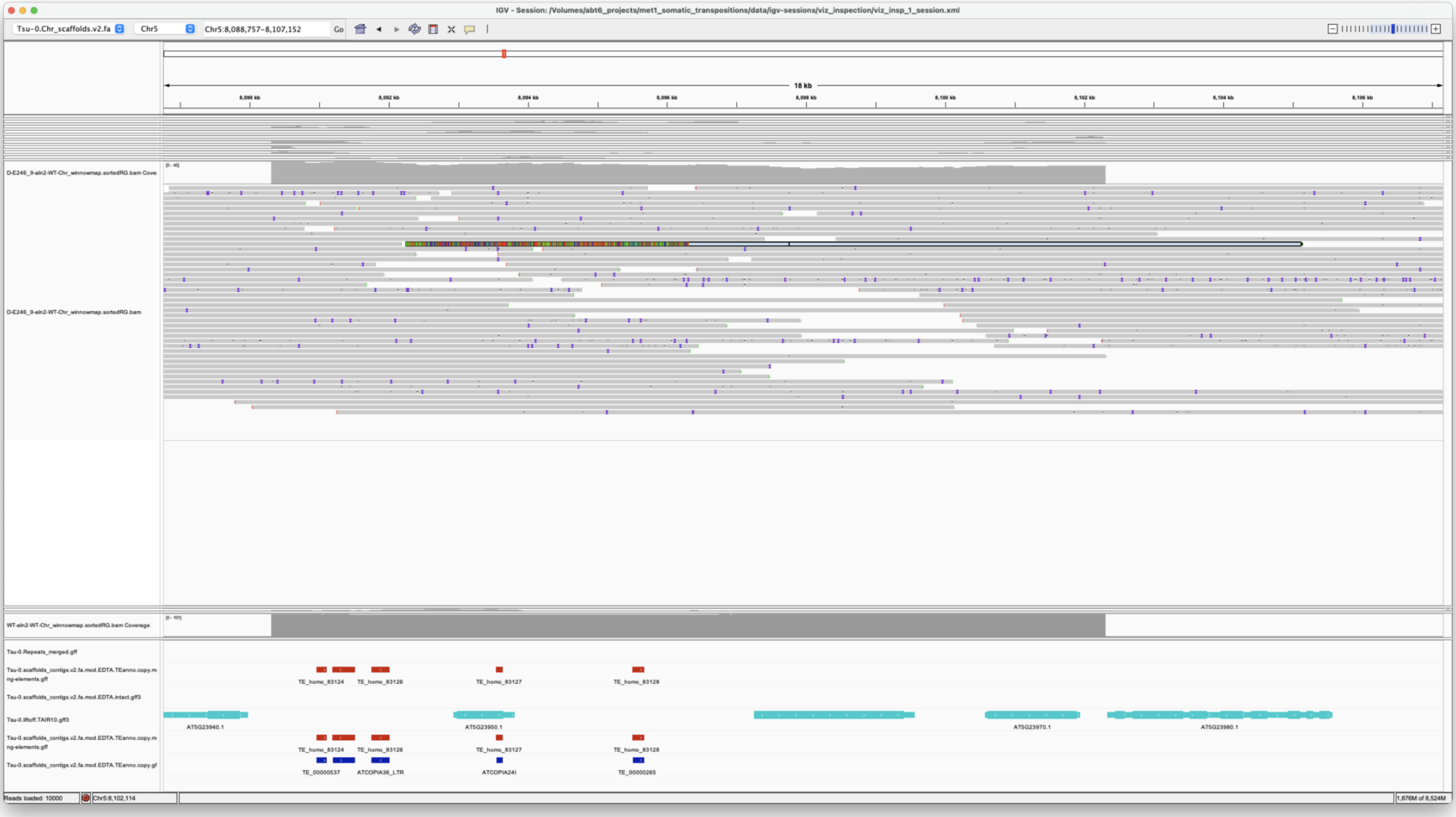

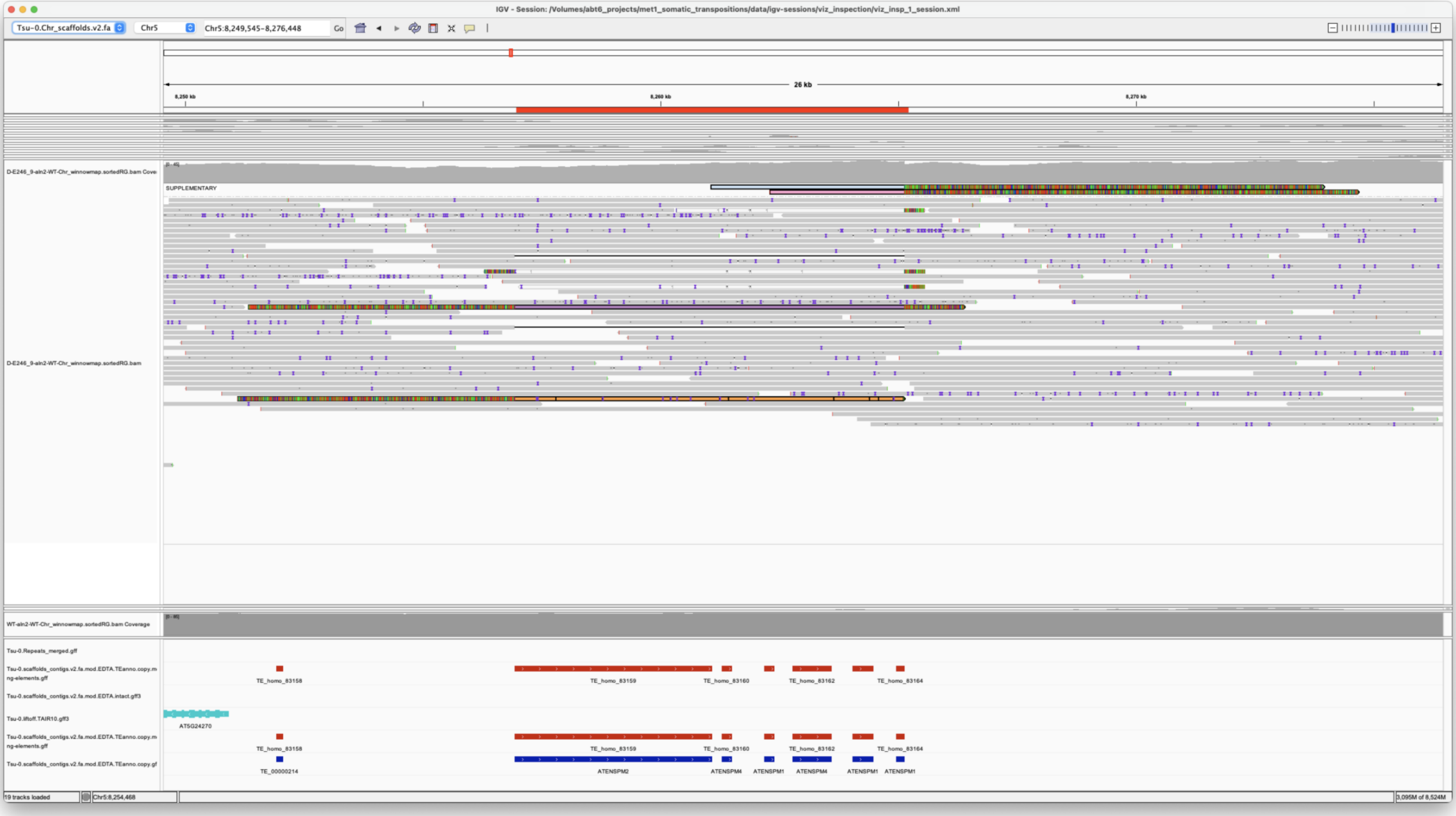

Partial

Confirmed

Chr5 15437880 15437880 - 1 Chr5;19152829;19160826;VANDAL21 m64079_240212_113350/130941060/ccs met1_09

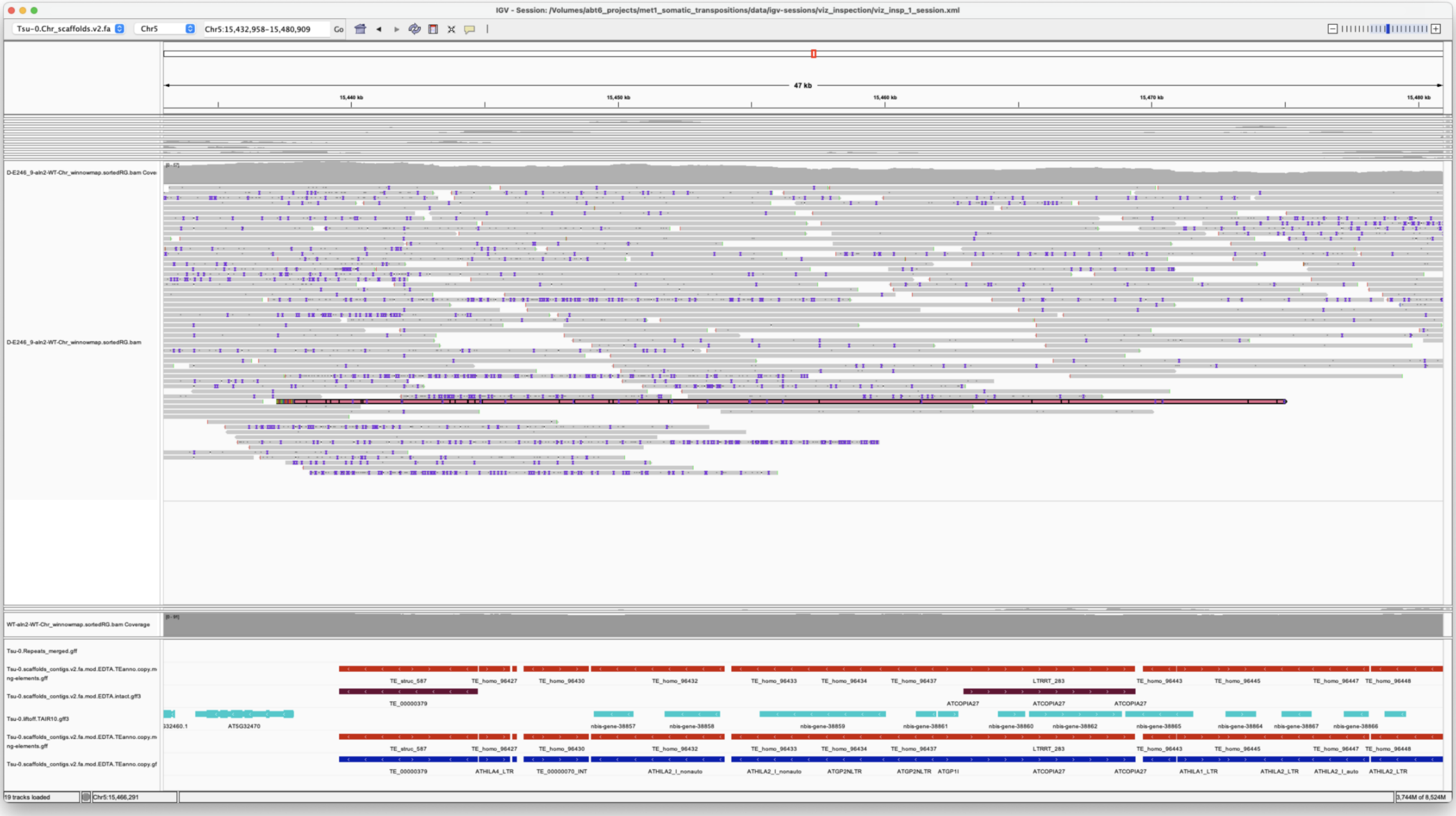

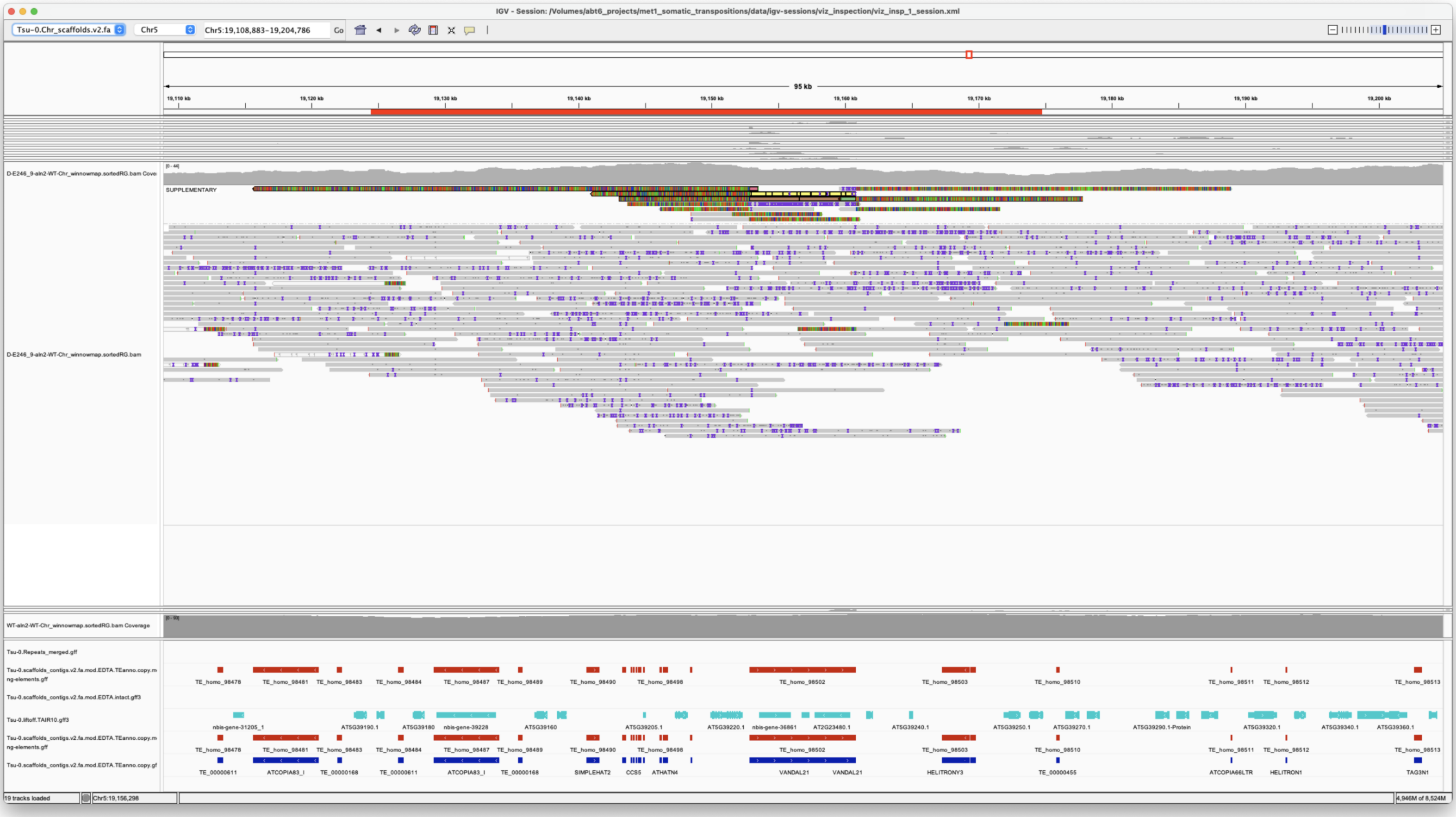

Partial

Confirmed

Chr5 15931318 15931318 - 1 Chr1;11941106;11946436;ATCOPIA93_Evade m64079_221220_112036/35193719/ccs met1_09

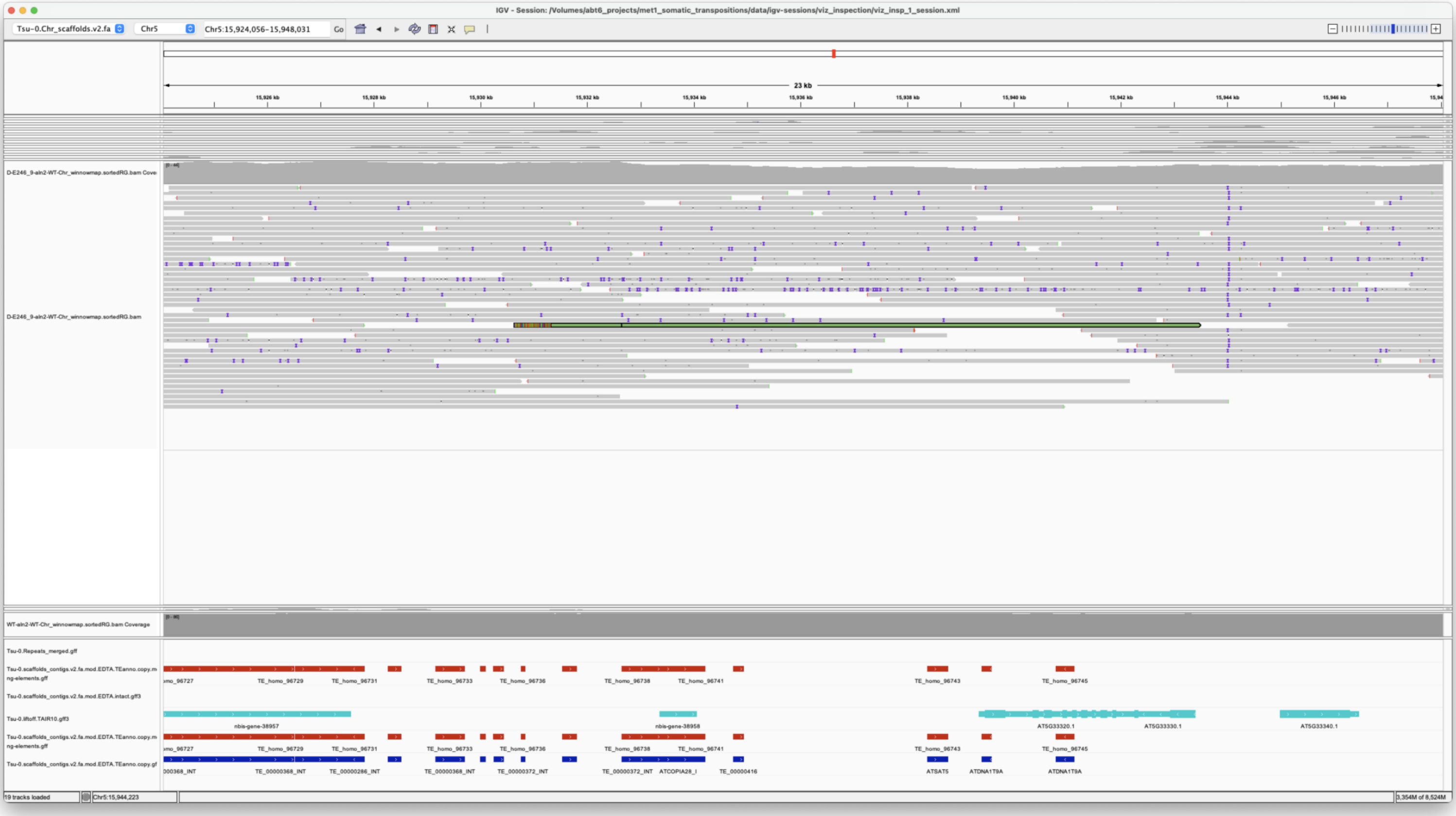

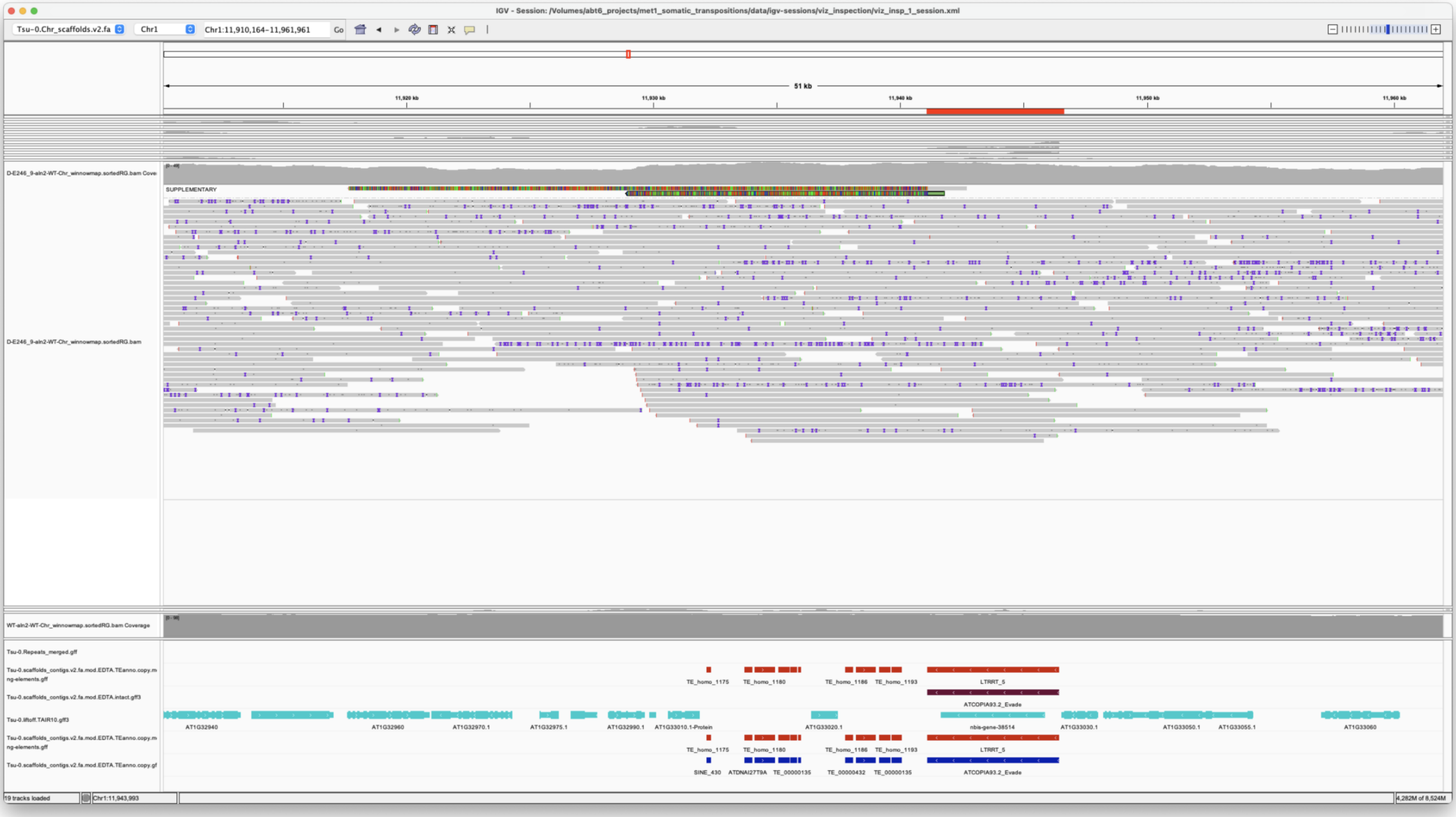

Partial

Confirmed

Additional
not detected

> m64079_221220_112036/147851825/ccs

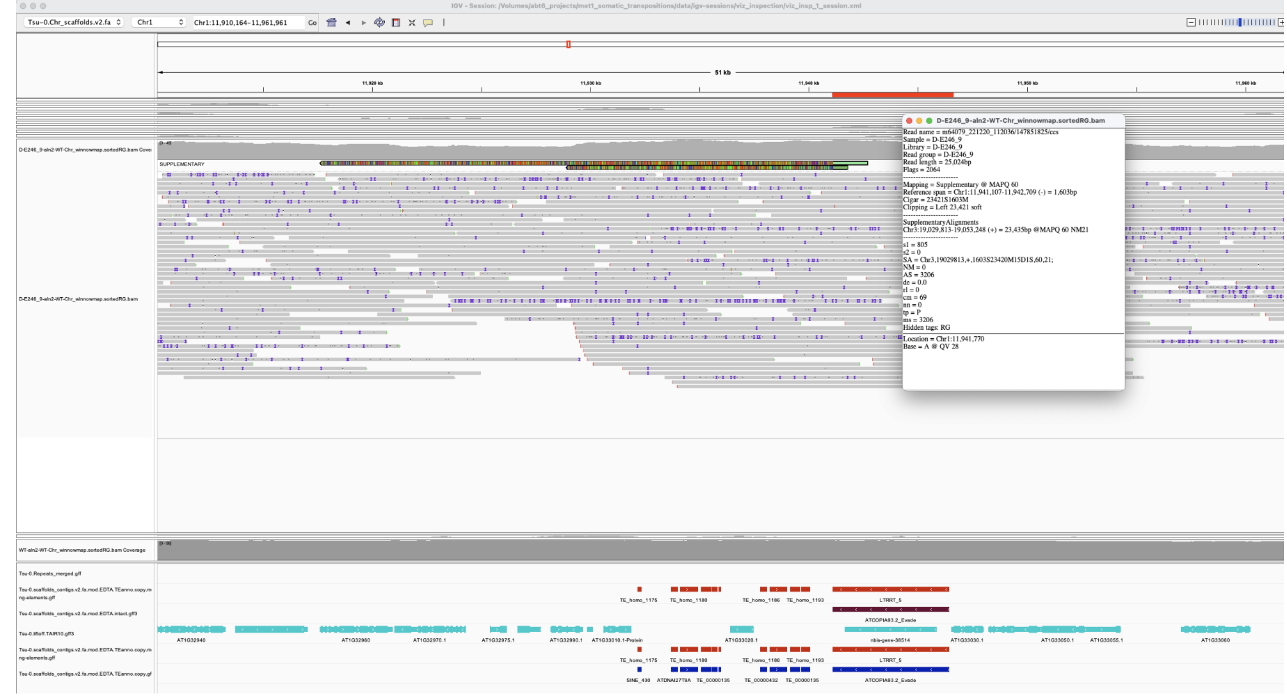

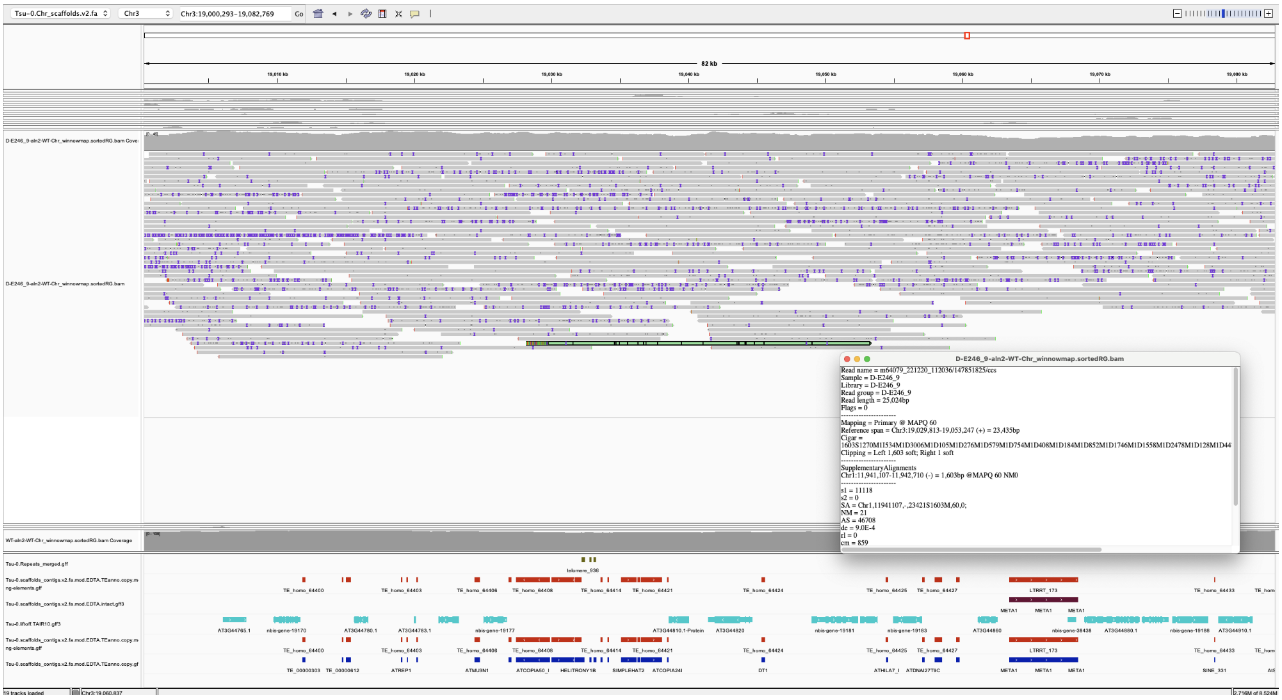

Partial

Confirmed

Chr5 19151530 19151530 + 1 Chr5;19877283;19884298;VANDAL21 m64079_221220_112036/148048259/ccs met1_09

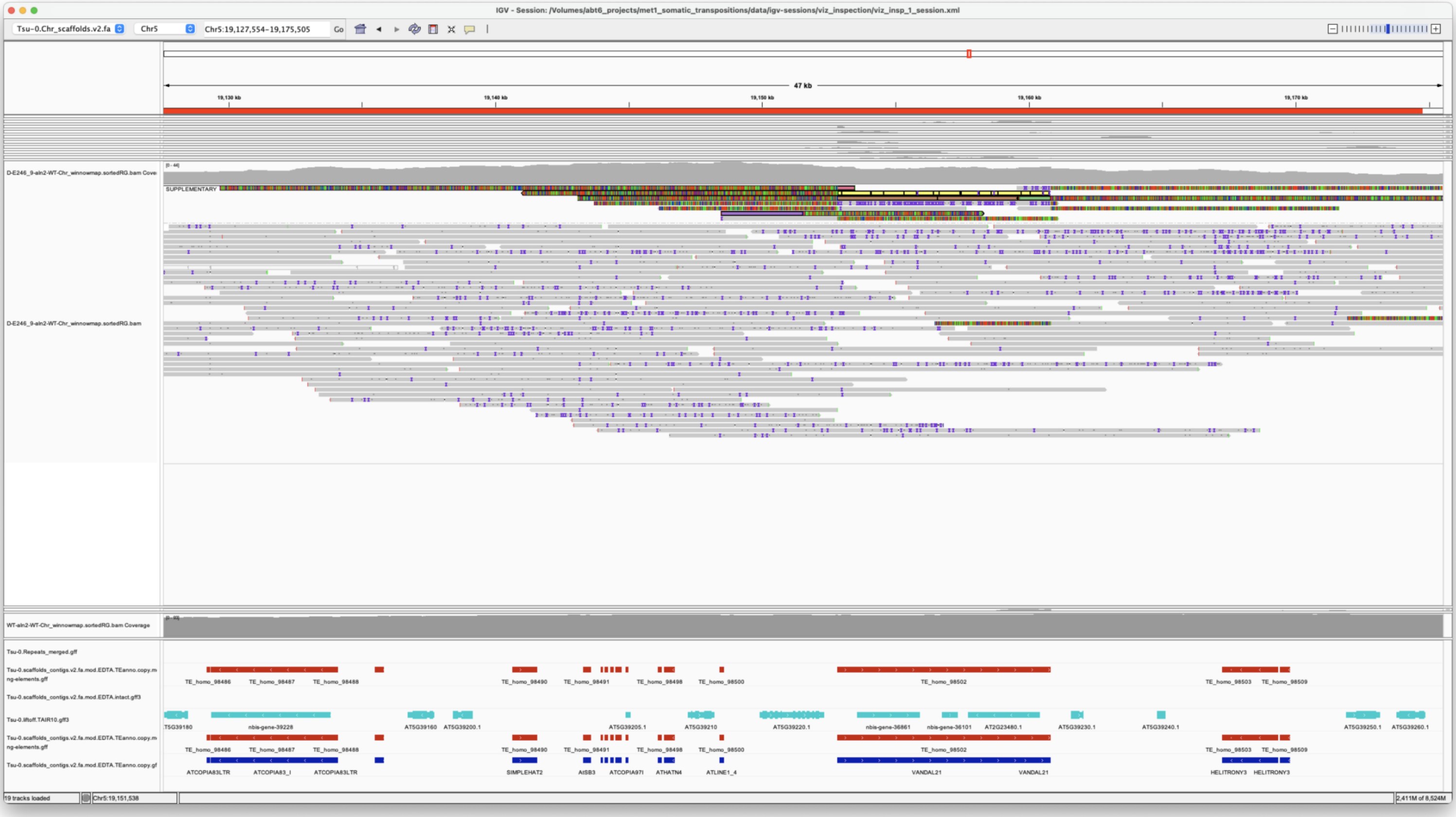

REARRANGEMENT WITH THE OTHER V21 locus subjected to rearrangements, Alternative transpositions

Hypermutable region

Rearrangement

unsupported

> Chr5 19171951 19171951 + 1 Chr5;19152829;19160826;VANDAL21 m64079_221220_112036/112722986/ccs met1_09

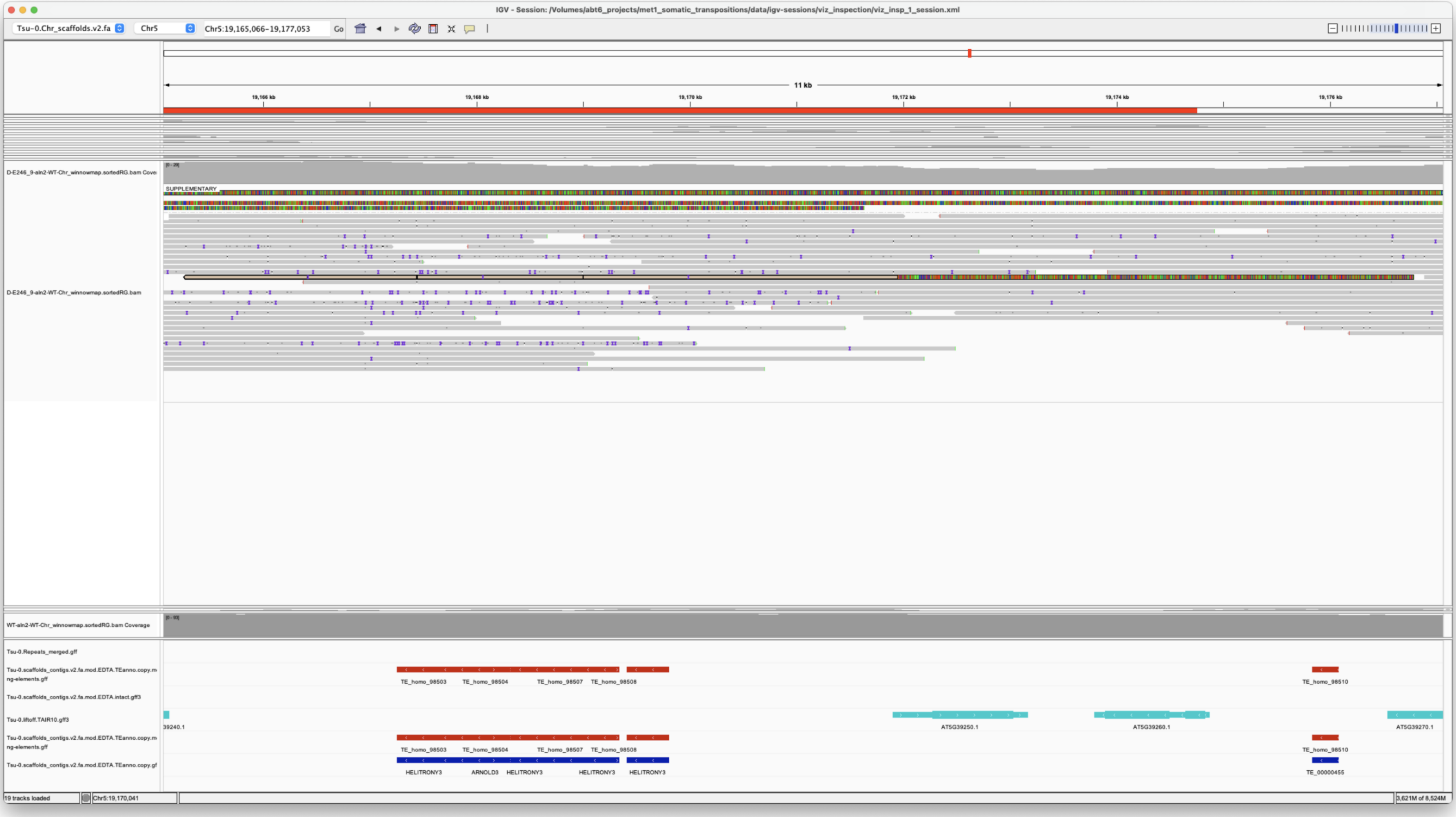

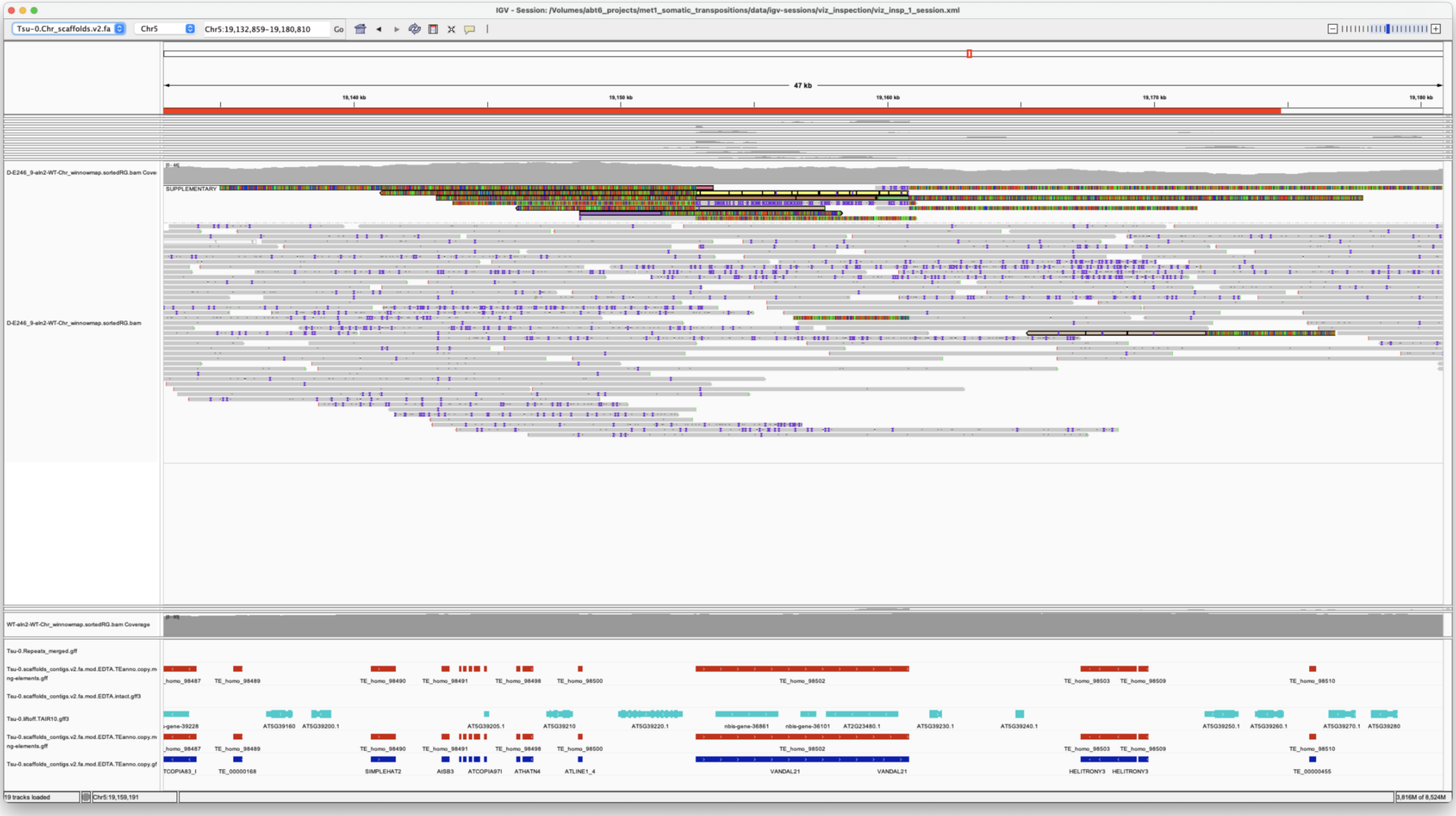

Probably too close to be a transposition

Rearrangement

unsupported

---

met1_10

> ## DIFFICULT CASE TO AUTOMATE: m64079_221220_112036/101450167/ccs 0 836 Chr5 15063906 15064742 16743 + ATHILA1.1_I_nonauto m64079_221220_112036/101450167/ccs 836 8838 Chr5 19152825 19160827 16743 + VANDAL21 m64079_221220_112036/101450167/ccs 8836 16742 Chr5 15064732 15072638 16743 + .

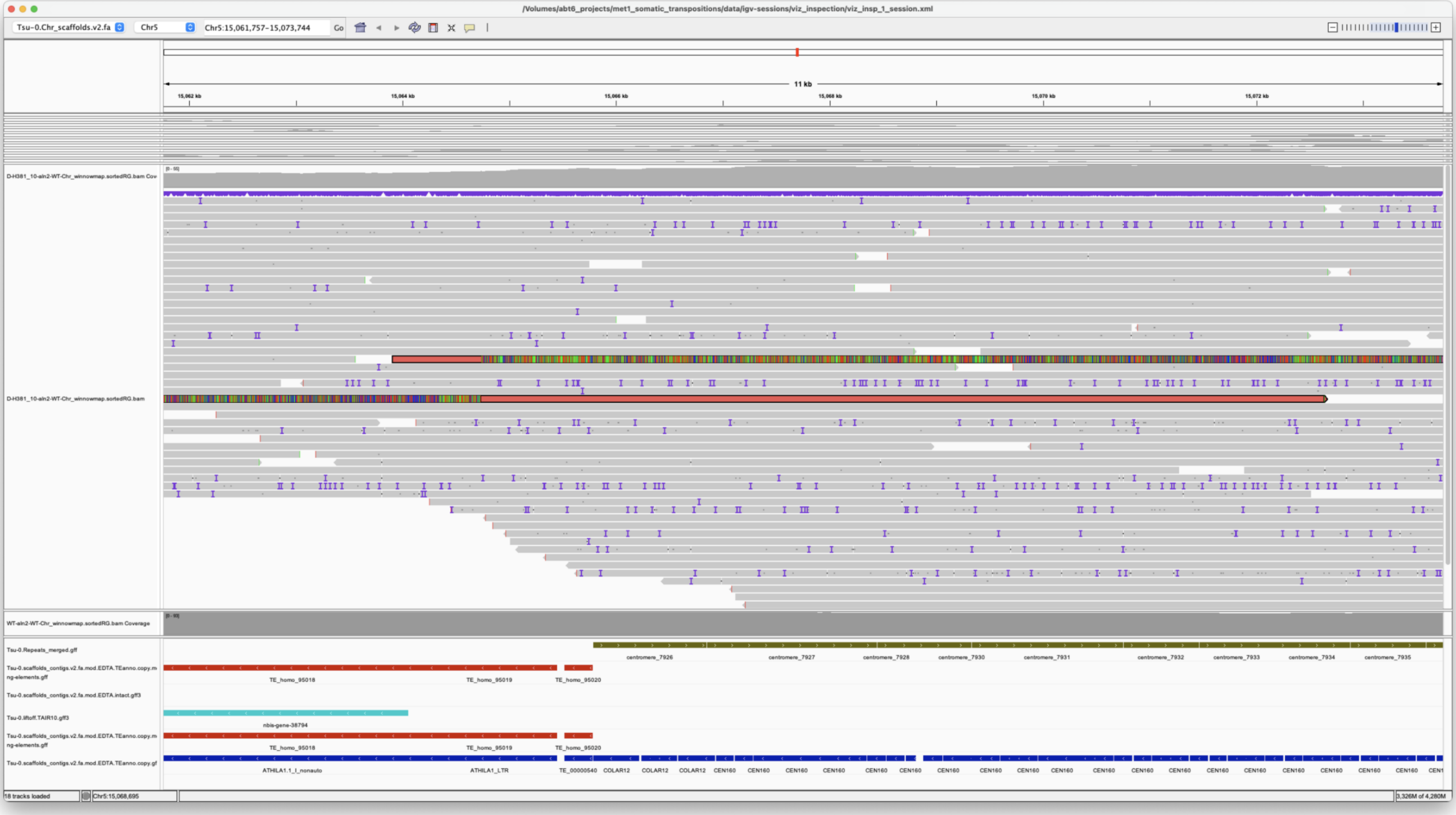

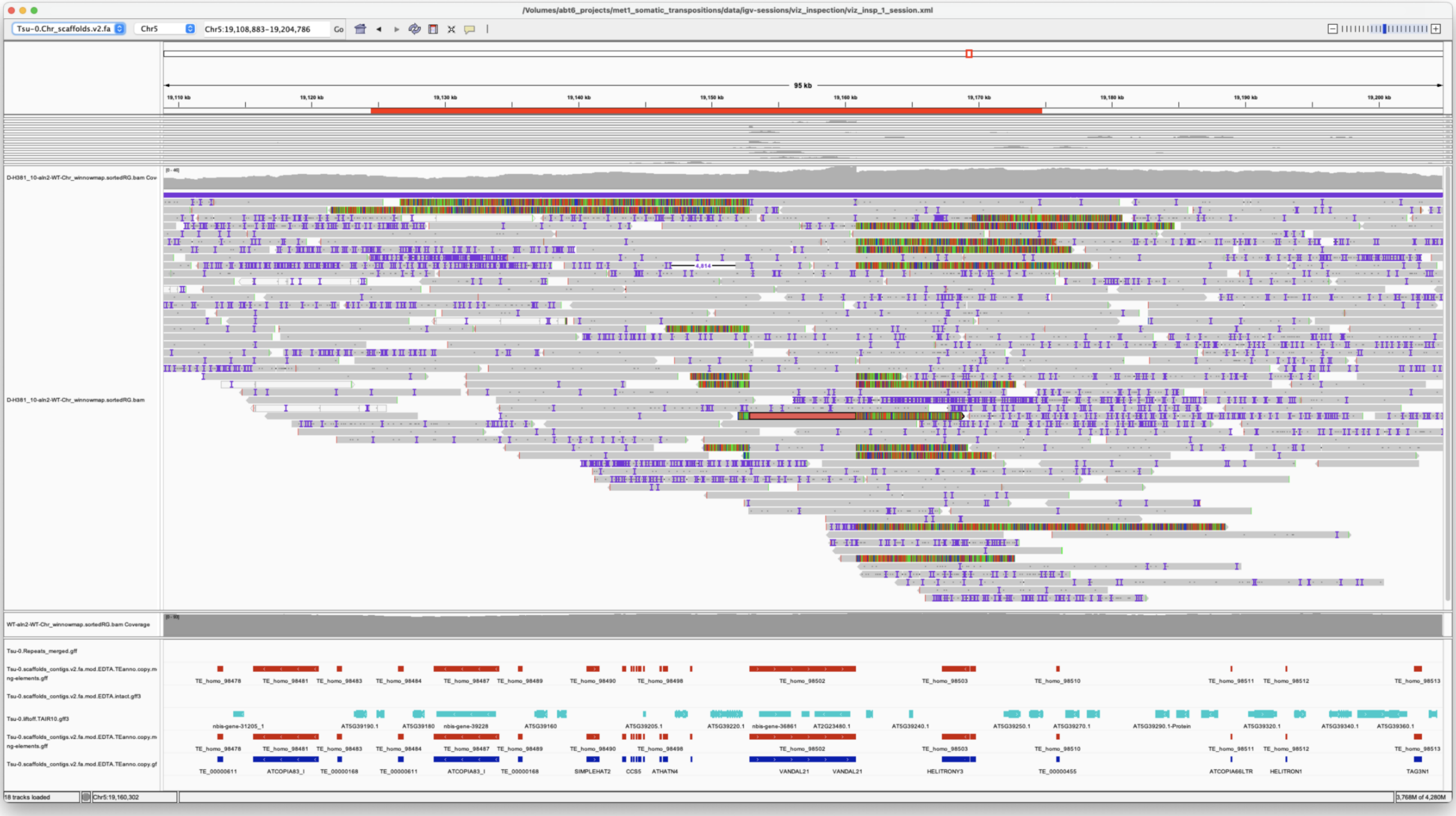

Central
TSD

Confirmed

> **DIFFICULT CASE TO AUTOMATE: m64079_221220_112036/102368776/ccs 0 551 Chr3 20269803 20270354 10204 - Chr3:20278947..20263318 m64079_221220_112036/102368776/ccs 551 1575 Chr5 875413 876437 10204 + PutativeDIFFICULT CASE TO AUTOMATE: m64079_221220_112036/102368776/ccs 0 551 Chr3 20269803 20270354 10204 - Chr3:20278947..20263318 m64079_221220_112036/102368776/ccs 551 1575 Chr5 875413 876437 10204 + PACm64079_221220_112036/102368776/ccs 1570 10204 Chr3 20261179 20269814 10204 - . met1_10PAC m64079_221220_112036/102368776/ccs 1570 10204 Chr3 20261179 20269814 10204 - . met1_10**

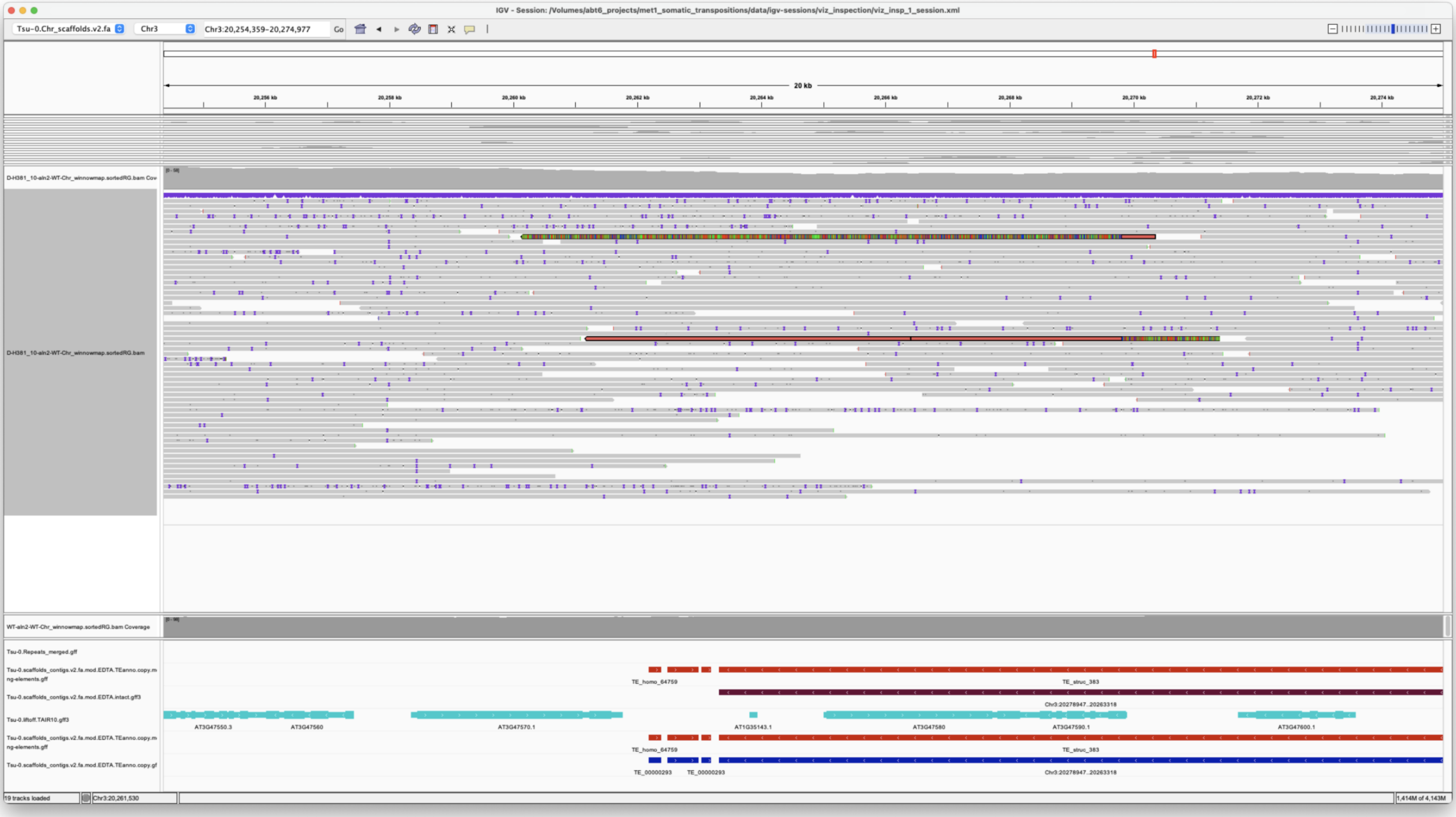

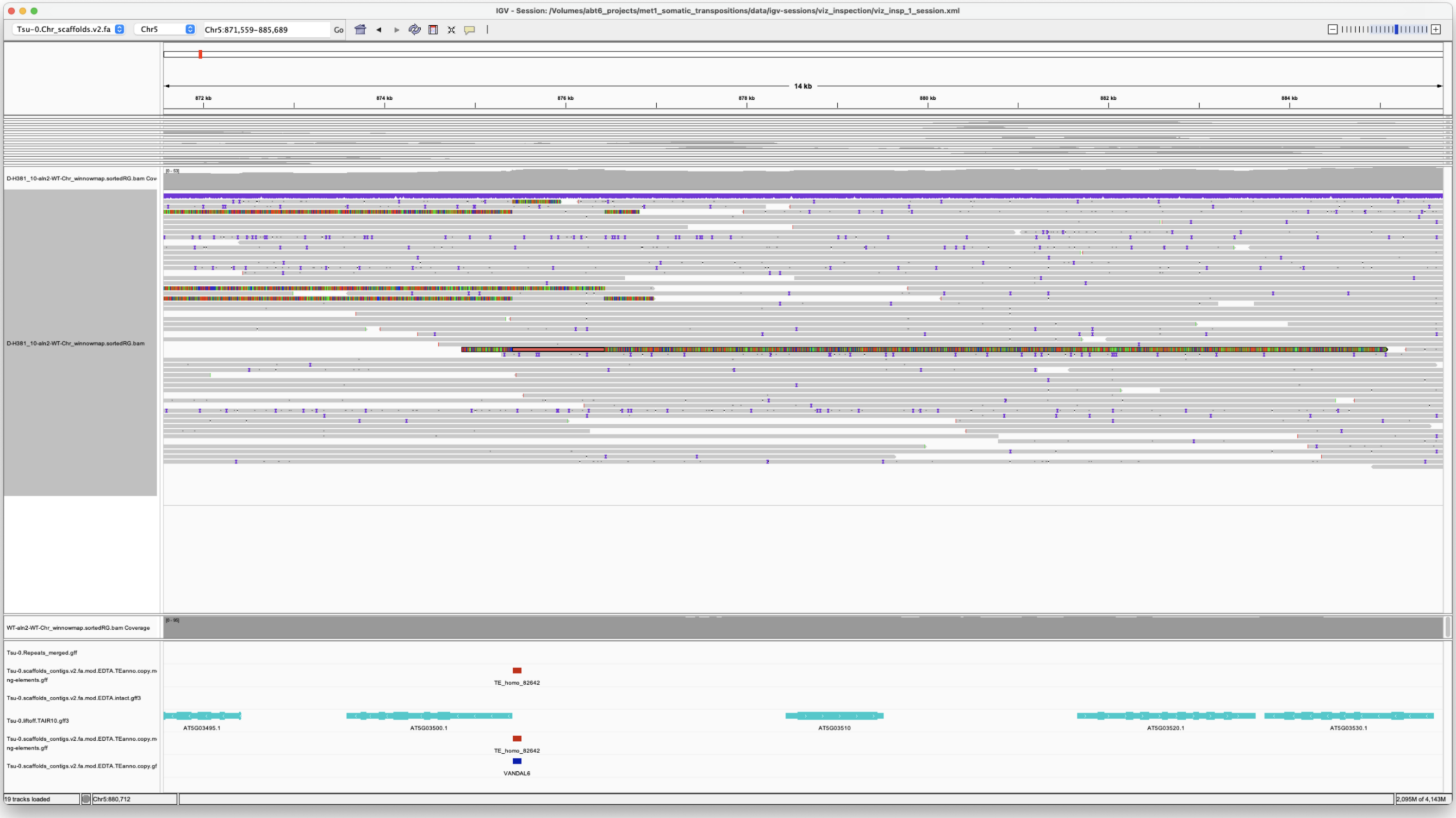

Central
TSD

Confirmed

> DIFFICULT CASE TO AUTOMATE: m64079_221220_112036/14289475/ccs 0 1181 Chr3 20158137 20159318 14163 - VANDAL6 m64079_221220_112036/14289475/ccs 1413 9206 Chr3 20158132 20165927 14163 + VANDAL6 m64079_221220_112036/14289475/ccs 9206 14163 Chr3 20161193 20166150 14163 - VANDAL6

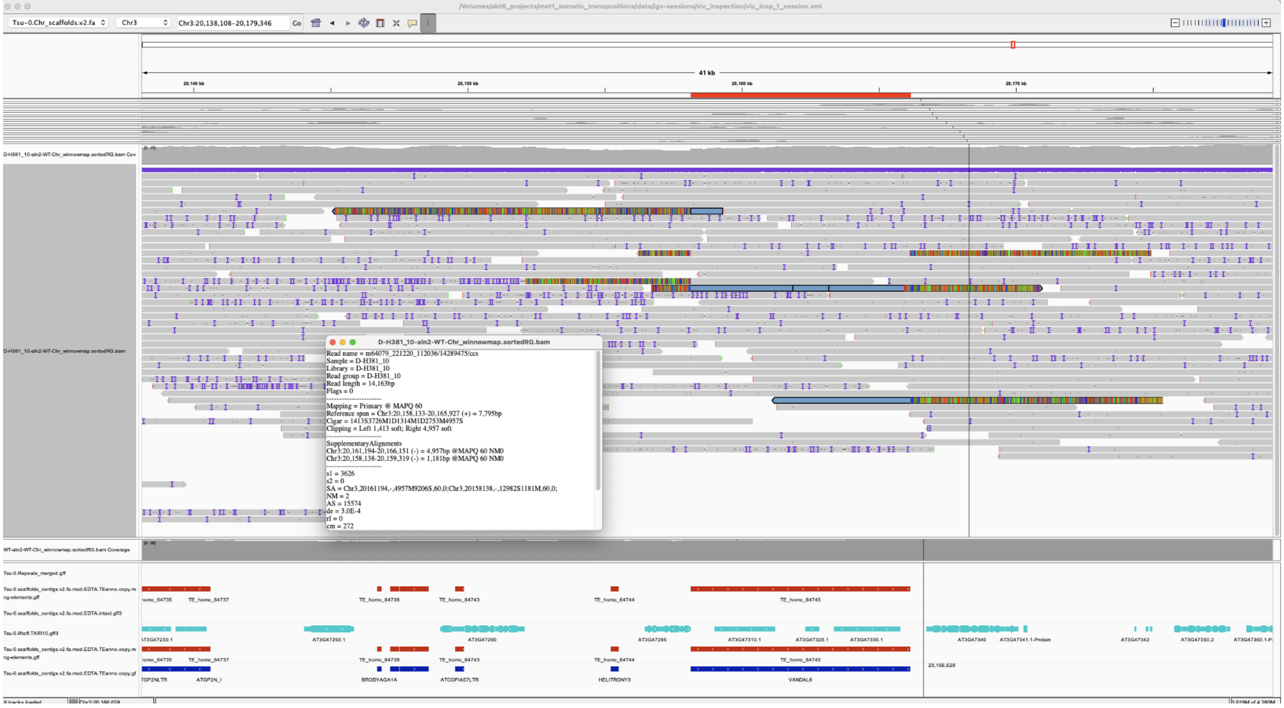

it is a rearrangement inversion with duplication!

unsupported

Chr1 10454365 10454365 + 1 Chr5;875414;876434;PAC m64079_221220_112036/177408517/ccs met1_10

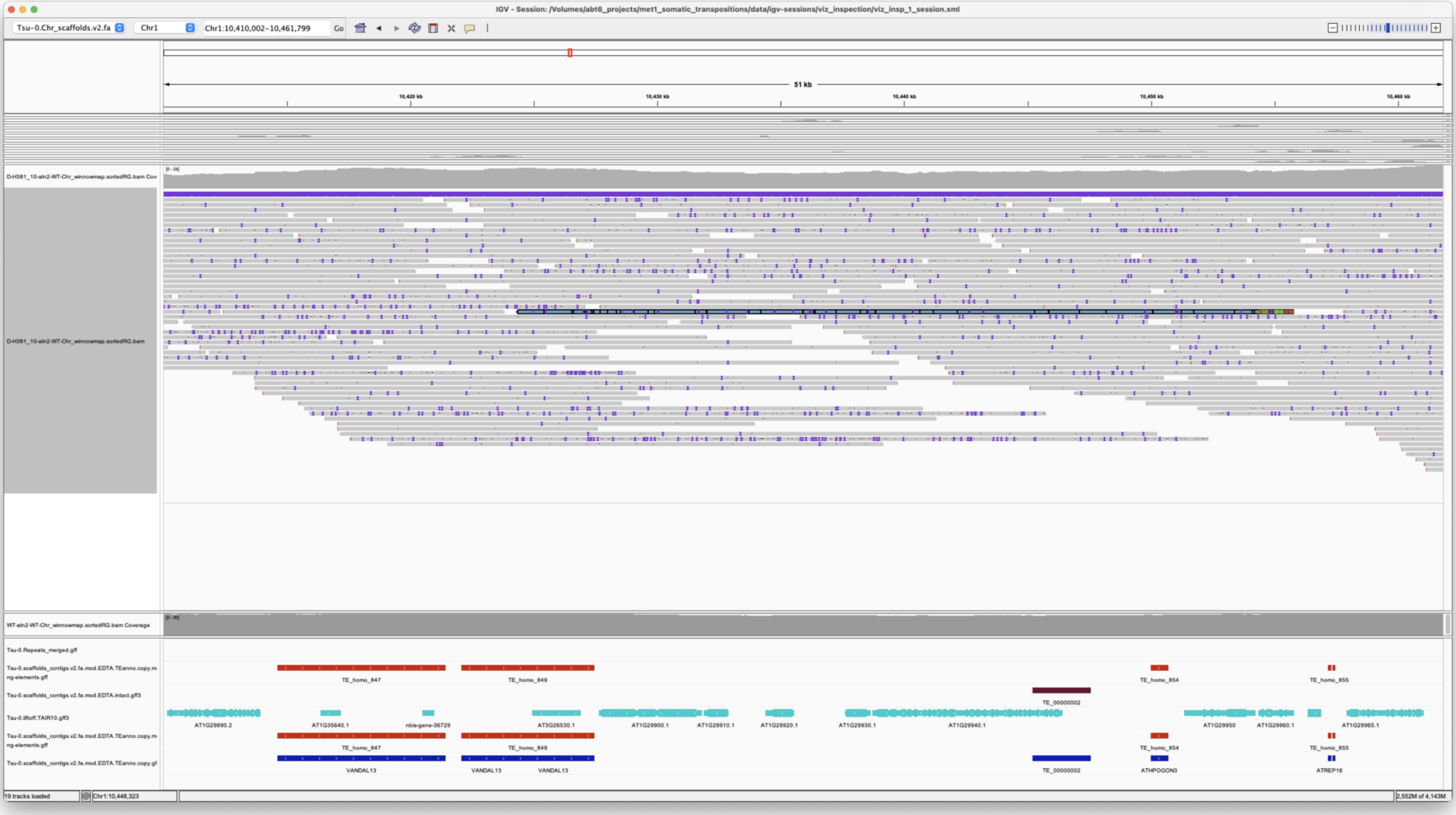

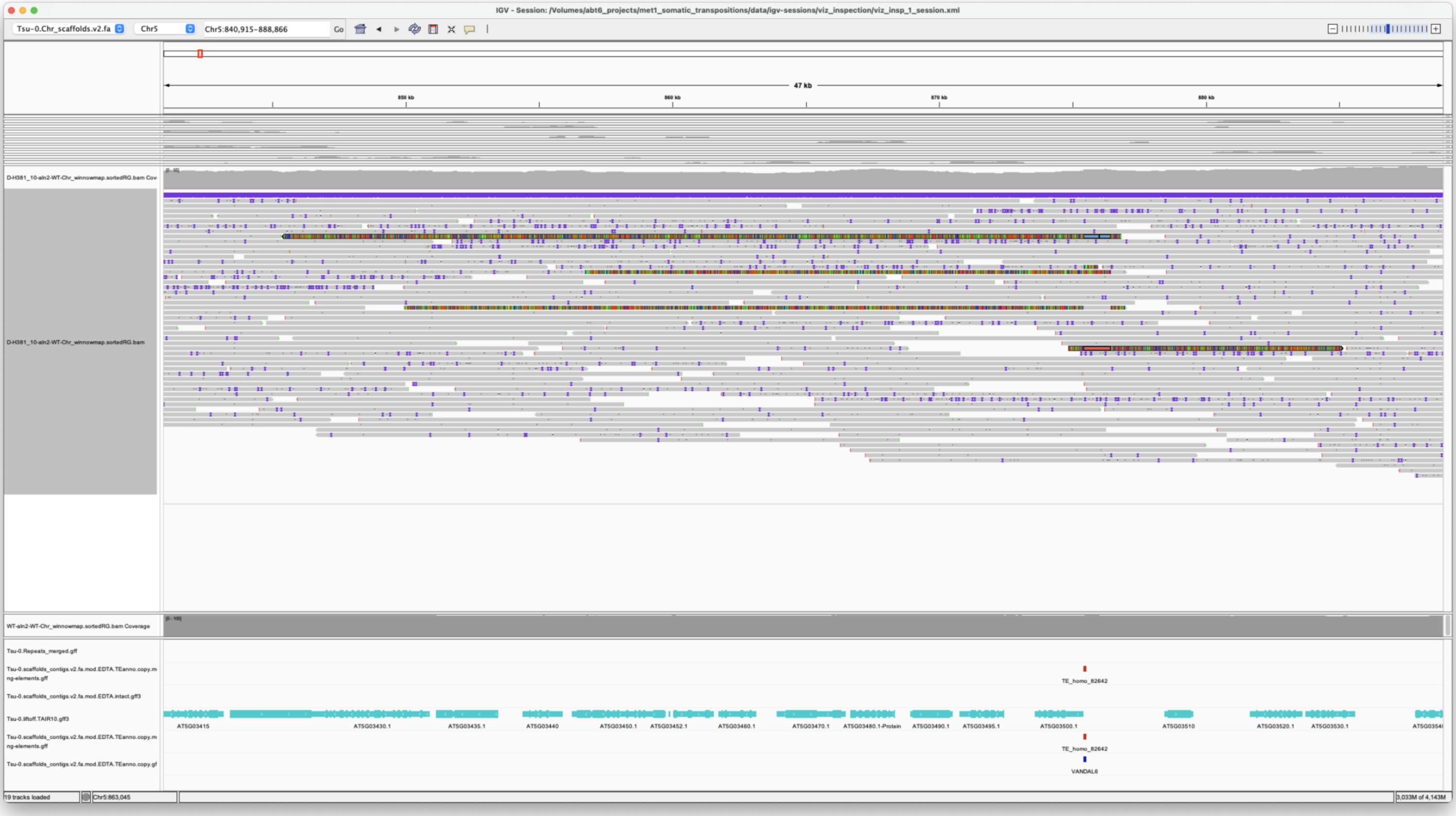

Partial

Confirmed

Chr1 3842584 3842586 -,-,- 3 Chr3;16344522;16352497;VANDAL6,Chr3;16344522;16352497;VANDAL6,Chr3;16344522;16352497;VANDAL6 m64079_221220_112036/145426603/ccs,m64079_221220_112036/86837412/ccs,m64079_240212_113350/93847777/ccs met1_10

Chr1 3842593 3842595 -,- 2 Chr3;16344522;16352497;VANDAL6,Chr3;16344522;16352497;VANDAL6 m64079_221220_112036/12387756/ccs,m64079_221220_112036/66126715/ccs met1_10

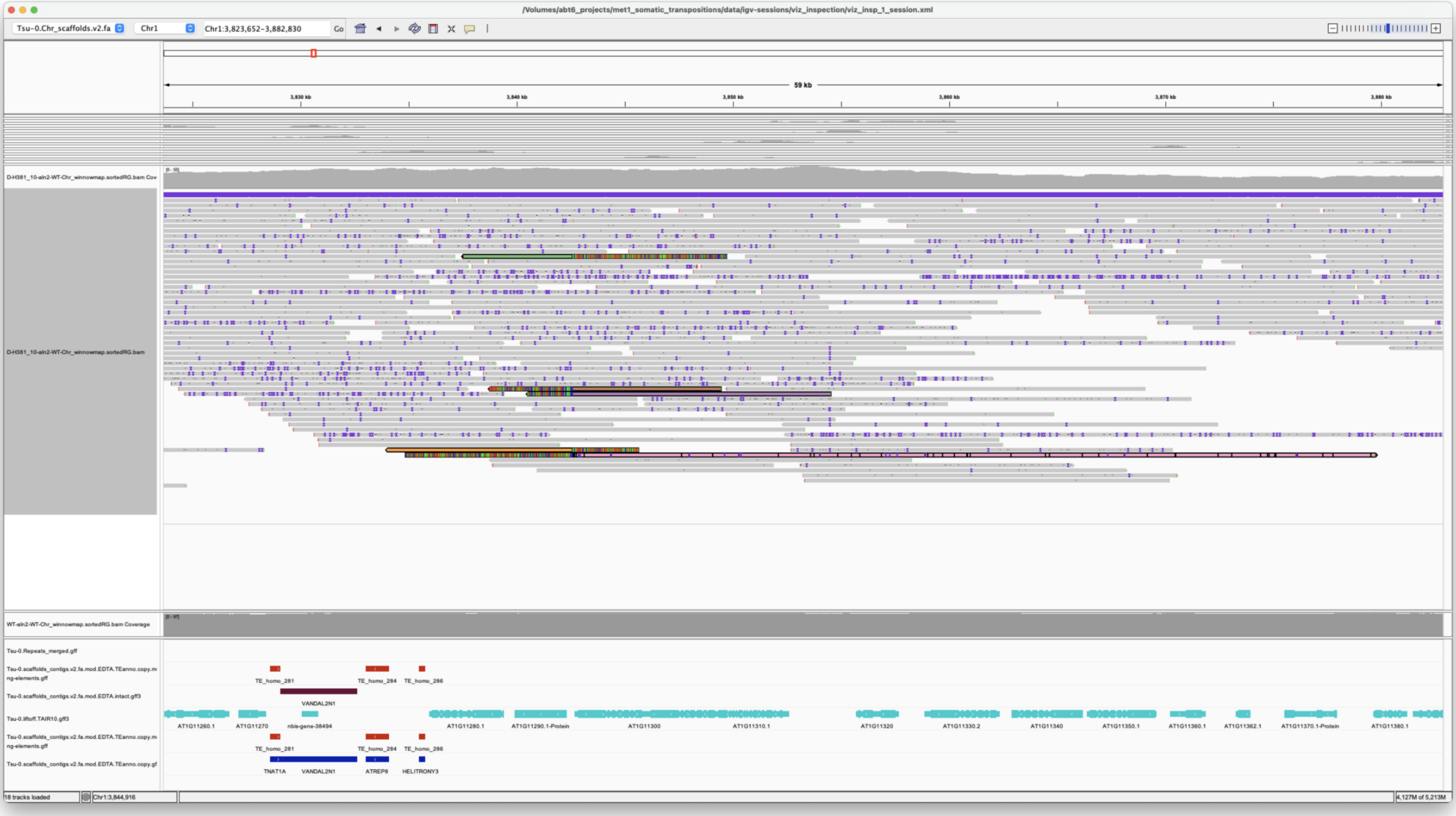

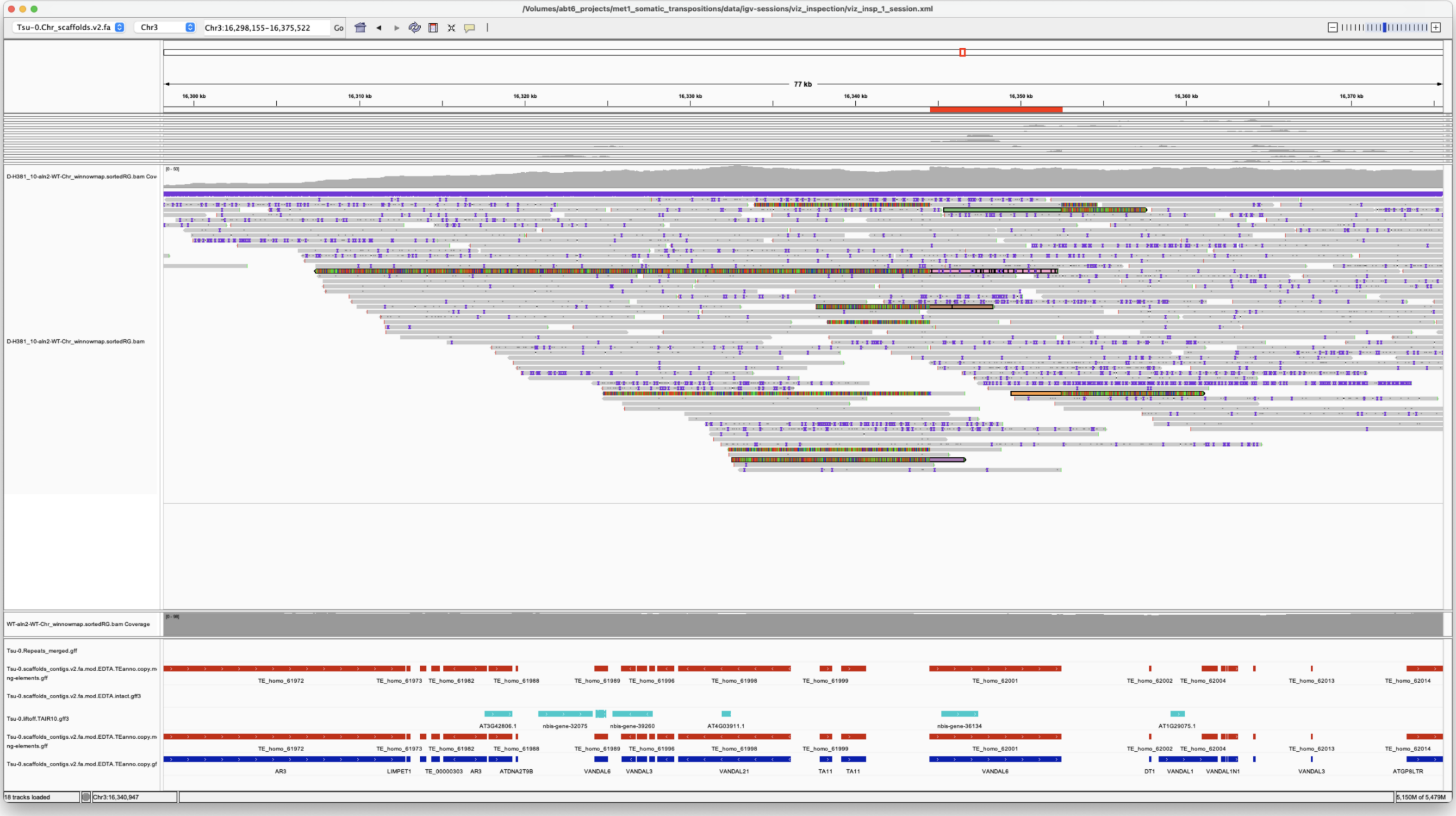

Partial

Confirmed

Chr1 23929316 23929316 + 1 Chr5;19152829;19160826;VANDAL21 m64079_221220_112036/163251438/ccs met1_10

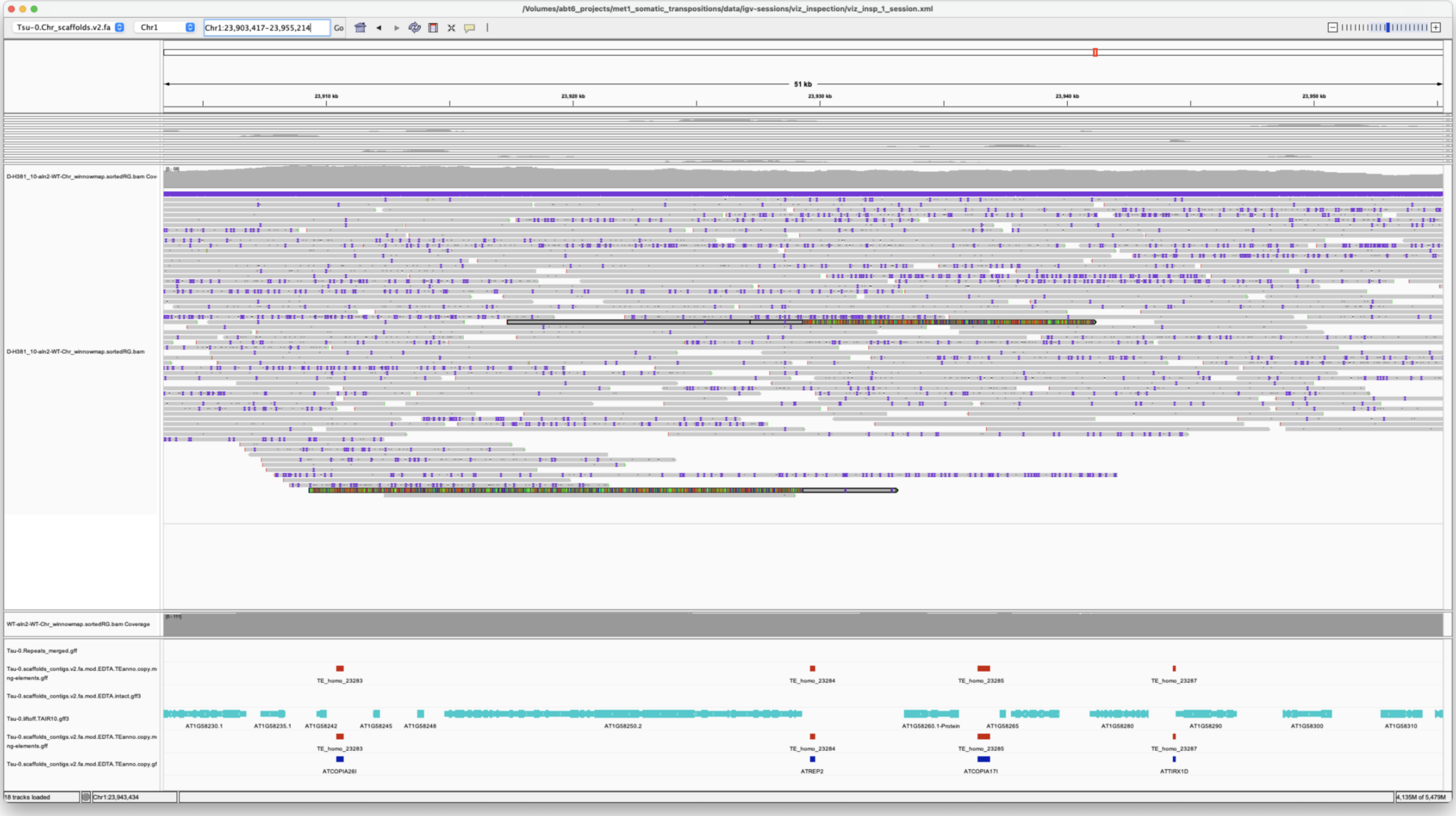

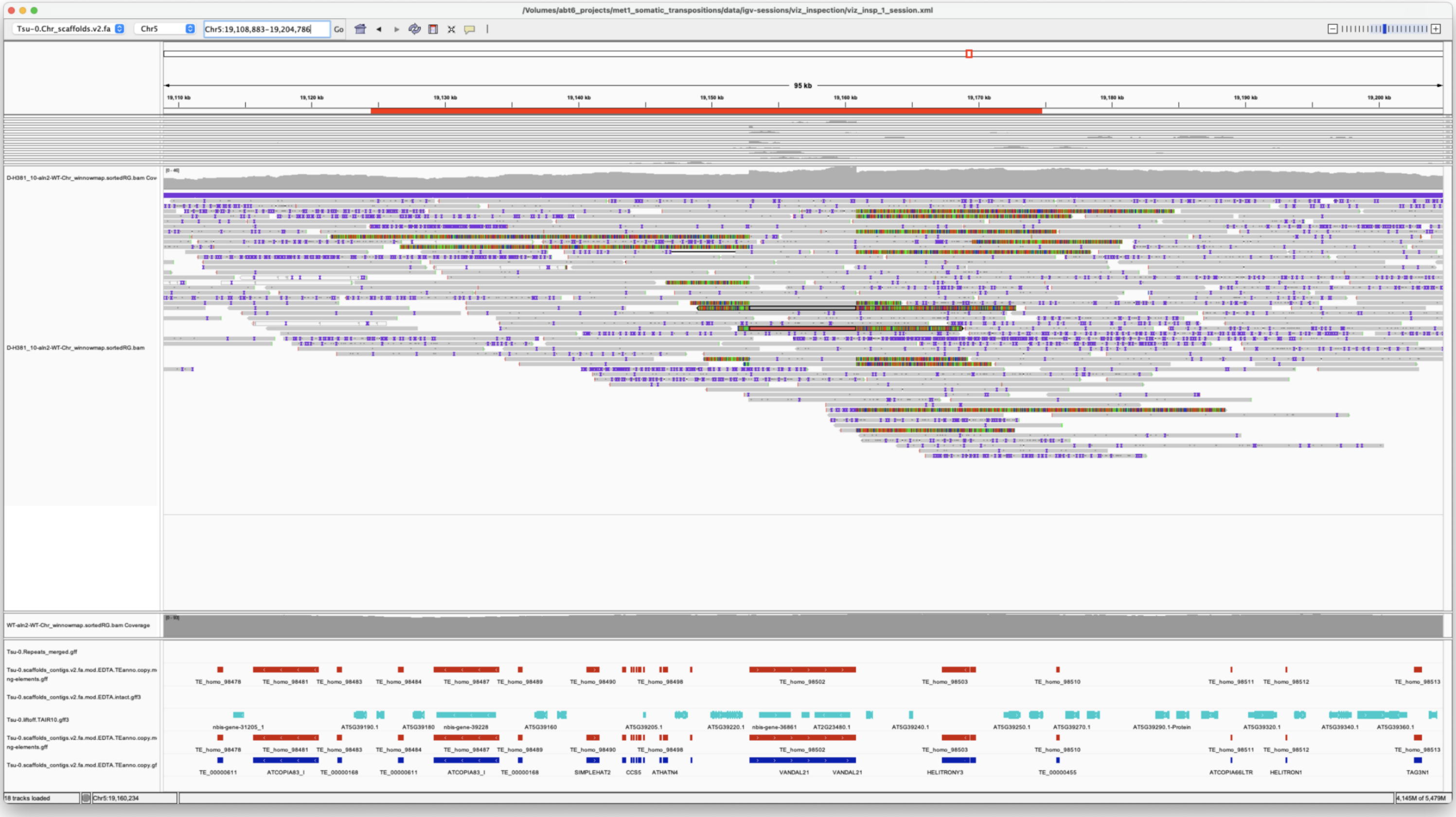

Central
TSD

Confirmed

Chr1 28965760 28965760 - 1 Chr1;11941106;11946436;ATCOPIA93_Evade m64079_221220_112036/143788169/ccs met1_10

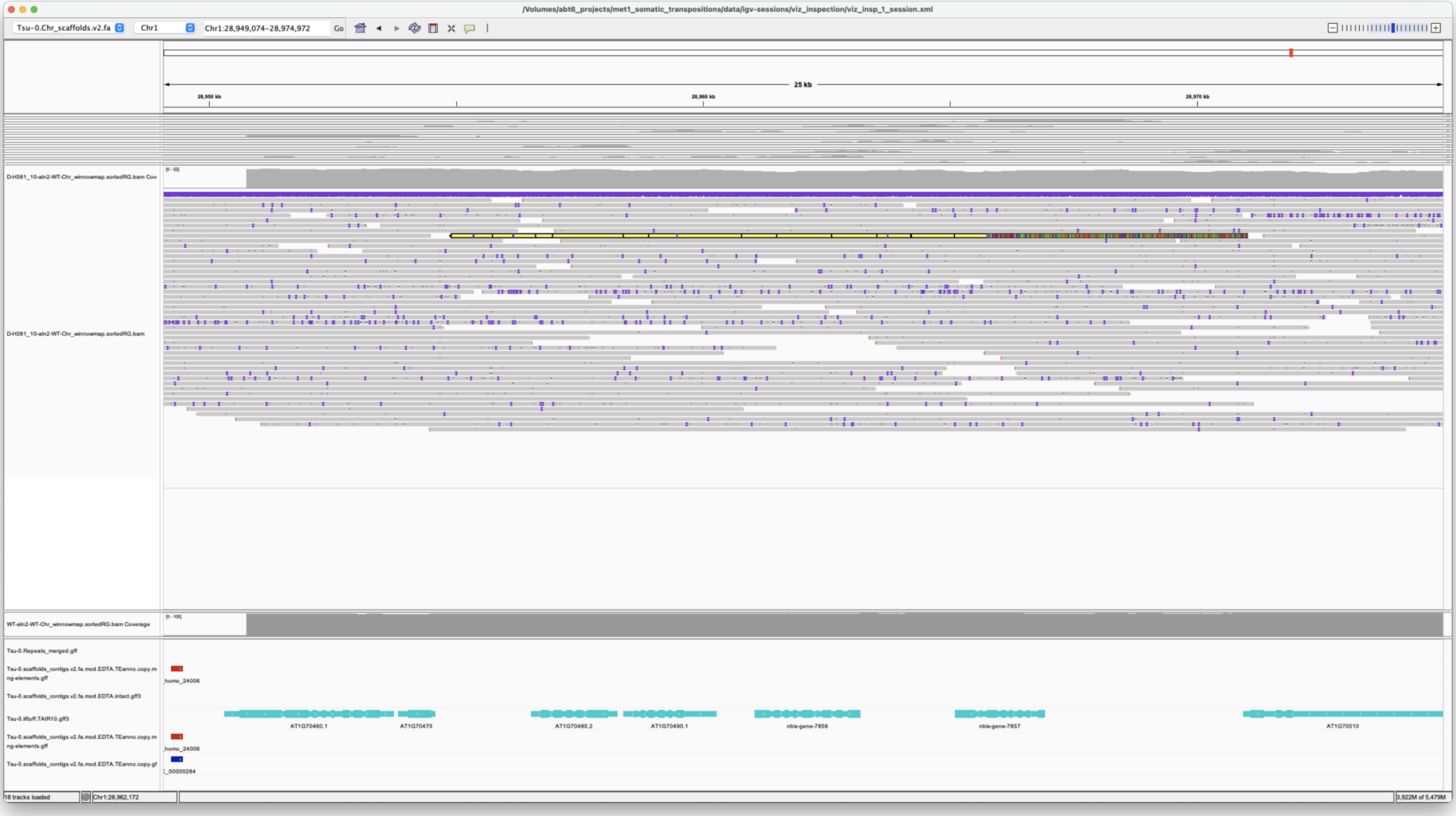

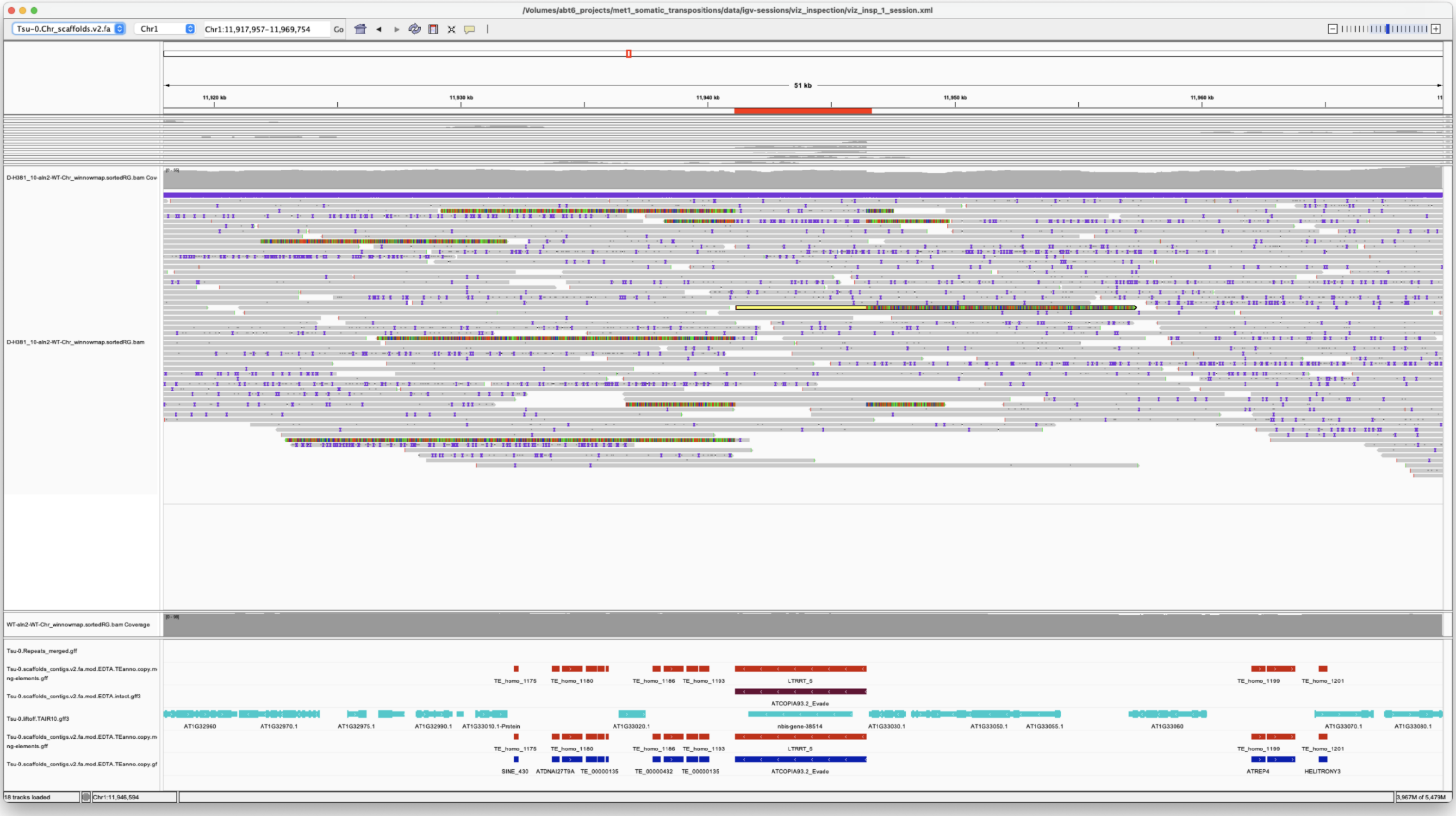

Partial

Confirmed

> Chr1 31760953 31760953 + 1 Chr3;20158137;20166150;VANDAL6 m64079_221220_112036/151979729/ccs met1_10

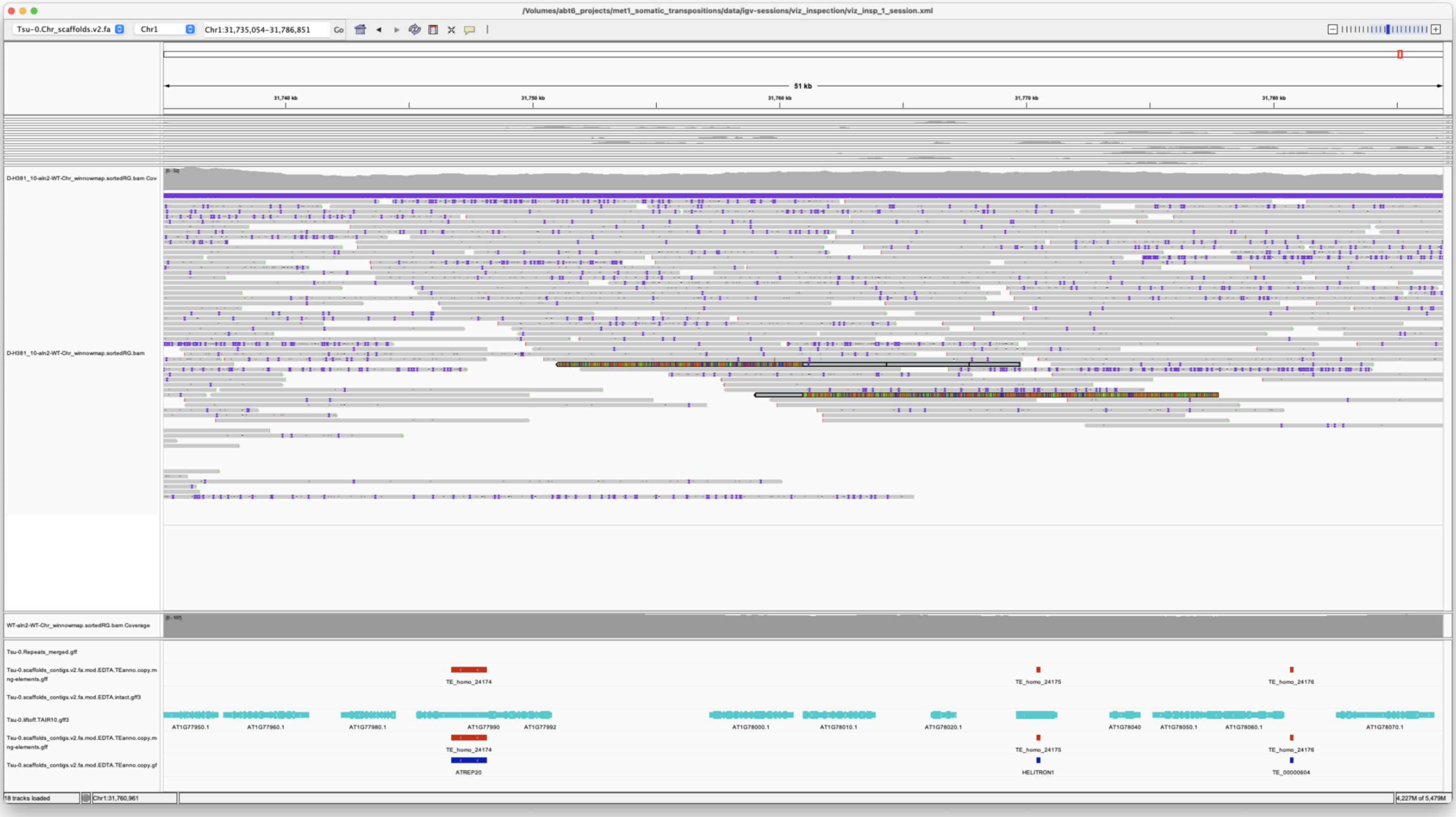

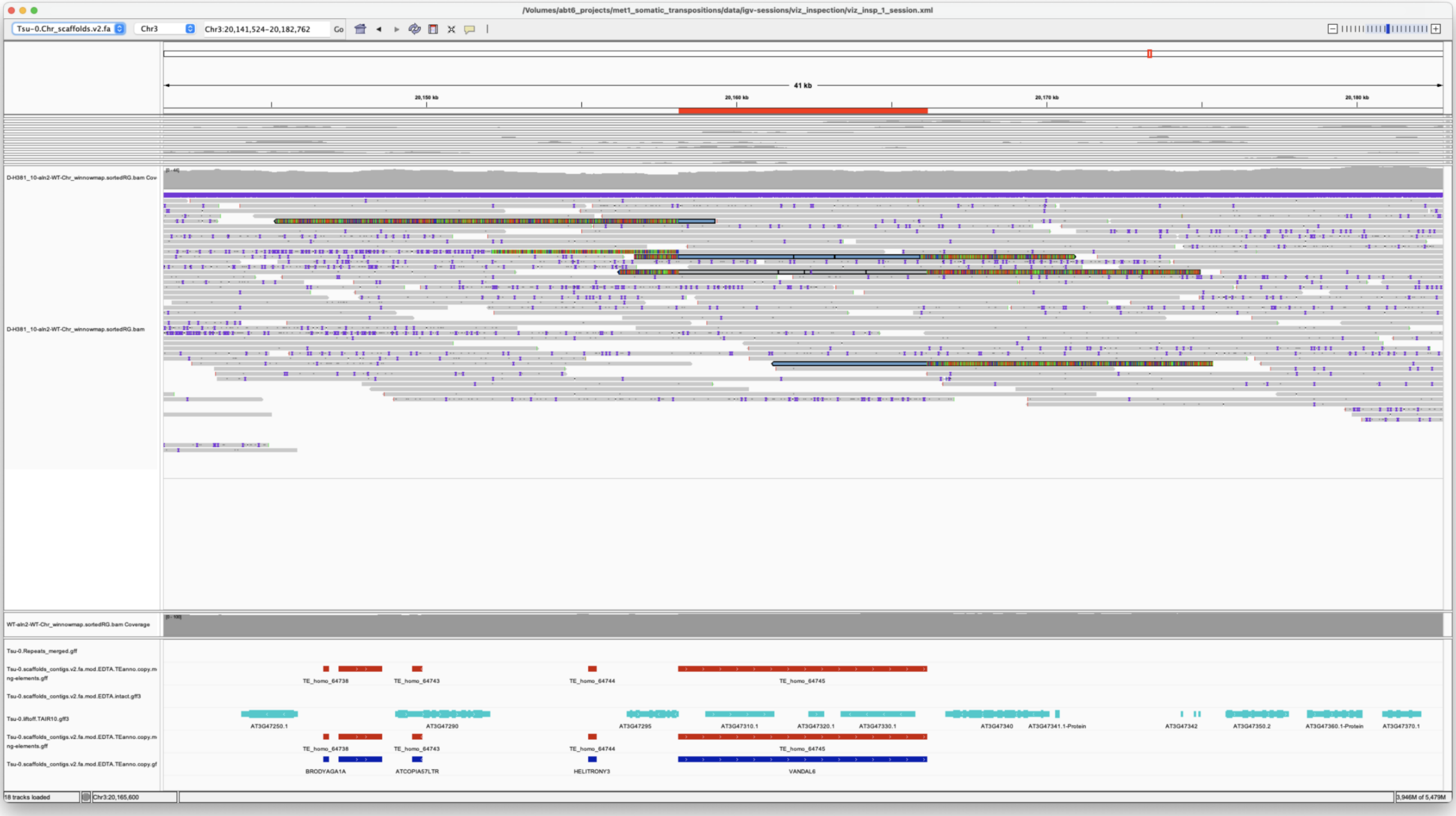

Central
TSD

Confirmed

Chr2 7303606 7303606 + 1 Chr5;19152829;19160826;VANDAL21 m64079_221220_112036/92866368/ccs met1_10

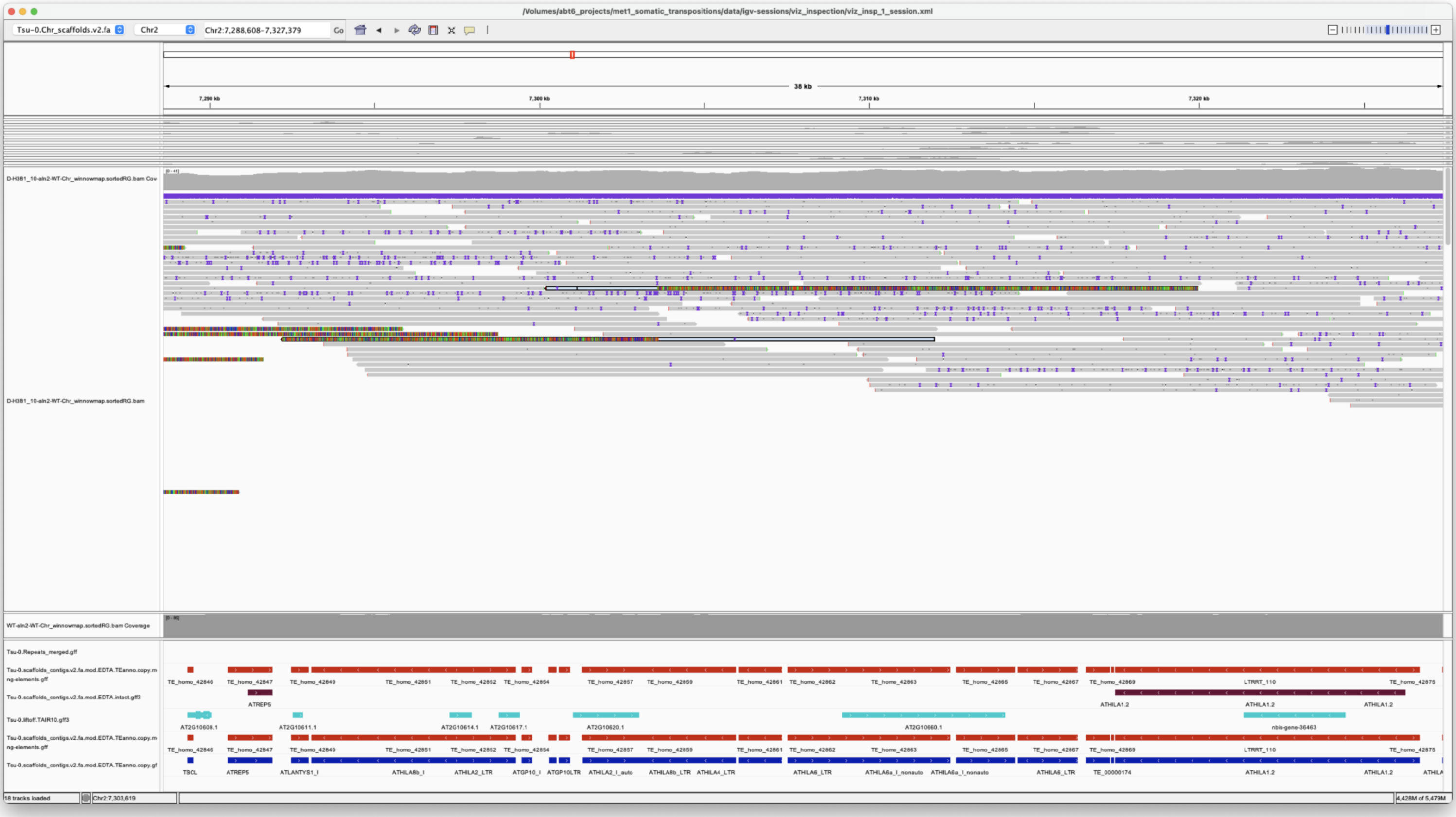

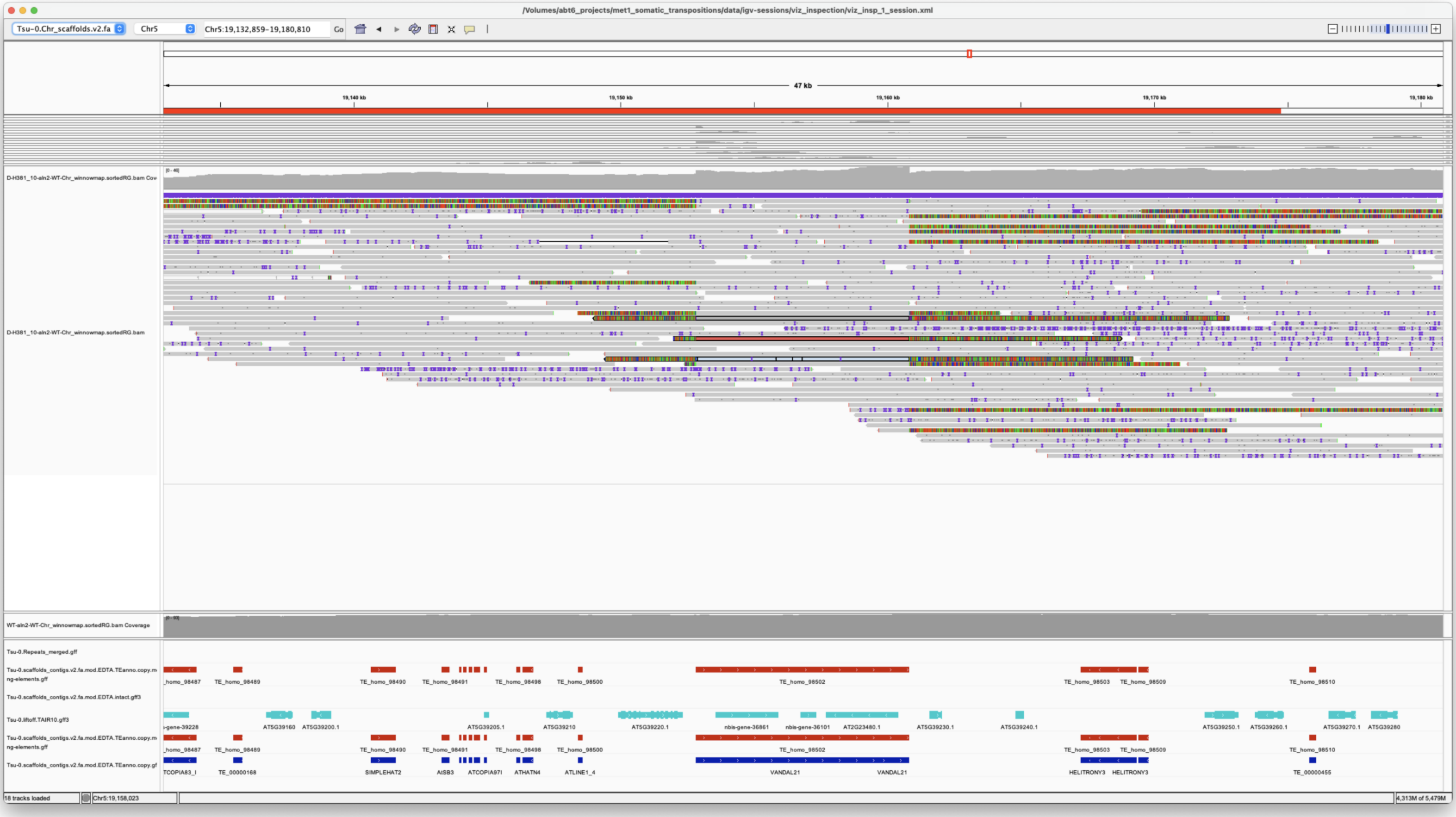

Central
TSD?

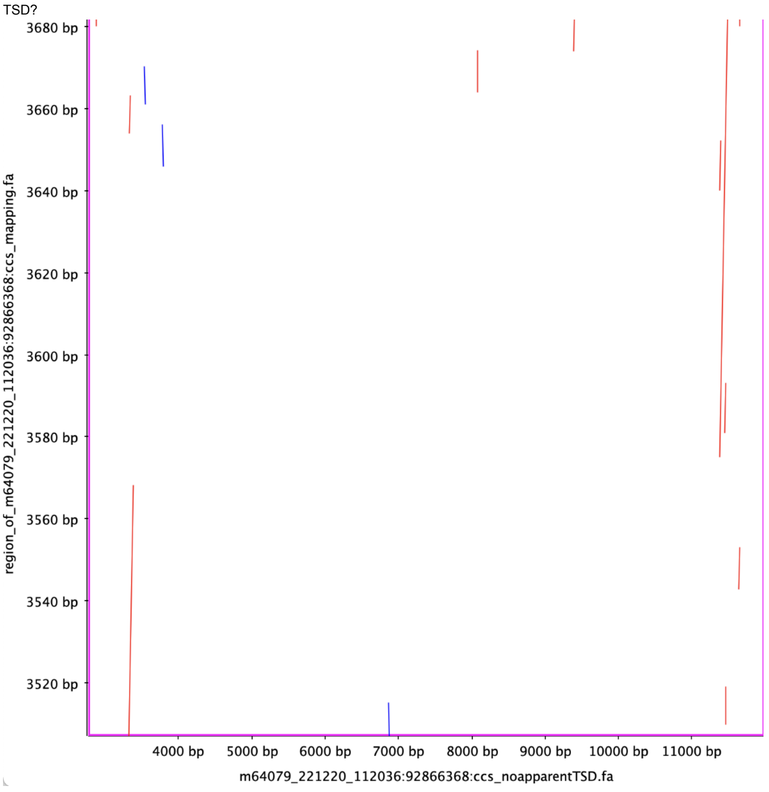

Confirmed

Chr2 8327863 8327863 + 1 Chr5;19152829;19160826;VANDAL21 m64079_221220_112036/82247748/ccs met1_10

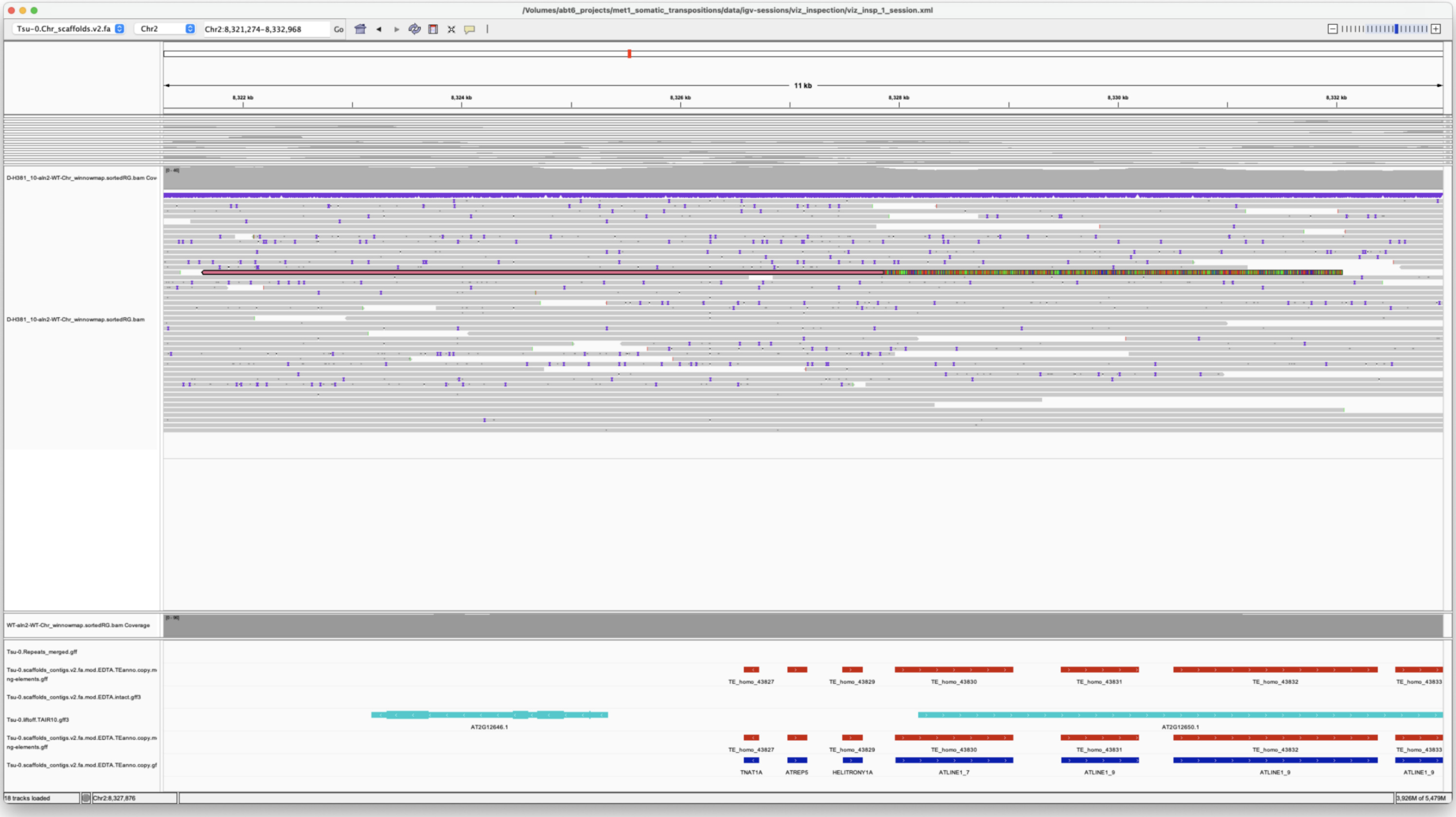

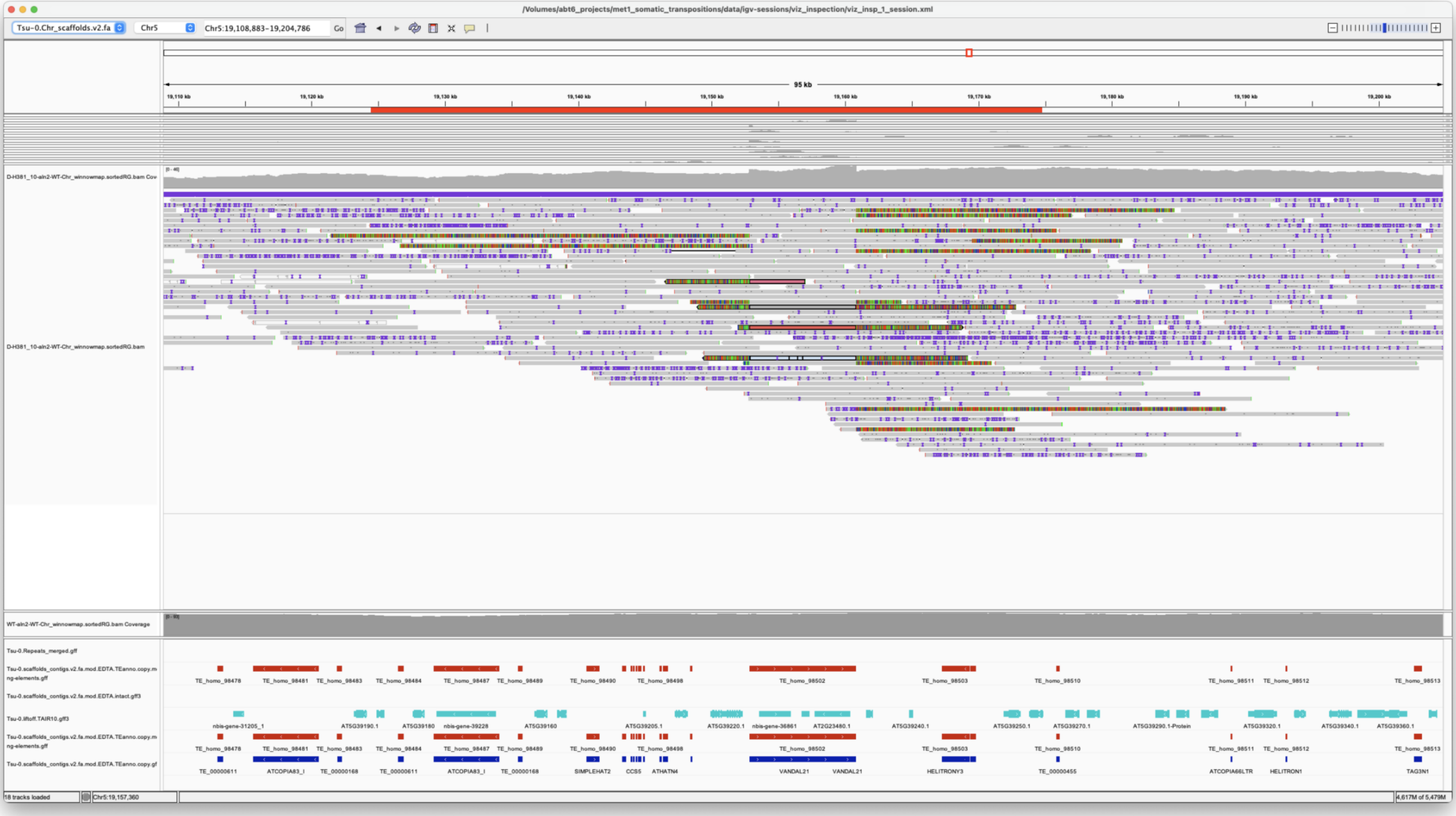

Partial

Confirmed

> Chr2 9375683 9375683 + 1 Chr1;11941106;11946436;ATCOPIA93_Evade m64079_221220_112036/78775709/ccs met1_10

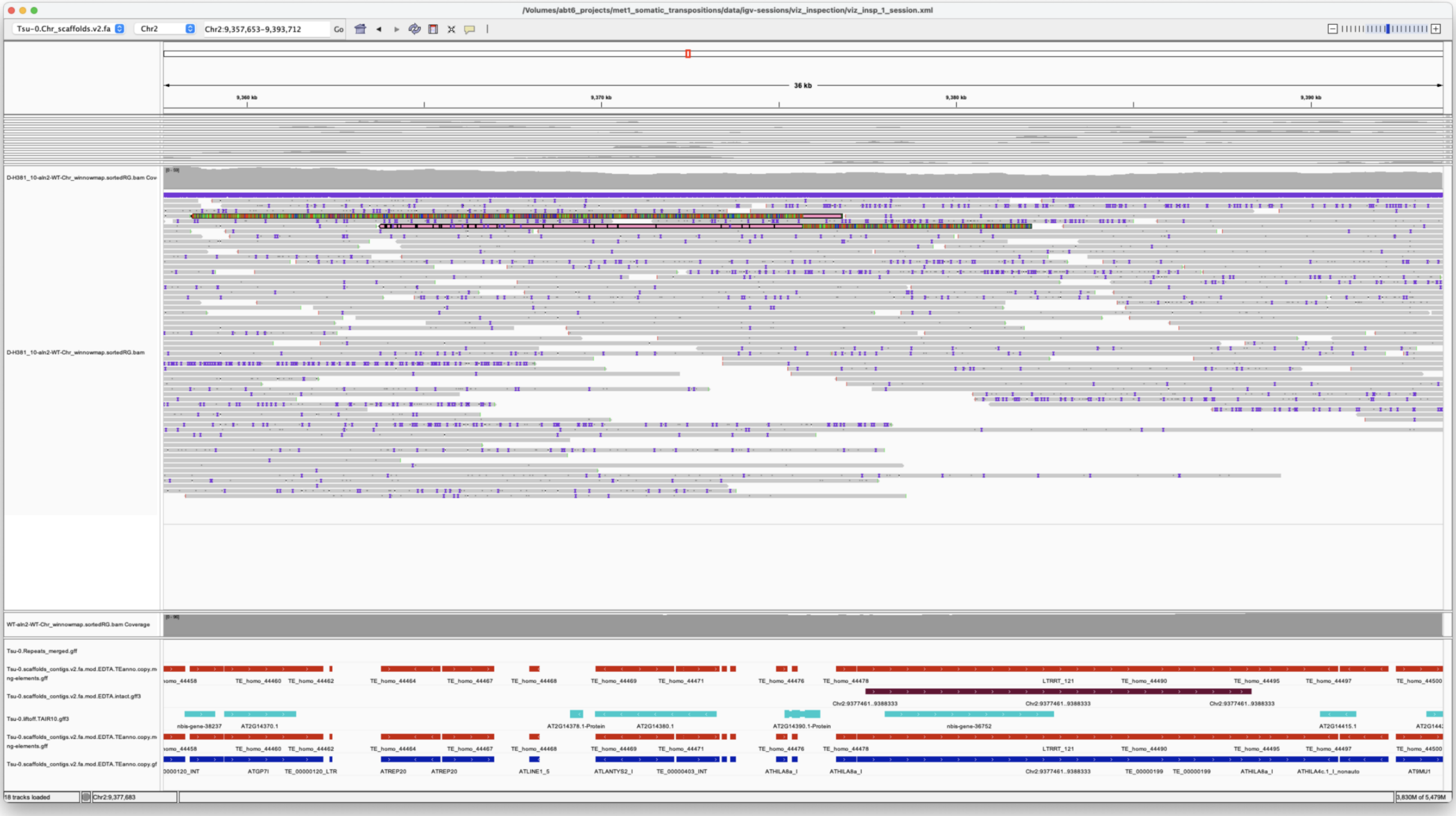

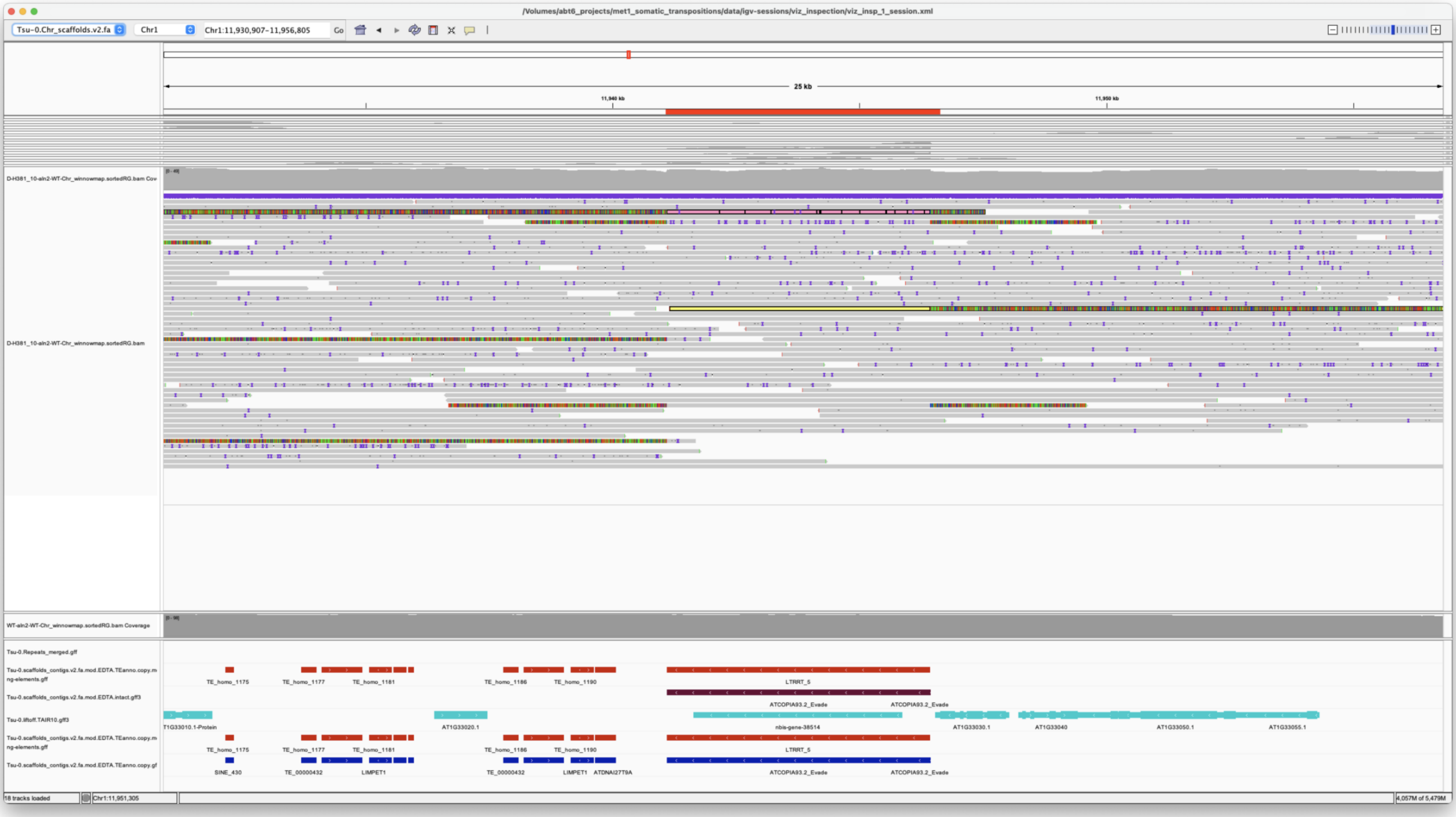

Central
TSD

Confirmed

Chr2 12740561 12740561 + 1 Chr1;11941106;11946436;ATCOPIA93_Evade m64079_221220_112036/102499387/ccs met1_10

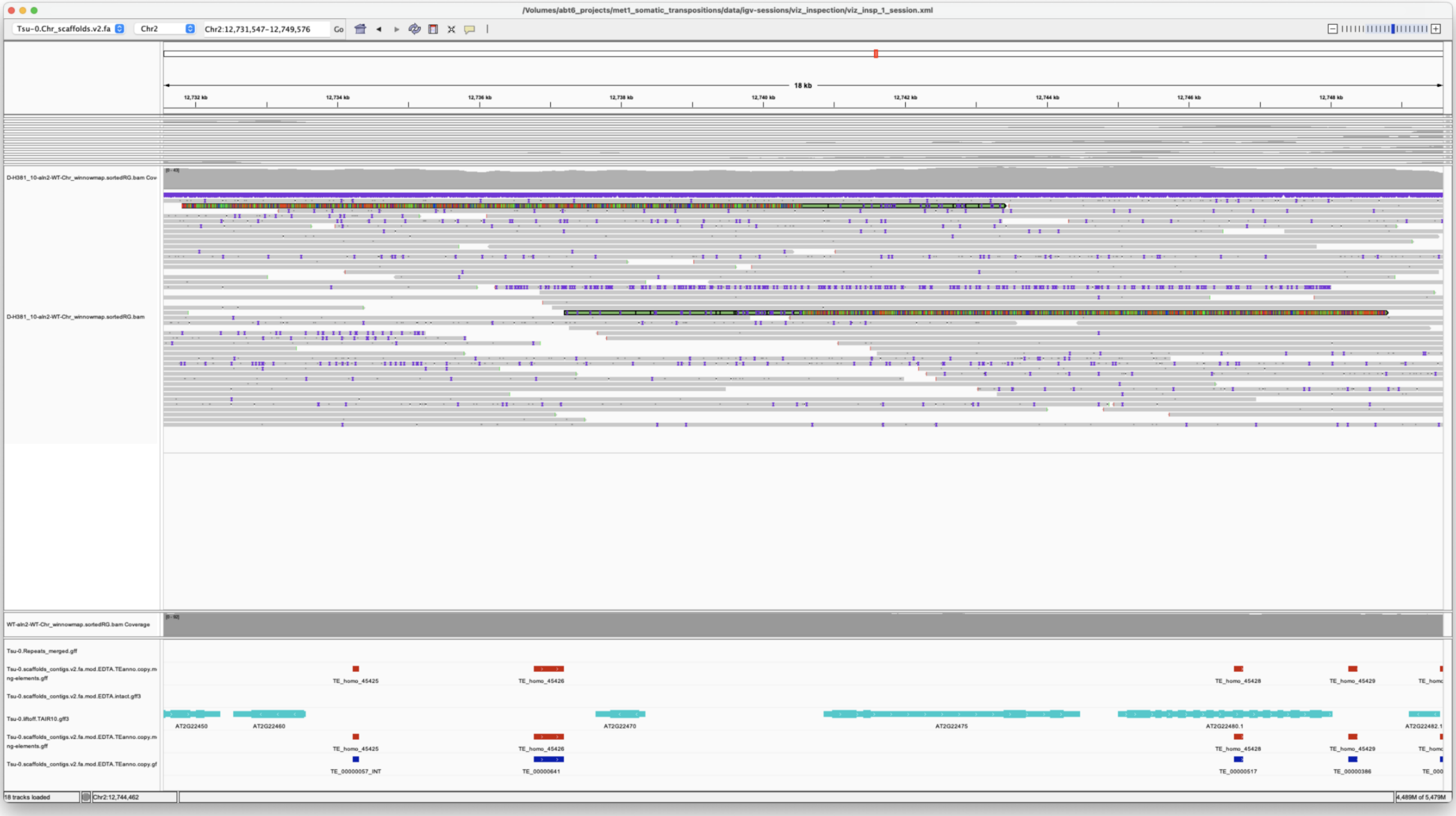

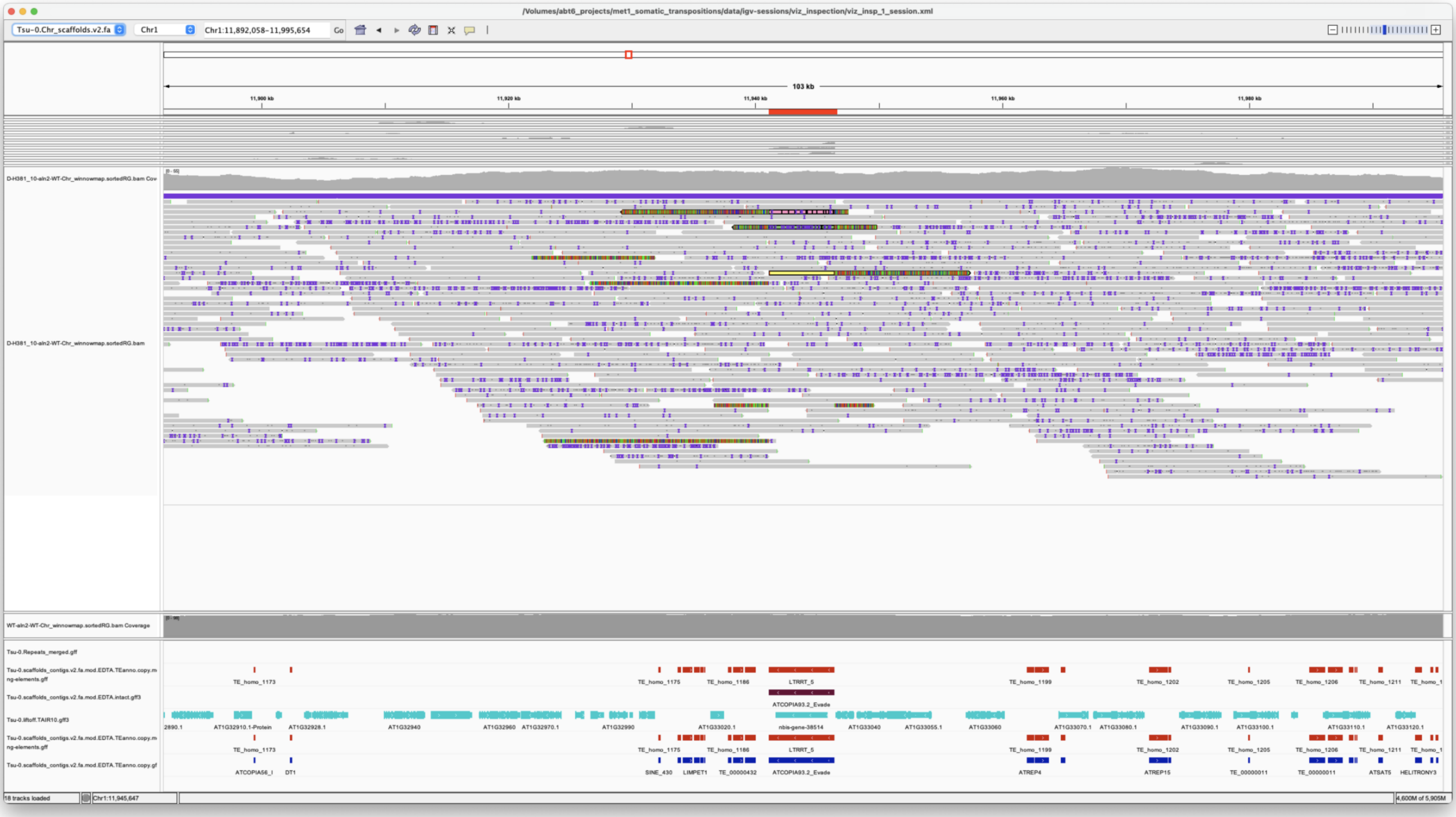

Central
TSD

Confirmed

Chr2 18907957 18907957 - 1 Chr1;11941106;11946436;ATCOPIA93_Evade m64079_221220_112036/35587392/ccs met1_10

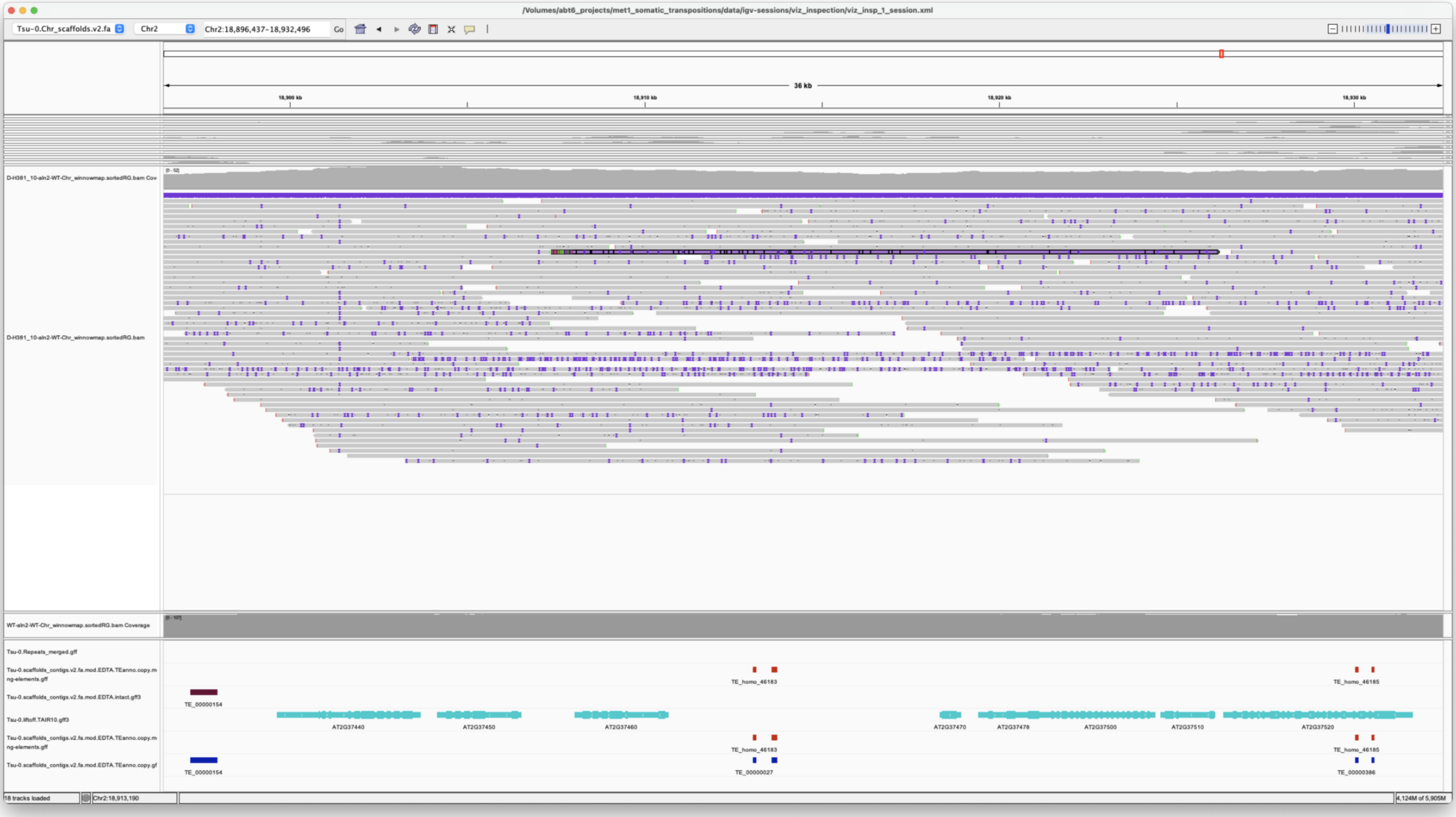

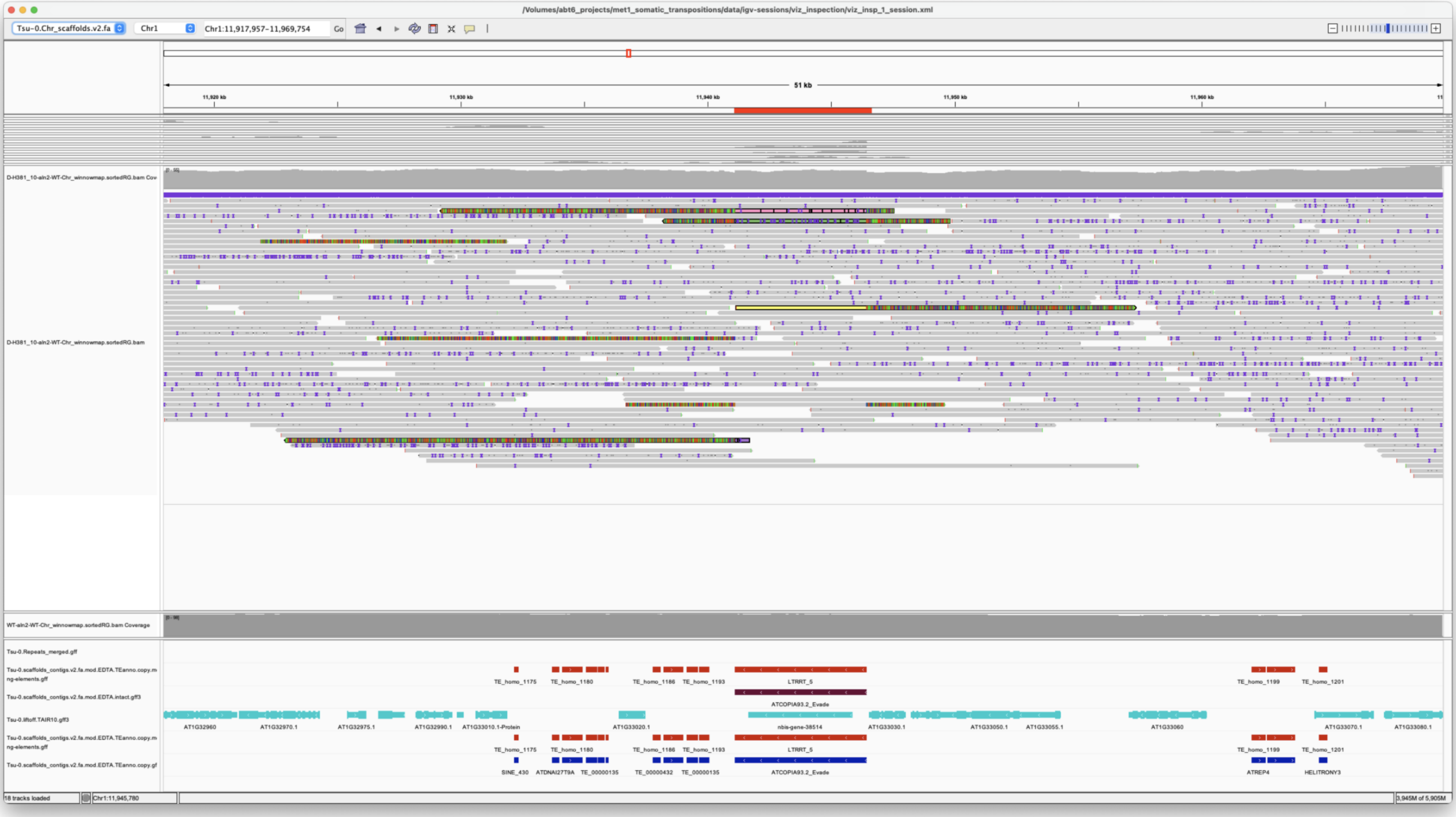

Partial

Confirmed

Chr3 143253 143253 + 1 Chr3;20158137;20166150;VANDAL6 m64079_221220_112036/14746100/ccs met1_10

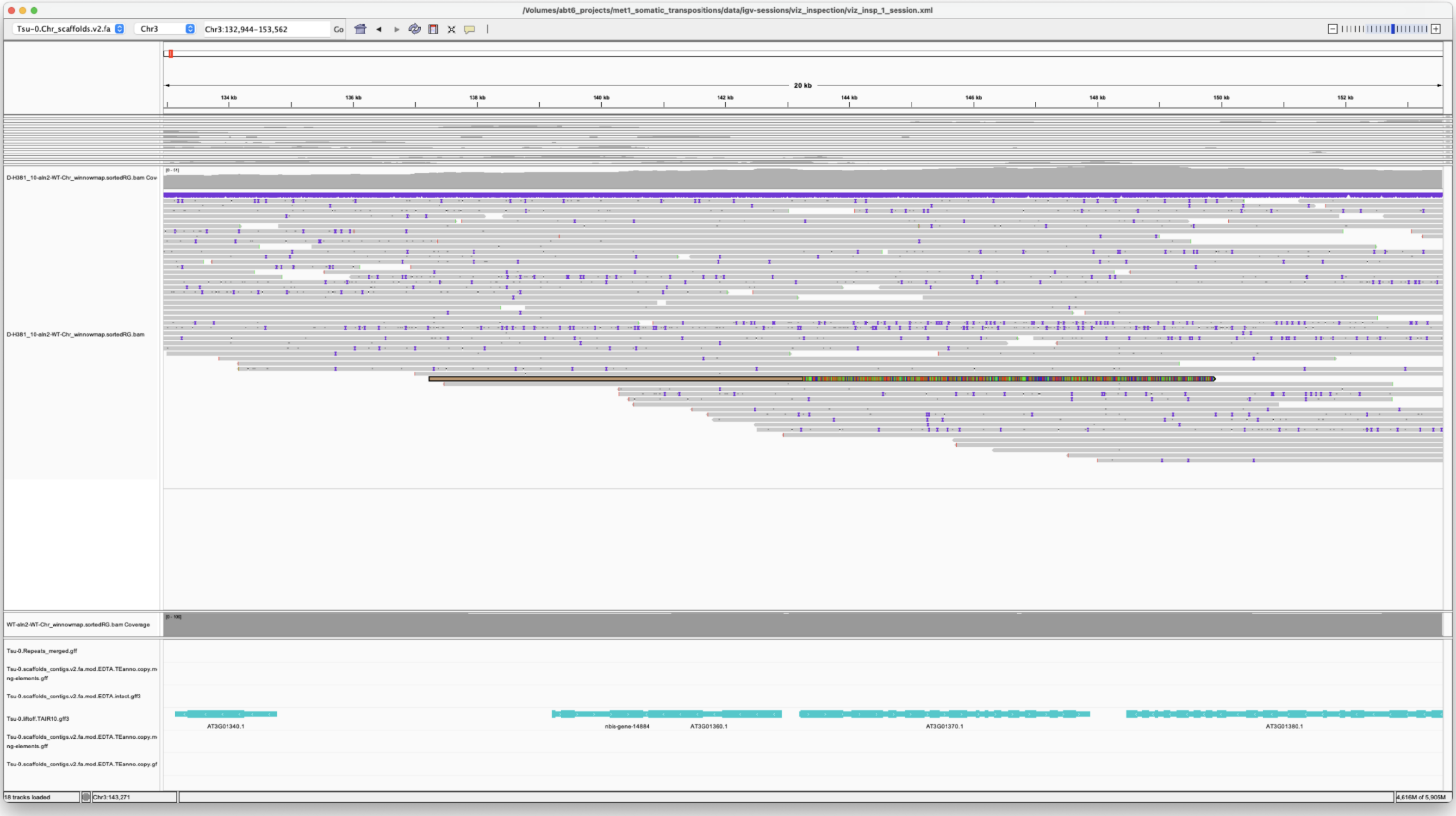

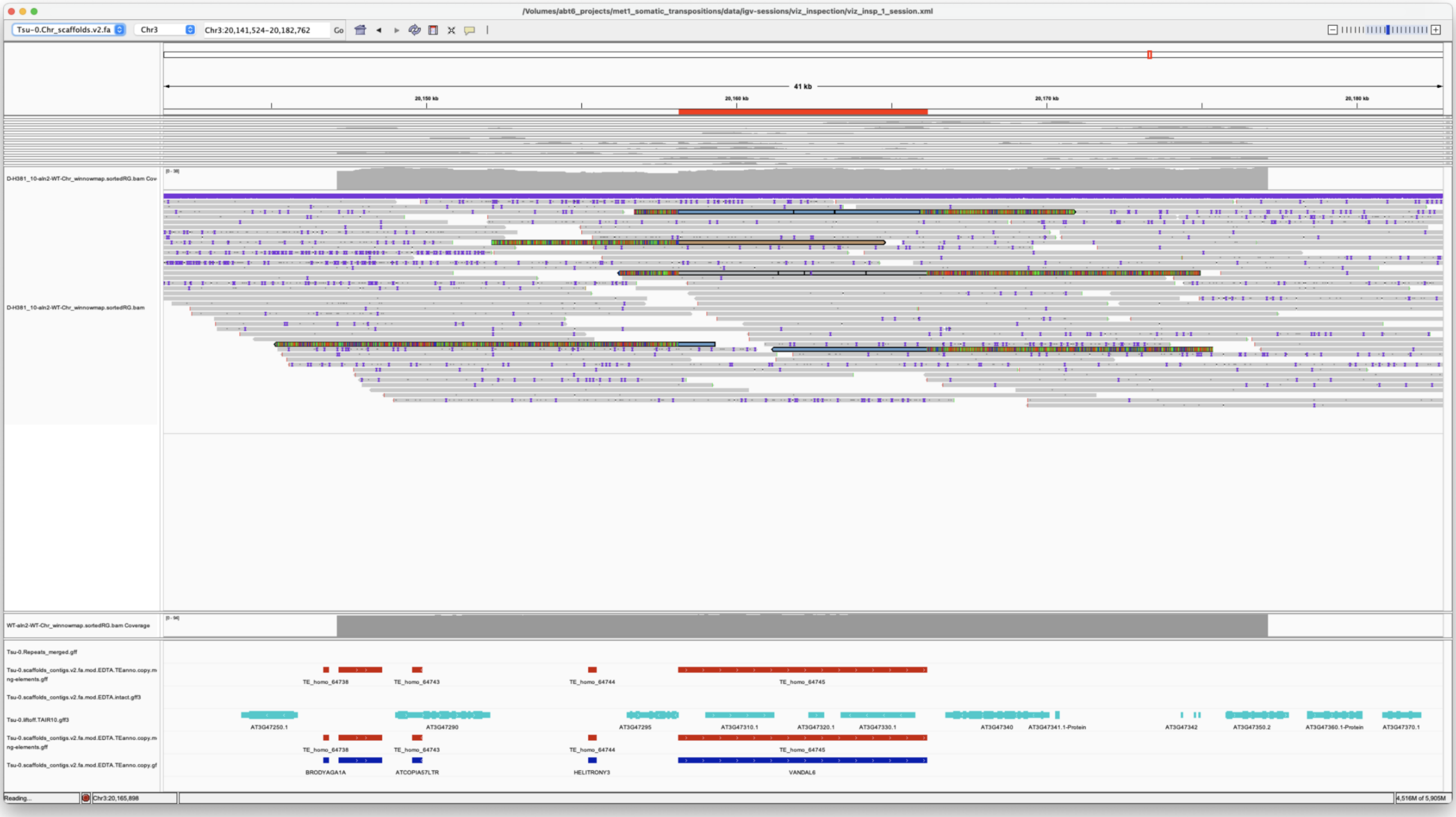

Partial

Confirmed

Chr3 24790888 24790888 + 1 Chr1;11941106;11946436;ATCOPIA93_Evade m64079_221220_112036/170920651/ccs met1_10

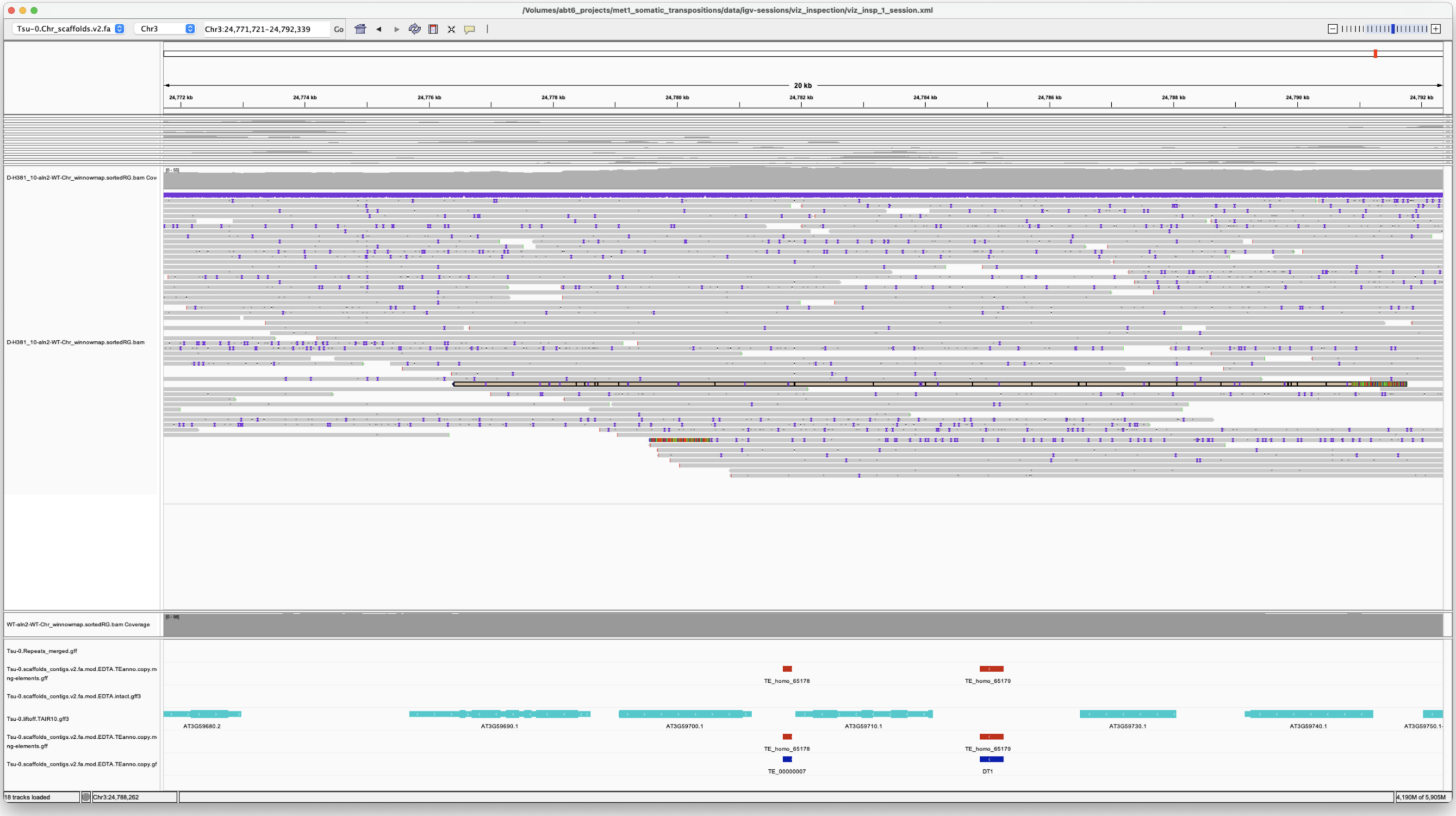

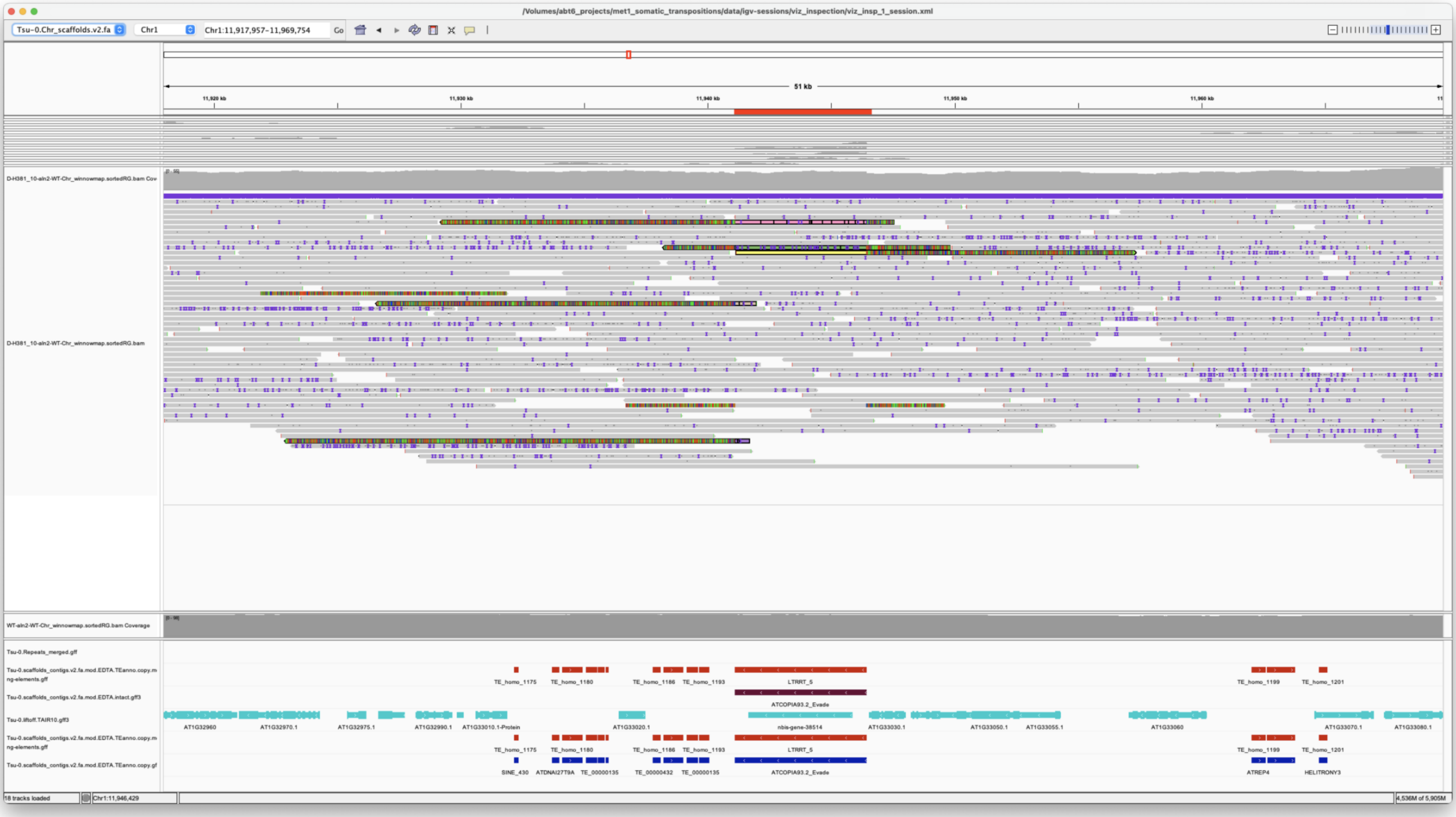

Partial

Confirmed

Chr3 24959115 24959115 - 1 Chr3;16344522;16352497;VANDAL6 m64079_240212_113350/19138681/ccs met1_10

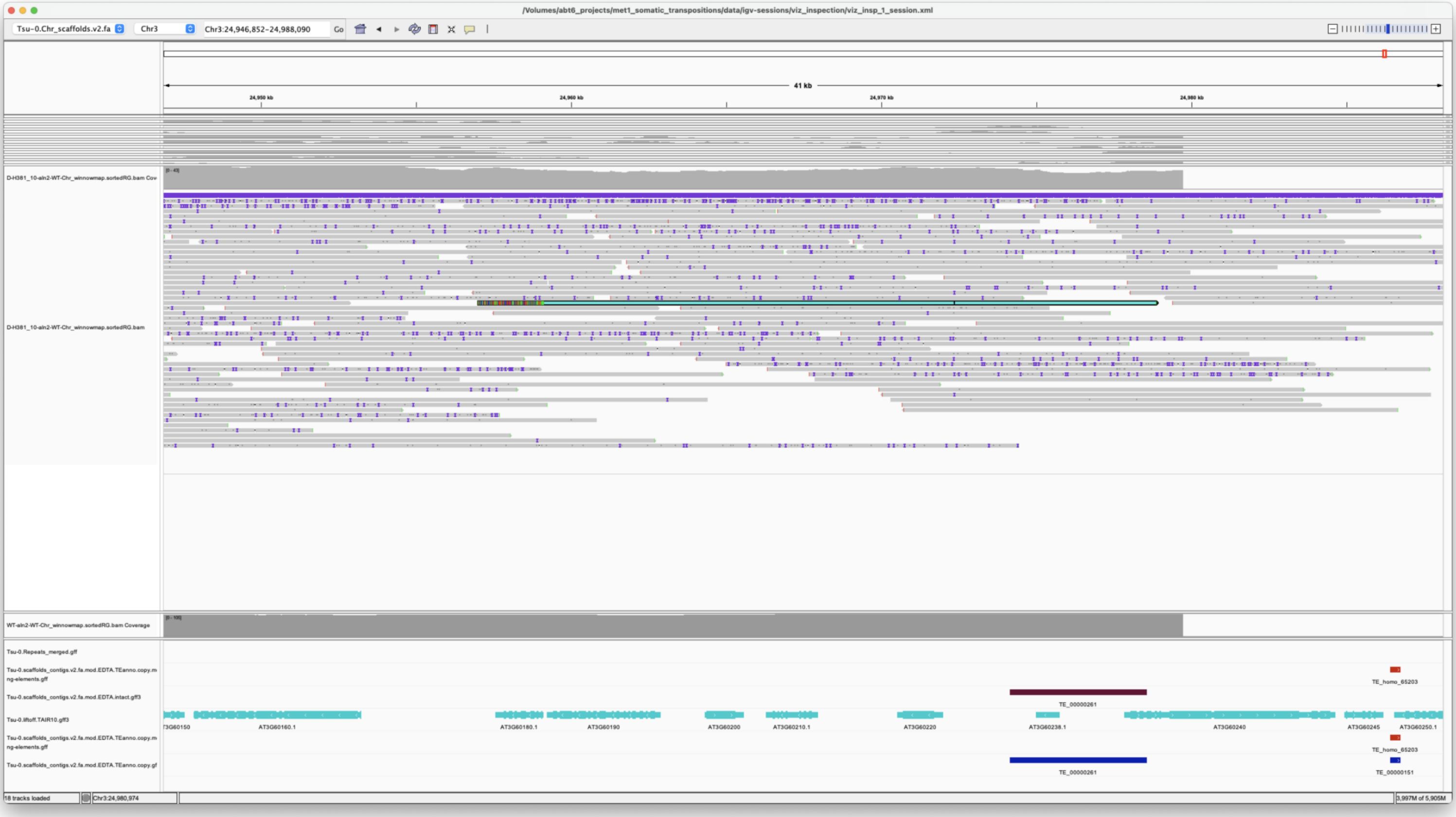

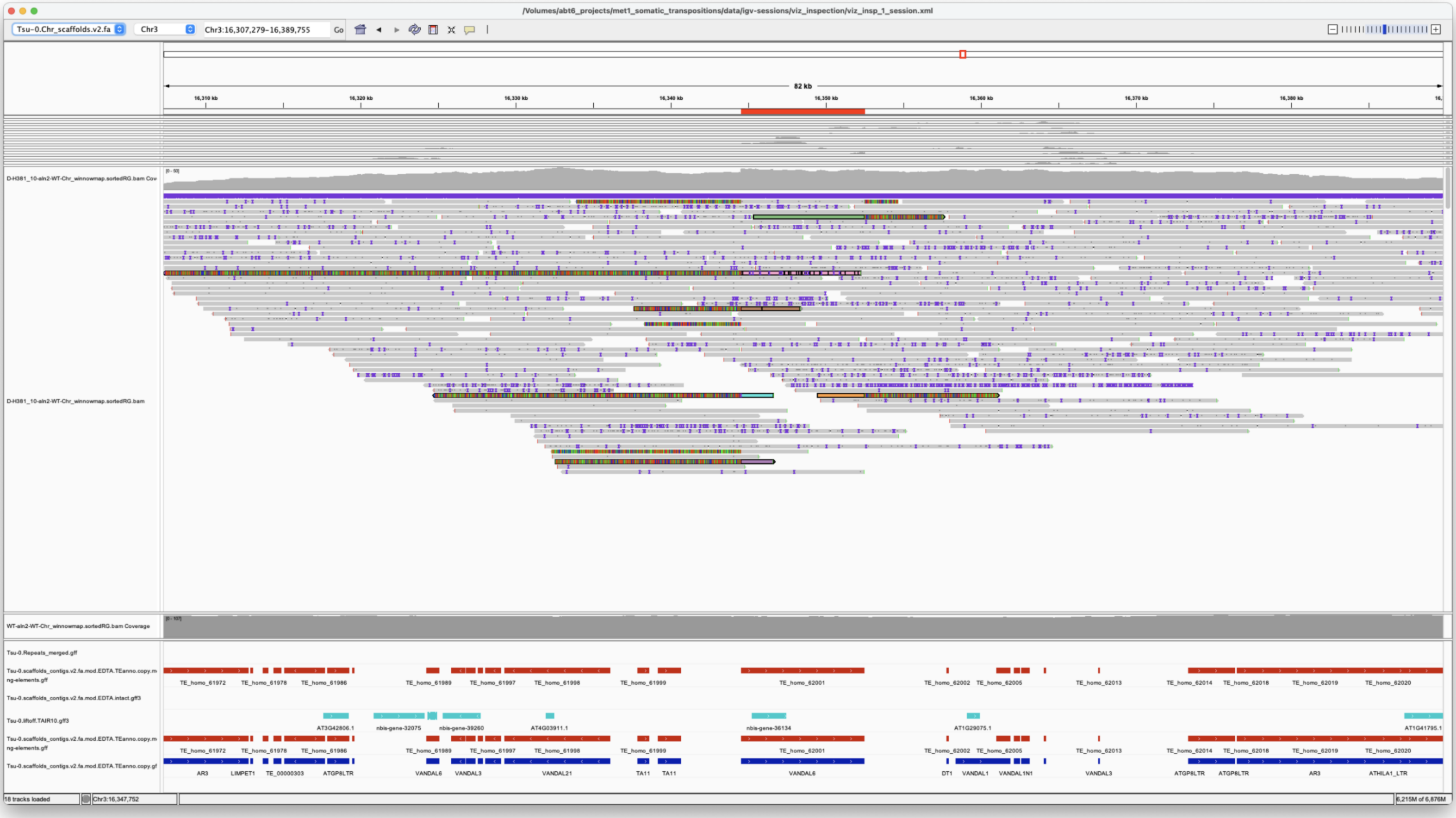

Partial

Confirmed

Chr4 2752044 2752044 - 1 Chr3;16344522;16352497;VANDAL6 m64079_221220_112036/154208565/ccs met1_10

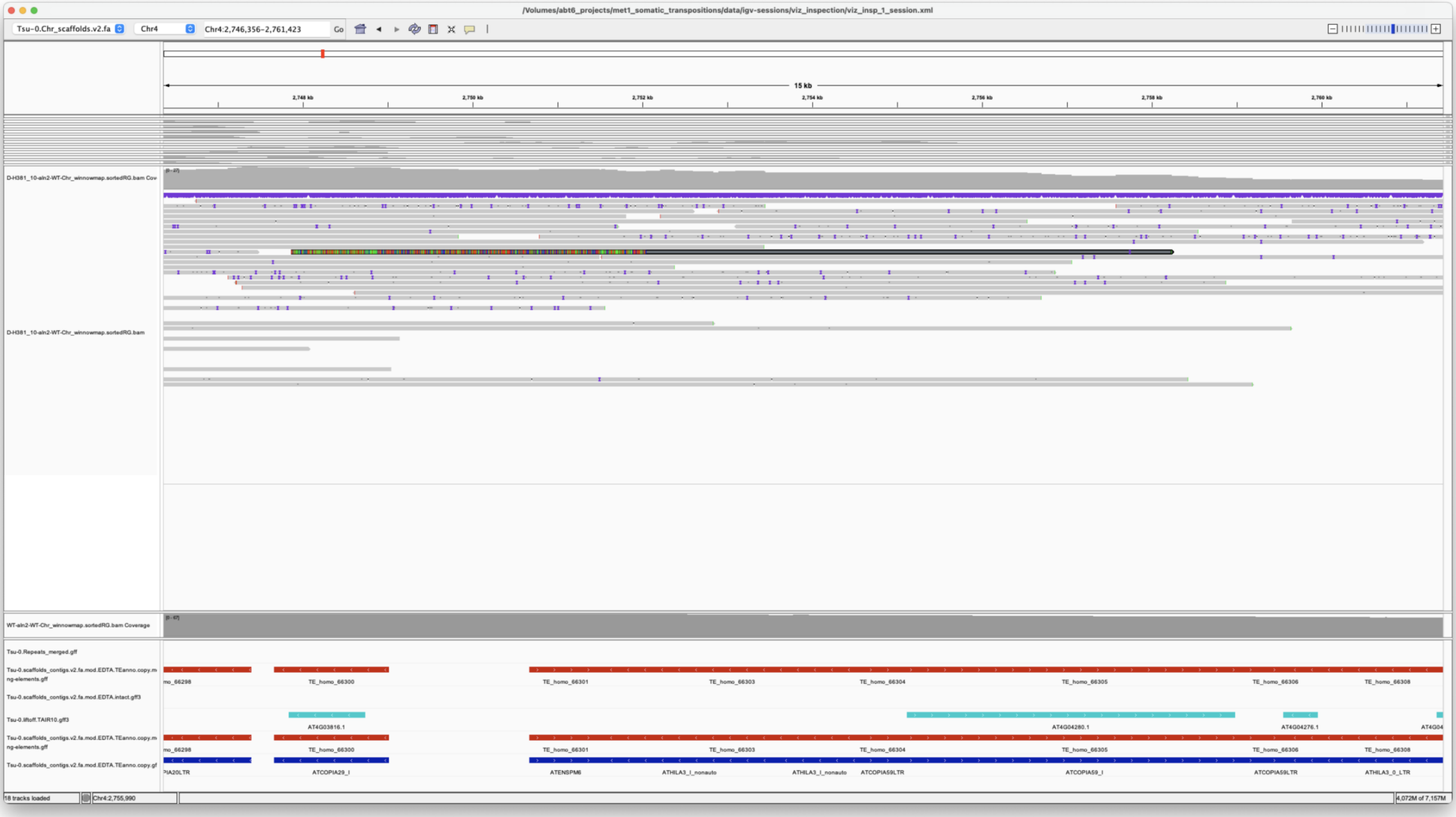

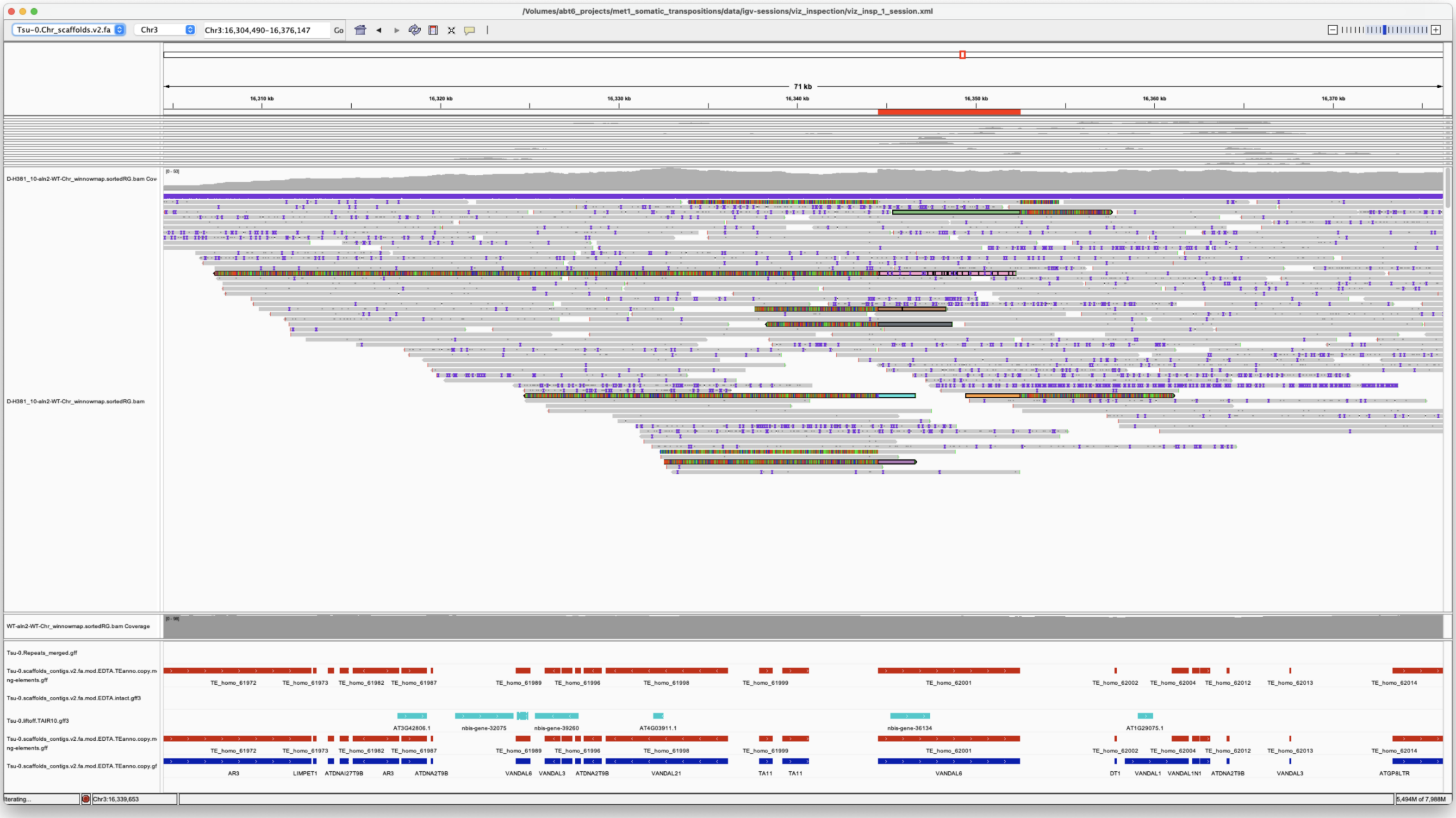

Partial

Confirmed

Chr4 2943152 2943152 + 1 Chr5;19152829;19160826;VANDAL21 m64079_221220_112036/172949806/ccs met1_10

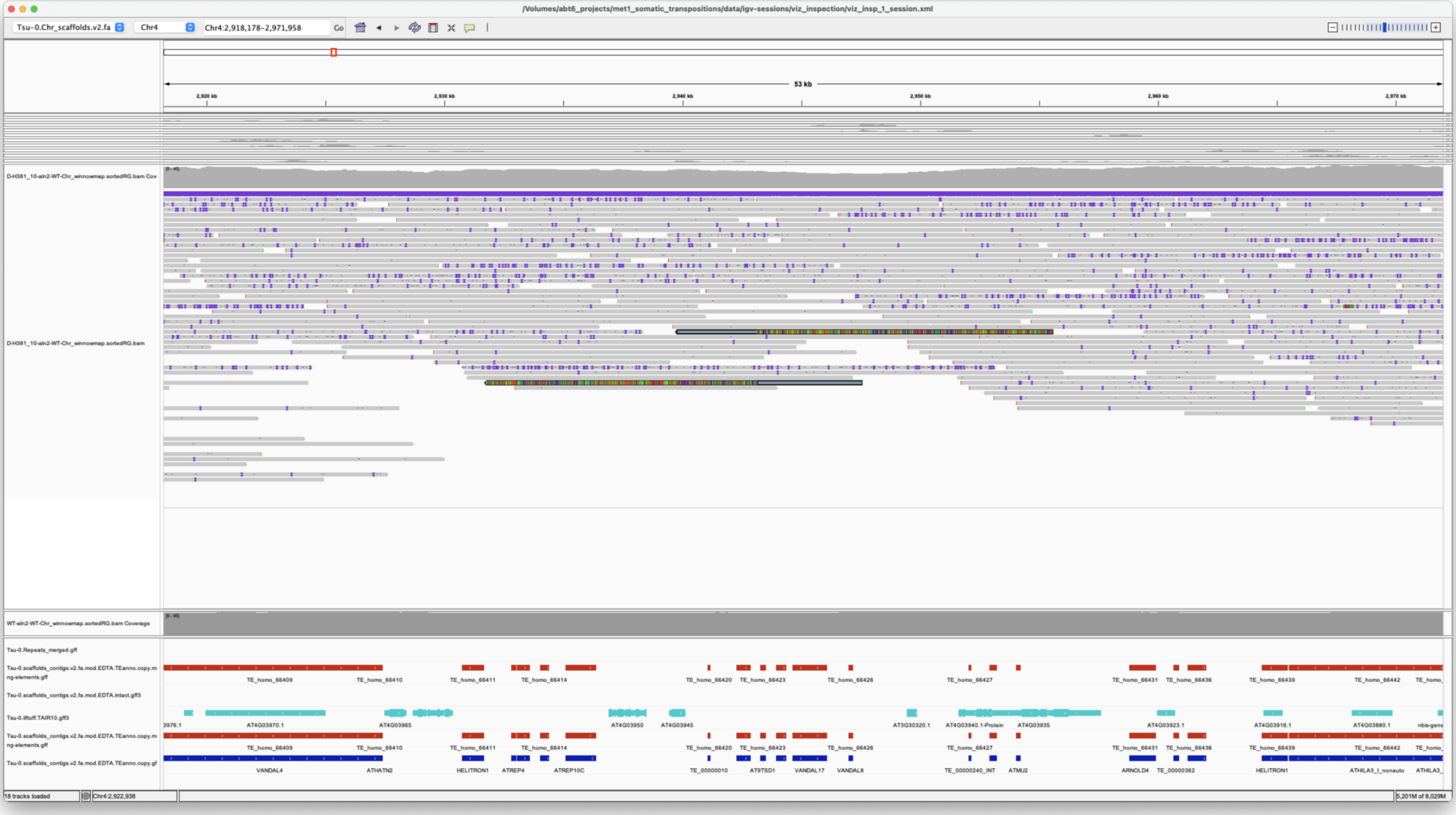

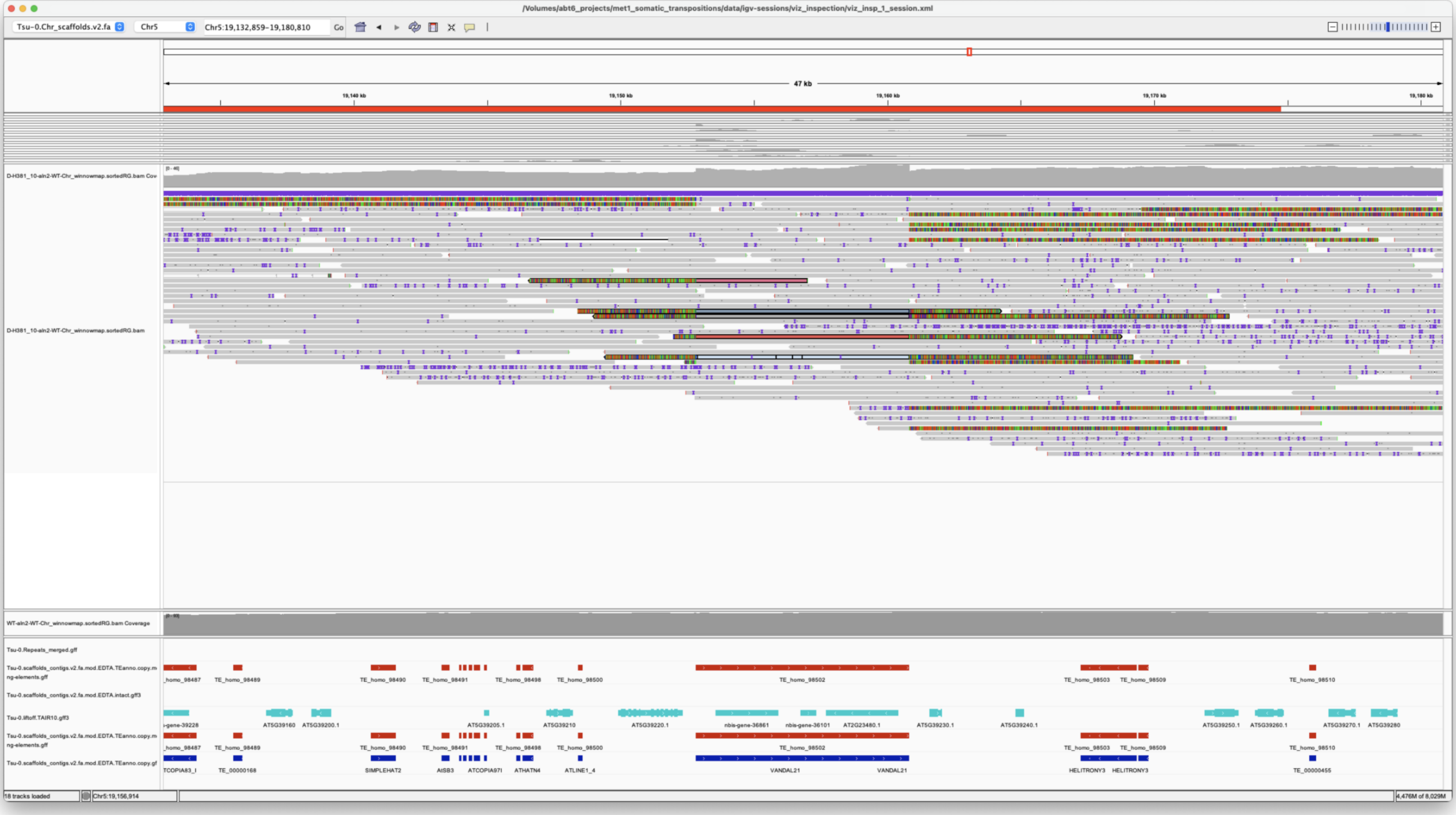

Central
TSD

Confirmed

Chr4 3242108 3242108 - 1 Chr5;19152829;19160826;VANDAL21 m64079_221220_112036/147587175/ccs met1_10

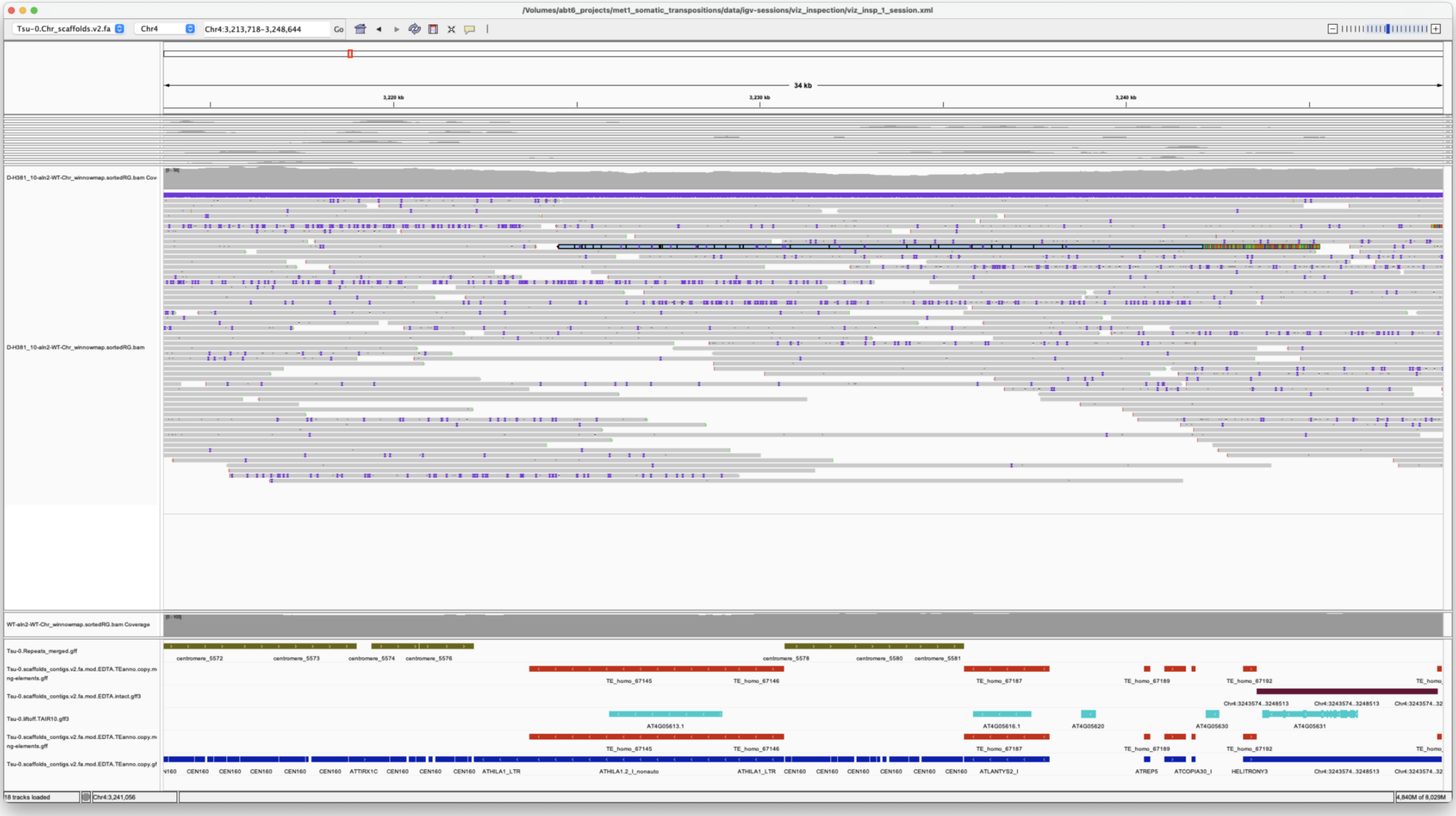

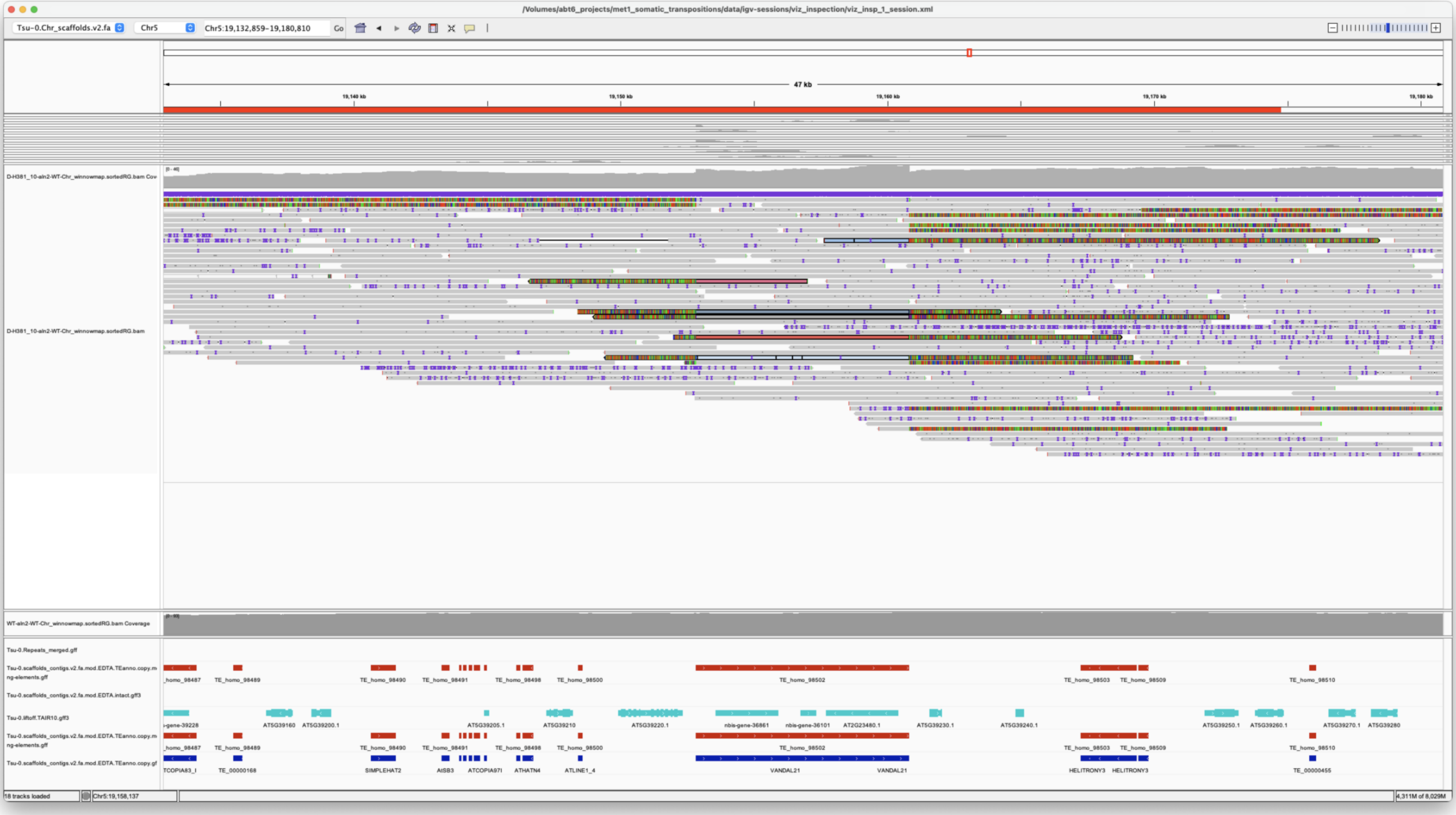

Partial

Confirmed

Chr4 3248337 3248337 - 1 Chr5;19152829;19160826;VANDAL21 m64079_221220_112036/917628/ccs met1_10

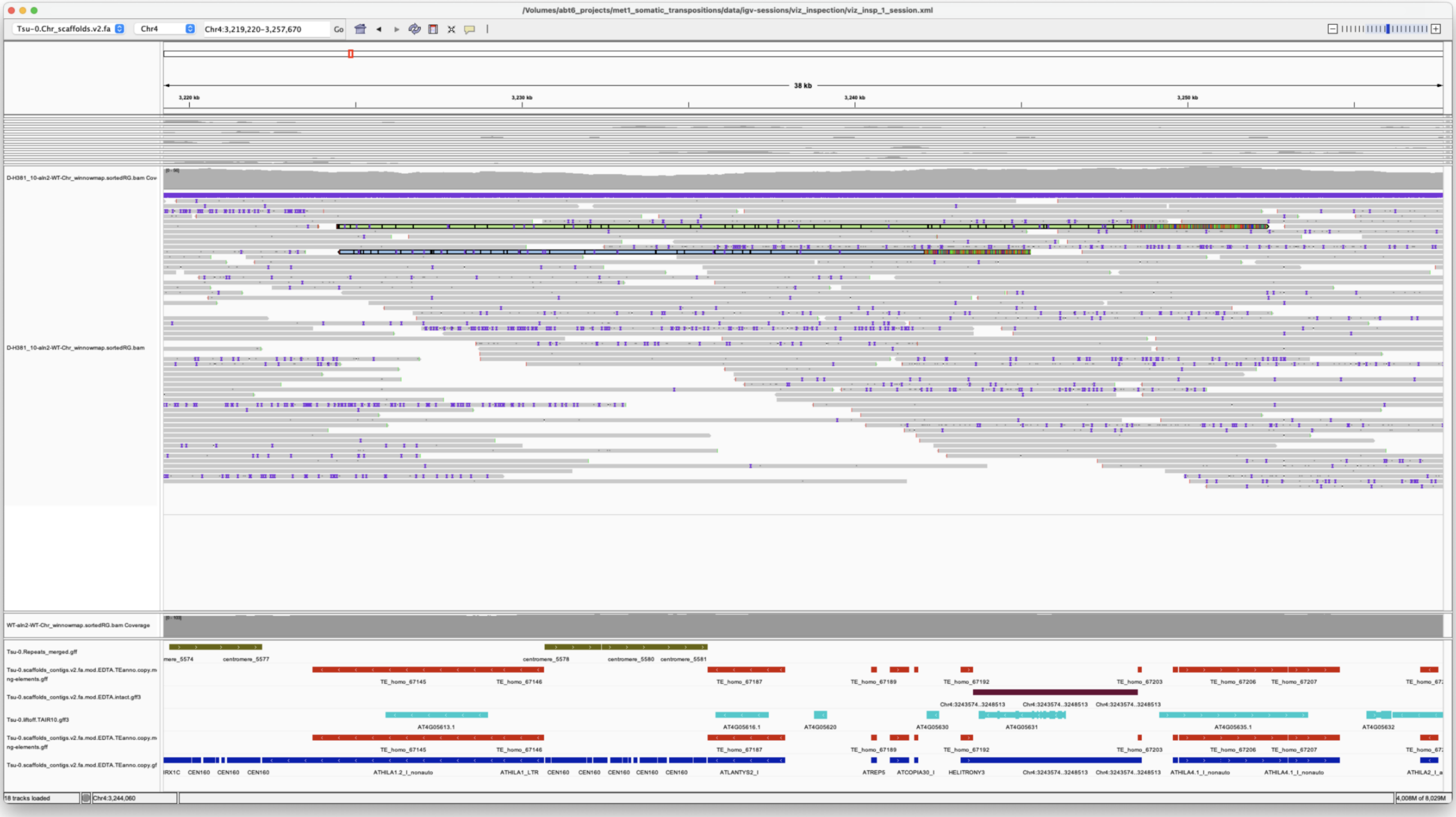

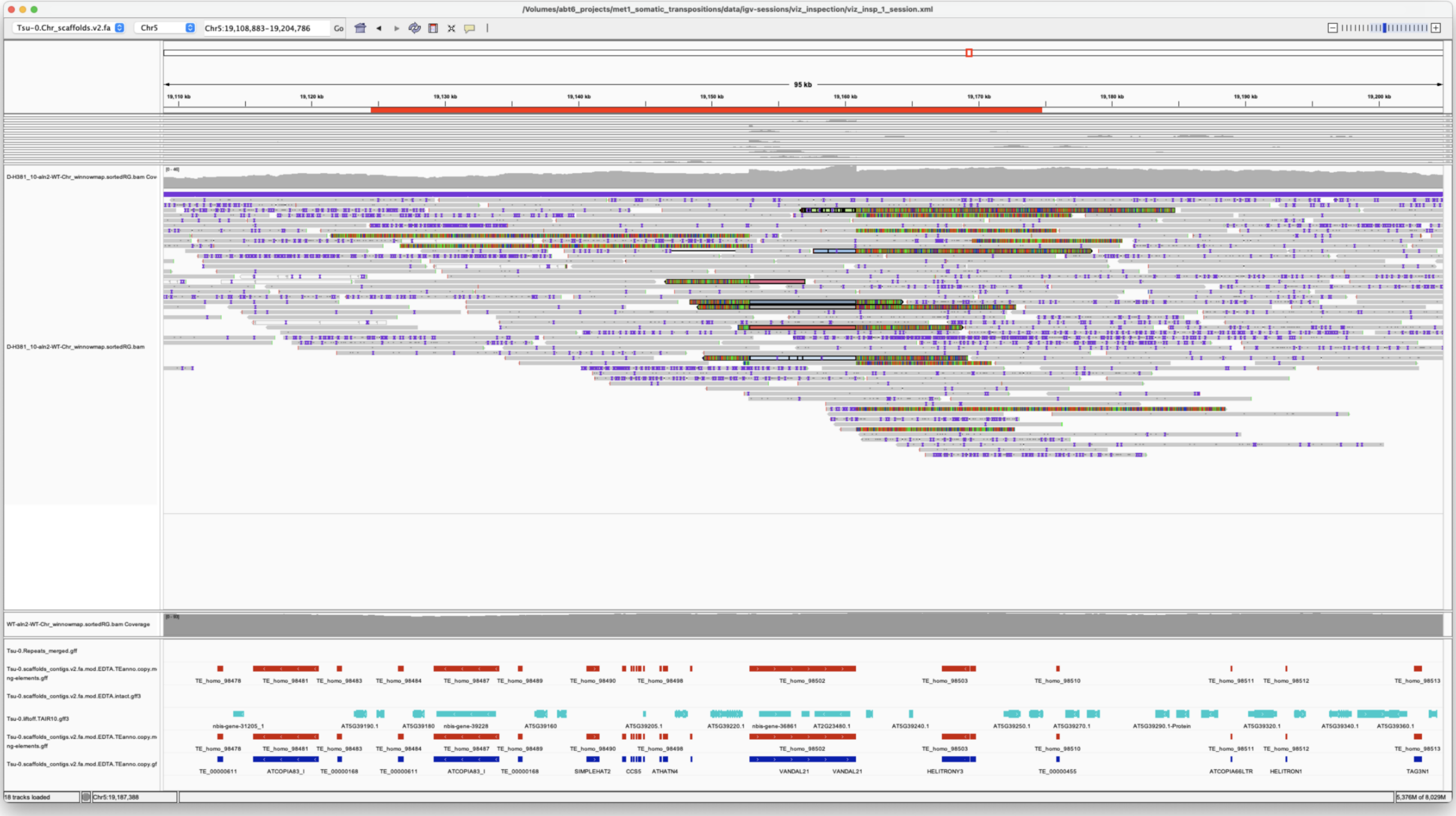

Most probably two independent VANDAL21 insertions close to each other

Partial

Confirmed

Chr4 10494251 10494251 - 1 Chr5;19152829;19160826;VANDAL21 m64079_221220_112036/131074130/ccs met1_10

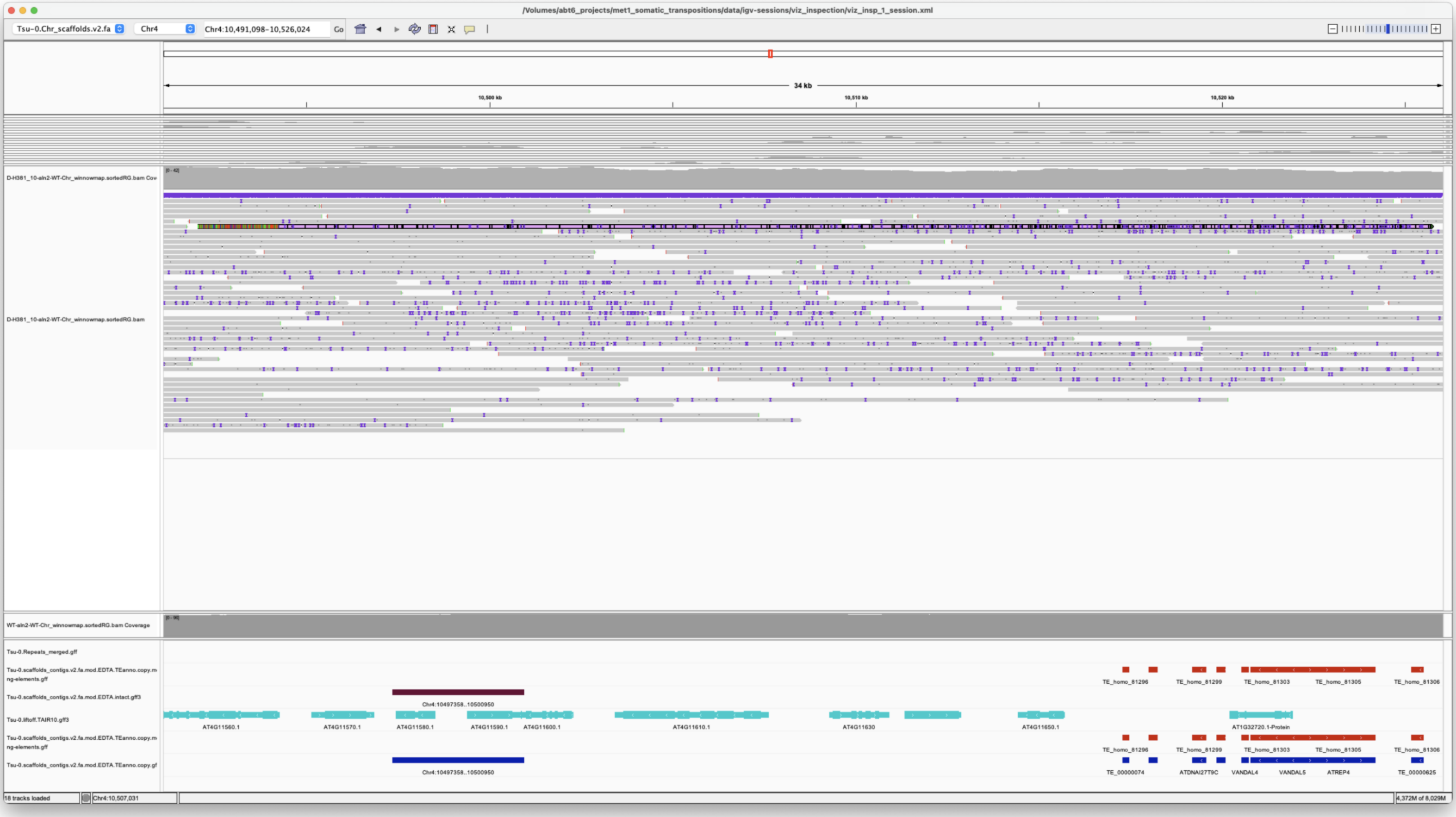

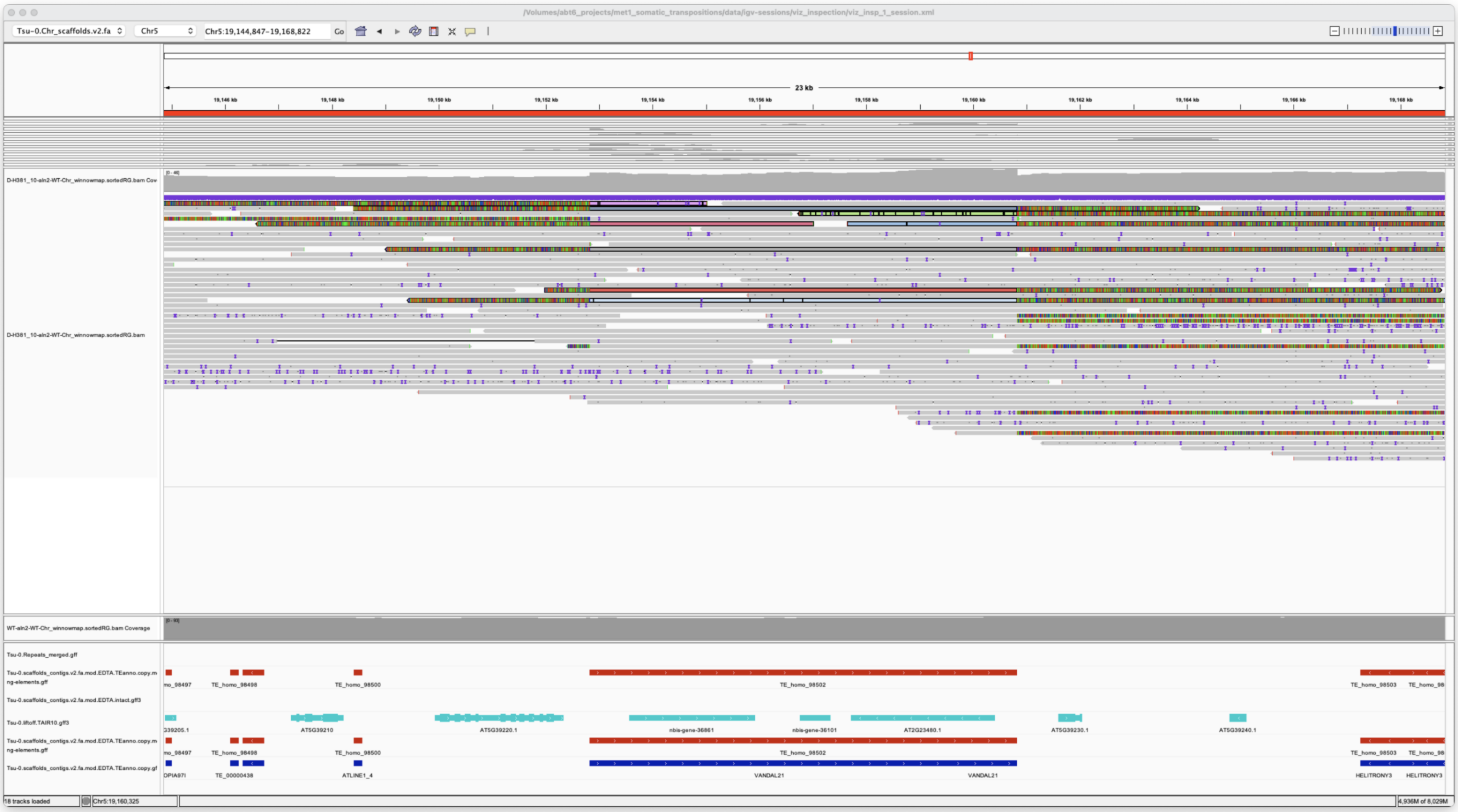

Partial

Confirmed

Chr4 11260296 11260296 - 1 Chr5;19152829;19160826;VANDAL21 m64079_221220_112036/47711675/ccs met1_10

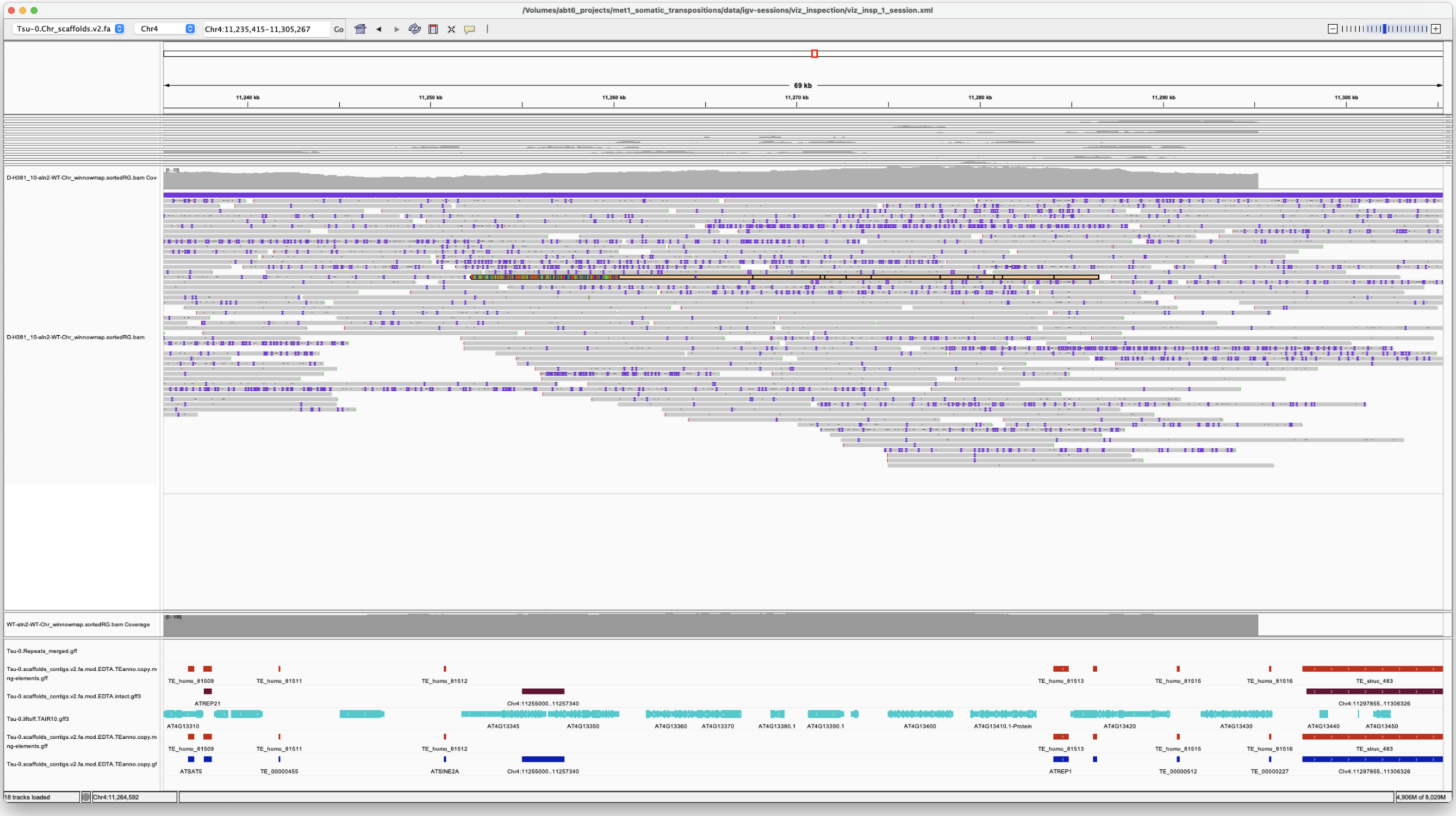

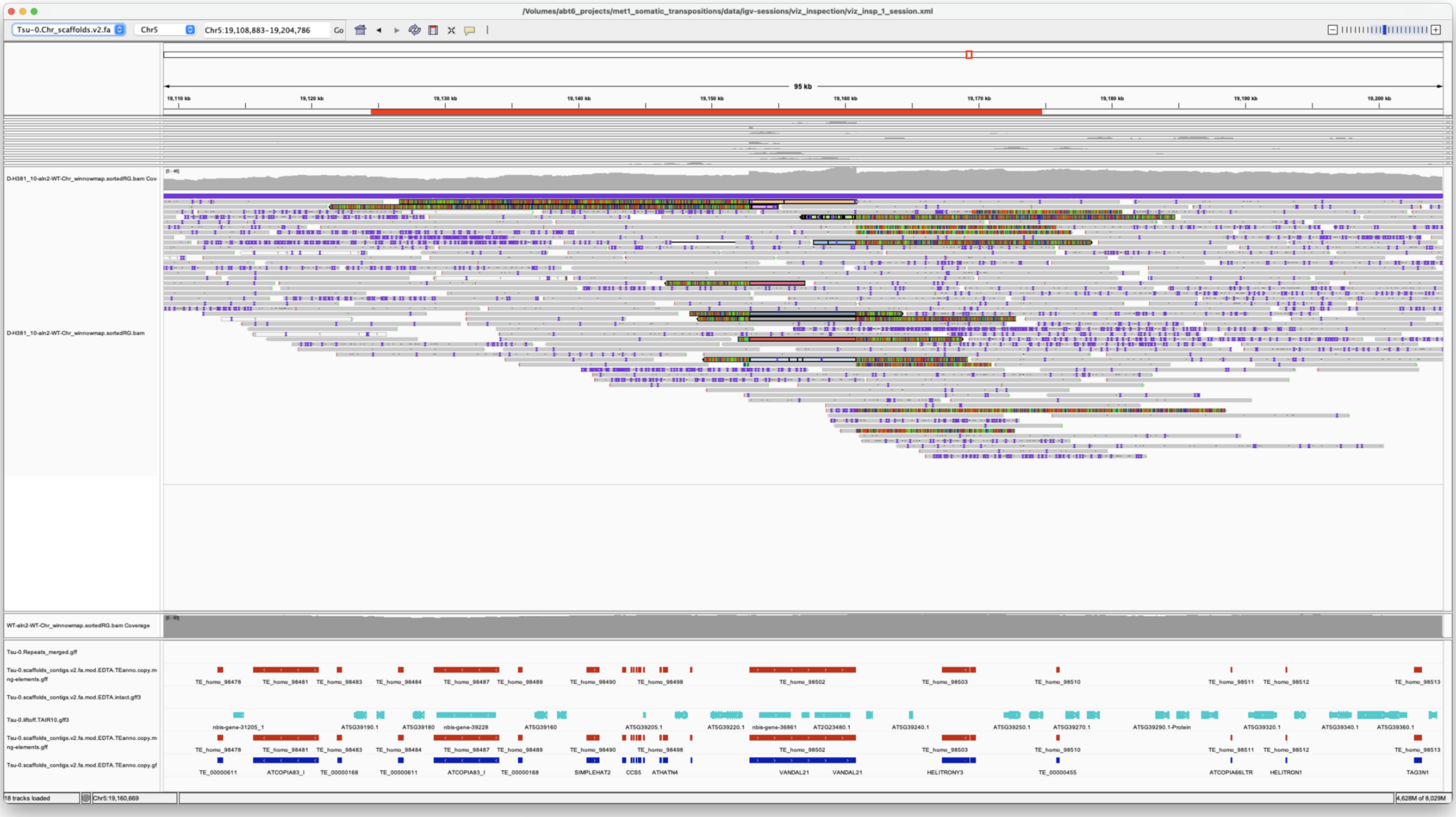

Partial

Confirmed

Chr4 12519914 12519914 + 1 Chr5;19152829;19160826;VANDAL21 m64079_221220_112036/148965309/ccs met1_10

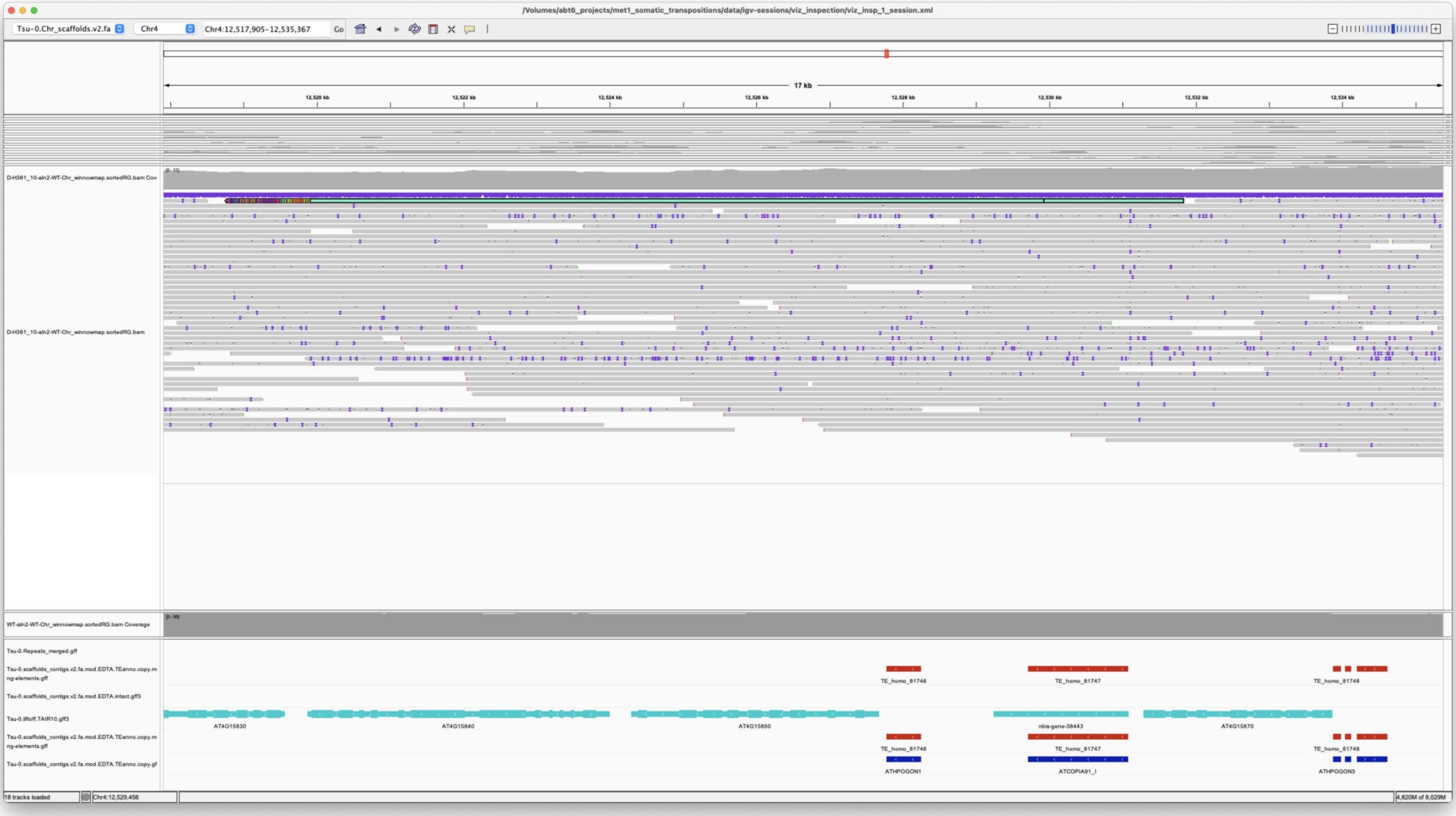

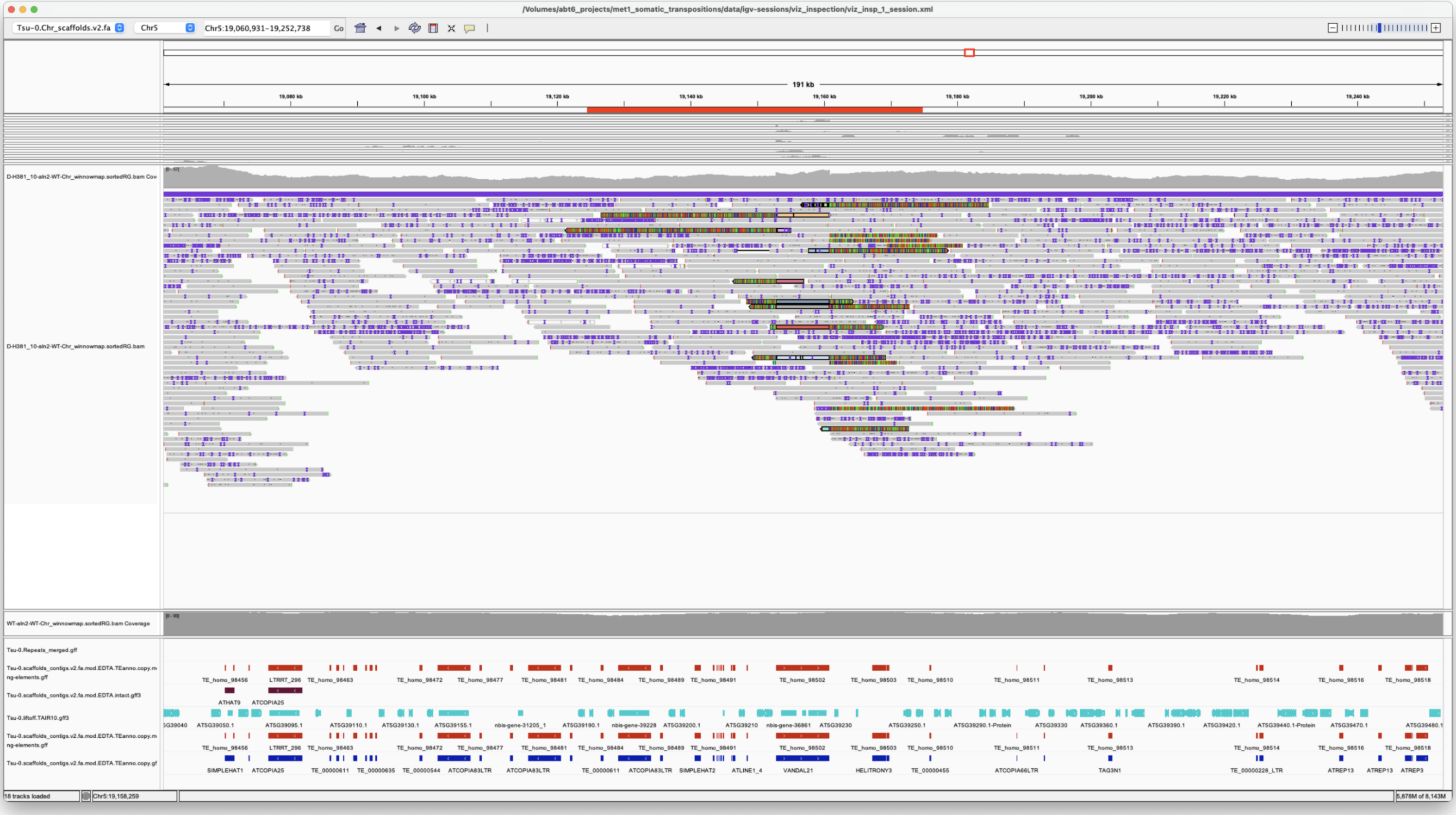

Partial

Confirmed

Chr4 16408459 16408459 + 1 Chr3;16344522;16352497;VANDAL6 m64079_221220_112036/77595626/ccs met1_10

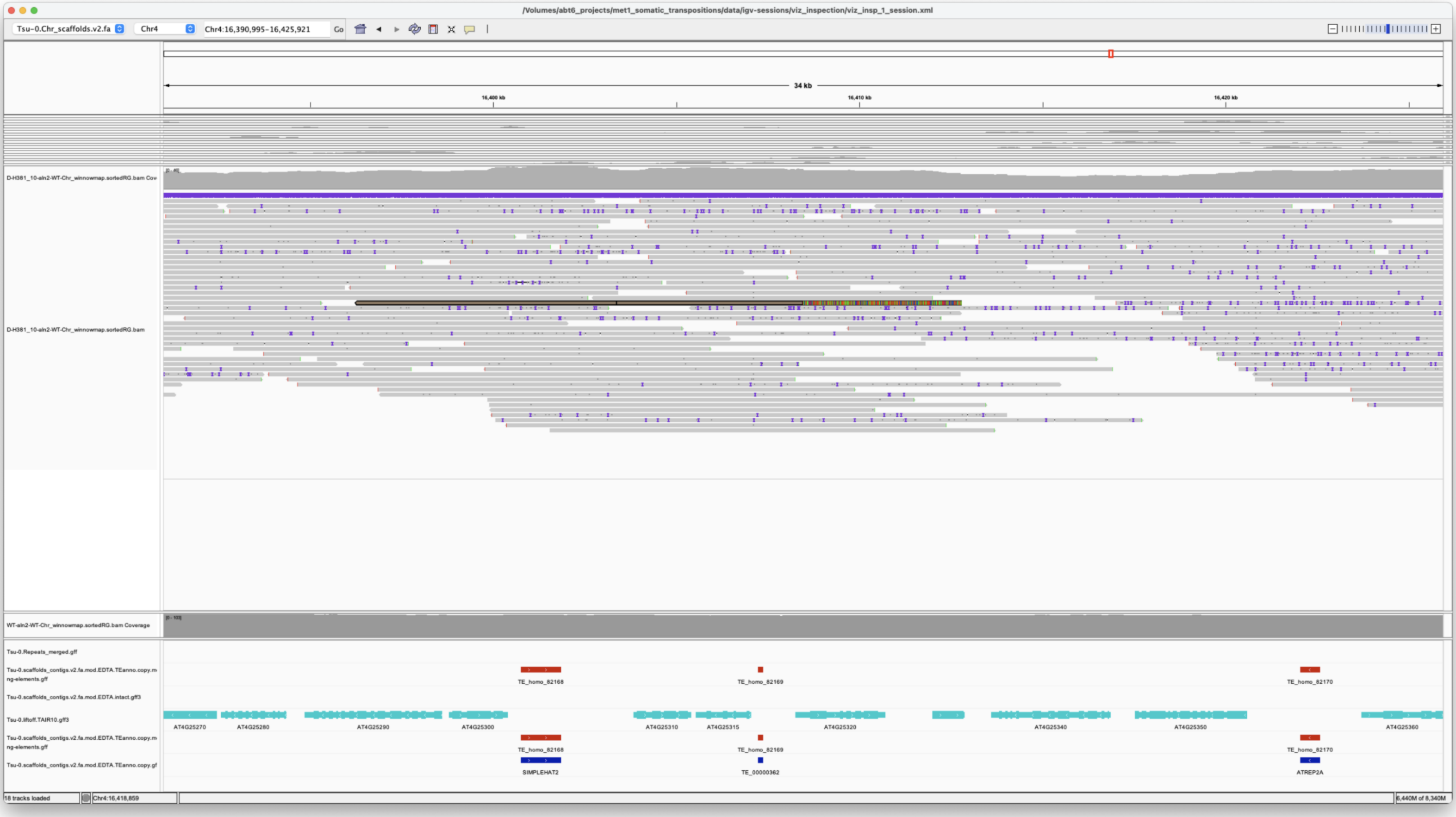

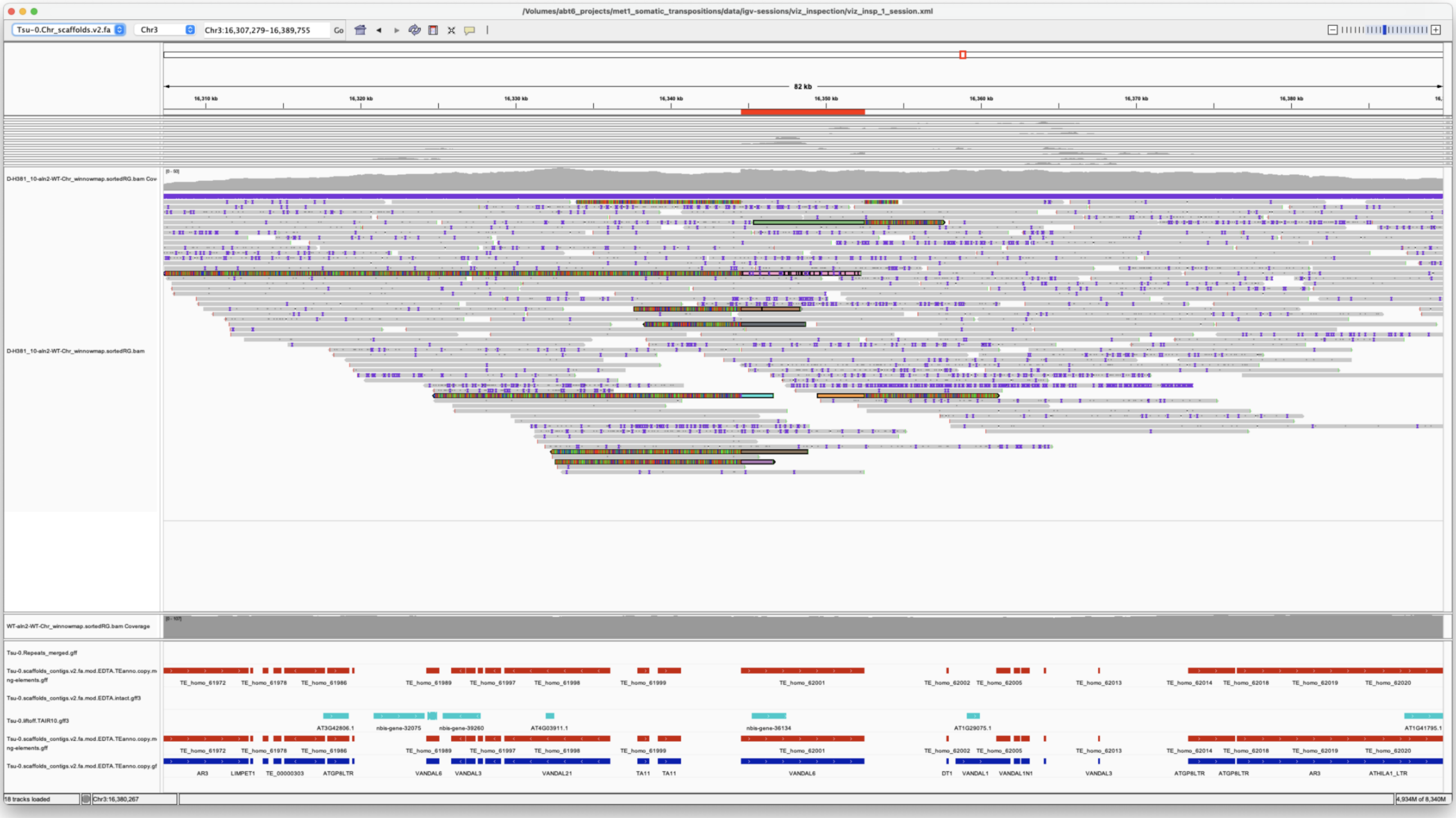

Partial

Confirmed

Chr4 20938887 20938887 - 1 Chr1;11941106;11946436;ATCOPIA93_Evade m64079_221220_112036/154403691/ccs met1_10

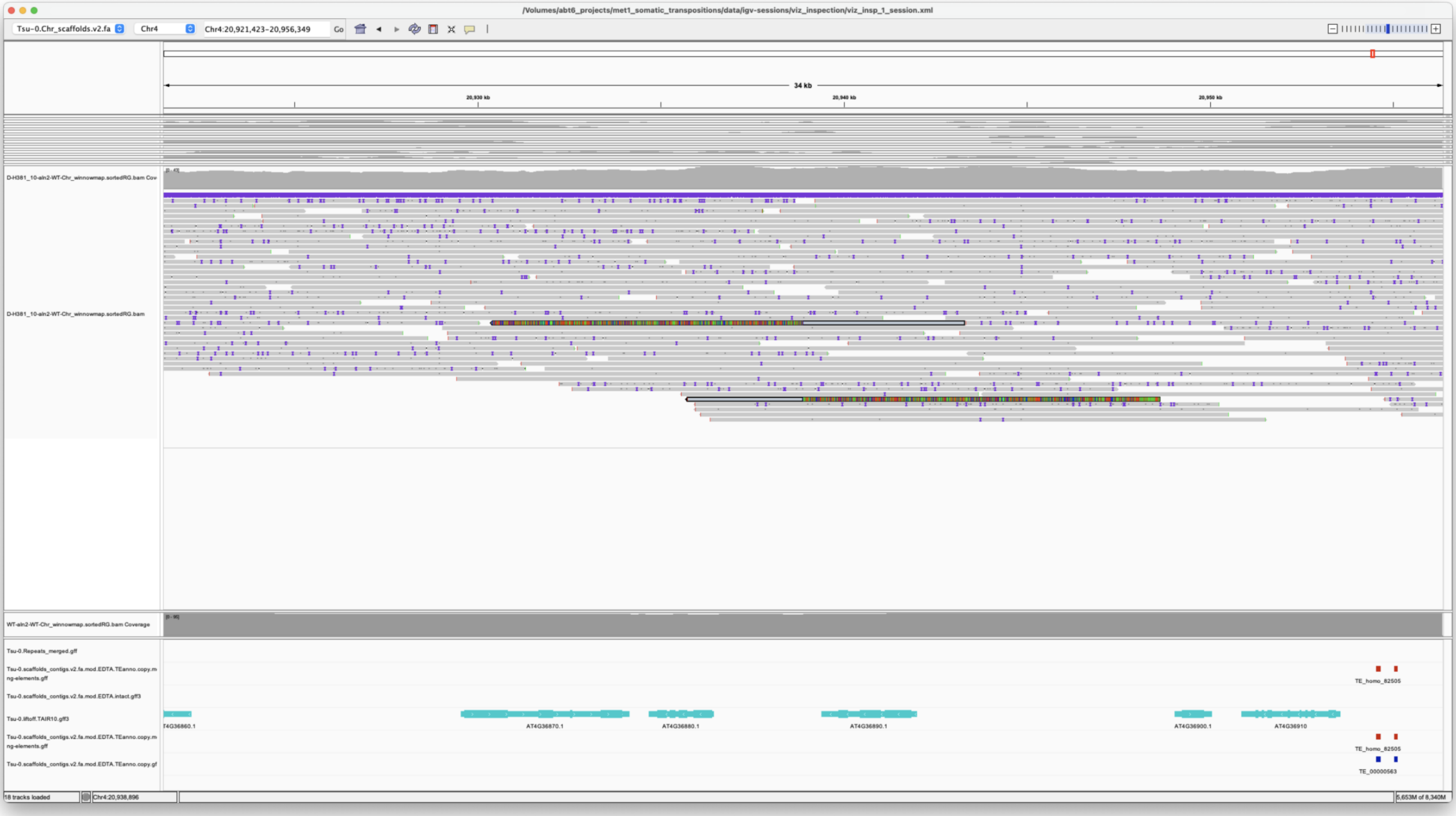

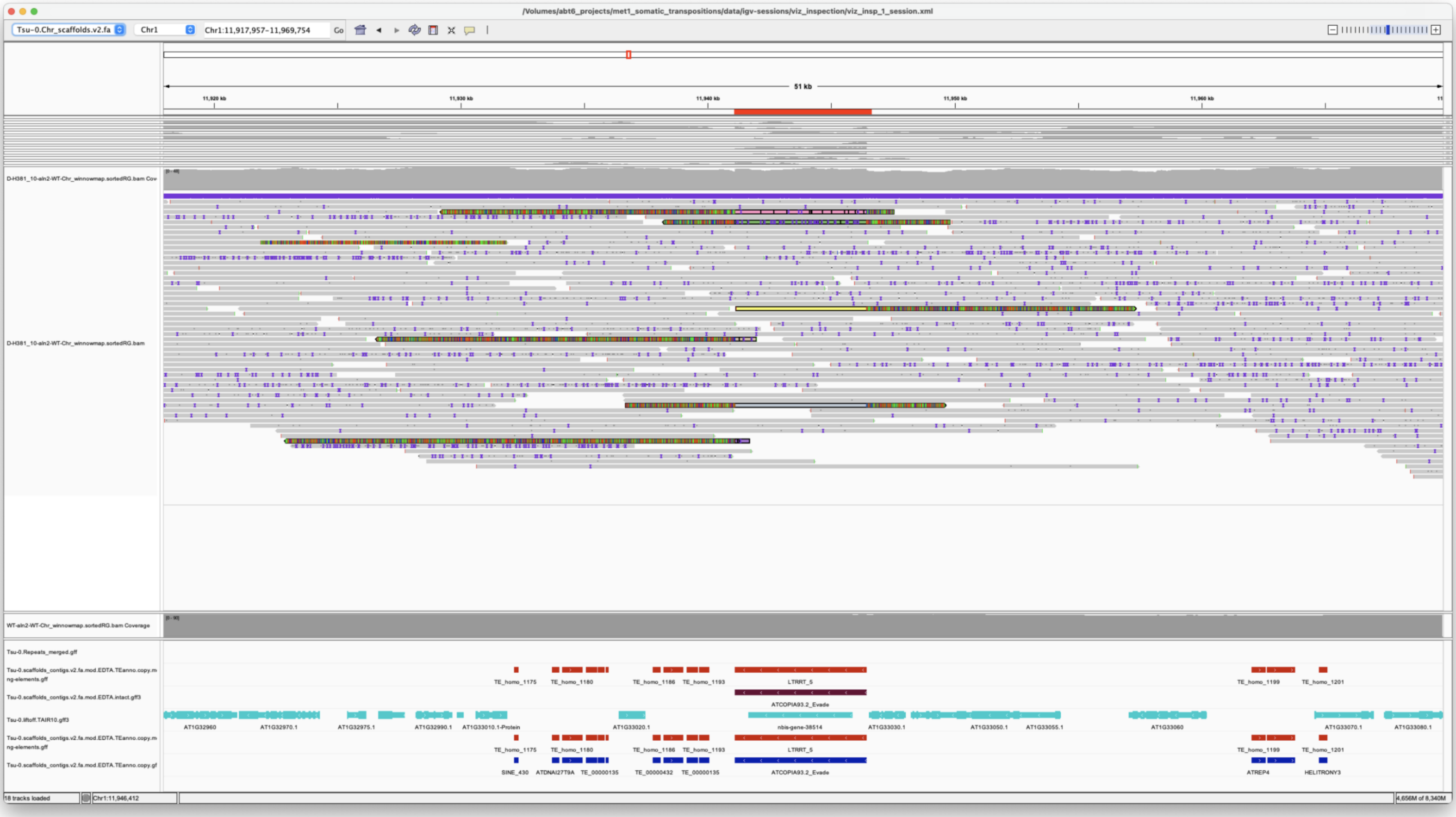

Central
TSD

Confirmed

Chr4 21486823 21486823 + 1 Chr3;16344522;16352497;VANDAL6 m64079_221220_112036/90048082/ccs met1_10

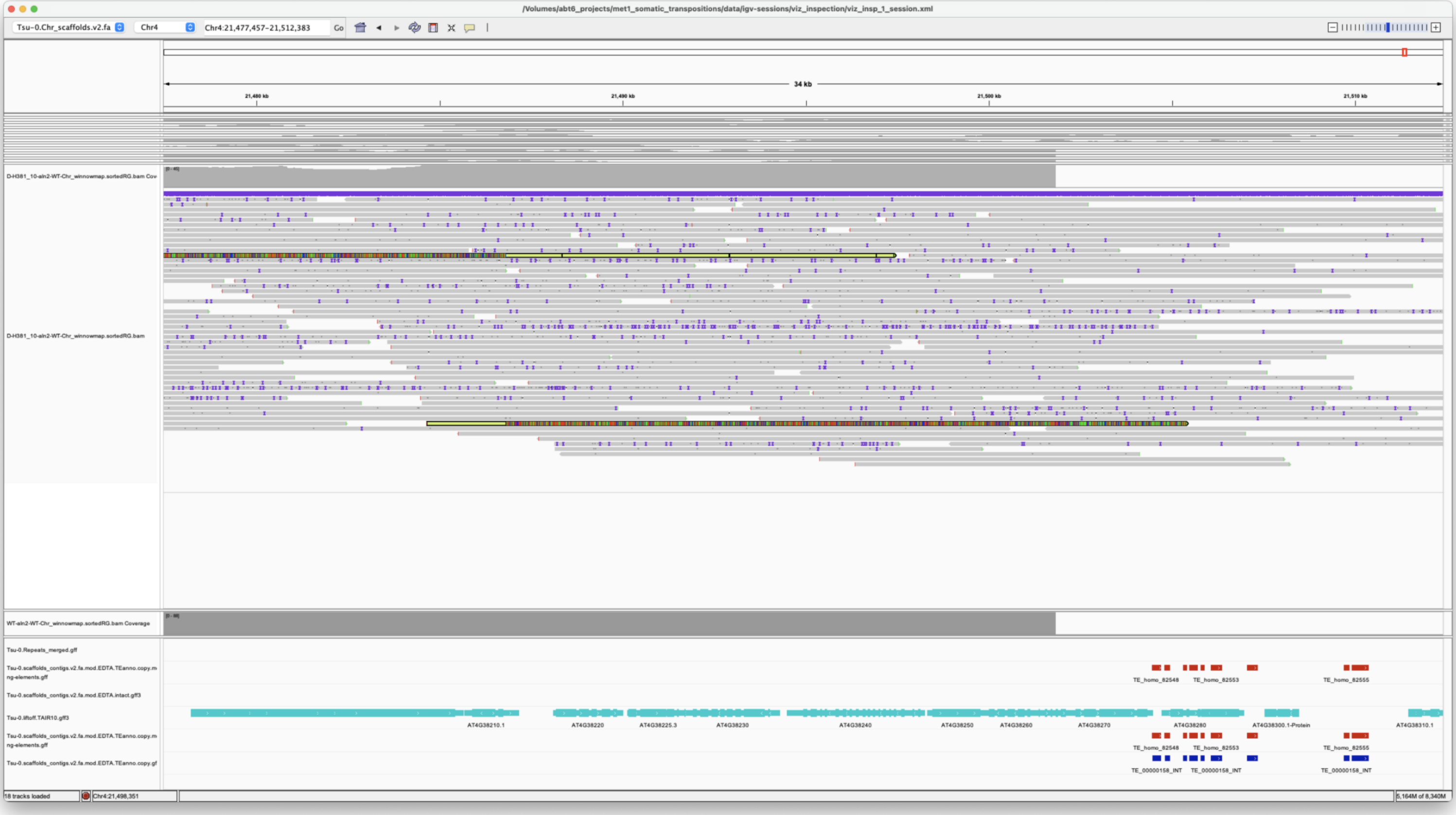

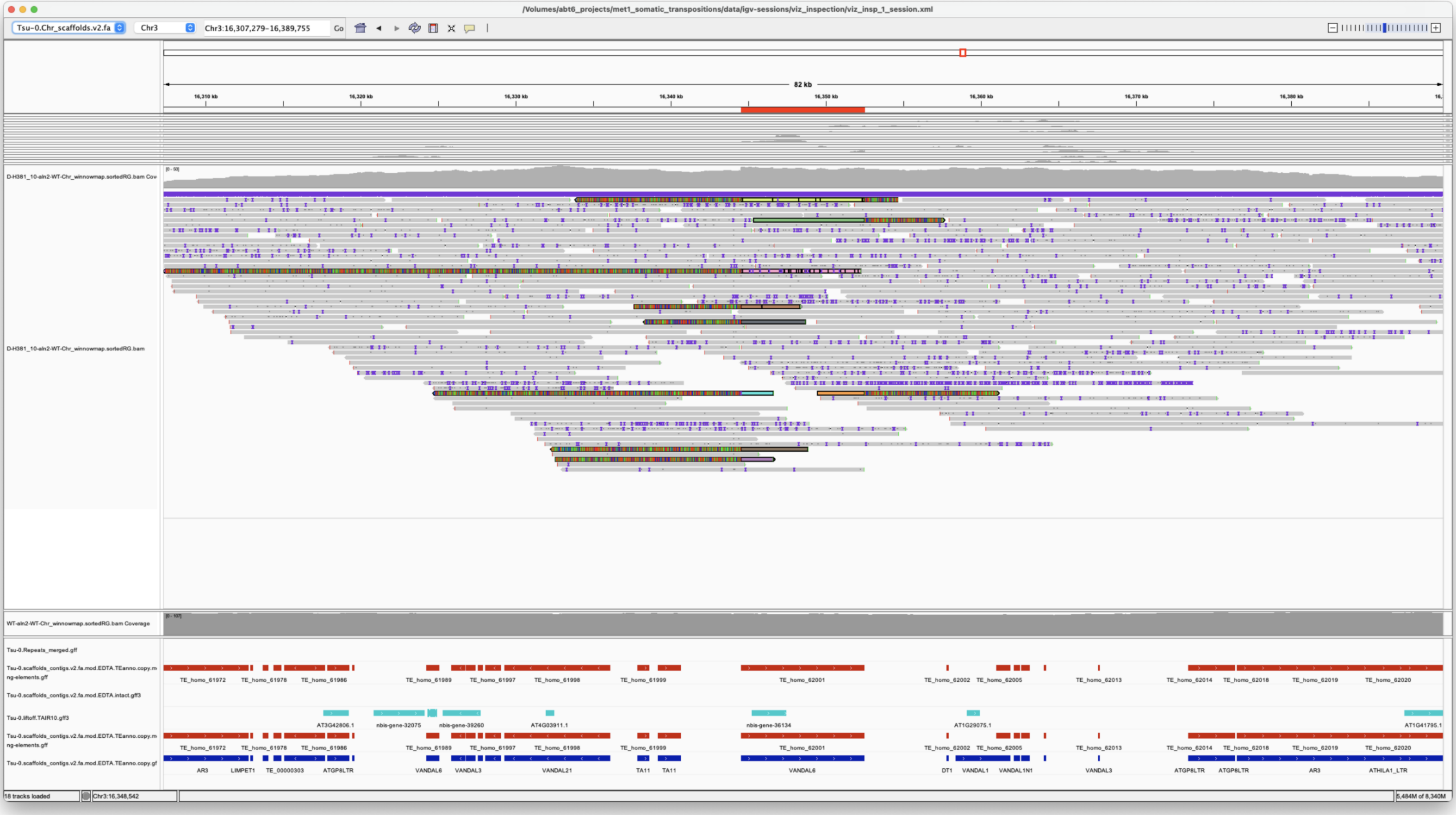

Central

Confirmed

Chr5 10254763 10254763 + 1 Chr5;19152829;19160826;VANDAL21 m64079_221220_112036/73926086/ccs met1_10

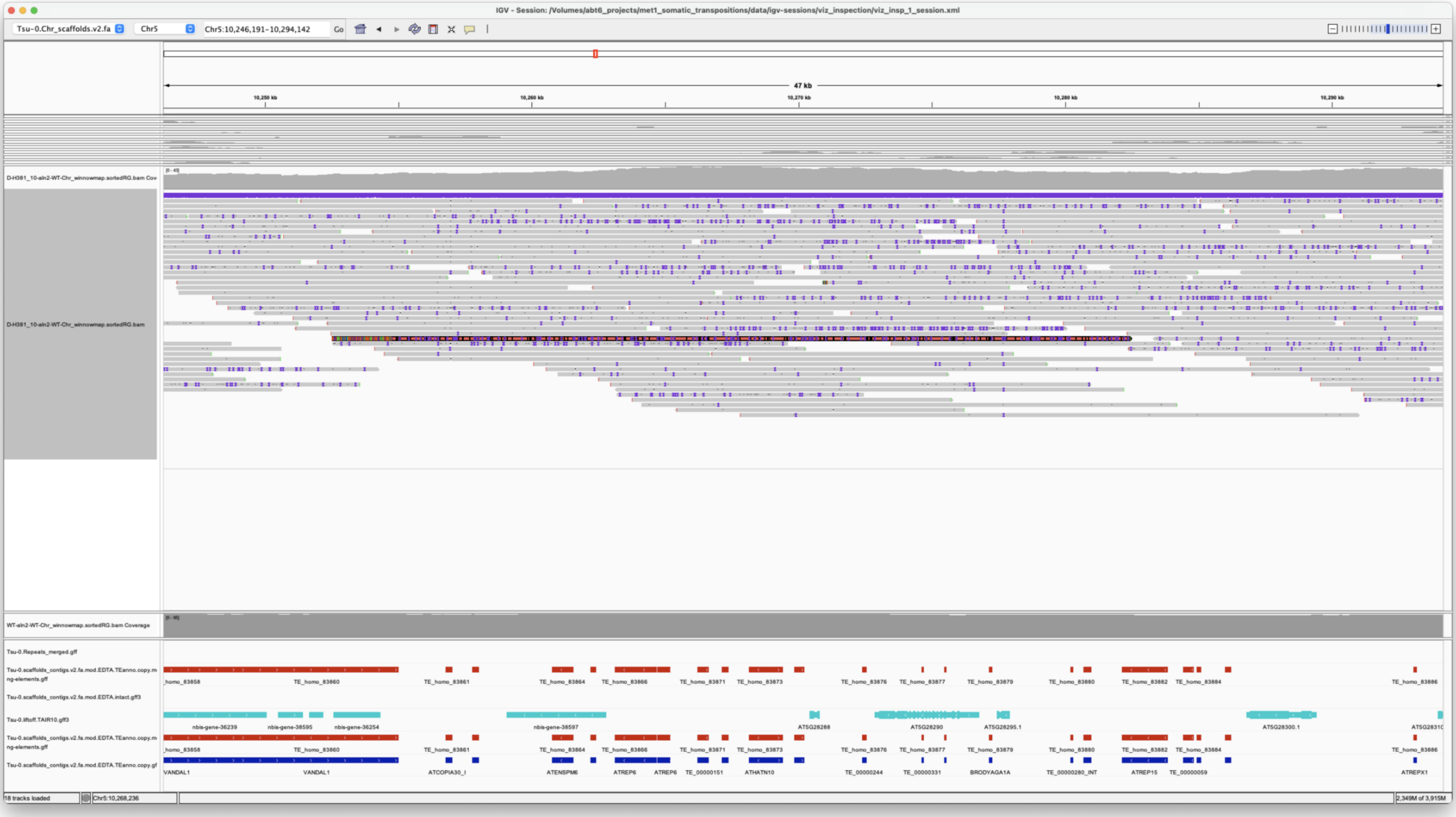

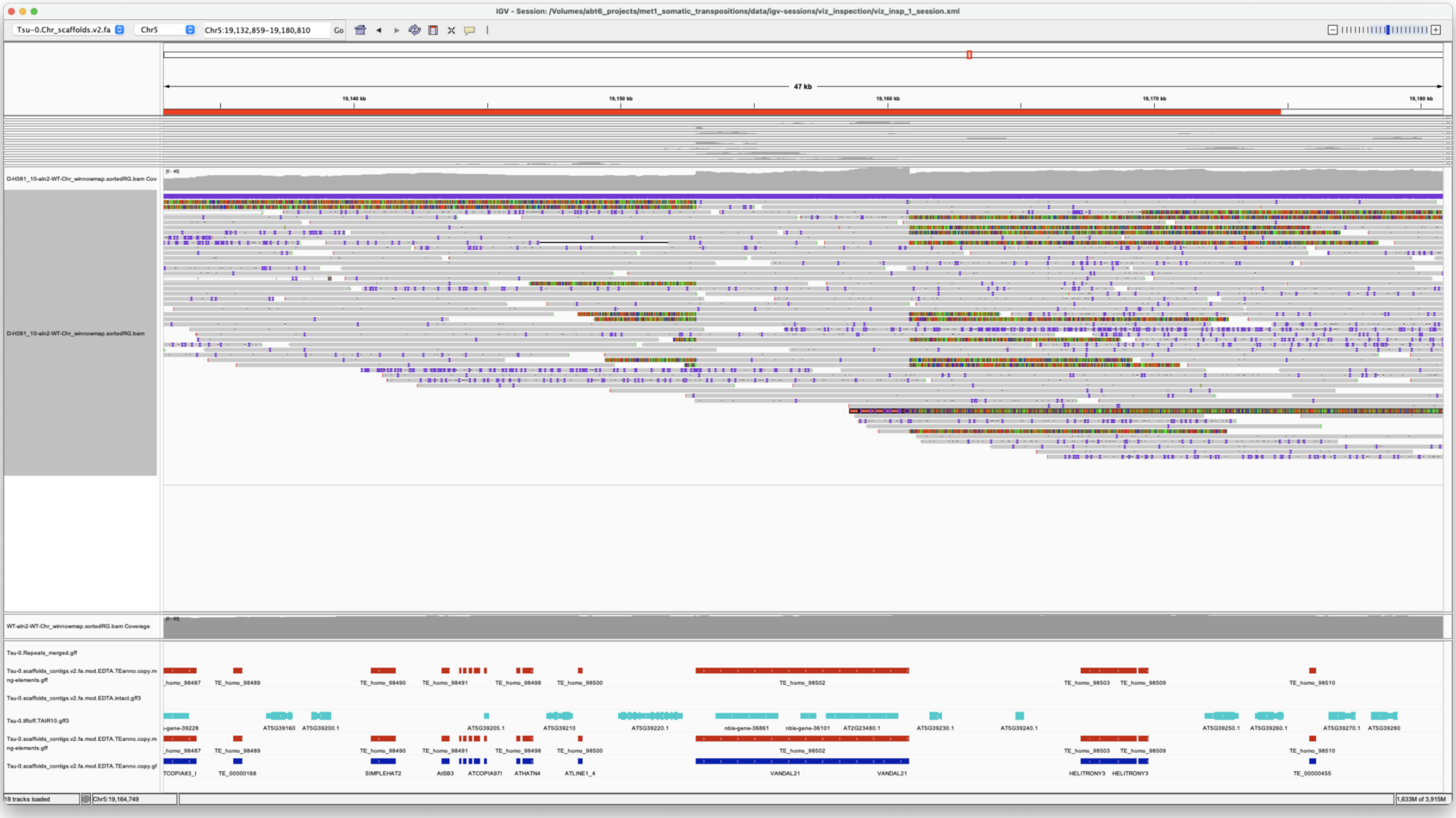

Partial

Confirmed

| Chr5 15974204 15974204 - 1 Chr5;19872565;19877095;VANDAL21 m64079_240212_113350/144048955/ccs met1_10

unsupported

| Chr5 18135651 18135651 - 1 Chr5;19872565;19877095;VANDAL21 m64079_240212_113350/166003235/ccs met1_10

unsupported

| Chr5 19865711 19865711 - 1 Chr5;19877283;19884298;VANDAL21 m64079_221220_112036/120390570/ccs met1_10

unsupported

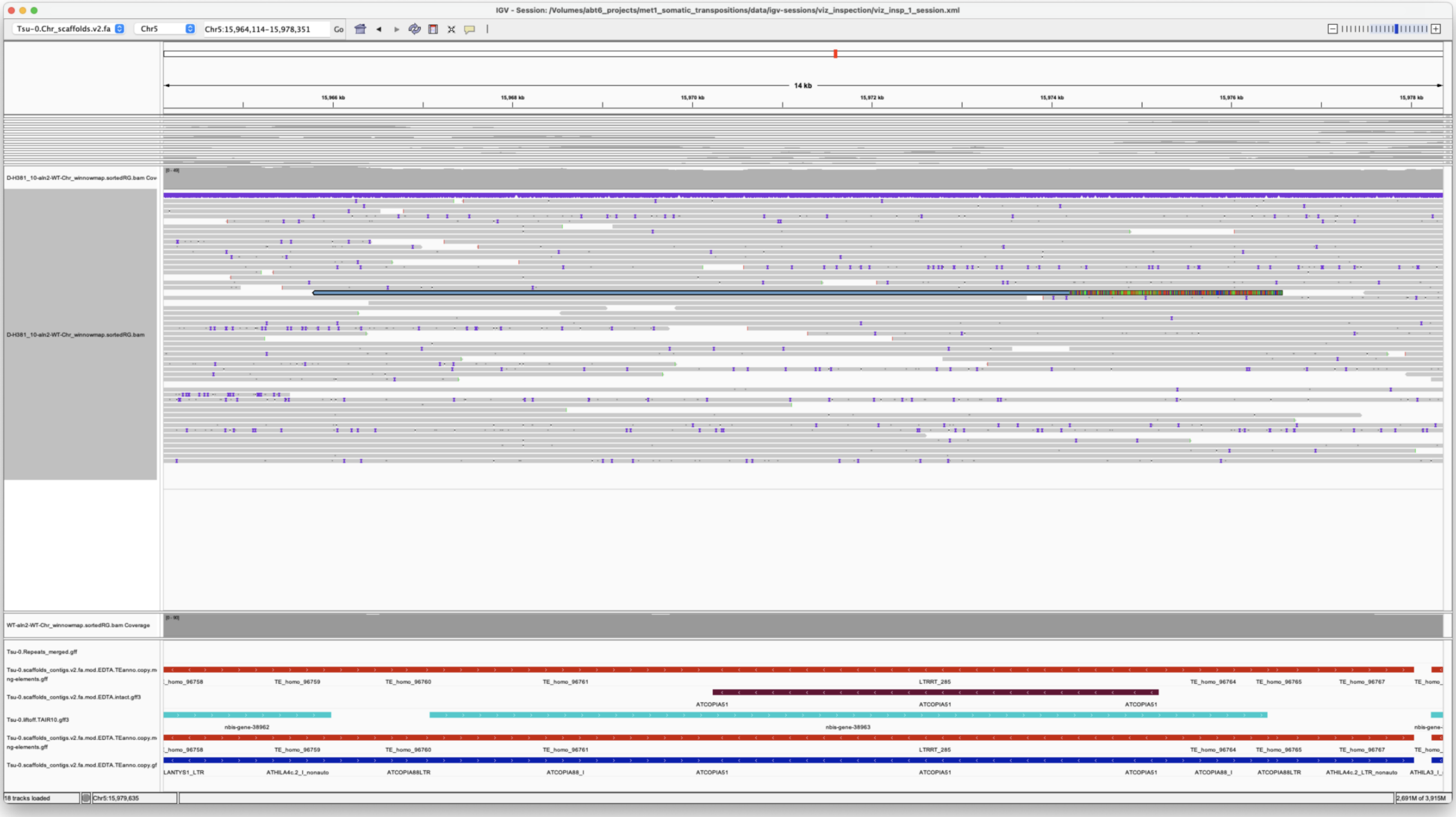
IGV - Session: /Volumes/abt6_projects/met1_somatic_transpositions/data/igv-sessions/viz_inspection/viz_insp_1_session.xml
Tsu-0.Chr_scaffolds.v2.fa
Chr5
Chr5:15,964,114-15,978,351
14 kb
D-H381_10-aln2-WT-Chr_winnowmap.sortedRG.bam
WT-aln2-WT-Chr_winnowmap.sortedRG.bam Coverage
Tsu-0.Repeats_merged.gff
Tsu-0.liftoff.TAIR10.gff3
ATCOPIA51
18 tracks loaded

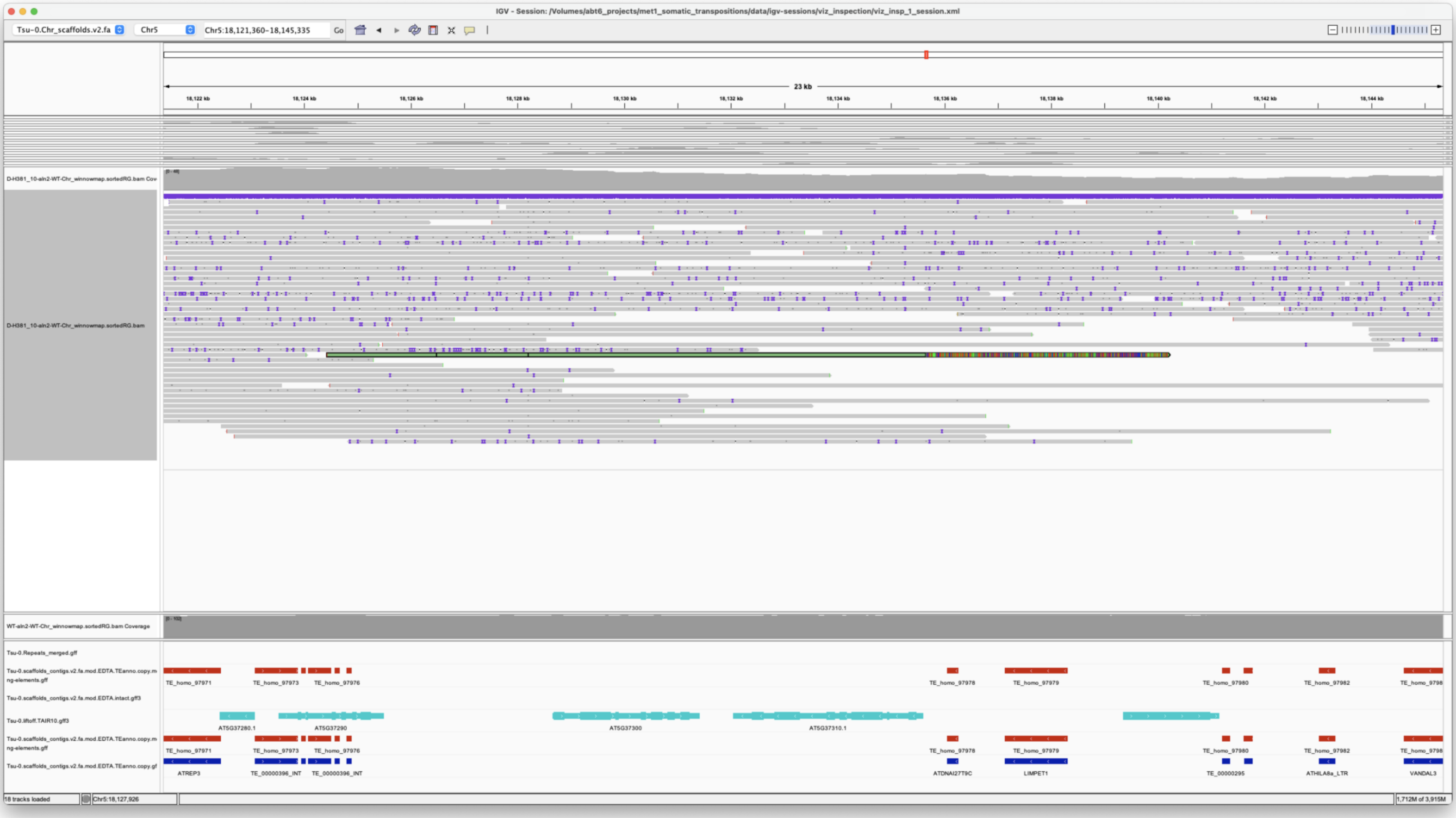
IGV - Session: /Volumes/abt6_projects/met1_somatic_transpositions/data/igv-sessions/viz_inspection/viz_insp_1_session.xml
Tsu-0.Chr_scaffolds.v2.fa
Chr5
Chr5:18,121,360-18,145,335
23 kb
D-H381_10-aln2-WT-Chr_winnowmap.sortedRG.bam
WT-aln2-WT-Chr_winnowmap.sortedRG.bam Coverage
Tsu-0.Repeats_merged.gff
Tsu-0.liftoff.TAIR10.gff3
ATREP3
LIMPET1
VANDAL3
18 tracks loaded

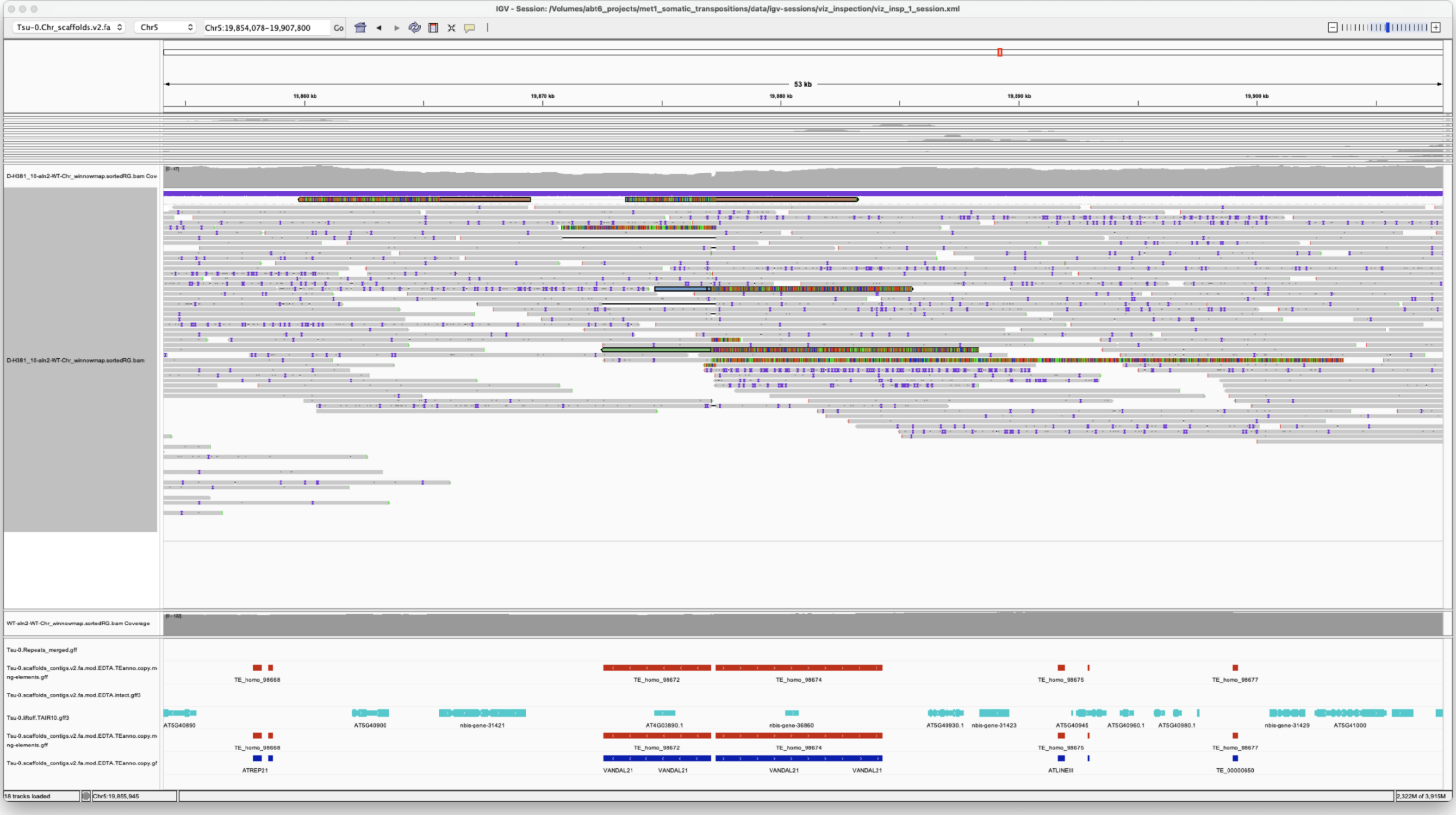

> Chr5 20104411 20104411 + 1 Chr5;19872565;19877095;VANDAL21 m64079_240212_113350/104006494/ccs met1_10

Hypermutable region

unsupported

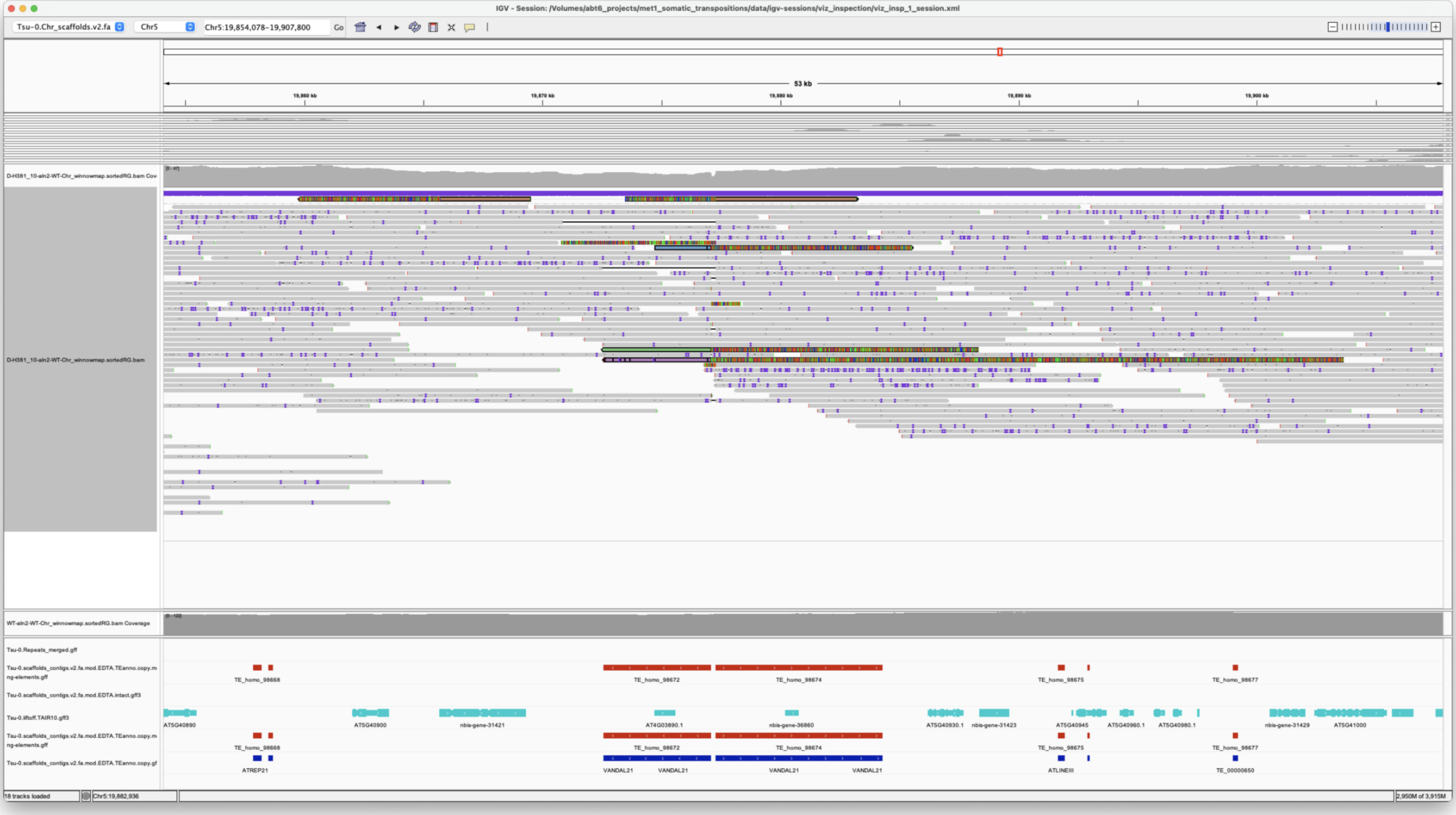

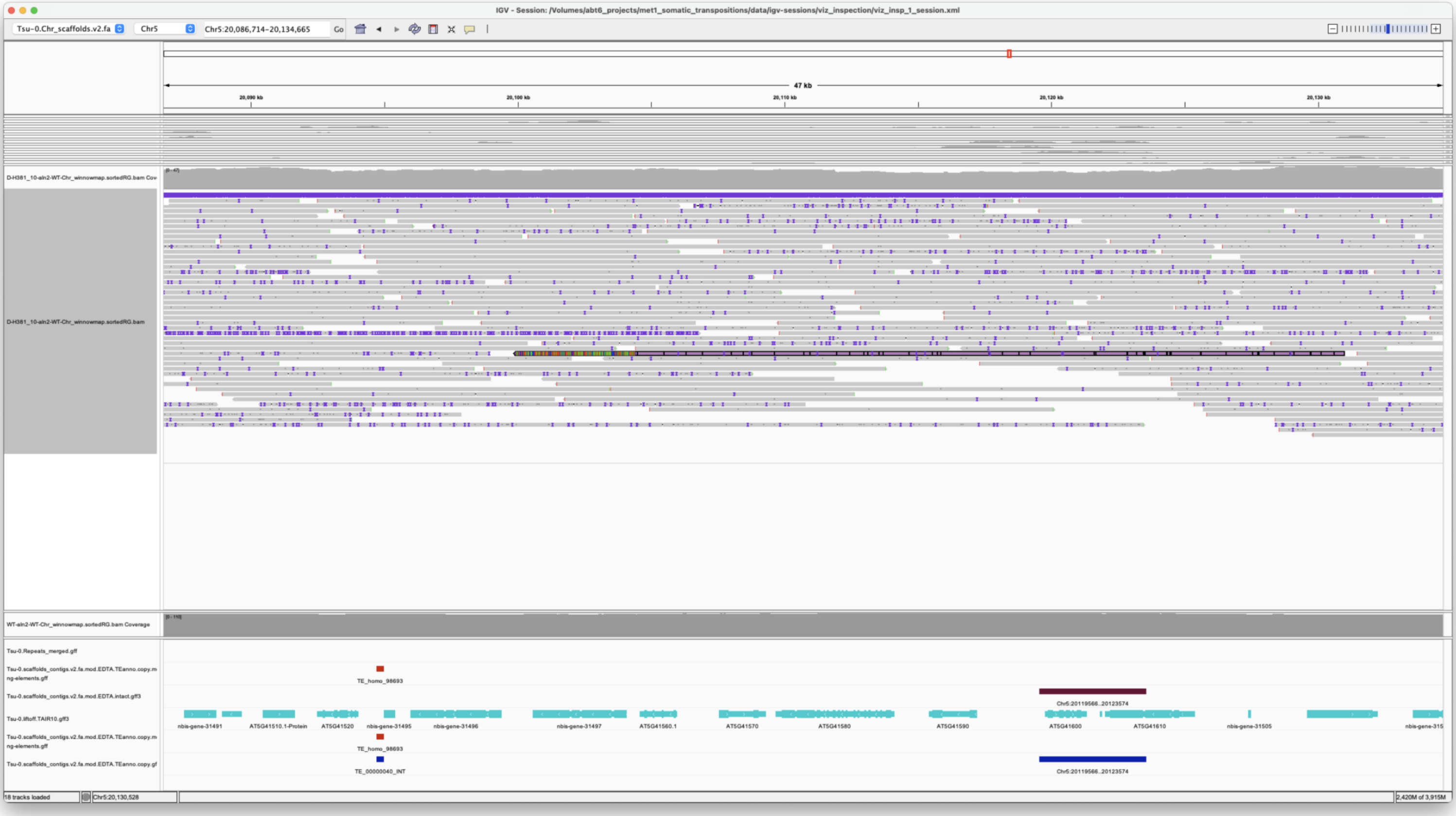

ALL REARRANGEMENTS

Rearrangement

Chr5 19909428 19909428 - 1 Chr5;19152829;19160826;VANDAL21 m64079_221220_112036/160170644/ccs met1_10

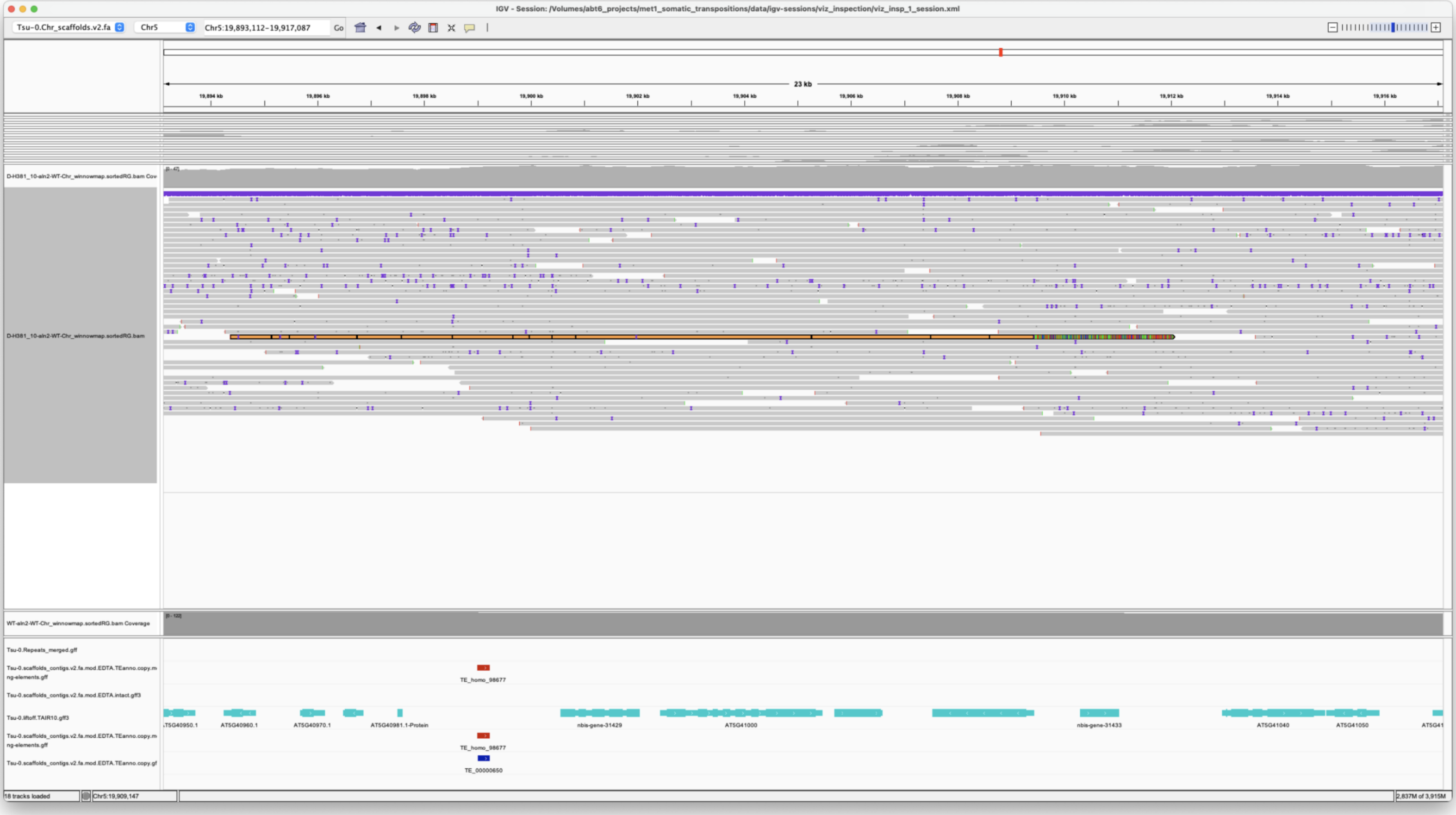

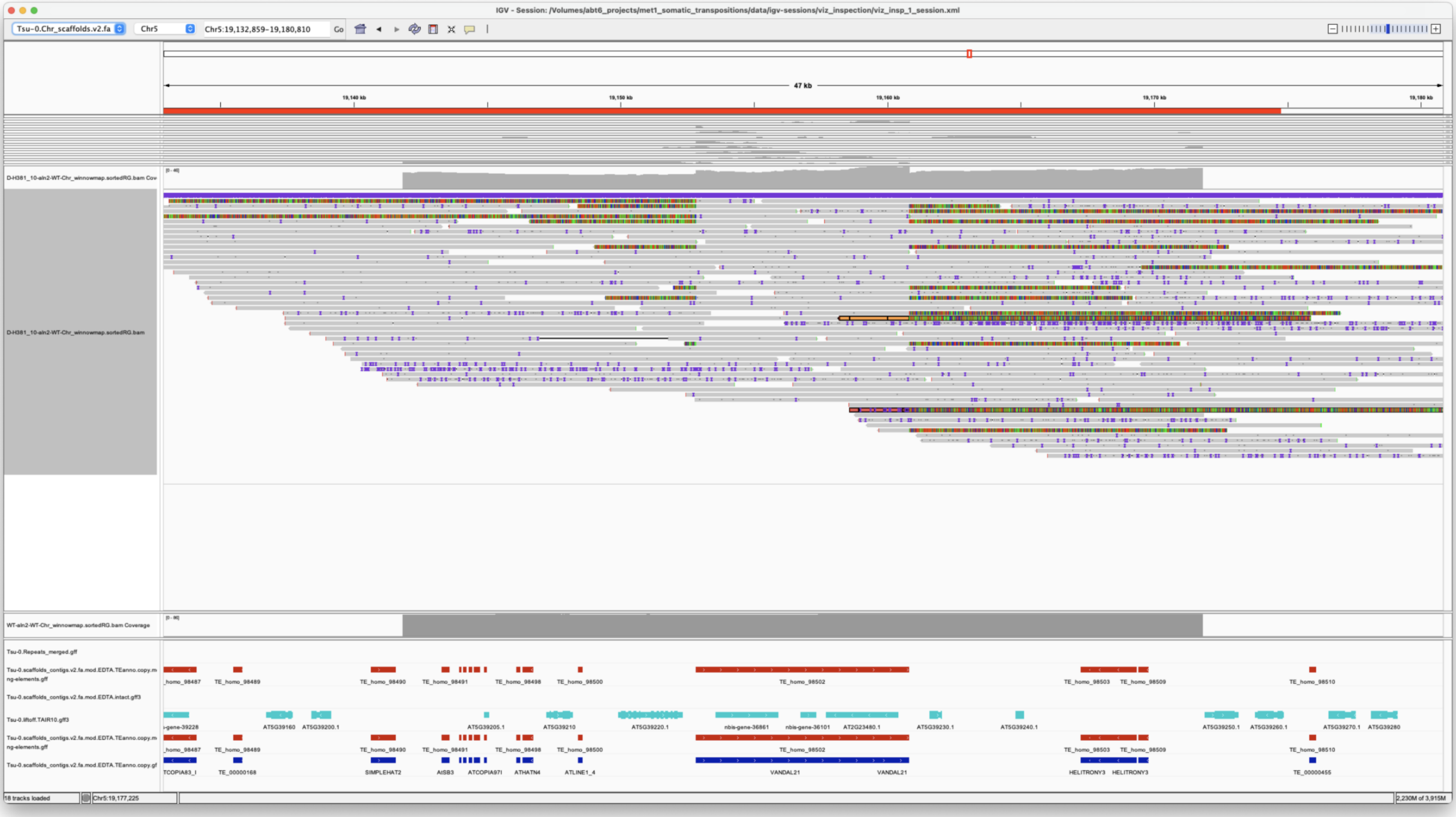

Partial

Confirmed

Chr5 23026038 23026038 - 1 Chr5;19152829;19160826;VANDAL21 m64079_221220_112036/113183496/ccs met1_10

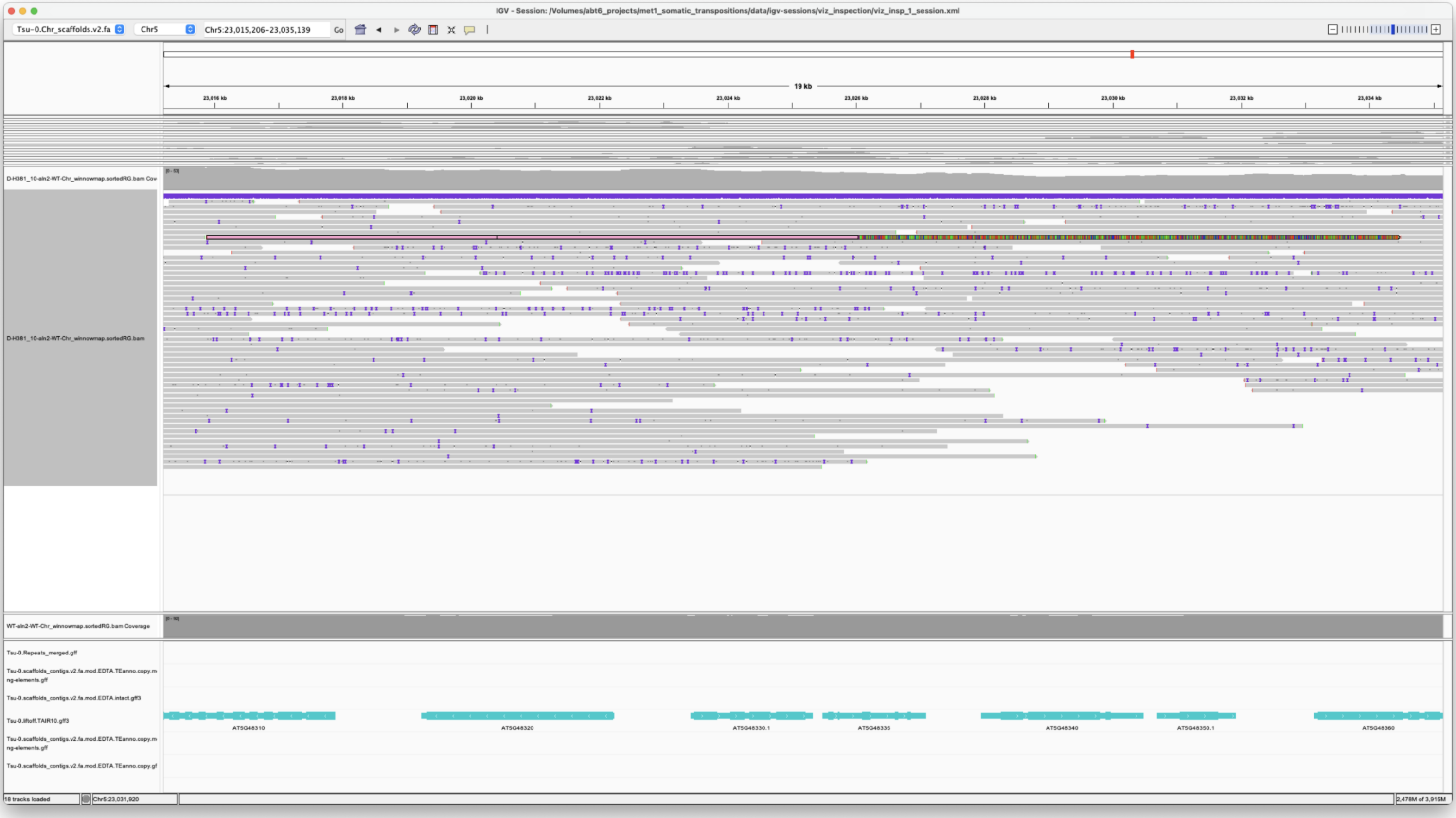

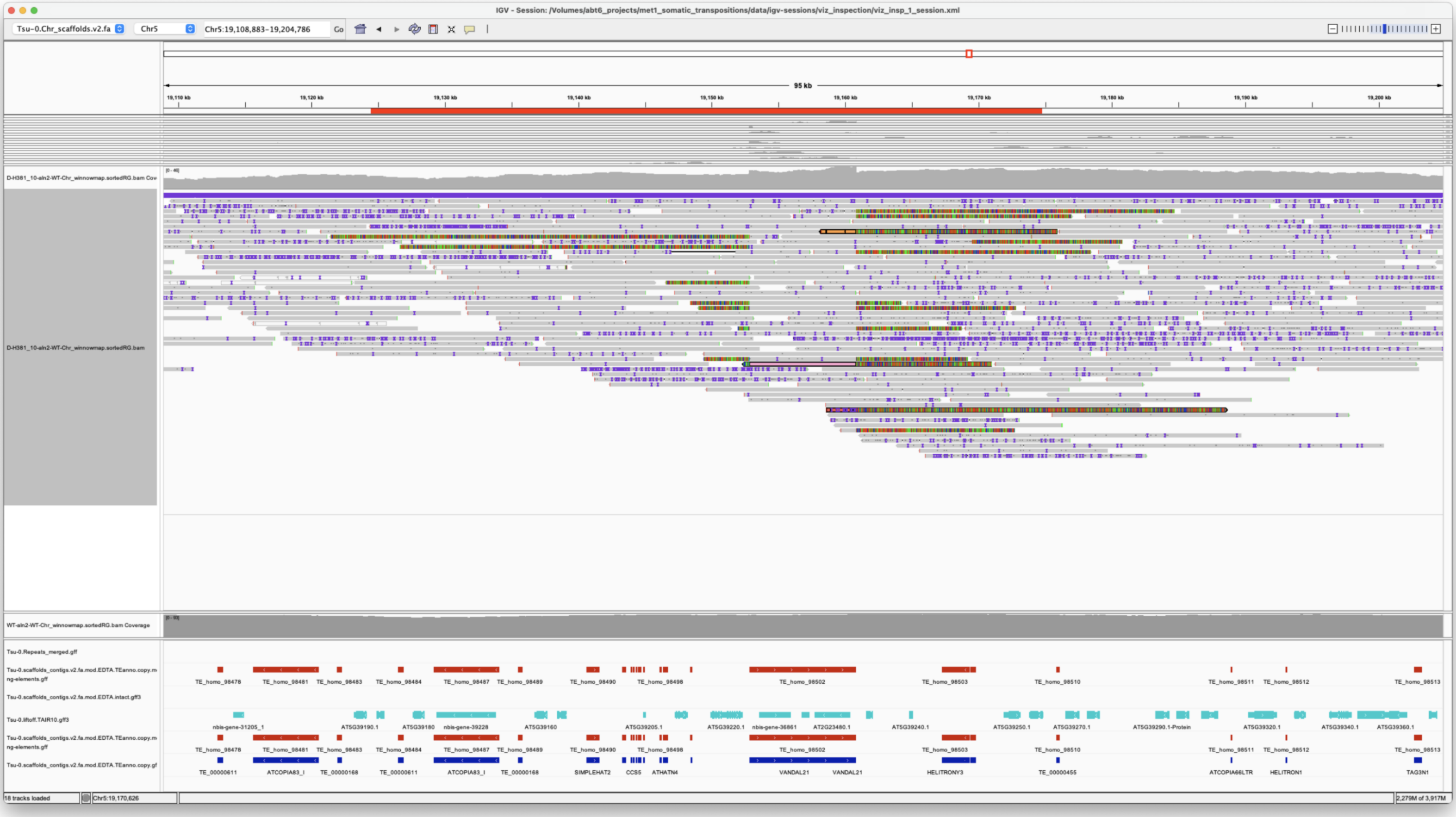

Partial

Confirmed

Chr5 29047155 29047155 + 1 Chr5;875414;876434;PAC m64079_221220_112036/118751517/ccs met1_10

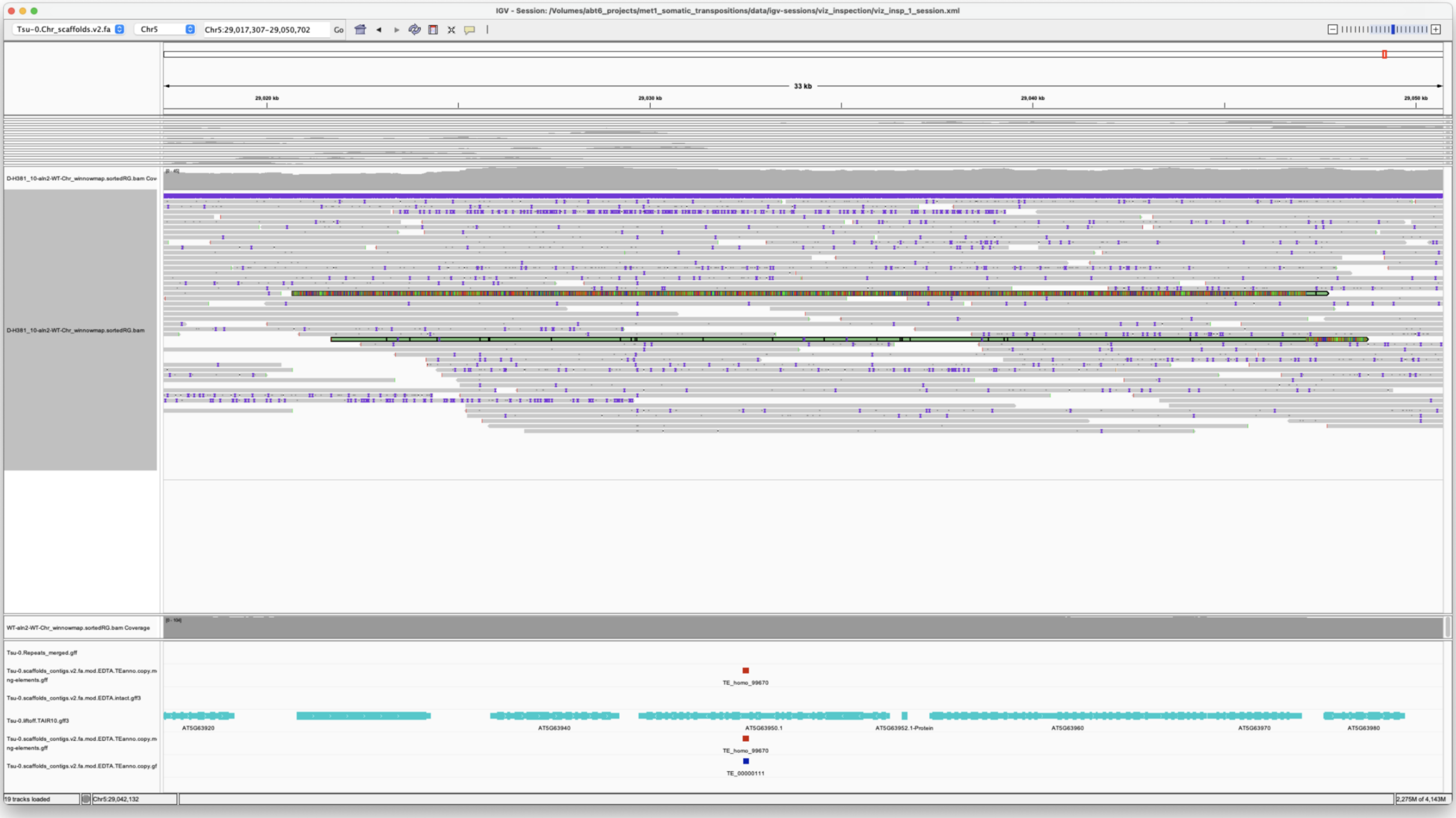

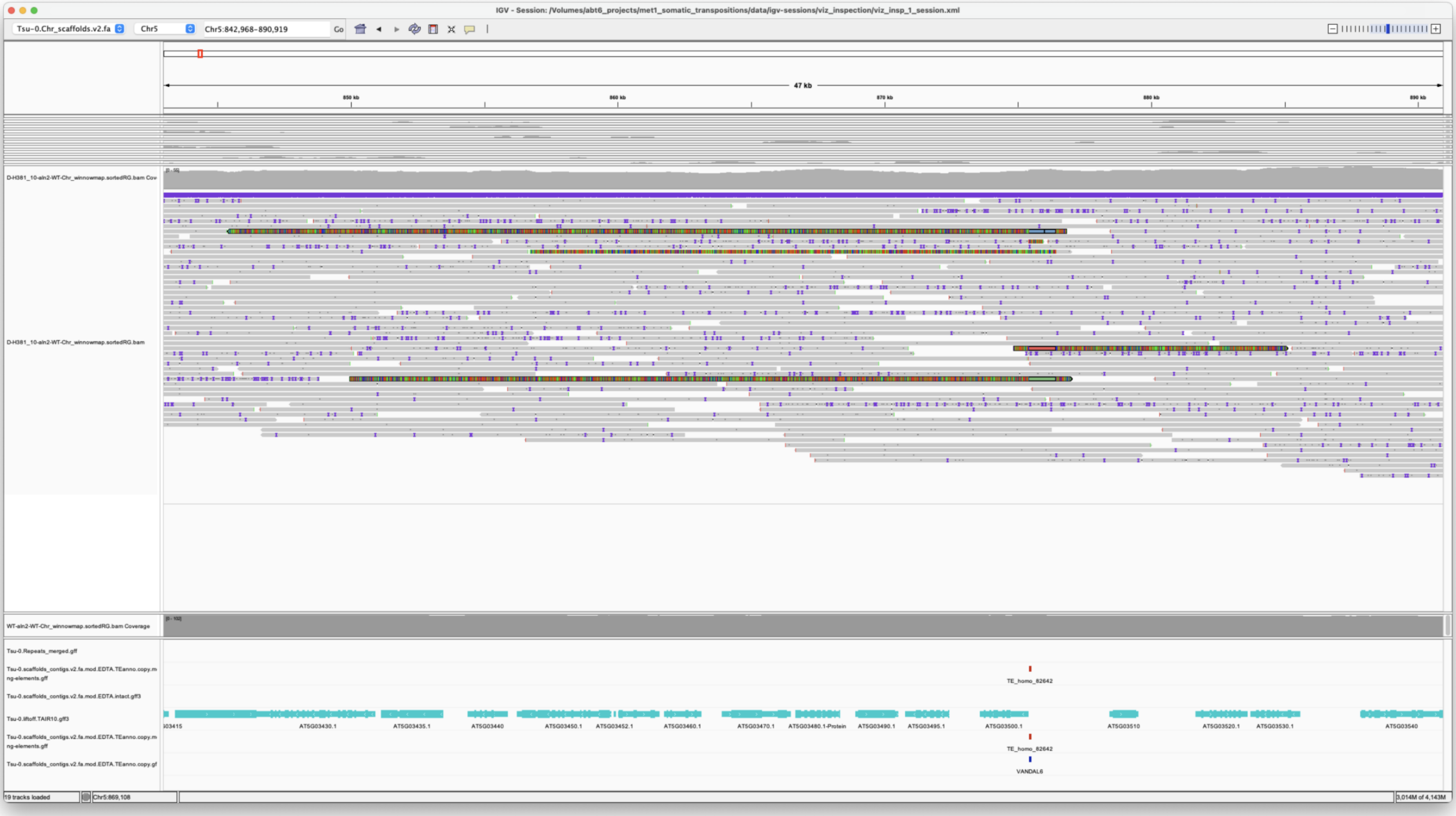

Partial

Confirmed

WT

Chr5 16330415 16330415 + 1 Chr4;2862691;2874240;ATLANTYS1_LTR m64079_220303_191344/2163209/ccs WT

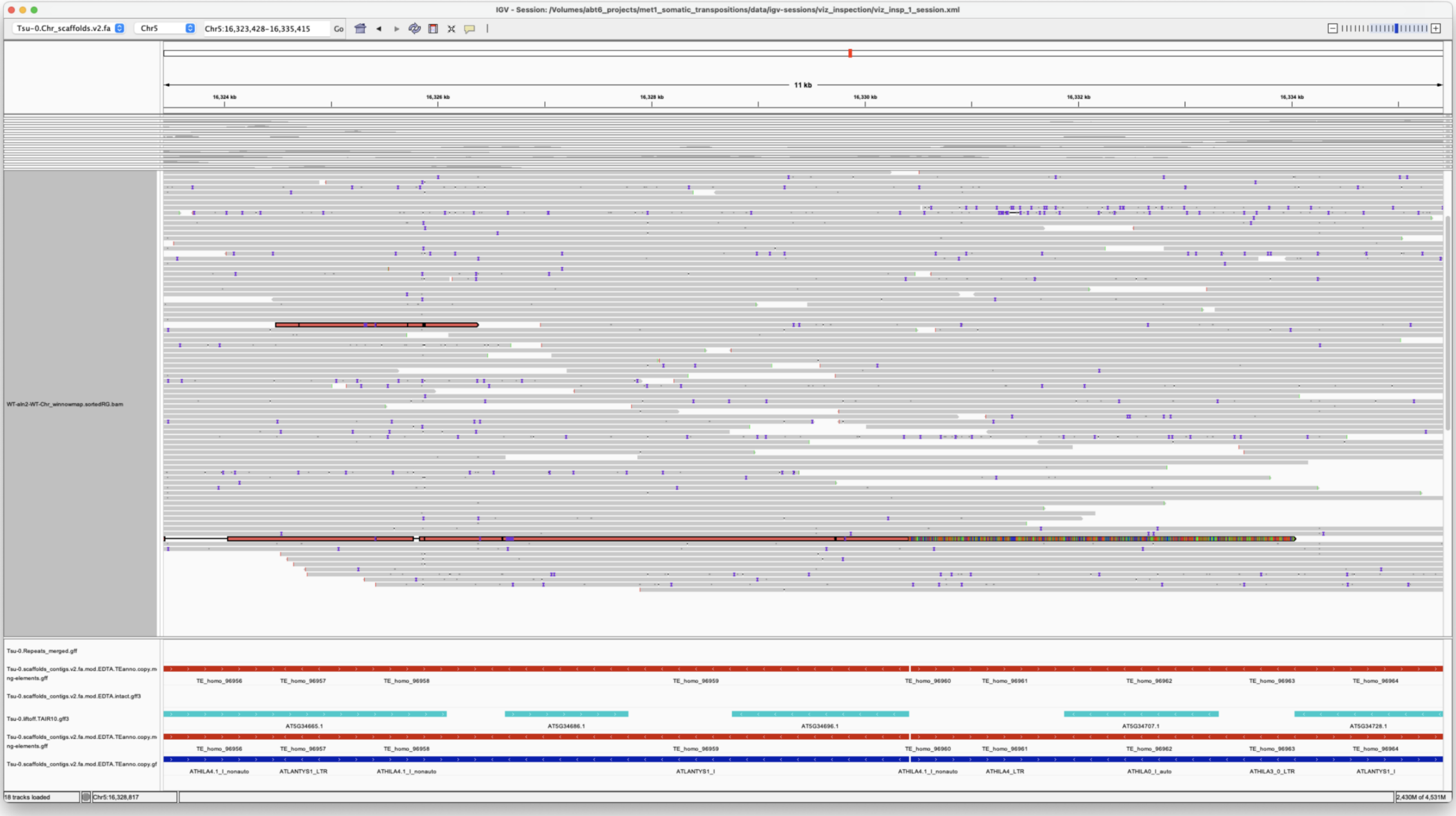

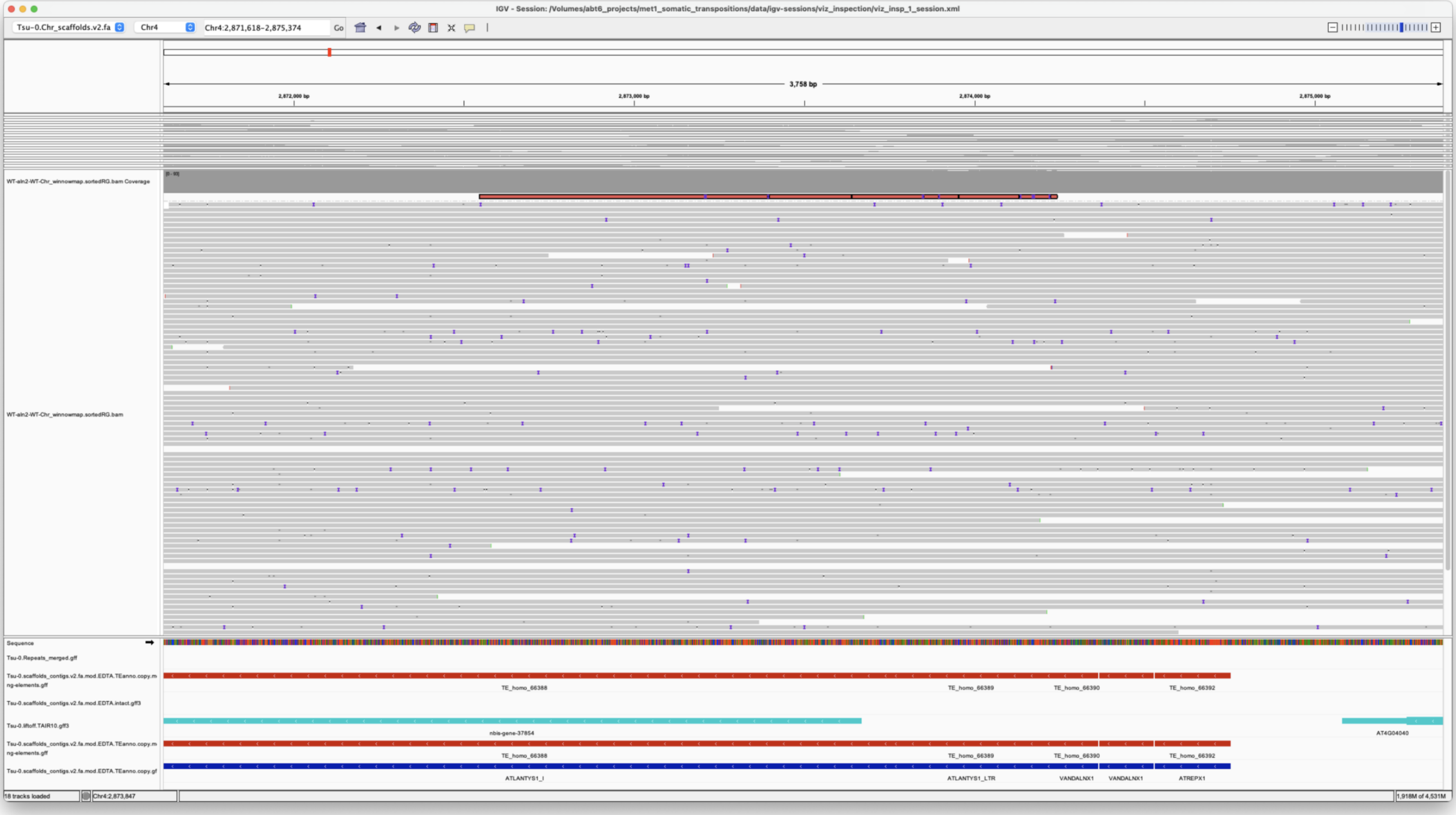

Probably just a rearrangement. It is not a whole TE insertion. Illegitimate recombination involved?

Rearrangement

Chr5 16958811 16958811 - 1 Chr5;18798099;18800344;ATREP5 m64079_220303_191344/148834725/ccs WT

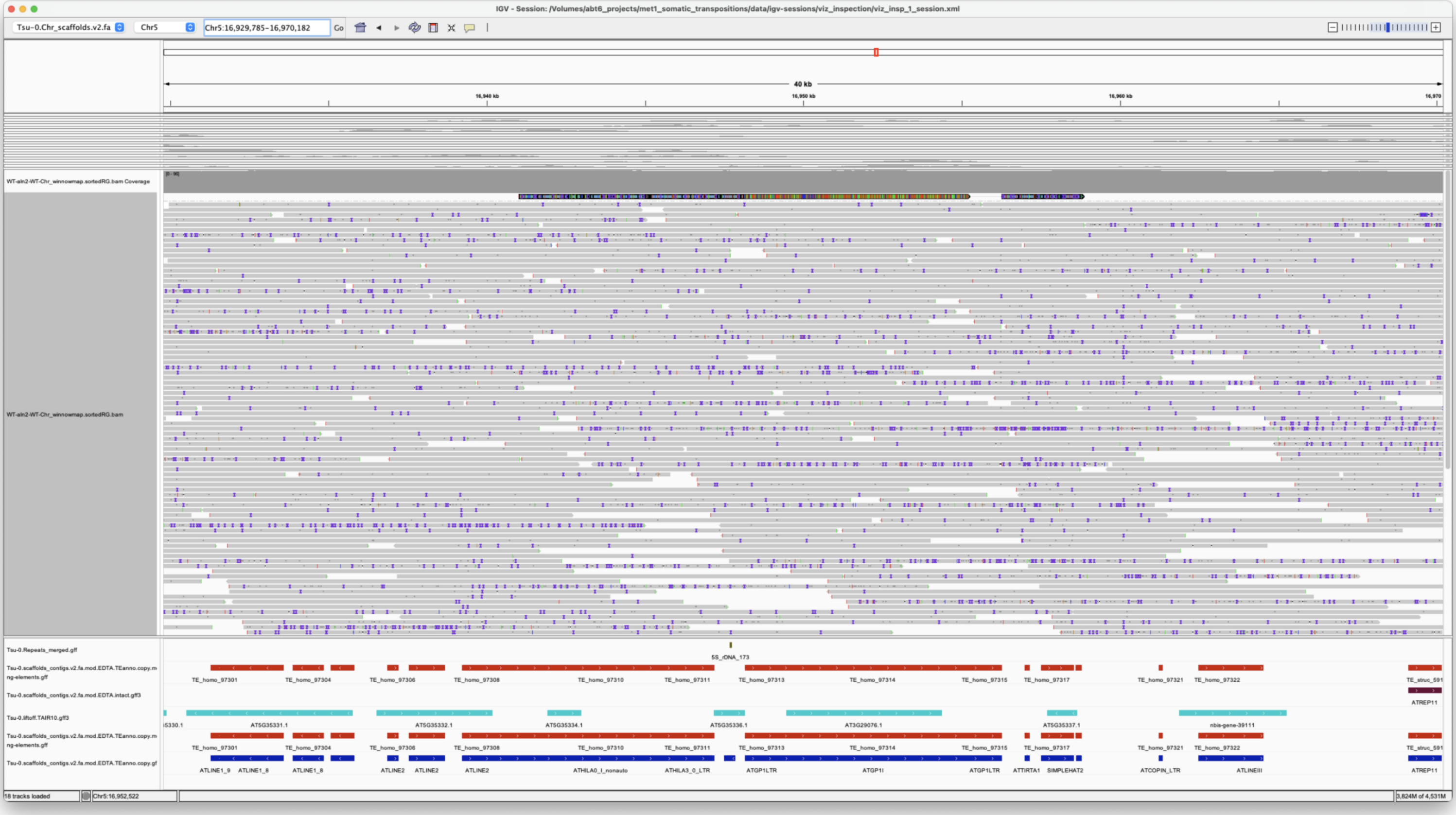

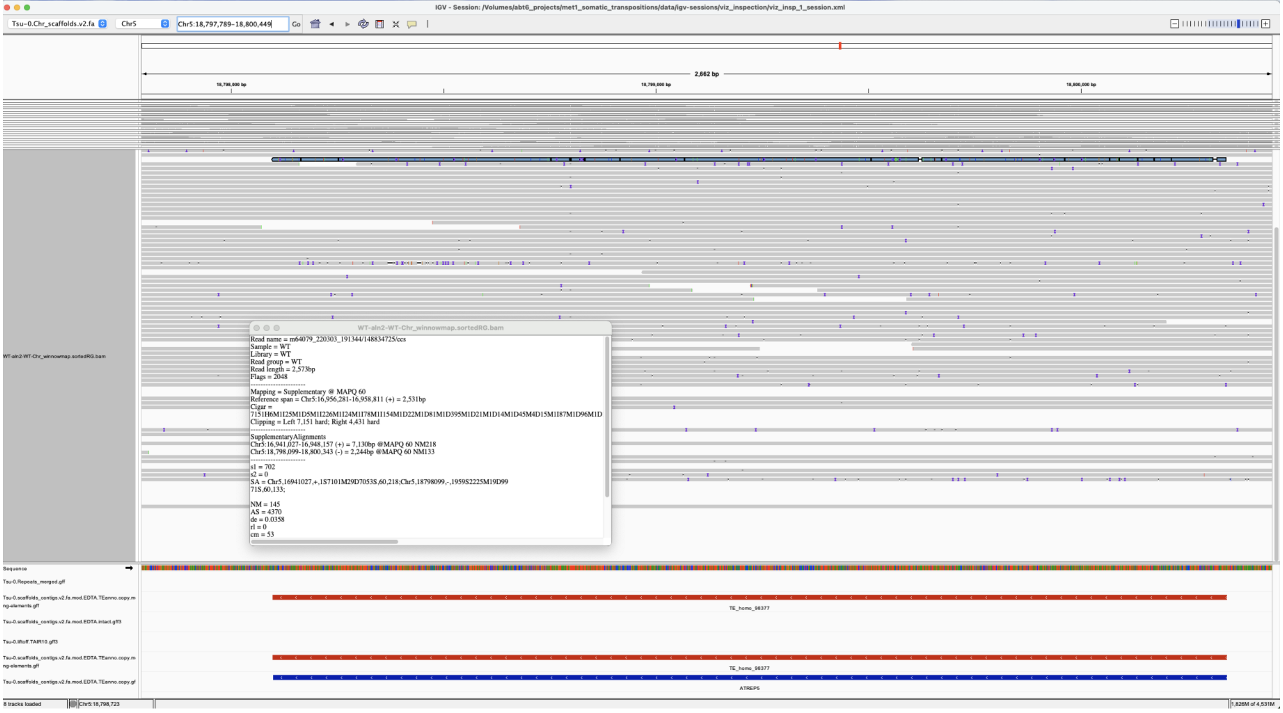

Not even Rearrangement

Something weird
