# Supplementary material for: Long-read detection of transposable element mobilization in the soma of hypomethylated Arabidopsis thaliana individuals
Source: Genome Biol. 2025 Jul 30;26:231. doi: 10.1186/s13059-025-03691-7 (PMC12312487; doi:10.1186/s13059-025-03691-7)
Supplement: Supplementary file 2 — Additional file 2. Visual inspection of somatic insertion and excision events, available at https://github.com/aerilli/Somatic-transposition_met1/tree/551df407370c6528225f404ba62a073dced14b08/Supplementary-Files/Visual_inspection. [file 13059_2025_3691_MOESM2_ESM.gz › Split_Supplementary-File4/File1_SupplementaryALN_Insertions/File1_SupplementaryALN_Insertions-1-68.pdf]

SupplementaryALN_Insertions
Merged elements

met1_01

Chr1 7457391 7457391 - 1 Chr1;11941102;11946441;ATCOPIA93_Evade m64079_221220_112036/9243376/ccs met1_01

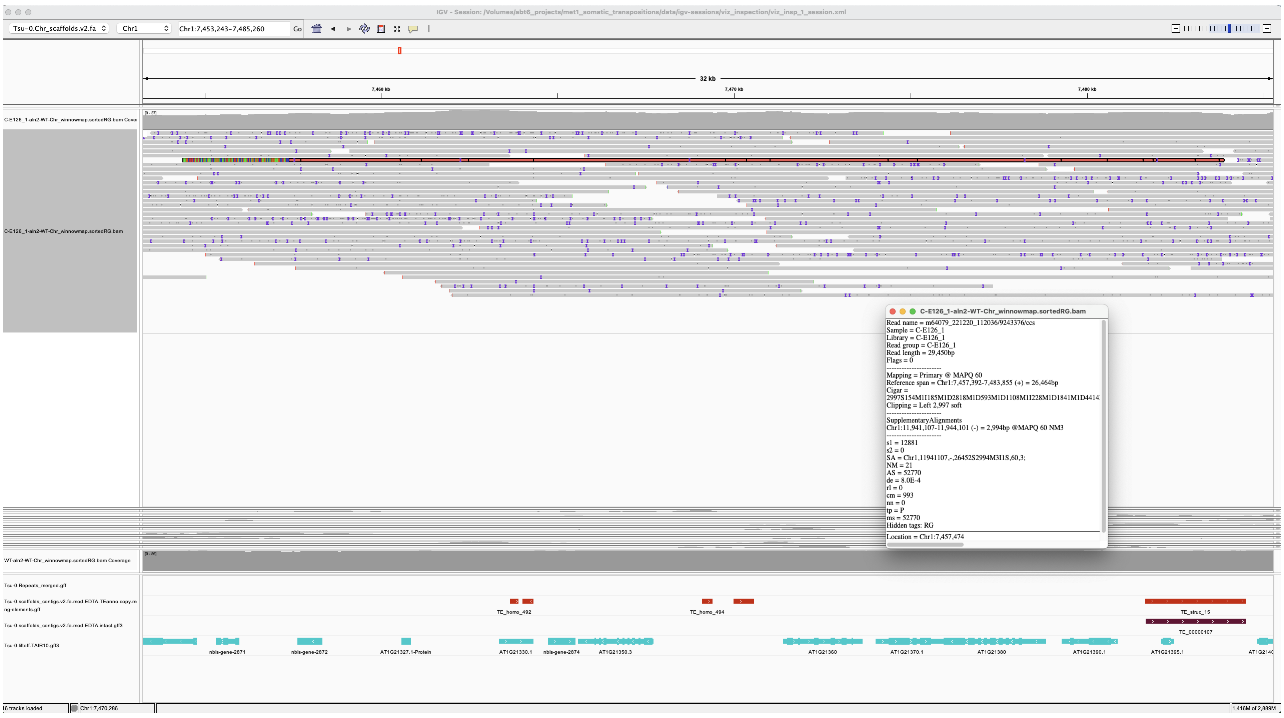

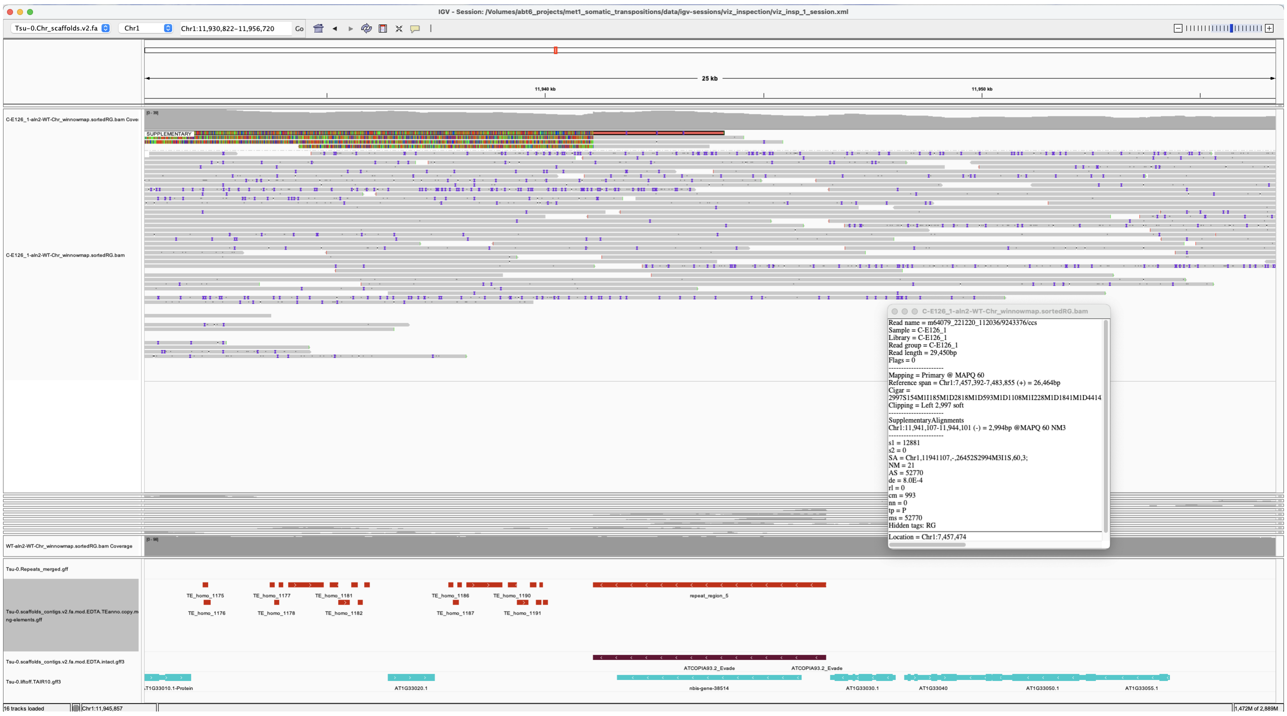

Partial

Confirmed

Chr1 10135363 10135363 + 1 Chr5;19152829;19160826;VANDAL21 m64079_221220_112036/136251212/ccs met1_01

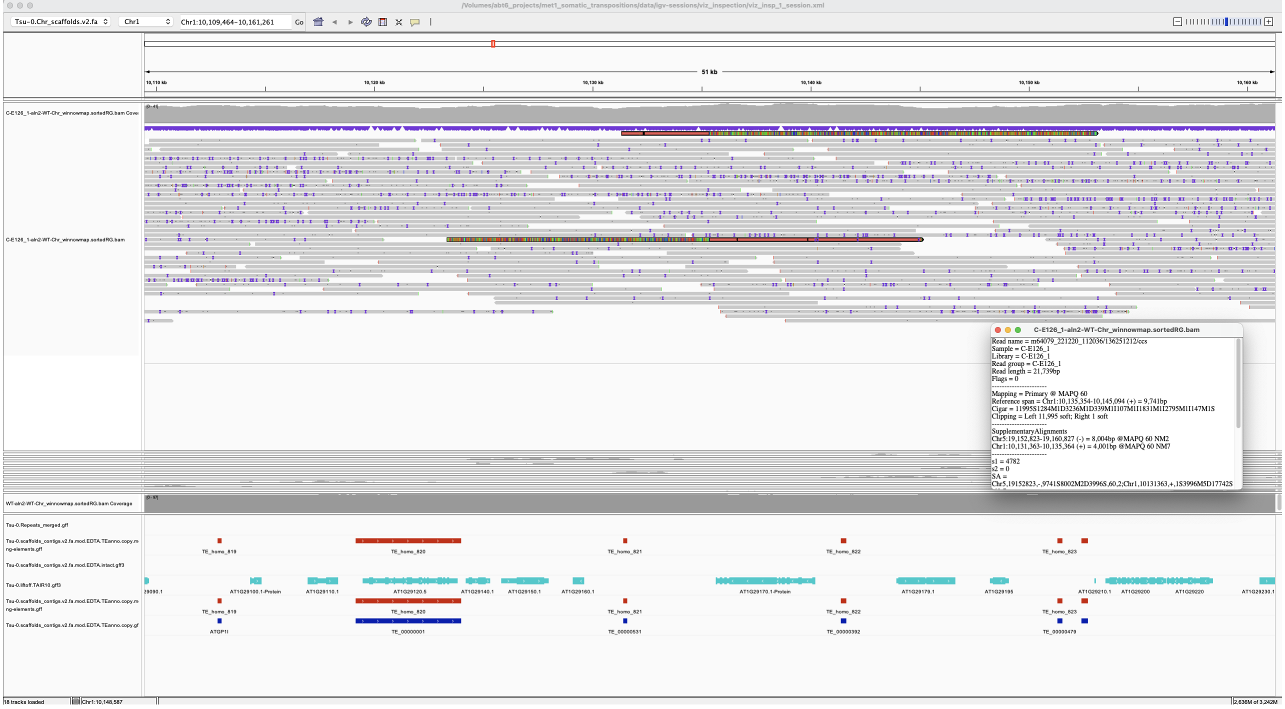

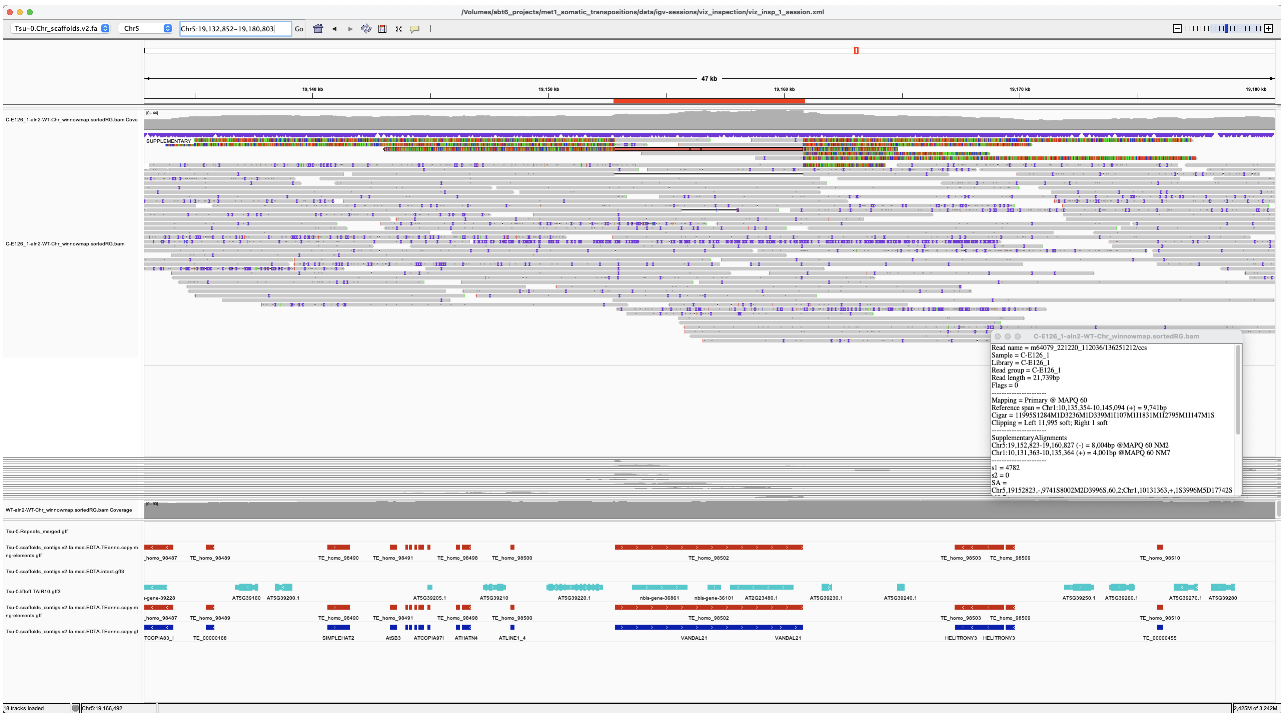

Central
TSD

Confirmed

TE_00000590 -> To be ignored for further cases!
High coverage, present also in WT!

Chloroplast

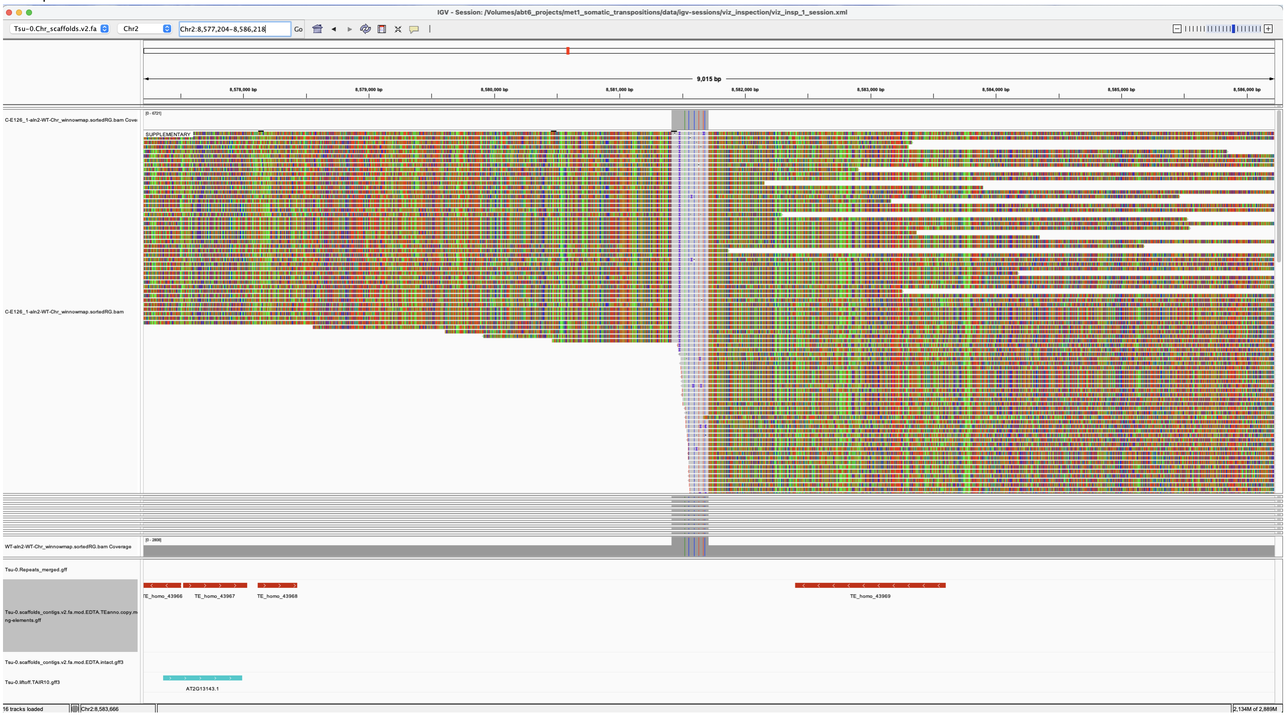

unsupported

Chr2 3983380 3983380 + 1 Chr5;19152829;19160826;VANDAL21 m64079_221220_112036/134744172/ccs met1_01

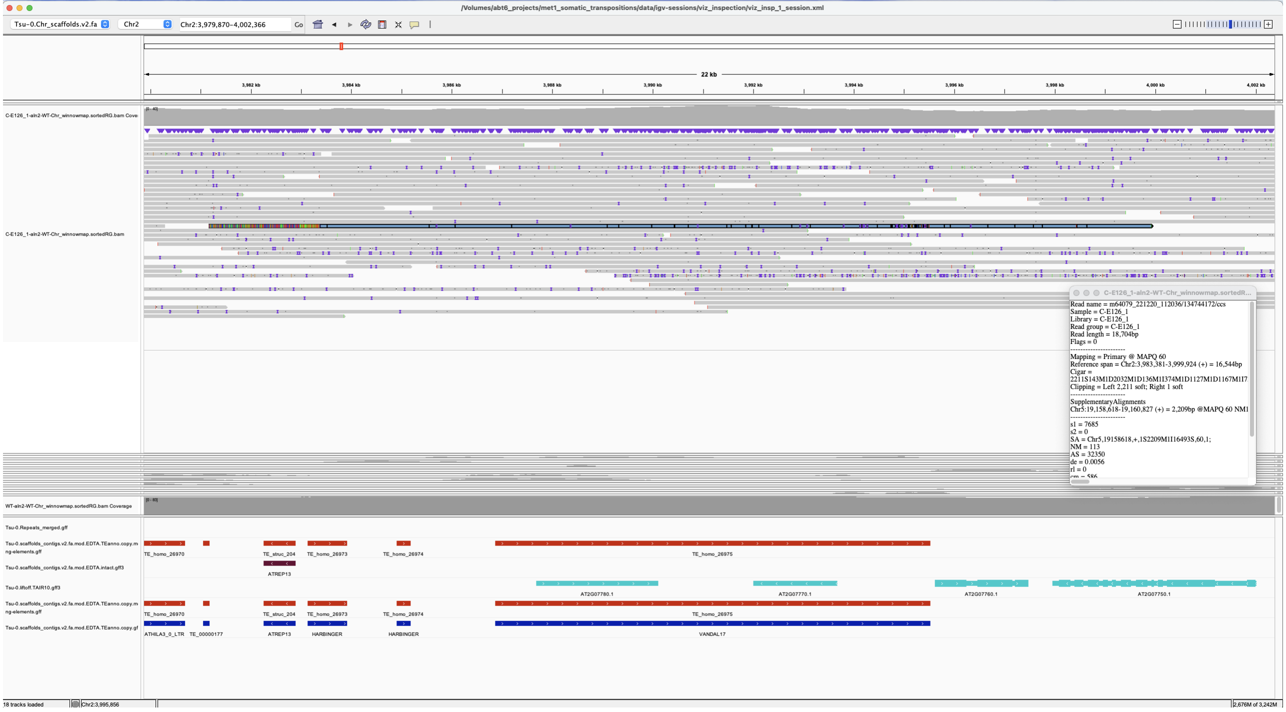

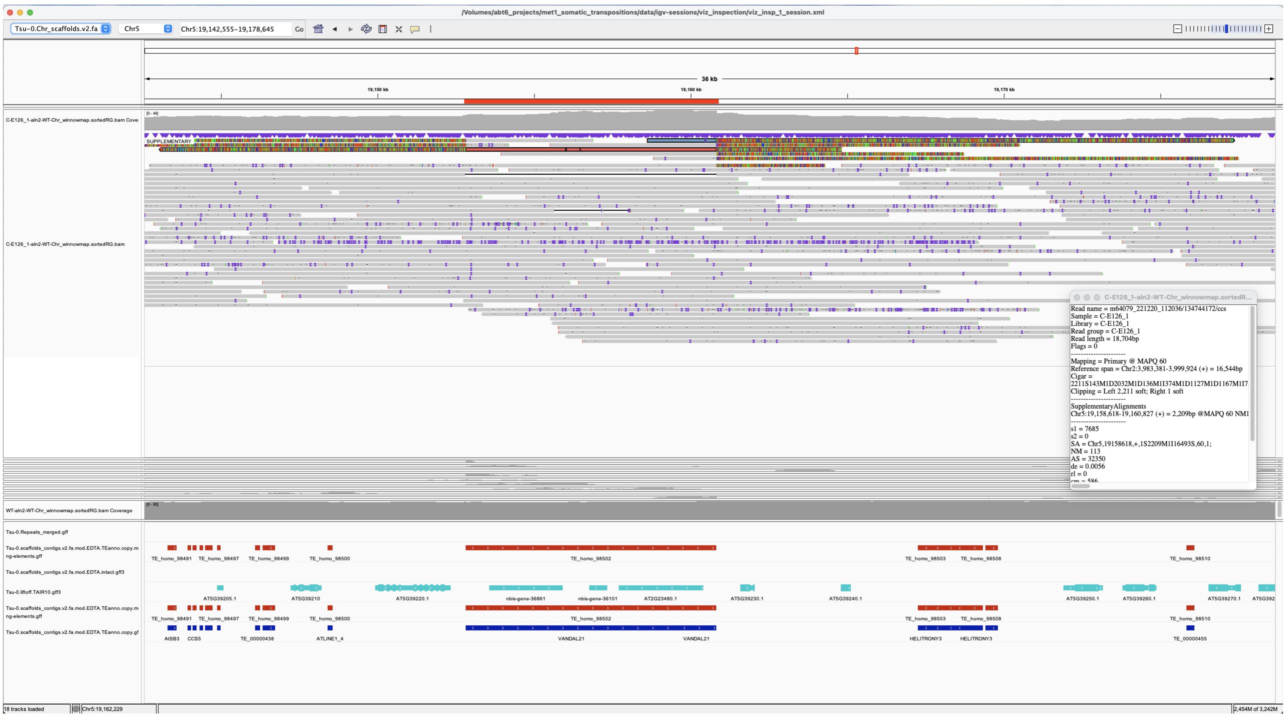

Partial

Confirmed

Chr2 8873653 8873653 + 1 Chr3;16344523;16352497;VANDAL6 m64079_221220_112036/162138239/ccs met1_01

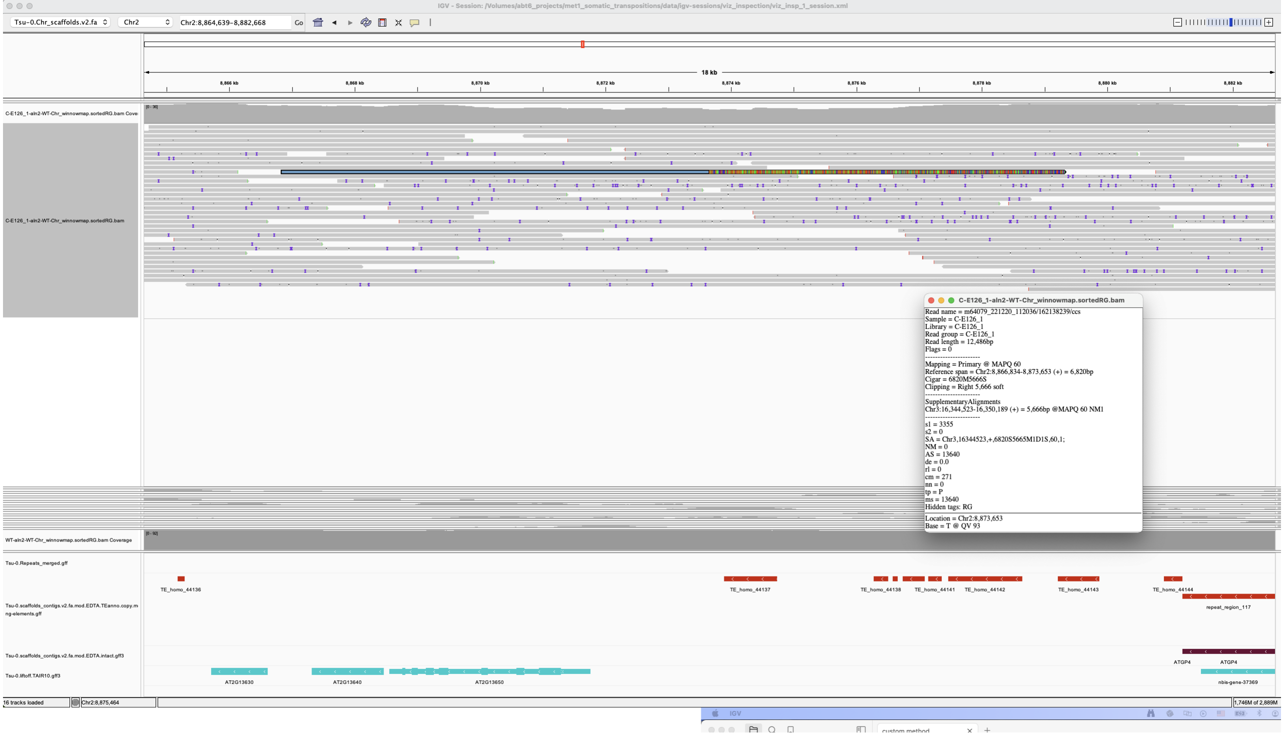

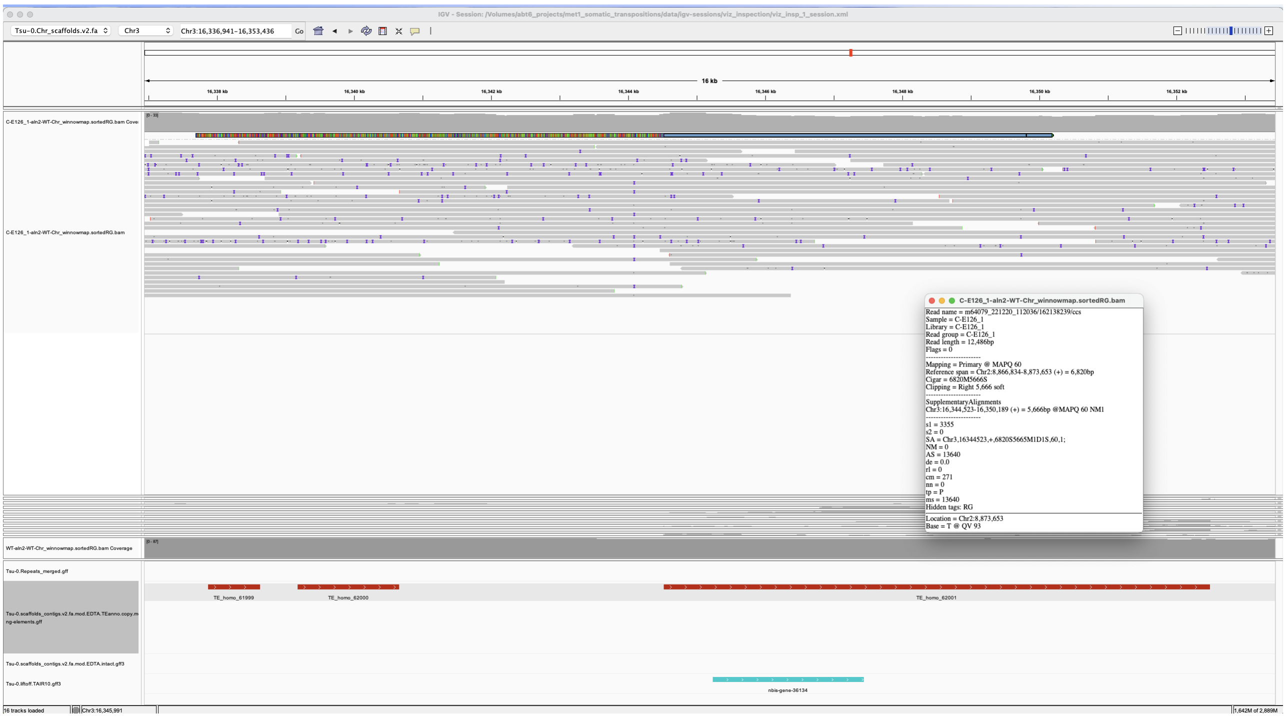

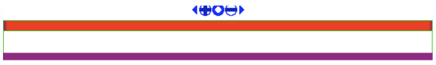

Name	From	To	Name	From	To	Class	Dir	Sim	Pos/Mm:Ts	Score
/tmp/censor.99432.tmp/data.ori	2	4888	VANDAL6	1	4828	DNA/MuDR	d	0.9635	1.9452	37004
/tmp/censor.99432.tmp/data.ori	4889	7976	VANDAL6	5302	8390	DNA/MuDR	d	0.9666	1.9167	24863

Partial

Confirmed

Chr2 11608326 11608326 - 1 Chr5;19152829;19160826;VANDAL21 m64079_221220_112036/4129671/ccs met1_01

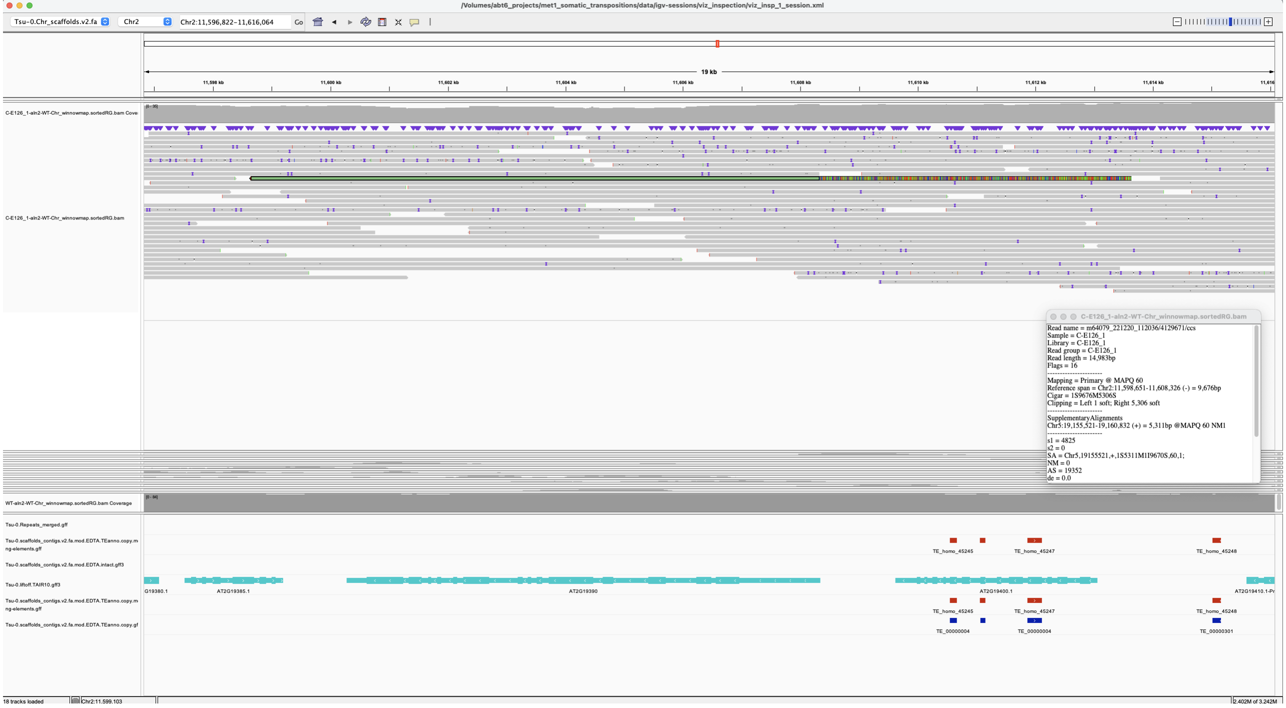

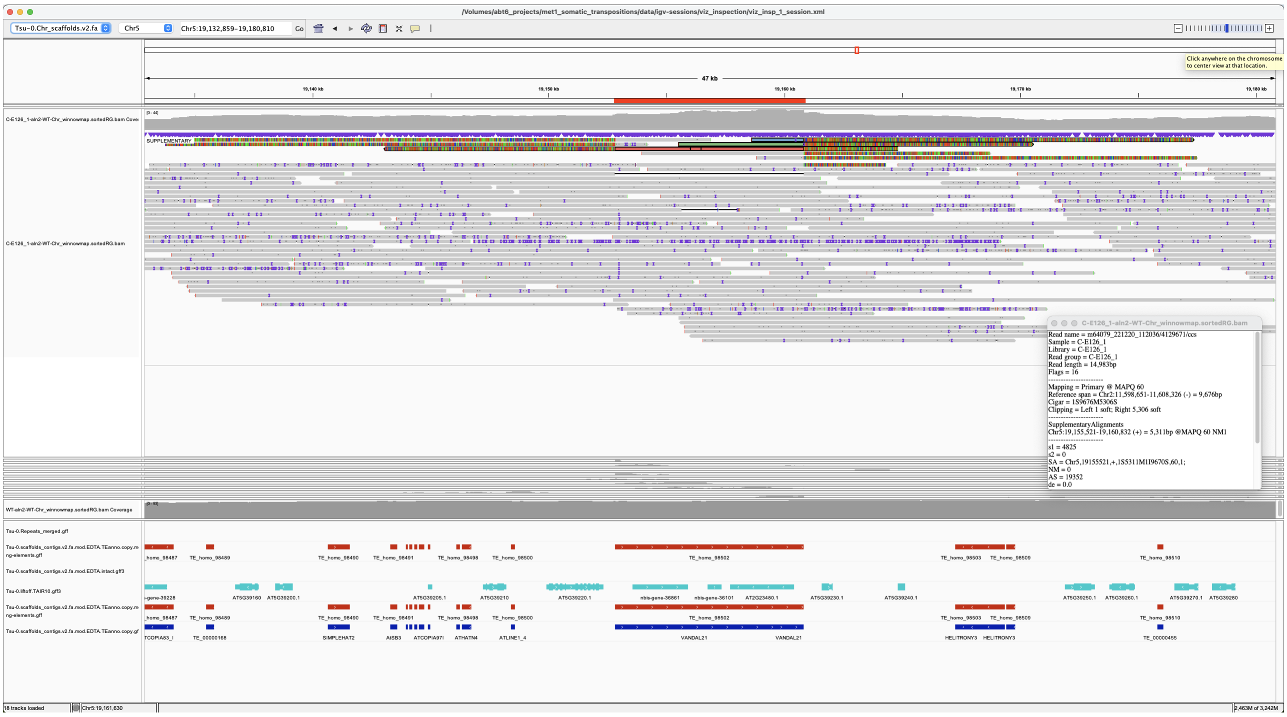

Partial

Confirmed

Chr2 12106042 12106042 + 1 Chr1;11941102;11946441;ATCOPIA93_Evade m64079_240212_113350/61409445/ccs met1_01

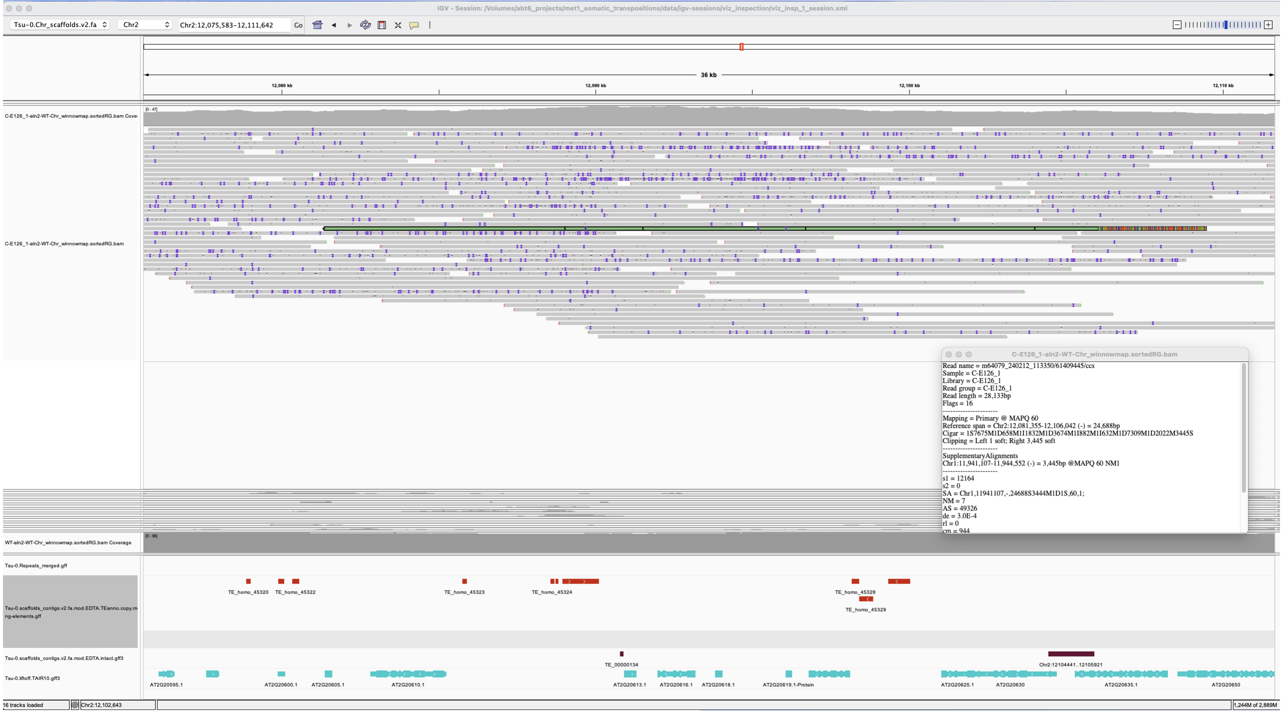

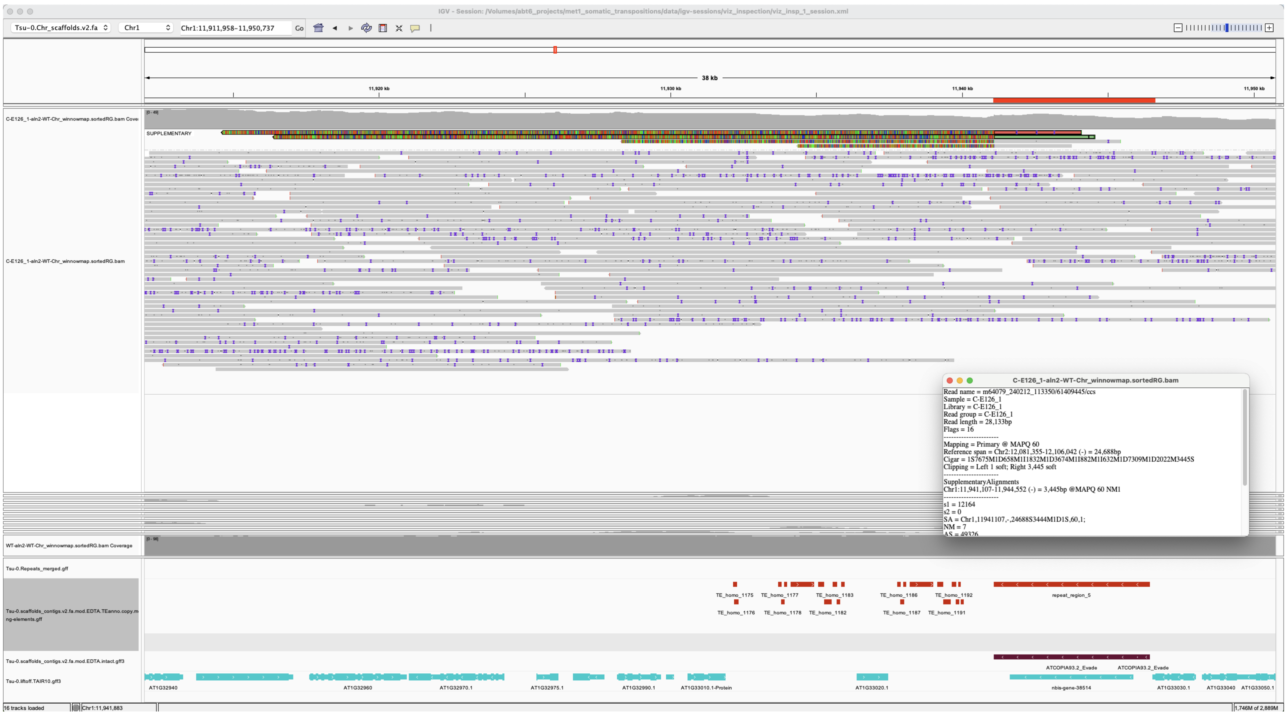

Partial

Confirmed

Chr3 4975400 4975400 - 1 Chr5;19152830;19159575;VANDAL21 m64079_221220_112036/104726604/ccs met1_01

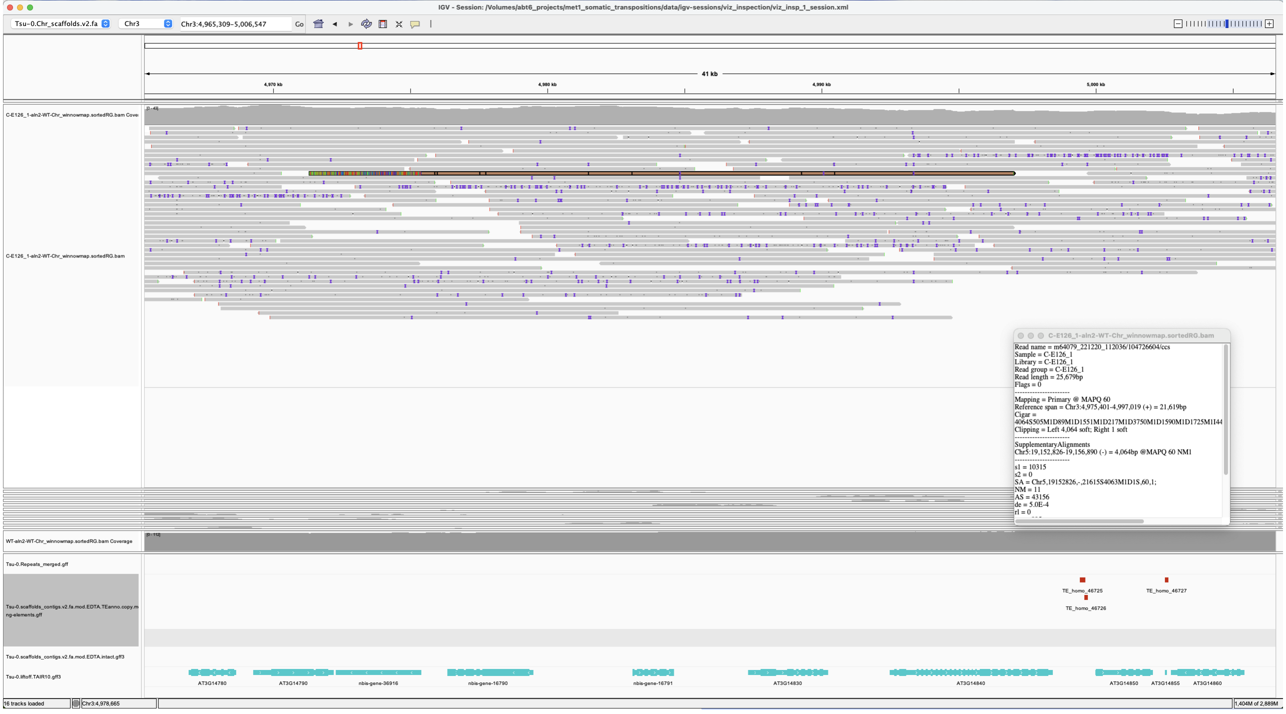

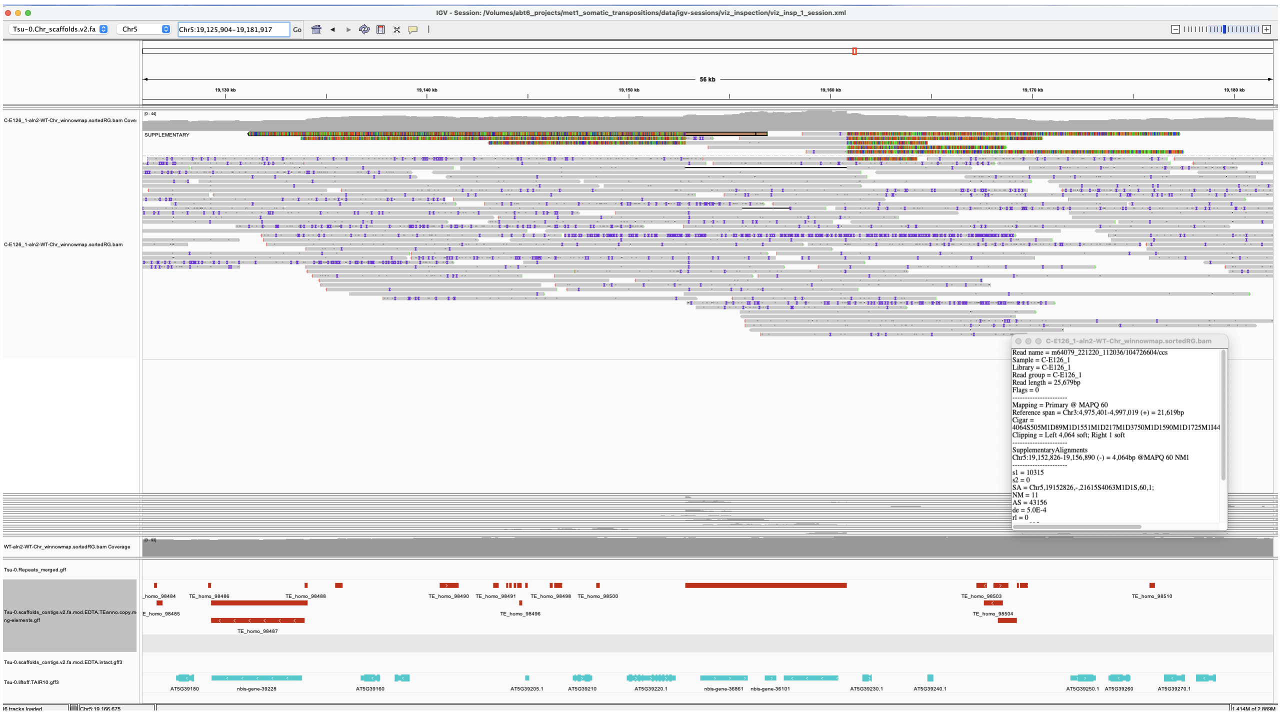

Partial

Confirmed

> Chr3 17324910 17324910 + 1 Chr5;19152829;19160826;VANDAL21 m64079_240212_113350/17236131/ccs met1_01

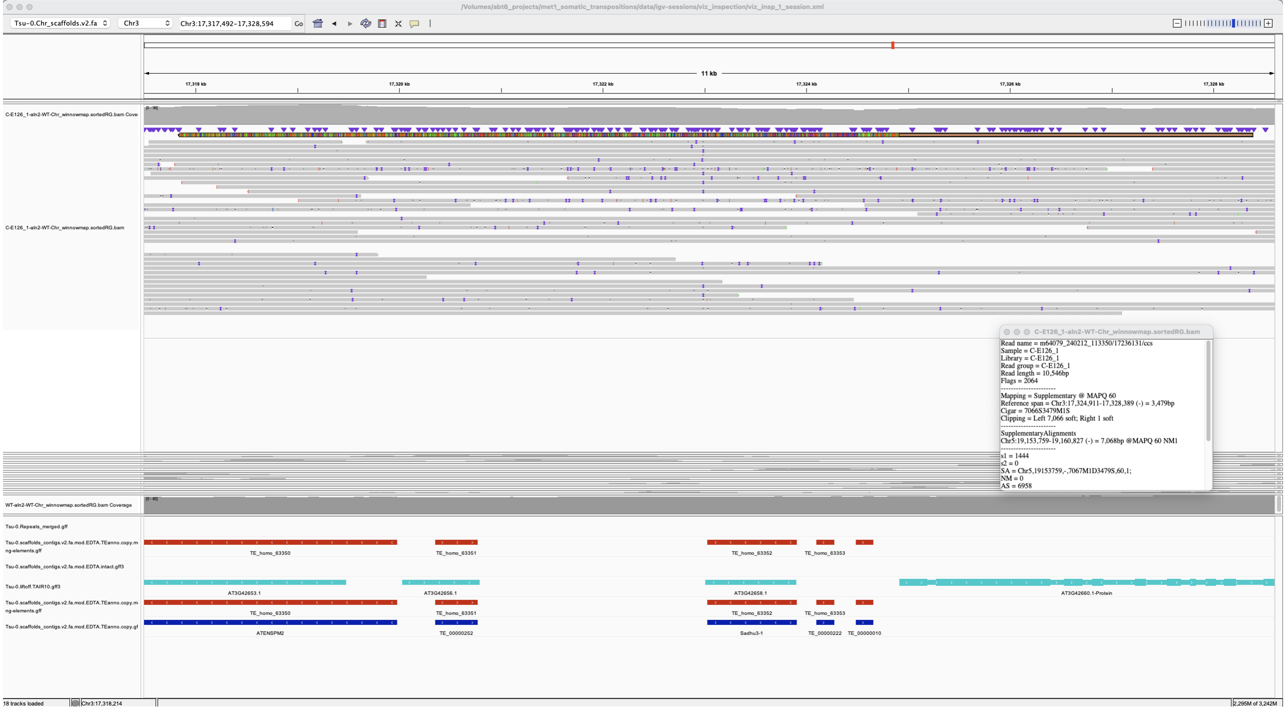

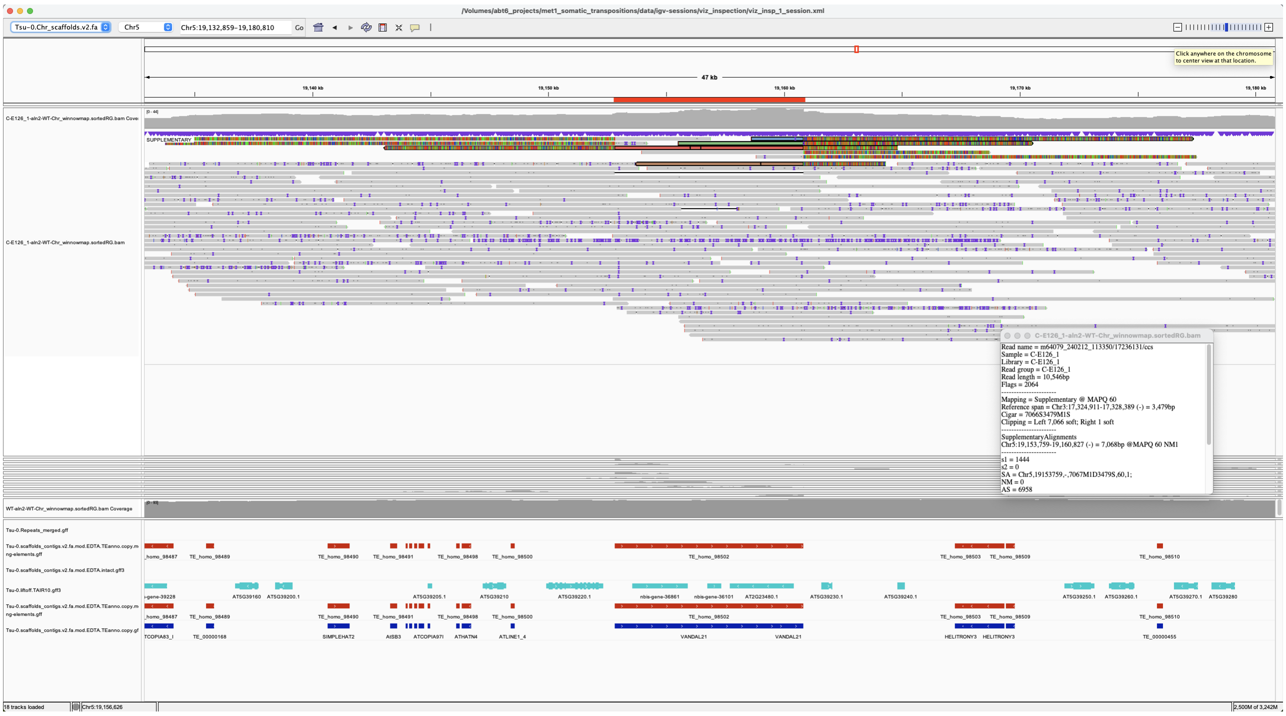

Partial

Confirmed

| Chr3 17758419 17758419 + 1 Chr5;19152829;19160826;VANDAL21 m64079_221220_112036/15204508/ccs met1_01

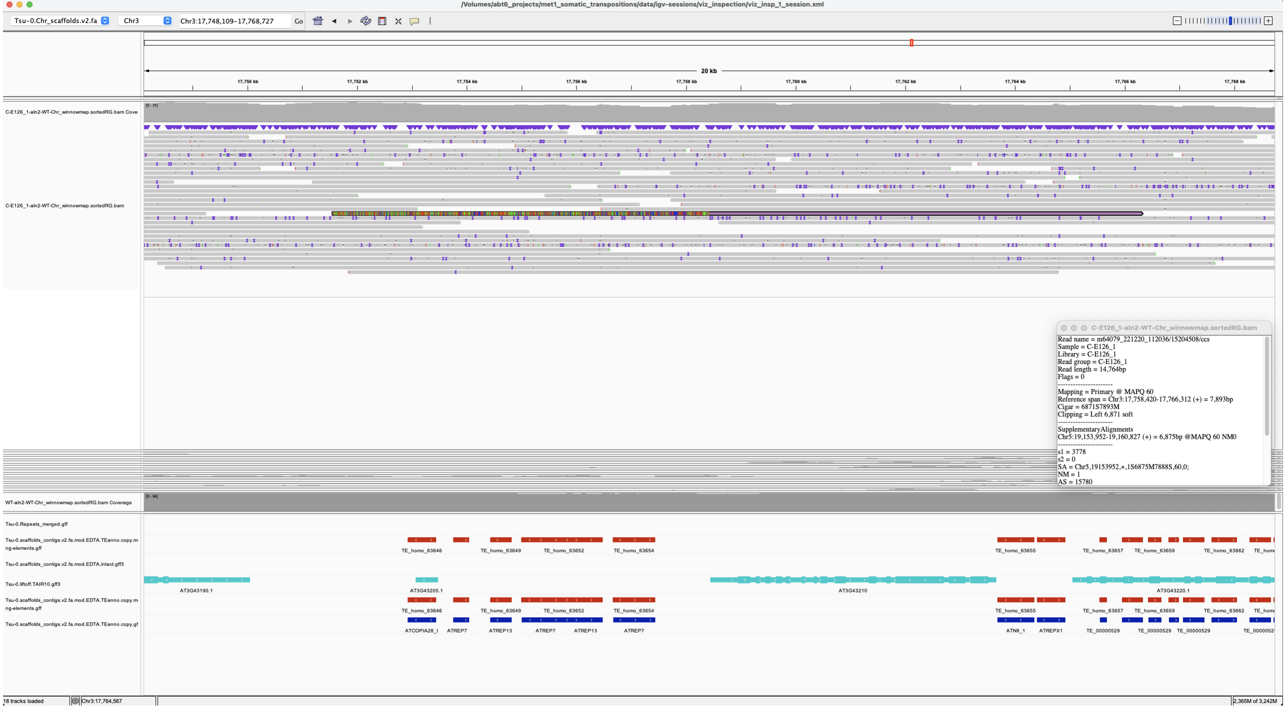

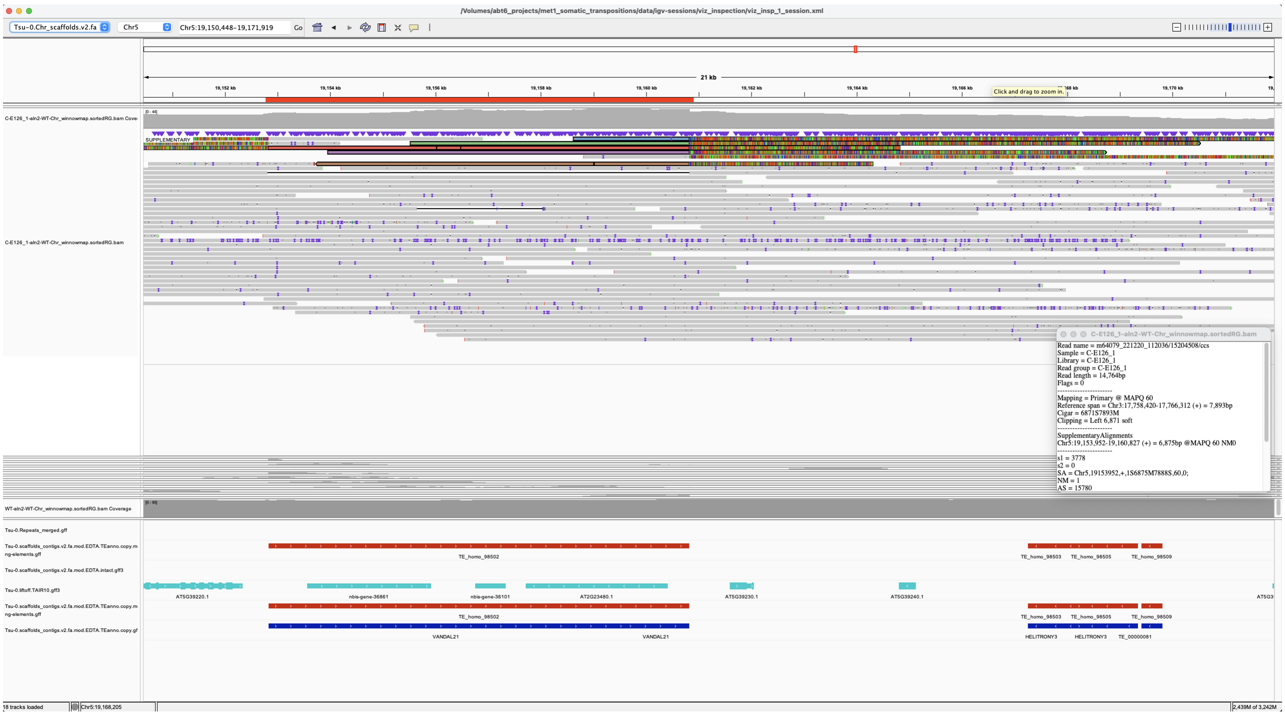

Partial

Confirmed

Chr3 21862257 21862257 - 1 Chr1;11941102;11946441;ATCOPIA93_Evade m64079_240212_113350/121307639/ccs met1_01

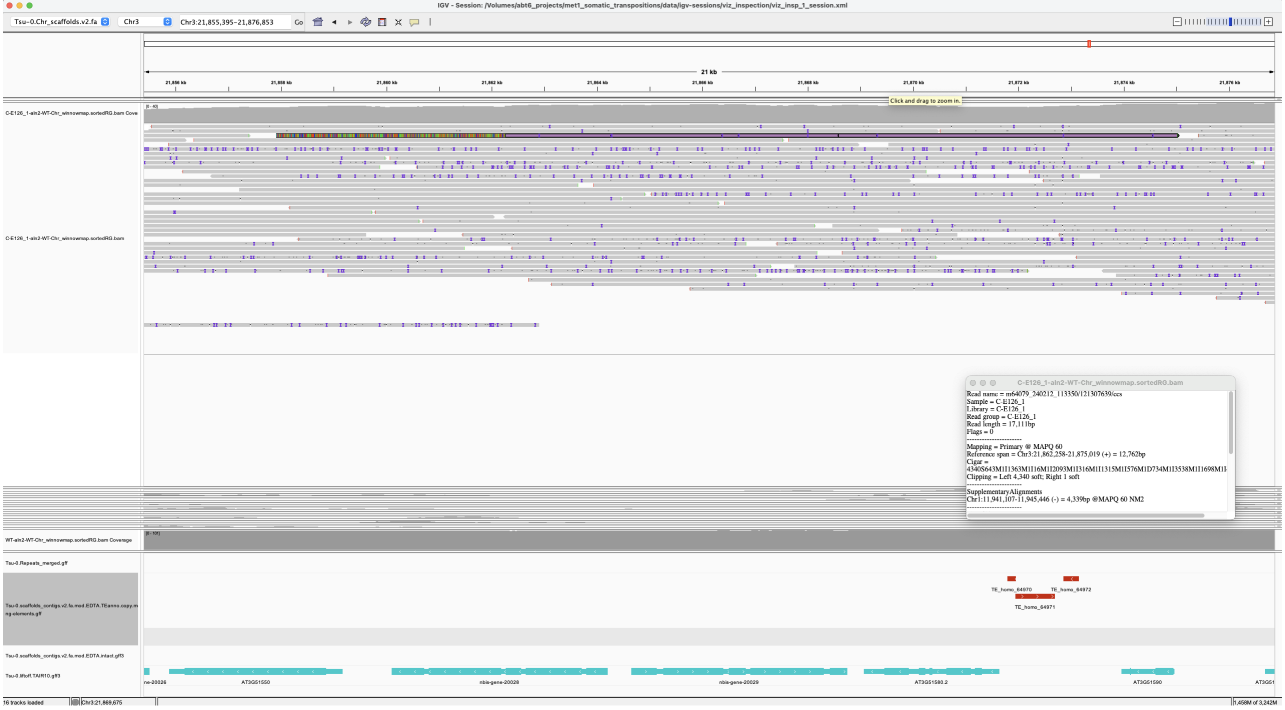

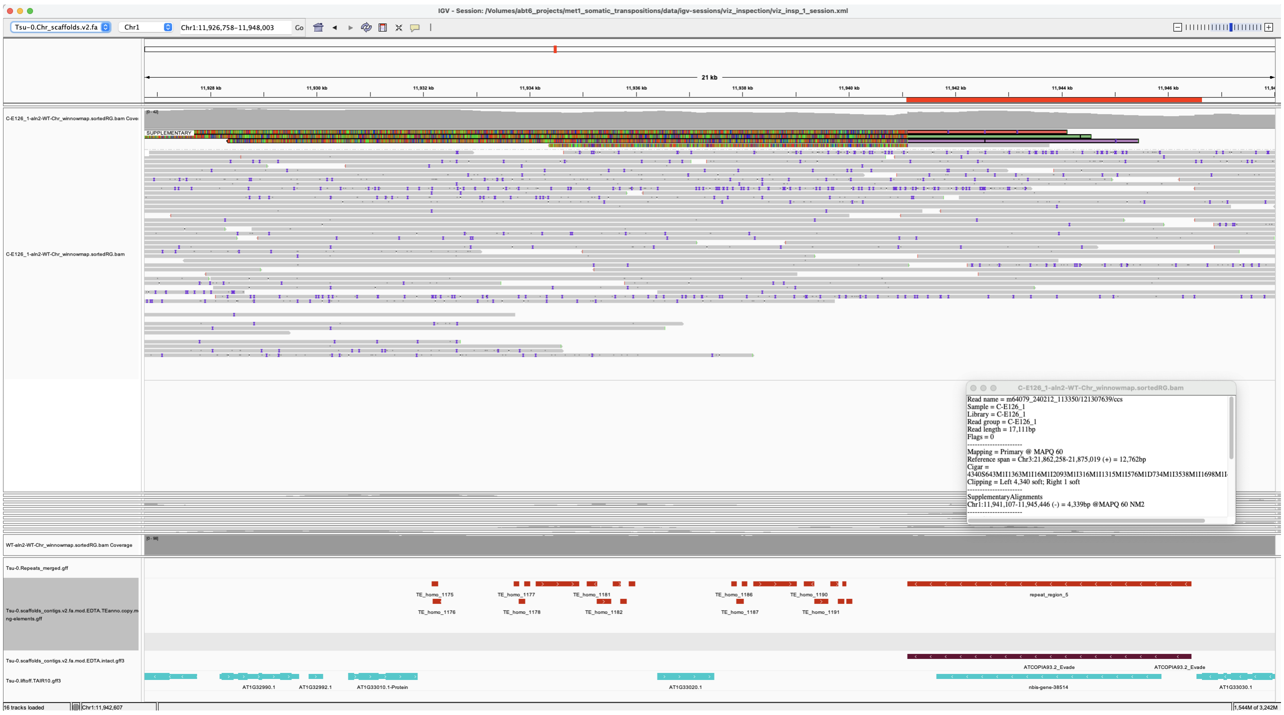

Partial

Confirmed

Chr5 19884281 19884281 + 1 Chr5;19872565;19877095;VANDAL21 m64079_240212_113350/84738381/ccs met1_01

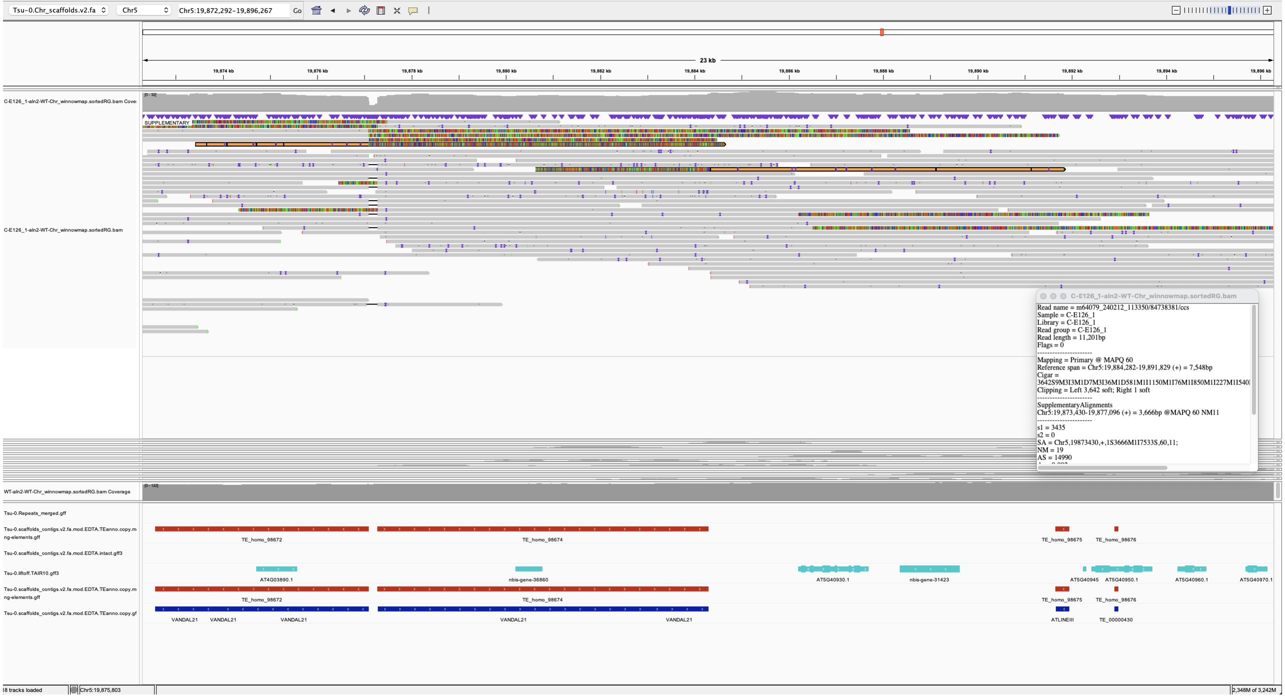

PROBABLY JUST A REARRANGEMENT!!

Hypermutable region

unsupported

Chr5 21335378 21335378 - 1 Chr5;19152829;19160826;VANDAL21 m64079_221220_112036/134611320/ccs met1_01

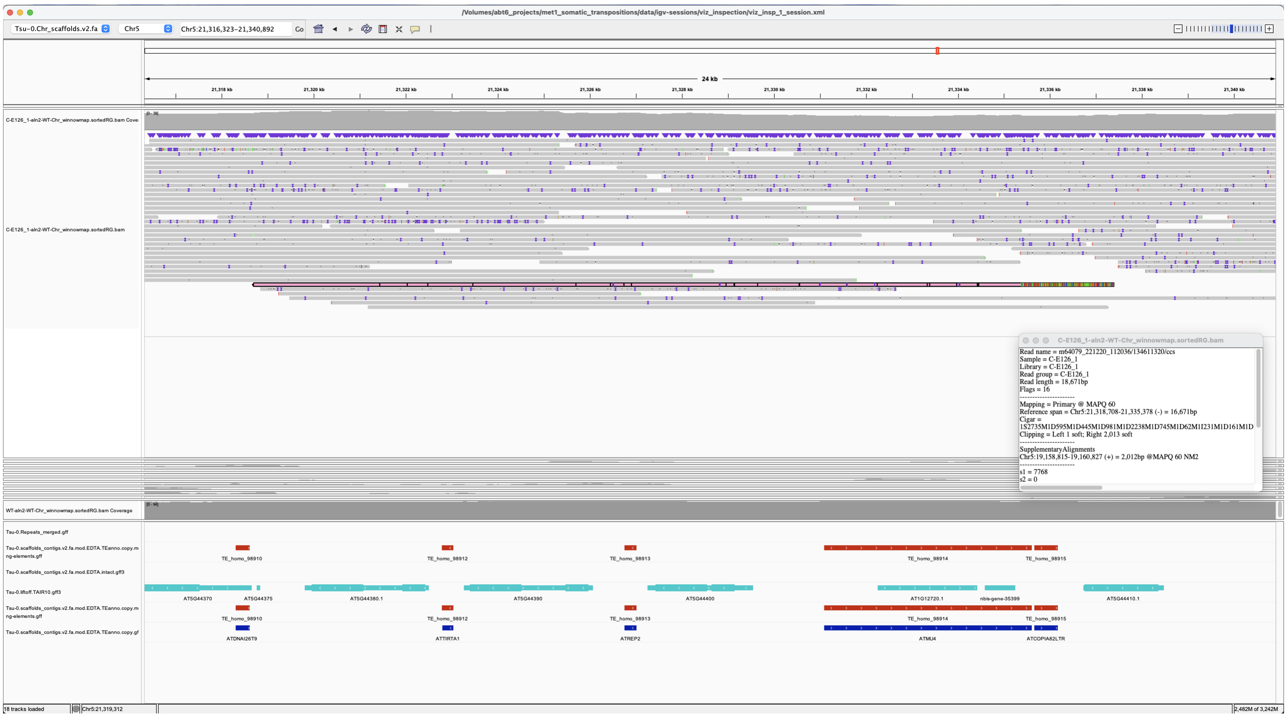

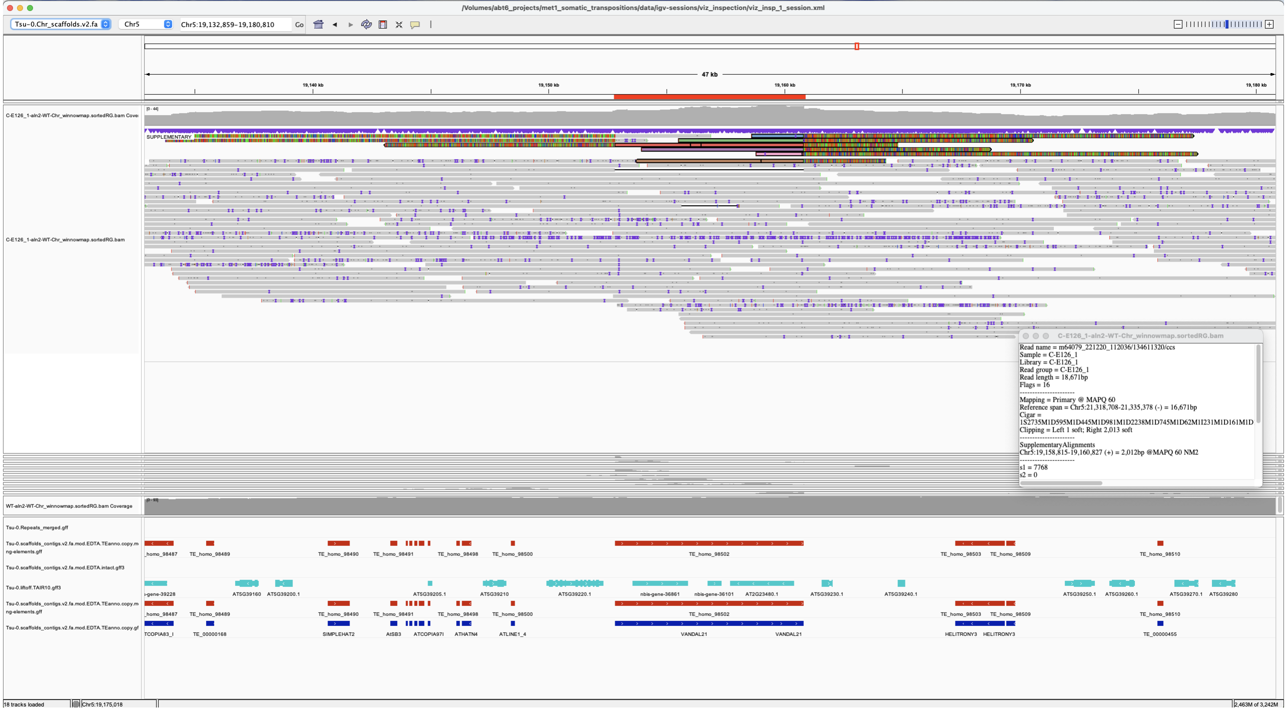

Partial

Confirmed

> Chr5 25216766 25216766 + 1 Chr5;19152830;19159575;VANDAL21 m64079_221220_112036/41550822/ccs met1_01

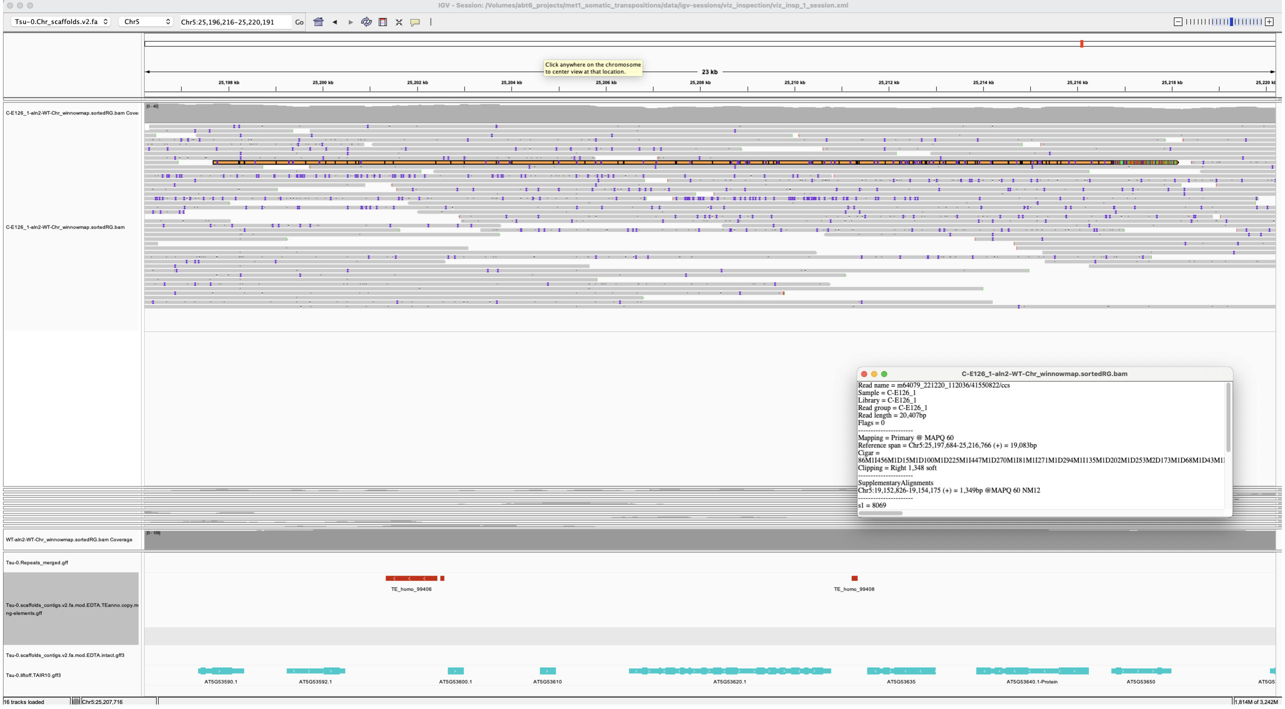

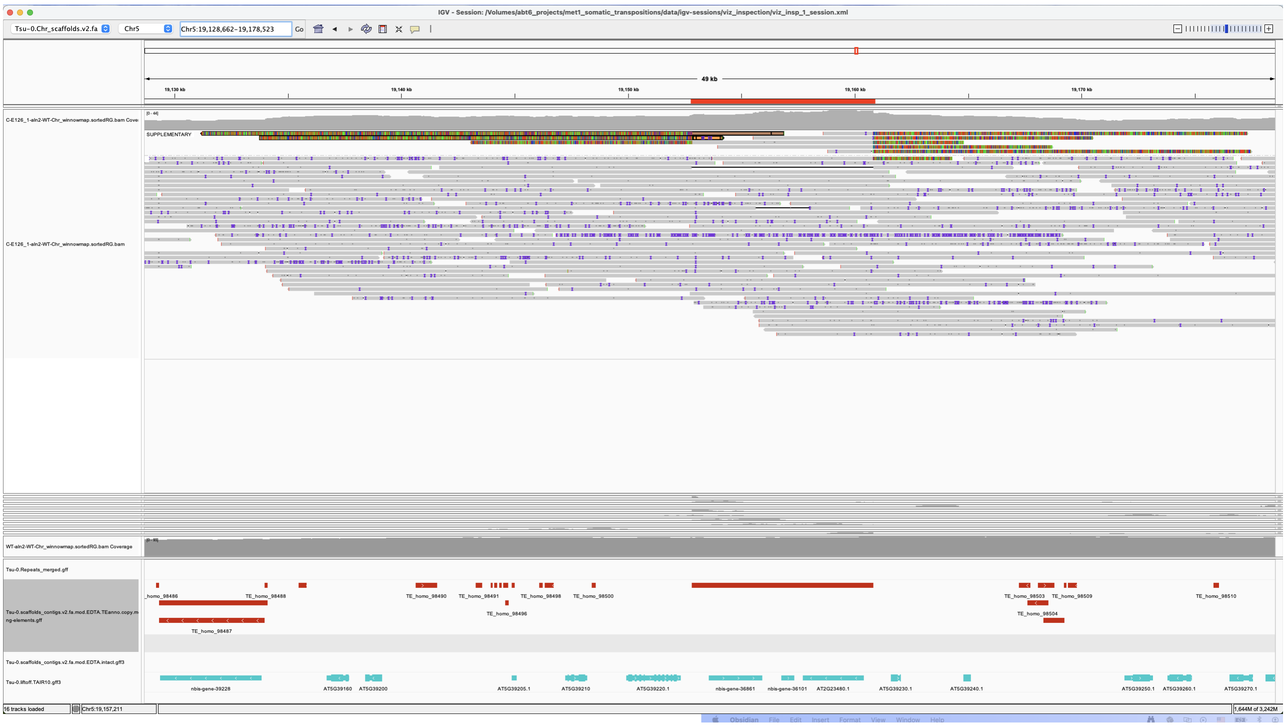

Partial

Confirmed

---

met1_02

> Chr1 4892937 4892937 + 1 Chr5;19152829;19160826;VANDAL21 m64079_221220_112036/121244451/ccs met1_02

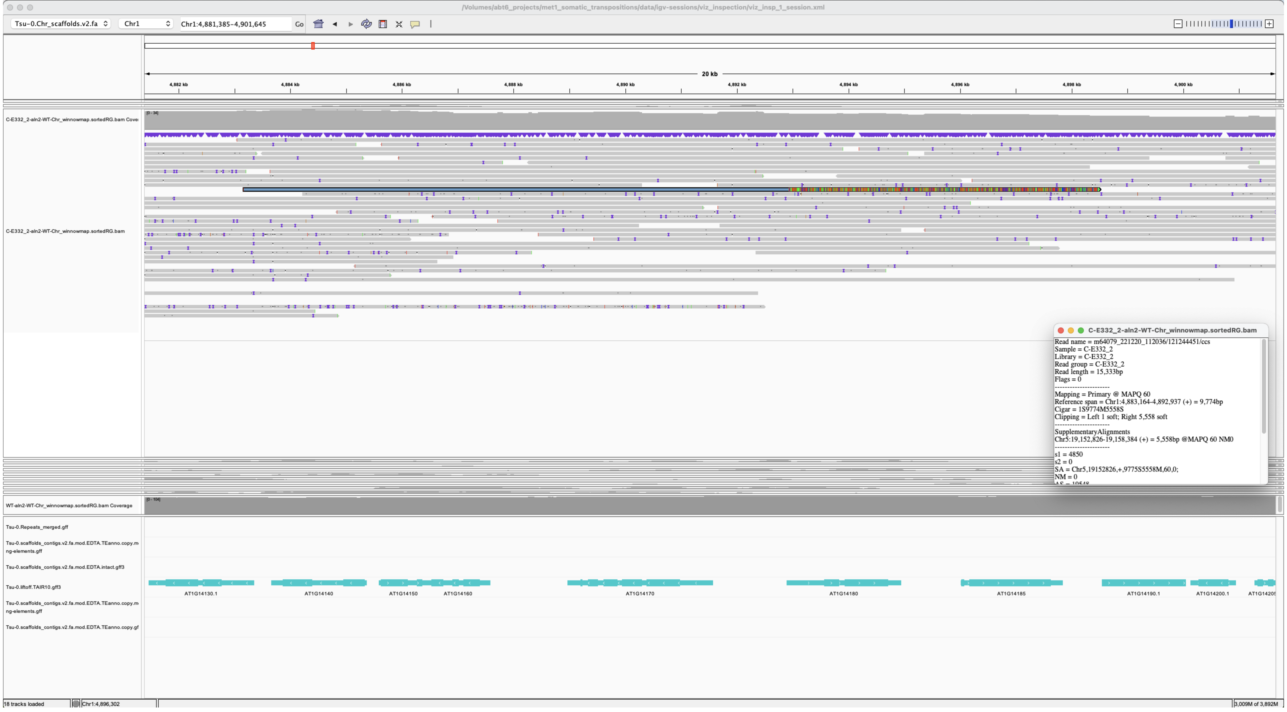

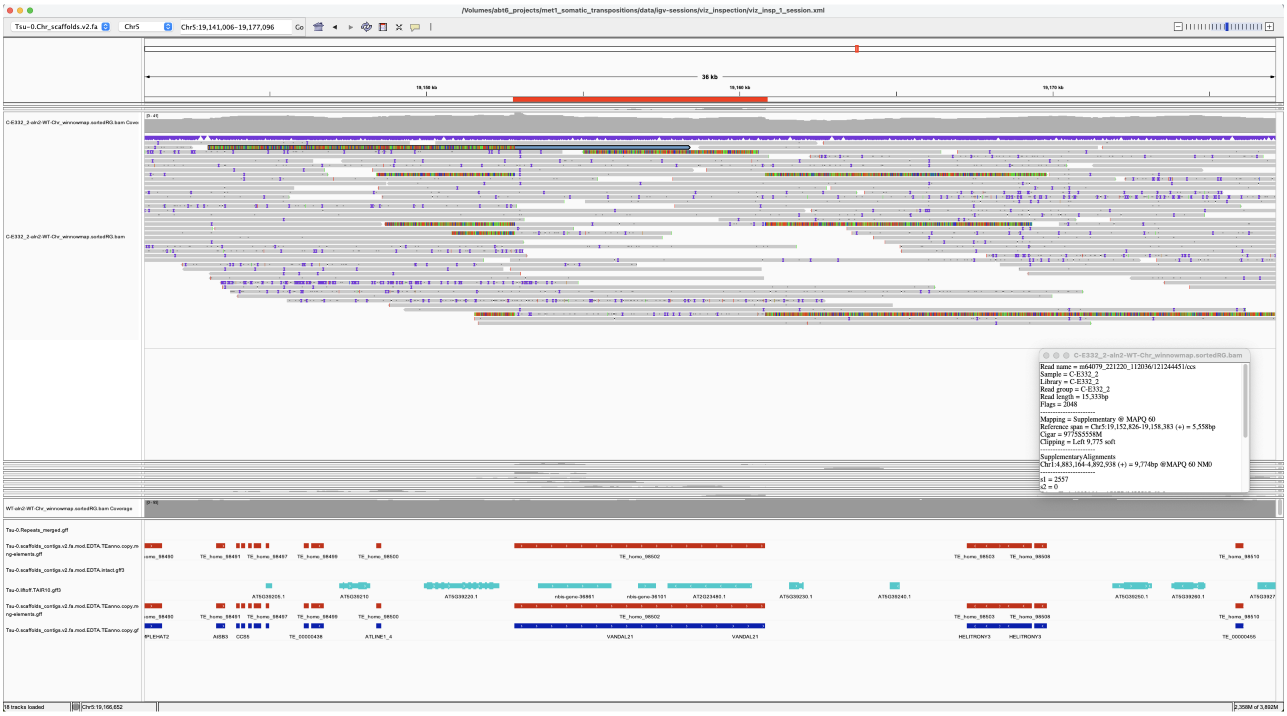

Partial

Confirmed

Chr1 5933328 5933328 + 1 Chr1;11941106;11946436;ATCOPIA93_Evade m64079_221220_112036/21432255/ccs met1_02

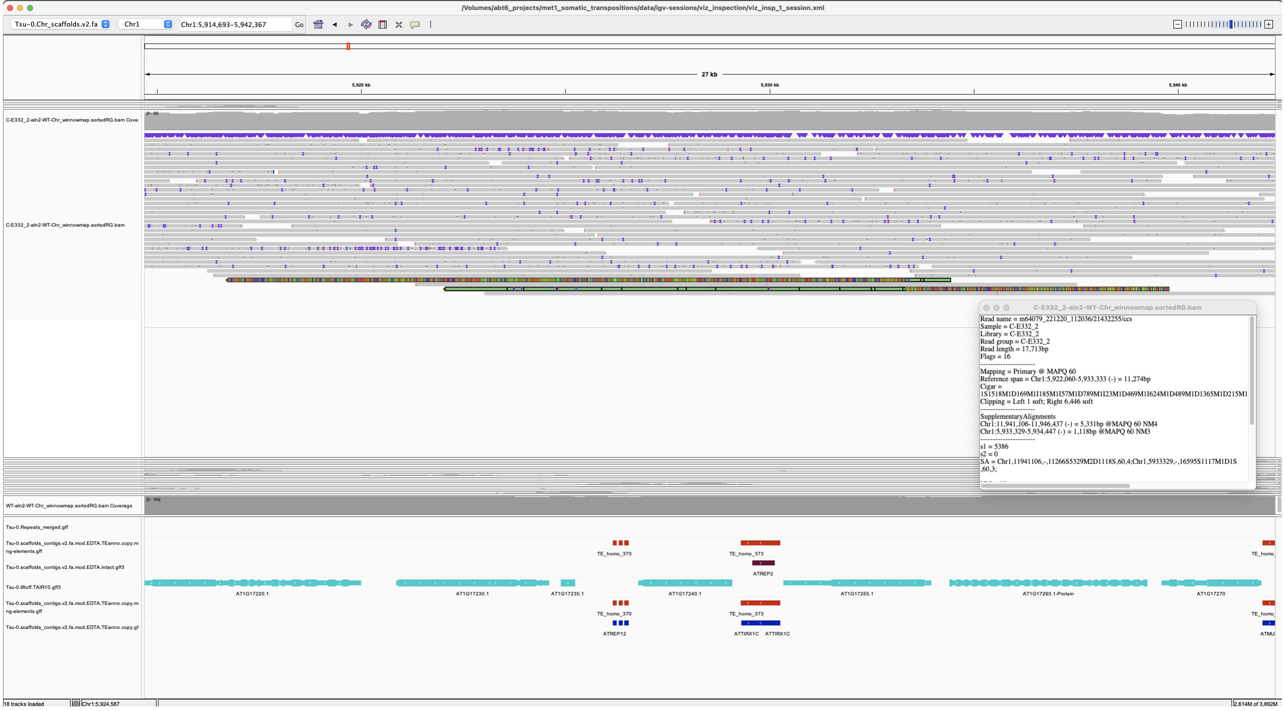

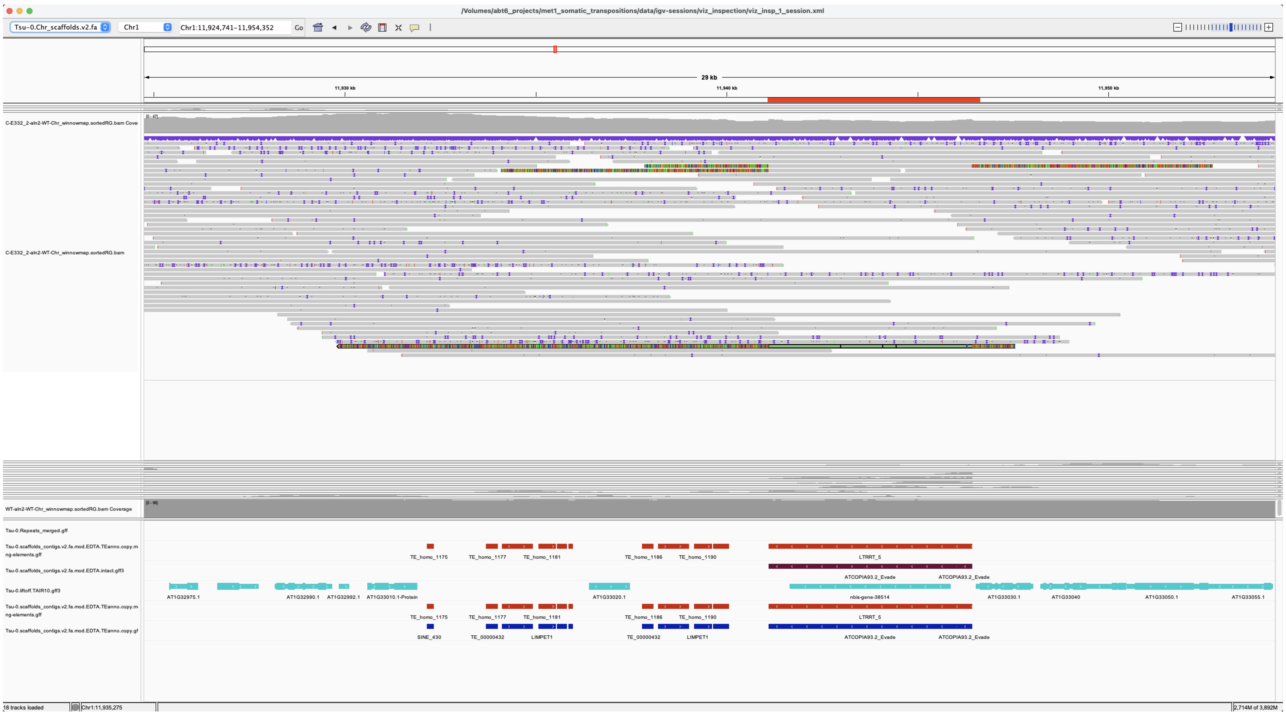

Central
TSD
Confirmed

Chr1 27873875 27873875 + 1 Chr1;11941106;11946436;ATCOPIA93_Evade m64079_221220_112036/123864804/ccs met1_02

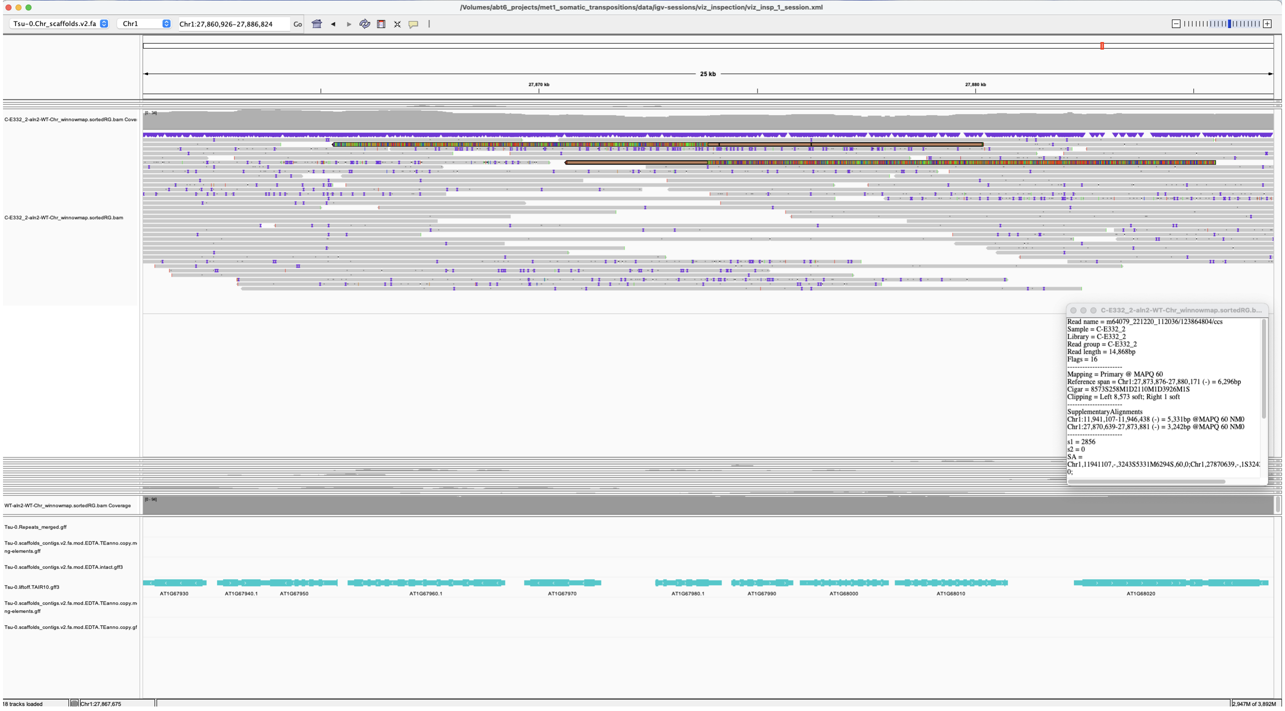

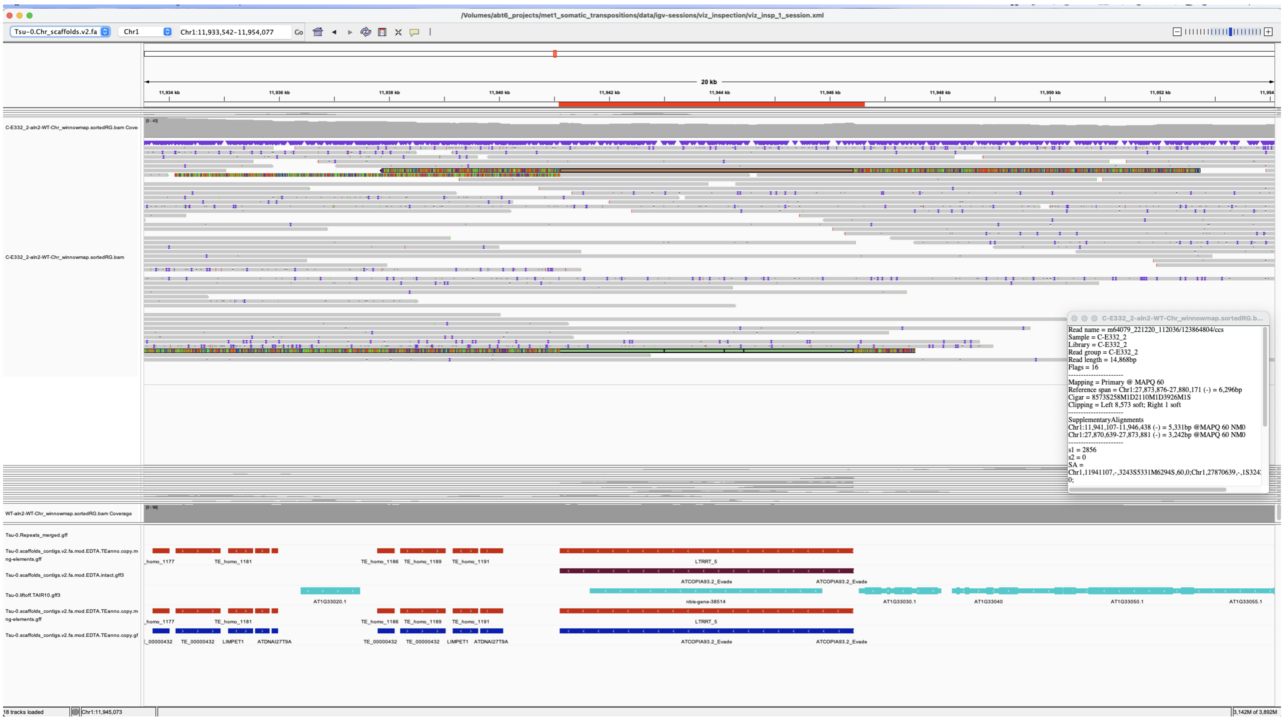

Central

TSD

Confirmed

Chr1 30871622 30871622 - 1 Chr3;16344522;16352497;VANDAL6 m64079_240212_113350/131334460/ccs met1_02

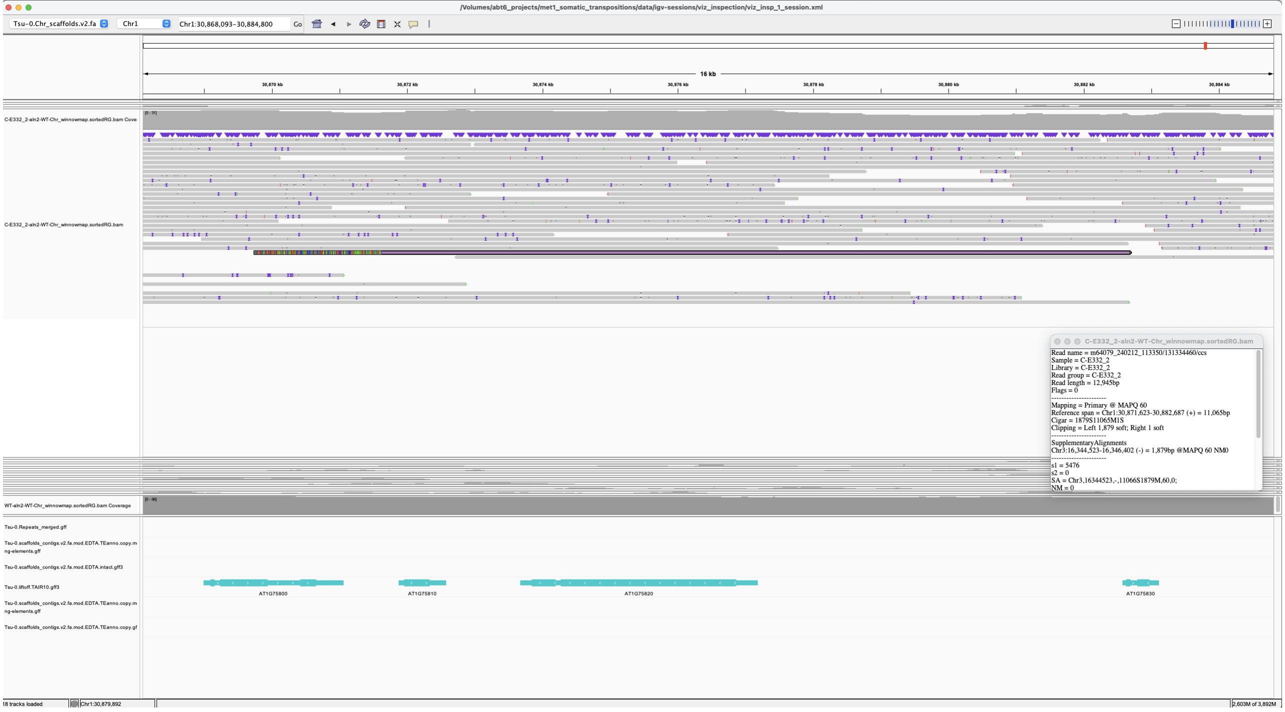

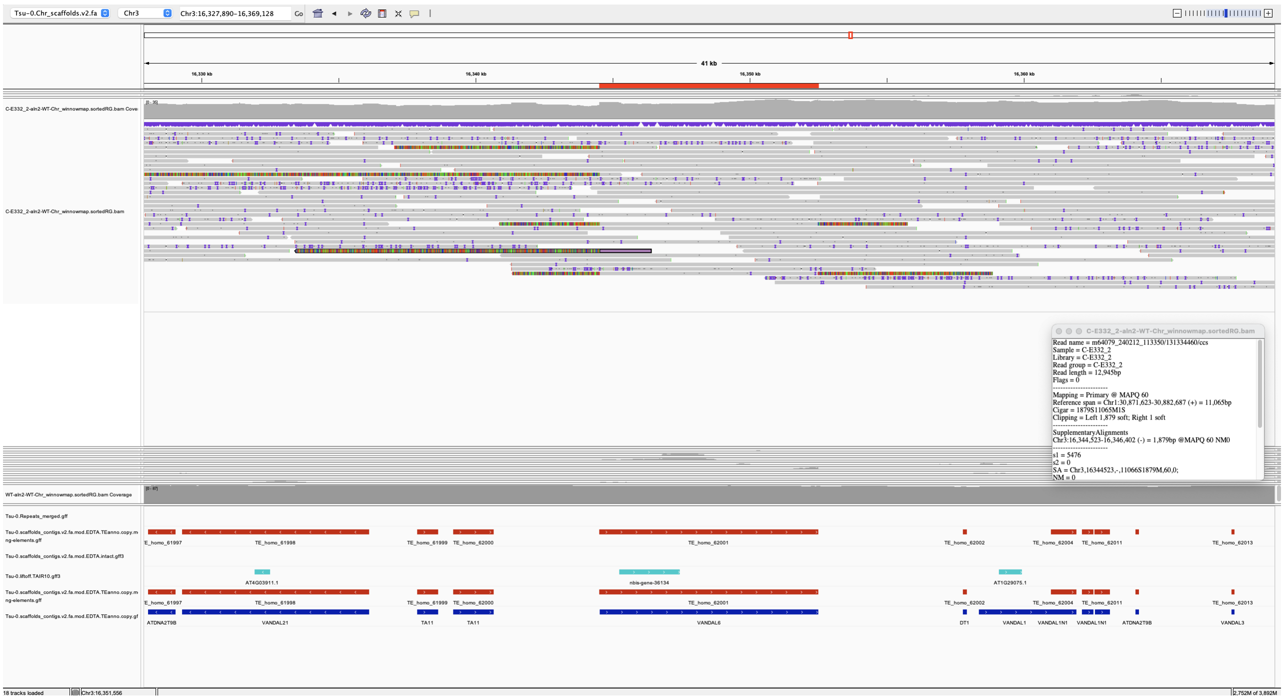

Partial

Confirmed

> Chr2 14466667 14466667 - 1 Chr3;16344522;16352497;VANDAL6 m64079_240212_113350/25559494/ccs met1_02

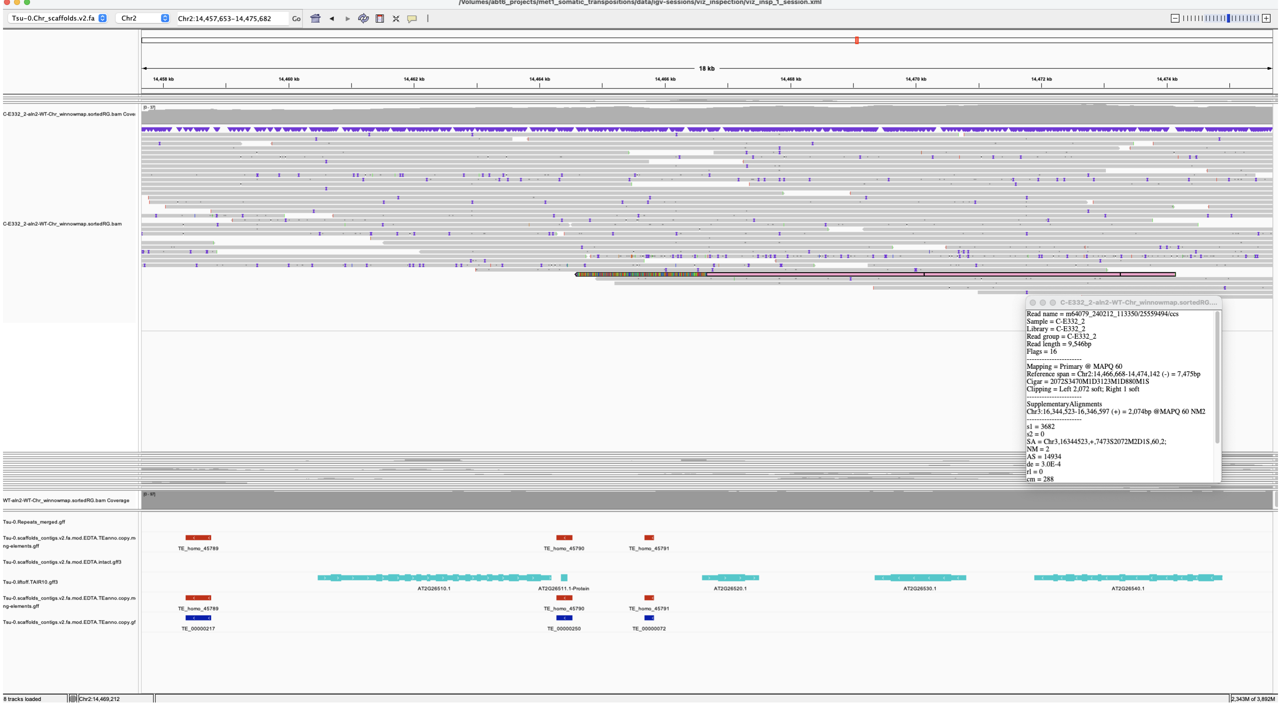

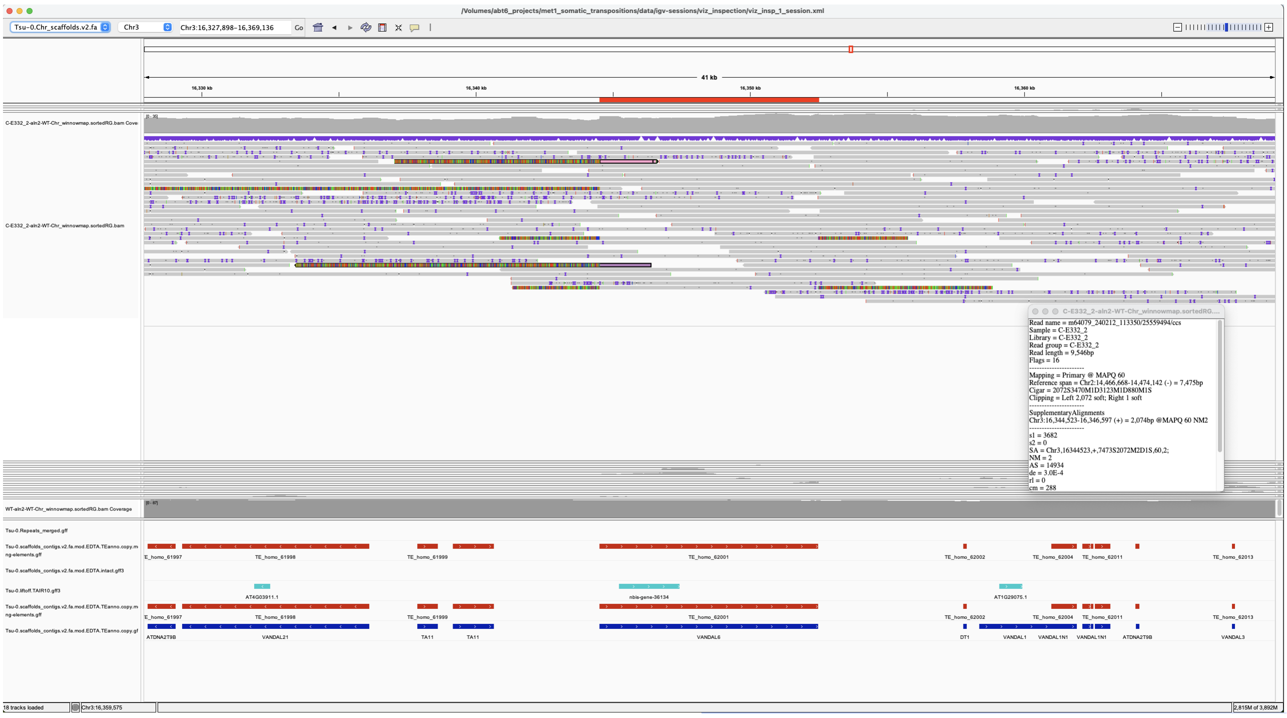

Partial

Confirmed

Chr2 17989858 17989858 - 1 Chr3;20158137;20166150;VANDAL6 m64079_221220_112036/330310/ccs met1_02

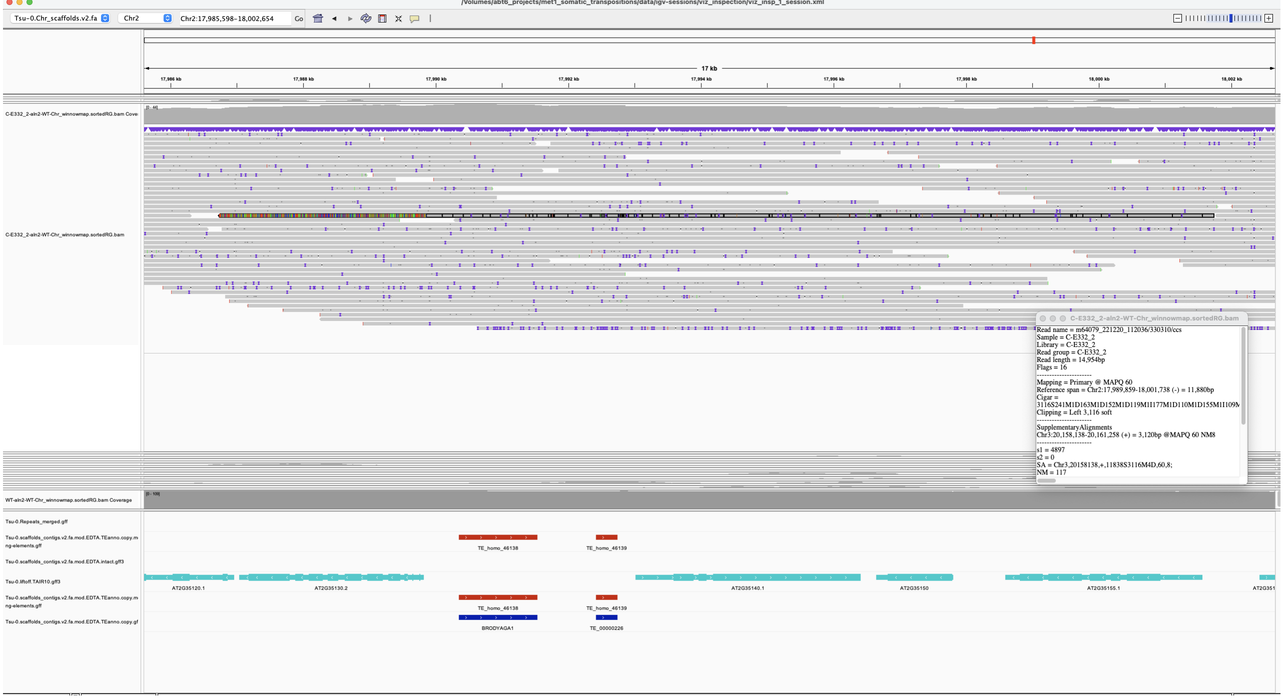

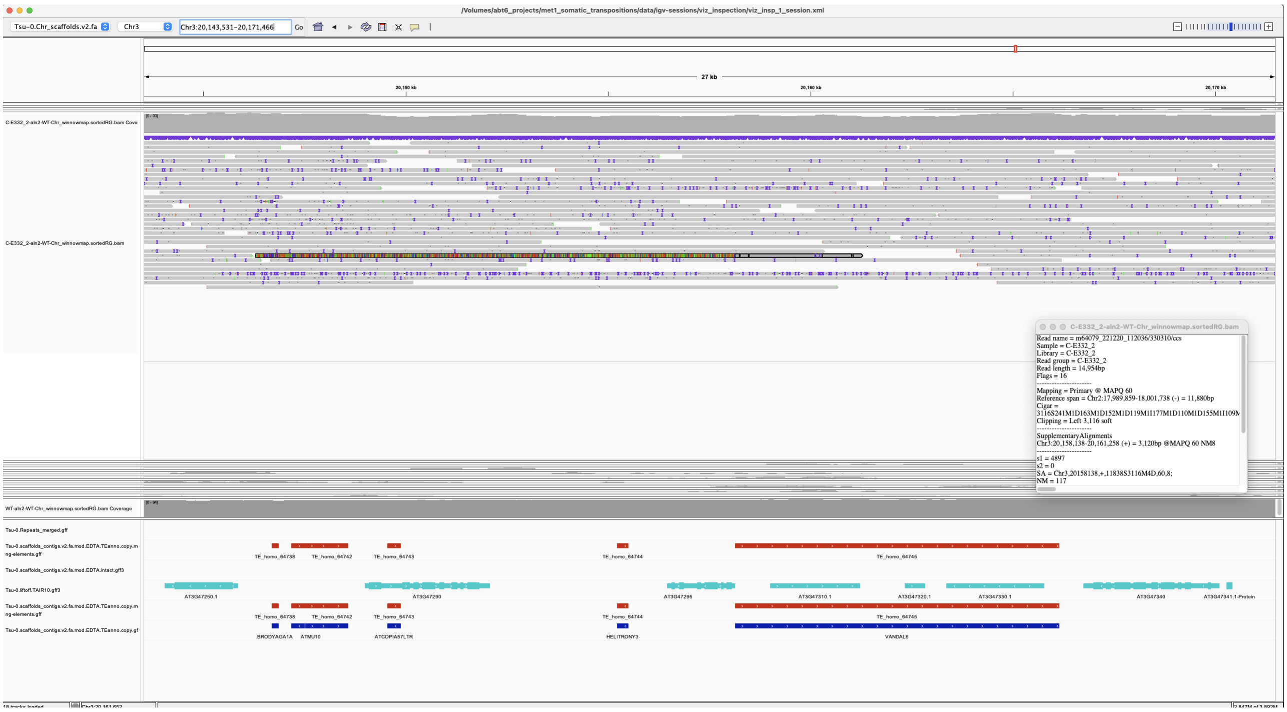

Partial

Confirmed

Chr2 22416805 22416805 + 1 Chr3;16344522;16352497;VANDAL6 m64079_221220_112036/139002086/ccs met1_02

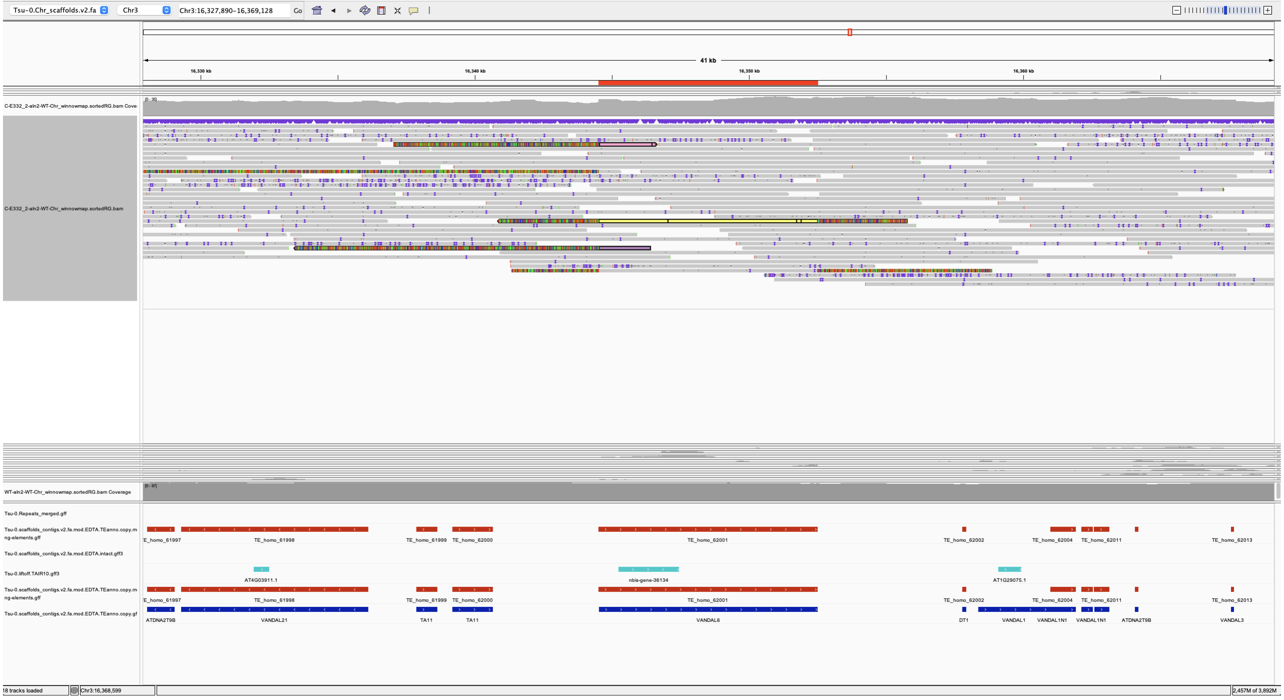

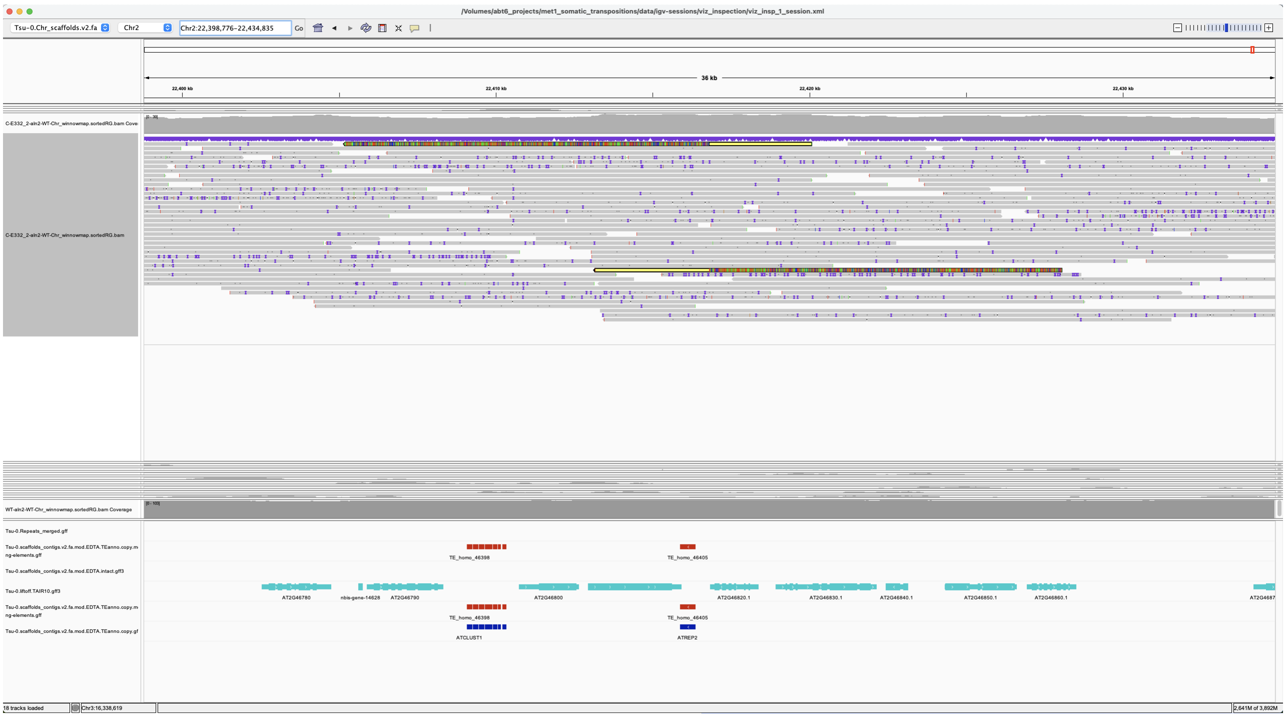

Central

TSD

Confirmed

Chr3 2816309 2816309 - 1 Chr1;11941106;11946436;ATCOPIA93_Evade m64079_240212_113350/99092948/ccs met1_02

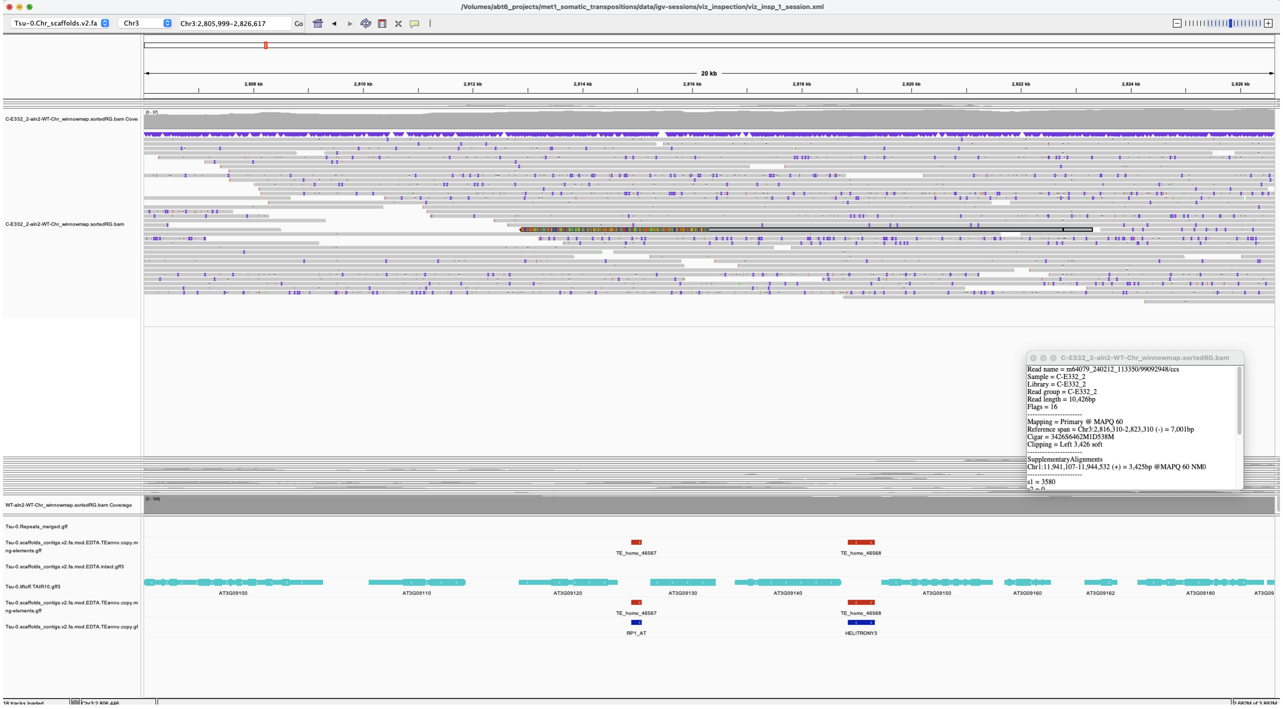

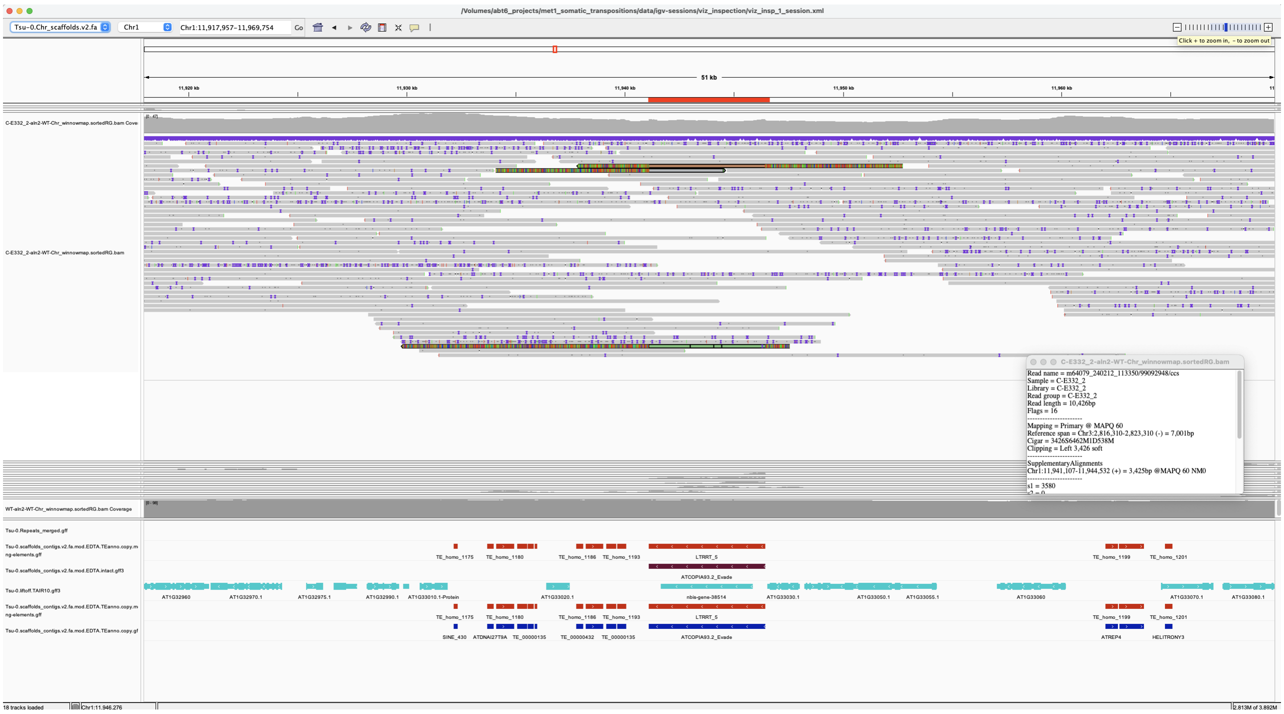

Partial

Confirmed

Chr3 8955326 8955326 + 1 Chr3;16344522;16352497;VANDAL6 m64079_221220_112036/180225706/ccs met1_02

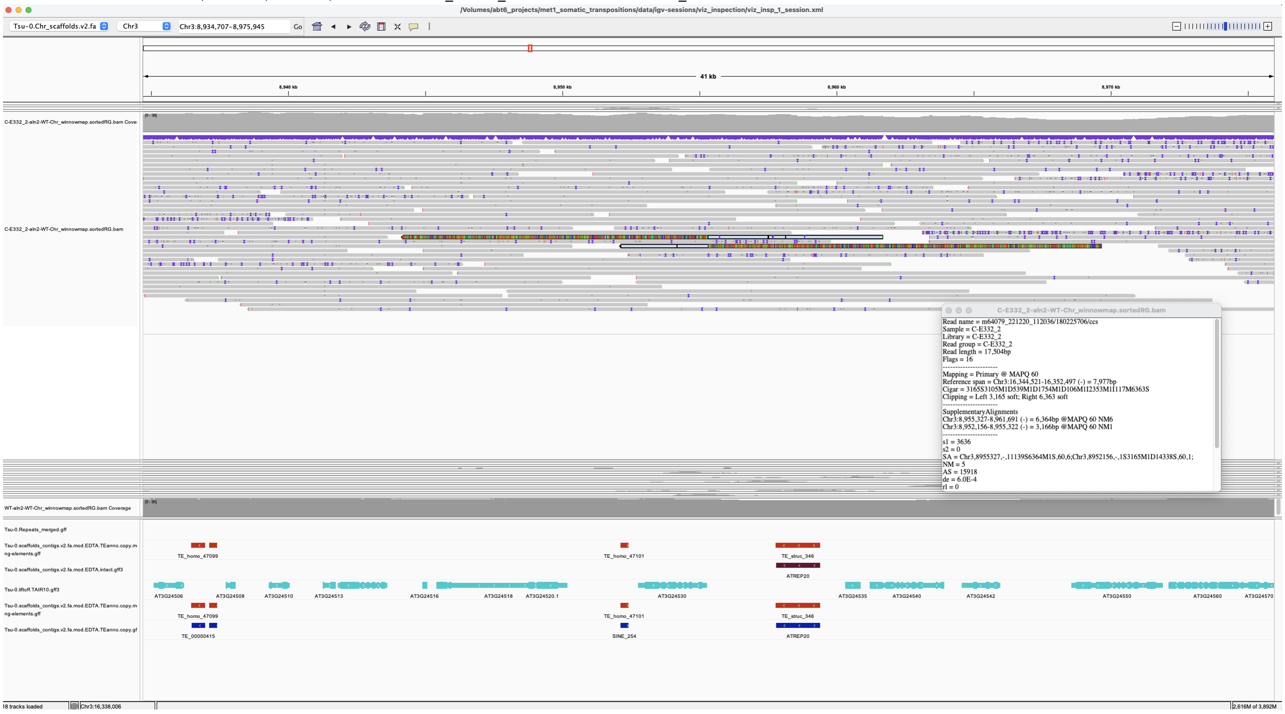

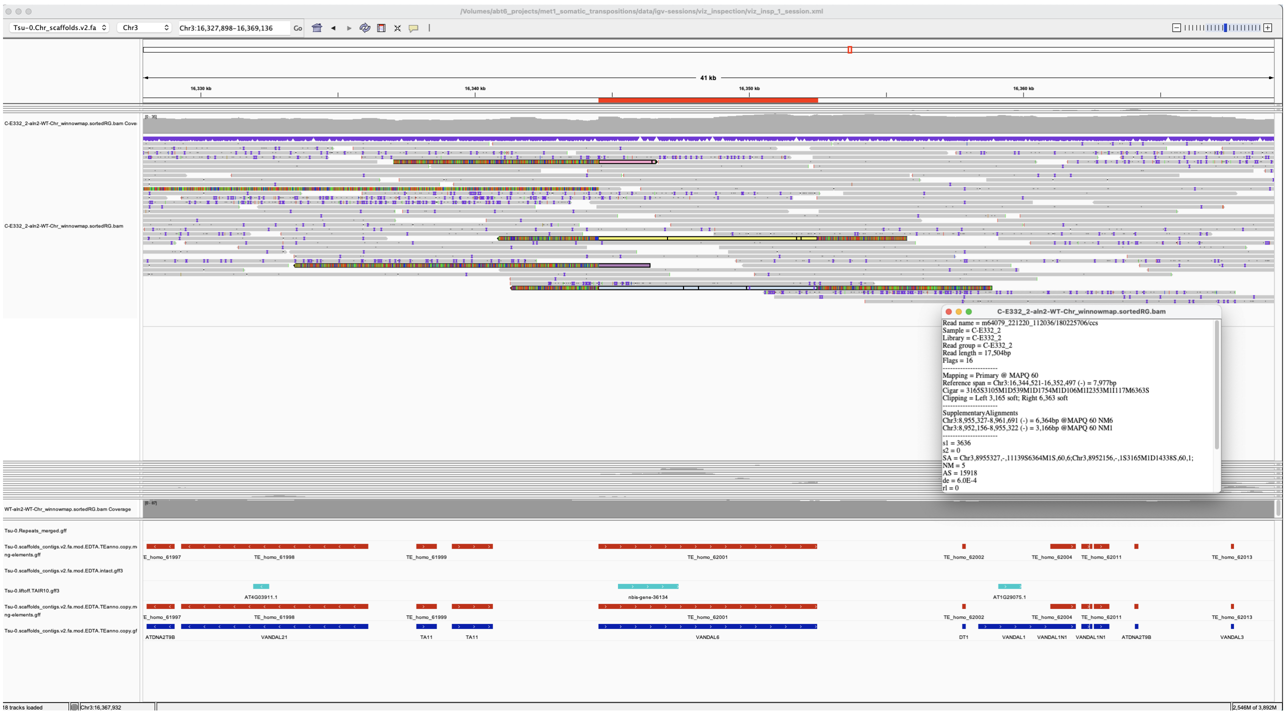

Central
TSD?

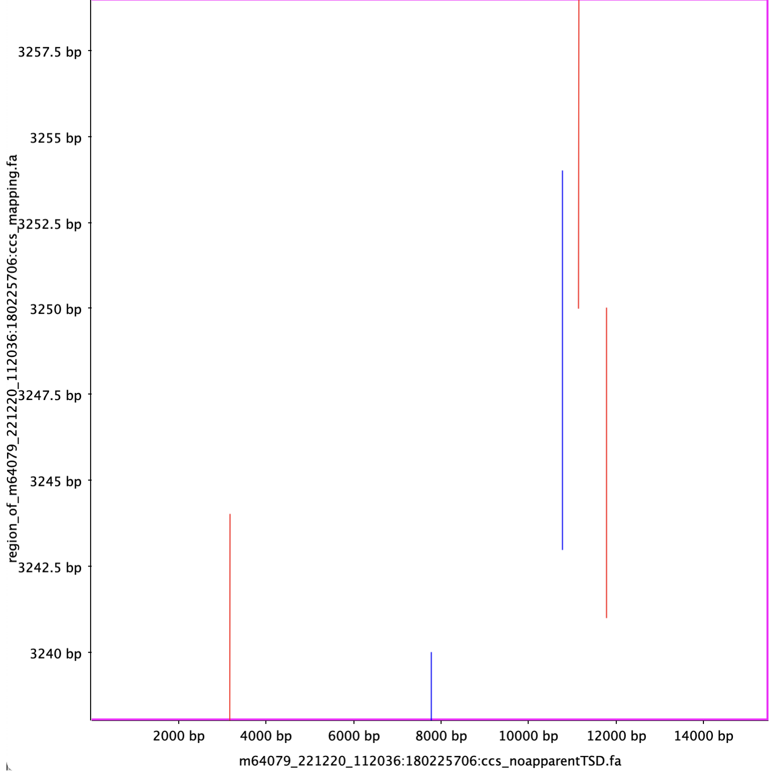

Confirmed

Chr3 18797334 18797334 + 1 Chr5;19152829;19160826;VANDAL21 m64079_240212_113350/39453910/ccs met1_02

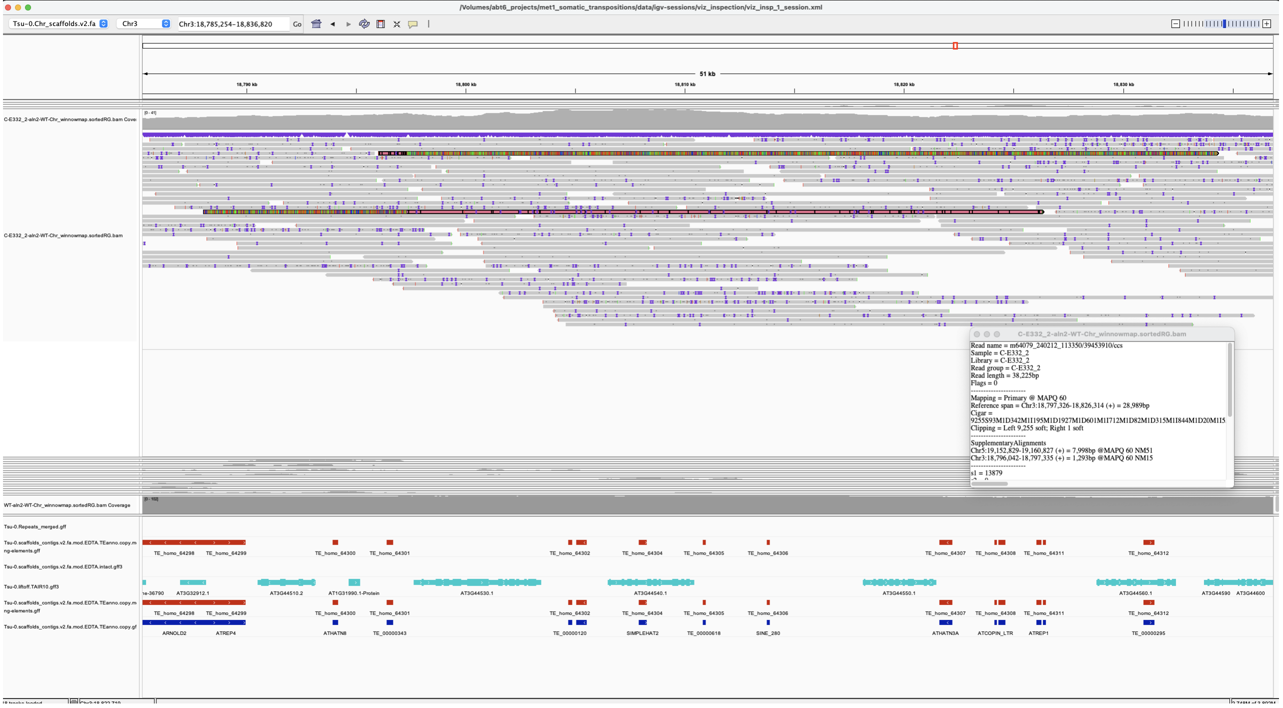

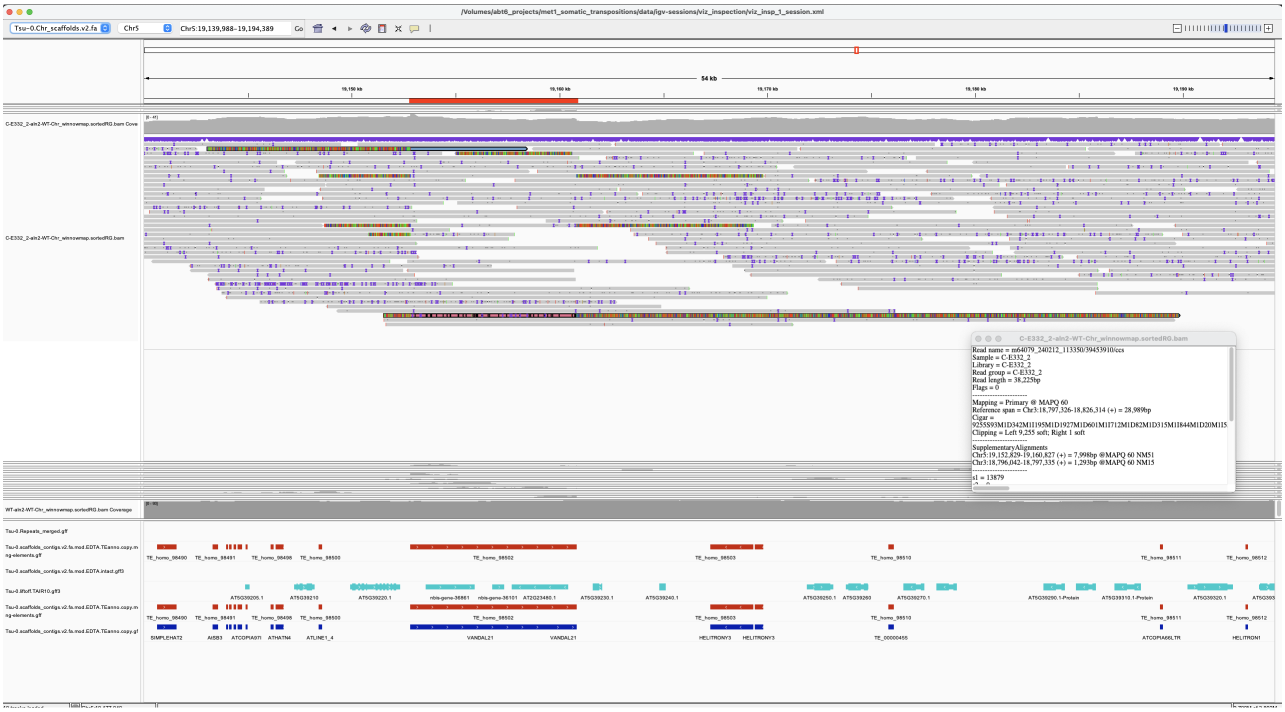

Central

TSD

Confirmed

> Chr4 586882 586882 + 1 Chr5;19152829;19160826;VANDAL21 m64079_221220_112036/120326434/ccs met1_02

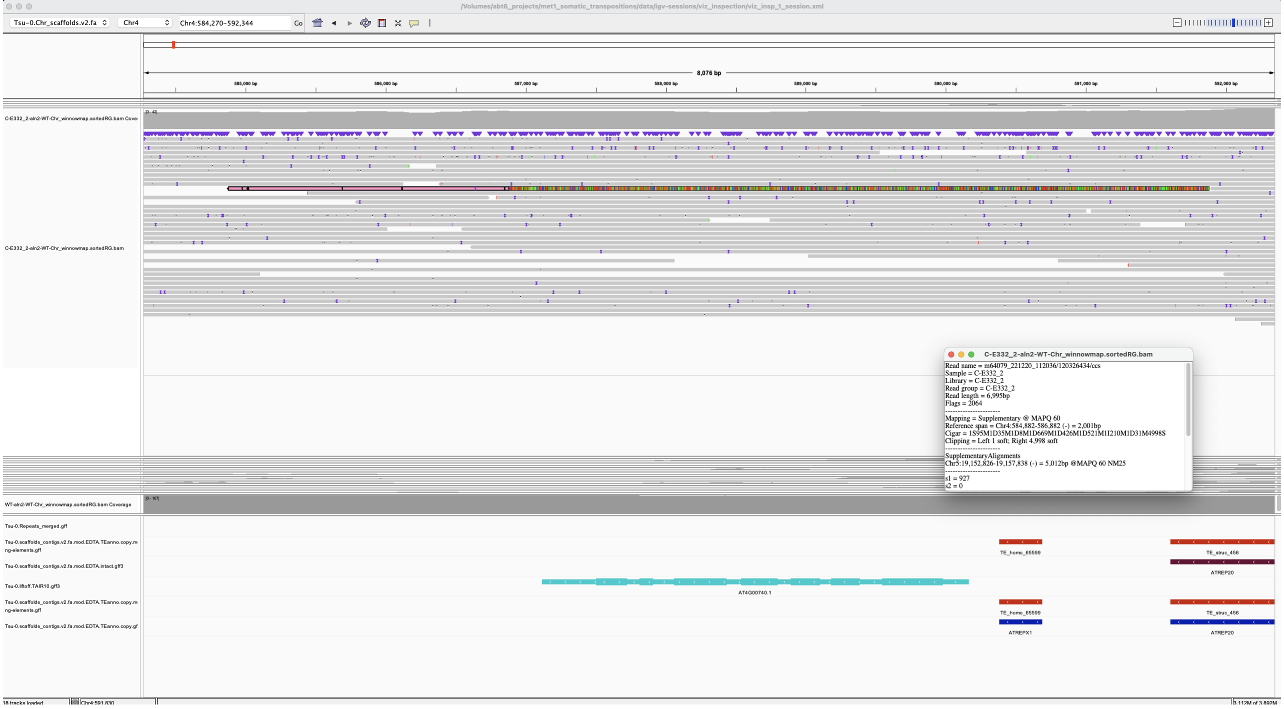

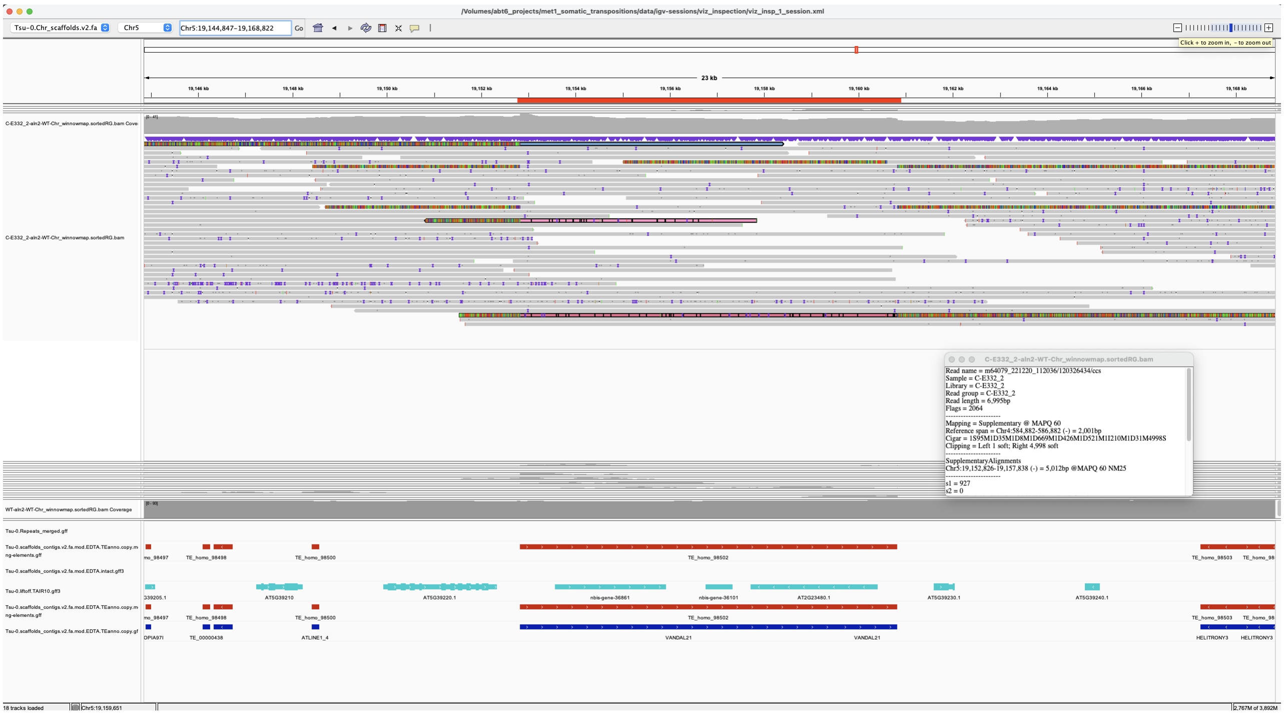

Partial

Confirmed

Chr4 19820190 19820190 + 1 Chr3;16344522;16352497;VANDAL6 m64079_240212_113350/23396363/ccs met1_02

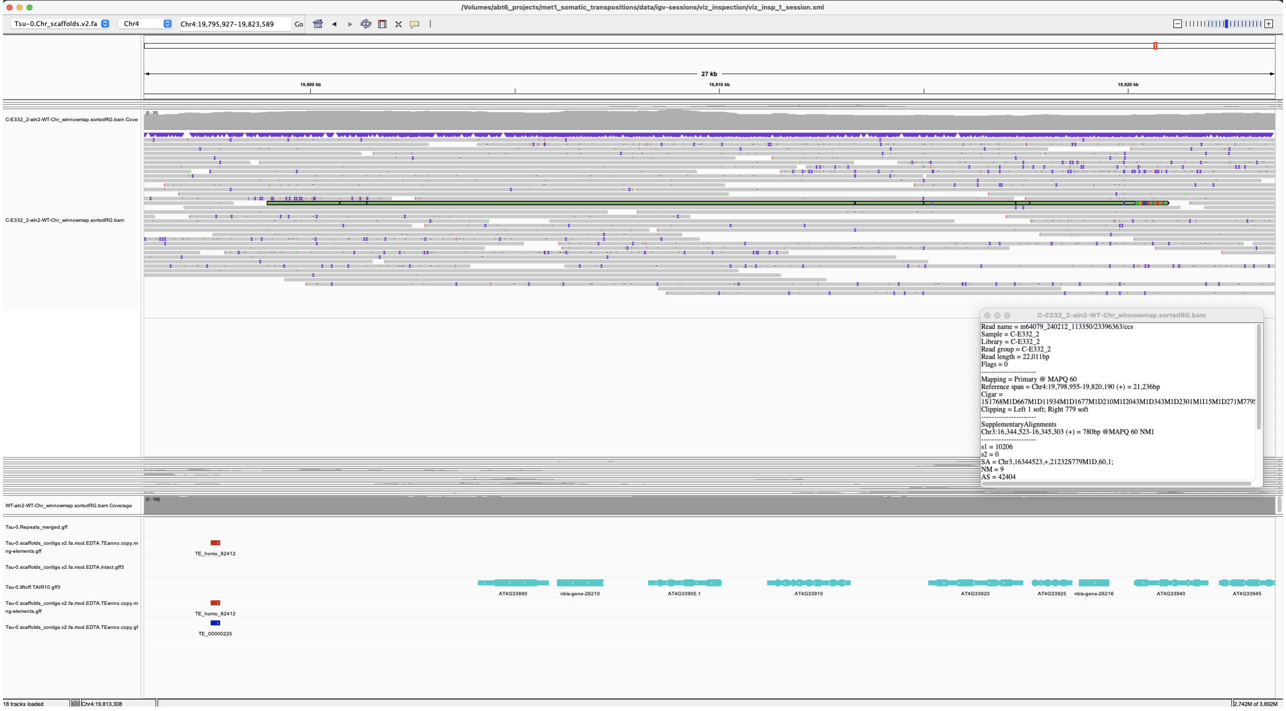

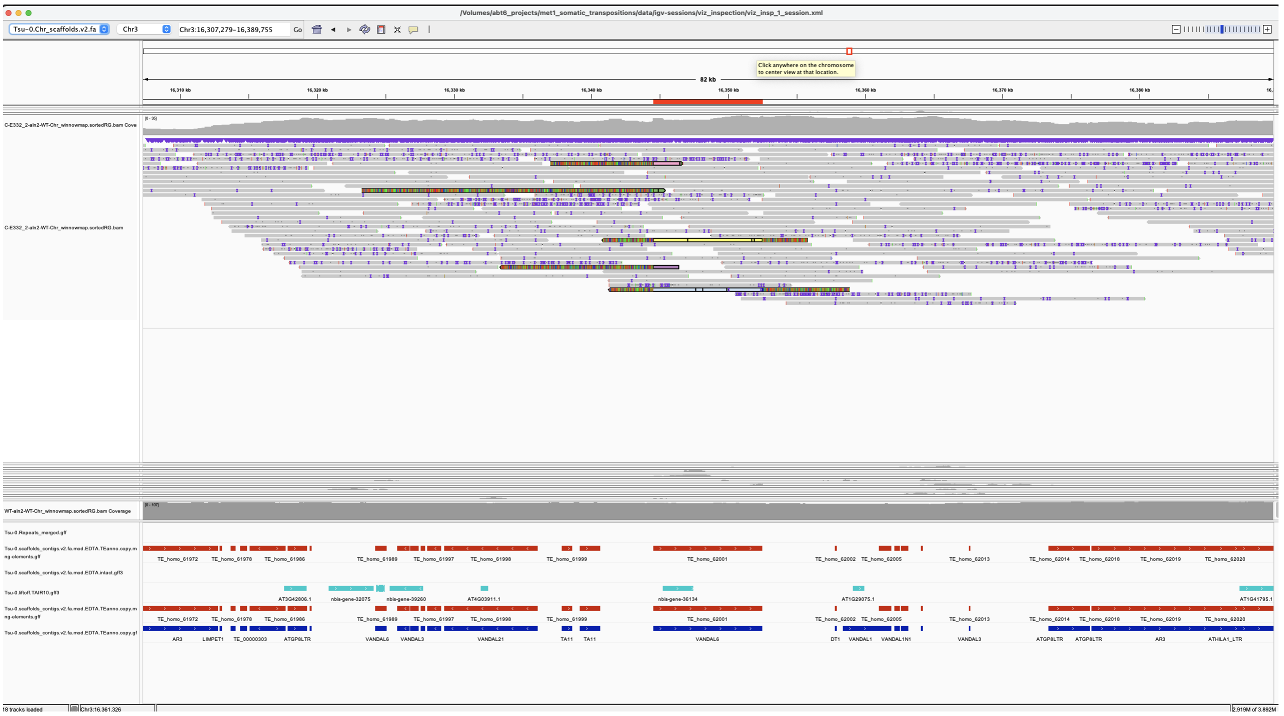

Confirmed

Chr5 15578614 15578614 + 1 Chr5;19152829;19160826;VANDAL21 m64079_221220_112036/49152575/ccs met1_02

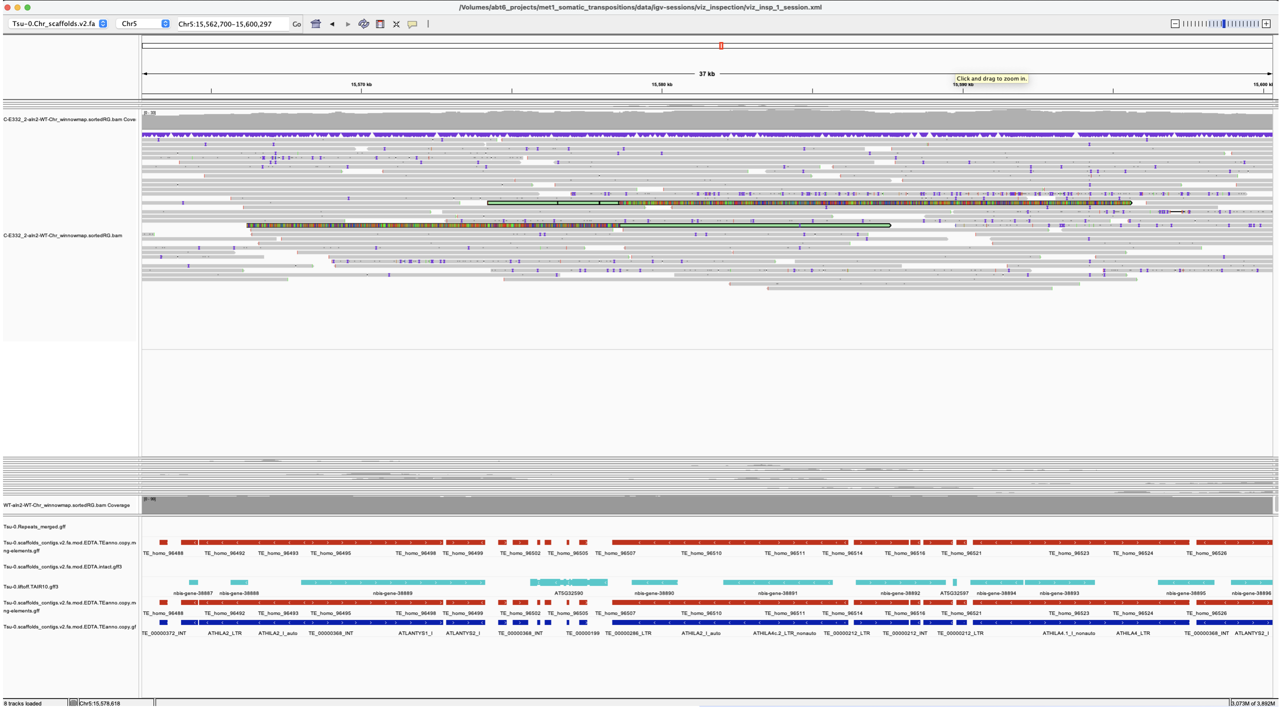

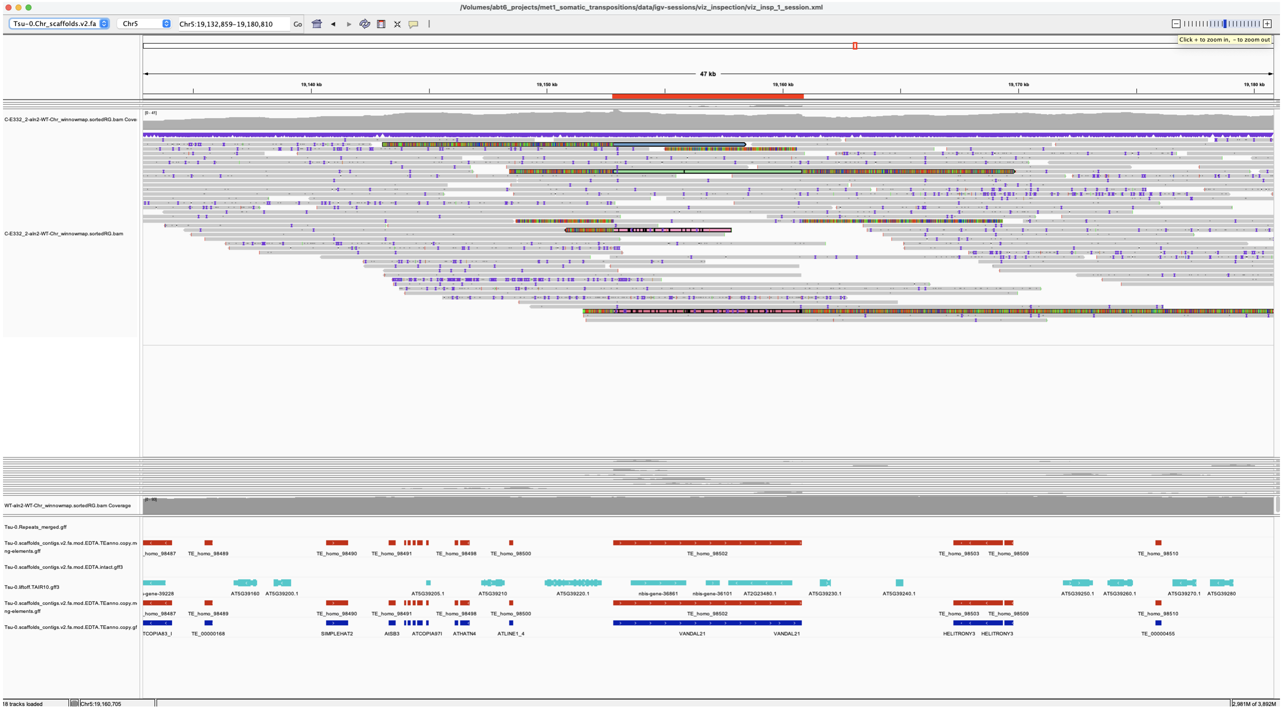

Central
TSD

Confirmed

Chr5 16487843 16487843 + 1 Chr5;19152829;19160826;VANDAL21 m64079_221220_112036/61344441/ccs met1_02

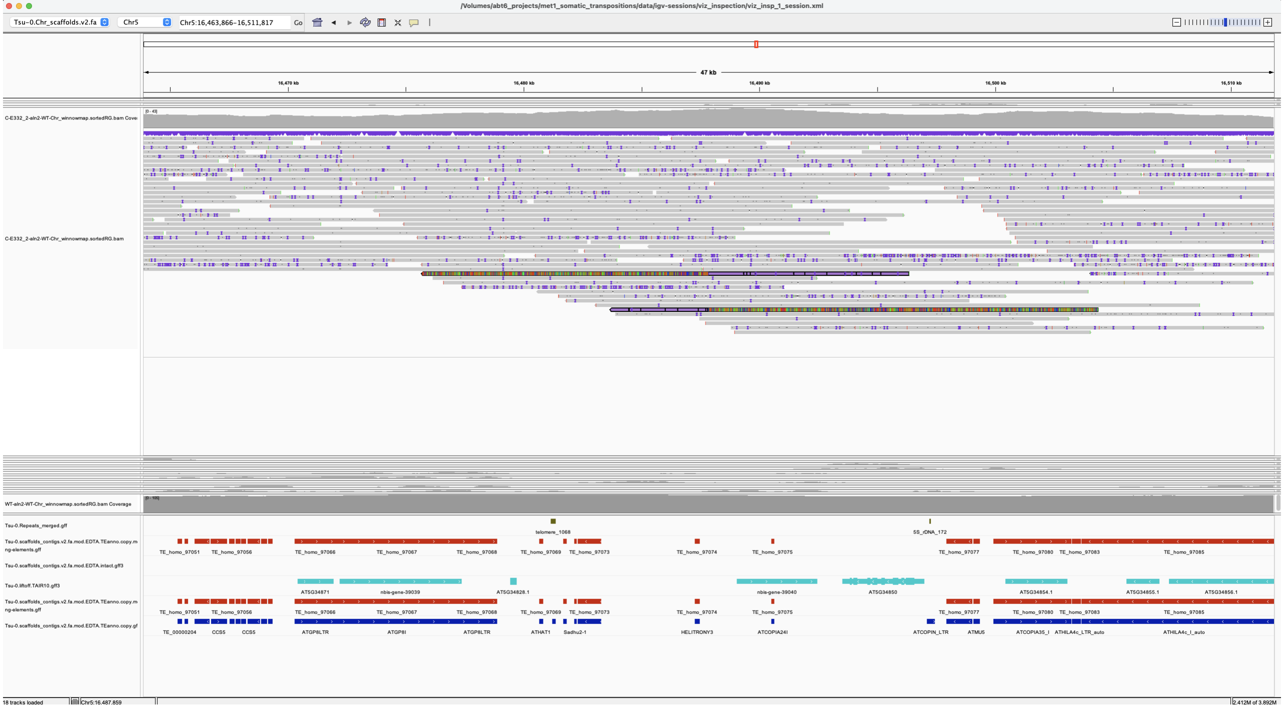

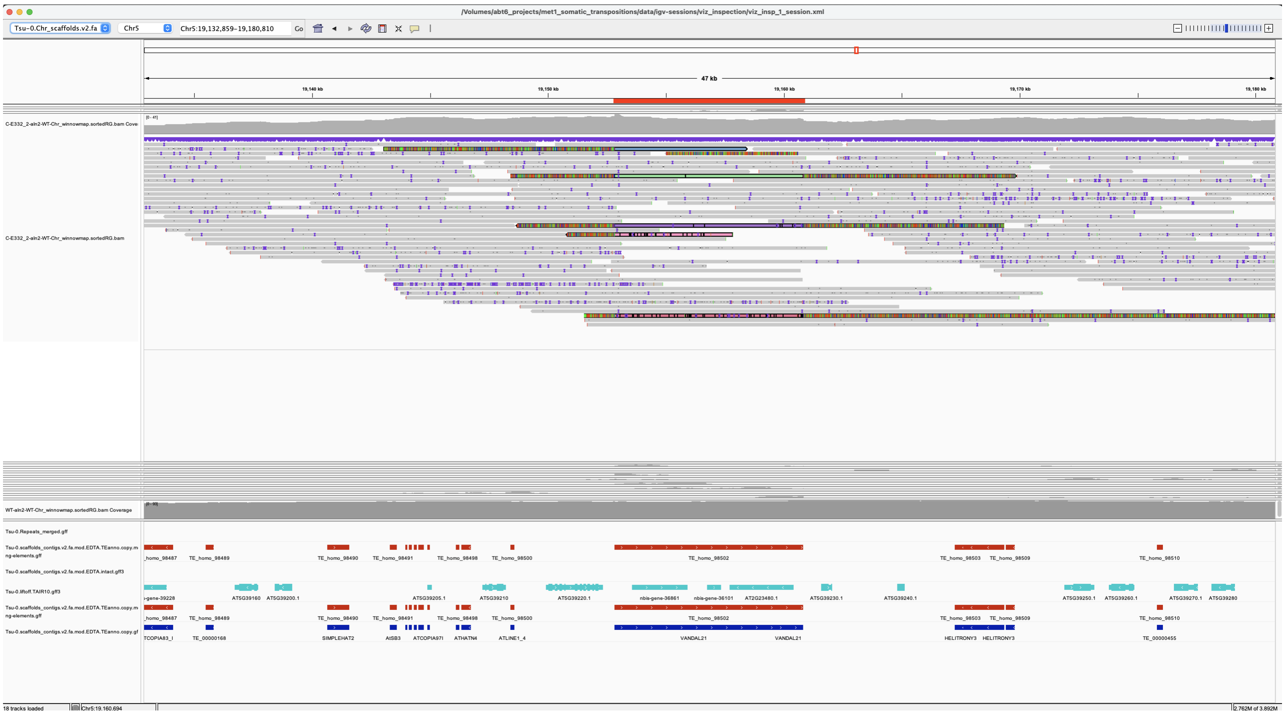

Central
TSD

Confirmed

Chr5 19915977 19915977 + 1 Chr5;19872565;19877095;VANDAL21 m64079_221220_112036/146147378/ccs met1_02

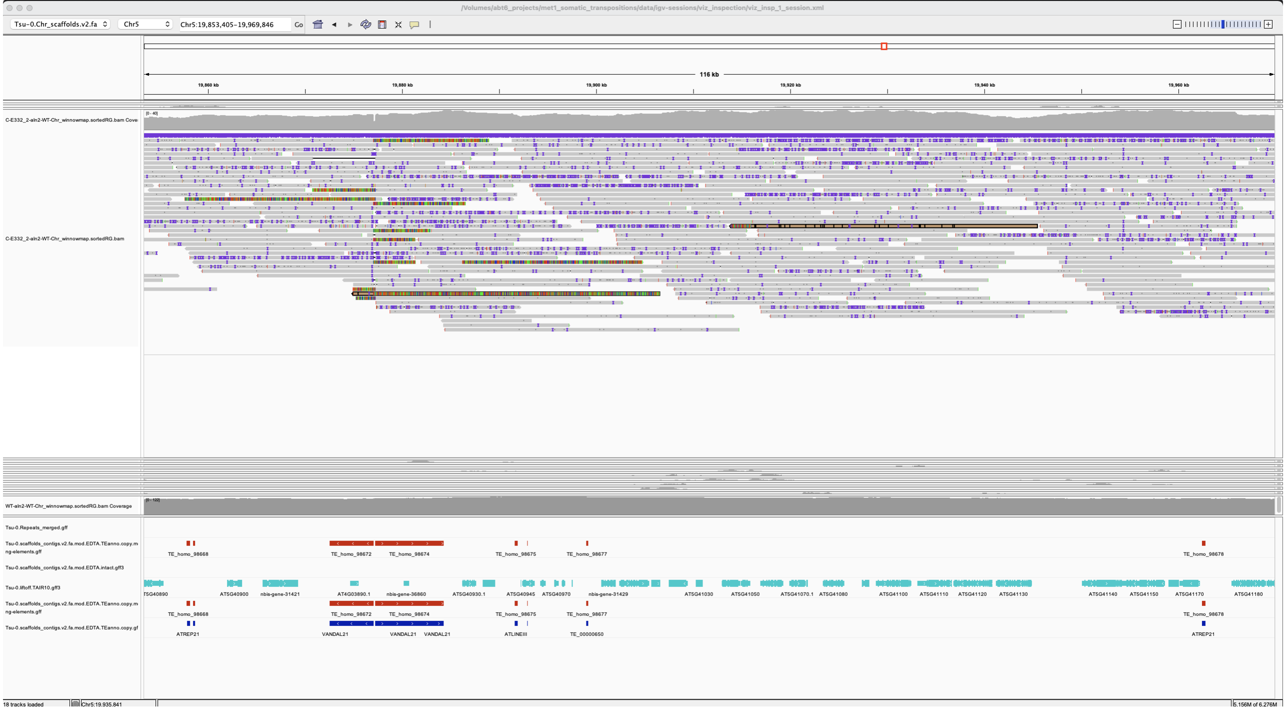

RE-arrangement
probably it is a big deletion

Hypermutable region

unsupported

Chr5 22640823 22640823 + 1 Chr5;19877283;19884298;VANDAL21 m64079_240212_113350/13566575/ccs met1_02

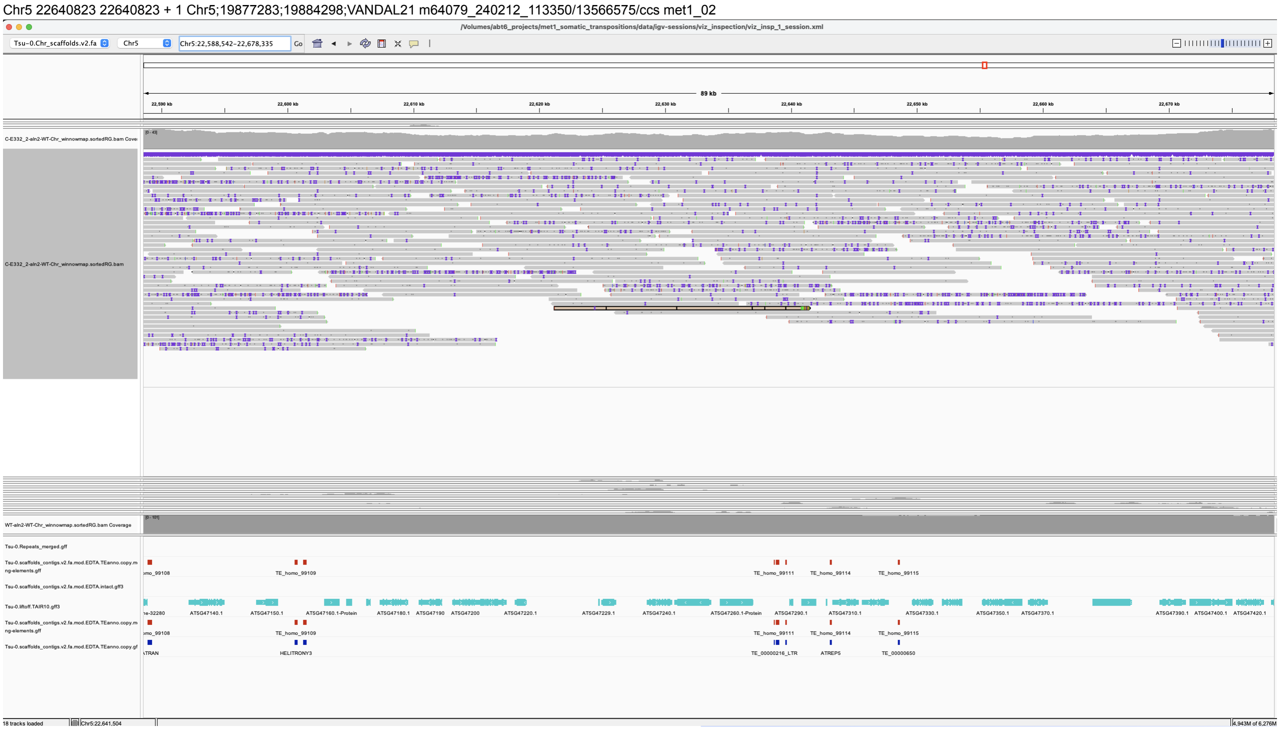

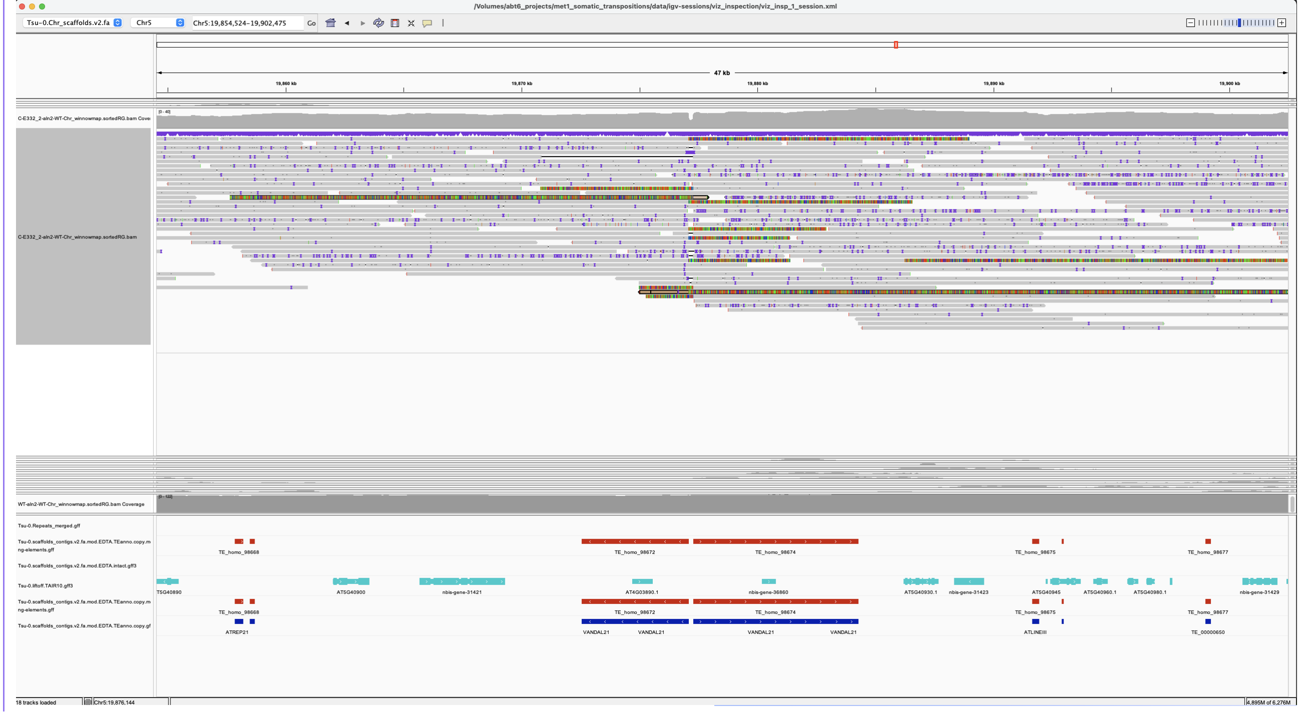

Deemed rearrangement

Hypermutable region

unsupported

---

met1_03

> **DIFFICULT CASE TO AUTOMATE: m64079_221220_112036/87294497/ccs 1 1557 Chr3 16018411 16019970 20934 - ATHILA7_I m64079_221220_112036/87294497/ccs 1557 6872 Chr1 11941106 11946436 20934 + ATCOPIA93.2_Evade m64079_221220_112036/87294497/ccs 6872 20933 Chr3 16004303 16018416 20934 - .**

EVD insertion

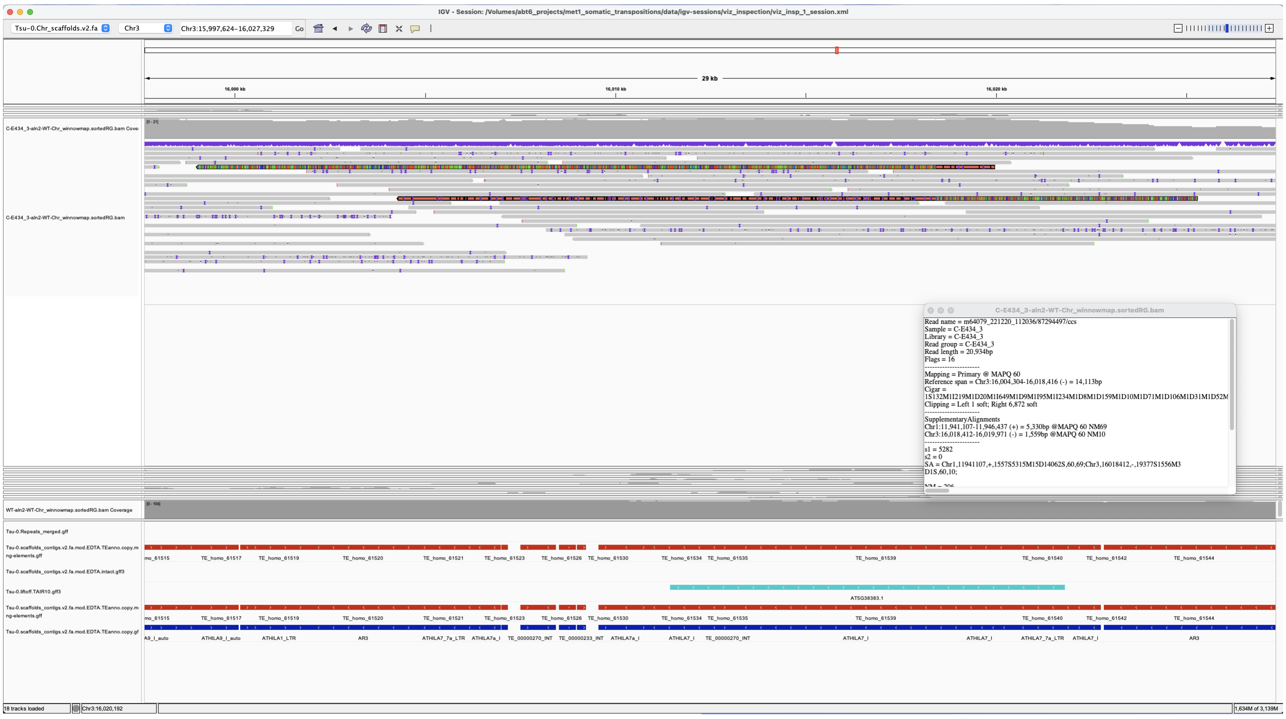

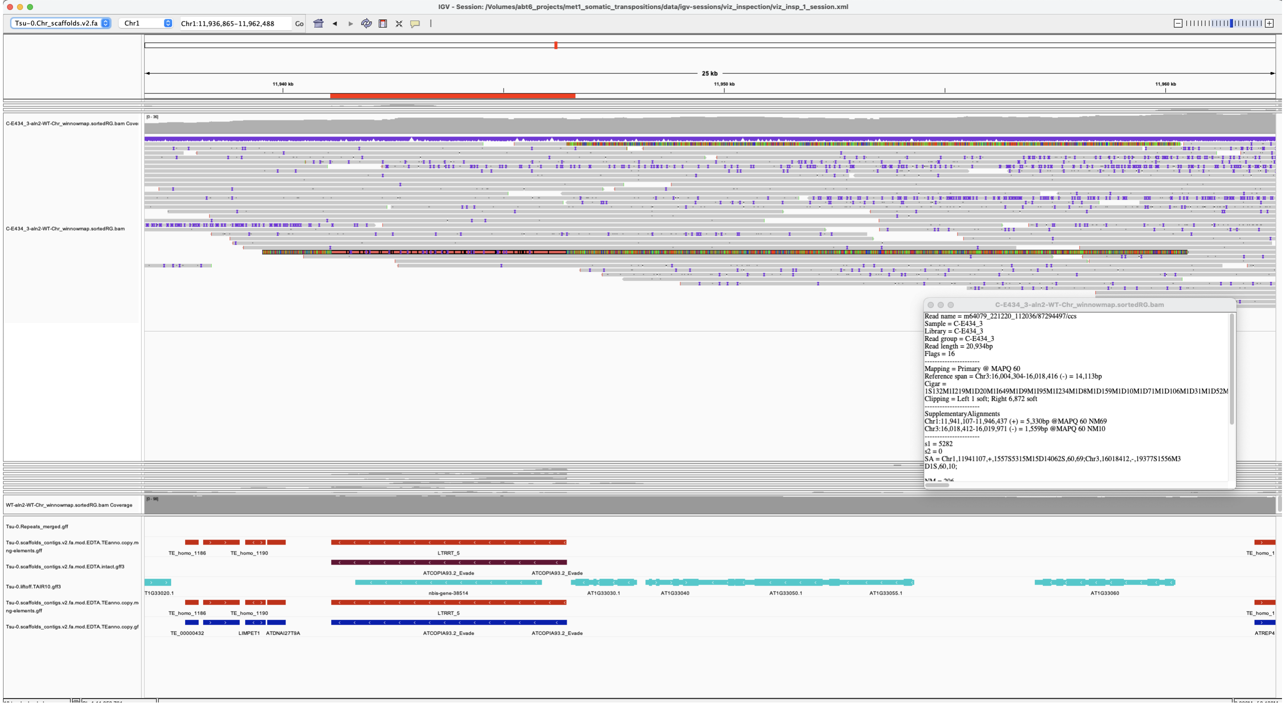

Central
TSD

Athila Centromeric

Confirmed

Chr2 9066366 9066366 + 1 Chr5;19152829;19160826;VANDAL21 m64079_240212_113350/7145316/ccs met1_03

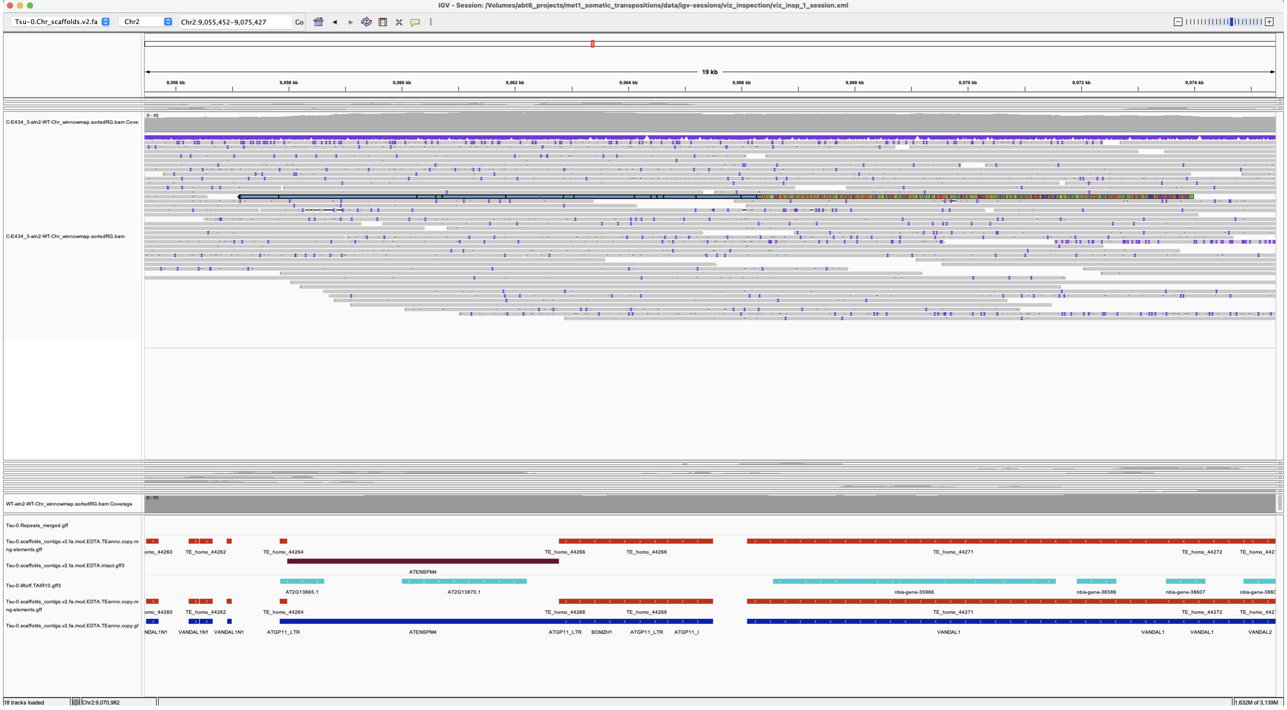

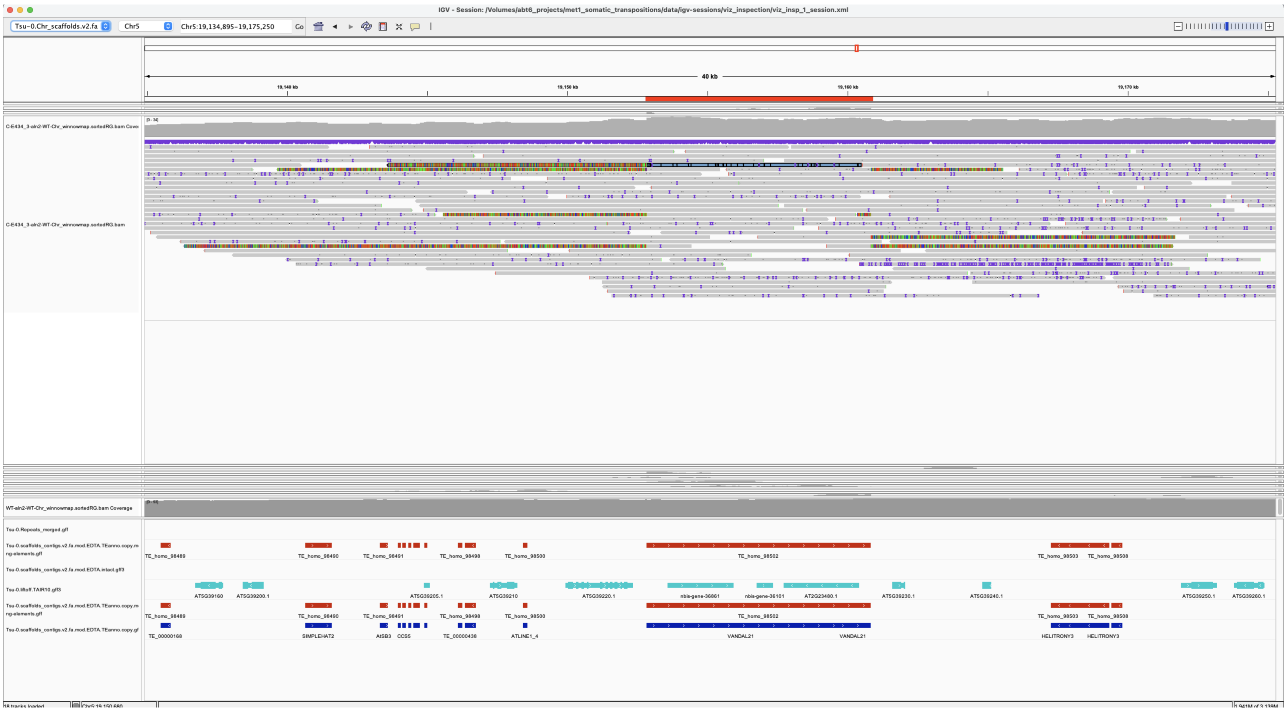

Partial

Confirmed

Chr2 22074634 22074634 - 1 Chr1;11941106;11946436;ATCOPIA93_Evade m64079_221220_112036/63113278/ccs met1_03

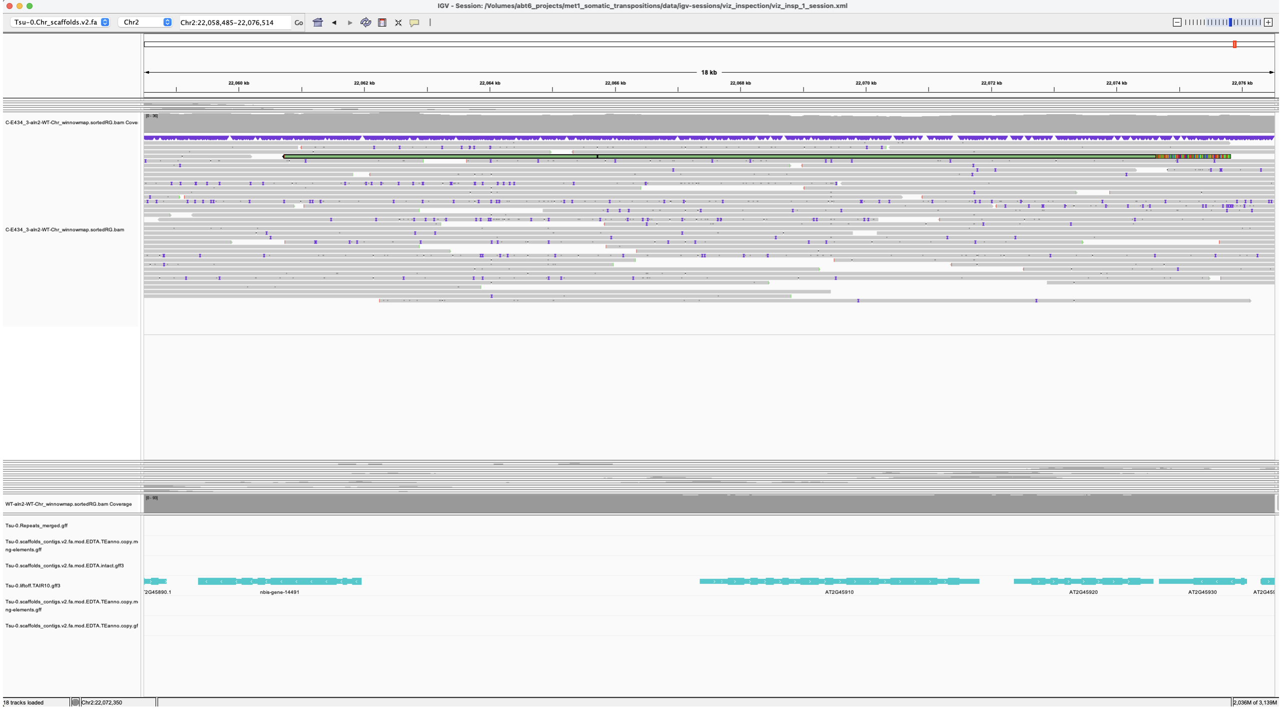

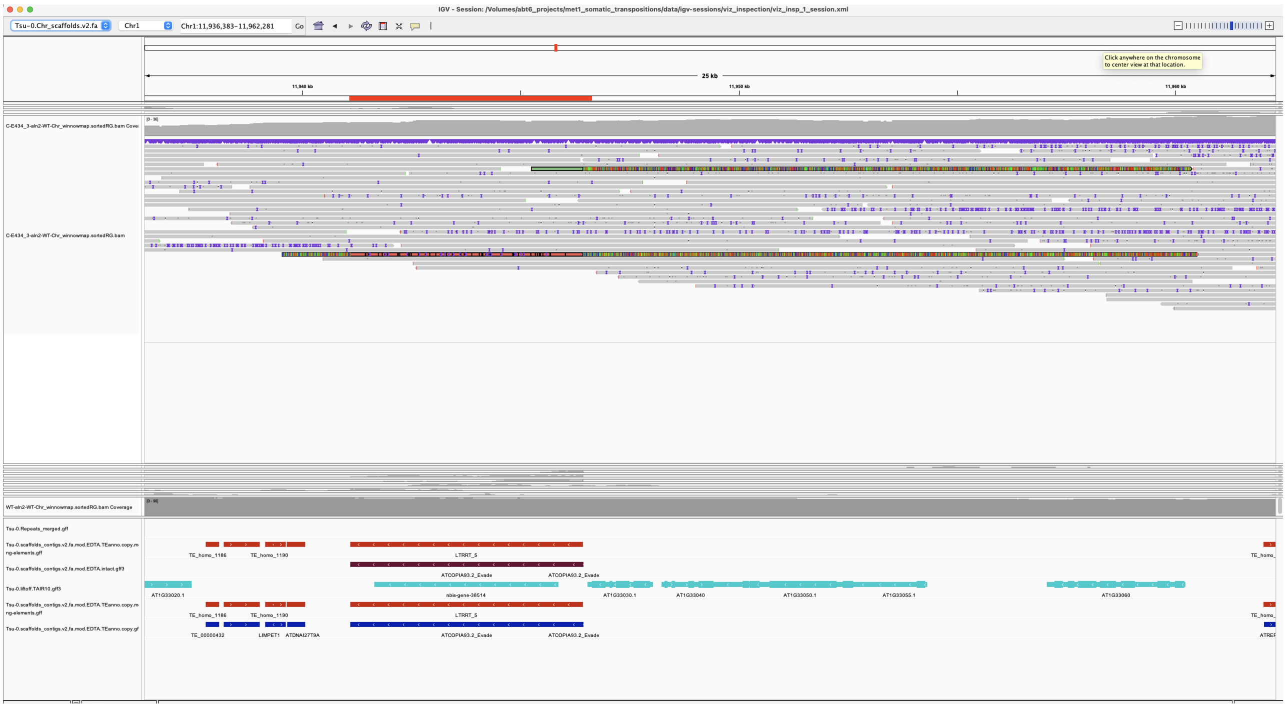

Partial

Confirmed

Chr4 4801792 4801792 - 1 Chr5;19152829;19160826;VANDAL21 m64079_240212_113350/153487970/ccs met1_03

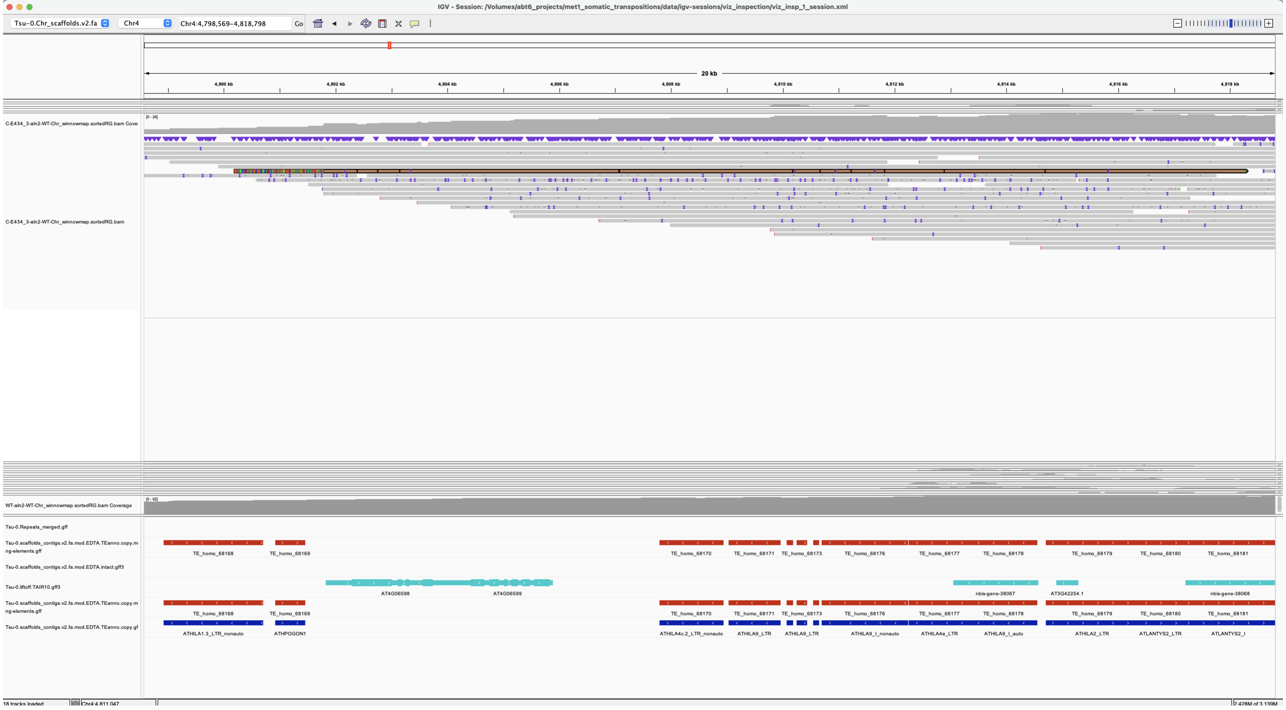

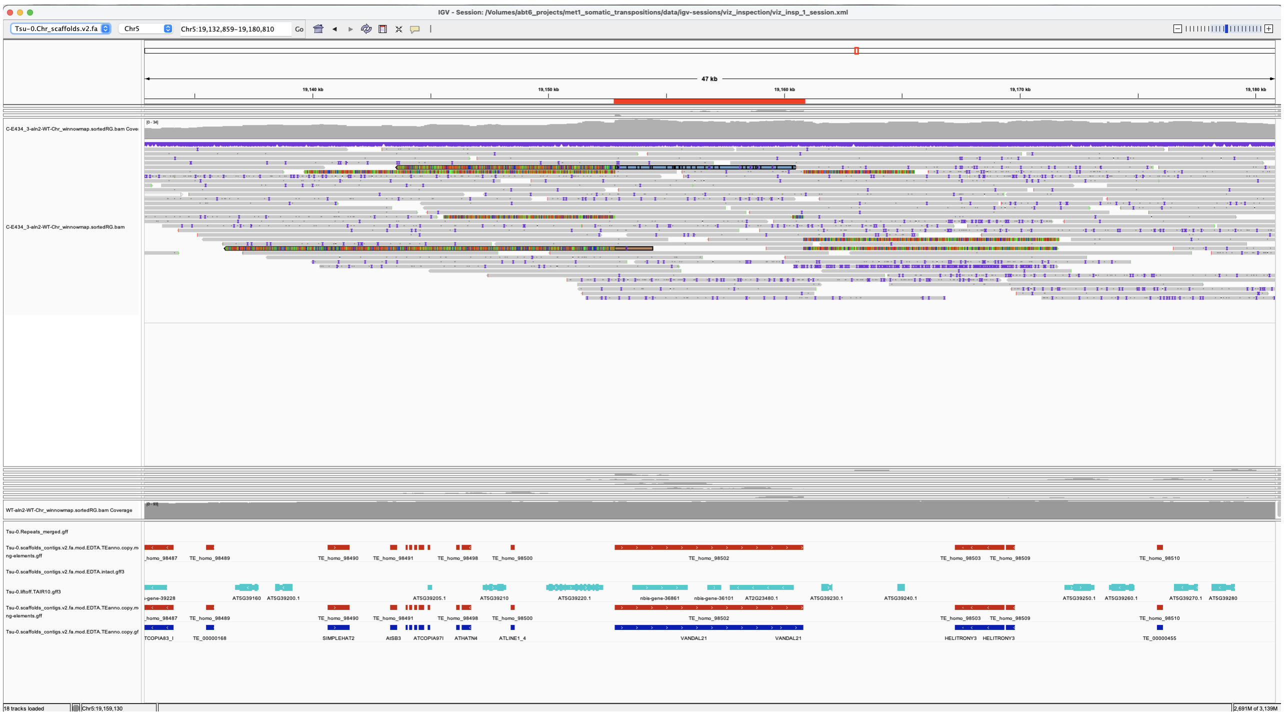

Partial

Confirmed

Chr4 10015343 10015343 + 1 Chr3;16344522;16352497;VANDAL6 m64079_221220_112036/81200381/ccs met1_03

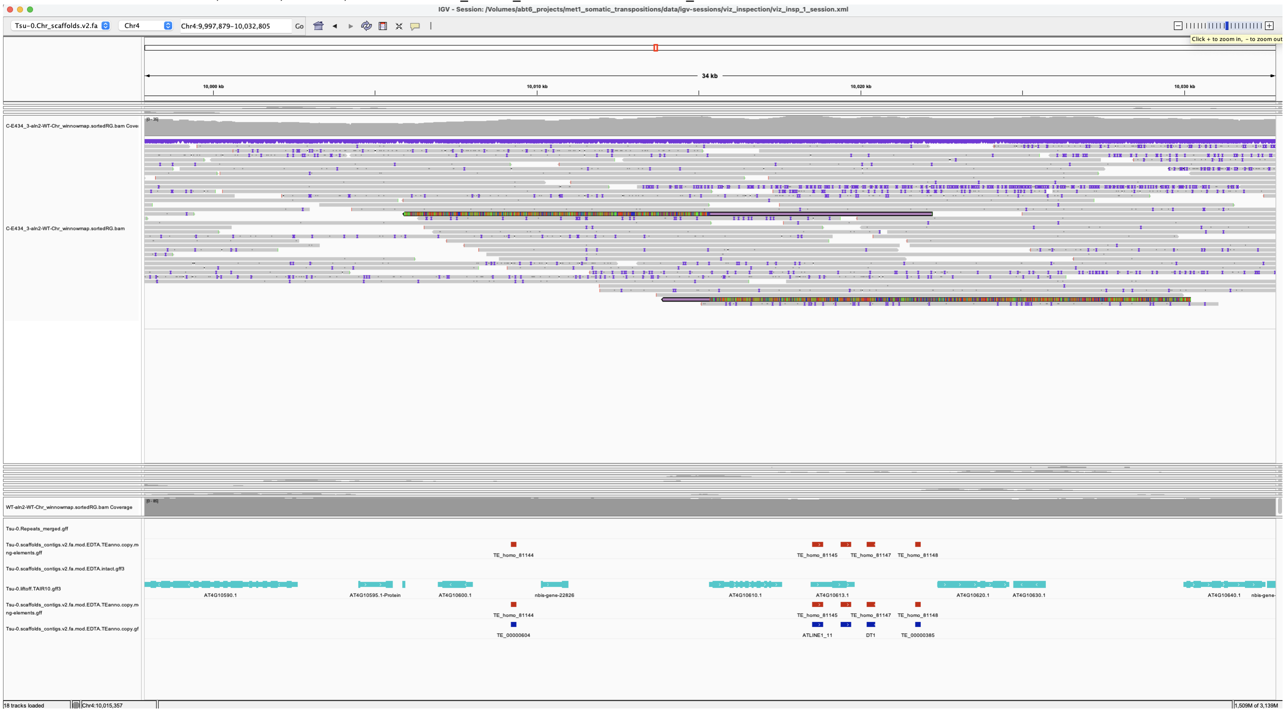

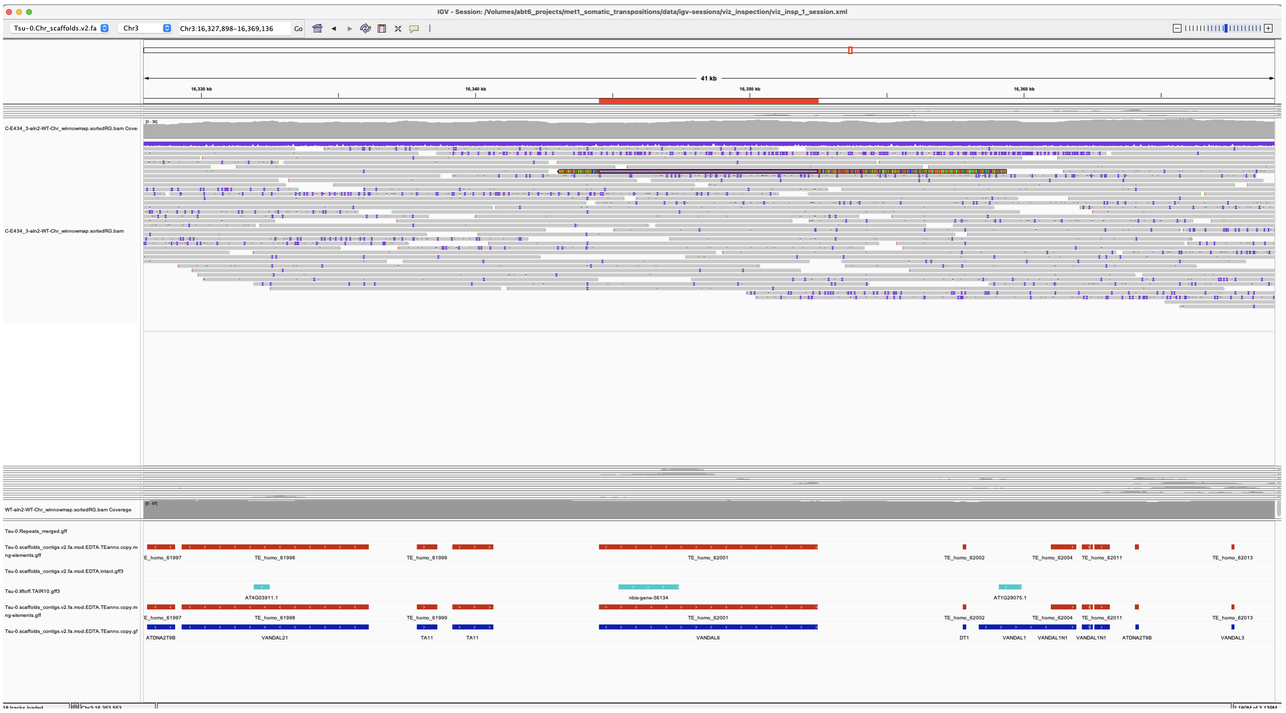

Central
TSD

Confirmed

Chr4 19683260 19683260 + 1 Chr3;20158137;20166150;VANDAL6 m64079_221220_112036/120521150/ccs met1_03

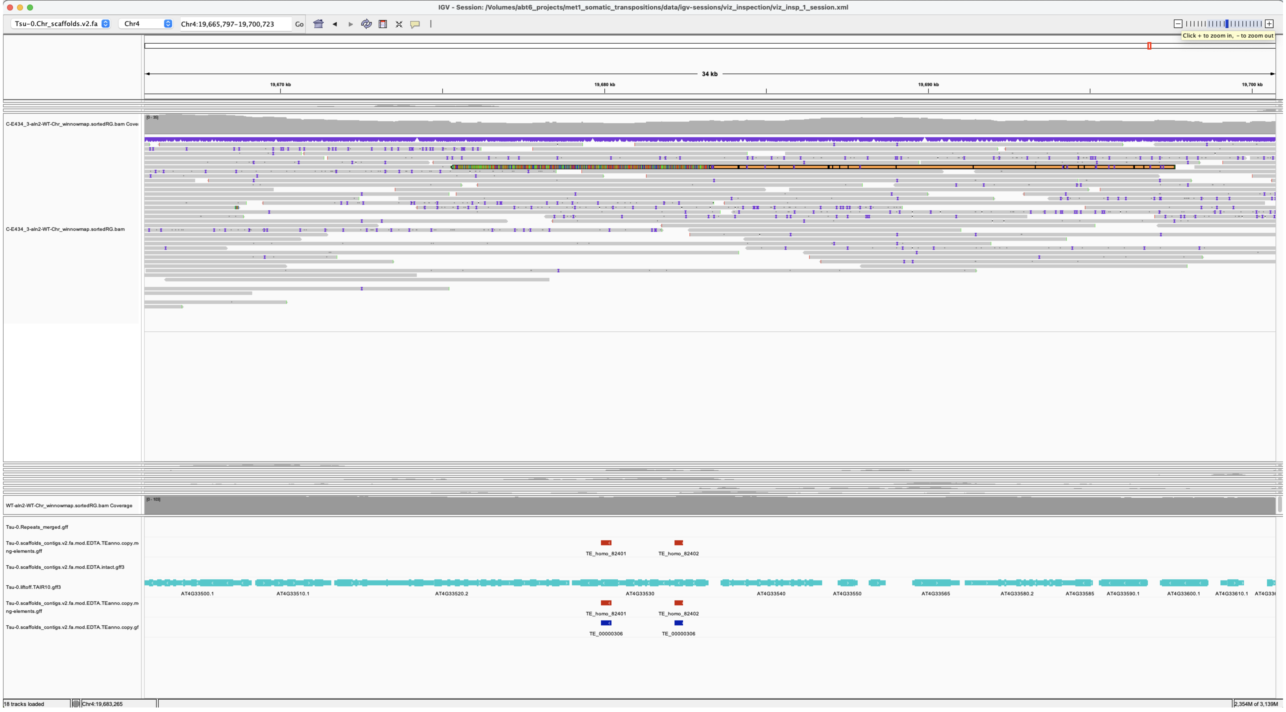

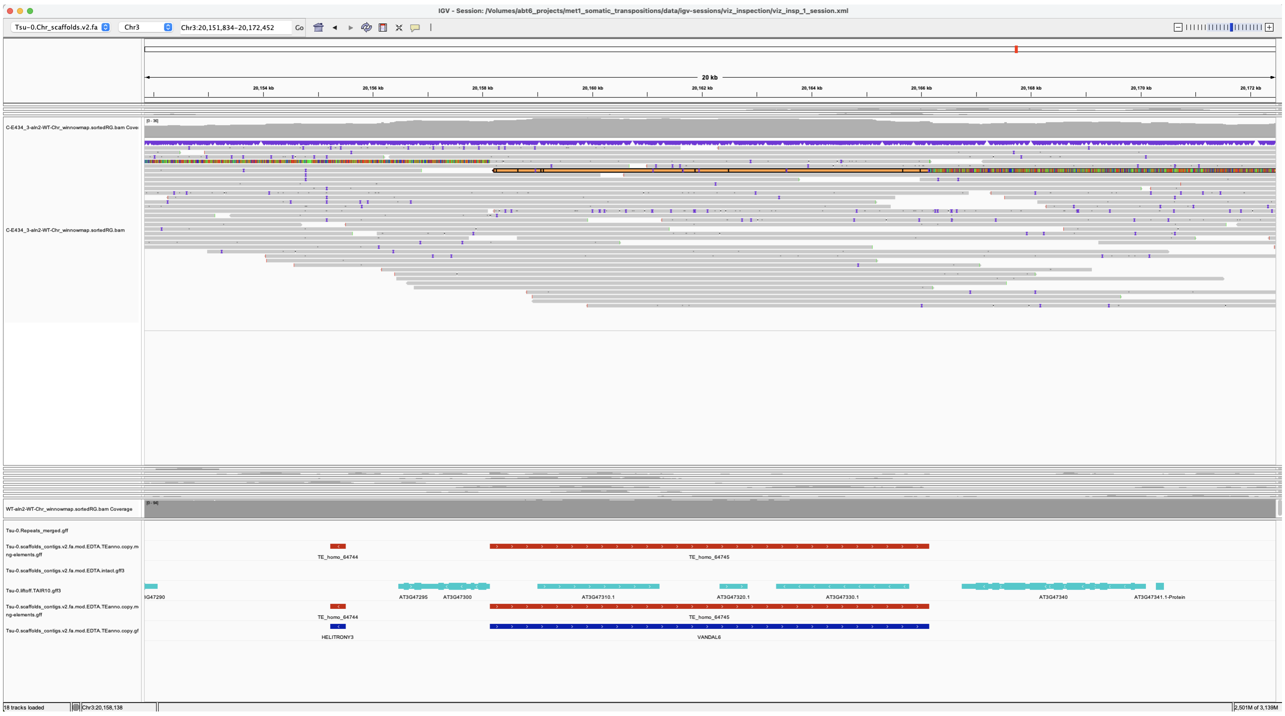

Partial

Confirmed

Chr5 4354556 4354556 - 1 Chr3;20158137;20166150;VANDAL6 m64079_240212_113350/32049639/ccs met1_03

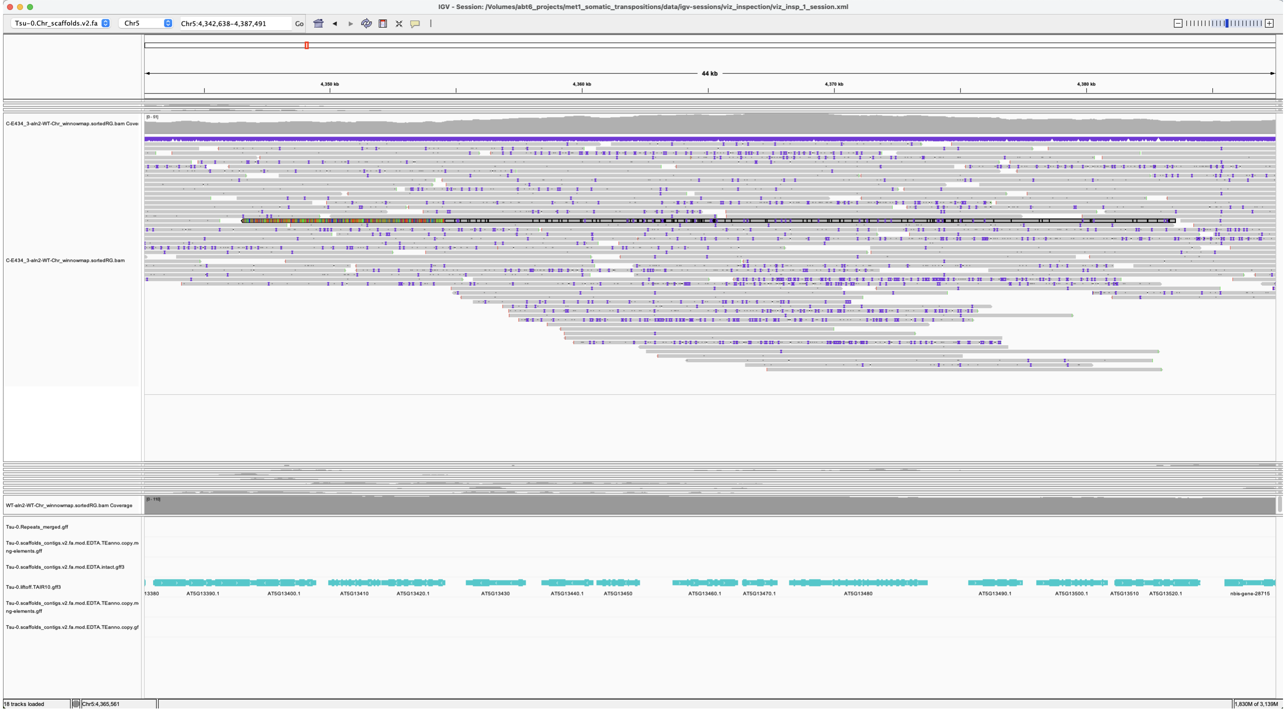

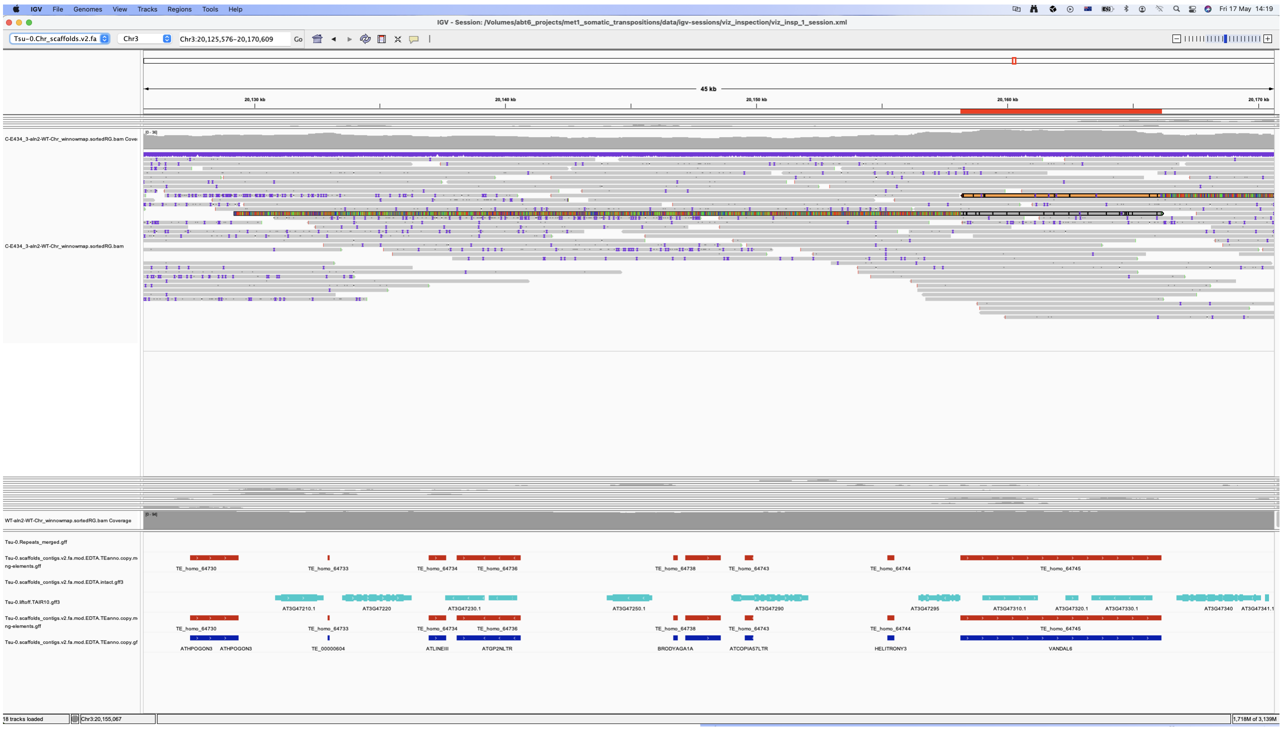

Partial

Confirmed

> Chr5 6593784 6593784 + 1 Chr5;19152829;19160826;VANDAL21 m64079_221220_112036/99747143/ccs met1_03

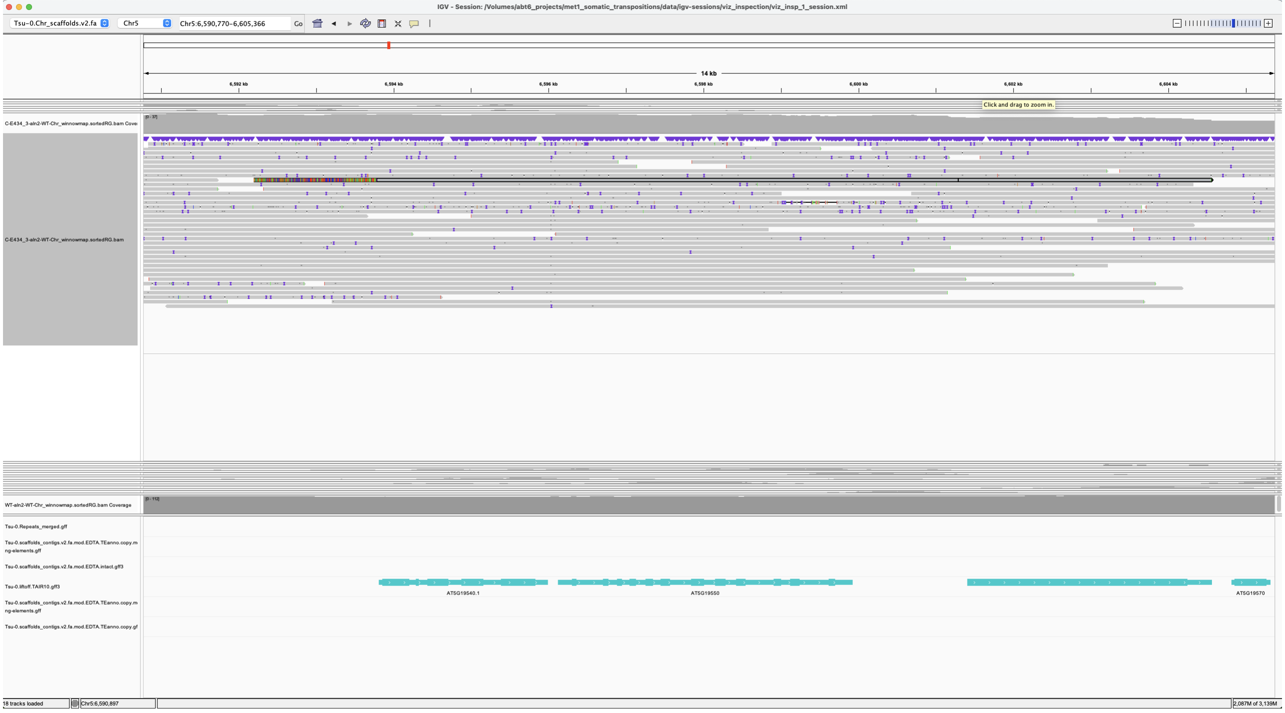

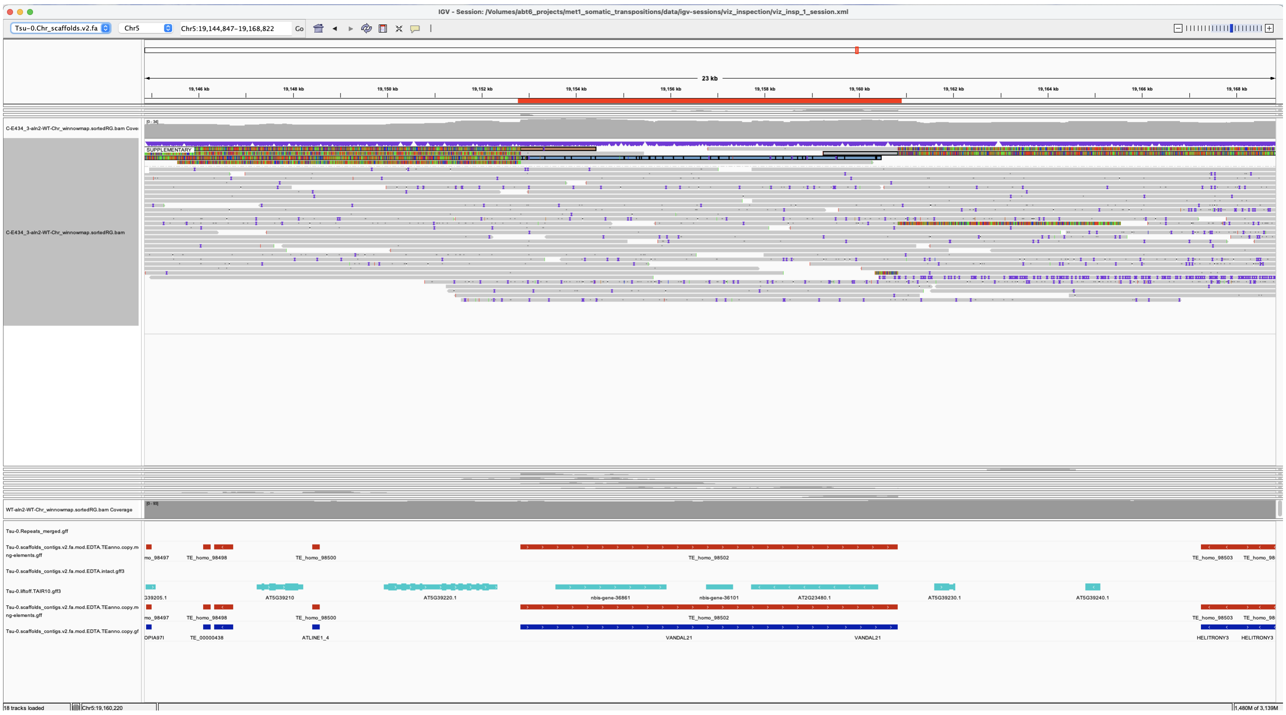

Partial

Confirmed

Chr5 9248081 9248081 - 1 Chr5;19877283;19884298;VANDAL21 m64079_240212_113350/19269311/ccs met1_03

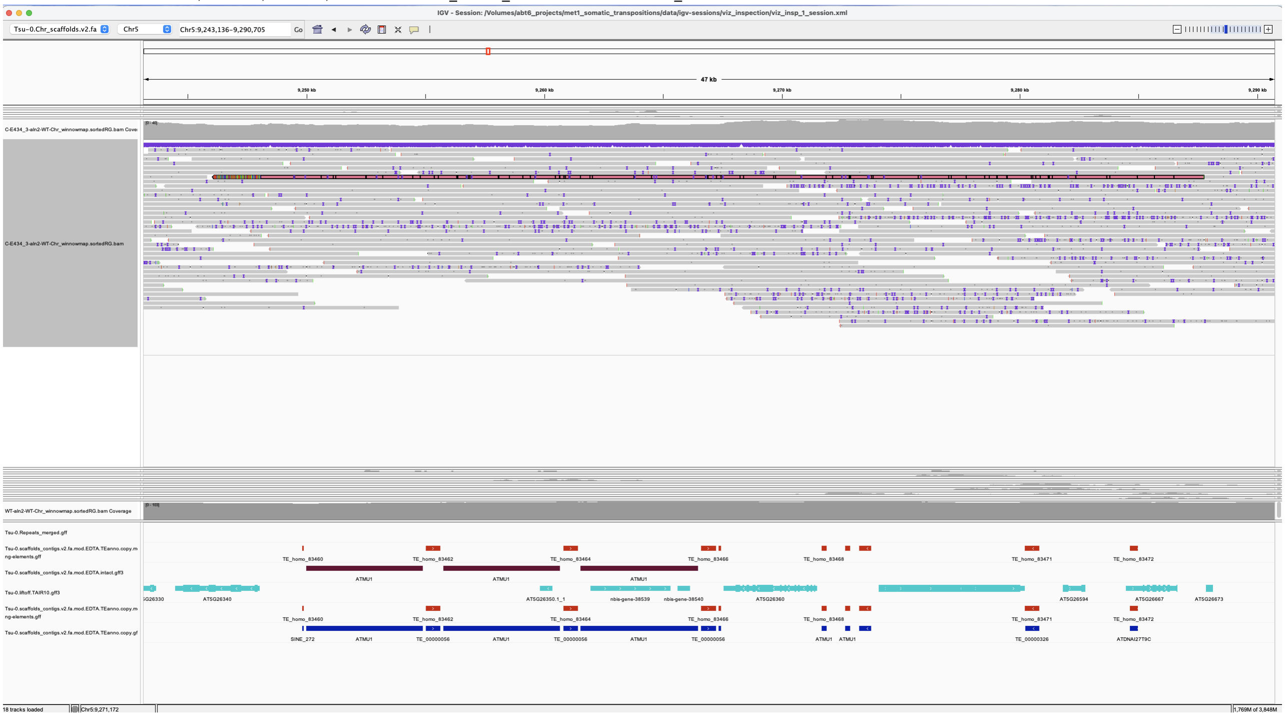

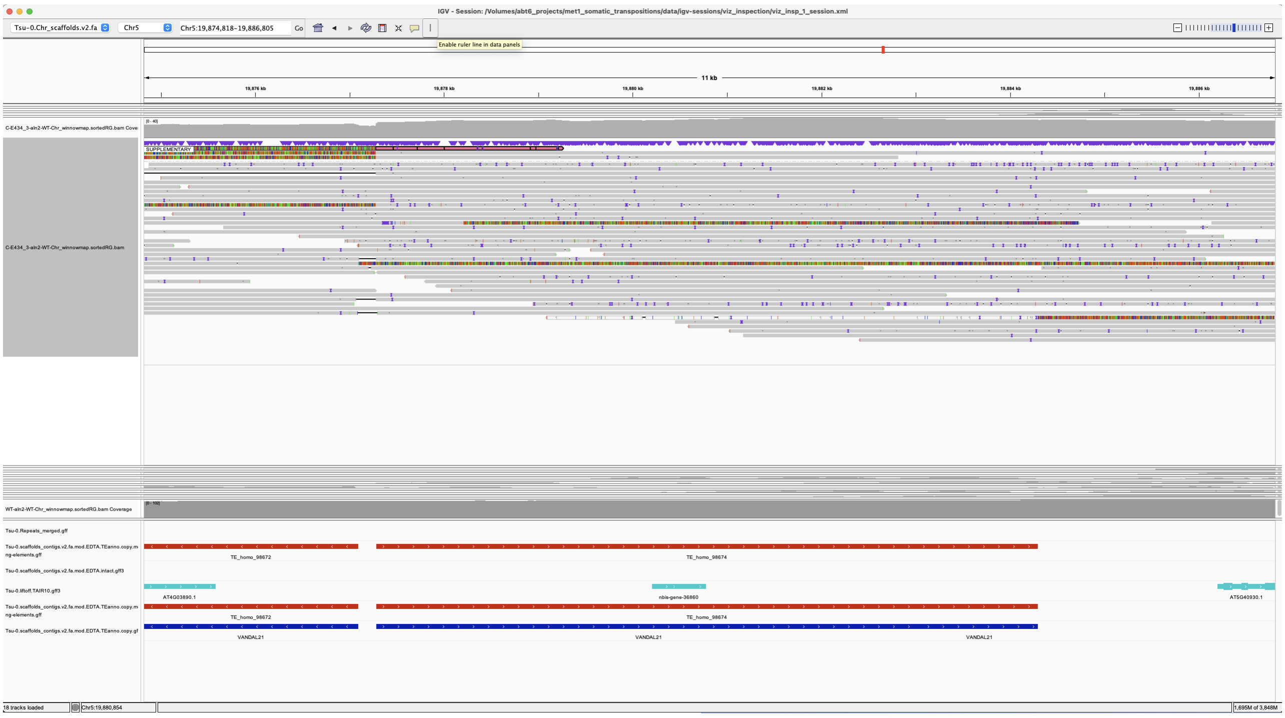

Probably rearrangement

Hypermutable Region
unsupported

Chr5 11090764 11090764 + 1 Chr5;19152829;19160826;VANDAL21 m64079_221220_112036/36374589/ccs met1_03

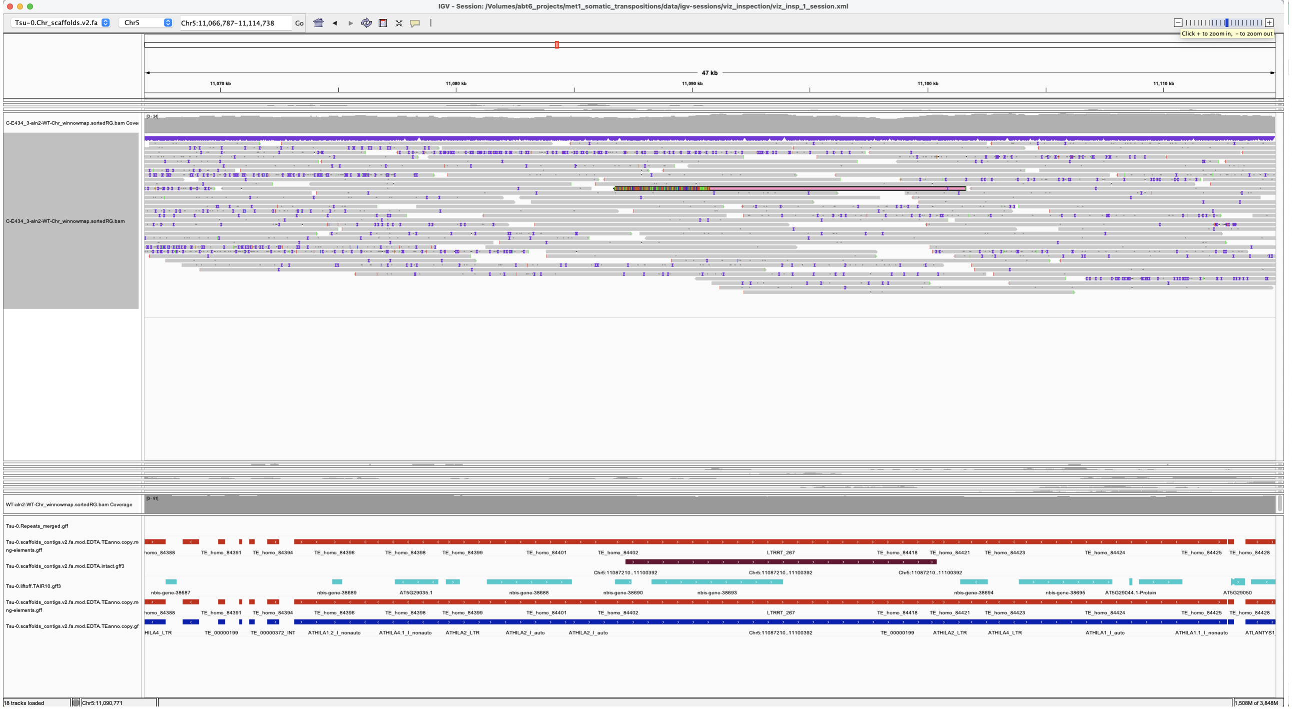

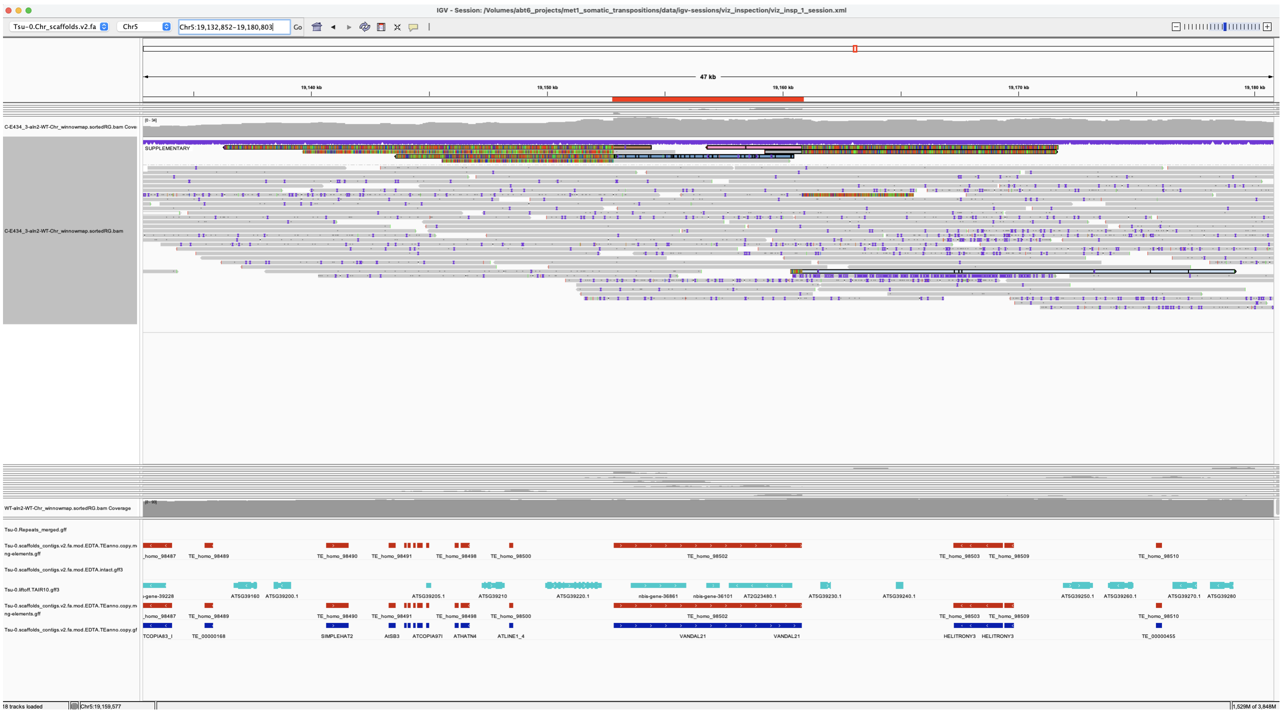

Partial

Confirmed

Chr5 15437876 15437876 - 1 Chr5;19152829;19160826;VANDAL21 m64079_240212_113350/9963280/ccs met1_03

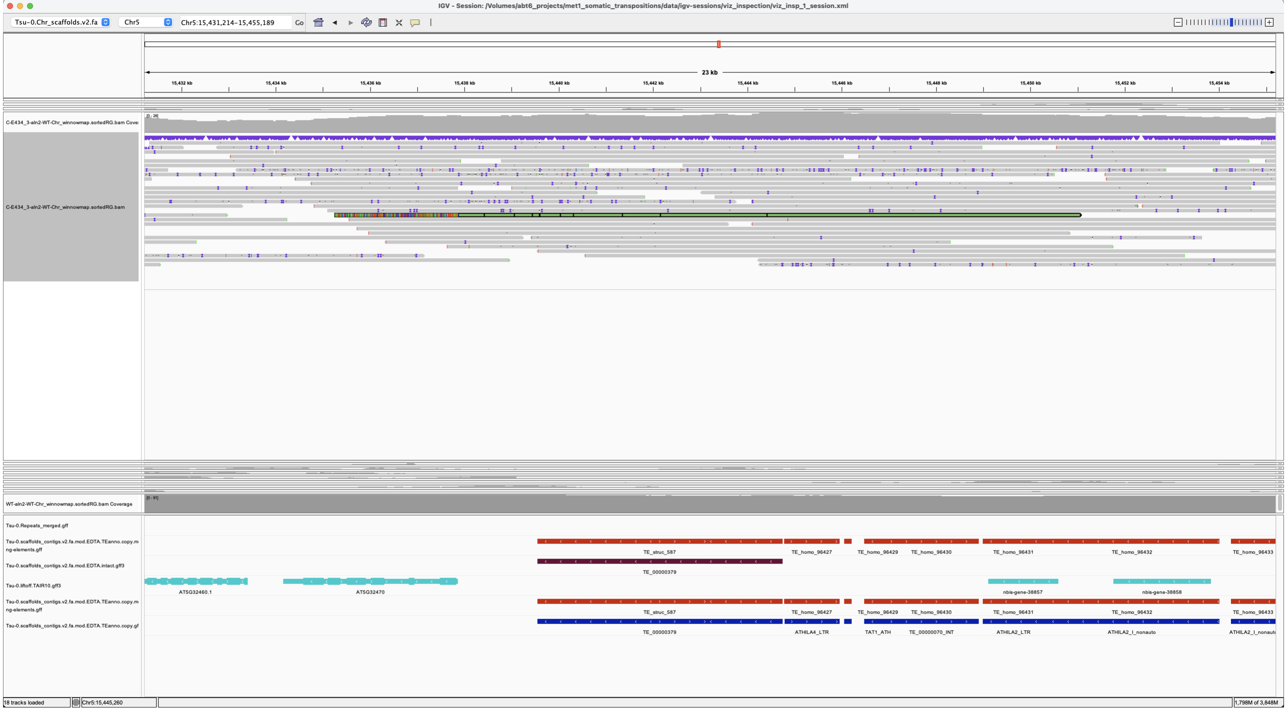

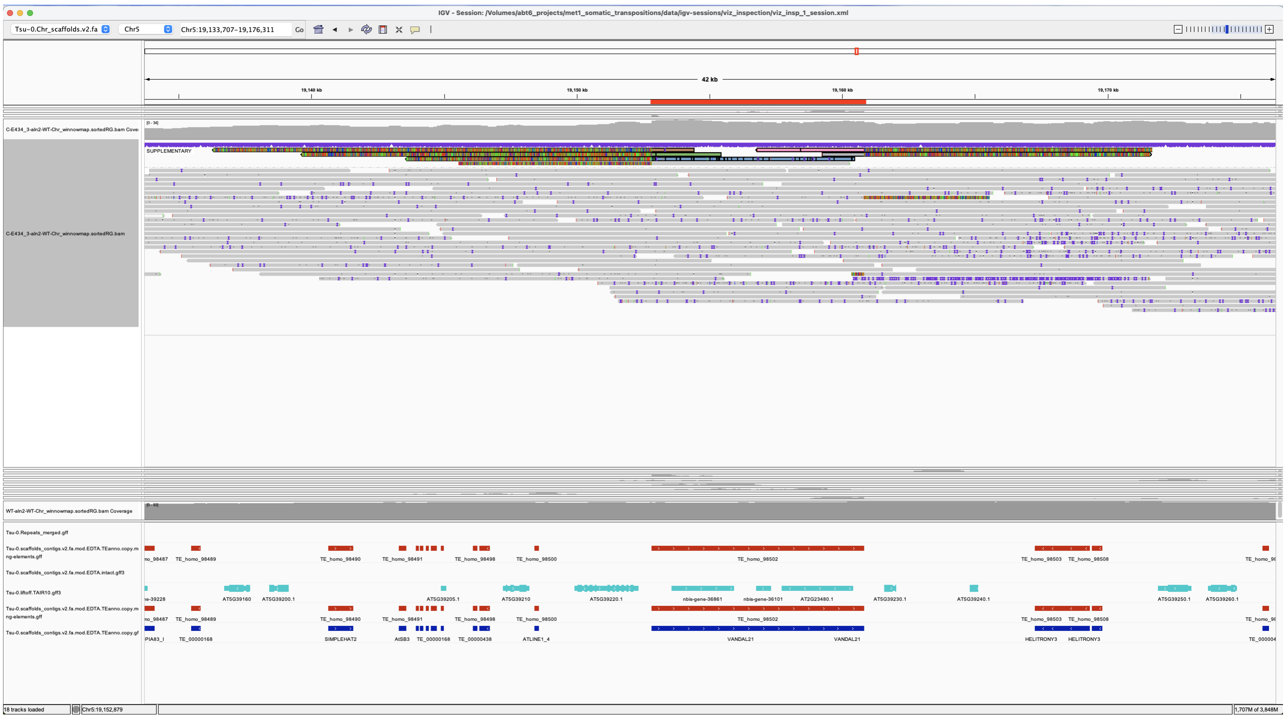

Partial

Confirmed

> Chr5 20436346 20436346 - 1 Chr5;19152829;19160826;VANDAL21 m64079_221220_112036/88738421/ccs met1_03

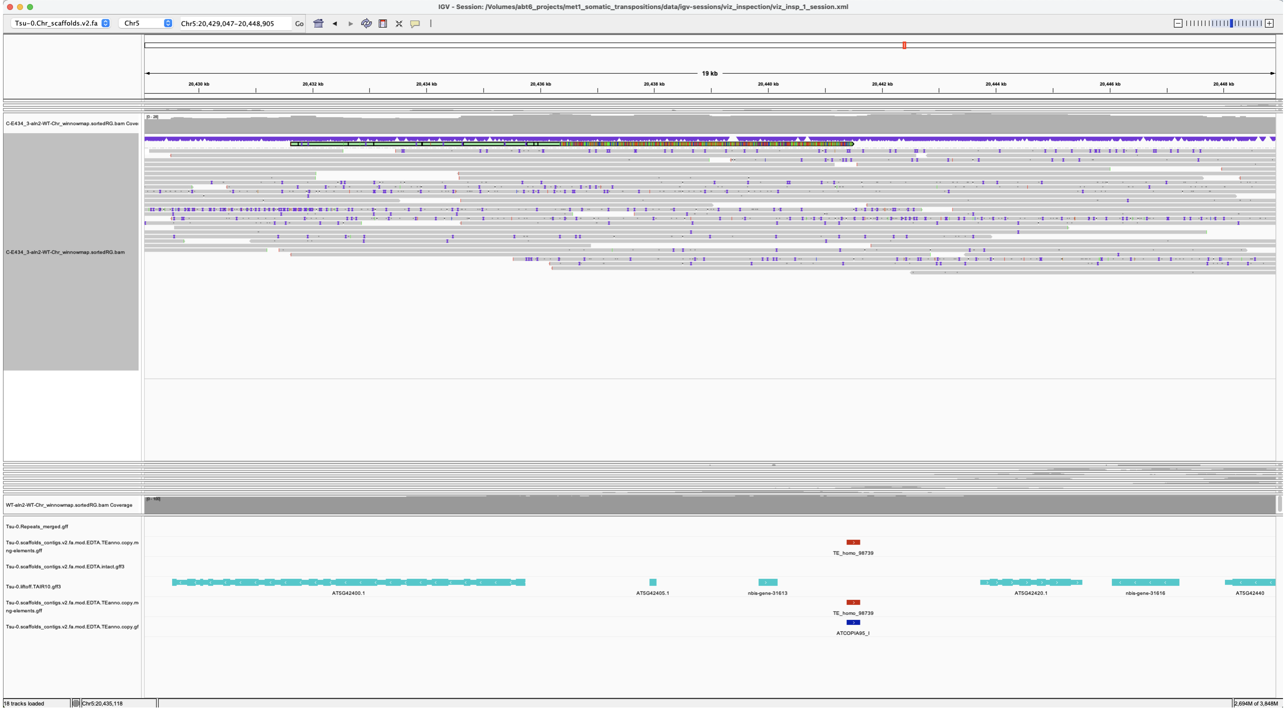

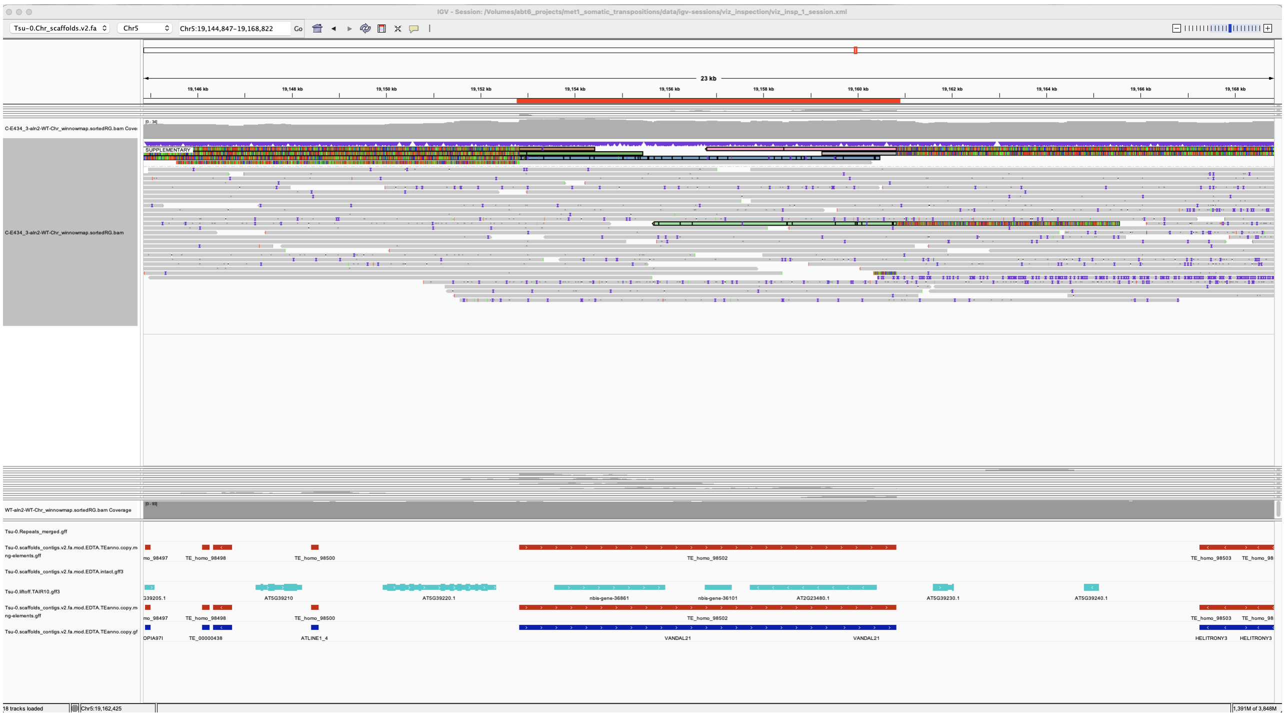

Partial

Confirmed

Chr5 20626476 20626476 + 1 Chr5;19877283;19884298;VANDAL21 m64079_240212_113350/60099338/ccs met1_03

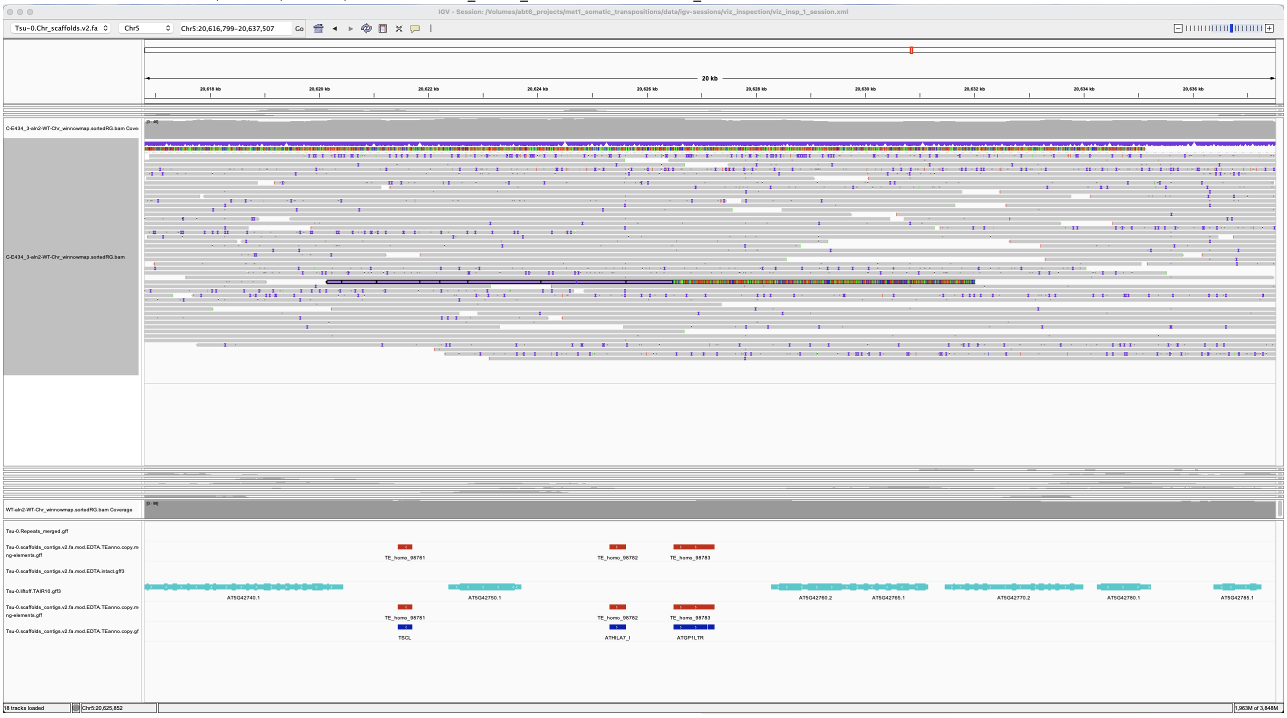

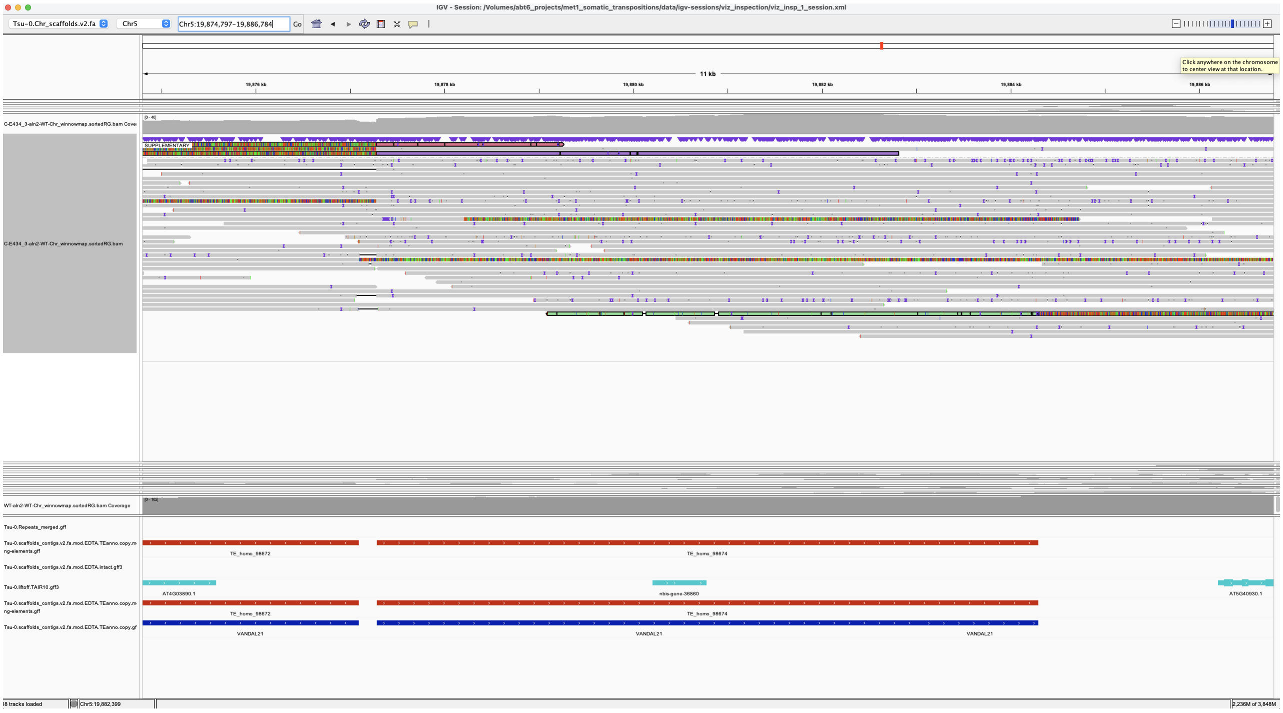

Rearrangement

Hypermutable region
unsupported

Chr5 28159472 28159472 + 1 Chr5;21419693;21425022;ATCOPIA93_Evade m64079_221220_112036/90638963/ccs met1_03

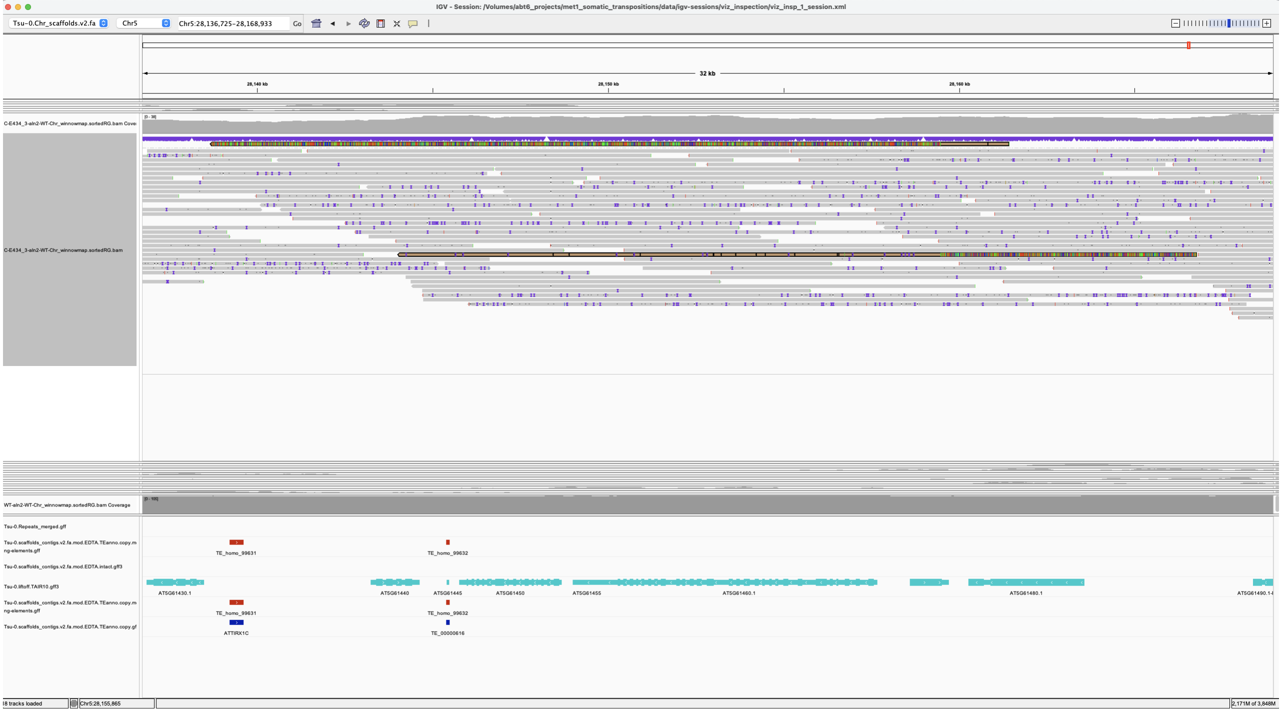

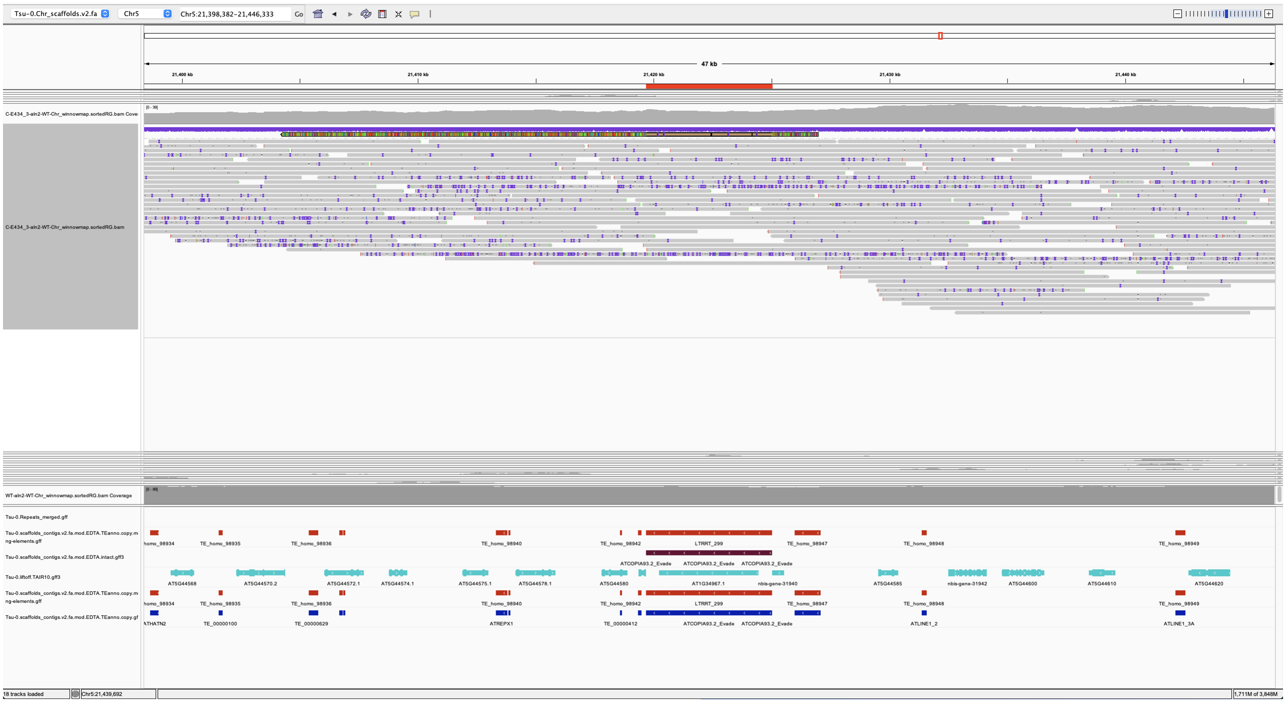

Central

TSD

Confirmed

---

met1_04

> **DIFFICULT CASE TO AUTOMATE: m64079_221220_112036/43976048/ccs 1 709 Chr5 19877278 19877986 9933 - VANDAL21 m64079_221220_112036/43976048/ccs 717 4877 Chr5 19872935 19877095 9933 + VANDAL21 m64079_221220_112036/43976048/ccs 4877 9933 Chr5 19867879 19872935 9933 - .**

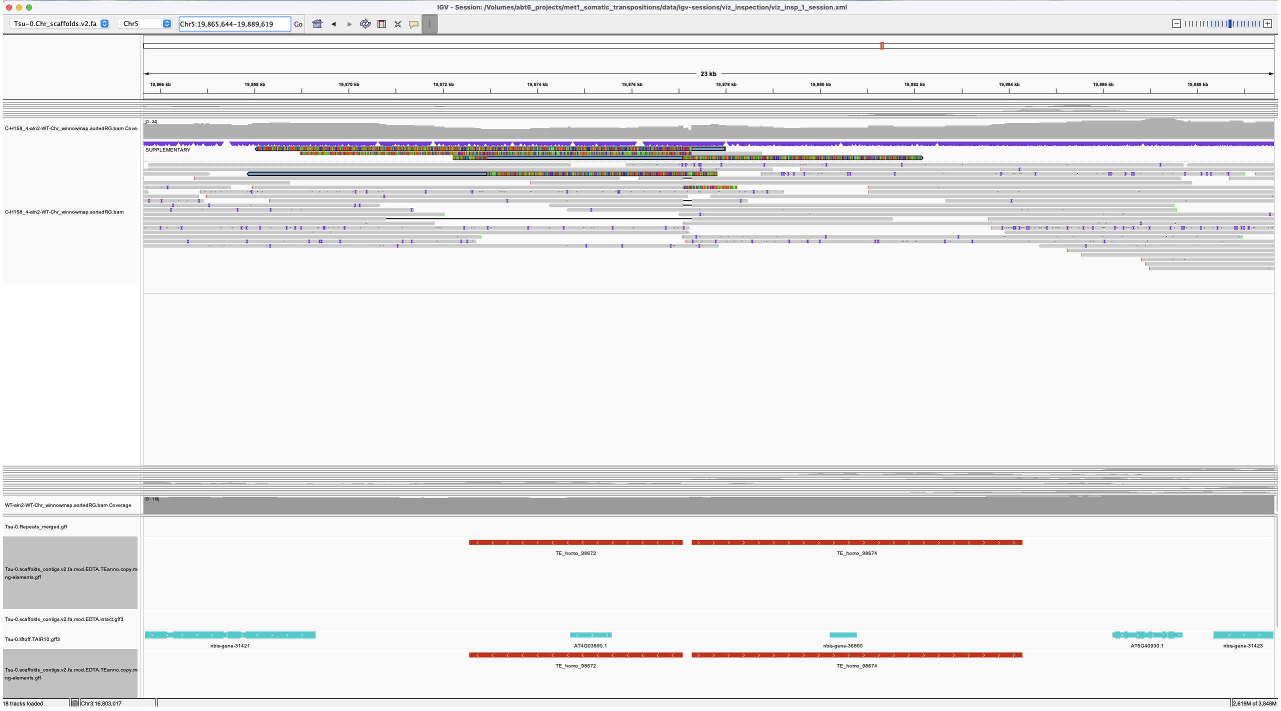

Rearrangements

It's the inversion of a VANDAL21

Hypermutable region

unsupported

> **DIFFICULT CASE TO AUTOMATE: m64079_221220_112036/61606528/ccs 1 6079 Chr3 16807887 16813968 15556 + ATHILA3_0_LTR m64079_221220_112036/61606528/ccs 7032 15381 Chr5 15871842 15881696 15556 + ATHILA2**

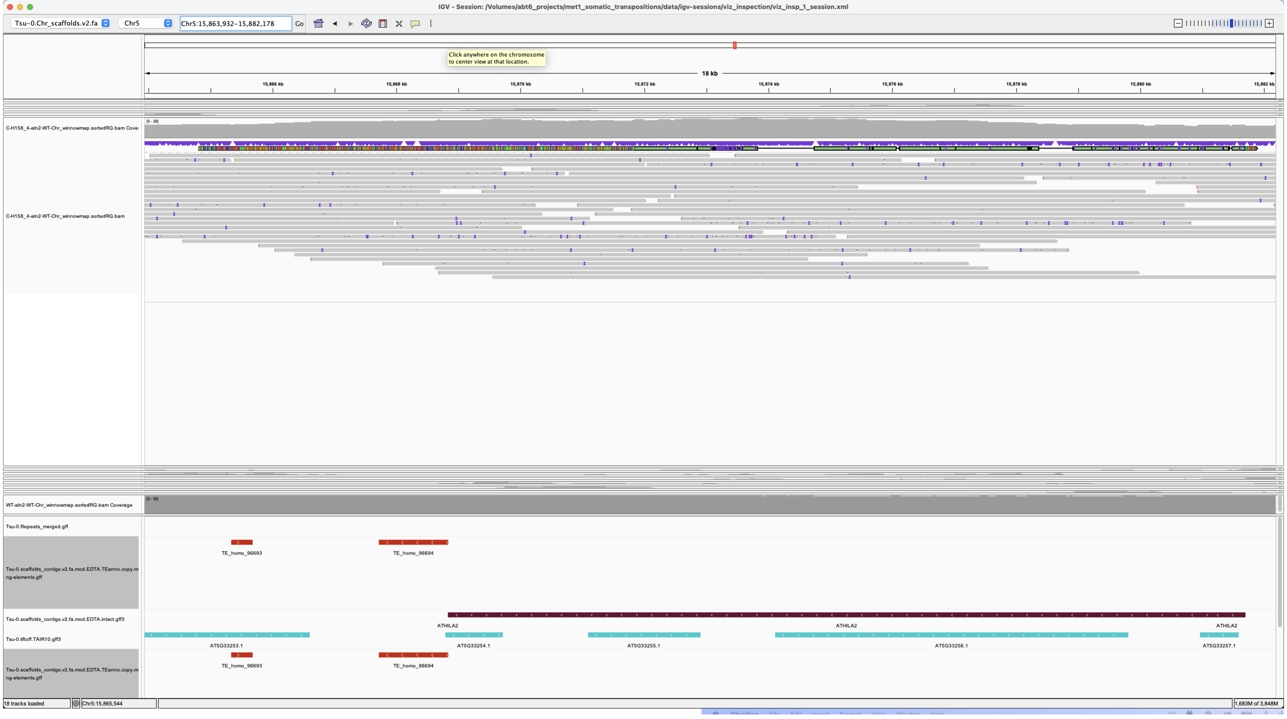

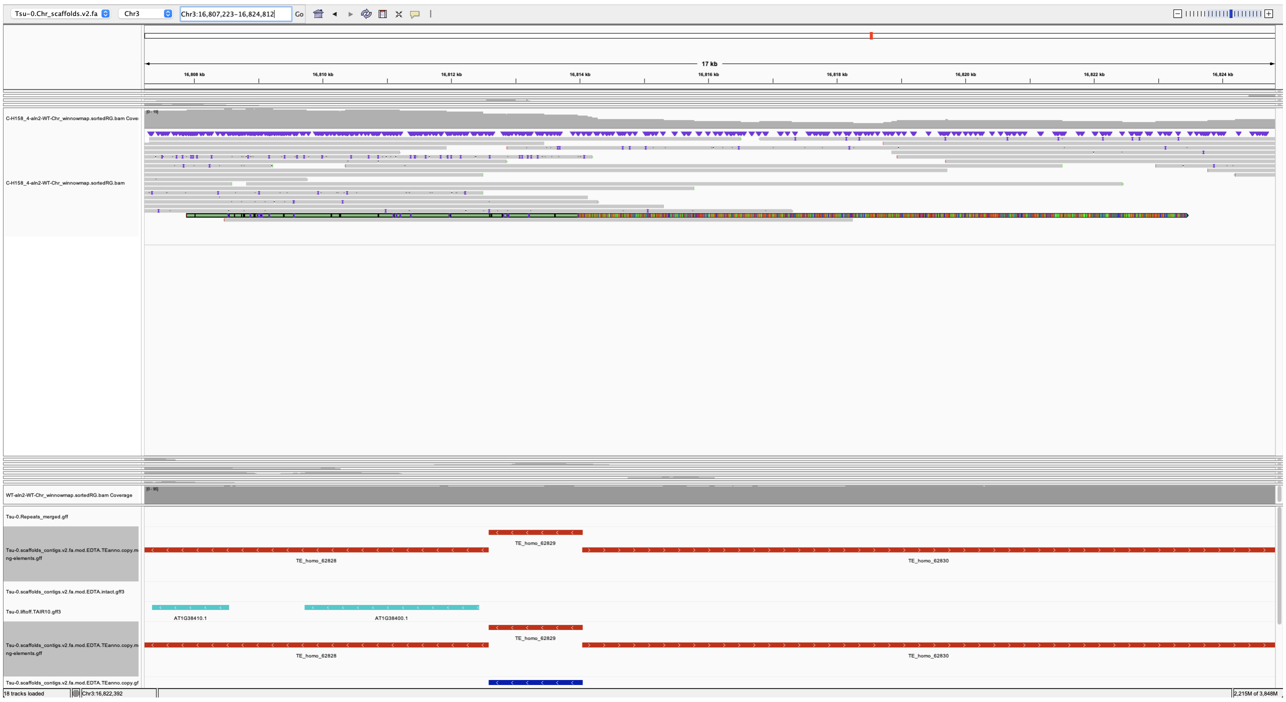

It can actually be a Athila mobilization but cannot be sure, the borders of the TE are not respected. Also many mutations.

Unsure

unsupported

Chr1 313952 313952 + 1 Chr3;16344522;16352497;VANDAL6 m64079_221220_112036/155912133/ccs met1_04

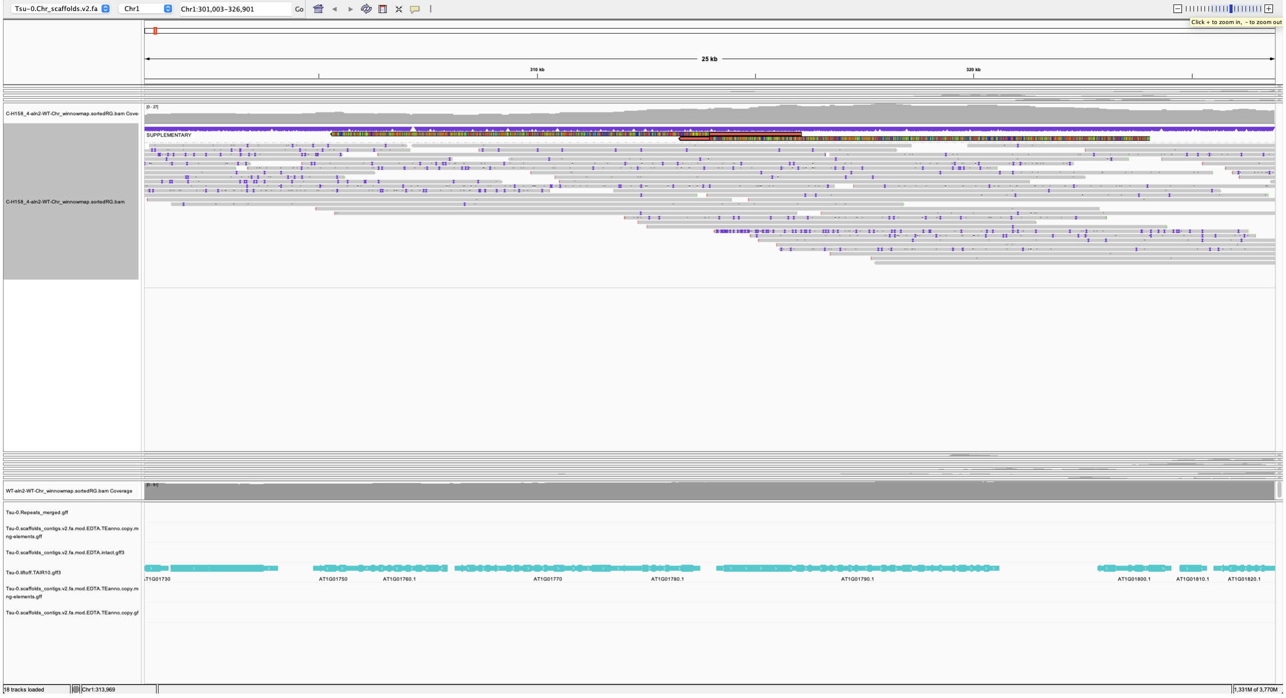

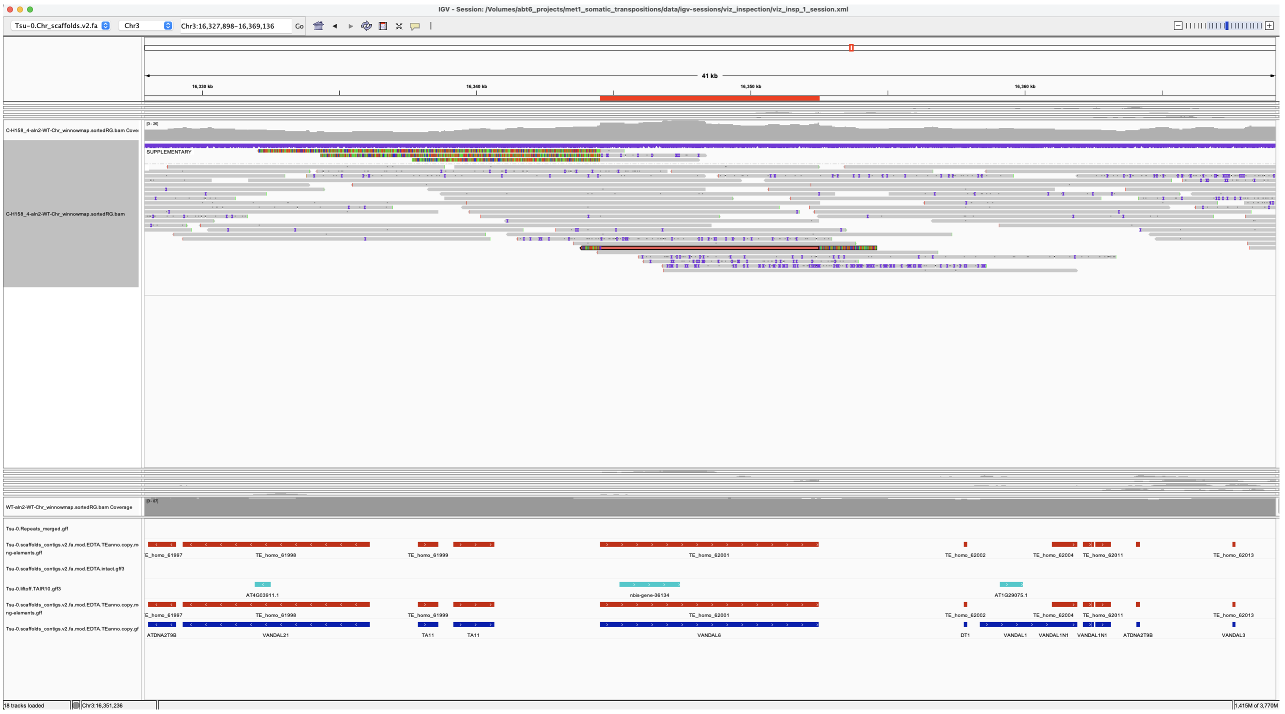

Central
TSD

Confirmed

> Chr1 14056050 14056050 + 1 Chr5;21419693;21425022;ATCOPIA93_Evade m64079_221220_112036/65735321/ccs met1_04

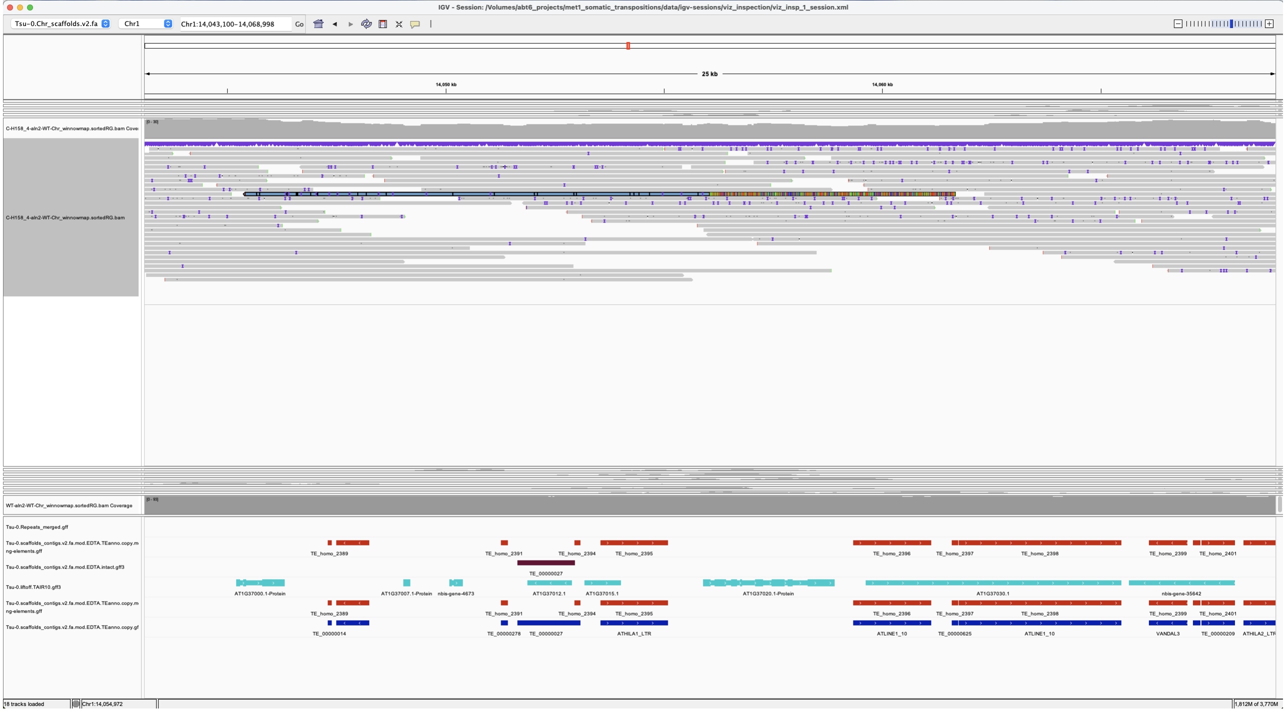

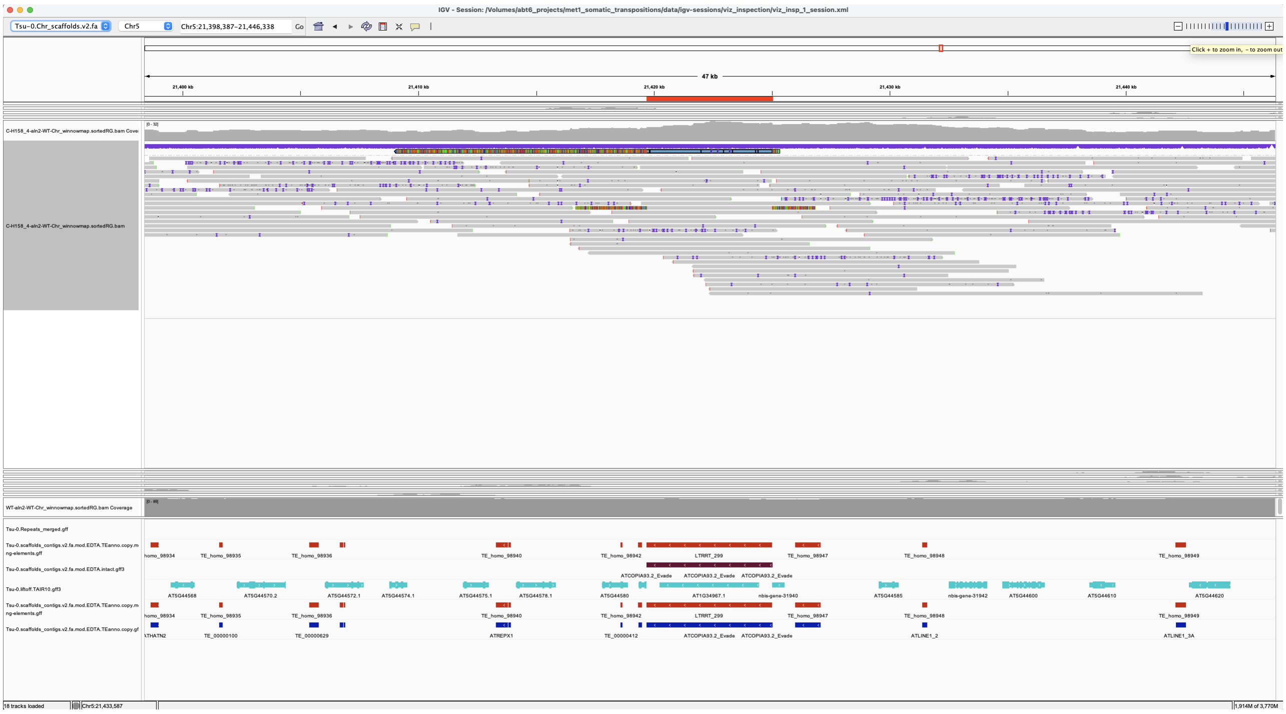

Partial

Confirmed

Chr2 6517591 6517591 - 1 Chr5;19152829;19160826;VANDAL21 m64079_221220_112036/167119051/ccs met1_04

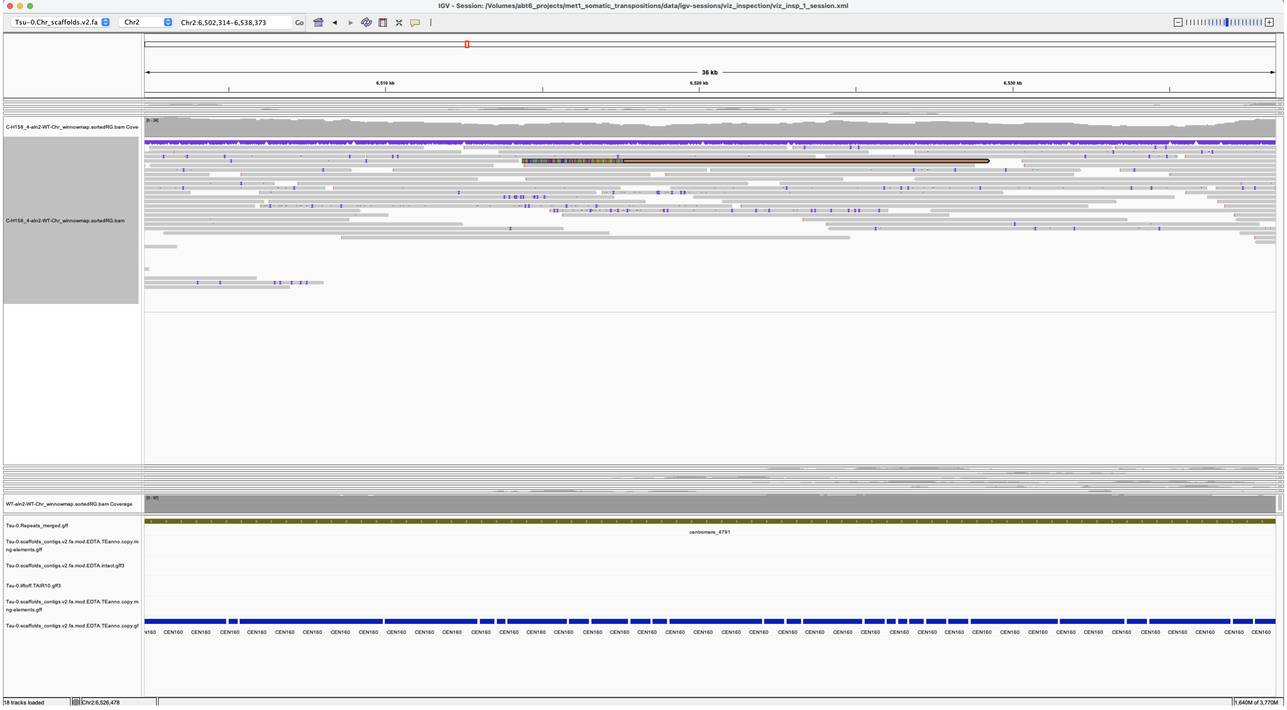

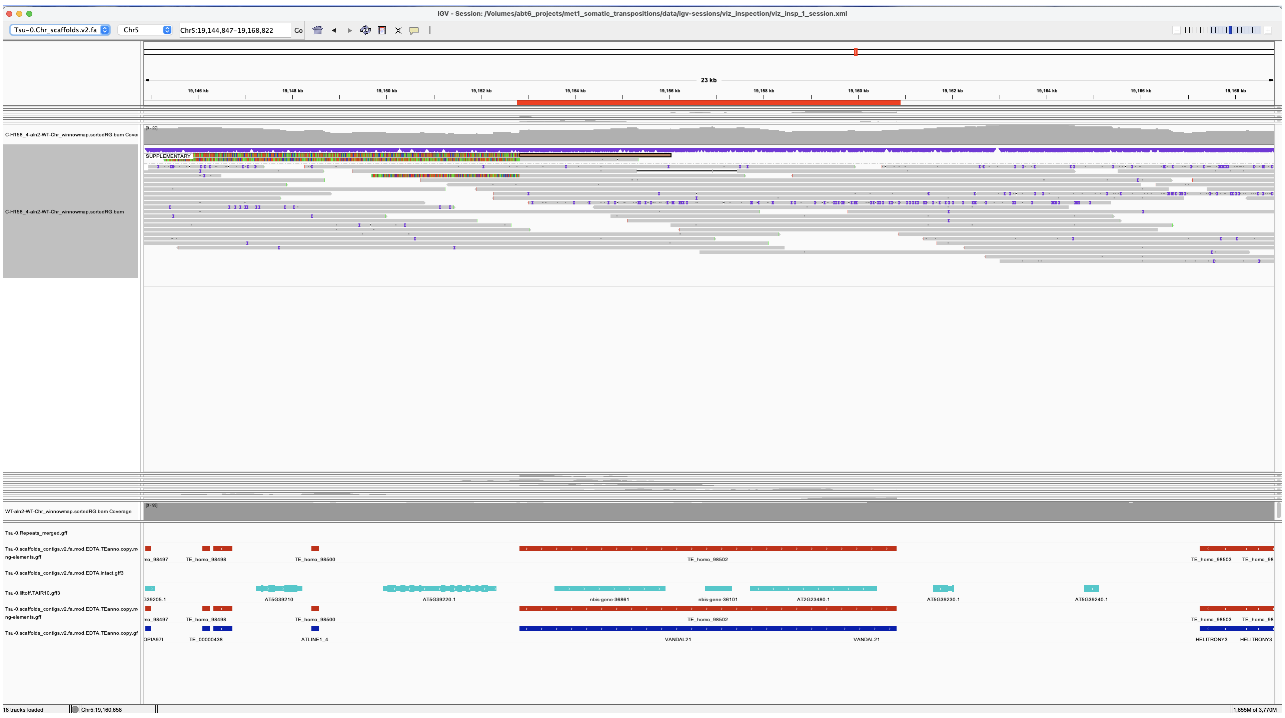

centromeric

Partial

Confirmed

Chr2 11738204 11738204 + 1 Chr3;16344522;16352497;VANDAL6 m64079_240212_113350/163775336/ccs met1_04

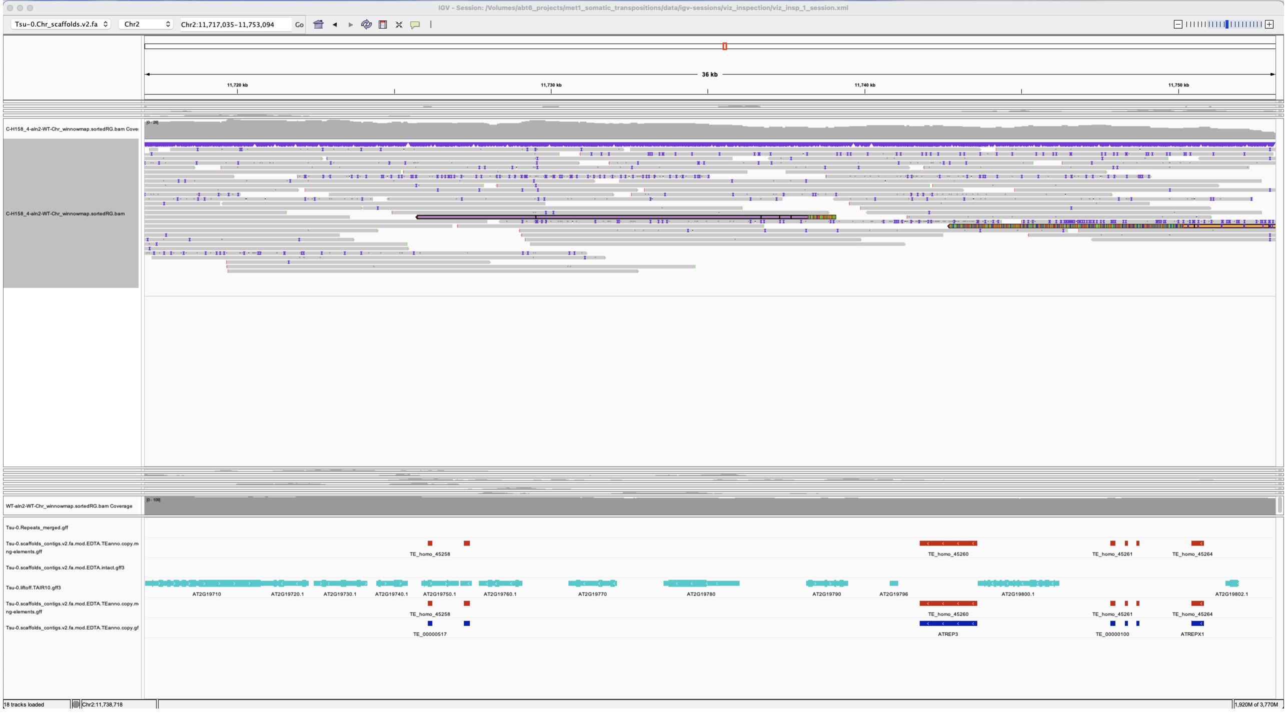

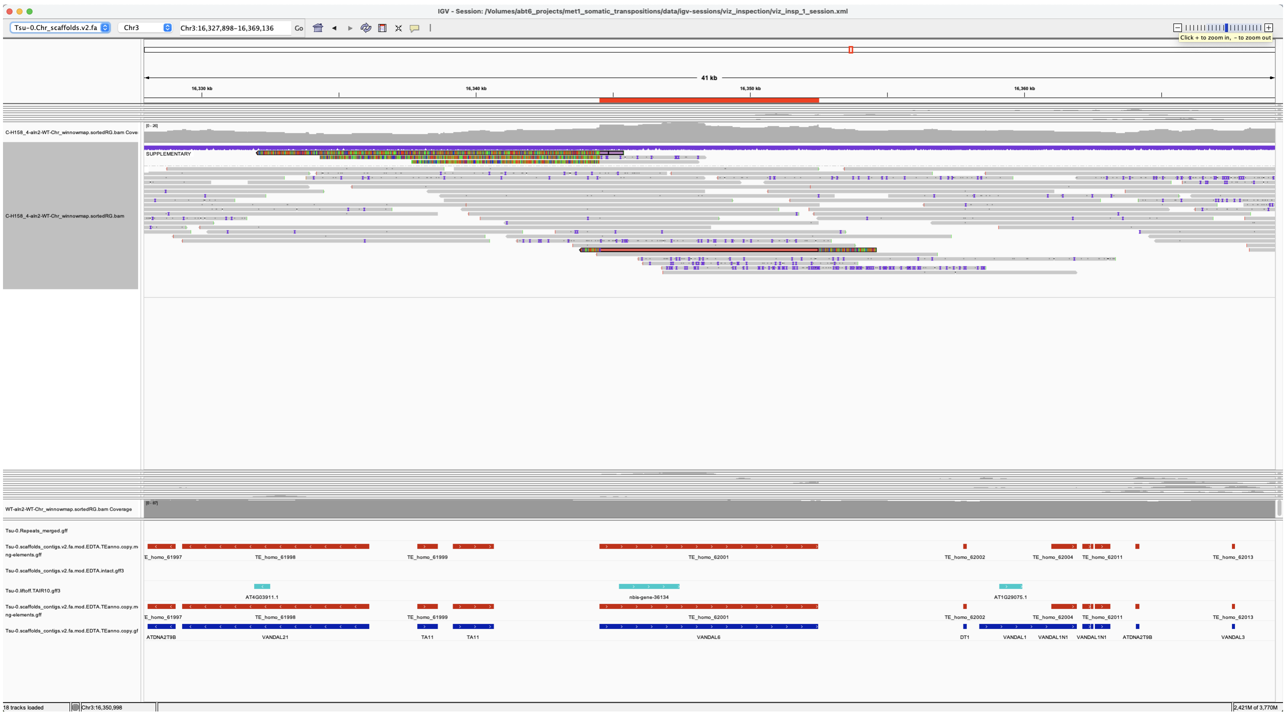

Partial

Confirmed

> Chr2 14445570 14445570 + 1 Chr3;20158137;20166150;VANDAL6 m64079_240212_113350/160827795/ccs met1_04

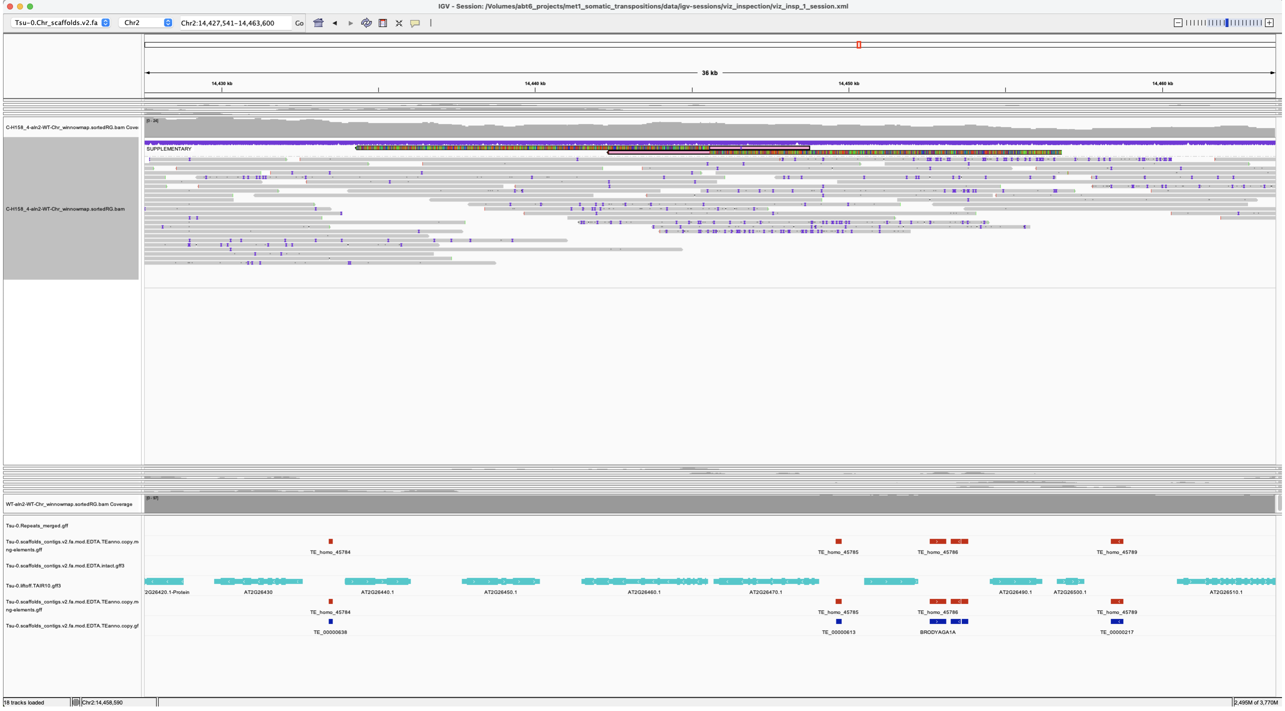

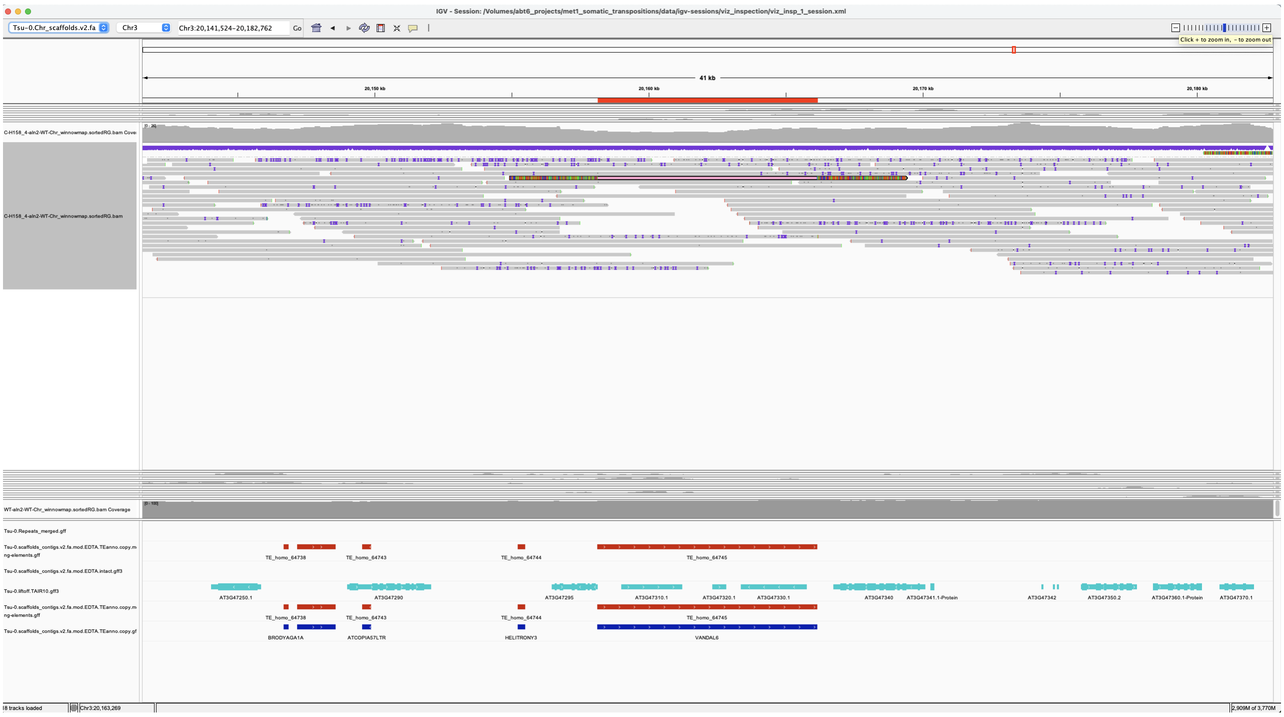

Central

TSD YES

Confirmed

> Chr3 24656824 24656824 + 1 Chr5;21419693;21425022;ATCOPIA93_Evade m64079_221220_112036/144245867/ccs met1_04

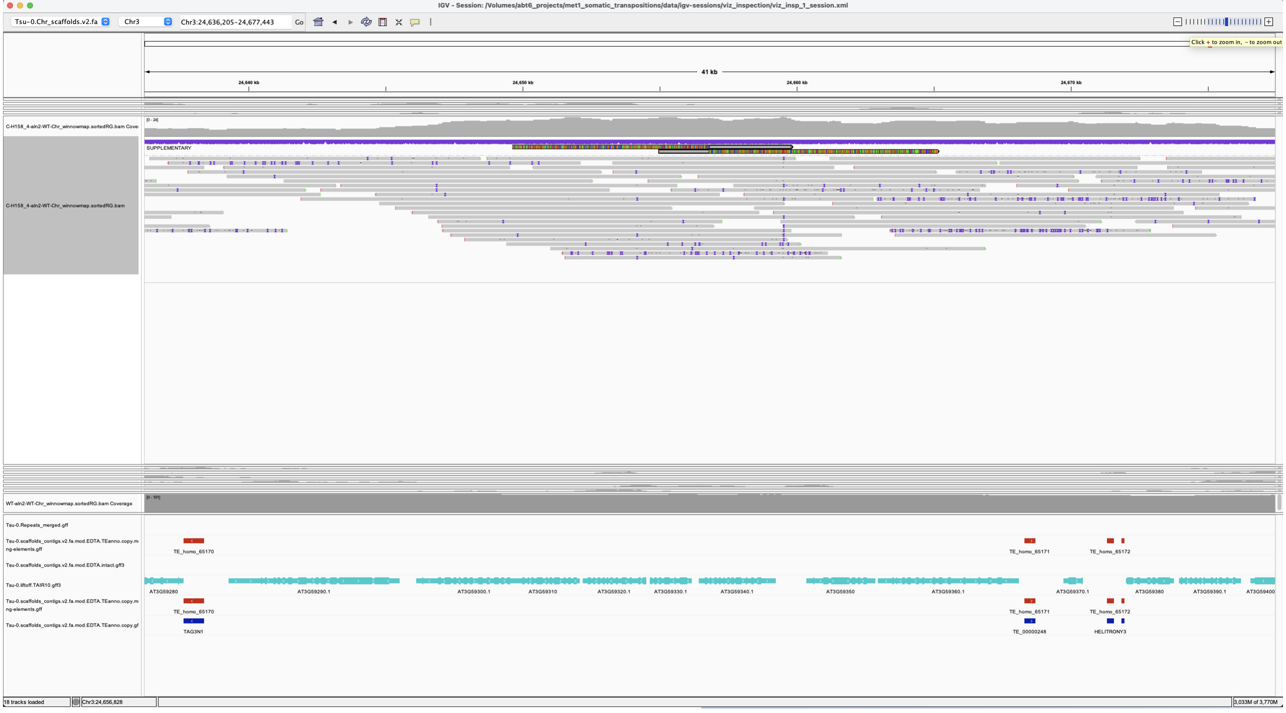

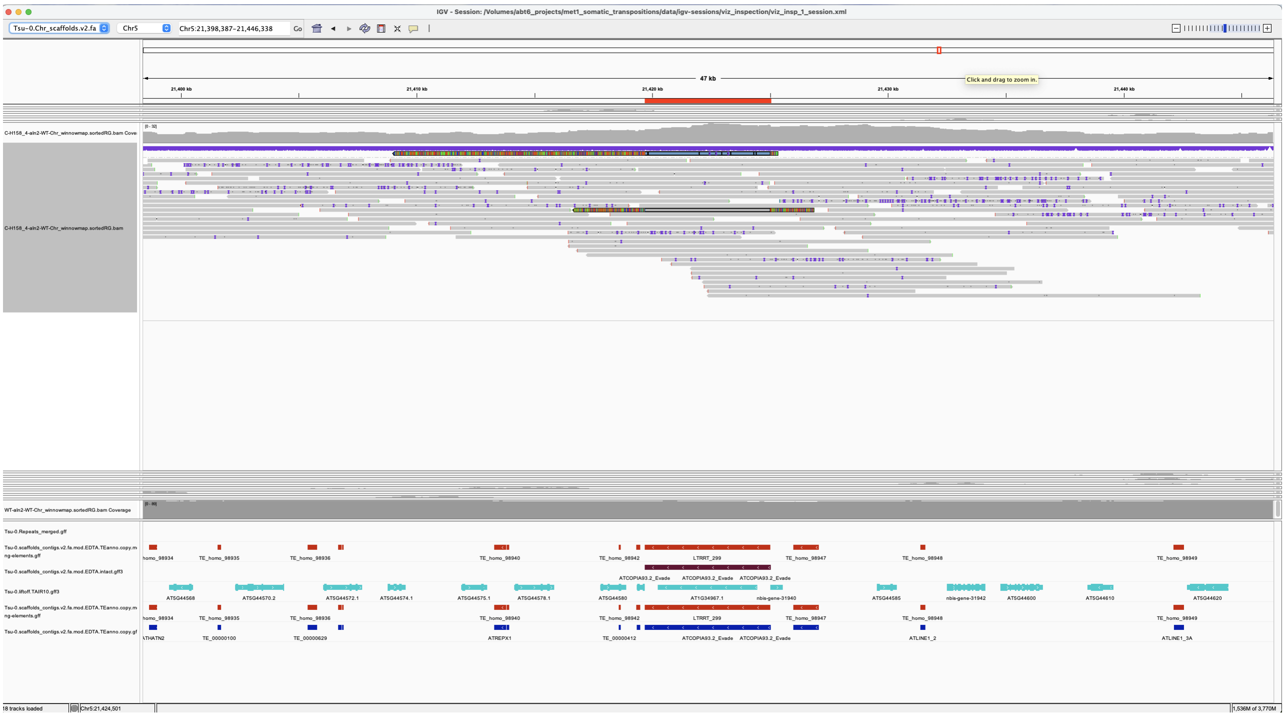

Central
TSD

Confirmed

Chr4 11762271 11762271 + 1 Chr3;16344522;16352497;VANDAL6 m64079_221220_112036/31525296/ccs met1_04

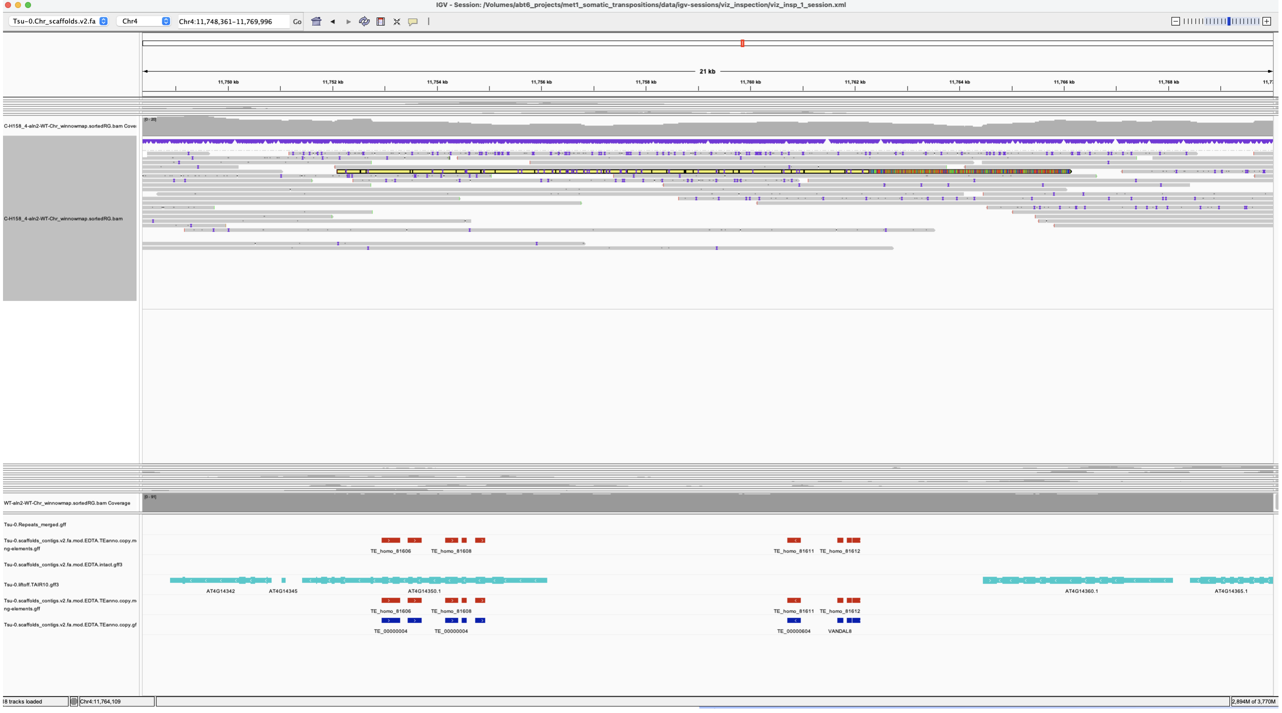

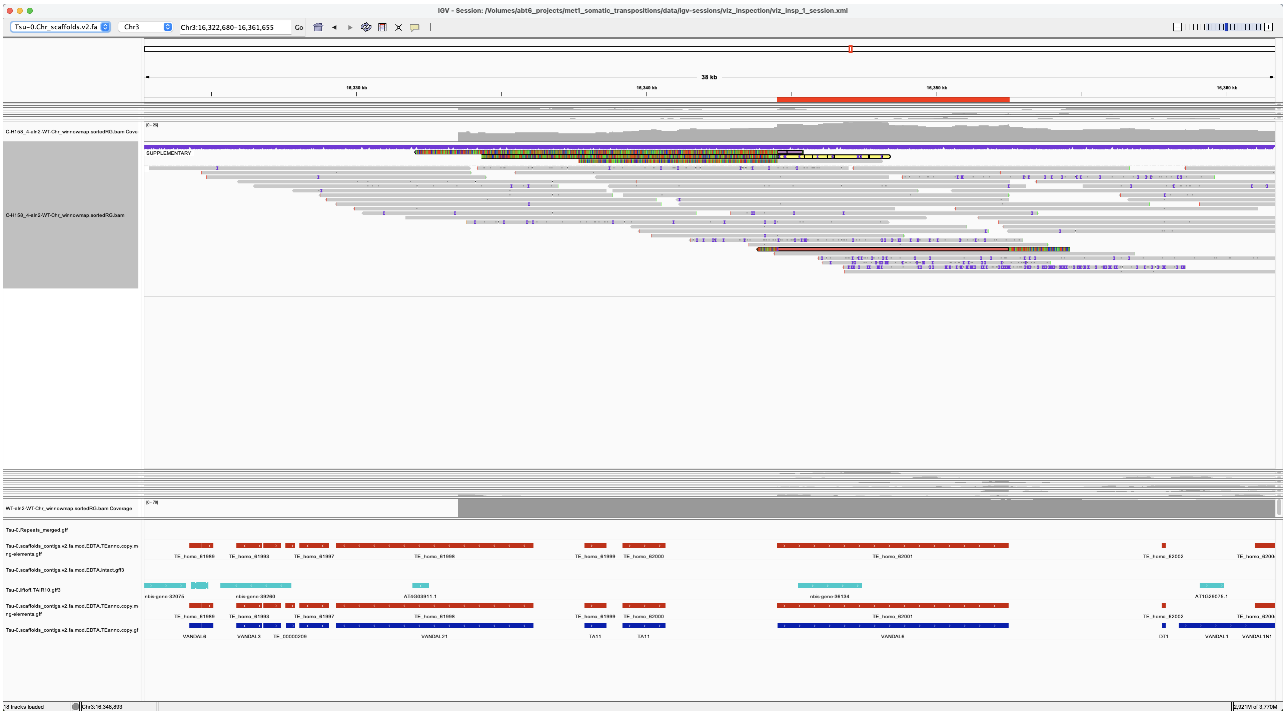

Partial

Confirmed

Chr4 15475942 15475942 - 1 Chr5;19877283;19884298;VANDAL21 m64079_240212_113350/7276043/ccs met1_04

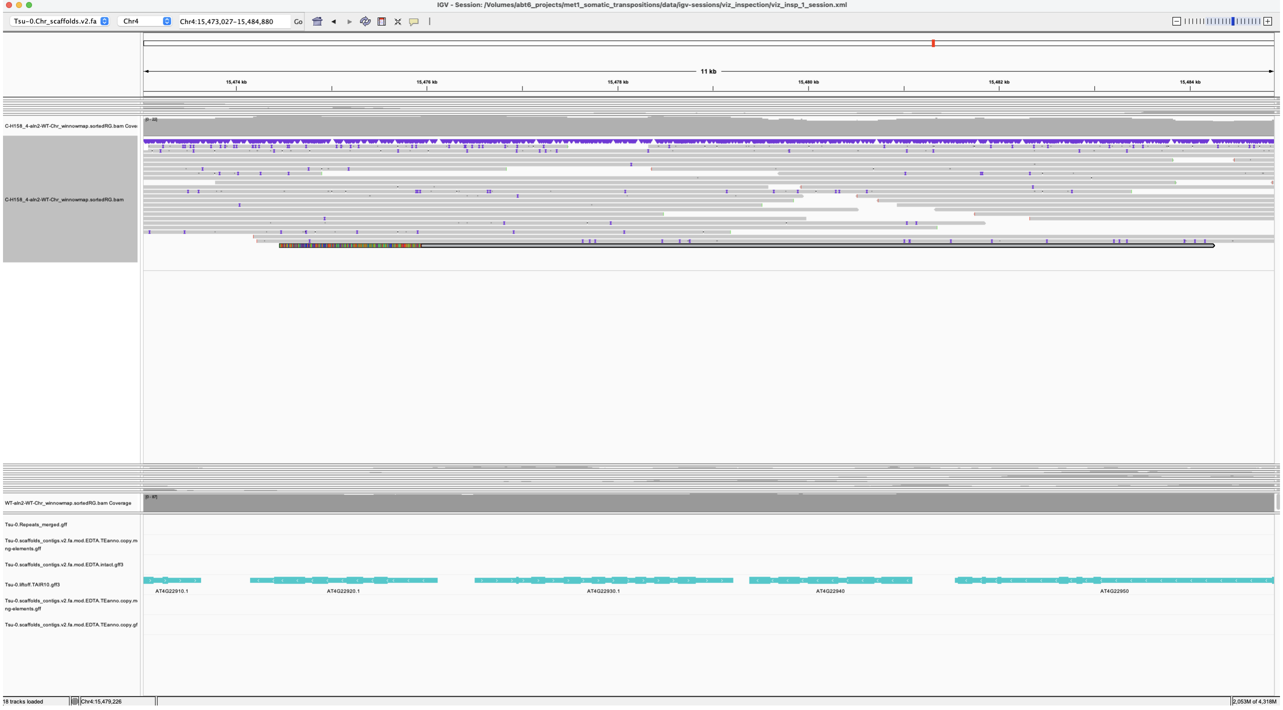

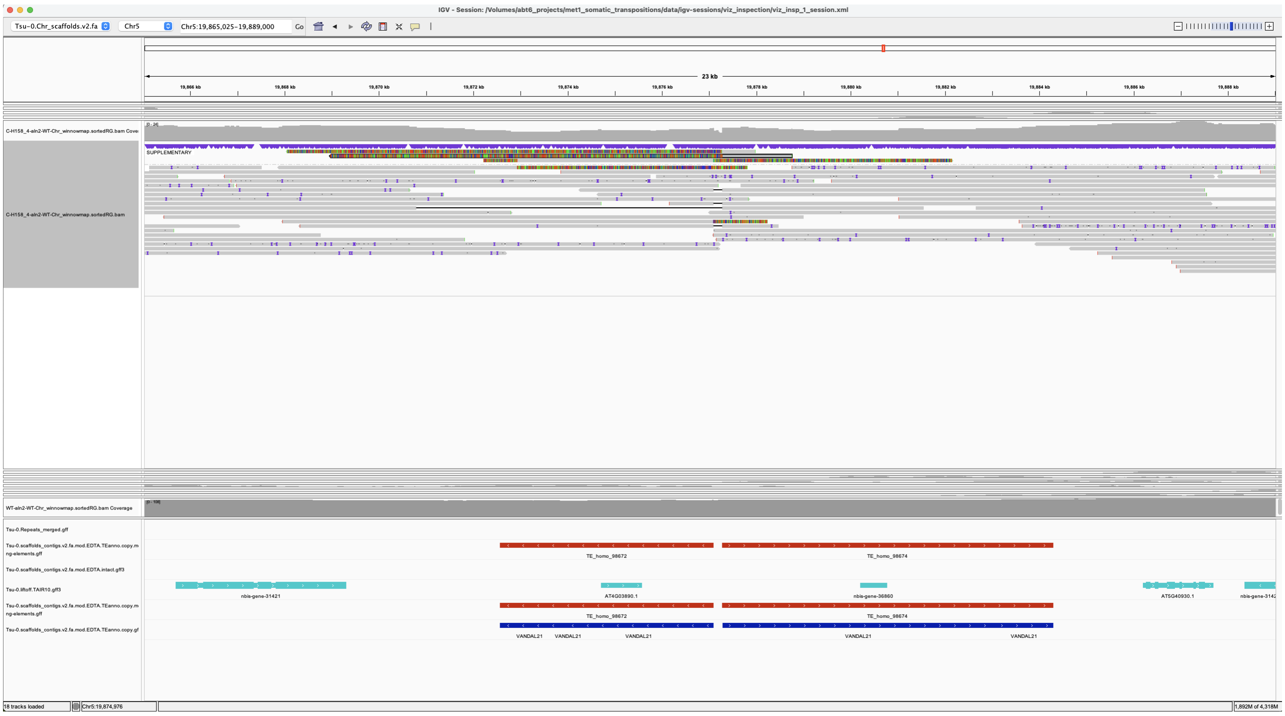

Hypermutable region

Re-arrangement

Chr5 4513218 4513218 + 1 Chr5;19152829;19160826;VANDAL21 m64079_240212_113350/87360859/ccs met1_04

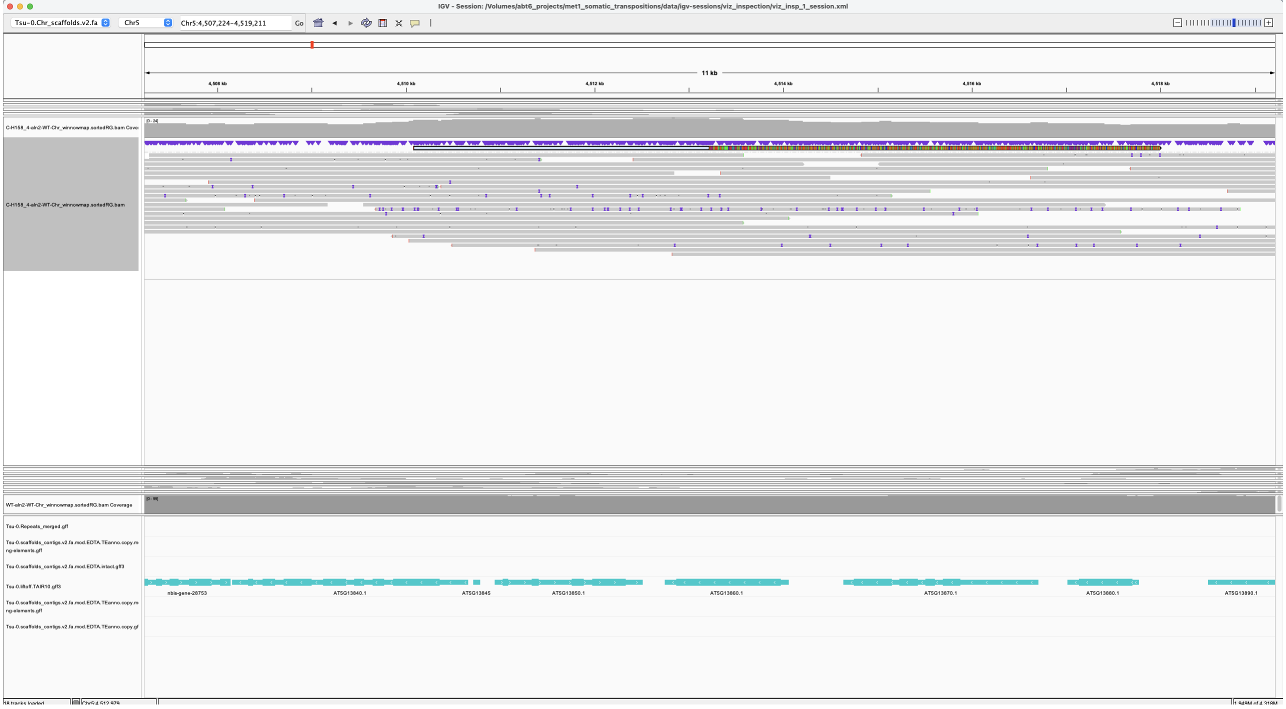

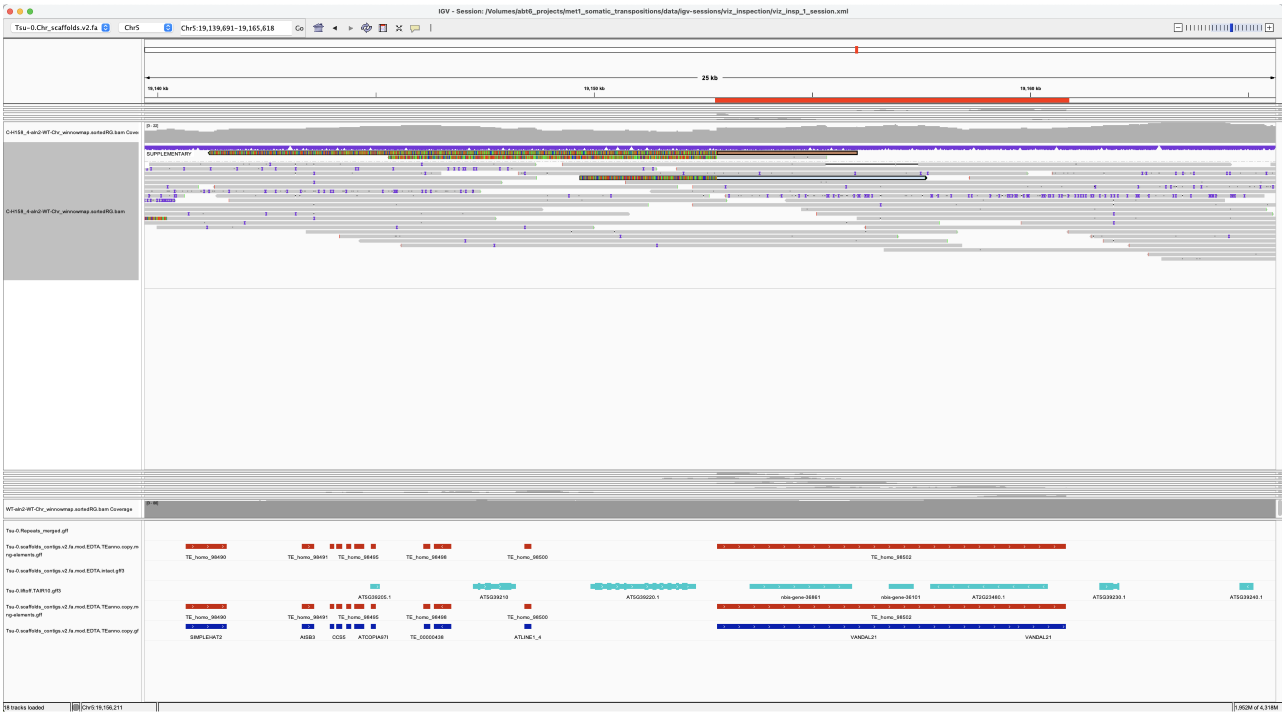

Partial

Confirmed

Chr5 6803479 6803479 - 1 Chr3;16344522;16352497;VANDAL6 m64079_221220_112036/133237592/ccs met1_04

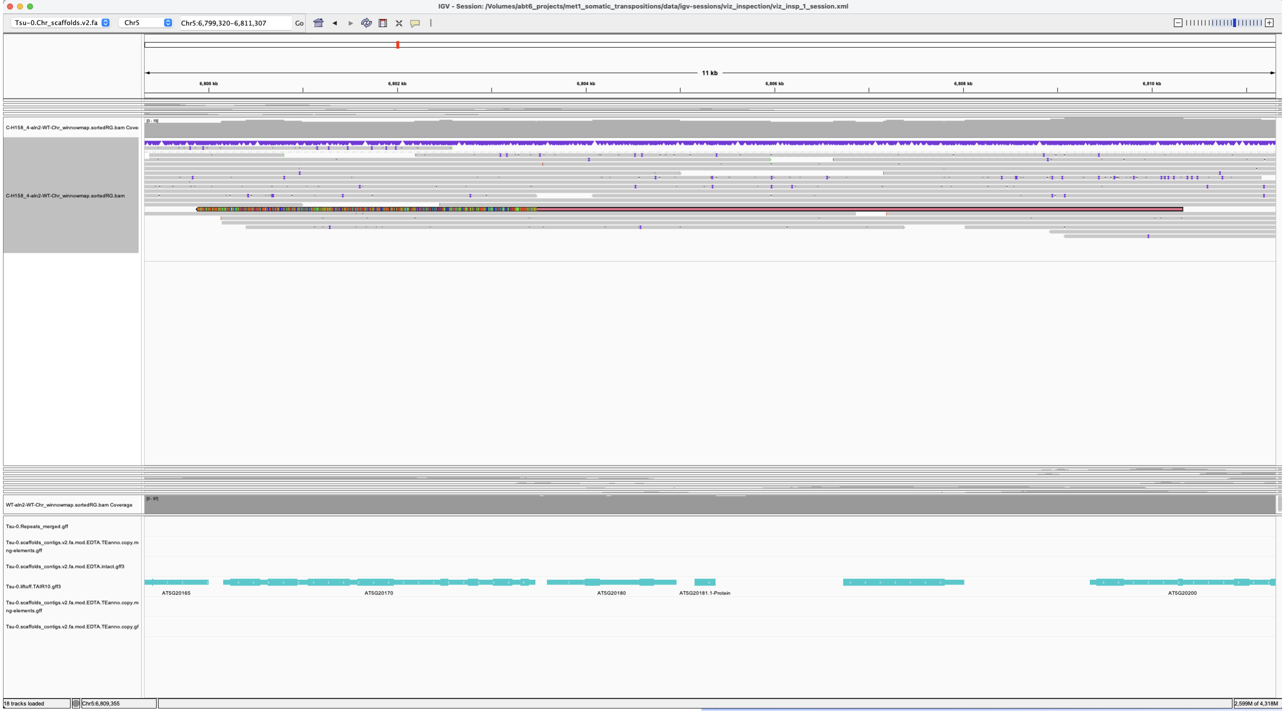

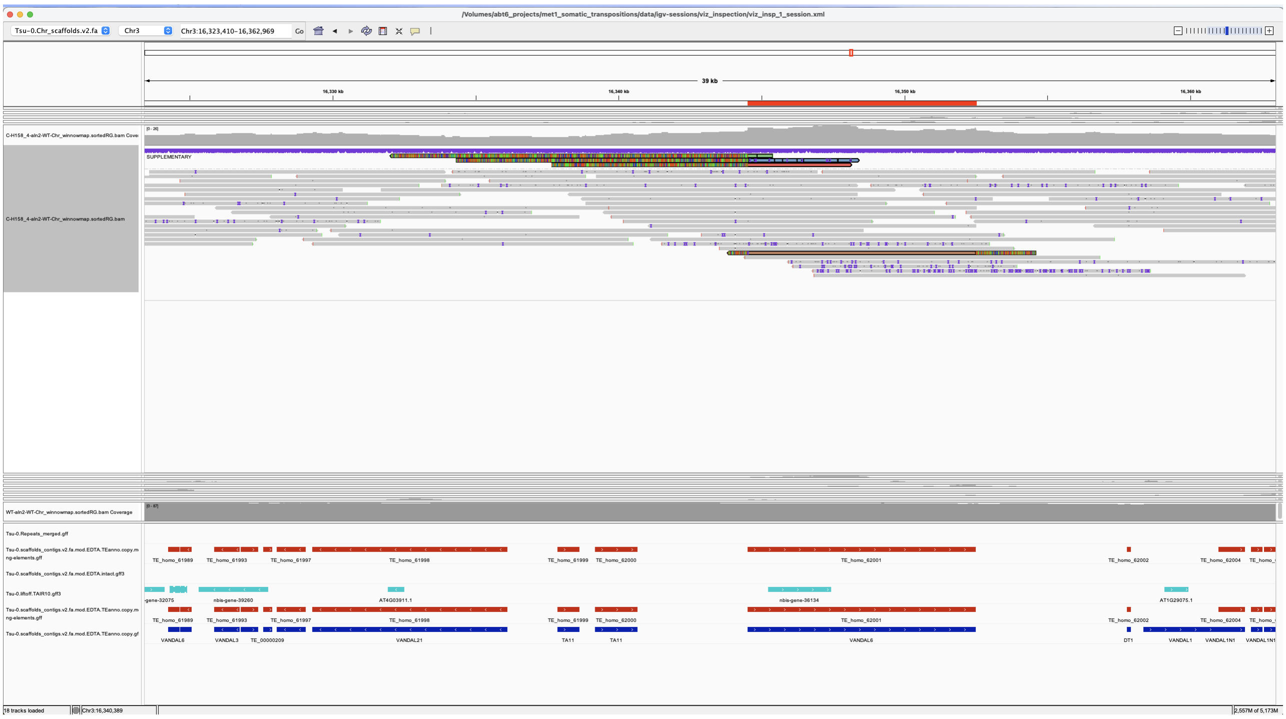

Partial

Confirmed

Chr5 19140216 19140216 - 1 Chr5;19152829;19160826;VANDAL21 m64079_221220_112036/50530220/ccs met1_04

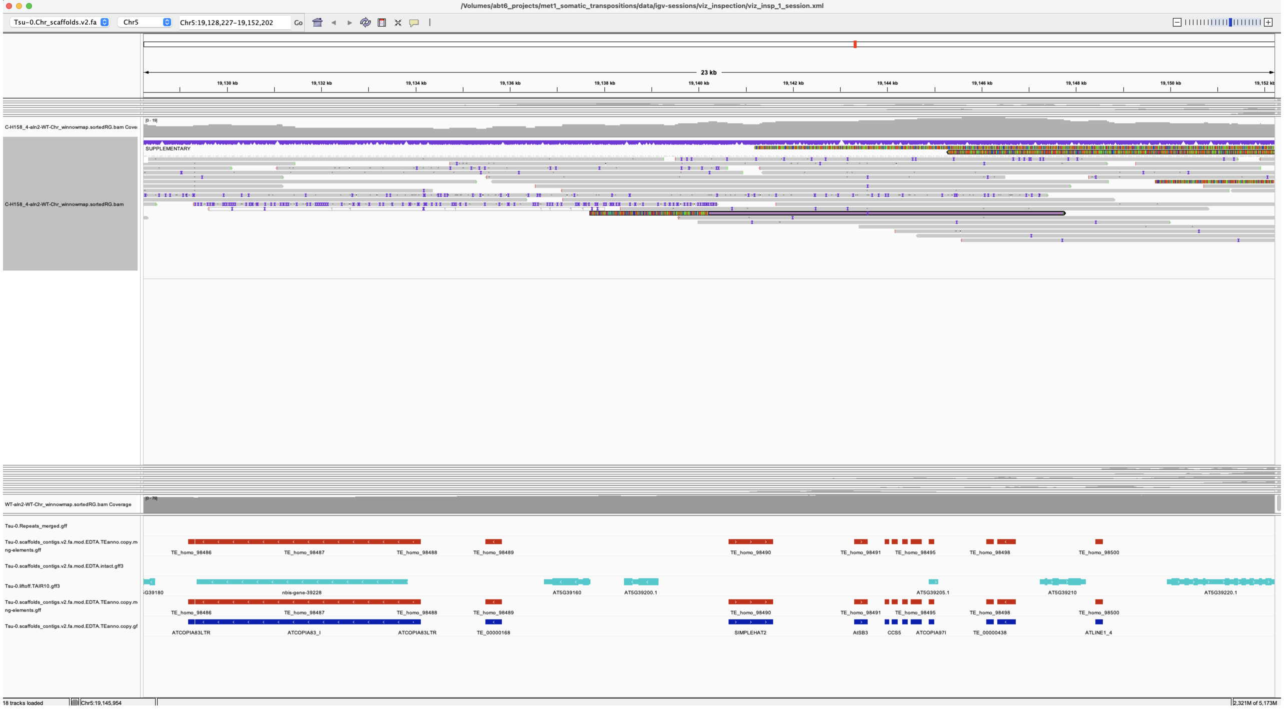

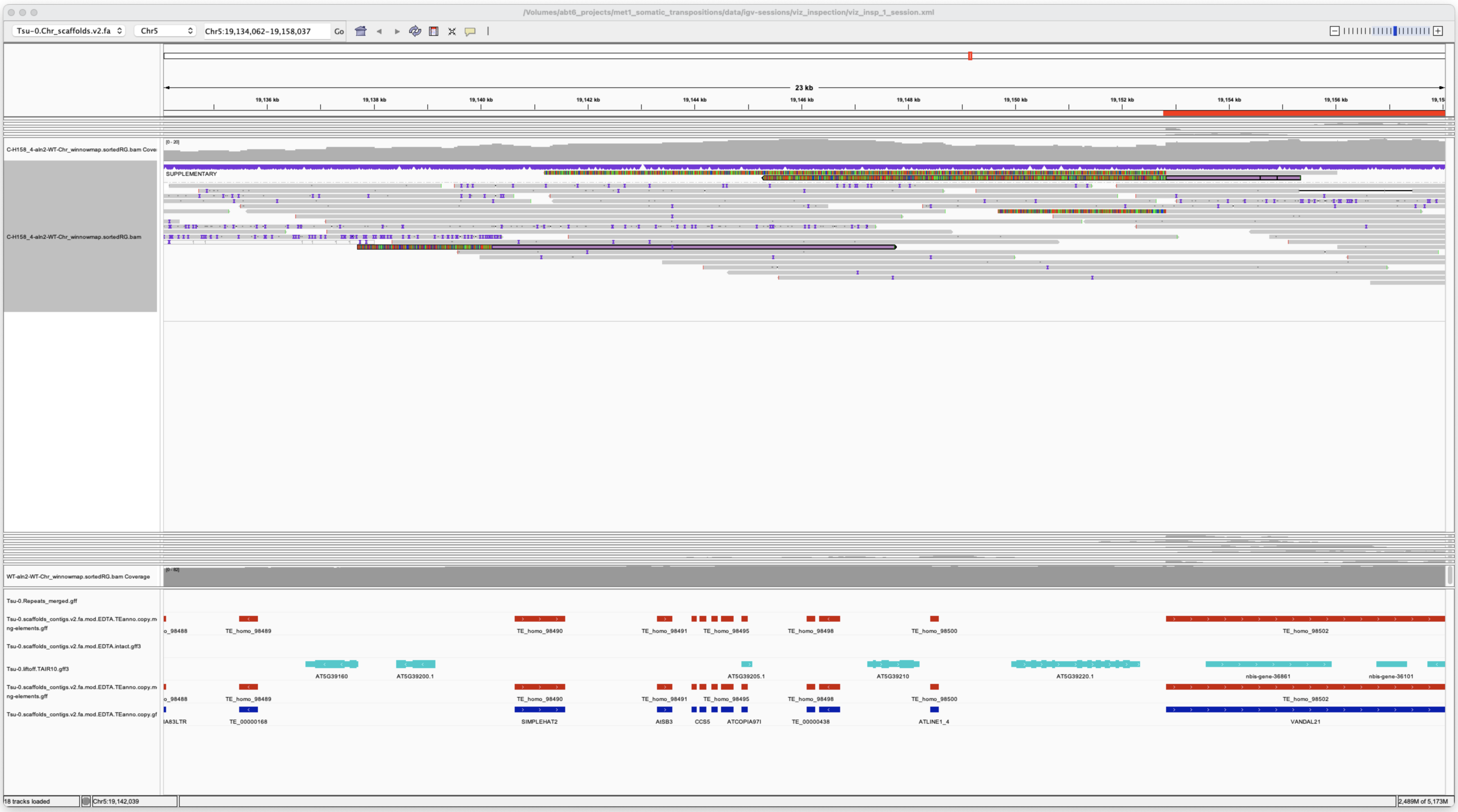

Most probably just a:

Rearrangement

unsupported

---

met1_05

DIFFICULT CASE TO AUTOMATE: m64079_221220_112036/49744130/ccs 0 660 Chr2 9100020 9100681 11914 - ATMU10 m64079_221220_112036/49744130/ccs 653 8652 Chr5 19152825 19160827 11914 + VANDAL21 m64079_221220_112036/49744130/ccs 8651 11914 Chr2 9096771 9100037 11914 - .

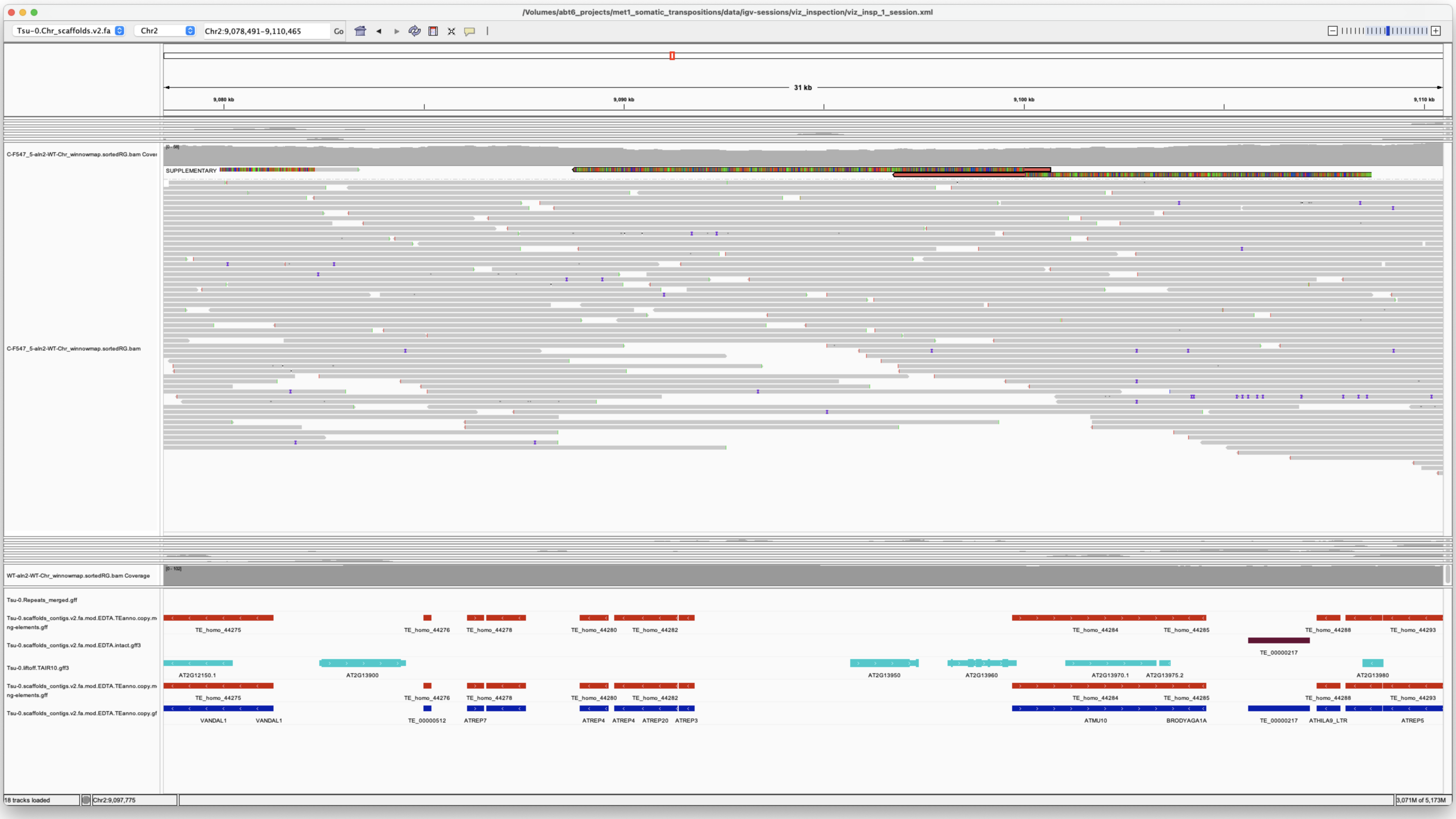

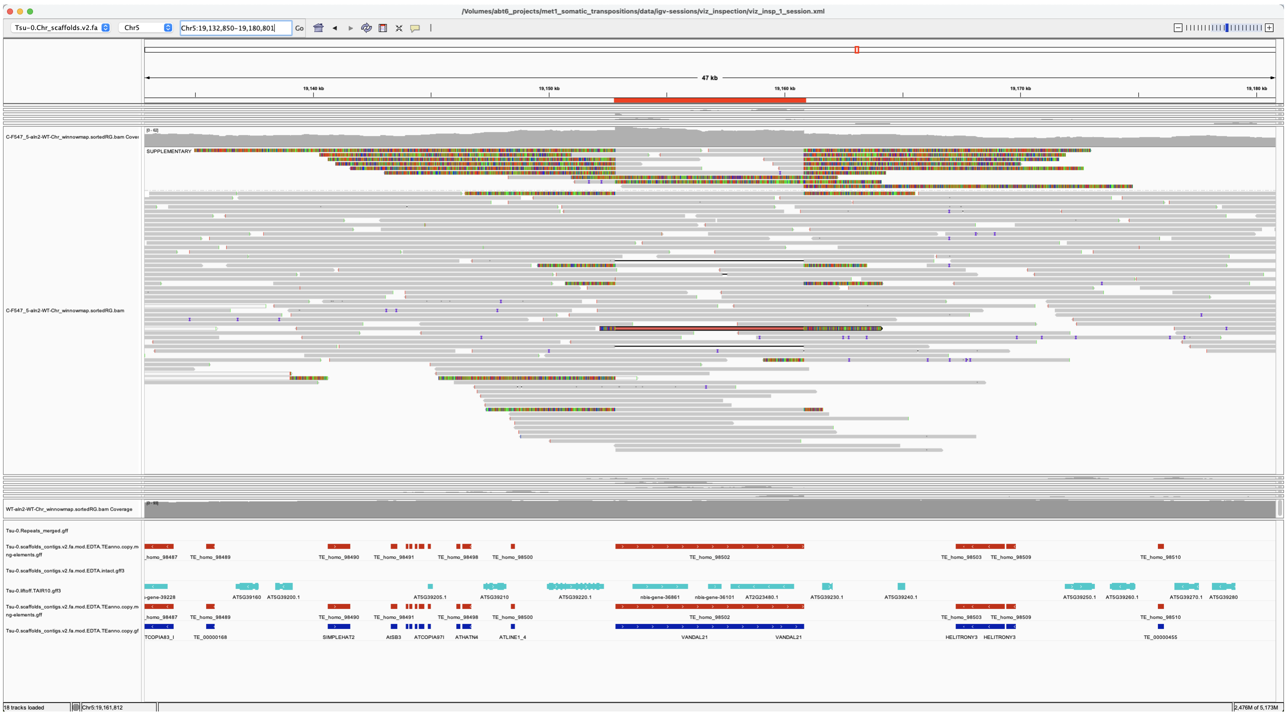

Central
TSD

Confirmed

DIFFICULT CASE TO AUTOMATE: m64079_240212_113350/122815669/ccs 1 2694 Chr5 19877279 19879977 14584 - VANDAL21 m64079_240212_113350/122815669/ccs 2694 14038 Chr5 19865736 19877095 14584 + . m64079_240212_113350/122815669/ccs 14037 14583 Chr5 19865200 19865746 14584 - .

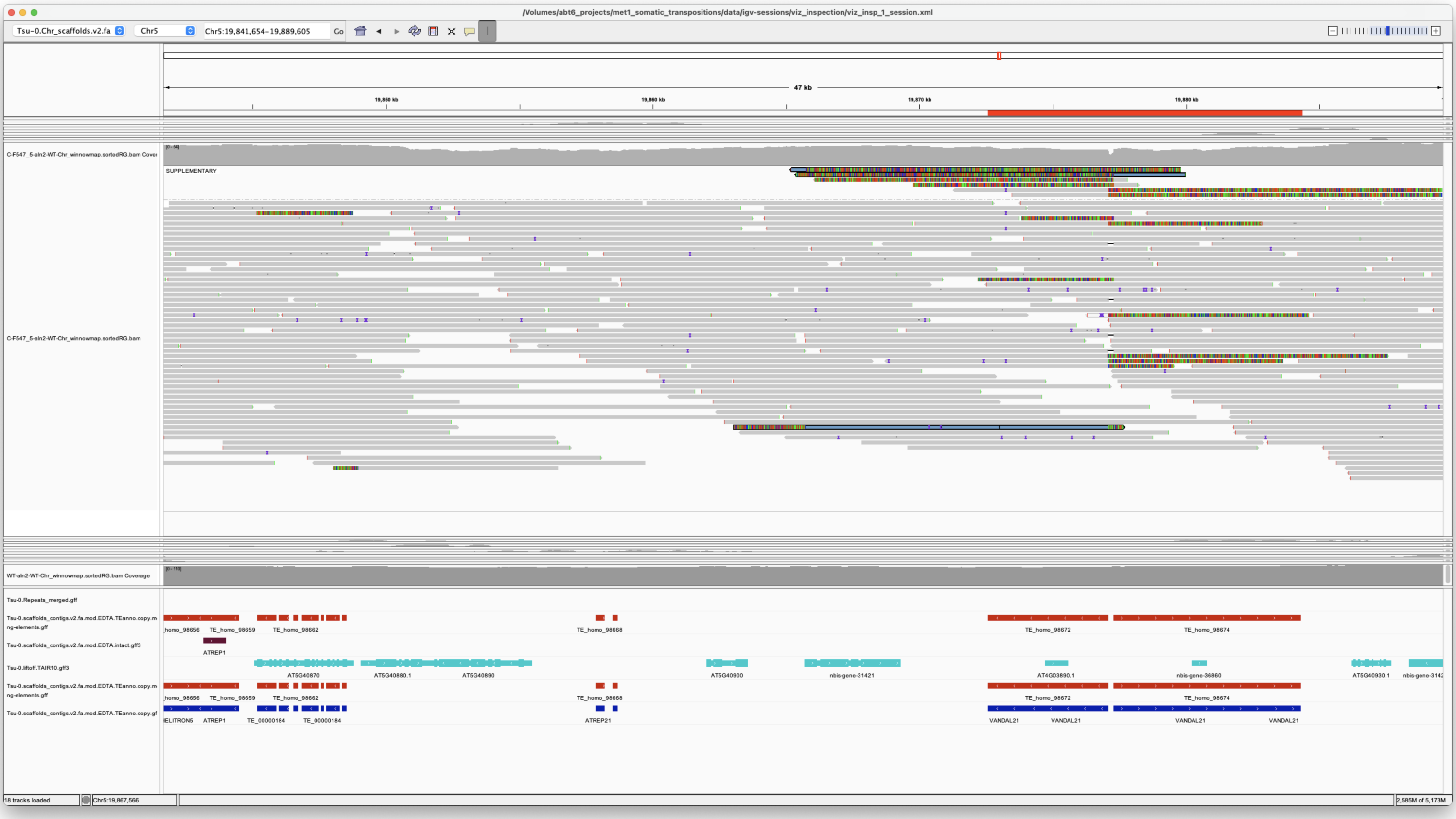

Hypermutable region

Rearrangement

unsupported

Chr1 3916144 3916144 - 1 Chr1;11941106;11946436;ATCOPIA93_Evade m64079_240212_113350/21496018/ccs met1_05

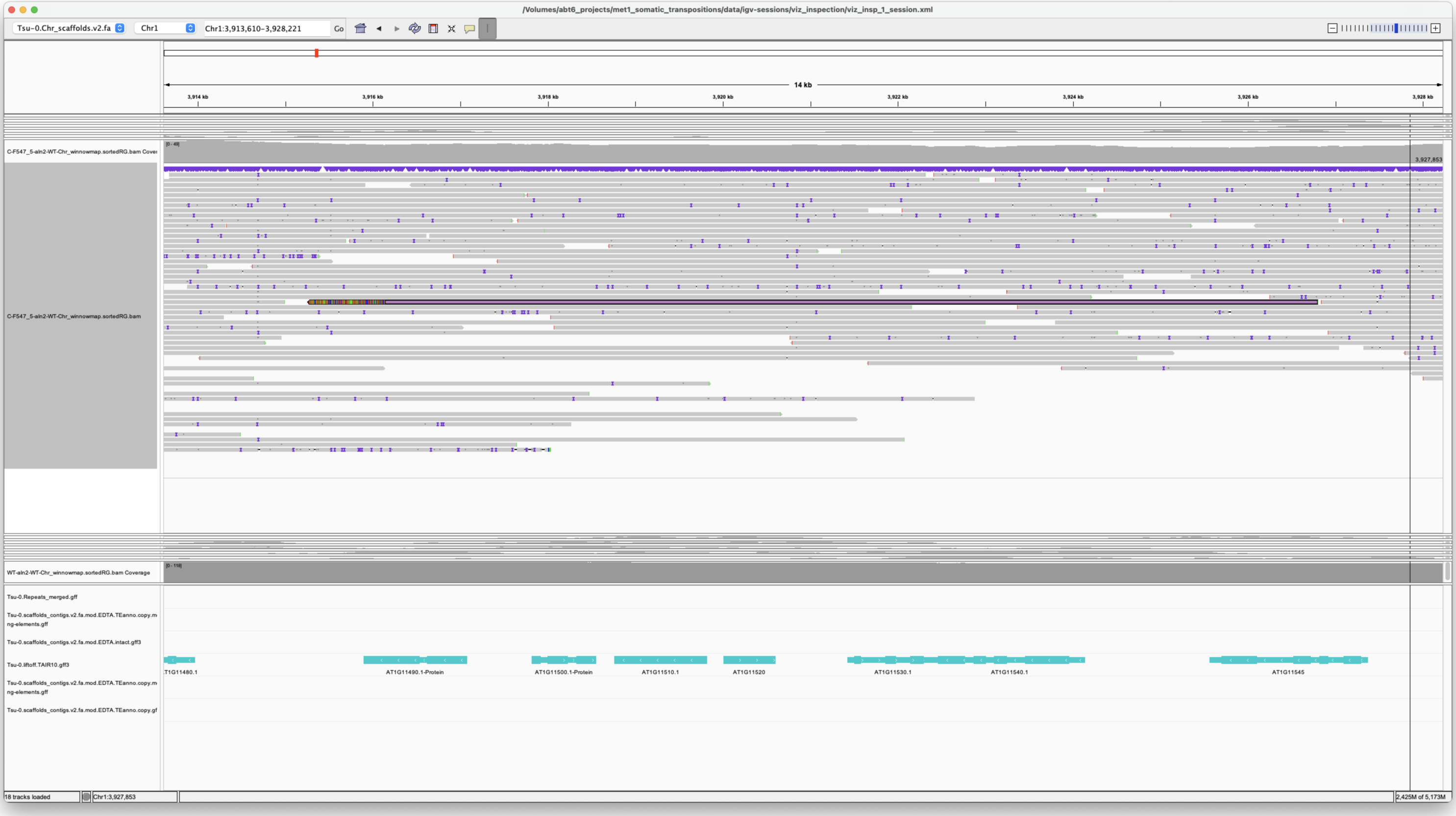

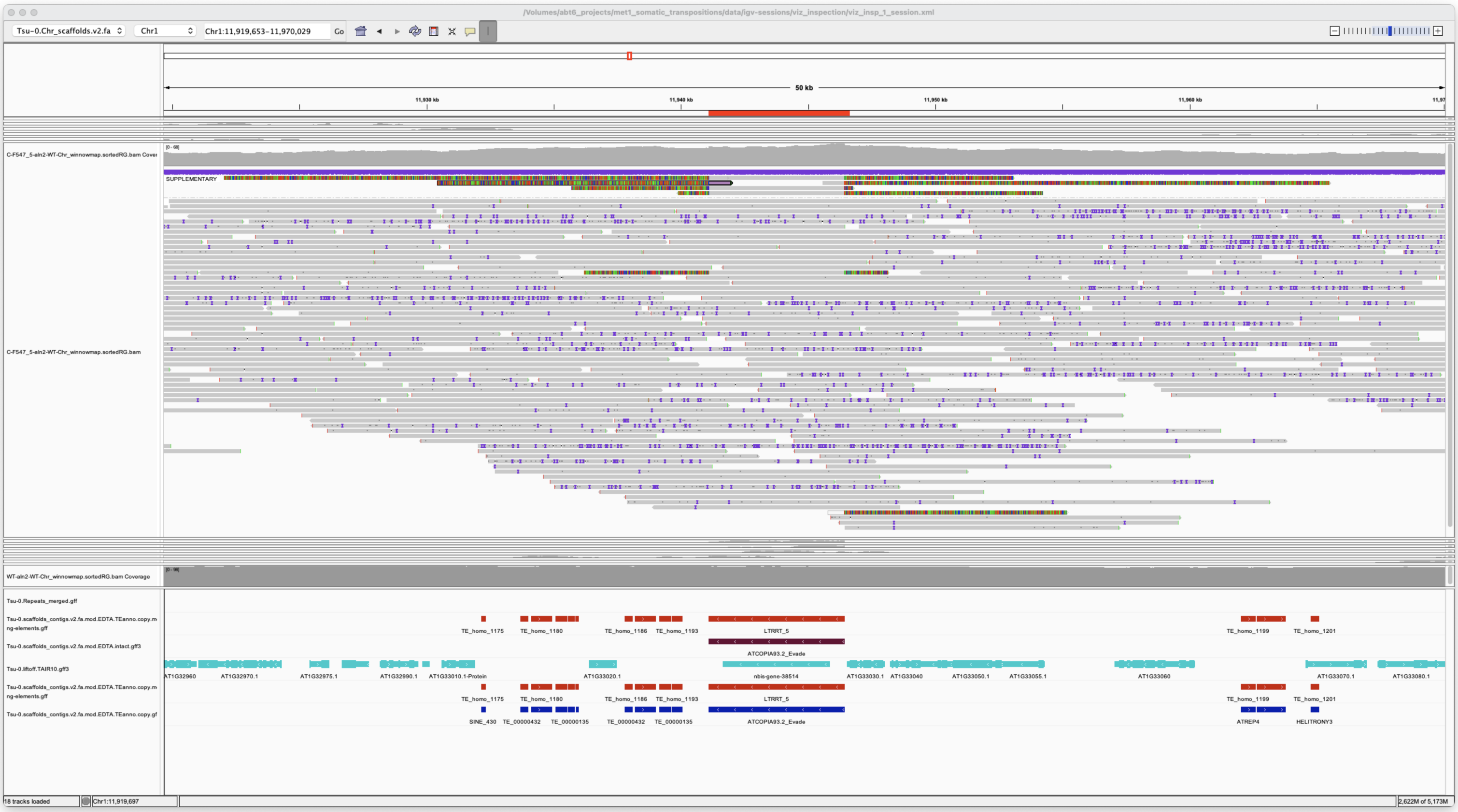

Partial

Confirmed

Chr1 6529799 6529799 - 1 Chr5;19152829;19160826;VANDAL21 m64079_221220_112036/19006919/ccs met1_05

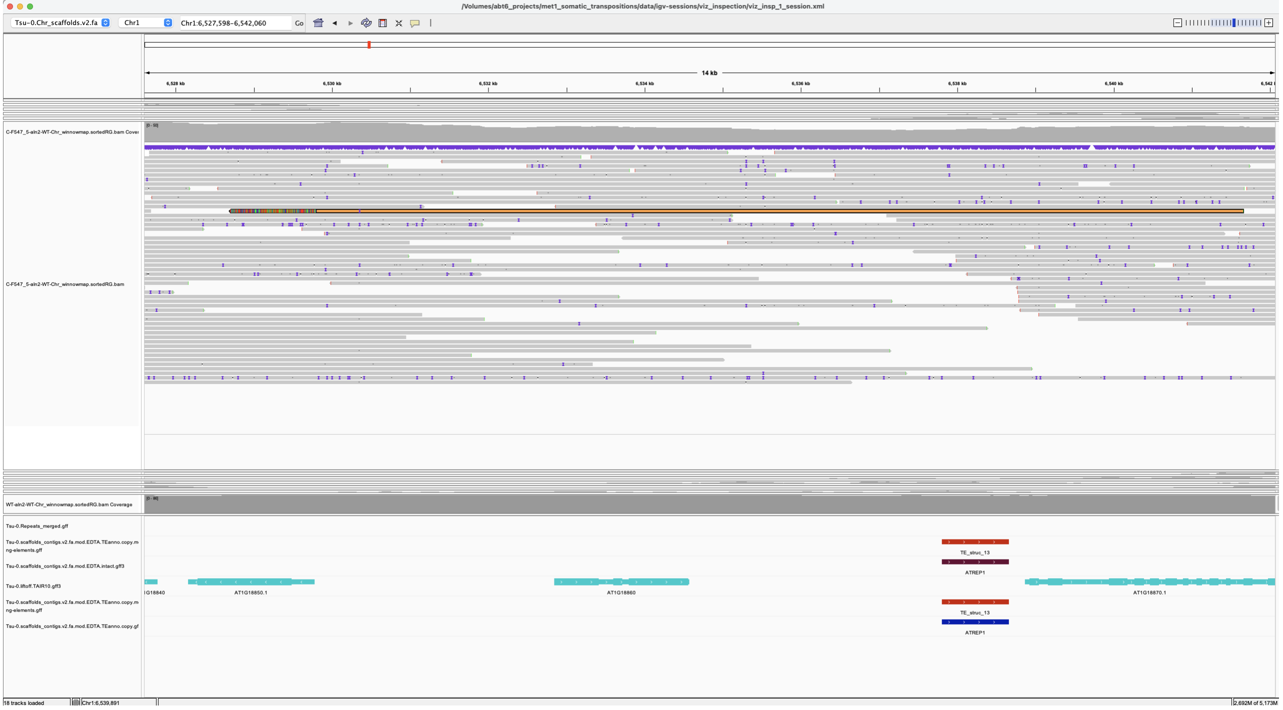

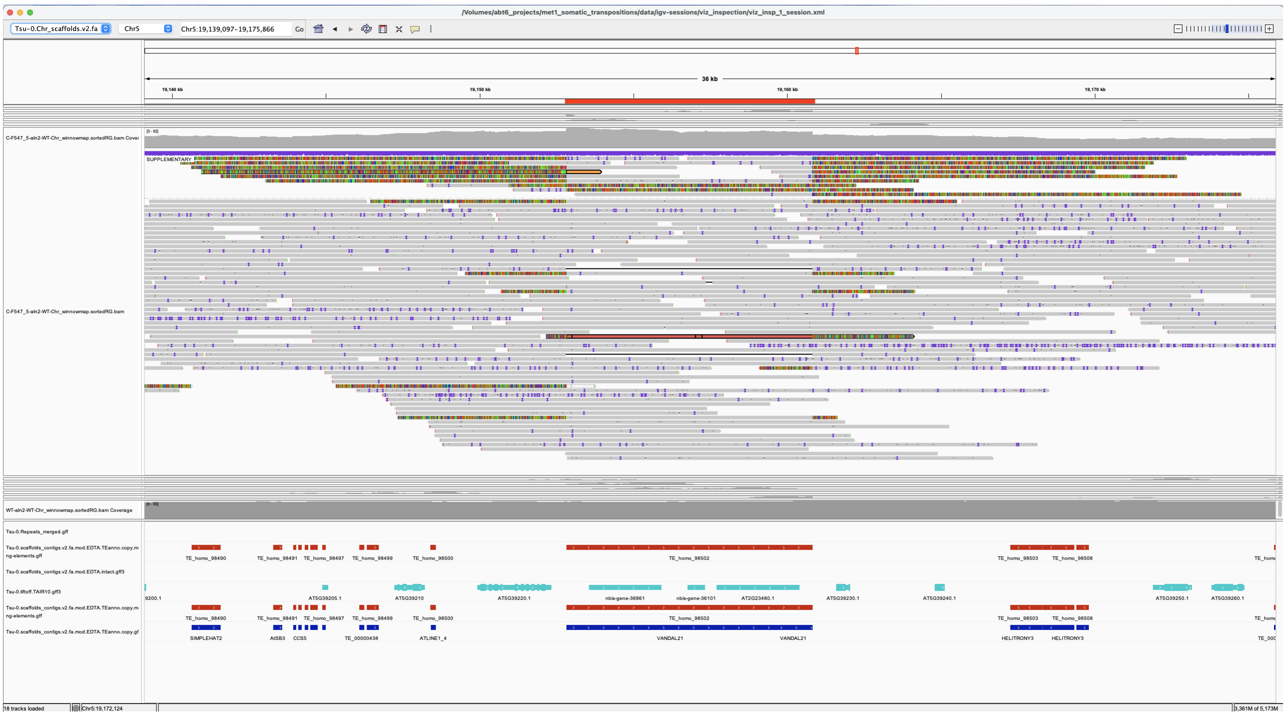

Partial

Confirmed

Chr1 8725235 8725235 + 1 Chr5;19152829;19160826;VANDAL21 m64079_240212_113350/126812806/ccs met1_05

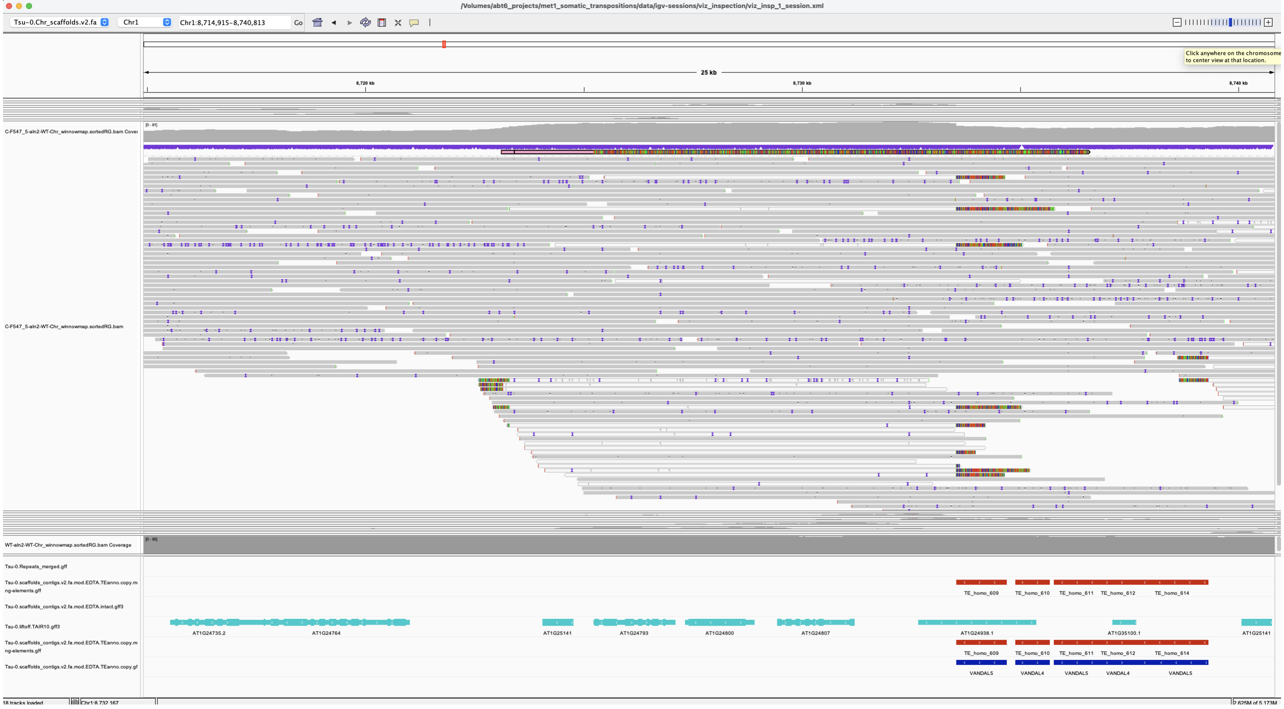

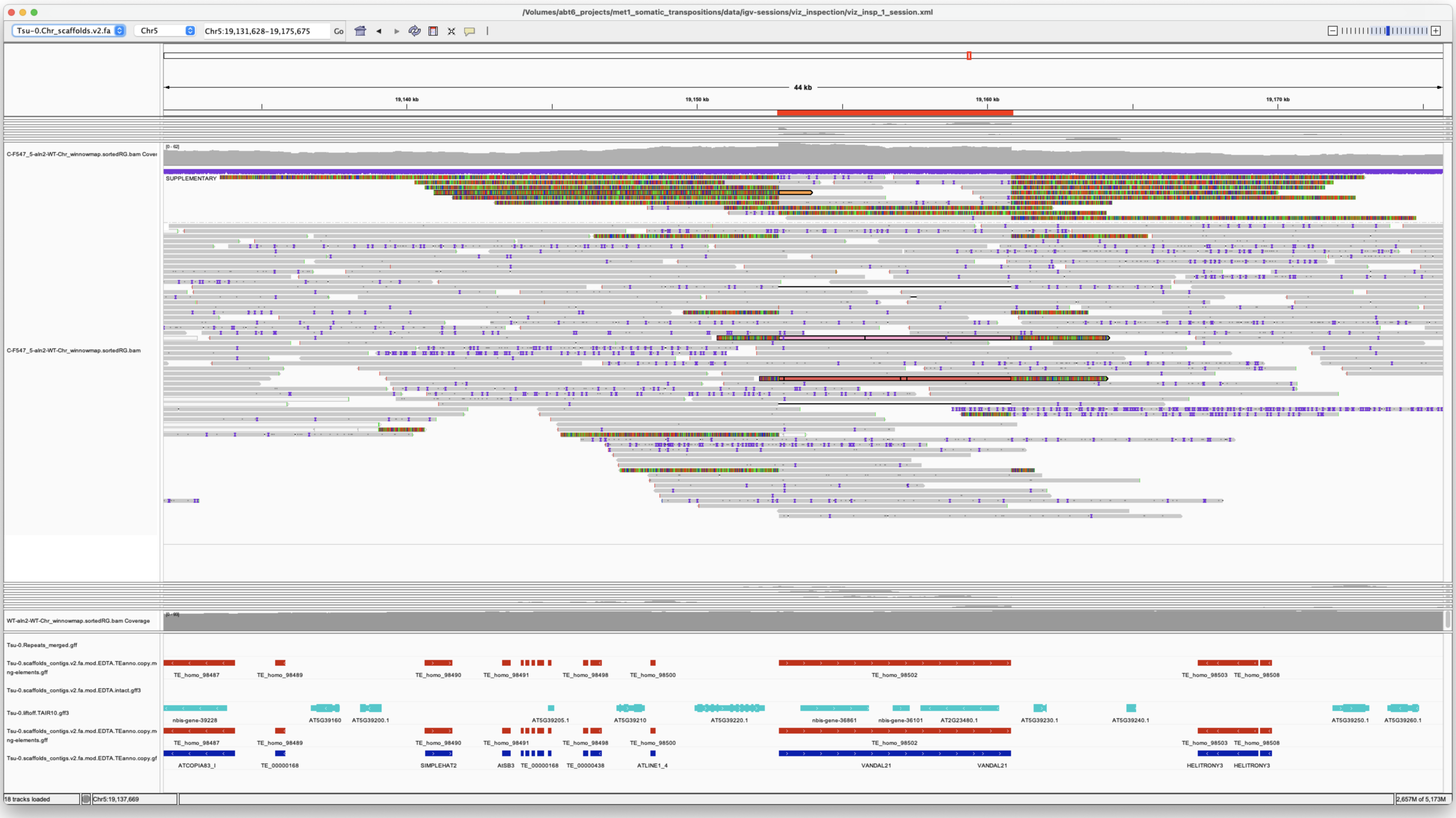

Partial

Confirmed

Chr1 19889718 19889718 + 1 Chr1;11941106;11946436;ATCOPIA93_Evade m64079_221220_112036/166660366/ccs met1_05

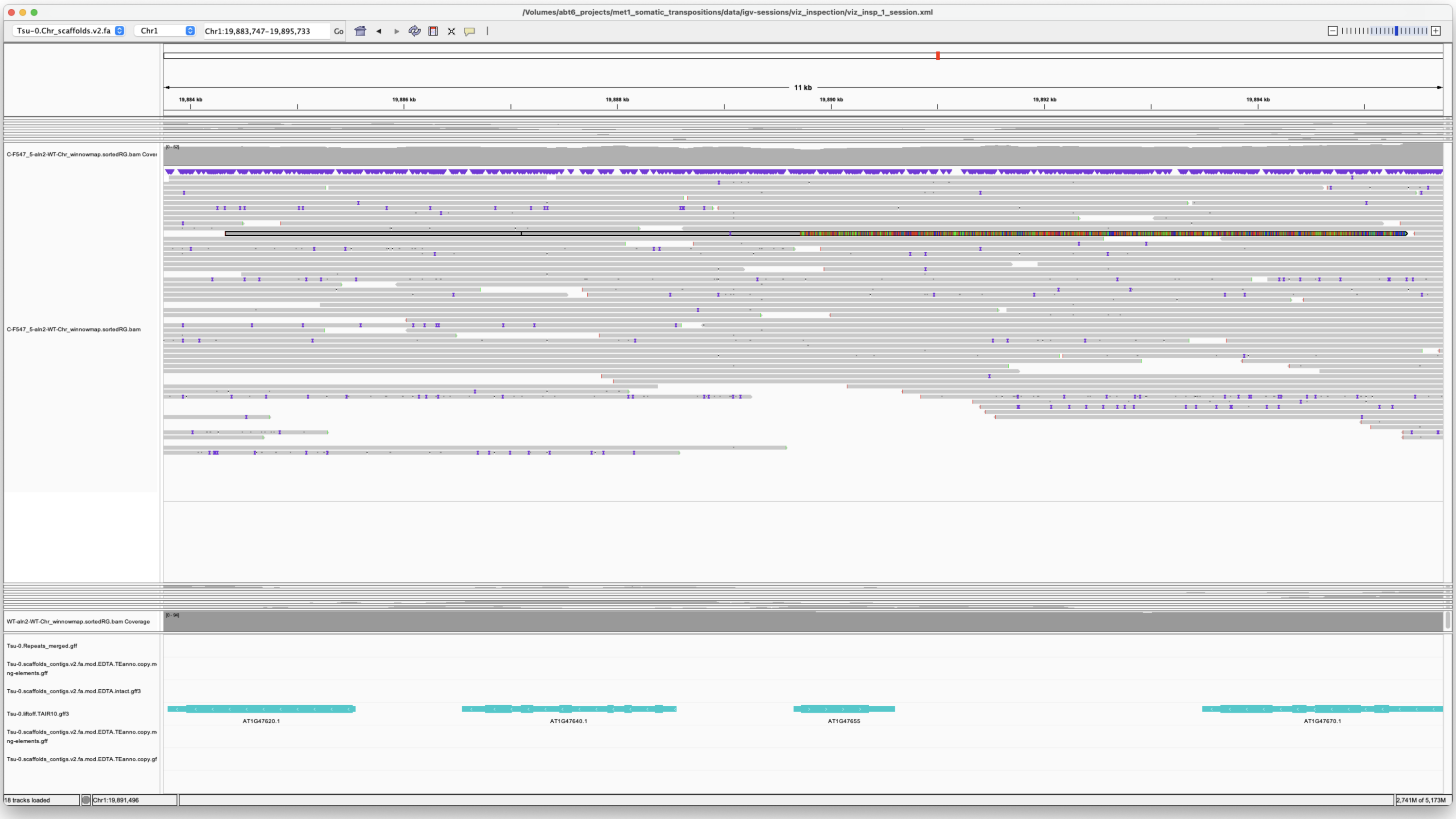

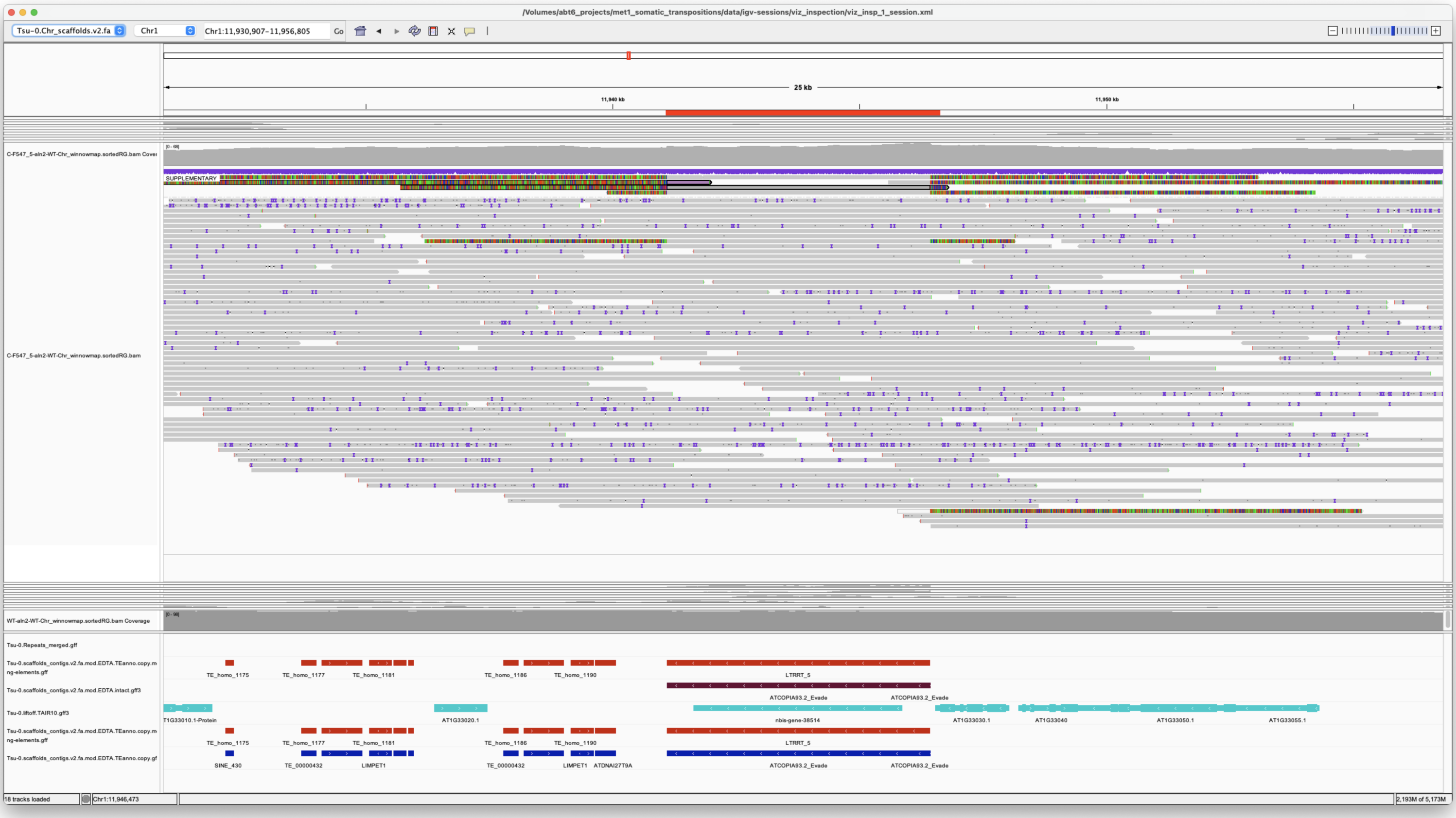

Partial

Confirmed

Chr1 23181200 23181200 + 1 Chr3;20158137;20166150;VANDAL6 m64079_240212_113350/171574467/ccs met1_05

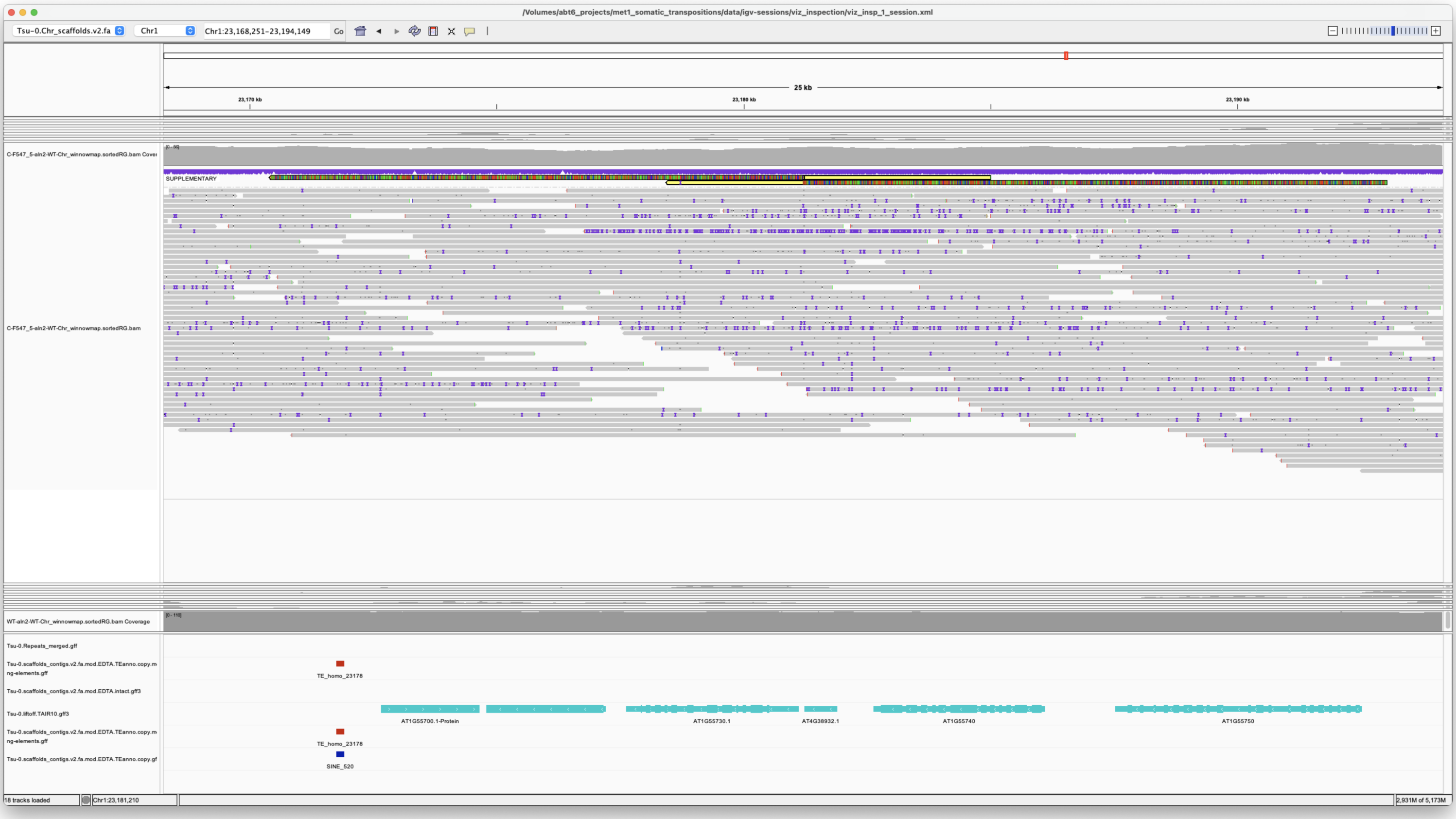

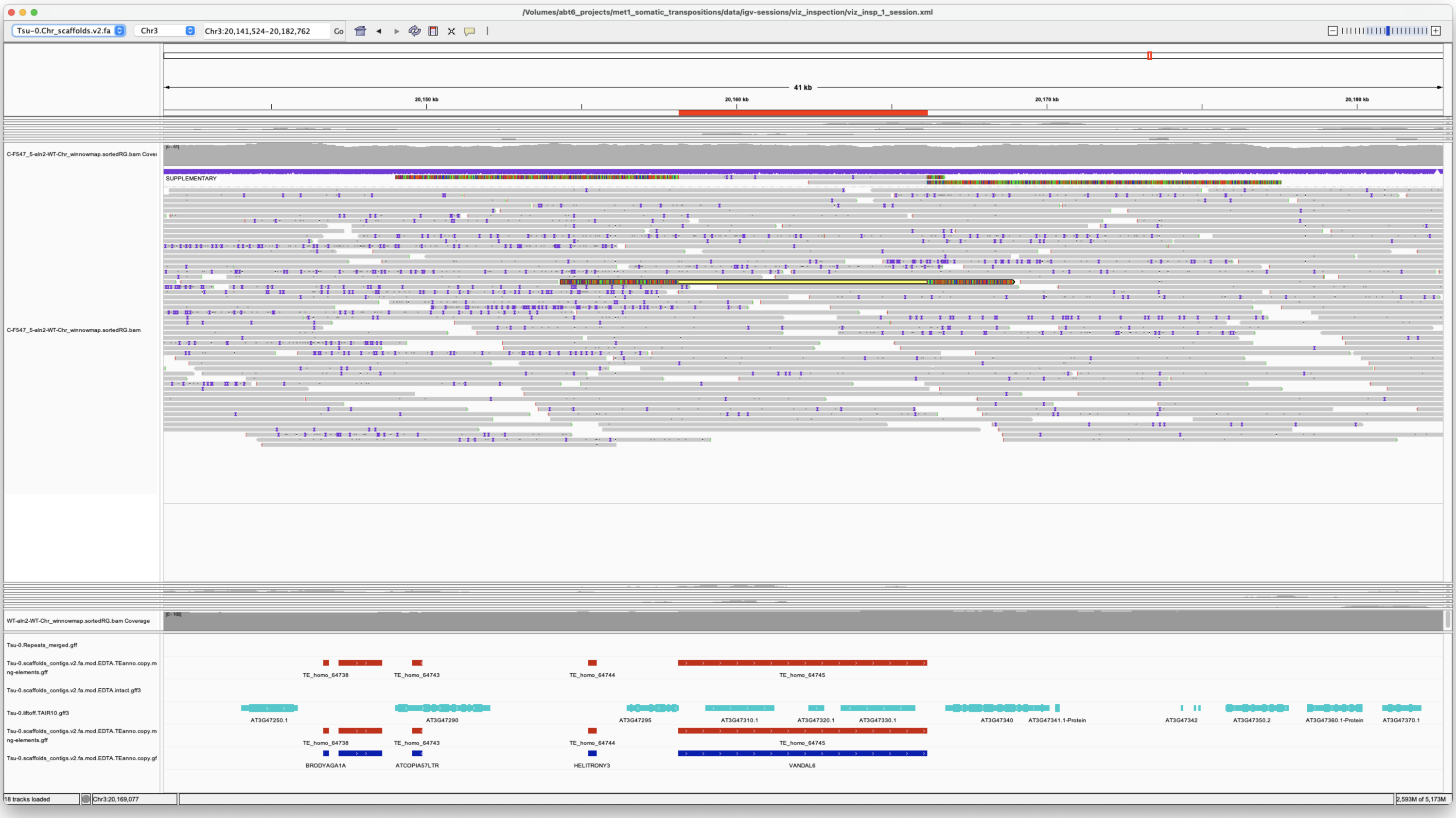

Central
TSD

Confirmed

Chr1 30316533 30316533 + 1 Chr3;16344522;16352497;VANDAL6 m64079_240212_113350/108331881/ccs met1_05

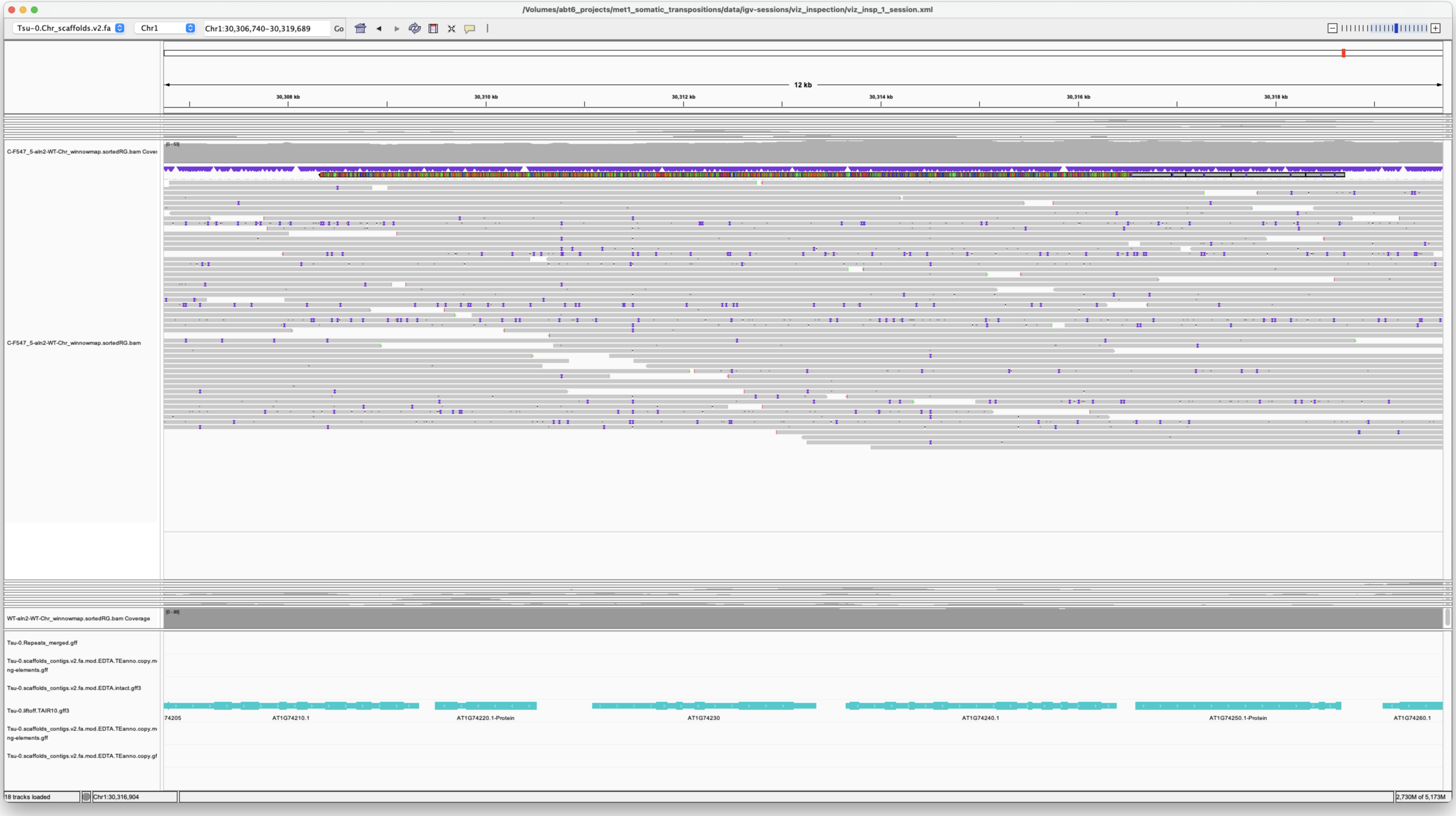

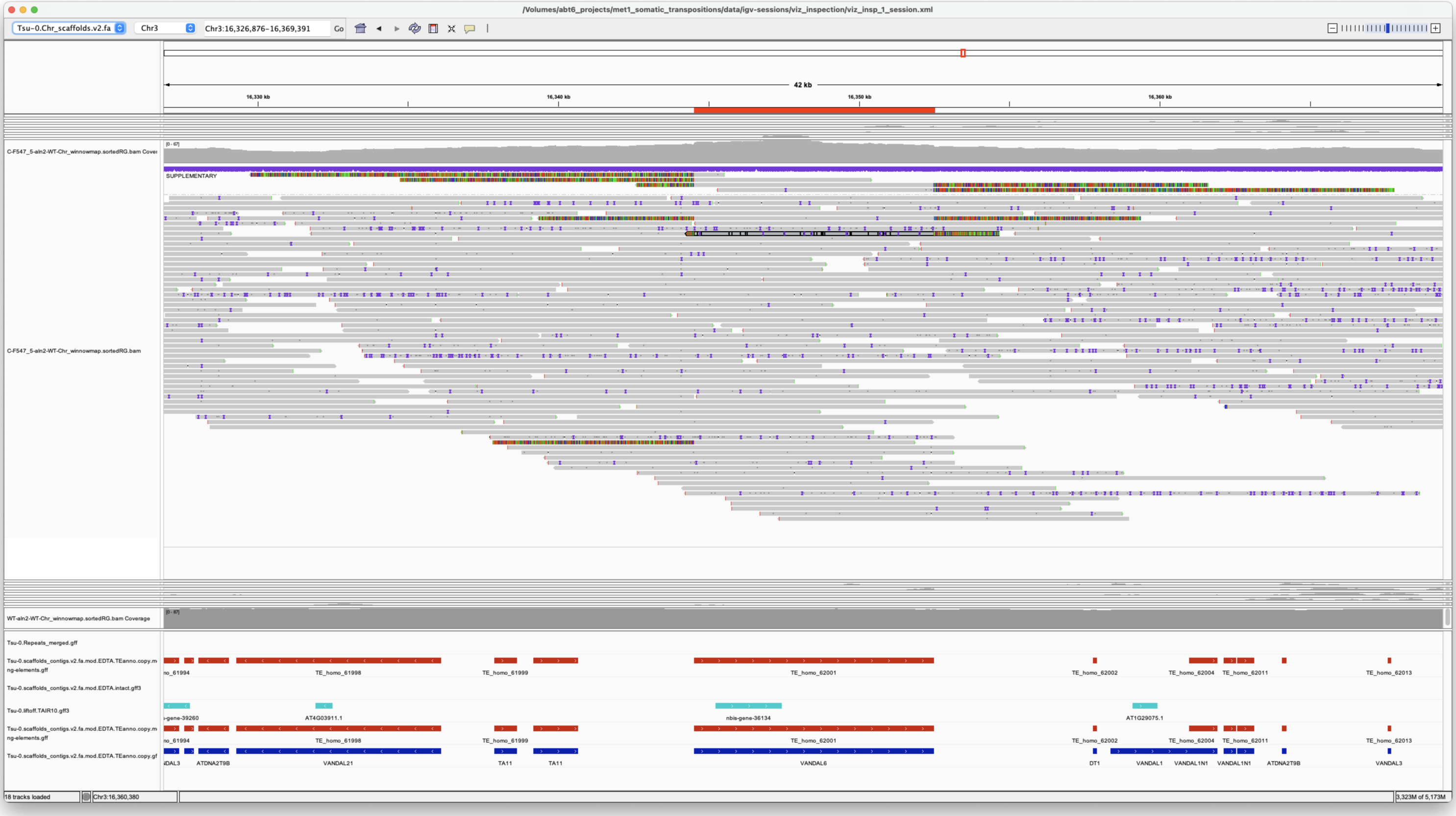

Partial

Confirmed

Chr1 32711196 32711196 + 1 Chr5;19152829;19160826;VANDAL21 m64079_240212_113350/5768130/ccs met1_05

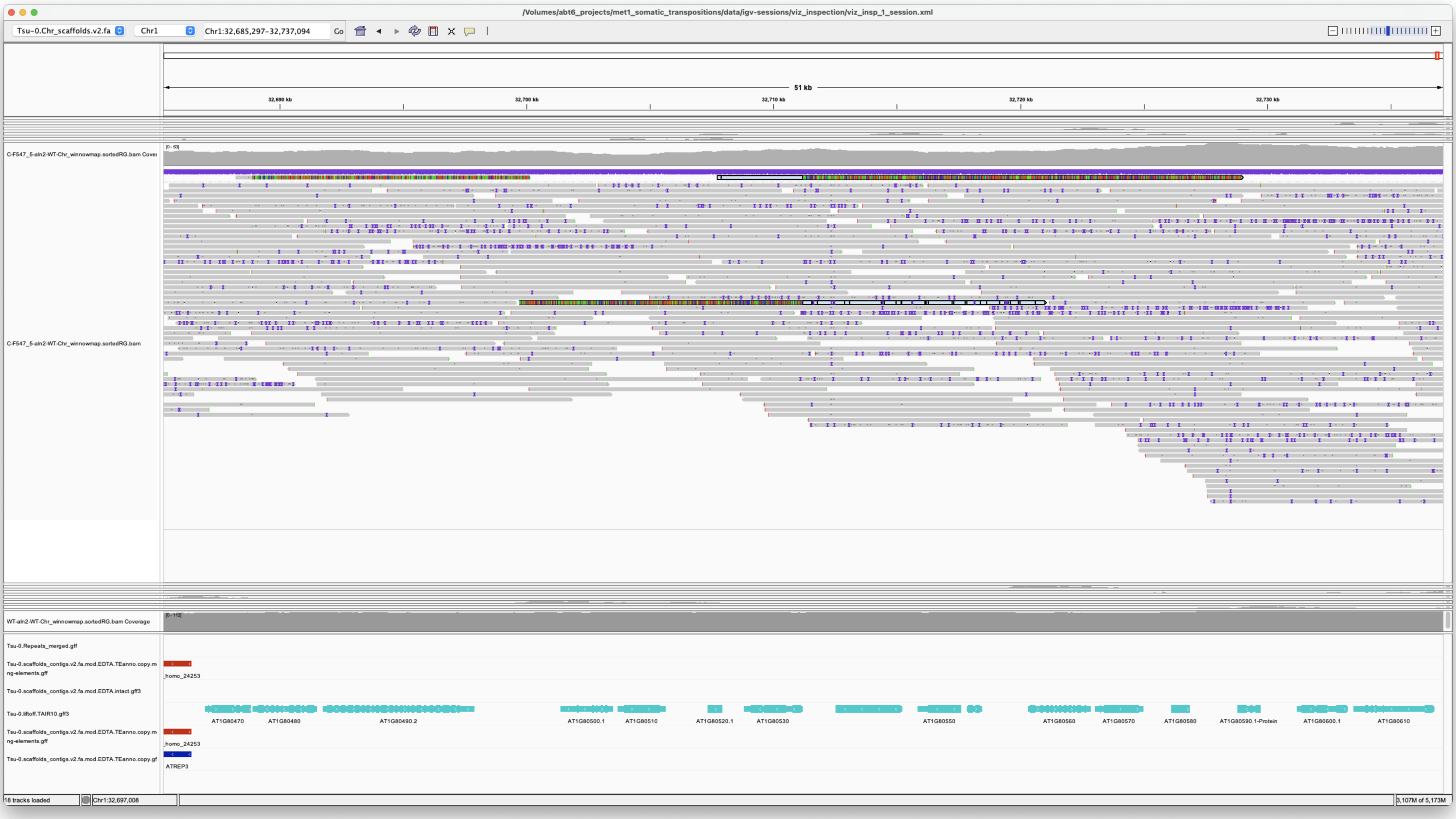

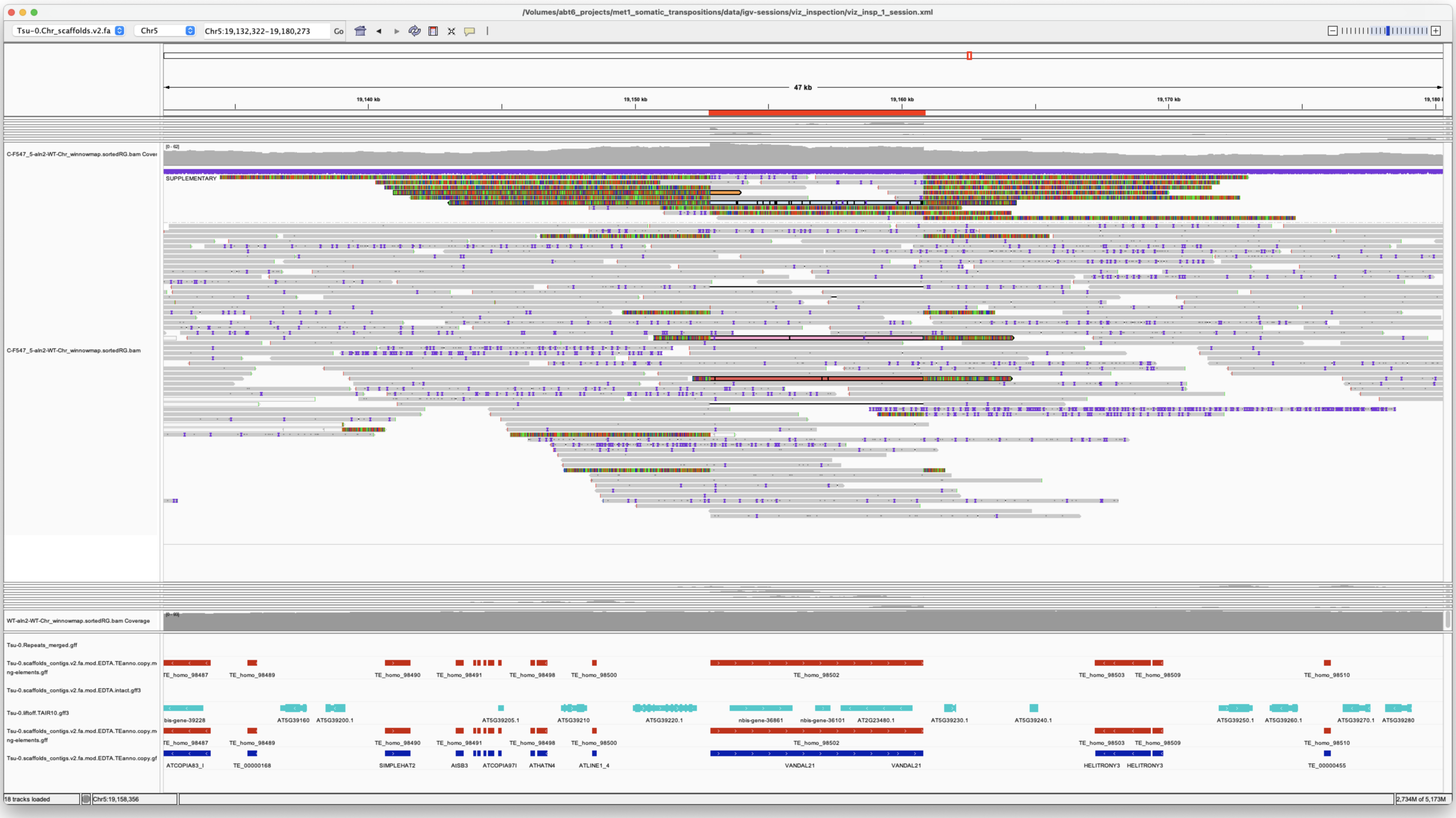

Central

TSD

Confirmed

Chr2 3092879 3092879 - 1 Chr5;19152829;19160826;VANDAL21 m64079_221220_112036/95683027/ccs met1_05

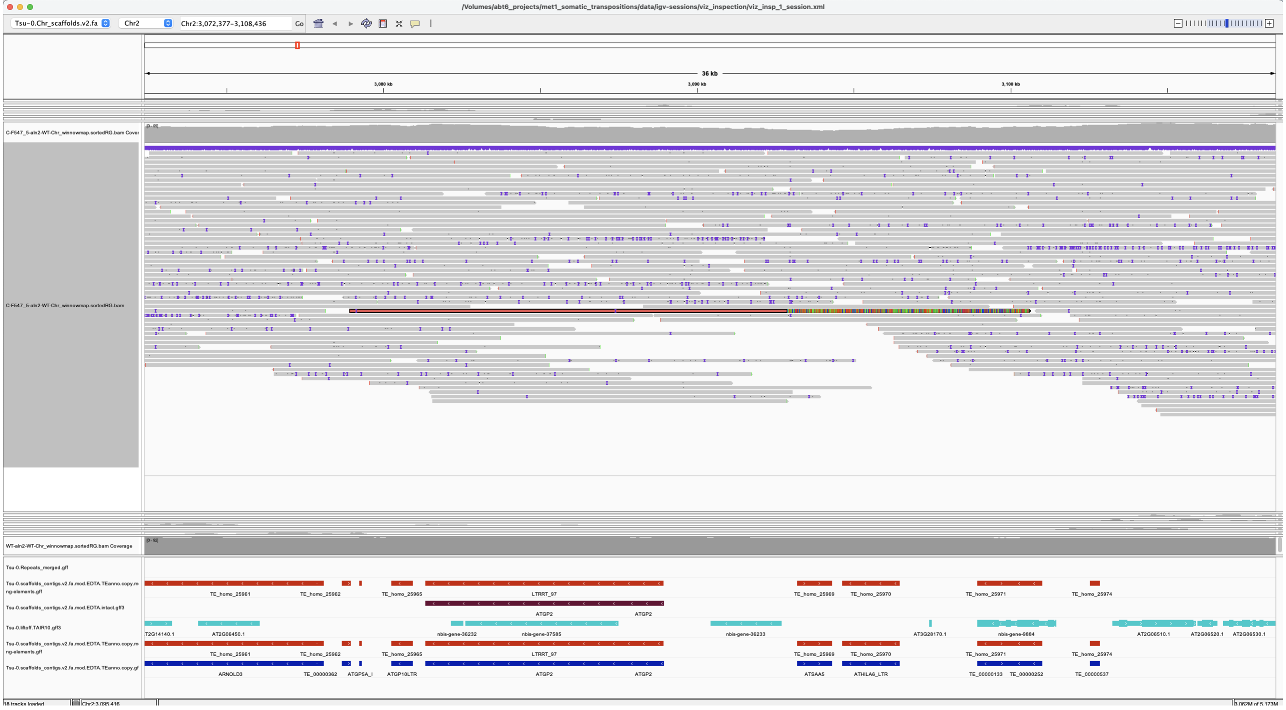

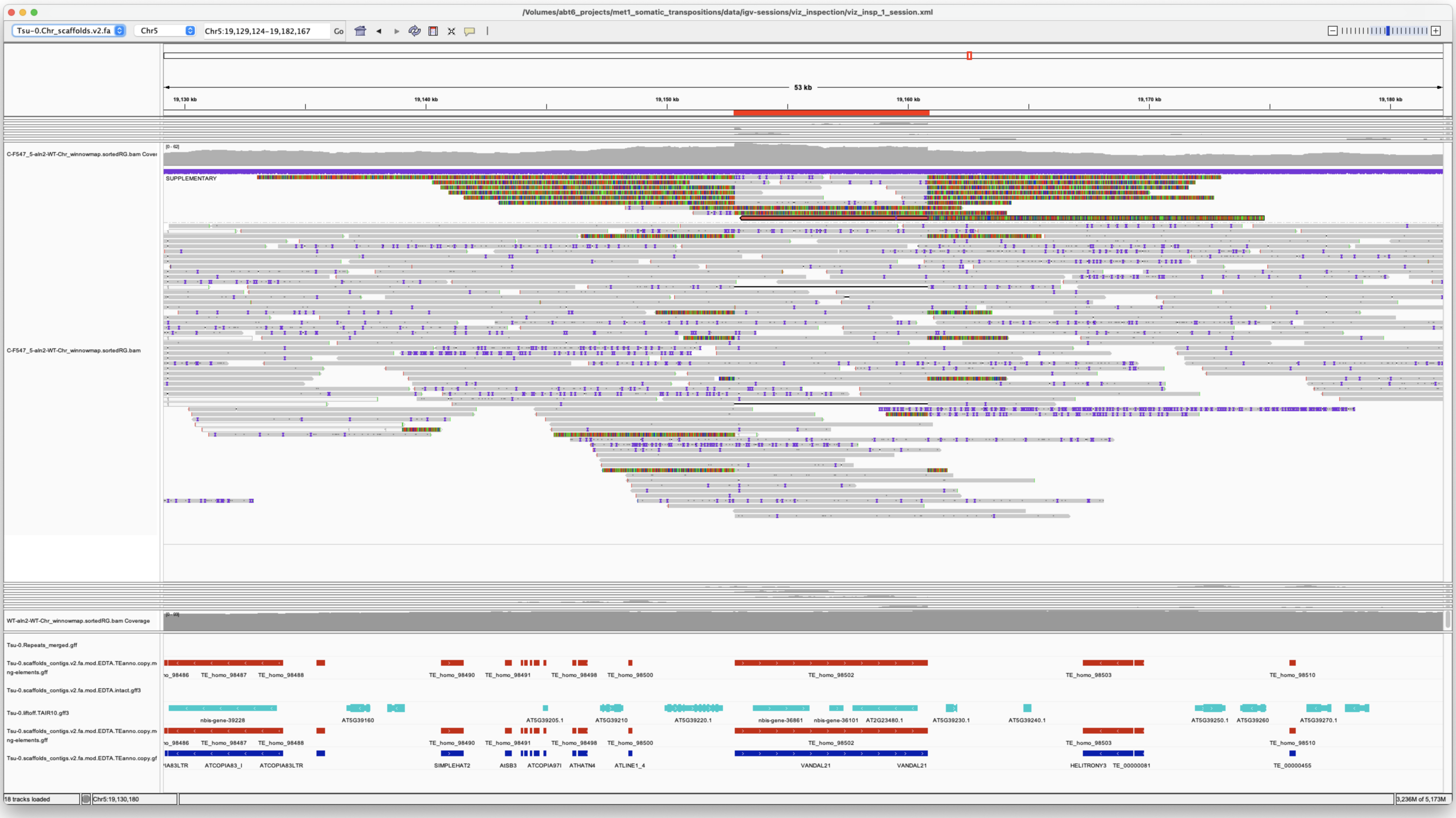

Partial

Confirmed

Chr2 7935840 7935840 + 1 Chr1;11941106;11946436;ATCOPIA93_Evade m64079_240212_113350/19138774/ccs met1_05
