# Supplementary material for: Long-read detection of transposable element mobilization in the soma of hypomethylated Arabidopsis thaliana individuals
Source: Genome Biol. 2025 Jul 30;26:231. doi: 10.1186/s13059-025-03691-7 (PMC12312487; doi:10.1186/s13059-025-03691-7)
Supplement: Supplementary file 2 — Additional file 2. Visual inspection of somatic insertion and excision events, available at https://github.com/aerilli/Somatic-transposition_met1/tree/551df407370c6528225f404ba62a073dced14b08/Supplementary-Files/Visual_inspection. [file 13059_2025_3691_MOESM2_ESM.gz › Split_Supplementary-File4/File1_SupplementaryALN_Insertions/File1_SupplementaryALN_Insertions-69-136.pdf]

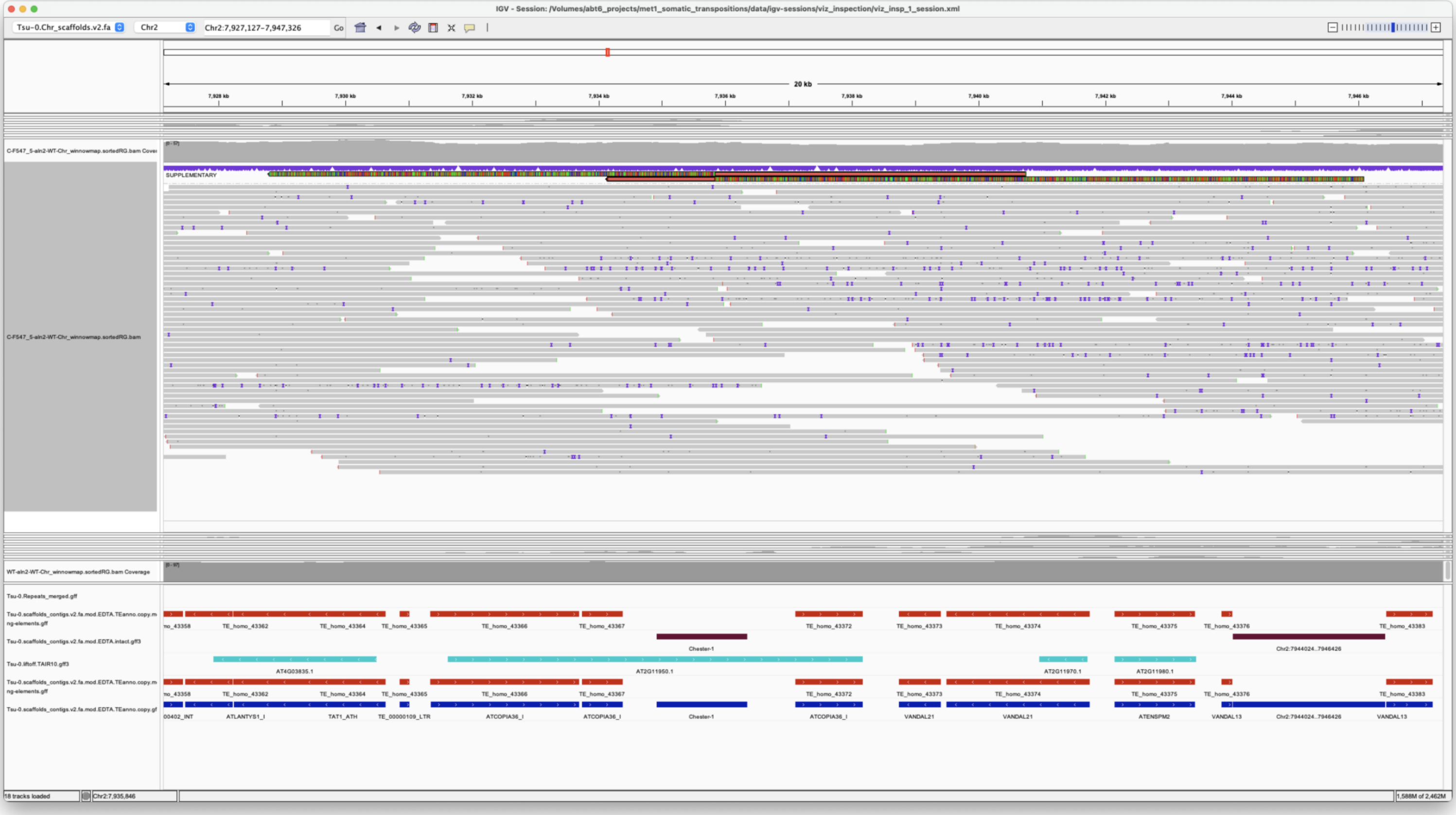

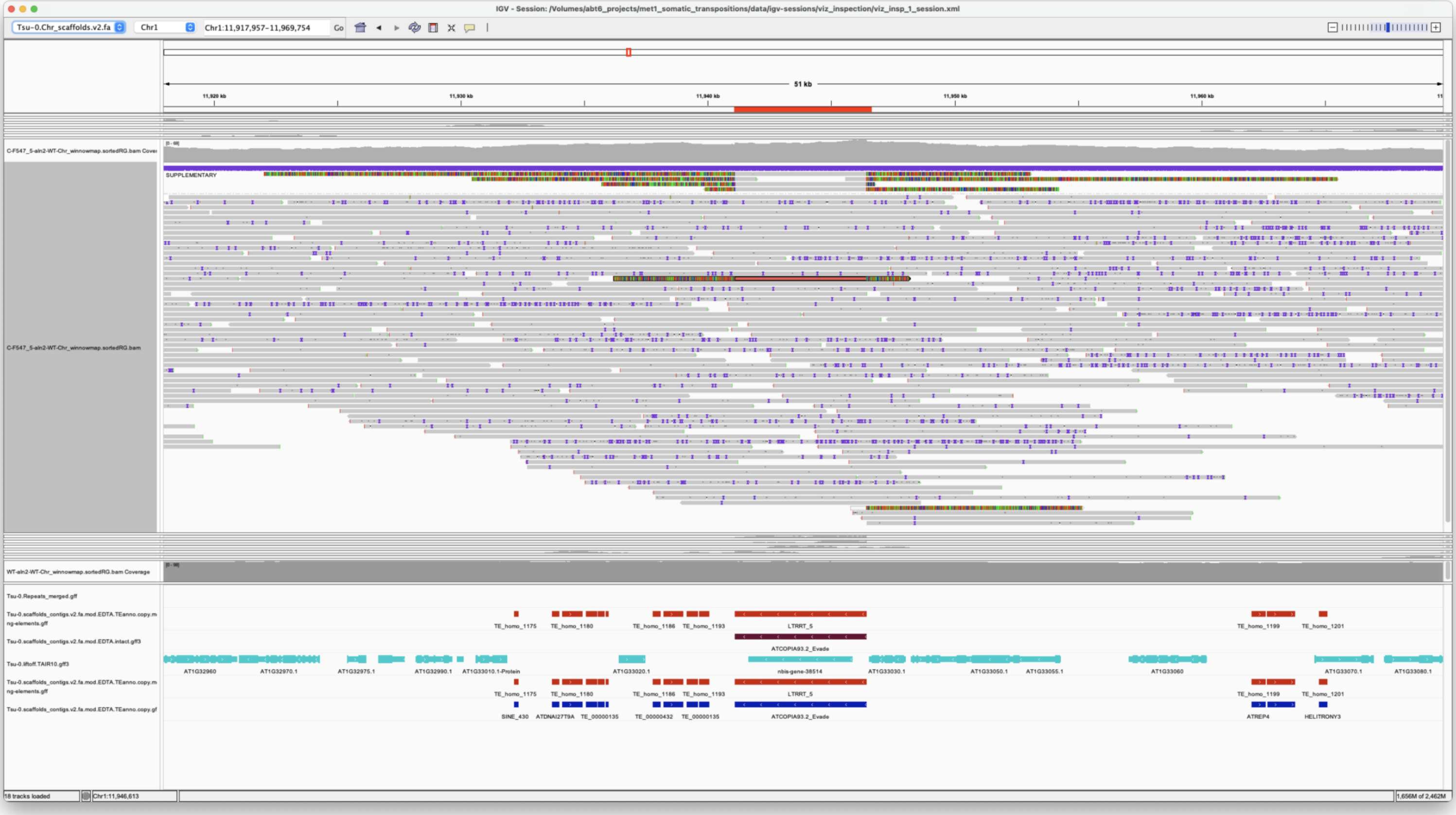

Central

TSD

Confirmed

Chr2 8113932 8113932 + 1 Chr5;19152829;19160826;VANDAL21 m64079_221220_112036/174786235/ccs met1_05

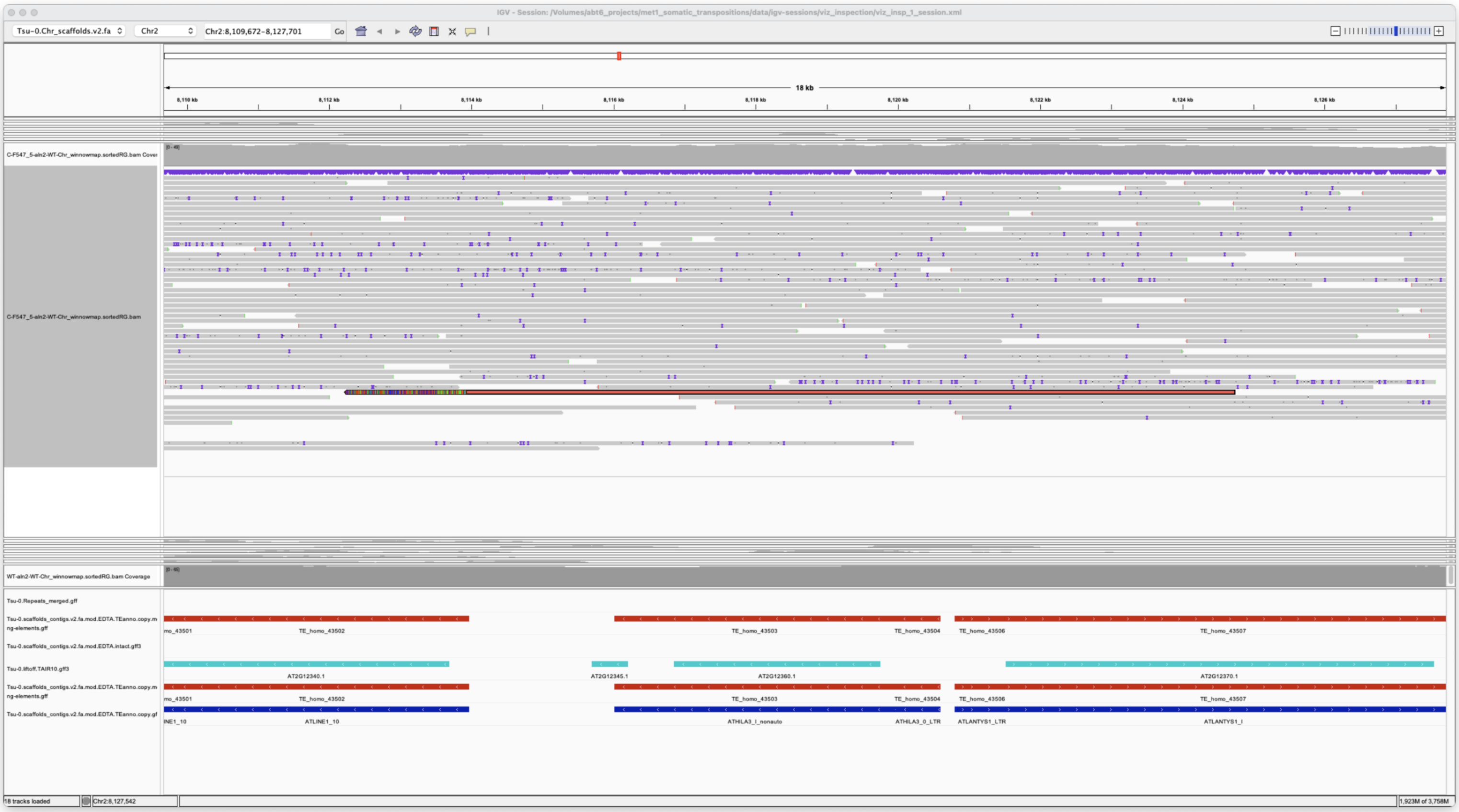

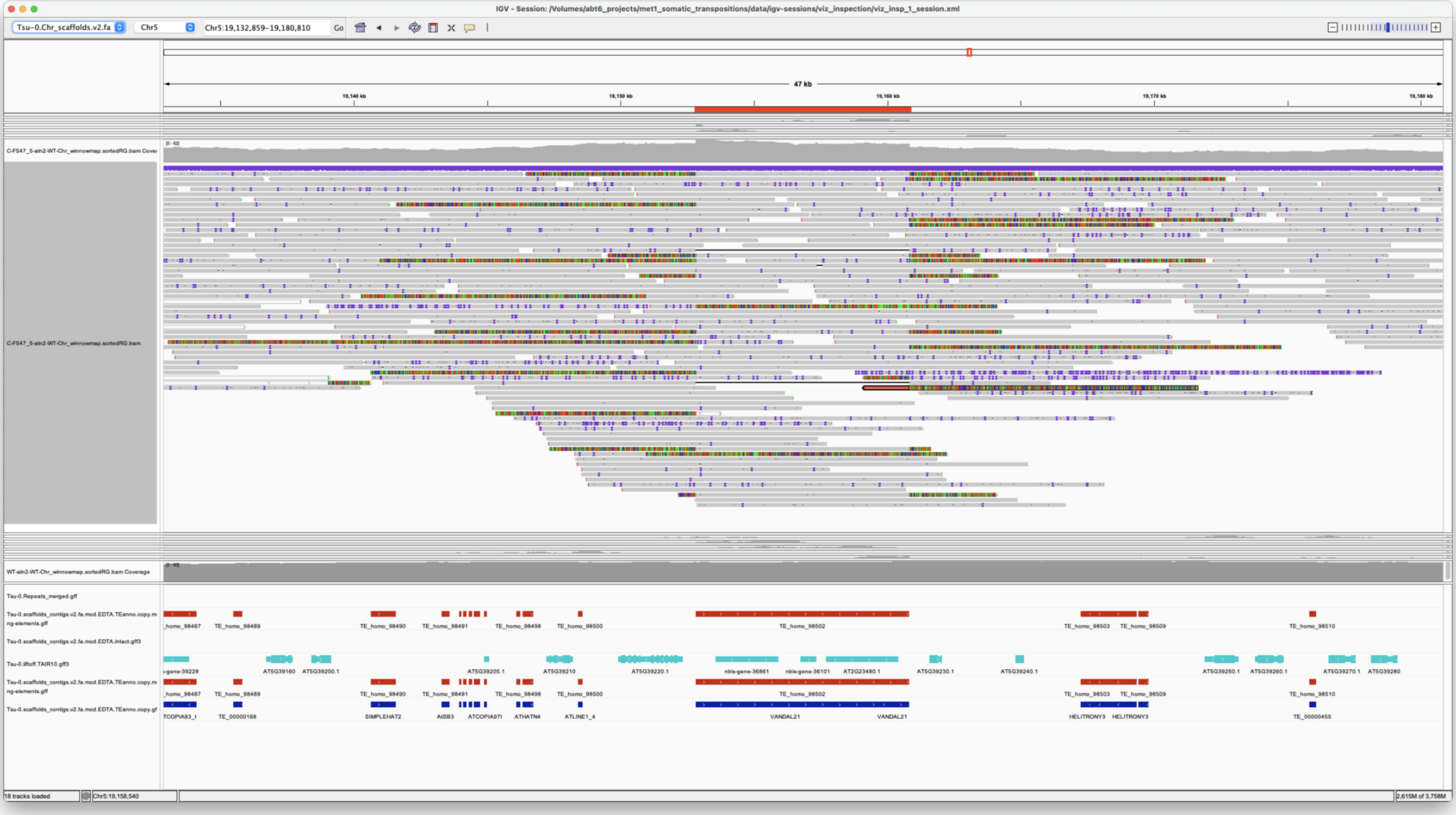

Partial

Confirmed

Chr2 9209529 9209529 - 1 Chr5;19152829;19160826;VANDAL21 m64079_221220_112036/43910439/ccs met1_05

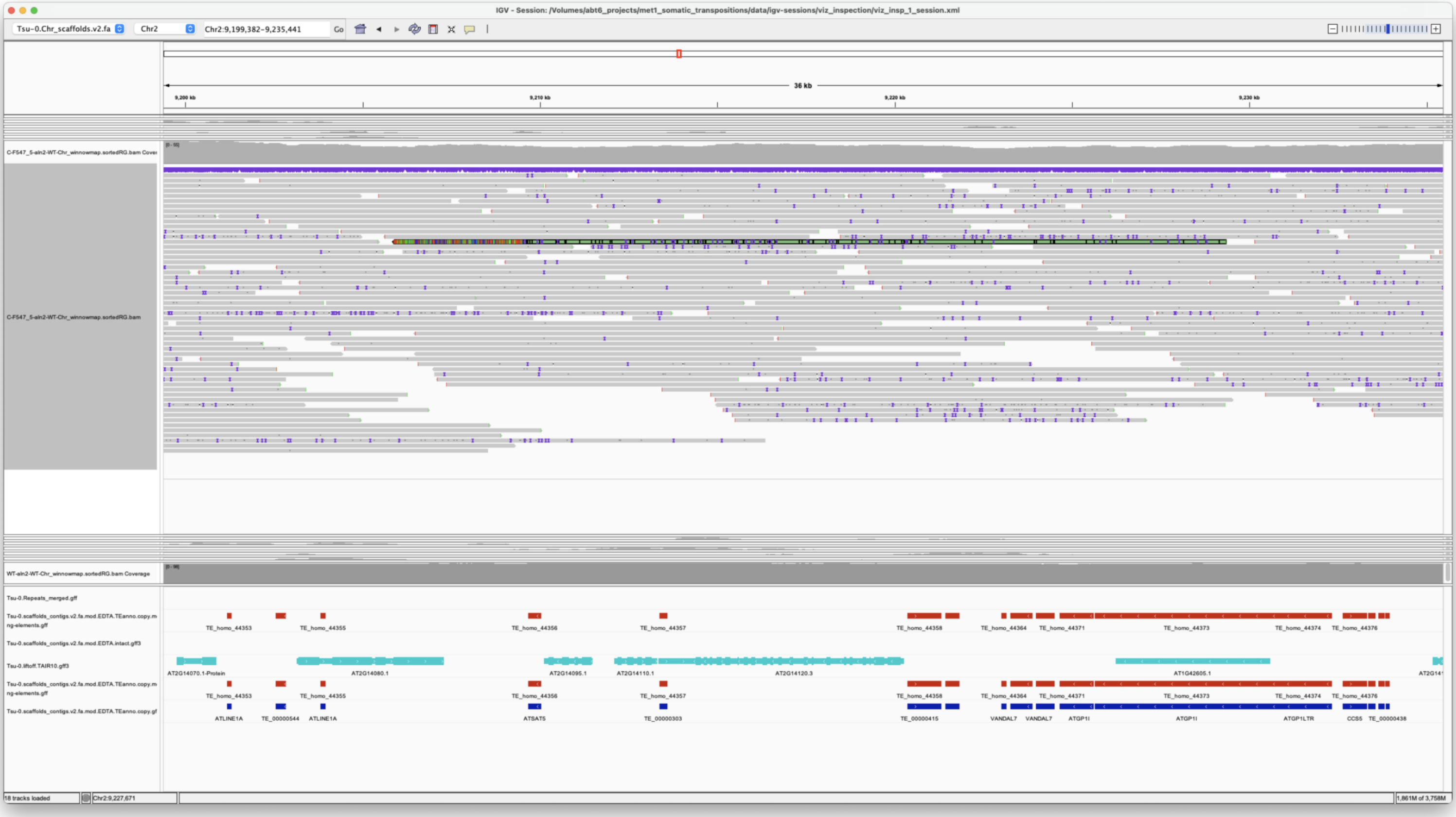

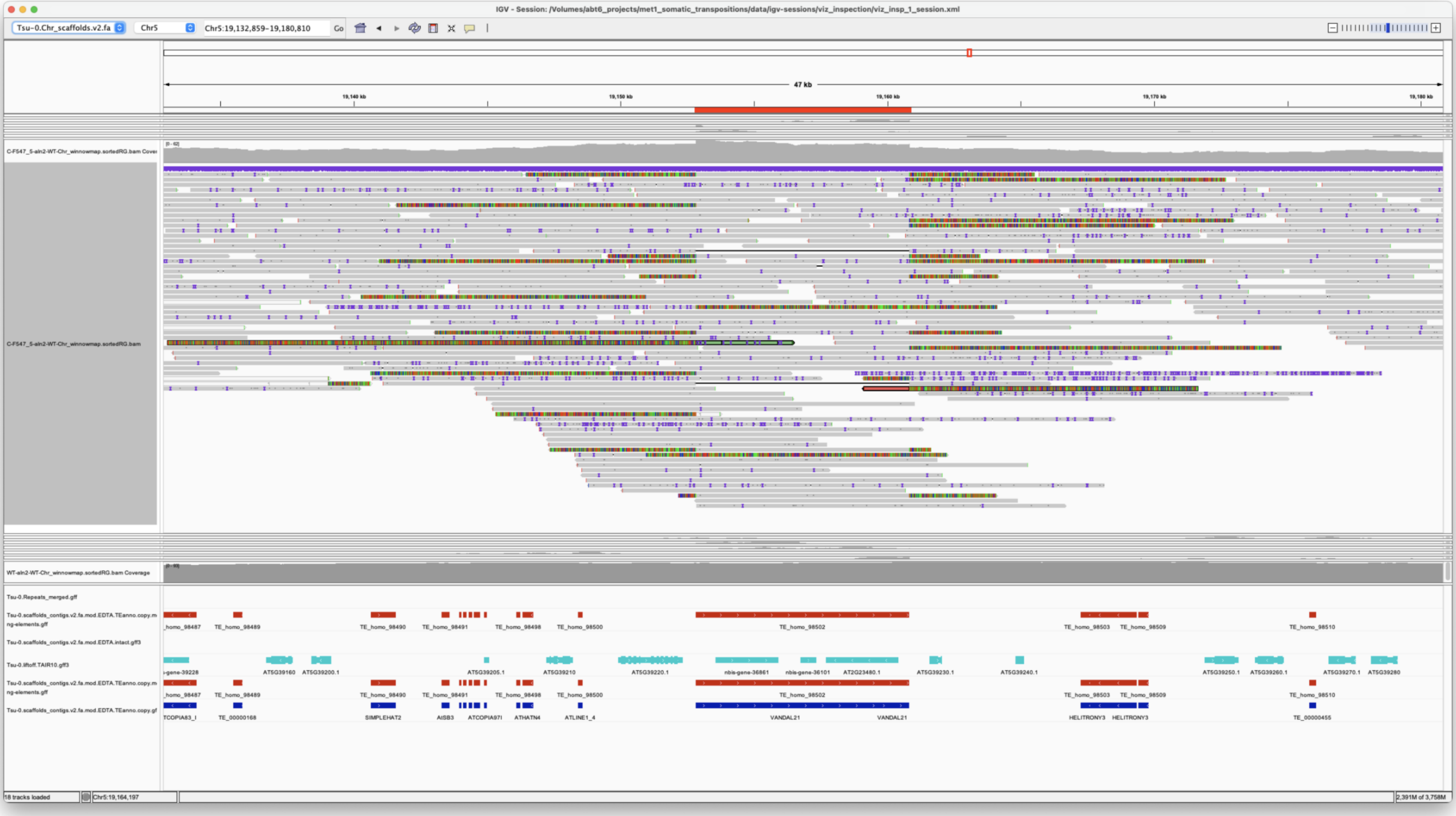

Partial

Confirmed

> Chr2 16316151 16316151 + 1 Chr1;11941106;11946436;ATCOPIA93_Evade m64079_221220_112036/155387511/ccs met1_05

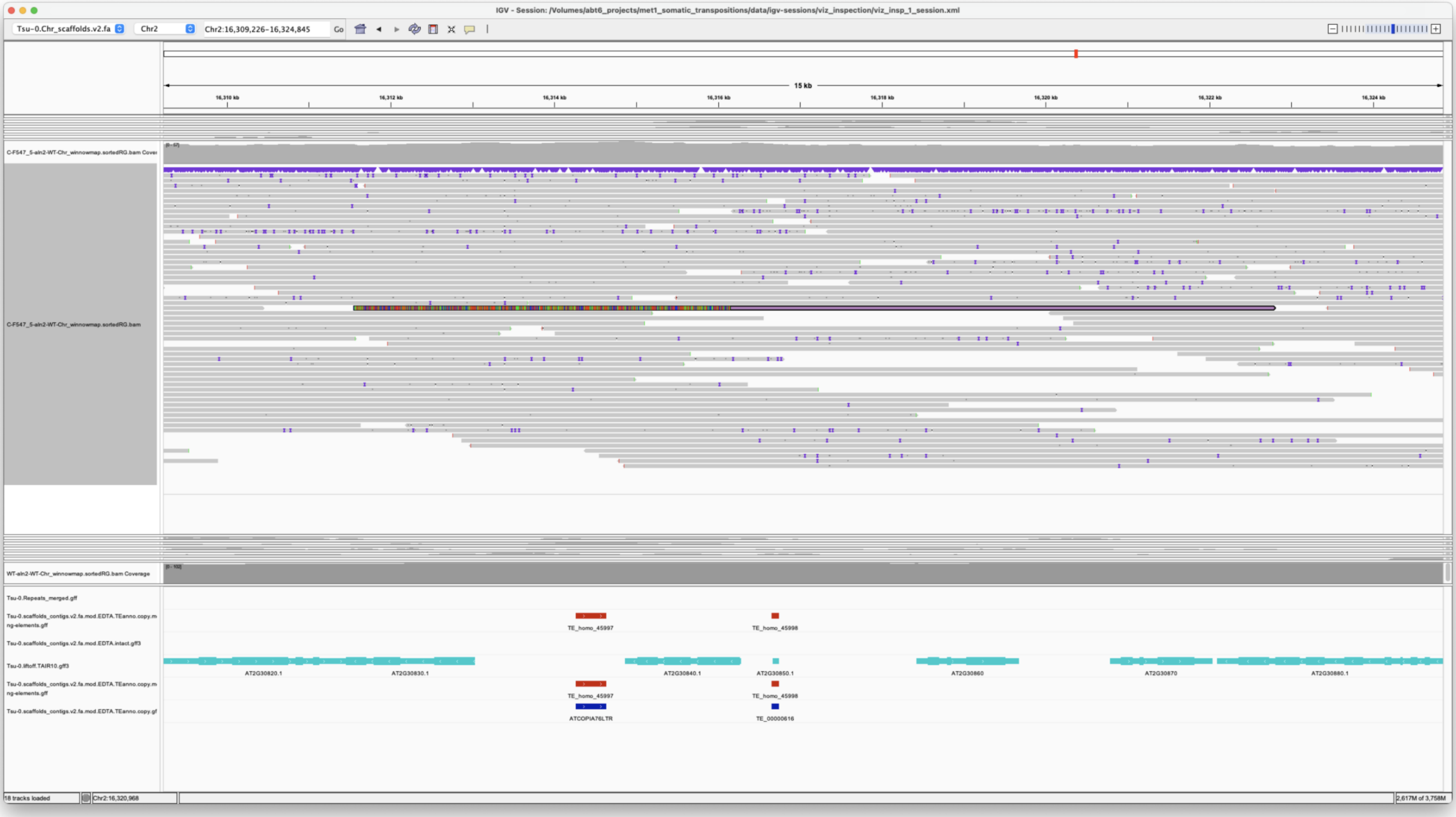

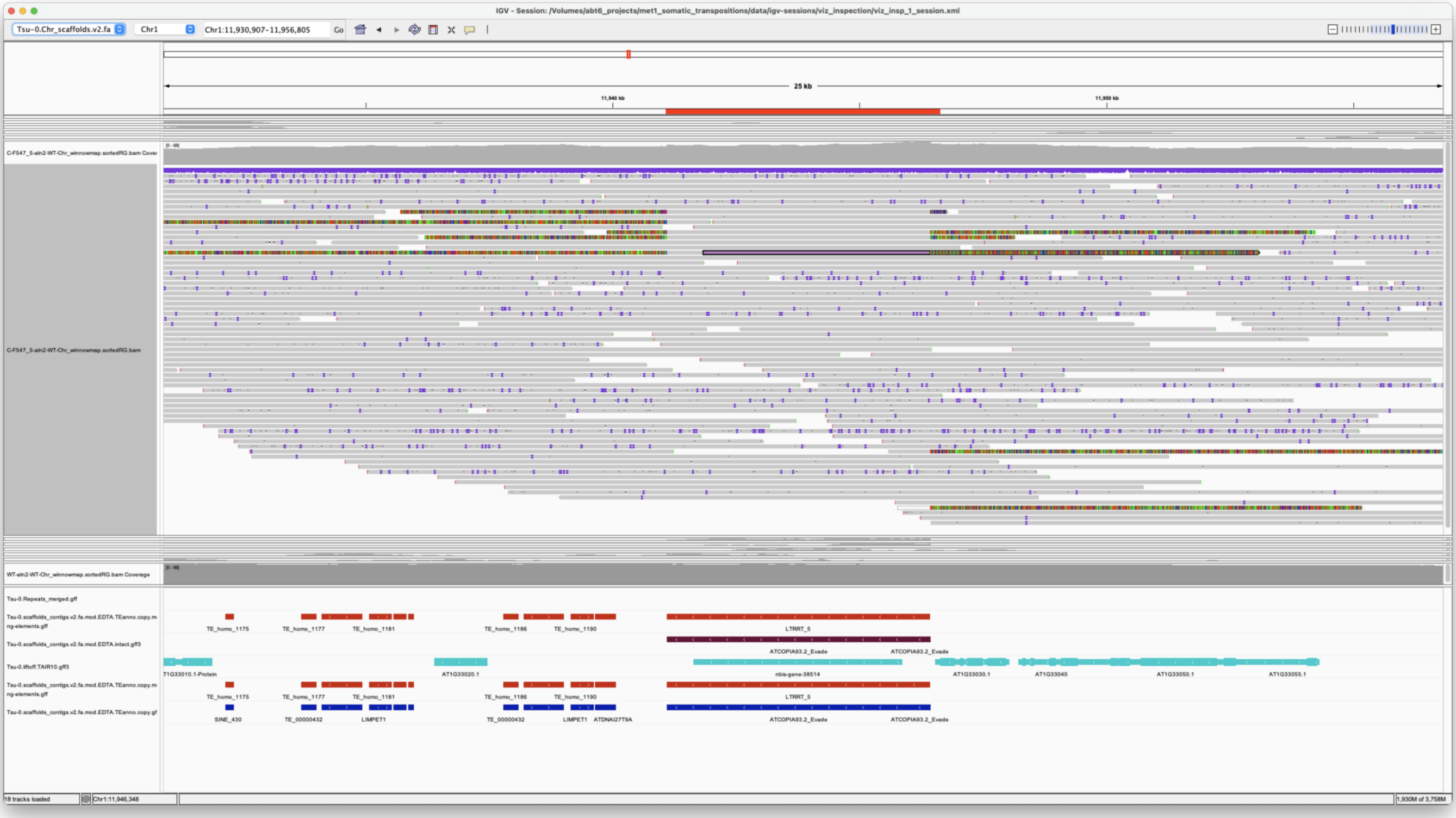

Partial

Confirmed

Chr2 20780007 20780007 - 1 Chr3;16344522;16352497;VANDAL6 m64079_240212_113350/27460723/ccs met1_05

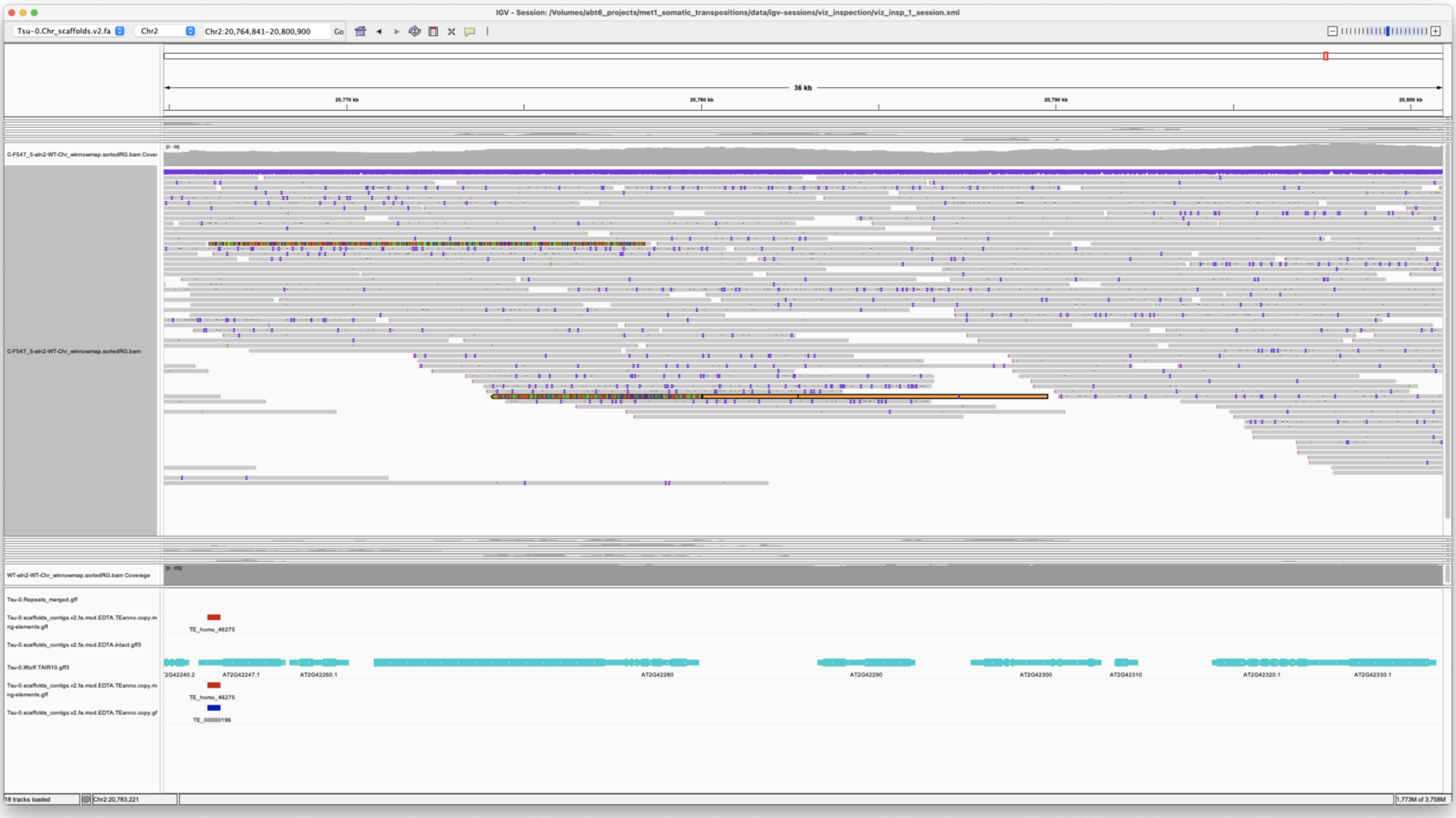

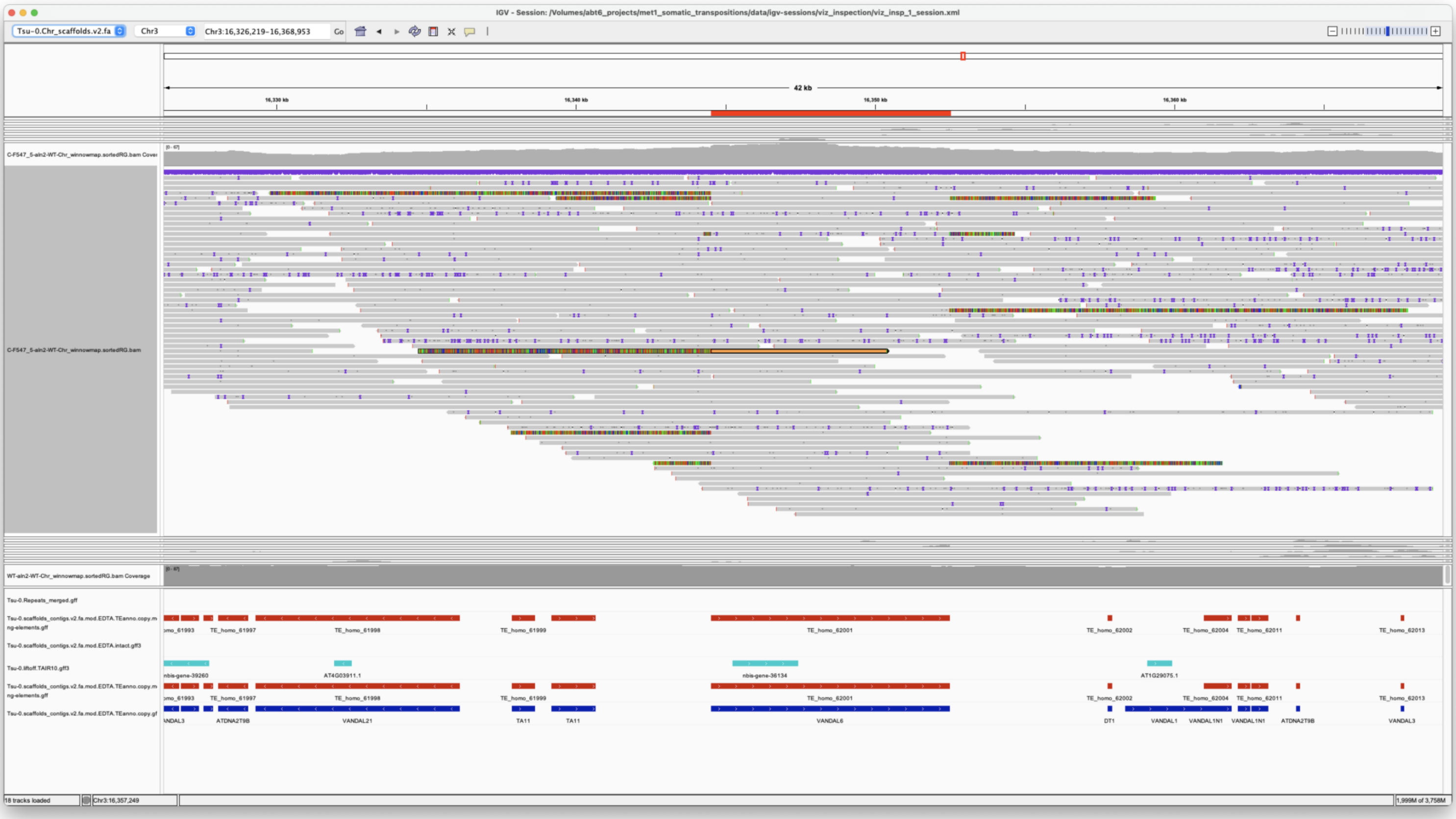

Partial

Confirmed

Chr3 2424704 2424704 + 1 Chr3;16344522;16352497;VANDAL6 m64079_240212_113350/76350204/ccs met1_05

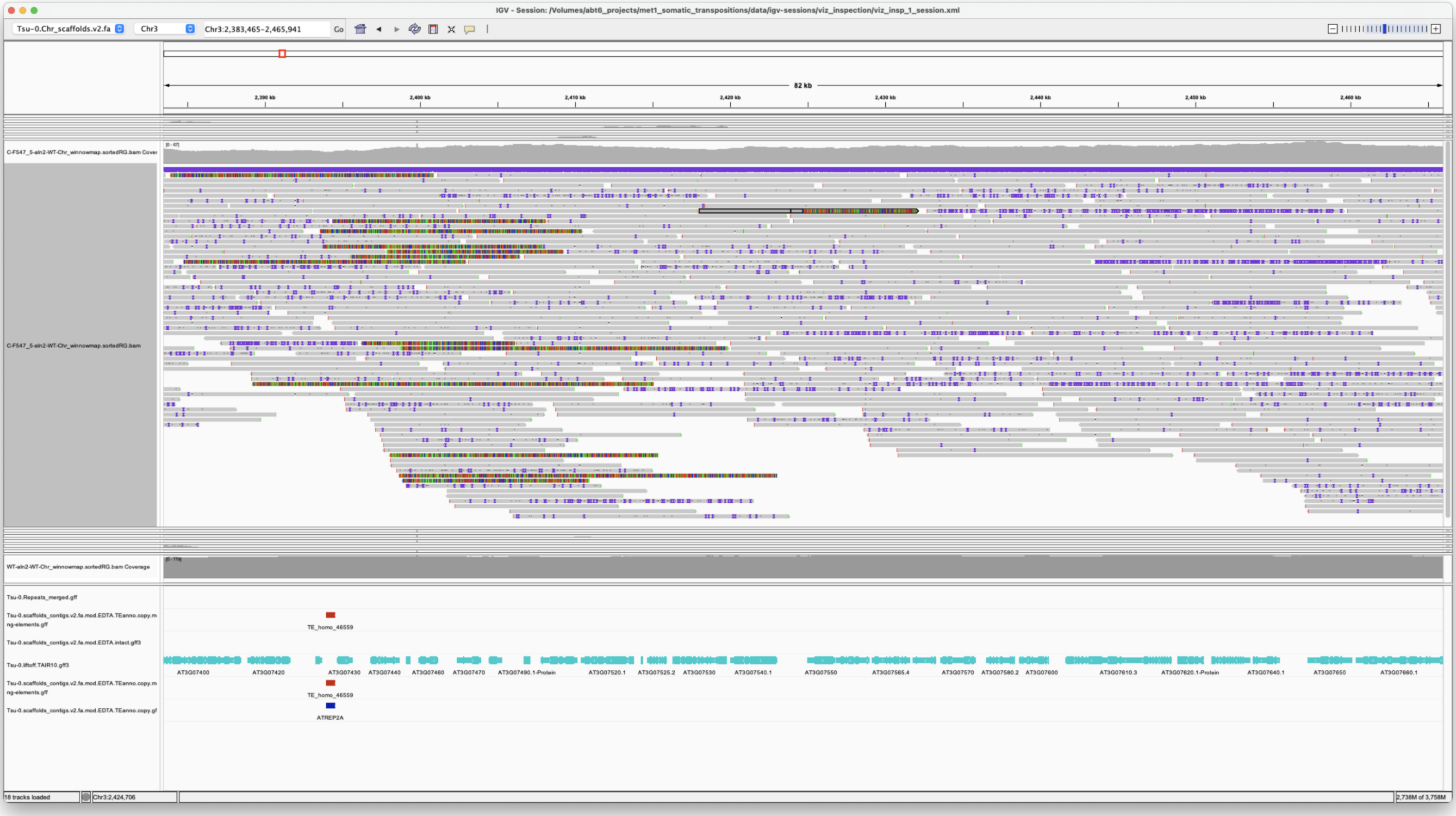

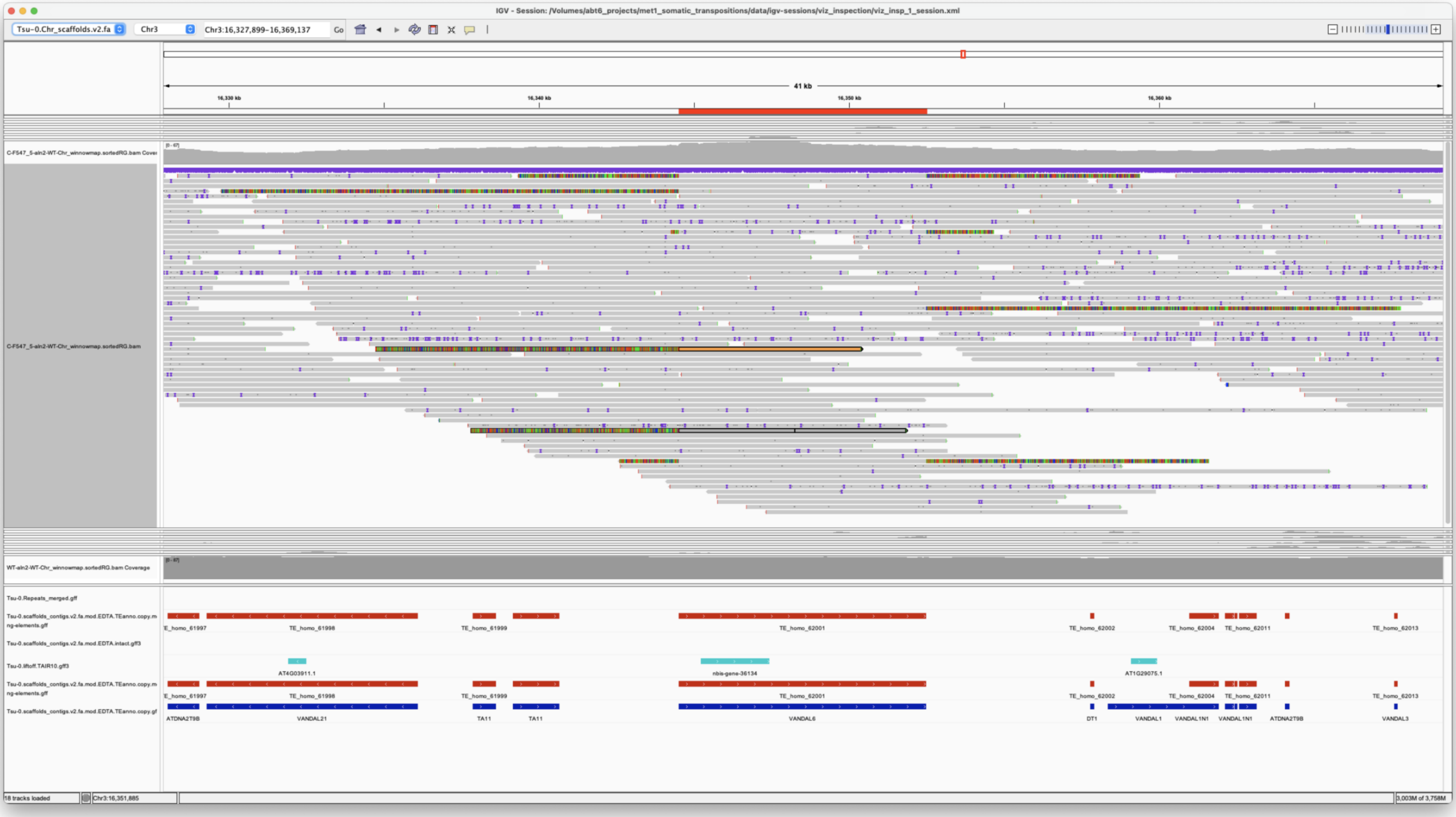

Partial

Confirmed

Chr3 2484266 2484266 - 1 Chr1;11941106;11946436;ATCOPIA93_Evade m64079_221220_112036/55051845/ccs met1_05

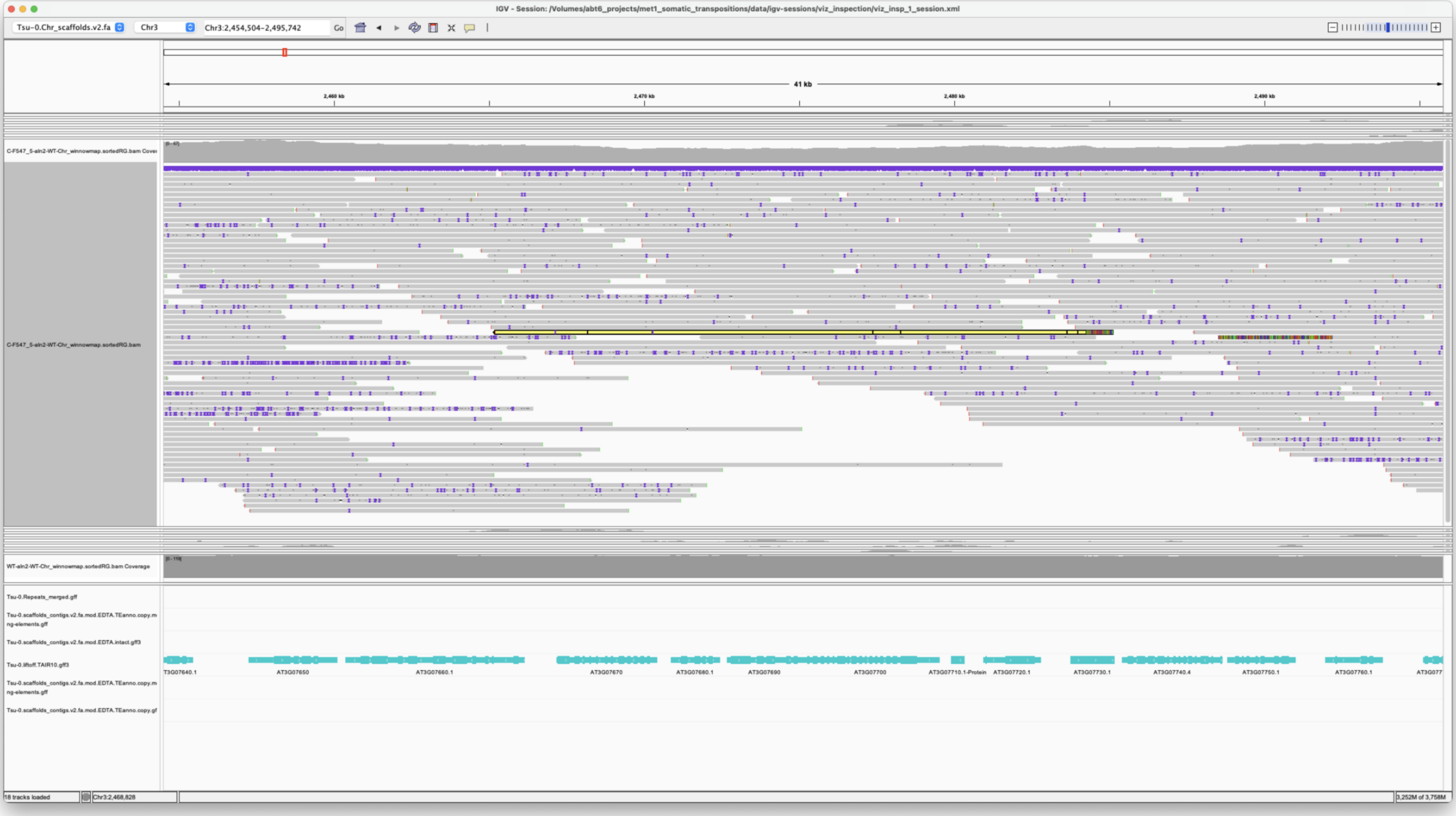

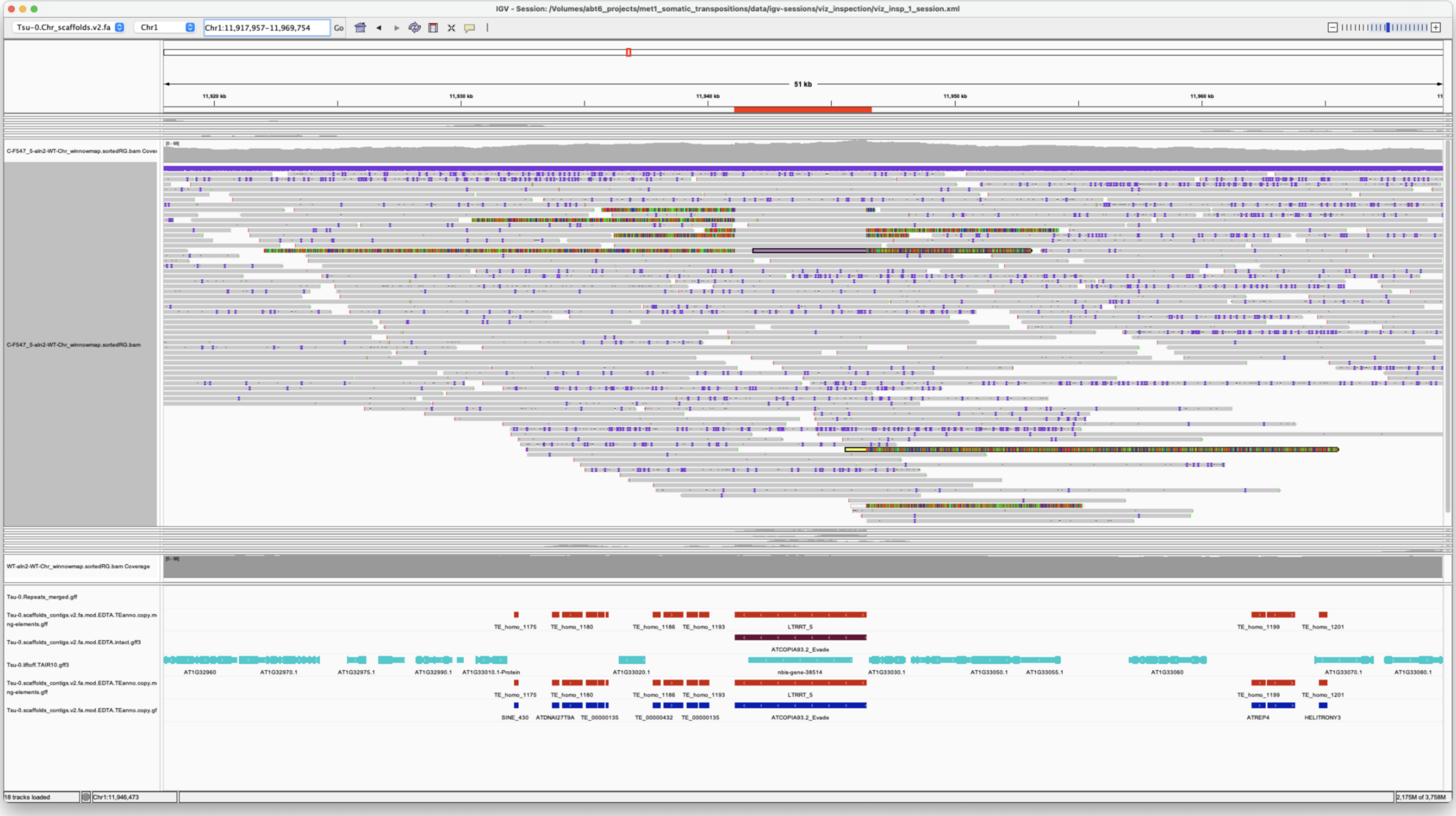

Partial

Confirmed

Chr3 2492197 2492197 - 1 Chr5;19152829;19160826;VANDAL21 m64079_221220_112036/115671051/ccs met1_05

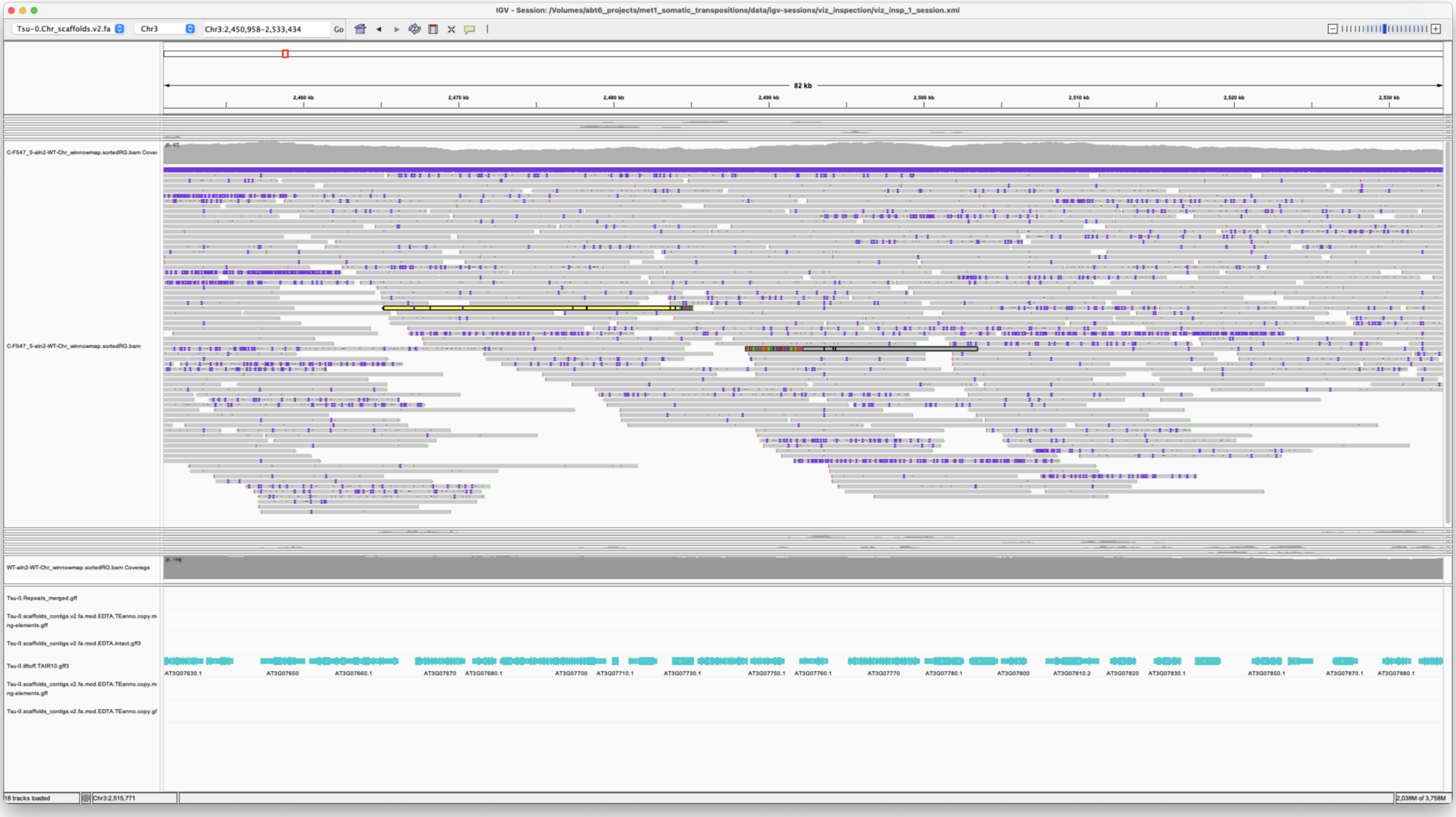

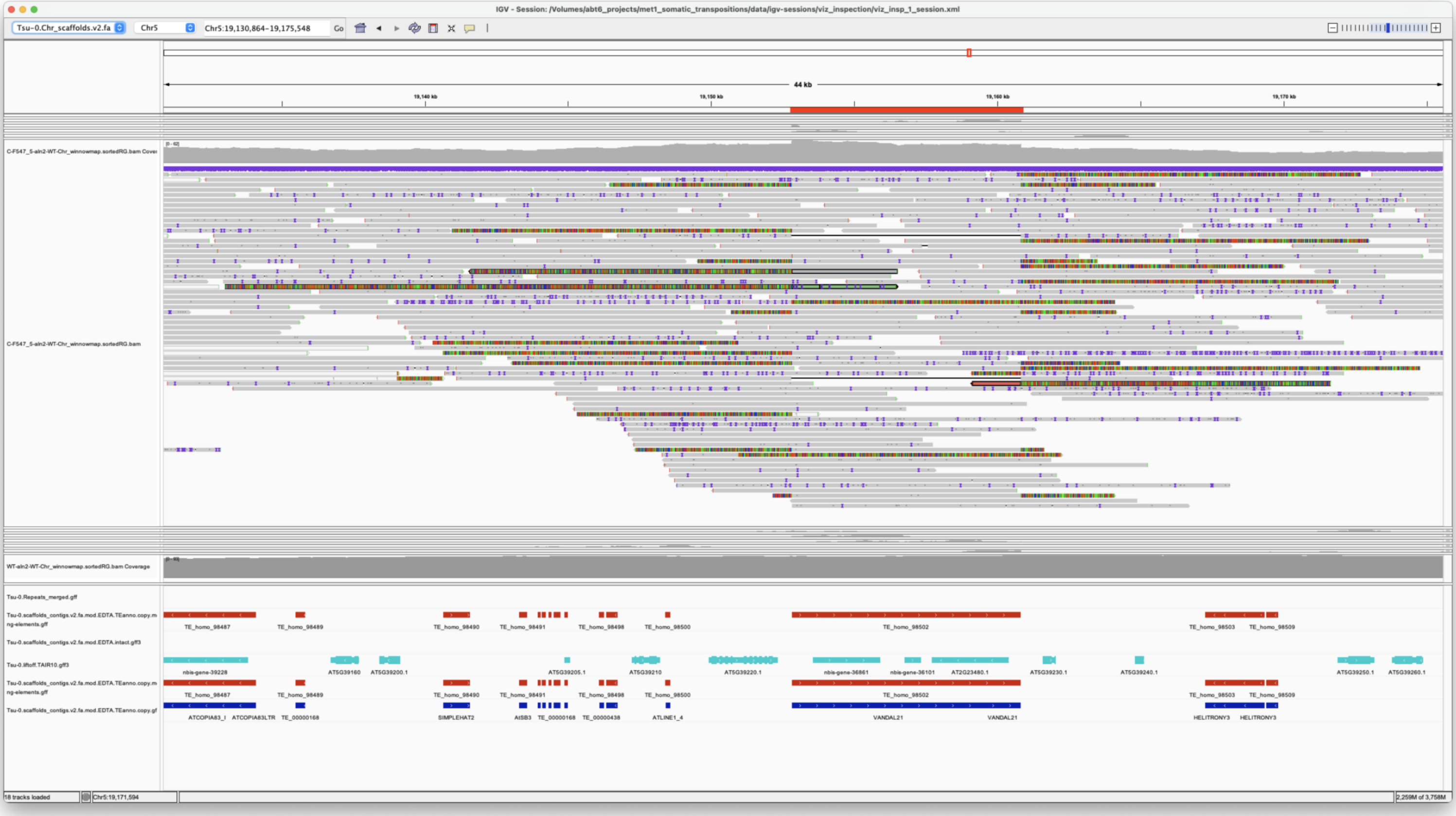

Partial

Confirmed

Chr3 3638915 3638915 + 1 Chr5;21419693;21425022;ATCOPIA93_Evade m64079_240212_113350/33423970/ccs met1_05

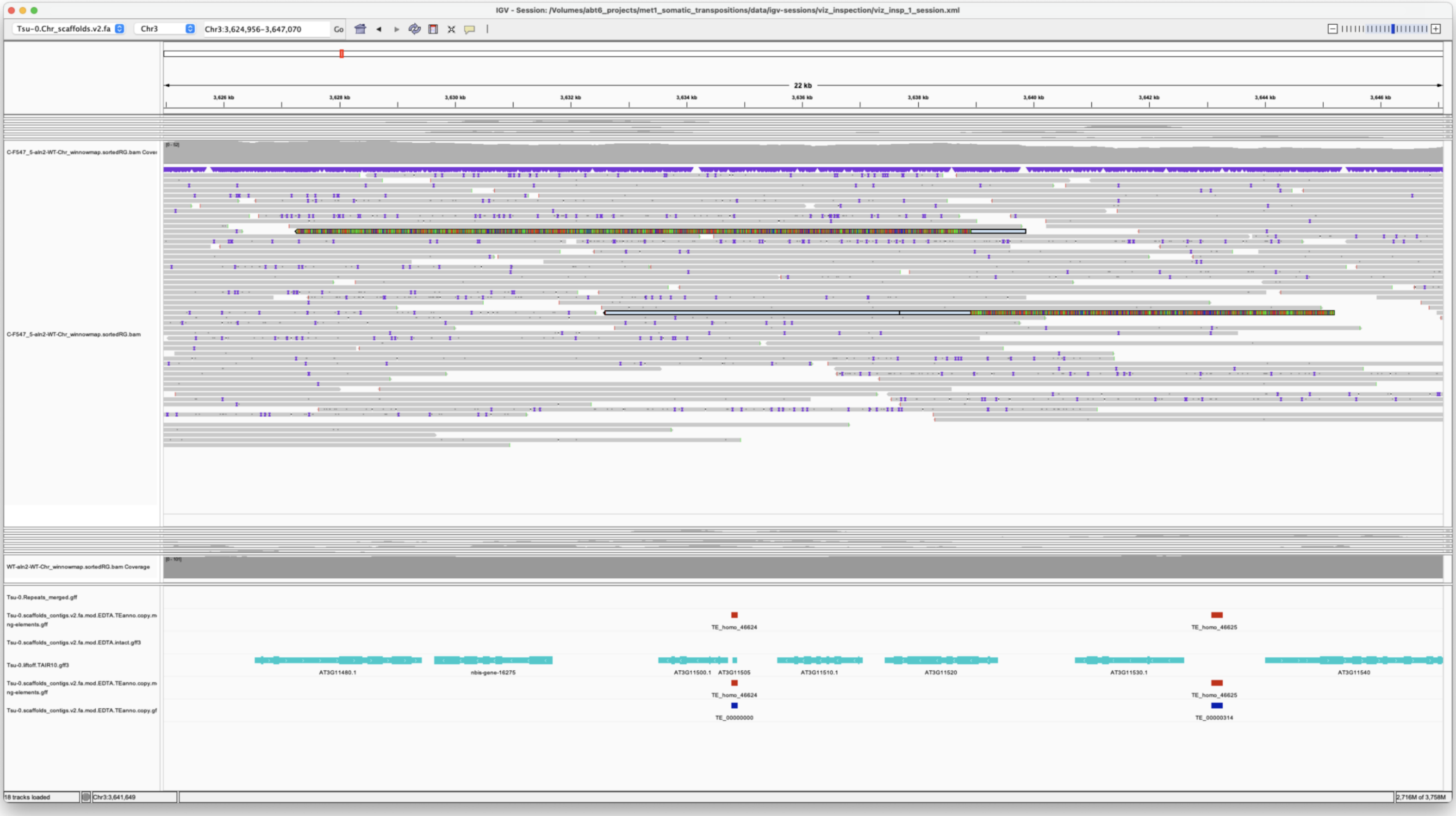

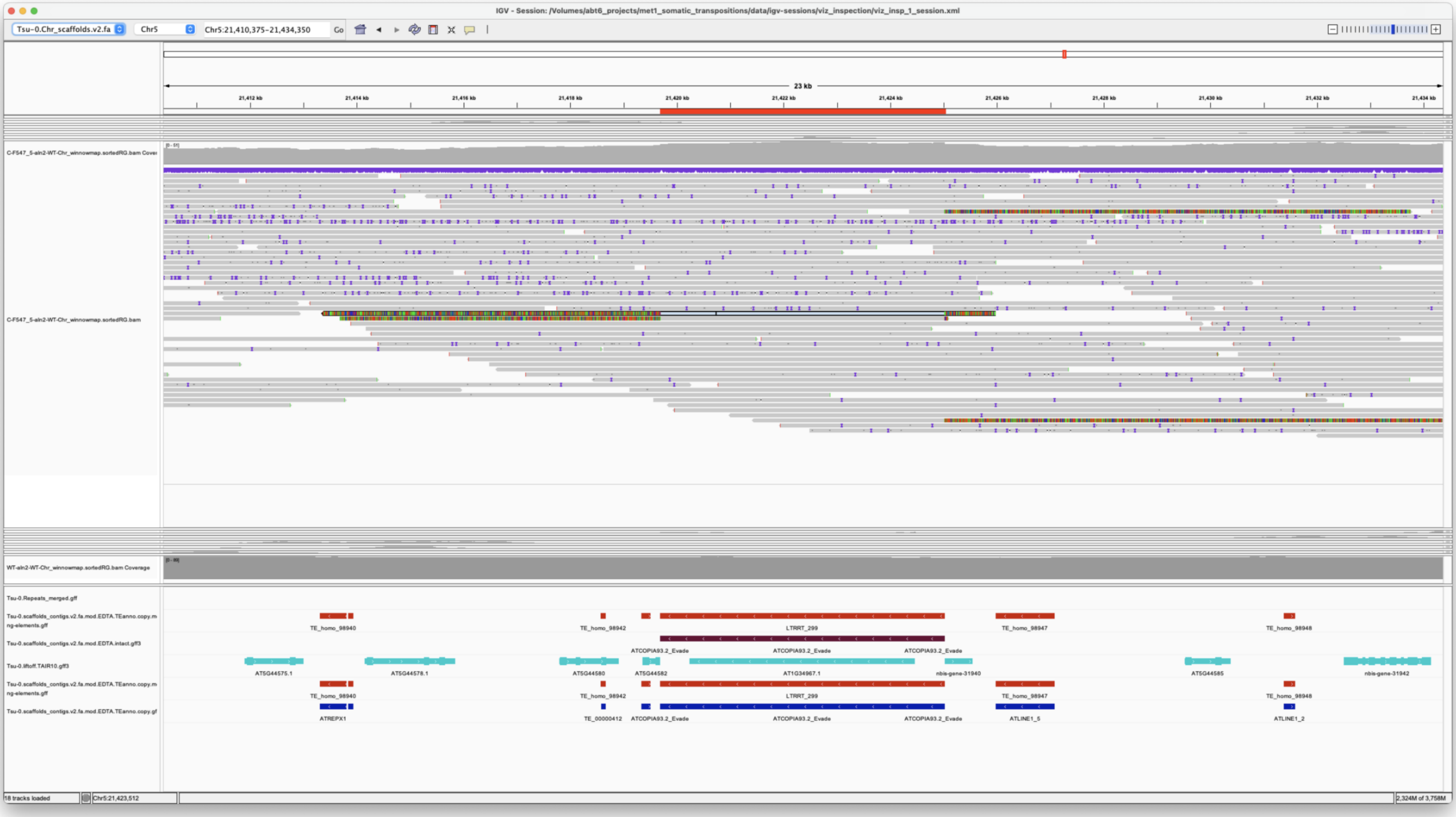

Central
TSD

Confirmed

Chr3 5381967 5381967 + 1 Chr3;20158137;20166150;VANDAL6 m64079_221220_112036/173738151/ccs met1_05

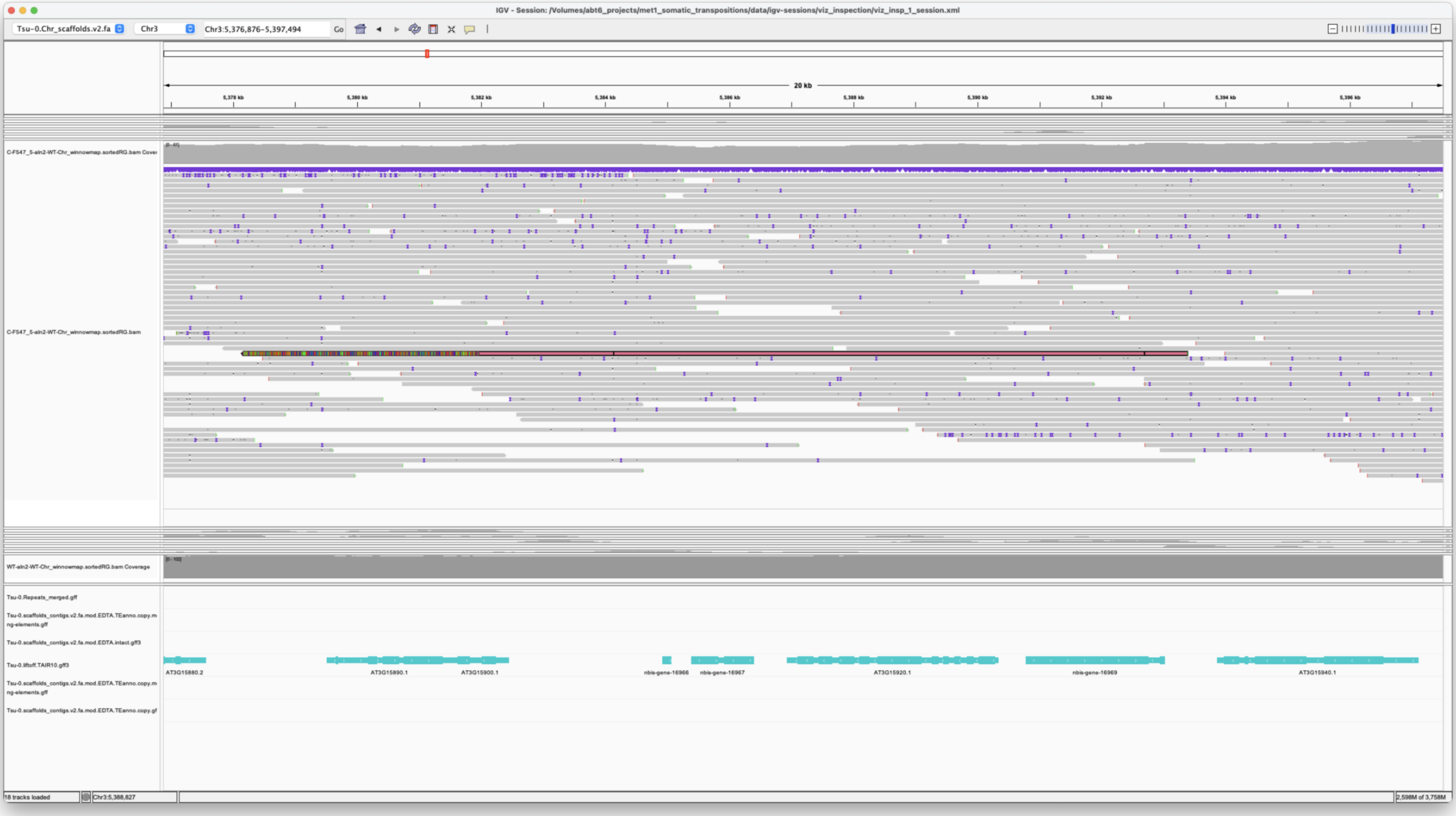

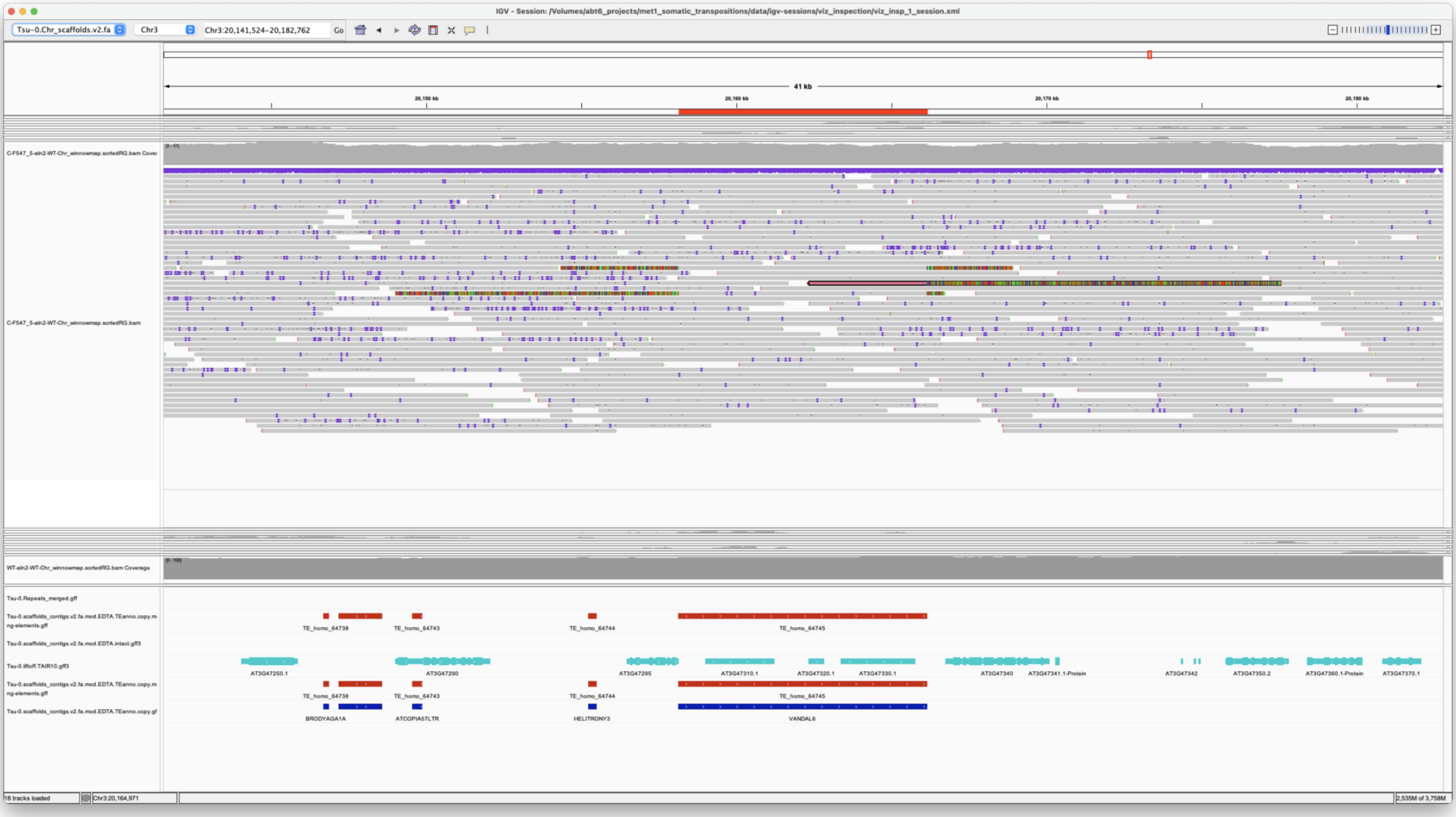

Partial

Confirmed

Chr3 13611705 13611705 + 1 Chr5;19152829;19160826;VANDAL21 m64079_221220_112036/53084508/ccs met1_05

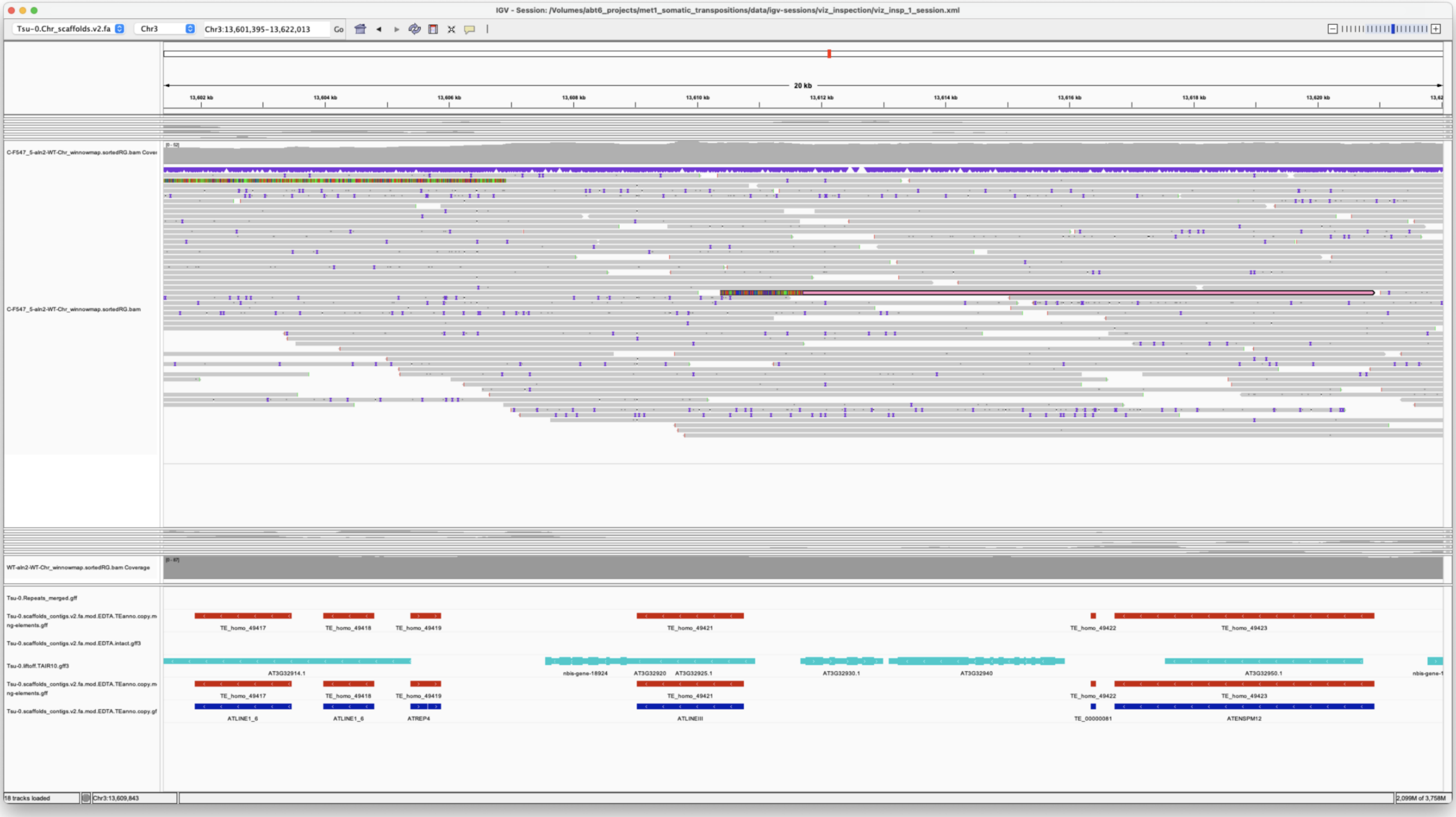

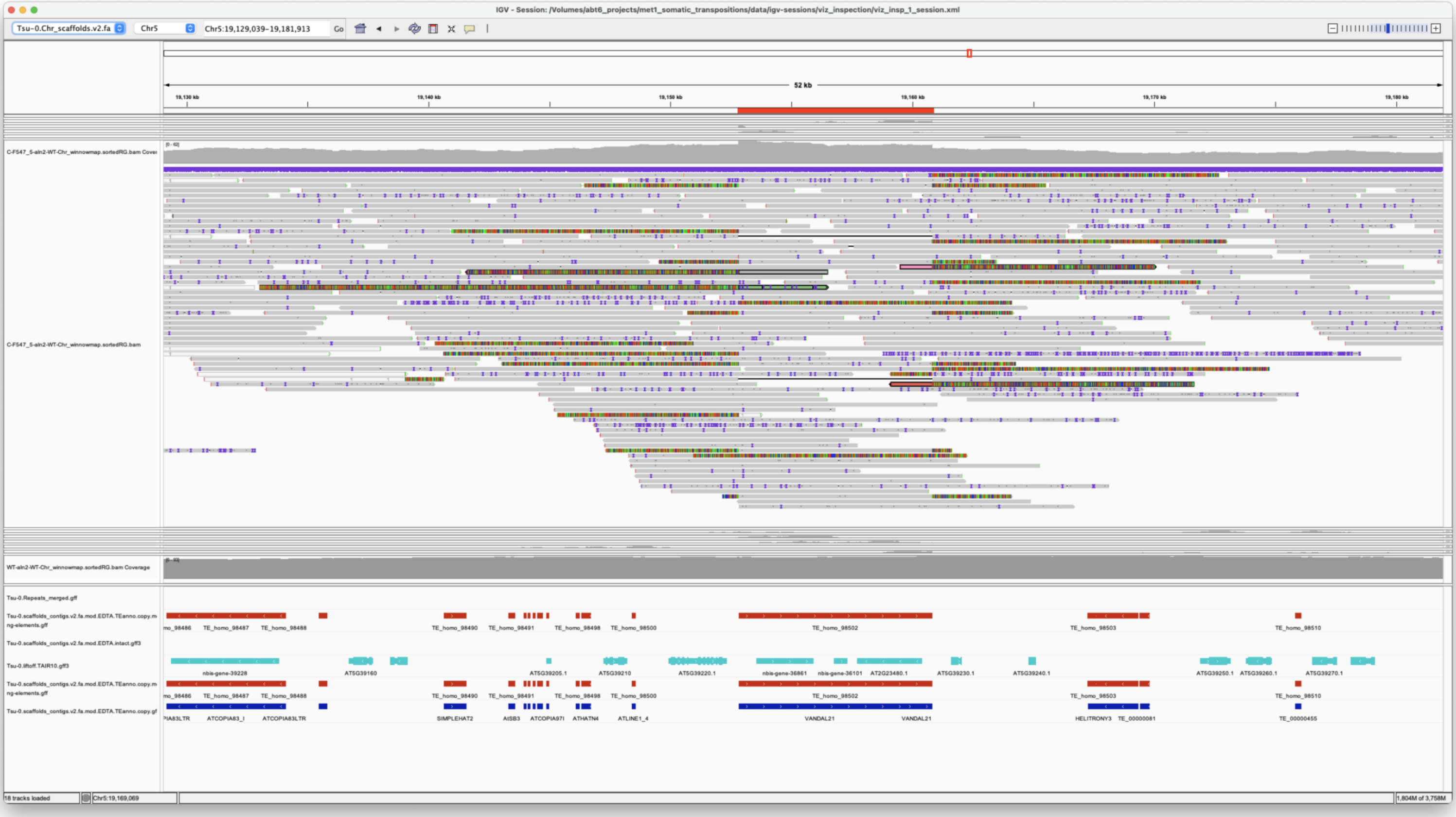

Partial

Confirmed

Chr3 21226953 21226953 + 1 Chr3;16344522;16352497;VANDAL6 m64079_221220_112036/133432670/ccs met1_05

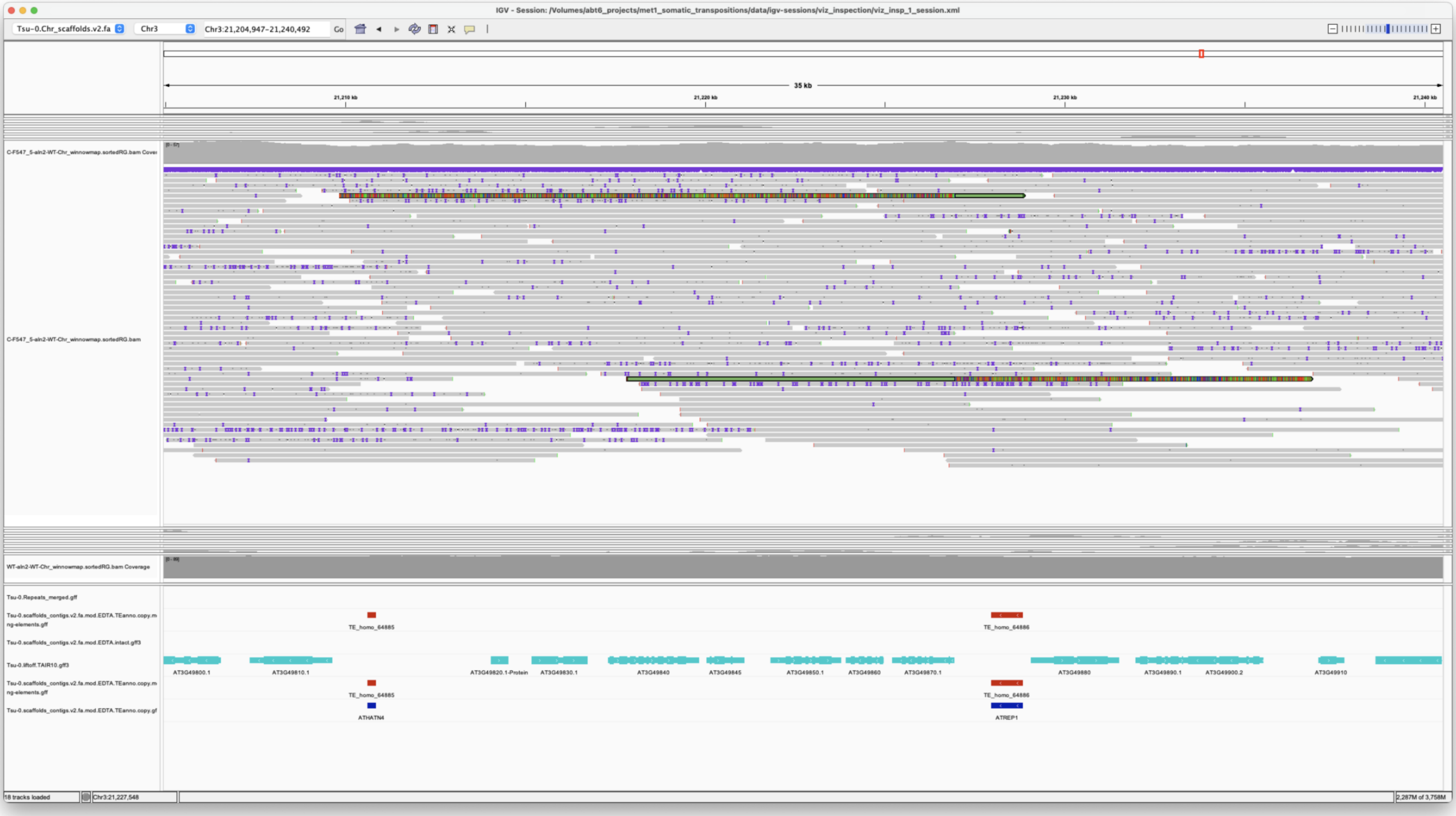

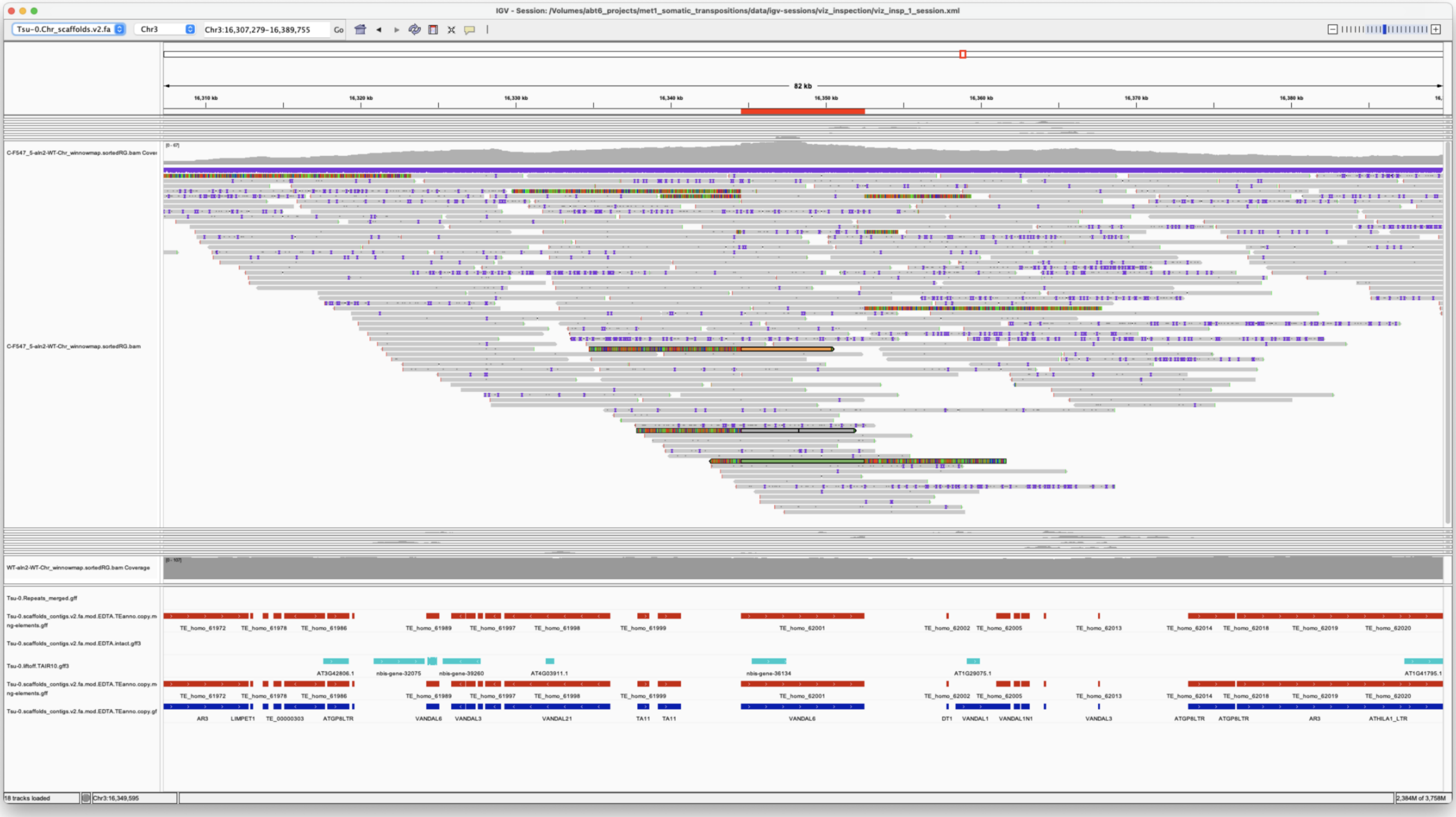

Central
TSD

Confirmed

Chr4 1123607 1123607 - 1 Chr1;11941106;11946436;ATCOPIA93_Evade m64079_240212_113350/8193103/ccs met1_05

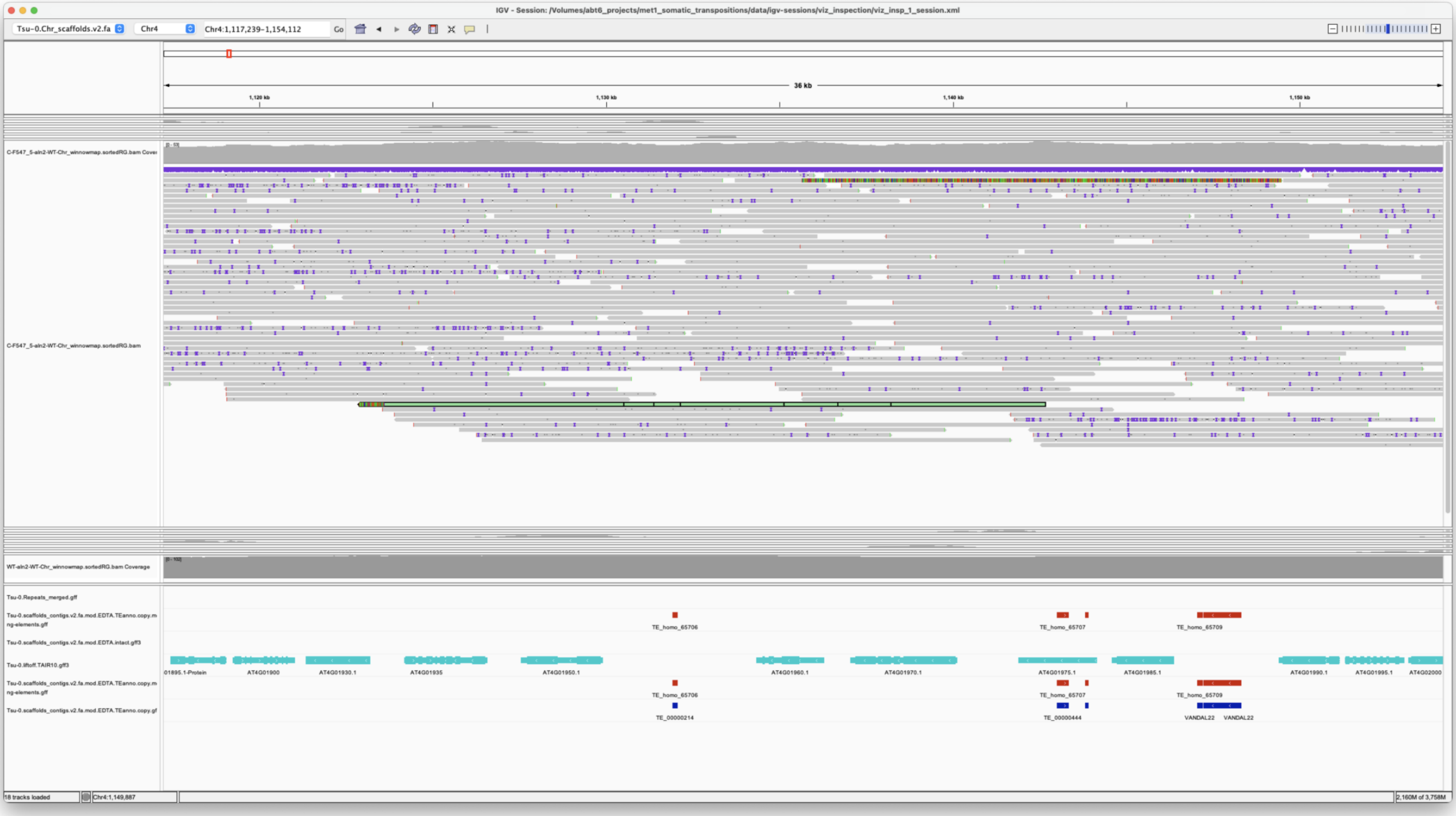

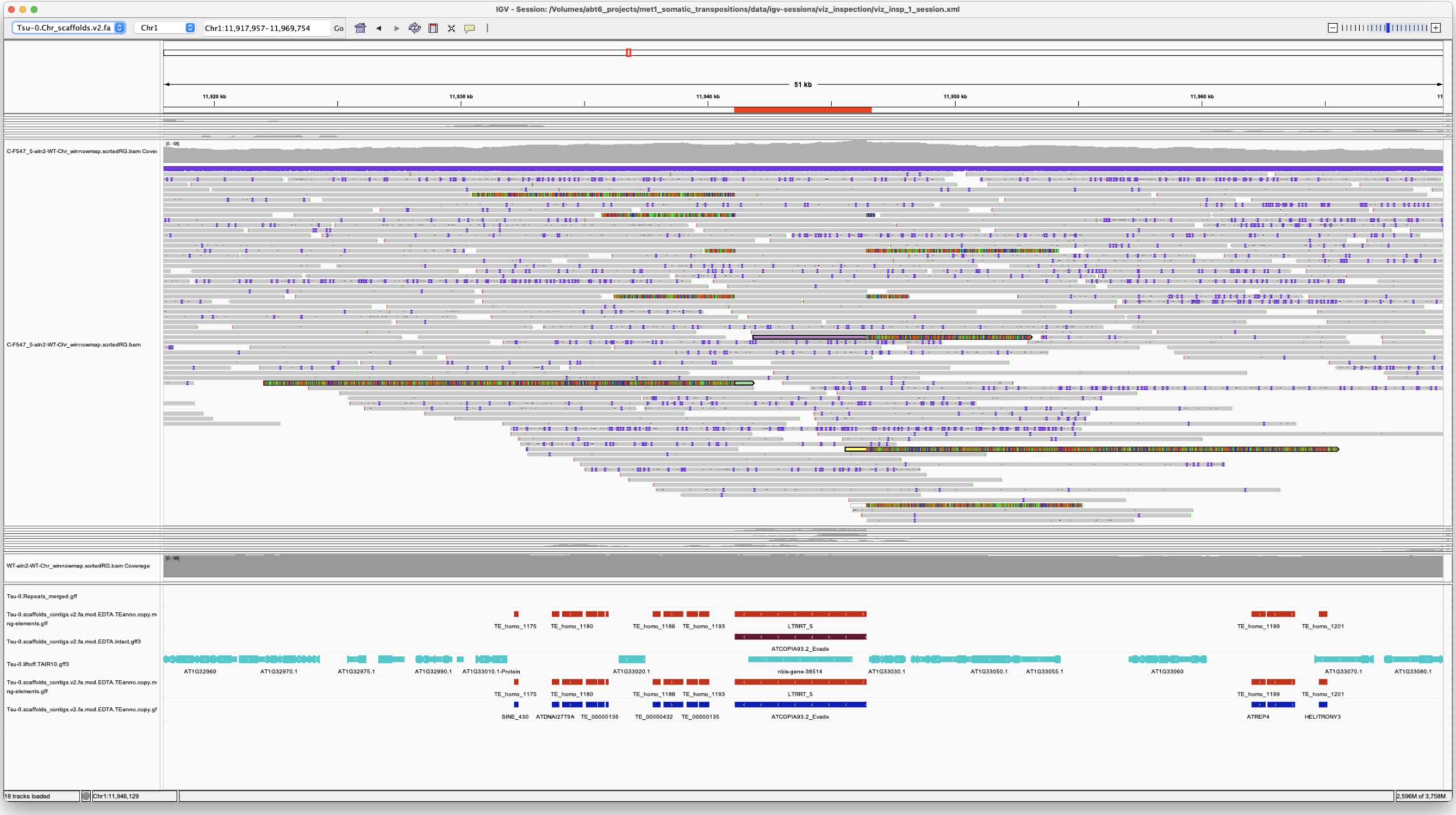

Partial

Confirmed

Chr4 1848707 1848707 - 1 Chr5;21419693;21425022;ATCOPIA93_Evade m64079_240212_113350/110233096/ccs met1_05

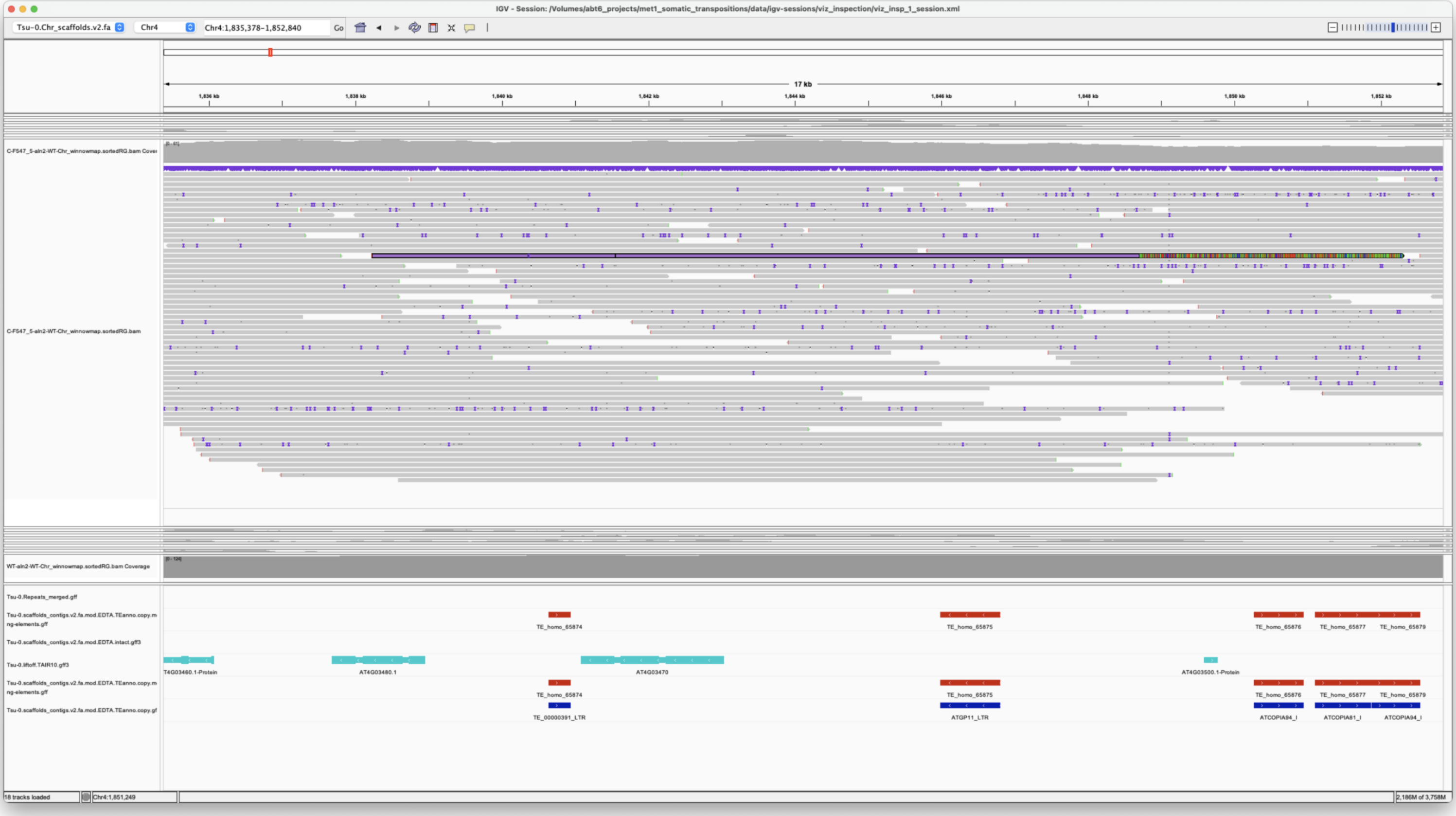

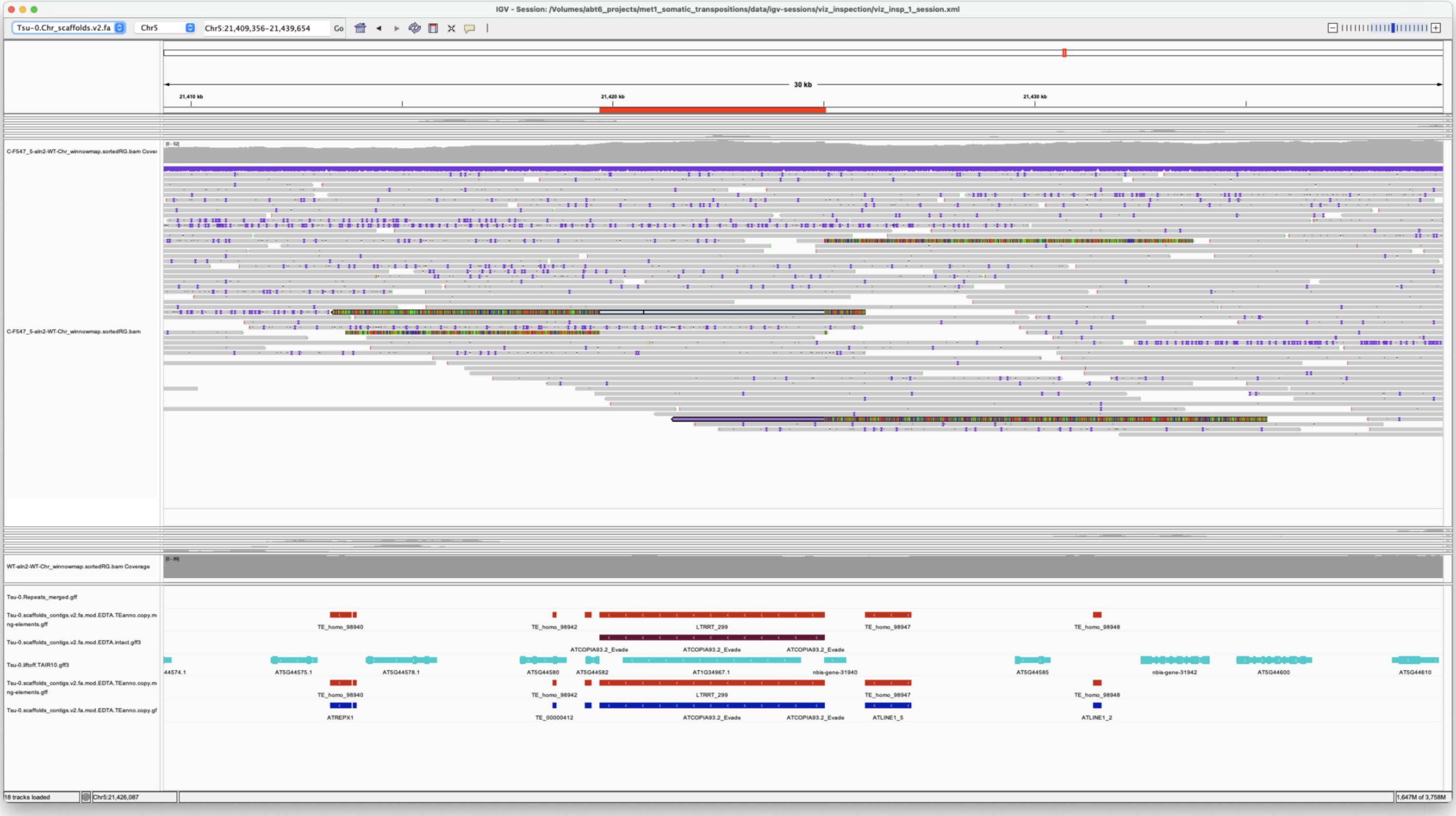

Partial

Confirmed

Chr4 9987688 9987688 - 1 Chr5;19152829;19160826;VANDAL21 m64079_240212_113350/135333141/ccs met1_05

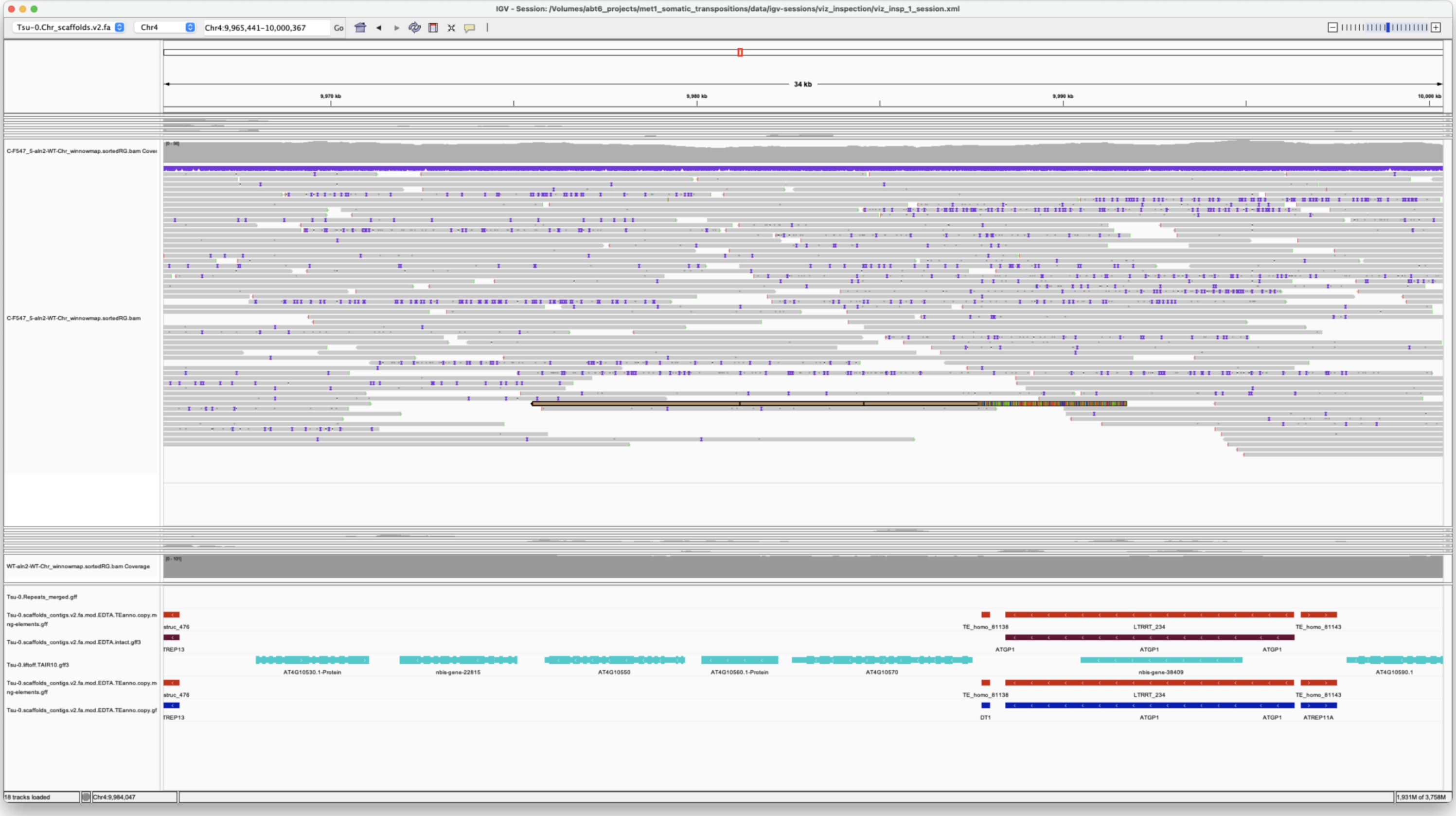

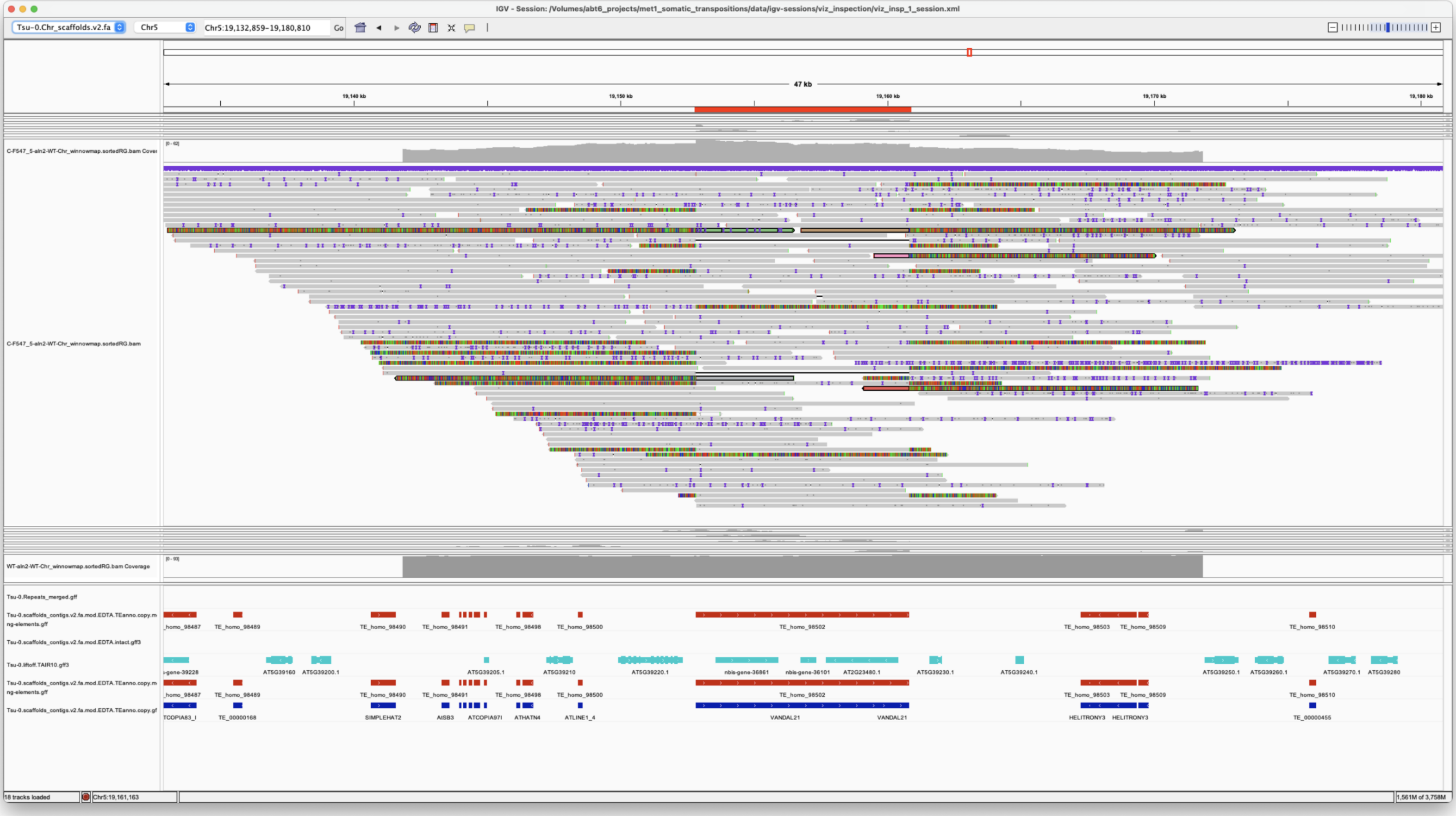

Partial

Confirmed

Chr4 17533761 17533761 - 1 Chr5;21419693;21425022;ATCOPIA93_Evade m64079_221220_112036/32049614/ccs met1_05

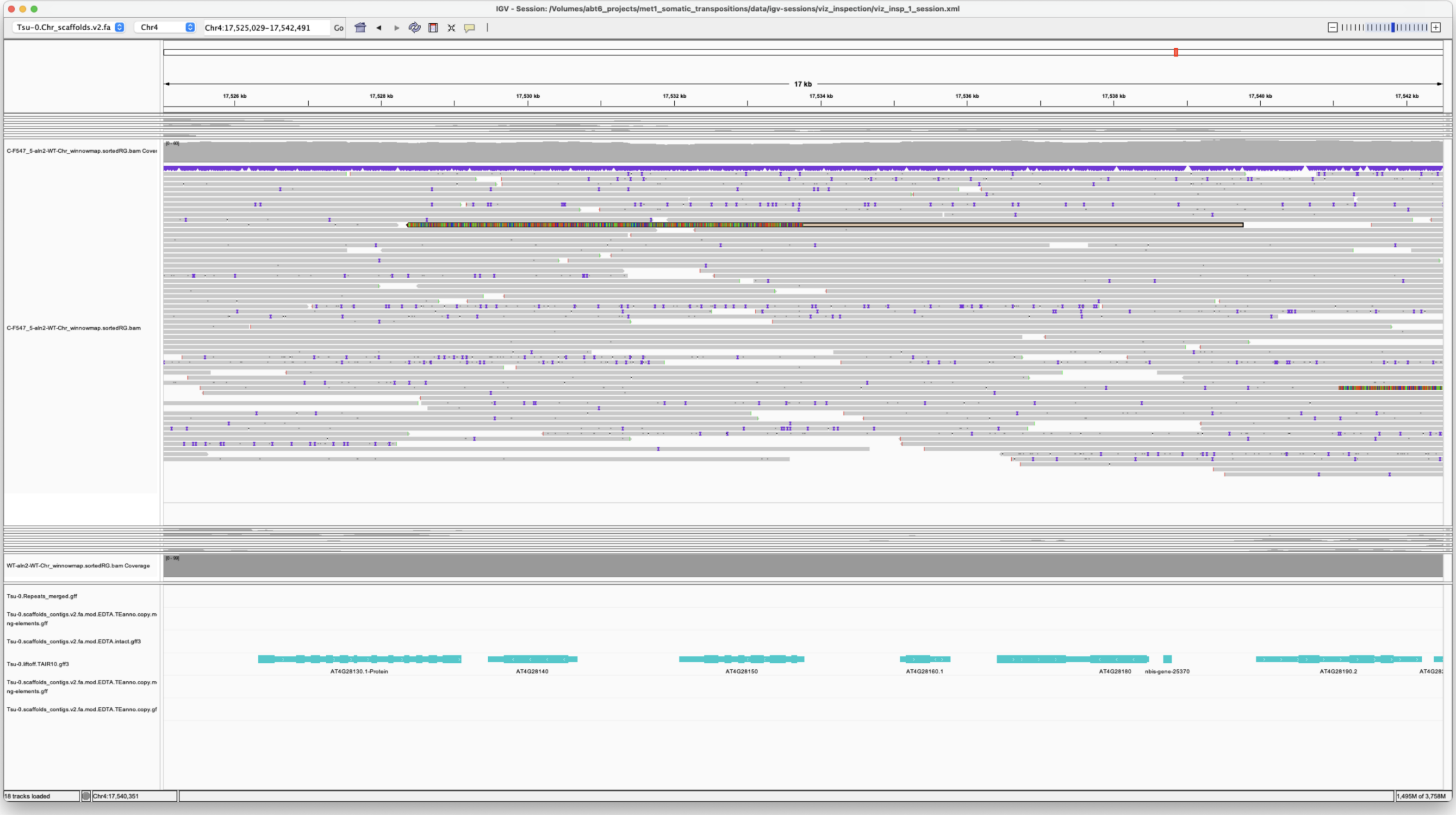

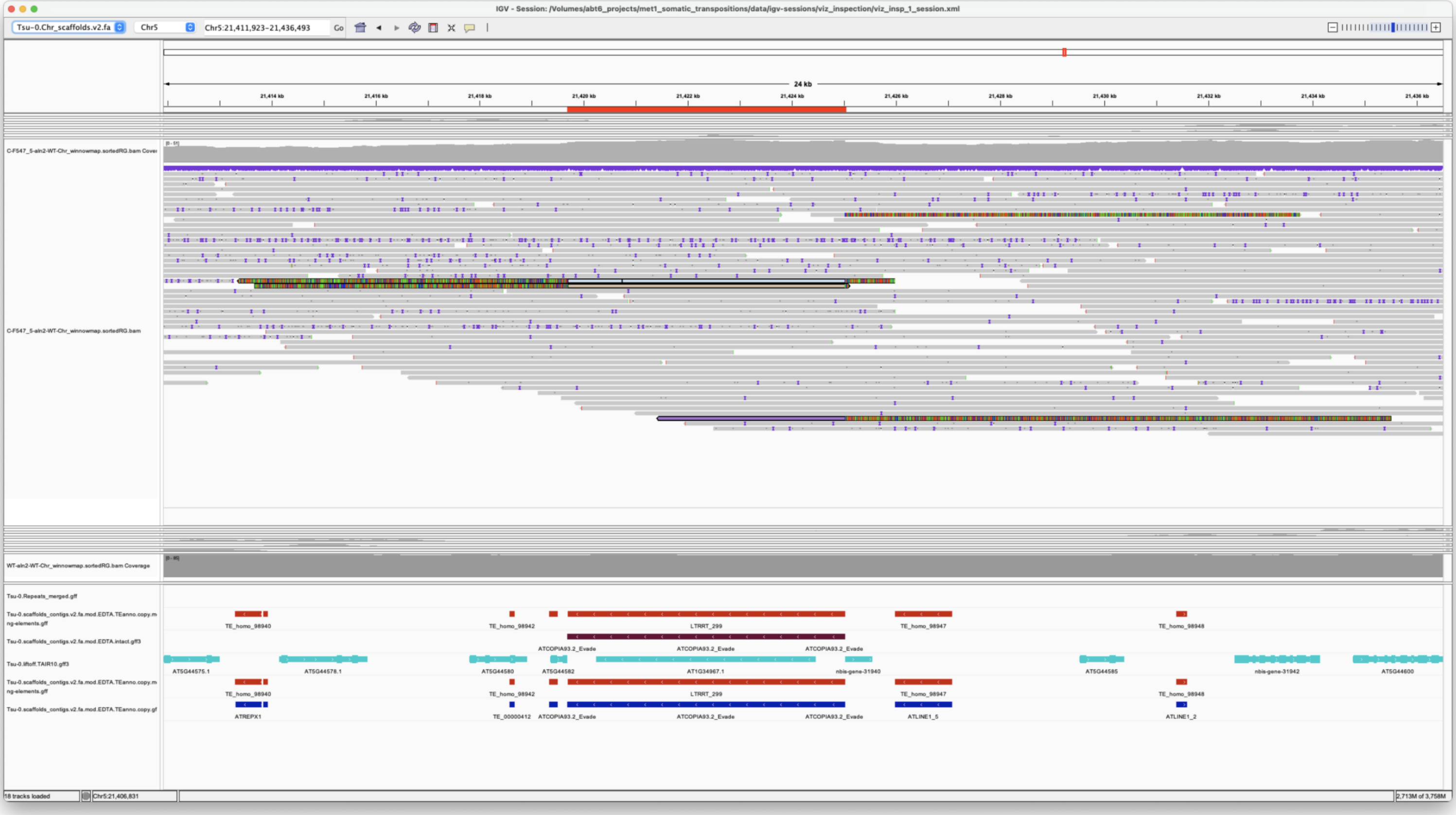

Partial

Confirmed

Chr4 18223745 18223745 + 1 Chr5;19152829;19160826;VANDAL21 m64079_221220_112036/177211687/ccs met1_05

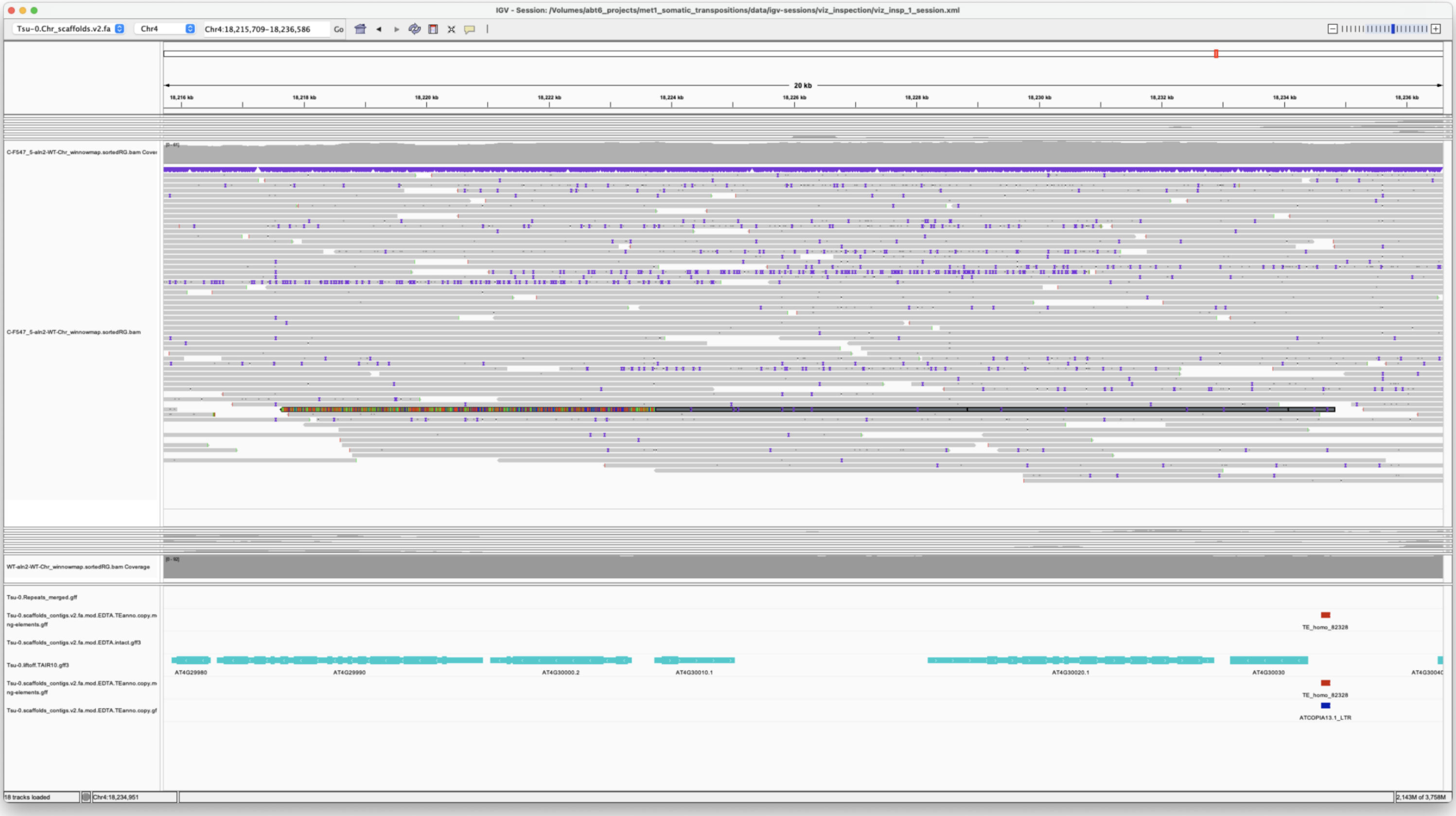

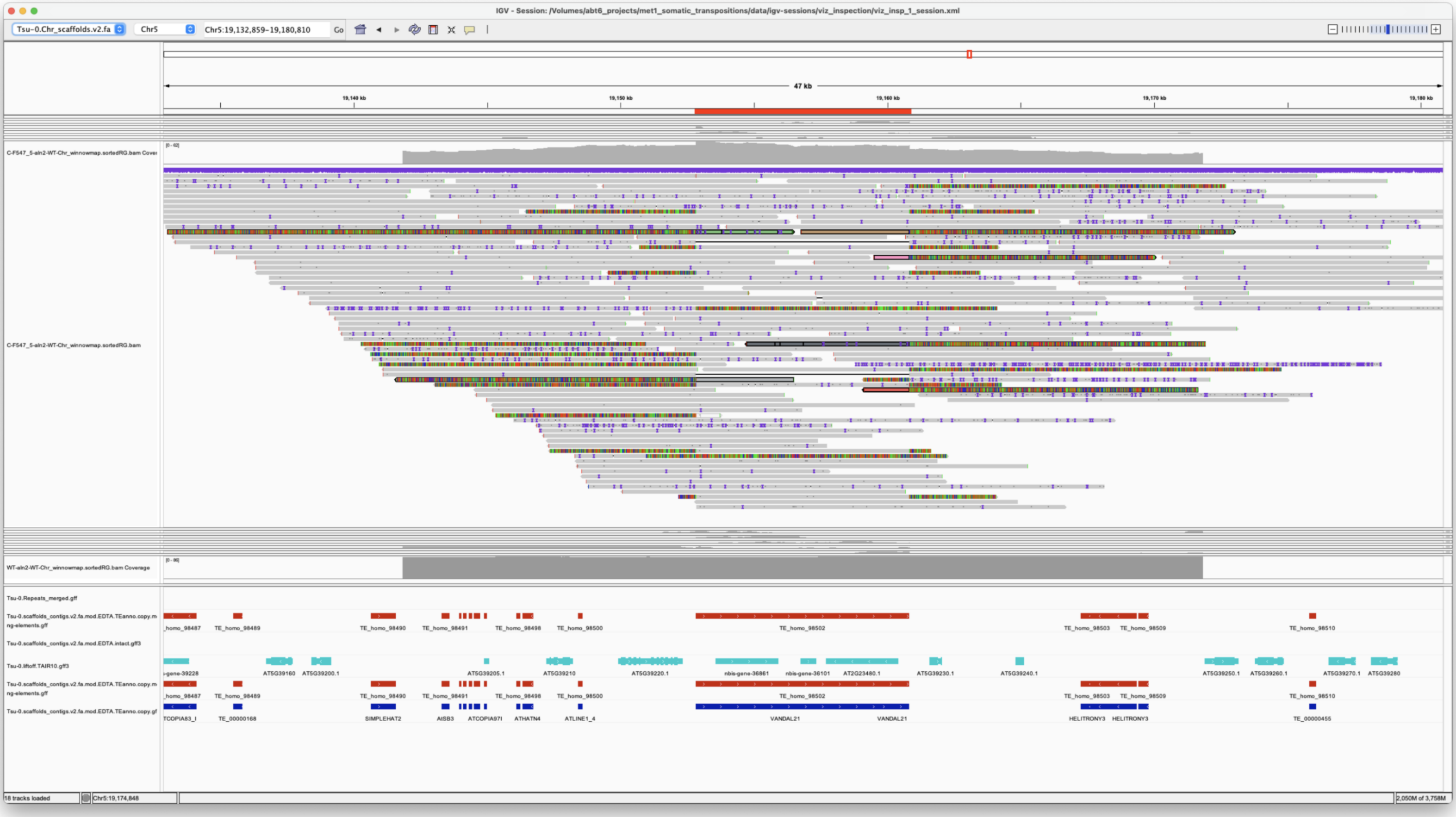

Partial

Confirmed

Chr4 19242778 19242778 - 1 Chr3;16344522;16352497;VANDAL6 m64079_221220_112036/132318972/ccs met1_05

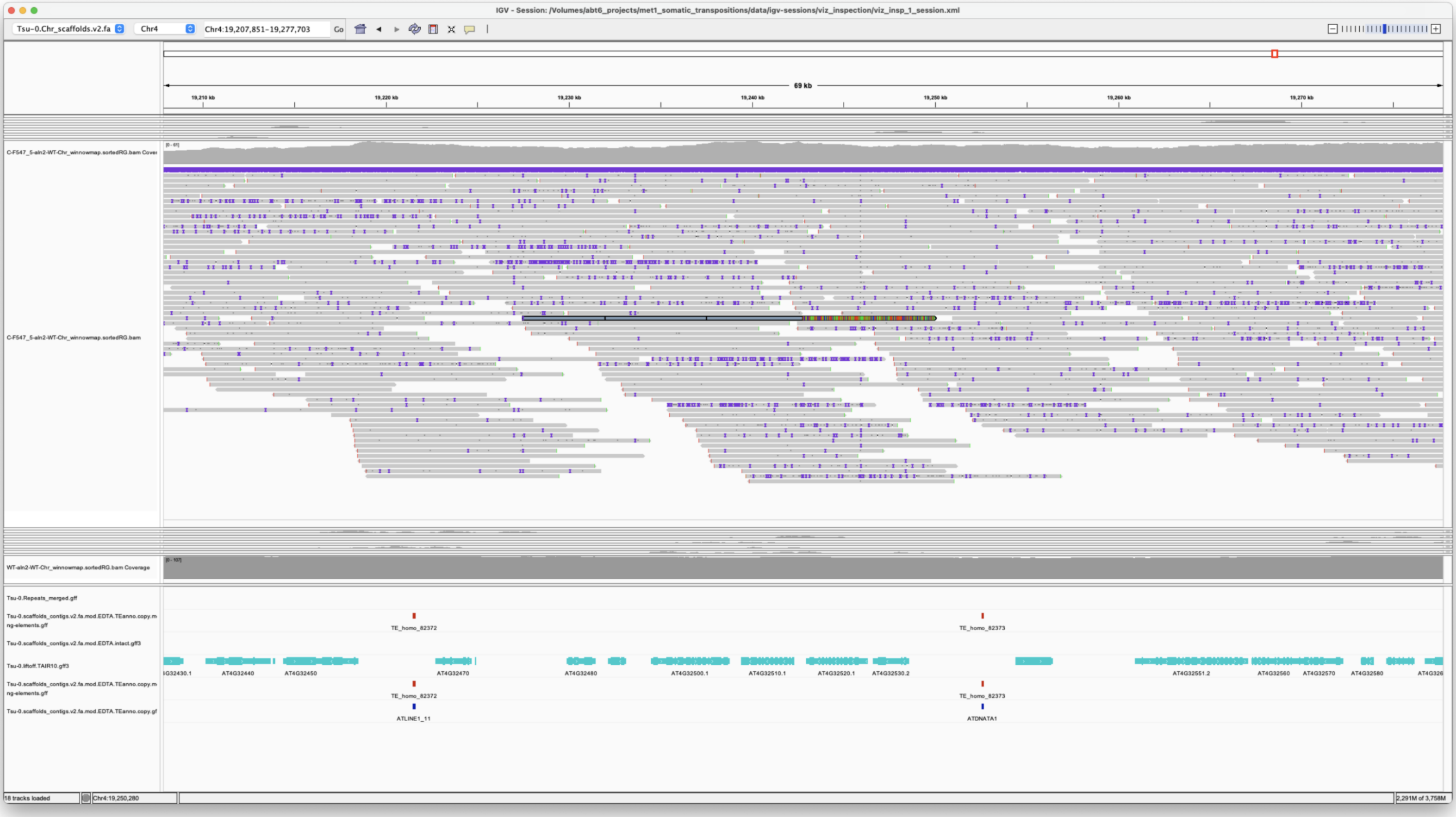

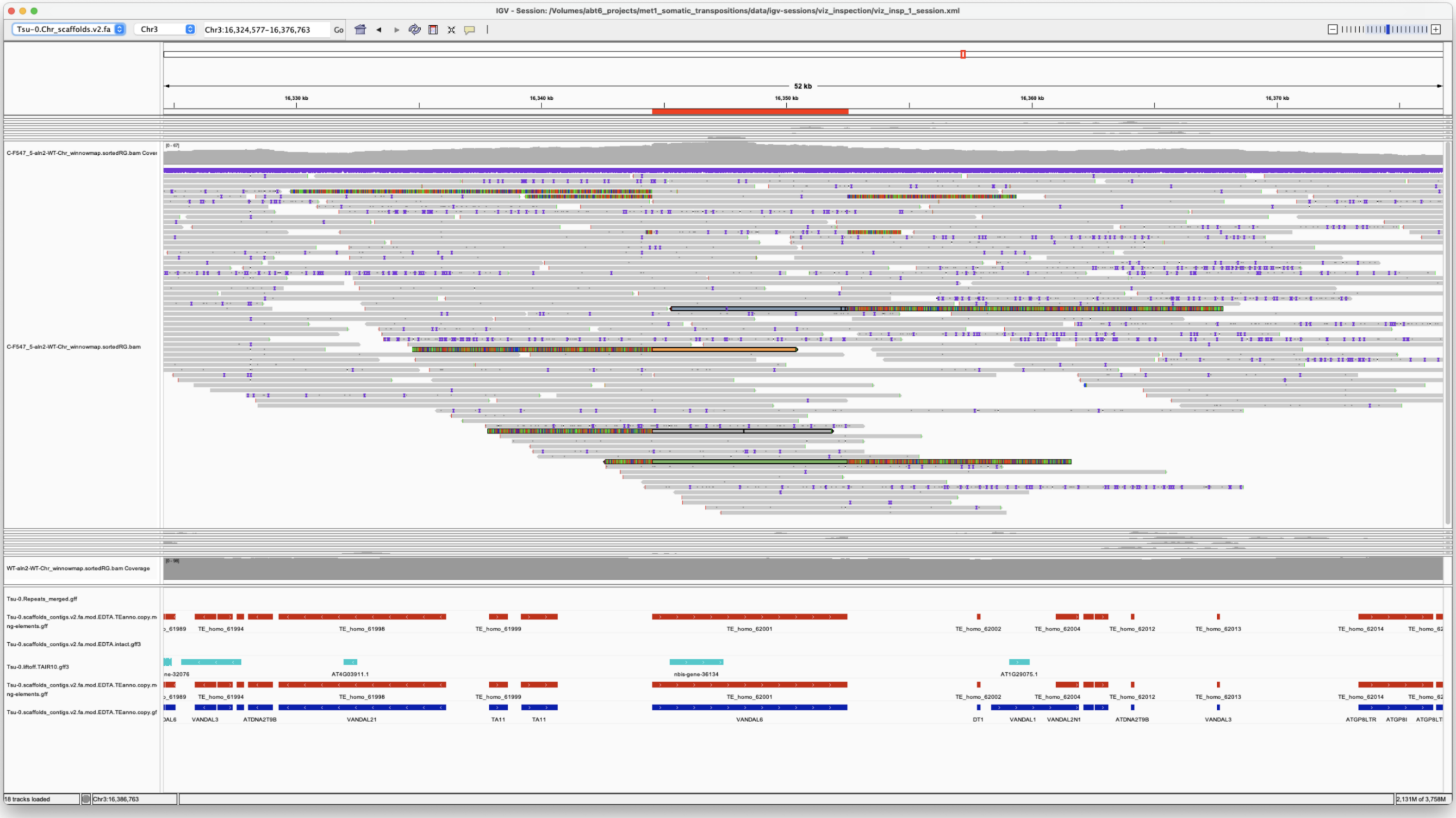

Partial

Confirmed

Chr5 5150522 5150522 + 1 Chr5;19152829;19160826;VANDAL21 m64079_221220_112036/177080054/ccs met1_05

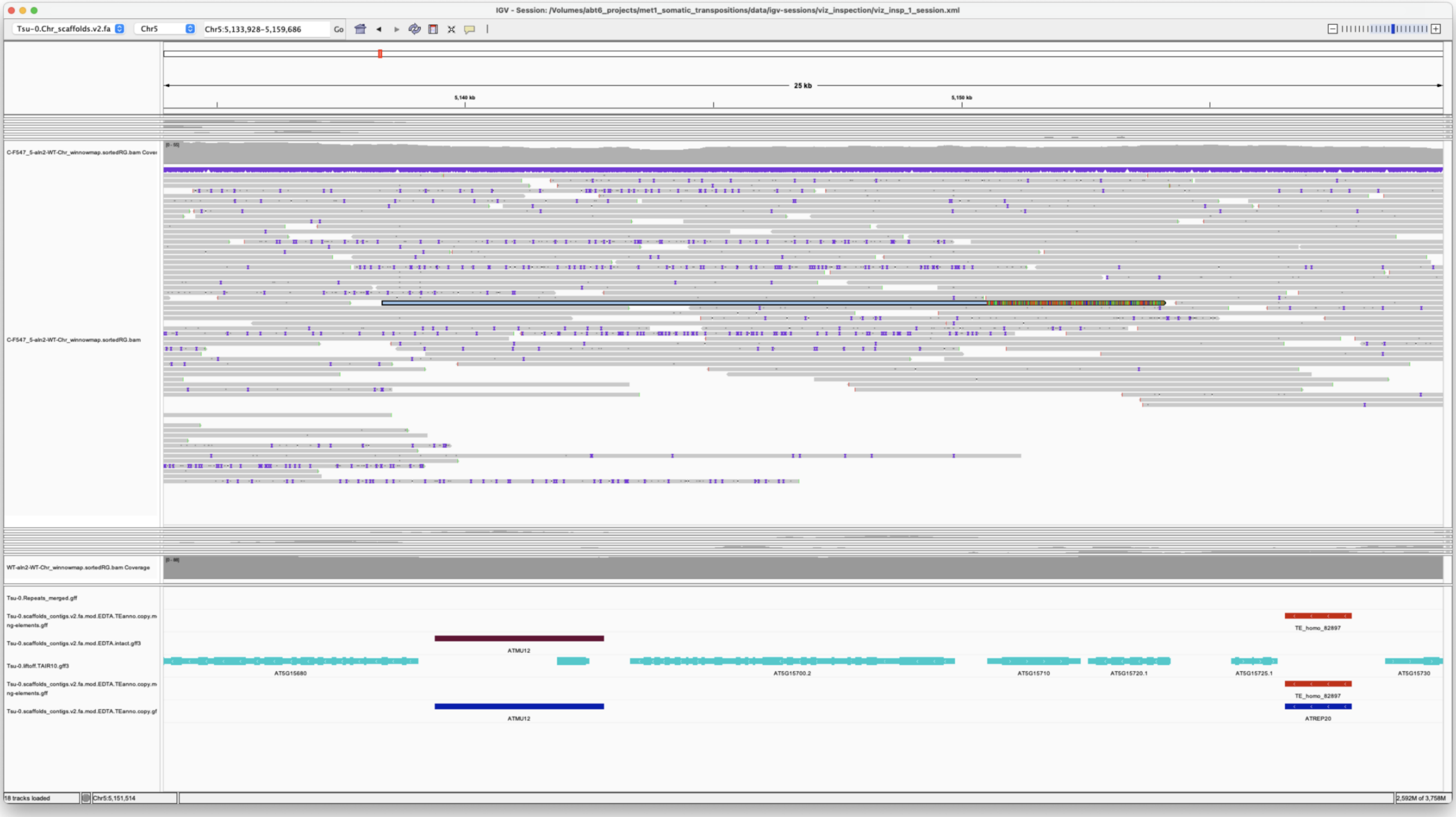

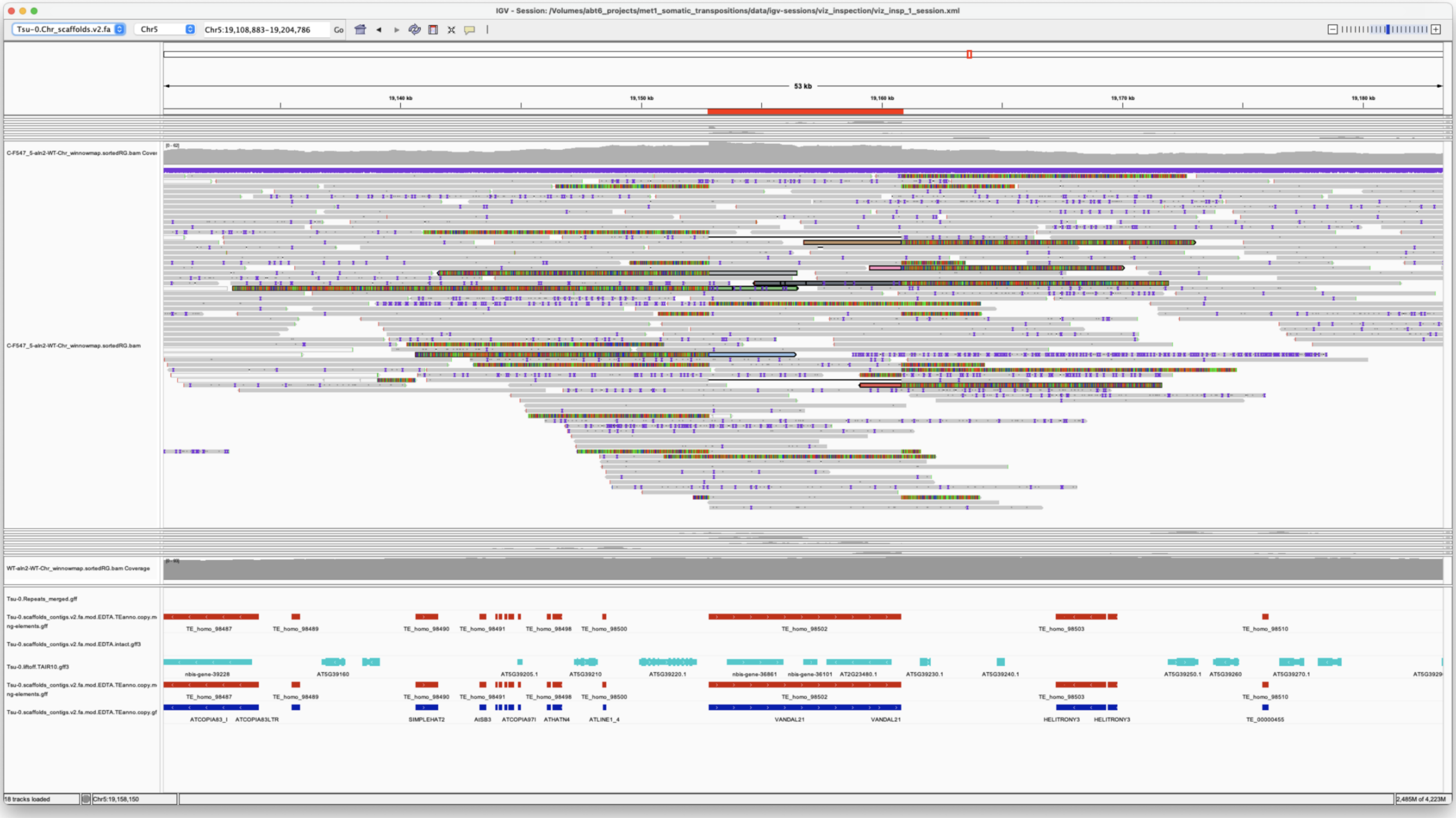

Partial

Confirmed

Chr5 5484748 5484748 + 1 Chr3;20158137;20166150;VANDAL6 m64079_221220_112036/65406206/ccs met1_05

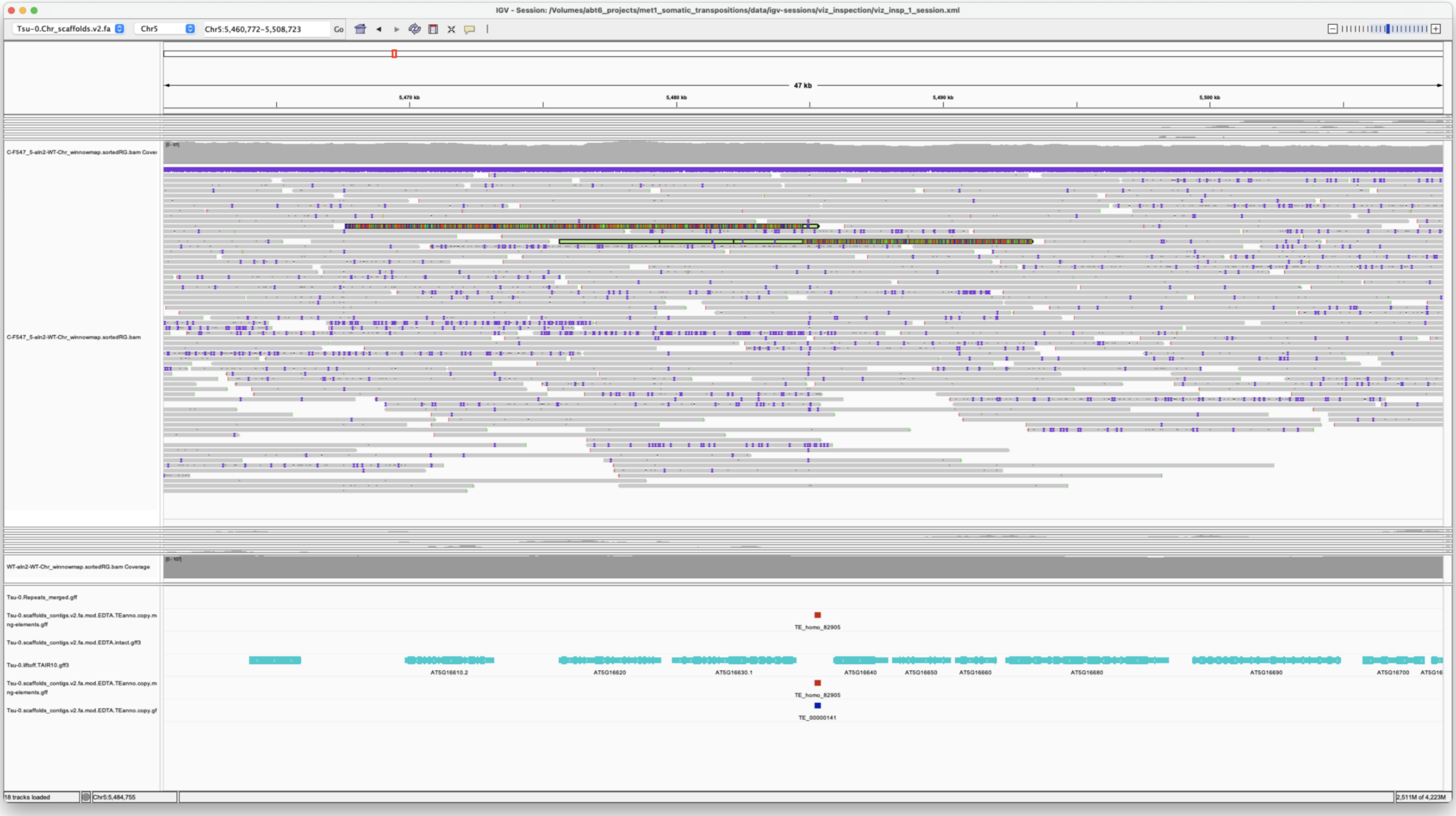

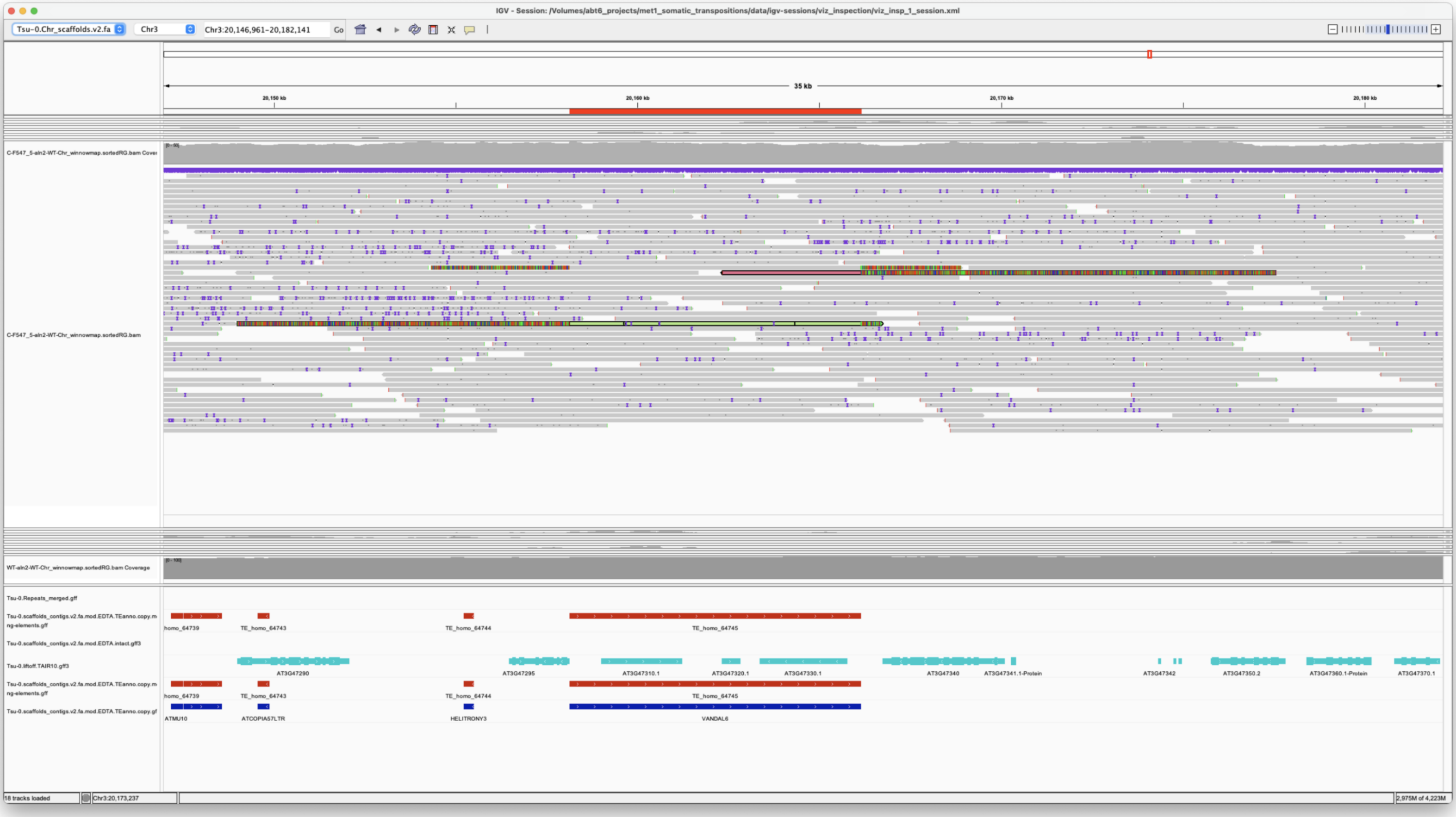

Central
TSD

Confirmed

Chr5 17413821 17413821 - 1 Chr5;19152829;19160826;VANDAL21 m64079_221220_112036/160761442/ccs met1_05

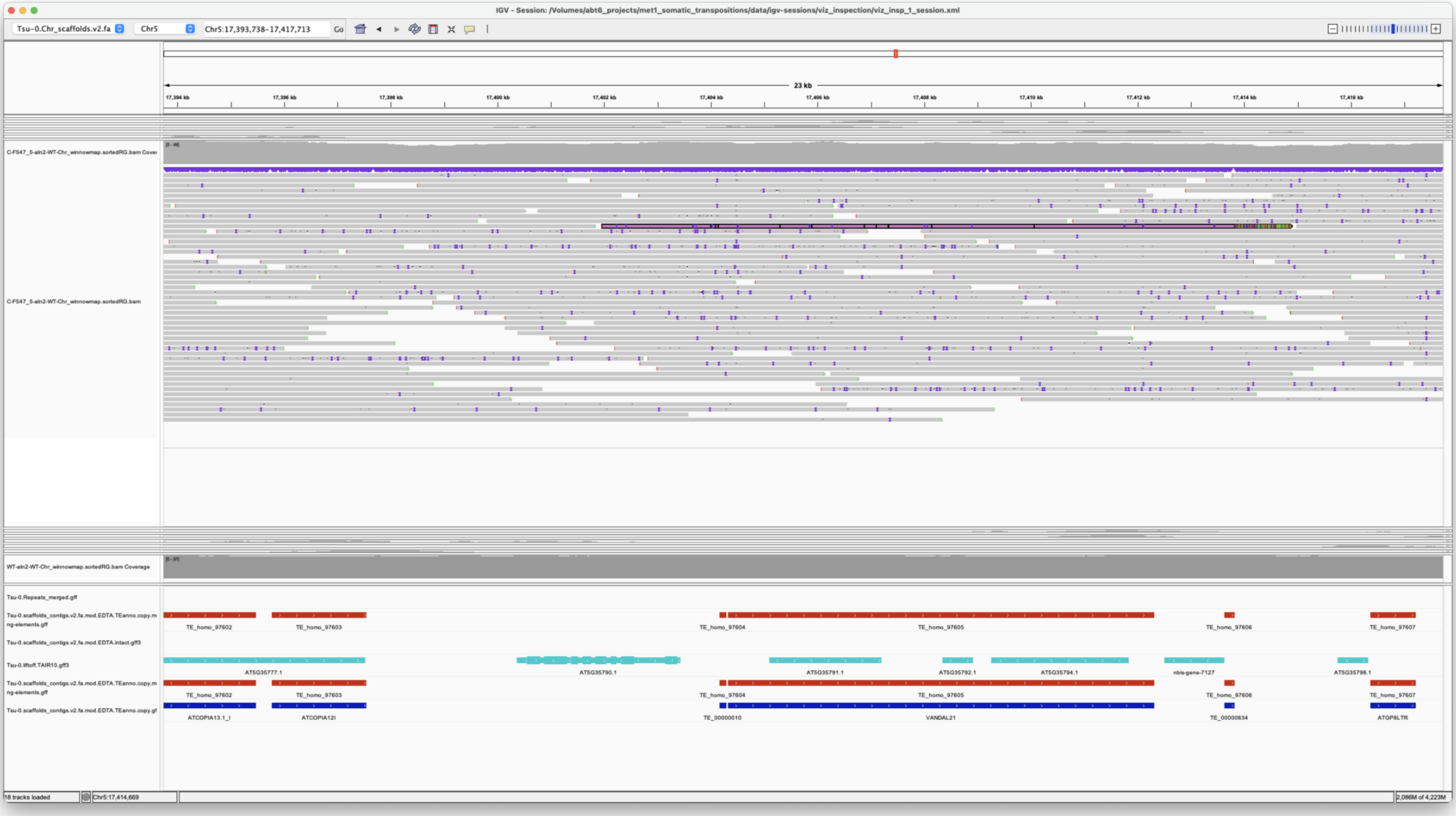

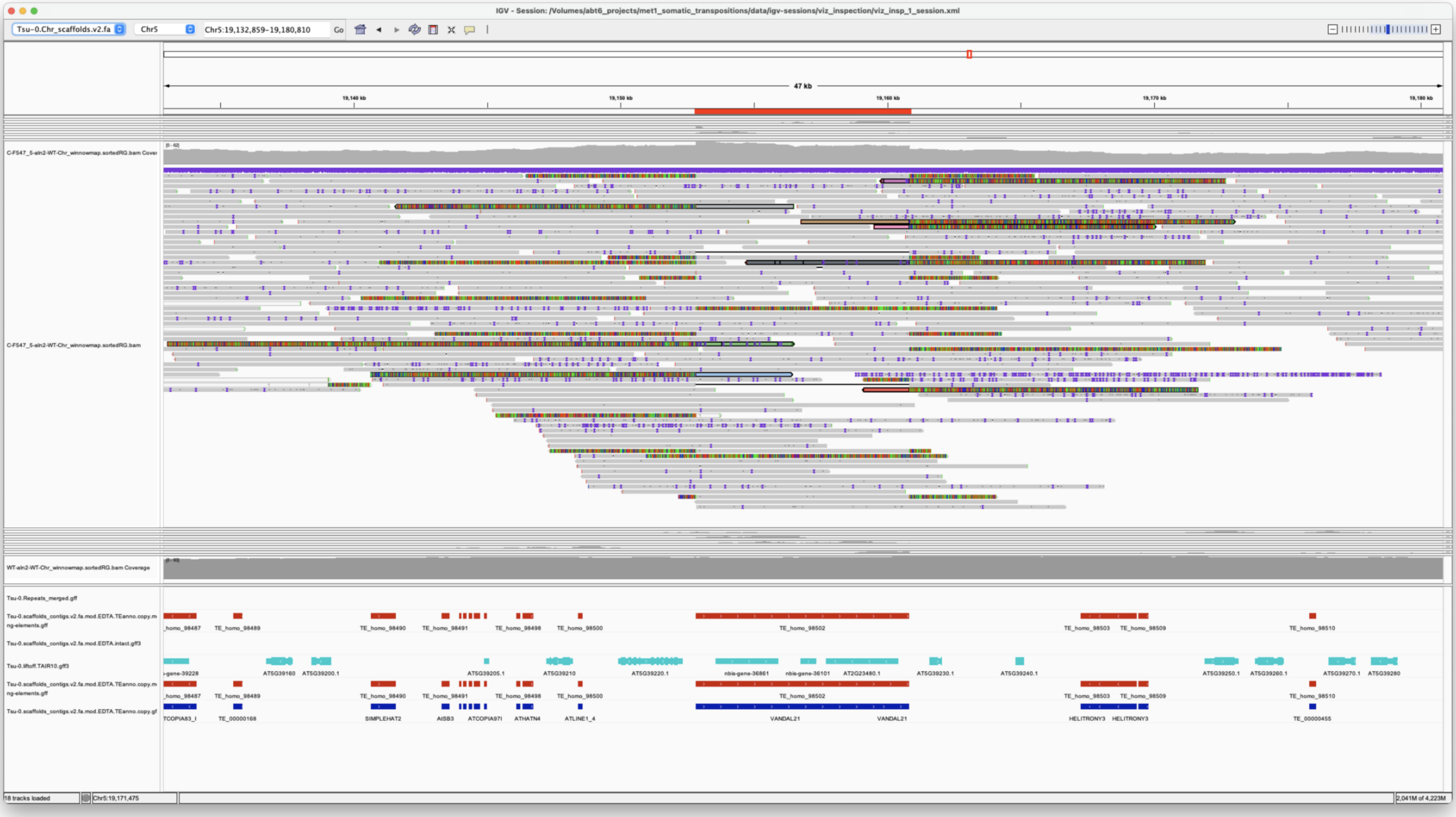

Confirmed

Chr5 19150958 19150958 + 1 Chr5;19152829;19160826;VANDAL21 m64079_221220_112036/34604722/ccs met1_05

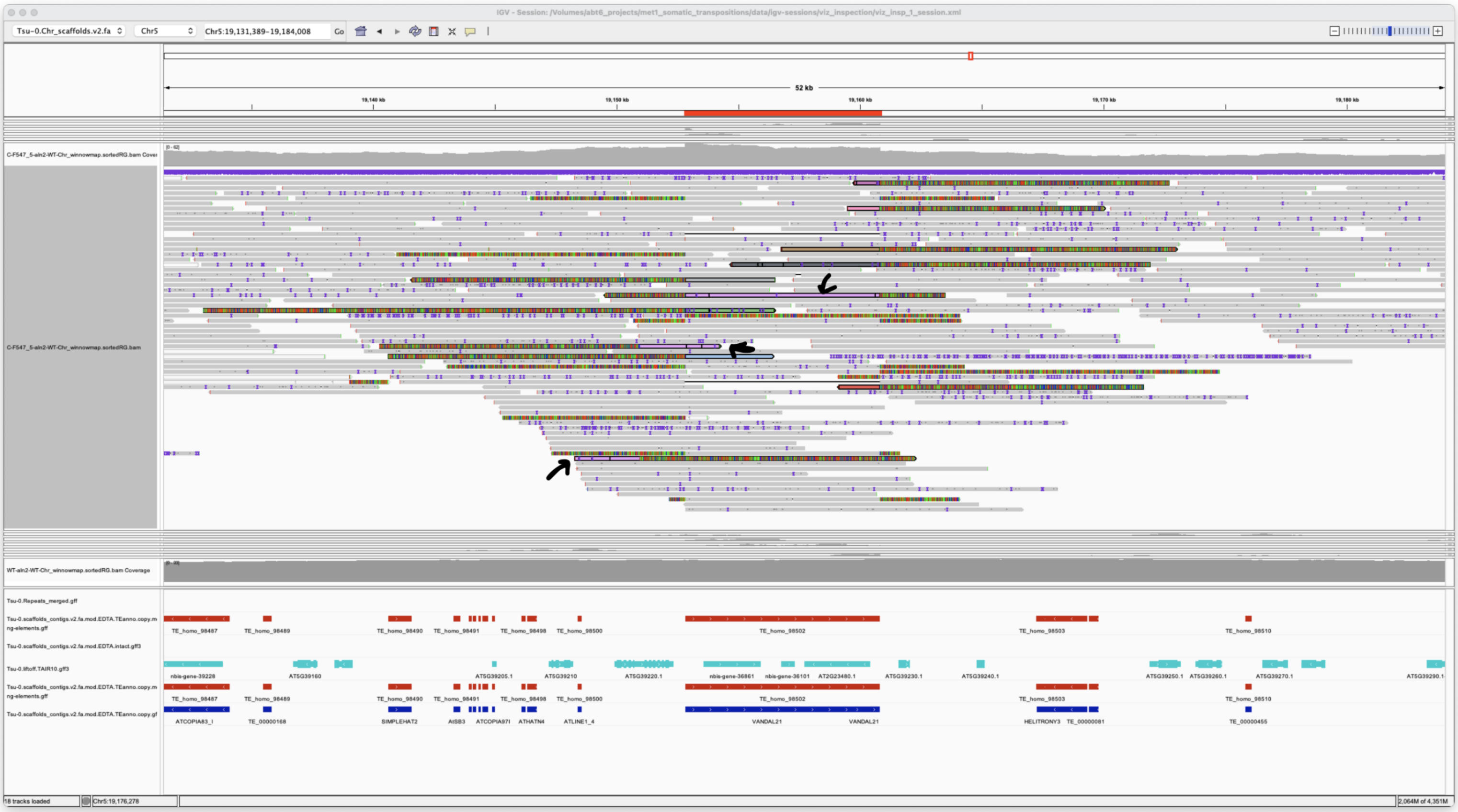

WEIRD!! EITHER REARRANGEMENT OR TRANSPOSITION VERY CLOSE!!

DEEMED TRANSPOSITION

Central
TSD

Confirmed

Chr5 19848968 19848968 - 1 Chr5;19877283;19884298;VANDAL21 m64079_240212_113350/177145737/ccs met1_05

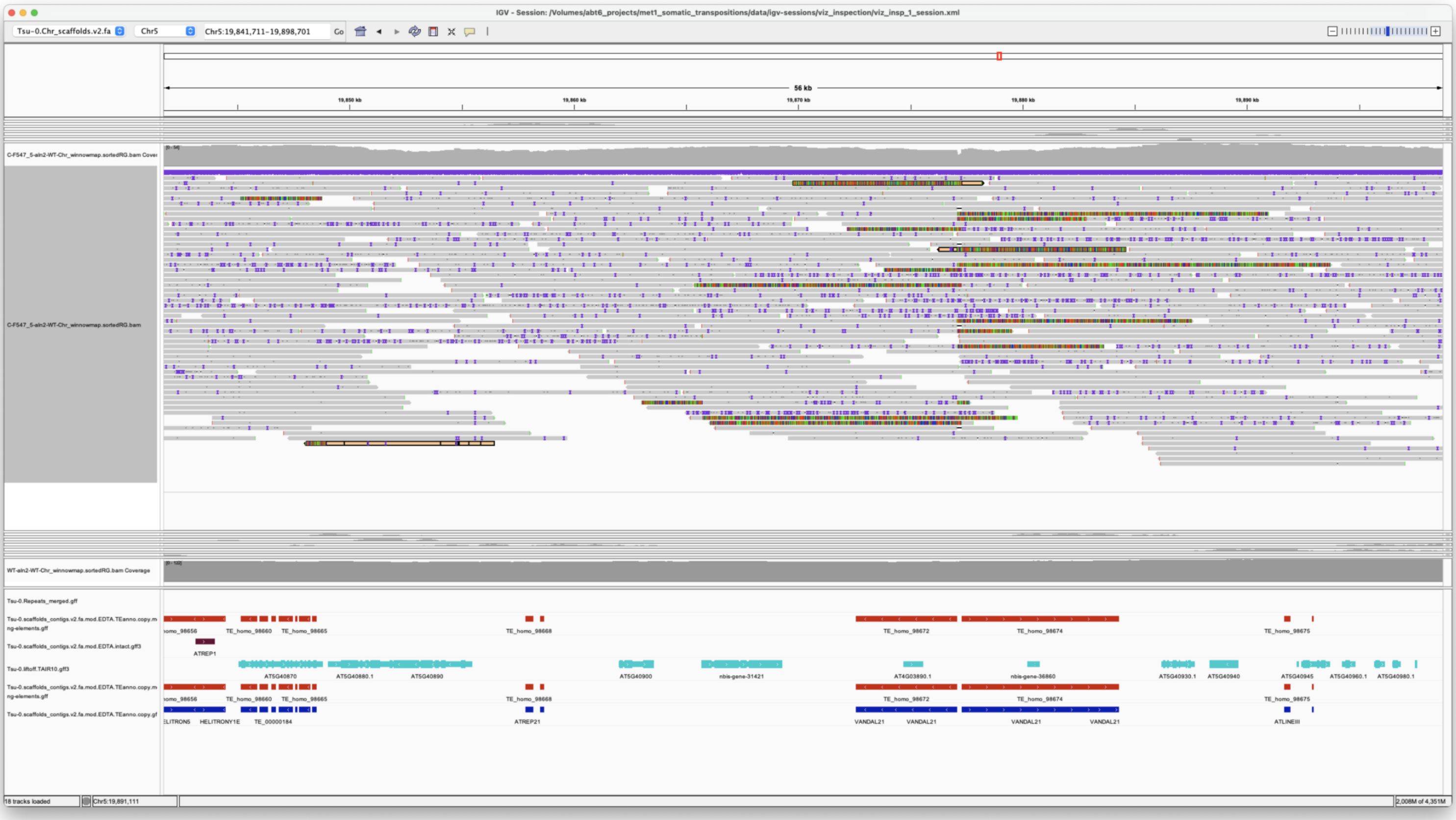

Hypermutable region

Rearrangement

unsupported

Chr5 28025713 28025713 + 1 Chr5;19152829;19160826;VANDAL21 m64079_221220_112036/76744176/ccs met1_05

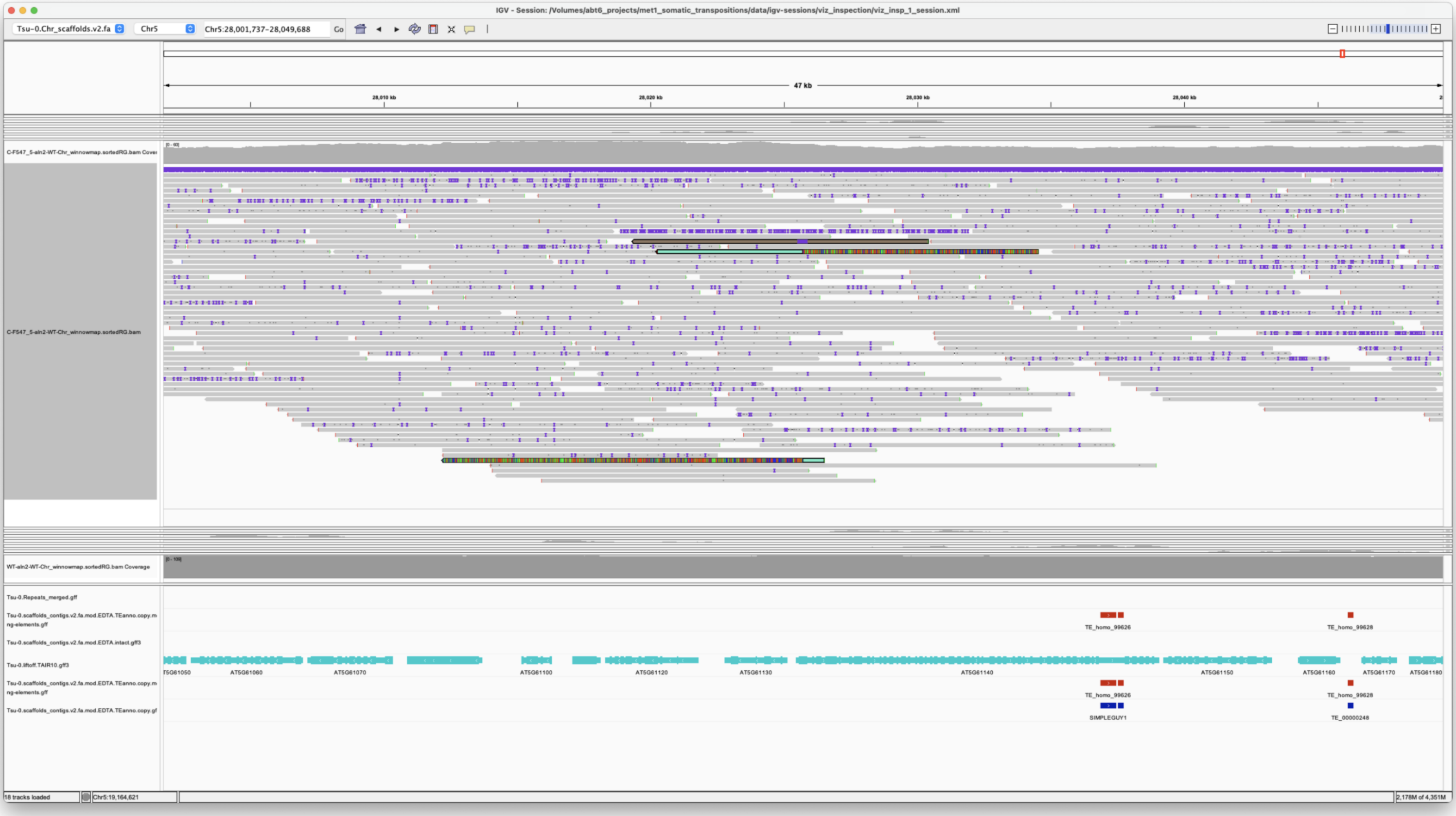

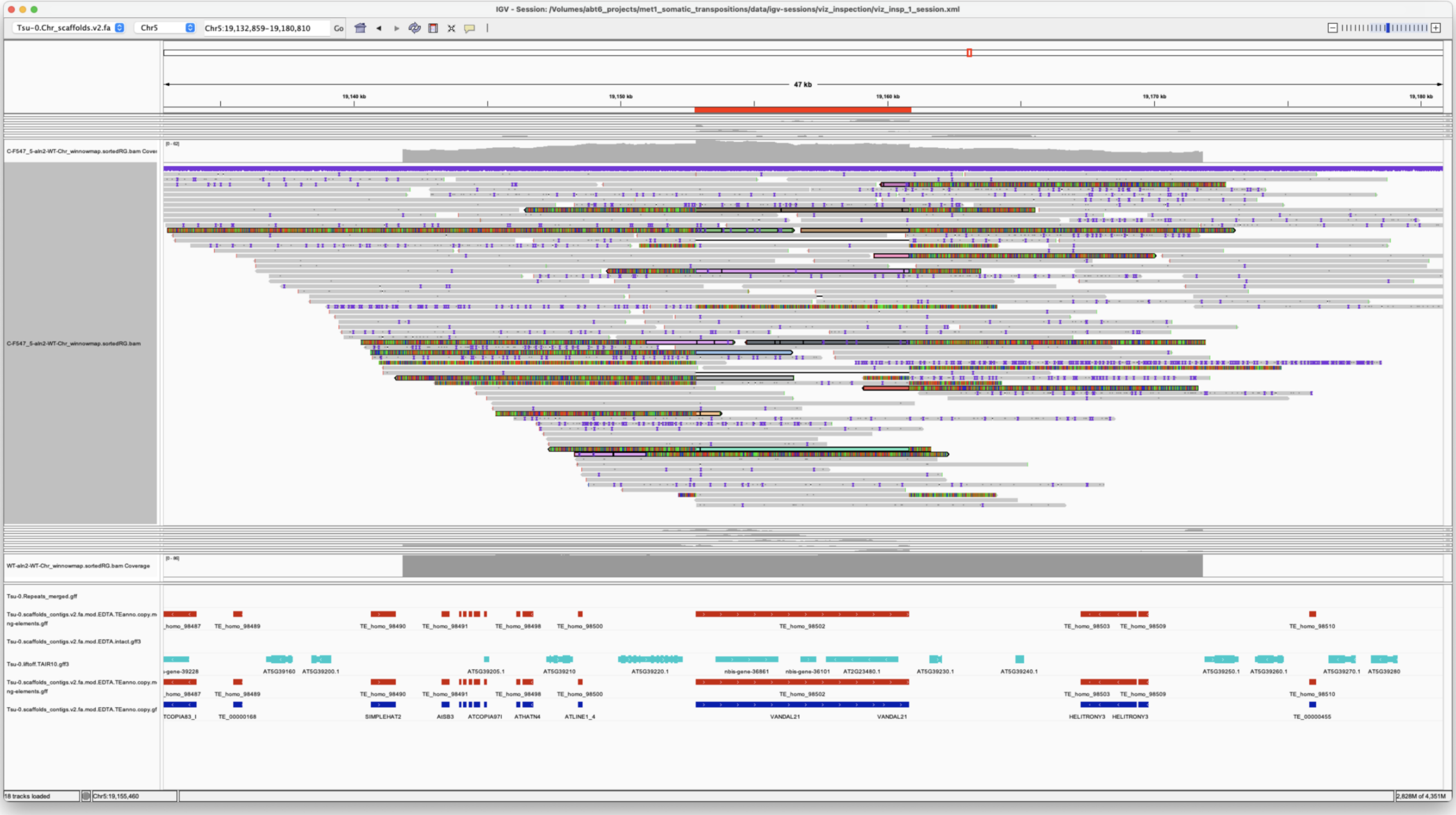

Also m64079_221220_112036/166528667/ccs explains the same insertion. But undetected by other methods 20.05.24

--> Corrected!! Coverage 2X

Central
TSD

Confirmed

Chr5 29885702 29885702 + 1 Chr3;16344522;16352497;VANDAL6 m64079_221220_112036/56953538/ccs met1_05

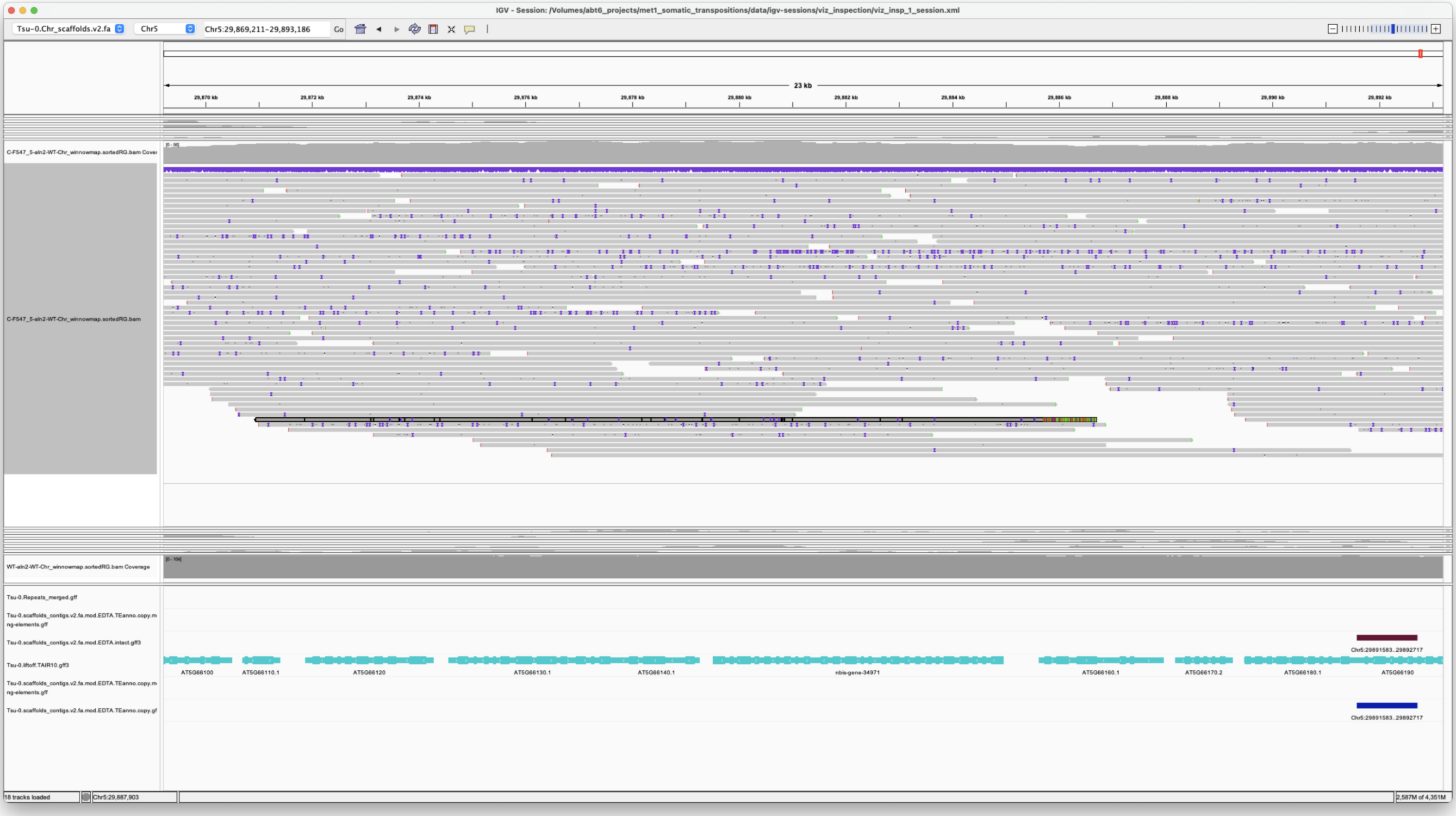

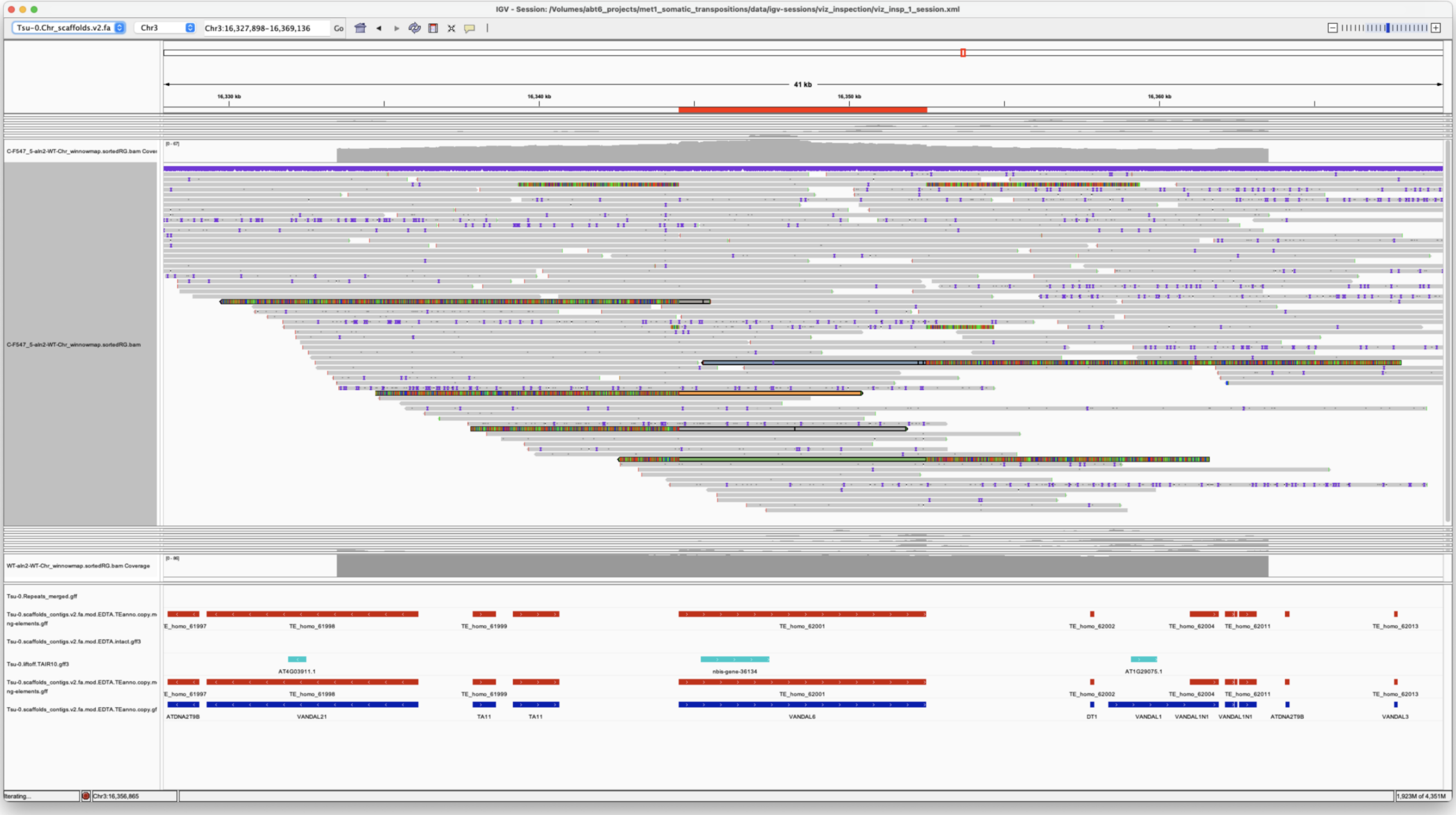

Partial

Confirmed

---

met1_06

> **DIFFICULT CASE TO AUTOMATE: m64079_221220_112036/24053759/ccs 0 3812 Chr3 13665998 13669810 10237 - ATLANTYS1_LTR m64079_221220_112036/24053759/ccs 3811 9141 Chr1 11941106 11946436 10237 + ATCOPIA93.2_Evade m64079_221220_112036/24053759/ccs 9141 10236 Chr3 13664909 13666004 10237 - ATLANTYS1_LTR**

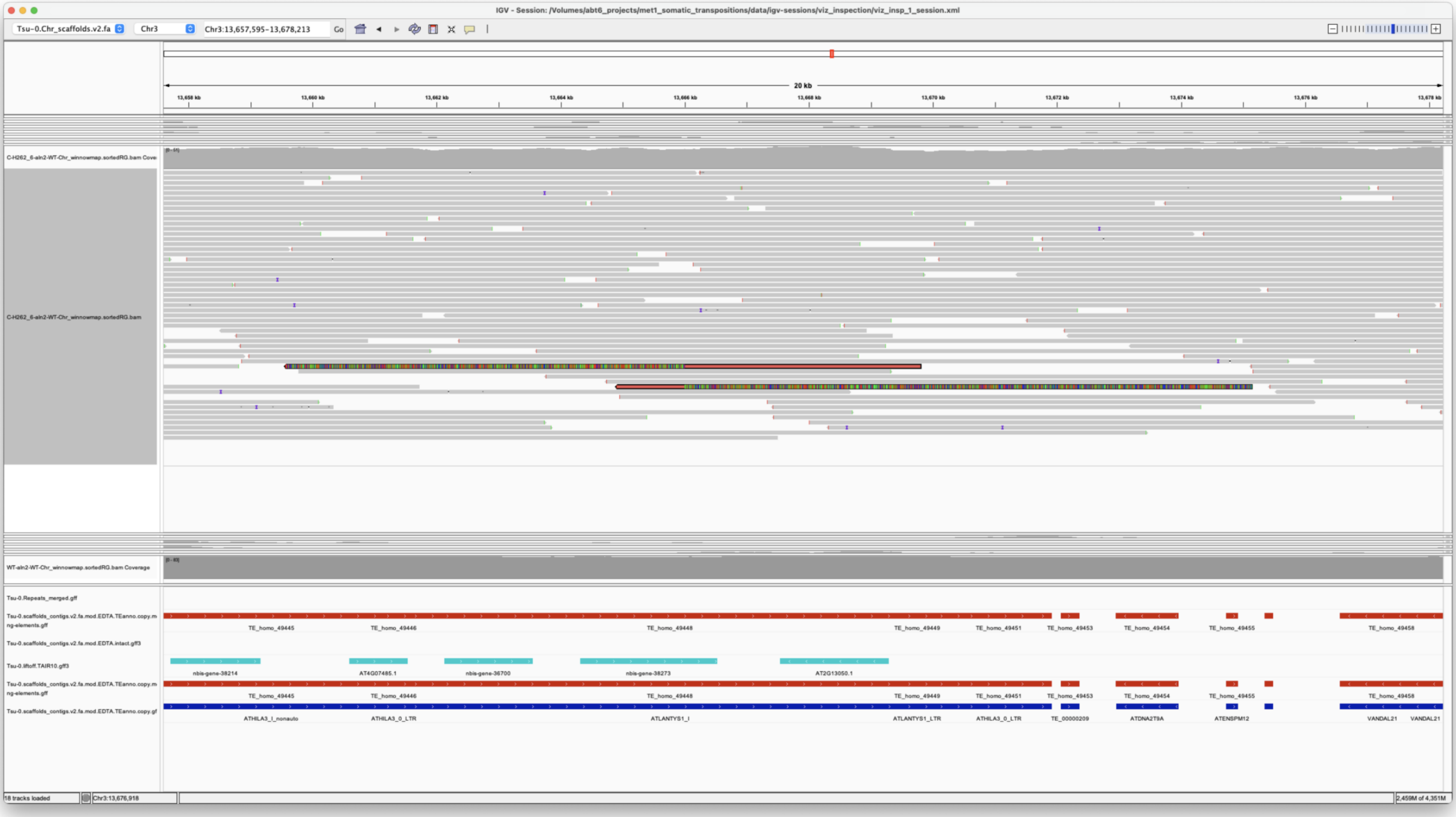

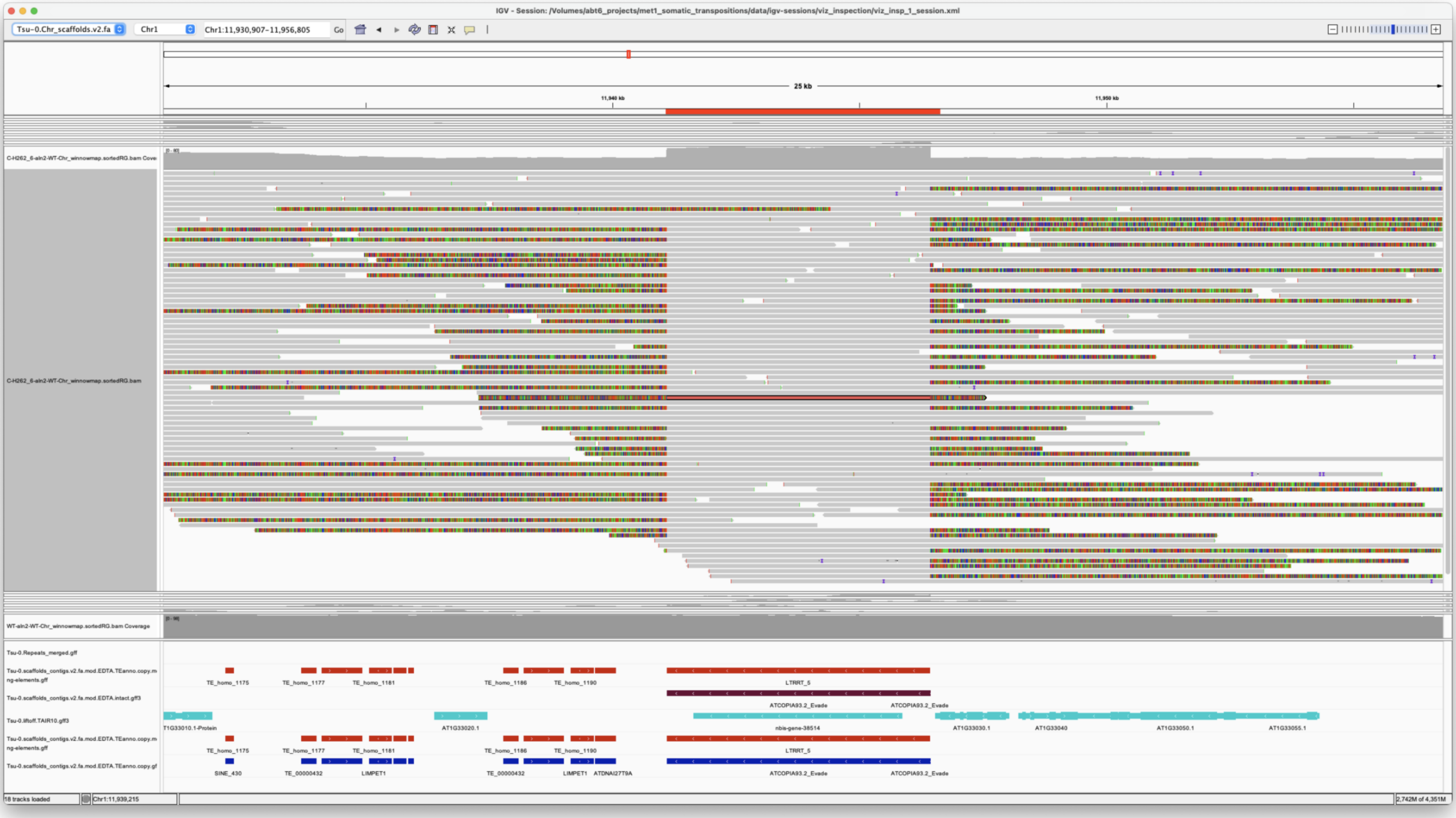

EVD insertion!

Central
TSD

Confirmed

Chr1 375331 375331 + 1 Chr5;19152829;19160826;VANDAL21 m64079_240212_113350/30476632/ccs met1_06

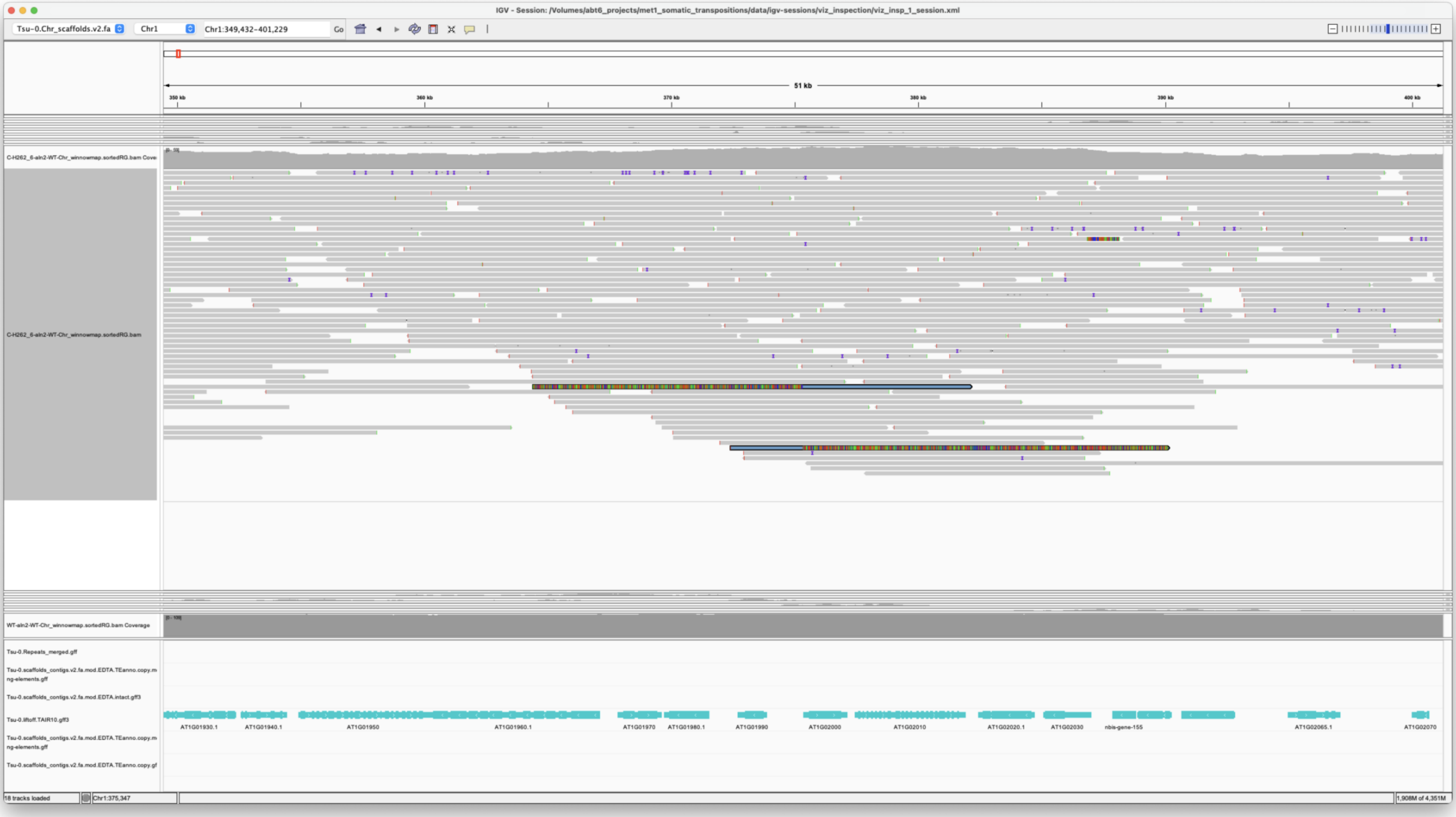

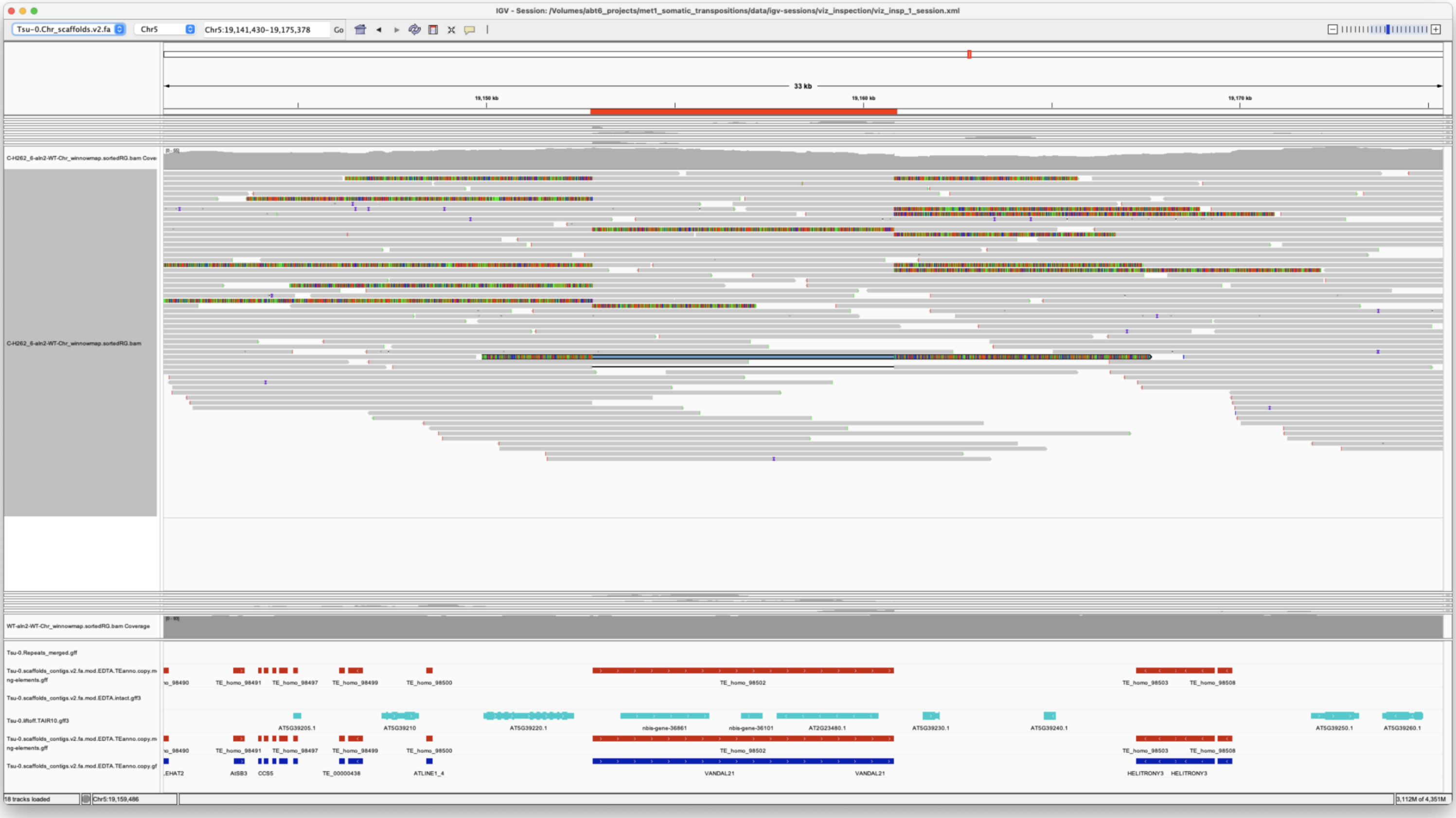

Central
TSD

Confirmed

Chr1 12724218 12724218 + 1 Chr3;16344522;16352497;VANDAL6 m64079_240212_113350/109709174/ccs met1_06

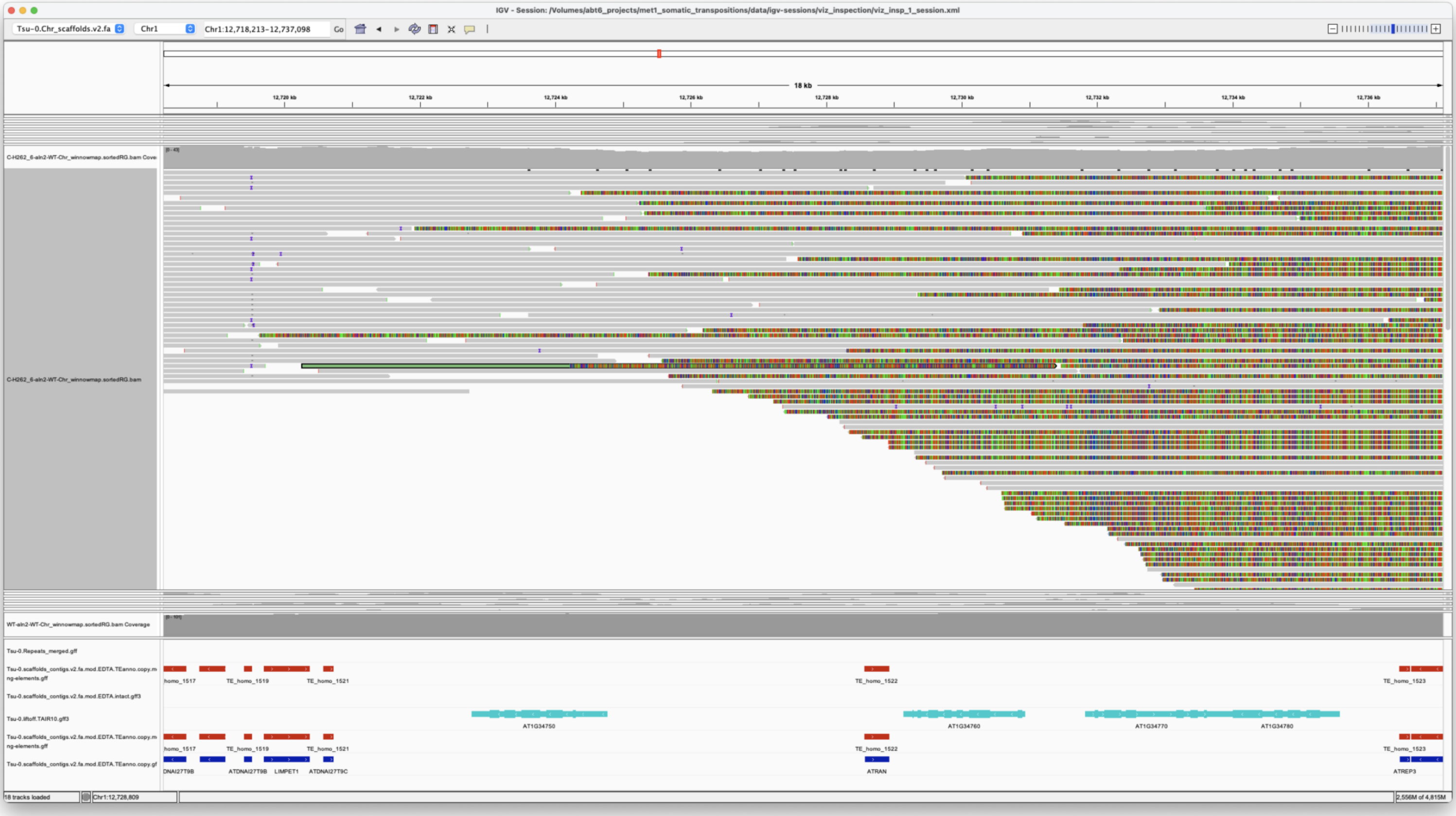

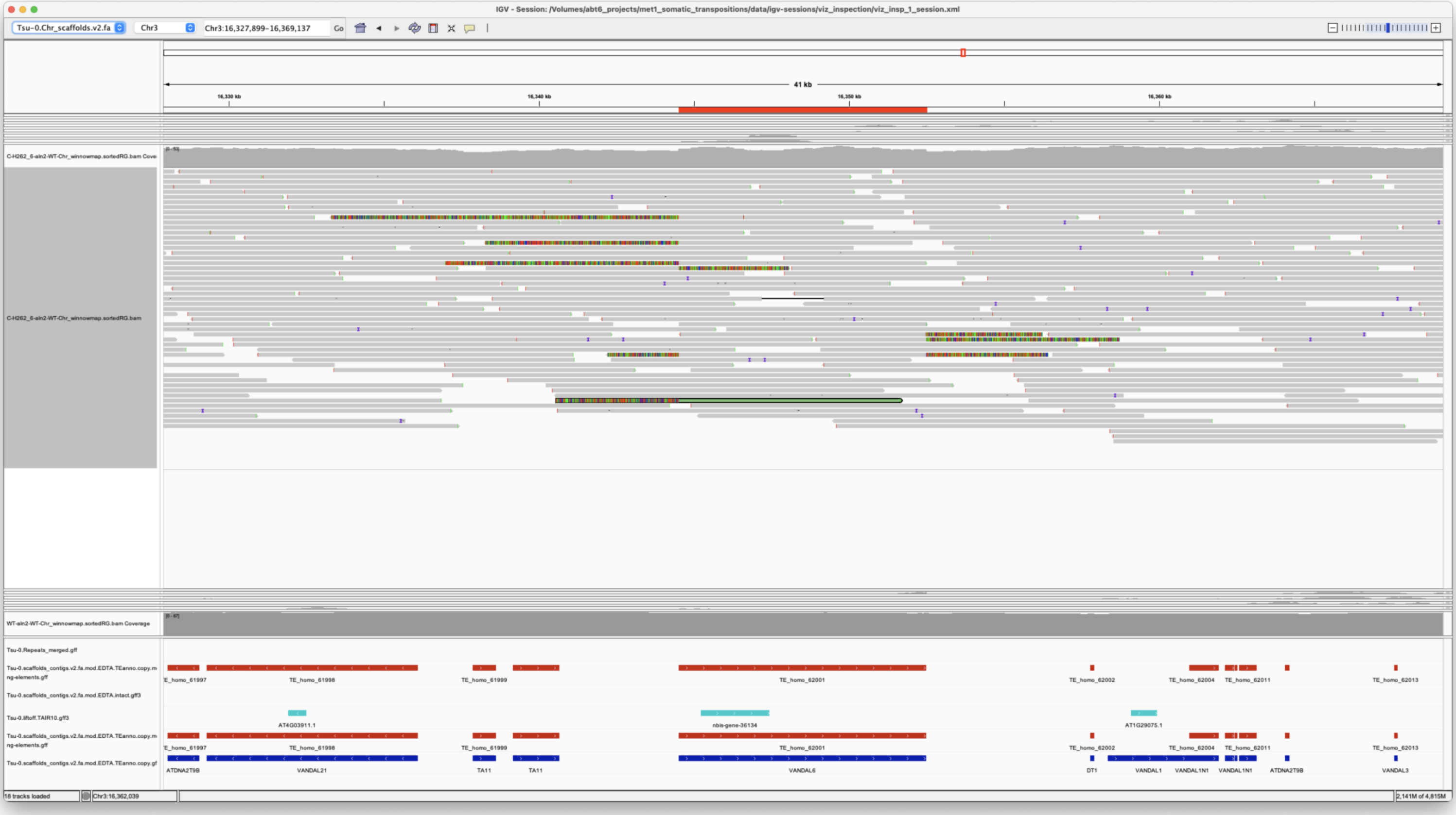

Next to Chloroplast insertion but seems ok

Partial

Confirmed

> Chr1 16161461 16161461 + 1 Chr5;19152829;19160826;VANDAL21 m64079_221220_112036/112133071/ccs met1_06

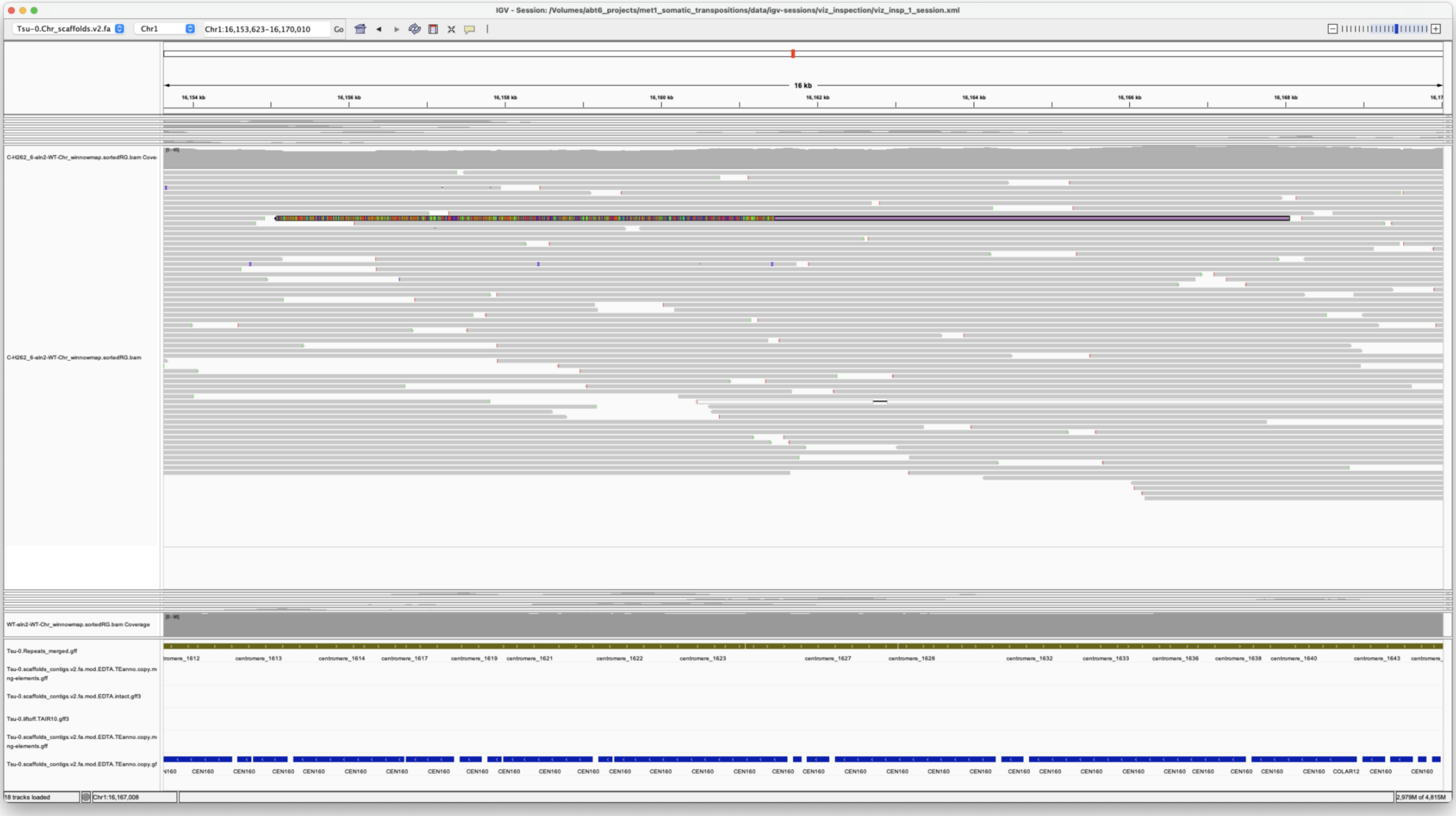

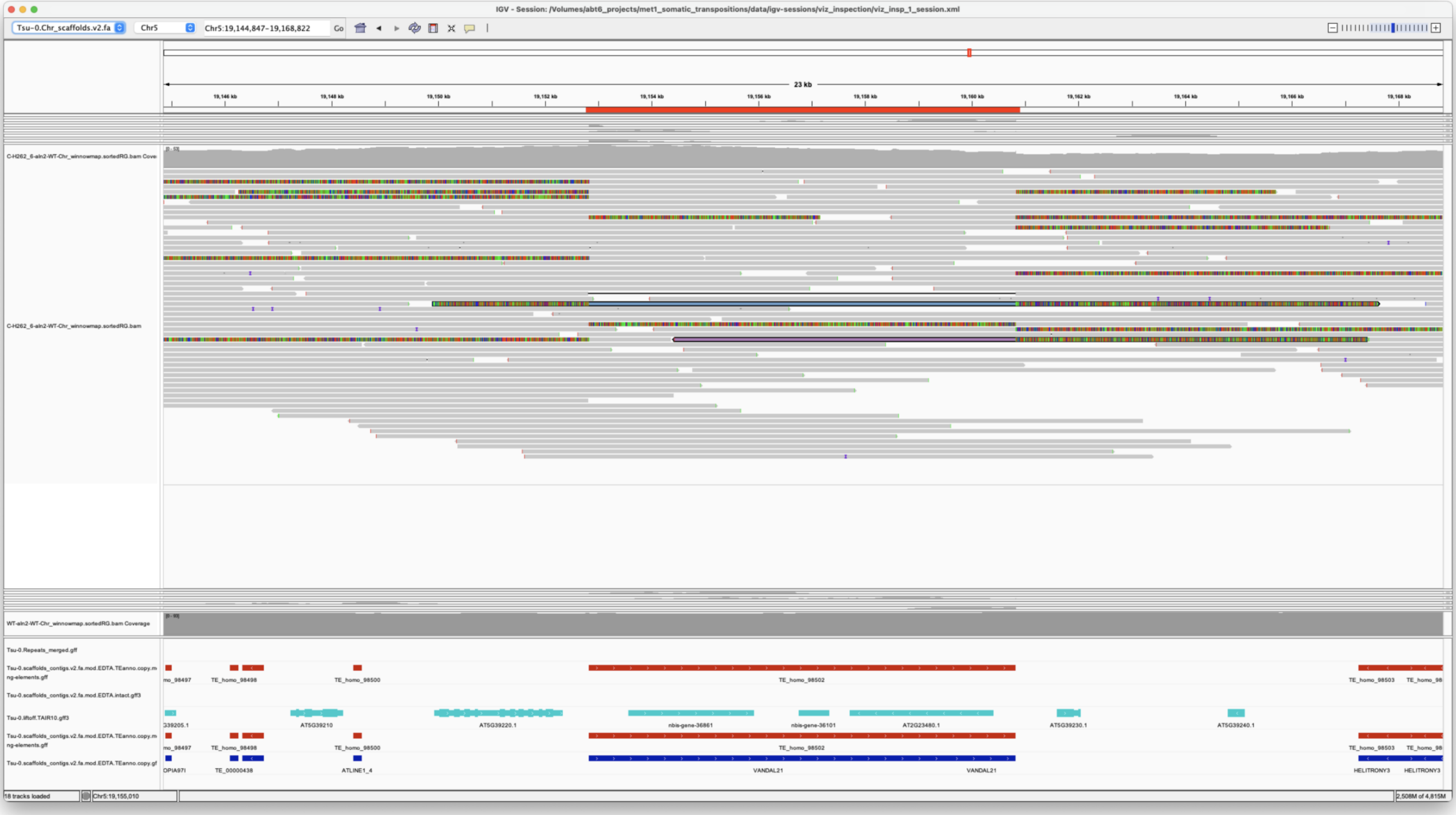

Centromeric

Partial

Confirmed

> Chr1 24282152 24282152 + 1 Chr2;8027483;8035680;VANDAL21 m64079_221220_112036/73794588/ccs met1_06

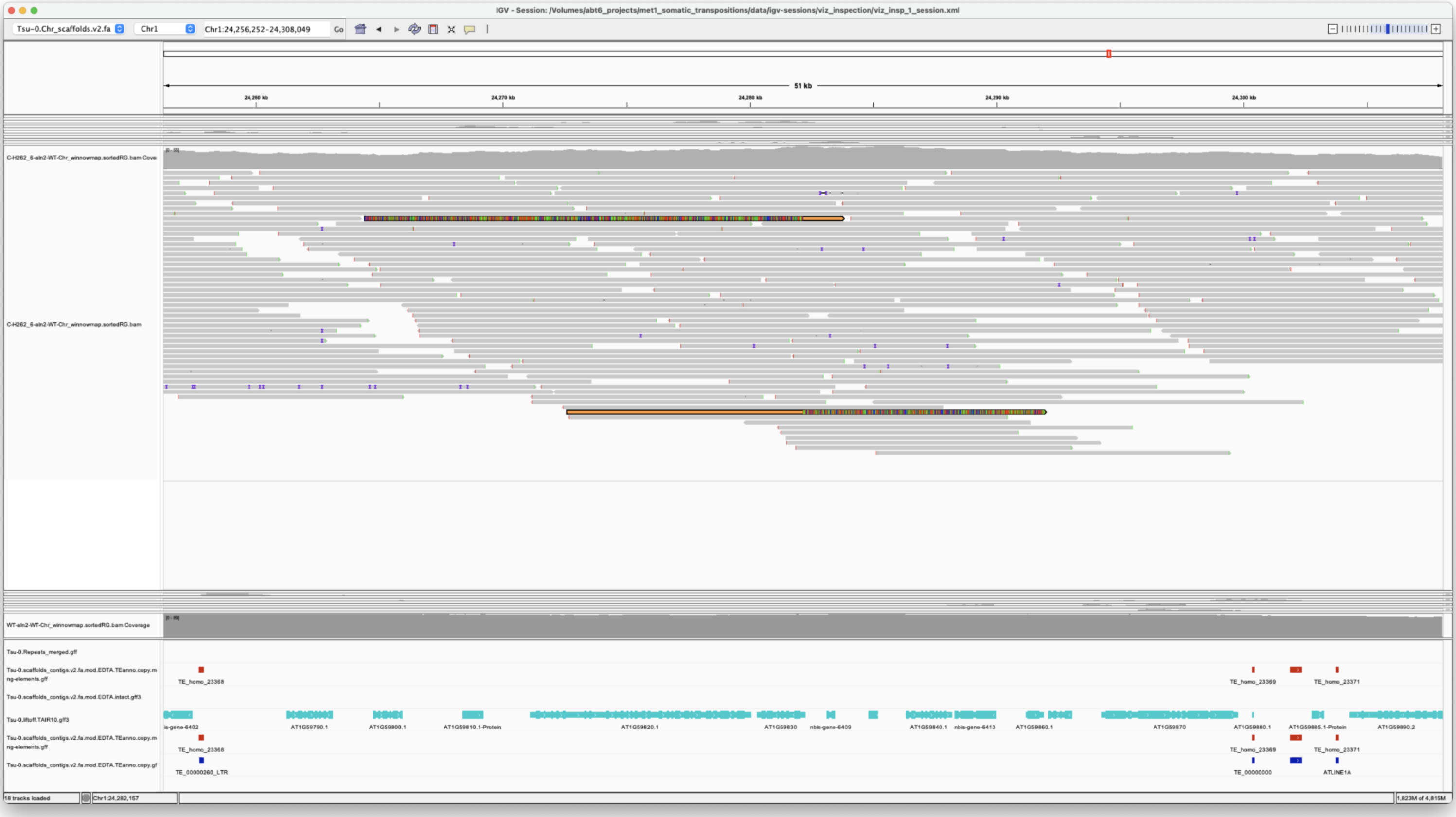

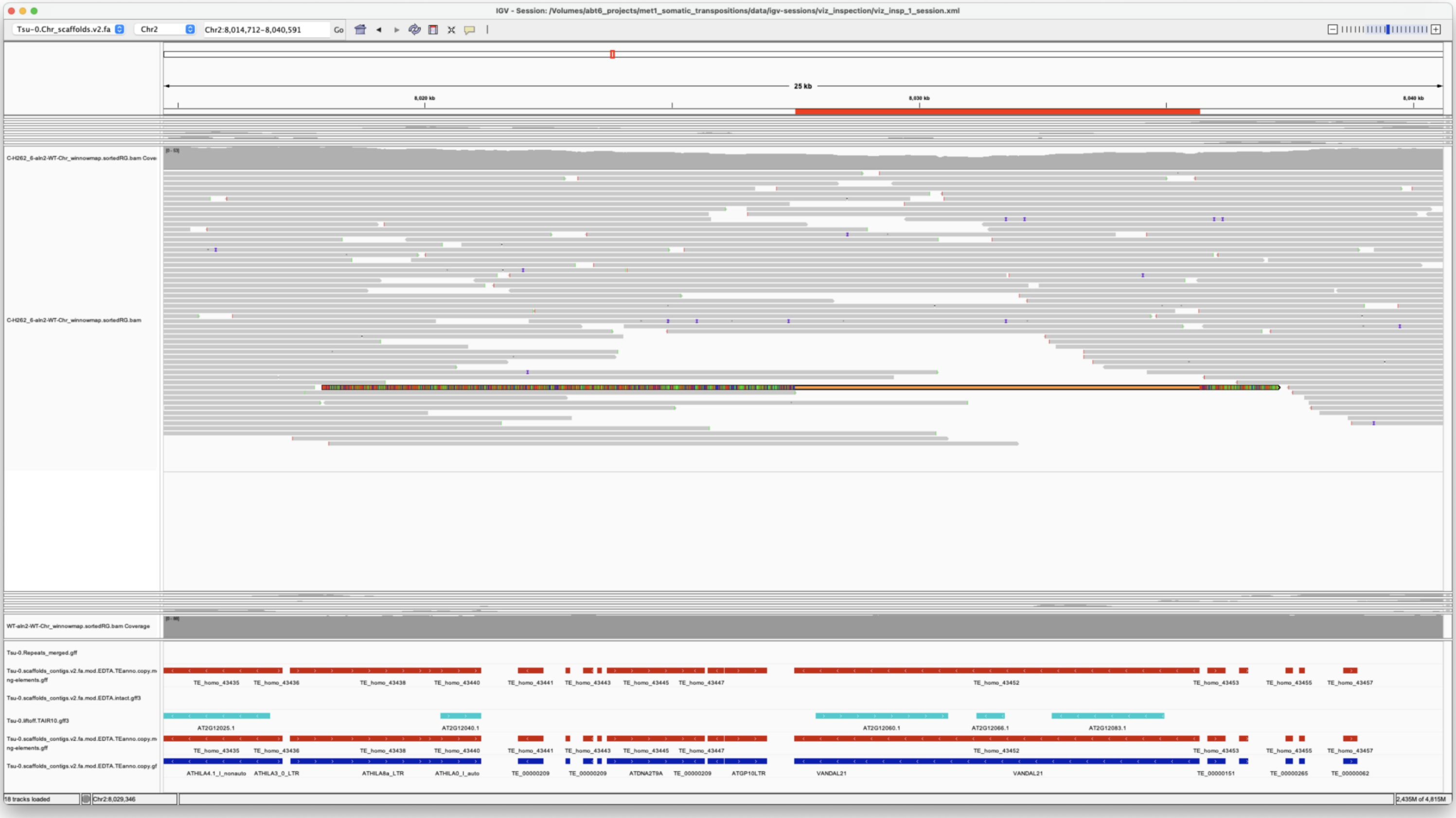

First instance of this TE so far

Partial

Confirmed

Chr1 26983849 26983849 + 1 Chr1;11941106;11946436;ATCOPIA93_Evade m64079_221220_112036/107217507/ccs met1_06

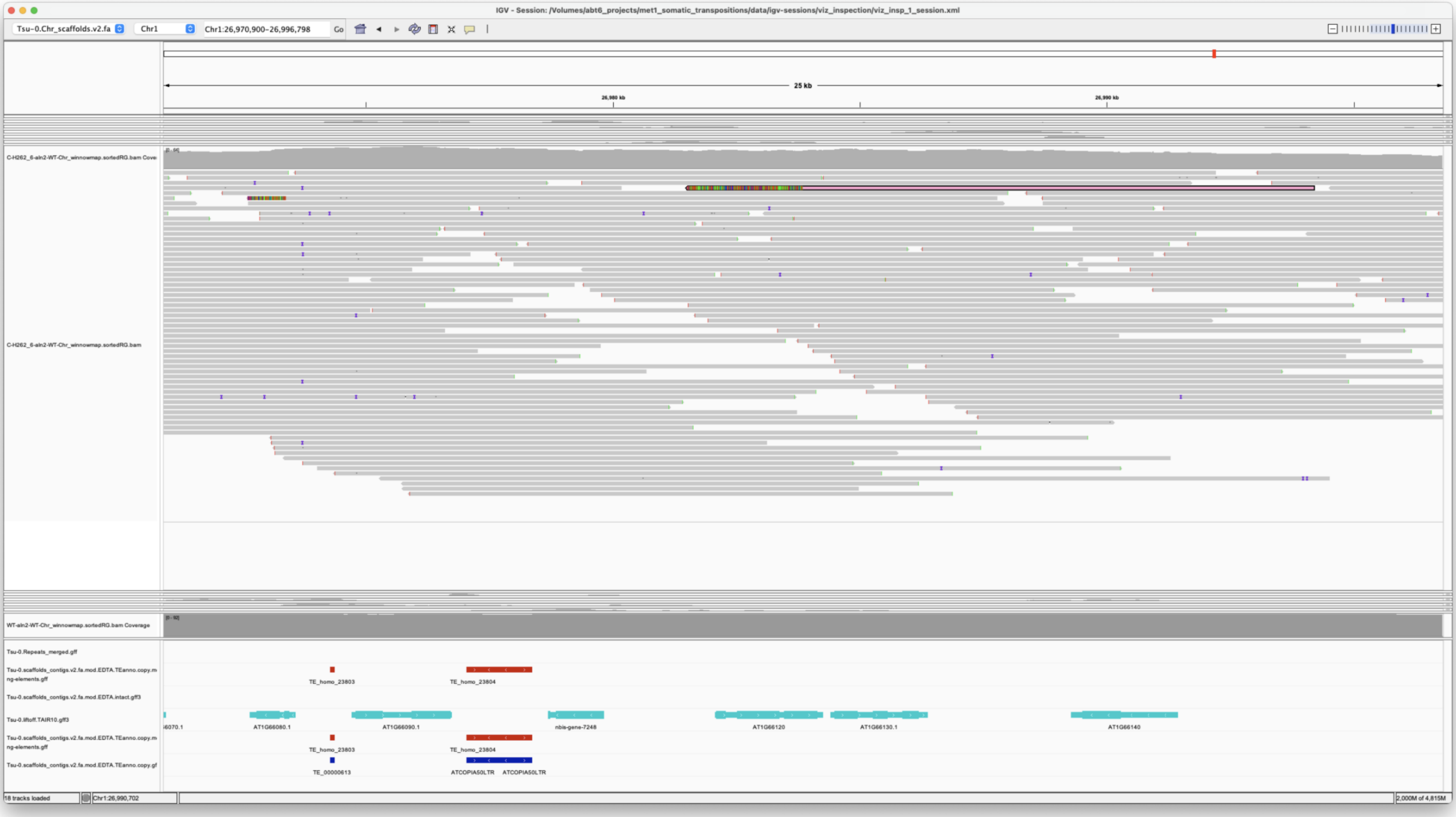

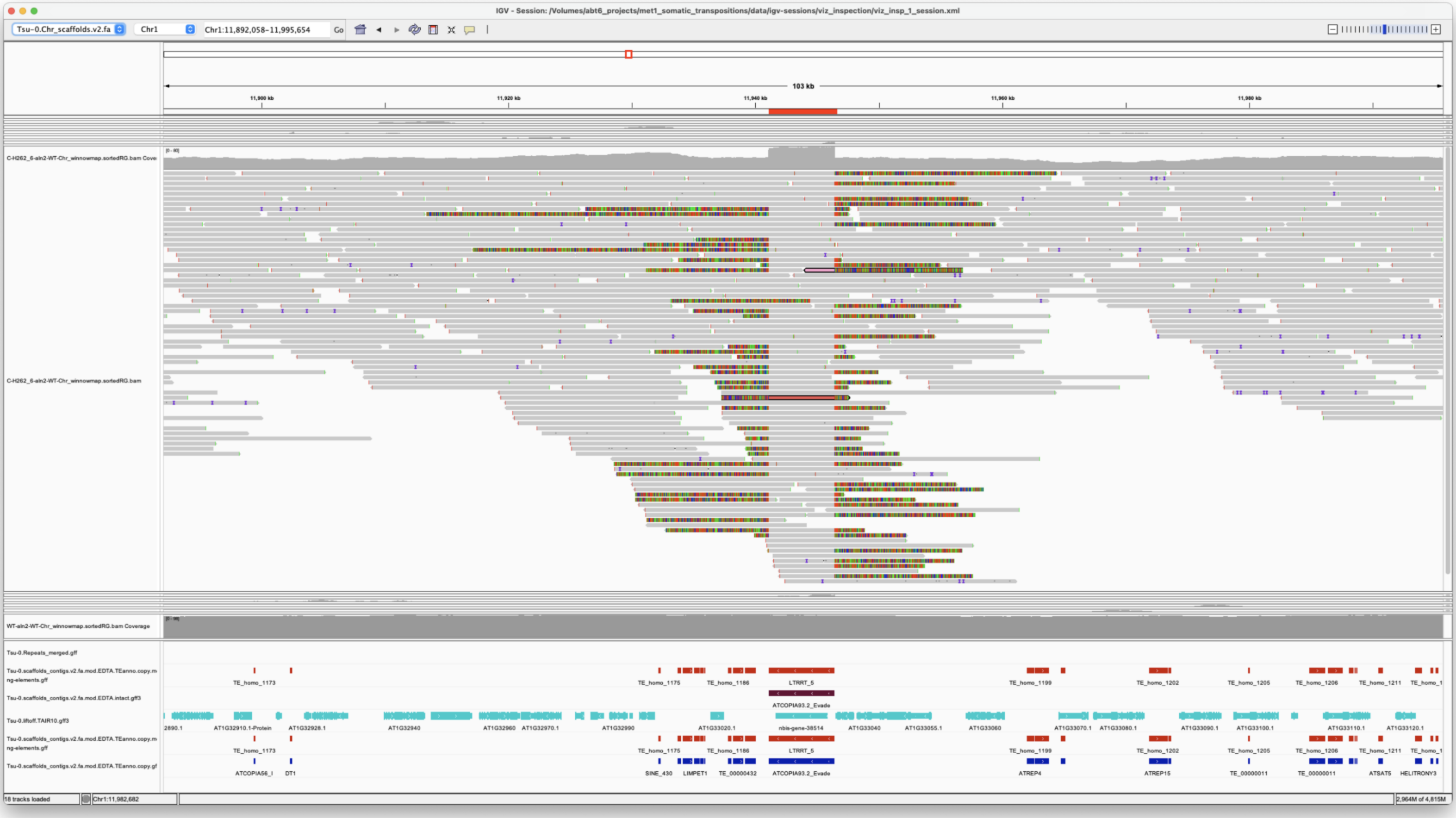

Partial

Confirmed

Chr1 27783933 27783933 + 1 Chr1;11941106;11946436;ATCOPIA93_Evade m64079_221220_112036/149487936/ccs met1_06

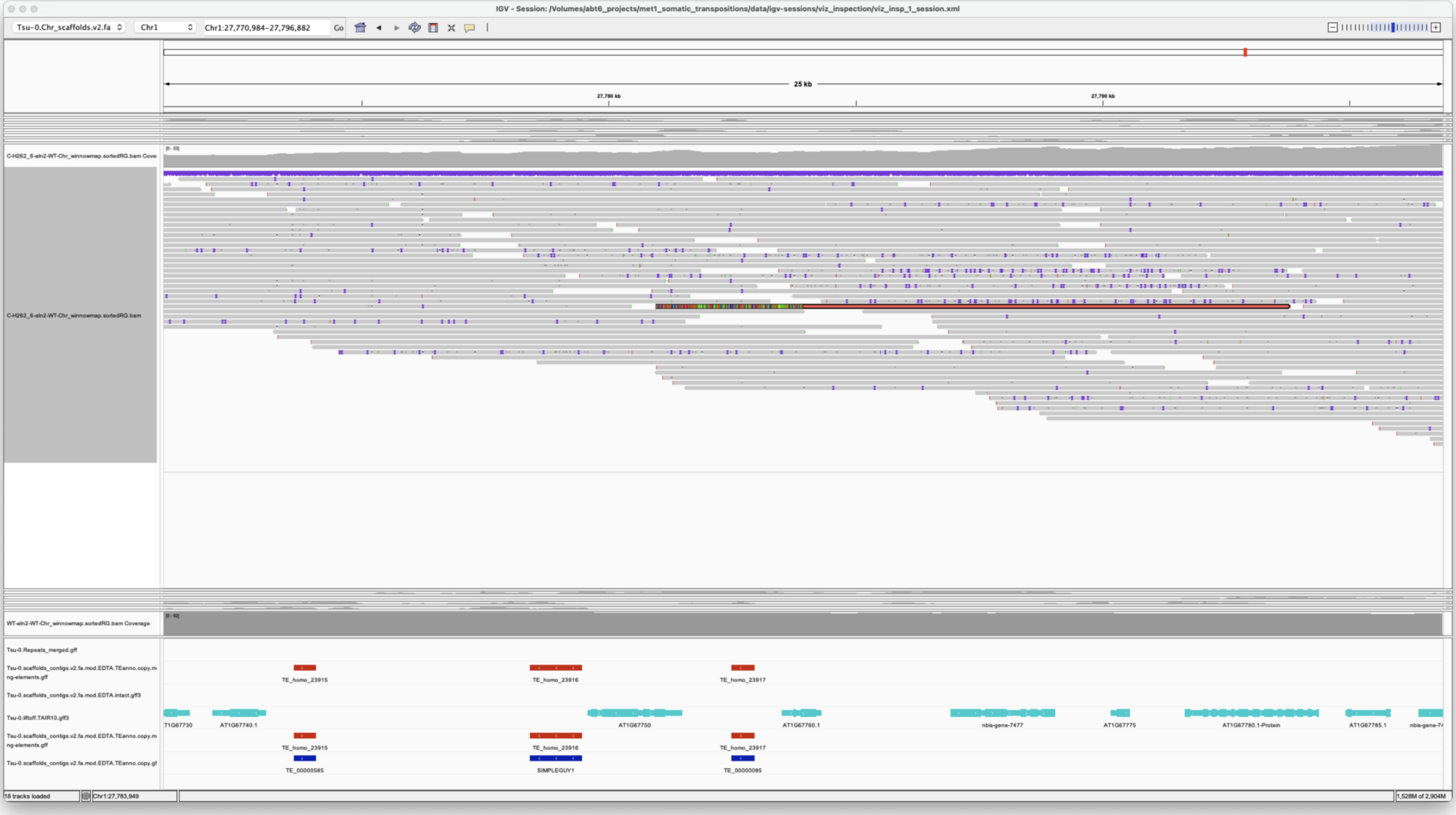

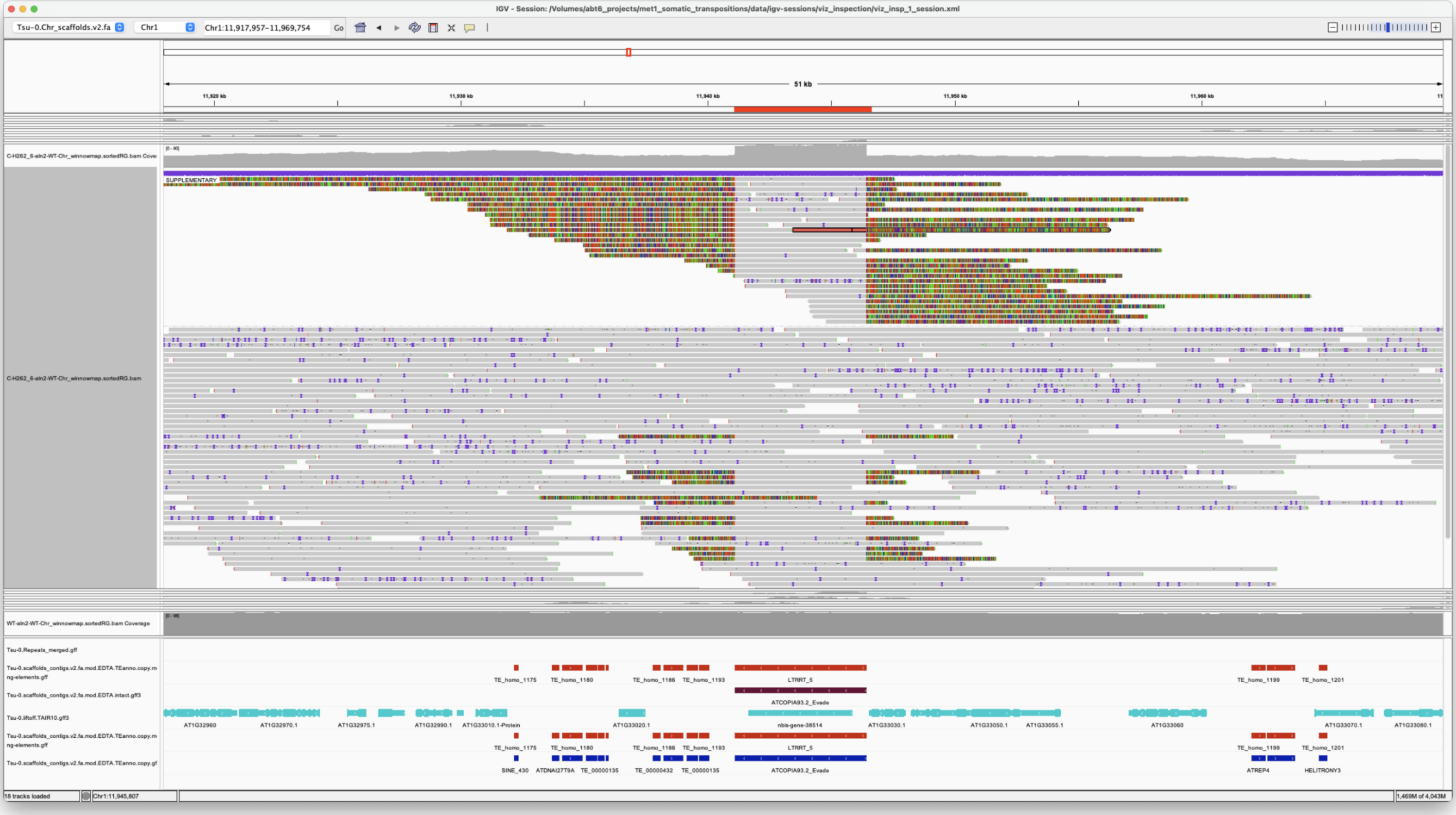

Partial

Confirmed

> Chr1 29612263 29612263 + 1 Chr1;11941106;11946436;ATCOPIA93_Evade m64079_221220_112036/91359146/ccs met1_06

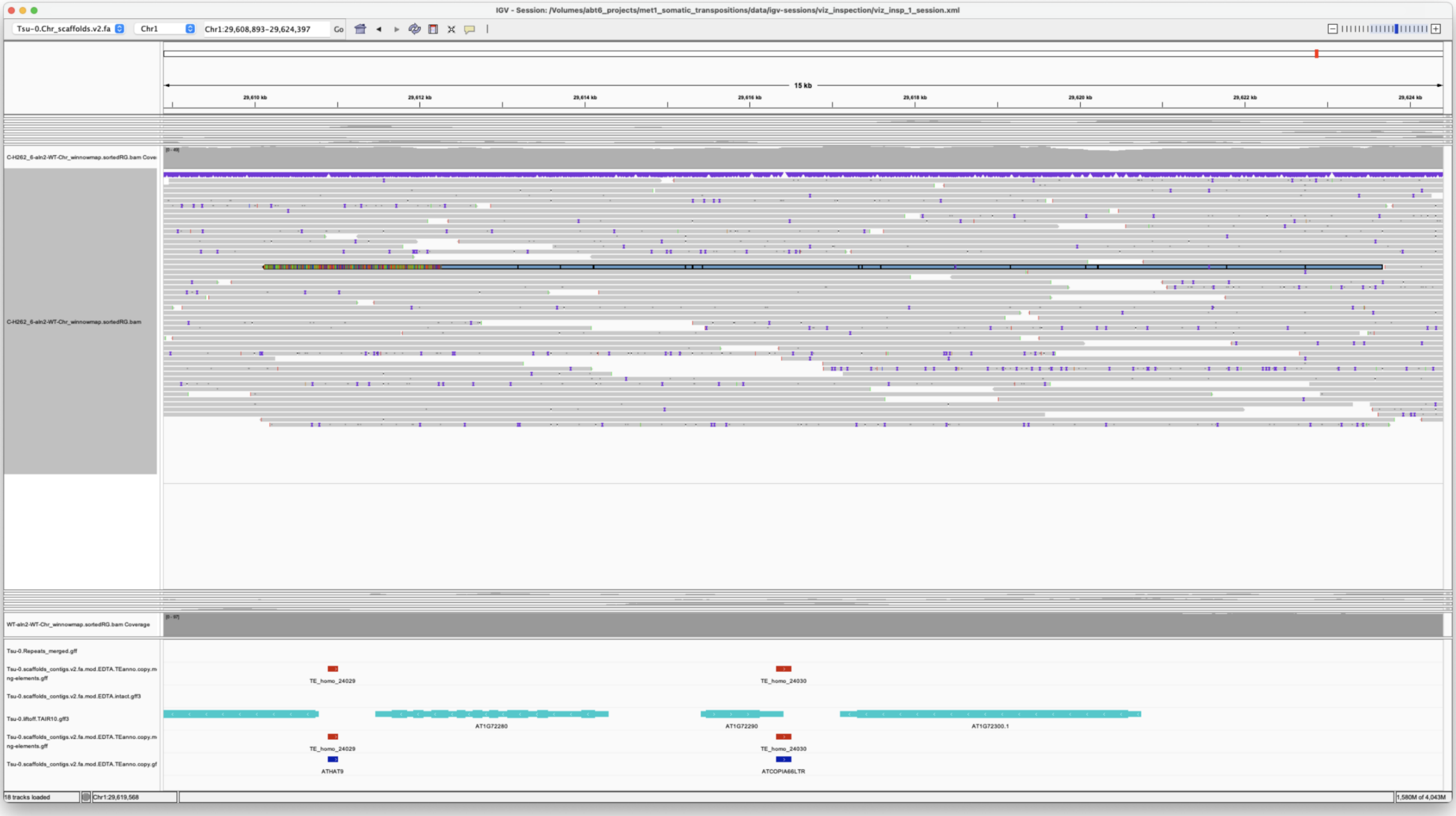

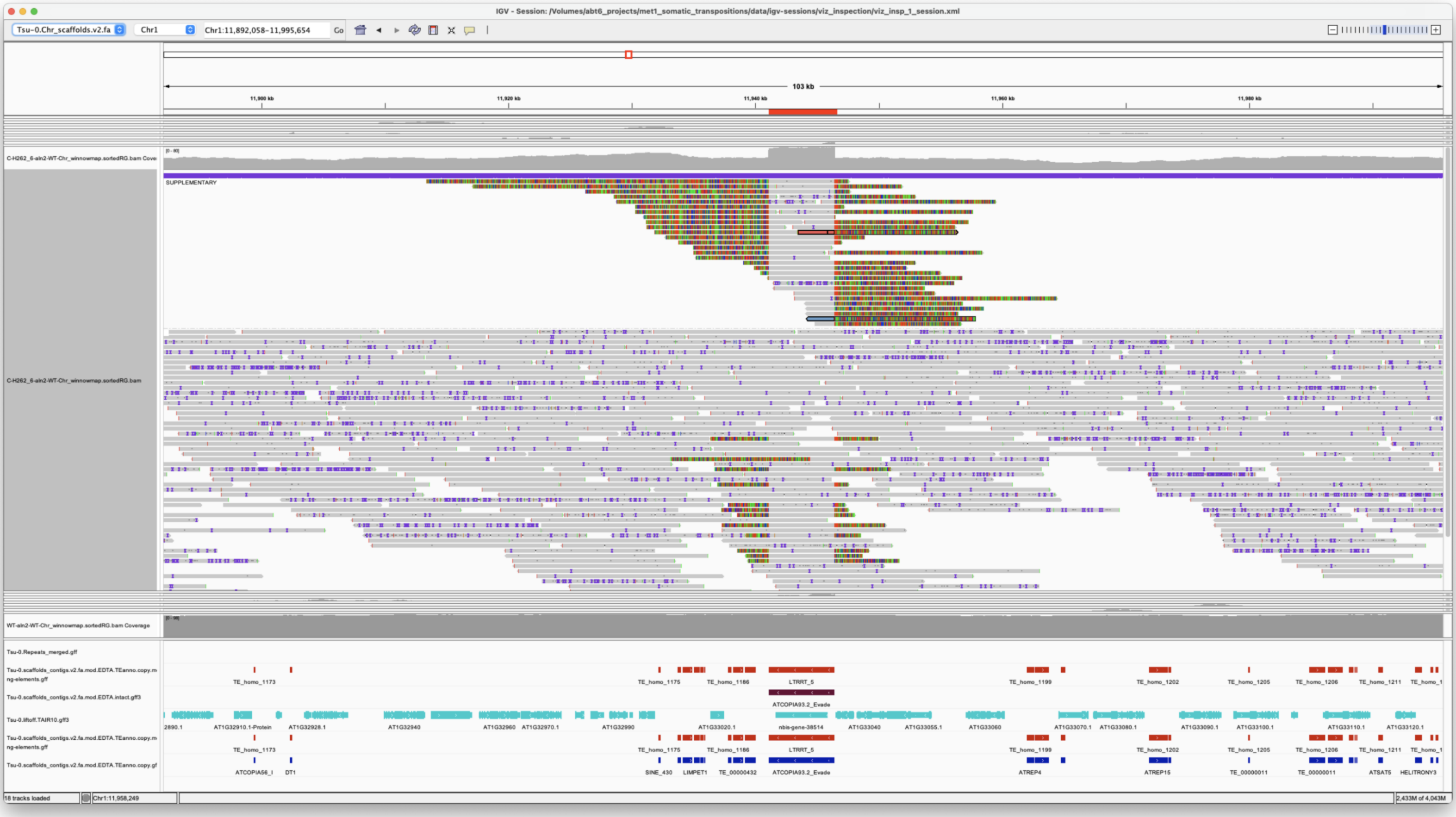

Partial

Confirmed

Chr1 31972454 31972454 - 1 Chr5;875414;876434;PAC m64079_240212_113350/82839561/ccs met1_06

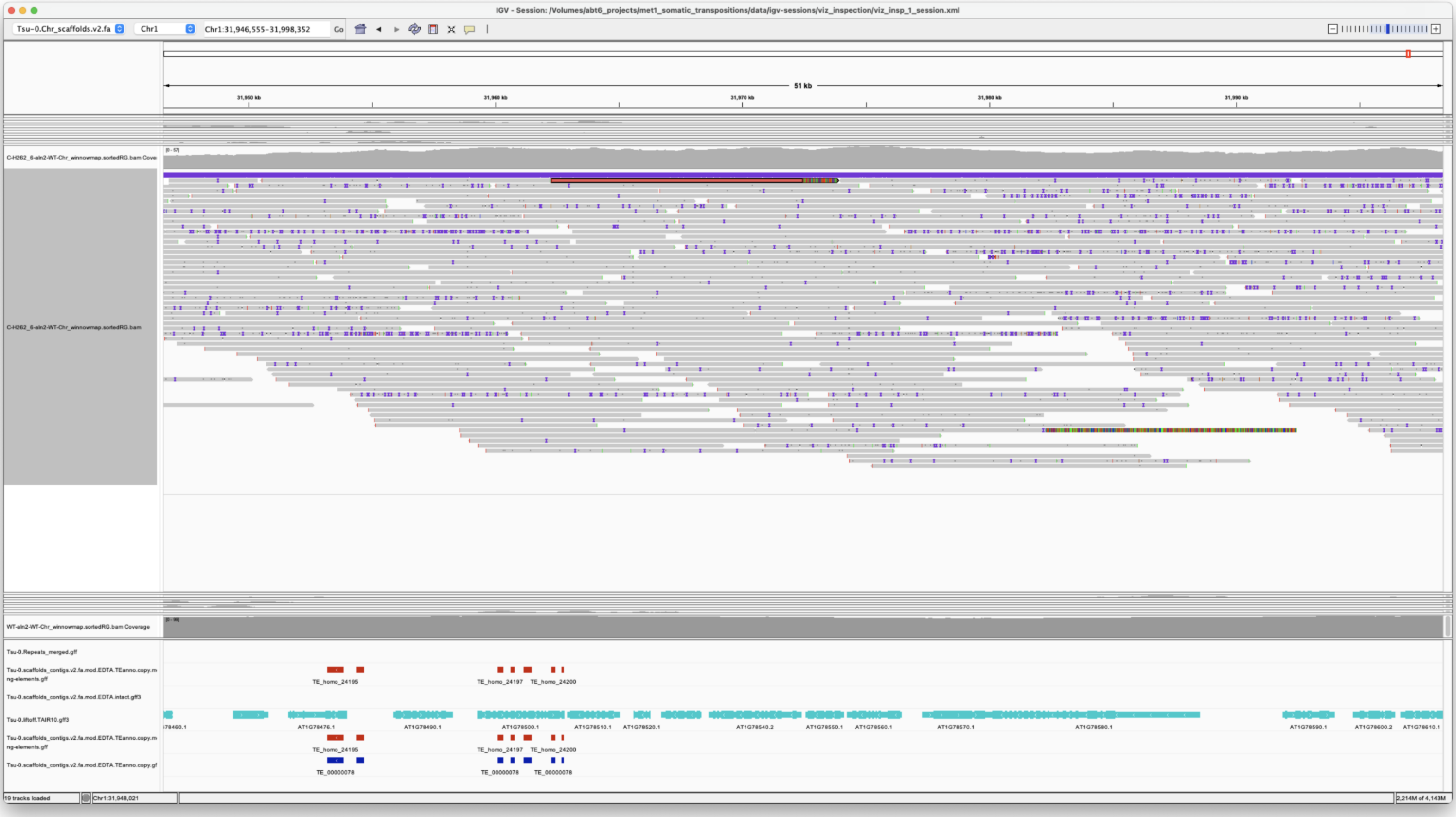

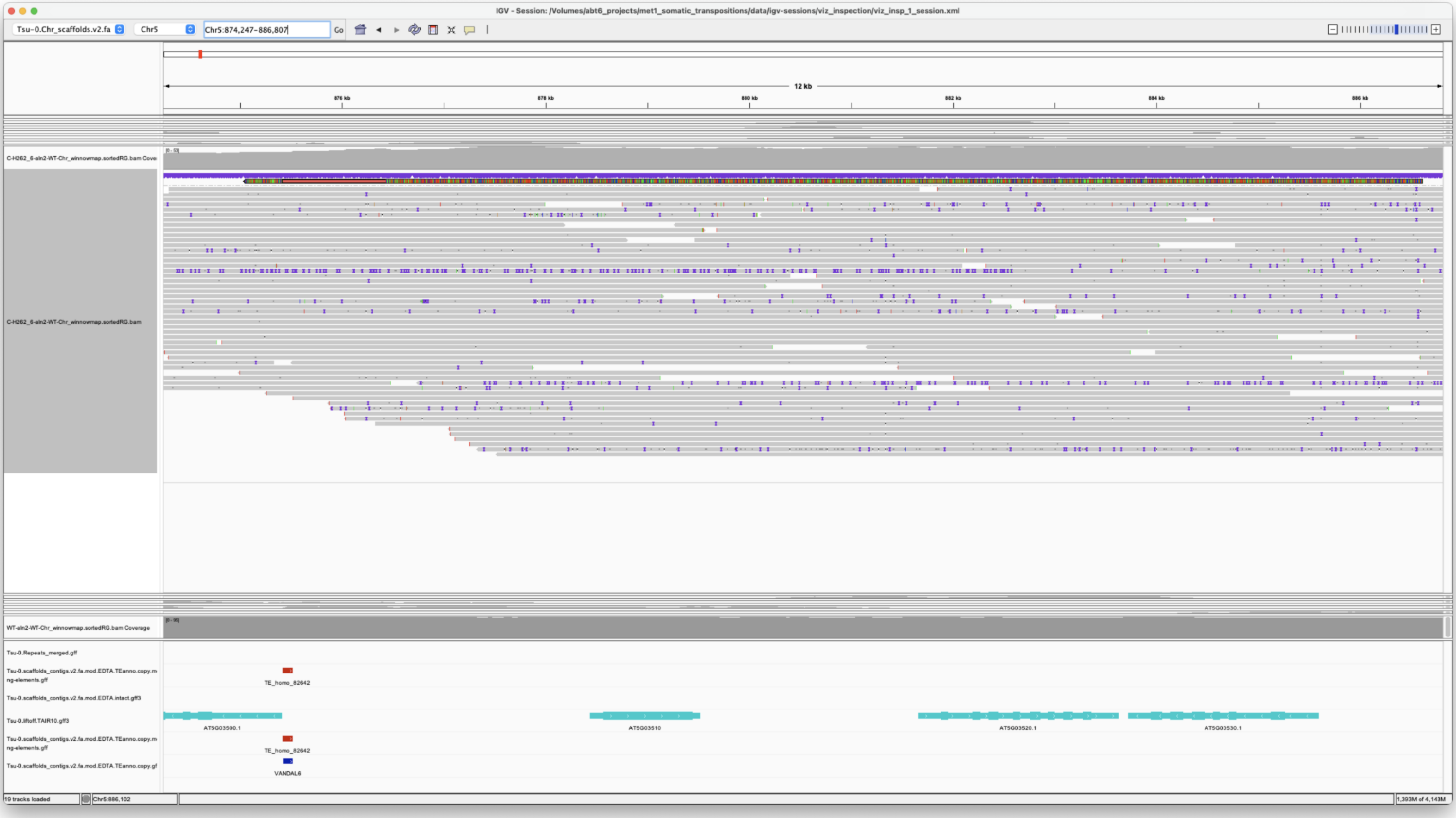

Annotation not present in the bottom tracks -> PAC

Partial

Confirmed

Chr2 1551971 1551971 + 1 Chr5;19152829;19160826;VANDAL21 m64079_221220_112036/157550110/ccs met1_06

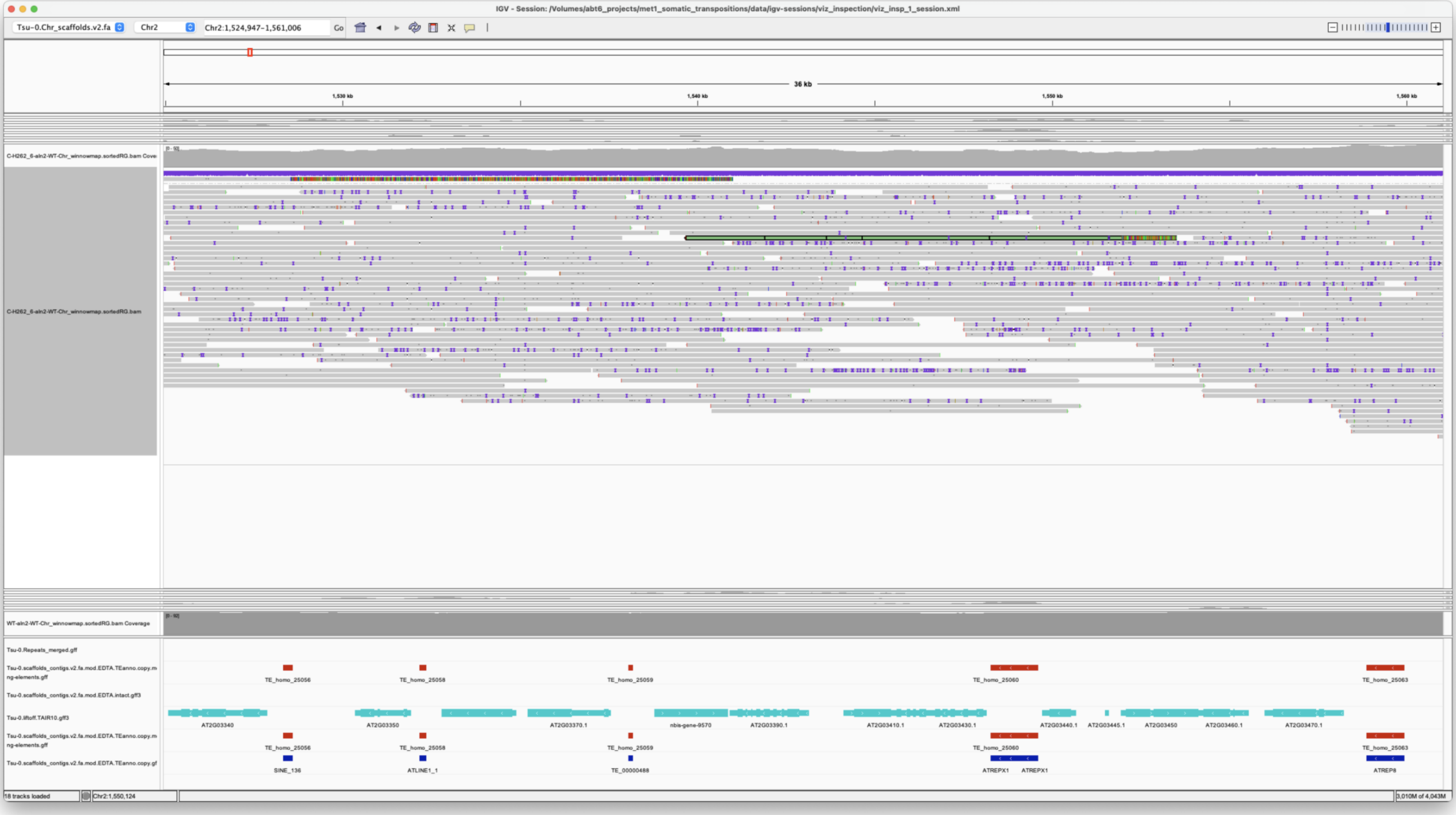

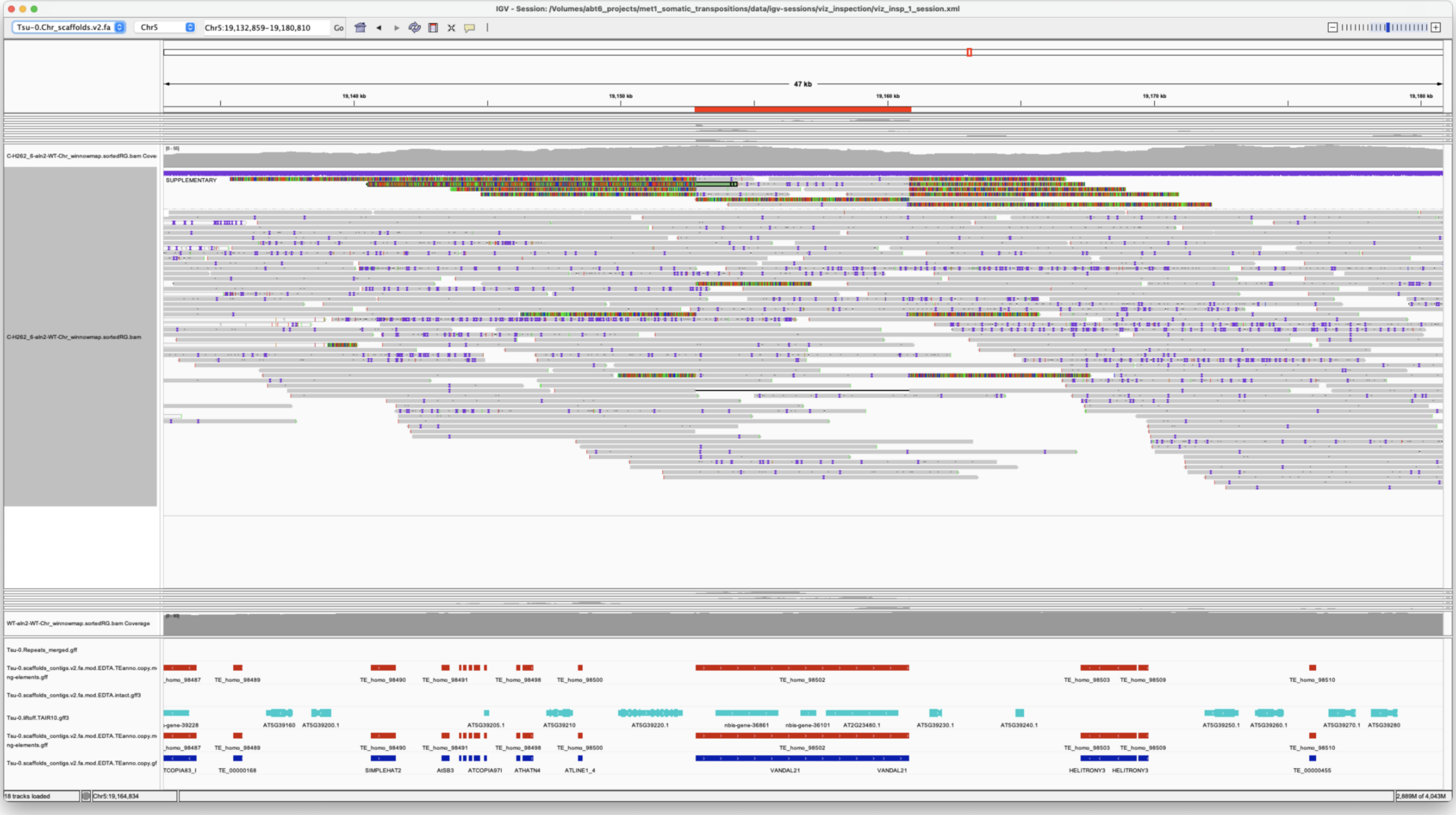

Partial

Confirmed

Chr2 8361870 8361870 - 1 Chr5;19152829;19160826;VANDAL21 m64079_221220_112036/122029913/ccs met1_06

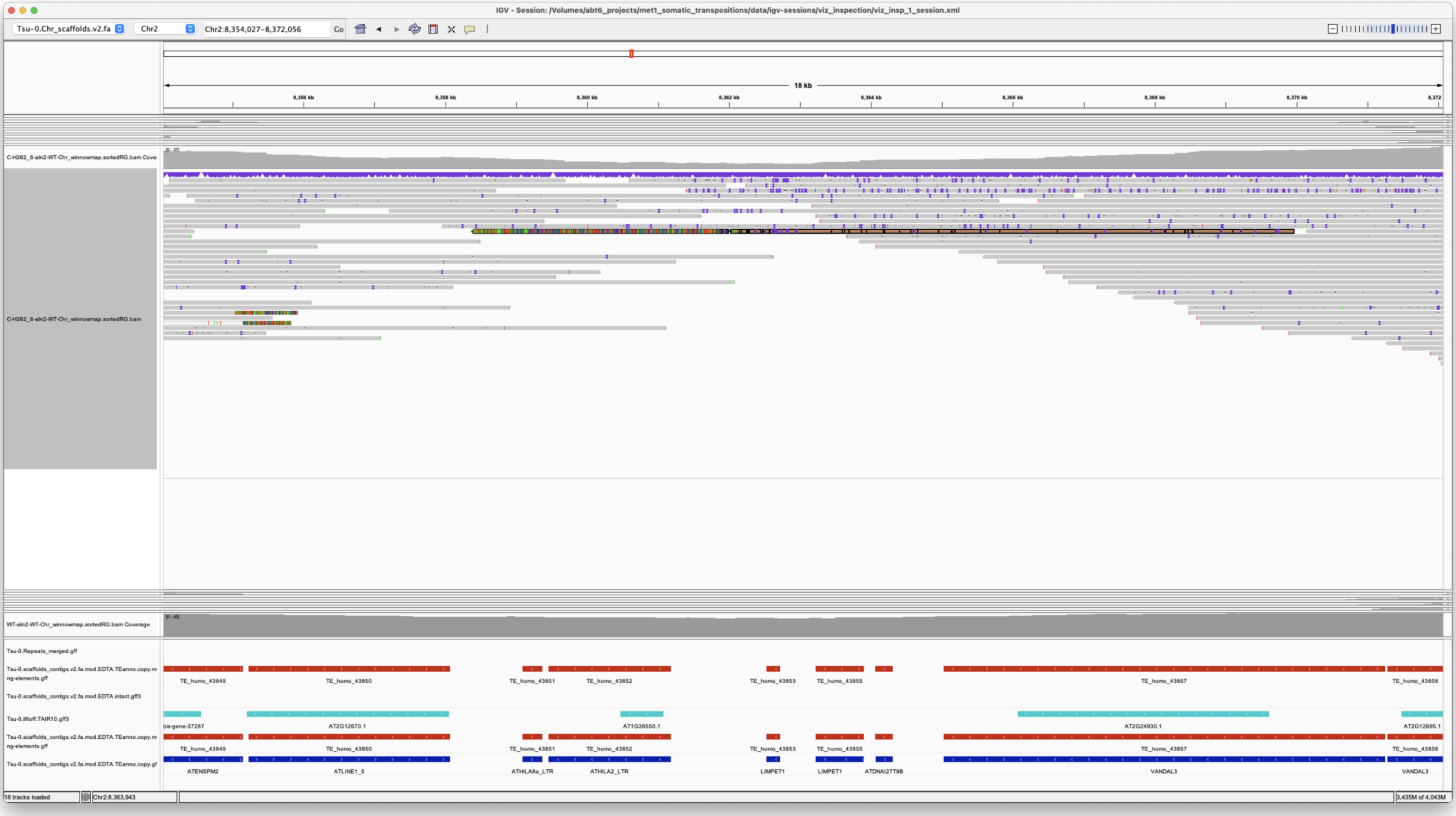

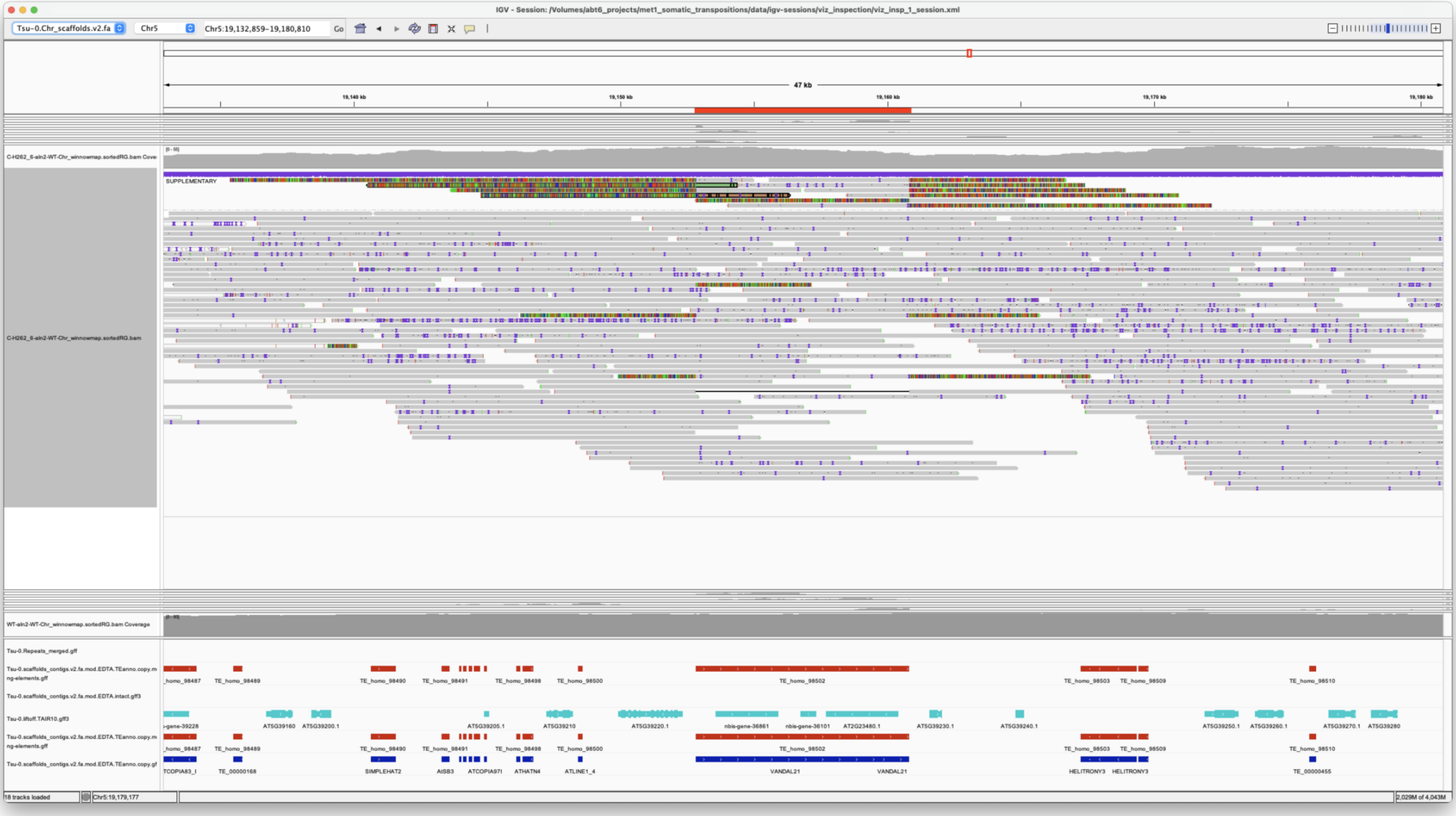

Partial

Confirmed

Chr2 8762630 8762630 - 1 Chr5;19152829;19160826;VANDAL21 m64079_221220_112036/21300945/ccs met1_06

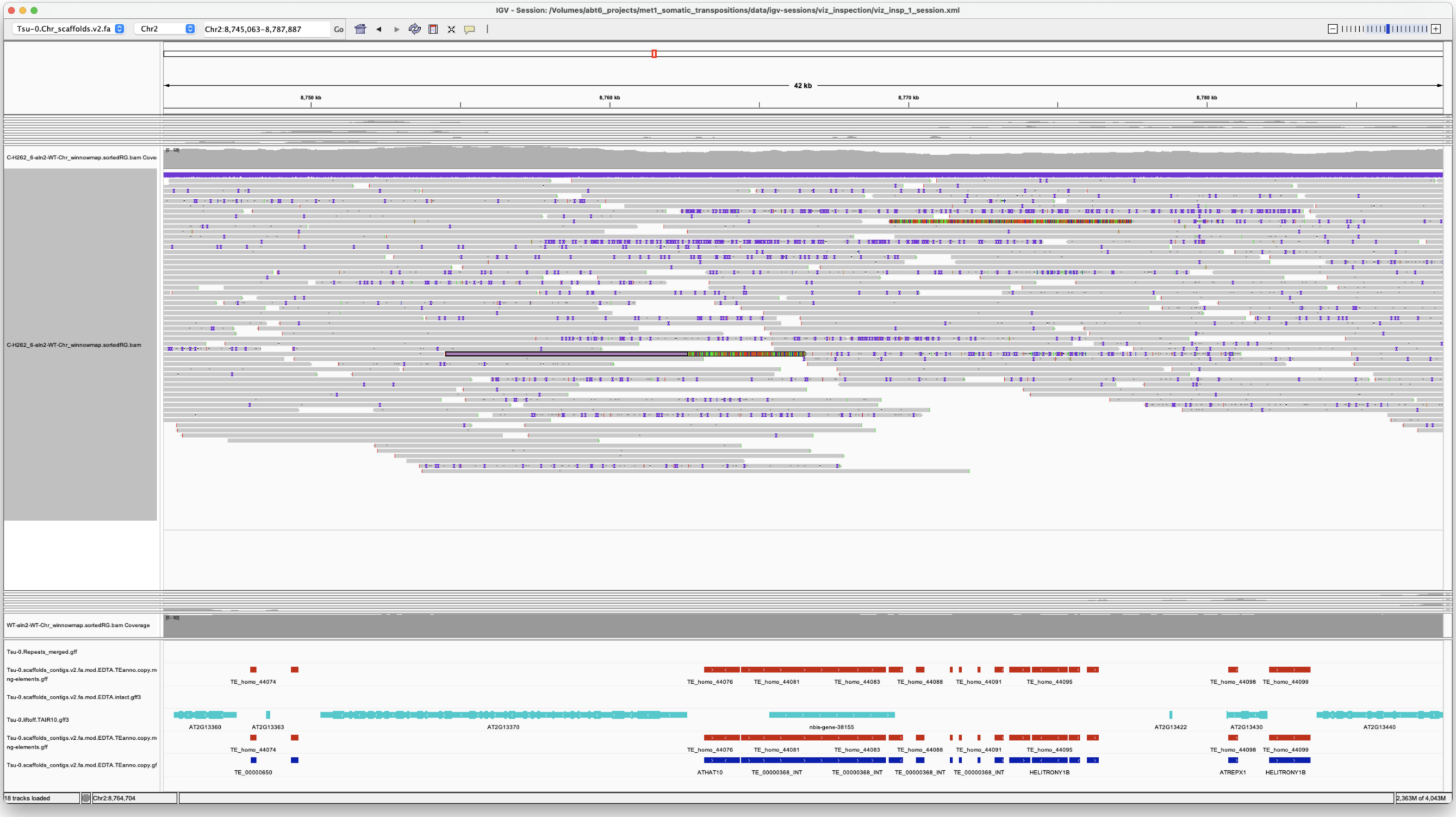

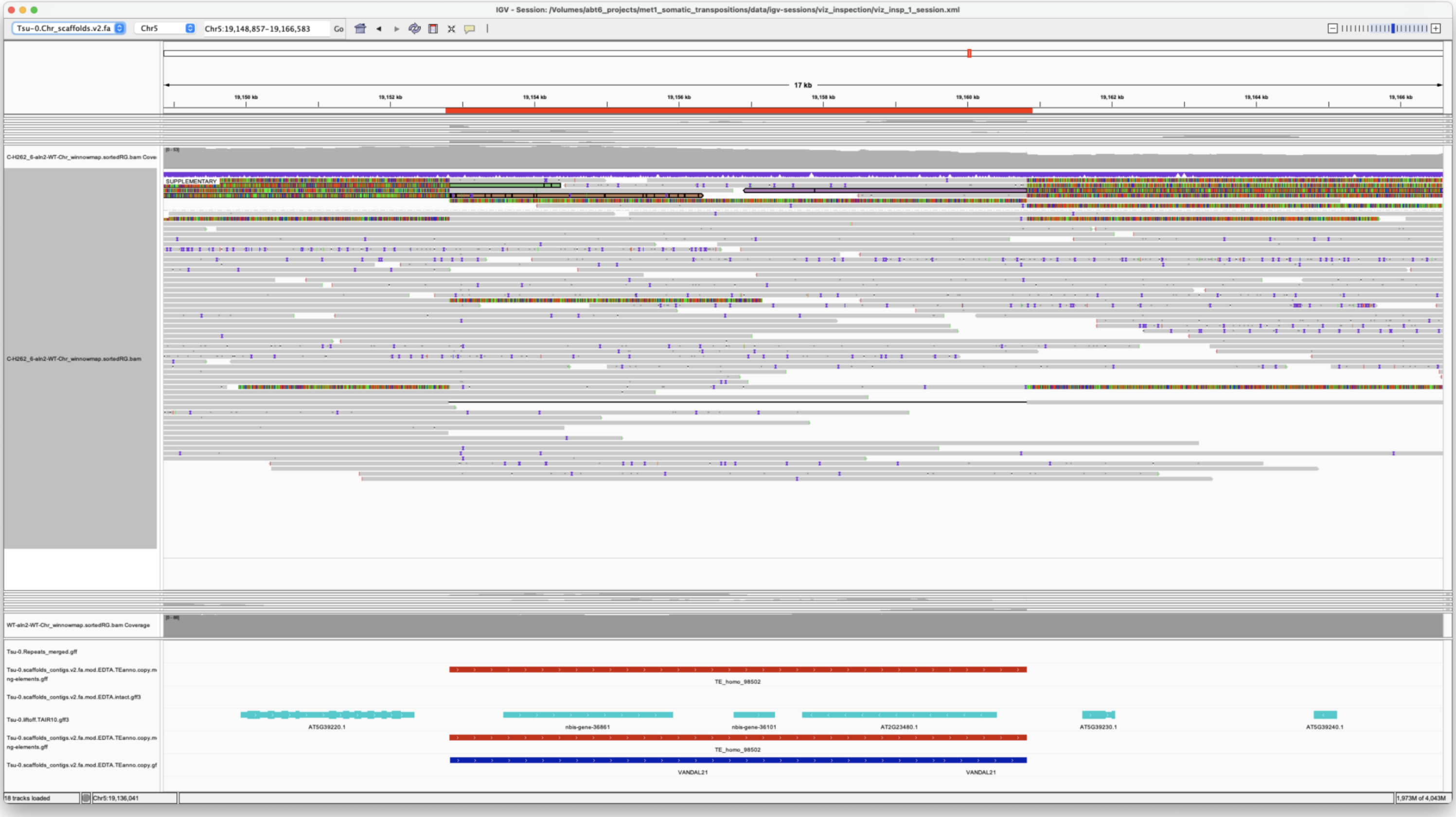

Partial

Confirmed

> Chr2 8821583 8821583 + 1 Chr5;19152829;19160826;VANDAL21 m64079_240212_113350/89457913/ccs met1_06

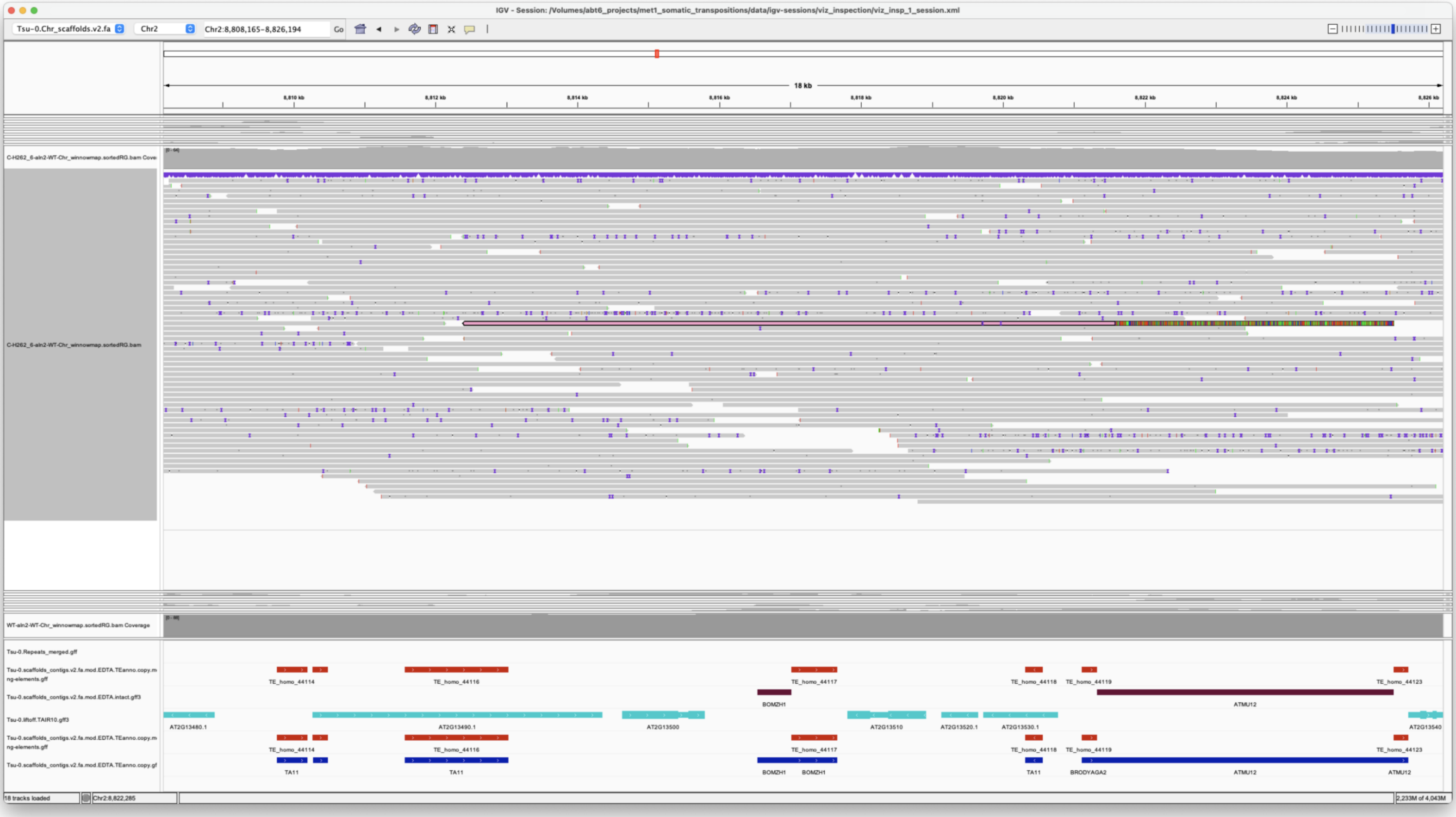

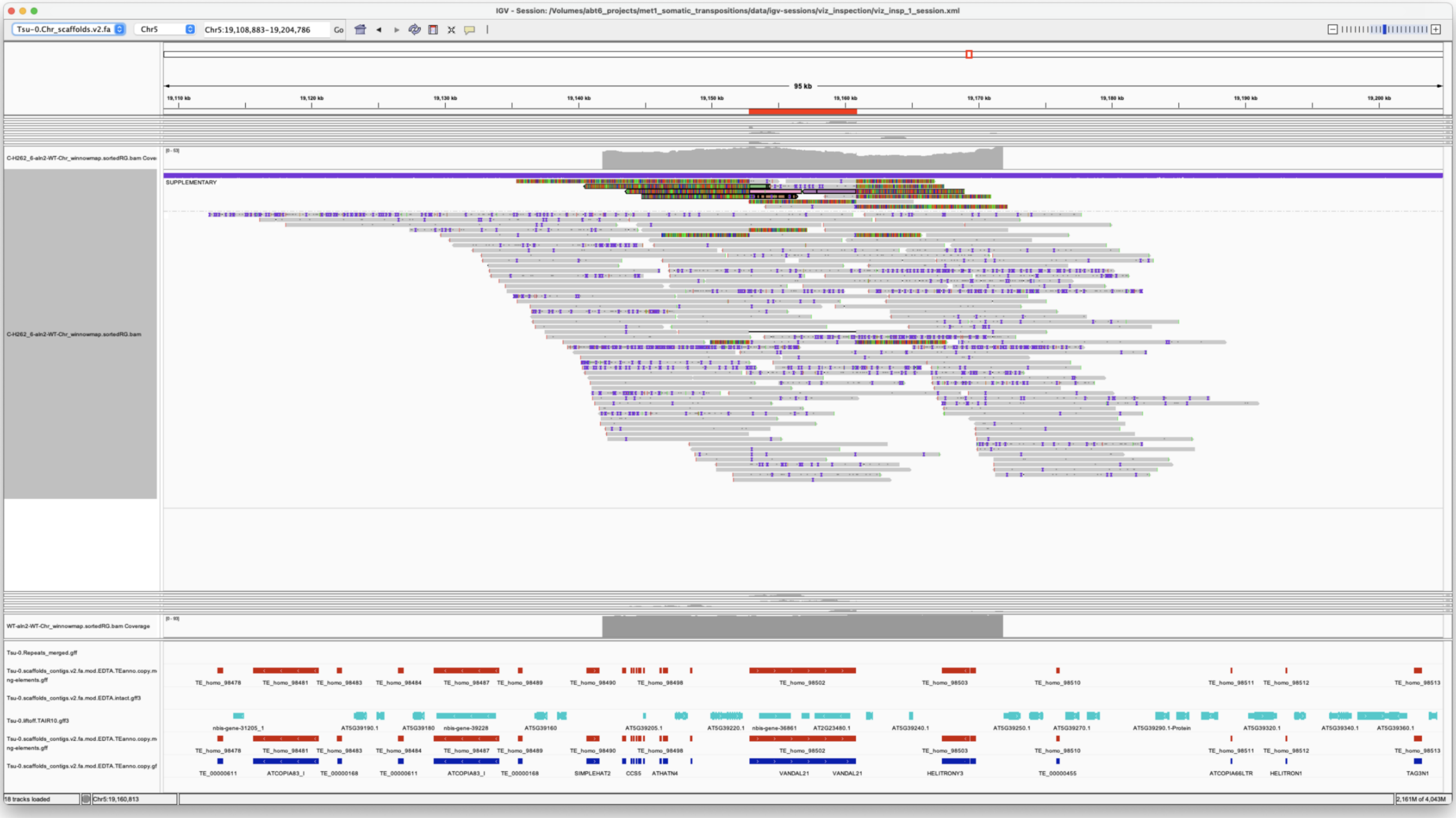

Partial

Confirmed

Chr2 16793393 16793393 - 1 Chr5;19152829;19160826;VANDAL21 m64079_221220_112036/117178755/ccs met1_06

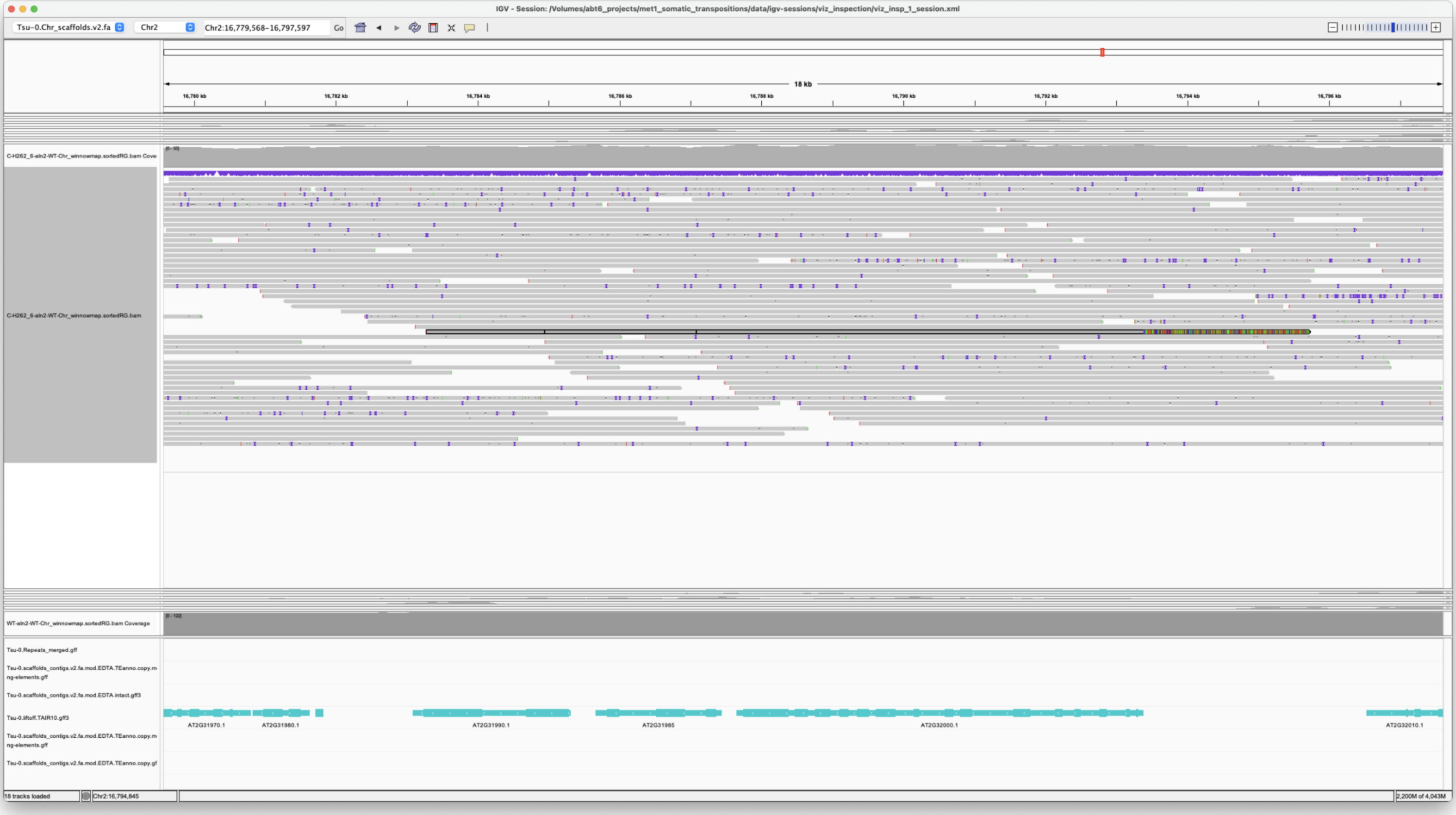

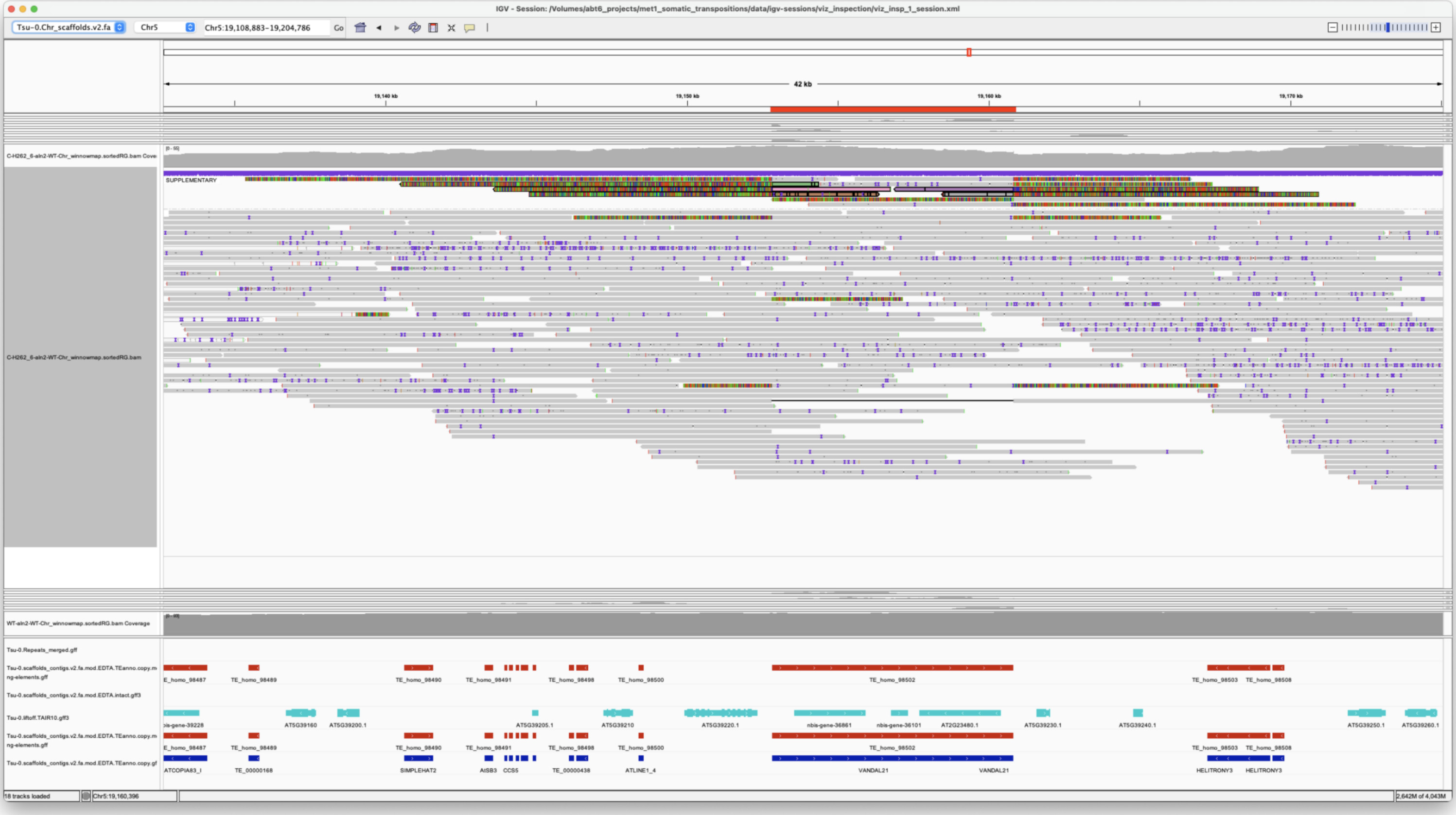

Partial

Confirmed

Chr2 17390044 17390044 - 1 Chr3;16344522;16352497;VANDAL6 m64079_221220_112036/76742734/ccs met1_06

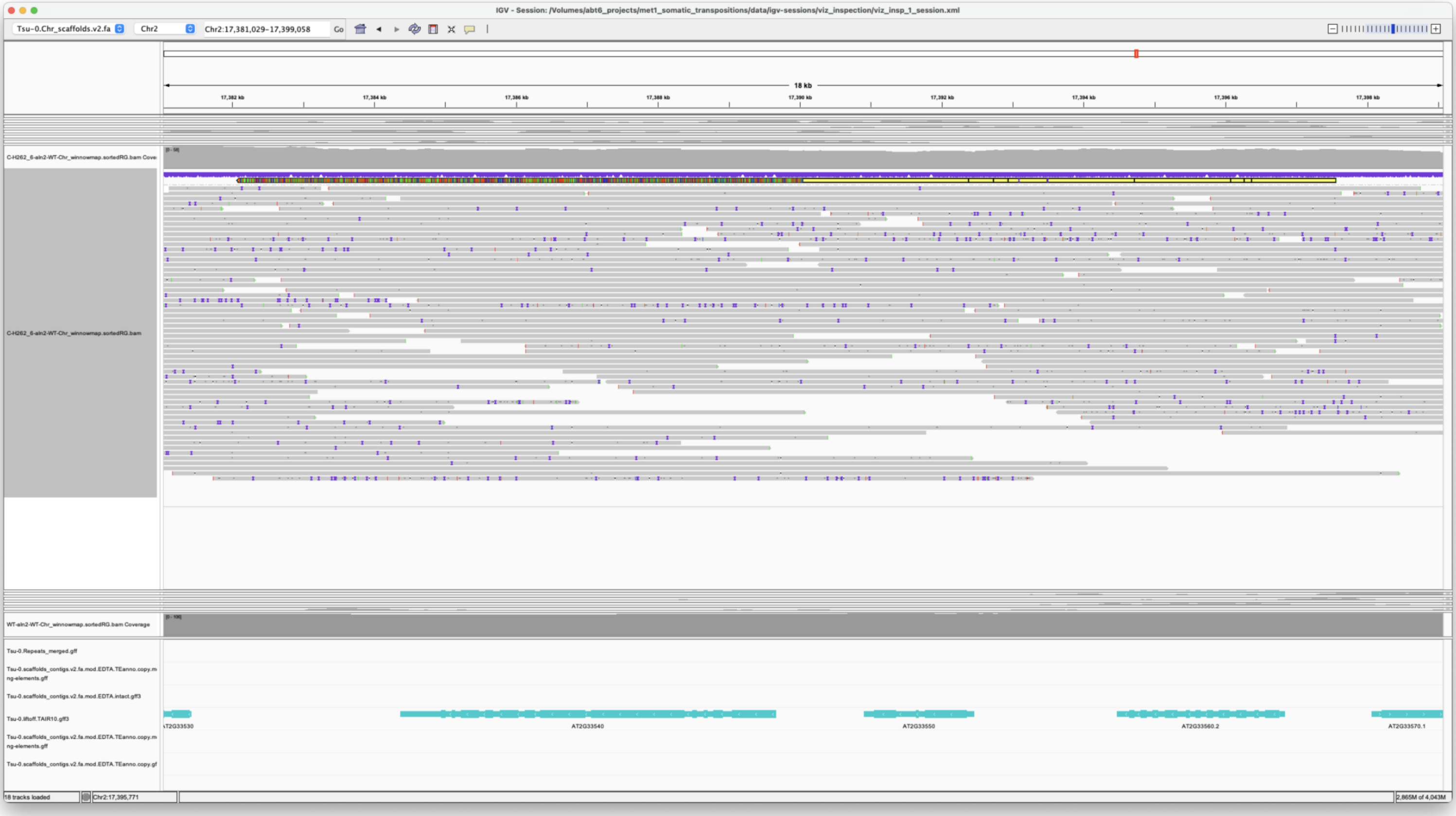

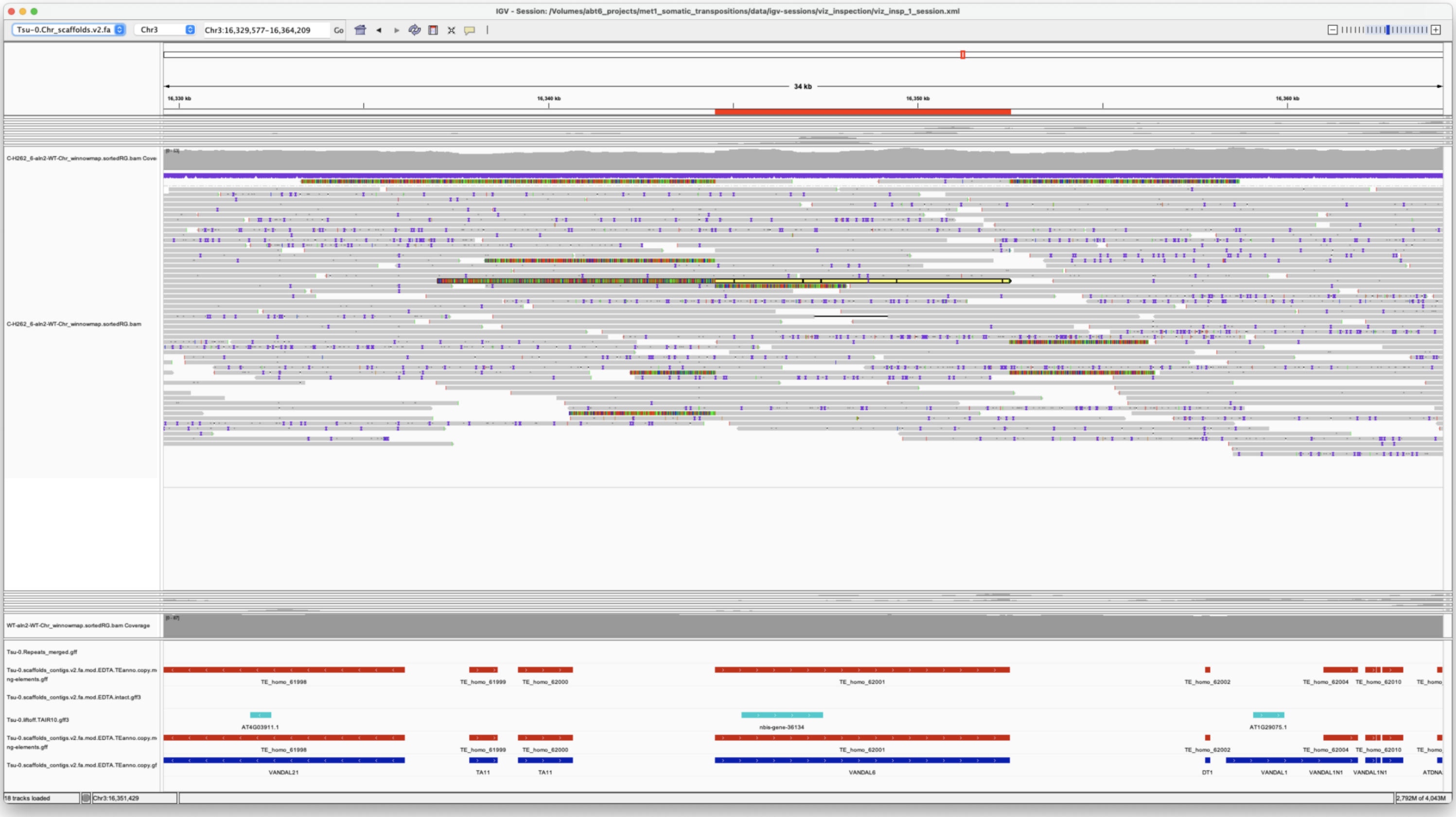

Partial

Confirmed

Chr2 17943470 17943470 - 1 Chr3;16344522;16352497;VANDAL6 m64079_240212_113350/1507864/ccs met1_06

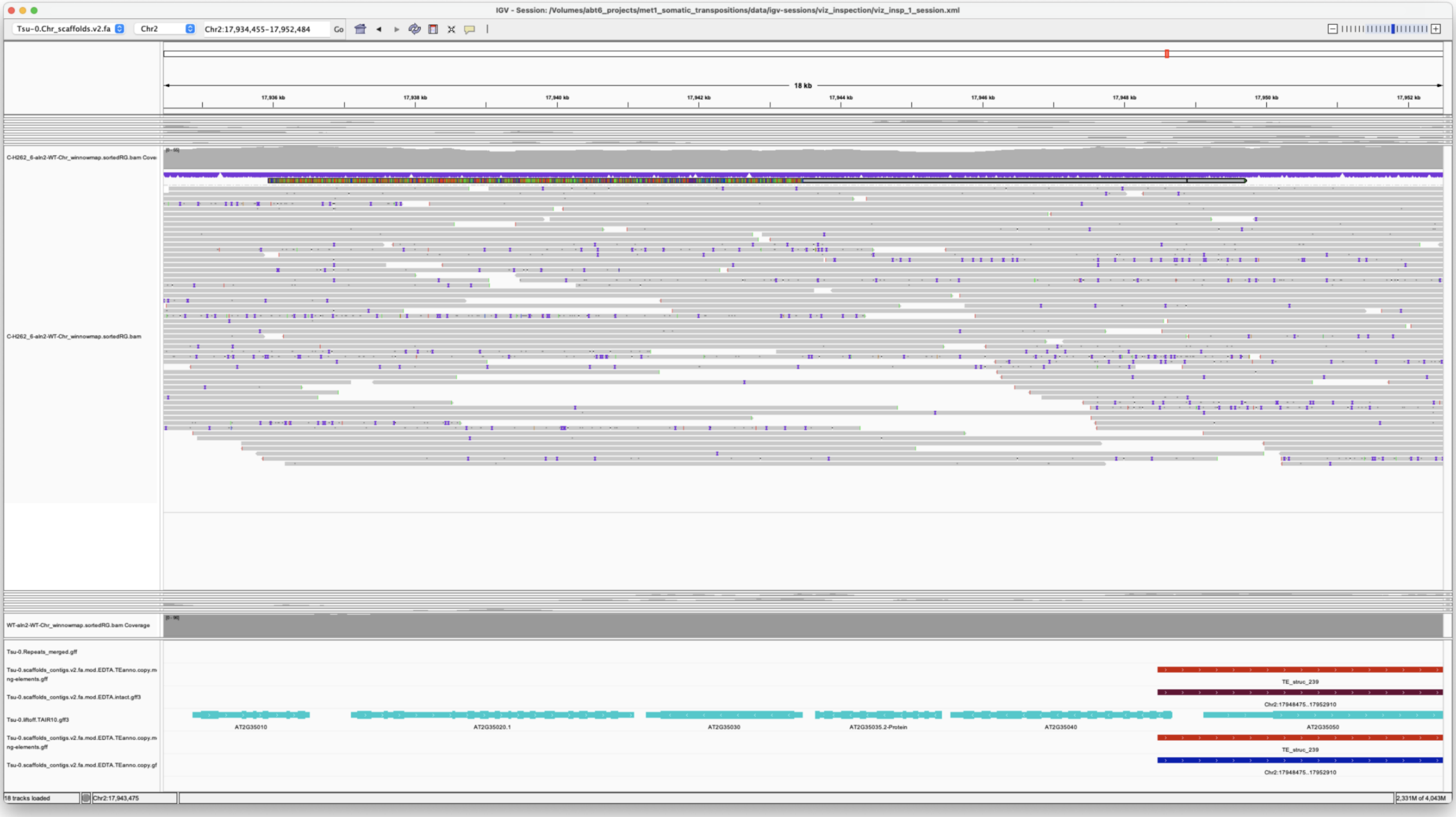

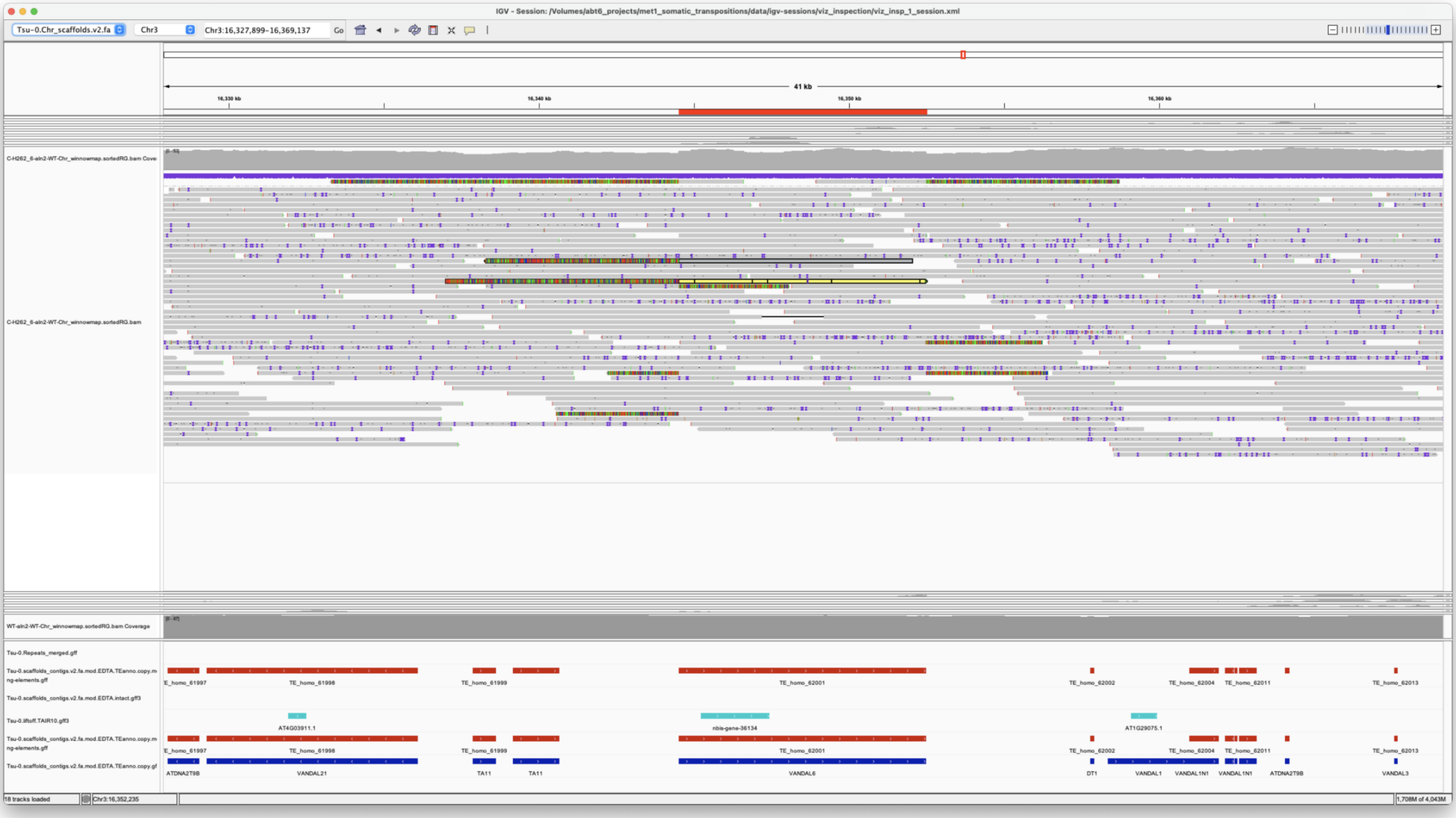

Partial

Confirmed

Chr2 17957374 17957374 - 1 Chr1;11941106;11946436;ATCOPIA93_Evade m64079_240212_113350/85527024/ccs met1_06

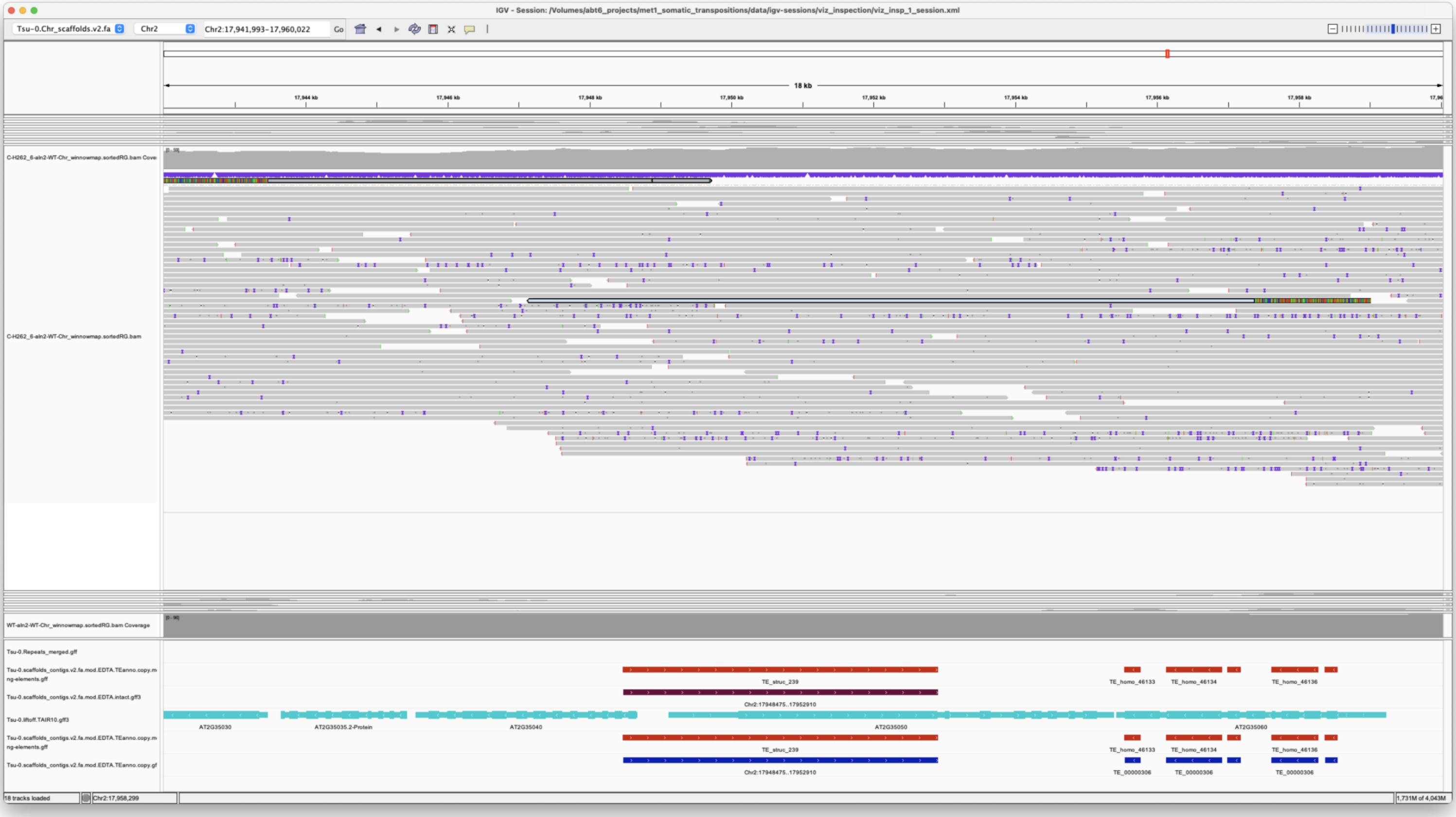

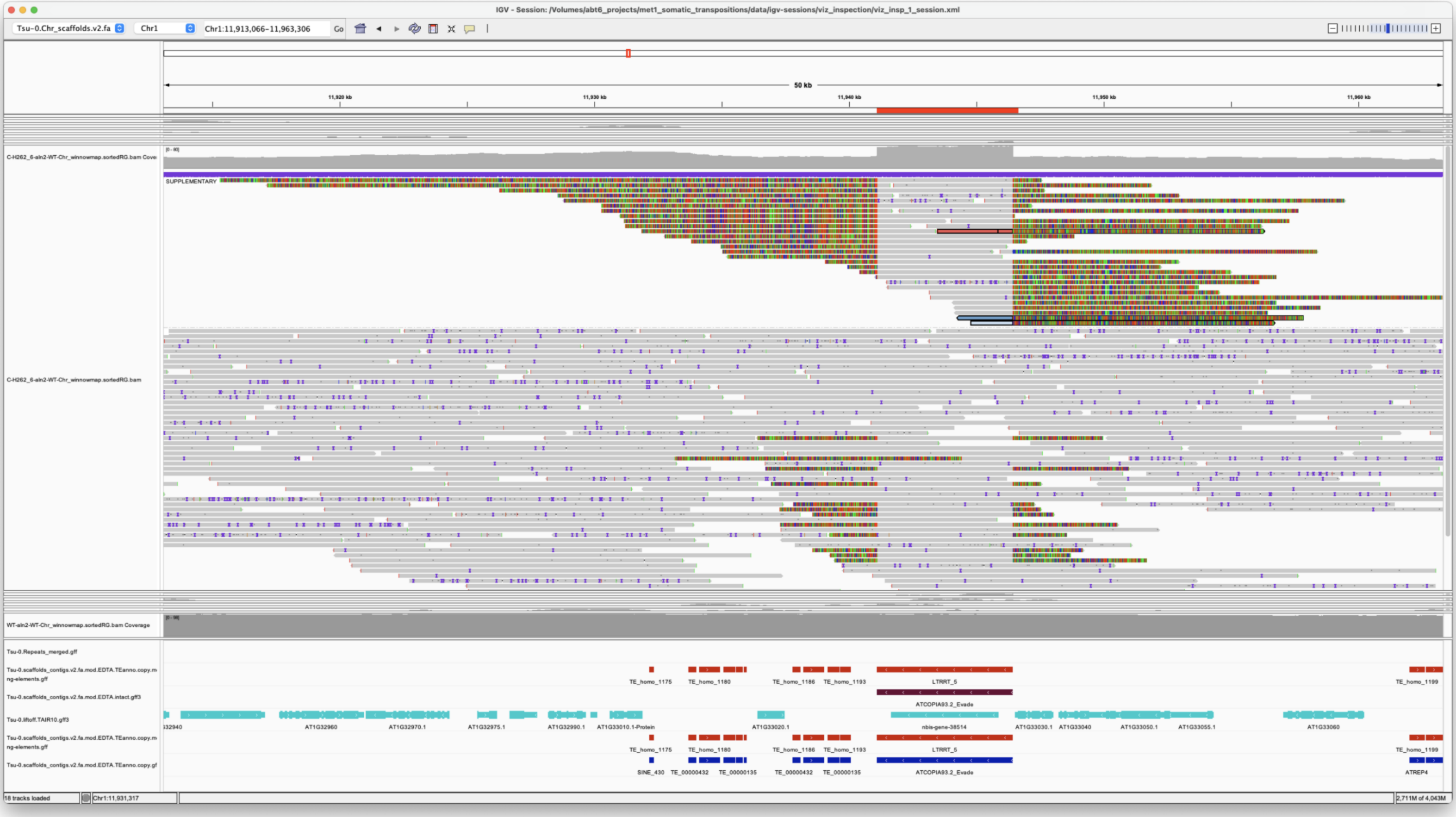

Partial

Confirmed

Chr2 18682044 18682044 + 1 Chr3;16344522;16352497;VANDAL6 m64079_221220_112036/17106667/ccs met1_06

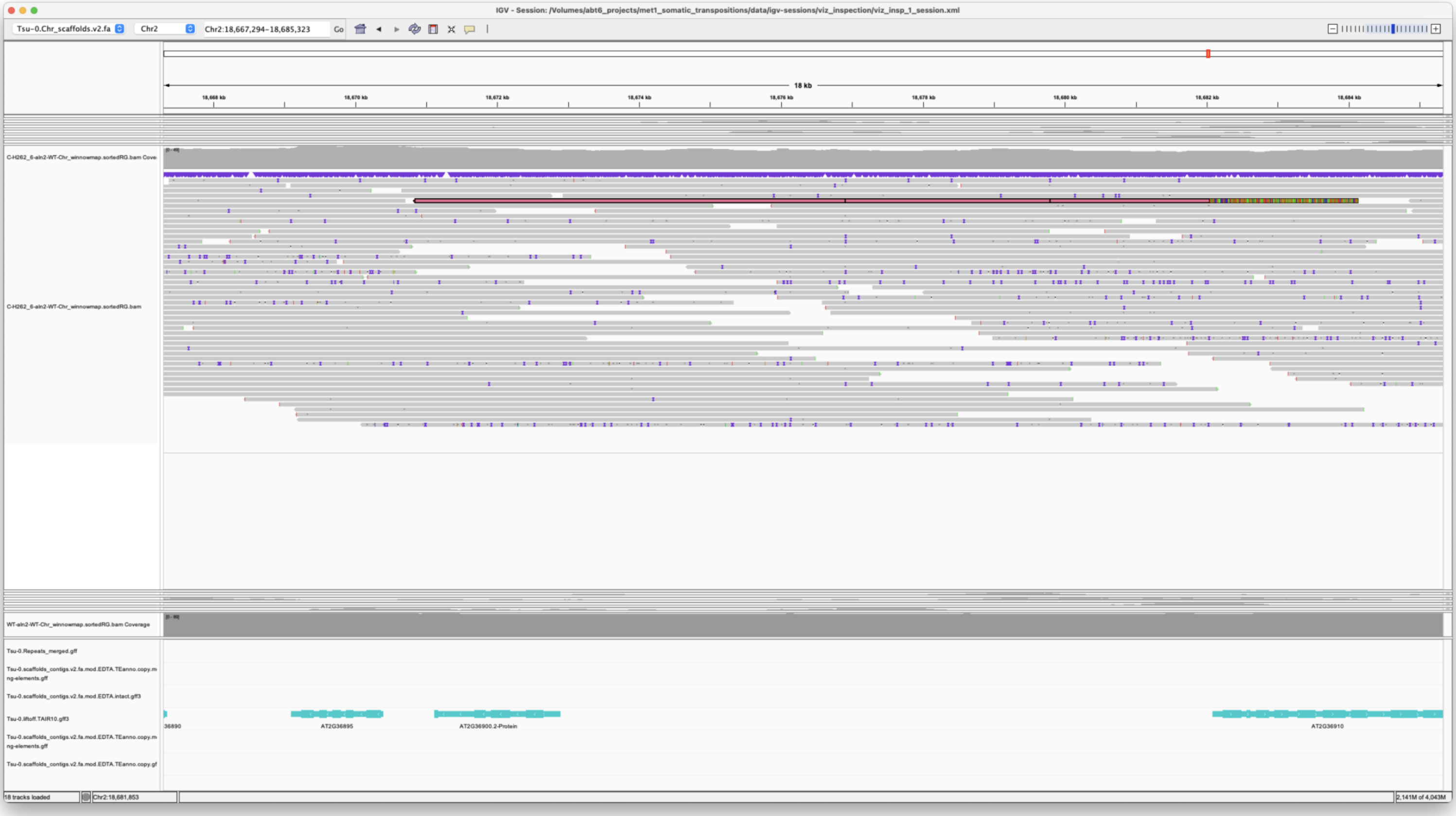

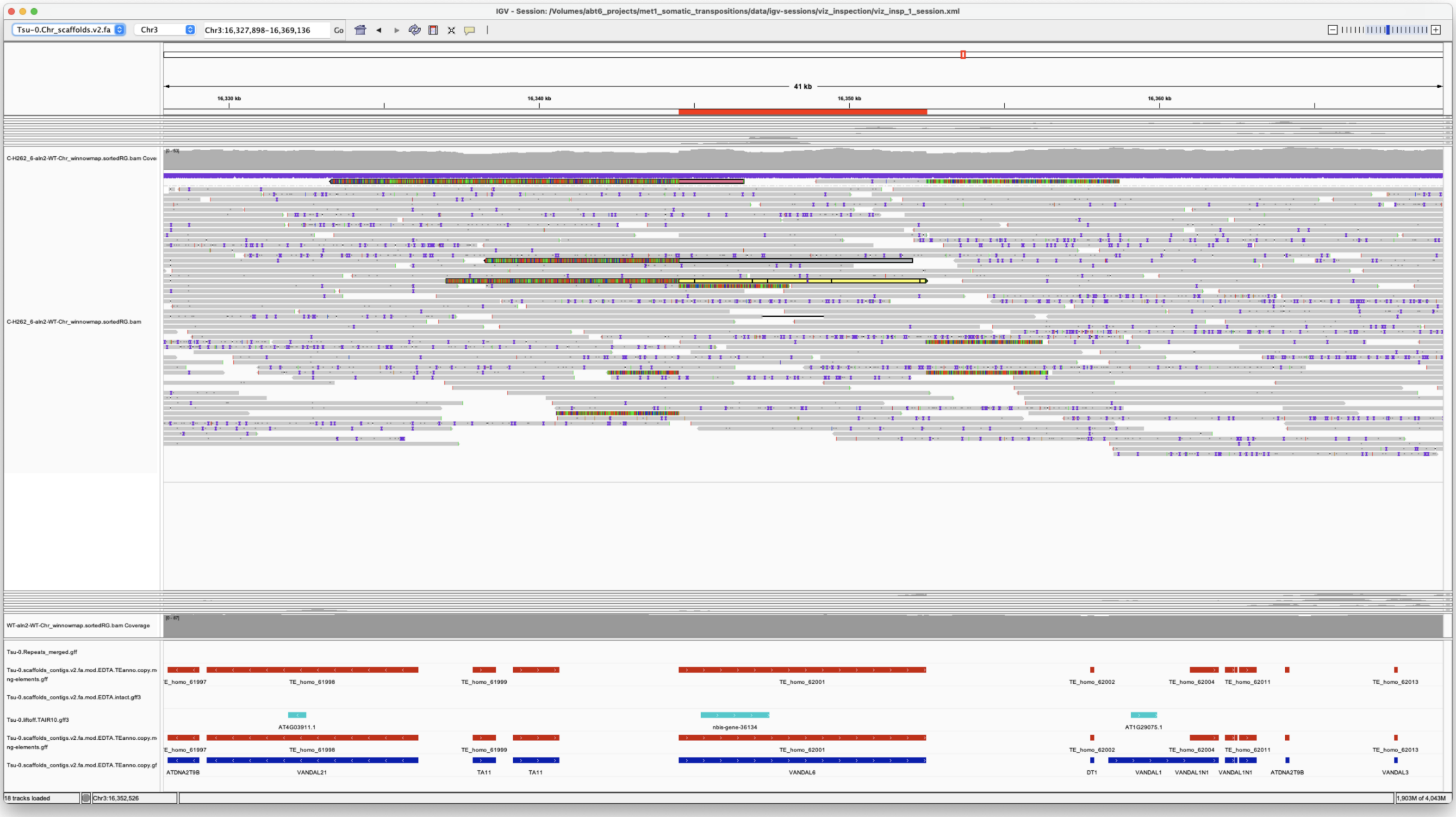

Partial

Confirmed

Chr3 891173 891173 + 1 Chr1;11941106;11946436;ATCOPIA93_Evade m64079_221220_112036/172623965/ccs met1_06

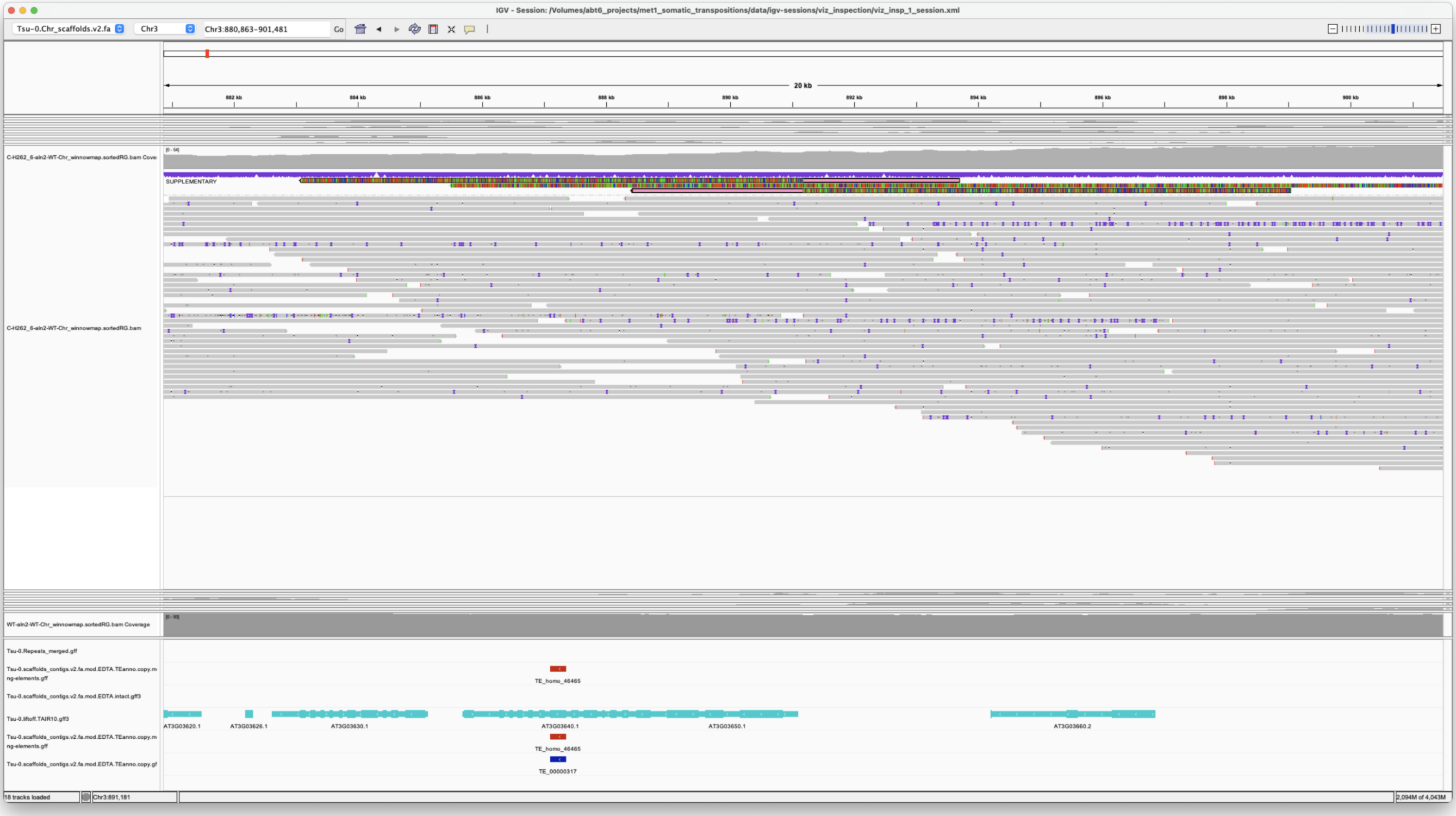

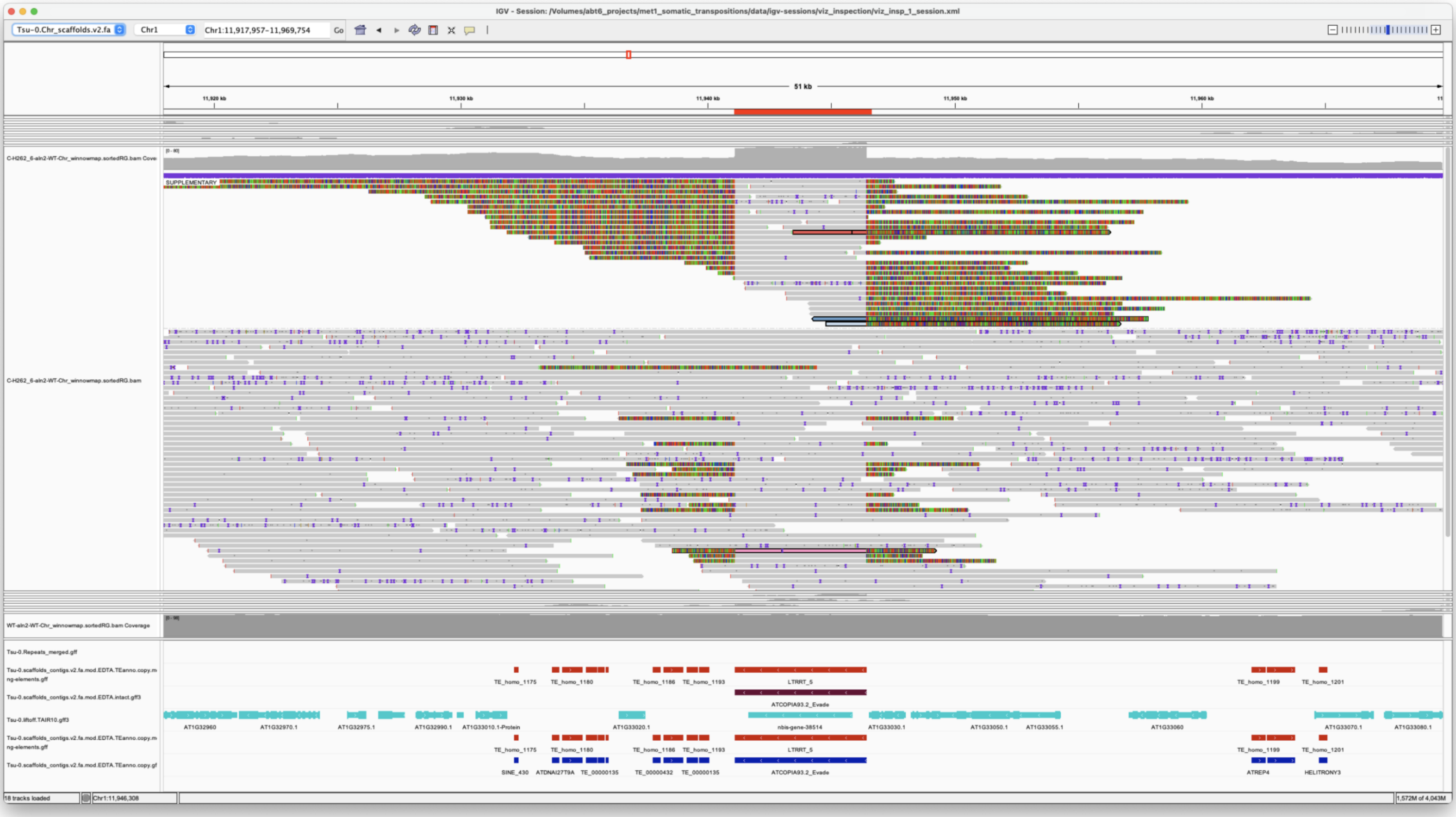

Central
TSD

Confirmed

Chr3 13352824 13352824 + 1 Chr1;11941106;11946436;ATCOPIA93_Evade m64079_221220_112036/169738614/ccs met1_06

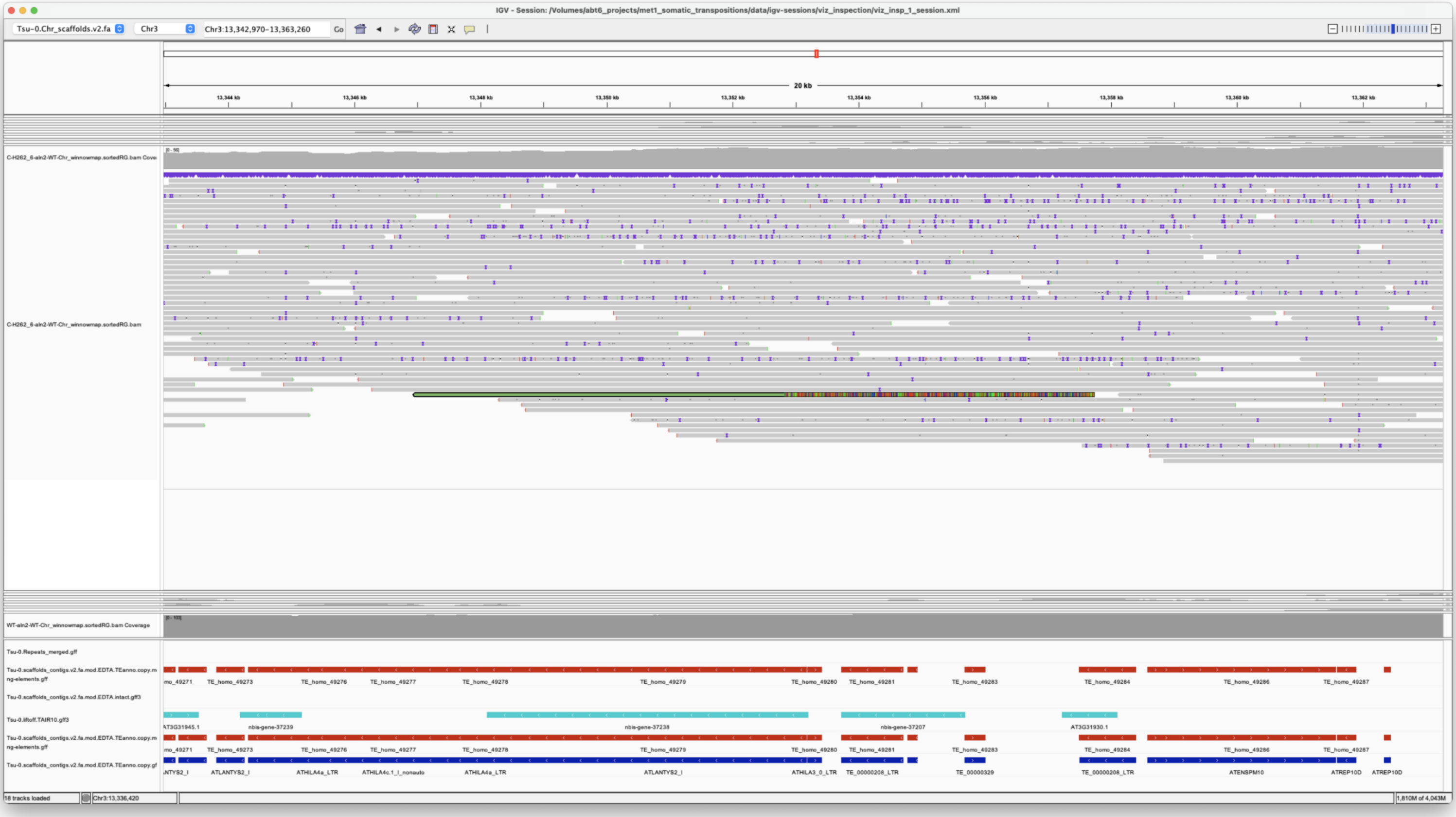

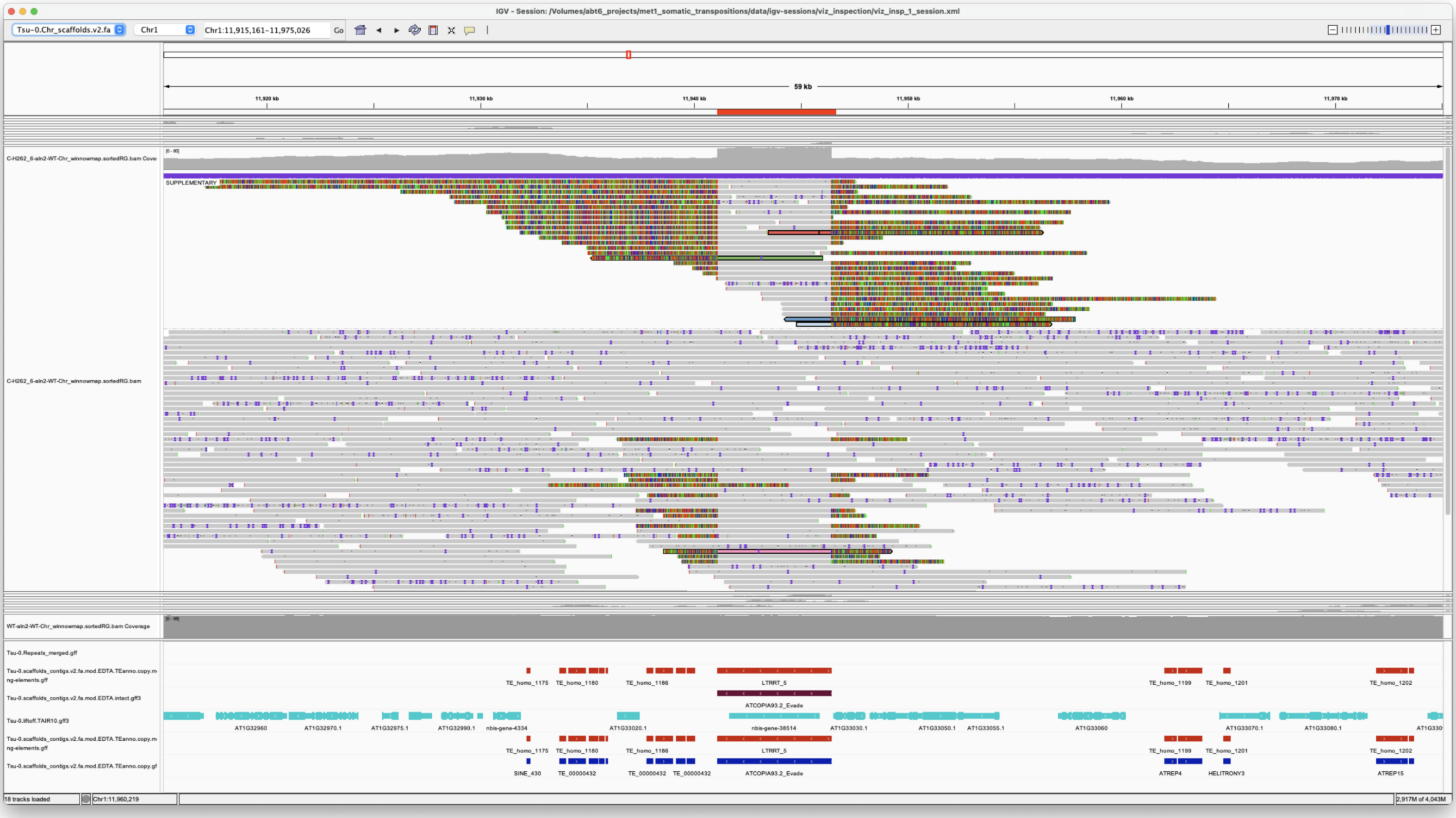

Partial

Confirmed

Chr3 13352824 13352824 + 1 Chr1;11941106;11946436;ATCOPIA93_Evade m64079_221220_112036/169738614/ccs met1_06

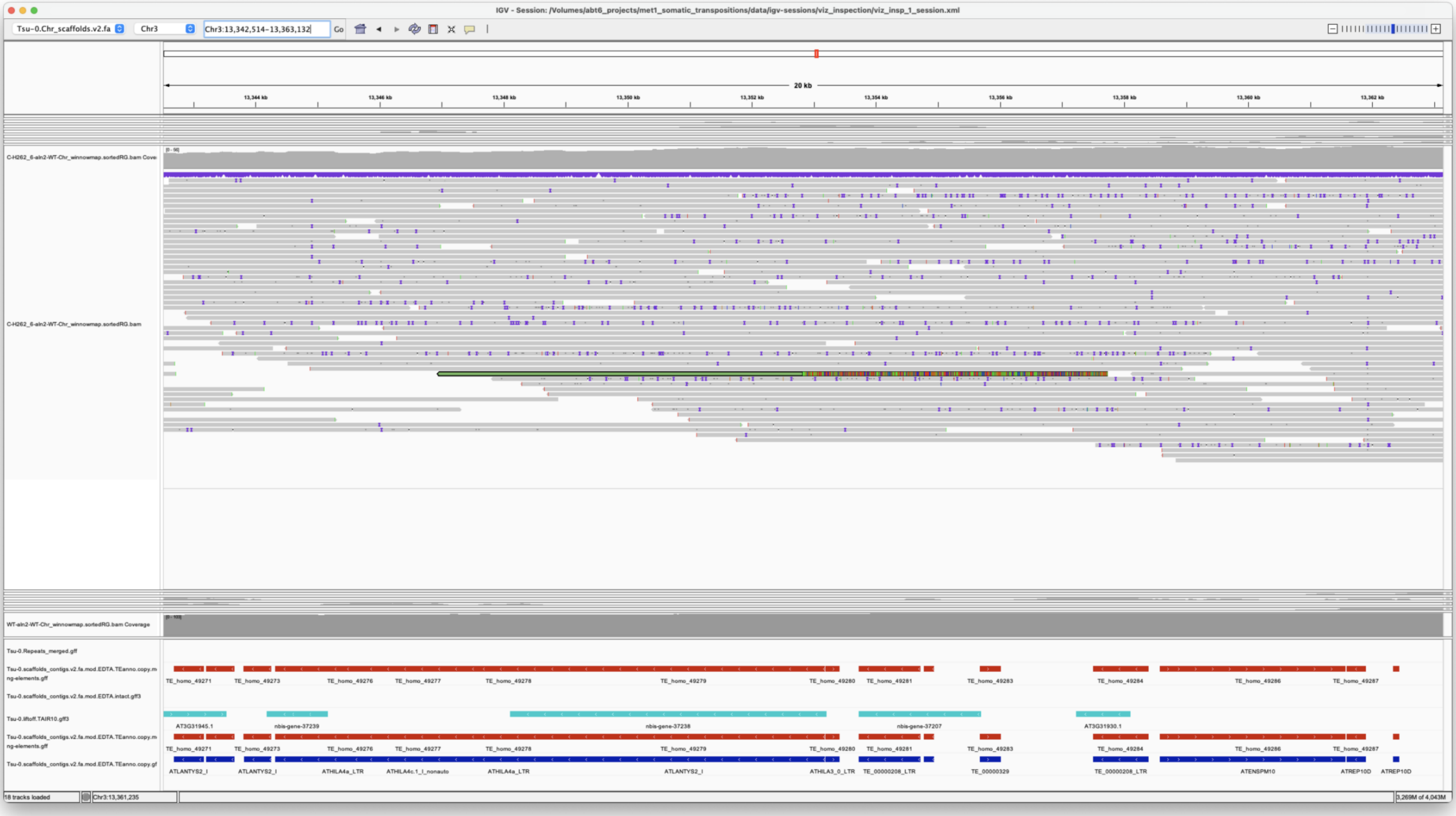

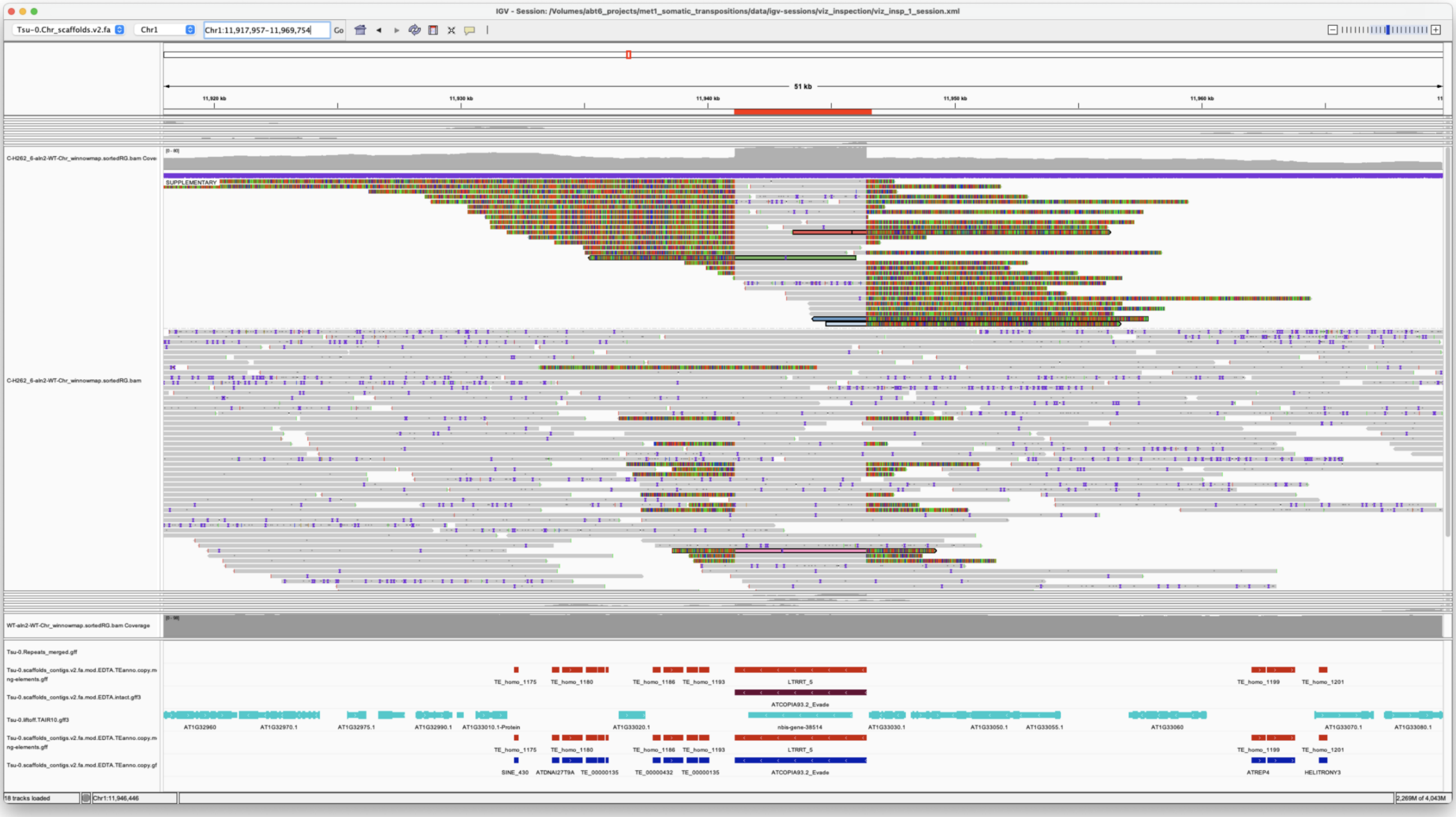

Partial

Confirmed

Chr3 16344523 16344523 - 1 Chr3;16344522;16352497;VANDAL6 m64079_221220_112036/71633312/ccs met1_06

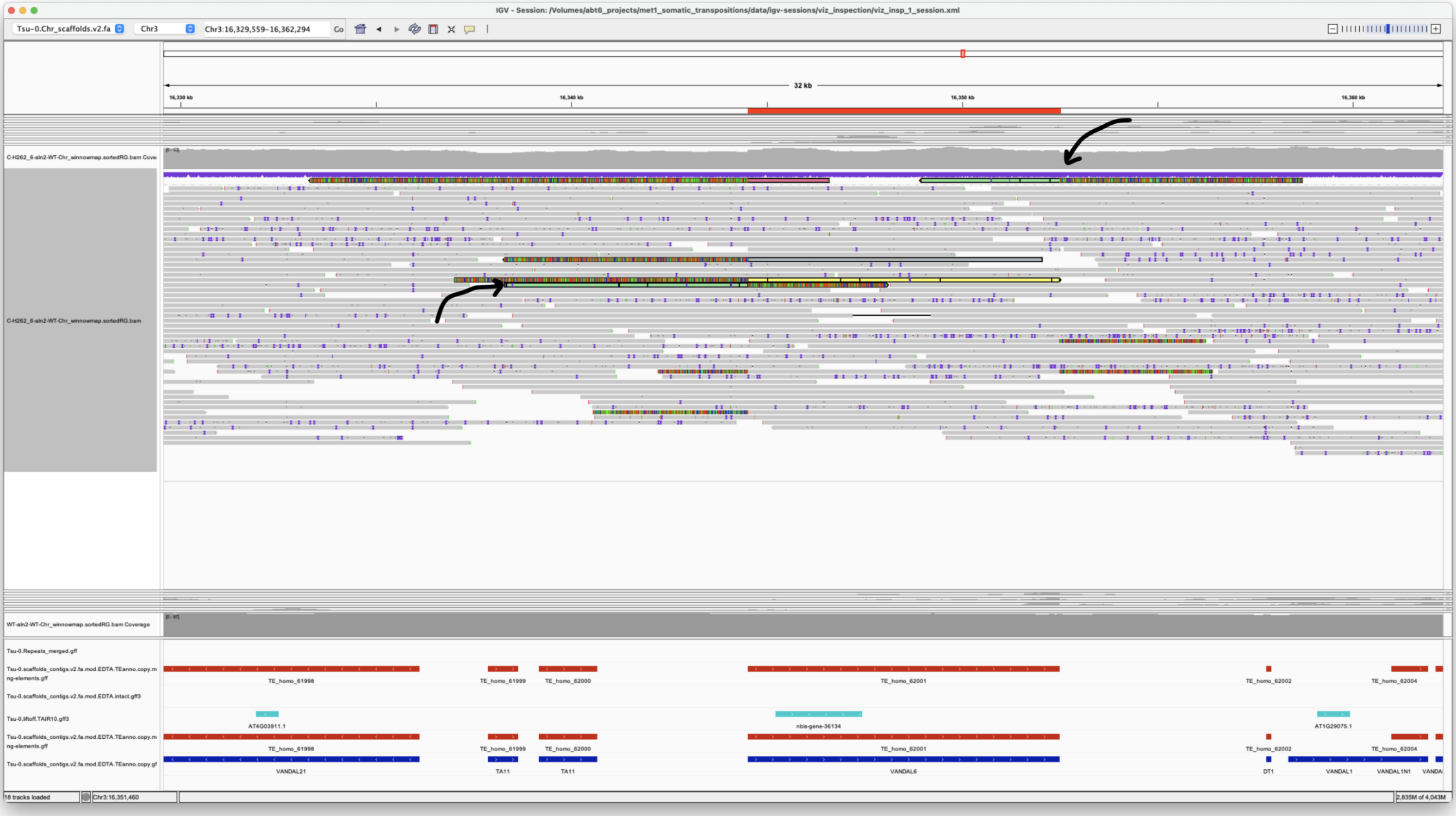

Rearrangement

unsupported

Chr4 2407651 2407651 + 1 Chr1;11941106;11946436;ATCOPIA93_Evade m64079_221220_112036/11666852/ccs met1_06

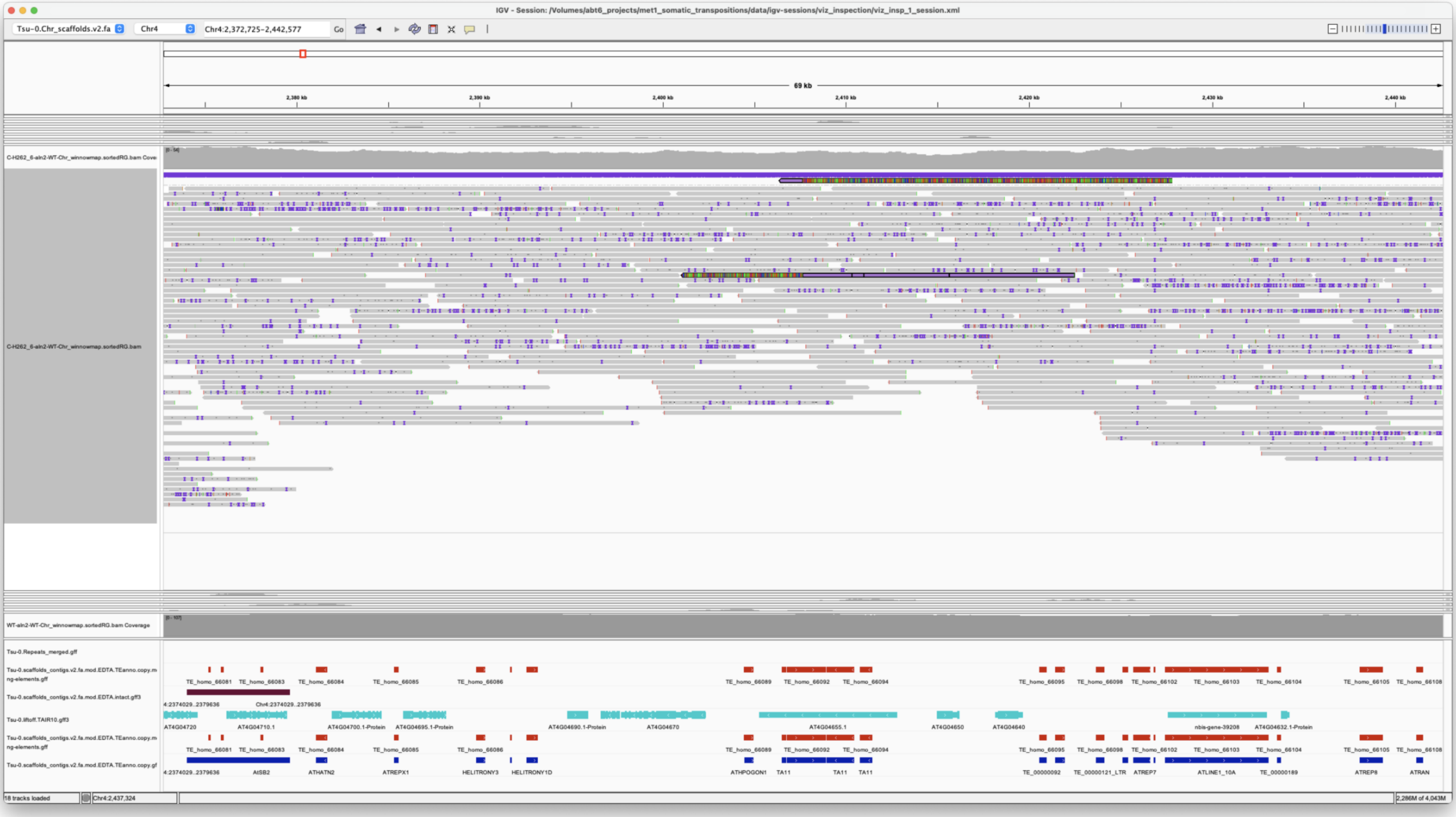

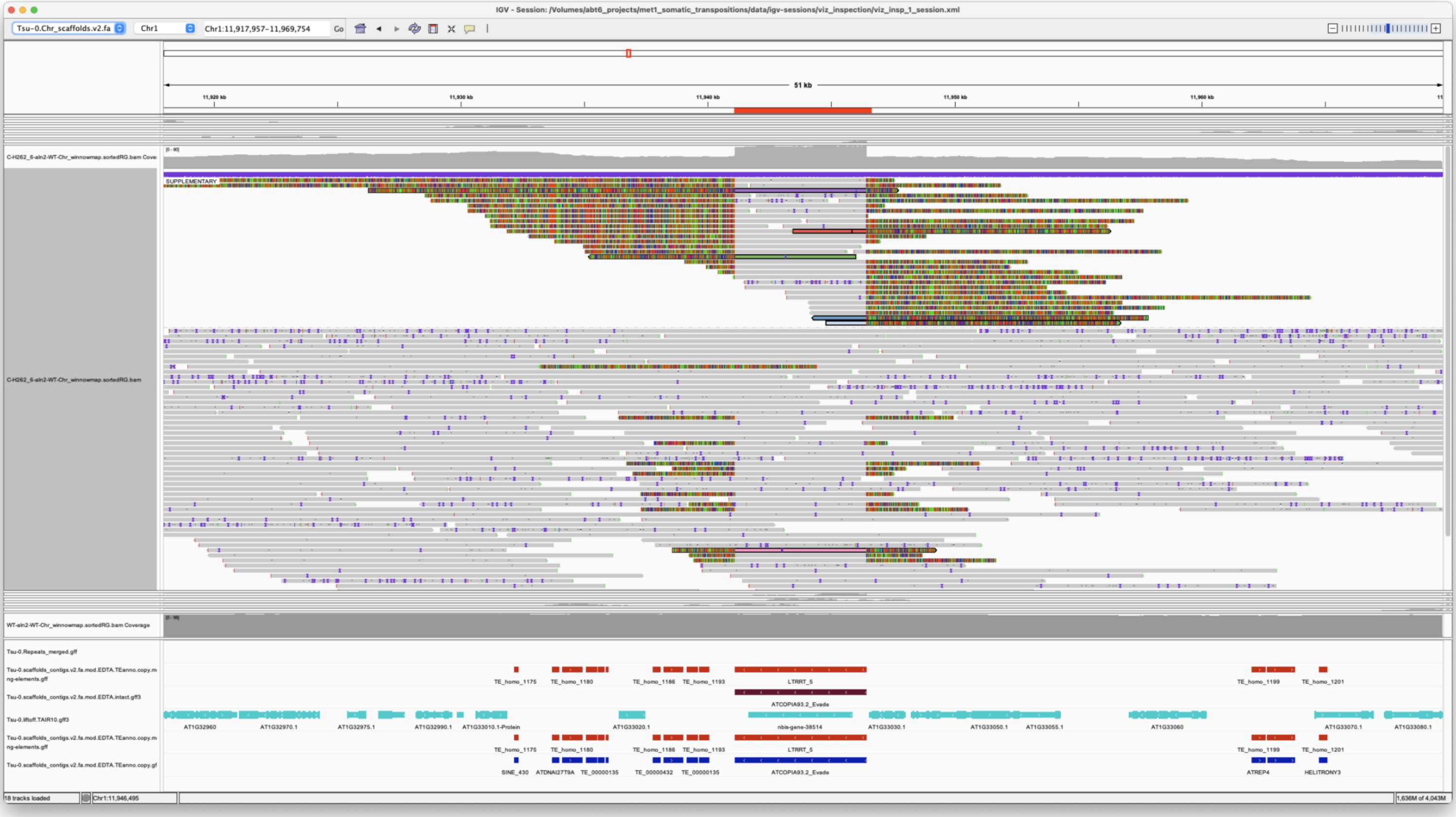

Central
TSD

Confirmed

Chr4 8851532 8851532 - 1 Chr1;11941106;11946436;ATCOPIA93_Evade m64079_240212_113350/147982772/ccs met1_06

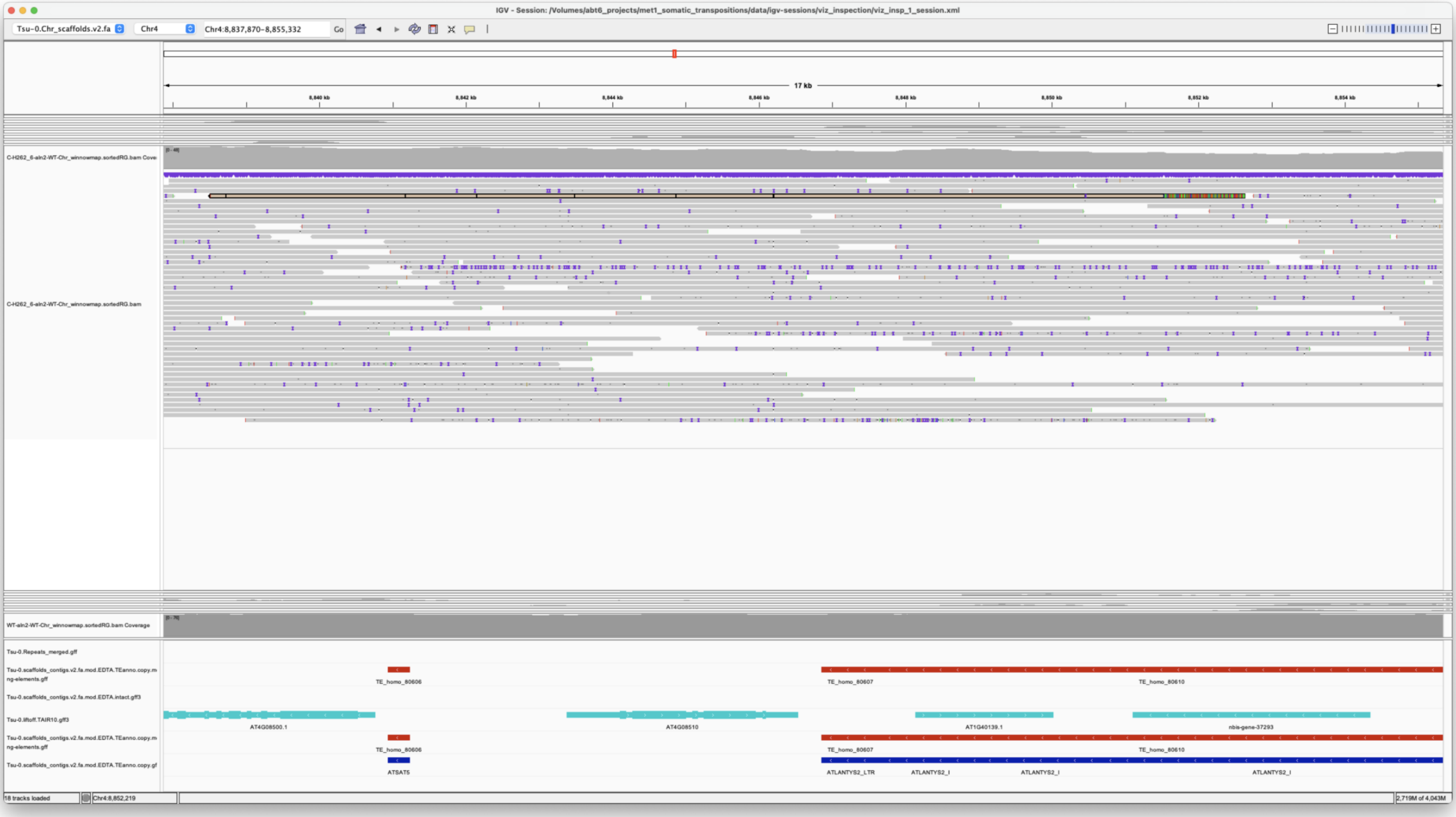

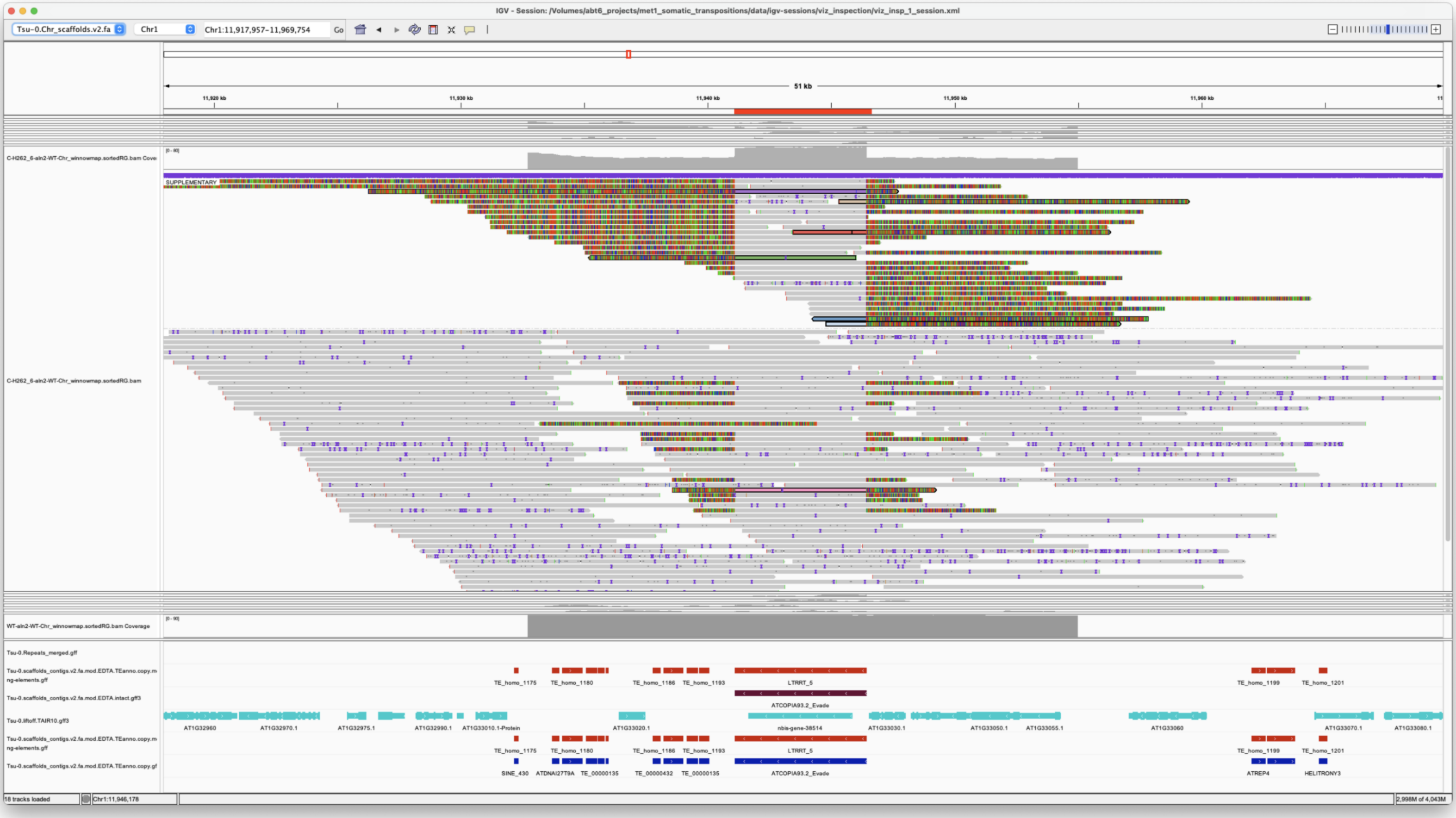

Partial

Confirmed

Chr4 10100102 10100102 + 1 Chr3;16344522;16352497;VANDAL6 m64079_221220_112036/27658802/ccs met1_06

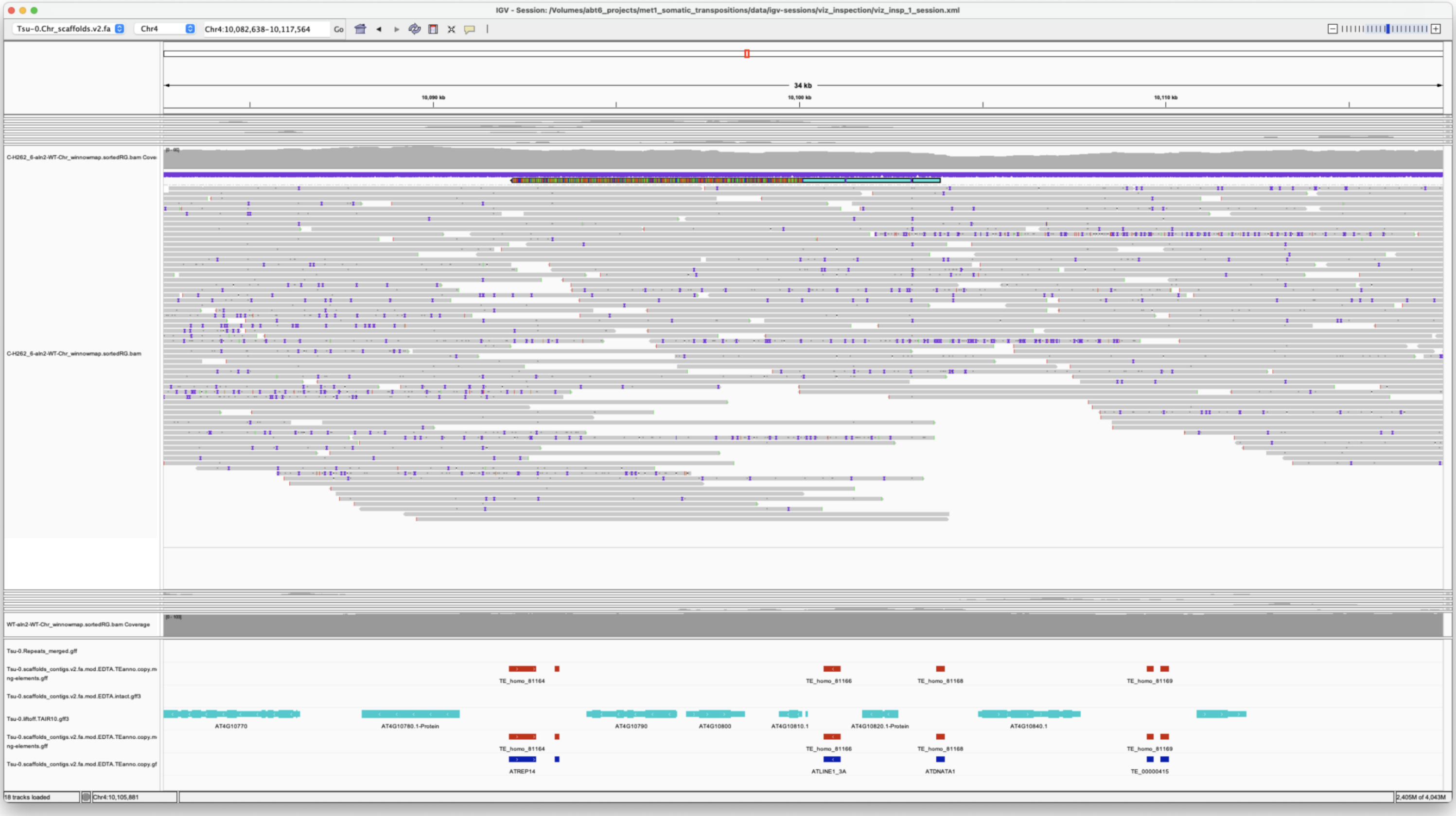

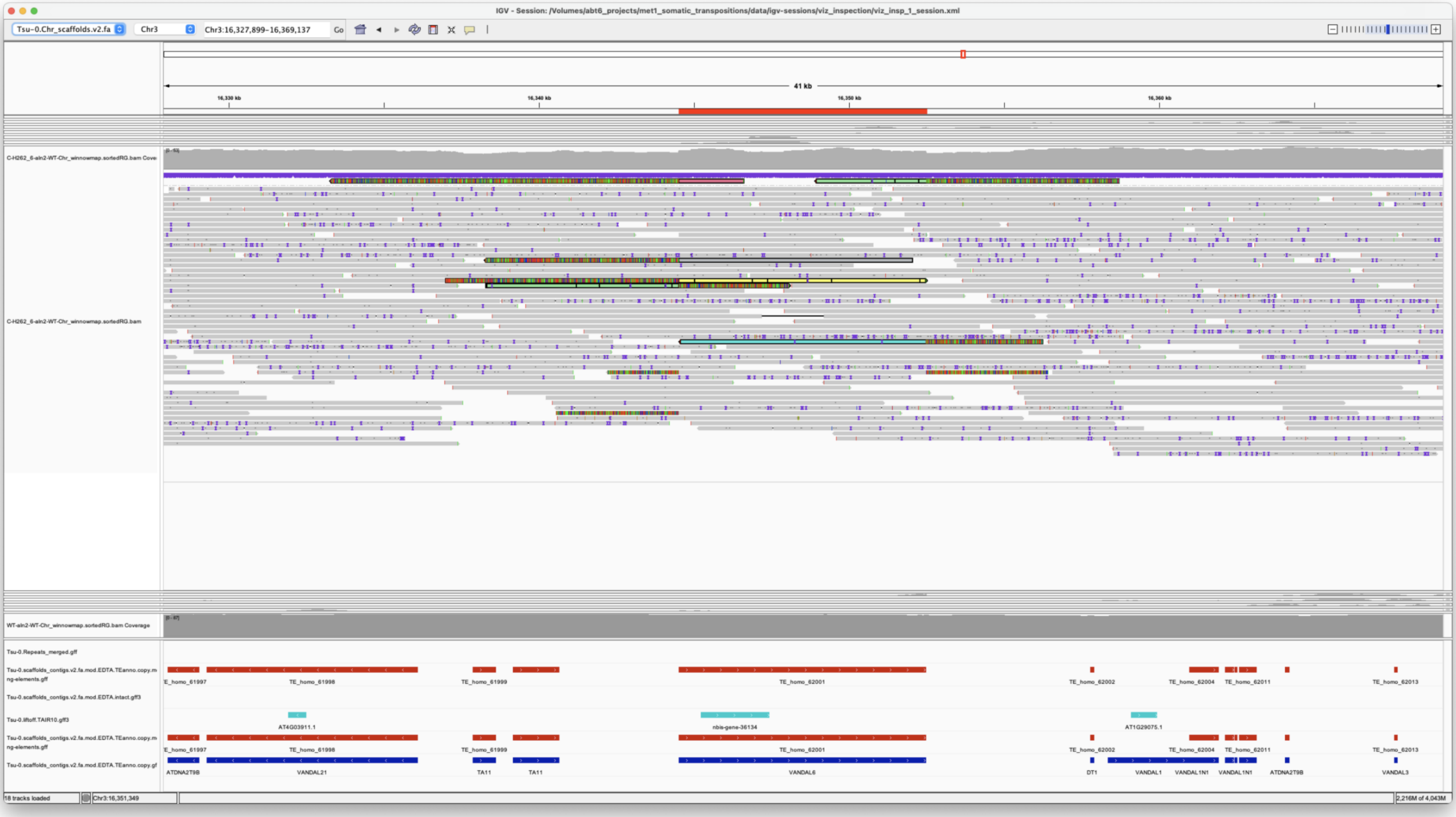

Partial

Confirmed

Chr4 16305632 16305632 - 1 Chr3;20158137;20166150;VANDAL6 m64079_240212_113350/51382961/ccs met1_06

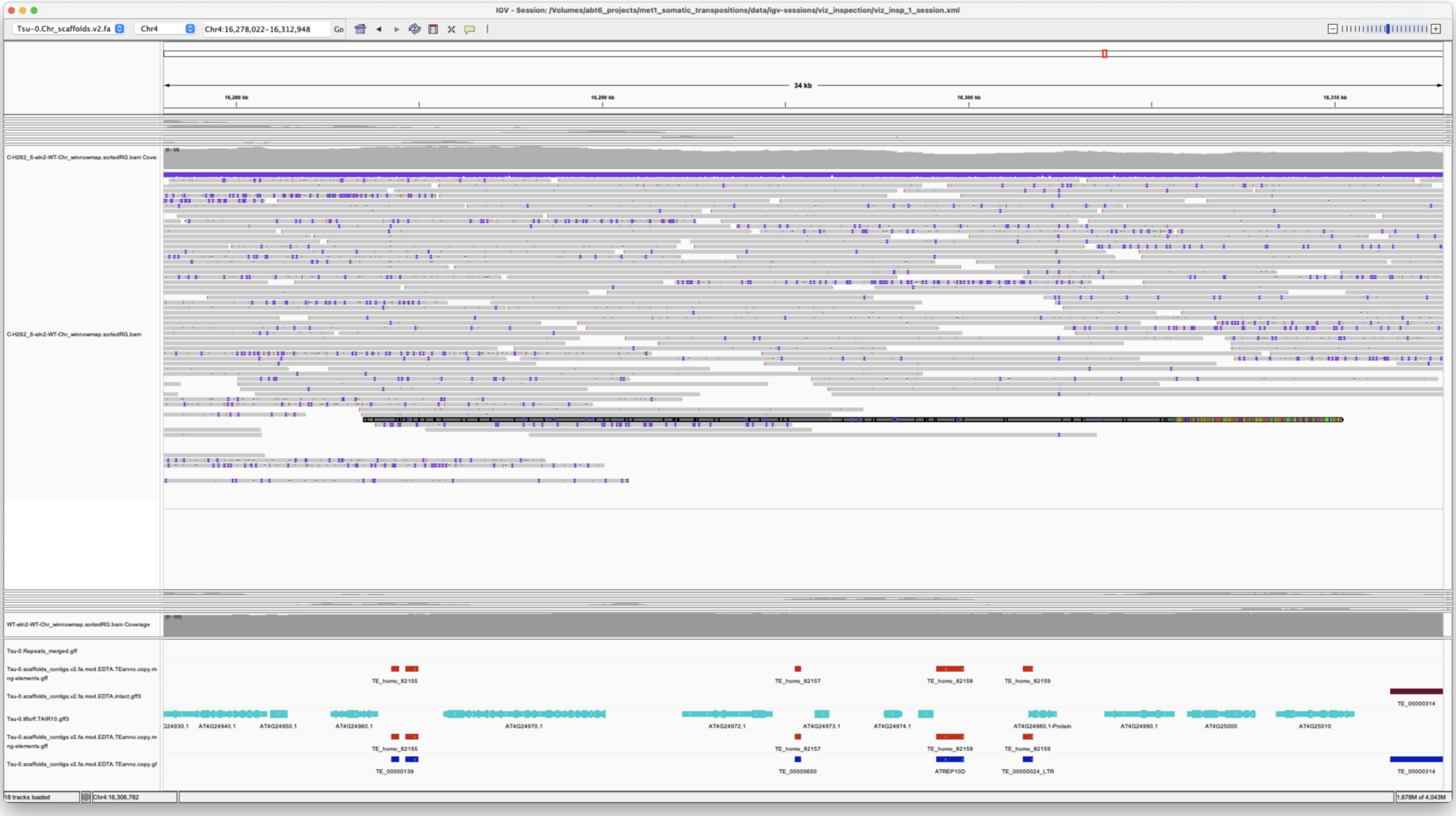

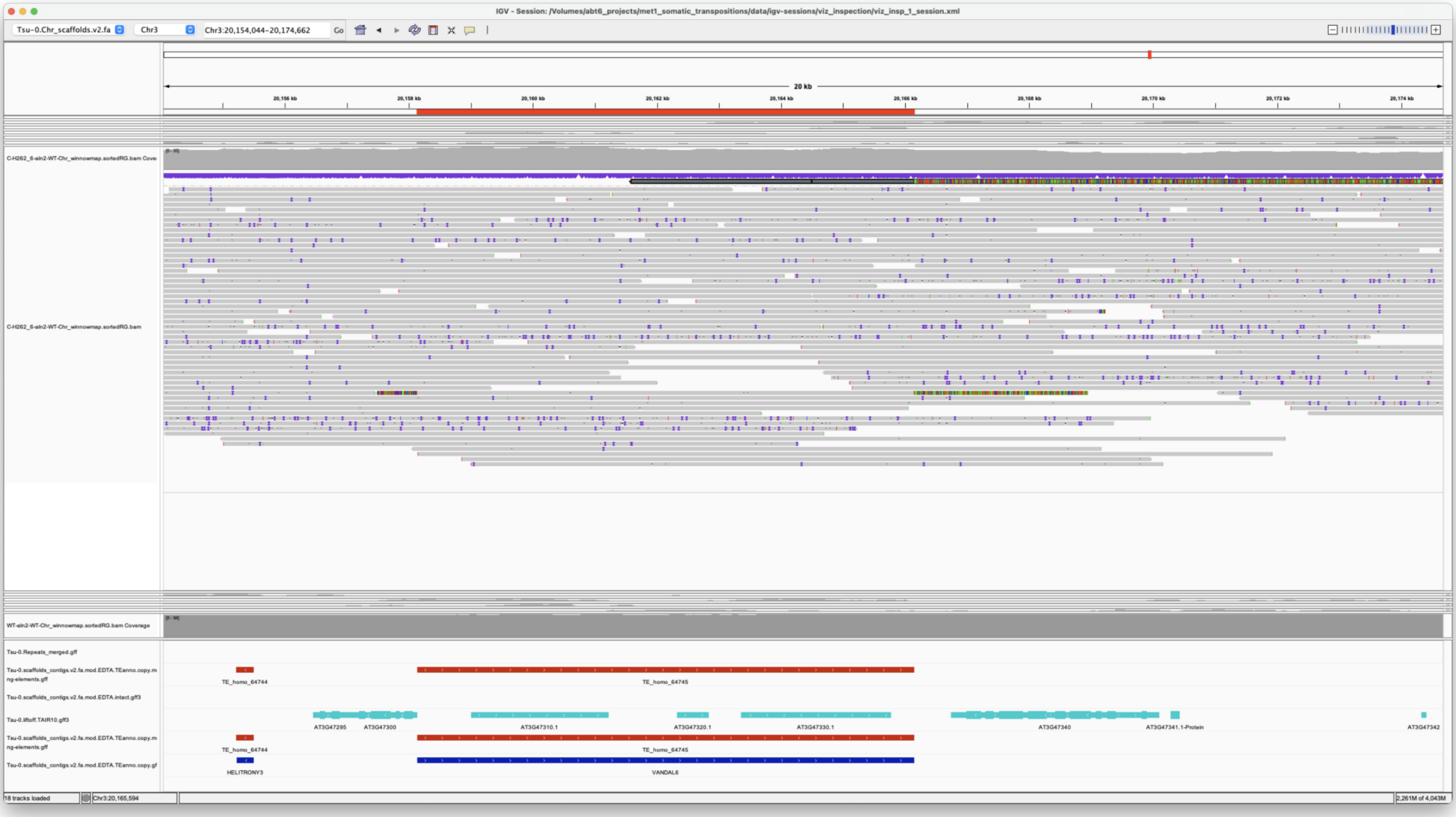

Partial

Confirmed

Chr4 19116429 19116429 + 1 Chr1;11941106;11946436;ATCOPIA93_Evade m64079_221220_112036/60293631/ccs met1_06

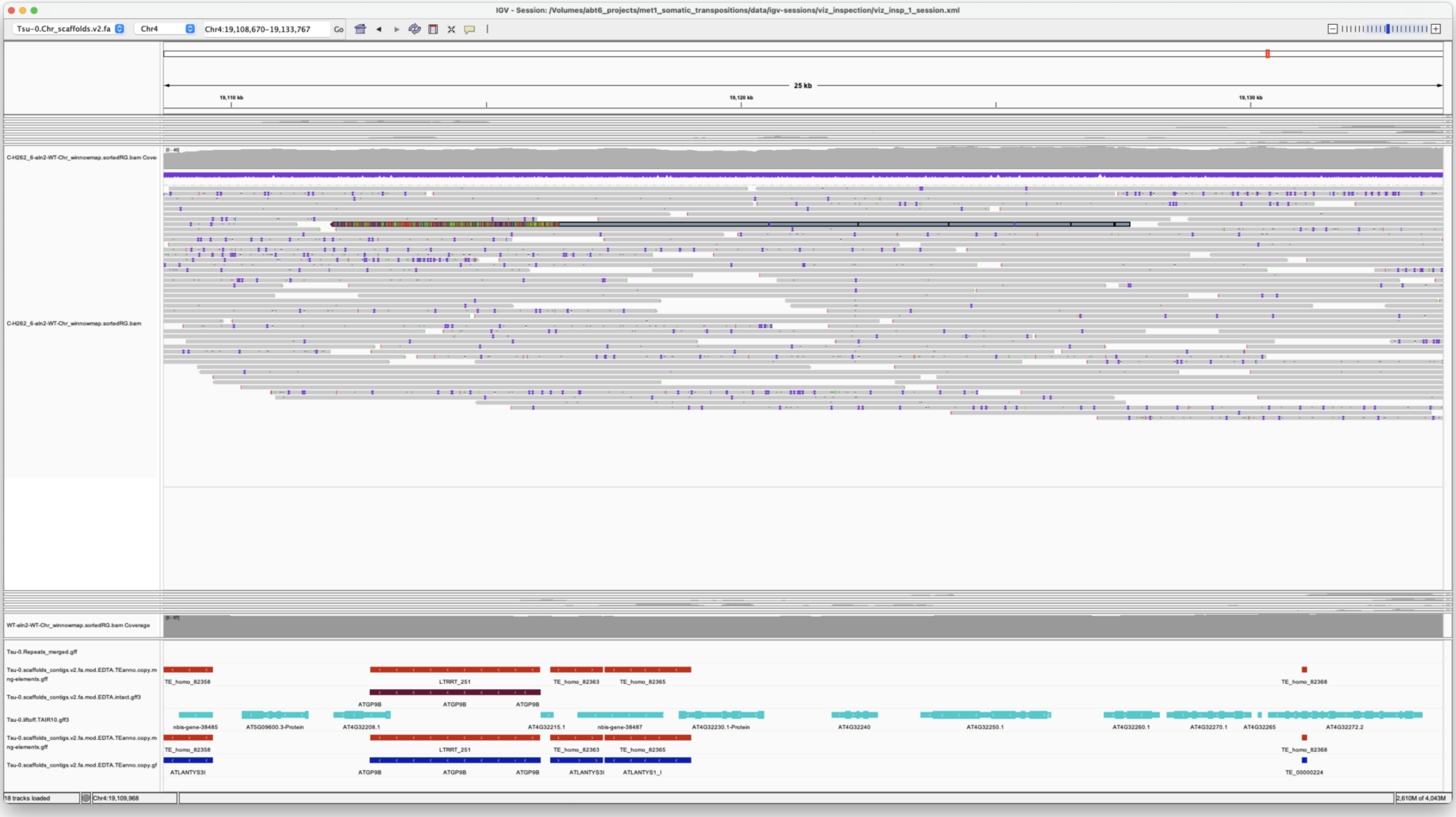

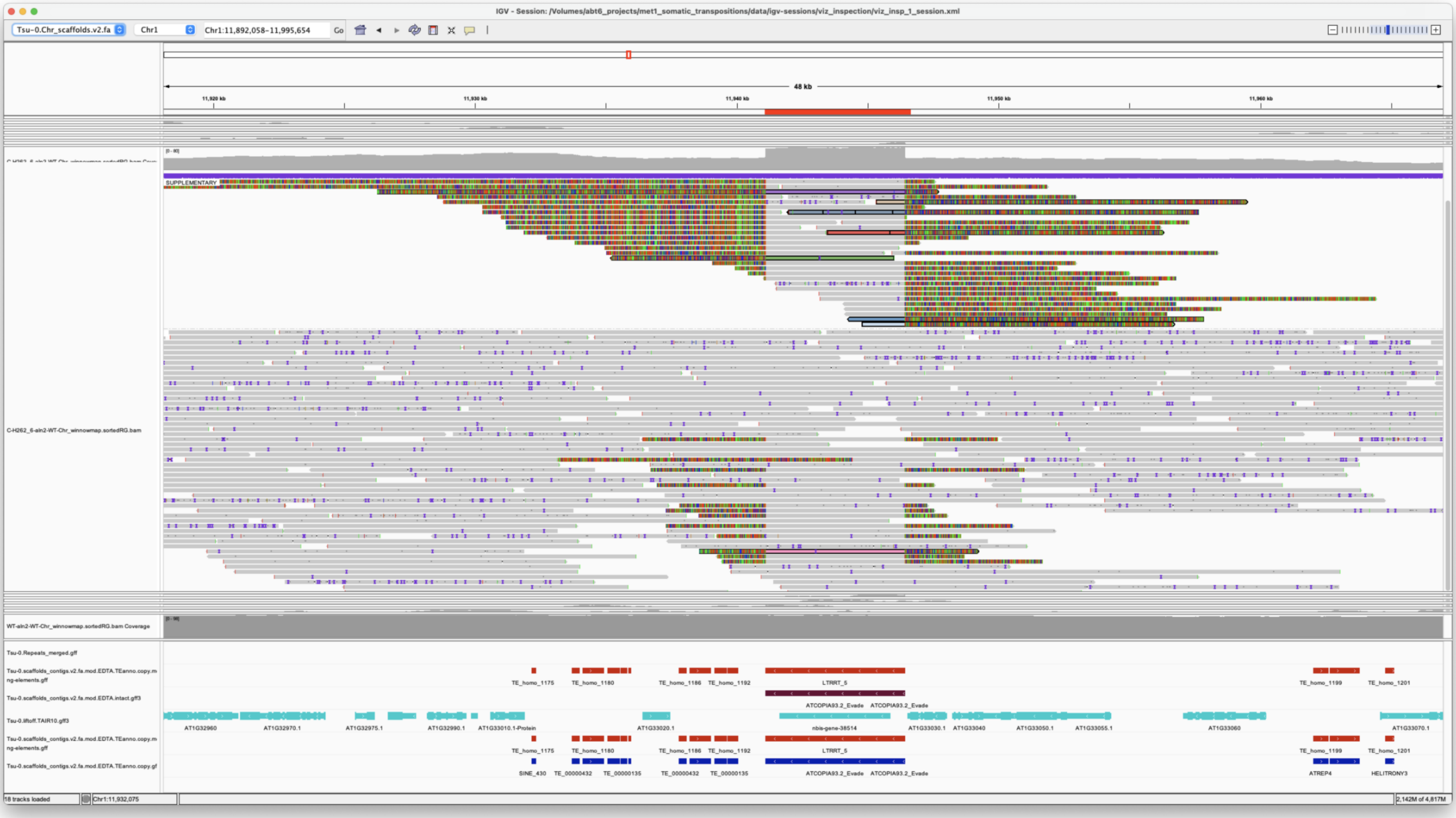

Partial

Confirmed

Chr5 1431423 1431423 - 1 Chr1;11941106;11946436;ATCOPIA93_Evade m64079_240212_113350/144902566/ccs met1_06

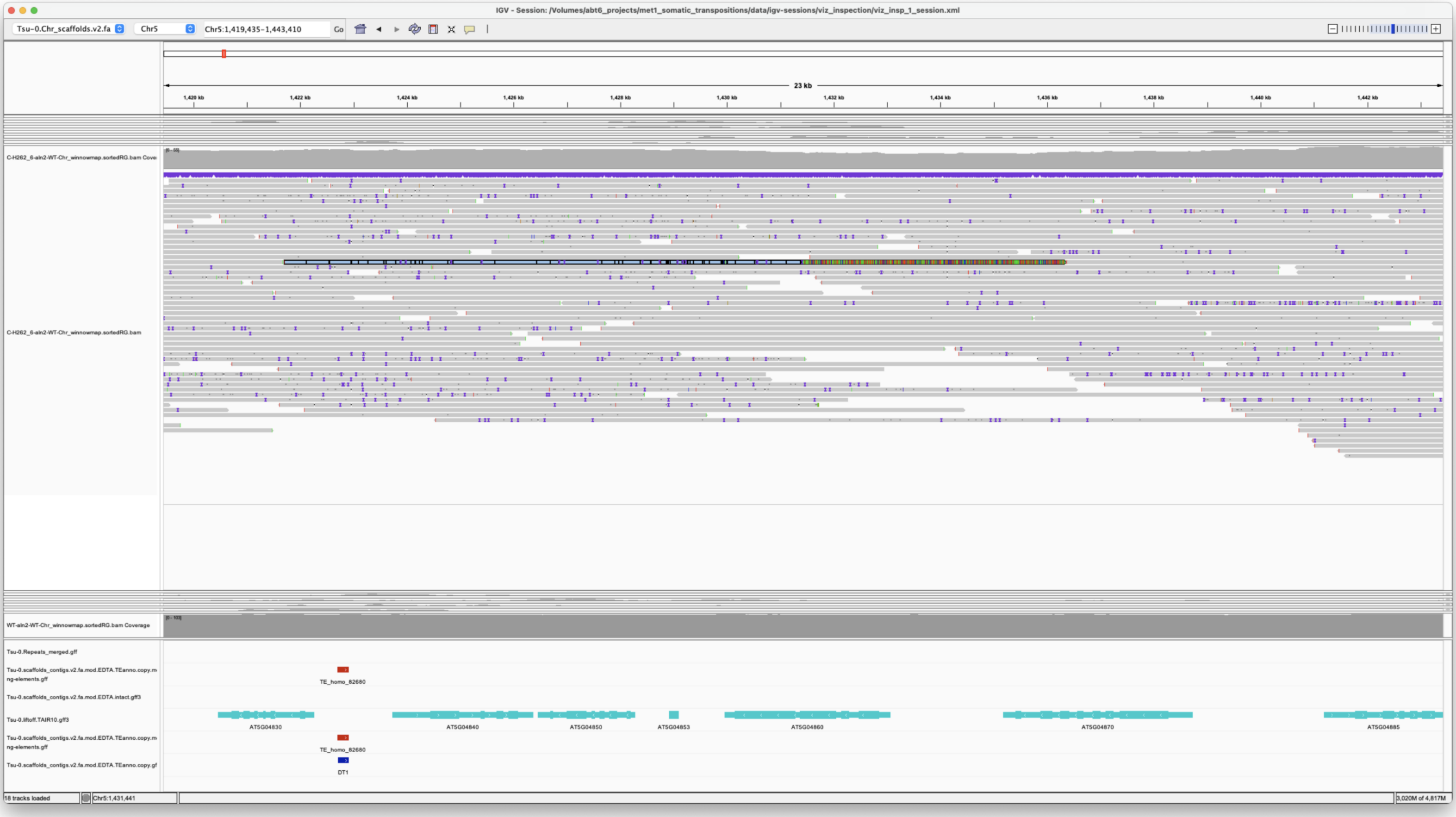

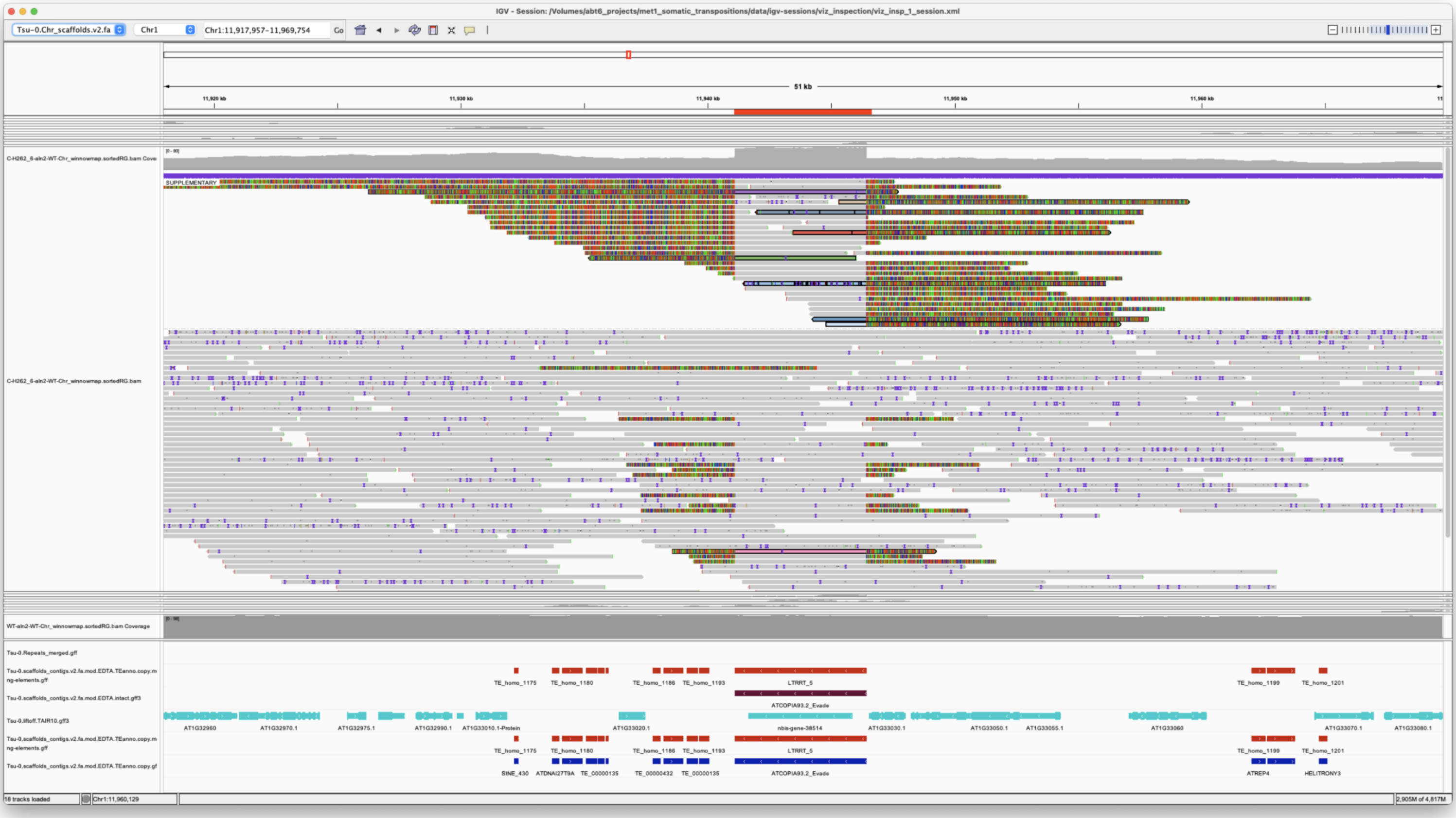

Partial

Confirmed

> Chr5 2092171 2092173 -,+,+,-,+,+,-,-,+,-,+,-,-,-,+,-,- 17
> Chr1;11941106;11946436;ATCOPIA93_Evade,Chr1;11941106;11946436;ATCOPIA93_Evade,Chr1;11941106;11946436;ATCOPIA93_Evade,Chr1;11941106;11946436;ATCOPIA93_Evade,Chr1;11941106;11946436;ATCOPIA93_Evade,Chr1;11941106;11946436;ATCOPIA93_Evade,Chr1;11941106;11946436;ATCOPIA93_Evade,Chr1;11941106;11946436;ATCOPIA93_Evade,Chr1;11941106;11946436;ATCOPIA93_Evade,Chr1;11941106;11946436;ATCOPIA93_Evade,Chr1;11941106;11946436;ATCOPIA93_Evade,Chr1;11941106;11946436;ATCOPIA93_Evade,Chr1;11941106;11946436;ATCOPIA93_Evade,Chr1;11941106;11946436;ATCOPIA93_Evade,Chr1;11941106;11946436;ATCOPIA93_Evade,Chr1;11941106;11946436;ATCOPIA93_Evade,Chr1;11941106;11946436;ATCOPIA93_Evade
> m64079_221220_112036/10617853/ccs,m64079_221220_112036/115280025/ccs,m64079_221220_112036/128780413/ccs,m64079_221220_112036/139004125/ccs,m64079_221220_112036/169477972/ccs,m64079_221220_112036/23266803/ccs,m64079_221220_112036/2820392/ccs,m64079_221220_112036/29165174/ccs,m64079_221220_112036/40699716/ccs,m64079_221220_112036/64357854/ccs,m64079_221220_112036/68419964/ccs,m64079_240212_113350/1051213/ccs,m64079_240212_113350/112198306/ccs,m64079_240212_113350/170198066/ccs,m64079_240212_113350/1902341/ccs,m64079_240212_113350/54200326/ccs,m64079_240212_113350/76546947/ccs met1_06

> Chr5 2092178 2092180 -,-,-,-,+,-,-,- 8
> Chr1;11941106;11946436;ATCOPIA93_Evade,Chr1;11941106;11946436;ATCOPIA93_Evade,Chr1;11941106;11946436;ATCOPIA93_Evade,Chr1;11941106;11946436;ATCOPIA93_Evade,Chr1;11941106;11946436;ATCOPIA93_Evade,Chr1;11941106;11946436;ATCOPIA93_Evade,Chr1;11941106;11946436;ATCOPIA93_Evade,Chr1;11941106;11946436;ATCOPIA93_Evade
> m64079_221220_112036/10225440/ccs,m64079_221220_112036/137887894/ccs,m64079_221220_112036/152306417/ccs,m64079_221220_112036/173999879/ccs,m64079_221220_112036/95683723/ccs,m64079_240212_113350/111936583/ccs,m64079_240212_113350/21234447/ccs,m64079_240212_113350/77202747/ccs met1_06

UNCLEAR IF IT IS TWO OR ONE INSERTIONS, OR ONE INSERTION PLUS REARRANGEMENT
Because different insertion directions.

ALSO THERE ARE UNDETECTED INSERTIONS!!

m64079_221220_112036/10617853/ccs -

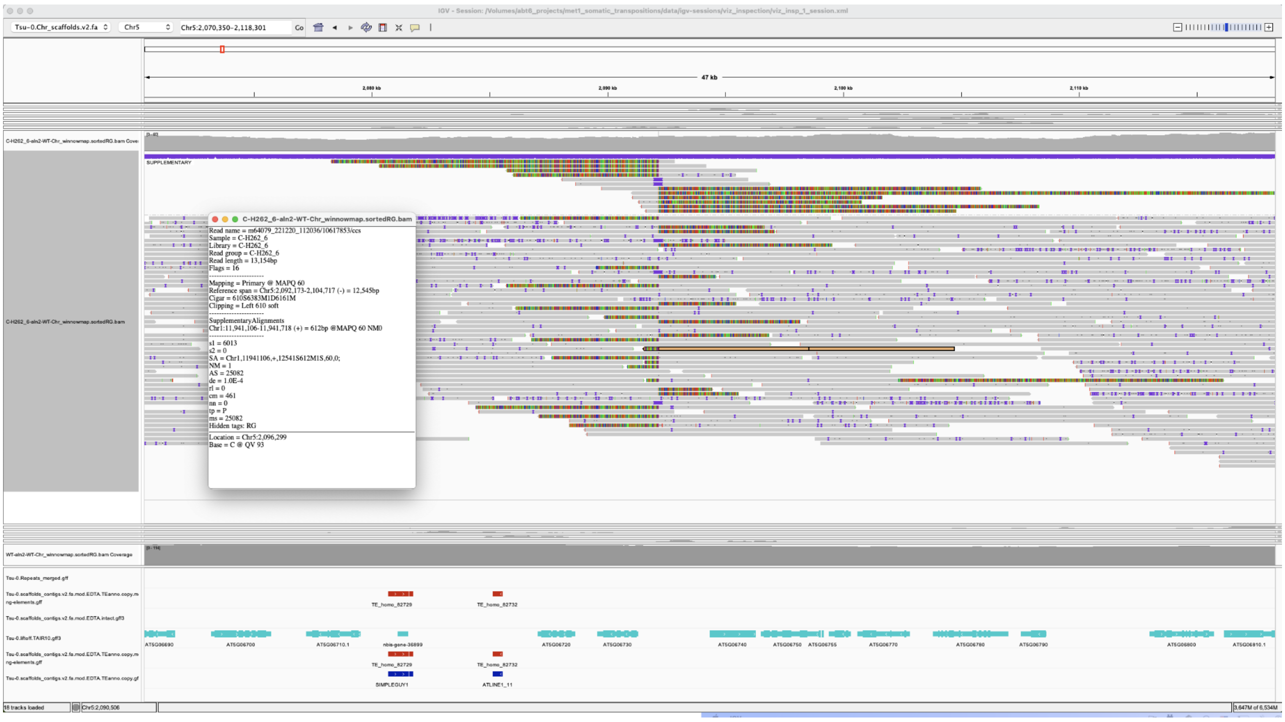

m64079_221220_112036/115280025/ccs -

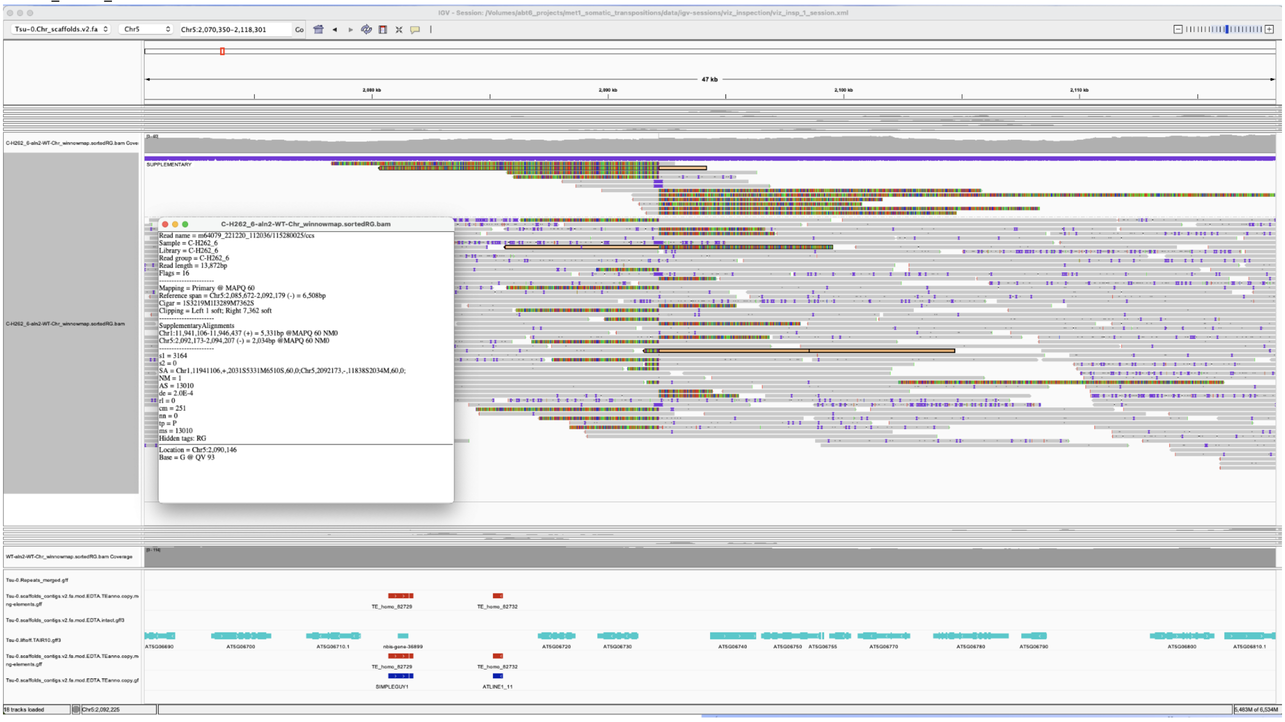

m64079_221220_112036/128780413/ccs -

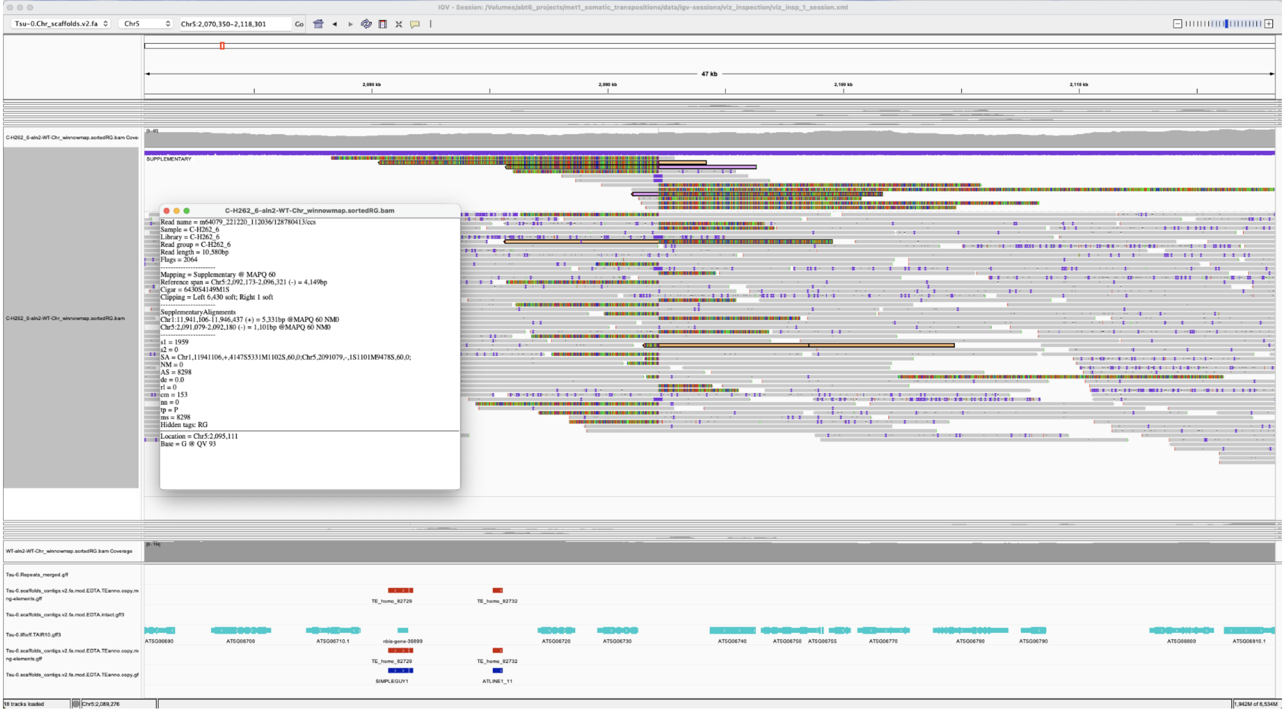

m64079_221220_112036/139004125/ccs -

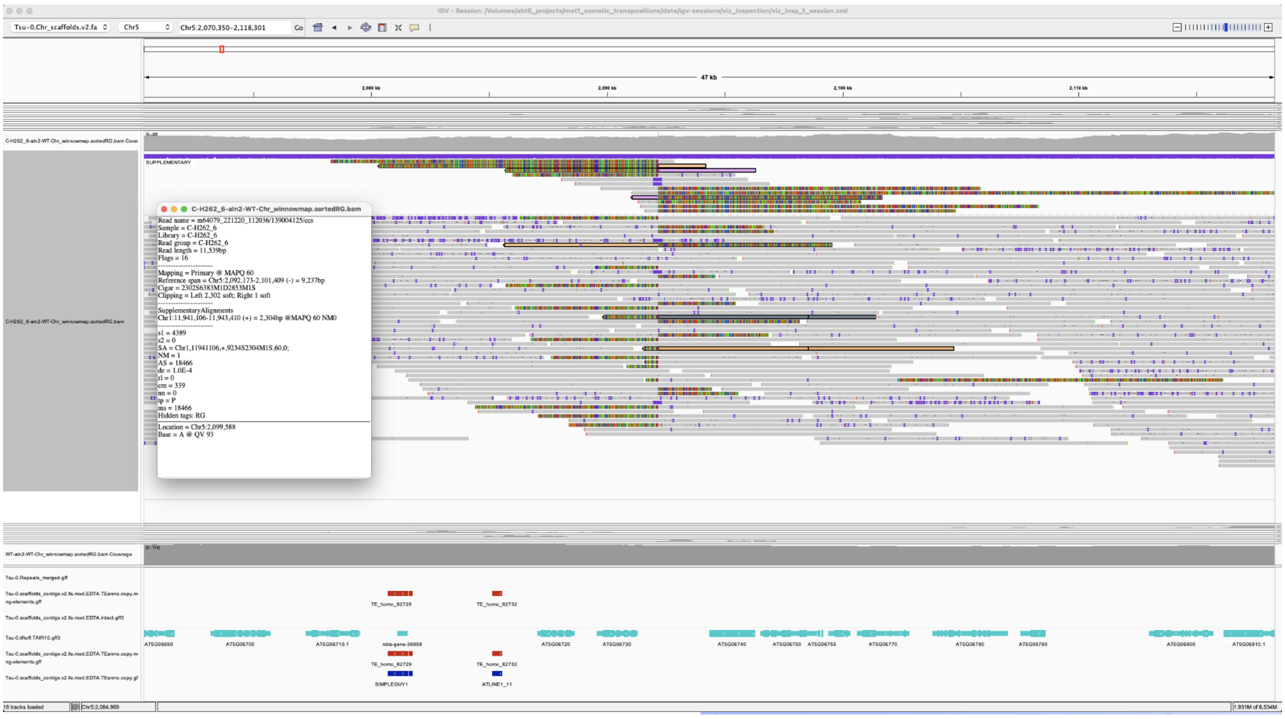

m64079_221220_112036/169477972/ccs -

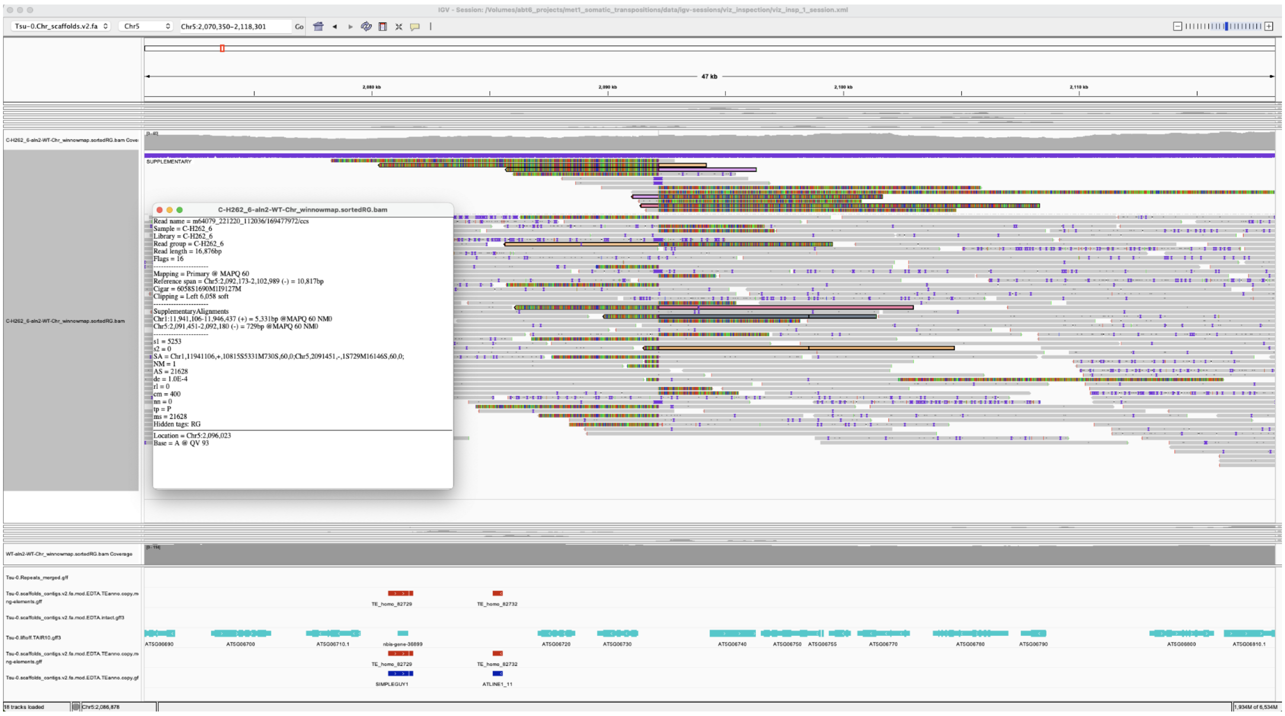

m64079_221220_112036/23266803/ccs -

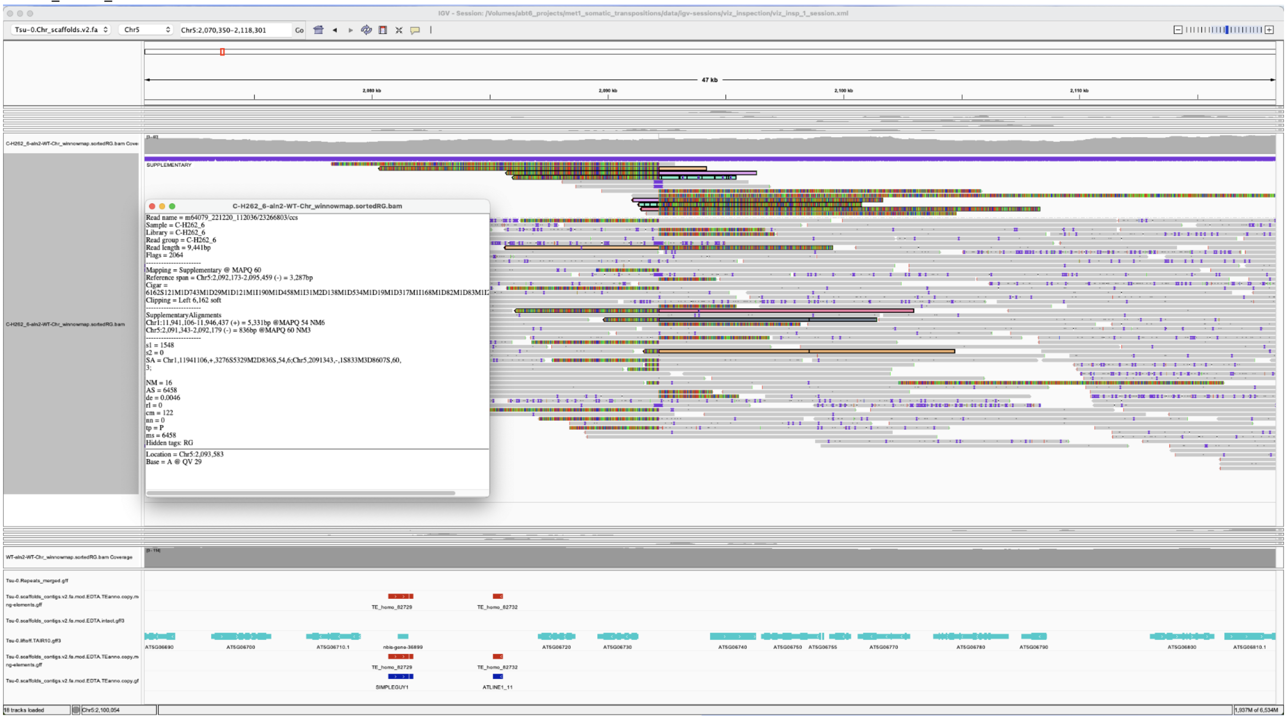

m64079_221220_112036/2820392/ccs -

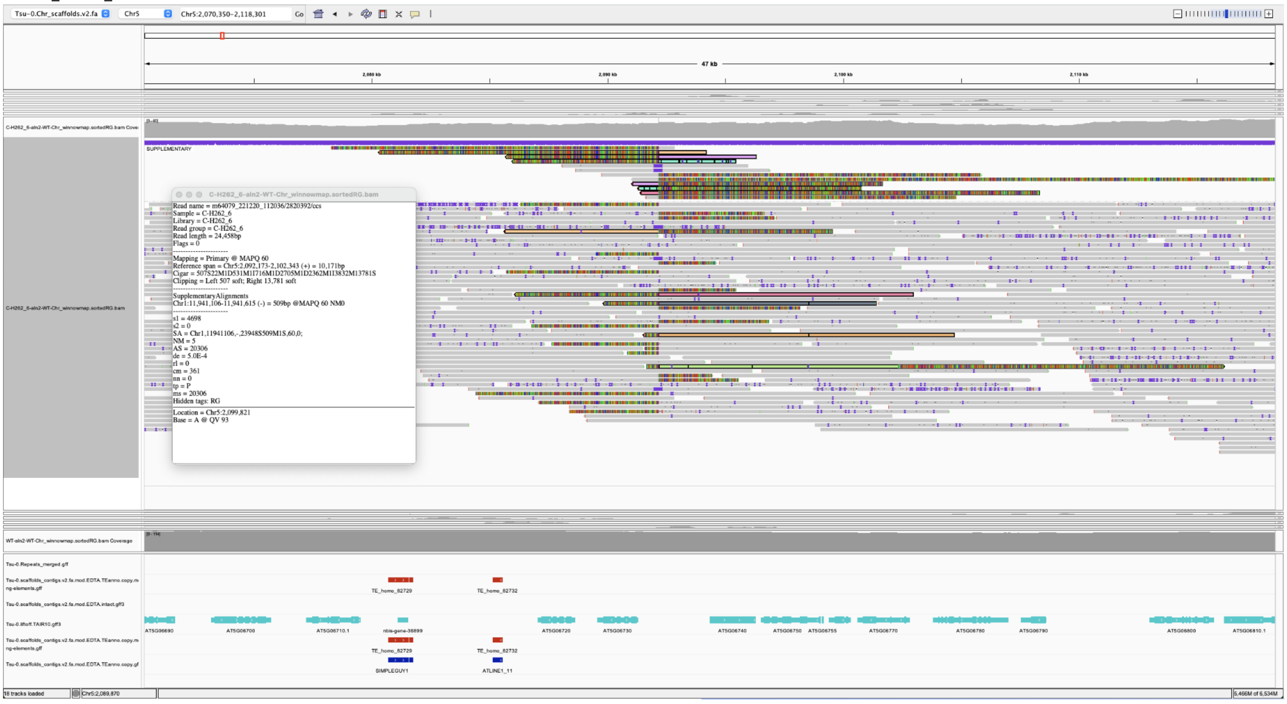

^^^ There is 13kb of right clipped read that is not mapping to anything! WTF!!!

Exclude

m64079_221220_112036/29165174/ccs -

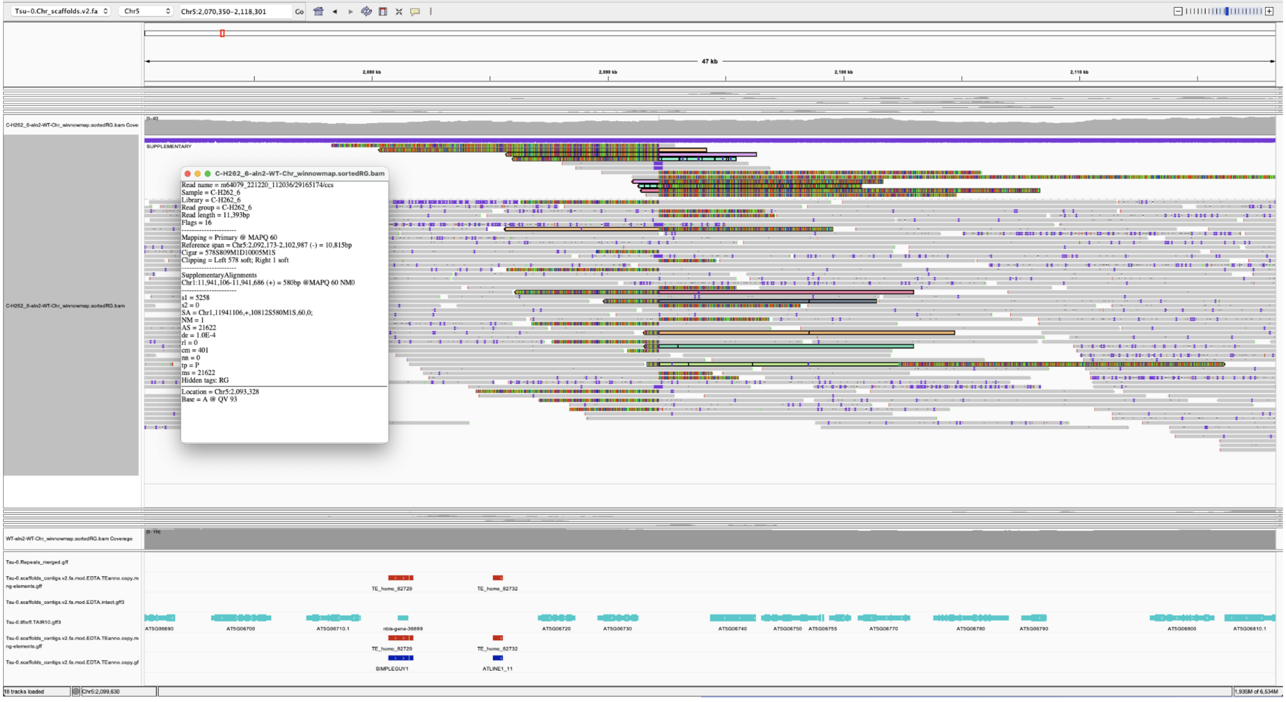

m64079_221220_112036/40699716/ccs -

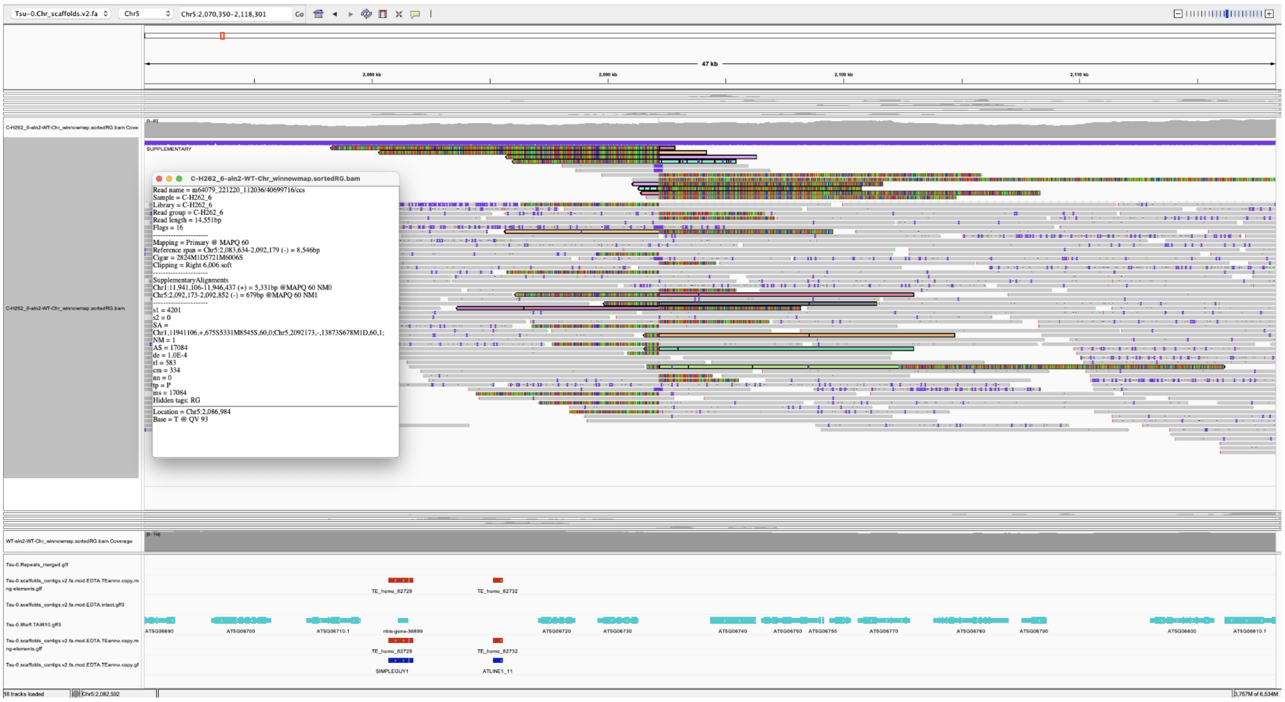

m64079_221220_112036/64357854/ccs -

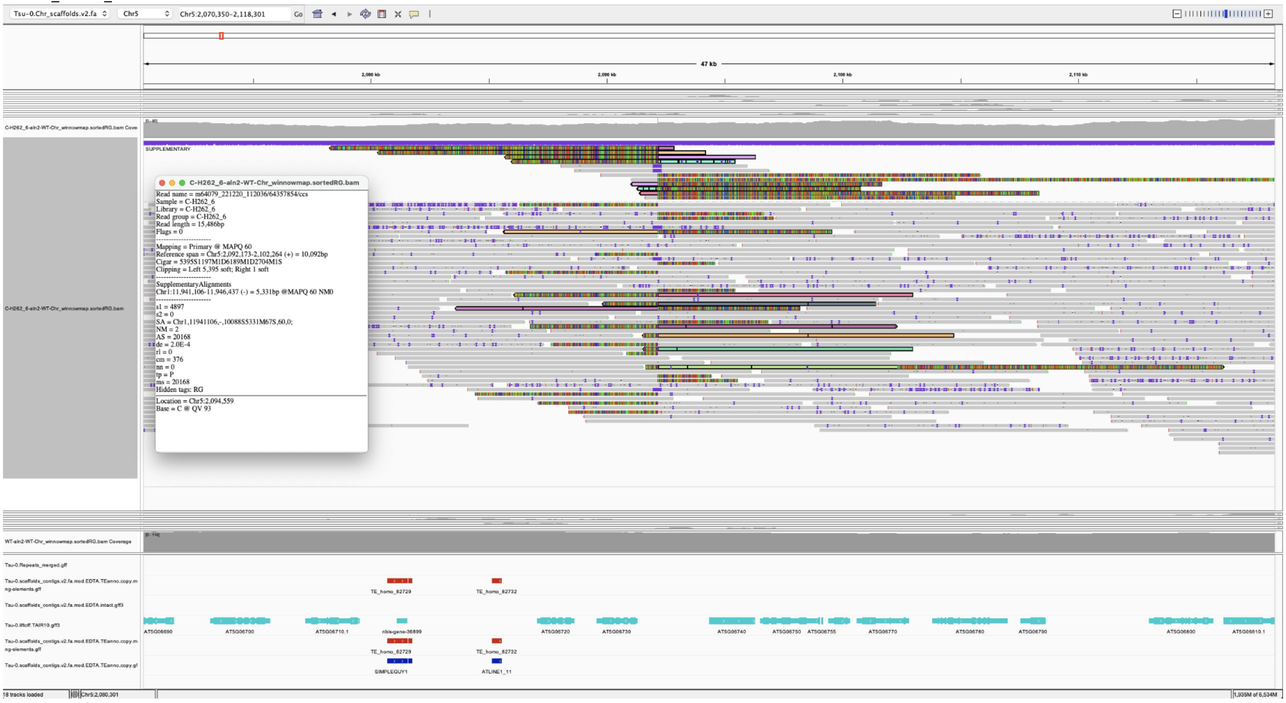

m64079_221220_112036/68419964/ccs -

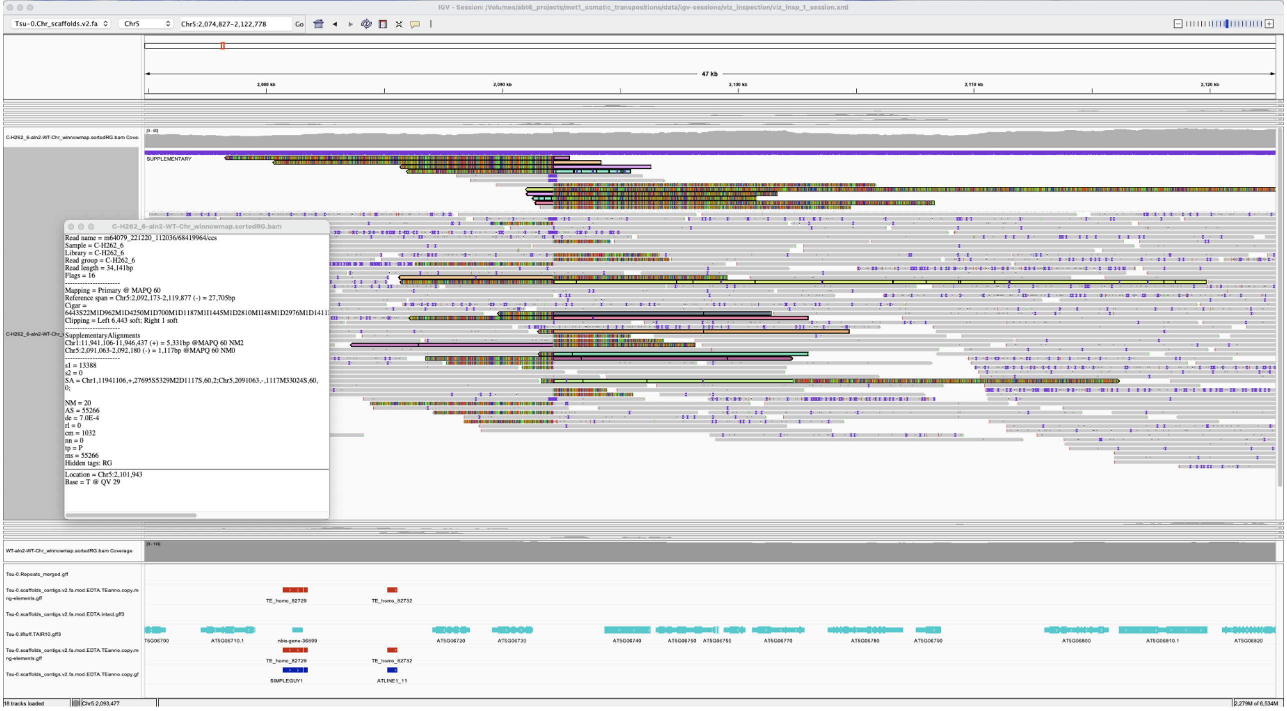

m64079_240212_113350/1051213/ccs -

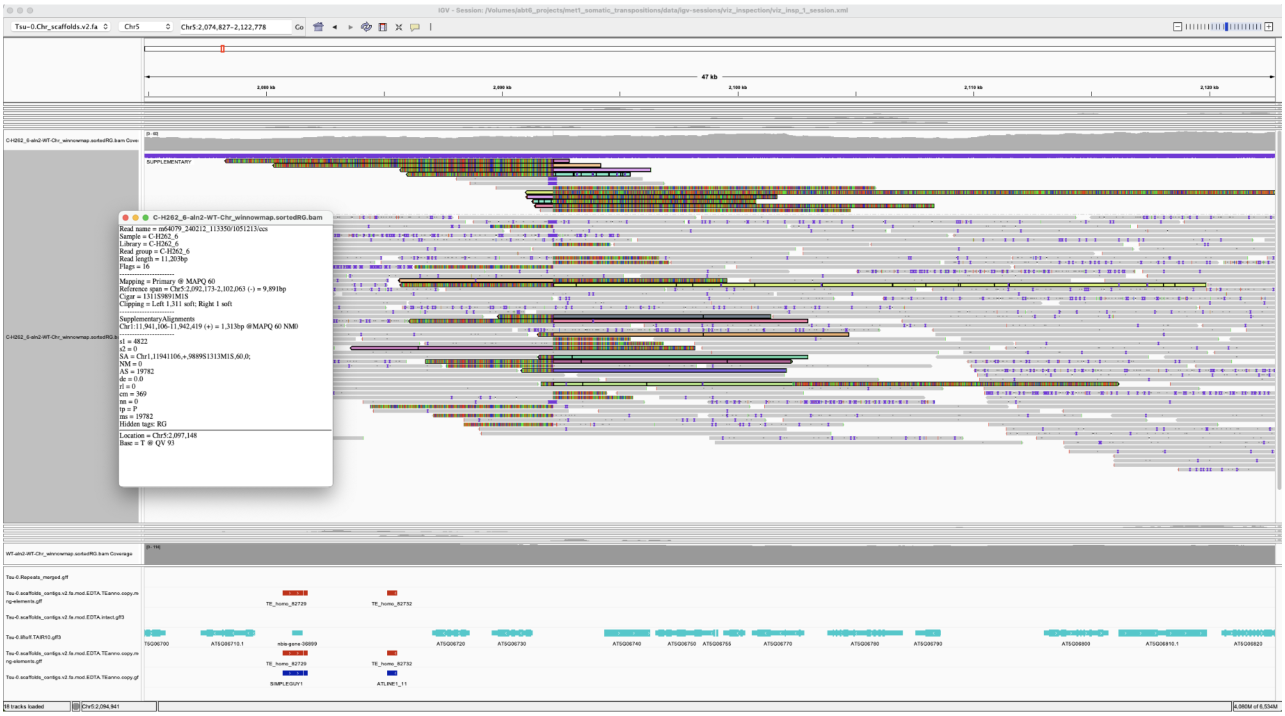

m64079_240212_113350/112198306/ccs

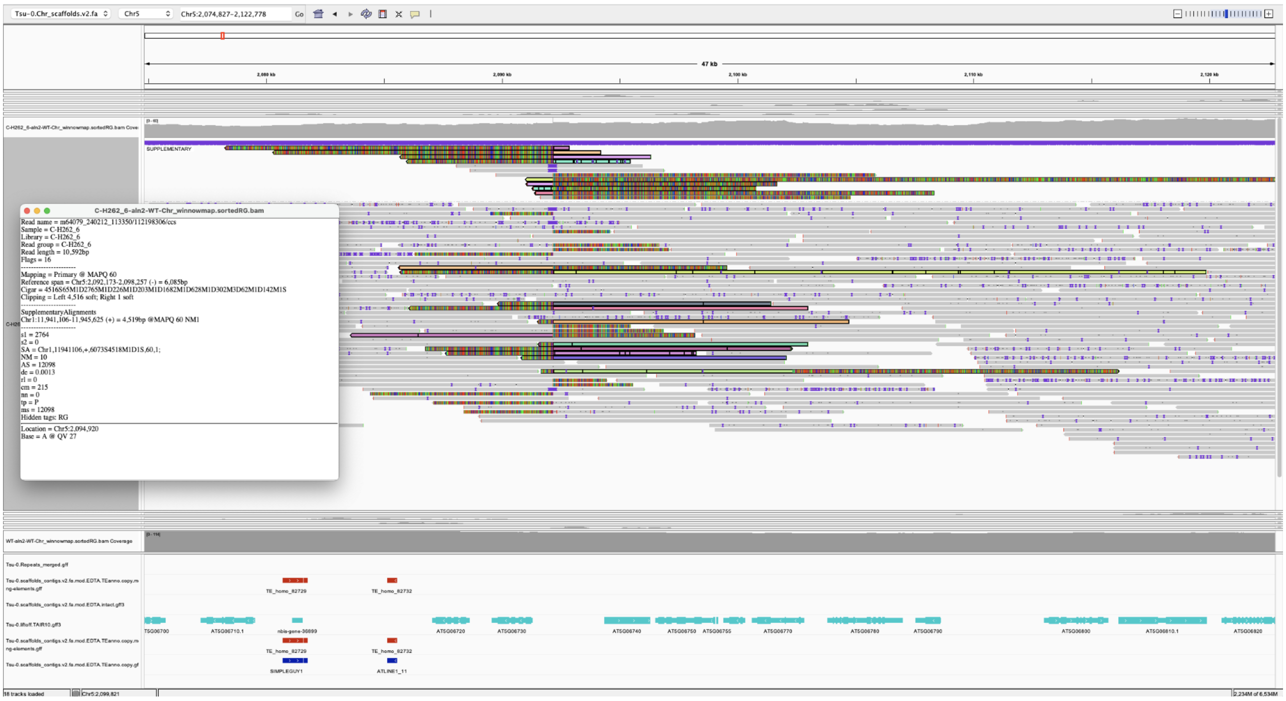

m64079_240212_113350/170198066/ccs -

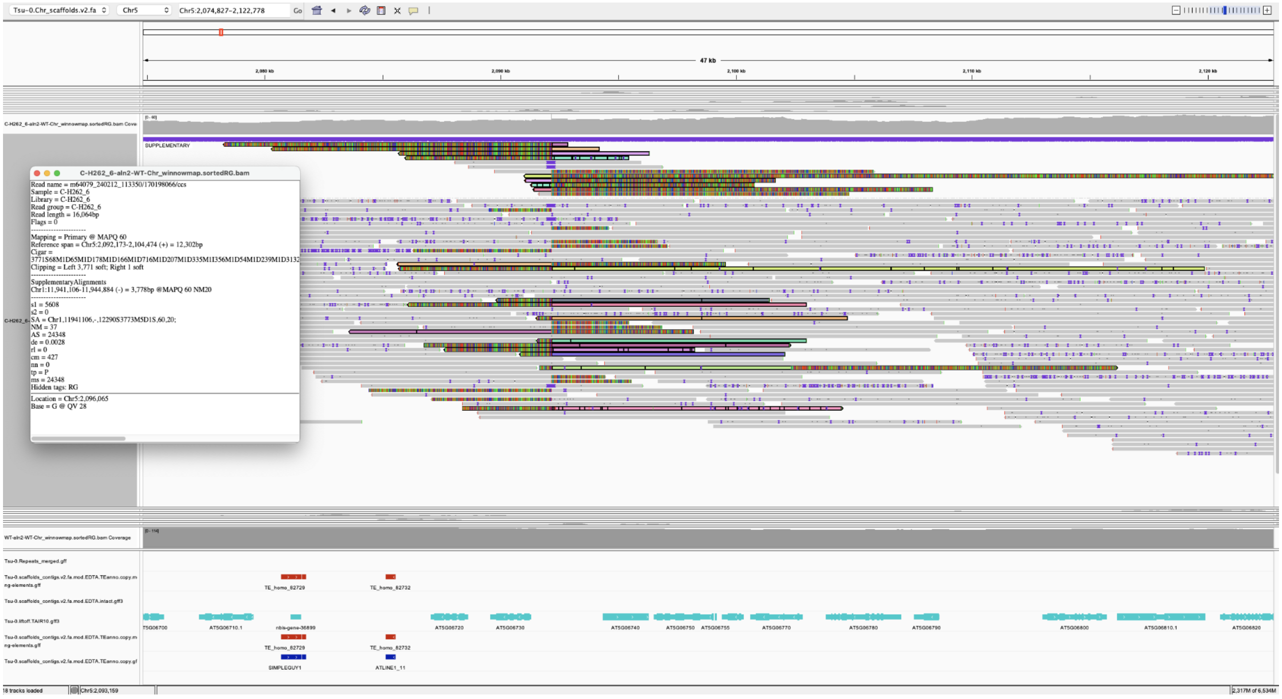

m64079_240212_113350/1902341/ccs -

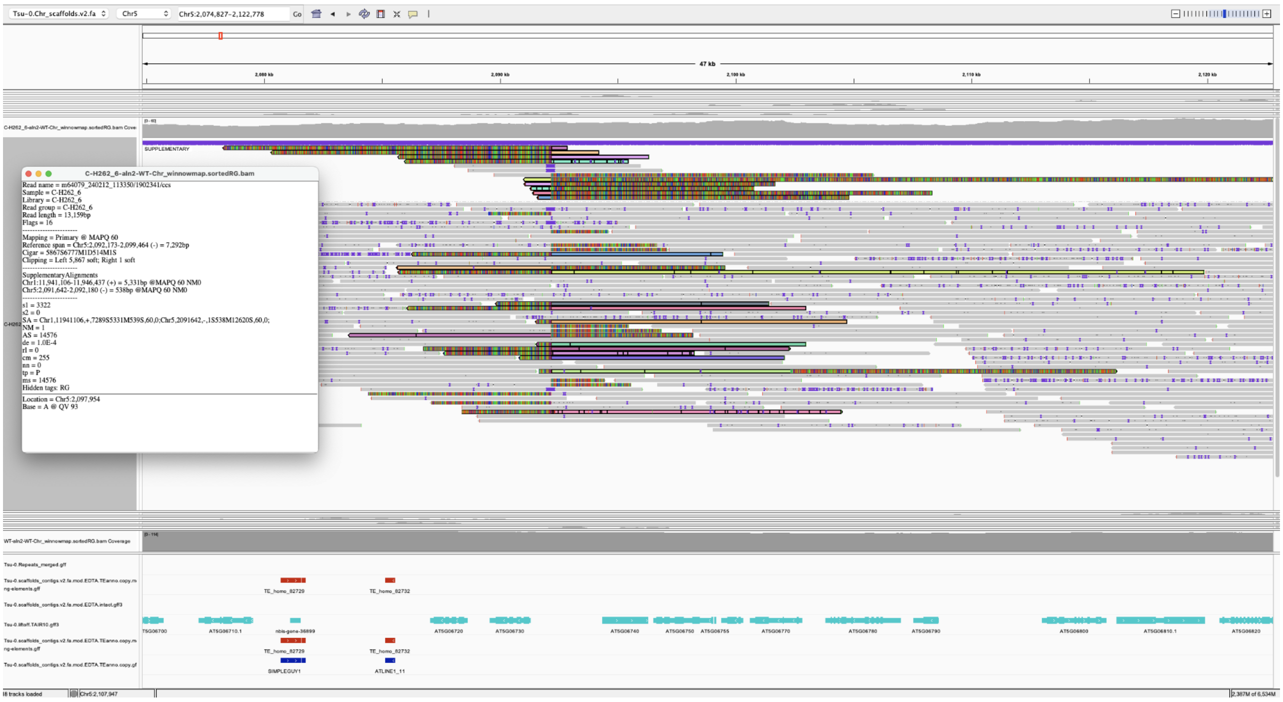

m64079_240212_113350/54200326/ccs

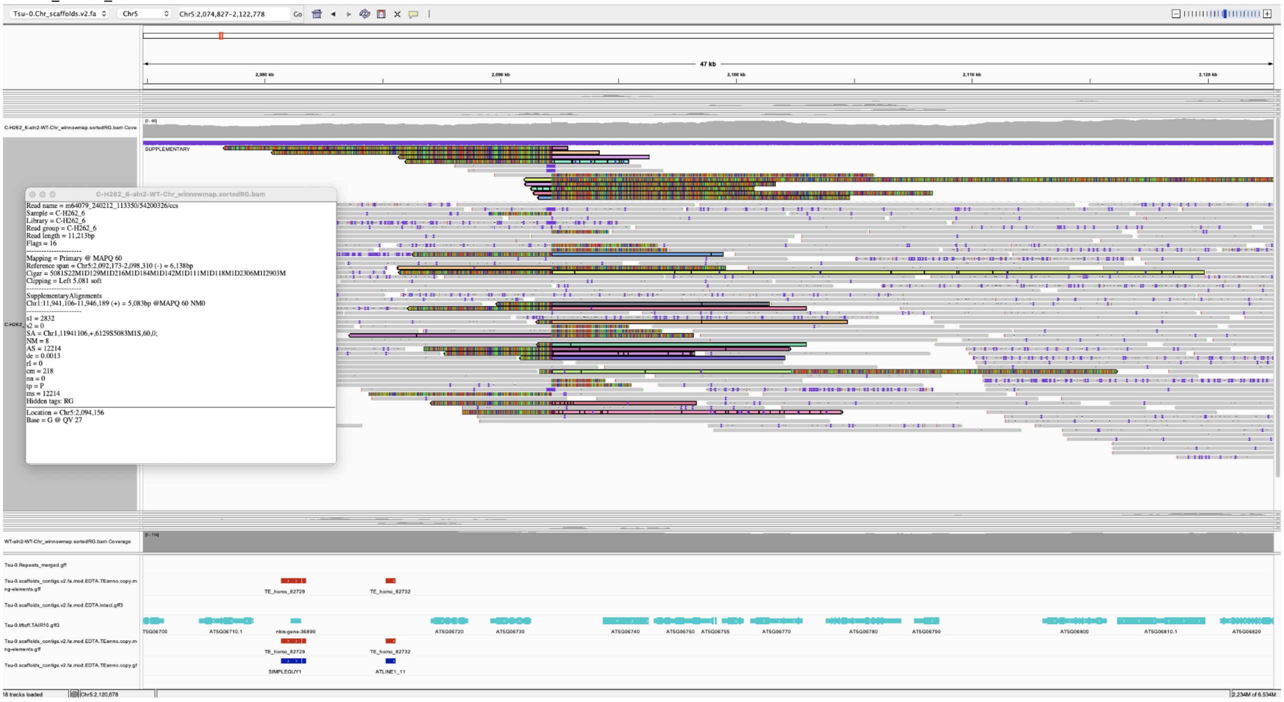

m64079_240212_113350/76546947/ccs

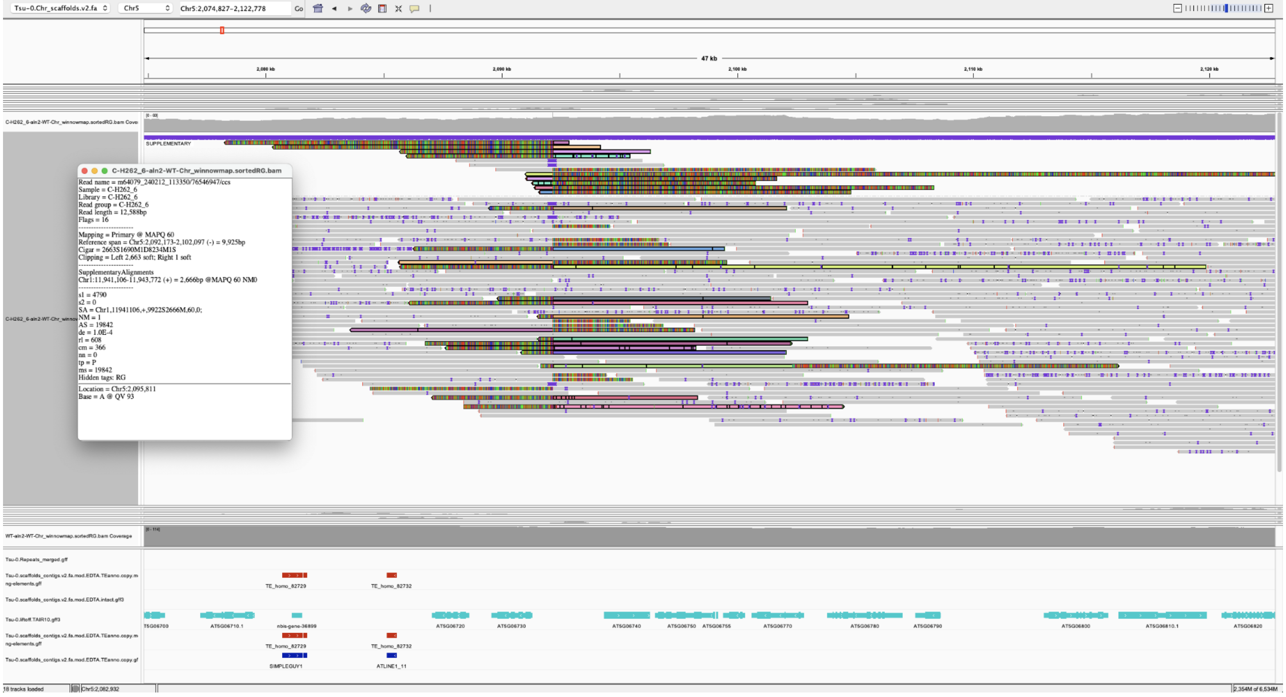

m64079_221220_112036/10225440/ccs

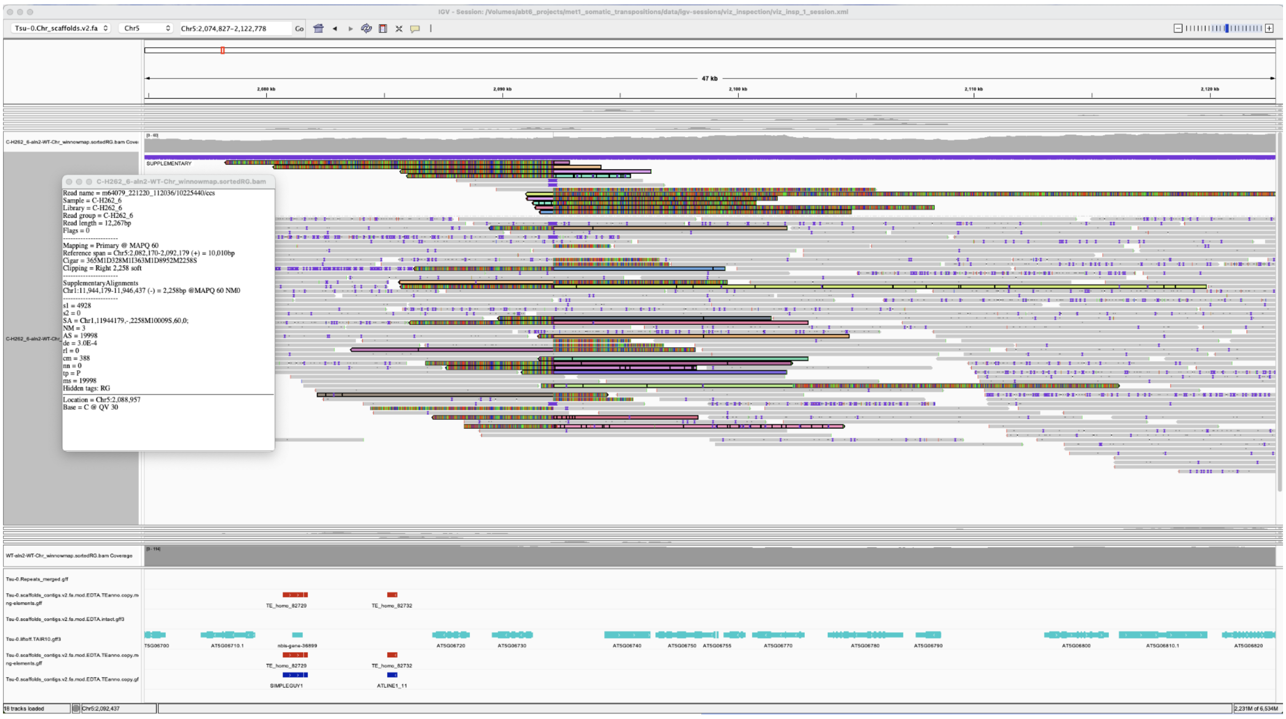

m64079_221220_112036/137887894/ccs

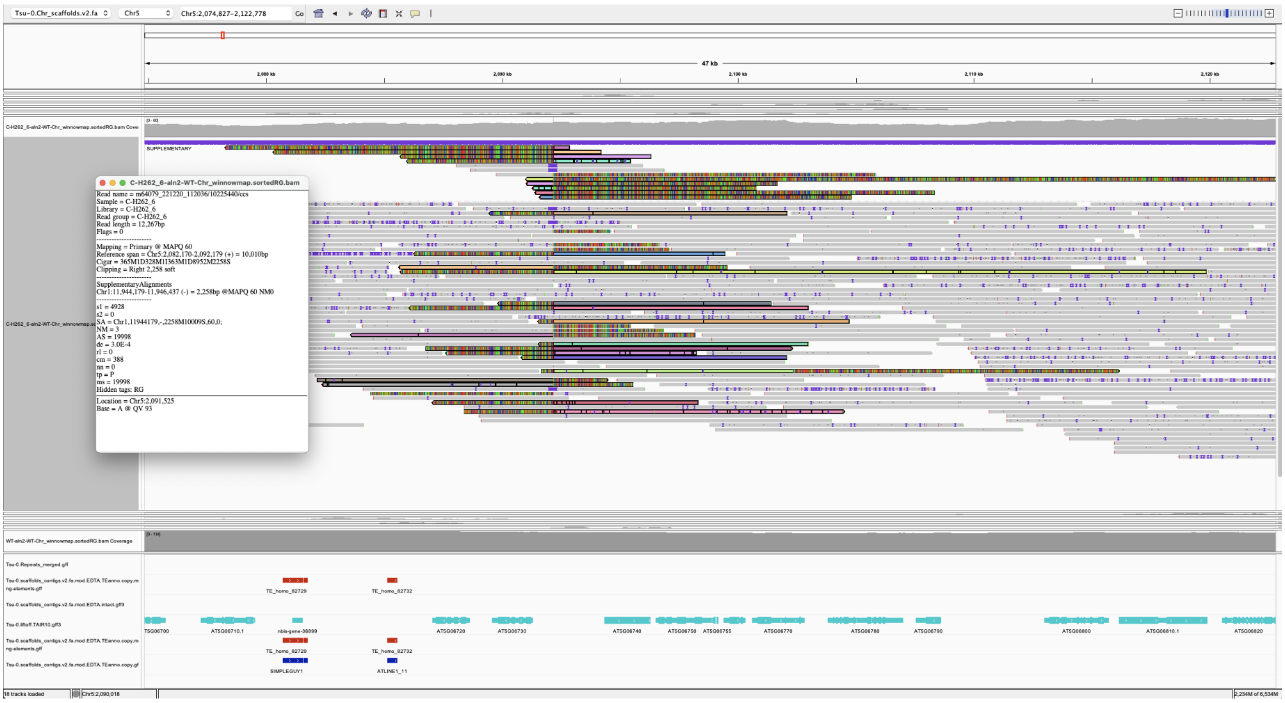

m64079_221220_112036/152306417/ccs,

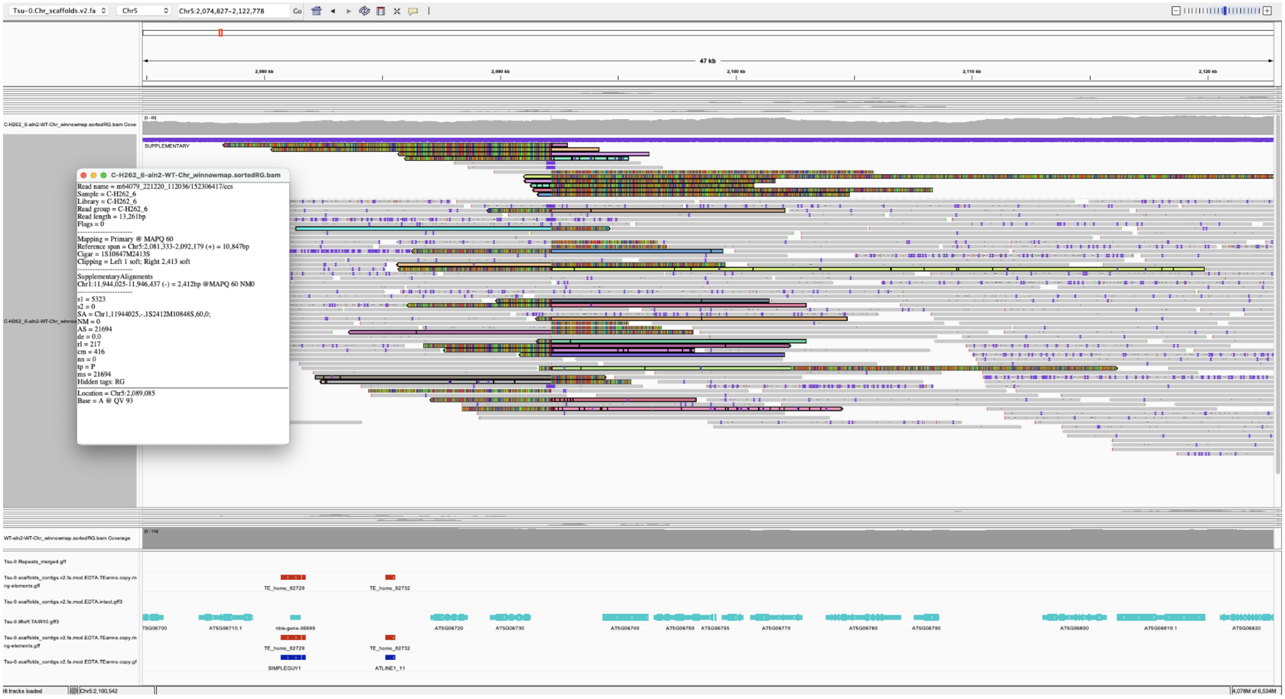

m64079_221220_112036/173999879/ccs

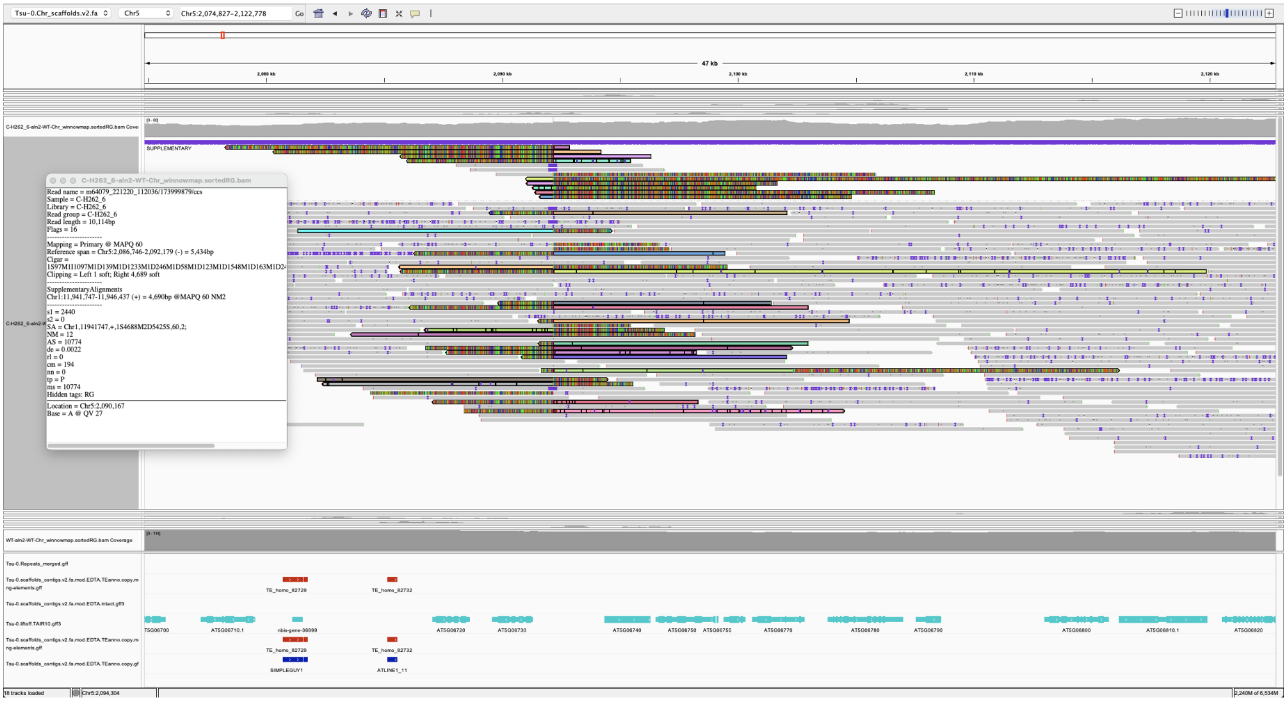

m64079_221220_112036/95683723/ccs

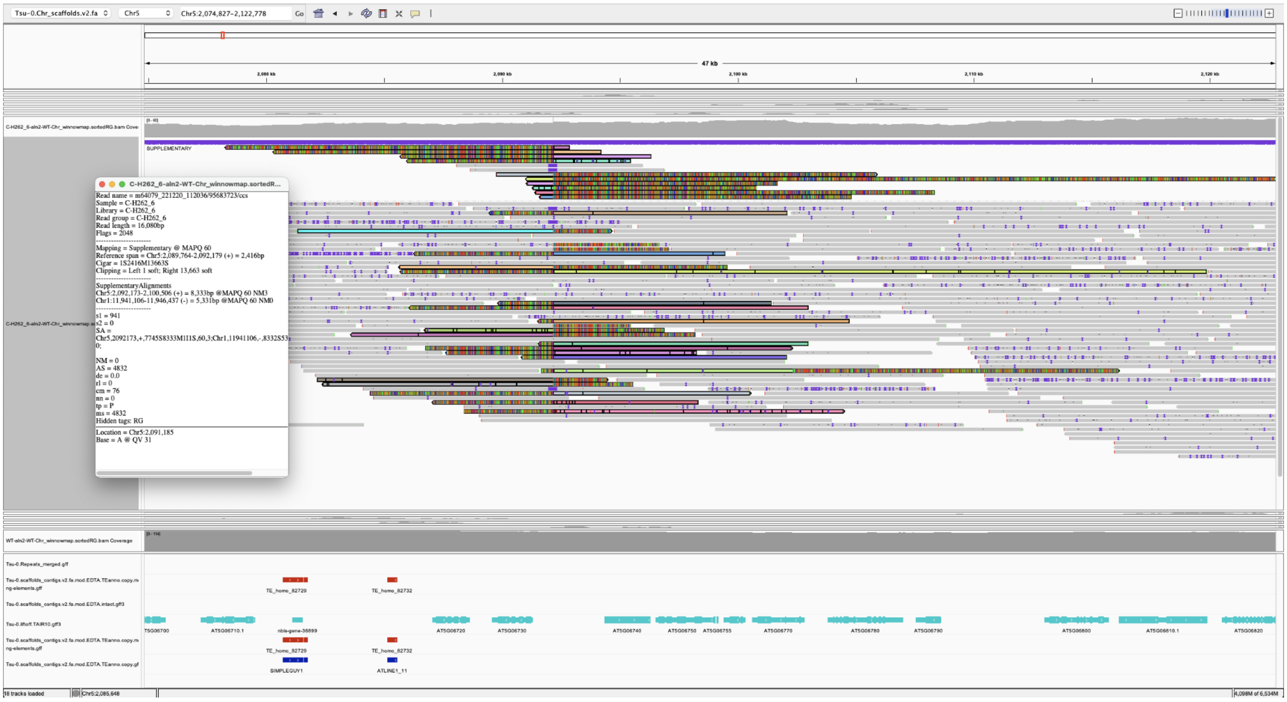

m64079_240212_113350/111936583/ccs

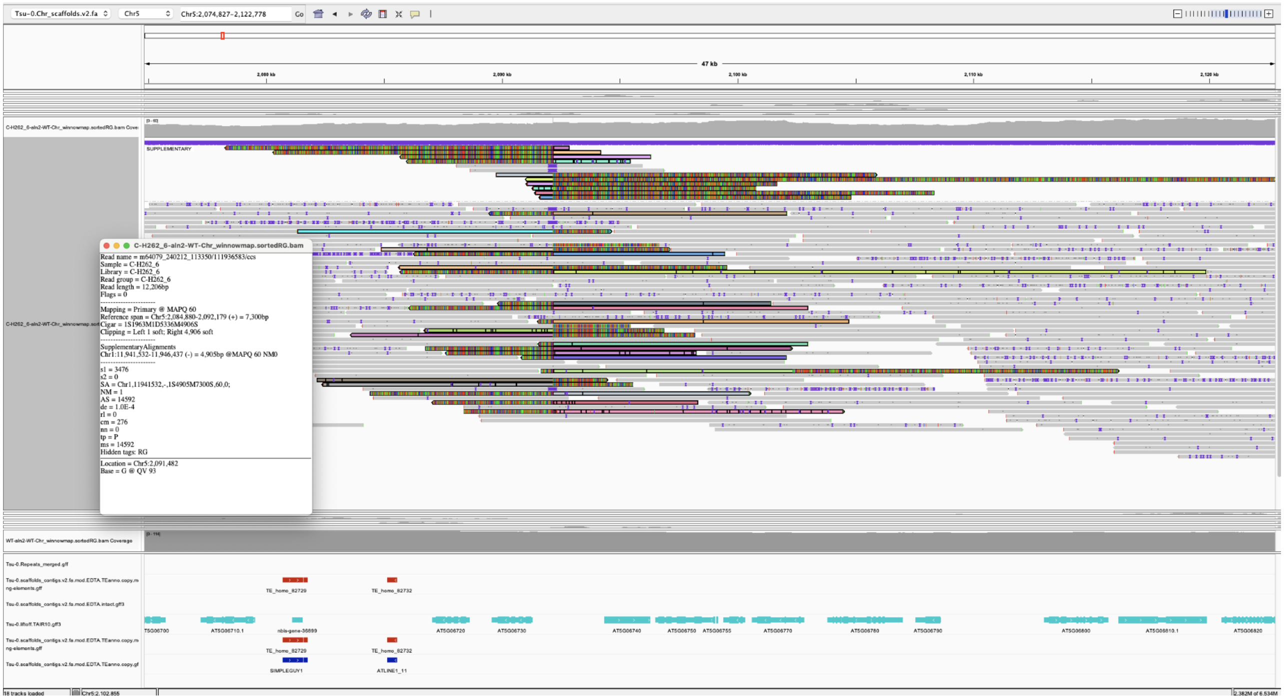

m64079_240212_113350/21234447/ccs

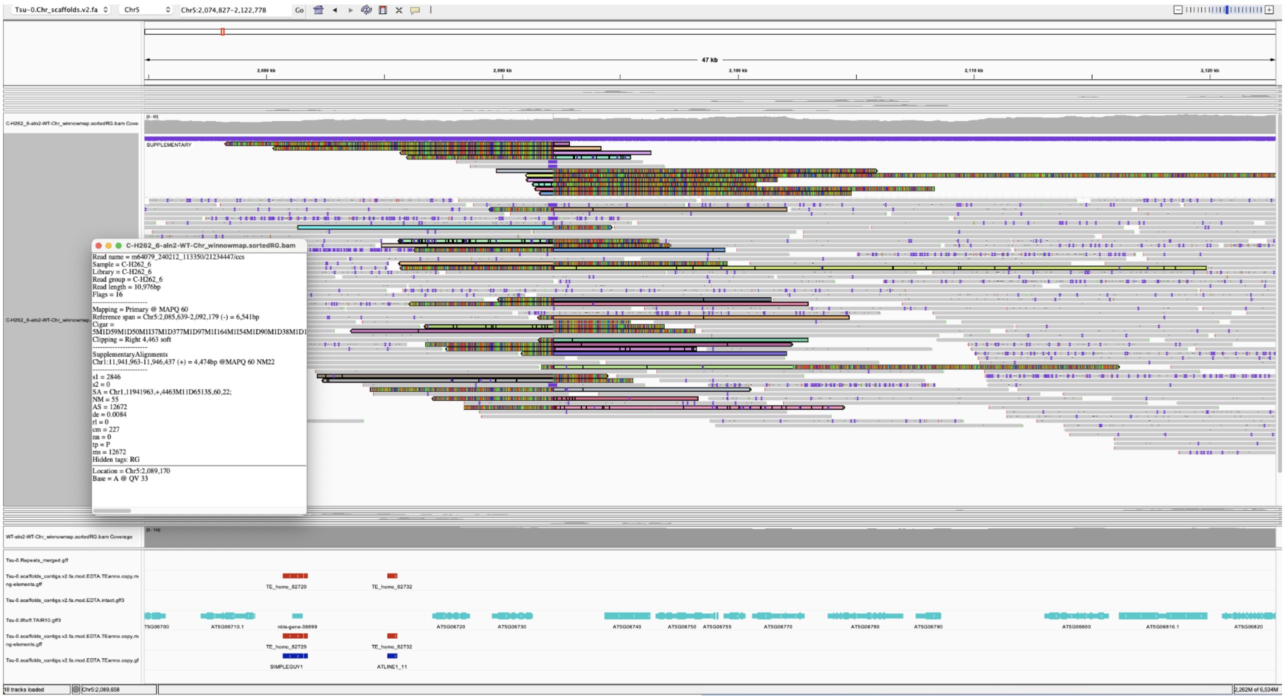

m64079_240212_113350/77202747/ccs

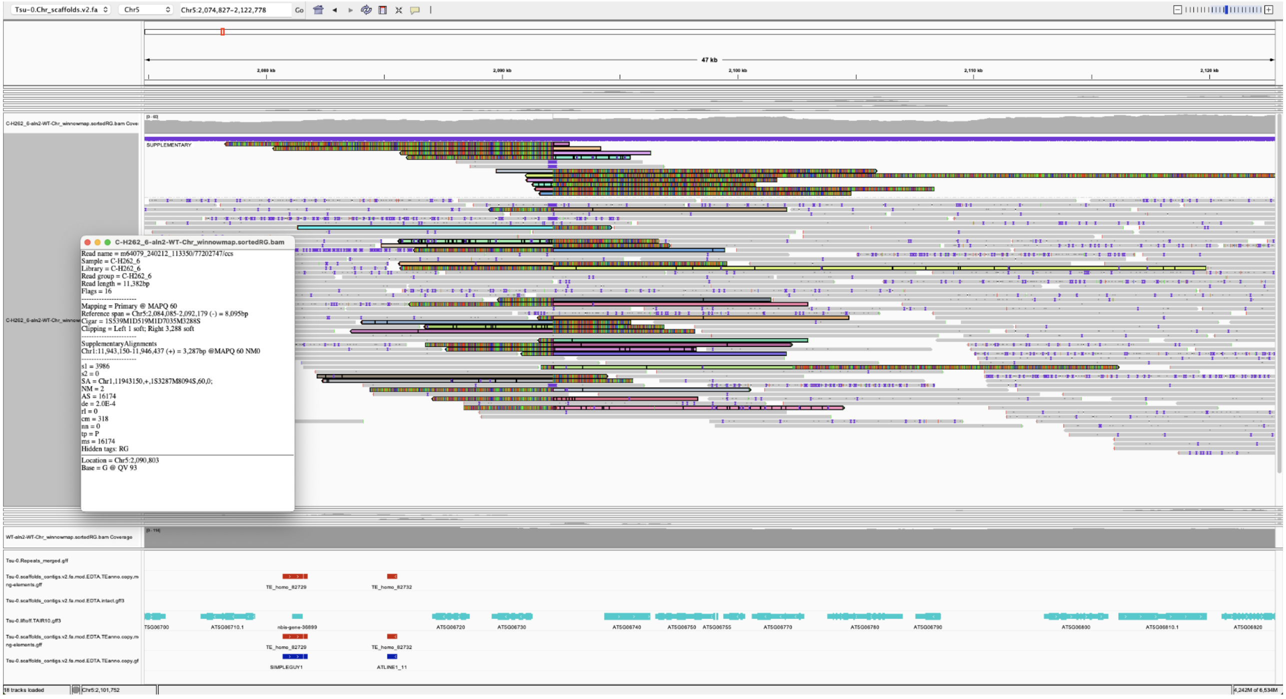

AND ADDITIONAL 5 READS WITH INSERTIONS

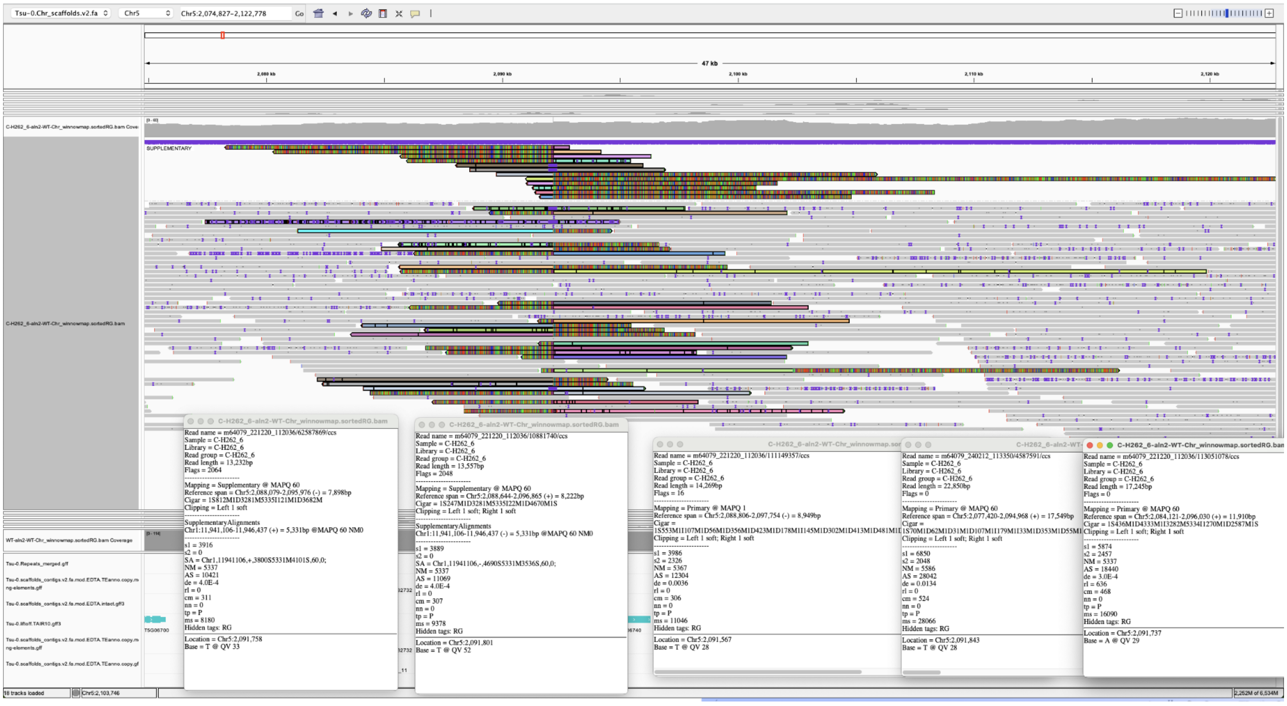

m64079_221220_112036/62587869/ccs,m64079_221220_112036/10881740/ccs,m64079_221220_112036/111149357/ccs,m64079_240212_113350/4587591/ccs,m64079_221220_112036/113051078/ccs

Mixed
(centrally and partially resolved insertions)
TSD

Chr5 10016551 10016551 + 1 Chr1;11941106;11946436;ATCOPIA93_Evade m64079_221220_112036/105122289/ccs met1_06

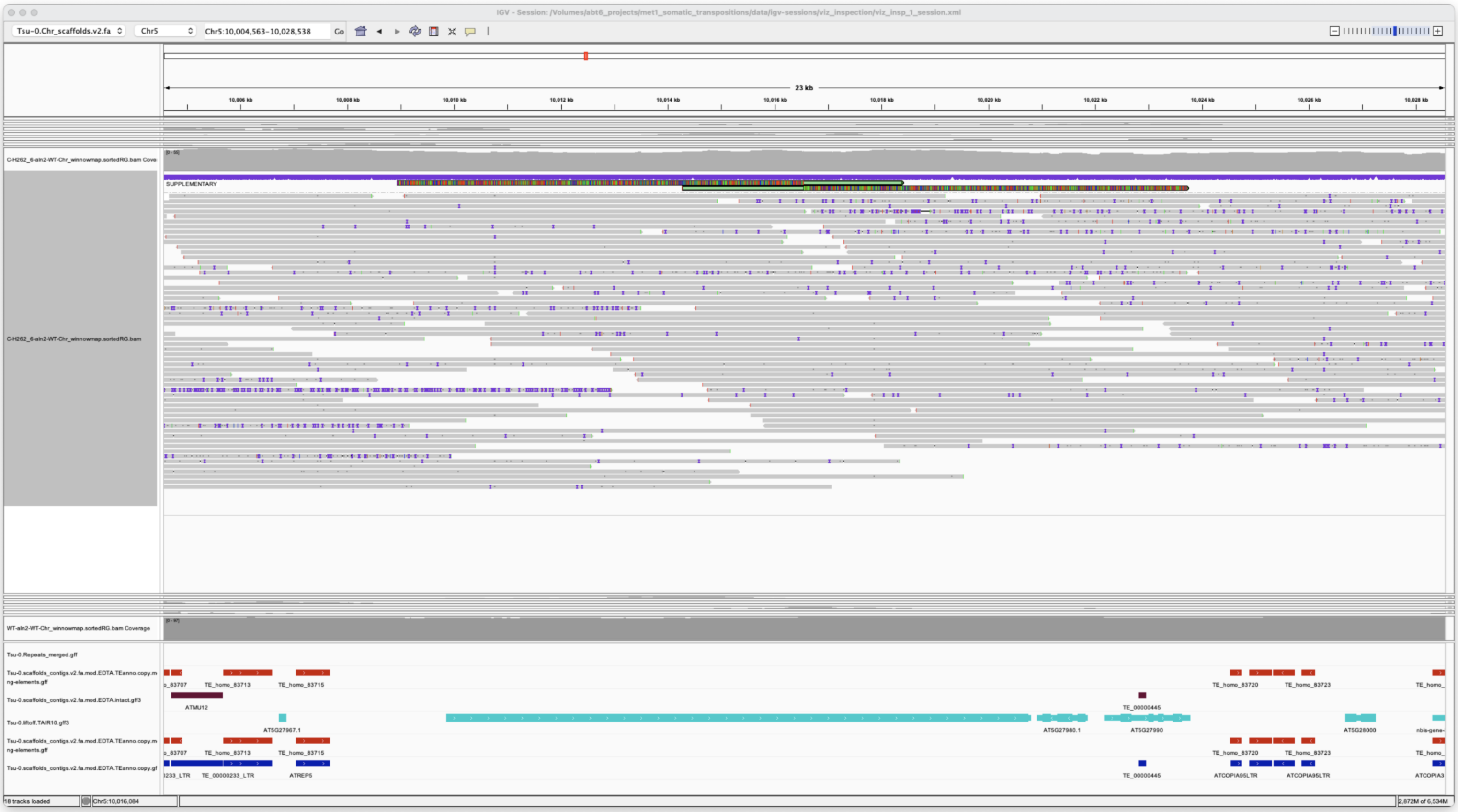

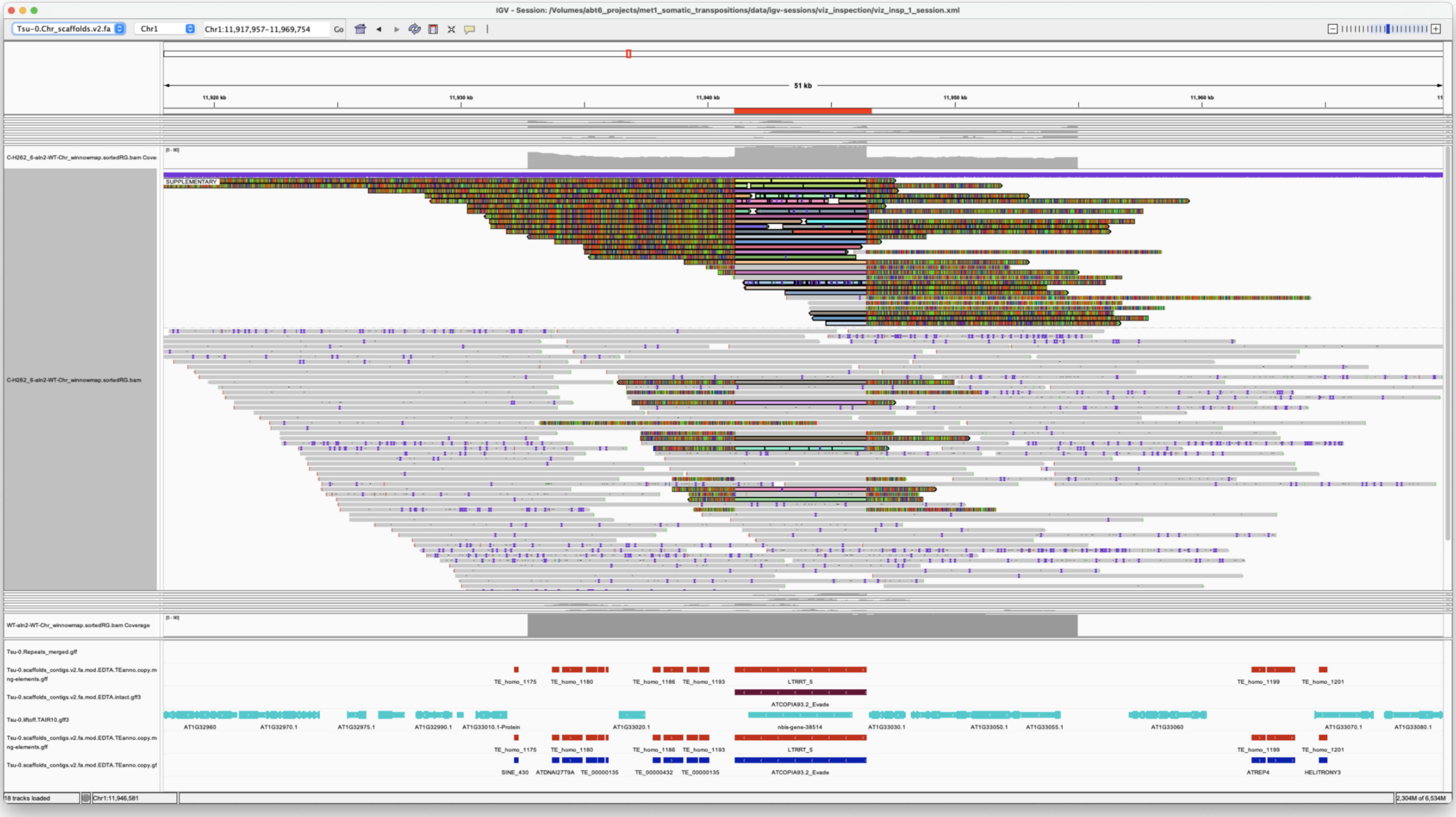

Central
TSD

Confirmed

Chr5 13897713 13897713 + 1 Chr1;11941106;11946436;ATCOPIA93_Evade m64079_221220_112036/42534900/ccs met1_06

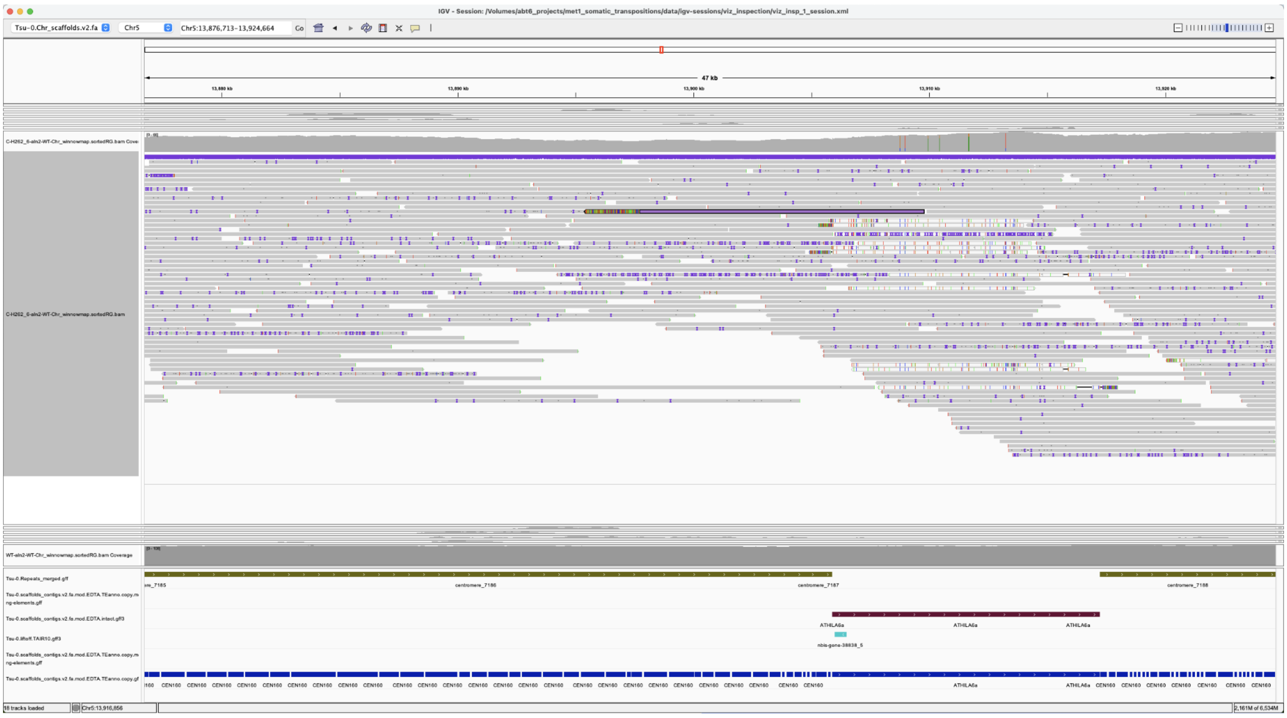

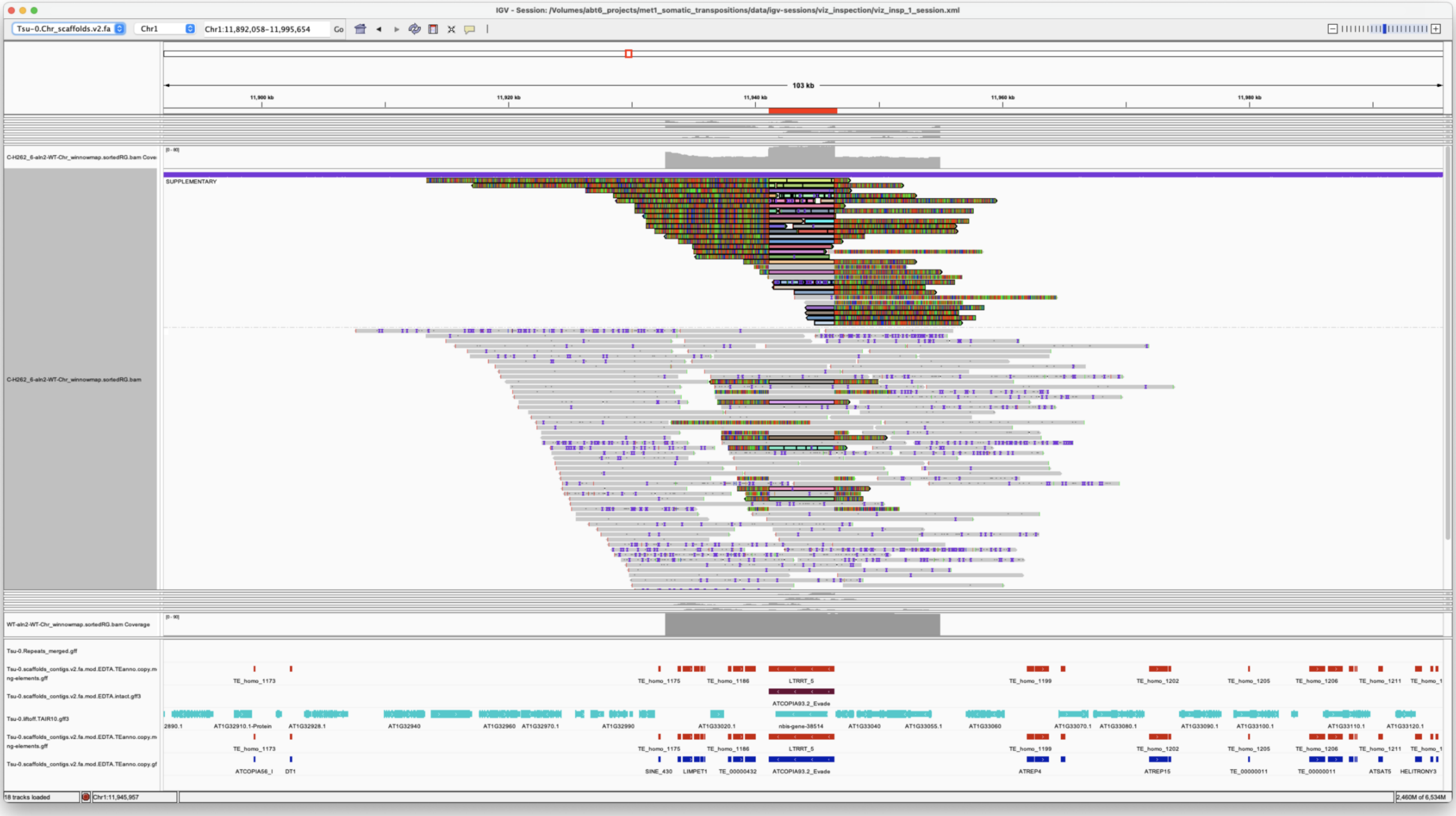

centromeric

Partial

Confirmed

Chr5 15260691 15260691 + 1 Chr1;11941106;11946436;ATCOPIA93_Evade m64079_221220_112036/87950948/ccs met1_06

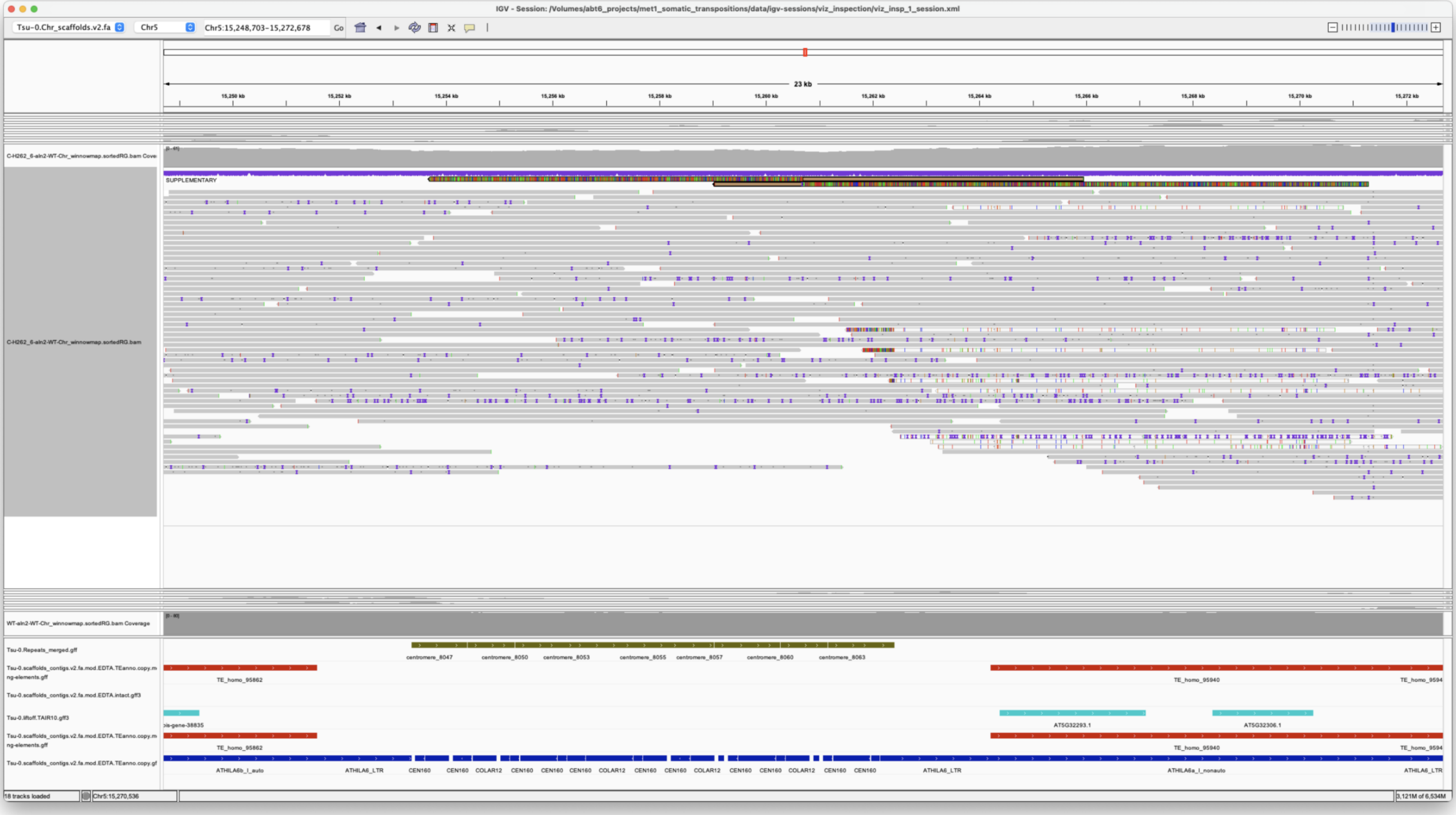

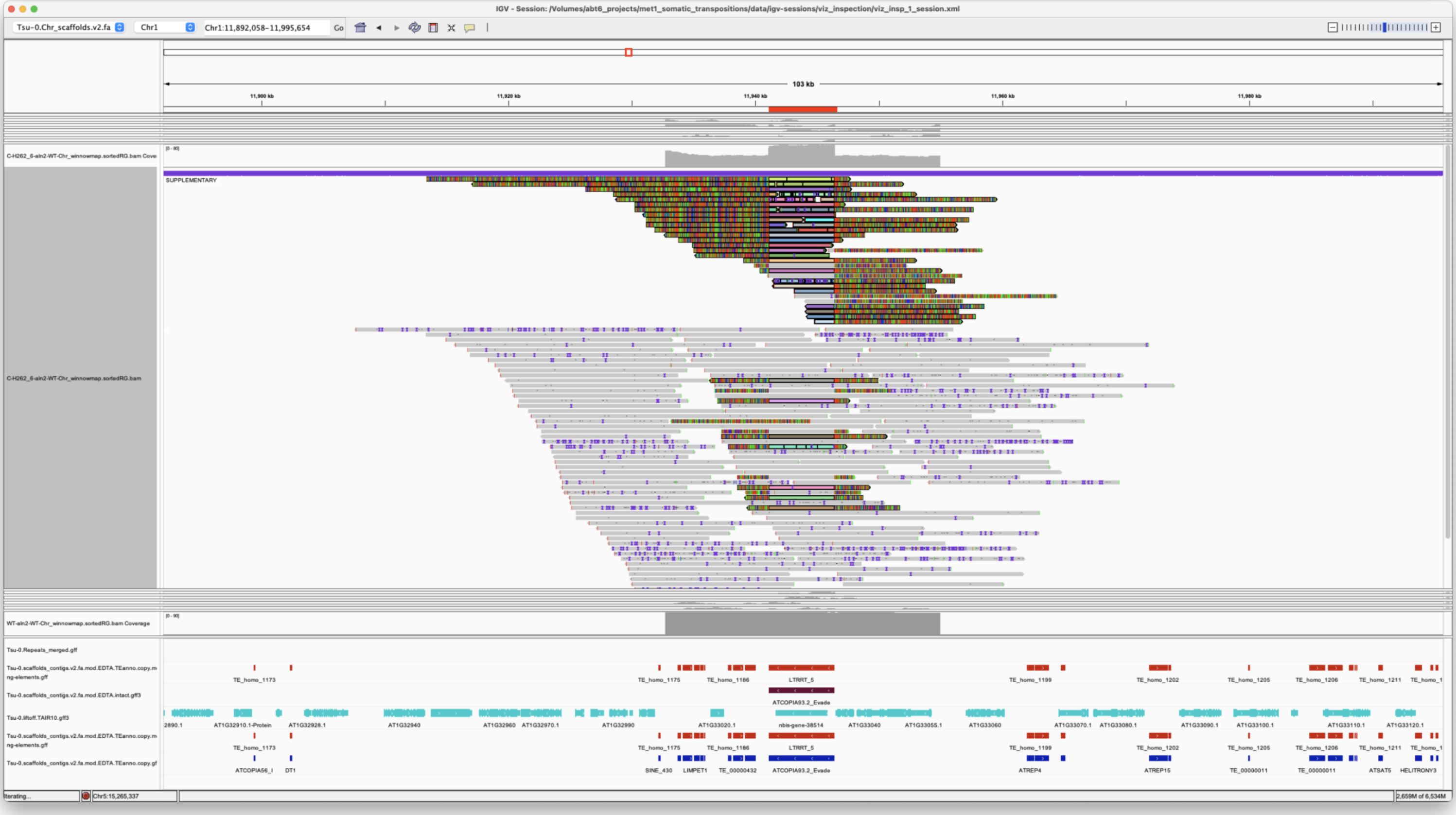

Centromeric

Central
TSD

Confirmed

Chr5 16993113 16993113 - 1 Chr5;19152829;19160826;VANDAL21 m64079_240212_113350/105449174/ccs met1_06

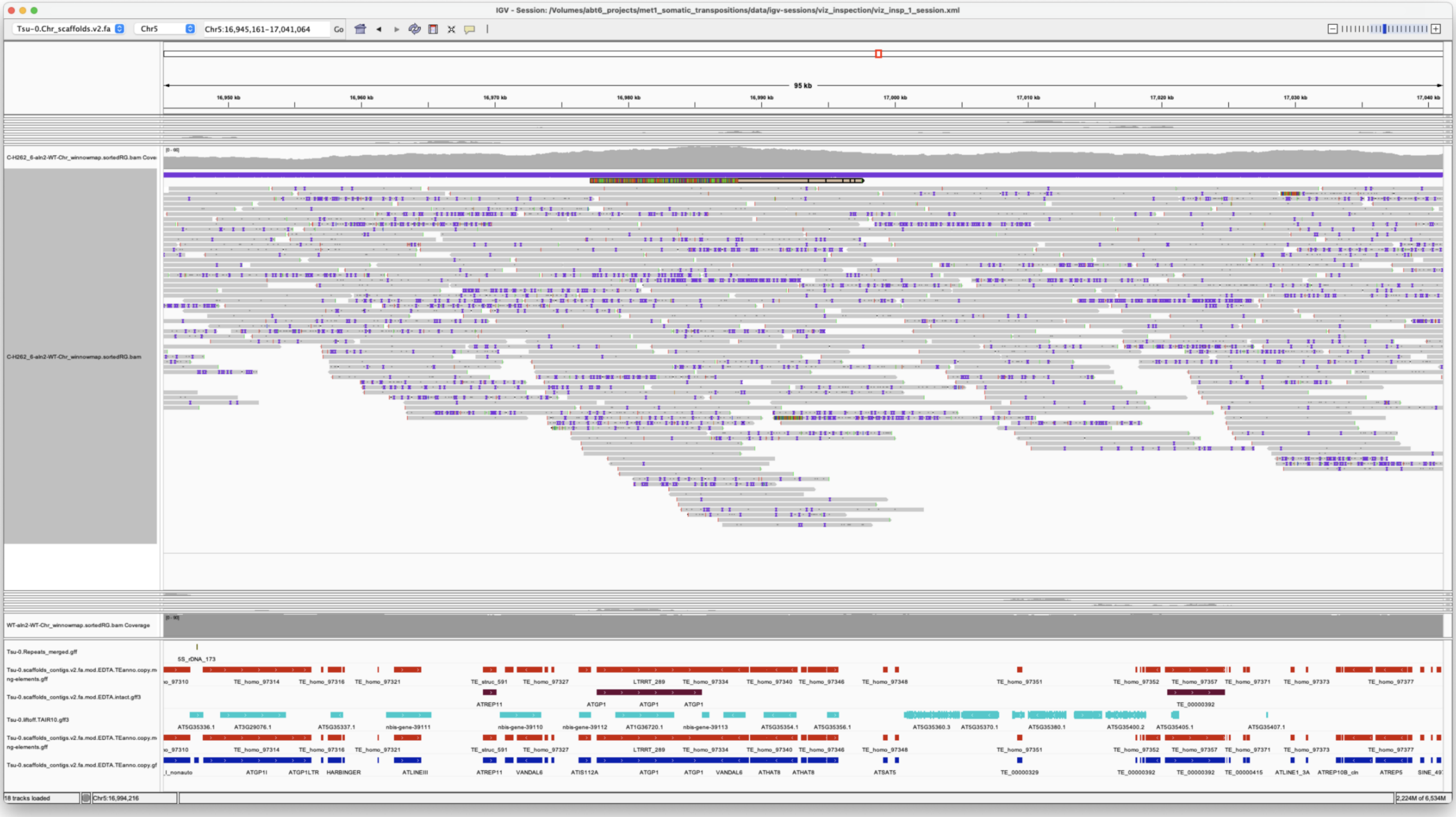

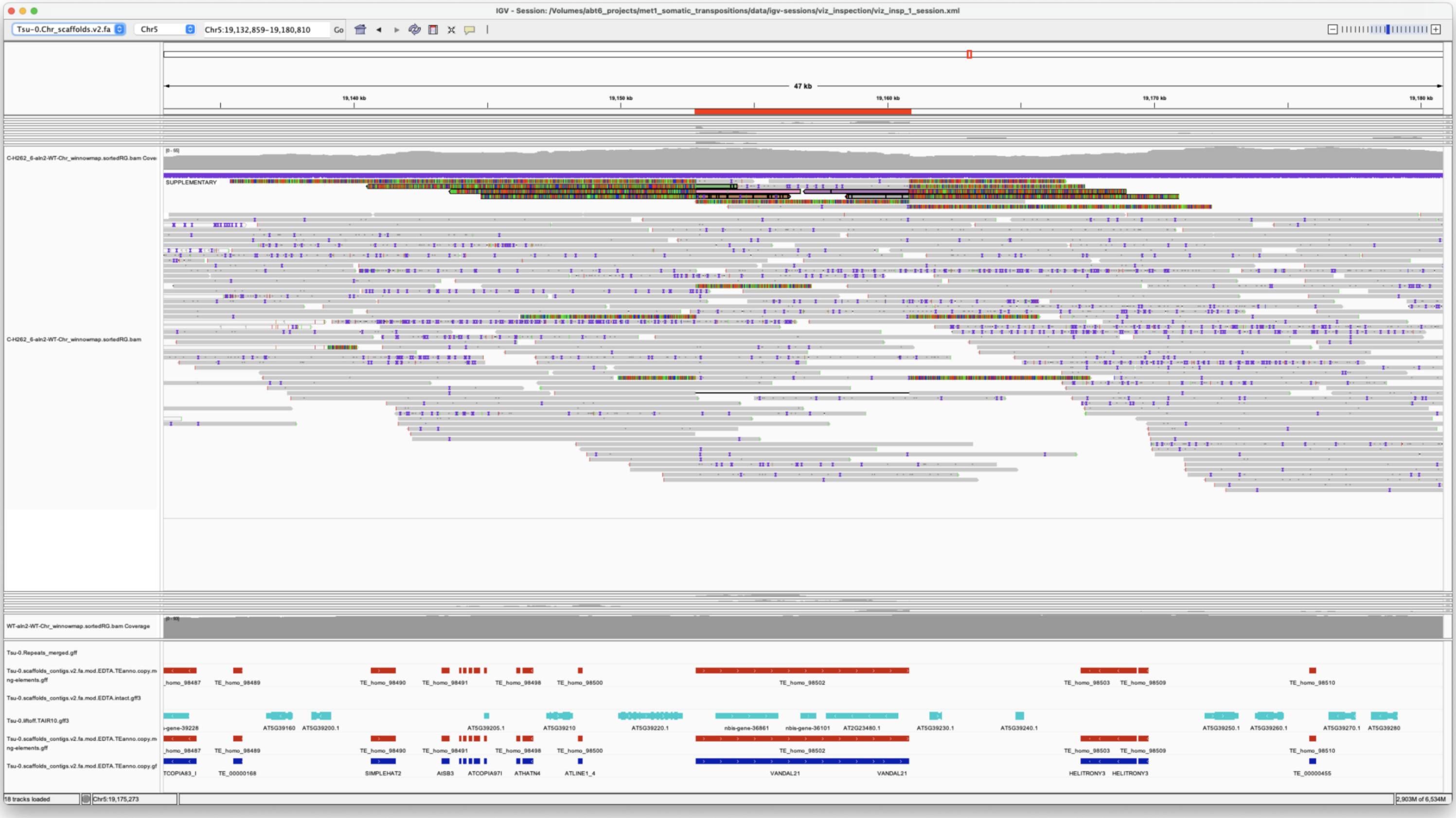

Partial

Confirmed

> Chr5 19075915 19075915 - 1 Chr1;11941106;11946436;ATCOPIA93_Evade m64079_221220_112036/67177263/ccs met1_06

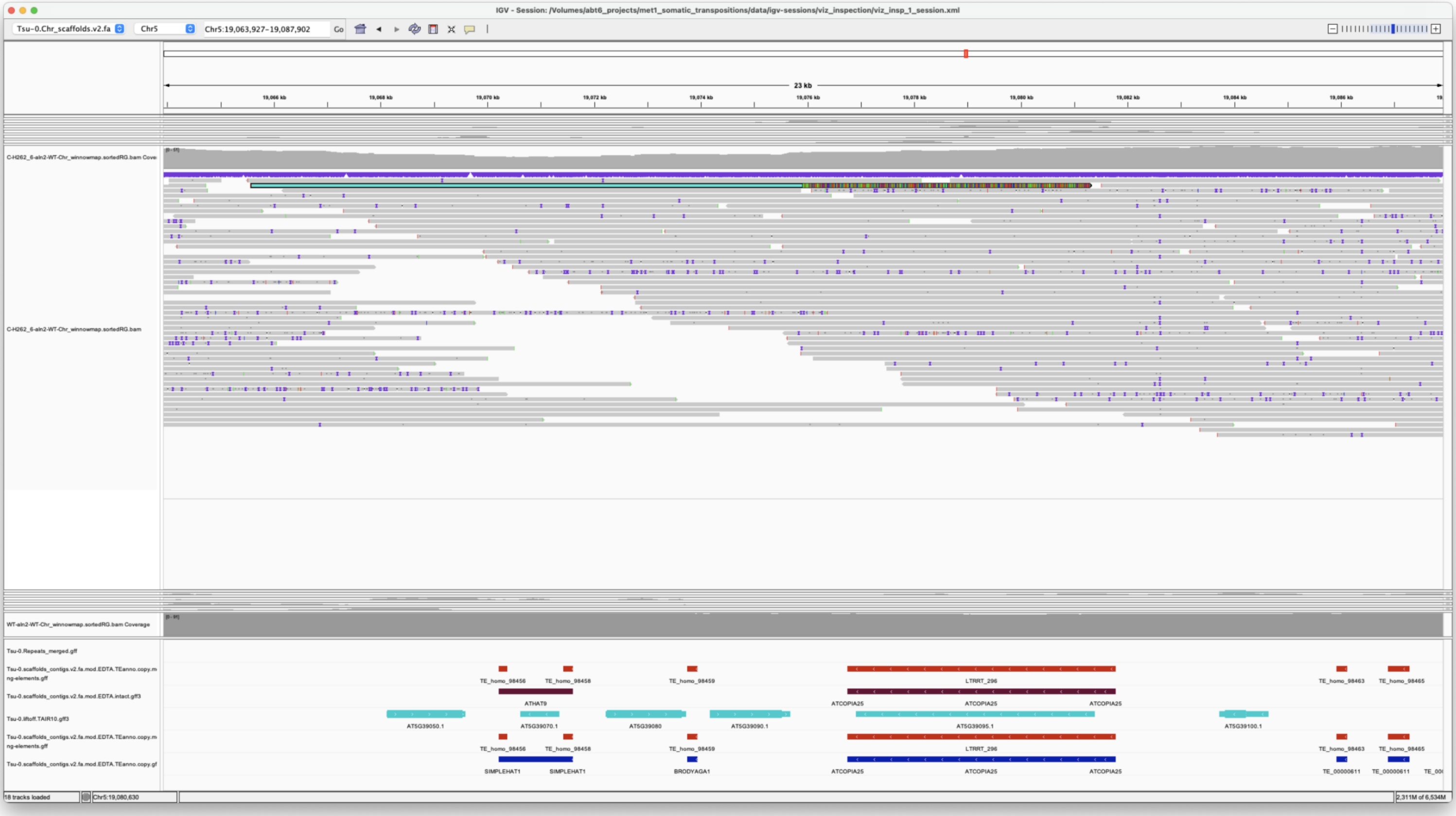

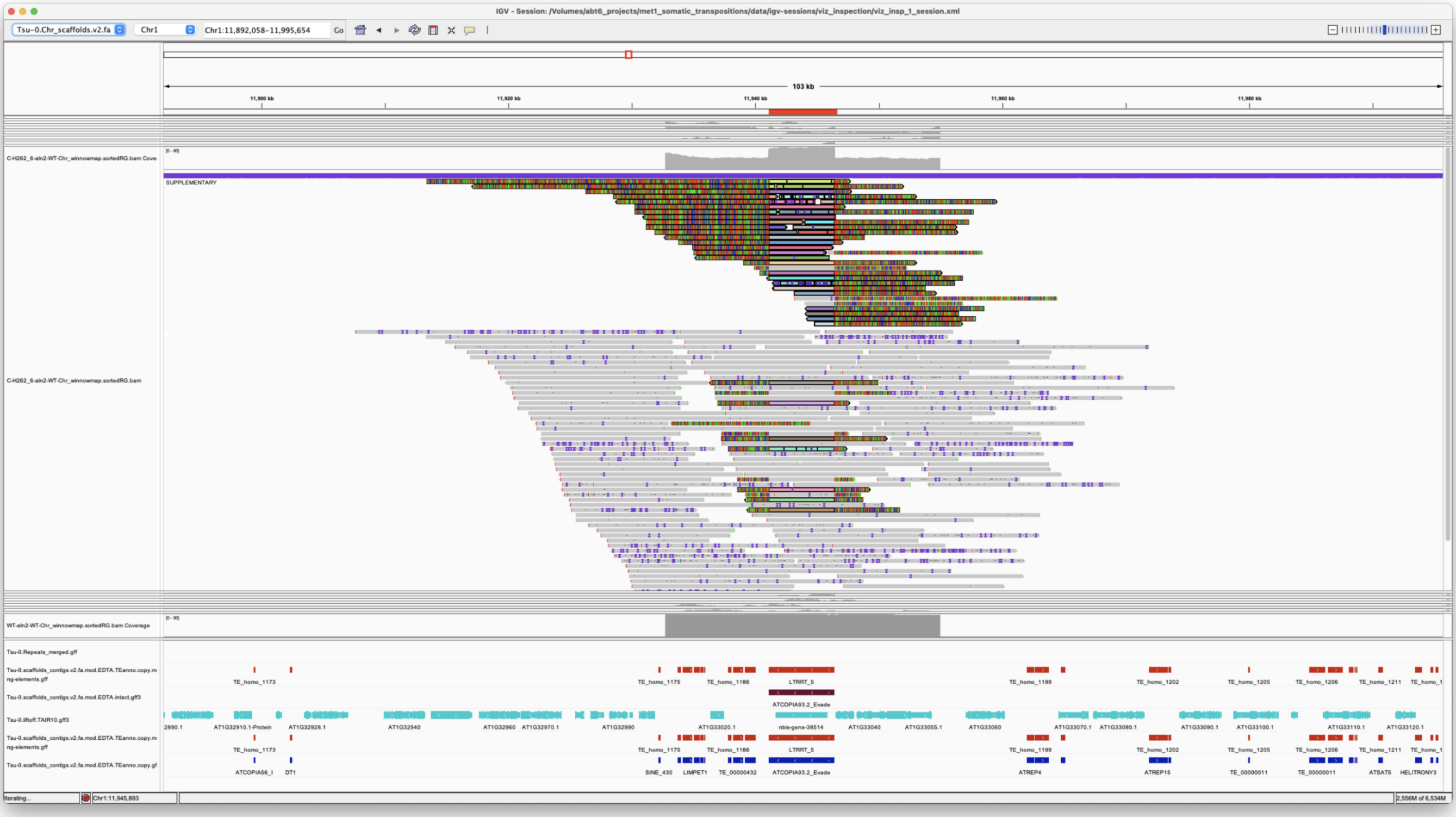

Partial

Confirmed

Chr5 19511048 19511048 + 1 Chr1;11941106;11946436;ATCOPIA93_Evade m64079_240212_113350/89262058/ccs met1_06

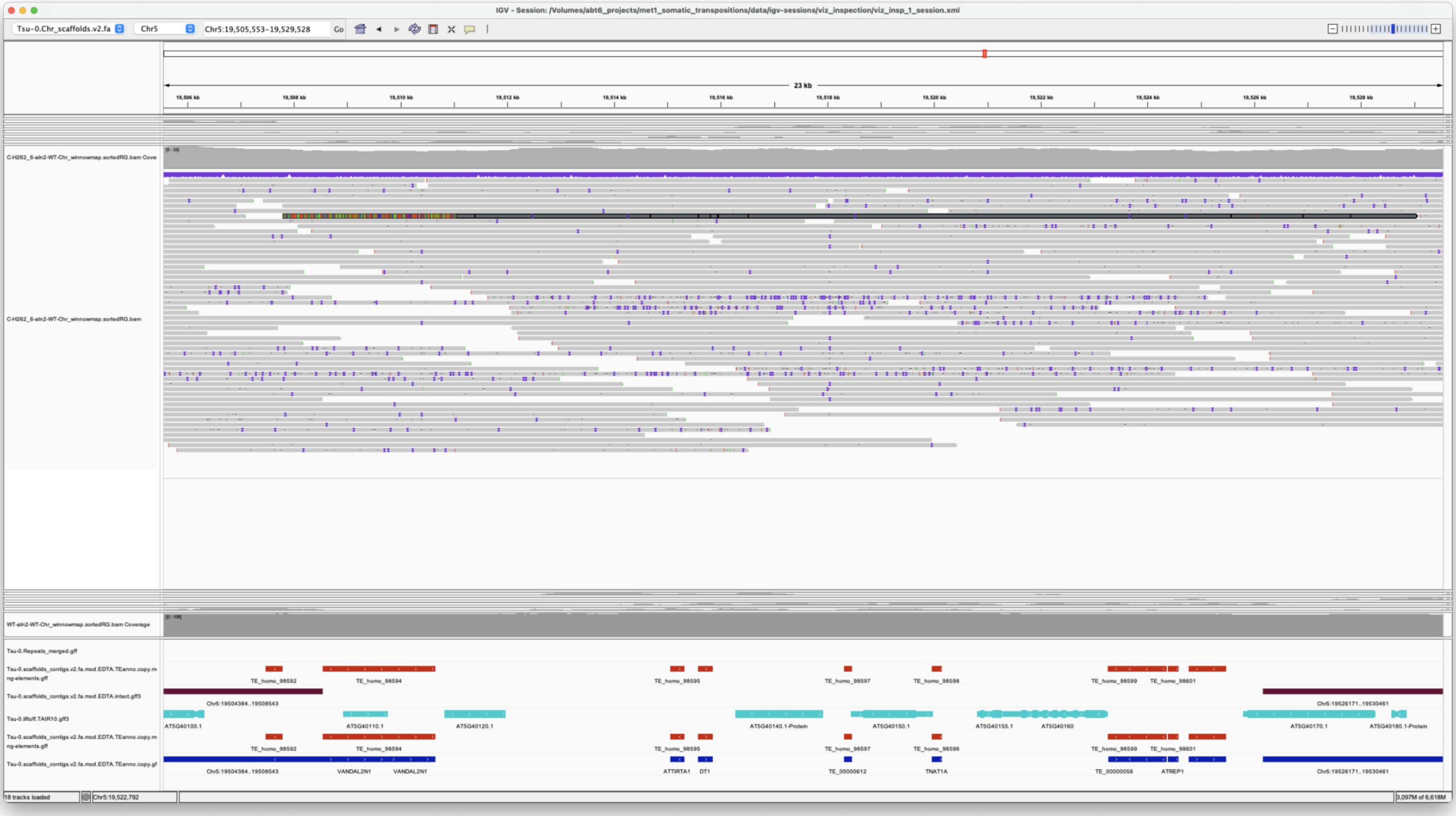

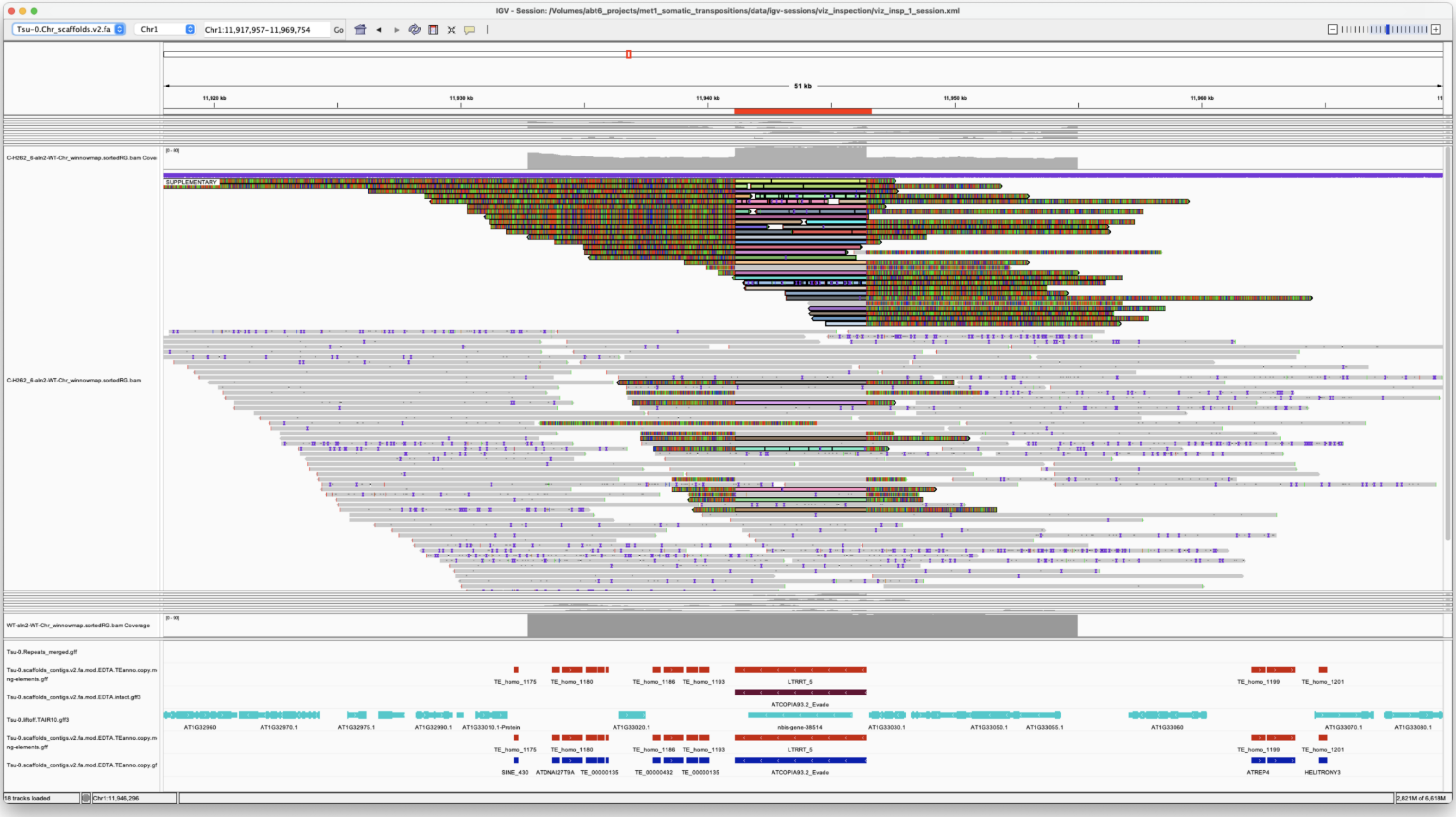

Partial

Confirmed

Chr5 19782252 19782252 - 1 Chr5;19152829;19160826;VANDAL21 m64079_240212_113350/164102149/ccs met1_06
