# Supplementary material for: Long-read detection of transposable element mobilization in the soma of hypomethylated Arabidopsis thaliana individuals
Source: Genome Biol. 2025 Jul 30;26:231. doi: 10.1186/s13059-025-03691-7 (PMC12312487; doi:10.1186/s13059-025-03691-7)
Supplement: Supplementary file 2 — Additional file 2. Visual inspection of somatic insertion and excision events, available at https://github.com/aerilli/Somatic-transposition_met1/tree/551df407370c6528225f404ba62a073dced14b08/Supplementary-Files/Visual_inspection. [file 13059_2025_3691_MOESM2_ESM.gz › Split_Supplementary-File4/File_4-5_Synteny-check/File4_Synteny-check_Col-0-TEs-in-Tsu-0.pdf]

Synteny-check_Col-0-TEs-in-Tsu-0

Chr1 11148307 11152649 ATCOPIA51

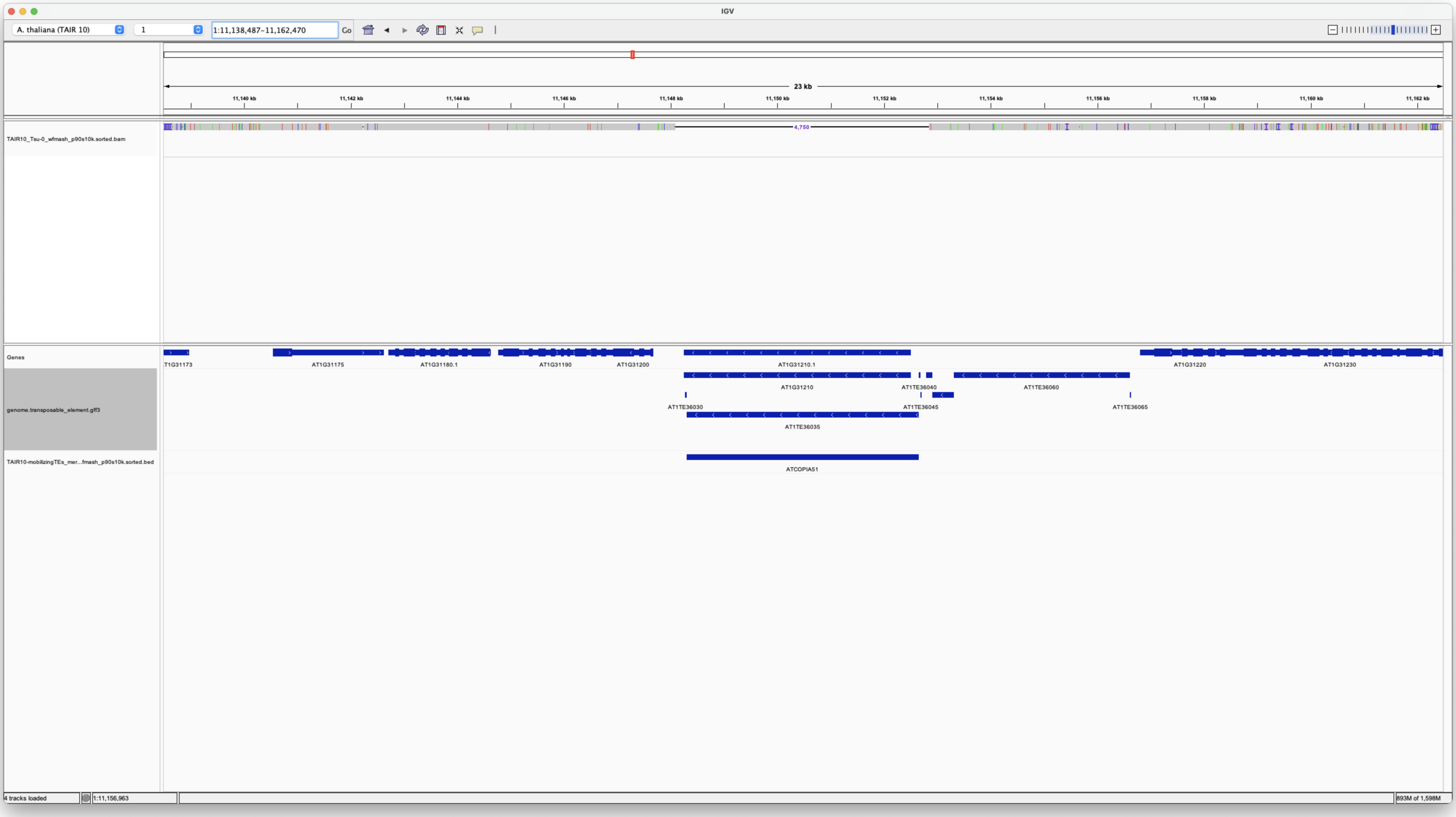

No Tsu-0

Chr1 12755059 12760395 ATCOPIA93

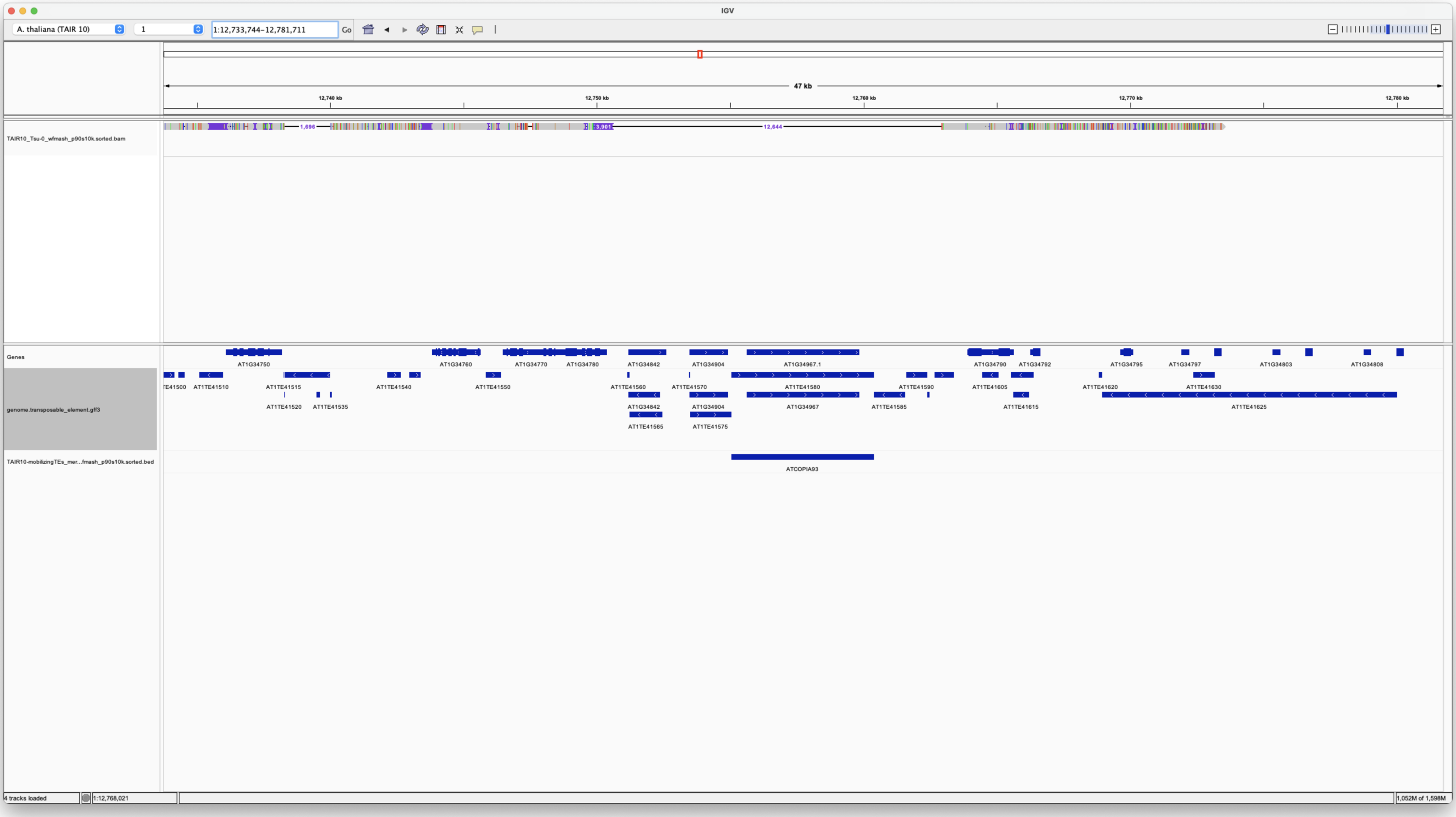

No Tsu-0

Chr1 17203925 17206318 ATCOPIA63

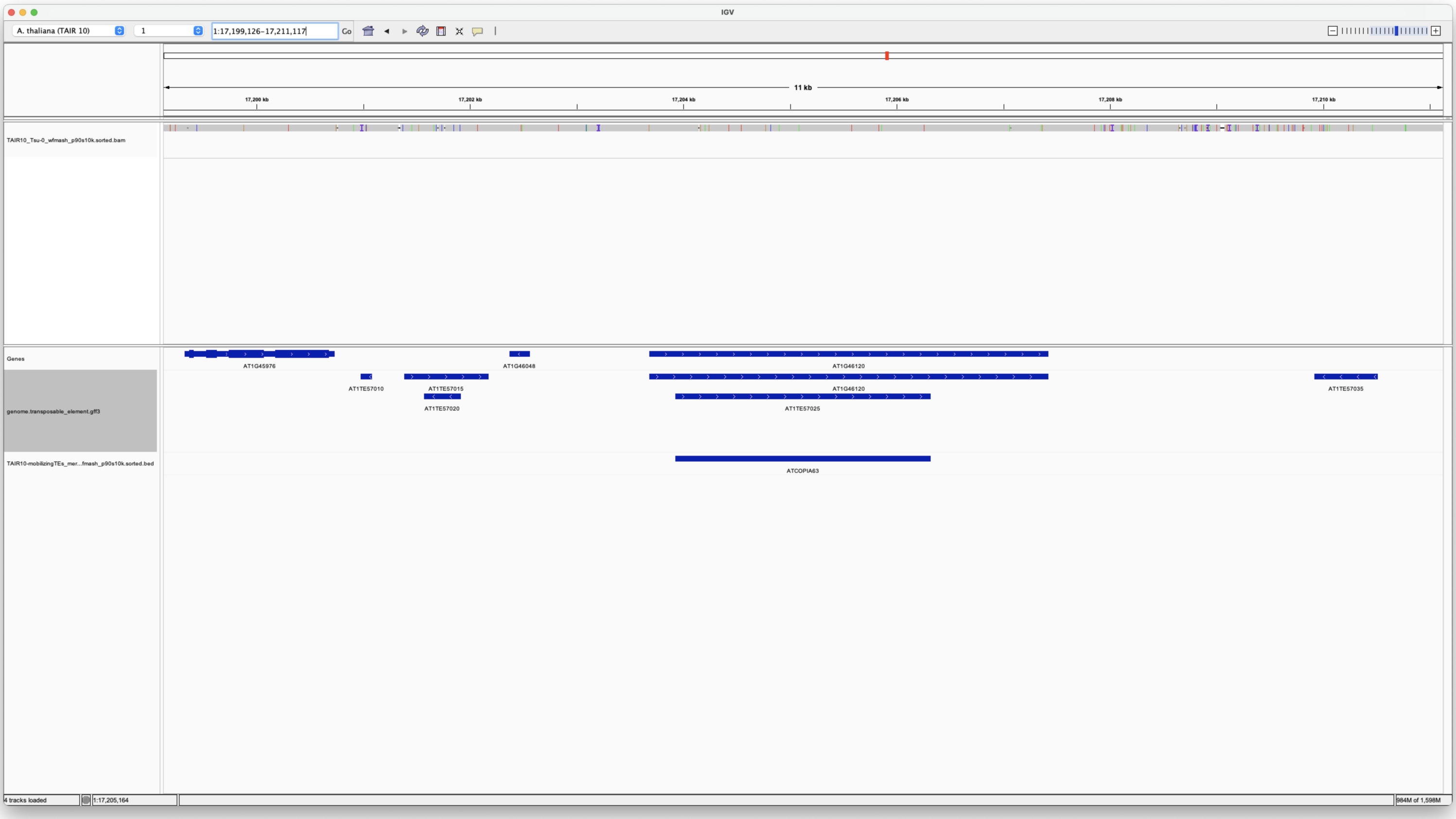

YES Tsu-0

Chr1 21796937 21801751 ATRE1

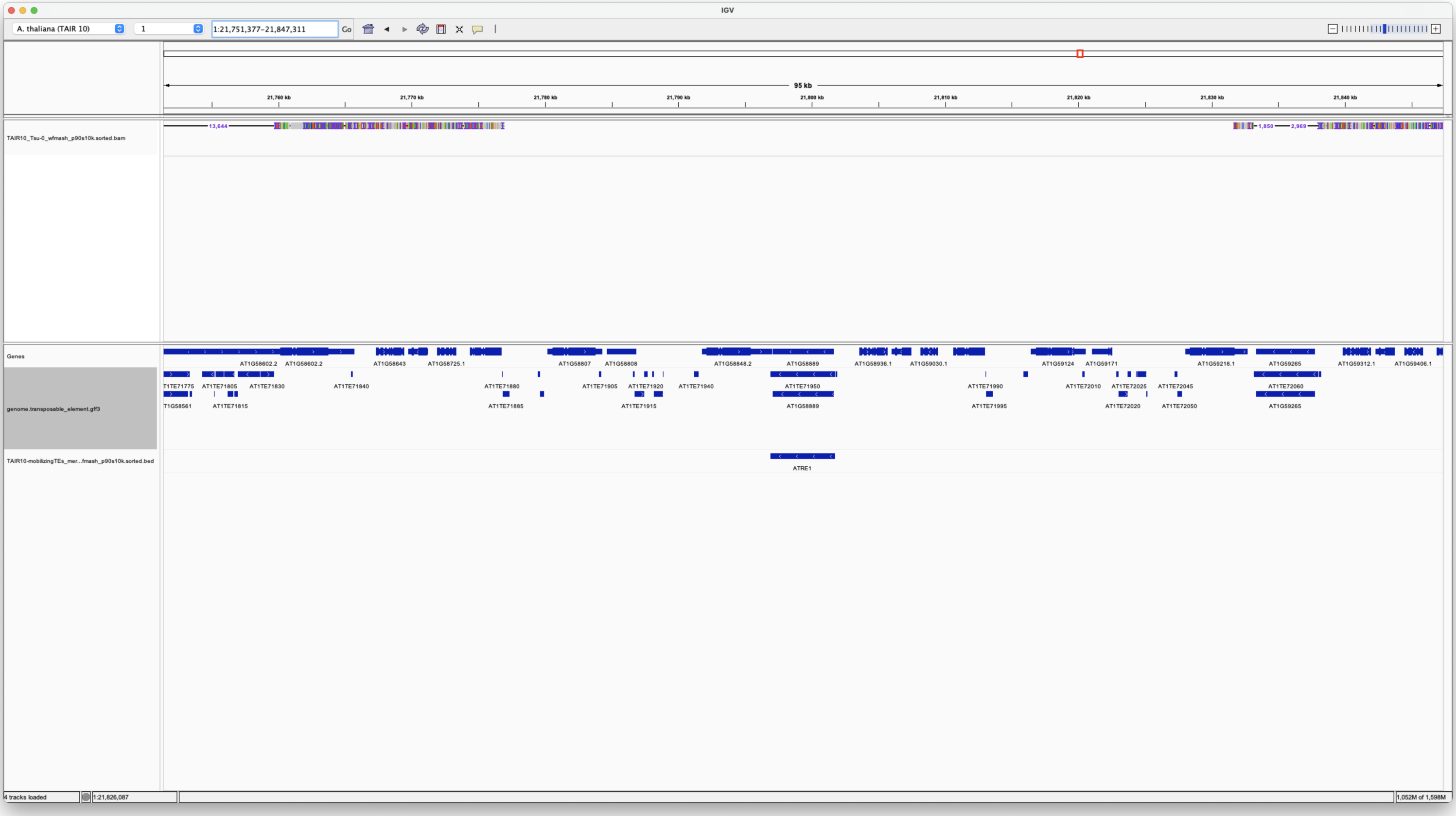

No Tsu-0

Chr2 10001479 10009658 VANDAL21

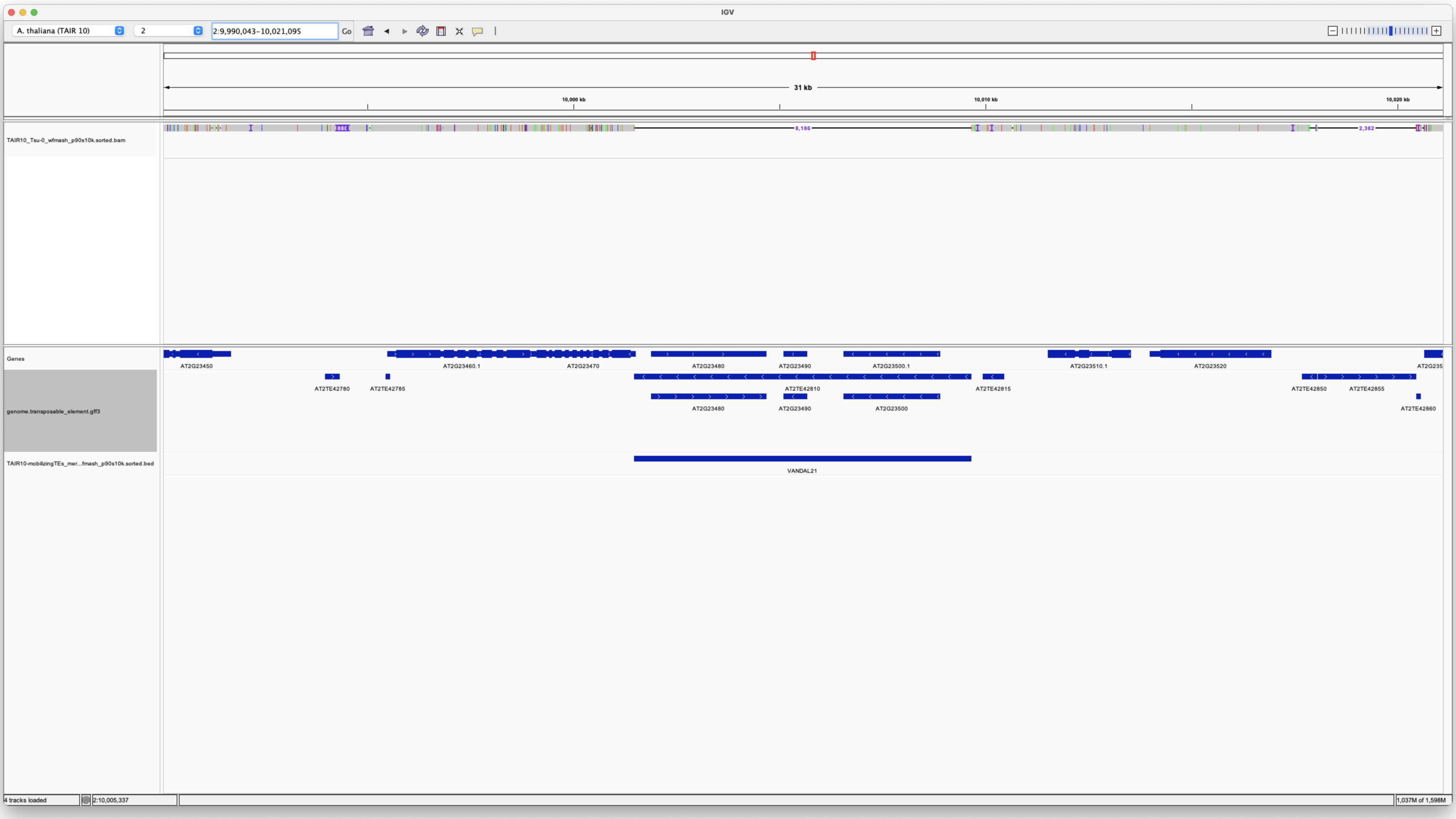

No Tsu-0

Chr2 34379 36436 ATGP2N

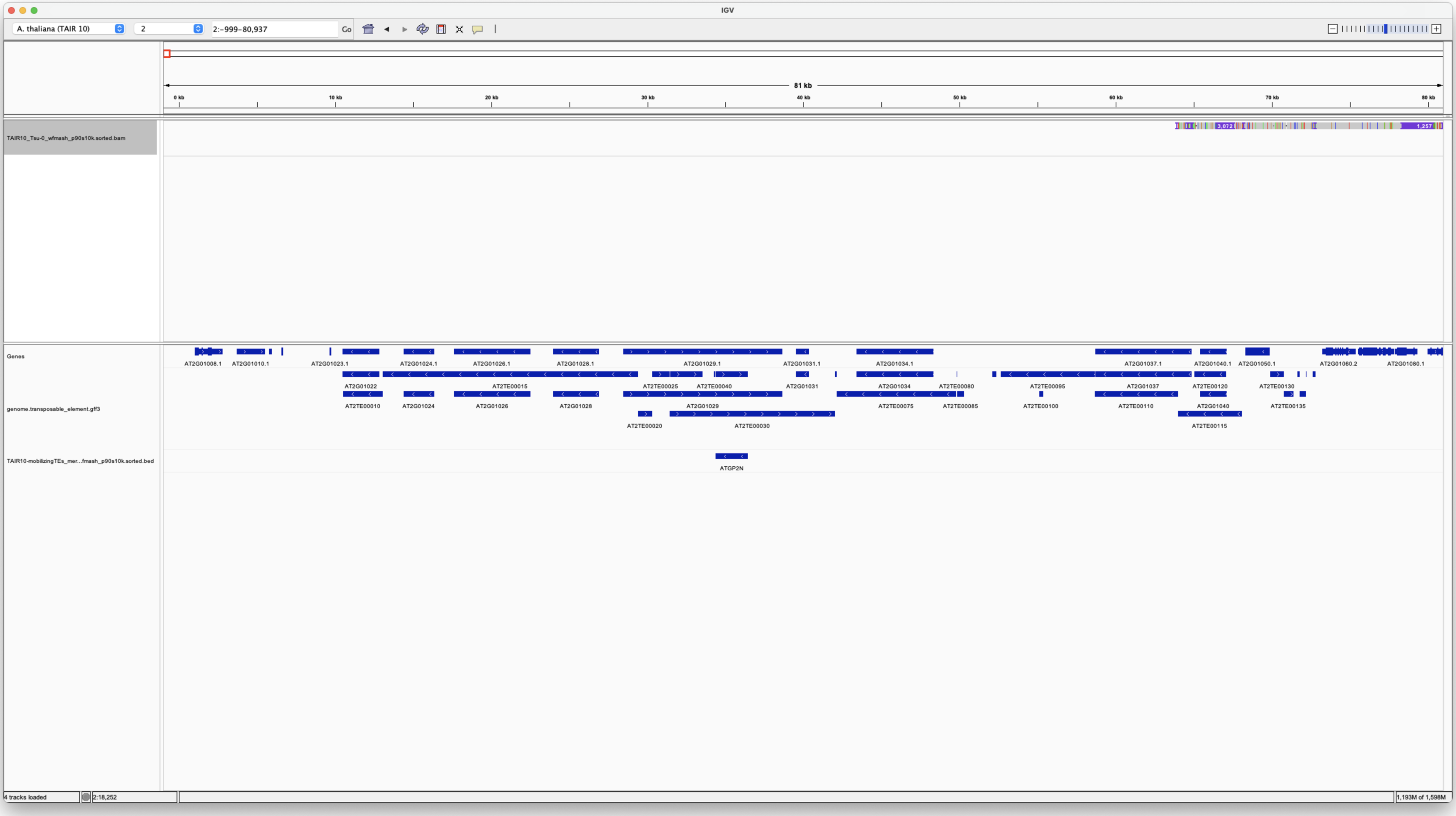

> Very telomeric -> dubious

No Tsu-0

Chr2 4900802 4909281 ATENSPM3

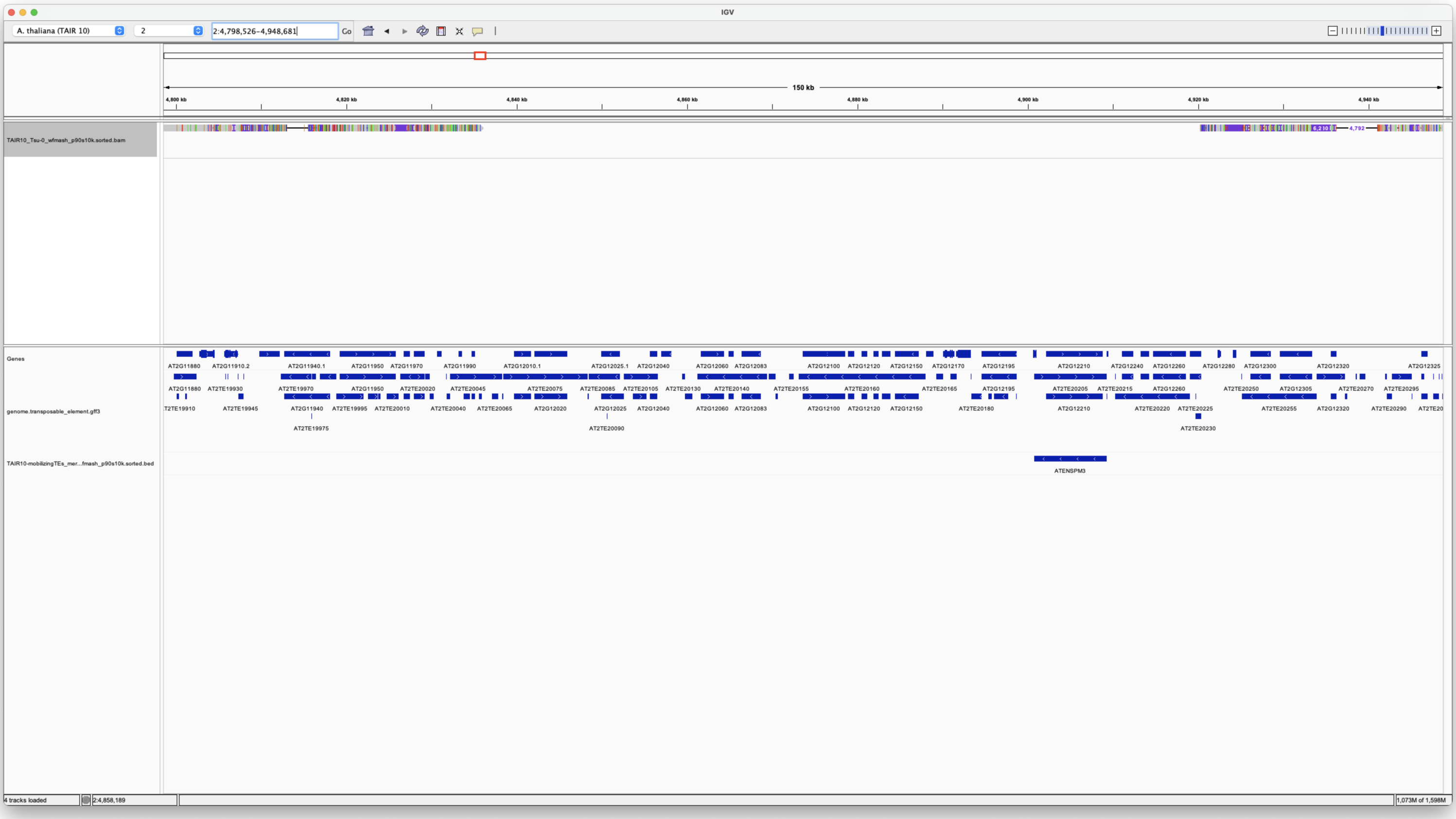

No Tsu-0

Chr2 5852698 5857428 ATCOPIA13

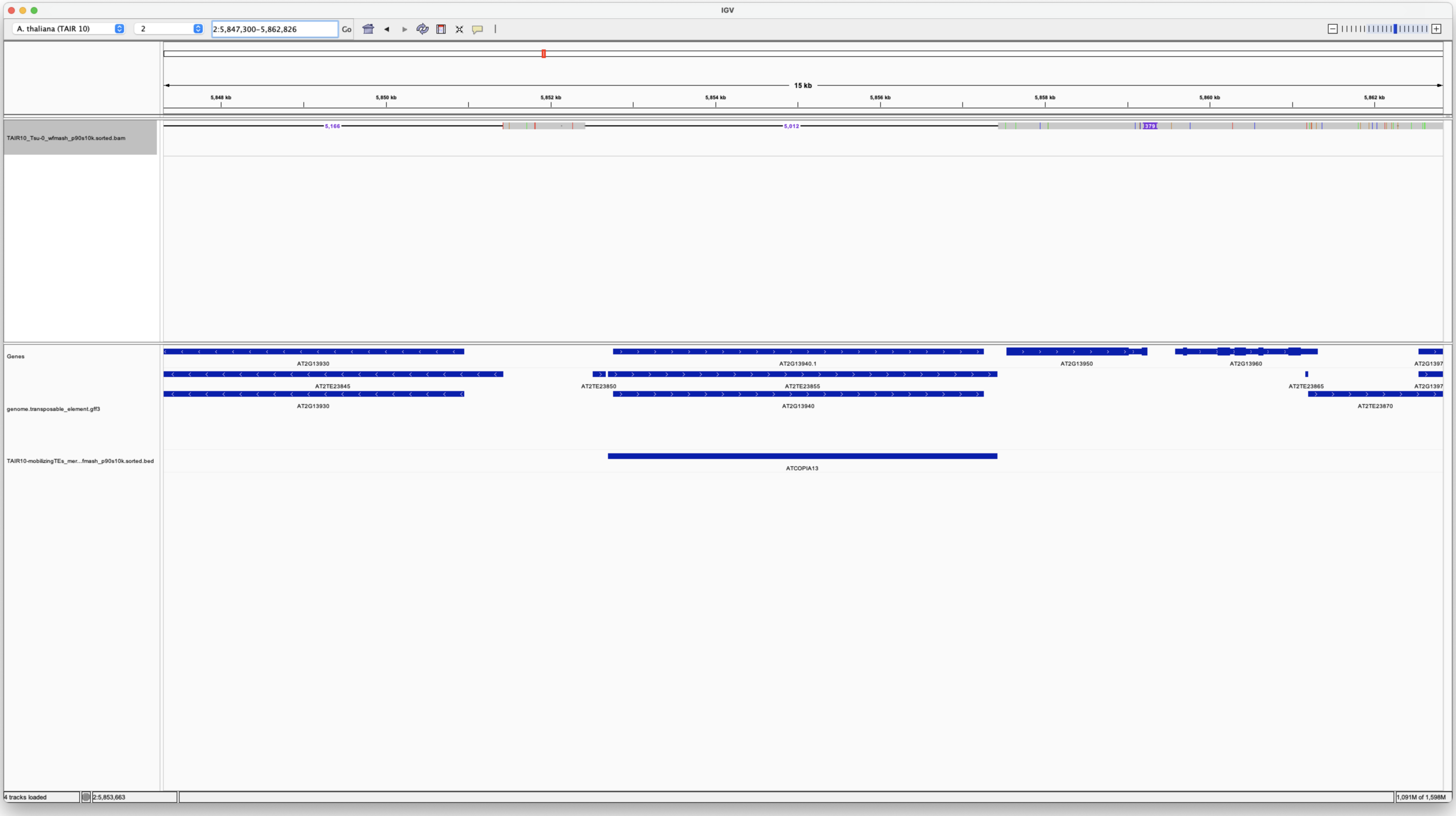

No Tsu-0

Chr3 15283647 15283911 ATENSPM3

Chr3 15283666 15286610 Pack-CACTA2a

Chr3 15286419 15286632 ATENSPM3

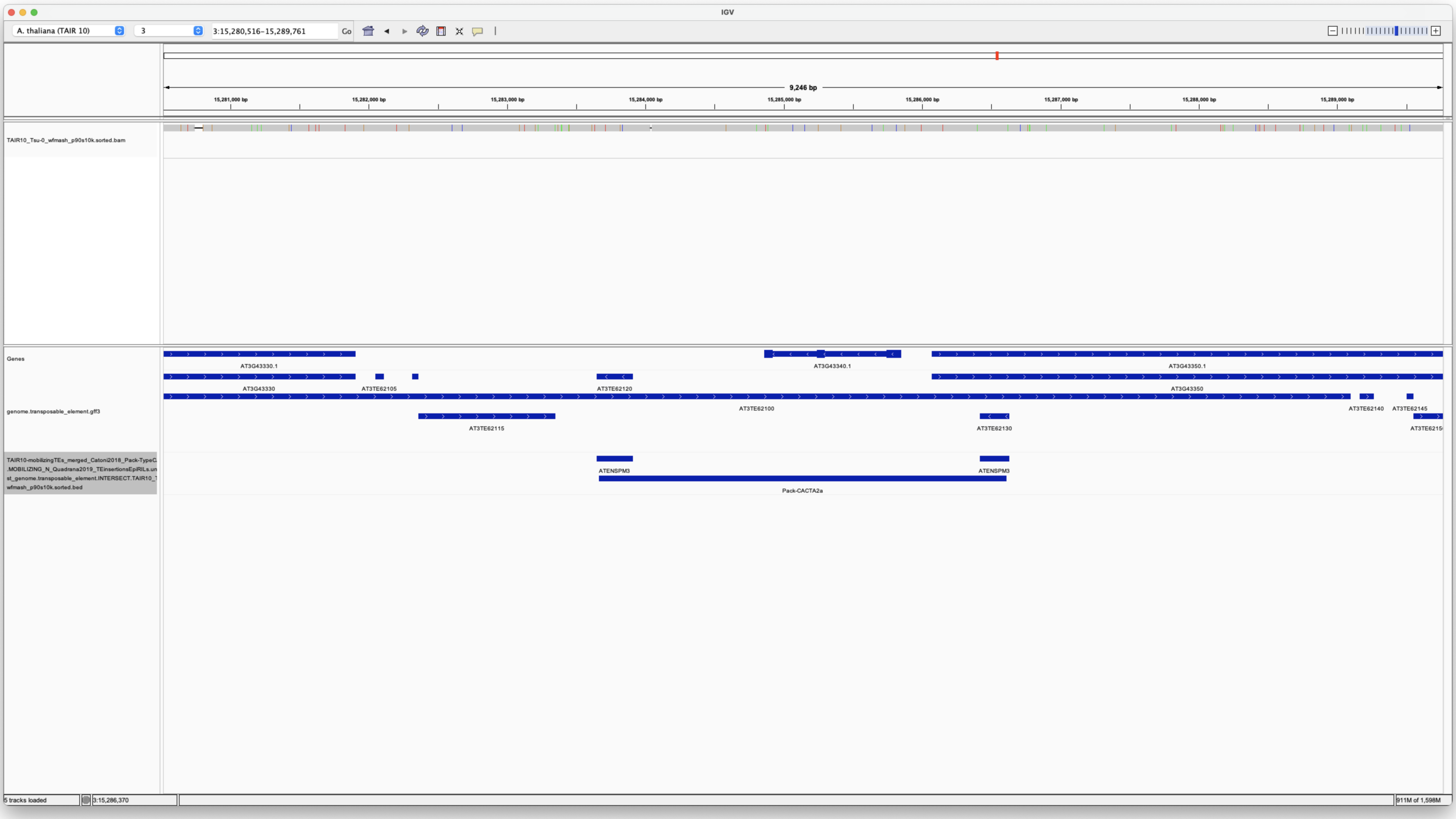

YES Tsu-0

Chr4 4358128 4358579 ATENSPM3

Chr4 4358131 4360293 Pack-CACTA1a

Chr4 4359956 4360293 ATENSPM3

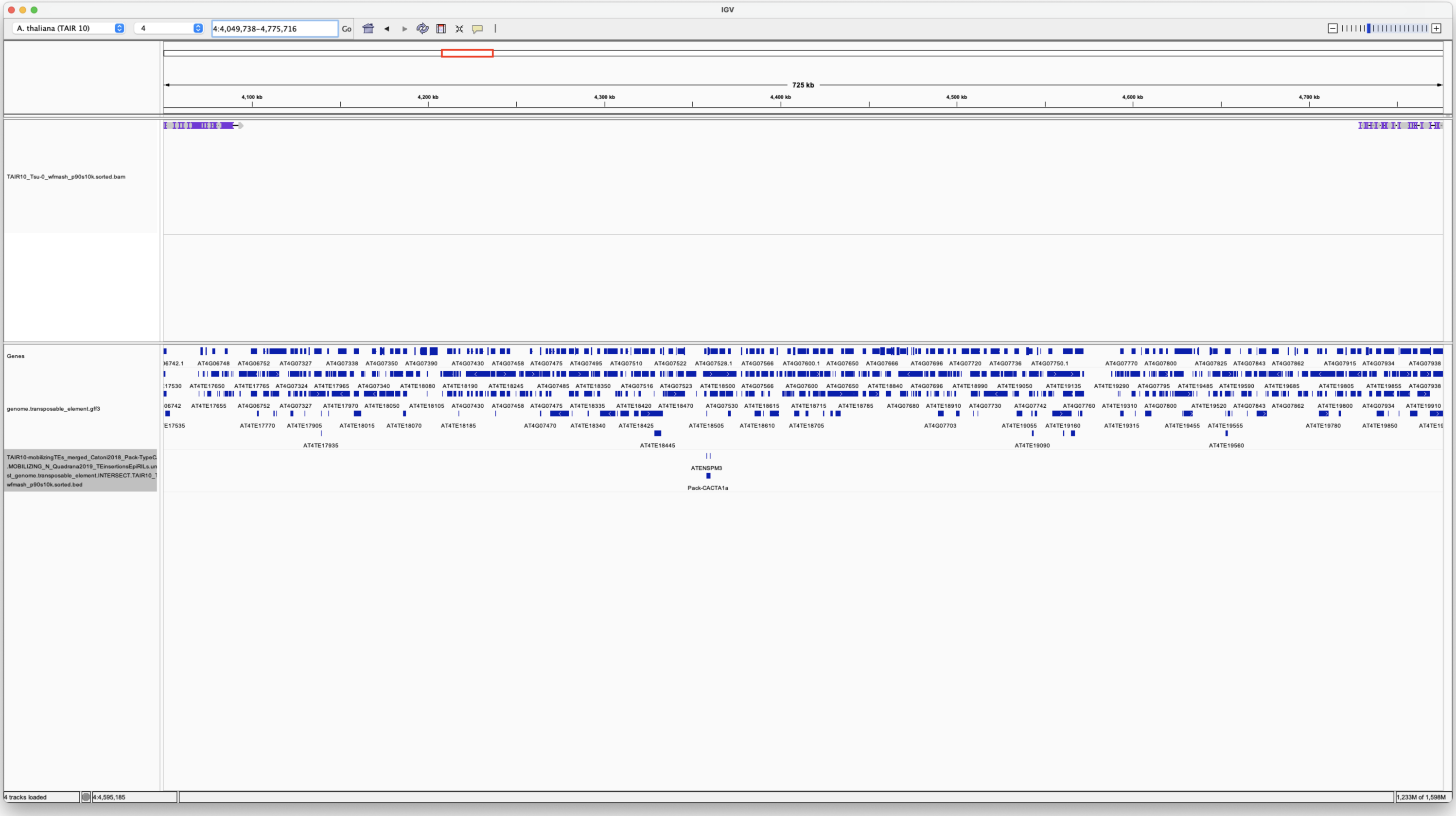

No Tsu-0

Chr5 13960645 13968612 VANDAL21

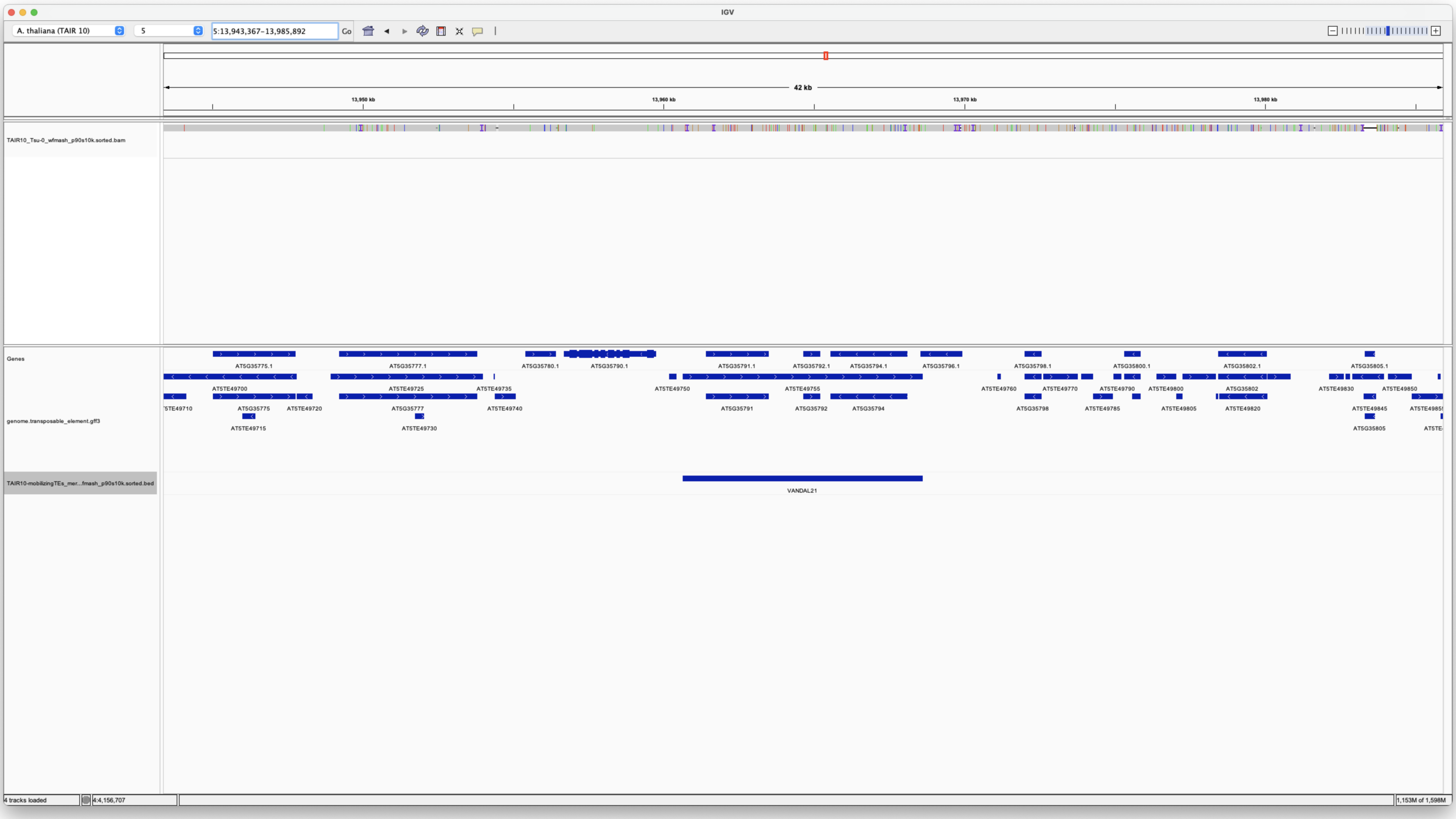

YES Tsu-0

Chr5 18142155 18146898 ATCOPIA21

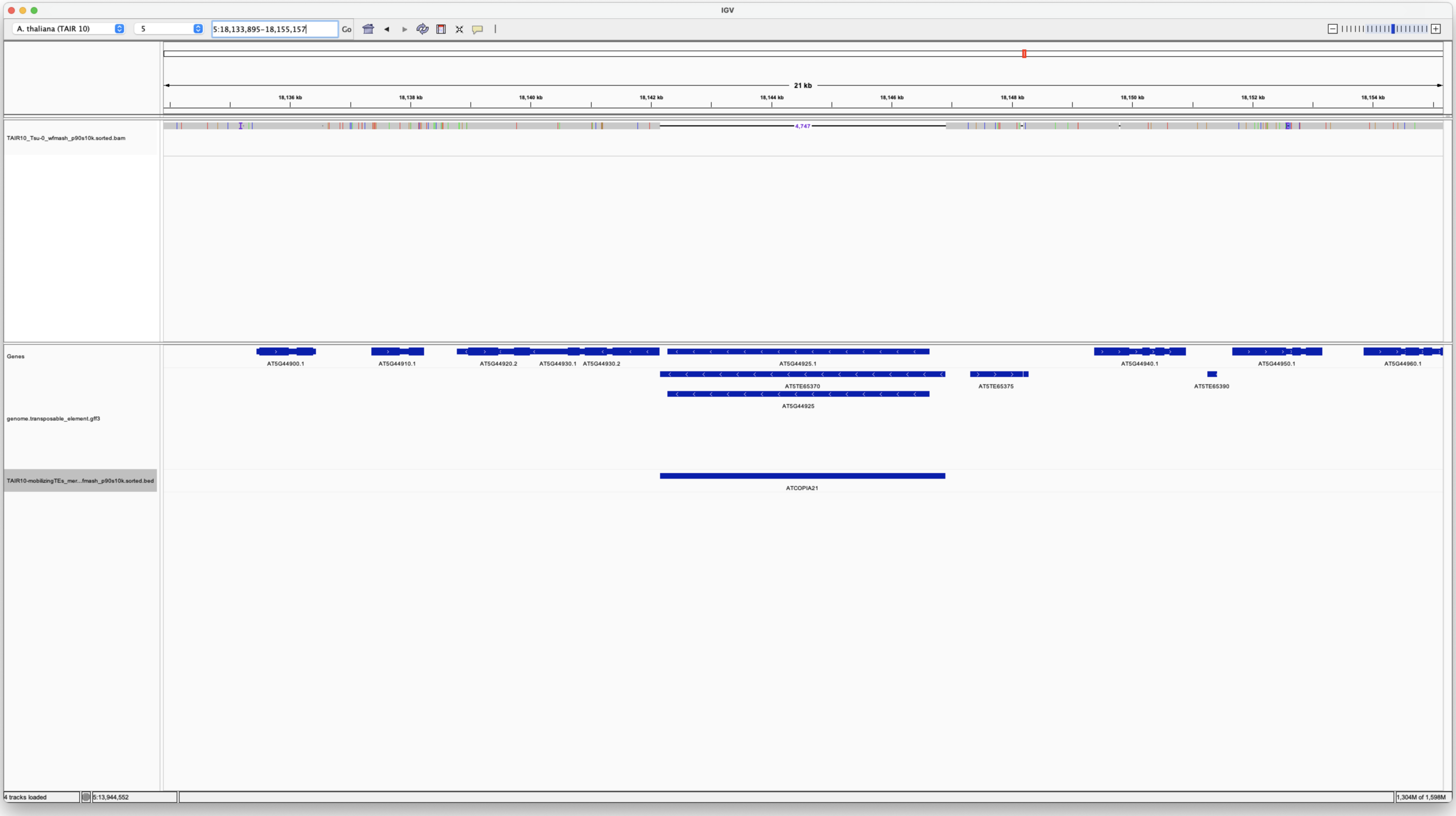

No Tsu-0

Chr5 18490605 18495234 ATCOPIA31

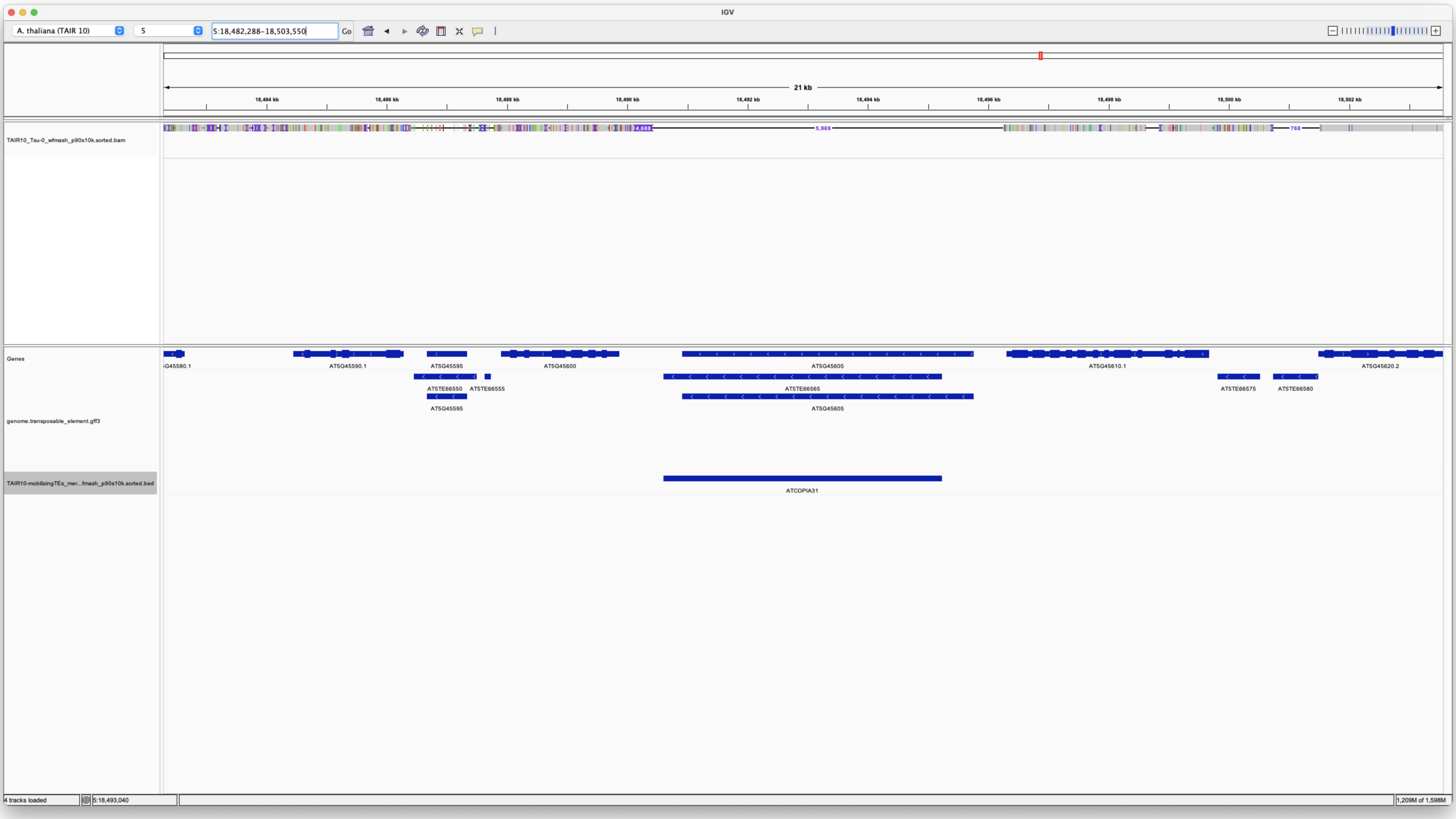

No Tsu-0

Chr5 5629977 5635310 ATCOPIA93

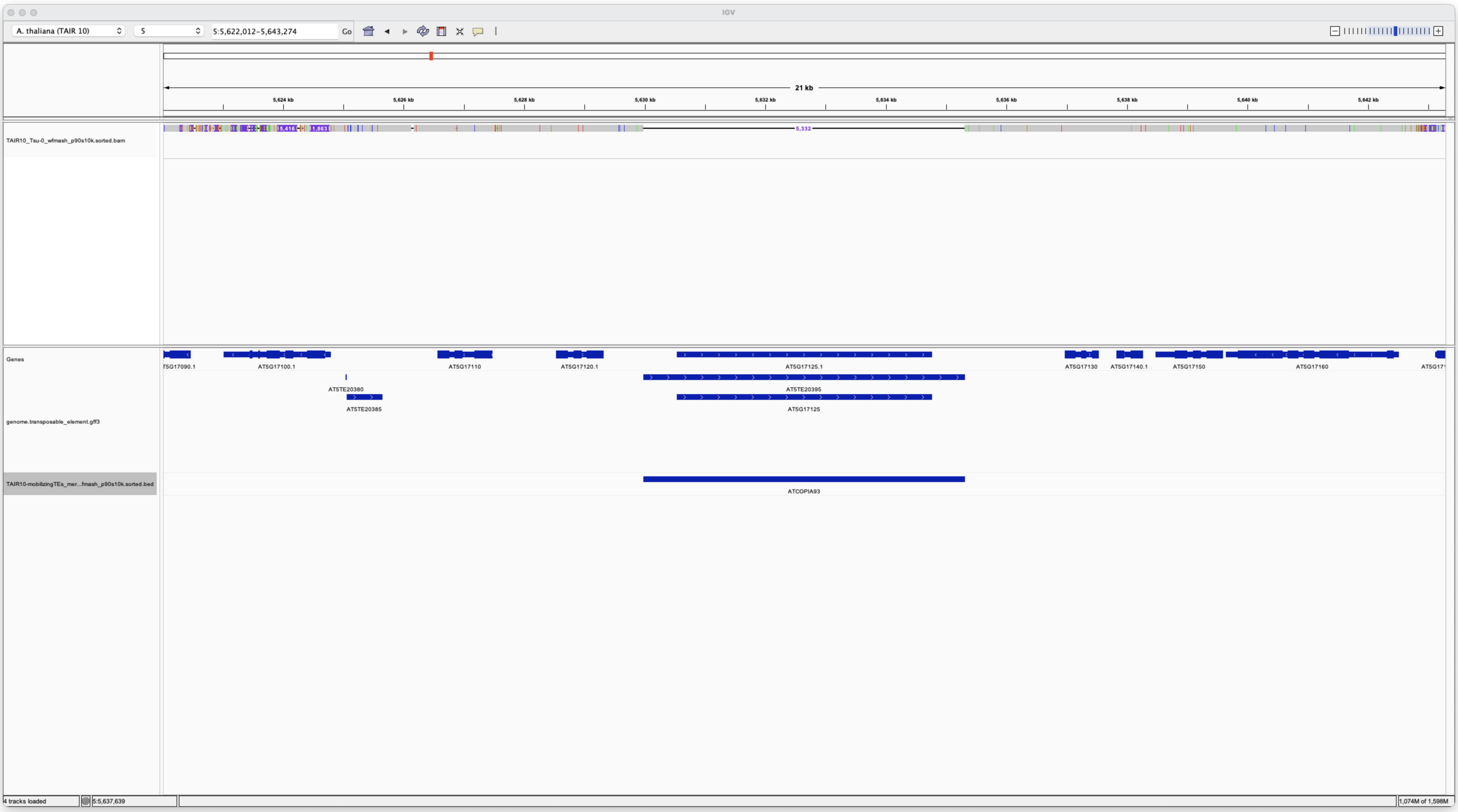

No Tsu-0

Chr5 9272139 9274532 ATCOPIA63

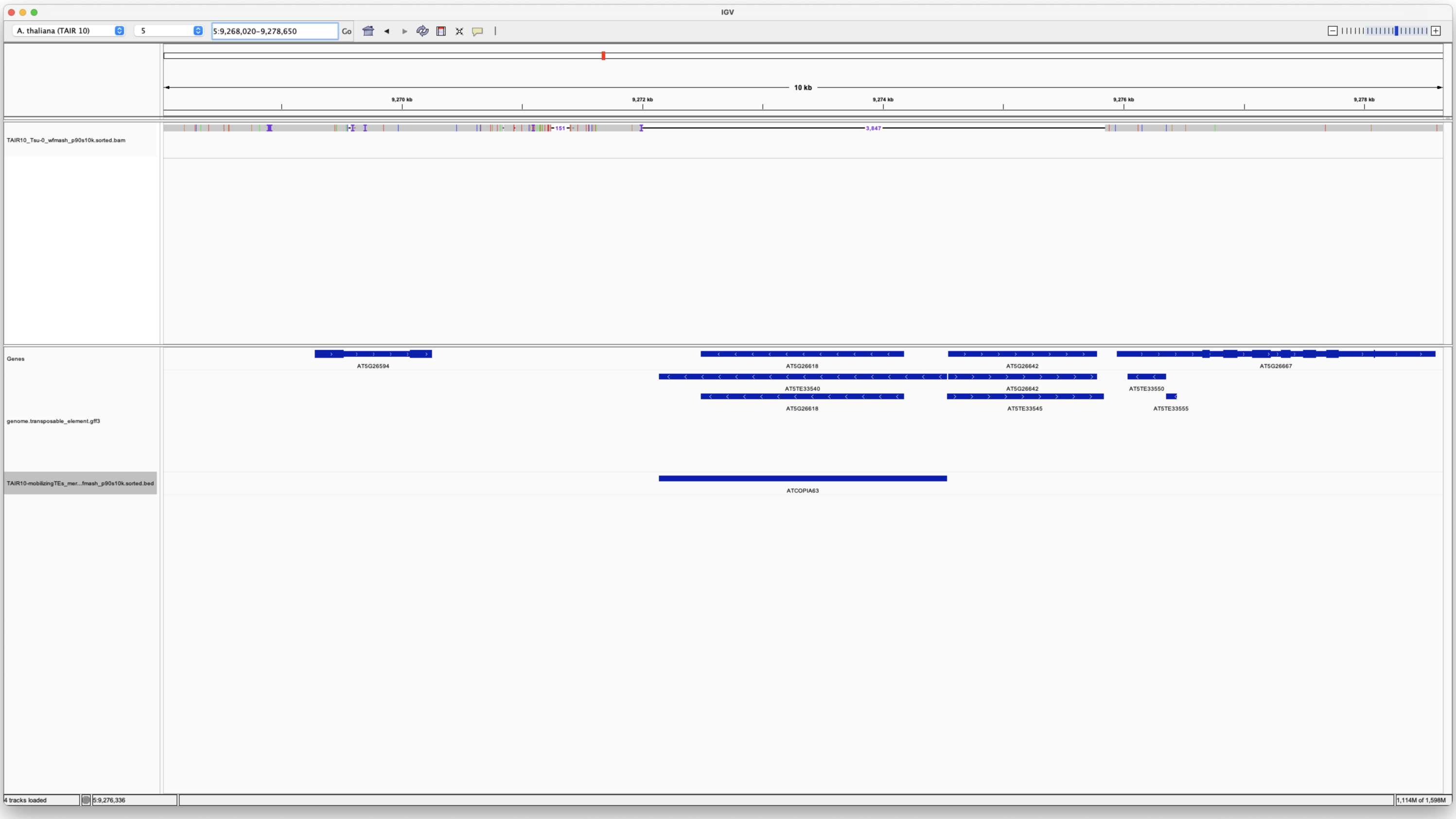

No Tsu-0

Tsukahara

Chr1 13818975 13823475 AT1G36590

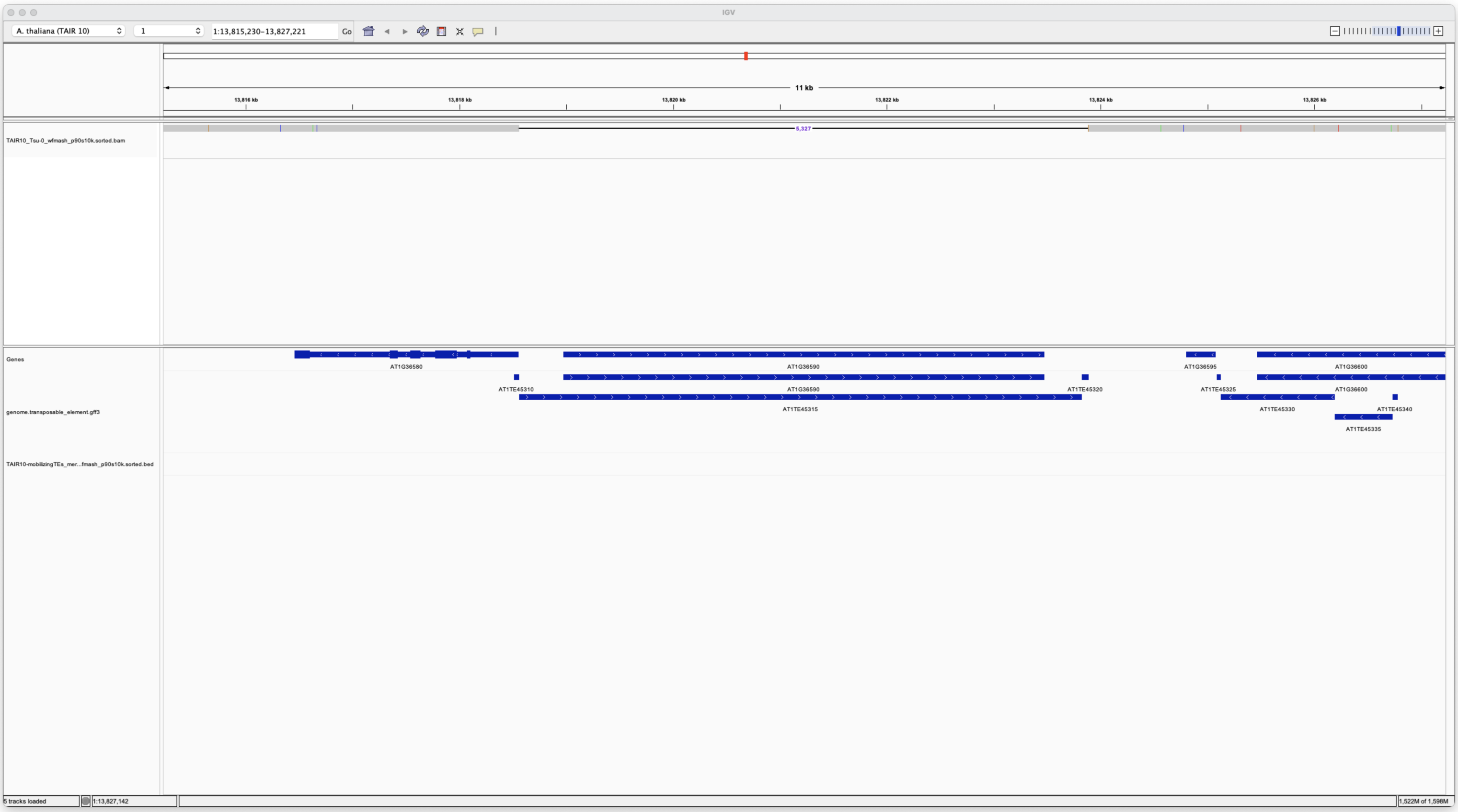

No Tsu-0

Chr1 29377751 29380183 AT1G78095

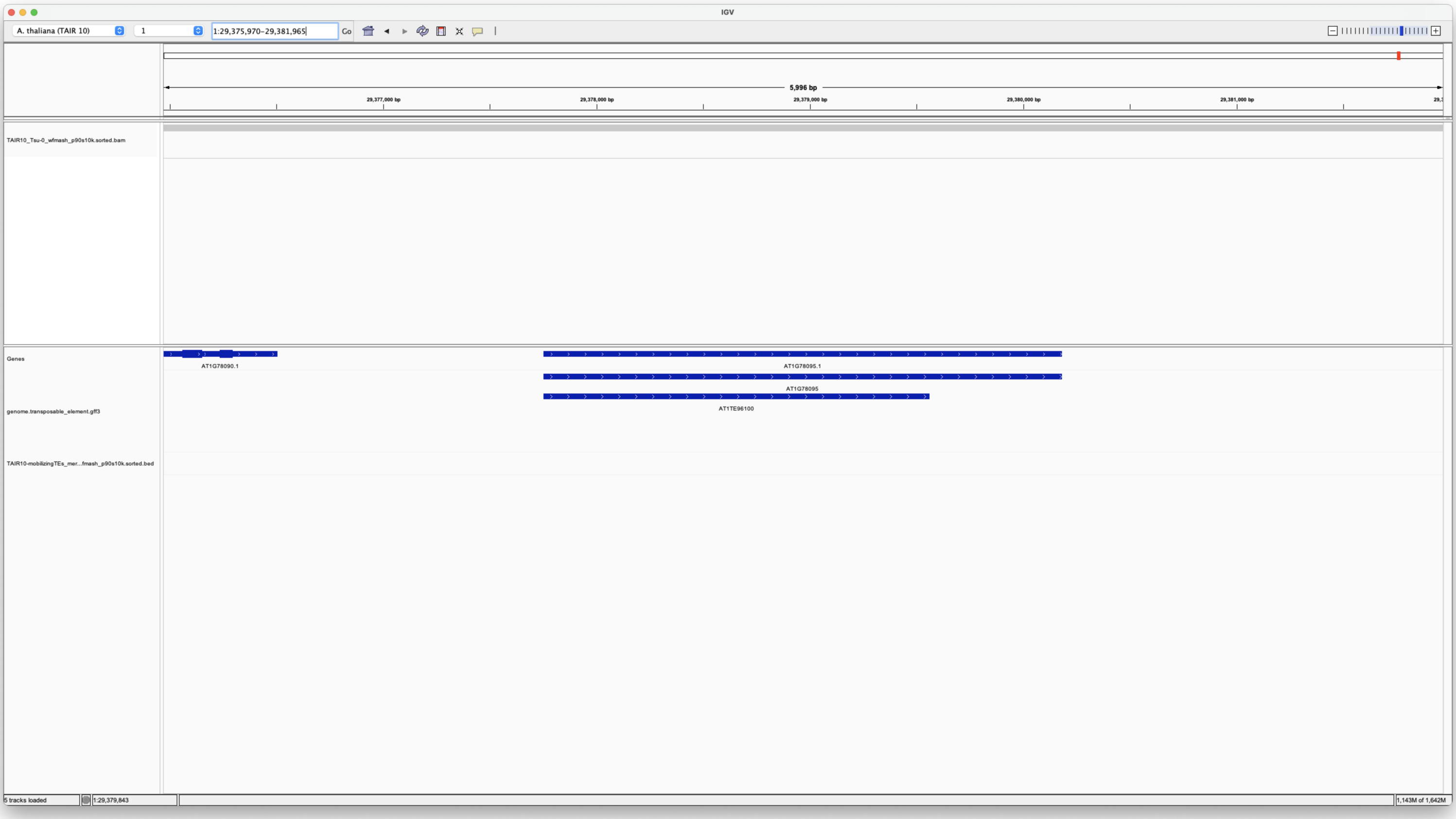

YES Tsu-0

Chr3 3807726 3812227 AT3G11970

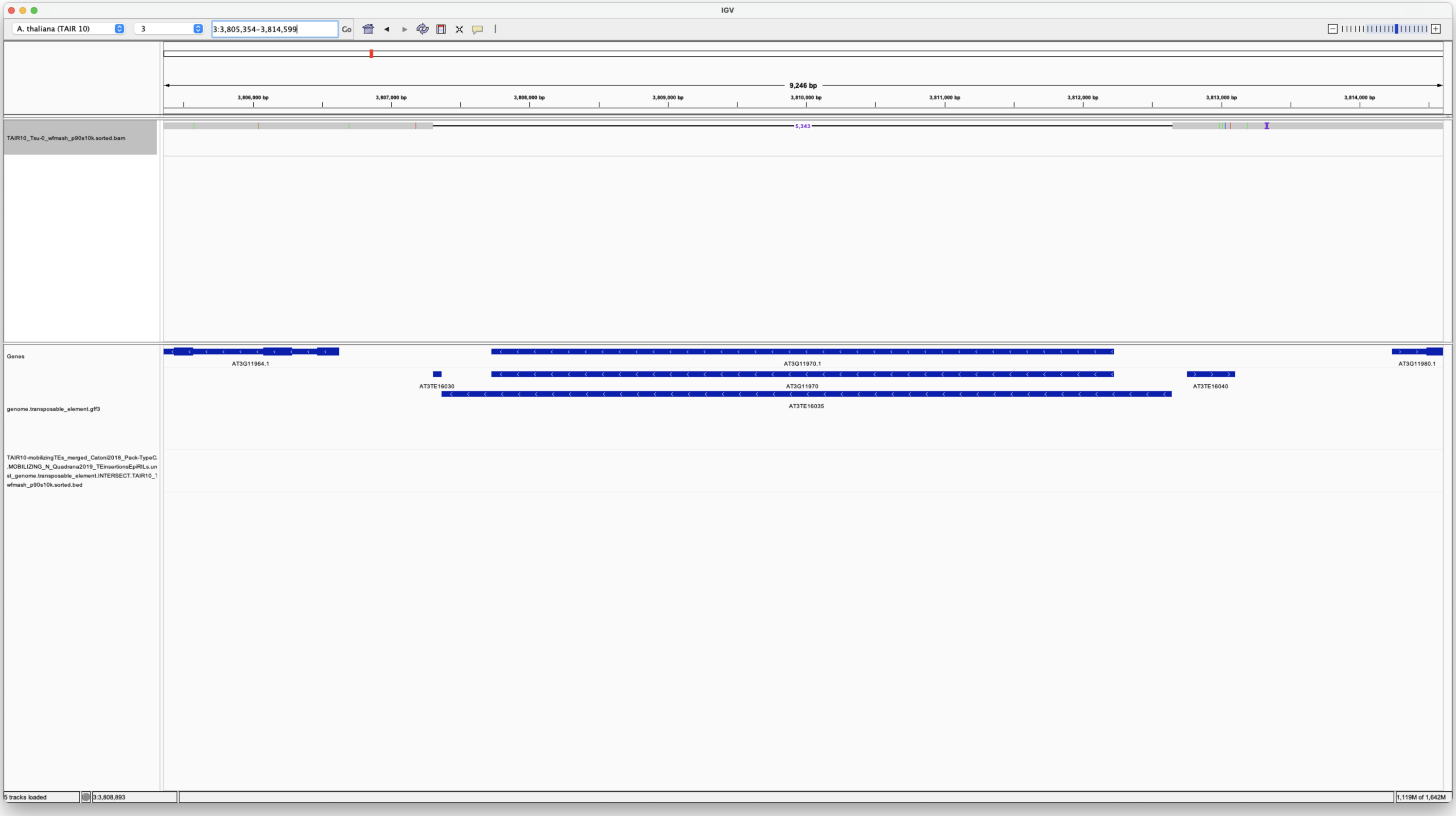

No Tsu-0

Chr4 5542430 5544862 AT4G08680

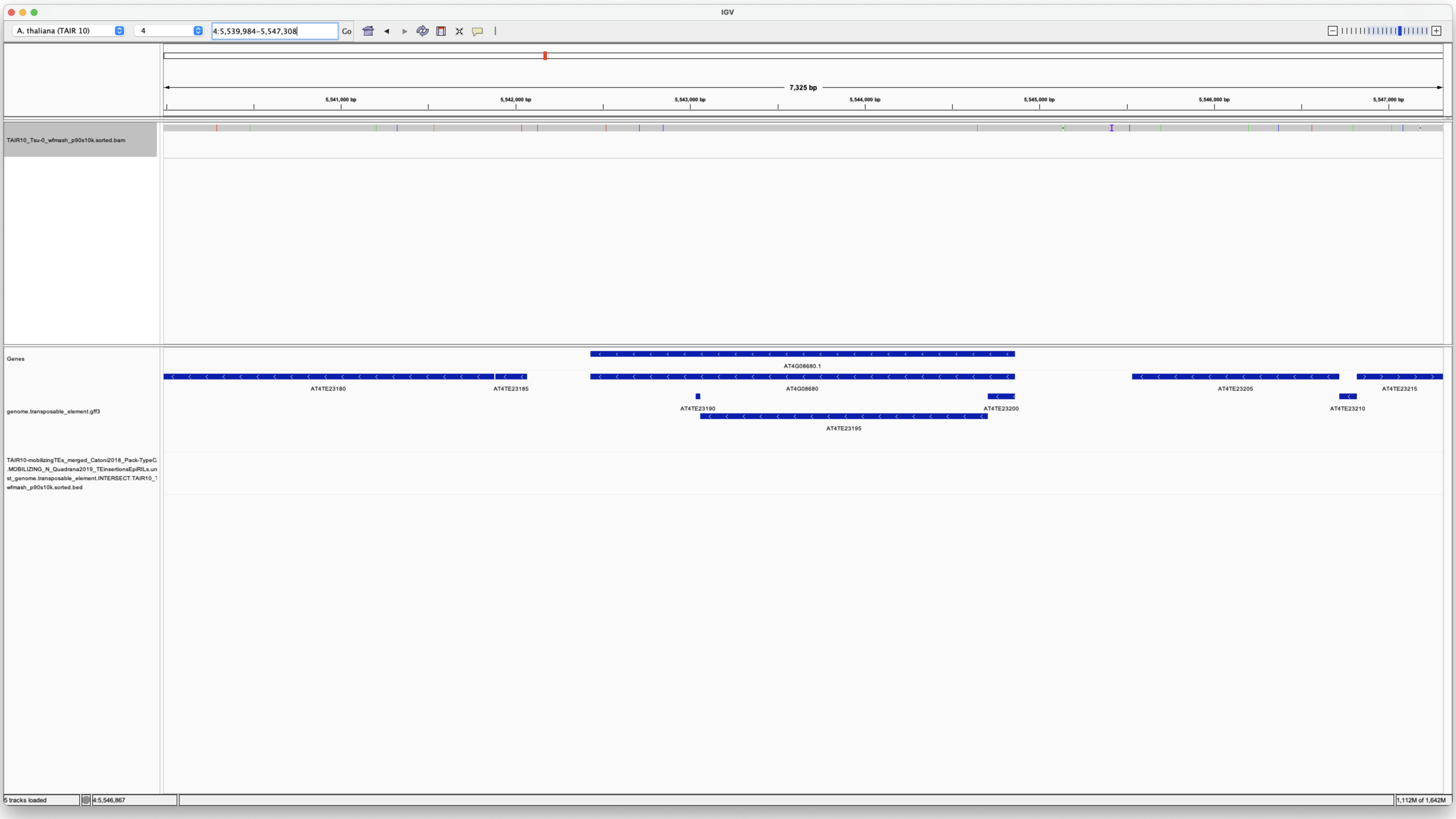

YES Tsu-0

Miura

Chr1 12942824 12947010 AT1TE42210

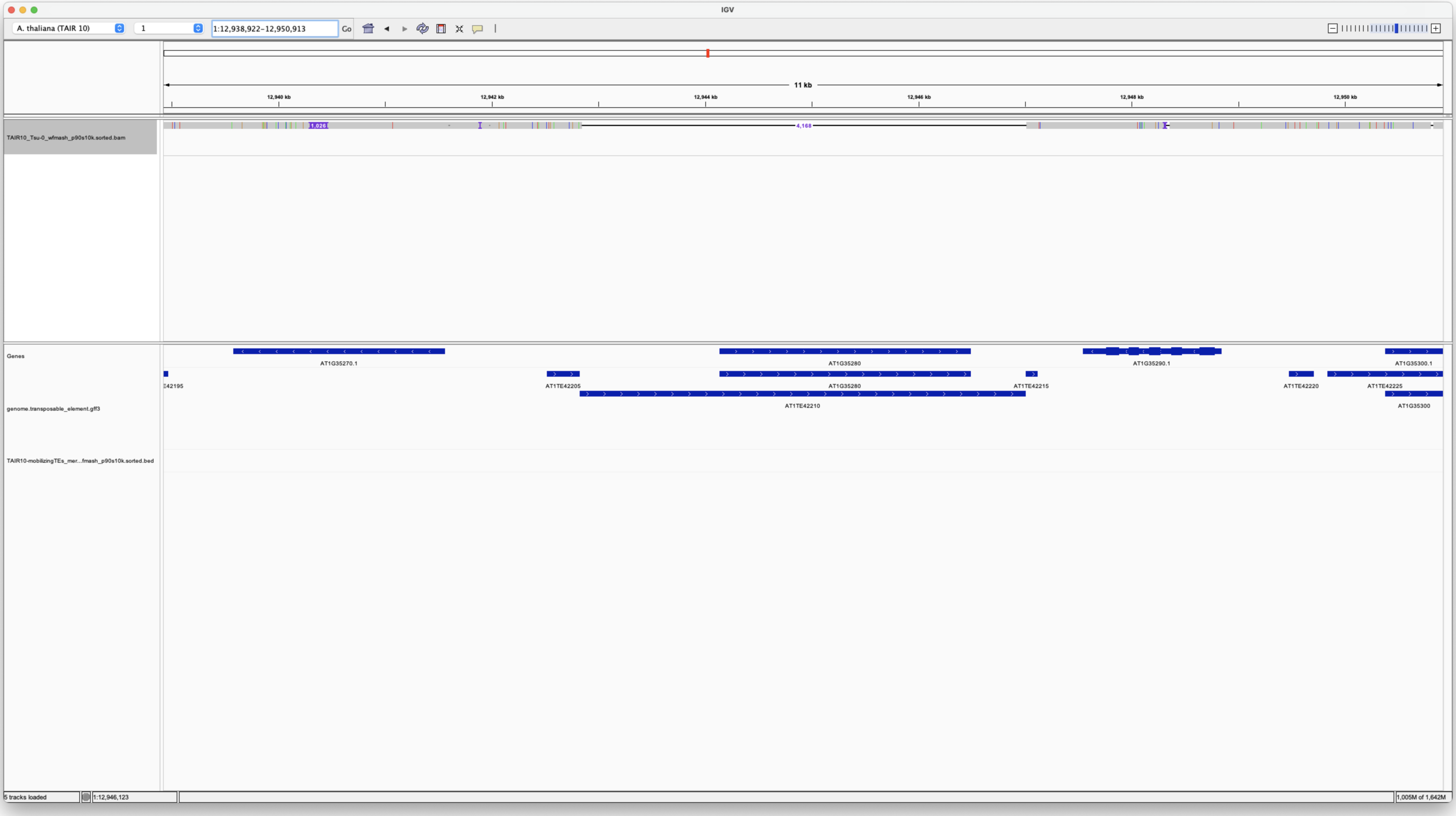

No Tsu-0

ATMU5_Comp

overlaps with ATMU1 (AT4G08680)

Quadrana et al., 2018

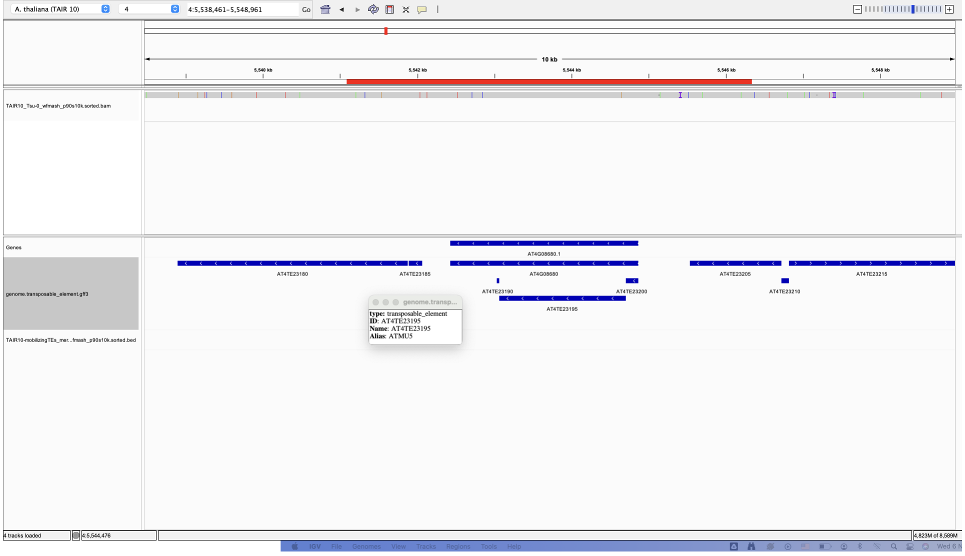
A. thaliana (TAIR 10)
4
4:5,538,461-5,548,961
Go
10 kb
5,540 kb
5,542 kb
5,544 kb
5,546 kb
5,548 kb
TAIR10_Tsu-0_wfmash_p90s10k.sorted.bam
Genes
genome.transposable_element.gff3
TAIR10-mobilizingTEs_mer...fmash_p90s10k.sorted.bed
AT4G08680.1
AT4TE23180
AT4TE23185
AT4G08680
AT4TE23205
AT4TE23215
AT4TE23190
AT4TE23200
AT4TE23210
AT4TE23195
type: transposable_element
ID: AT4TE23195
Name: AT4TE23195
Alias: ATMU5
4 tracks loaded
4:5,544,476
4,823M of 8,589M

Yes Tsu-0
