# Supplementary material for: Long-read detection of transposable element mobilization in the soma of hypomethylated Arabidopsis thaliana individuals
Source: Genome Biol. 2025 Jul 30;26:231. doi: 10.1186/s13059-025-03691-7 (PMC12312487; doi:10.1186/s13059-025-03691-7)
Supplement: Supplementary file 2 — Additional file 2. Visual inspection of somatic insertion and excision events, available at https://github.com/aerilli/Somatic-transposition_met1/tree/551df407370c6528225f404ba62a073dced14b08/Supplementary-Files/Visual_inspection. [file 13059_2025_3691_MOESM2_ESM.gz › Split_Supplementary-File4/File2_CIGAR_Insertions/File2_CIGAR_Insertions_1-33.pdf]

# CIGAR_Insertions
# Merged elements

## met1_01

> Chr1 32282511 32282511 m64079_221220_112036/81594785/ccs Chr5 875414 876434
> Chr5|875414|876433|ID=TE_MANUAL_02;Name=PAC;classification=DNA/DTC;sequence_ontology=MANUAL;identity=MANUAL;method=MANUAL;ID=TE_MANUAL_02;sequence_ontology=MANUAL met1_01

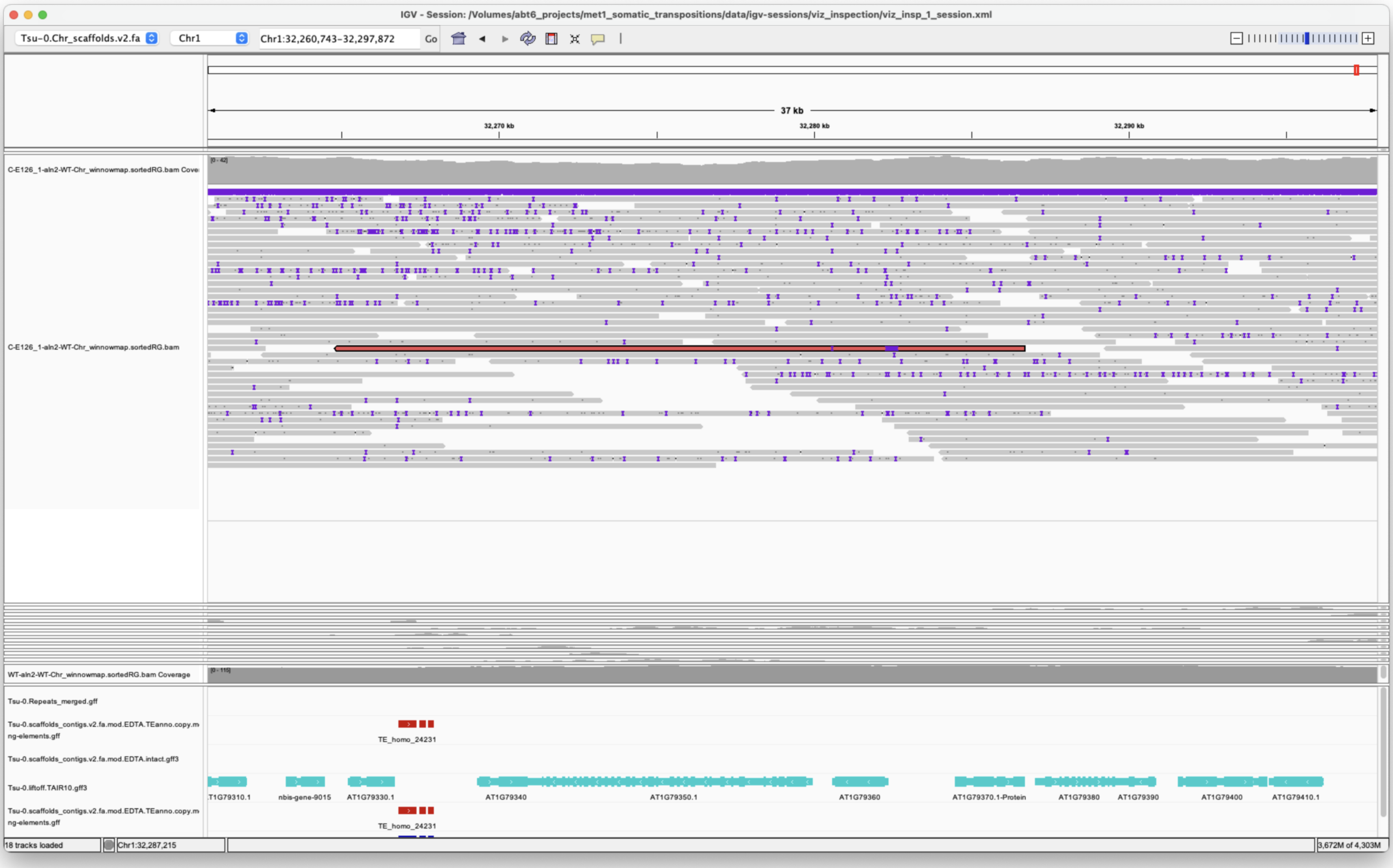


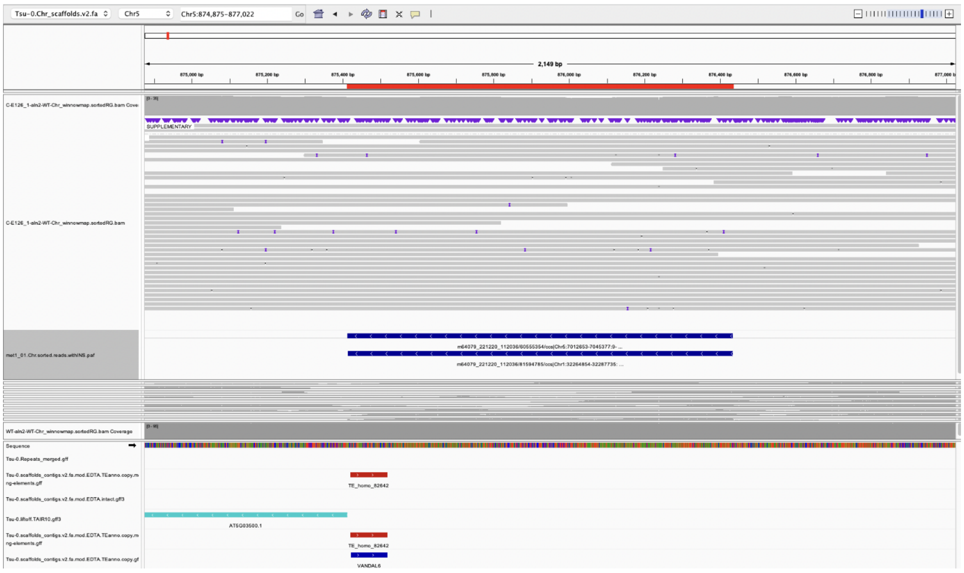


*Confirmed*

> Chr5 7026319 7026319 m64079_221220_112036/60555354/ccs Chr5 875413 876434
> Chr5|875414|876433|ID=TE_MANUAL_02;Name=PAC;classification=DNA/DTC;sequence_ontology=MANUAL;identity=MANUAL;method=MANUAL;ID=TE_MANUAL_02;sequence_ontology=MANUAL met1_01

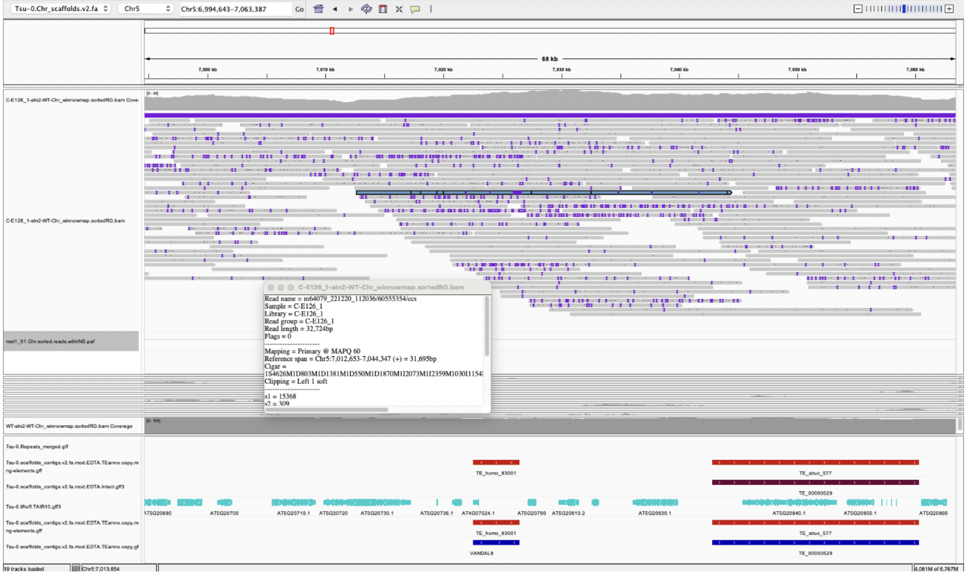


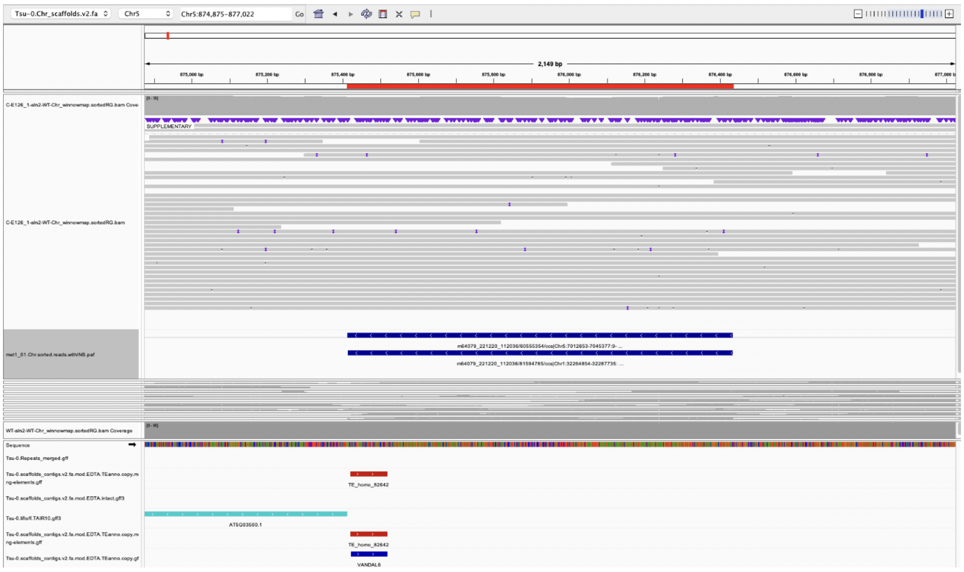


*Confirmed*

> Chr5 15902949 15902949 m64079_221220_112036/45091048/ccs Chr5 19152825 19160826 Chr5|19152829|19160825|ID=TE_homo_95640;Name=VANDAL21;classification=DNA/Mutator;sequence_ontology=SO:0002280;identity=0.976;method=homology;ID=TE_homo_98501;sequence_ontology=SO:0002280|ID=TE_homo_95641;Name=VANDAL21;classification=DNA/Mutator;sequence_ontology=SO:0002280;identity=0.966;method=homology;ID=TE_homo_98502;sequence_ontology=SO:0002280 met1_01

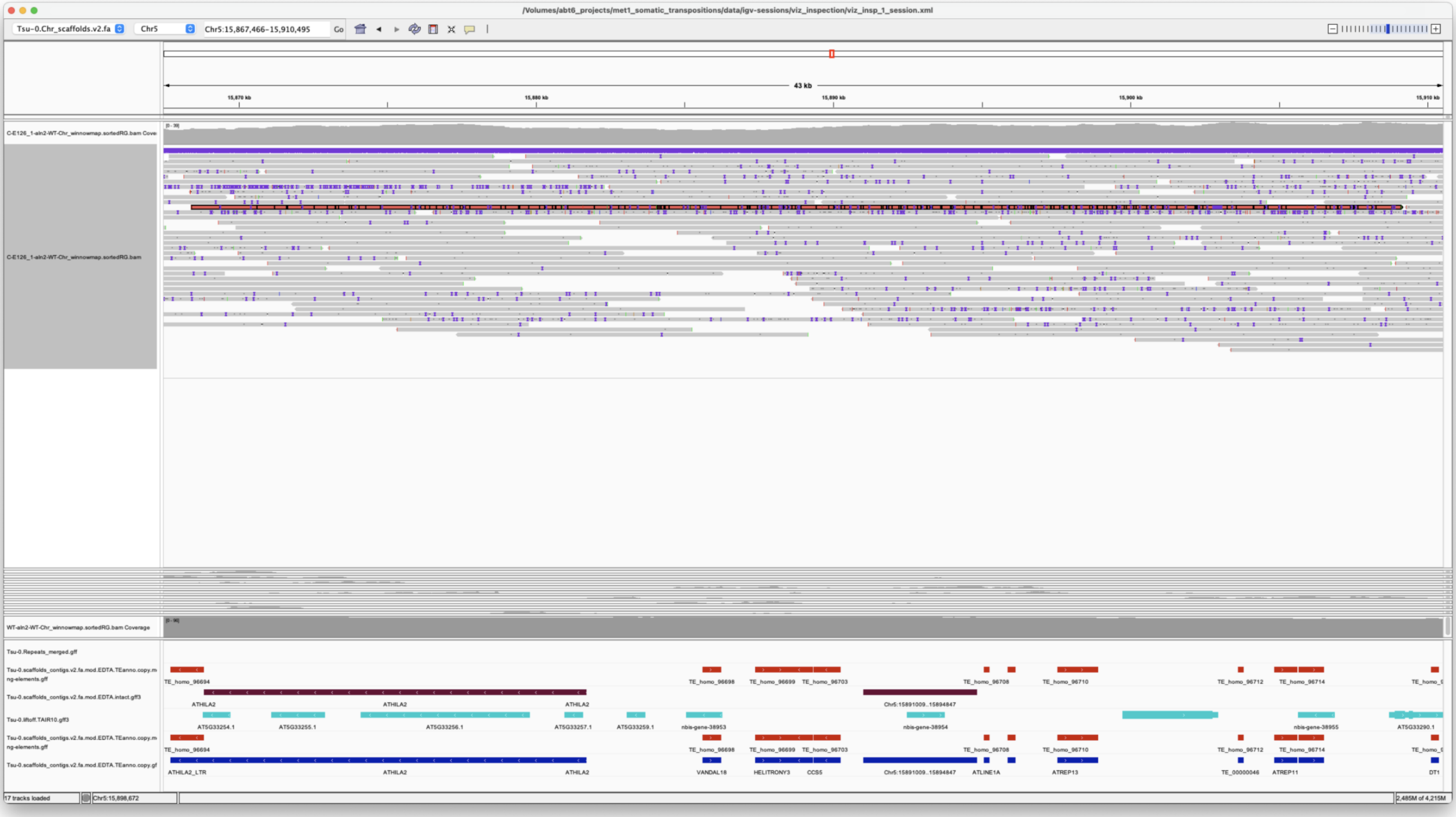

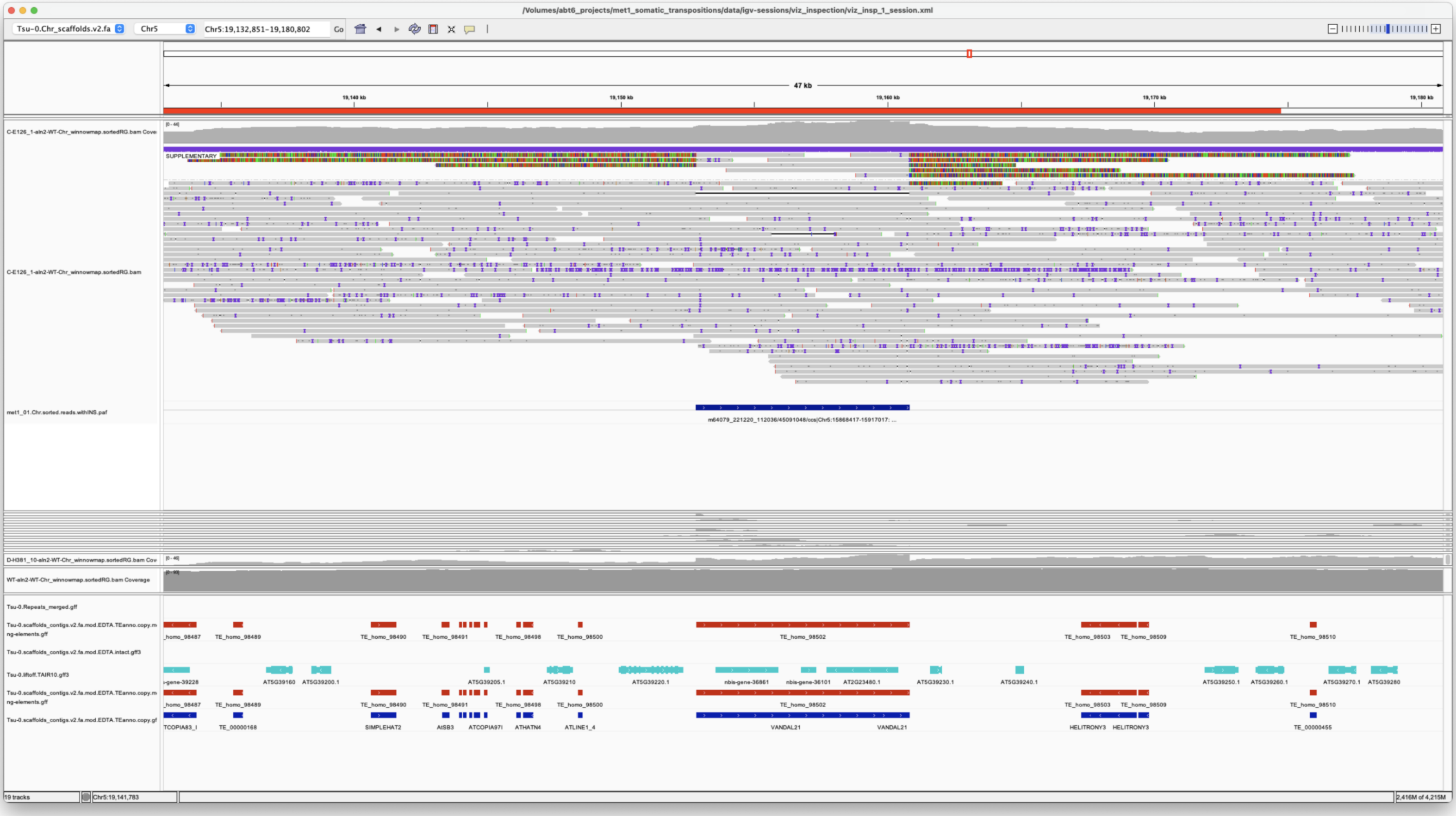

VANDAL21 insertion

*Confirmed*

---

---

---

## met1_02

> Chr1 6039355 6039355 m64079_240212_113350/111674010/ccs Chr3 16344522 16352496 Chr3|16344522|16352496|ID=TE_homo_60420;Name=VANDAL6;classification=DNA/Mutator;sequence_ontology=SO:0002280;identity=0.969;method=homology;ID=TE_homo_62001;sequence_ontology=SO:0002280 met1_02

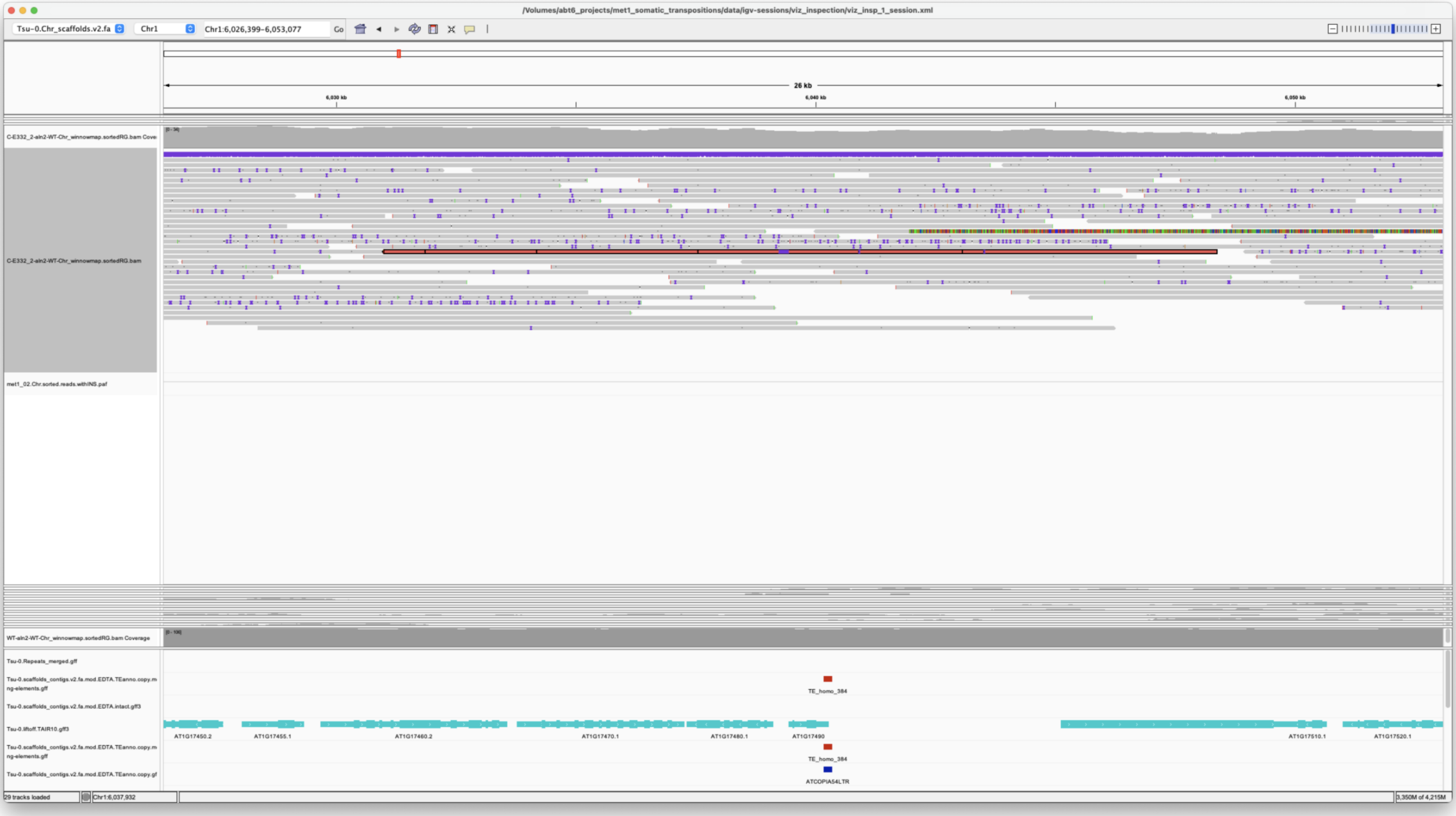


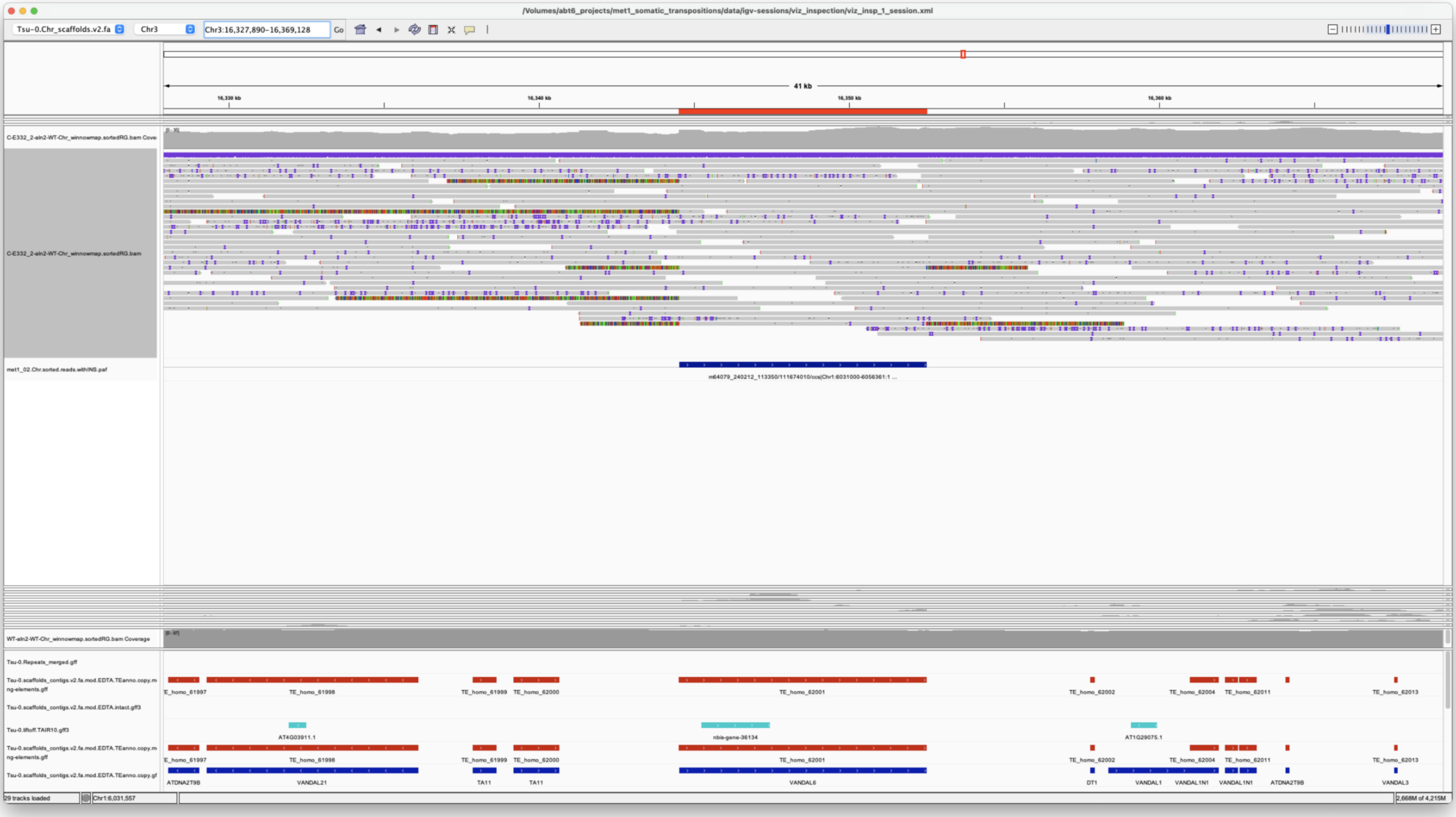


*Confirmed*

> Chr1 20639051 20639051 m64079_221220_112036/15731028/ccs Chr5 875413 876434 Chr5|875414|876433|ID=TE_MANUAL_02;Name=PAC;classification=DNA/DTC;sequence_ontology=MANUAL;identity=MANUAL;method=MANUAL;ID=TE_MANUAL_02;sequence_ontology=MANUAL met1_02

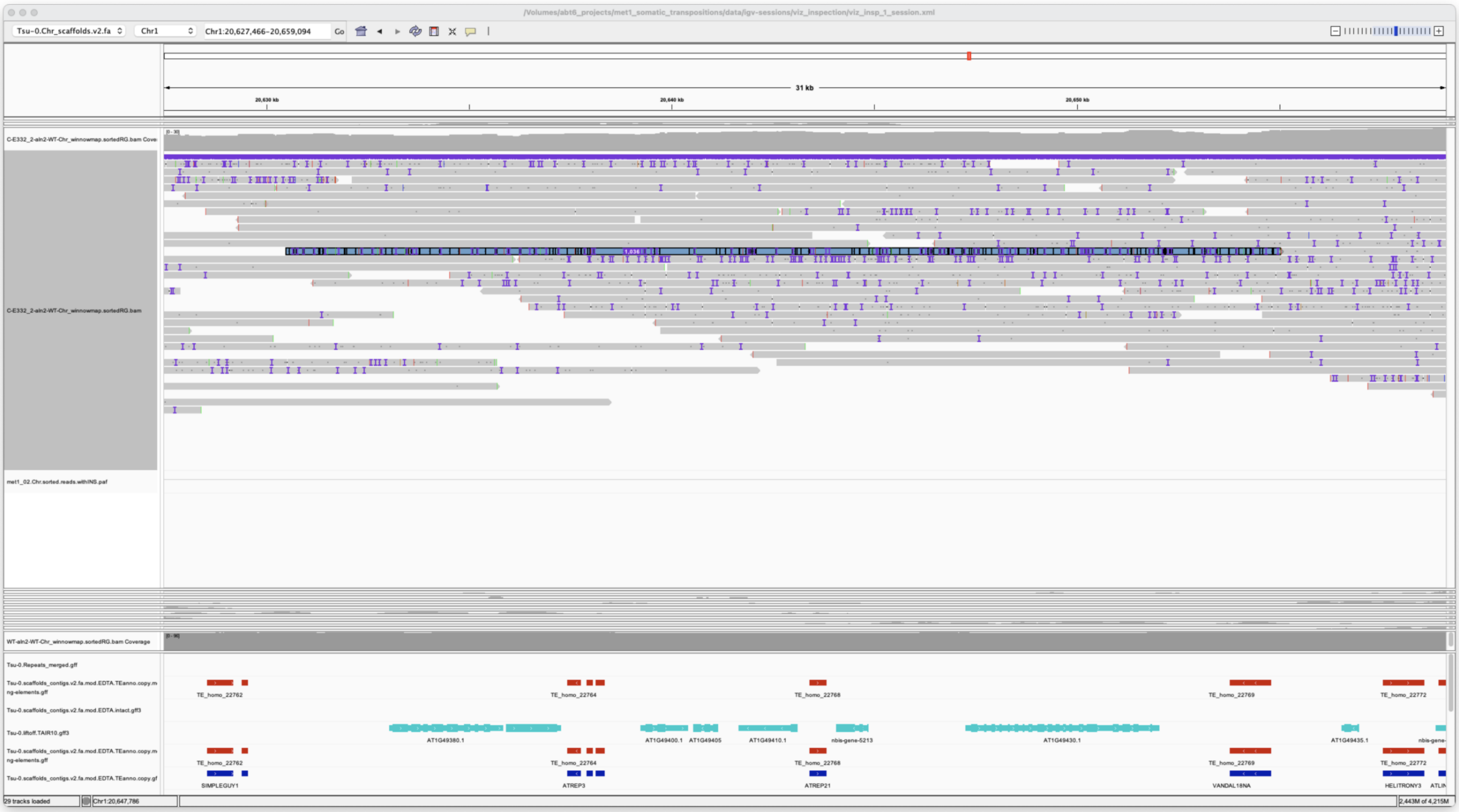


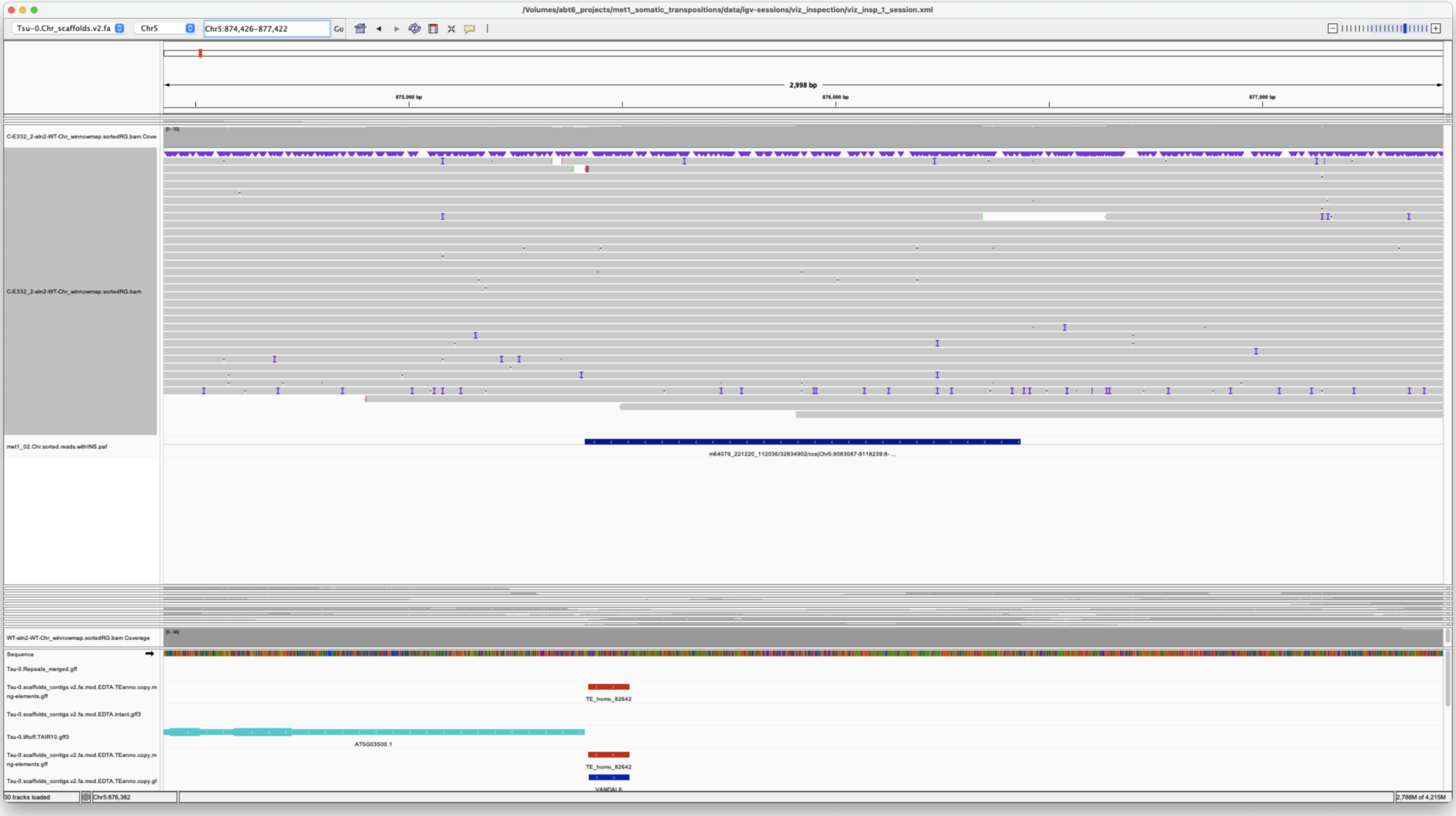


*Confirmed*

> Chr1 32269307 32269307 m64079_240212_113350/161613456/ccs Chr5 19152823 19160826 Chr5|19152829|19160825|ID=TE_homo_95640;Name=VANDAL21;classification=DNA/Mutator;sequence_ontology=SO:0002280;identity=0.976;method=homology;ID=TE_homo_98501;sequence_ontology=SO:0002280|ID=TE_homo_95641;Name=VANDAL21;classification=DNA/Mutator;sequence_ontology=SO:0002280;identity=0.966;method=homology;ID=TE_homo_98502;sequence_ontology=SO:0002280 met1_02

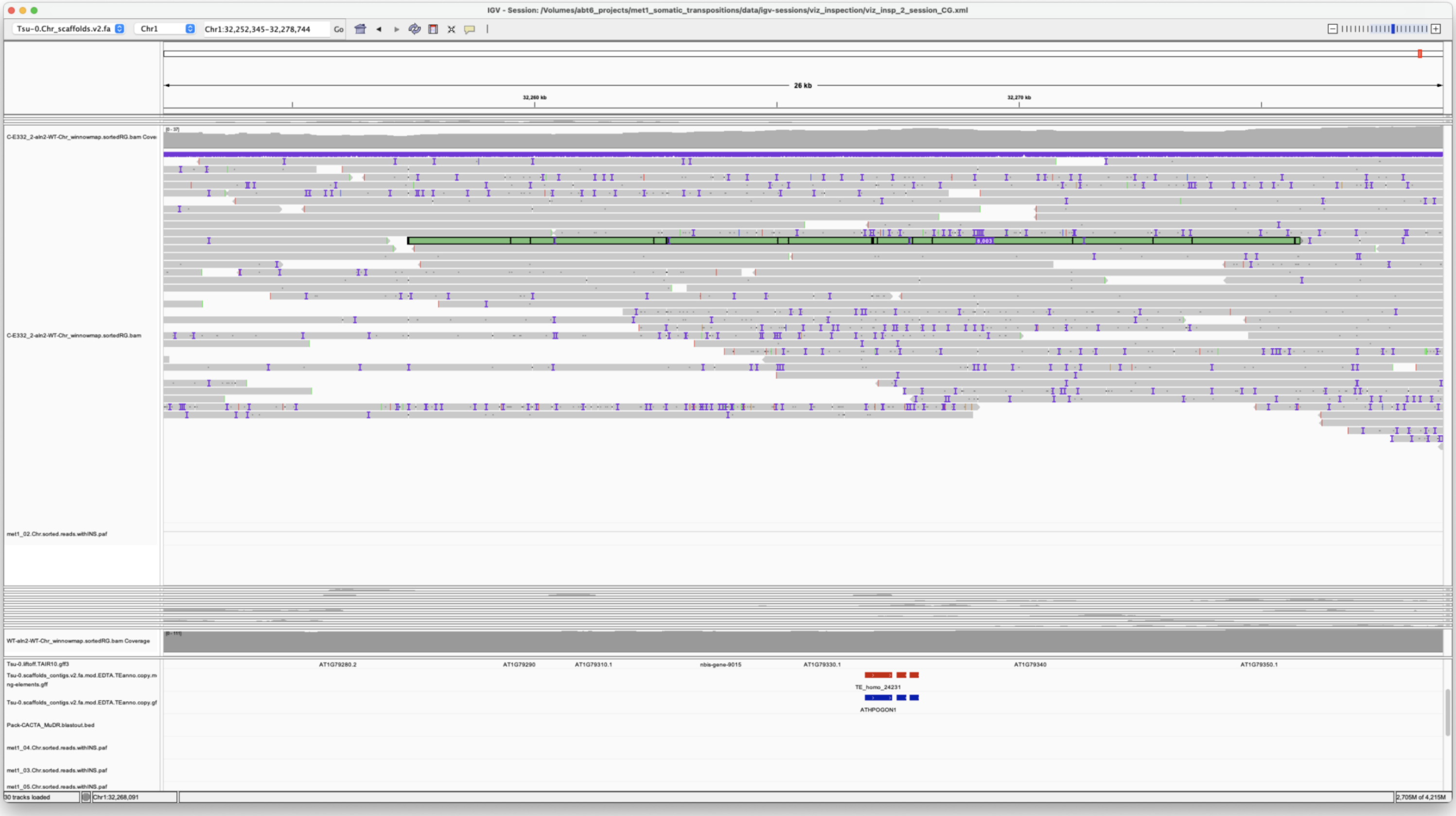


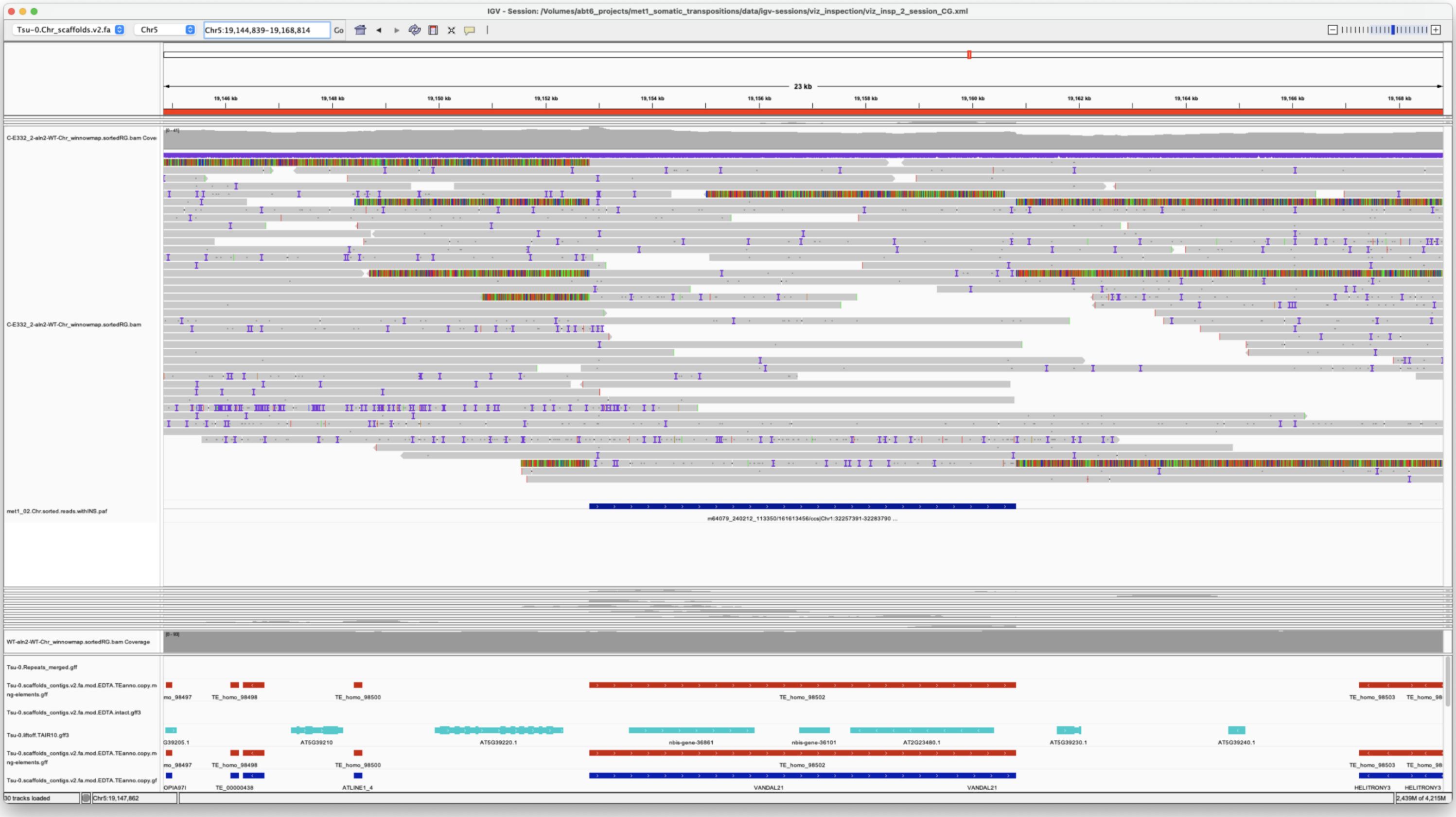


*Confirmed*

---

> Chr4 7023215 7023217
> m64079_221220_112036/29032572/ccs**m64079_221220_112036/41615485/ccs**m64079_221220_112036/42140263/ccs**m64079_221220_112036/60687065/ccs**m64079_240212_113350/120195183/ccs**m64079_240212_113350/15402943/ccs Chr4 7023215 7024283 .|-1|-2|.**.|-1|-2|.**.|-1|-2|.**.|-1|-2|.**.|-1|-2|.**.|-1|-2|. met1_02
> Chr4 7023225 7023225 m64079_221220_112036/5113584/ccs Chr4 7023224 7024291 .|-1|-2|. met1_02
> Chr4 7024209 7024209 m64079_221220_112036/47776303/ccs Chr4 7023140 7024207 .|-1|-2|. met1_02
> Chr4 7024279 7024279 m64079_221220_112036/10813820/ccs Chr4 7023210 7024277 .|-1|-2|. met1_02

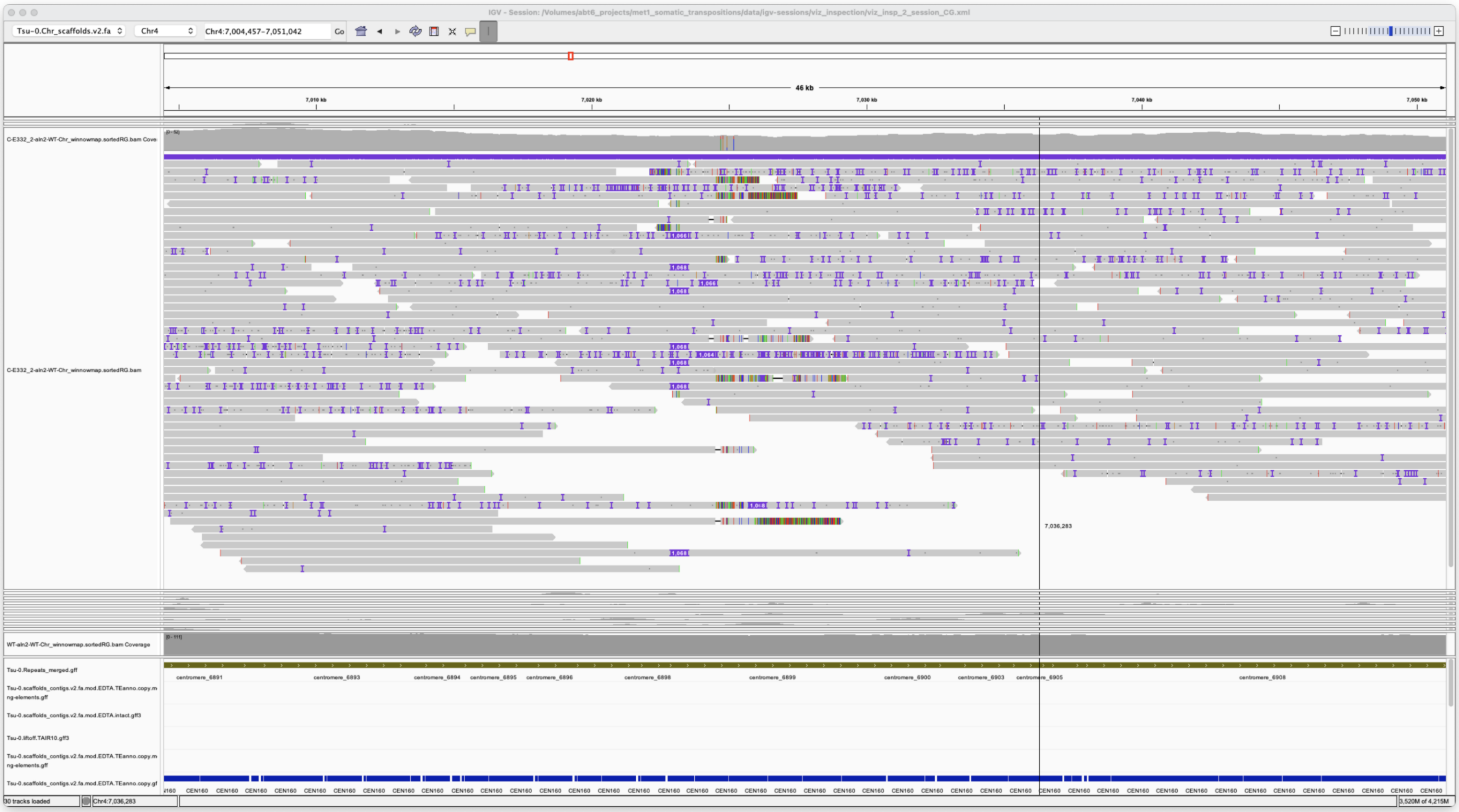

Centromeric rearrangements

*unsupported*

*Rearrangement*

---

> Chr5 9101100 9101100 m64079_221220_112036/32834902/ccs Chr5 875413 876434
> Chr5|875414|876433|ID=TE_MANUAL_02;Name=PAC;classification=DNA/DTC;sequence_ontology=MANUAL;identity=MANUAL;method=MANUAL;ID=TE_MANUAL_02;sequence_ontology=MANUAL met1_02

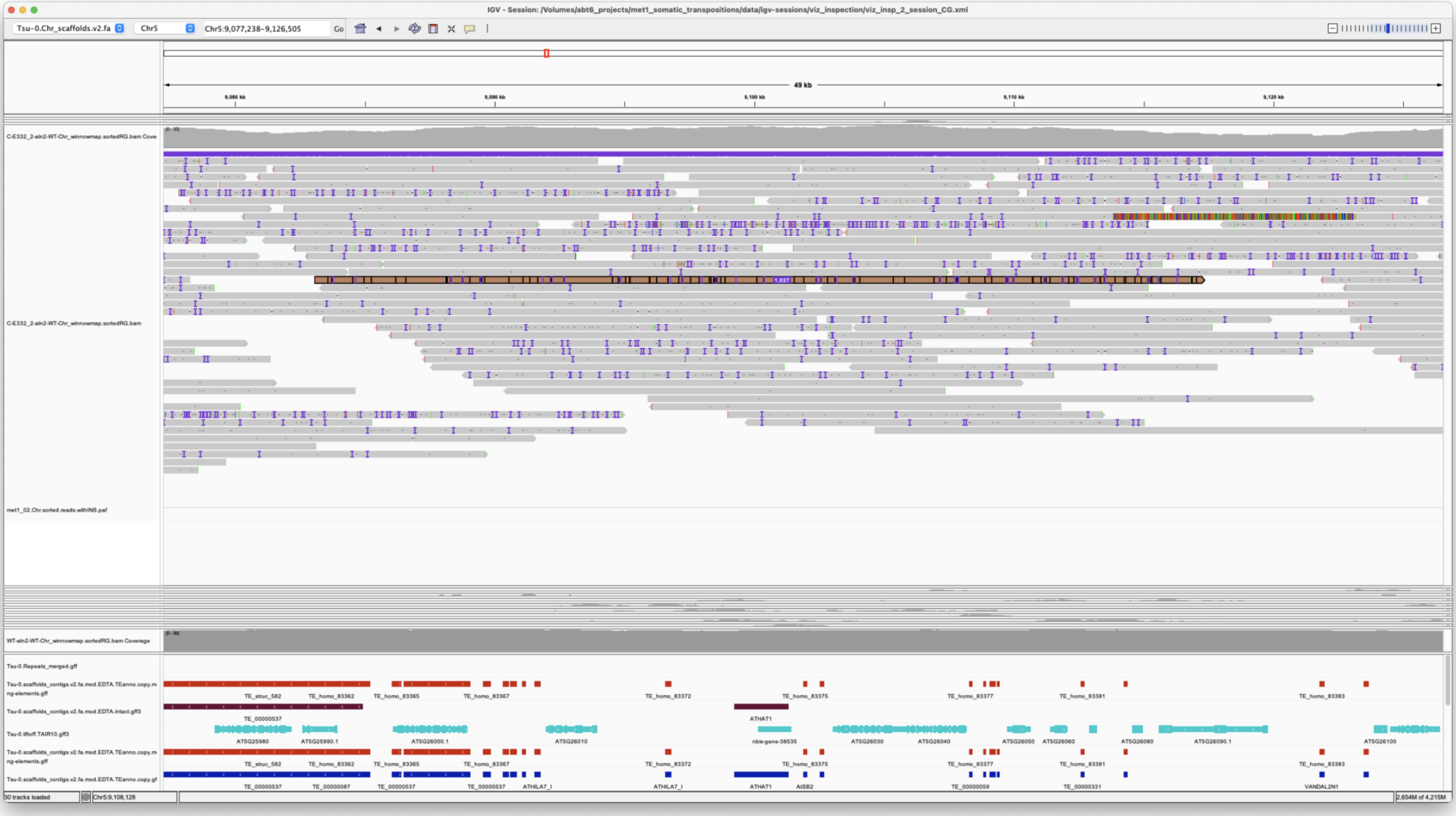

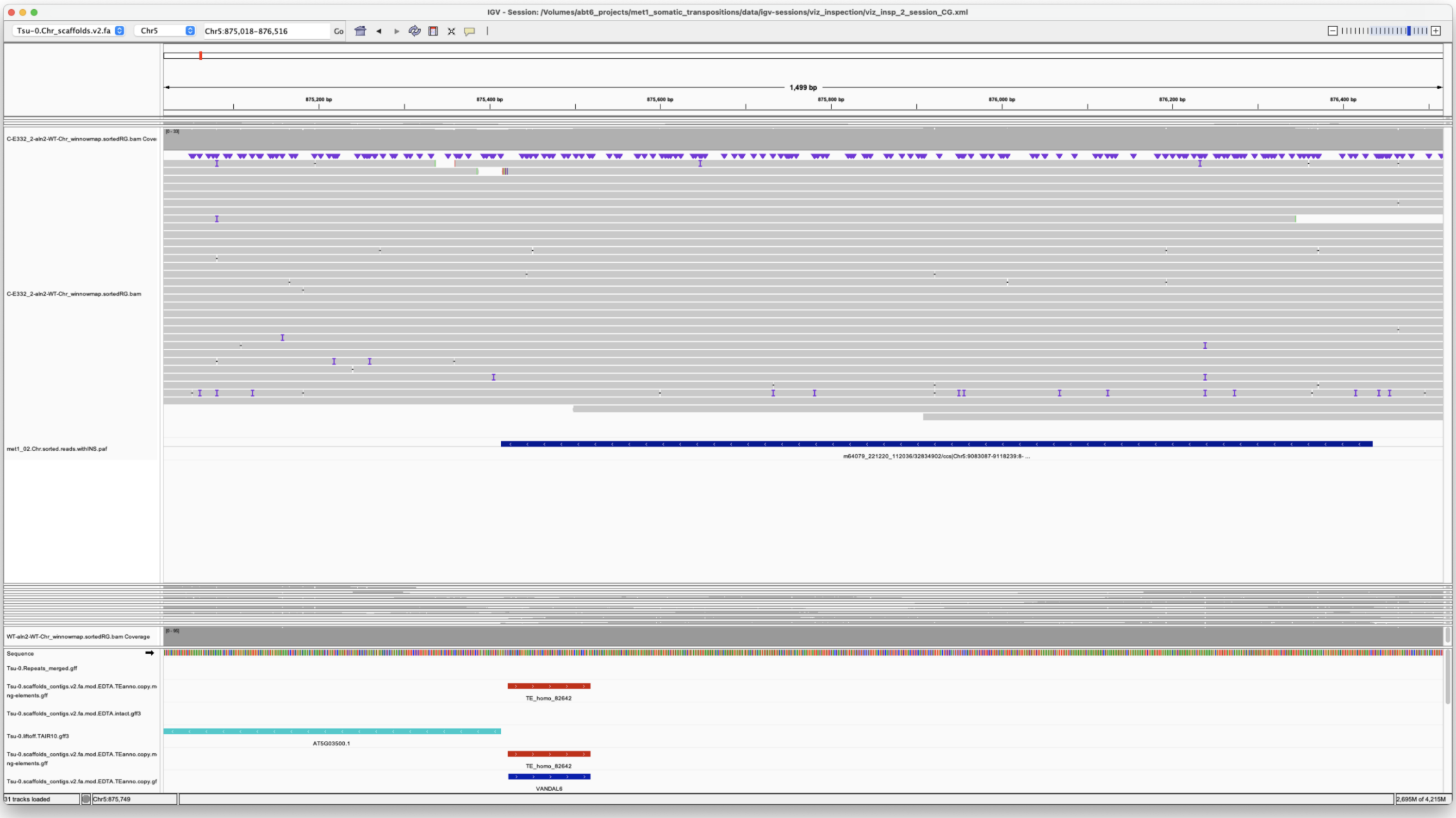


*Confirmed*

## met1_03

> Chr2 6768455 6768455 m64079_221220_112036/112396489/ccs Chr2 6768454 6769622 Chr2|6734994|6775797|ID=TE_homo_41523;Name=ATMSAT1;classification=Satellite/Satellite;sequence_ontology=SO:0000005;identity=0.934;method=homology;ID=TE_homo_42370;sequence_ontology=SO:0000005 met1_03

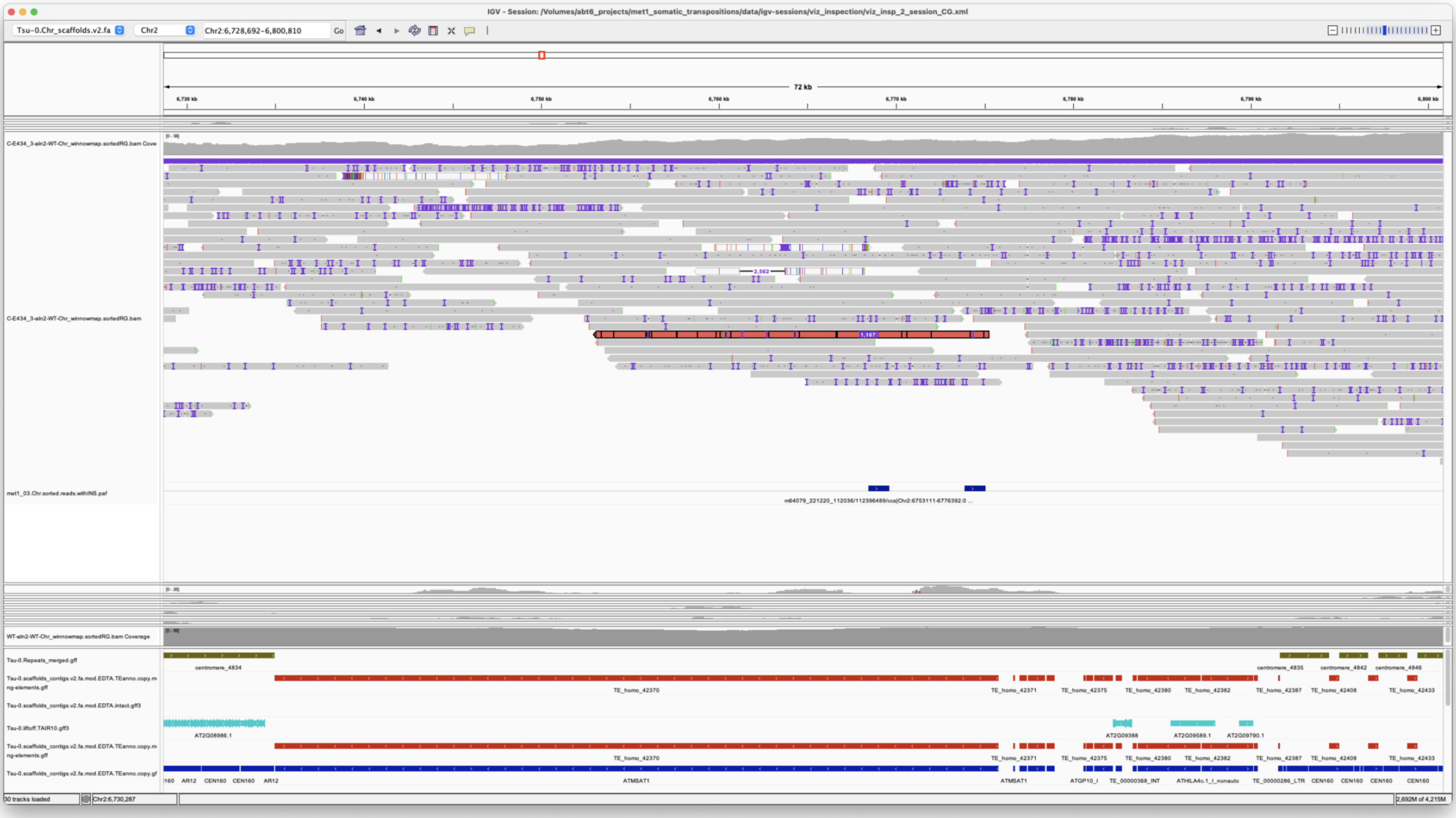


Satellite rearrangement

*unsupported*

*Rearrangement*

## met1_04

**NONE**

## met1_05

> Chr1 7025171 7025171 m64079_221220_112036/97912206/ccs Chr3 16344522 16352504
> Chr3|16344522|16352496|ID=TE_homo_60420;Name=VANDAL6;classification=DNA/Mutator;sequence_ontology=SO:0002280;identity=0.969;method=homology;ID=TE_homo_62001;sequence_ontology=SO:0002280 met1_05

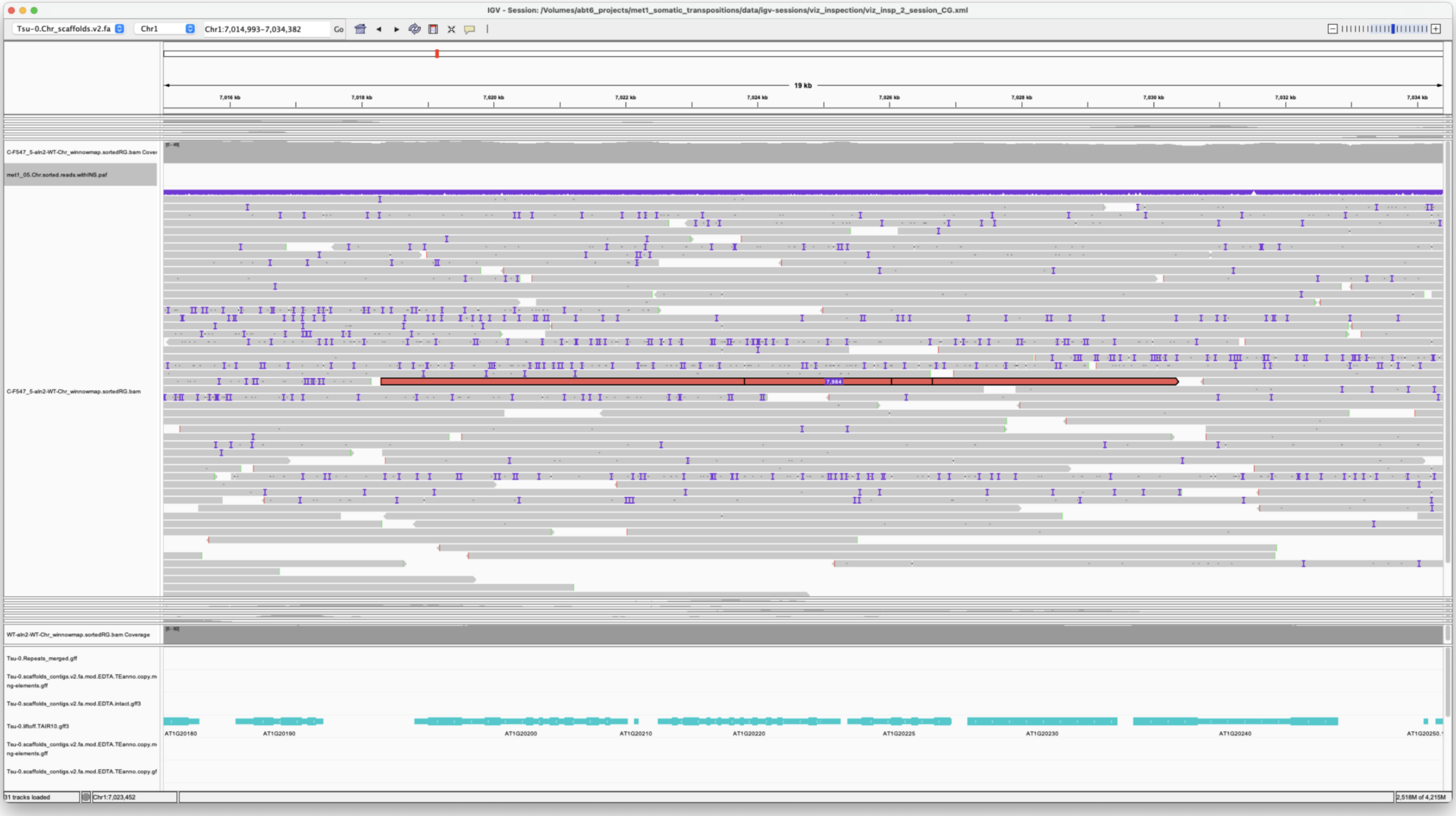

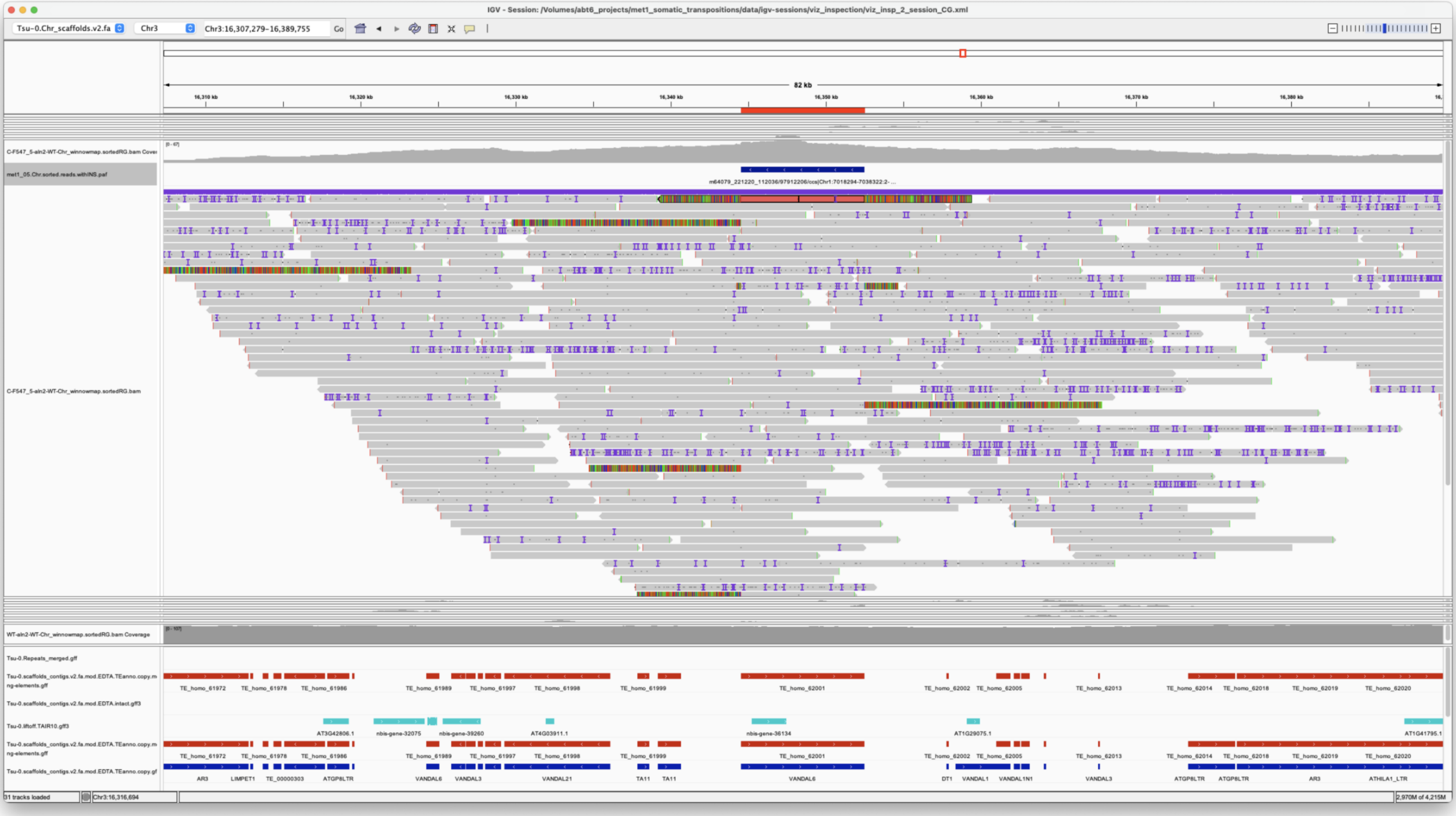

*Confirmed*

Chr2 11071303 11071303 m64079_240212_113350/170396218/ccs Chr1 11941106 11946436 Chr1|11941106|11946435|ID=LTRRT_5;Name=ATCOPIA93.2_Evade;Classification=LTR/Copia;Sequence_ontology=SO:0002264;ltr_identity=1.0000;Method=structural;motif=TACA;tsd=ATATG met1_05

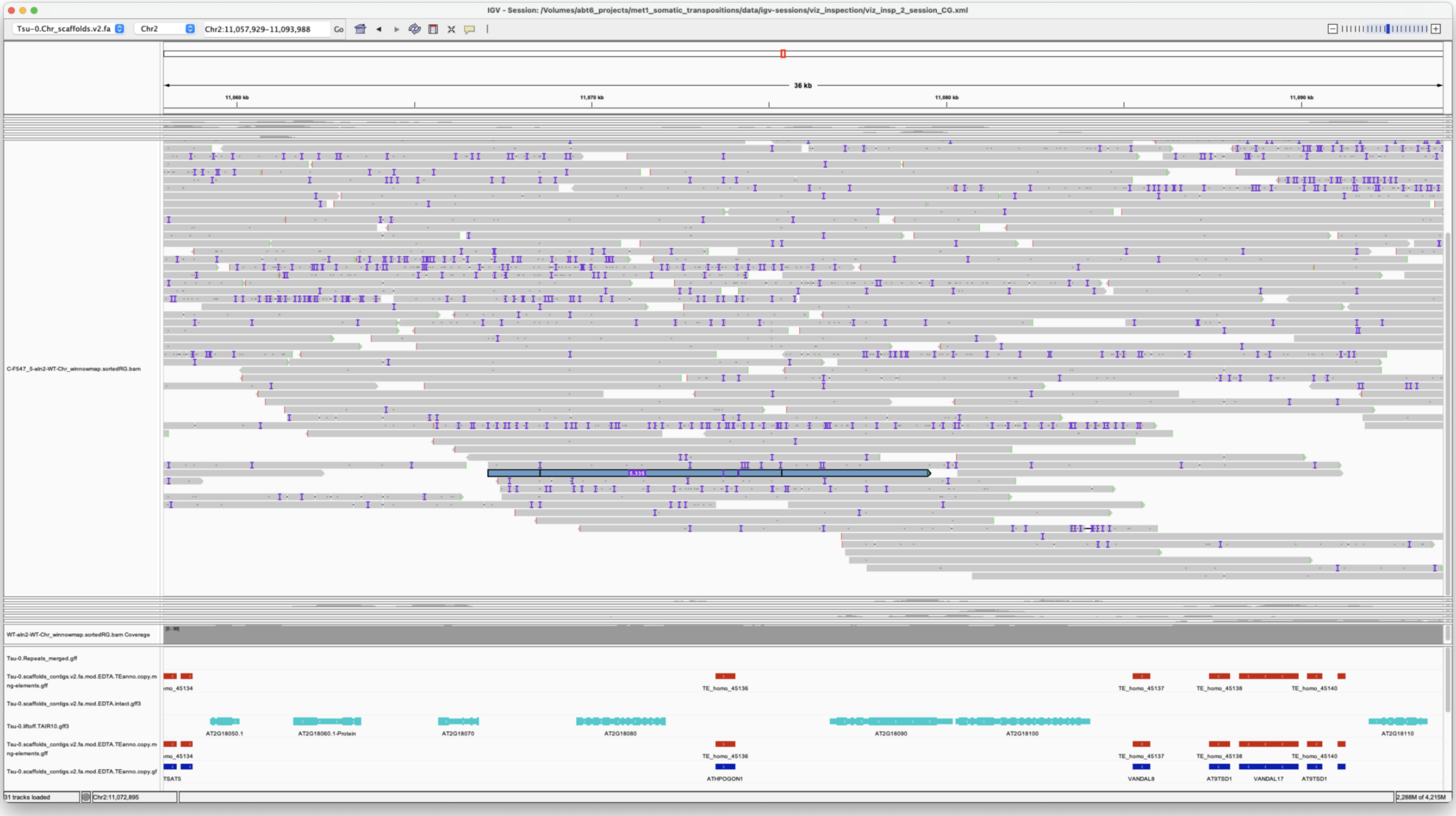

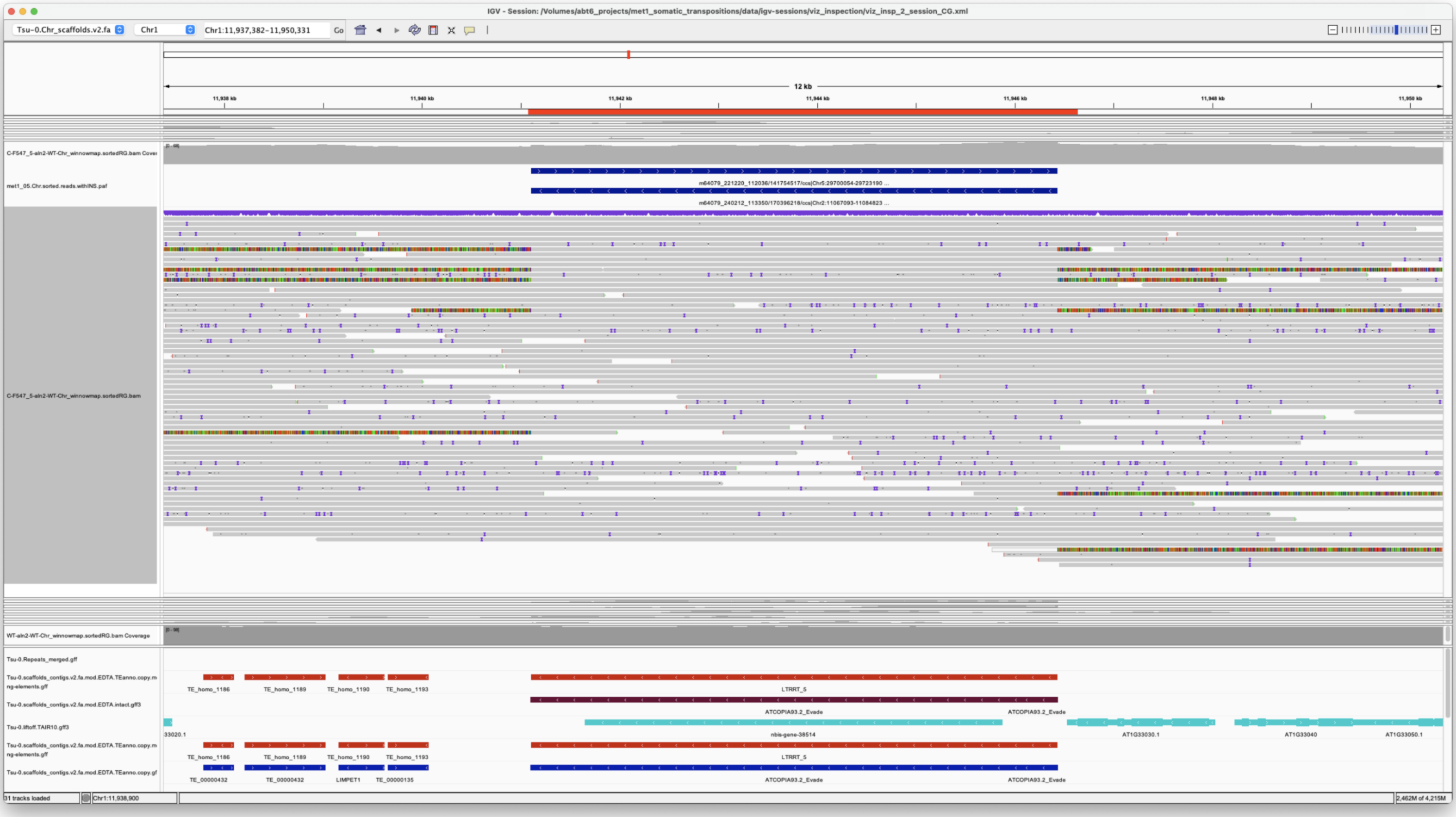

*Confirmed*

Chr3 2809693 2809693 m64079_240212_113350/9307420/ccs Chr5 875413 876434 Chr5|875414|876433|ID=TE_MANUAL_02;Name=PAC;classification=DNA/DTC;sequence_ontology=MANUAL;identity=MANUAL;method=MANUAL;ID=TE_MANUAL_02;sequence_ontology=MANUAL met1_05

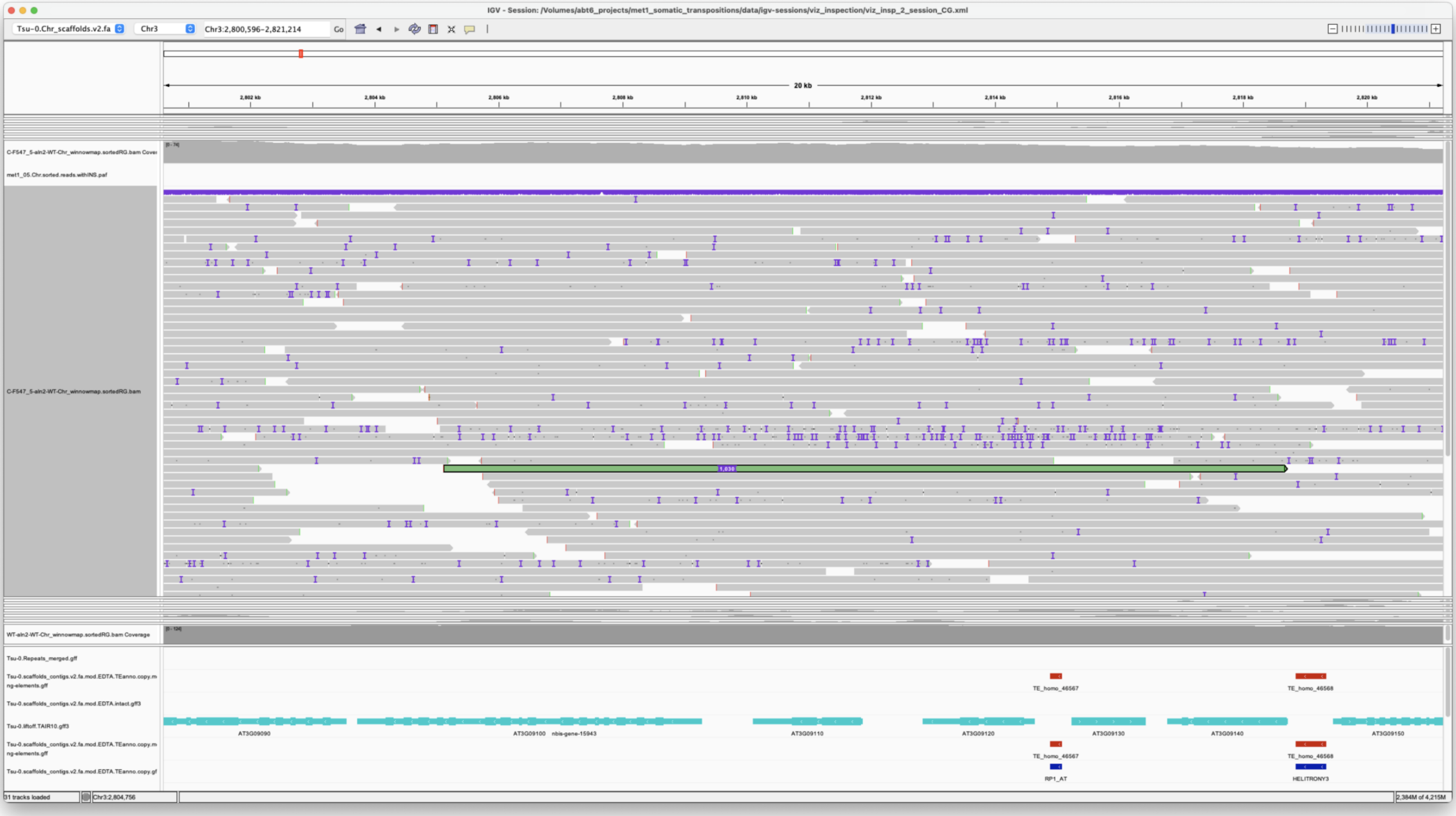

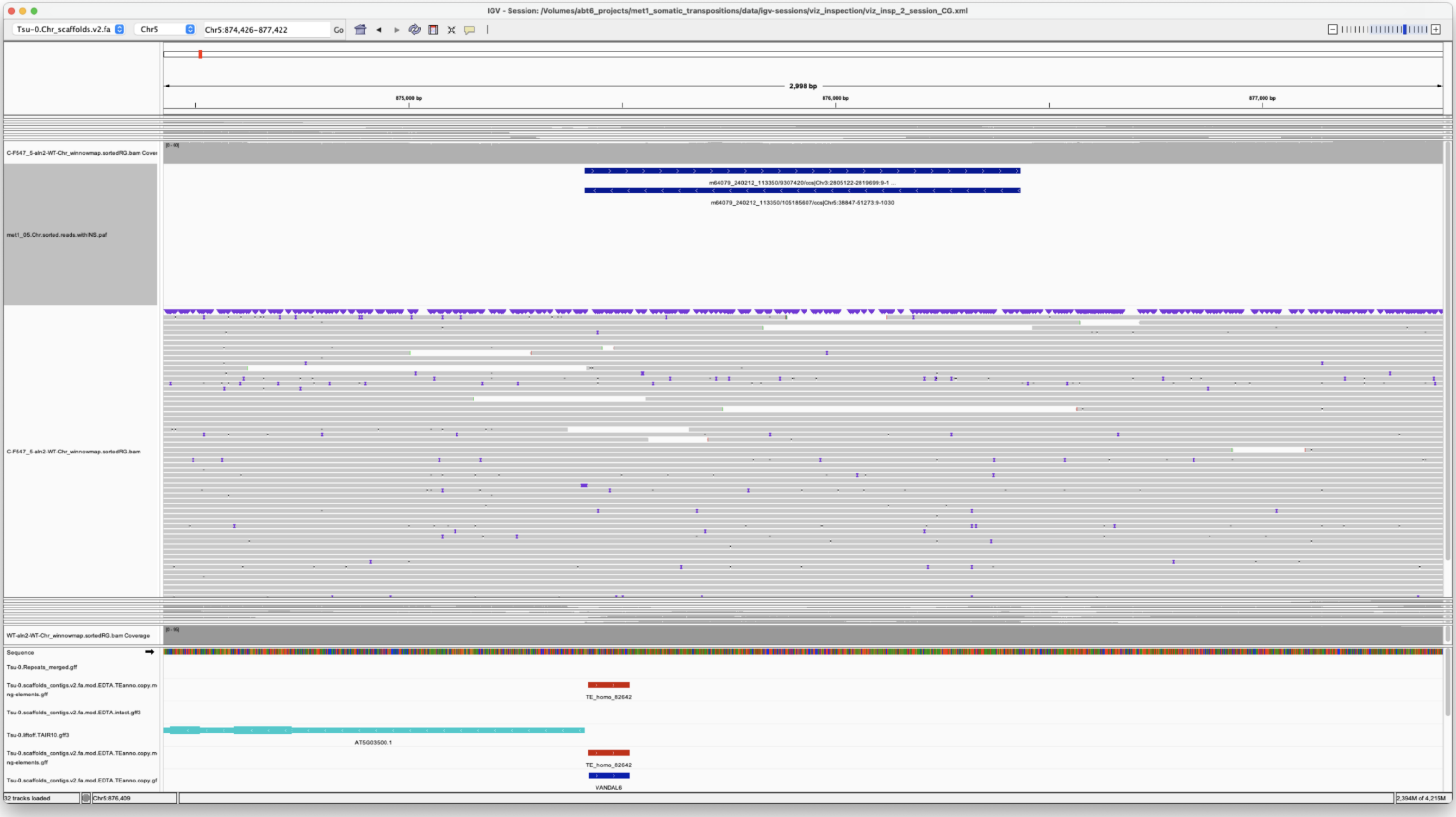

Pack-Type

*Confirmed*

---

> Chr5 49106 49106 m64079_240212_113350/105185607/ccs Chr5 875413 876434
> Chr5|875414|876433|ID=TE_MANUAL_02;Name=PAC;classification=DNA/DTC;sequence_ontology=MANUAL;identity=MANUAL;method=MANUAL;ID=TE_MANUAL_02;sequence_ontology=MANUAL met1_05

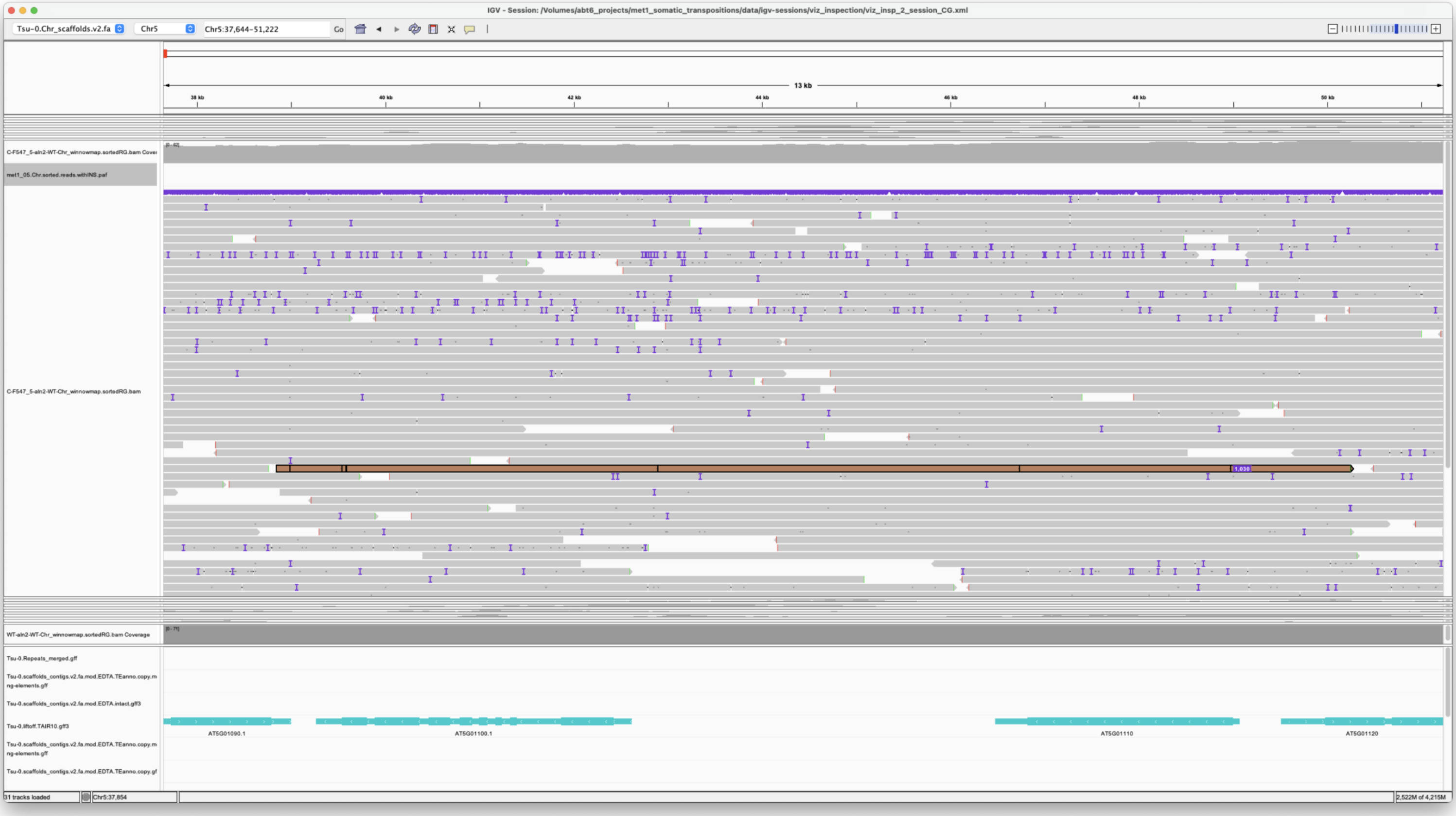


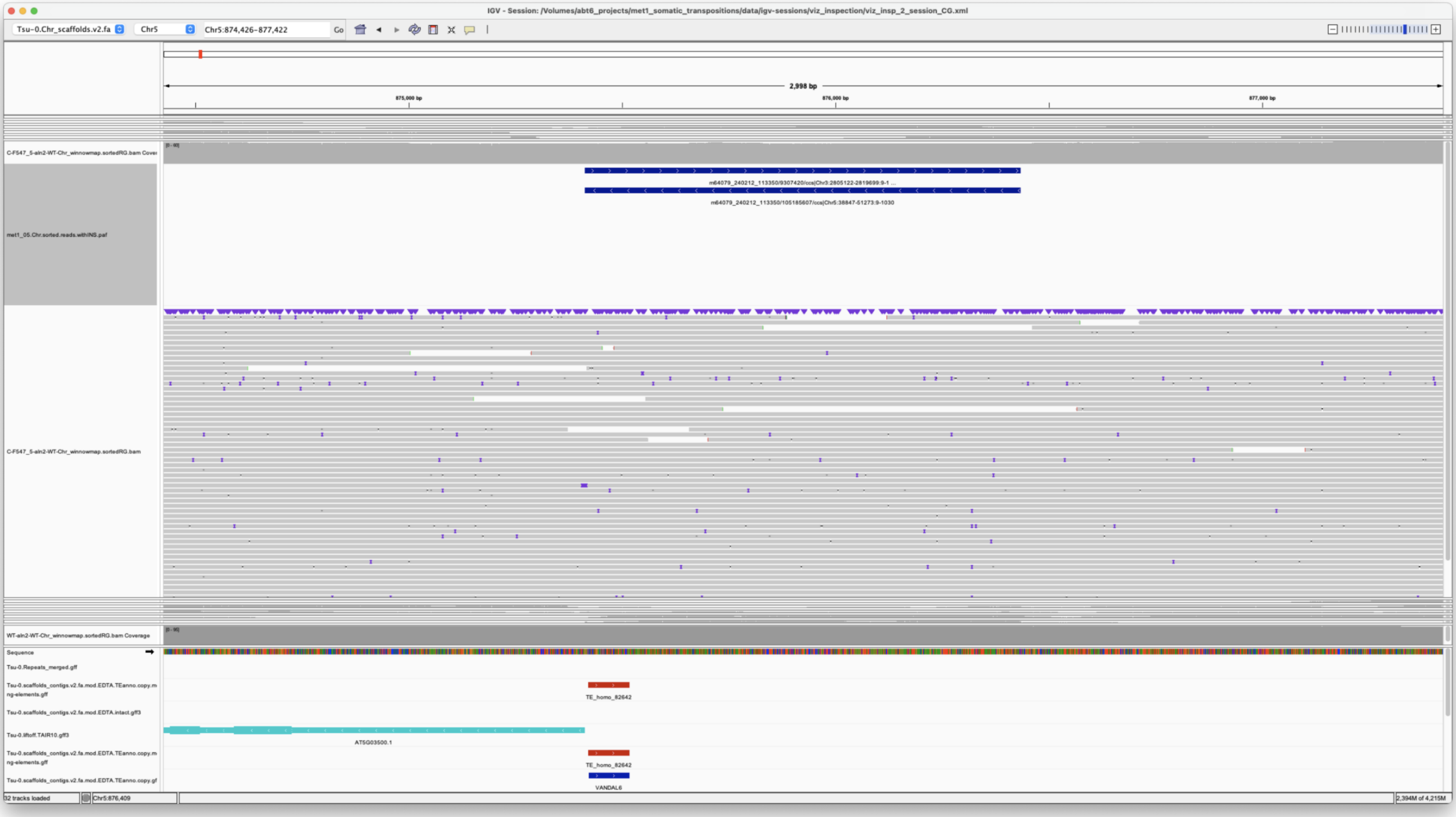


Pack-Type

*Confirmed*

---

> Chr5 28025714 28025714 m64079_221220_112036/166528667/ccs Chr5 19152825 19160826 Chr5|19152829|19160825|ID=TE_homo_95640;Name=VANDAL21;classification=DNA/Mutator;sequence_ontology=SO:0002280;identity=0.976;method=homology;ID=TE_homo_98501;sequence_ontology=SO:0002280|ID=TE_homo_95641;Name=VANDAL21;classification=DNA/Mutator;sequence_ontology=SO:0002280;identity=0.966;method=homology;ID=TE_homo_98502;sequence_ontology=SO:0002280 met1_05

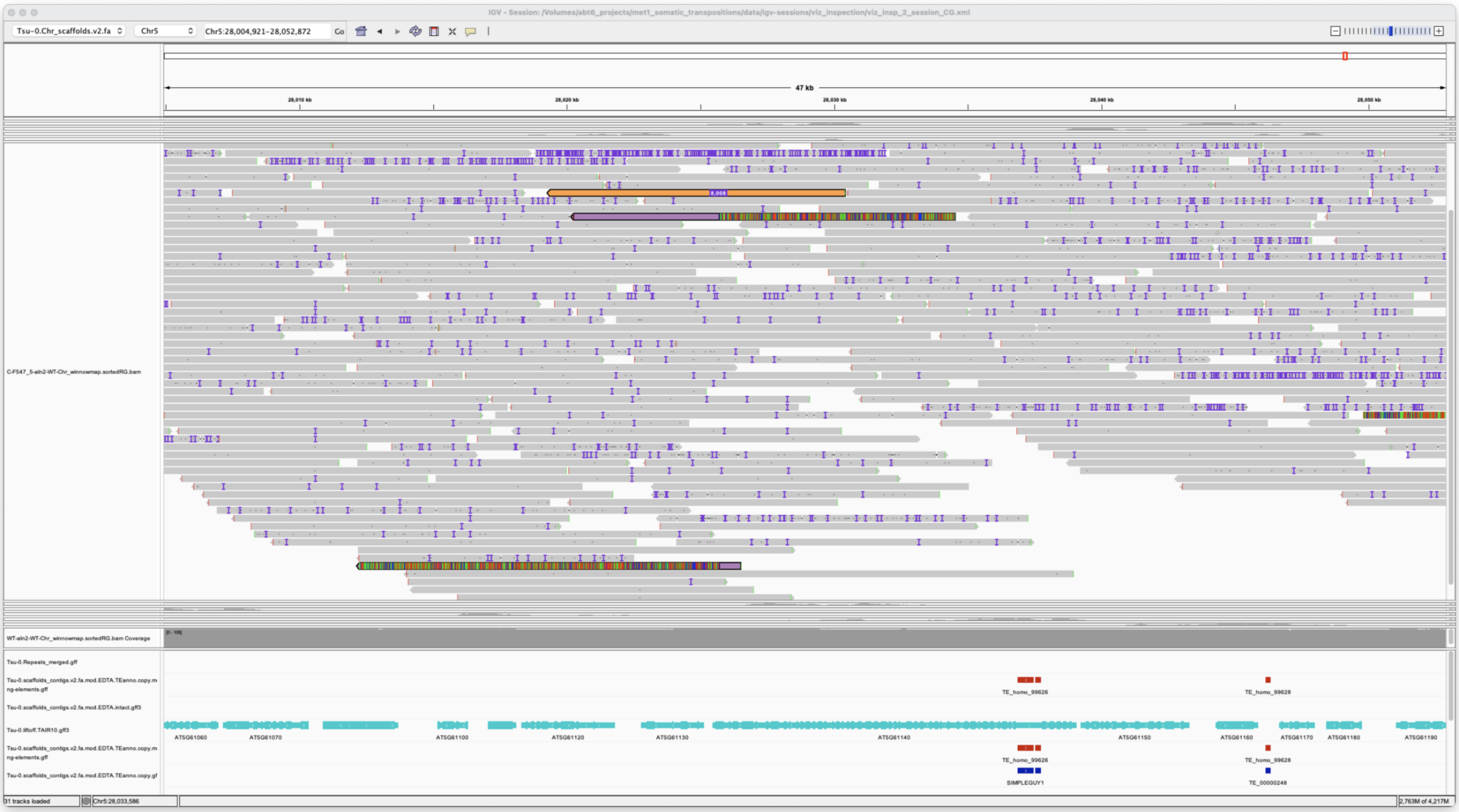

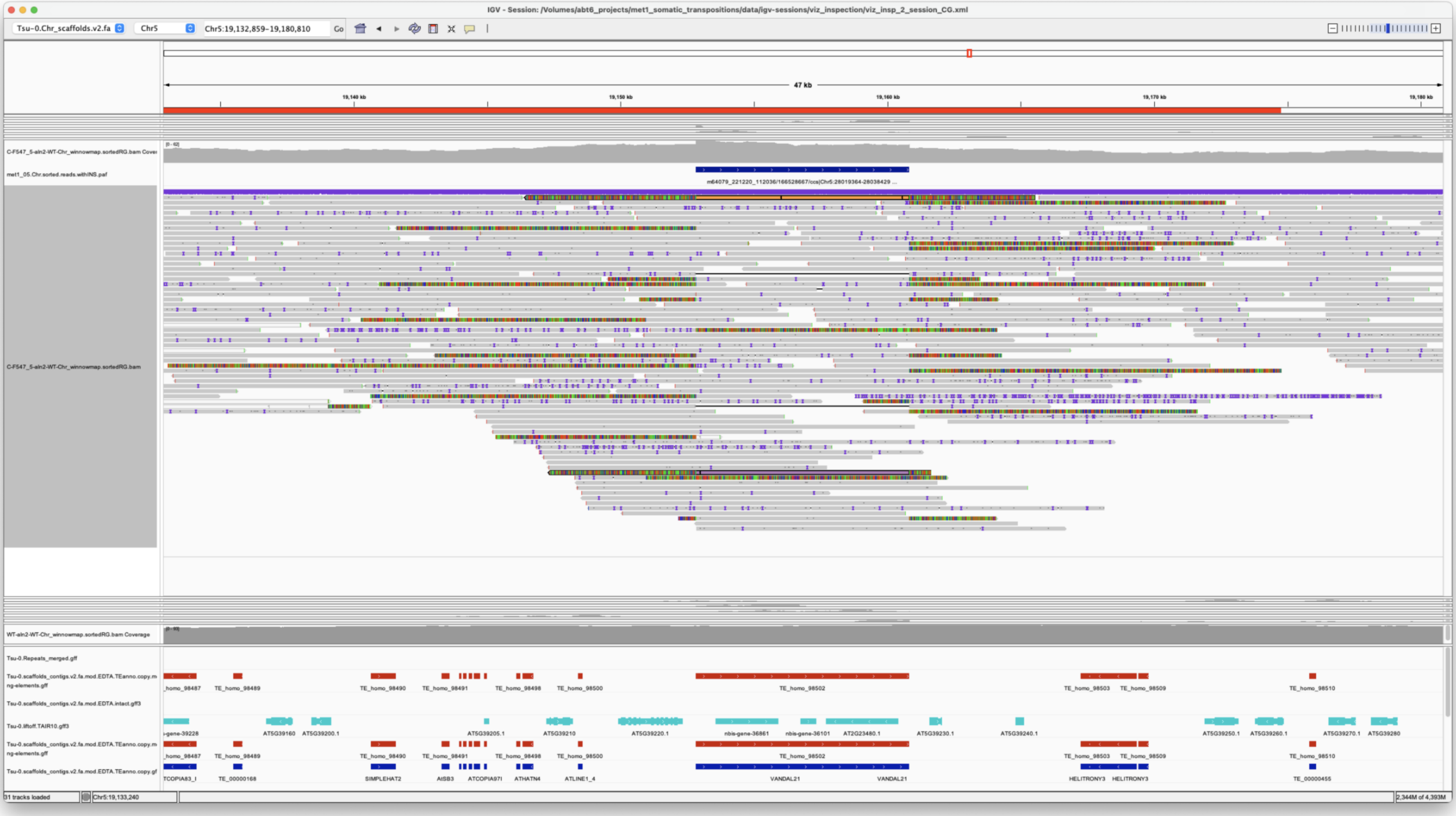

**2X coverage for insertion**

*Confirmed*

---

> Chr5 29702875 29702875 m64079_221220_112036/141754517/ccs Chr1 11941106 11946436 Chr1|11941106|11946435|ID=LTRRT_5;Name=ATCOPIA93.2_Evade;Classification=LTR/Copia;Sequence_ontology=SO:0002264;ltr_identity=1.0000;Method=structural;motif=TACA;tsd=ATATG met1_05

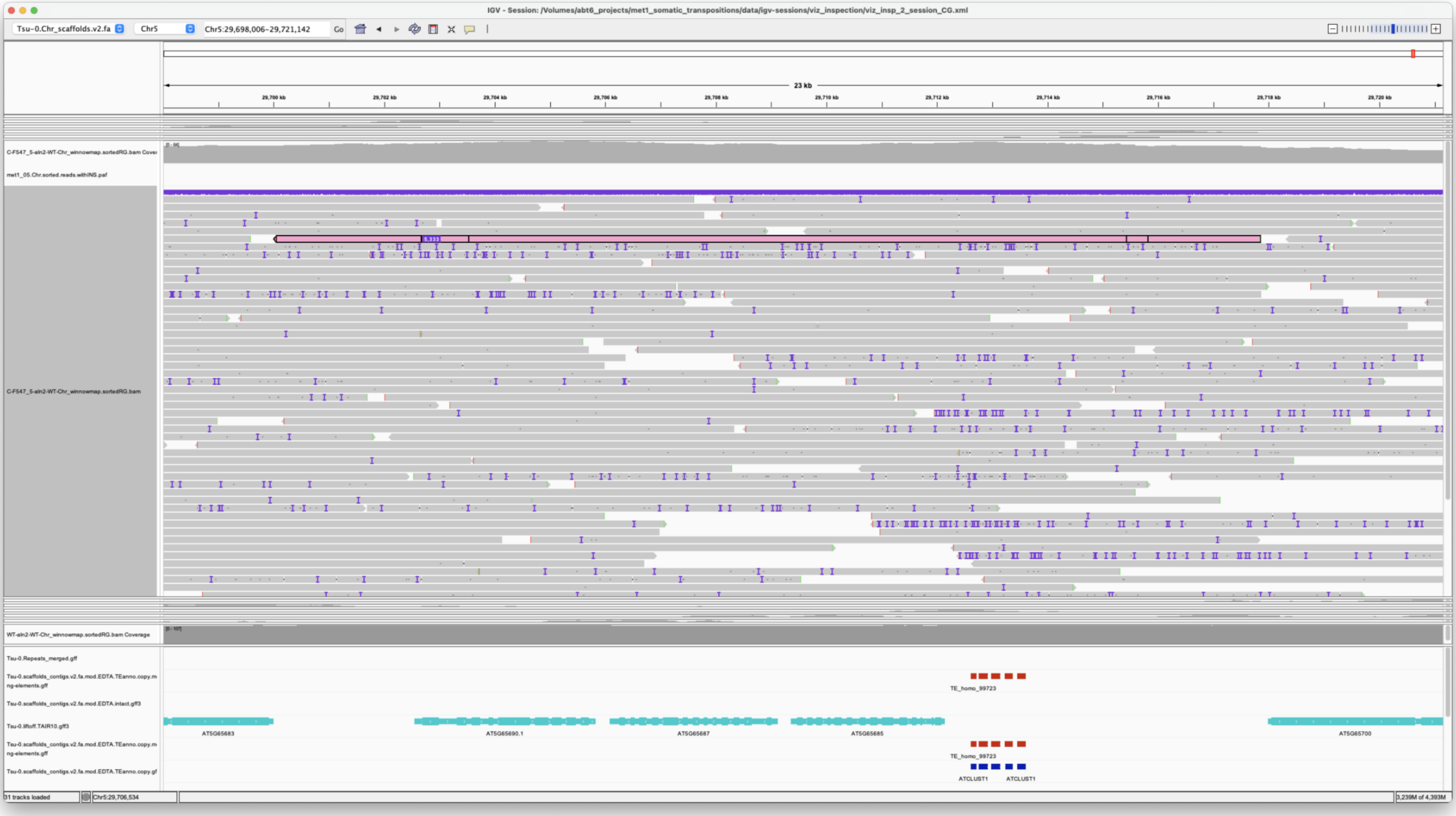


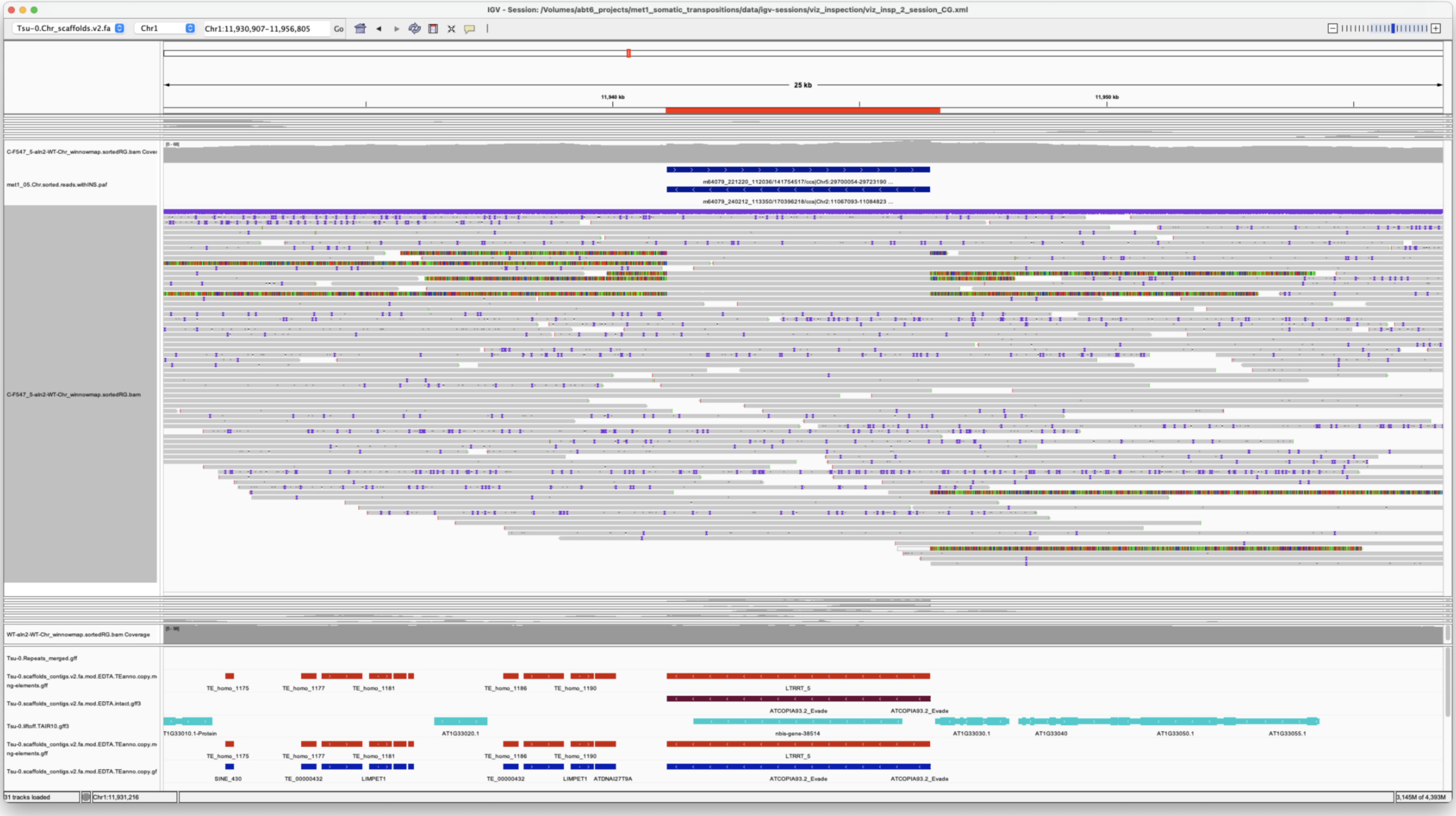


*Confirmed*

---

---

## met1_06

---

Chr1 17891752 17891752 m64079_221220_112036/12321302/ccs Chr5 19152826 19160834 Chr5|19152829|19160825|ID=TE_homo_95640;Name=VANDAL21;classification=DNA/Mutator;sequence_ontology=SO:0002280;identity=0.976;method=homology;ID=TE_homo_98501;sequence_ontology=SO:0002280|ID=TE_homo_95641;Name=VANDAL21;classification=DNA/Mutator;sequence_ontology=SO:0002280;identity=0.966;method=homology;ID=TE_homo_98502;sequence_ontology=SO:0002280 met1_06

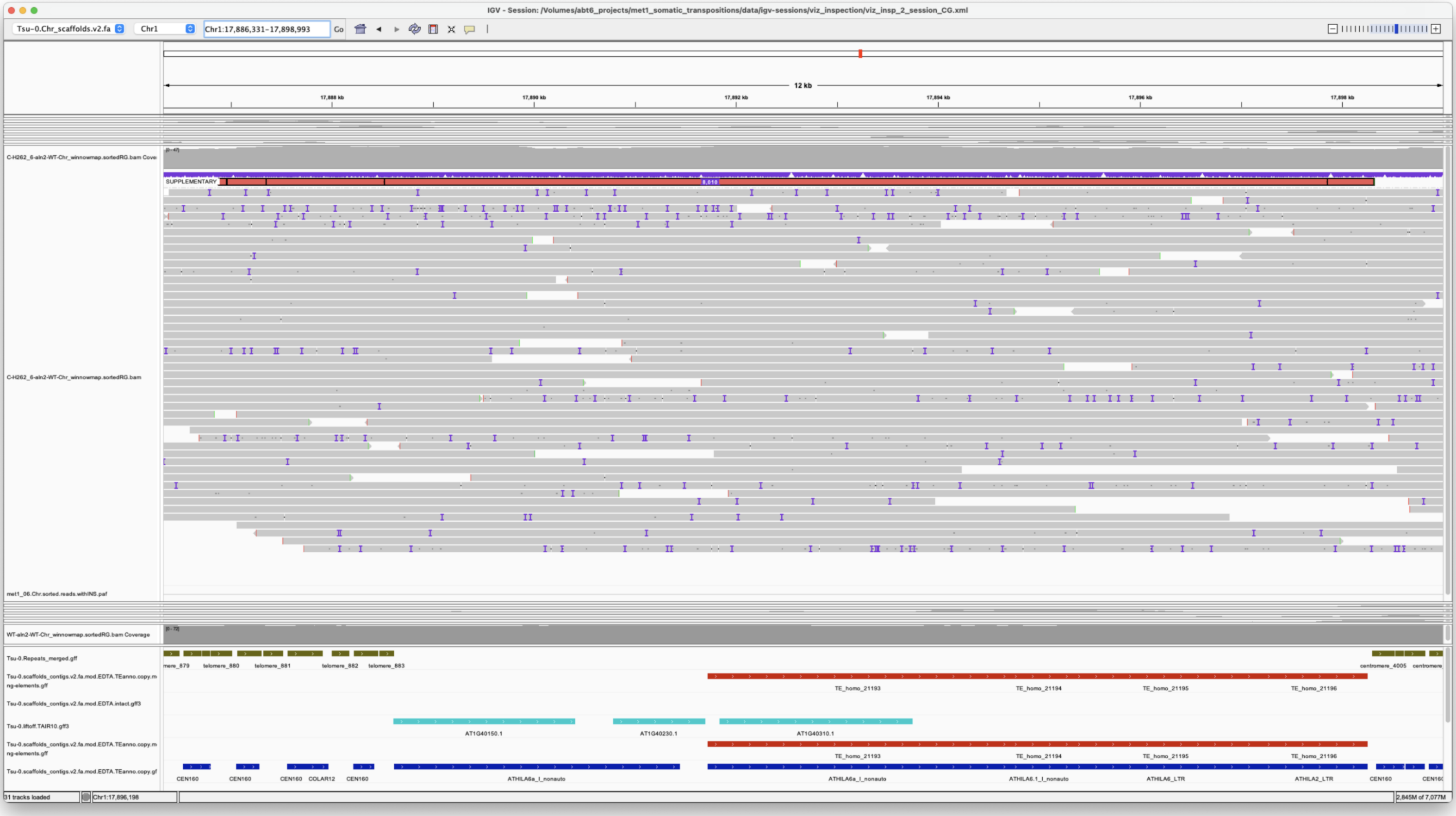


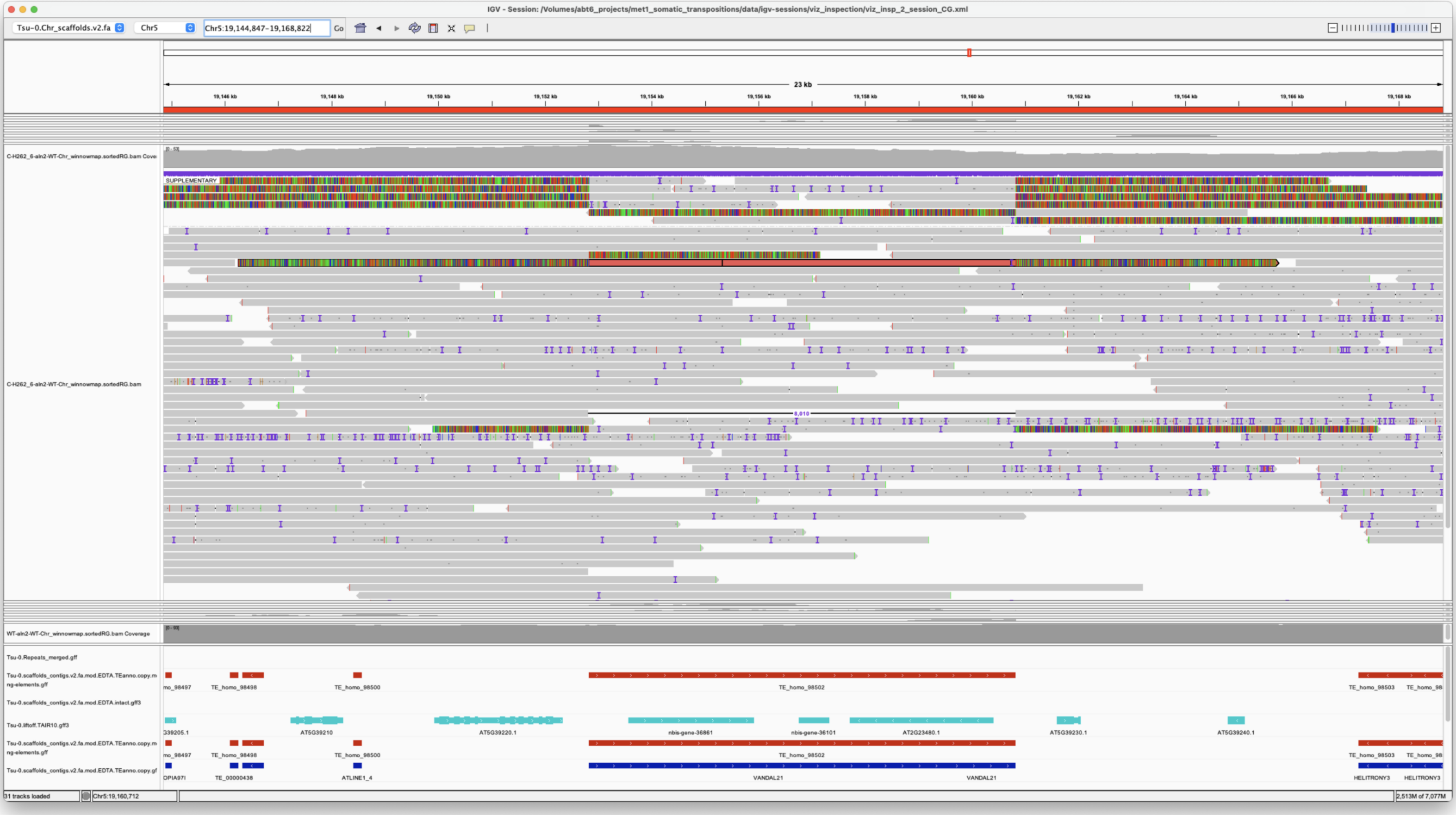


*Confirmed*

> Chr1 31010416 31010416 m64079_221220_112036/97584208/ccs Chr5 875412 876434 Chr5|875414|876433|ID=TE_MANUAL_02;Name=PAC;classification=DNA/DTC;sequence_ontology=MANUAL;identity=MANUAL;method=MANUAL;ID=TE_MANUAL_02;sequence_ontology=MANUAL met1_06

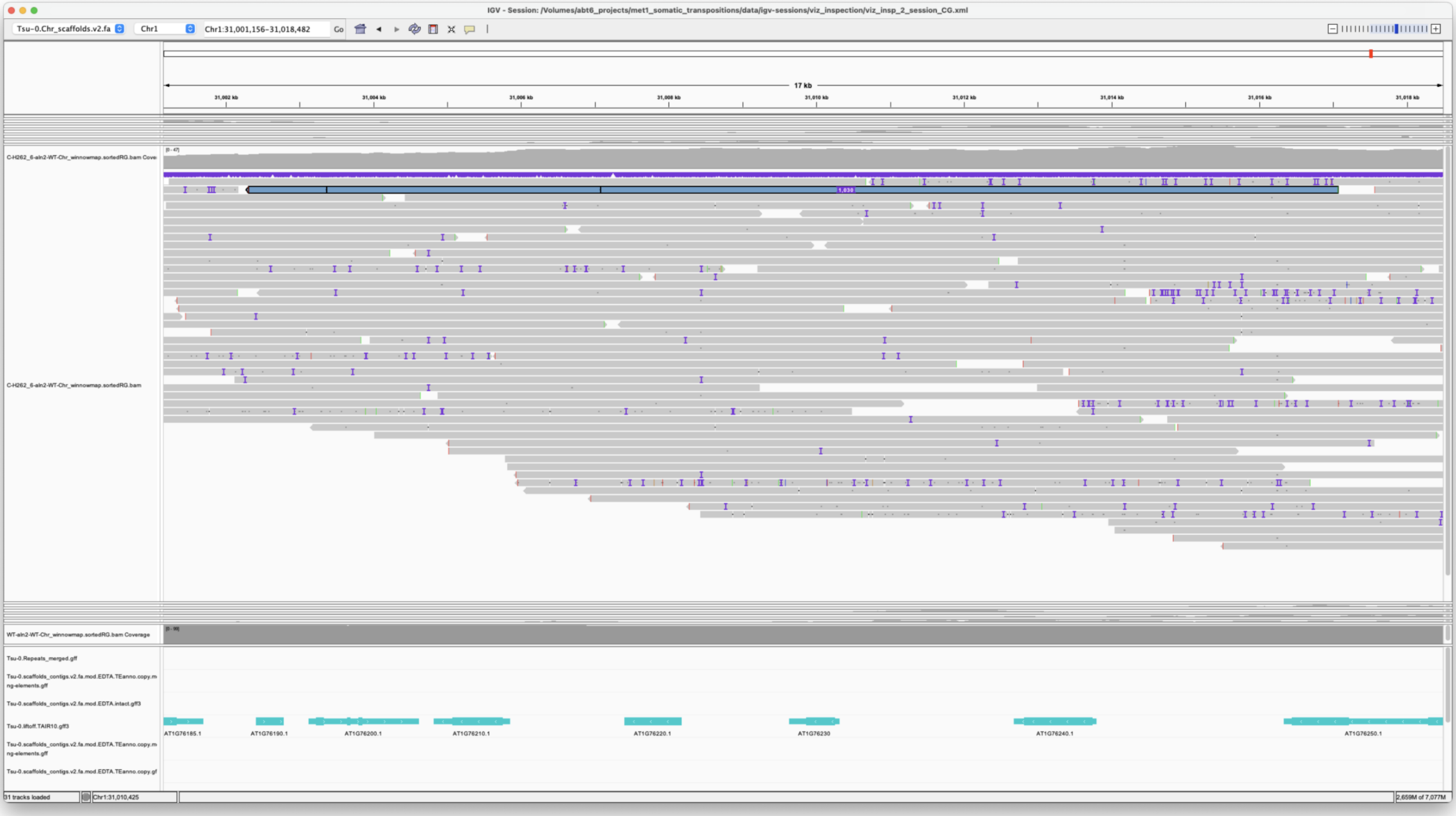


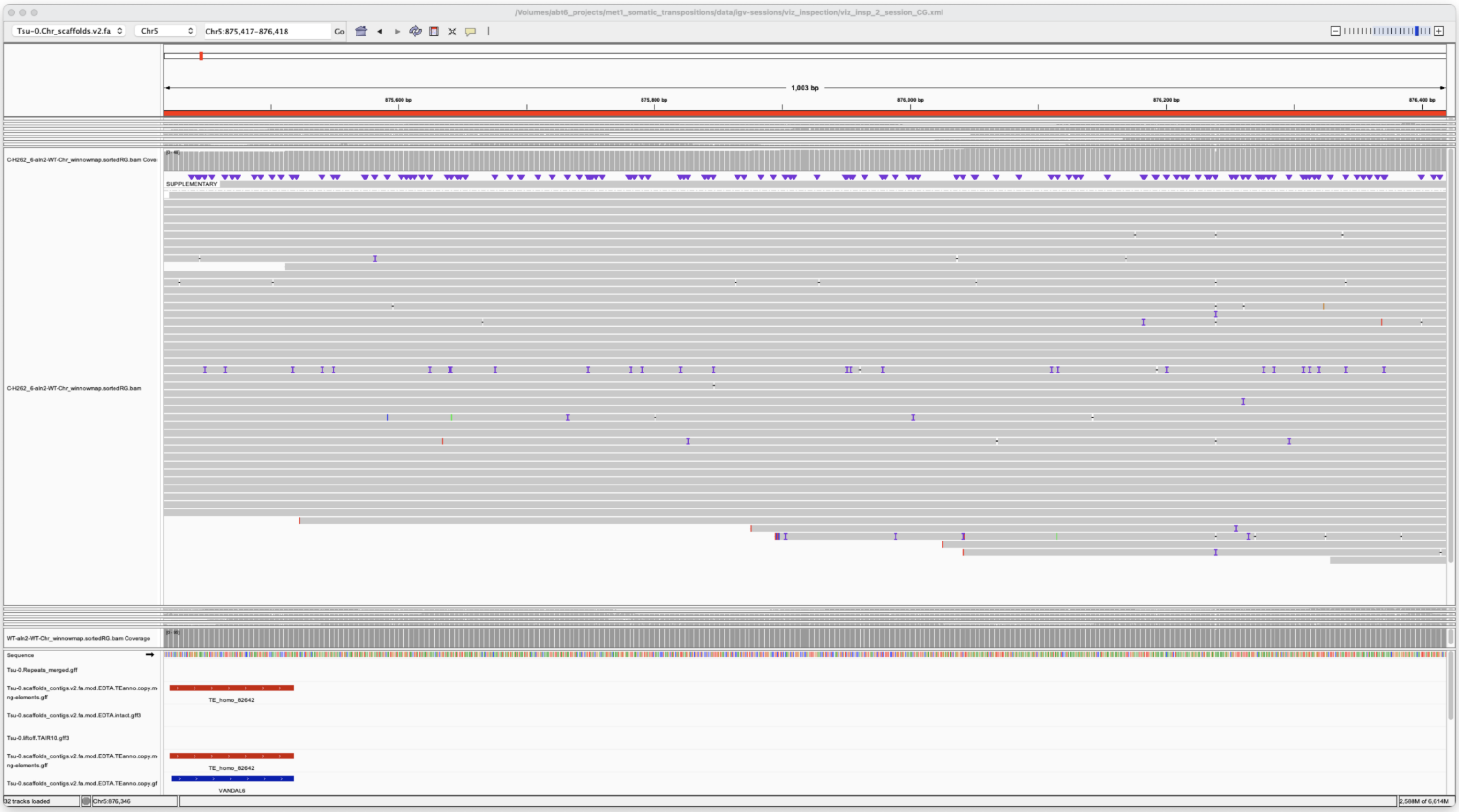


*Confirmed*

Chr2 2187852 2187852 m64079_221220_112036/39389477/ccs Chr1 11941106 11946435
Chr1|11941106|11946435|ID=LTRRT_5;Name=ATCOPIA93.2_Evade;Classification=LTR/Copia;Sequence_ontology=SO:0002264;ltr_identity=1.0000;Method=structural;motif=TACA;tsd=ATATG met1_06

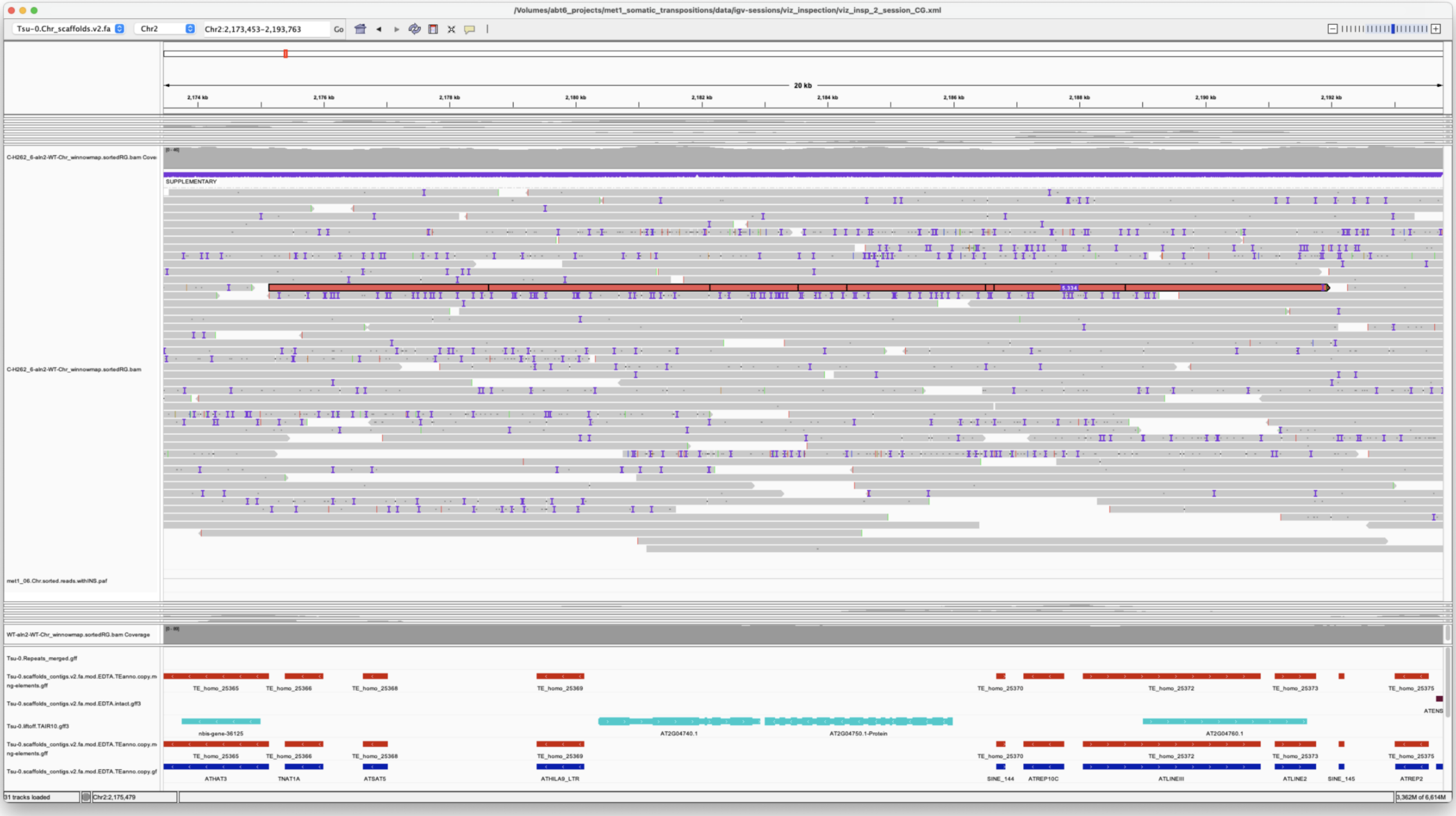


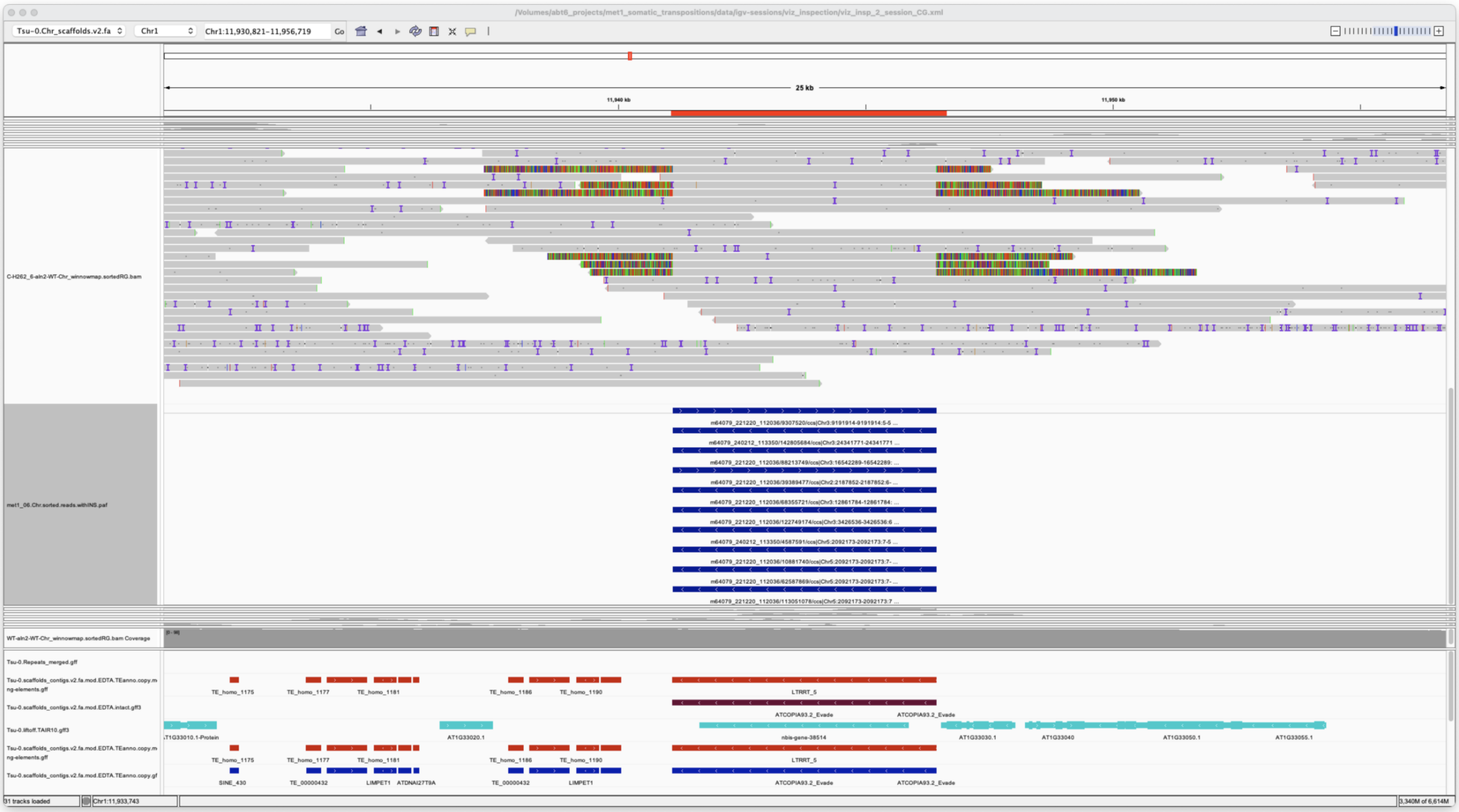


*Confirmed*

> Chr3 3426536 3426536 m64079_221220_112036/122749174/ccs Chr1 11941107 11946436
> Chr1|11941106|11946435|ID=LTRRT_5;Name=ATCOPIA93.2_Evade;Classification=LTR/Copia;Sequence_ontology=SO:0002264;ltr_identity=1.0000;Method=structural;motif=TACA;tsd=ATATG met1_06

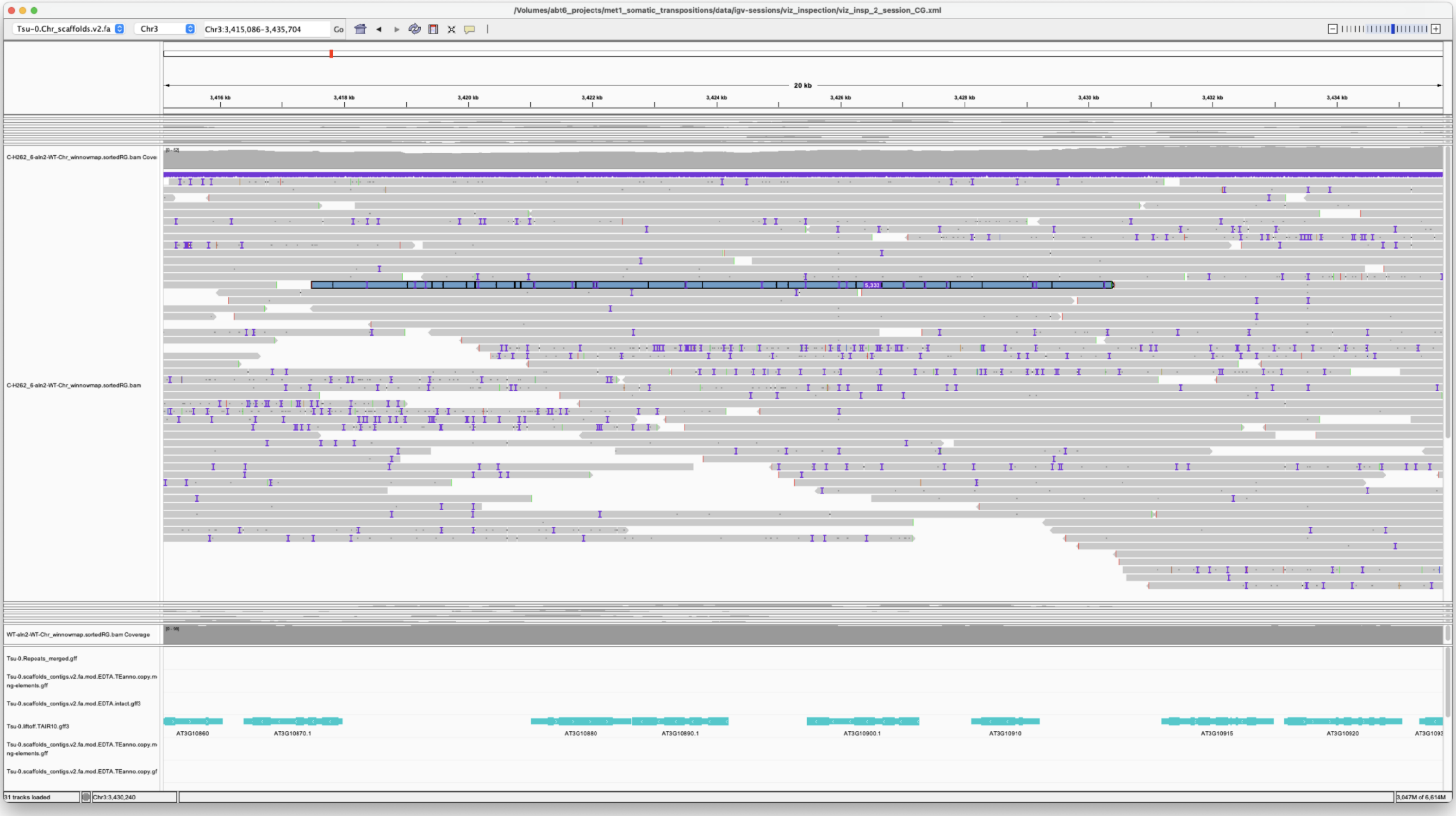


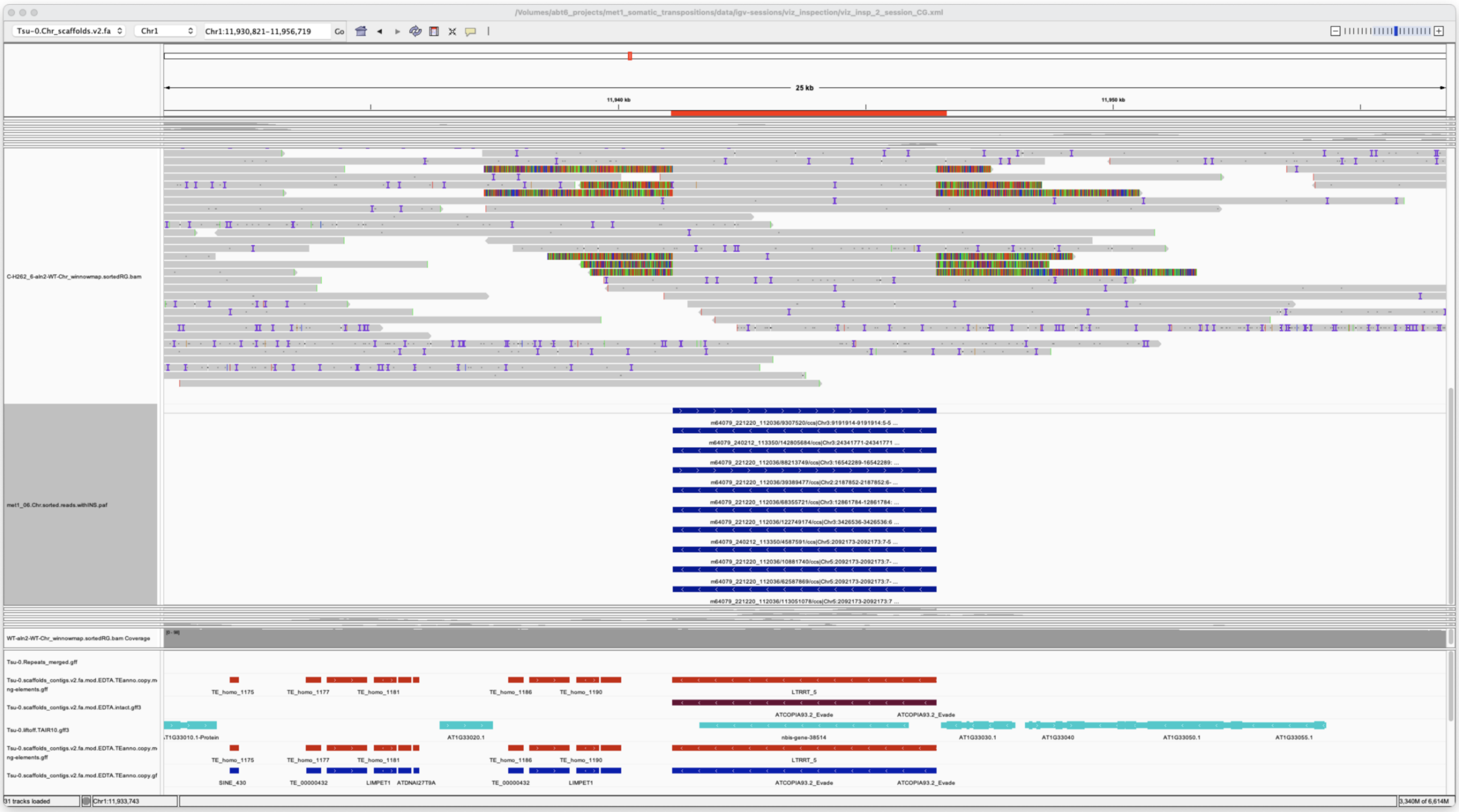


*Confirmed*

Chr3 9191914 9191914 m64079_221220_112036/9307520/ccs Chr1 11941106 11946436
Chr1|11941106|11946435|ID=LTRRT_5;Name=ATCOPIA93.2_Evade;Classification=LTR/Copia;Sequence_ontology=SO:0002264;ltr_identity=1.0000;Method=structural;motif=TACA;tsd=ATATG met1_06

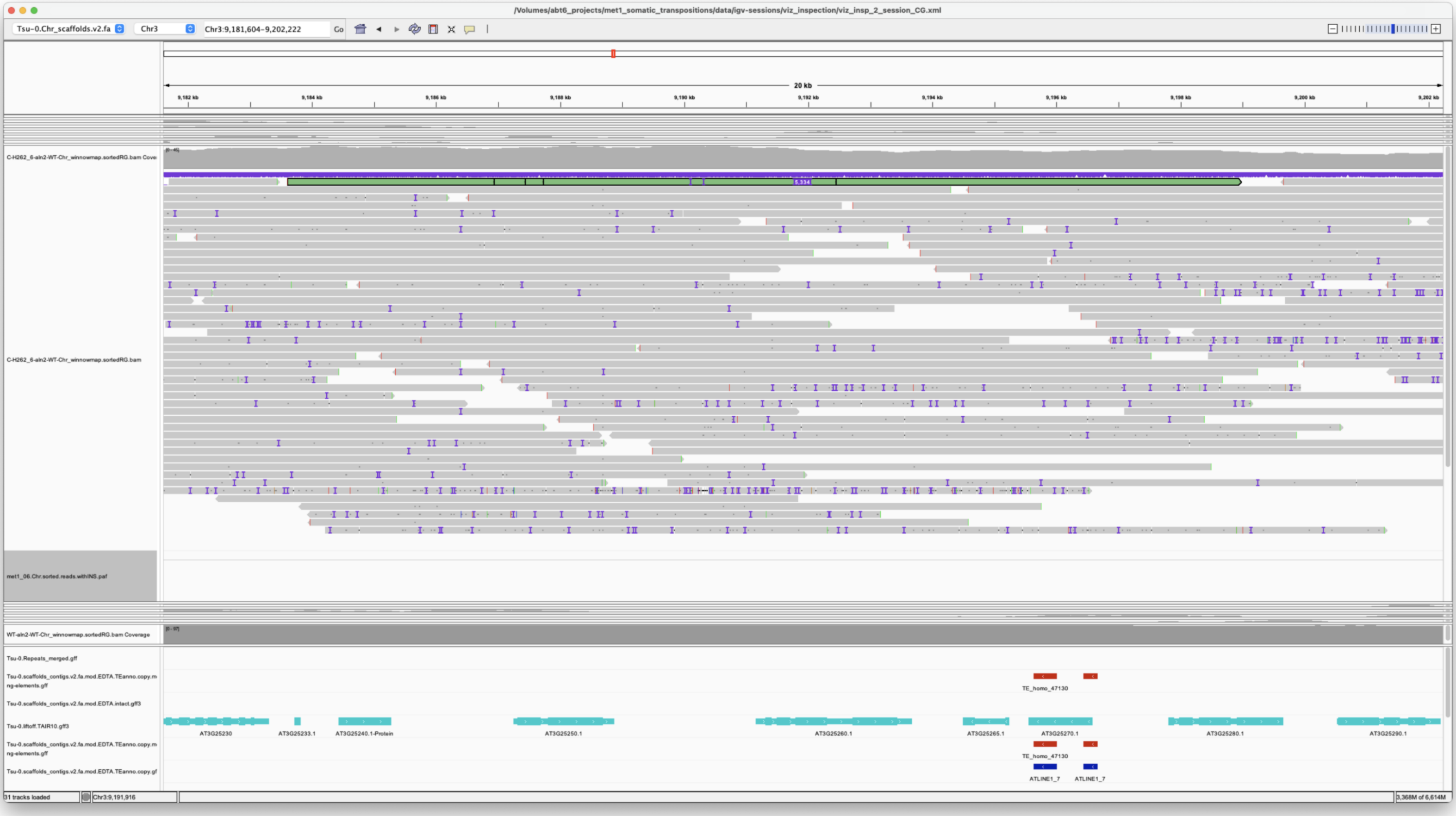


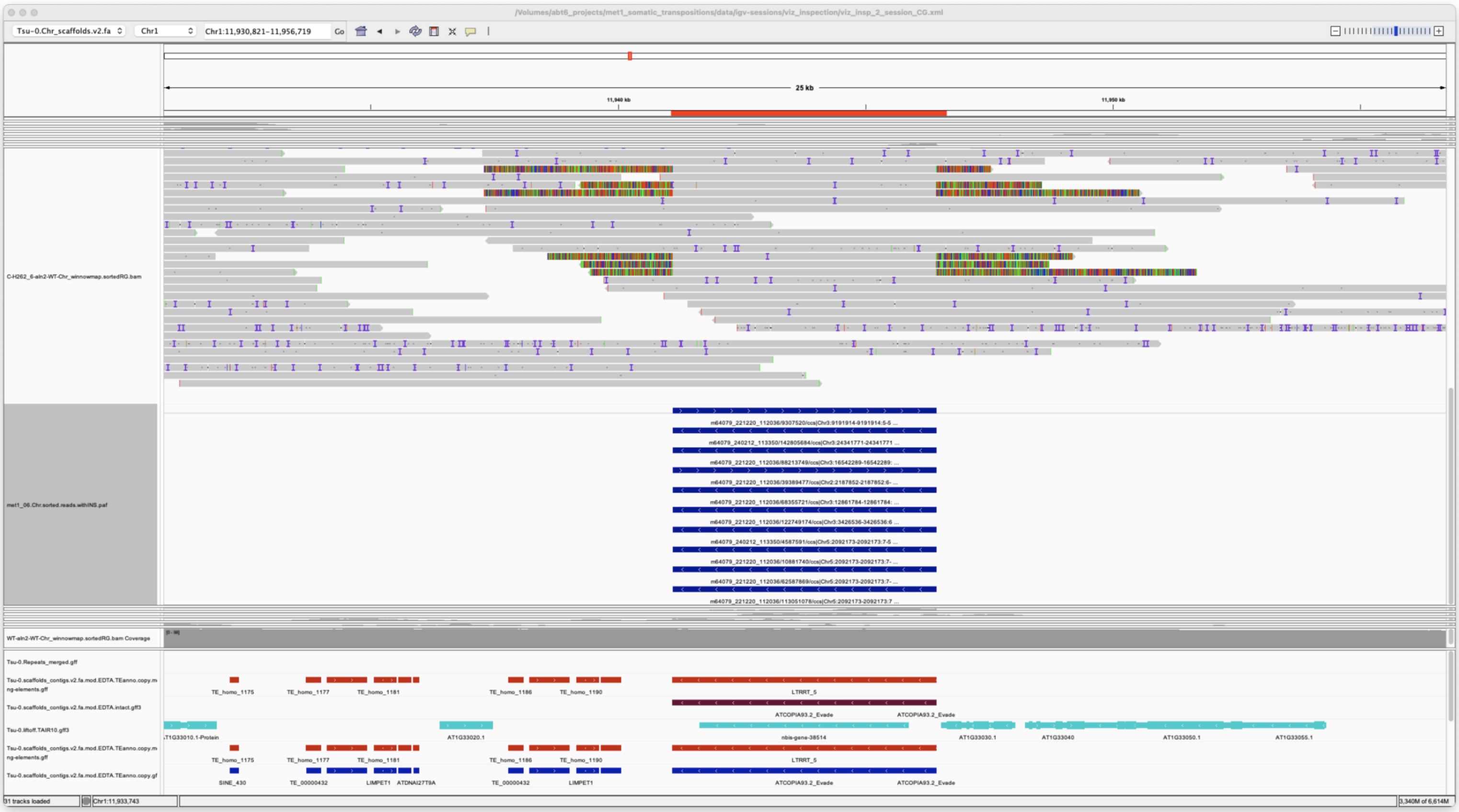


*Confirmed*

---

> Chr3 12861784 12861784 m64079_221220_112036/68355721/ccs Chr1 11941107 11946437
> Chr1|11941106|11946435|ID=LTRRT_5;Name=ATCOPIA93.2_Evade;Classification=LTR/Copia;Sequence_ontology=SO:0002264;ltr_identity=1.0000;Method=structural;motif=TACA;tsd=ATATG met1_06

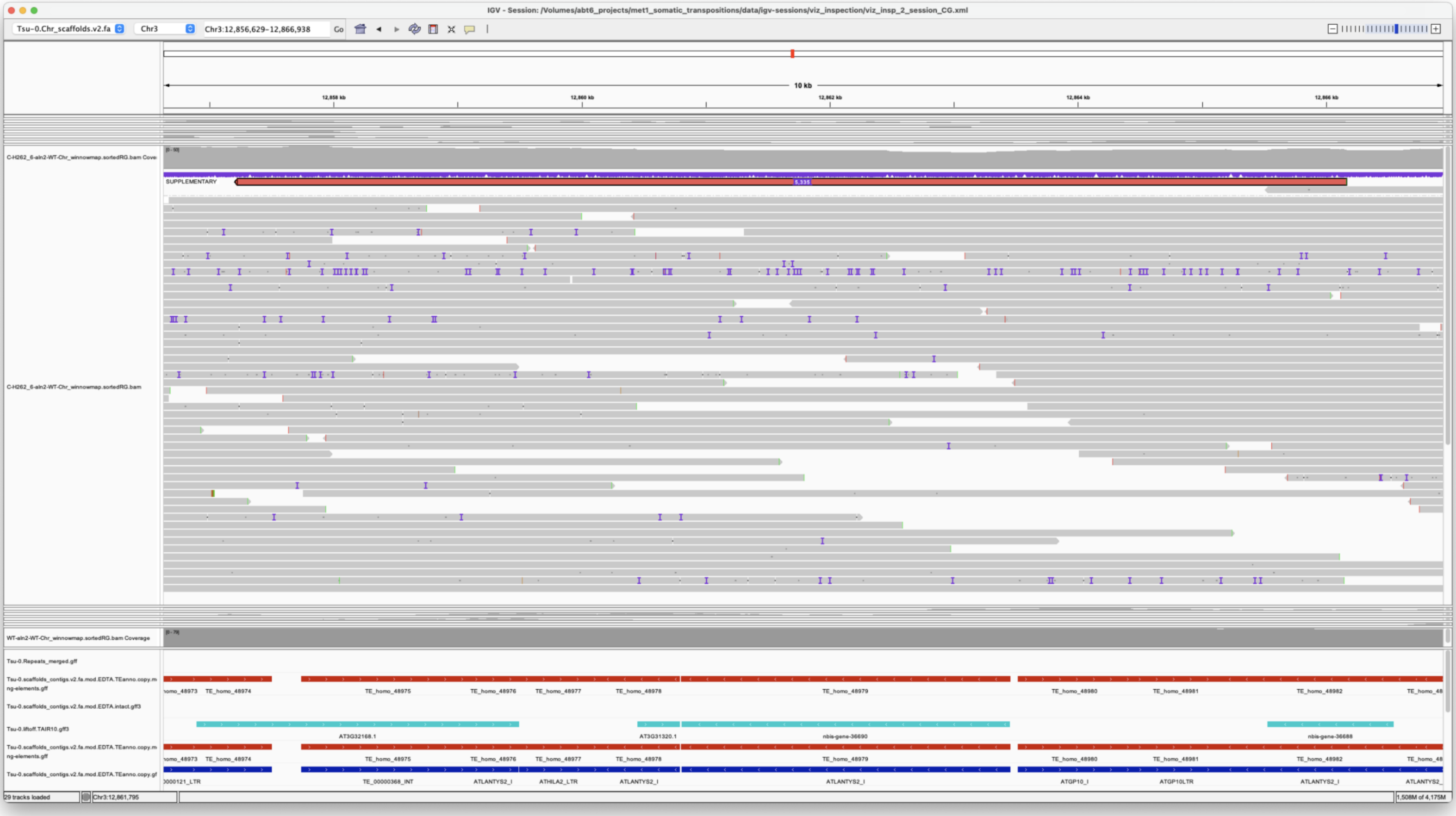


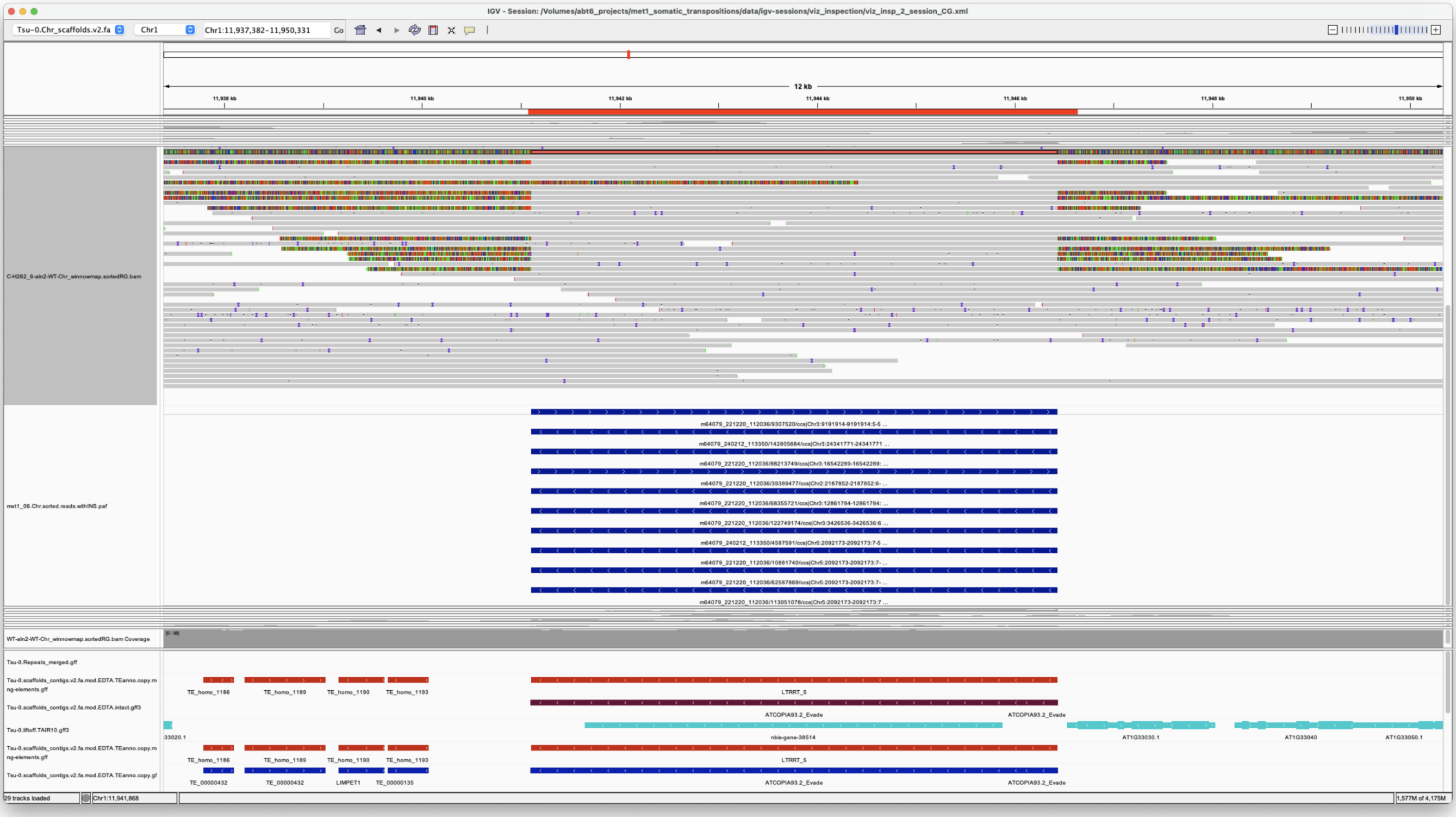


*Confirmed*

---

> Chr3 16542289 16542289 m64079_221220_112036/88213749/ccs Chr1 11941106 11946436
> Chr1|11941106|11946435|ID=LTRRT_5;Name=ATCOPIA93.2_Evade;Classification=LTR/Copia;Sequence_ontology=SO:0002264;ltr_identity=1.0000;Method=structural;motif=TACA;tsd=ATATG met1_06

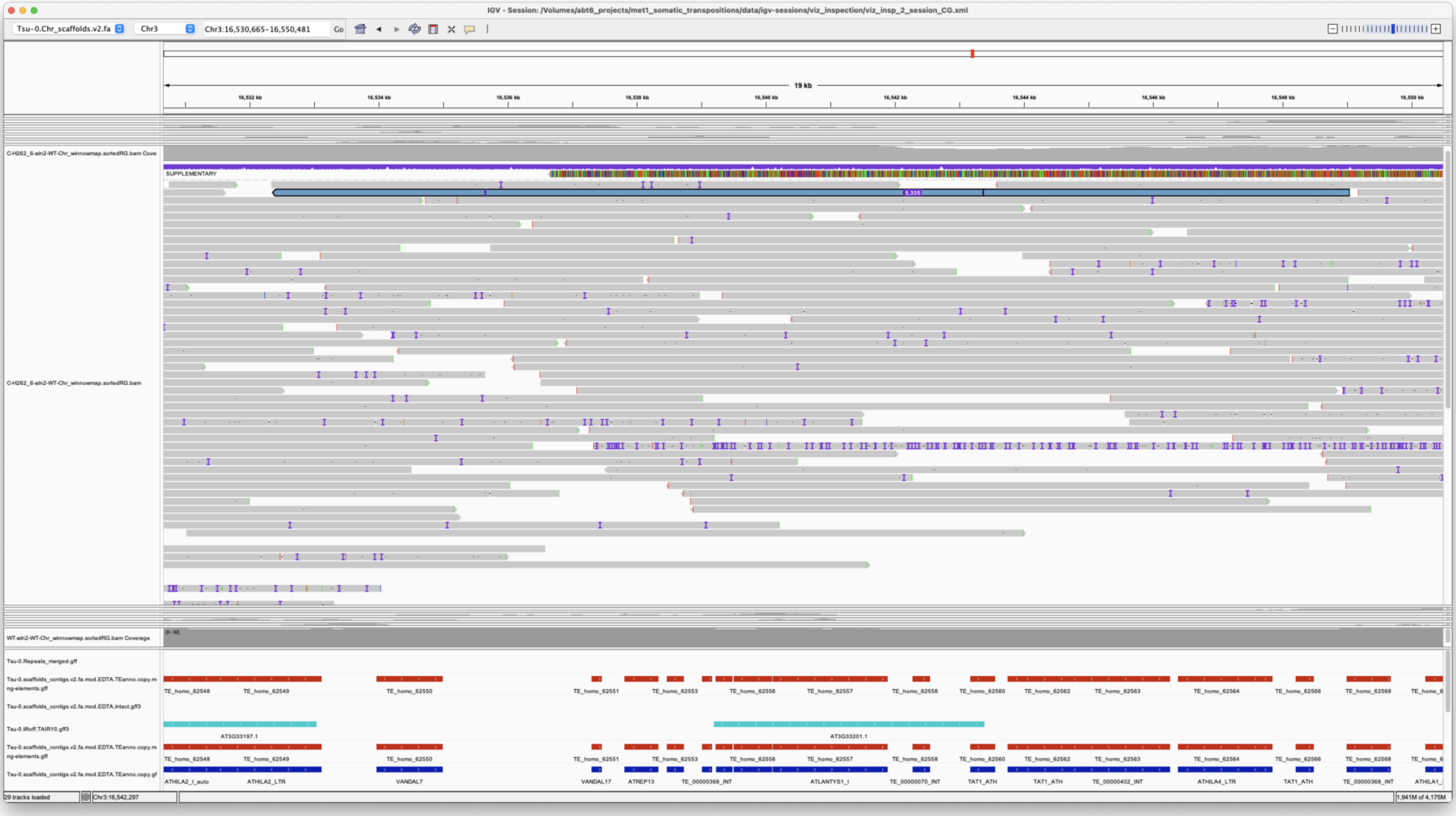


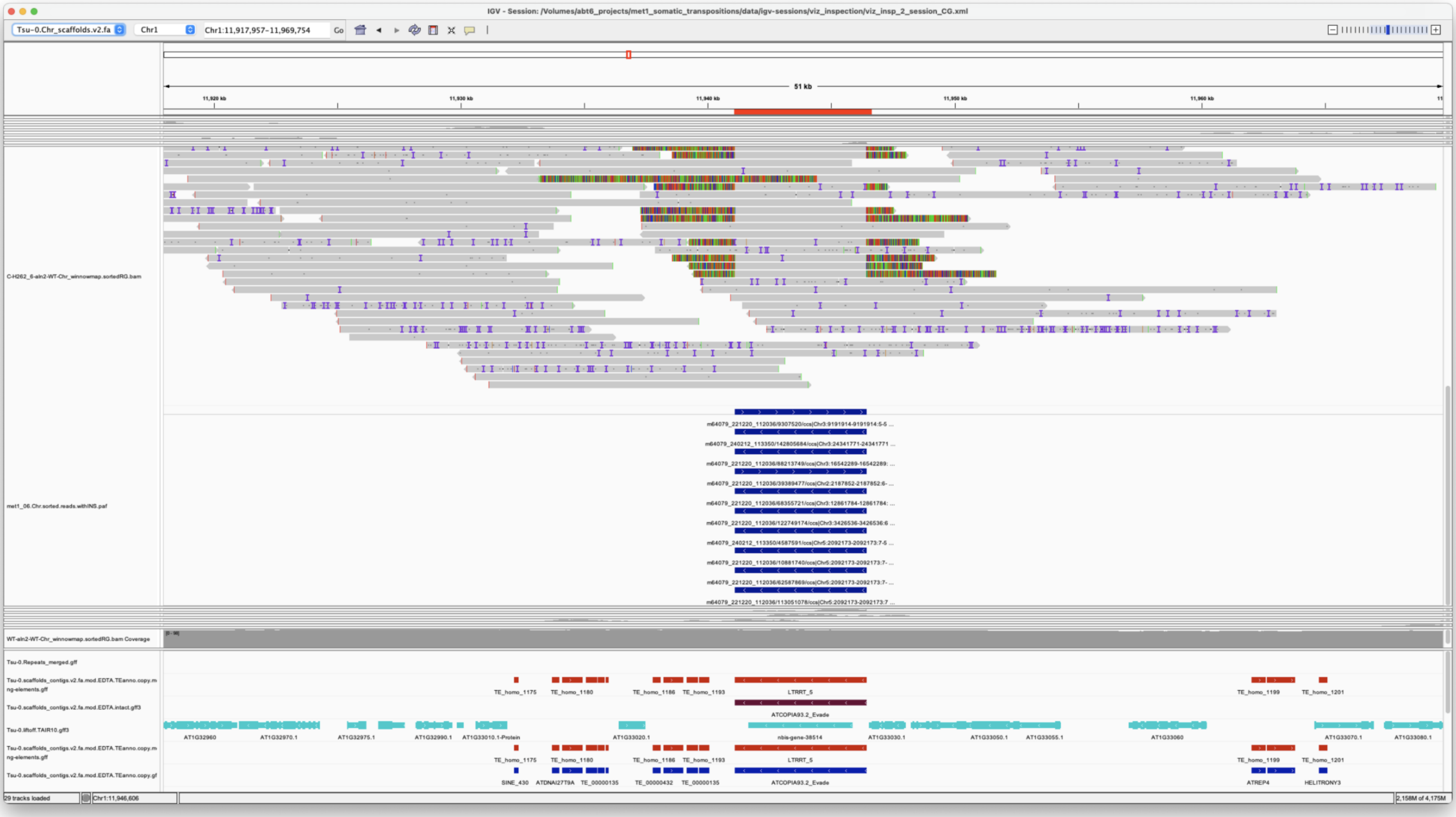


*Confirmed*

---

> Chr3 24341771 24341771 m64079_240212_113350/142805684/ccs Chr1 11941106 11946436
> Chr1|11941106|11946435|ID=LTRRT_5;Name=ATCOPIA93.2_Evade;Classification=LTR/Copia;Sequence_ontology=SO:0002264;ltr_identity=1.0000;Method=structural;motif=TACA;tsd=ATATG met1_06

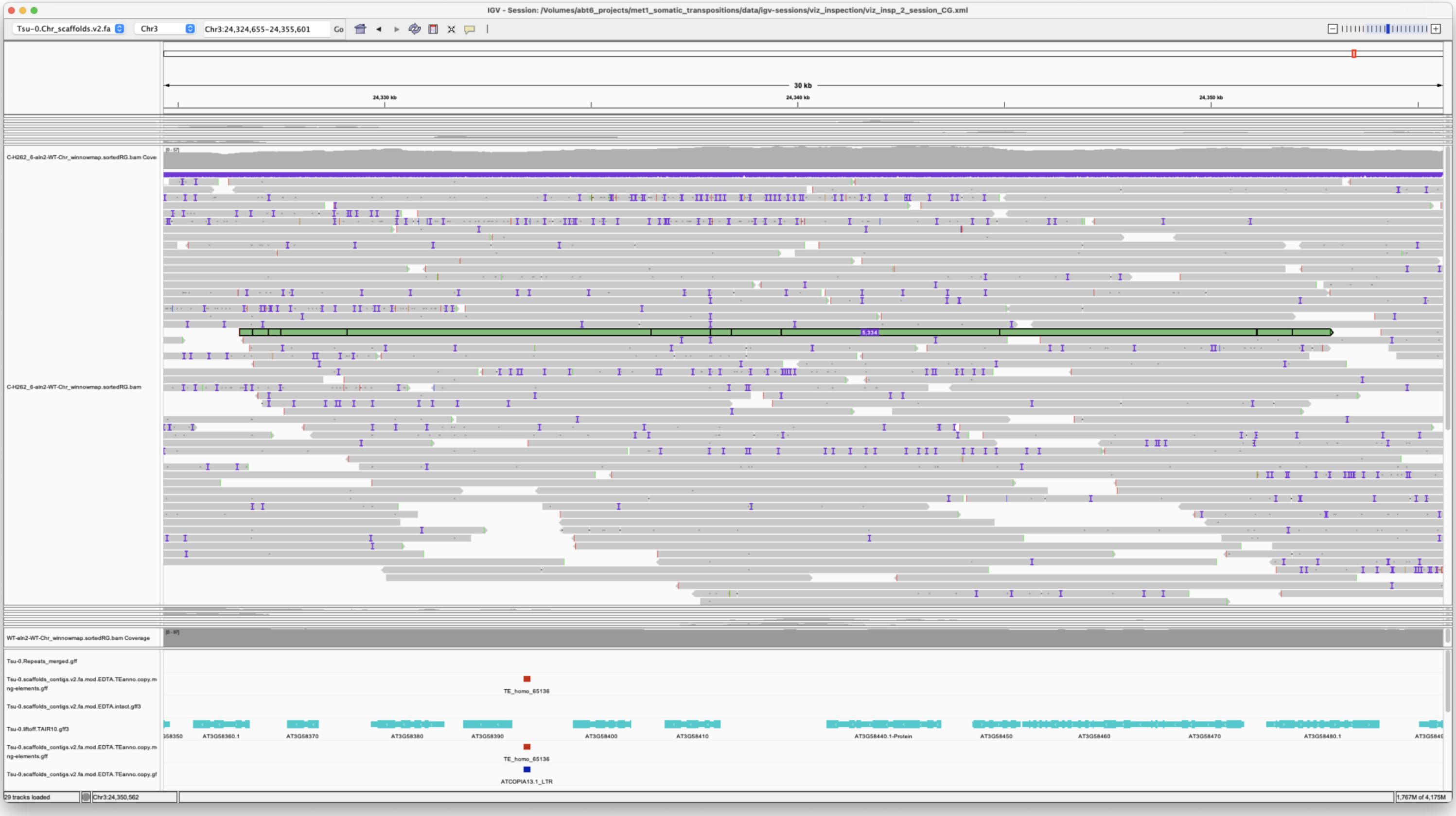


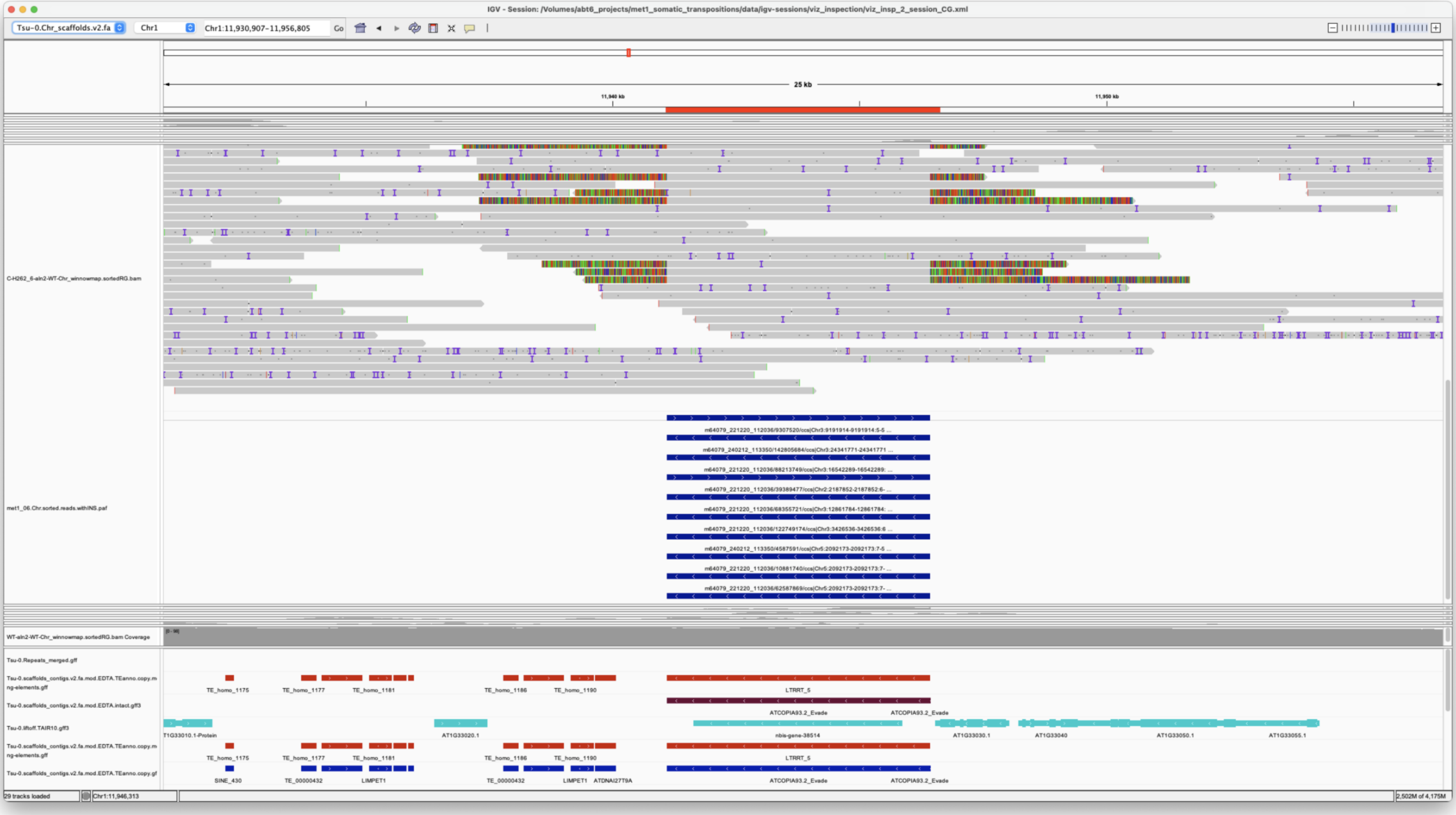


*Confirmed*

---

Chr4 6070355 6070355 m64079_221220_112036/121309221/ccs Chr4 6067864 6070355 .|-1|-2|. met1_06

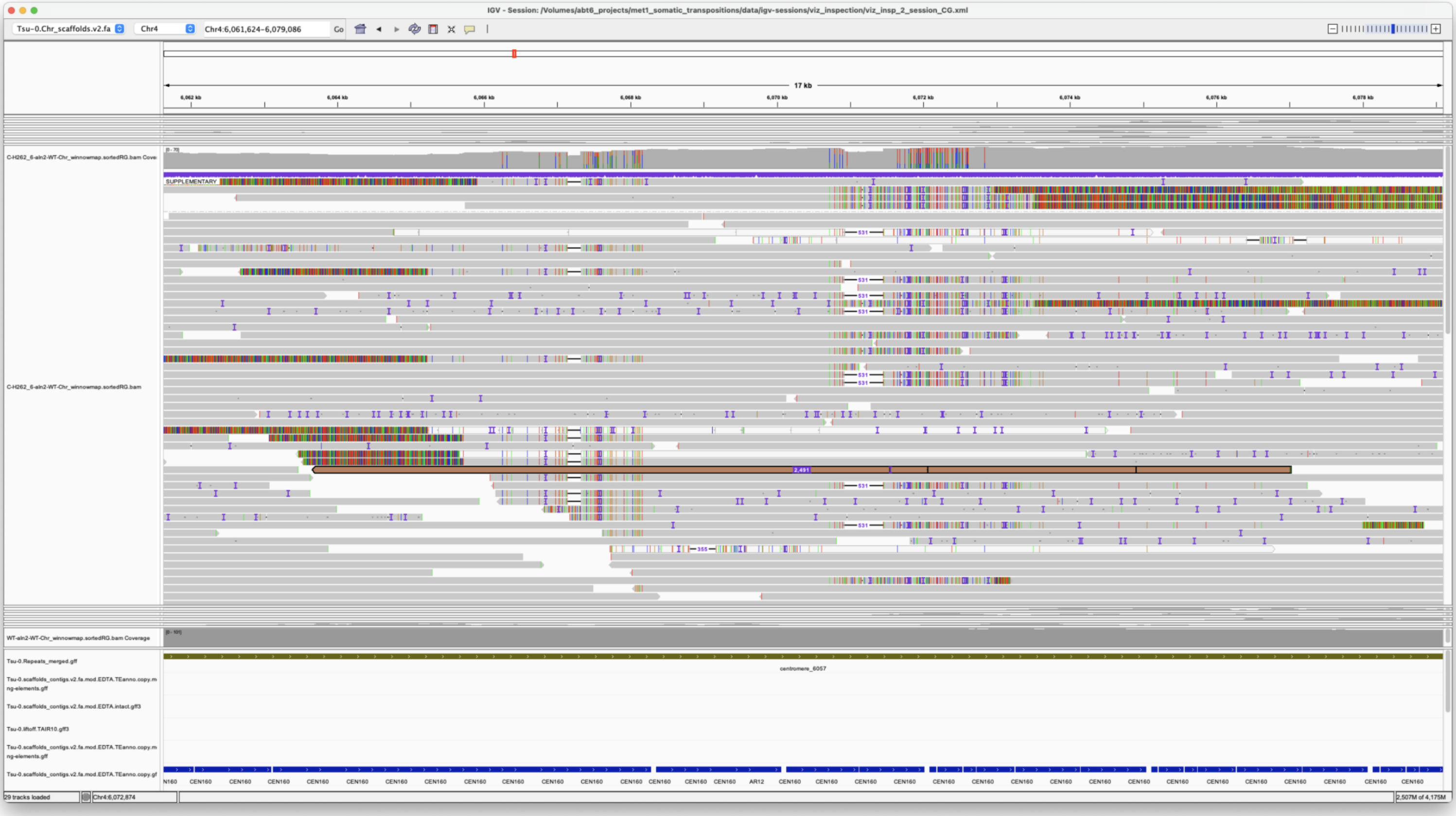


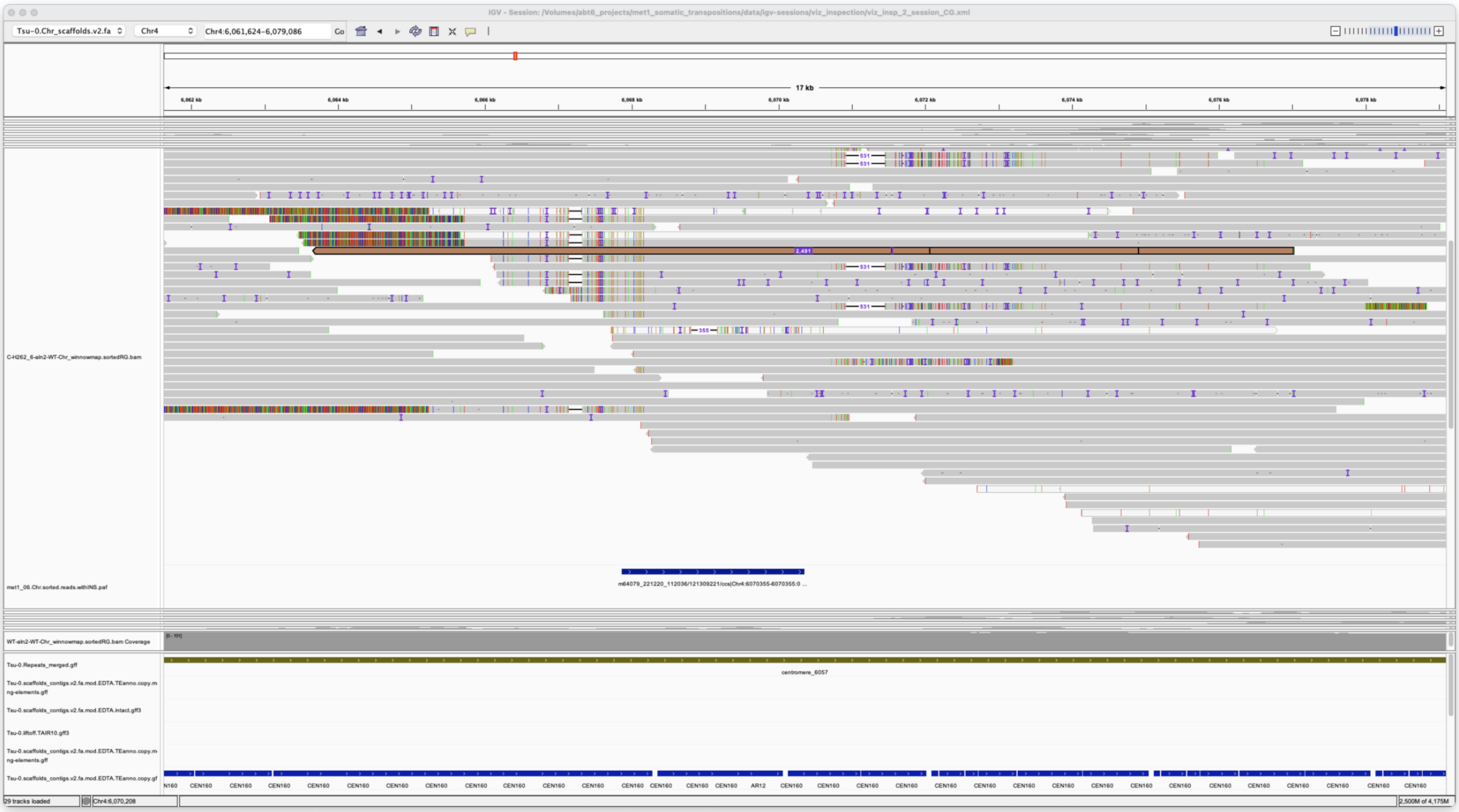


Centromeric rearrangement

*unsupported*

---

> Chr4 12391953 12391953 m64079_240212_113350/99025857/ccs Chr5 875413 876434
> Chr5|875414|876433|ID=TE_MANUAL_02;Name=PAC;classification=DNA/DTC;sequence_ontology=MANUAL;identity=MANUAL;method=MANUAL;ID=TE_MANUAL_02;sequence_ontology=MANUAL met1_06

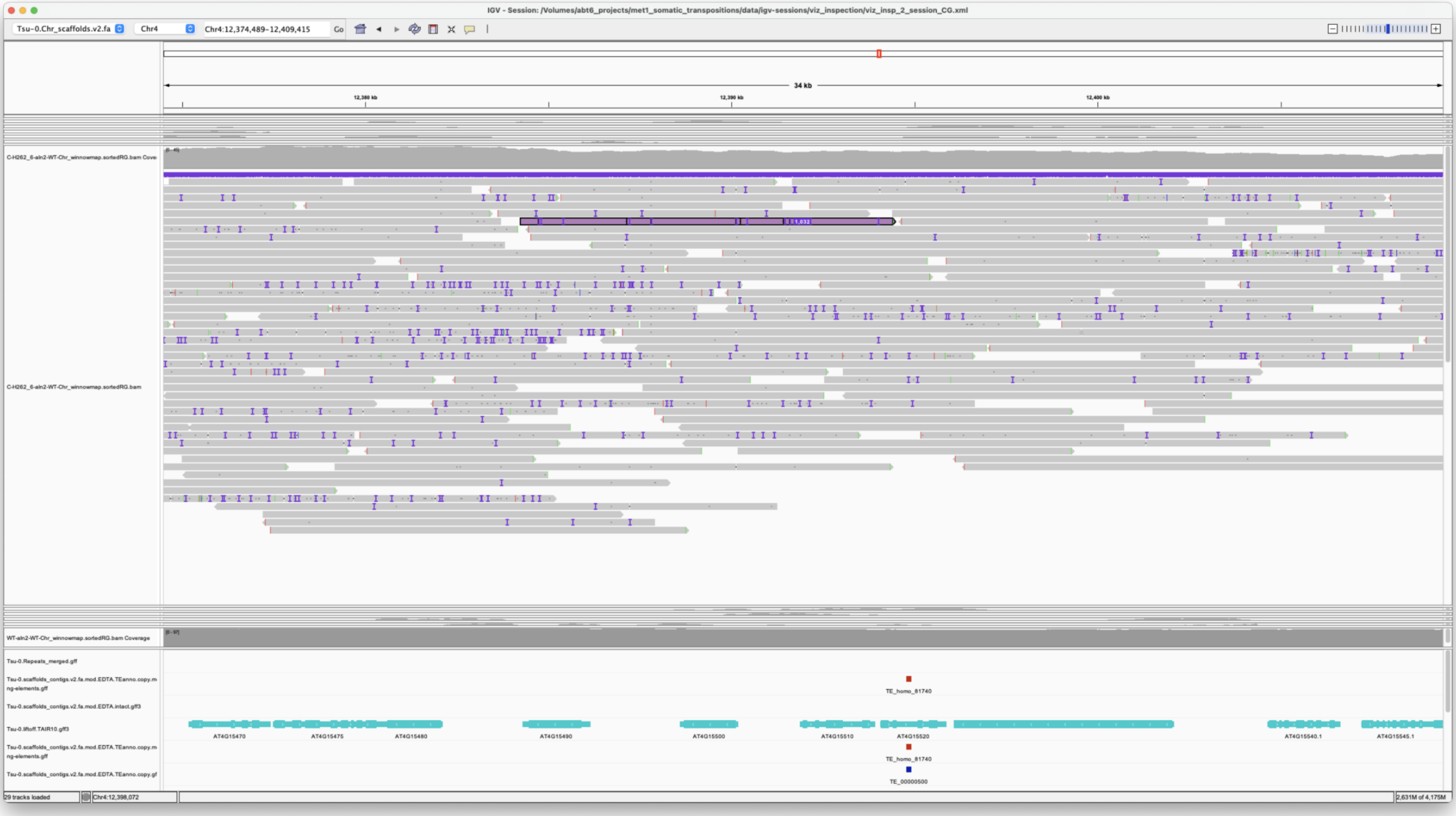


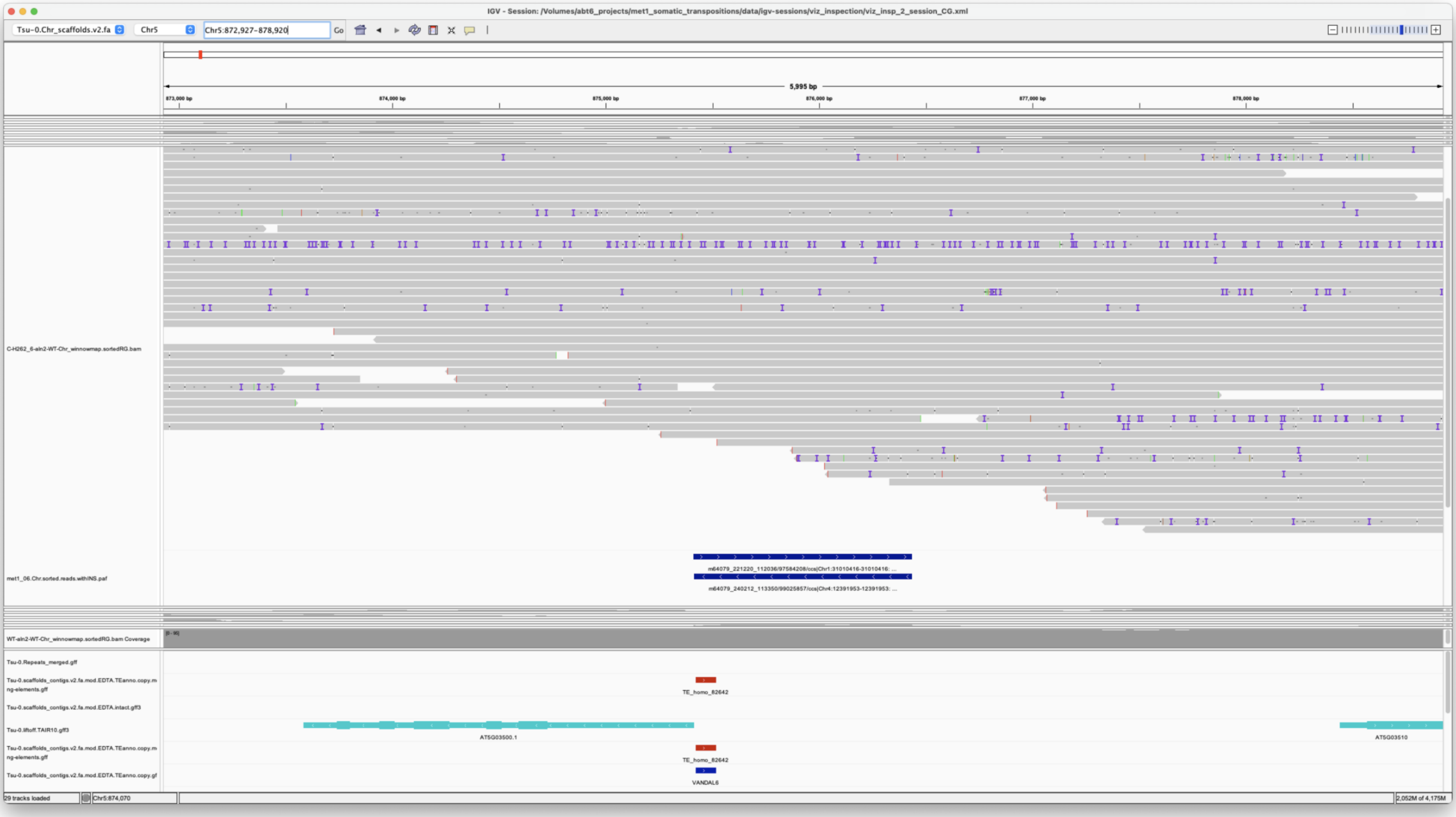


*Confirmed*

---

> Chr5 2092172 2092174 m64079_221220_112036/10881740/ccs**m64079_221220_112036/113051078/ccs**m64079_221220_112036/62587869/ccs**m64079_240212_113350/4587591/ccs Chr1 11941108 11946436 Chr1|11941106|11946435|ID=LTRRT_5;Name=ATCOPIA93.2_Evade;Classification=LTR/Copia;Sequence_ontology=SO:0002264;ltr_identity=1.0000;Method=structural;motif=TACA;tsd=ATATG**Chr1|11941106|11946435|ID=LTRRT_5;Name=ATCOPIA93.2_Evade;Classification=LTR/Copia;Sequence_ontology=SO:0002264;ltr_identity=1.0000;Method=structural;motif=TACA;tsd=ATATG**Chr1|11941106|11946435|ID=LTRRT_5;Name=ATCOPIA93.2_Evade;Classification=LTR/Copia;Sequence_ontology=SO:0002264;ltr_identity=1.0000;Method=structural;motif=TACA;tsd=ATATG**Chr1|11941106|11946435|ID=LTRRT_5;Name=ATCOPIA93.2_Evade;Classification=LTR/Copia;Sequence_ontology=SO:0002264;ltr_identity=1.0000;Method=structural;motif=TACA;tsd=ATATG met1_06

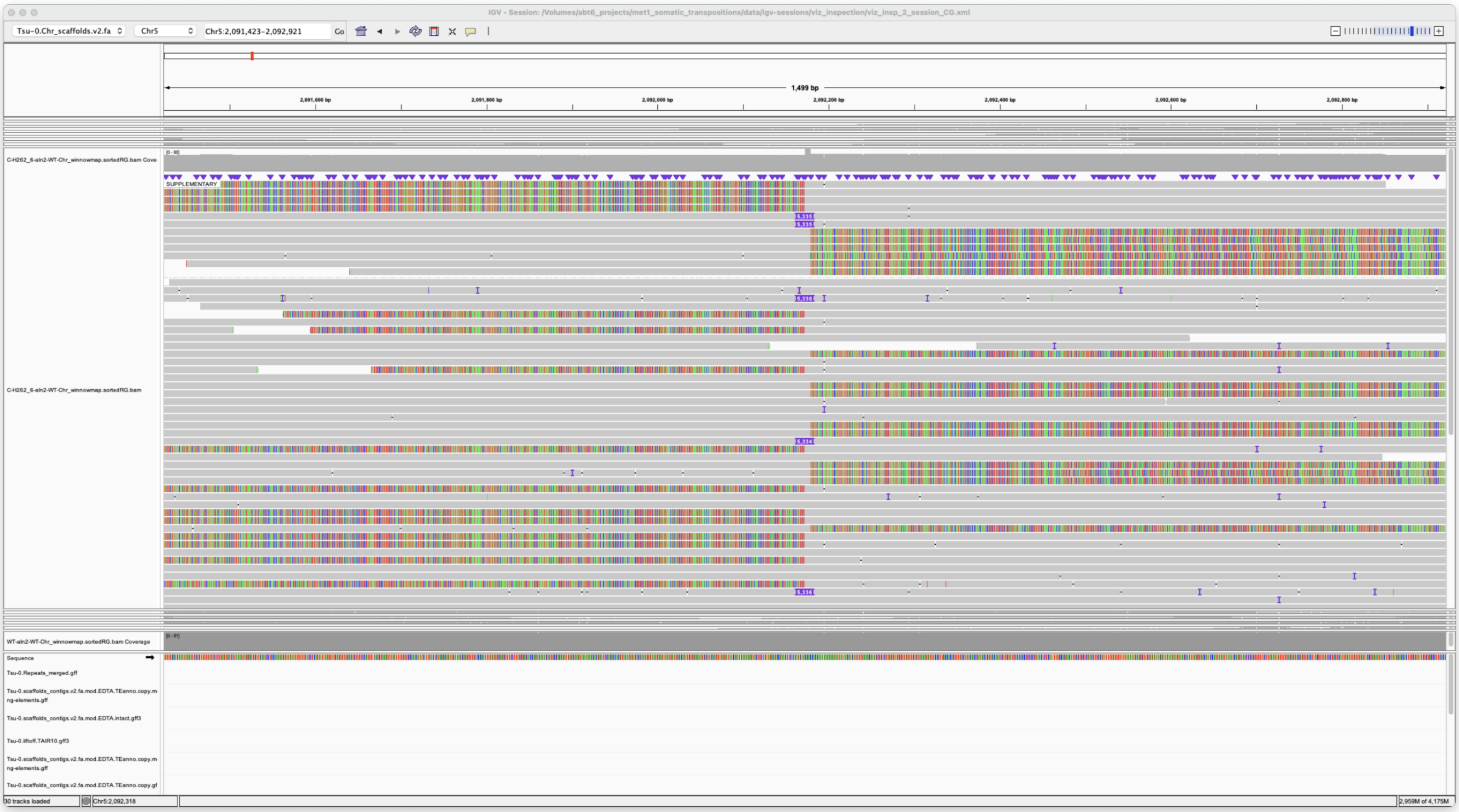

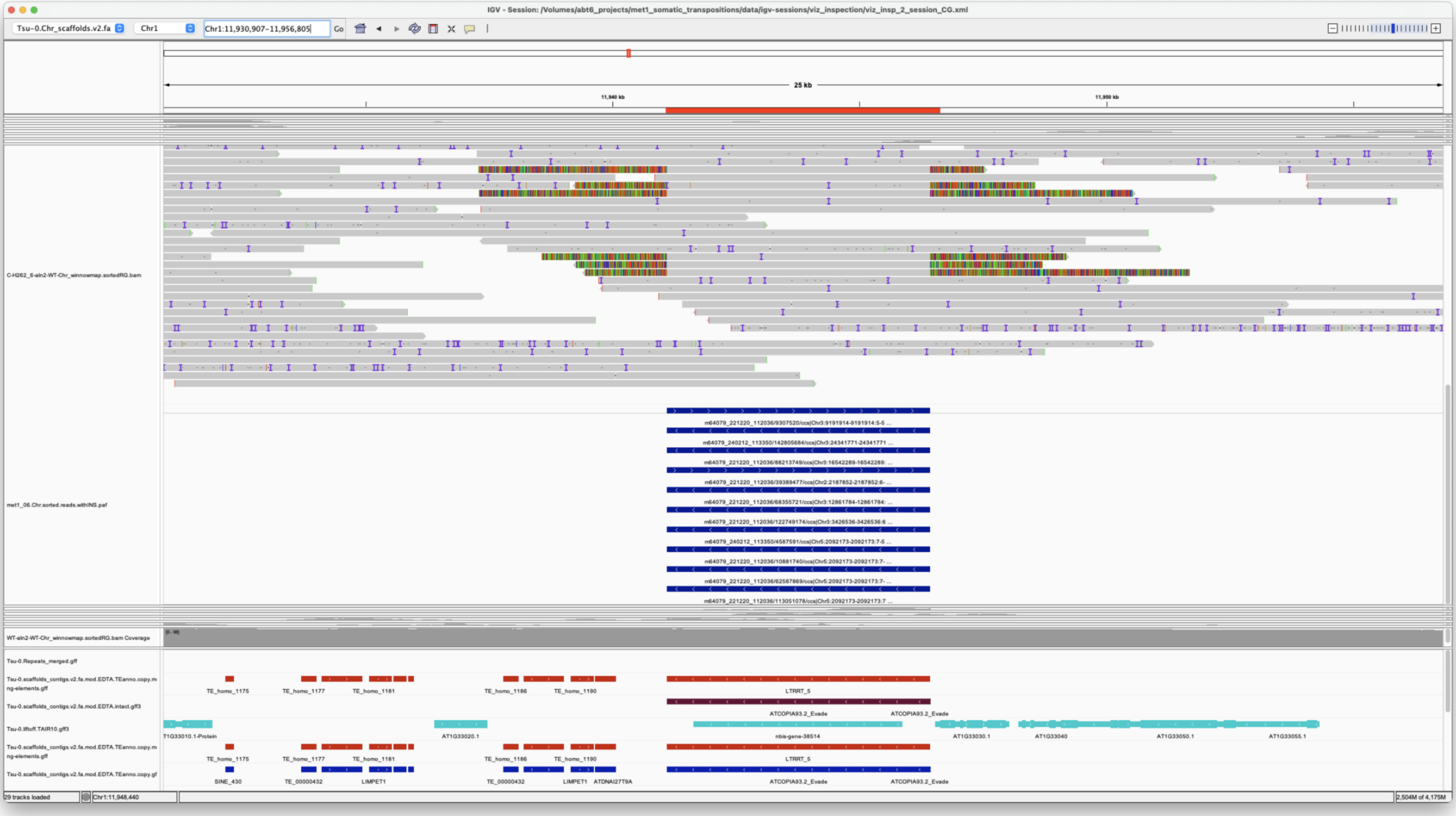

*Confirmed*

Chr5 23005790 23005790 m64079_221220_112036/79759803/ccs Chr5 8256941 8265133
Chr5|8256939|8265132|ID=TE_MANUAL_01;Name=CAC2A;classification=DNA/DTC;sequence_ontology=MANUAL;identity=MANUAL;method=MANUAL;ID=TE_MANUAL_01;sequence_ontology=MANUAL met1_06

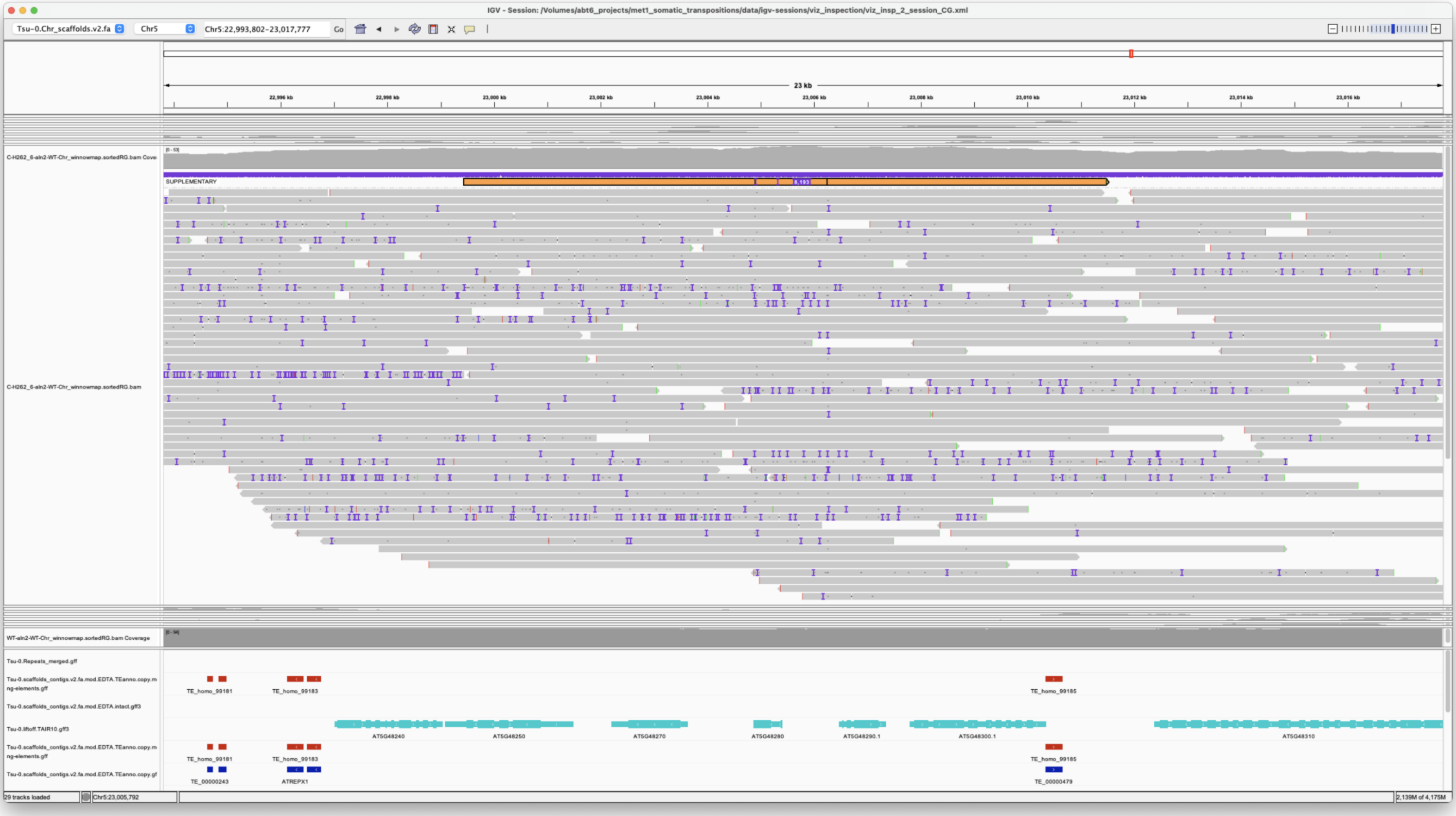

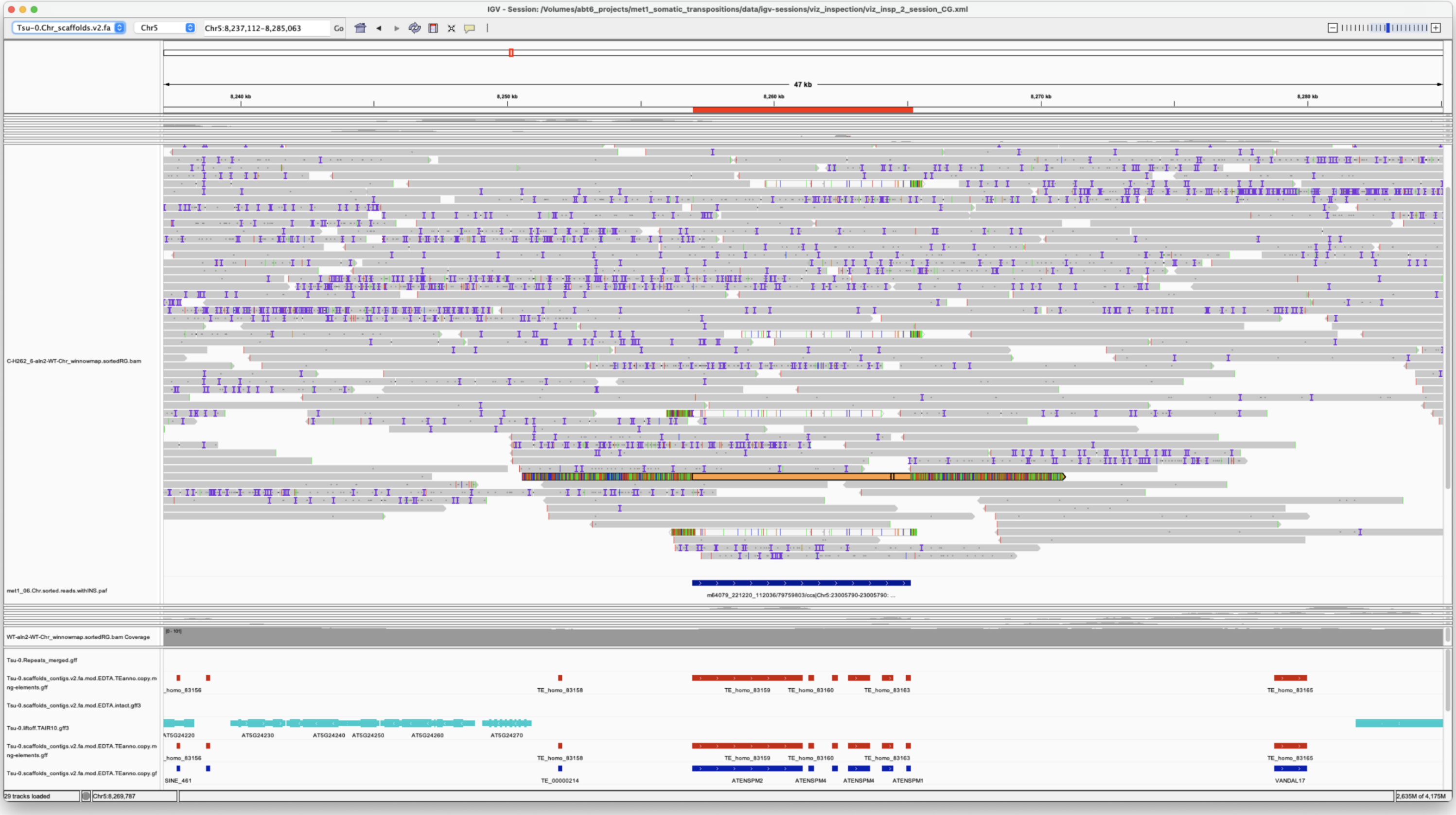

*Confirmed*

## met1_07

> Chr1 276643 276643 m64079_240212_113350/83624893/ccs Chr5 875415 876434
> Chr5|875414|876433|ID=TE_MANUAL_02;Name=PAC;classification=DNA/DTC;sequence_ontology=MANUAL;identity=MANUAL;method=MANUAL;ID=TE_MANUAL_02;sequence_ontology=MANUAL met1_07

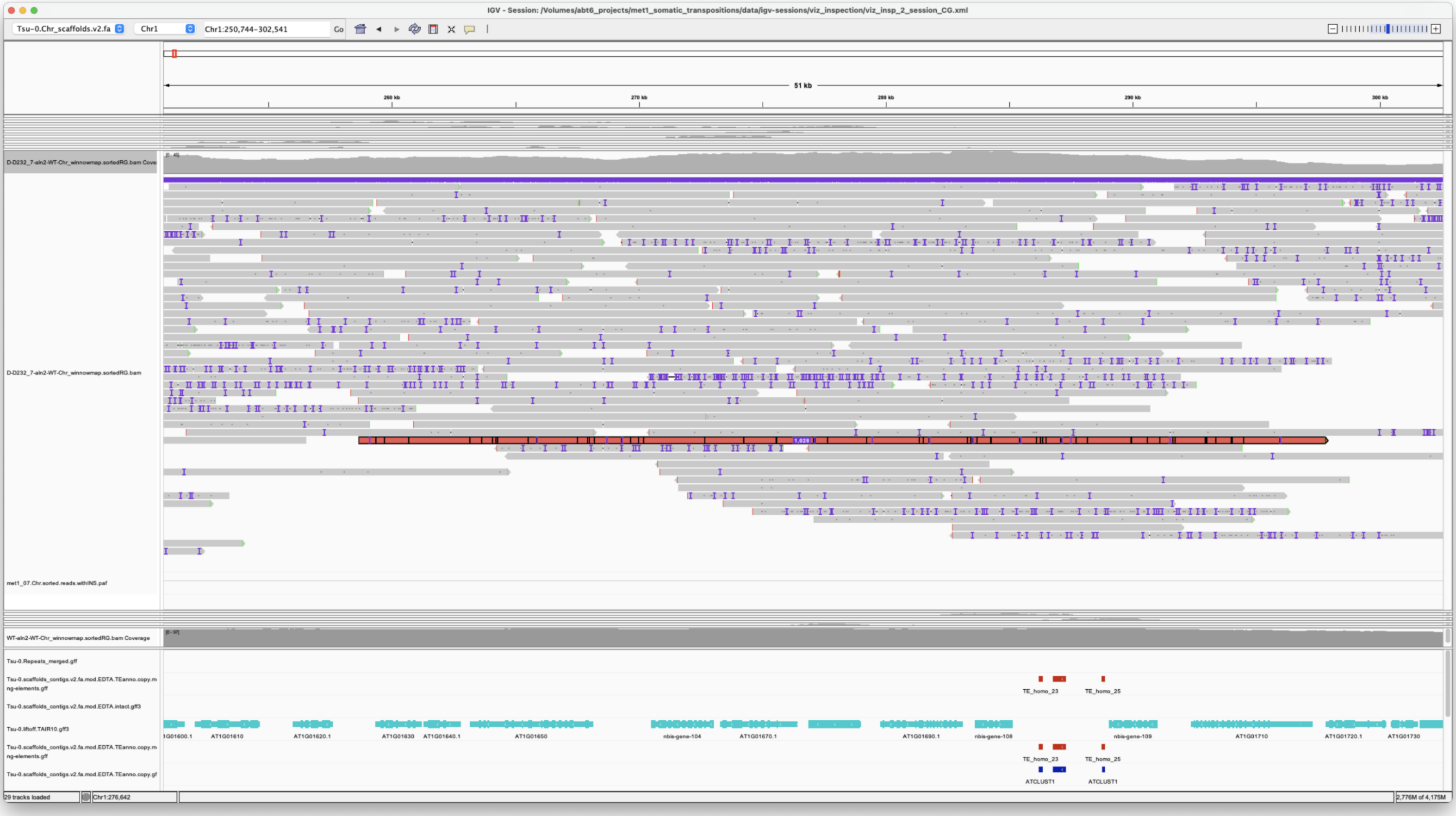

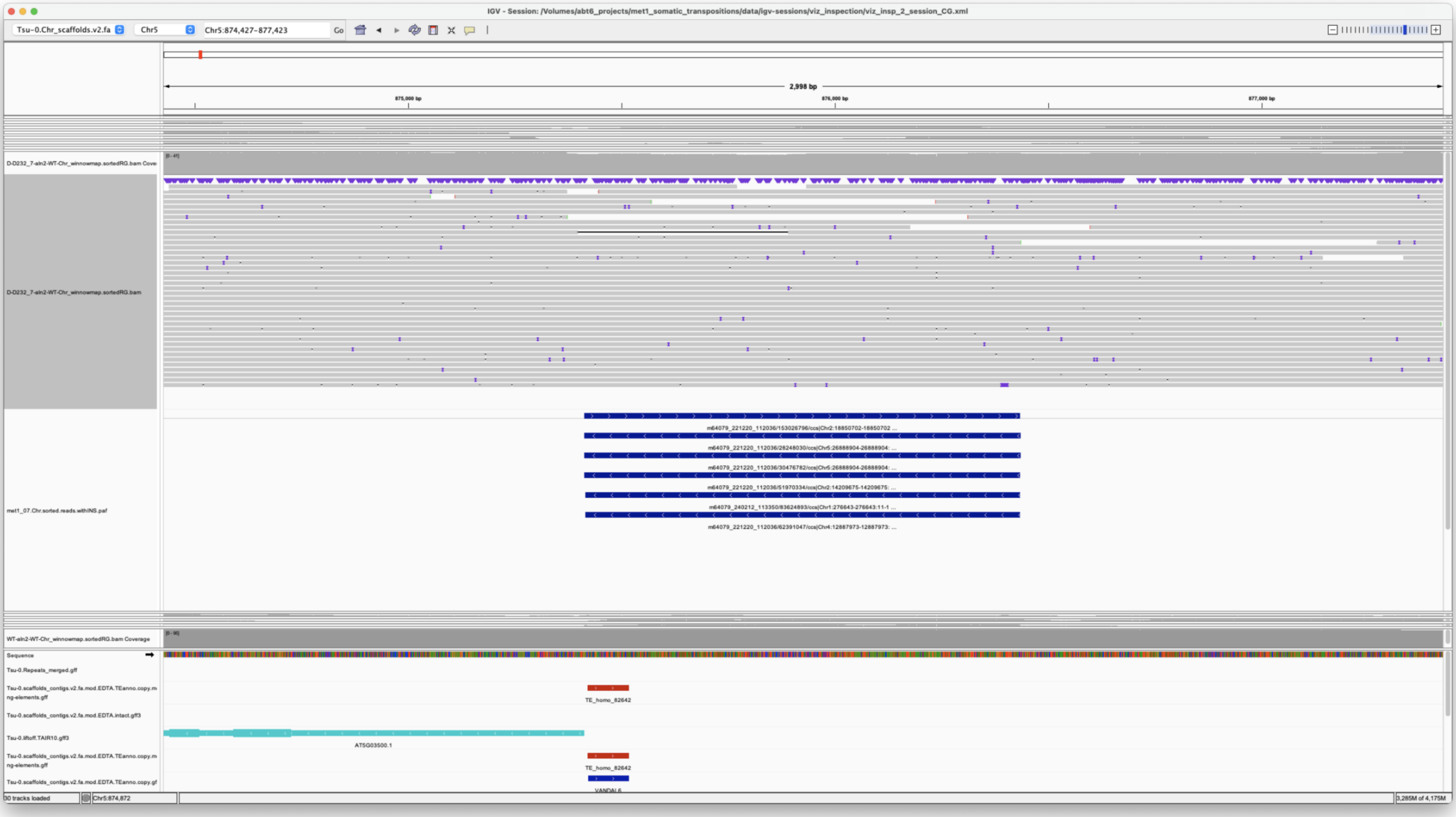

*Confirmed*

---

> Chr1 8551345 8551345 m64079_221220_112036/173541973/ccs Chr1 11941106 11946436
> Chr1|11941106|11946435|ID=LTRRT_5;Name=ATCOPIA93.2_Evade;Classification=LTR/Copia;Sequence_ontology=SO:0002264;ltr_identity=1.0000;Method=structural;motif=TACA;tsd=ATATG met1_07

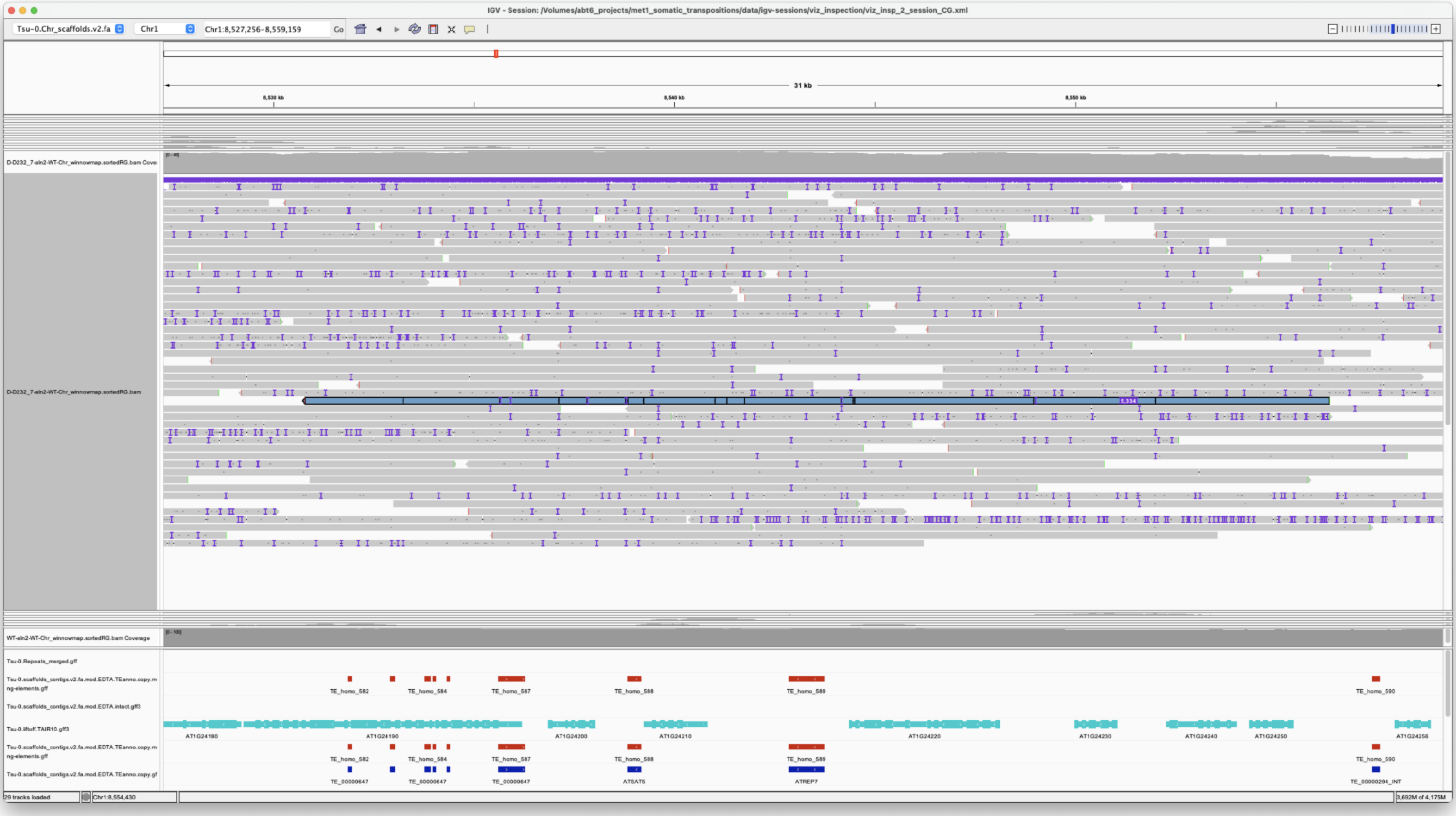


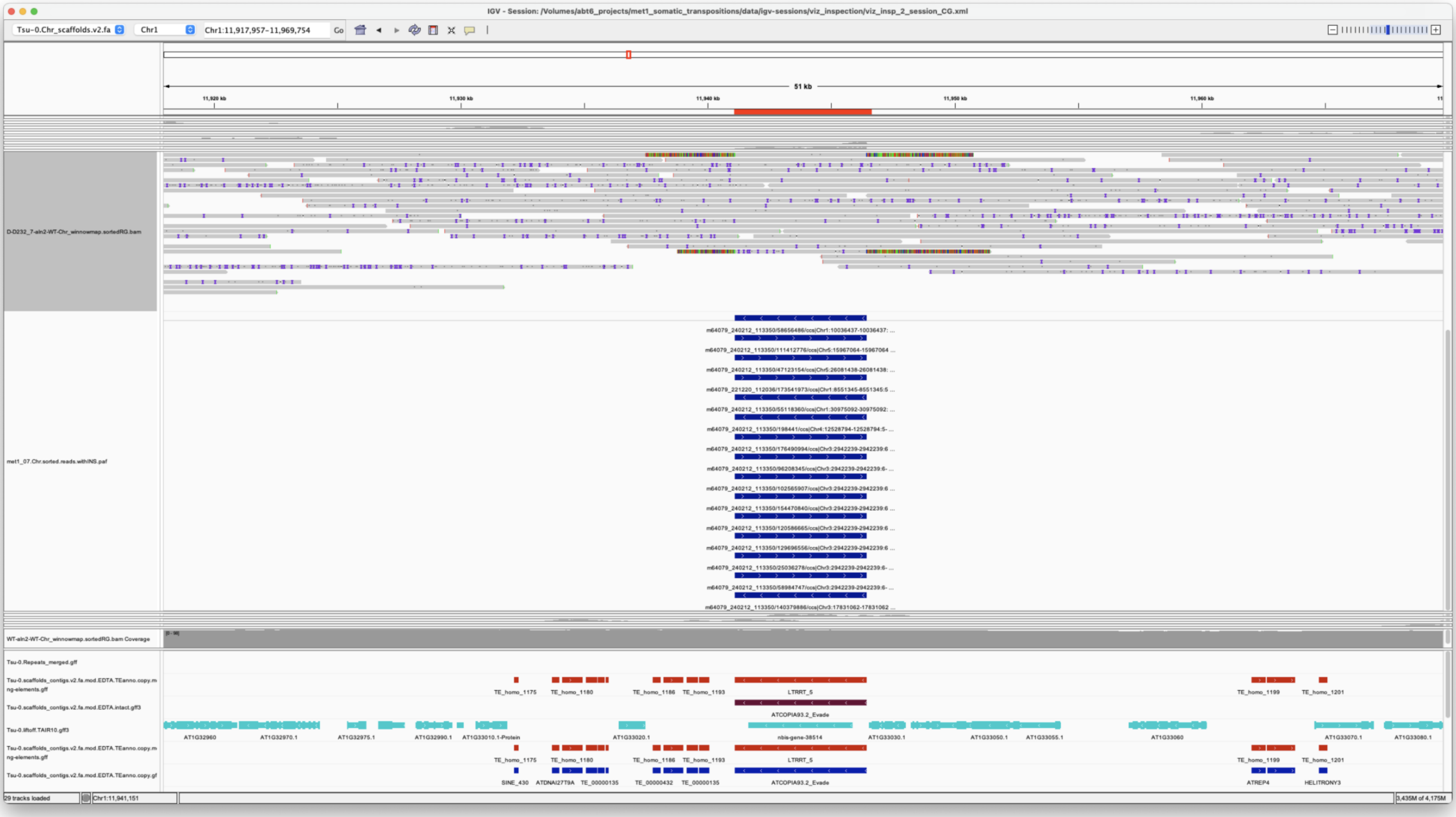


*Confirmed*

Chr1 10036437 10036437 m64079_240212_113350/58656486/ccs Chr1 11941106 11946437 Chr1|11941106|11946435|ID=LTRRT_5;Name=ATCOPIA93.2_Evade;Classification=LTR/Copia;Sequence_ontology=SO:0002264;ltr_identity=1.0000;Method=structural;motif=TACA;tsd=ATATG met1_07

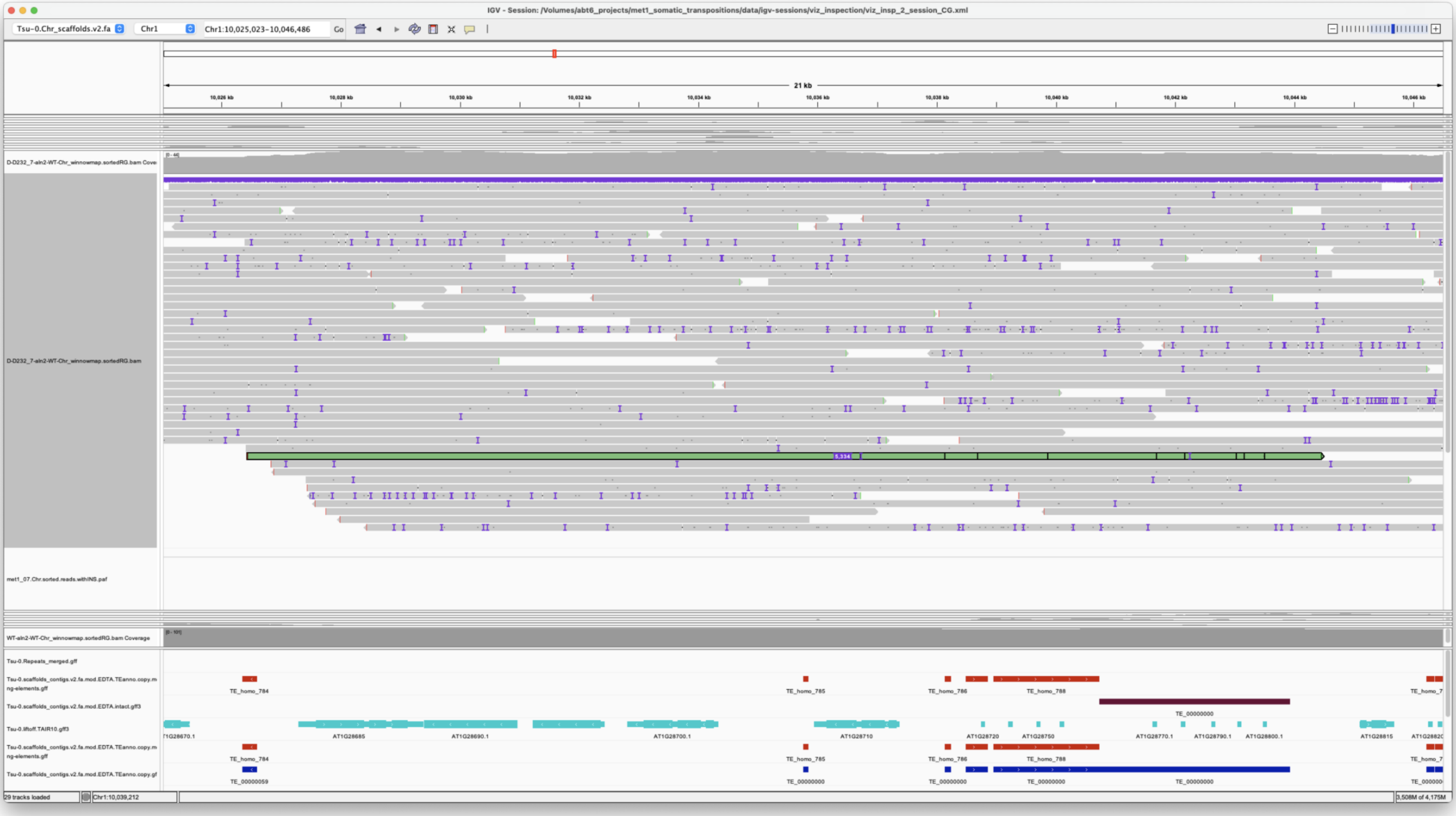


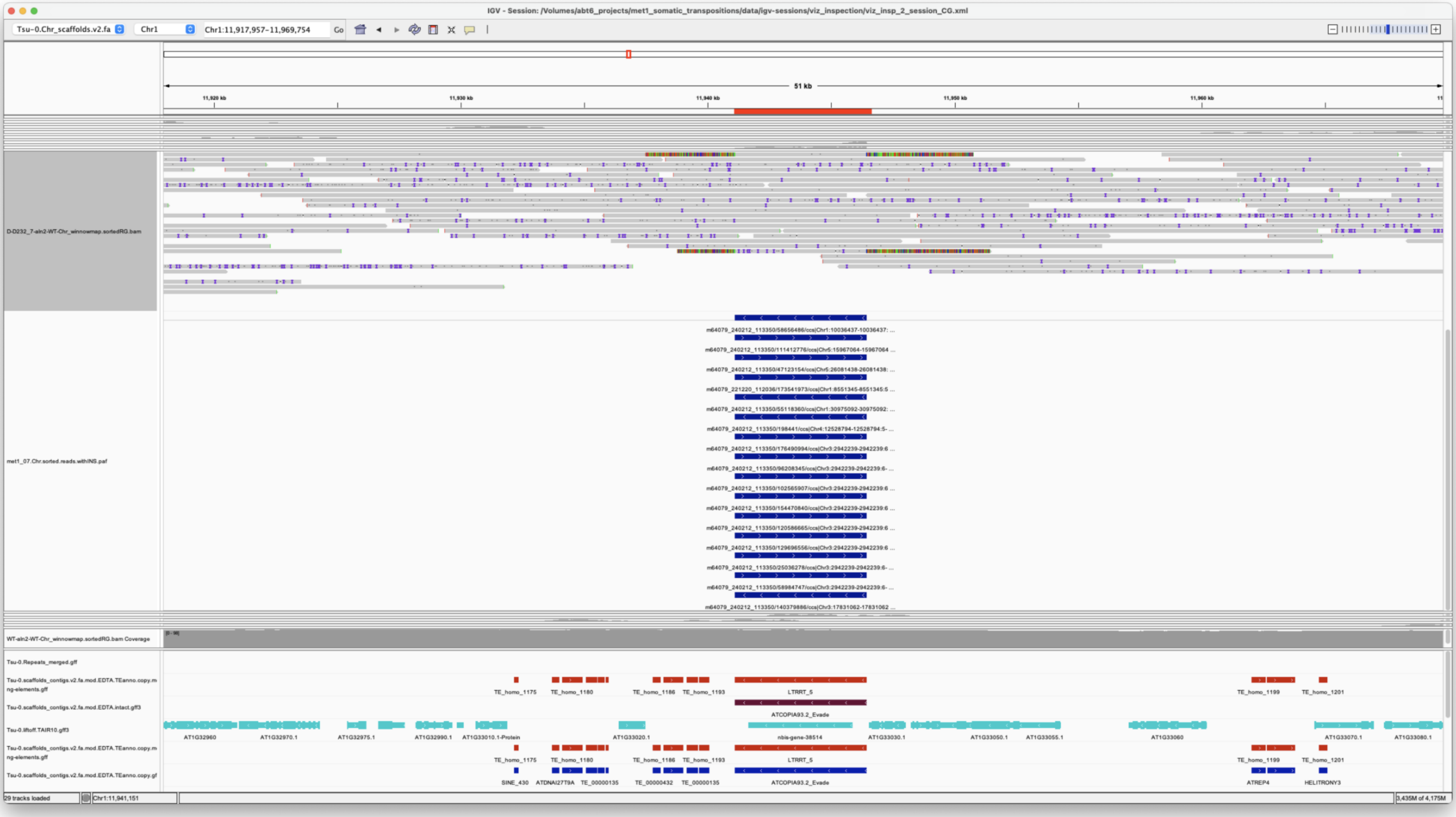


*Confirmed*

> Chr1 24744044 24744044 m64079_221220_112036/22151649/ccs Chr3 16344525 16352497
> Chr3|16344522|16352496|ID=TE_homo_60420;Name=VANDAL6;classification=DNA/Mutator;sequence_ontology=SO:0002280;identity=0.969;method=homology;ID=TE_homo_62001;sequence_ontology=SO:0002280 met1_07

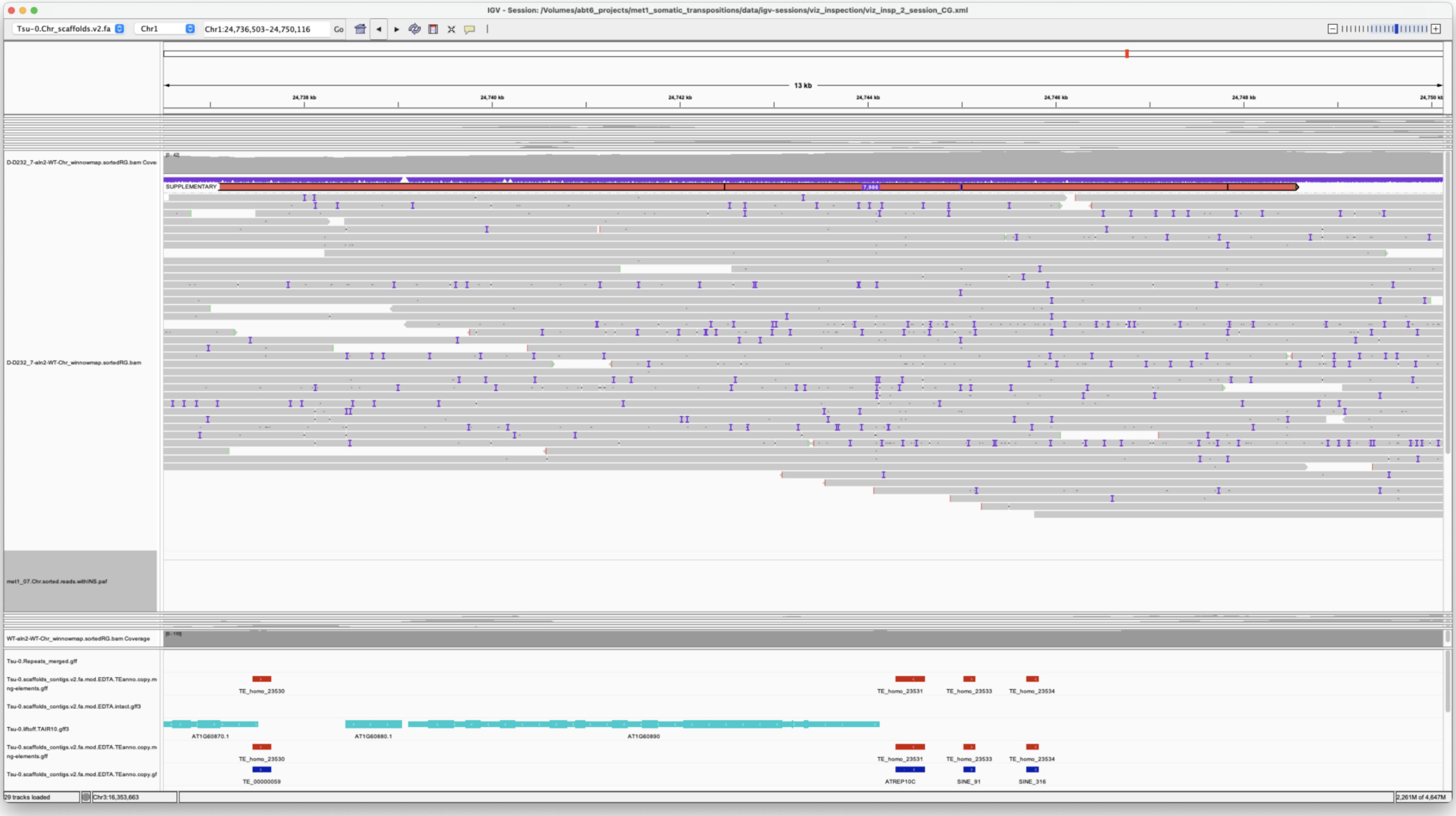


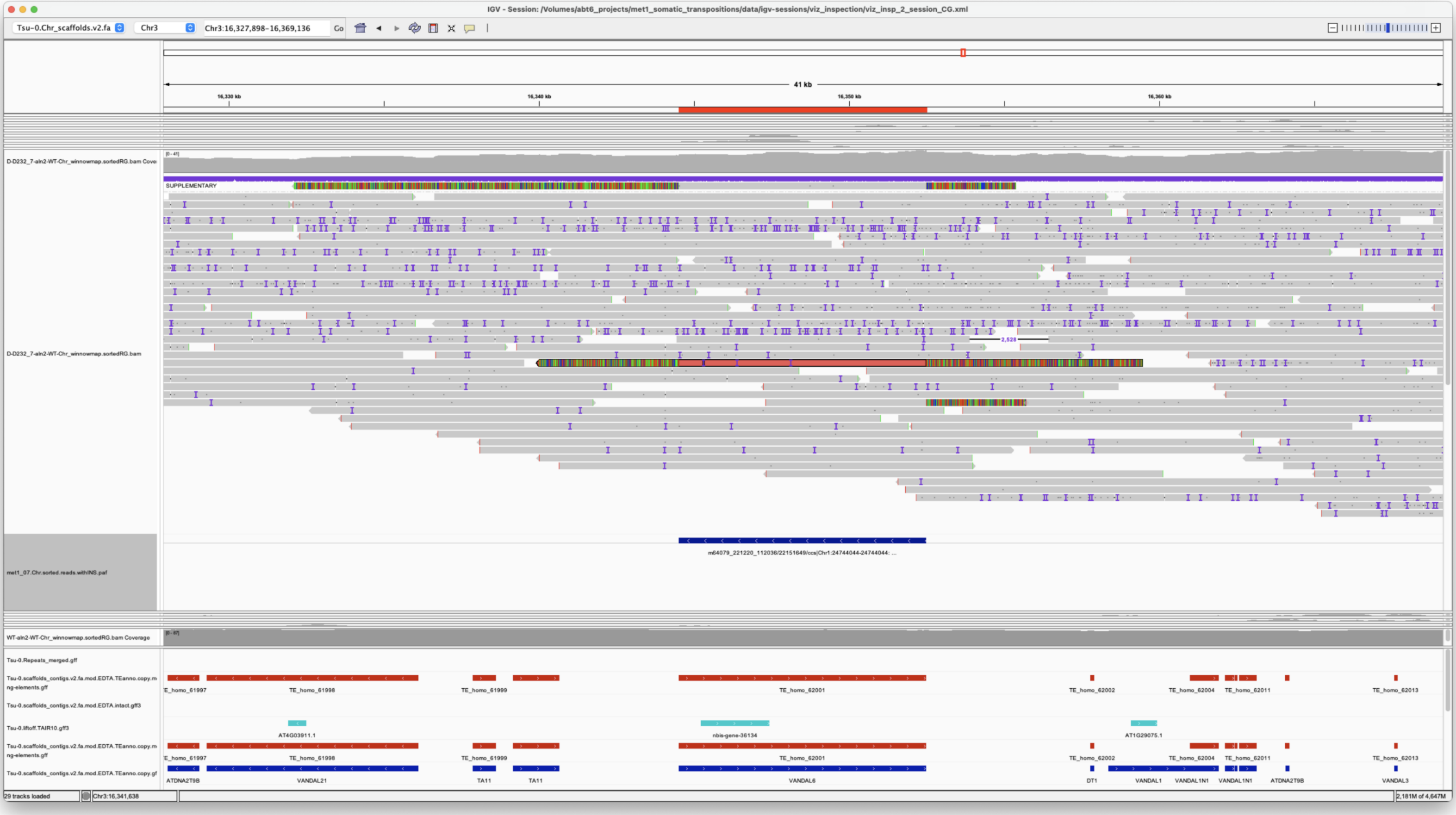


*Confirmed*

Chr1 30975092 30975092 m64079_240212_113350/55118360/ccs Chr5 21419693 21425022 Chr5|21419693|21425021|ID=LTRRT_299;Name=ATCOPIA93.2_Evade;Classification=LTR/Copia;Sequence_ontology=SO:0002264;ltr_identity=1.0000;Method=structural;motif=TACA;tsd=GGACA met1_07

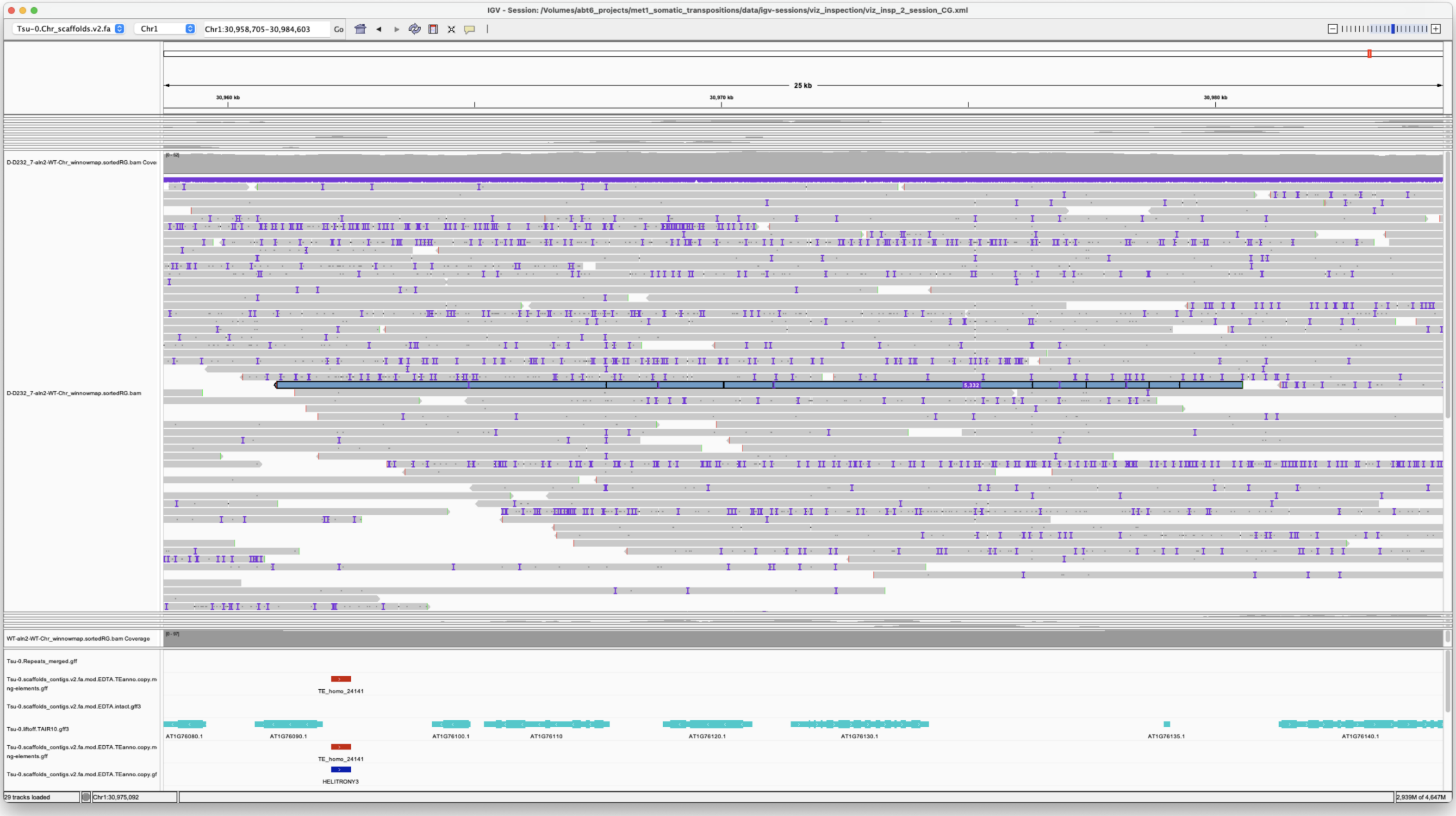


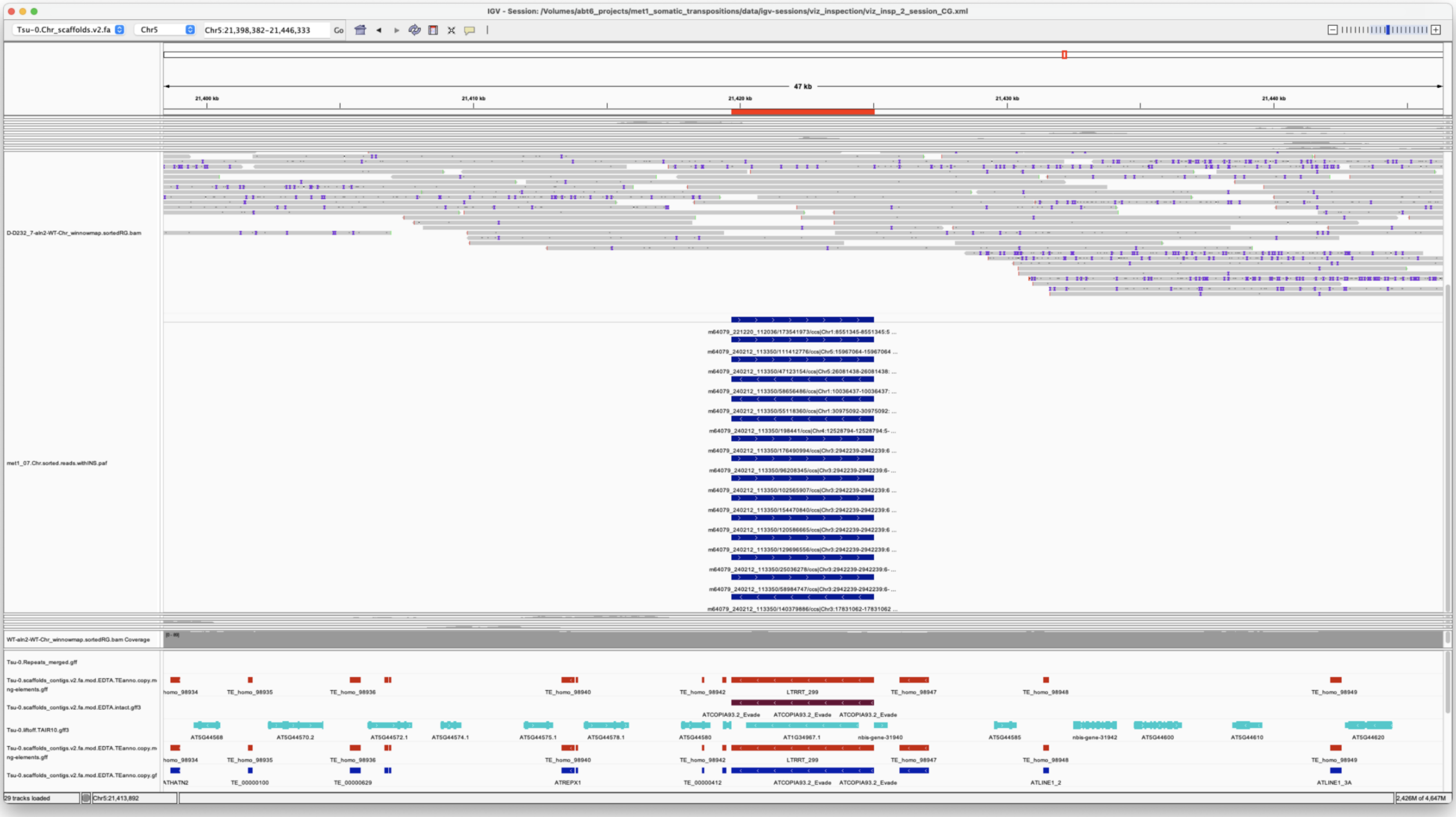


*Confirmed*

> Chr2 14209675 14209675 m64079_221220_112036/51970334/ccs Chr5 875413 876434
> Chr5|875414|876433|ID=TE_MANUAL_02;Name=PAC;classification=DNA/DTC;sequence_ontology=MANUAL;identity=MANUAL;method=MANUAL;ID=TE_MANUAL_02;sequence_ontology=MANUAL met1_07

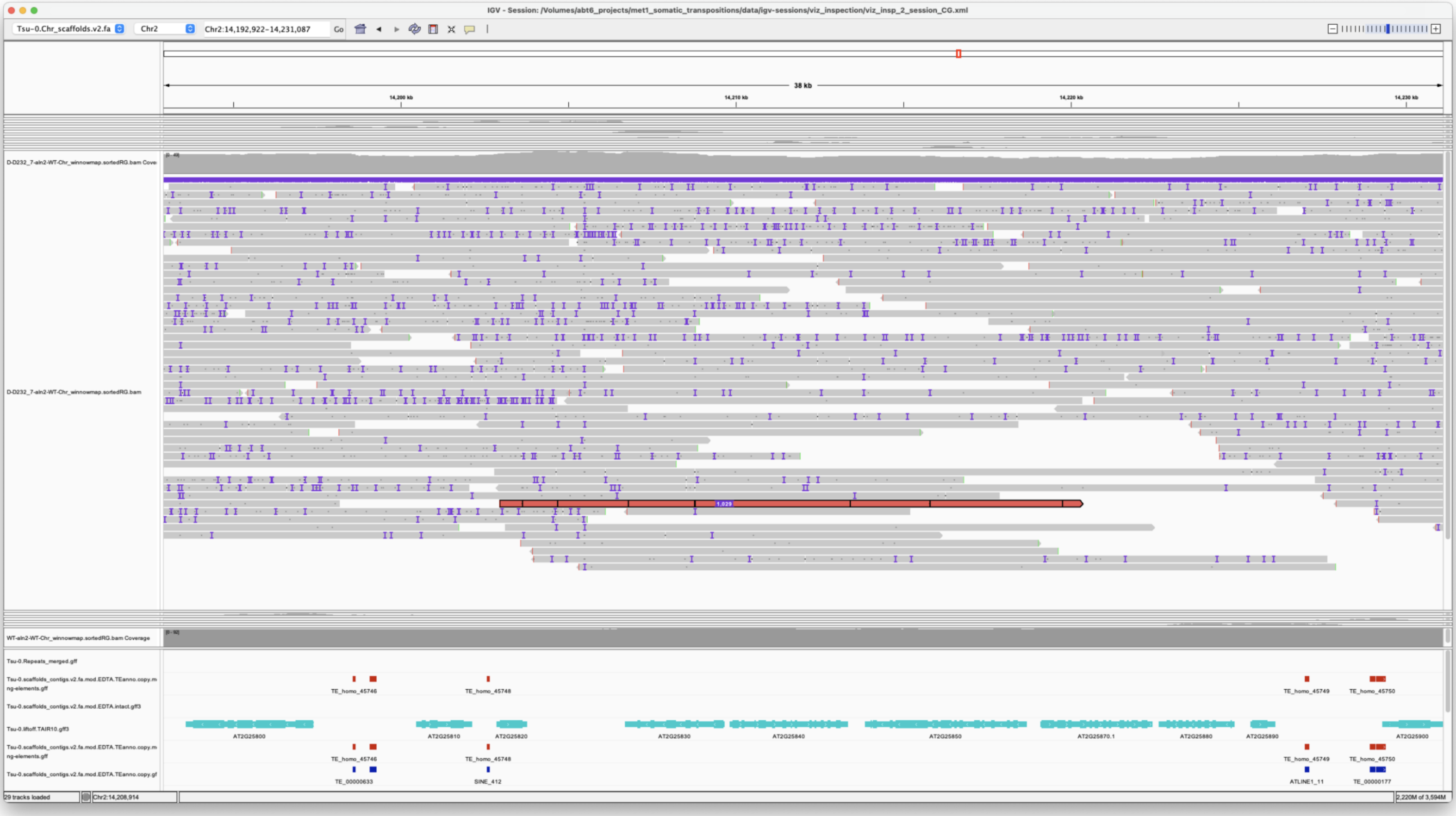

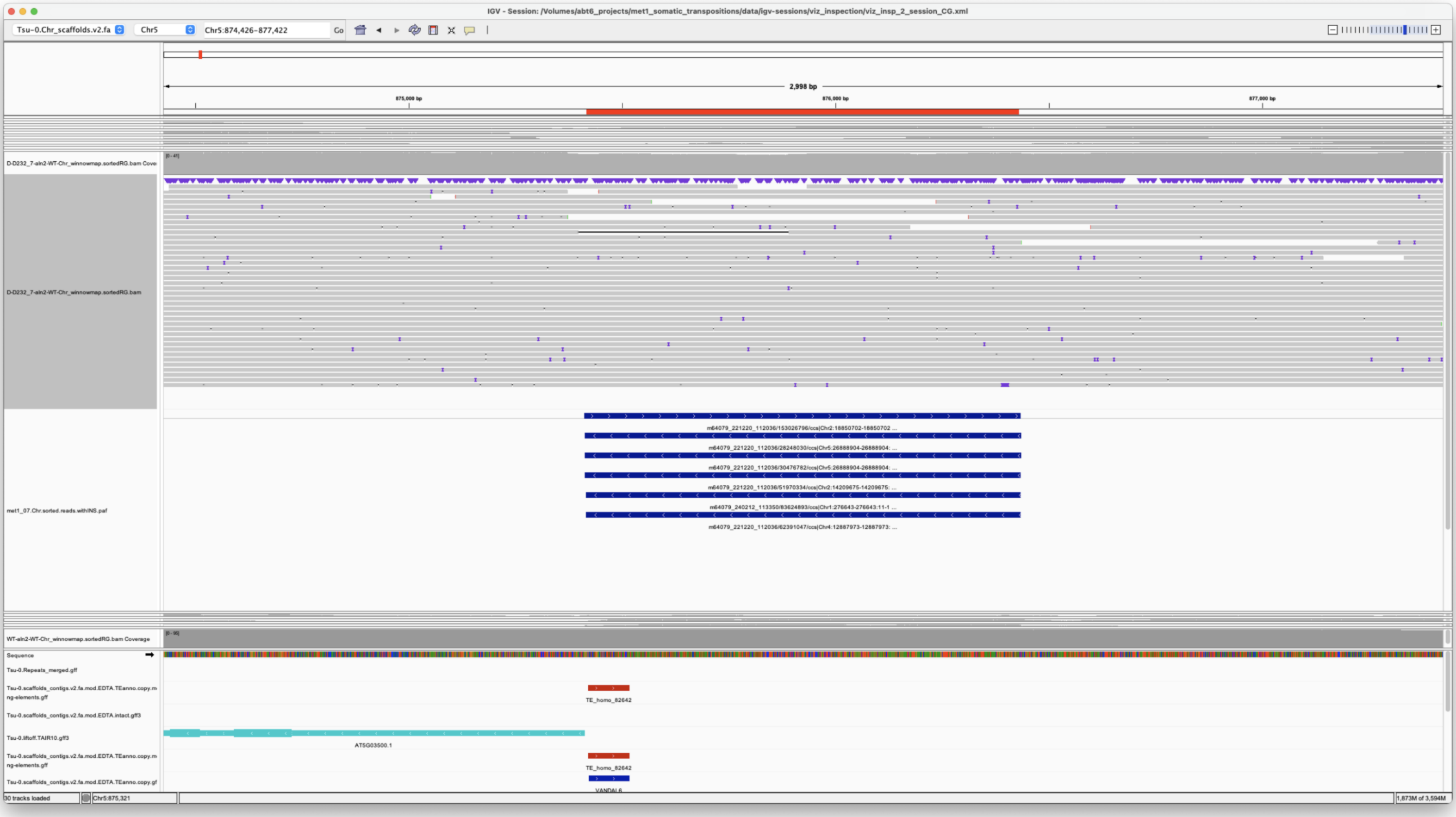

*Confirmed*

> Chr2 18850702 18850702 m64079_221220_112036/153026796/ccs Chr5 875412 876434
> Chr5|875414|876433|ID=TE_MANUAL_02;Name=PAC;classification=DNA/DTC;sequence_ontology=MANUAL;identity=MANUAL;method=MANUAL;ID=TE_MANUAL_02;sequence_ontology=MANUAL met1_07

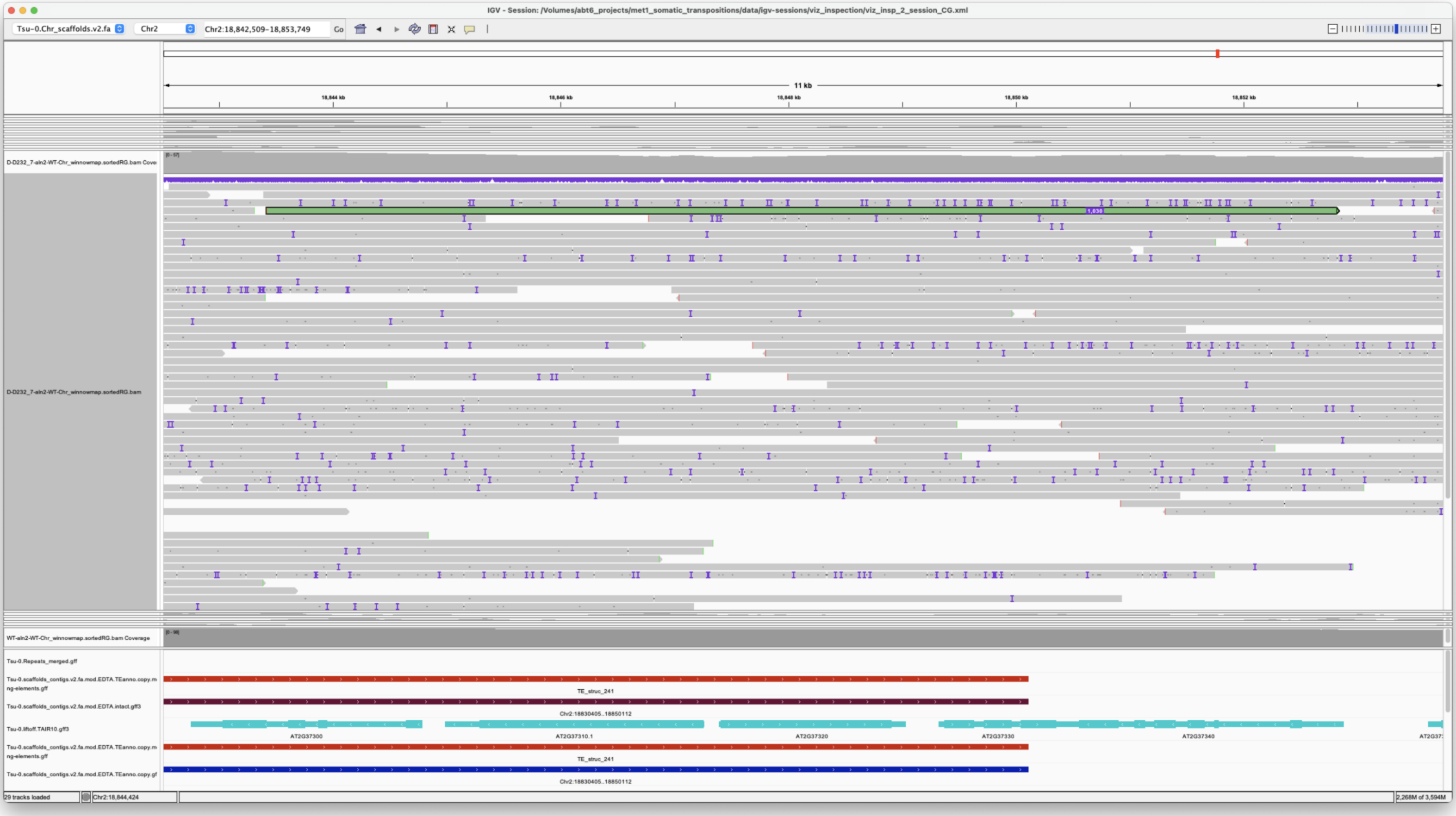


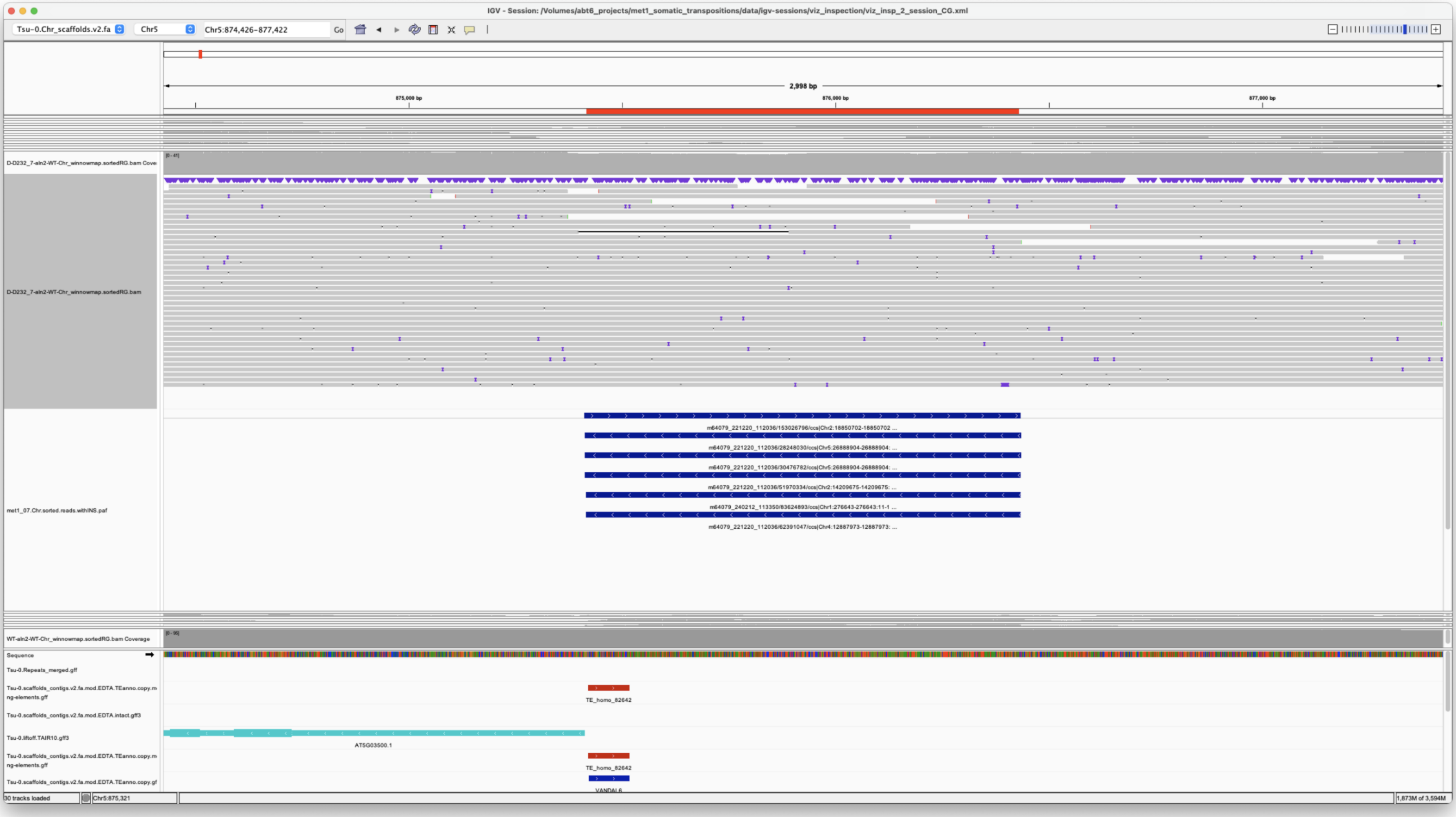


*Confirmed*

---

> Chr3 2942238 2942240
> m64079_240212_113350/102565907/ccs**m64079_240212_113350/120586665/ccs**m64079_240212_113350/129696556/ccs**m64079_240212_113350/154470840/ccs**m64079_240212_113350/176490994/ccs**m64079_240212_113350/25036278/ccs**m64079_240212_113350/58984747/ccs**m64079_240212_113350/96208345/ccs Chr1 11941106 11946435**
> **Chr1|11941106|11946435|ID=LTRRT_5;Name=ATCOPIA93.2_Evade;Classification=LTR/Copia;Sequence_ontology=SO:0002264;ltr_identity=1.0000;Method=structural;motif=TACA;tsd=ATATG**Chr1|11941106|11946435|ID=LTRRT_5;Name=ATCOPIA93.2_Evade;Classification=LTR/Copia;Sequence_ontology=SO:0002264;ltr_identity=1.0000;Method=structural;motif=TACA;tsd=ATATG**Chr1|11941106|11946435|ID=LTRRT_5;Name=ATCOPIA93.2_Evade;Classification=LTR/Copia;Sequence_ontology=SO:0002264;ltr_identity=1.0000;Method=structural;motif=TACA;tsd=ATATG**Chr1|11941106|11946435|ID=LTRRT_5;Name=ATCOPIA93.2_Evade;Classification=LTR/Copia;Sequence_ontology=SO:0002264;ltr_identity=1.0000;Method=structural;motif=TACA;tsd=ATATG**Chr1|11941106|11946435|ID=LTRRT_5;Name=ATCOPIA93.2_Evade;Classification=LTR/Copia;Sequence_ontology=SO:0002264;ltr_identity=1.0000;Method=structural;motif=TACA;tsd=ATATG**Chr1|11941106|11946435|ID=LTRRT_5;Name=ATCOPIA93.2_Evade;Classification=LTR/Copia;Sequence_ontology=SO:0002264;ltr_identity=1.0000;Method=structural;motif=TACA;tsd=ATATG**Chr1|11941106|11946435|ID=LTRRT_5;Name=ATCOPIA93.2_Evade;Classification=LTR/Copia;Sequence_ontology=SO:0002264;ltr_identity=1.0000;Method=structural;motif=TACA;tsd=ATATG**Chr1|11941106|11946435|ID=LTRRT_5;Name=ATCOPIA93.2_Evade;Classification=LTR/Copia;Sequence_ontology=SO:0002264;ltr_identity=1.0000;Method=structural;motif=TACA;tsd=ATATG met1_07
