# Supplementary material for: Long-read detection of transposable element mobilization in the soma of hypomethylated Arabidopsis thaliana individuals
Source: Genome Biol. 2025 Jul 30;26:231. doi: 10.1186/s13059-025-03691-7 (PMC12312487; doi:10.1186/s13059-025-03691-7)
Supplement: Supplementary file 2 — Additional file 2. Visual inspection of somatic insertion and excision events, available at https://github.com/aerilli/Somatic-transposition_met1/tree/551df407370c6528225f404ba62a073dced14b08/Supplementary-Files/Visual_inspection. [file 13059_2025_3691_MOESM2_ESM.gz › Split_Supplementary-File4/File2_CIGAR_Insertions/File2_CIGAR_Insertions_34-66.pdf]

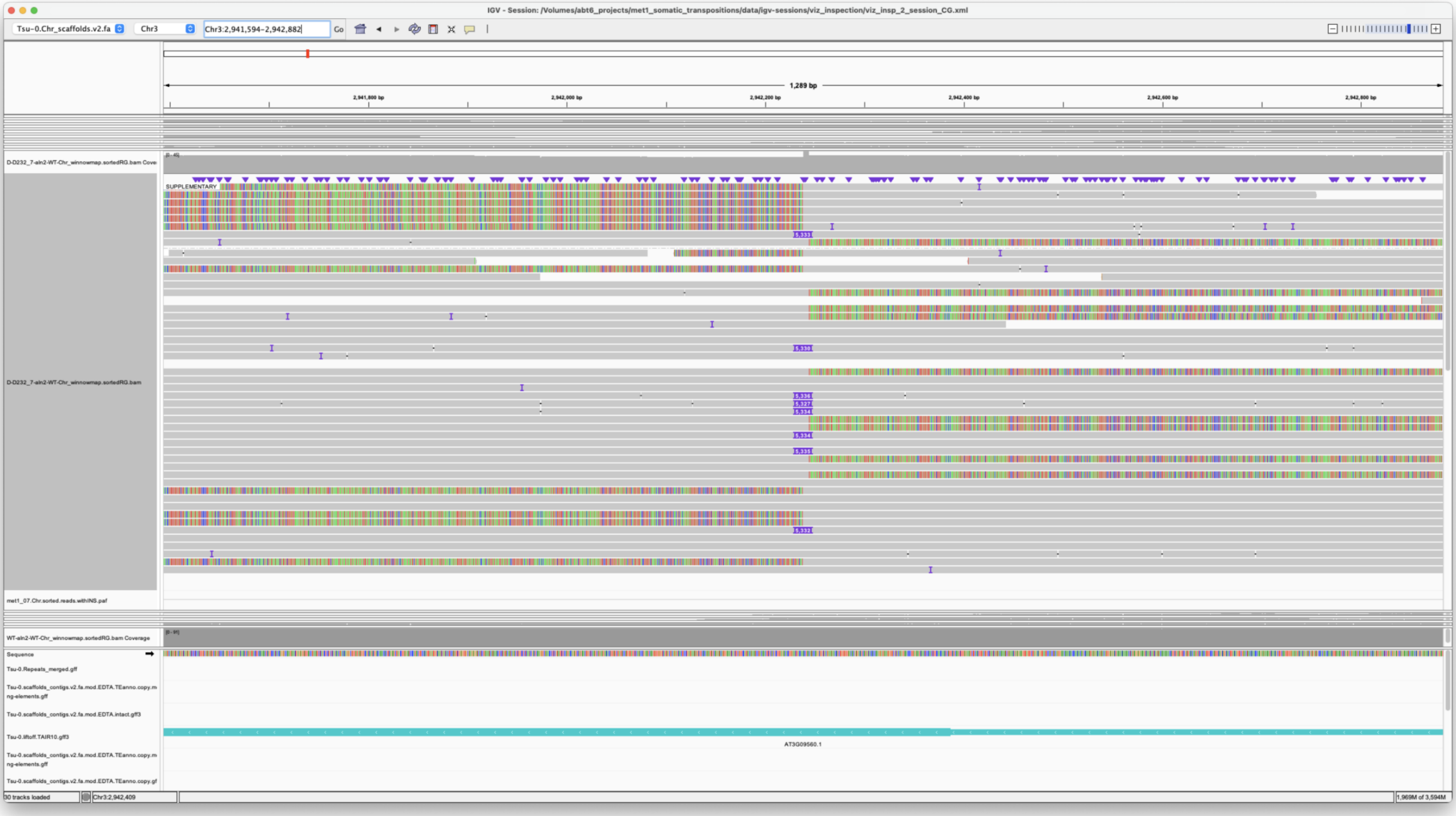

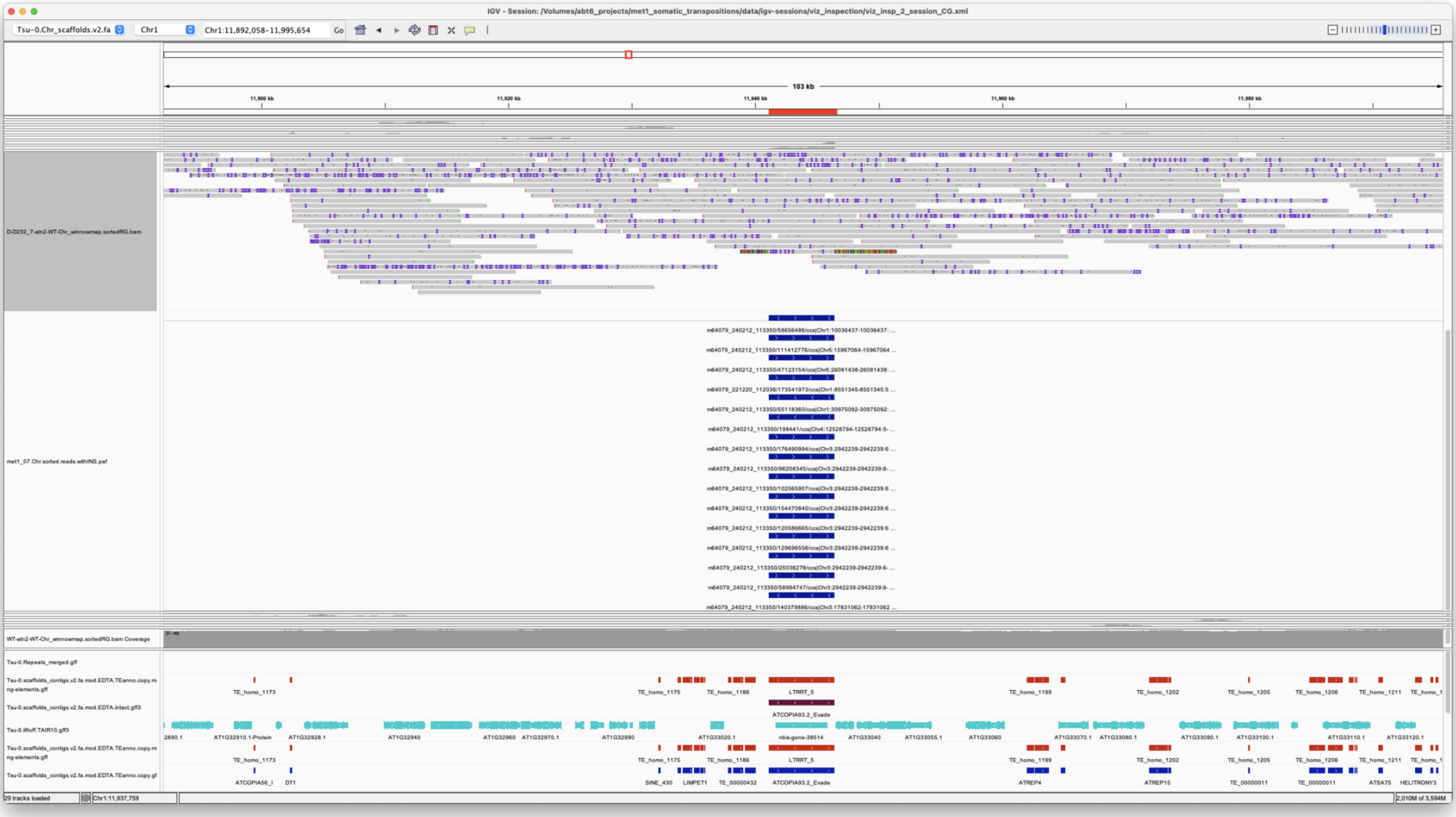

Confirmed

Chr3 17831062 17831062 m64079_240212_113350/140379886/ccs Chr1 11941107 11946436 Chr1|11941106|11946435|ID=LTRRT_5;Name=ATCOPIA93.2_Evade;Classification=LTR/Copia;Sequence_ontology=SO:0002264;ltr_identity=1.0000;Method=structural;motif=TACA;tsd=ATATG met1_07

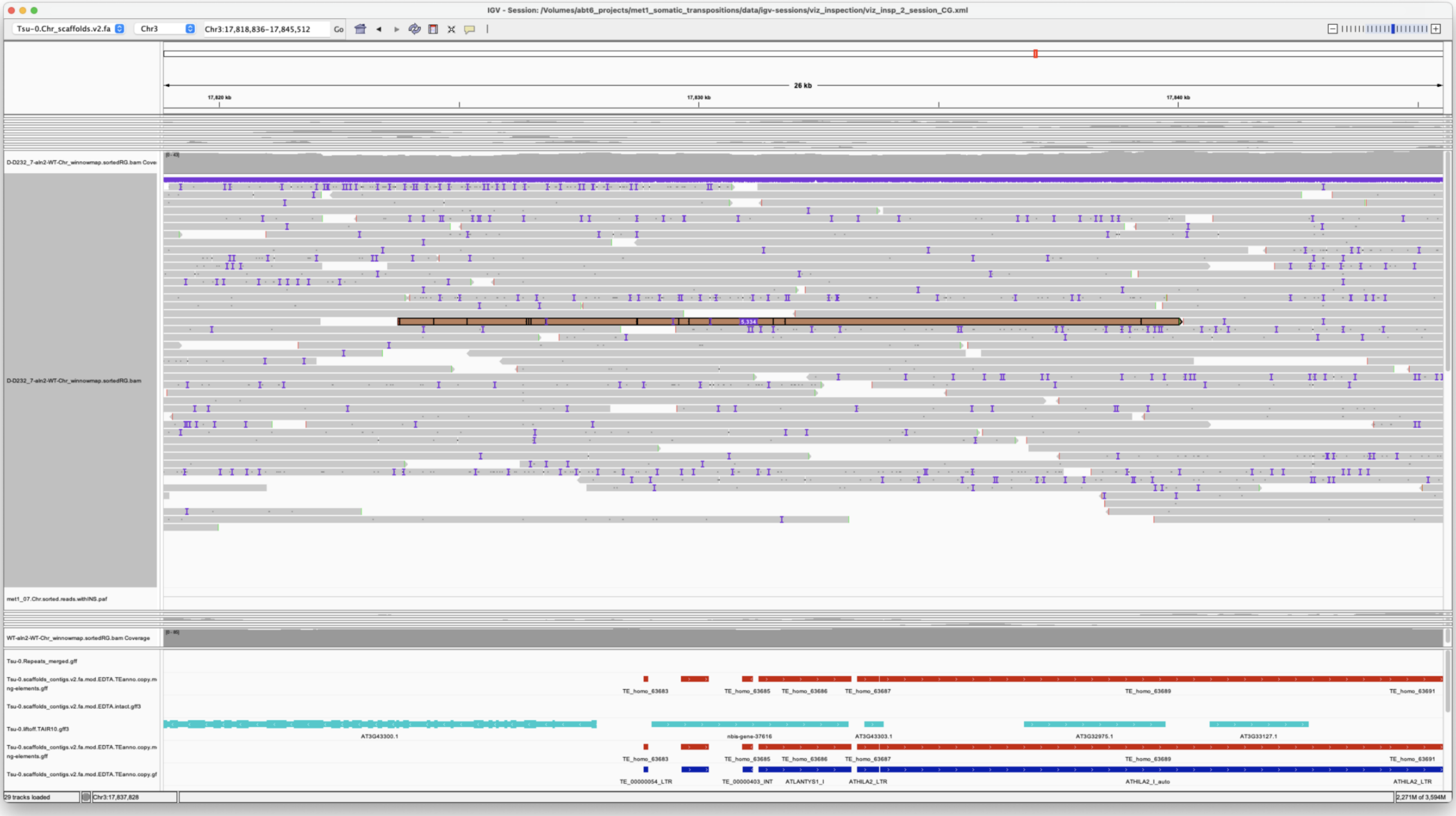

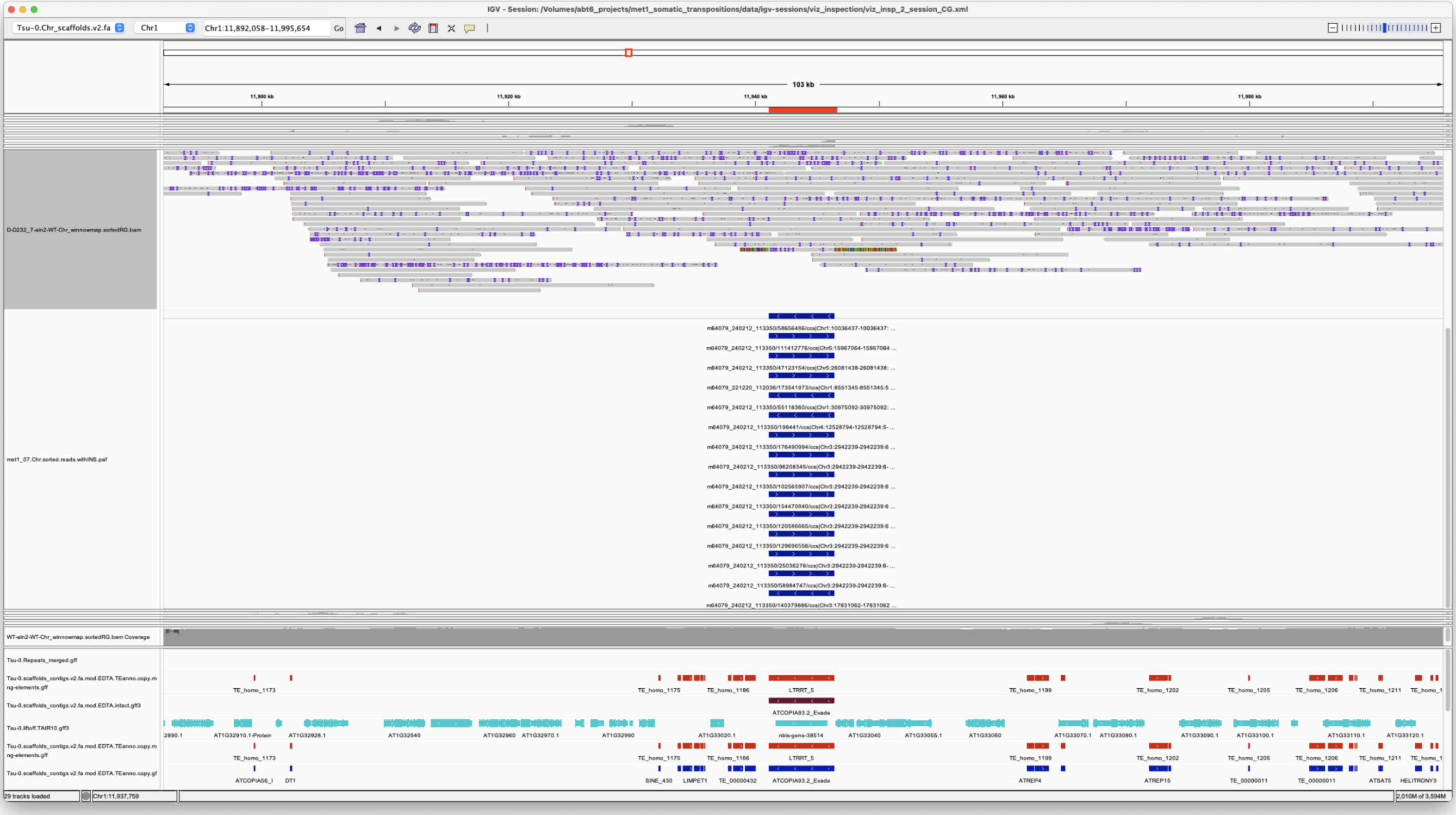

Confirmed

> Chr4 12528794 12528794 m64079_240212_113350/198441/ccs Chr1 11941106 11946436 Chr1|11941106|11946435|ID=LTRRT_5;Name=ATCOPIA93.2_Evade;Classification=LTR/Copia;Sequence_ontology=SO:0002264;ltr_identity=1.0000;Method=structural;motif=TACA;tsd=ATATG met1_07

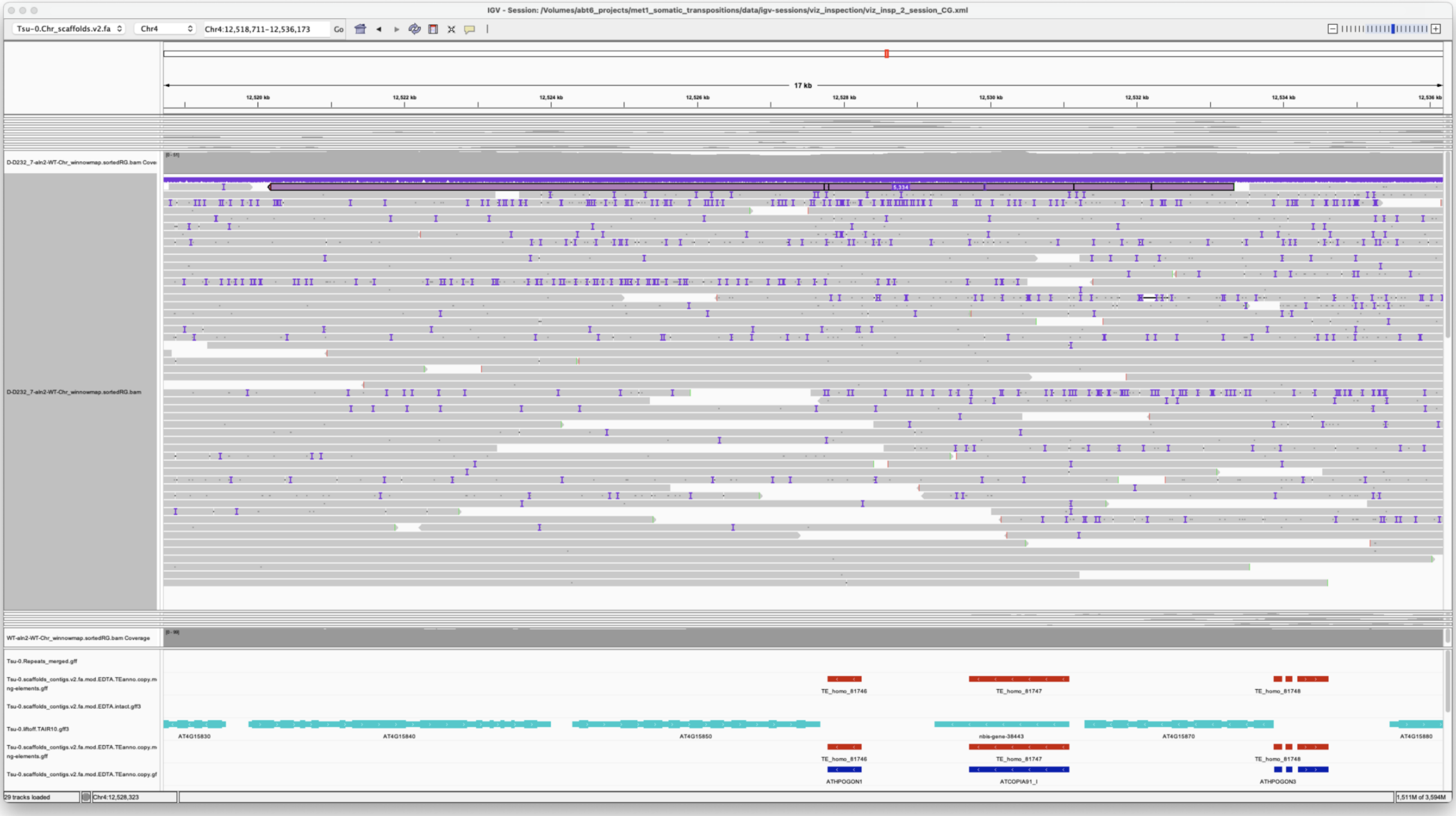

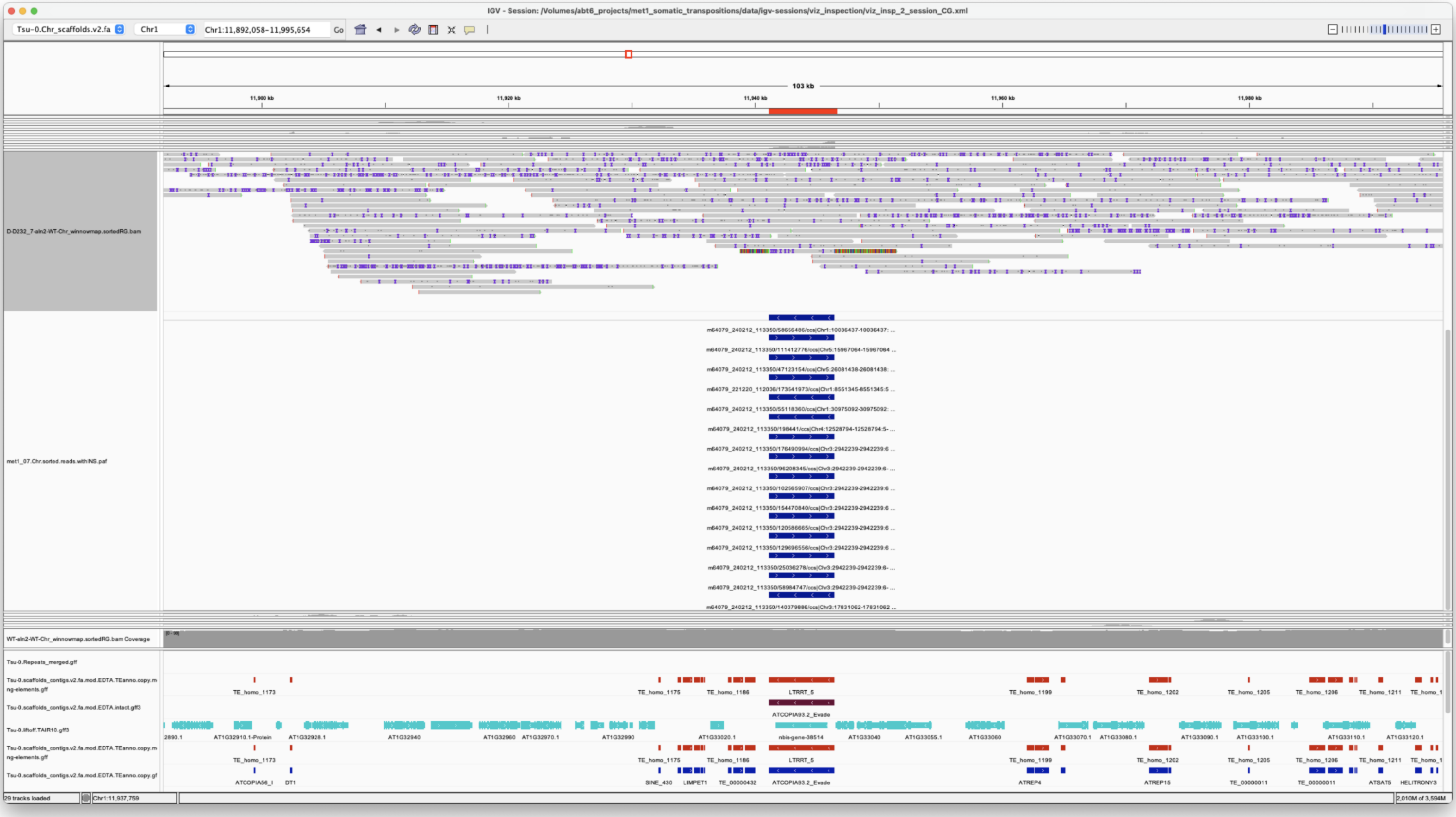

Confirmed

Chr4 12887973 12887973 m64079_221220_112036/62391047/ccs Chr5 875415 876434 Chr5|875414|876433|ID=TE_MANUAL_02;Name=PAC;classification=DNA/DTC;sequence_ontology=MANUAL;identity=MANUAL;method=MANUAL;ID=TE_MANUAL_02;sequence_ontology=MANUAL met1_07

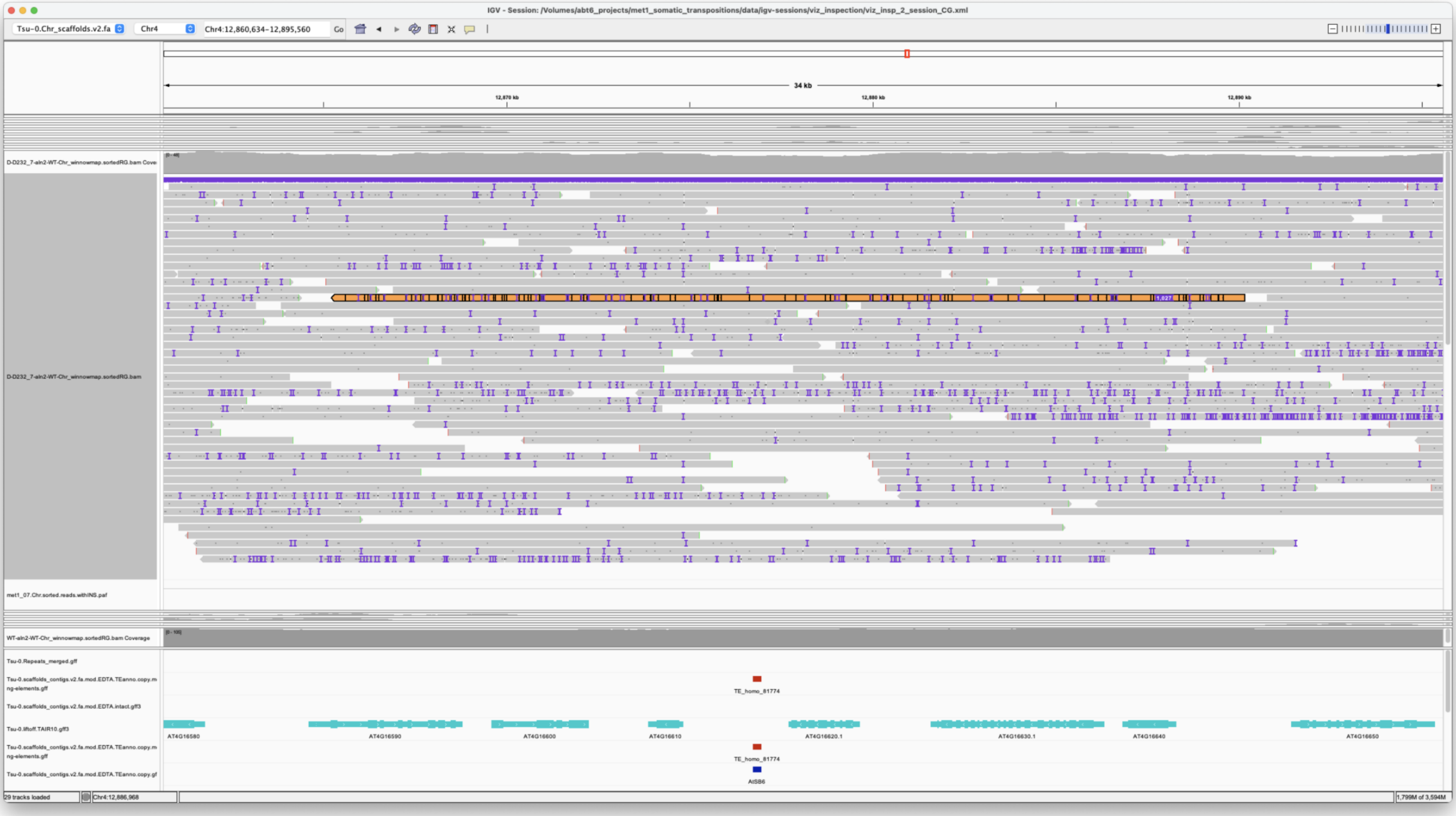

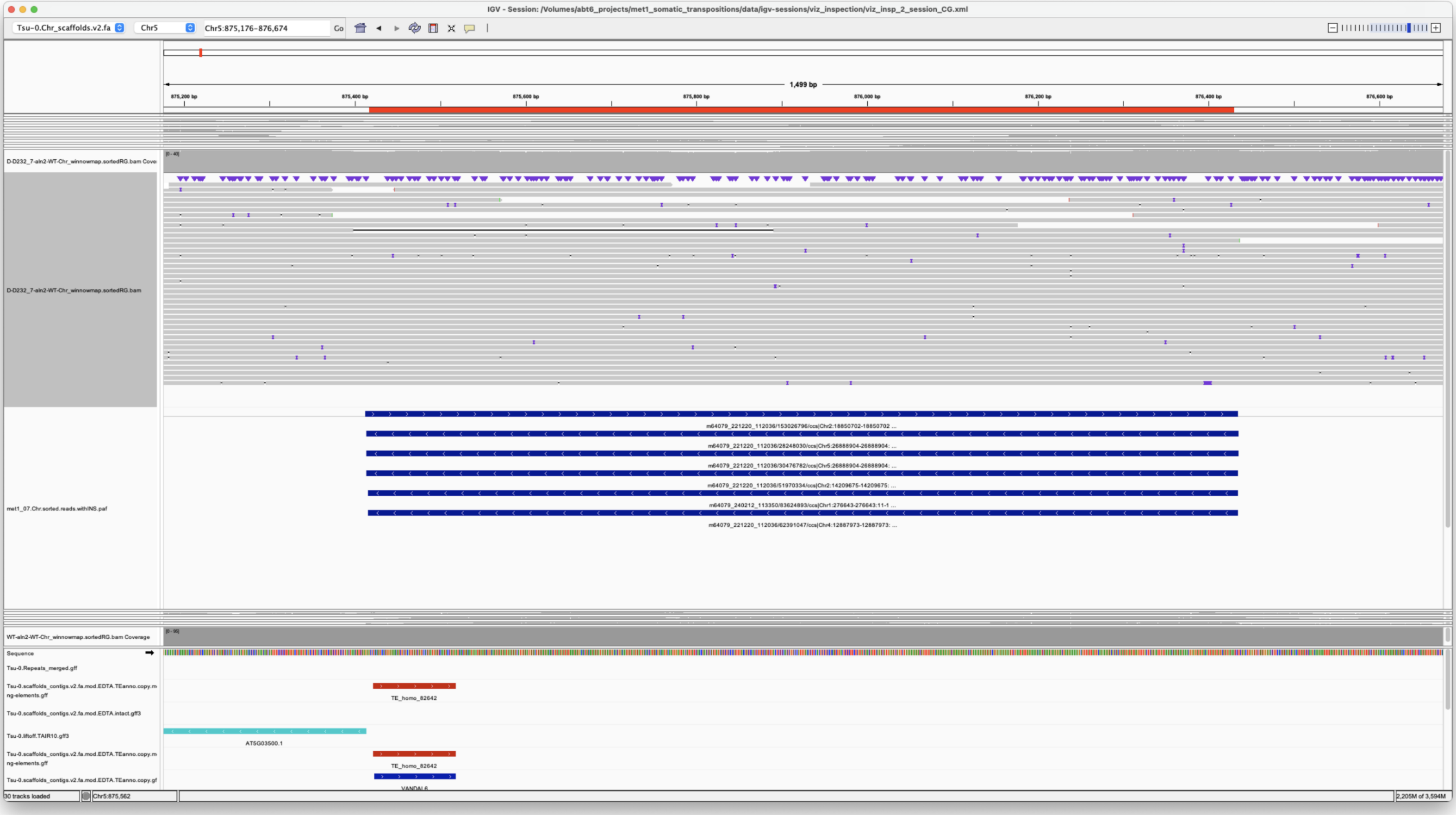

Confirmed

> Chr5 15967064 15967064 m64079_240212_113350/111412776/ccs Chr1 11941106 11946436 Chr1|11941106|11946435|ID=LTRRT_5;Name=ATCOPIA93.2_Evade;Classification=LTR/Copia;Sequence_ontology=SO:0002264;ltr_identity=1.0000;Method=structural;motif=TACA;tsd=ATATG met1_07

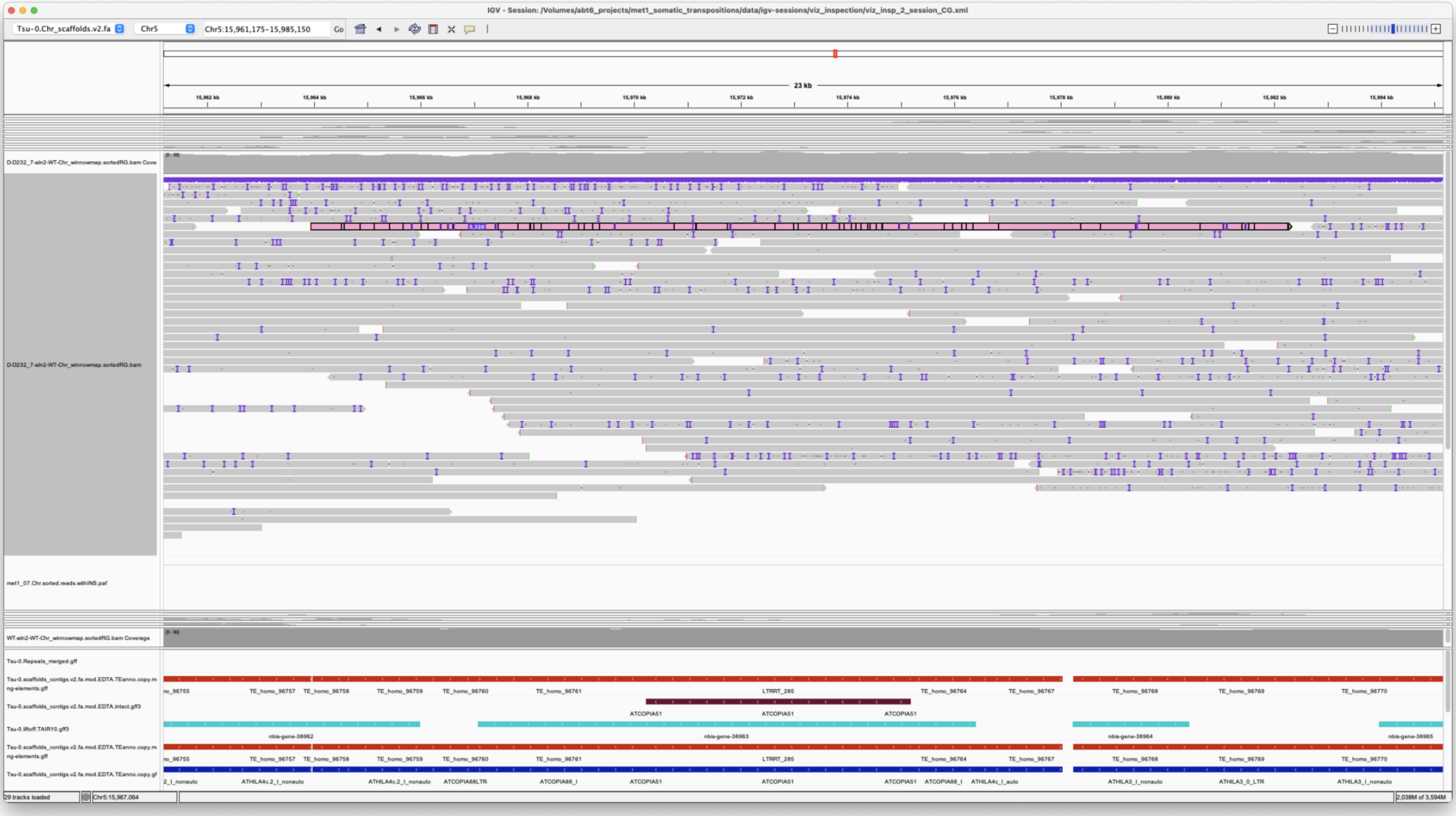

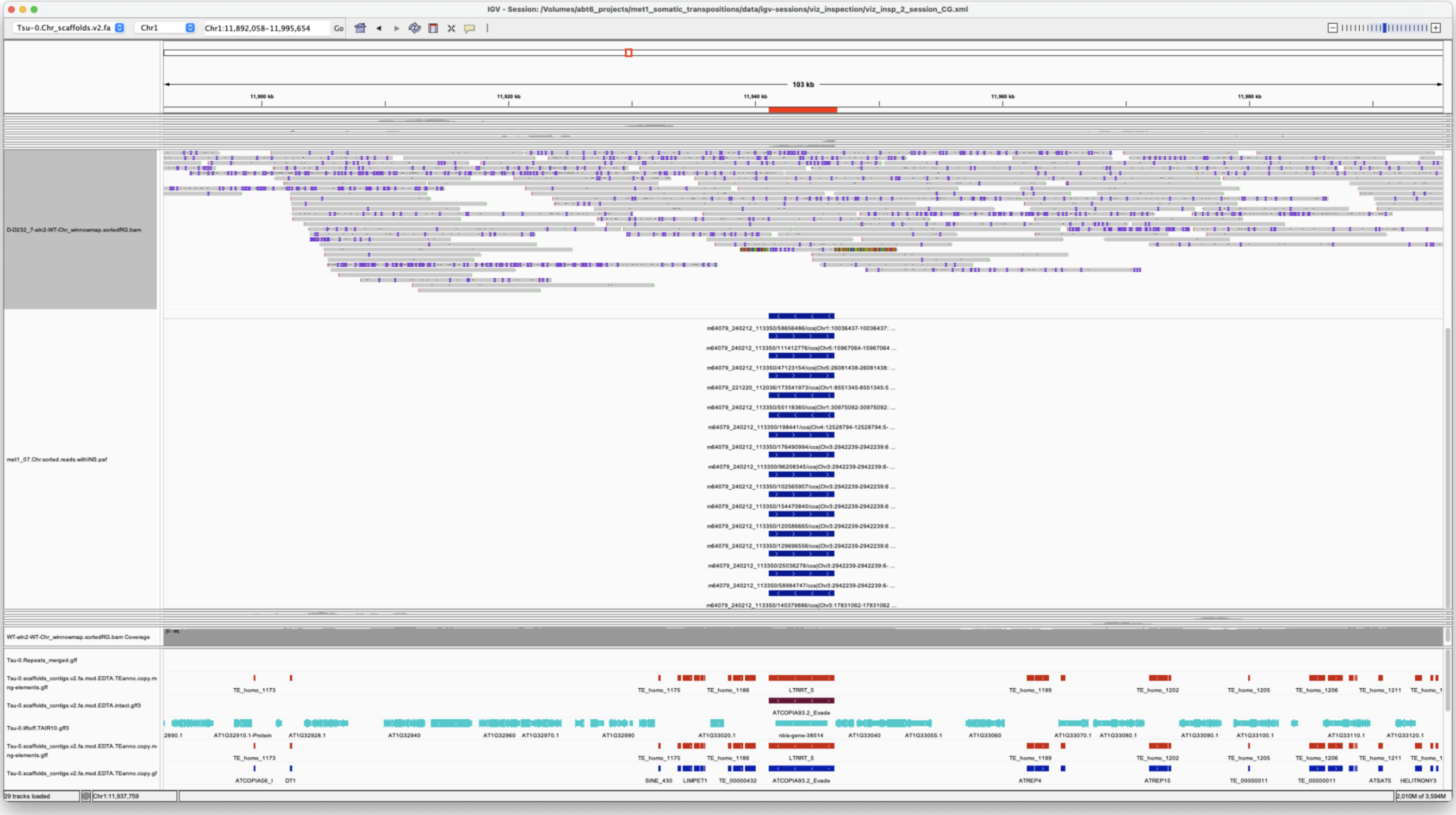

Confirmed

Chr5 26081438 26081438 m64079_240212_113350/47123154/ccs Chr1 11941106 11946436
Chr1|11941106|11946435|ID=LTRRT_5;Name=ATCOPIA93.2_Evade;Classification=LTR/Copia;Sequence_ontology=SO:0002264;ltr_identity=1.0000;Method=structural;motif=TACA;tsd=ATATG met1_07

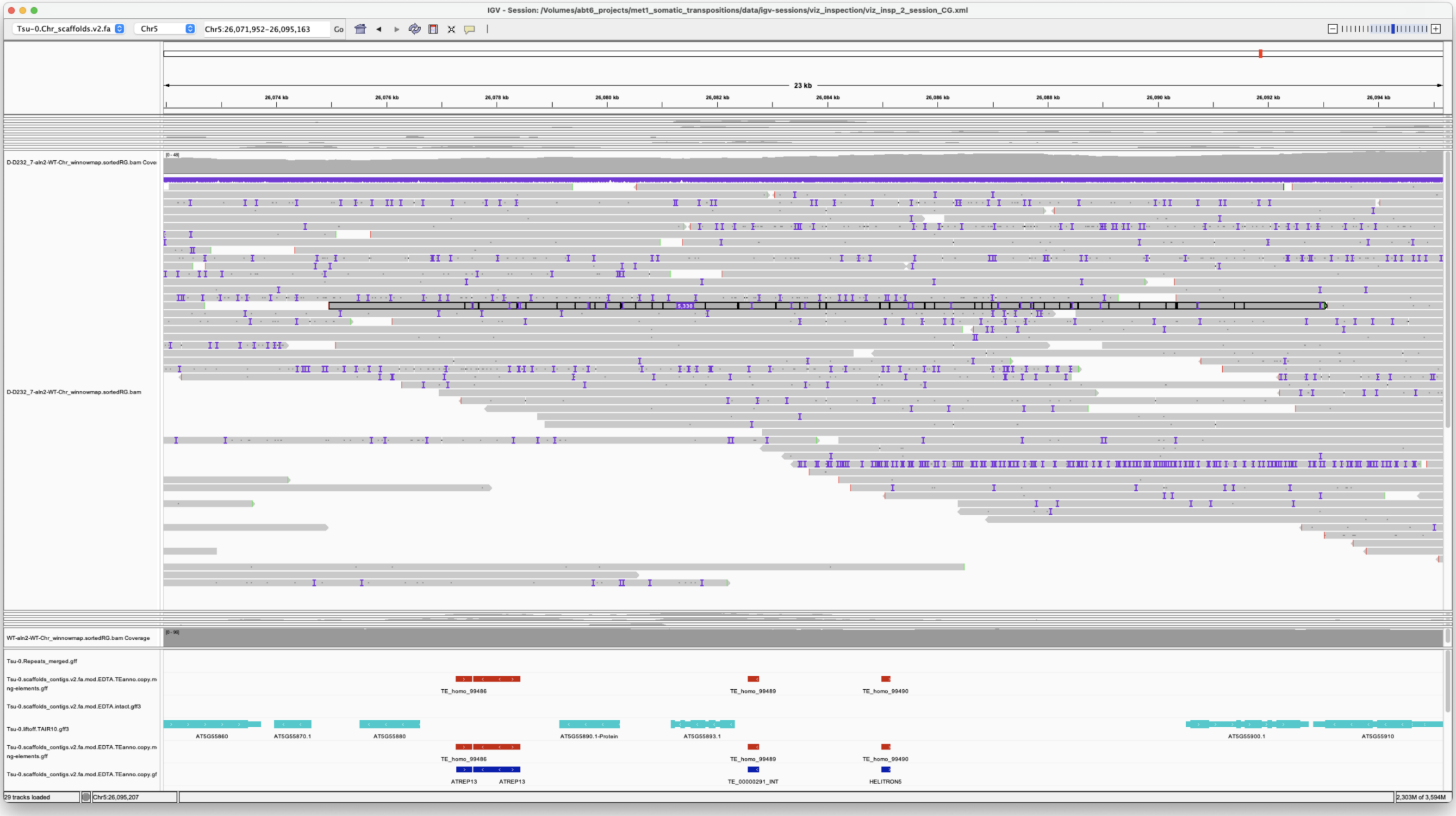

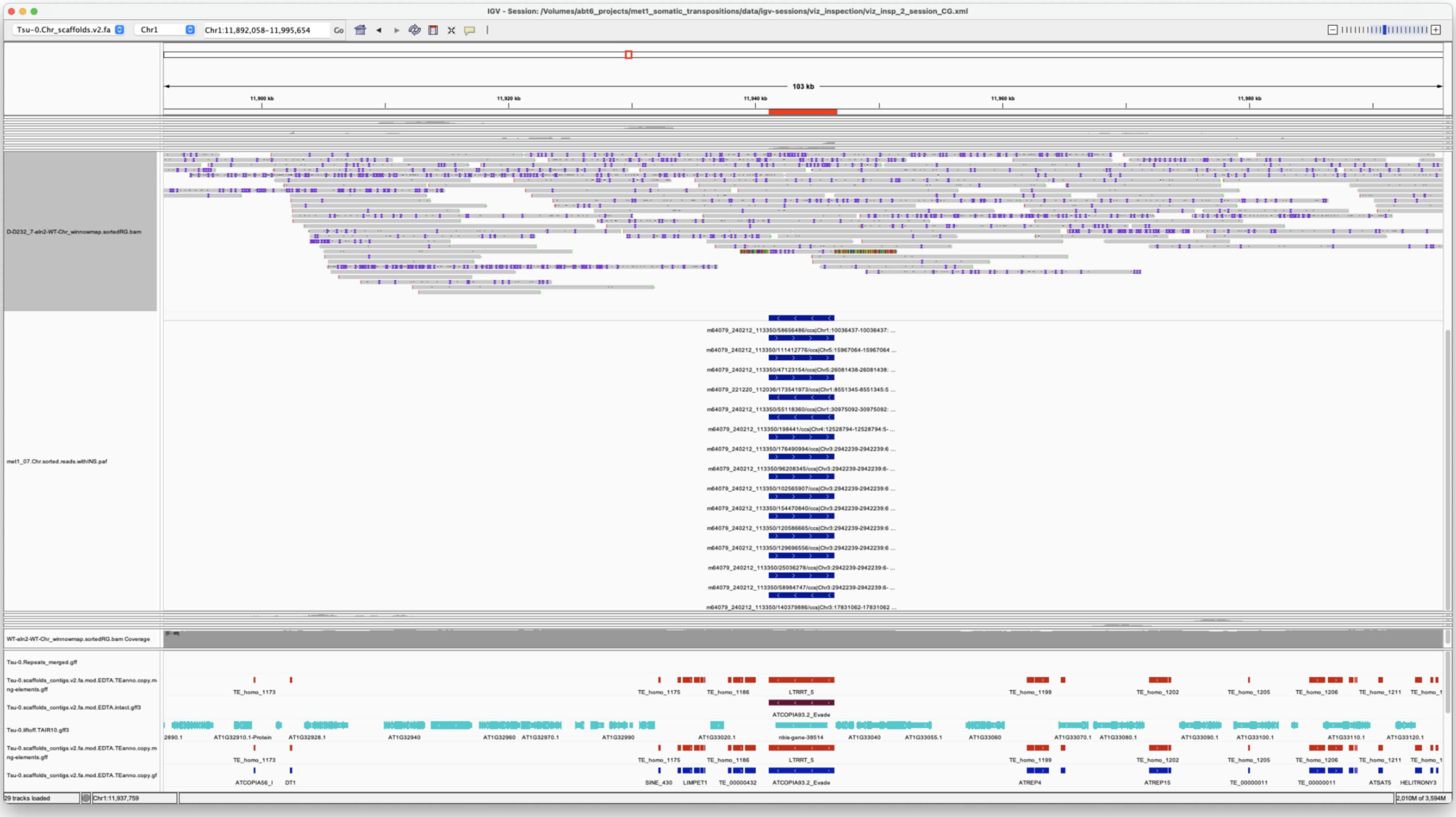

Confirmed

> Chr5 26888903 26888905 m64079_221220_112036/28248030/ccs**m64079_221220_112036/30476782/ccs Chr5 875413 876435 Chr5|875414|876433|ID=TE_MANUAL_02;Name=PAC;classification=DNA/DTC;sequence_ontology=MANUAL;identity=MANUAL;method=MANUAL;ID=TE_MANUAL_02;sequence_ontology=MANUAL**Chr5|875414|876433|ID=TE_MANUAL_02;Name=PAC;classification=DNA/DTC;sequence_ontology=MANUAL;identity=MANUAL;method=MANUAL;ID=TE_MANUAL_02;sequence_ontology=MANUAL met1_07

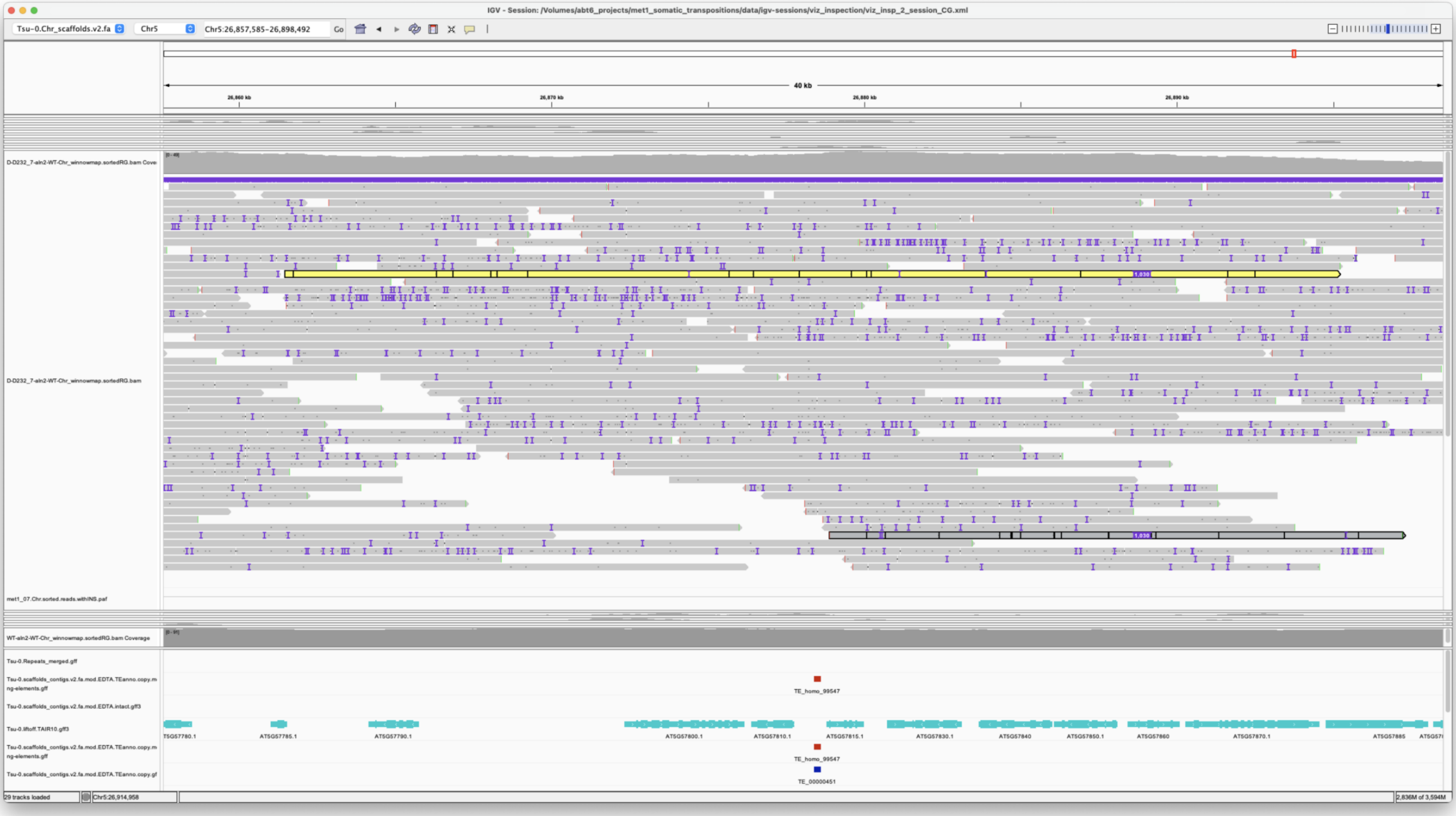

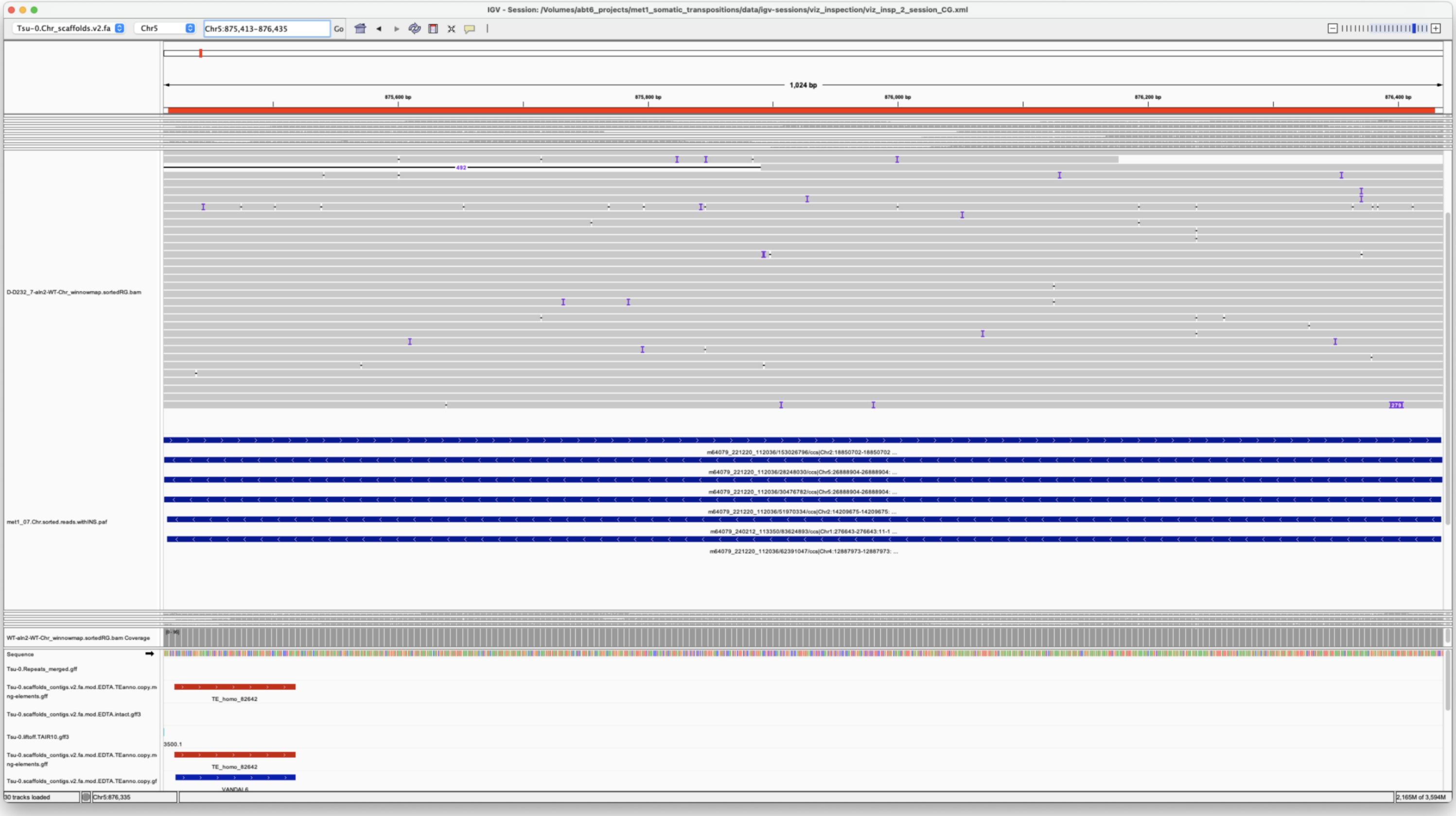

Beautiful

Confirmed

---

met1_08

> Chr2 14533452 14533452 m64079_221220_112036/101452109/ccs Chr1 11941106 11946441
> Chr1|11941106|11946435|ID=LTRRT_5;Name=ATCOPIA93.2_Evade;Classification=LTR/Copia;Sequence_ontology=SO:0002264;ltr_identity=1.0000;Method=structural;motif=TACA;tsd=ATATG met1_08

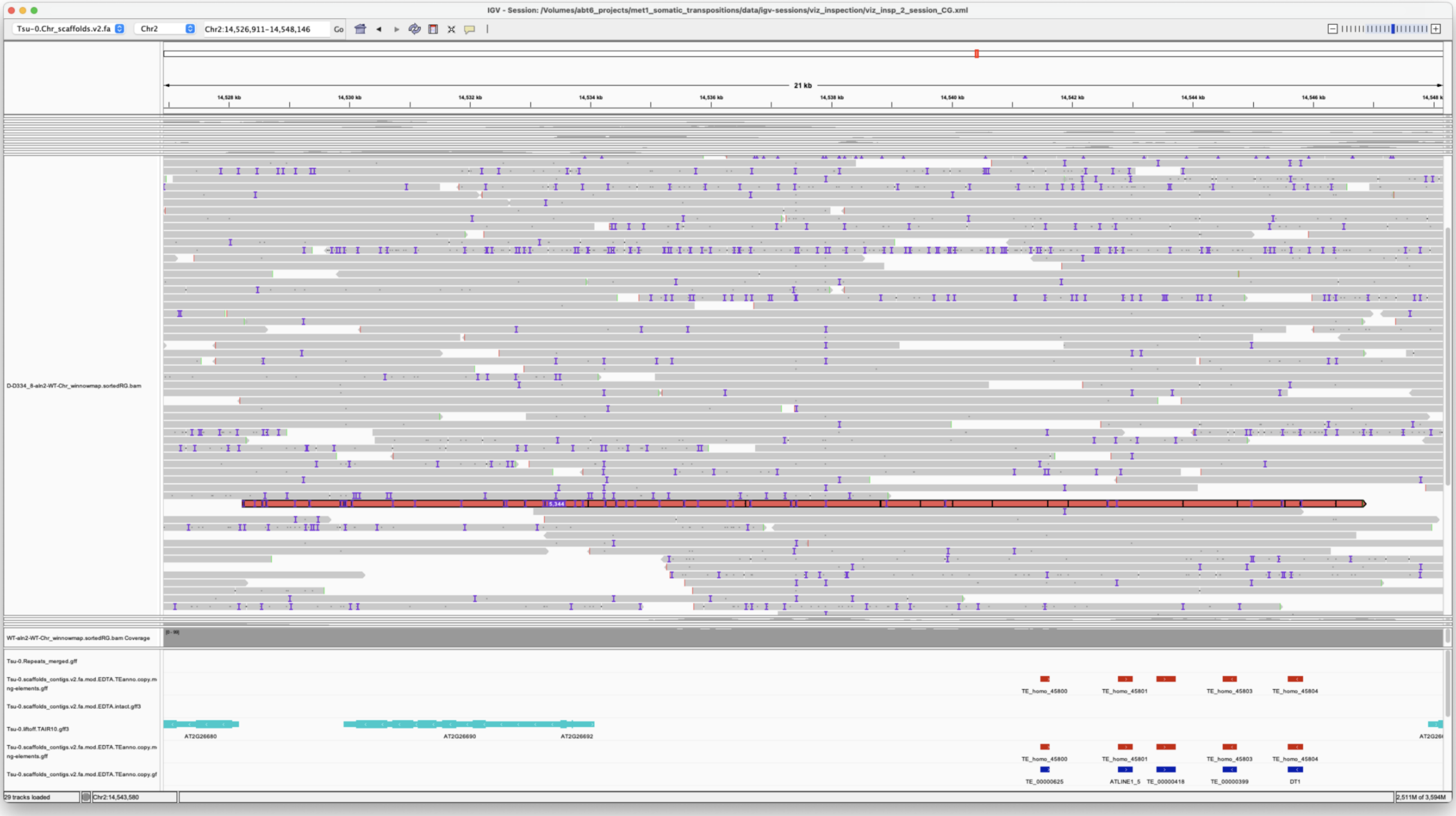

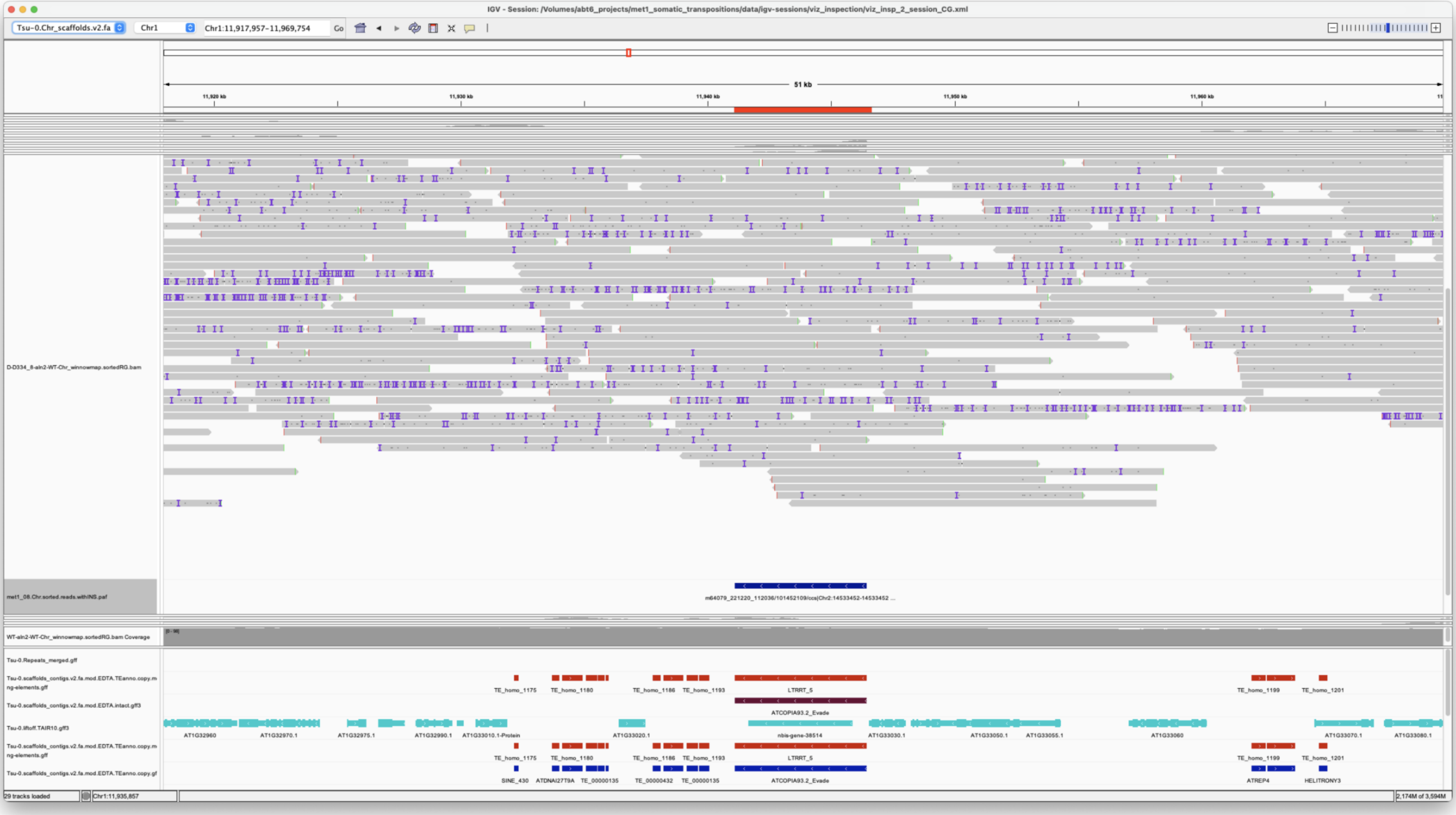

Confirmed

Chr3 6900522 6900522 m64079_221220_112036/152111366/ccs Chr5 875413 876434 Chr5|875414|876433|ID=TE_MANUAL_02;Name=PAC;classification=DNA/DTC;sequence_ontology=MANUAL;identity=MANUAL;method=MANUAL;ID=TE_MANUAL_02;sequence_ontology=MANUAL met1_08

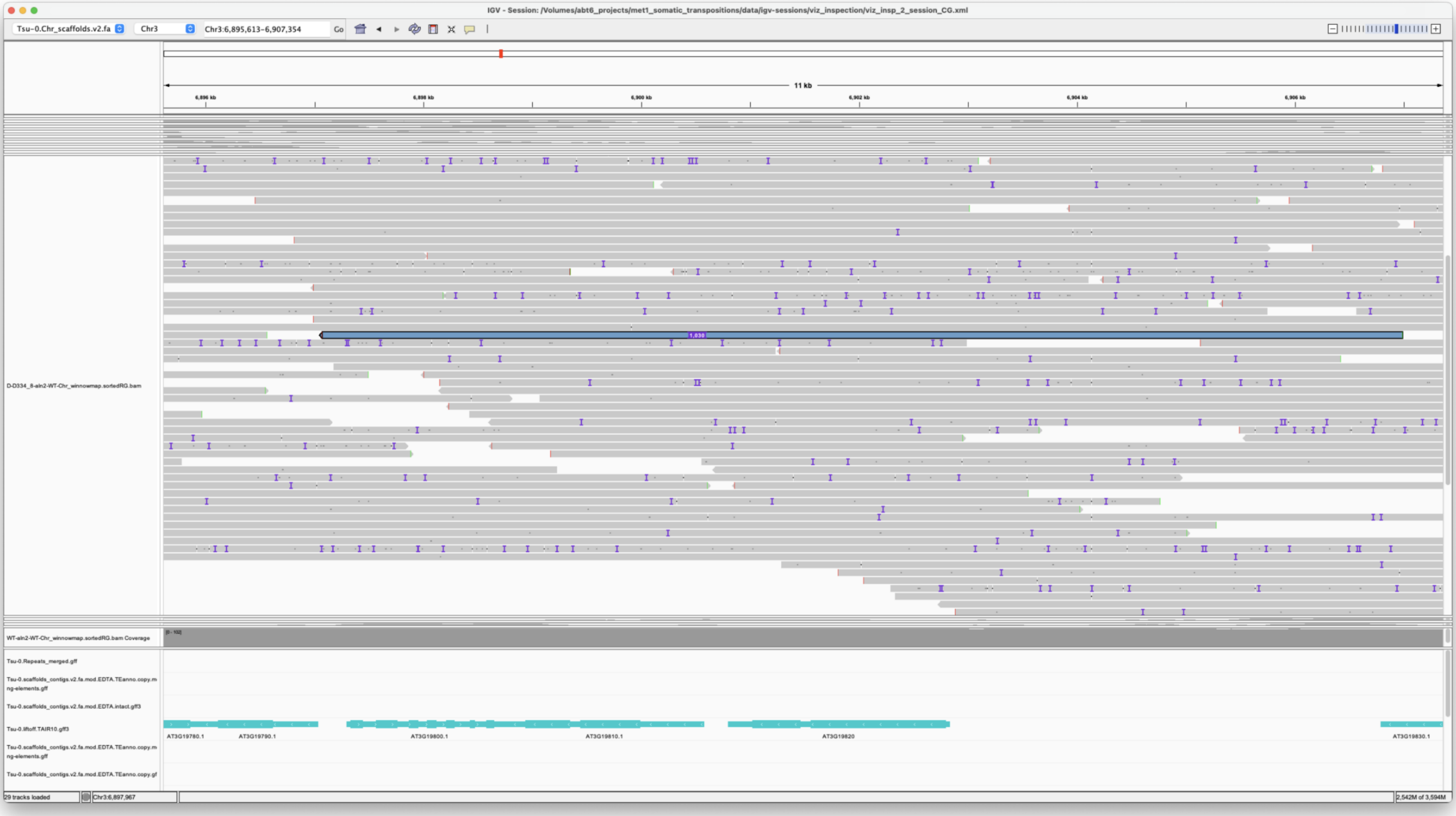

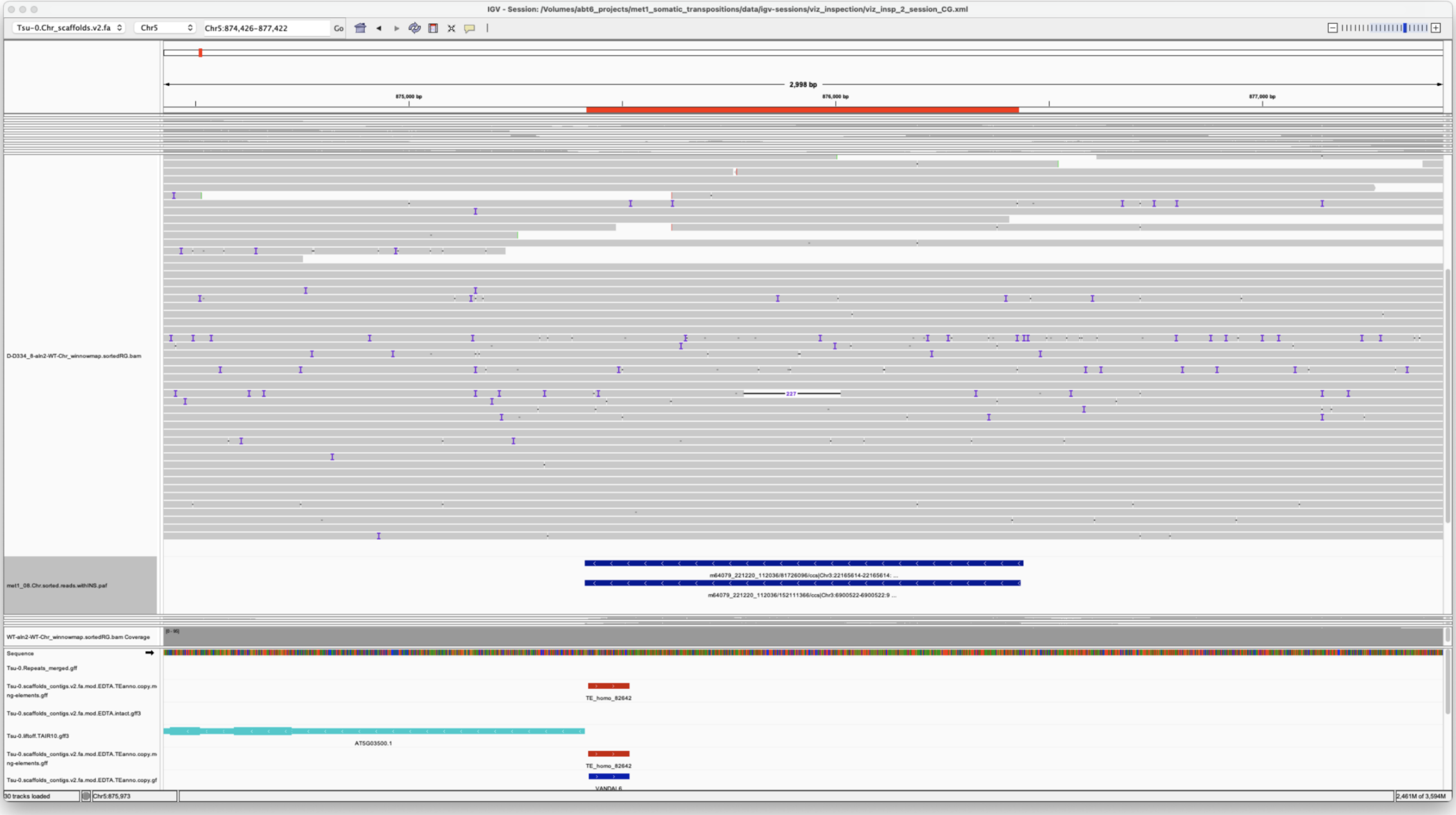

Confirmed

Chr3 22165614 22165614 m64079_221220_112036/81726096/ccs Chr5 875413 876441
Chr5|875414|876433|ID=TE_MANUAL_02;Name=PAC;classification=DNA/DTC;sequence_ontology=MANUAL;identity=MANUAL;method=MANUAL;ID=TE_MANUAL_02;sequence_ontology=MANUAL met1_08

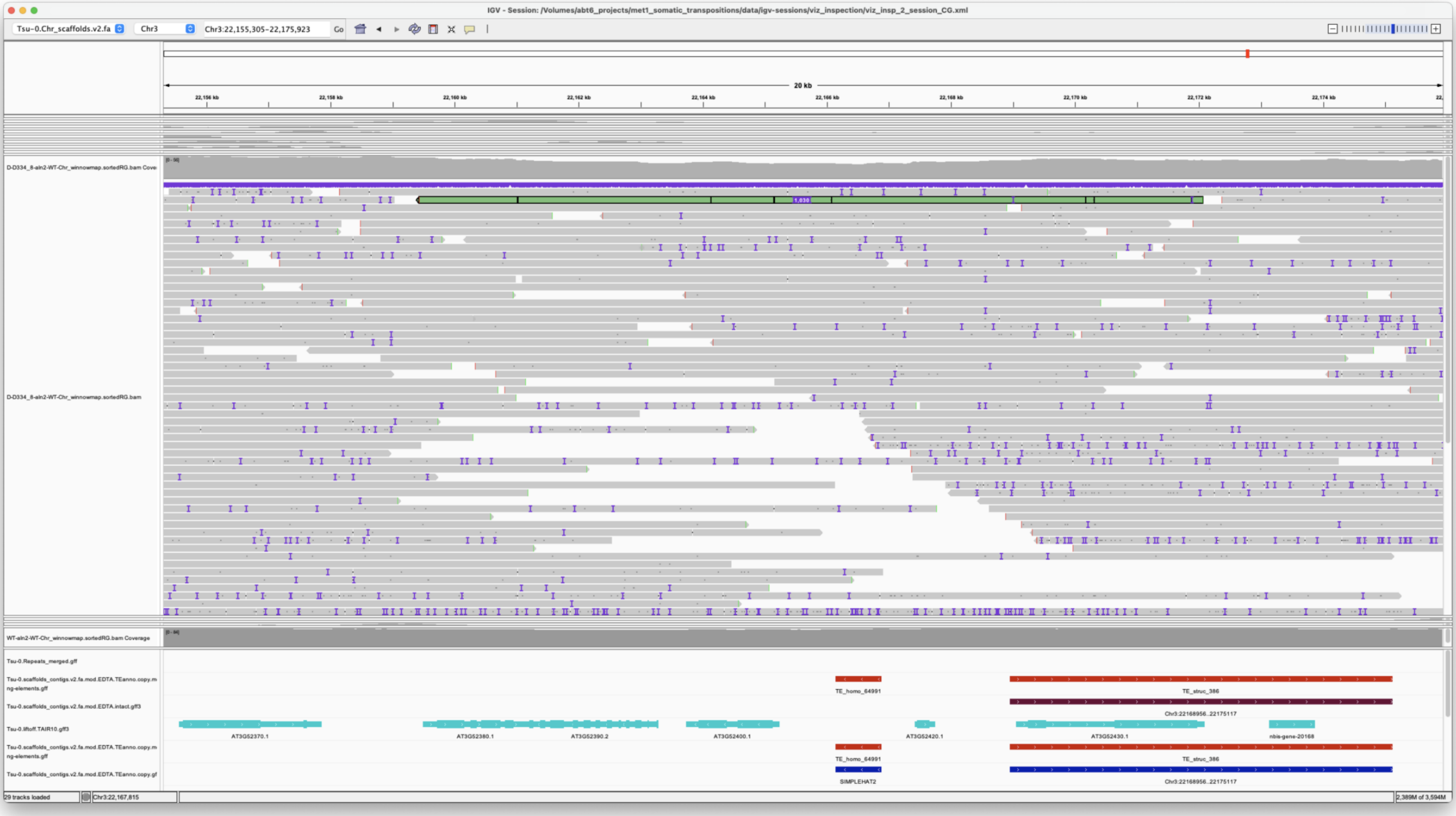

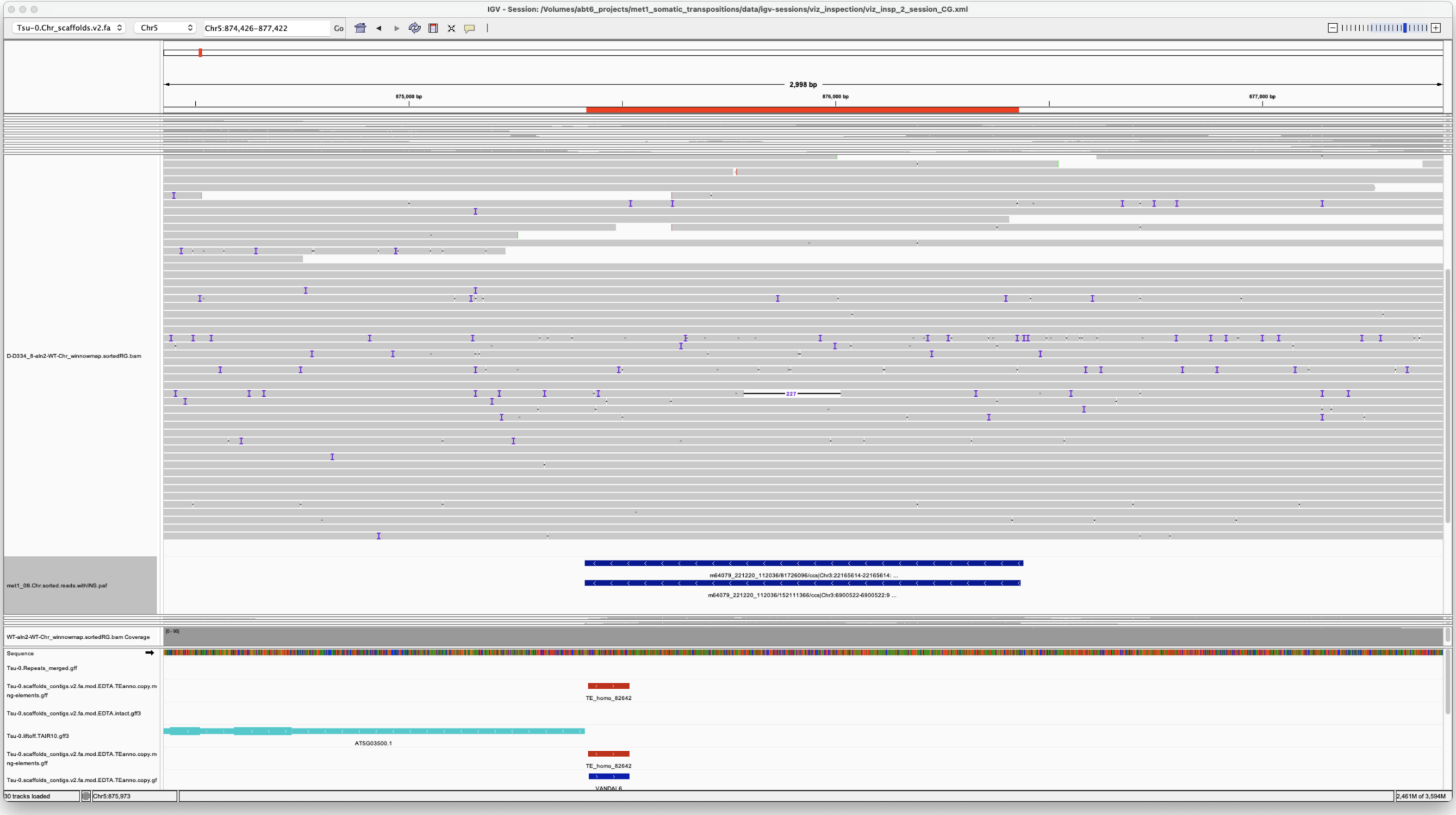

Confirmed

Chr5 18982271 18982271 m64079_221220_112036/93391083/ccs Chr5 19152825 19160826 Chr5|19152829|19160825|ID=TE_homo_95640;Name=VANDAL21;classification=DNA/Mutator;sequence_ontology=SO:0002280;identity=0.976;method=homology;ID=TE_homo_98501;sequence_ontology=SO:0002280|ID=TE_homo_95641;Name=VANDAL21;classification=DNA/Mutator;sequence_ontology=SO:0002280;identity=0.966;method=homology;ID=TE_homo_98502;sequence_ontology=SO:0002280 met1_08

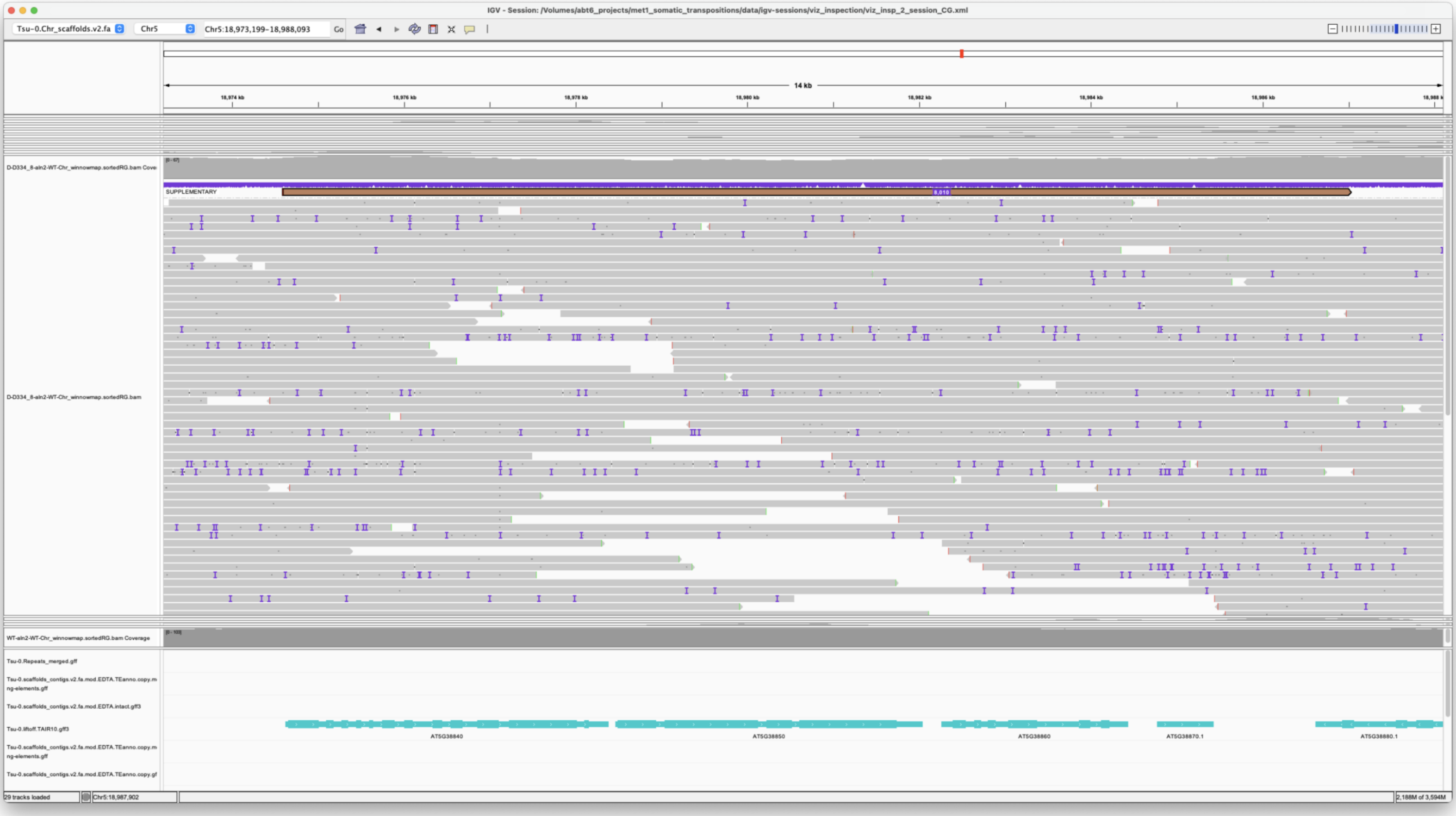

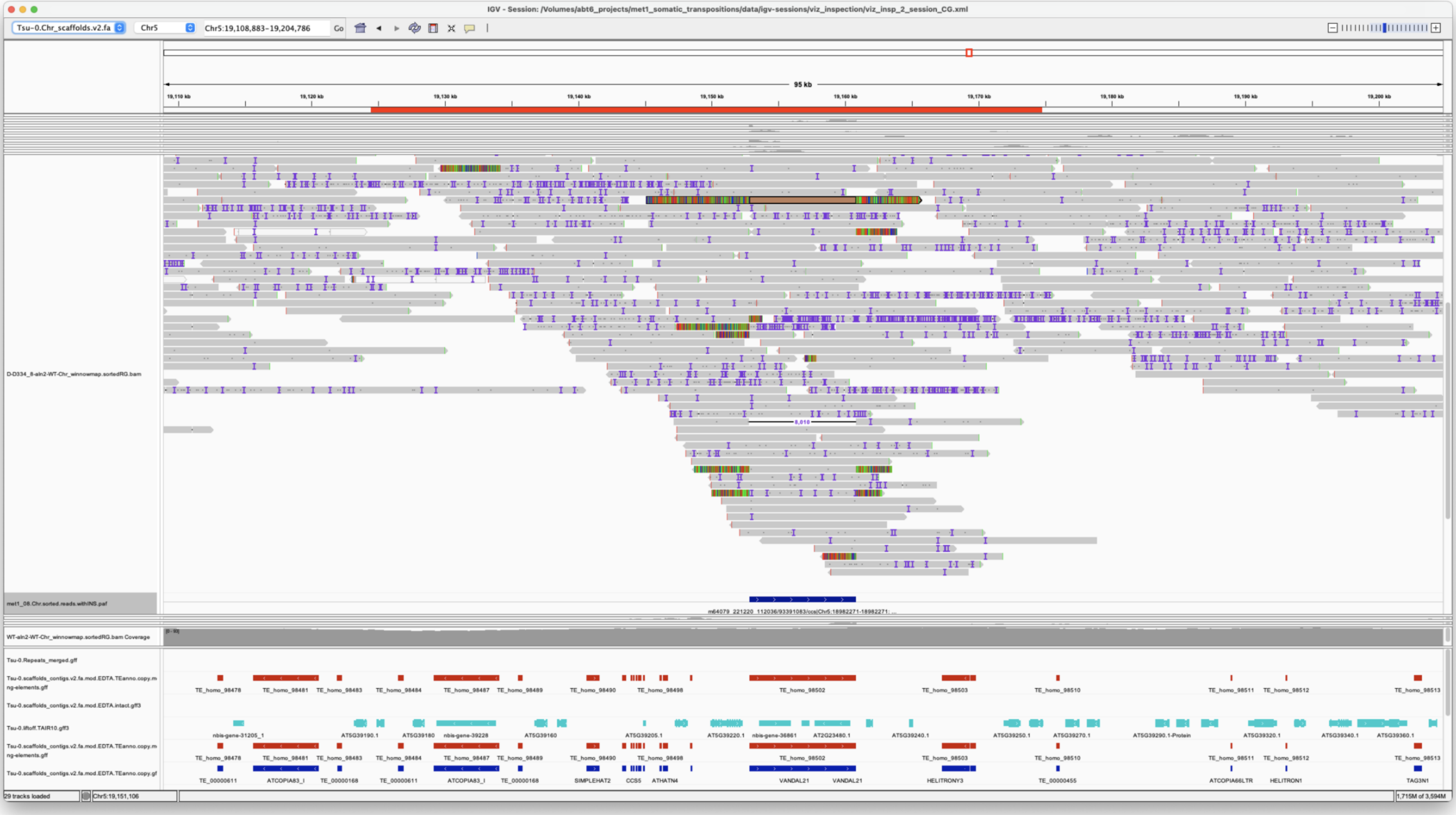

Confirmed

---

met1_09

> Chr1 7686553 7686553 m64079_221220_112036/113312127/ccs Chr5 875409 876434
> Chr5|875414|876433|ID=TE_MANUAL_02;Name=PAC;classification=DNA/DTC;sequence_ontology=MANUAL;identity=MANUAL;method=MANUAL;ID=TE_MANUAL_02;sequence_ontology=MANUAL met1_09

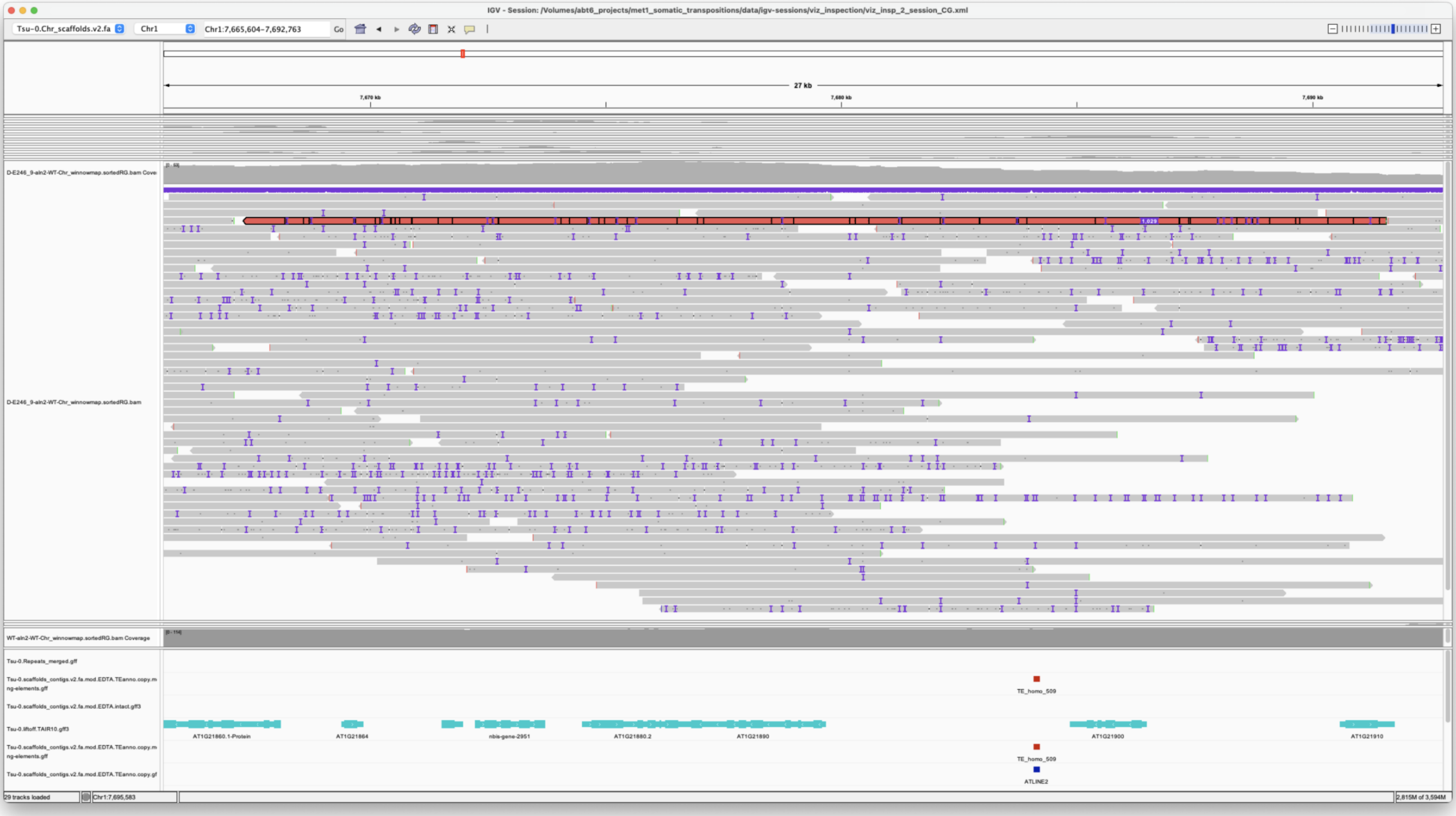

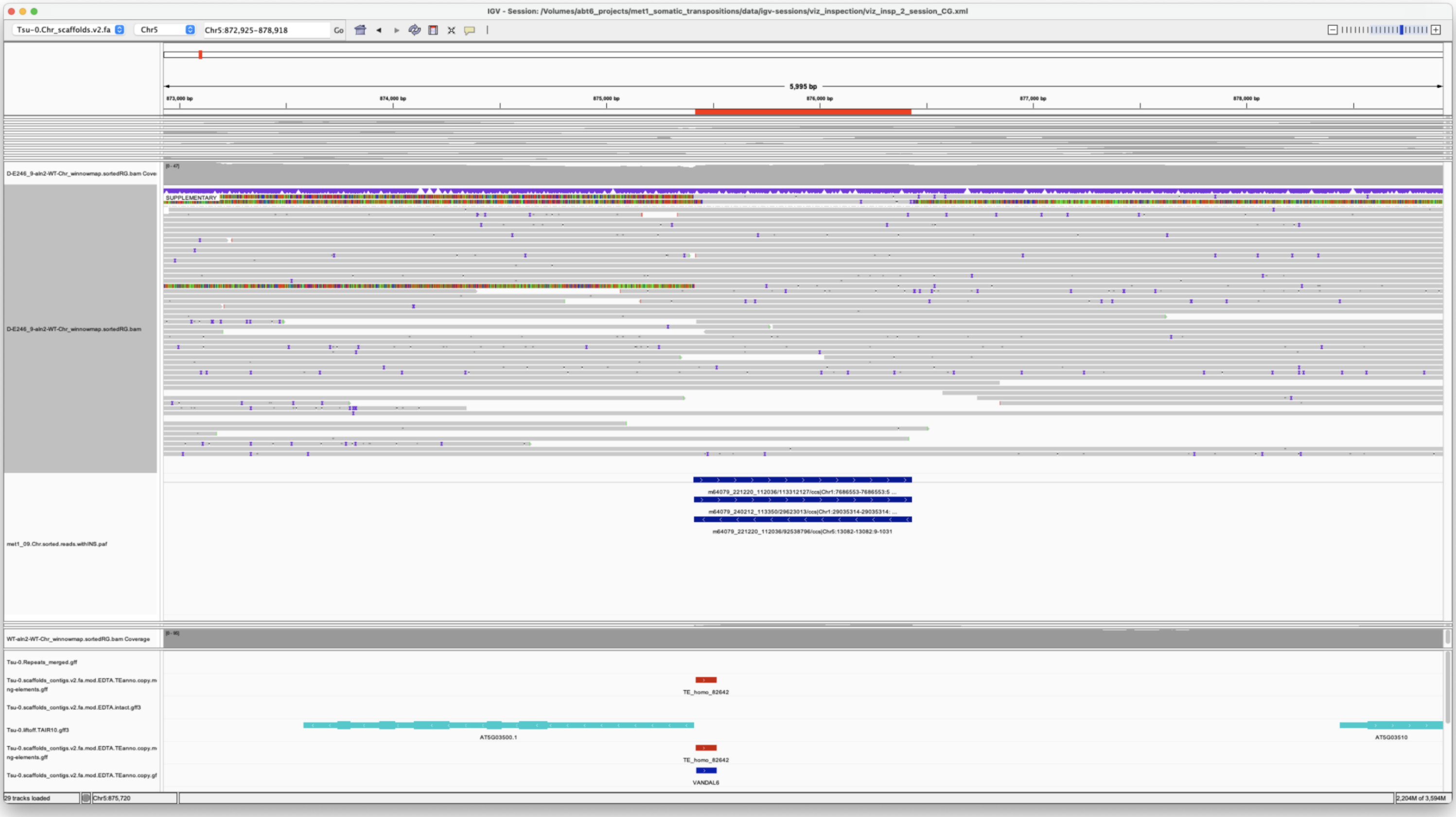

Confirmed

> Chr1 28846148 28846150 m64079_221220_112036/125503791/ccs Chr4 9036240 9040519 Chr4|9037080|9037164|ID=TE_homo_78576;Name=TE_00000569;classification=Unknown;sequence_ontology=SO:0001050;identity=0.841;method=homology;ID=TE_homo_80707;sequence_ontology=SO:0001050**Chr4|9037221|9037332|ID=TE_homo_78577;Name=TE_00000569;classification=Unknown;sequence_ontology=SO:0001050;identity=0.909;method=homology;ID=TE_homo_80708;sequence_ontology=SO:0001050**Chr4|9037391|9037501|ID=TE_homo_78578;Name=TE_00000569;classification=Unknown;sequence_ontology=SO:0001050;identity=0.927;method=homology;ID=TE_homo_80709;sequence_ontology=SO:0001050**Chr4|9037502|9038015|ID=TE_homo_78579;Name=VANDAL21;classification=DNA/Mutator;sequence_ontology=SO:0002280;identity=0.705;method=homology;ID=TE_homo_80710;sequence_ontology=SO:0002280**Chr4|9038028|9039749|ID=TE_homo_78580;Name=VANDAL21;classification=DNA/MULE-MuDR;sequence_ontology=SO:0002280;identity=0.795;method=homology;ID=TE_homo_80711;sequence_ontology=SO:0002280**Chr4|9039779|9040118|ID=TE_homo_78581;Name=VANDAL21;classification=DNA/MULE-MuDR;sequence_ontology=SO:0002280;identity=0.735;method=homology;ID=TE_homo_80712;sequence_ontology=SO:0002280**Chr4|9040114|9040303|ID=TE_homo_78582;Name=TE_00000579;classification=Unknown;sequence_ontology=SO:0001050;identity=0.937;method=homology;ID=TE_homo_80713;sequence_ontology=SO:0001050 met1_09

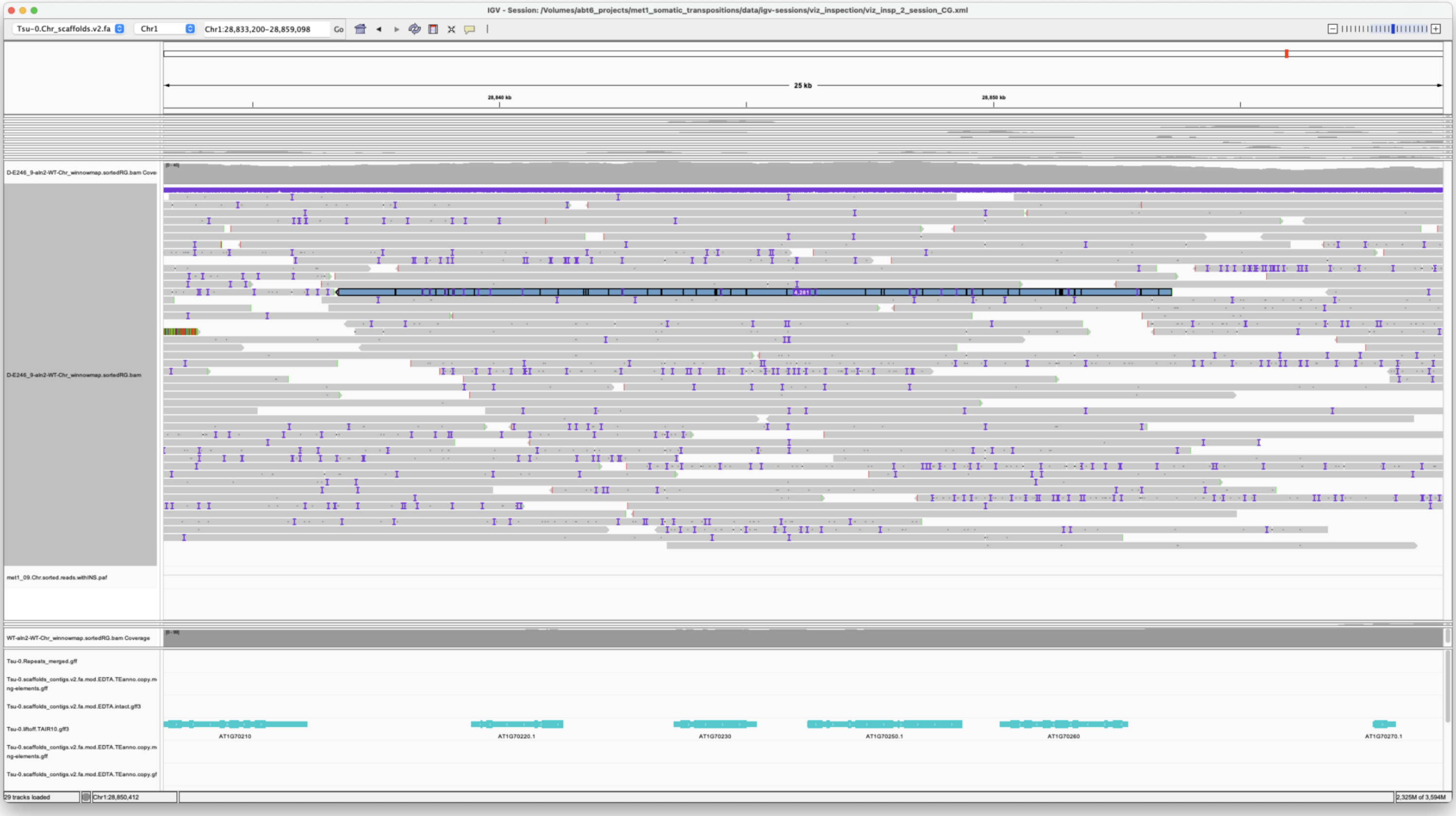

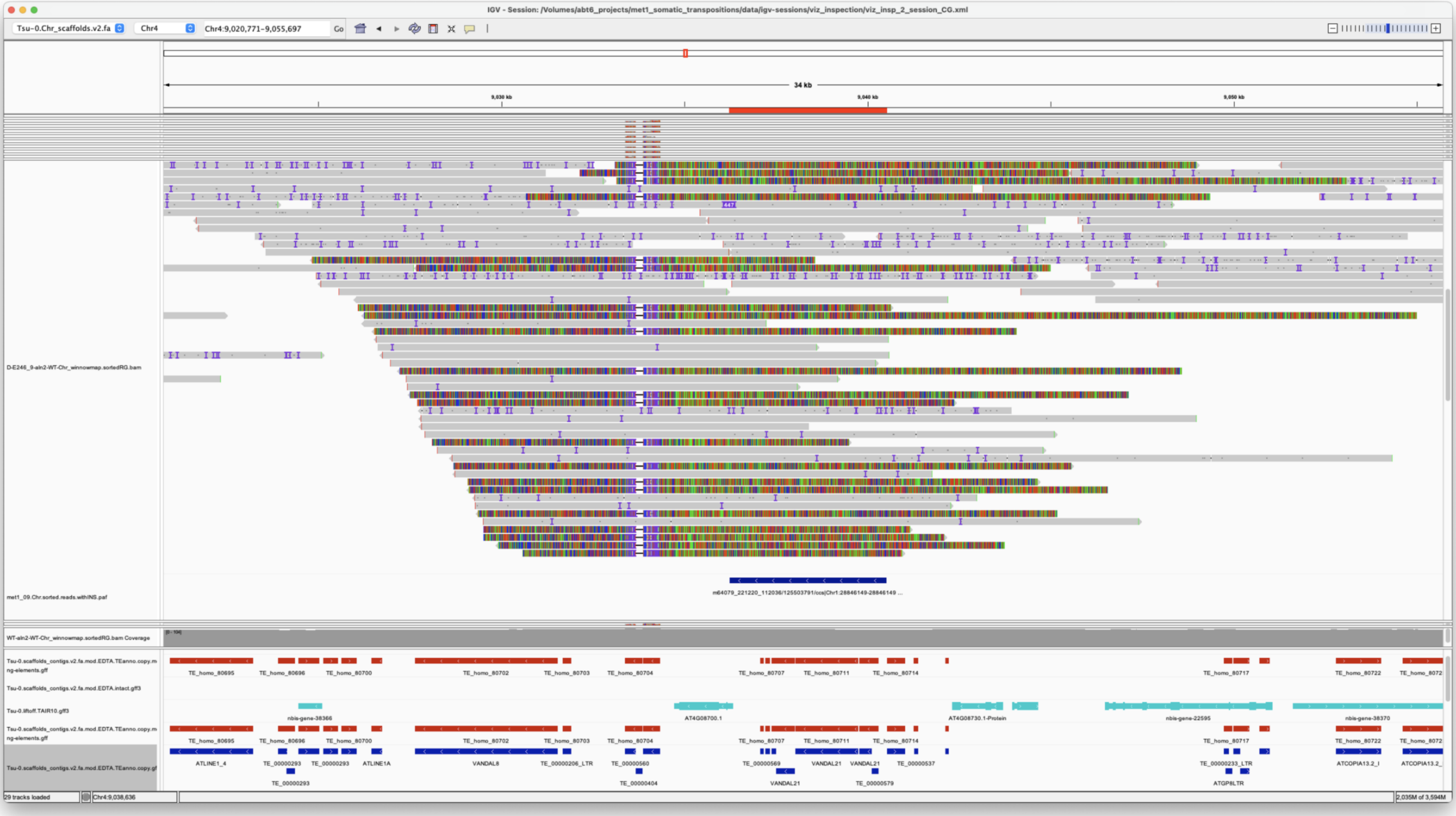

Presence of a difficult region nearby makes difficult to identify transpositions at this locus.
Also this VANDAL21 seems to be incorrectly annotated.

```
!!!To be manually corrected in main annotation.
```

But the presence of an insertion in central configuration is good evidence of mobilization of this element.
Also dotplot shows TSD

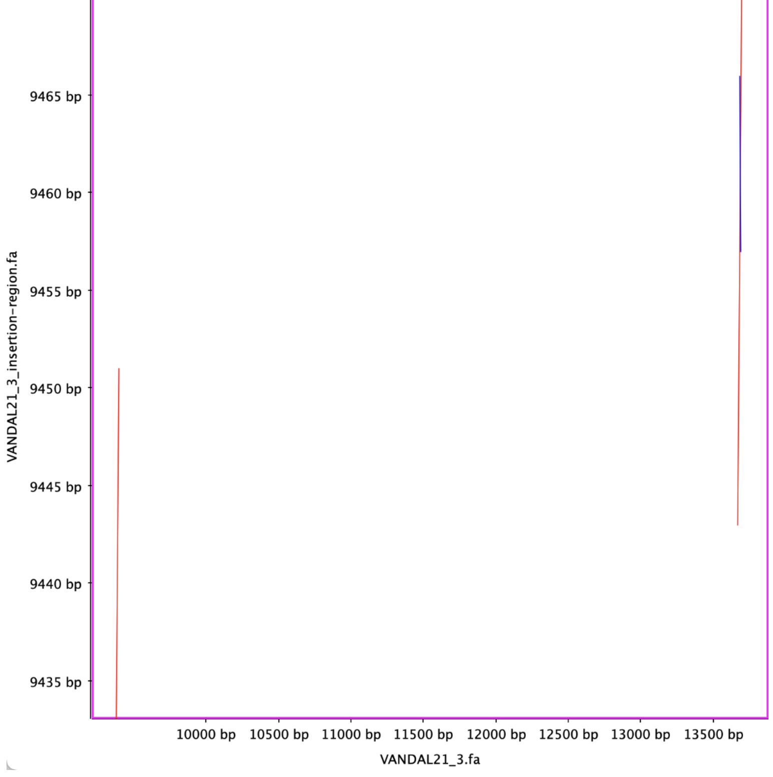

Confirmed

Chr1 29035314 29035314 m64079_240212_113350/29623013/ccs Chr5 875413 876434 Chr5|875414|876433|ID=TE_MANUAL_02;Name=PAC;classification=DNA/DTC;sequence_ontology=MANUAL;identity=MANUAL;method=MANUAL;ID=TE_MANUAL_02;sequence_ontology=MANUAL met1_09

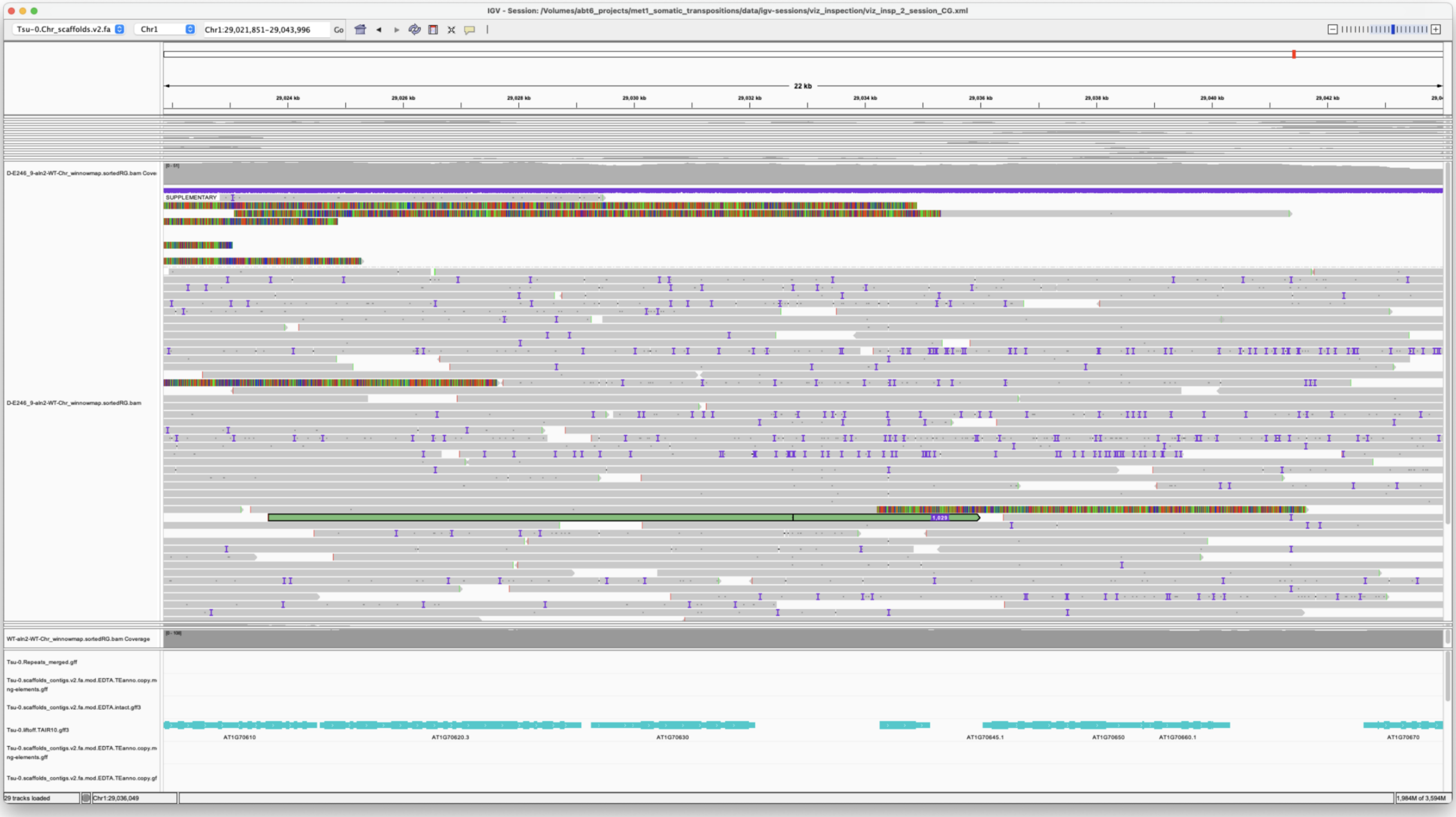

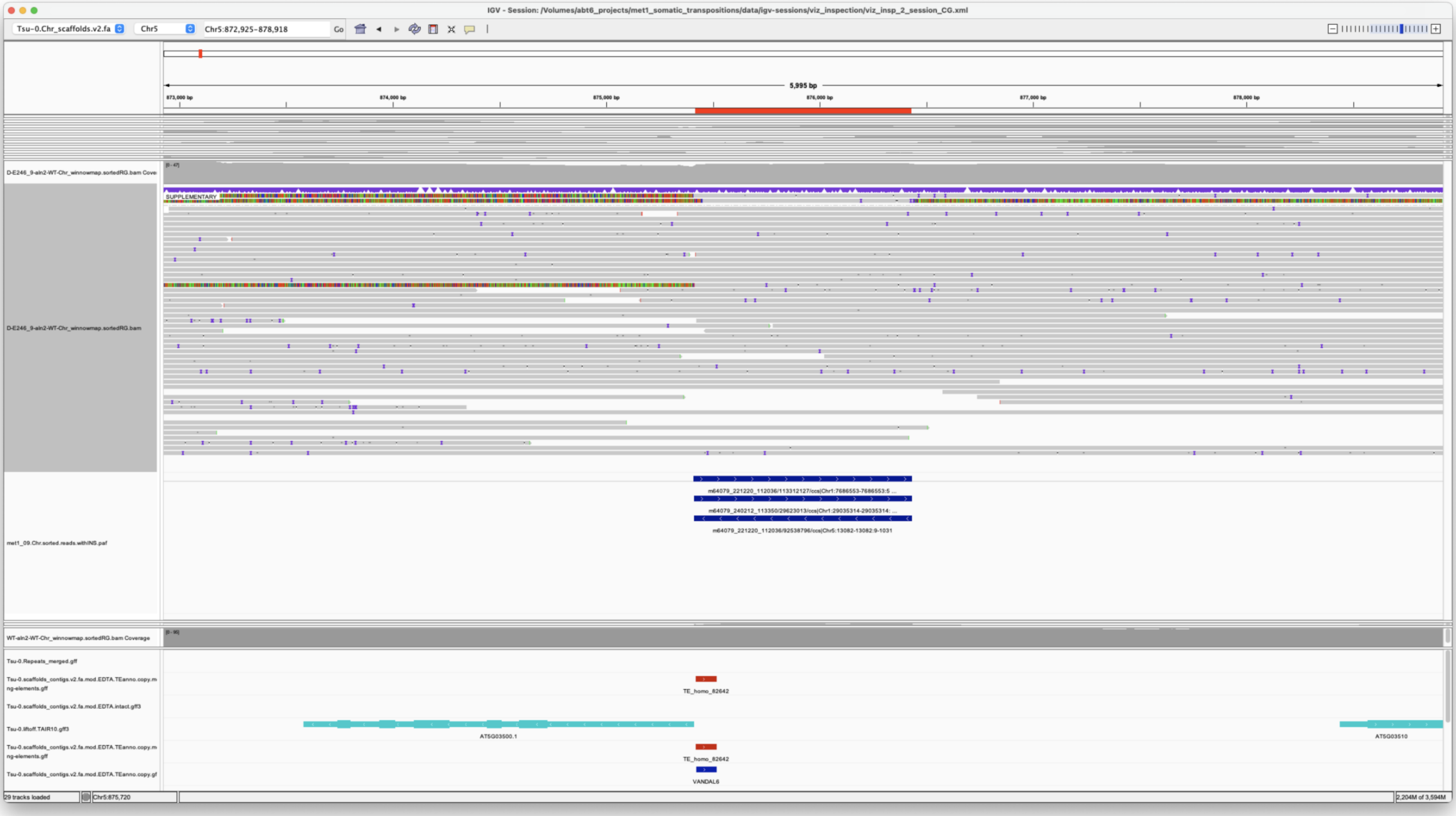

Confirmed

Chr5 13082 13082 m64079_221220_112036/92538796/ccs Chr5 875413 876434 Chr5|875414|876433|ID=TE_MANUAL_02;Name=PAC;classification=DNA/DTC;sequence_ontology=MANUAL;identity=MANUAL;method=MANUAL;ID=TE_MANUAL_02;sequence_ontology=MANUAL met1_09

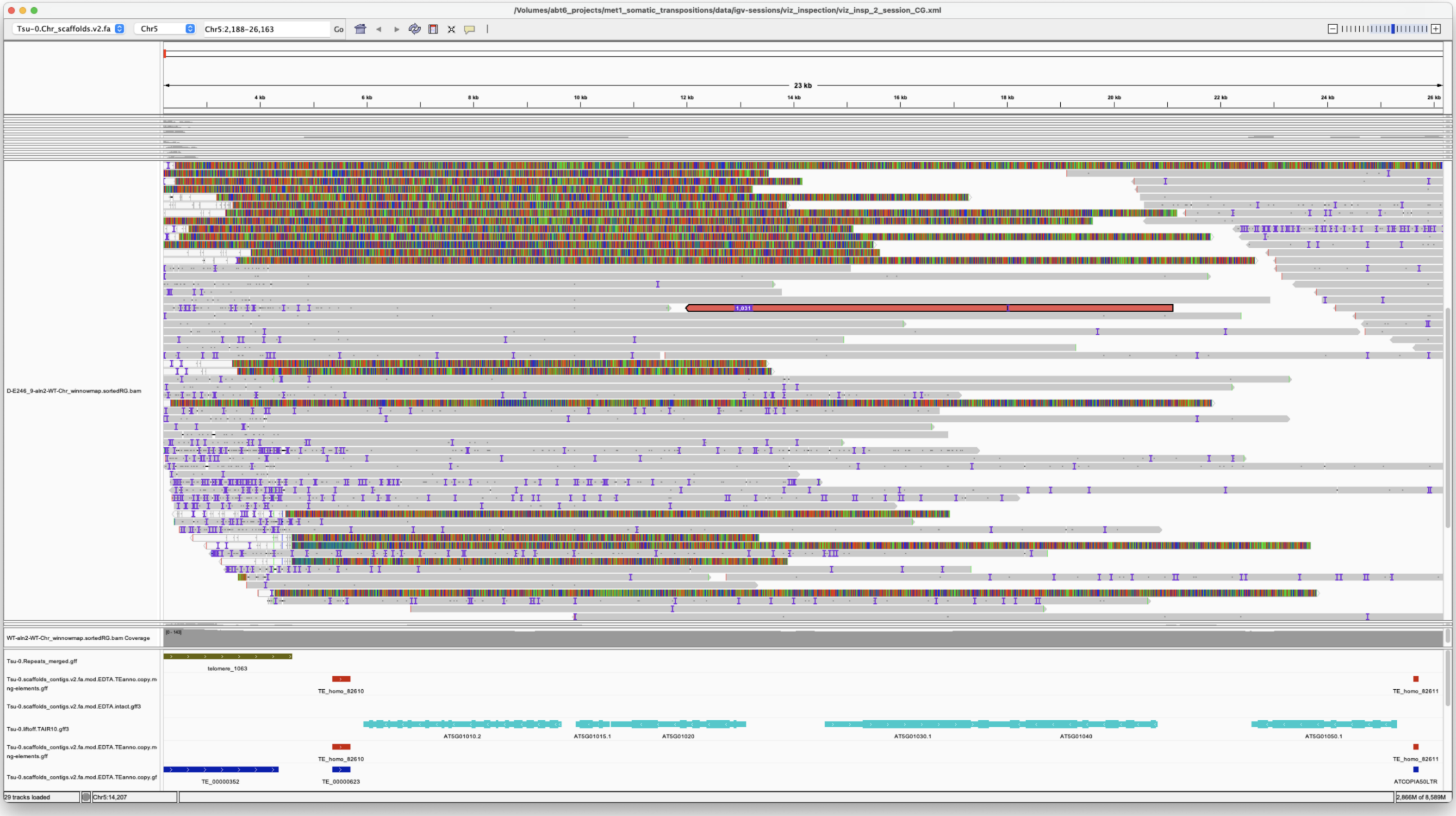

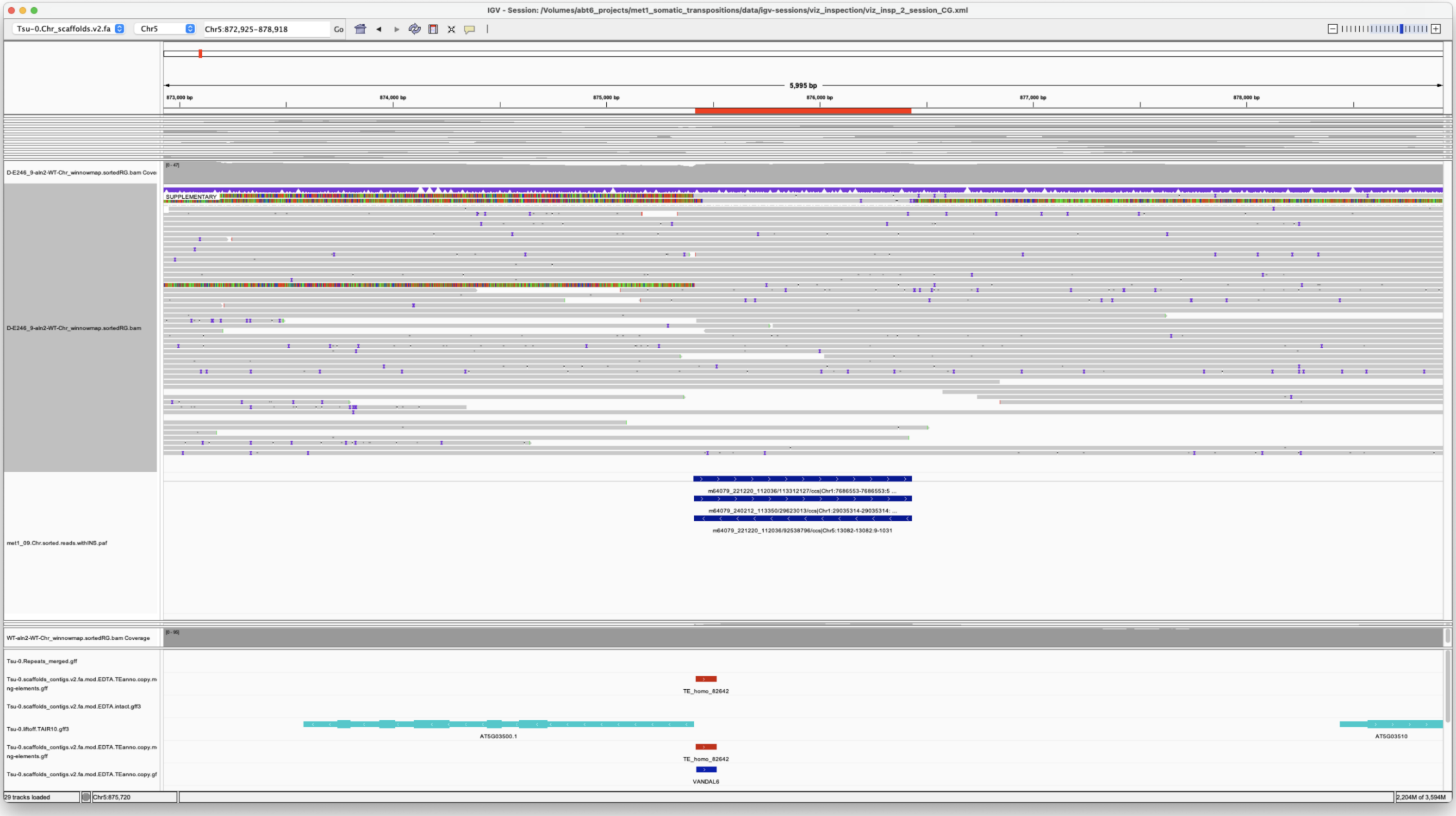

Very telomeric insertion

Confirmed

---

met1_10

> Chr1 6485187 6485187 m64079_240212_113350/152699302/ccs Chr5 875413 876434 Chr5|875414|876433|ID=TE_MANUAL_02;Name=PAC;classification=DNA/DTC;sequence_ontology=MANUAL;identity=MANUAL;method=MANUAL;ID=TE_MANUAL_02;sequence_ontology=MANUAL met1_10

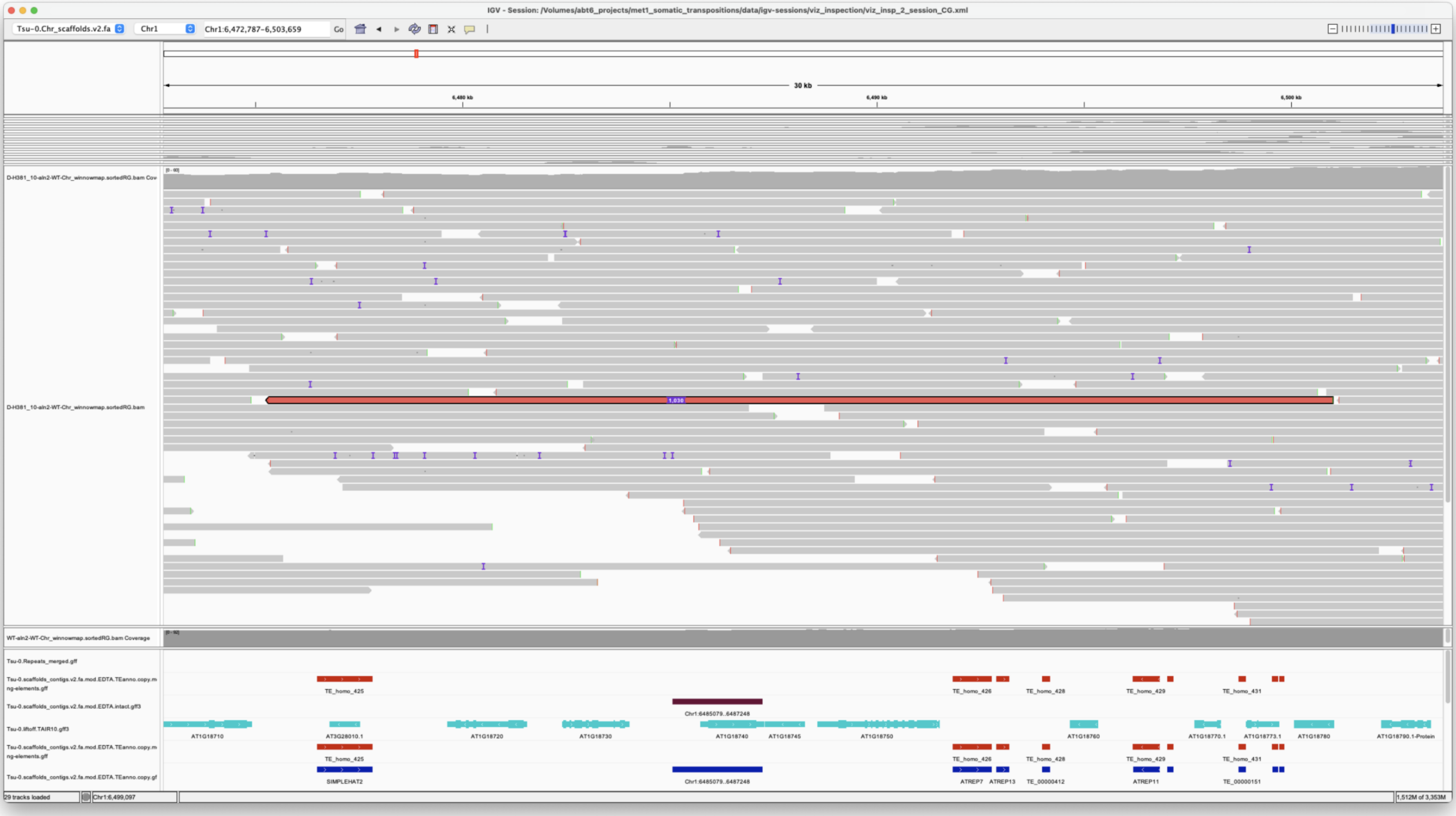

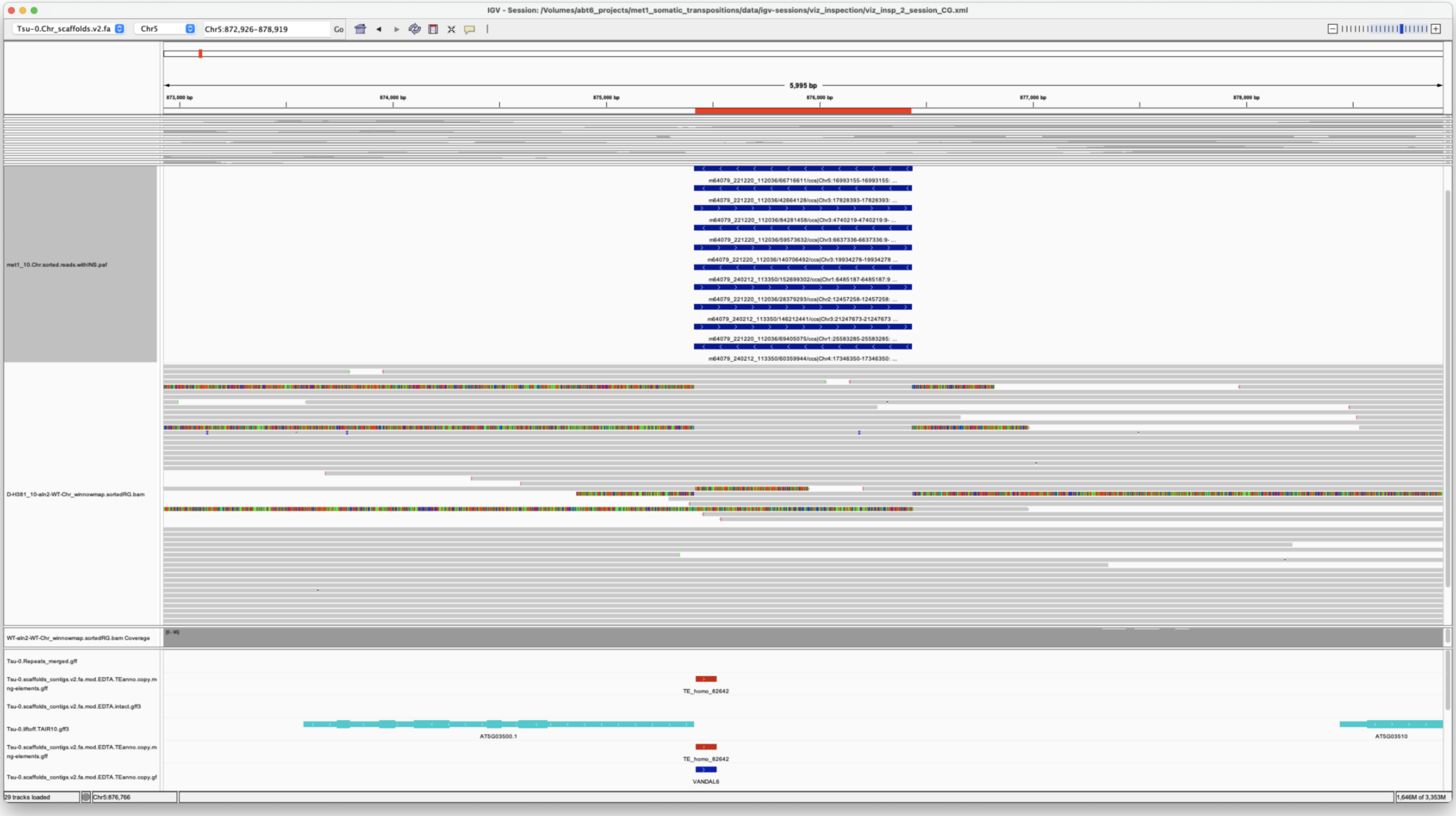

Confirmed

> Chr1 9954546 9954546 m64079_221220_112036/69664872/ccs Chr5 21419693 21425021 Chr5|21419693|21425021|ID=LTRRT_299;Name=ATCOPIA93.2_Evade;Classification=LTR/Copia;Sequence_ontology=SO:0002264;ltr_identity=1.0000;Method=structural;motif=TACA;tsd=GGACA met1_10

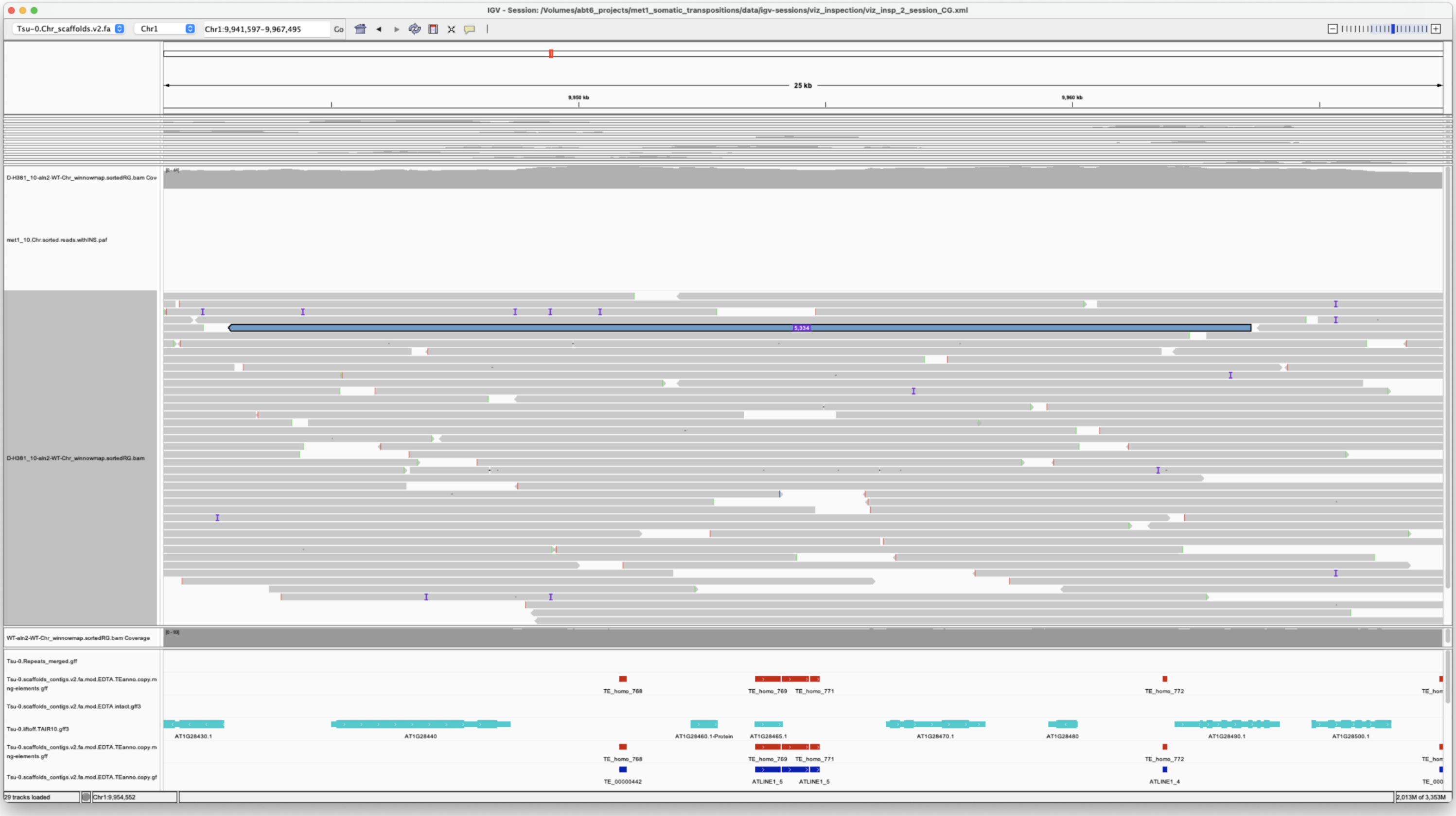

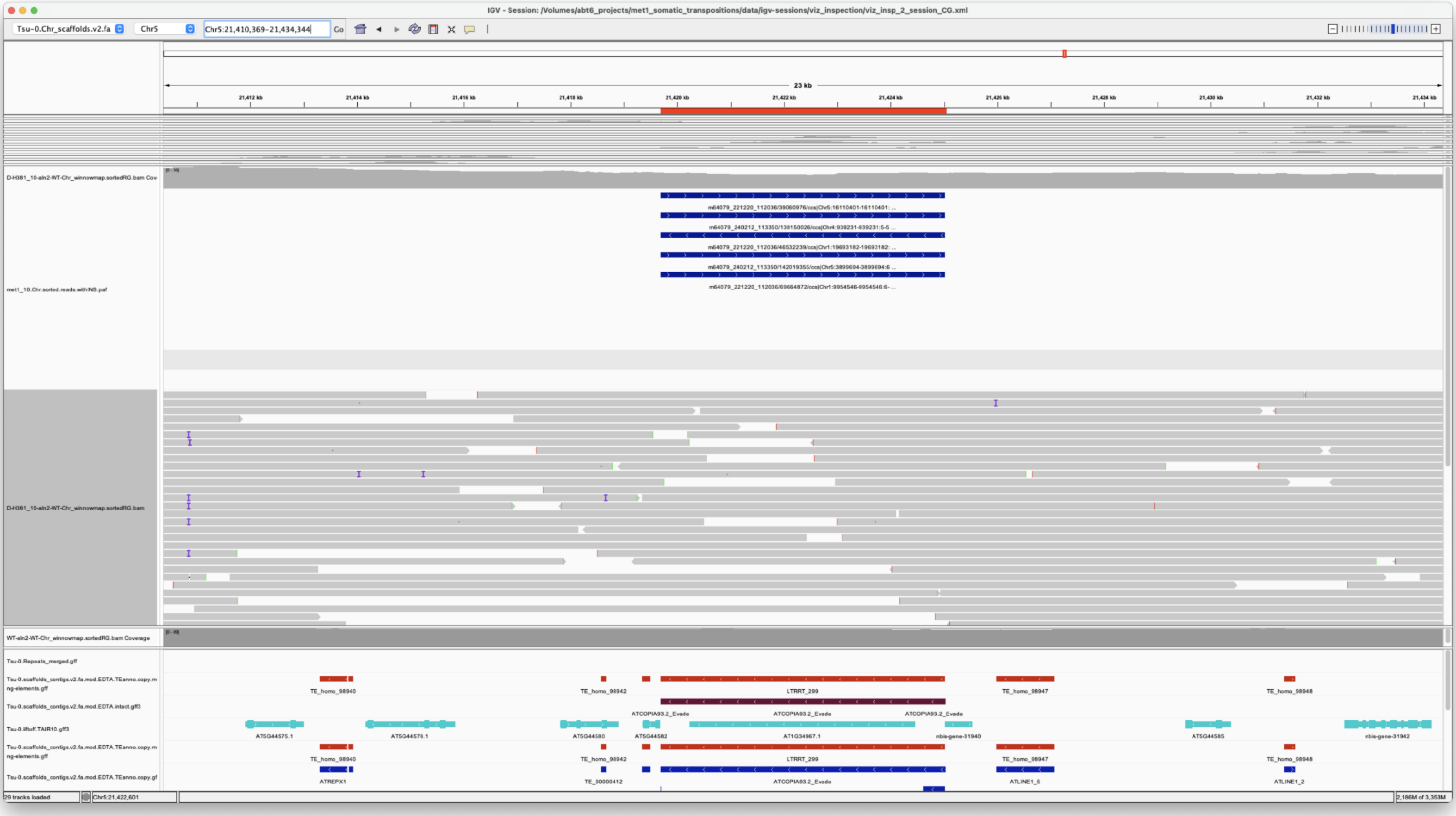

Confirmed

Chr1 19693182 19693182 m64079_221220_112036/46532239/ccs Chr5 21419693 21425022 Chr5|21419693|21425021|ID=LTRRT_299;Name=ATCOPIA93.2_Evade;Classification=LTR/Copia;Sequence_ontology=SO:0002264;ltr_identity=1.0000;Method=structural;motif=TACA;tsd=GGACA met1_10

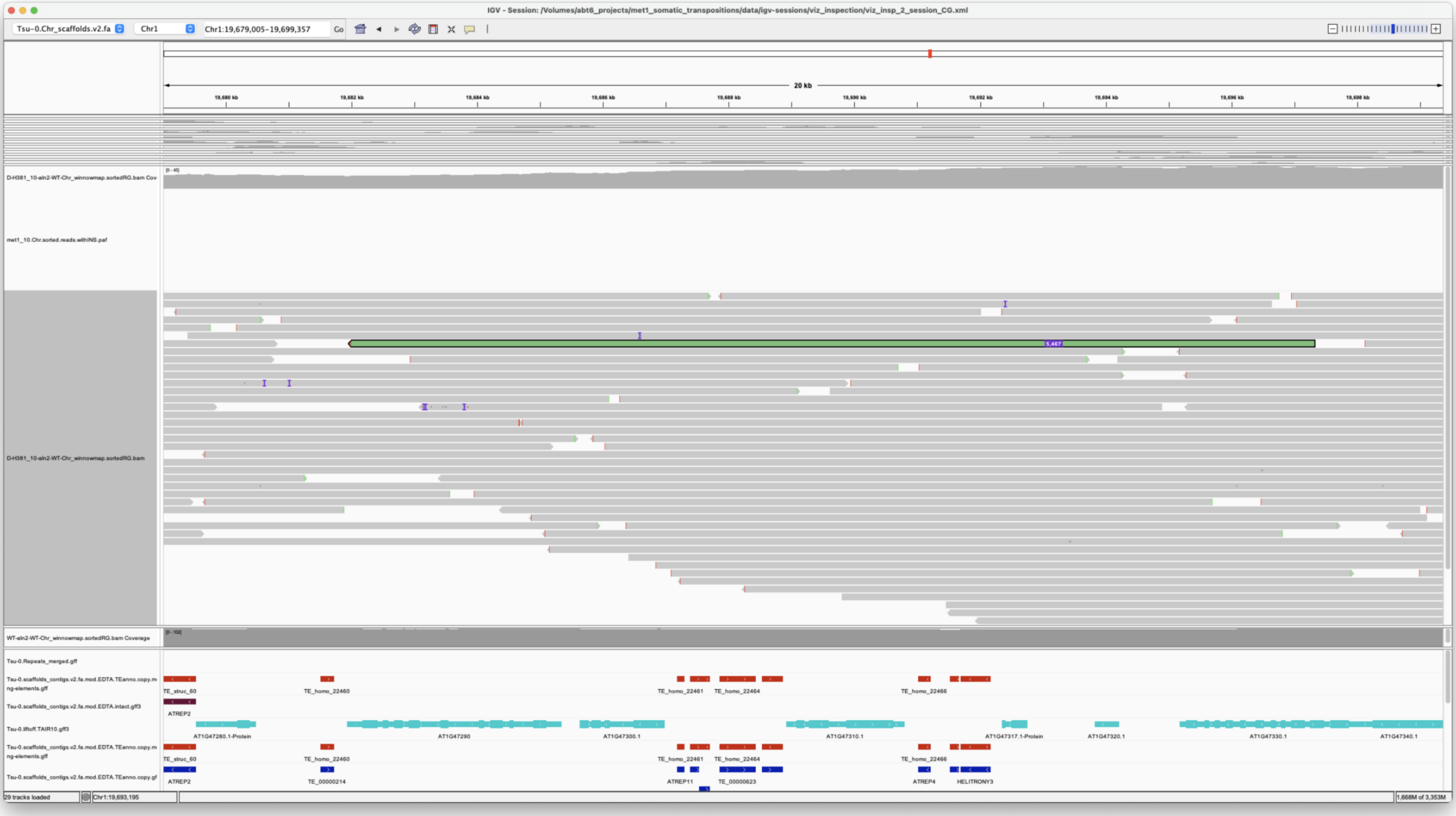

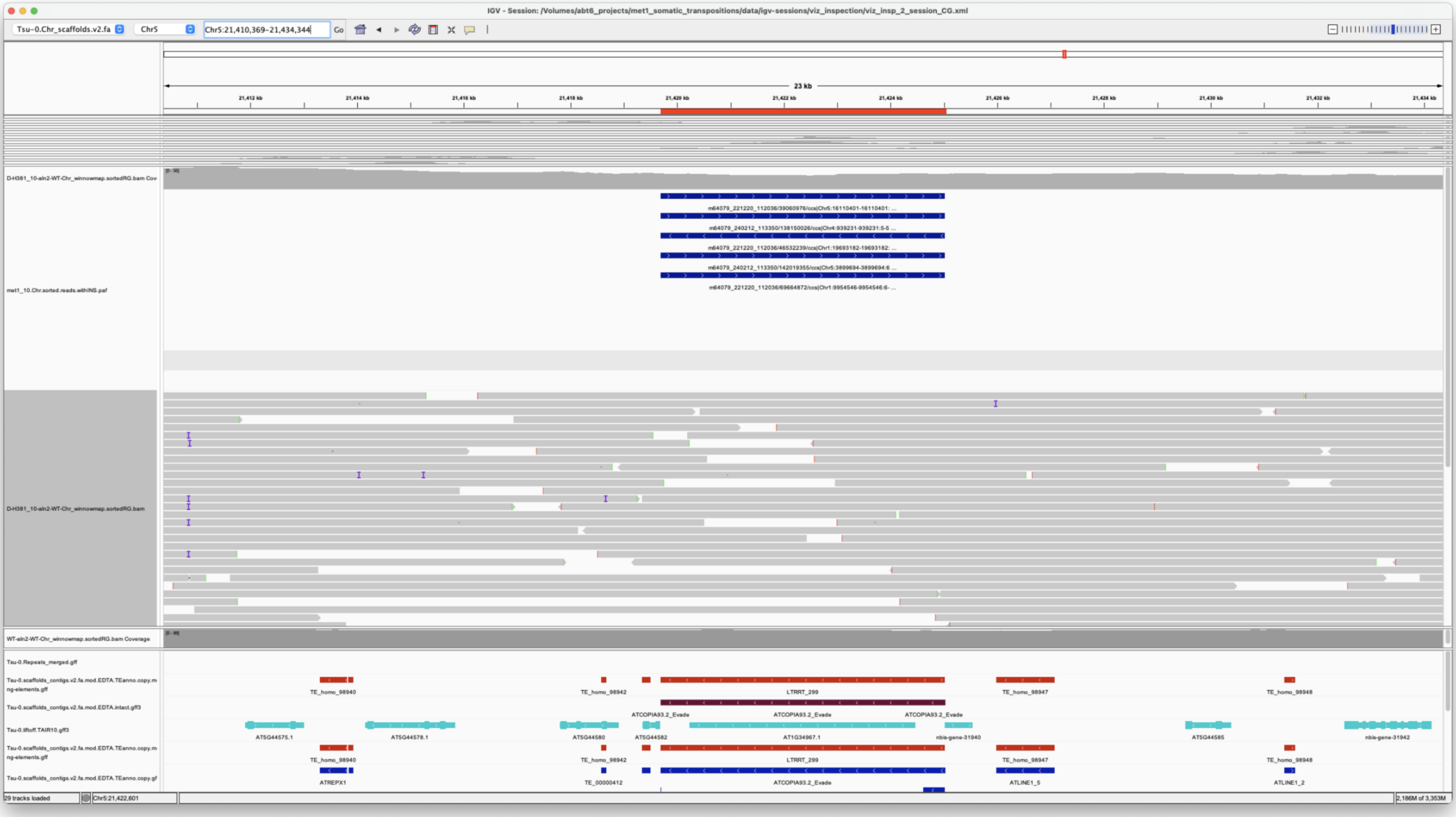

Confirmed

> Chr1 25583285 25583285 m64079_221220_112036/69405075/ccs Chr5 875413 876433 Chr5|875414|876433|ID=TE_MANUAL_02;Name=PAC;classification=DNA/DTC;sequence_ontology=MANUAL;identity=MANUAL;method=MANUAL;ID=TE_MANUAL_02;sequence_ontology=MANUAL met1_10

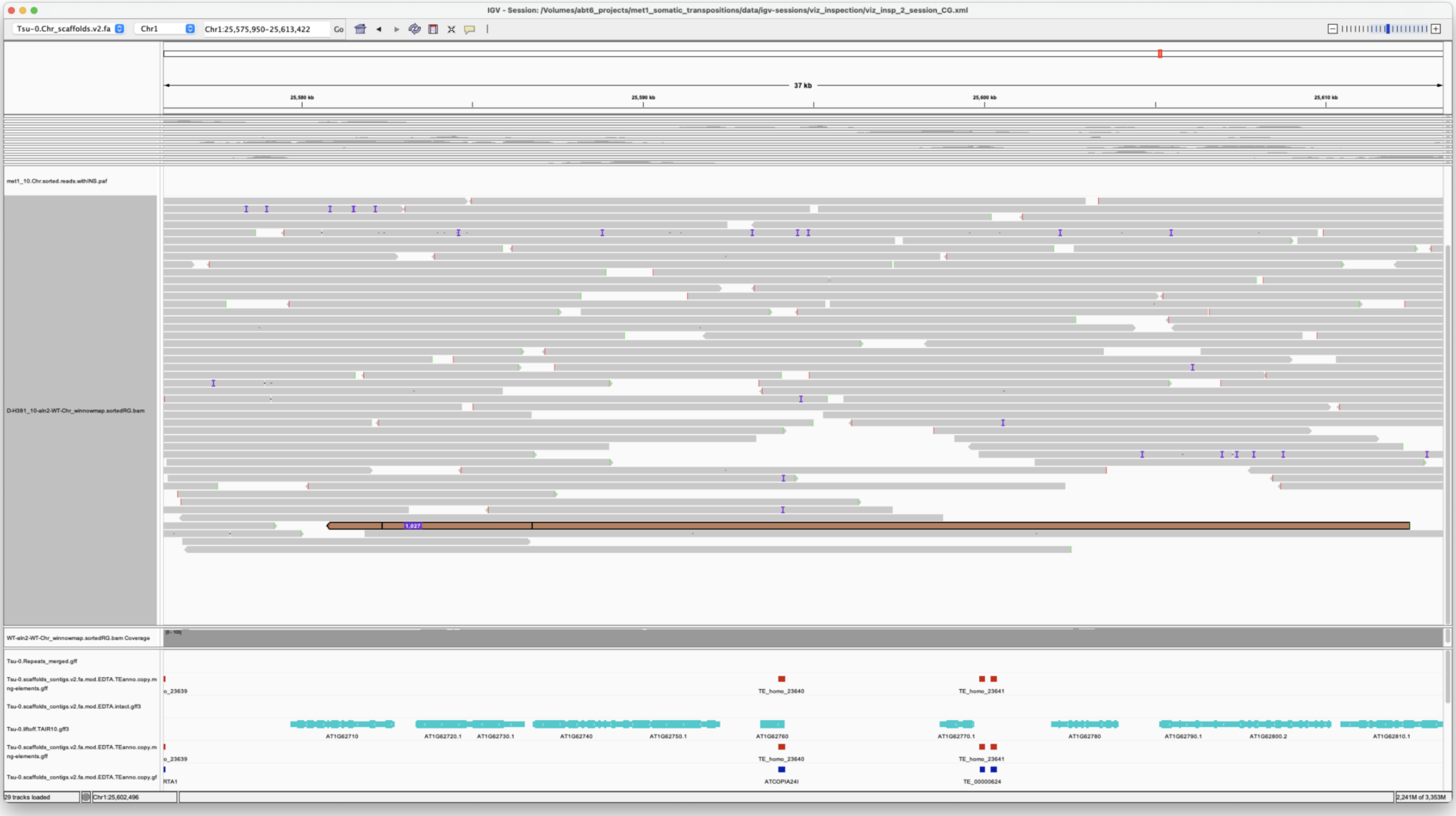

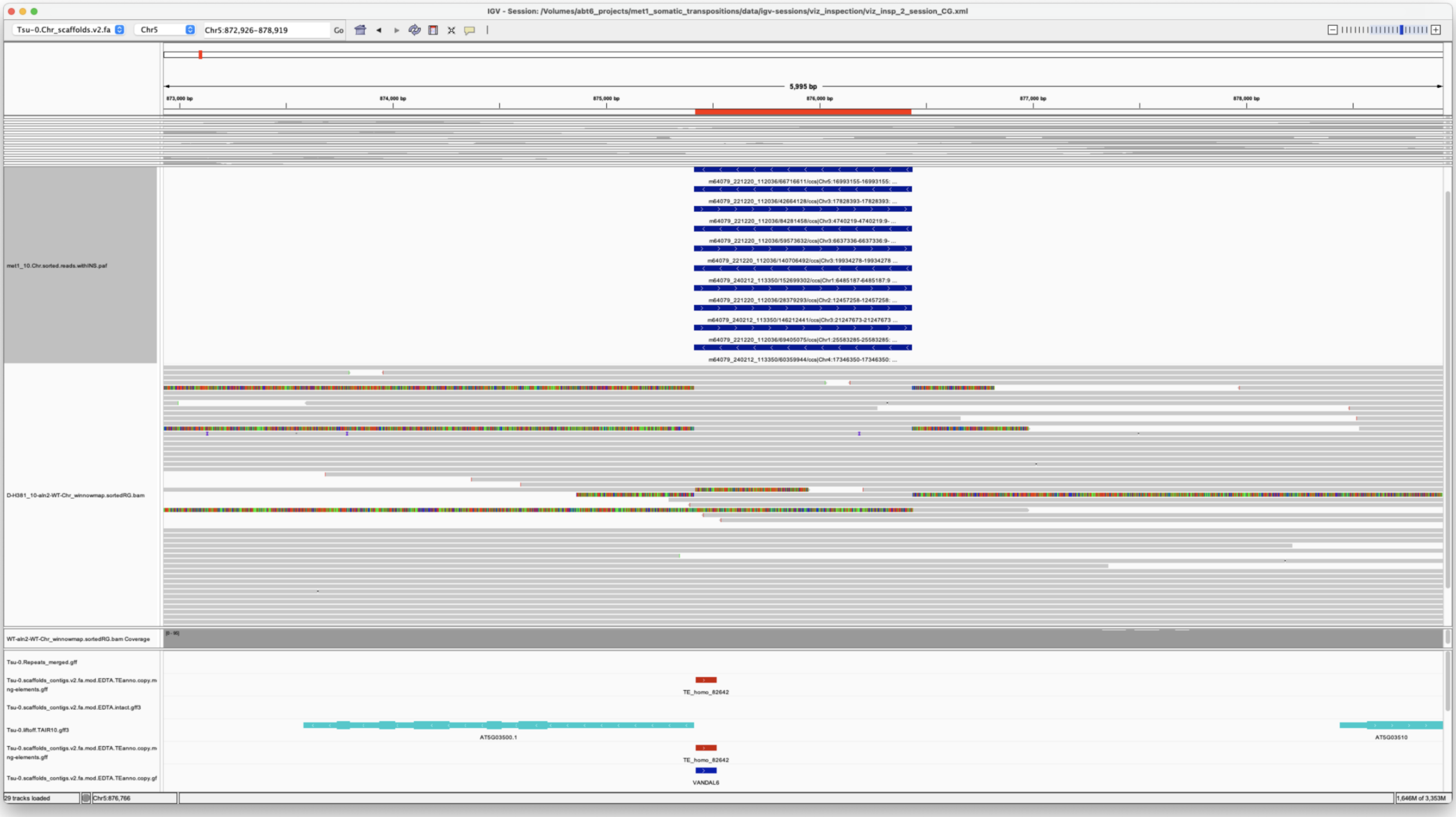

Confirmed

> Chr2 12457258 12457258 m64079_221220_112036/28379293/ccs Chr5 875413 876433 Chr5|875414|876433|ID=TE_MANUAL_02;Name=PAC;classification=DNA/DTC;sequence_ontology=MANUAL;identity=MANUAL;method=MANUAL;ID=TE_MANUAL_02;sequence_ontology=MANUAL met1_10

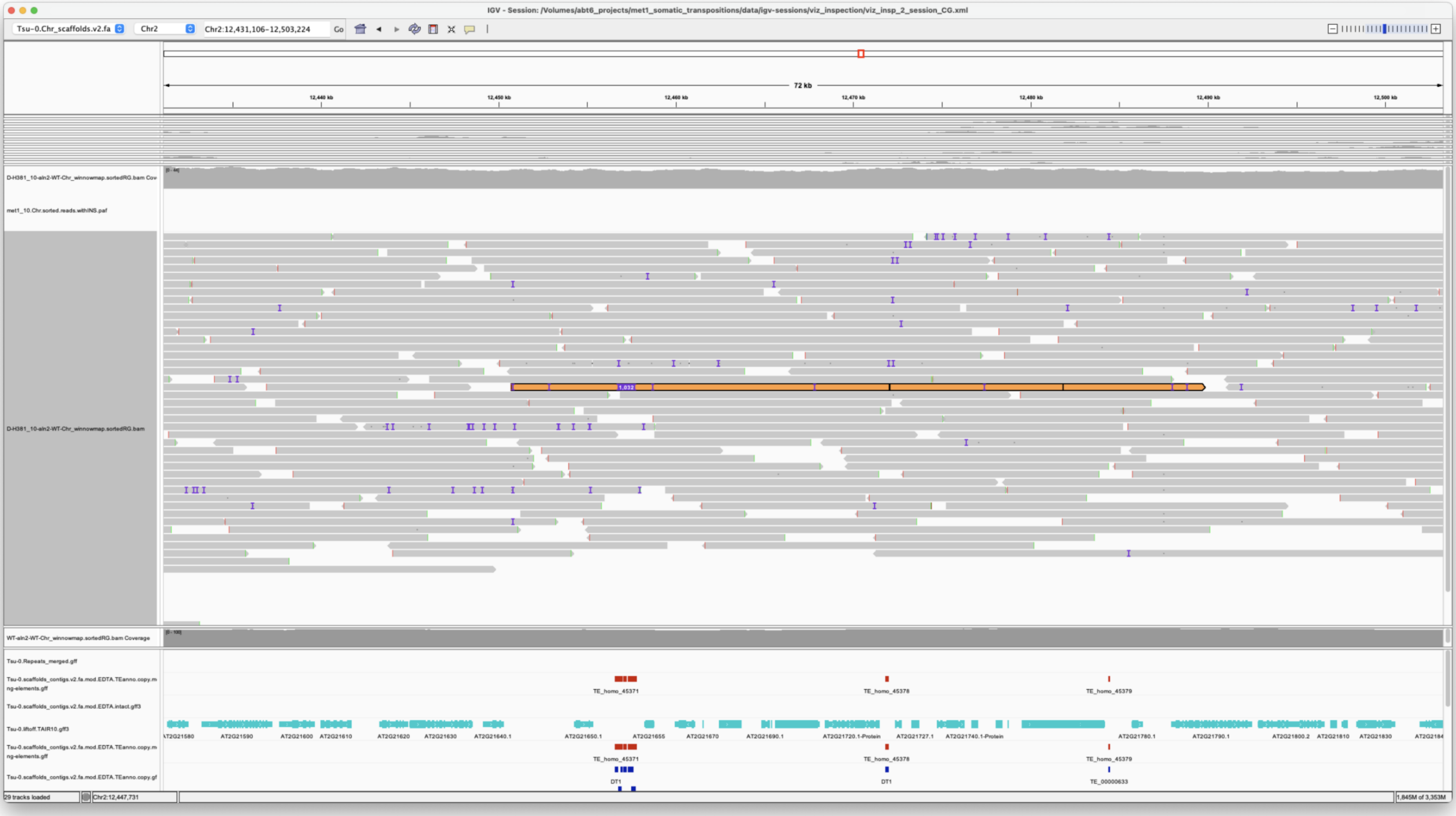

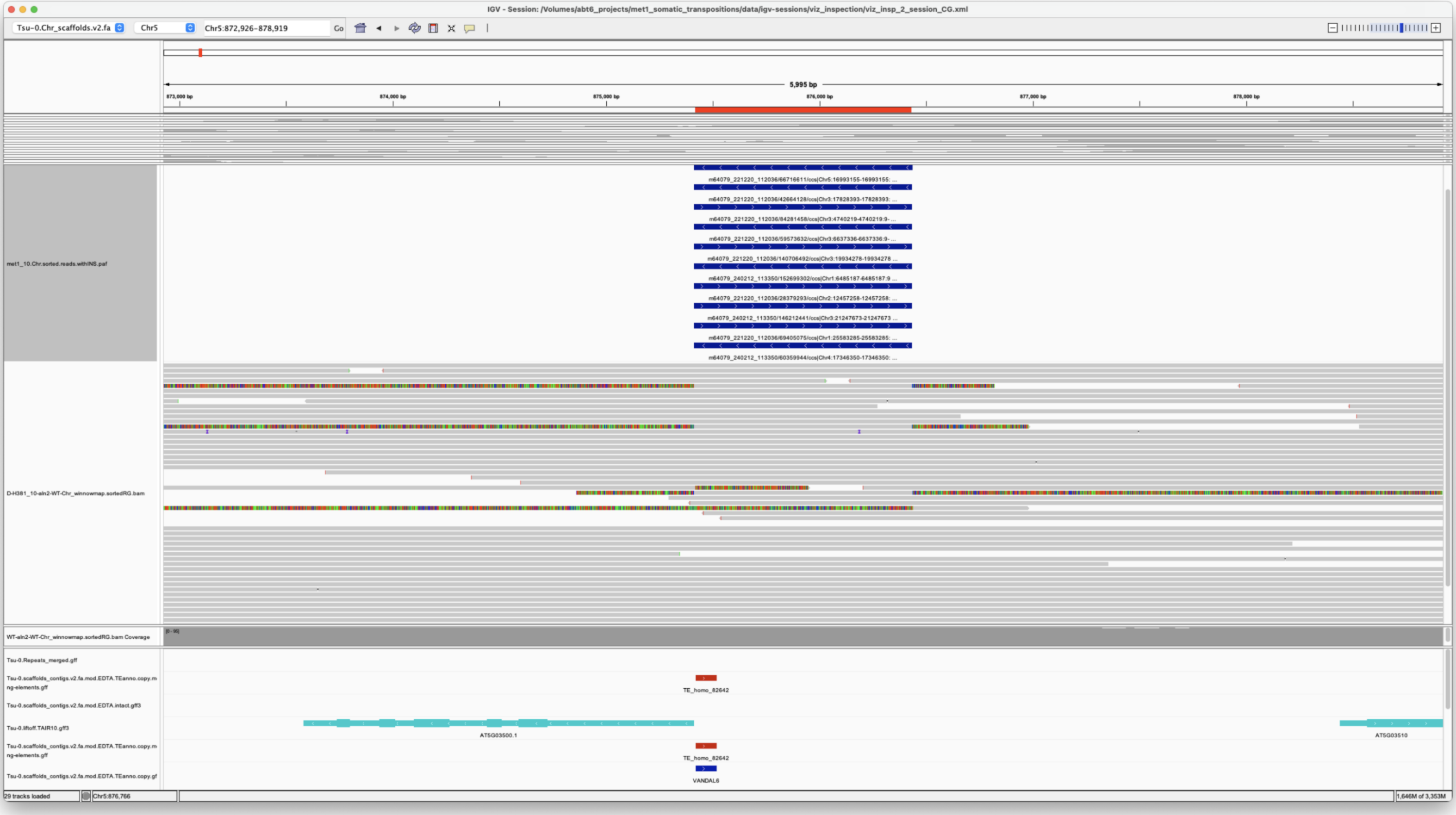

Confirmed

> Chr2 18394095 18394095 m64079_240212_113350/171707048/ccs Chr3 16344522 16352498 Chr3|16344522|16352496|ID=TE_homo_60420;Name=VANDAL6;classification=DNA/Mutator;sequence_ontology=SO:0002280;identity=0.969;method=homology;ID=TE_homo_62001;sequence_ontology=SO:0002280 met1_10

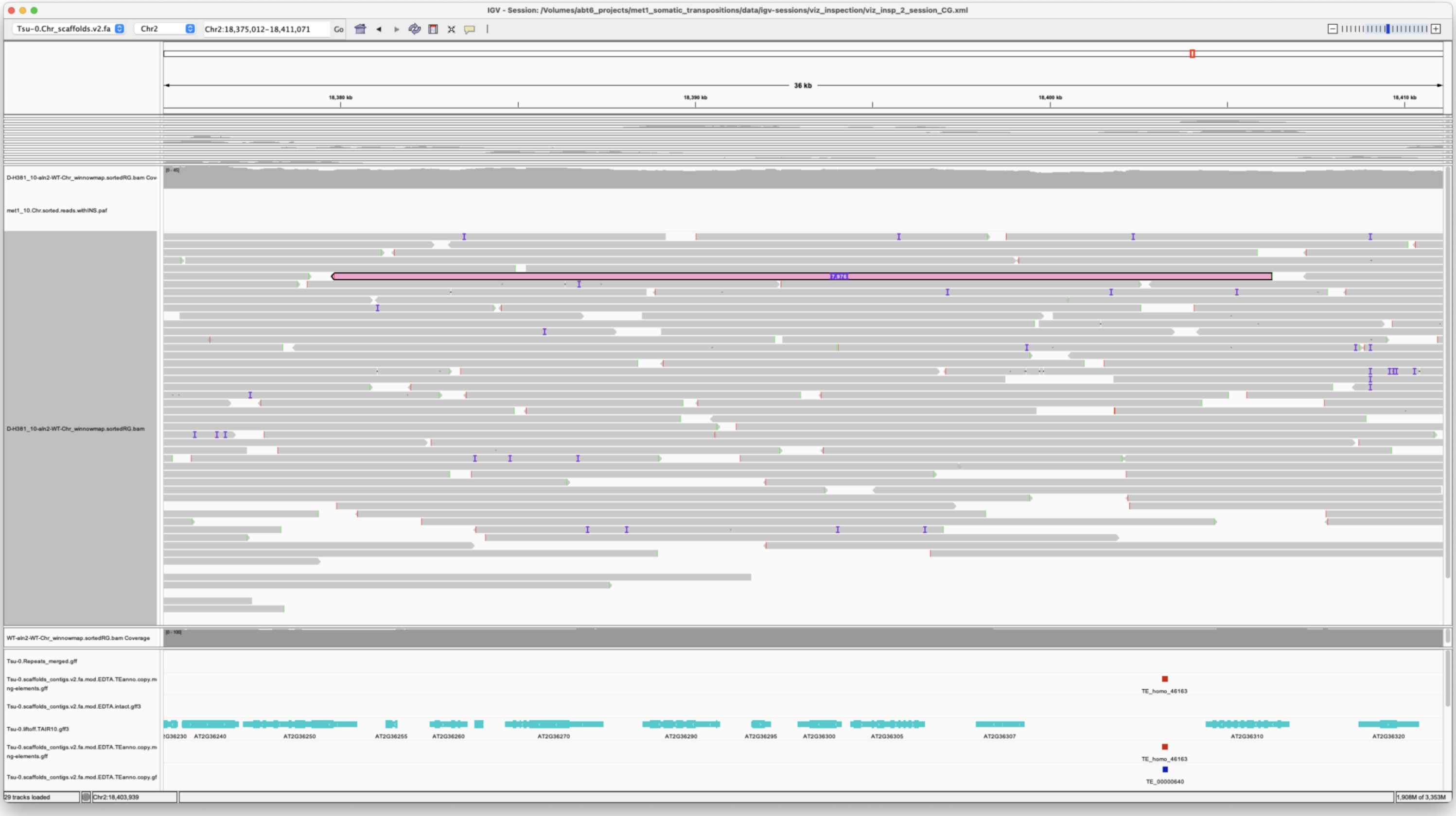

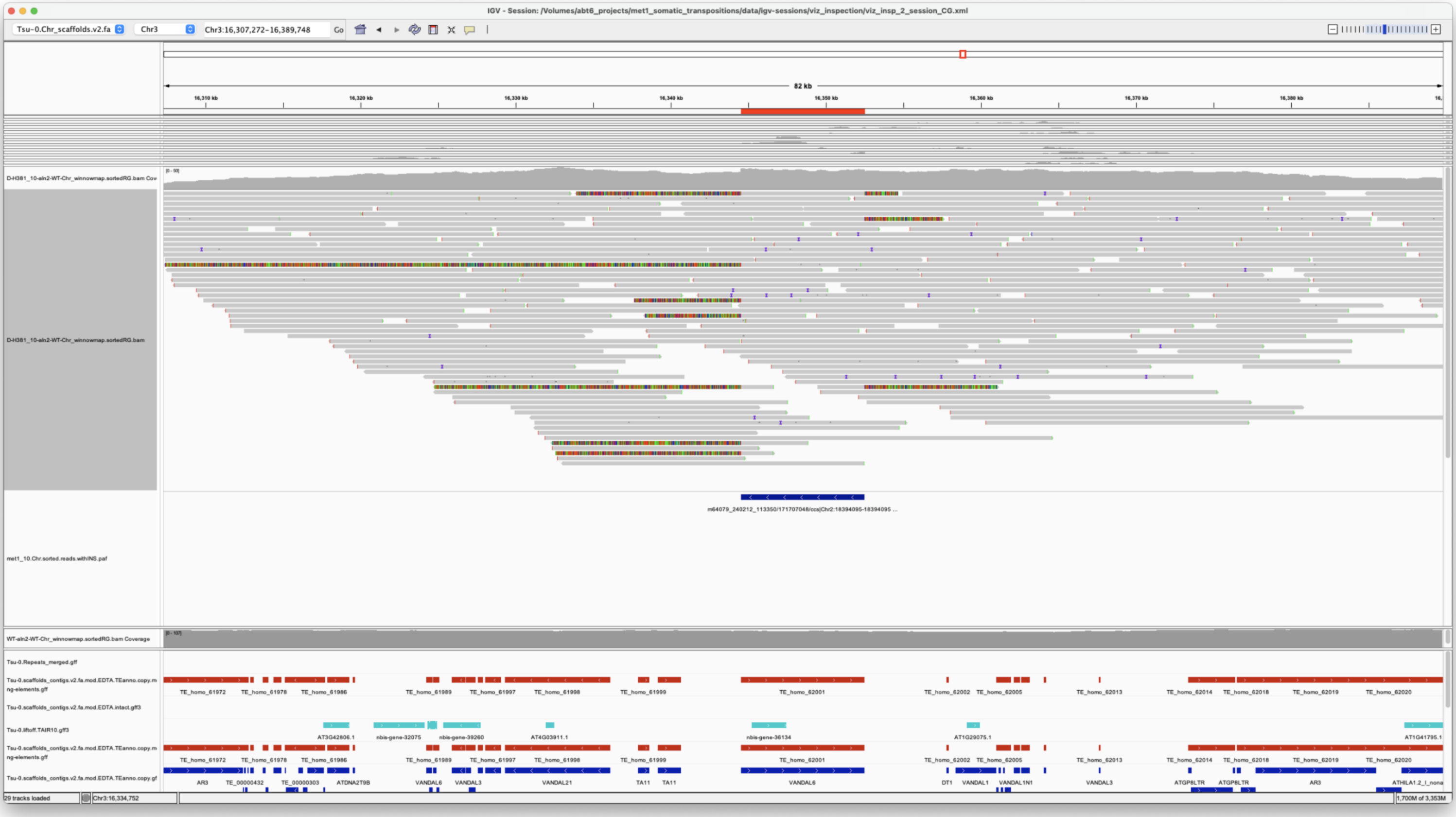

Confirmed

> Chr3 4740219 4740219 m64079_221220_112036/84281458/ccs Chr5 875413 876434
> Chr5|875414|876433|ID=TE_MANUAL_02;Name=PAC;classification=DNA/DTC;sequence_ontology=MANUAL;identity=MANUAL;method=MANUAL;ID=TE_MANUAL_02;sequence_ontology=MANUAL met1_10

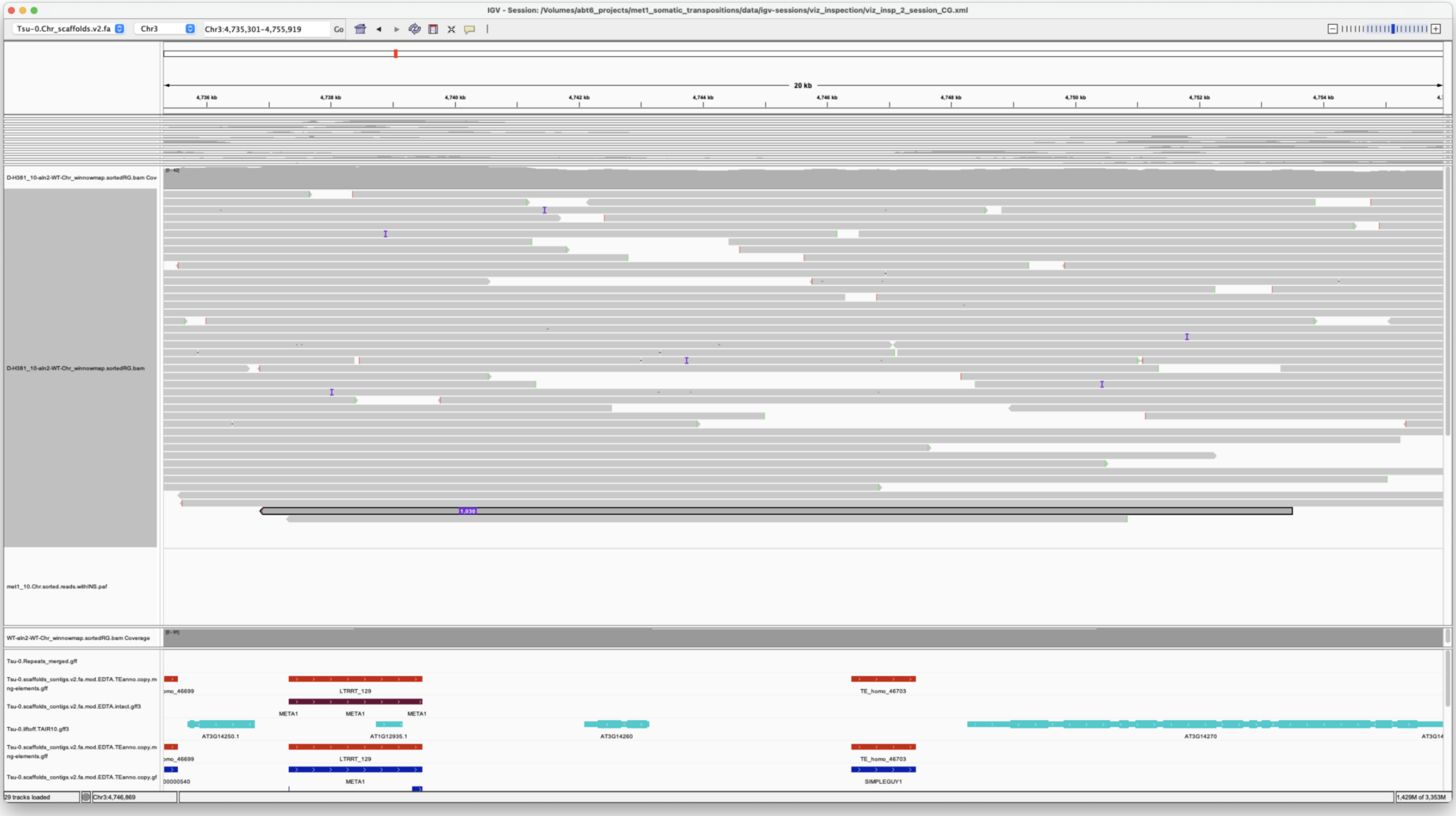

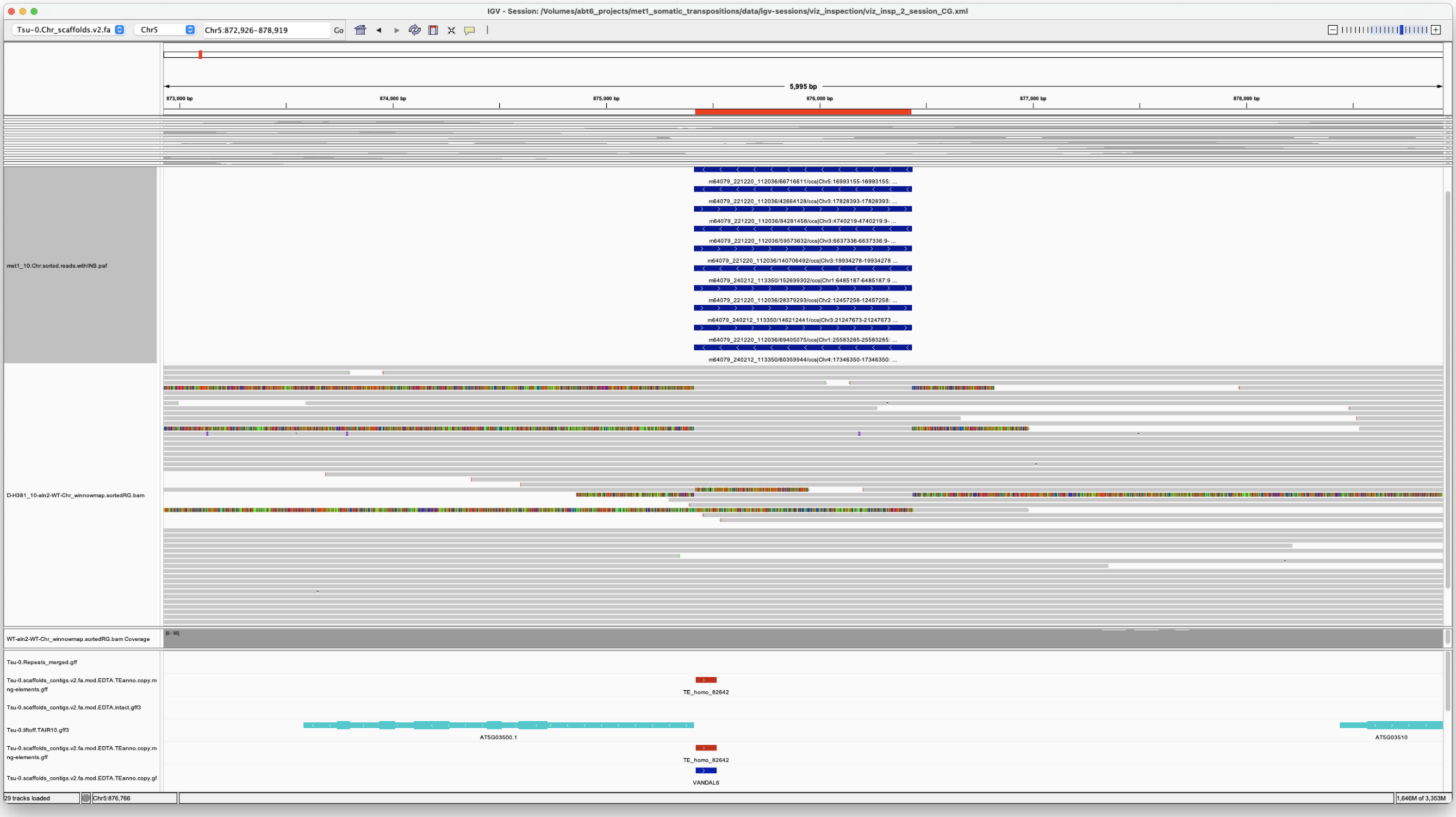

Confirmed

> Chr3 6637336 6637336 m64079_221220_112036/59573632/ccs Chr5 875413 876434
> Chr5|875414|876433|ID=TE_MANUAL_02;Name=PAC;classification=DNA/DTC;sequence_ontology=MANUAL;identity=MANUAL;method=MANUAL;ID=TE_MANUAL_02;sequence_ontology=MANUAL met1_10

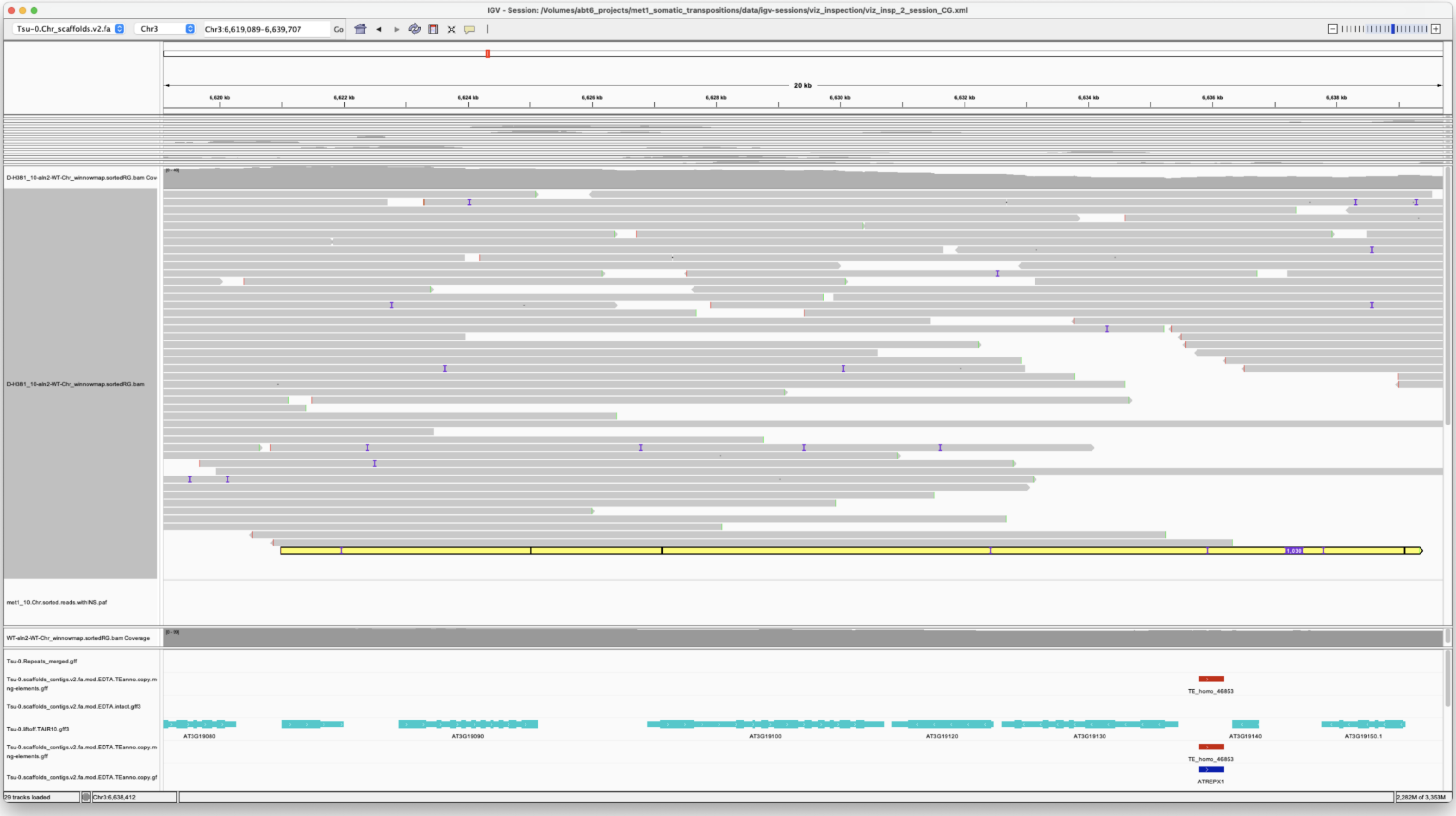

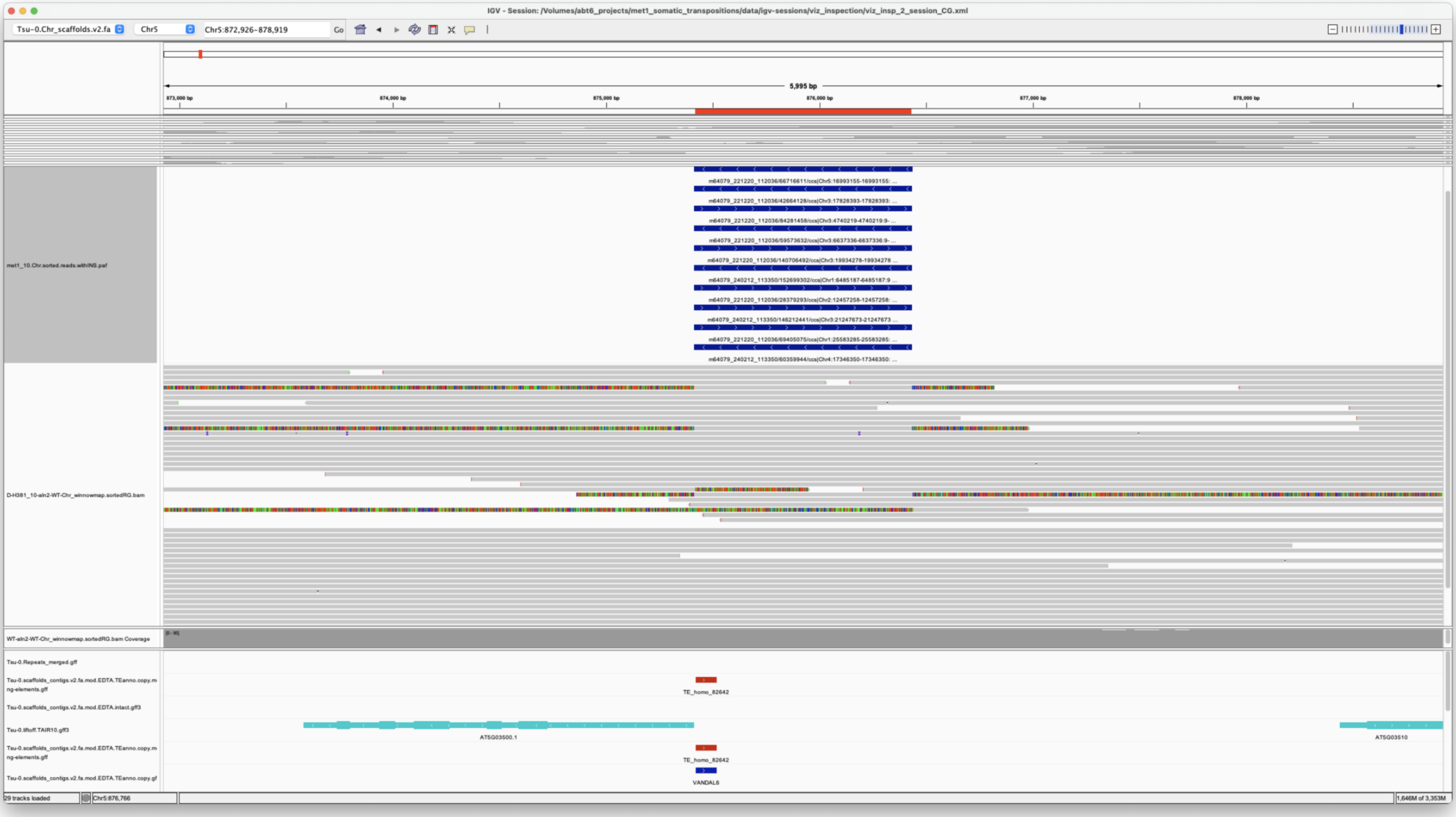

Confirmed

> Chr3 17828393 17828393 m64079_221220_112036/42664128/ccs Chr5 875413 876434 Chr5|875414|876433|ID=TE_MANUAL_02;Name=PAC;classification=DNA/DTC;sequence_ontology=MANUAL;identity=MANUAL;method=MANUAL;ID=TE_MANUAL_02;sequence_ontology=MANUAL met1_10

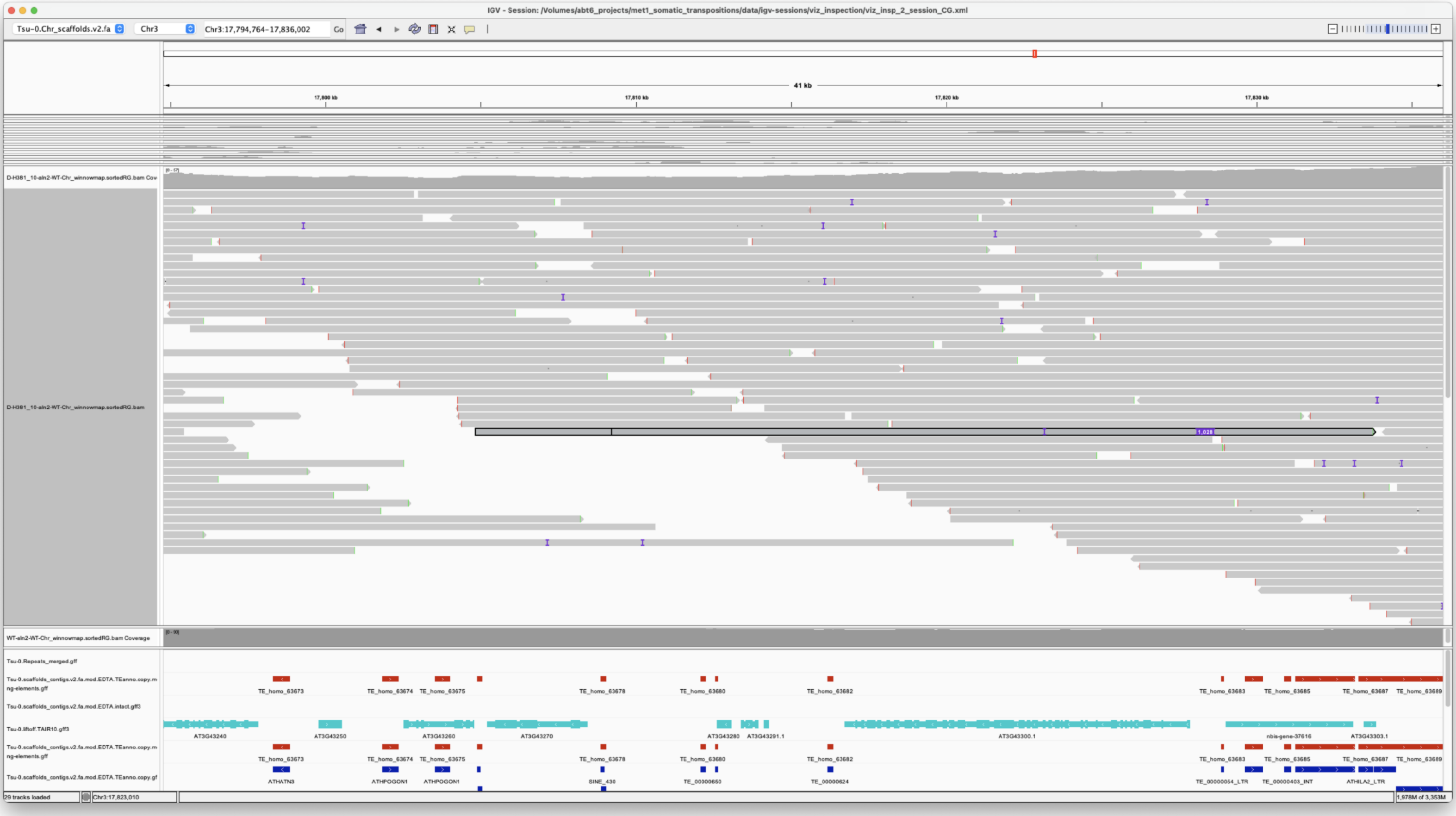

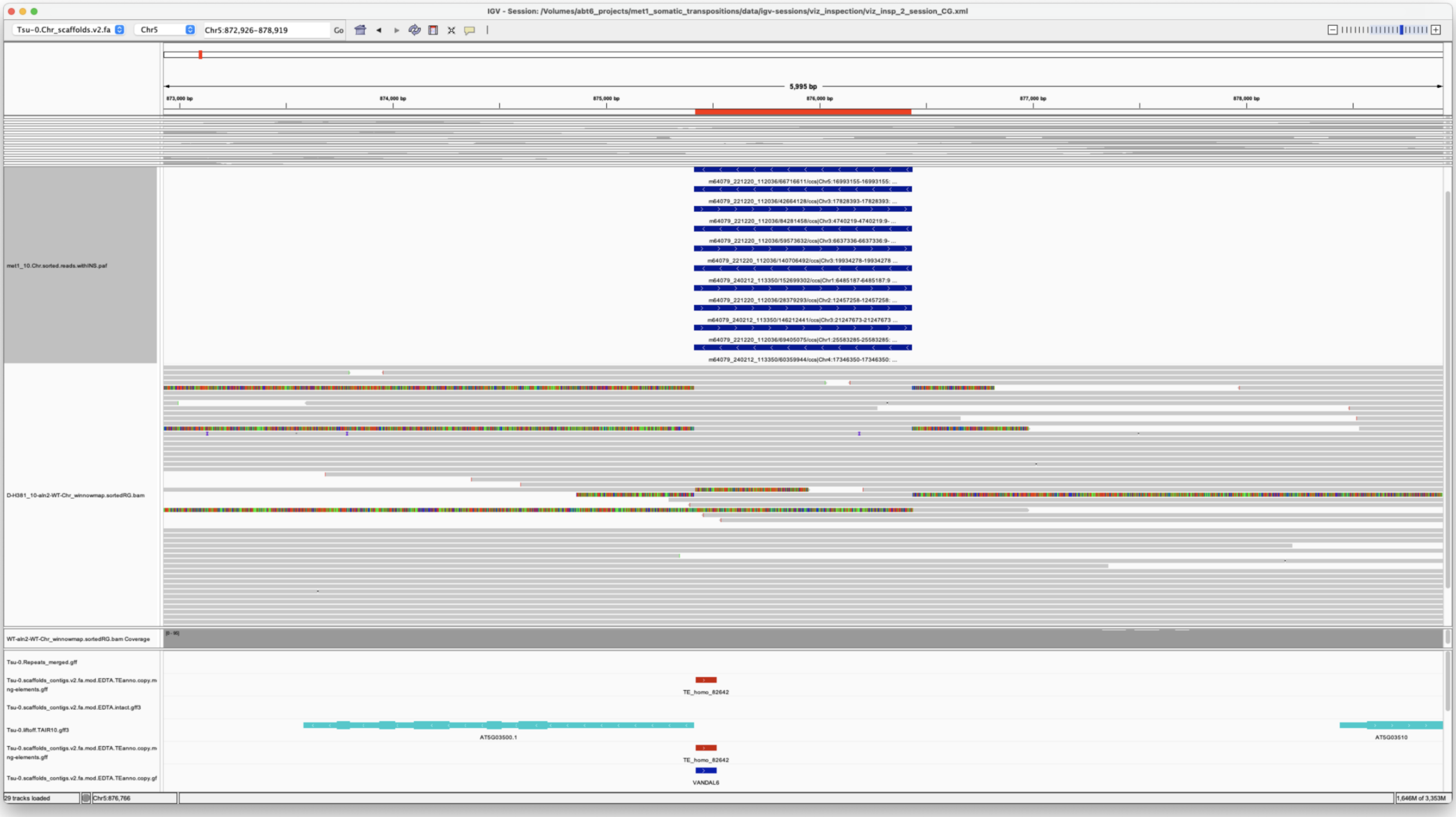

Confirmed

Chr3 19934278 19934278 m64079_221220_112036/140706492/ccs Chr5 875413 876434 Chr5|875414|876433|ID=TE_MANUAL_02;Name=PAC;classification=DNA/DTC;sequence_ontology=MANUAL;identity=MANUAL;method=MANUAL;ID=TE_MANUAL_02;sequence_ontology=MANUAL met1_10

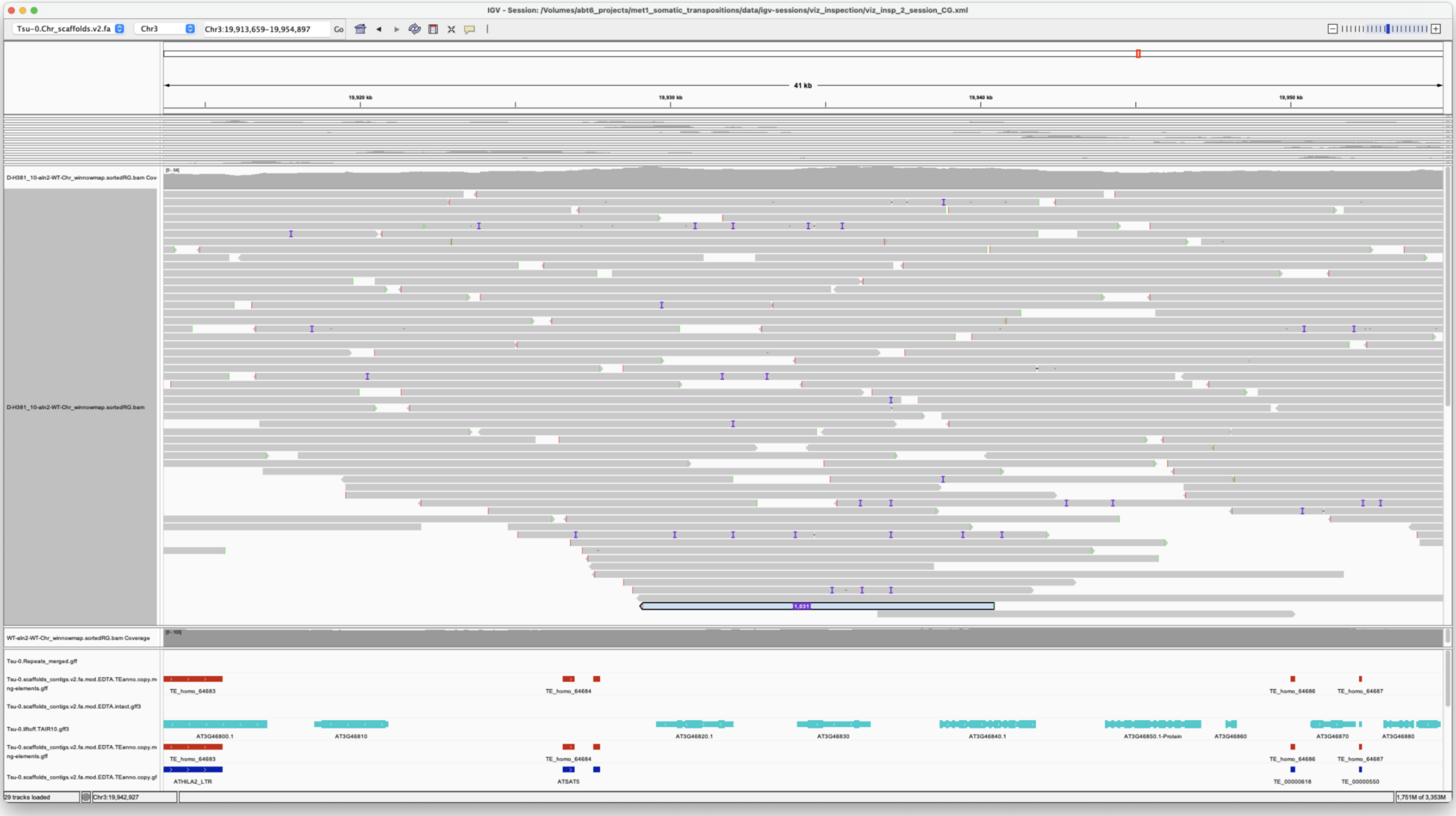

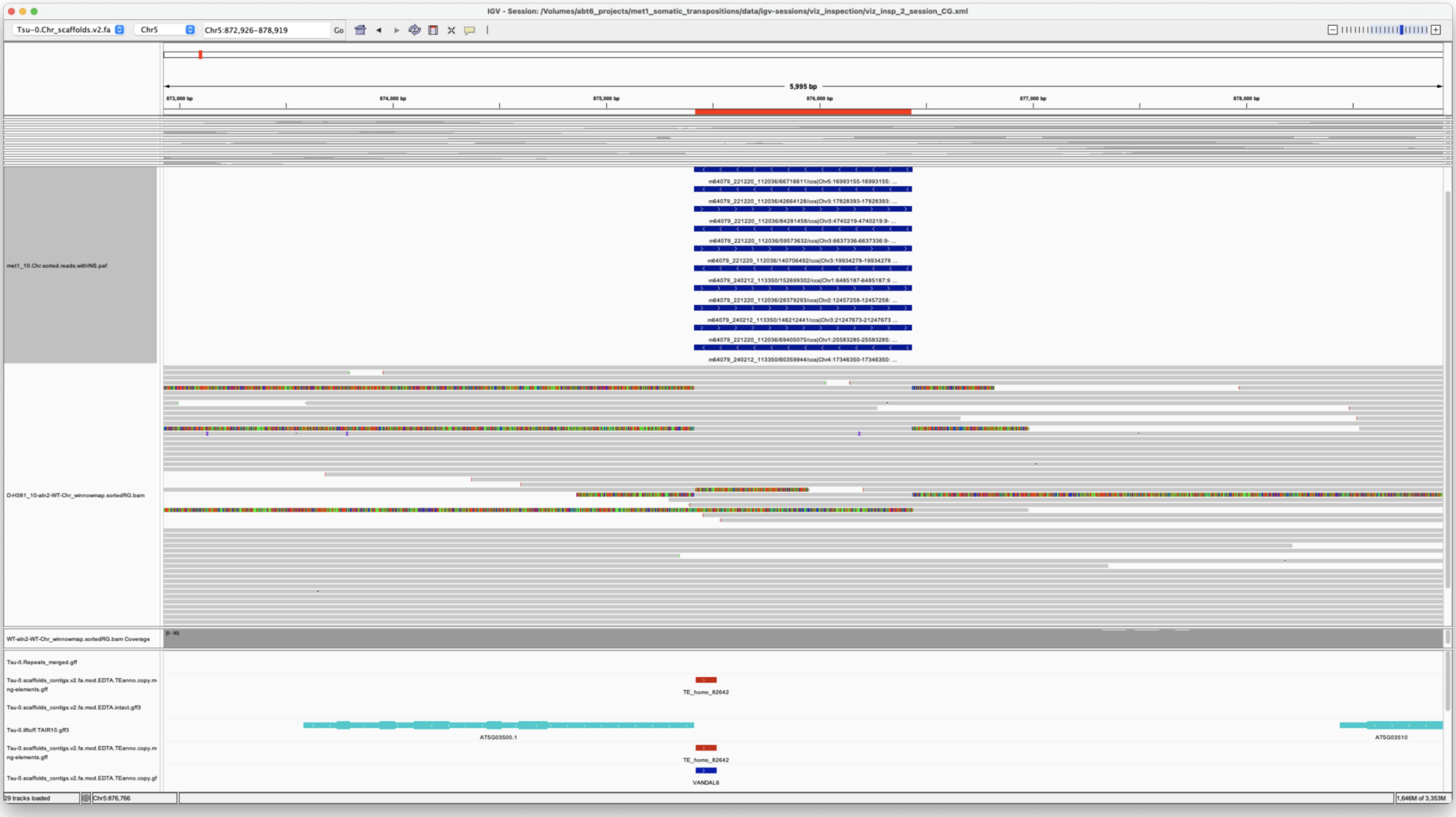

Confirmed

Chr3 21247673 21247673 m64079_240212_113350/146212441/ccs Chr5 875413 876433 Chr5|875414|876433|ID=TE_MANUAL_02;Name=PAC;classification=DNA/DTC;sequence_ontology=MANUAL;identity=MANUAL;method=MANUAL;ID=TE_MANUAL_02;sequence_ontology=MANUAL met1_10

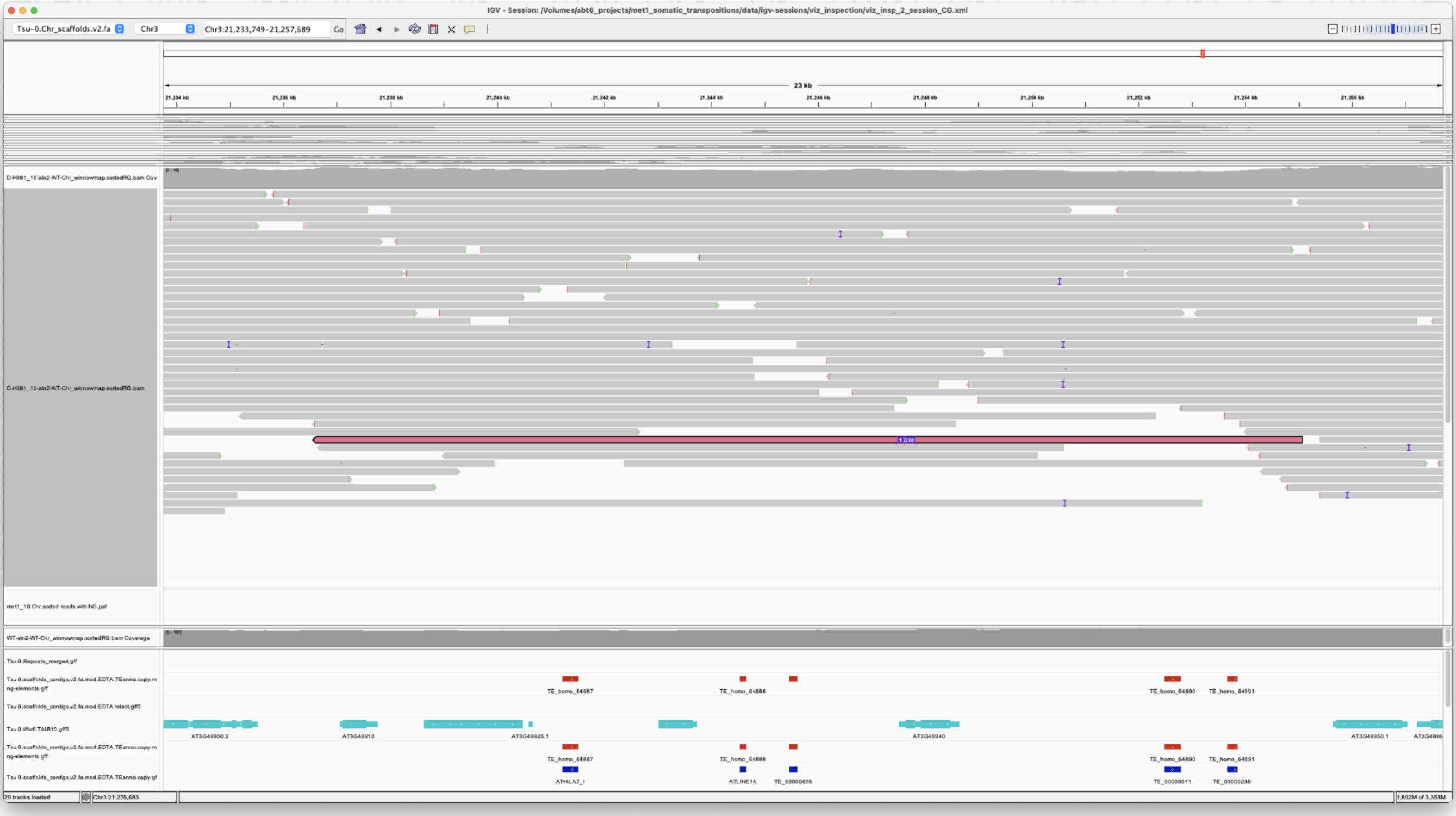

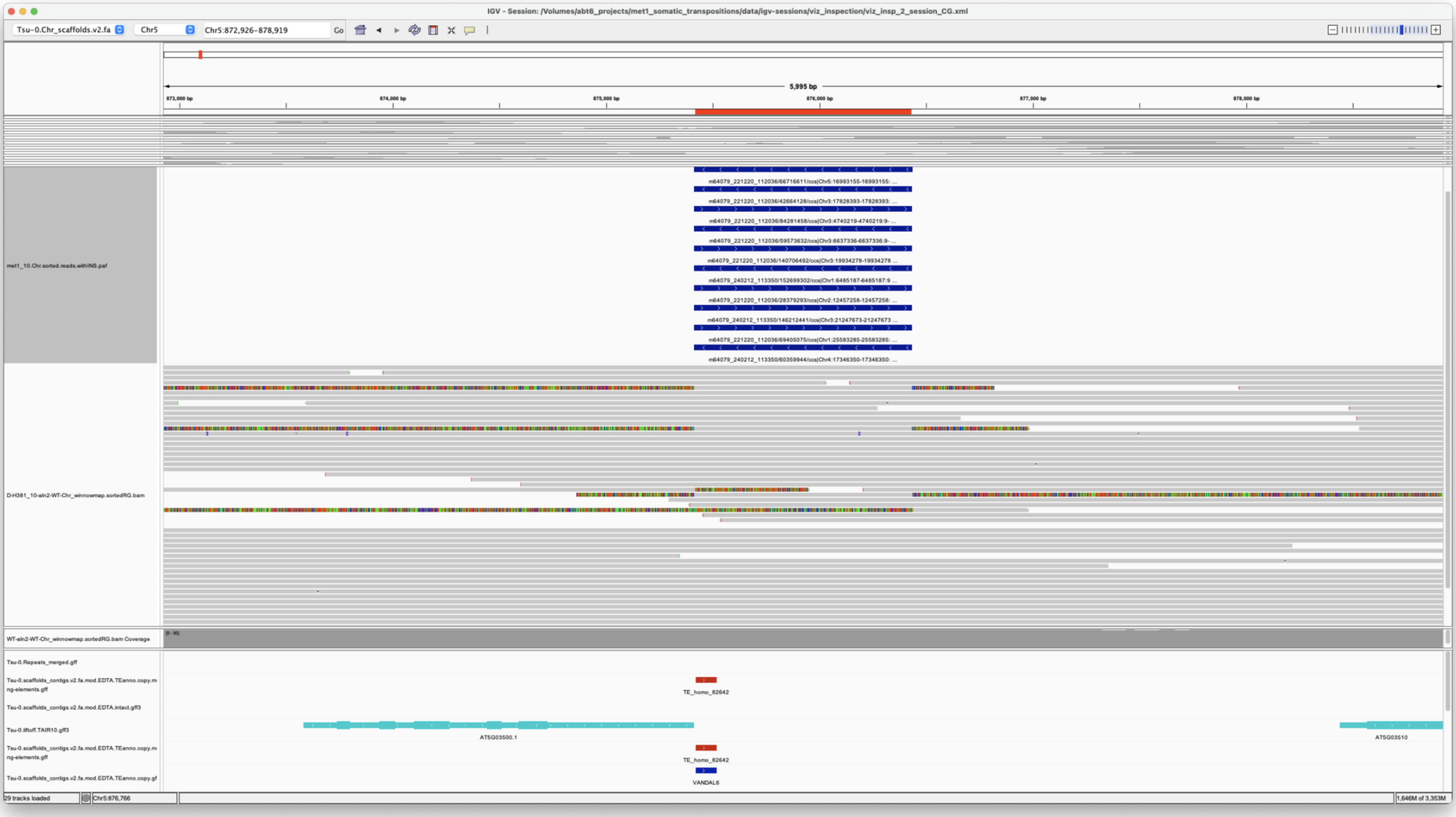

Confirmed

> Chr4 939231 939231 m64079_240212_113350/138150026/ccs Chr1 11941103 11946436
> Chr1|11941106|11946435|ID=LTRRT_5;Name=ATCOPIA93.2_Evade;Classification=LTR/Copia;Sequence_ontology=SO:0002264;ltr_identity=1.0000;Method=structural;motif=TACA;tsd=ATATG met1_10

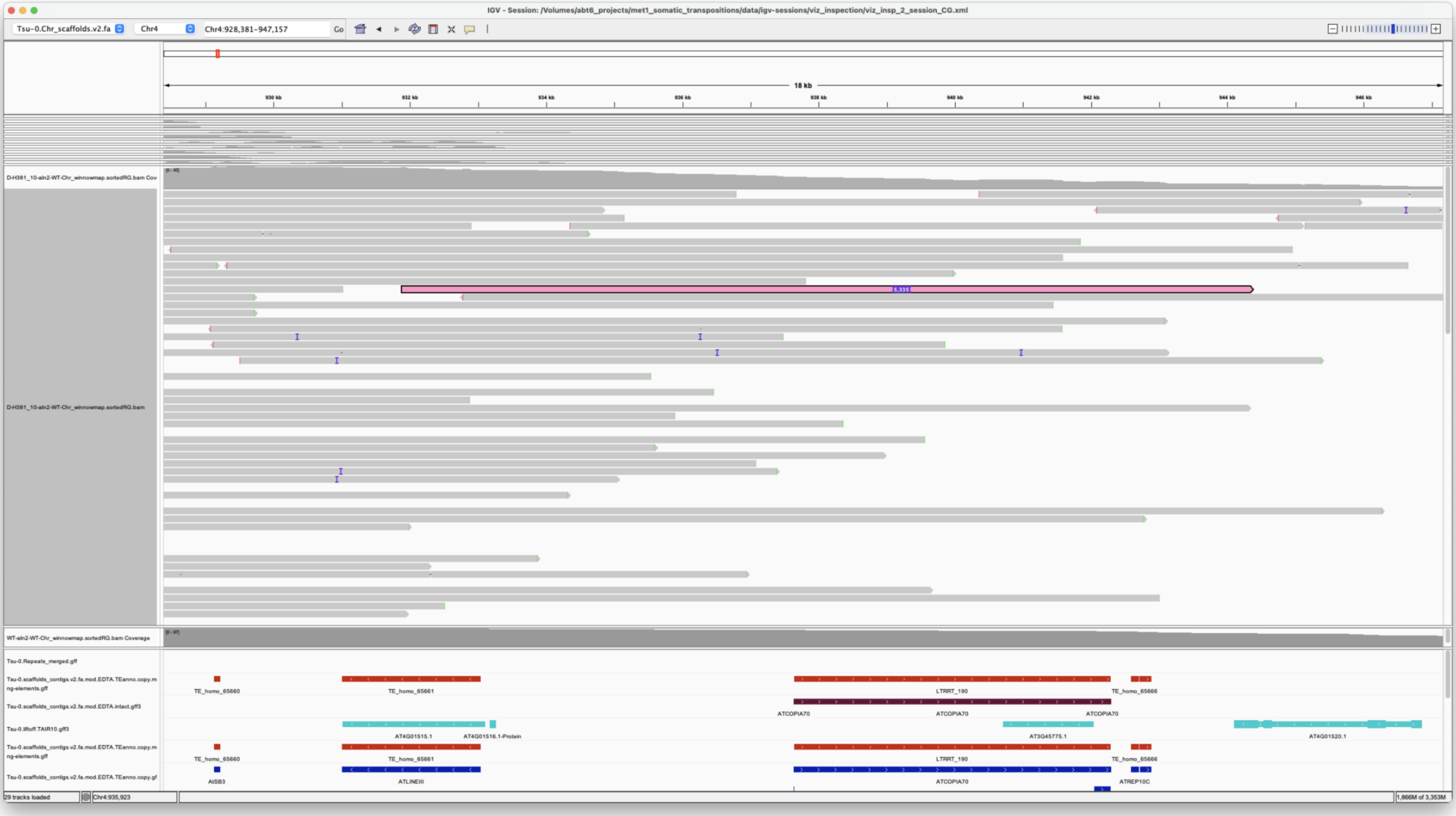

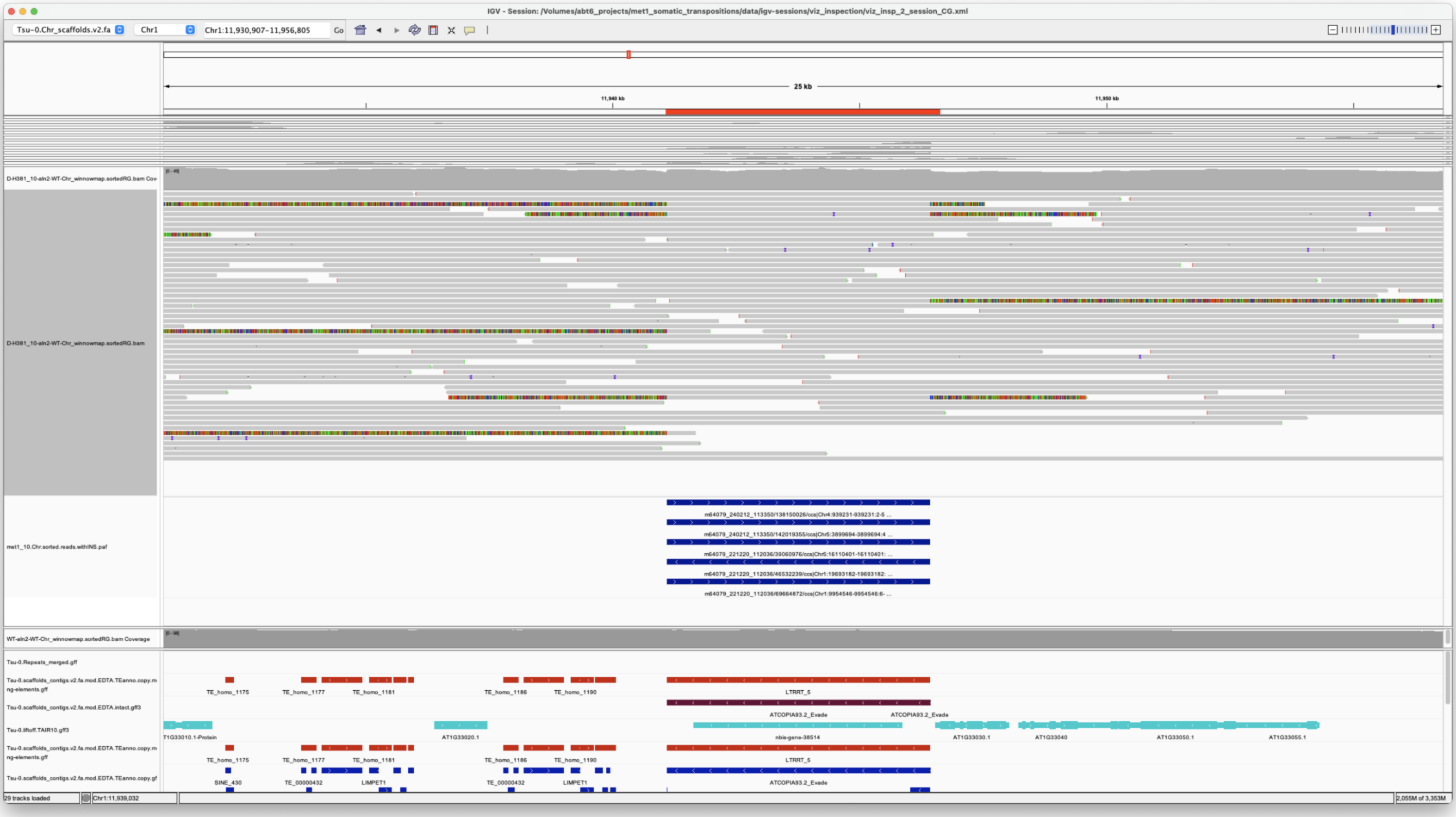

Confirmed

Chr4 17346350 17346350 m64079_240212_113350/60359944/ccs Chr5 875414 876434
Chr5|875414|876433|ID=TE_MANUAL_02;Name=PAC;classification=DNA/DTC;sequence_ontology=MANUAL;identity=MANUAL;method=MANUAL;ID=TE_MANUAL_02;sequence_ontology=MANUAL met1_10

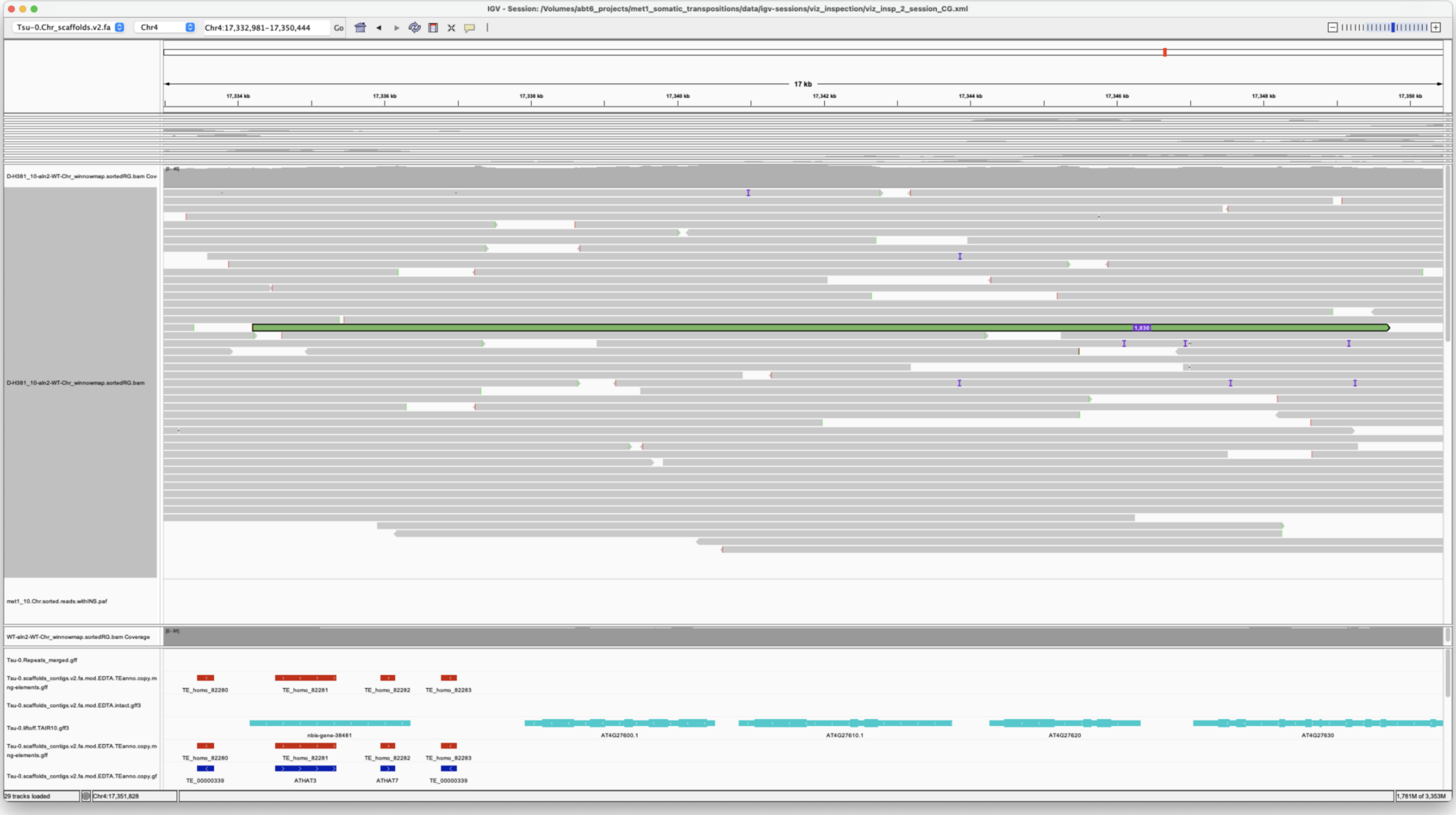

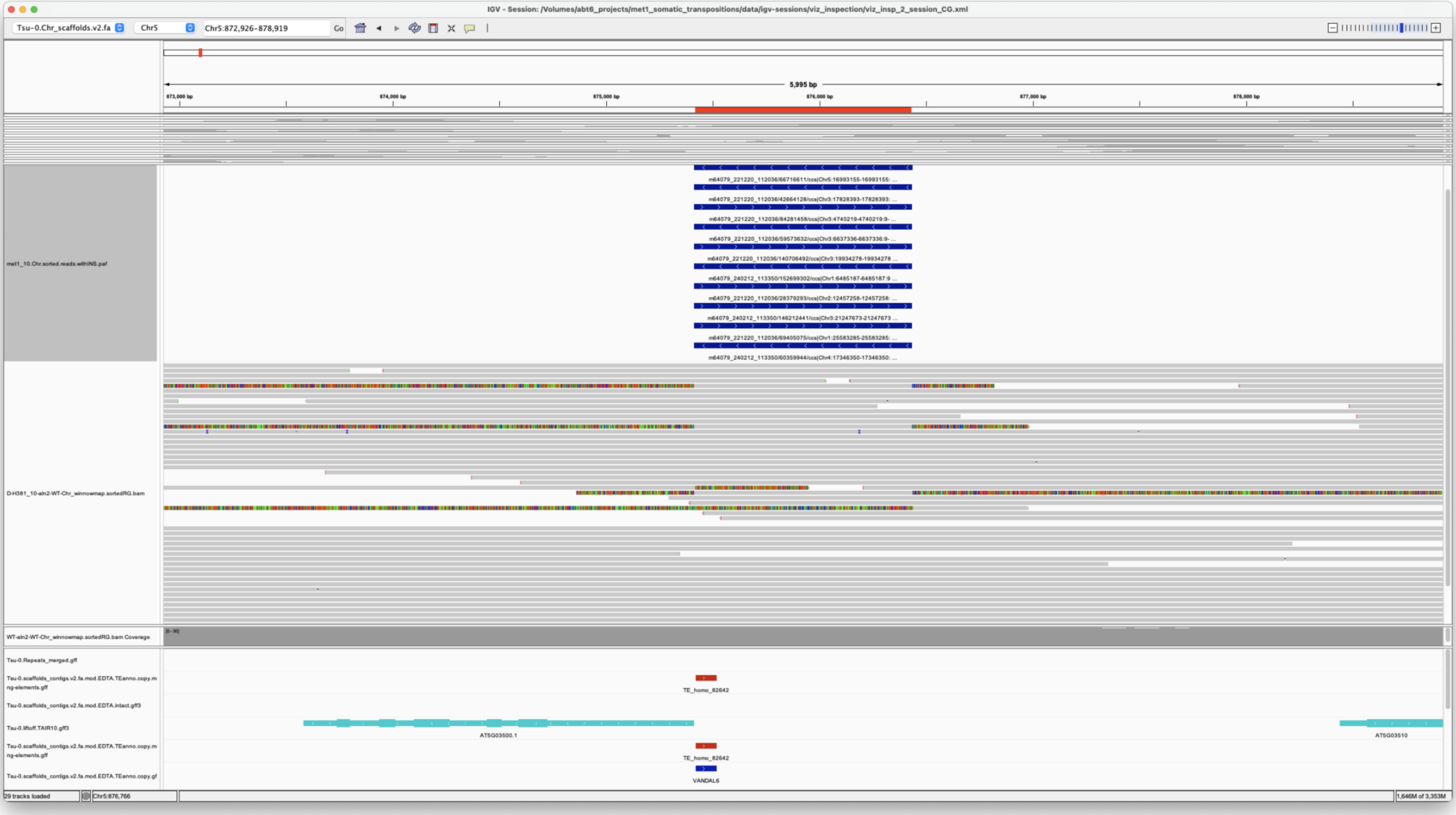

Confirmed

> Chr5 3899694 3899694 m64079_240212_113350/142019355/ccs Chr1 11941104 11946435
> Chr1|11941106|11946435|ID=LTRRT_5;Name=ATCOPIA93.2_Evade;Classification=LTR/Copia;Sequence_ontology=SO:0002264;ltr_identity=1.0000;Method=structural;motif=TACA;tsd=ATATG met1_10

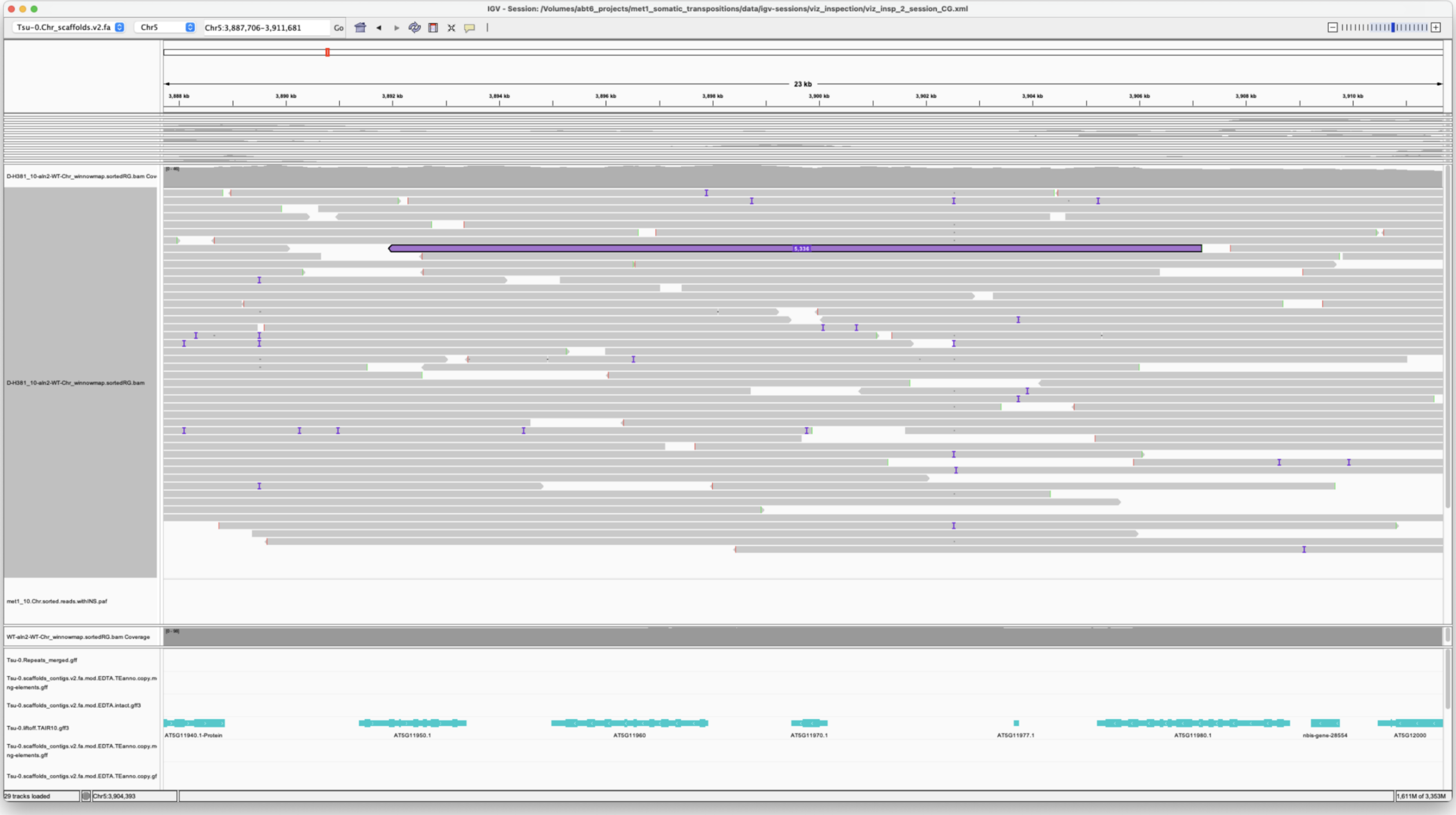

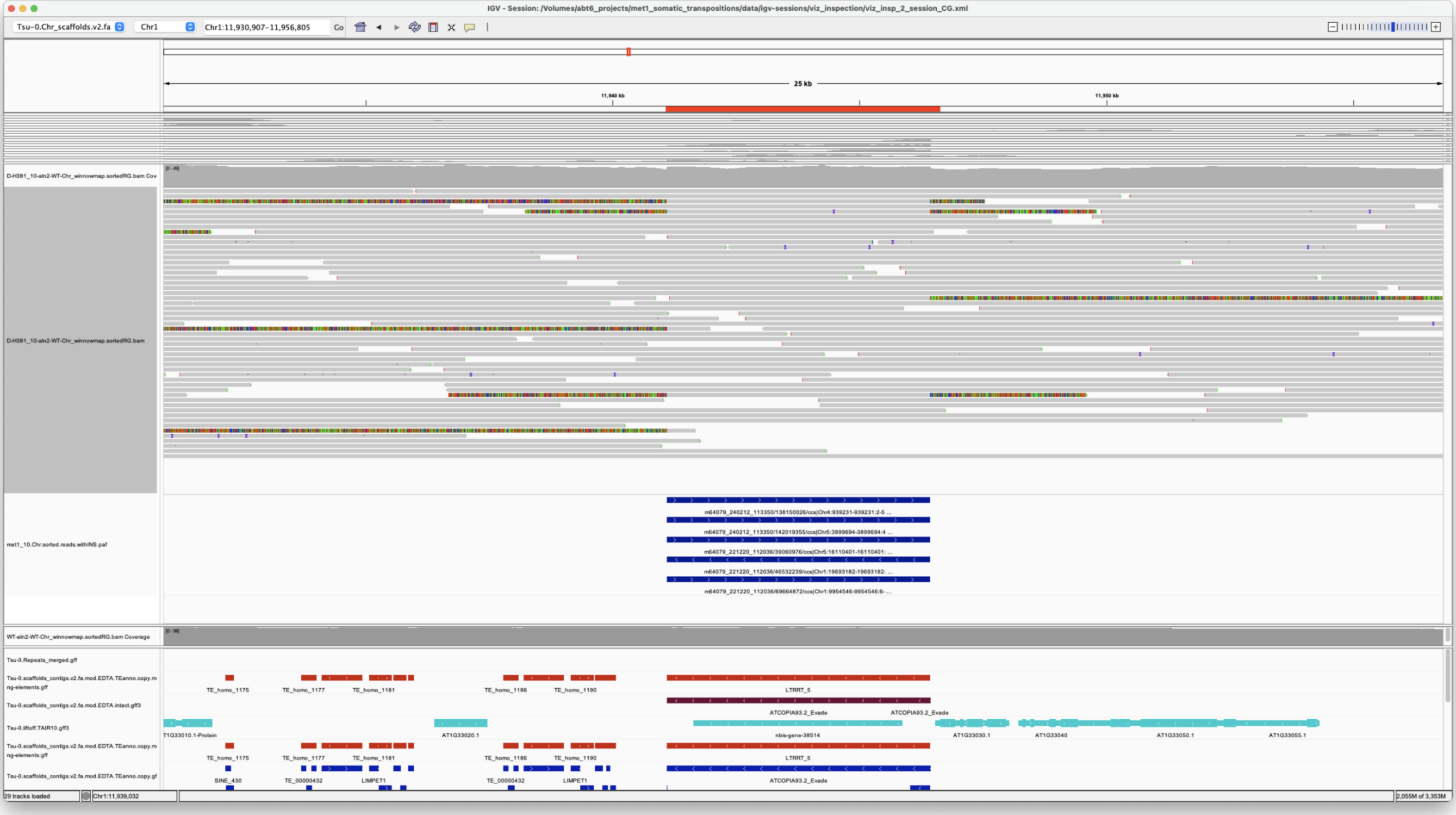

Confirmed

> Chr5 5803022 5803022 m64079_221220_112036/120455225/ccs Chr5 19152825 19160825 Chr5|19152829|19160825|ID=TE_homo_95640;Name=VANDAL21;classification=DNA/Mutator;sequence_ontology=SO:0002280;identity=0.976;method=homology;ID=TE_homo_98501;sequence_ontology=SO:0002280|ID=TE_homo_95641;Name=VANDAL21;classification=DNA/Mutator;sequence_ontology=SO:0002280;identity=0.966;method=homology;ID=TE_homo_98502;sequence_ontology=SO:0002280 met1_10

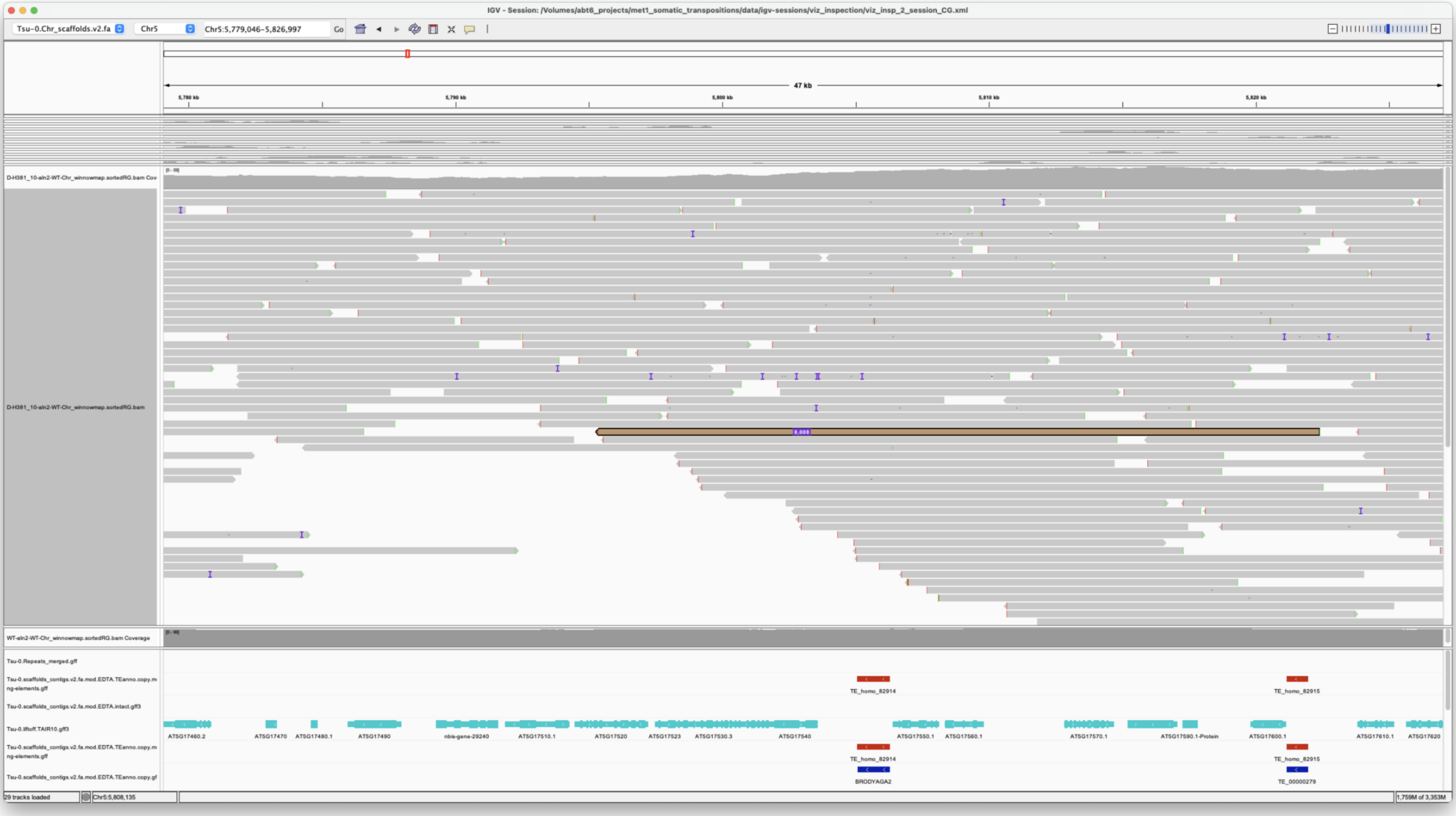

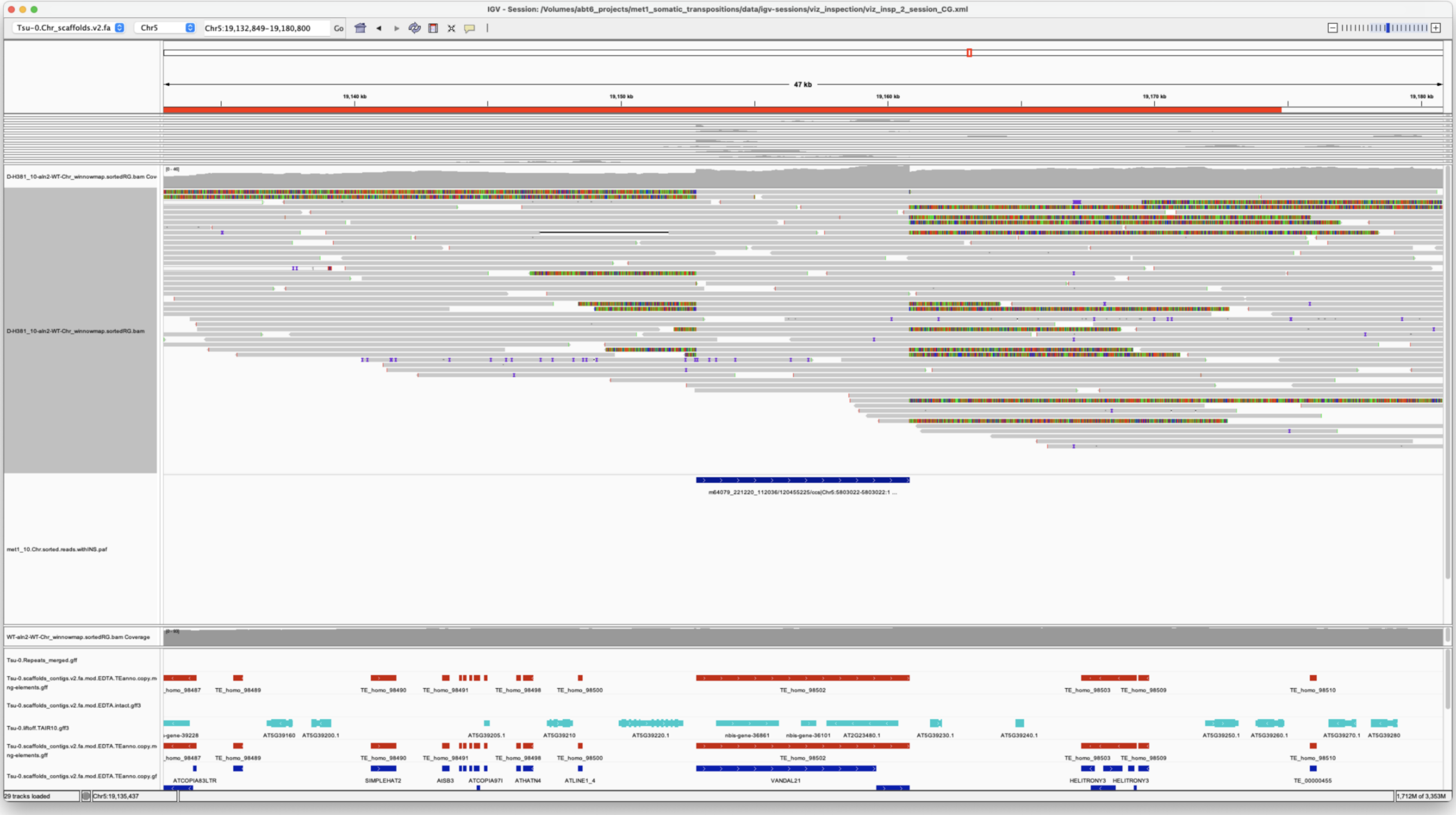

Confirmed

> Chr5 16110401 16110401 m64079_221220_112036/39060976/ccs Chr1 11941106 11946436 Chr1|11941106|11946435|ID=LTRRT_5;Name=ATCOPIA93.2_Evade;Classification=LTR/Copia;Sequence_ontology=SO:0002264;ltr_identity=1.0000;Method=structural;motif=TACA;tsd=ATATG met1_10

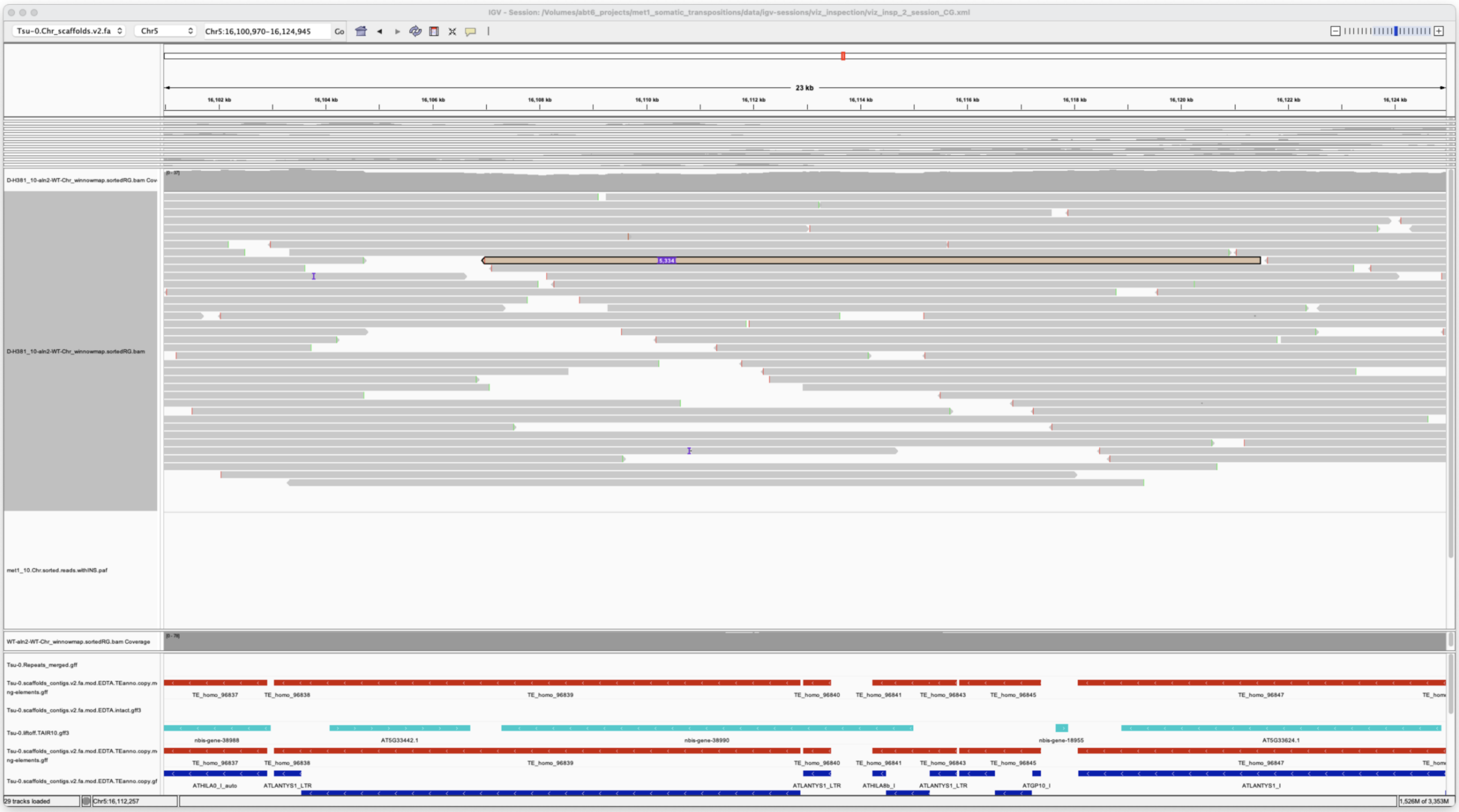

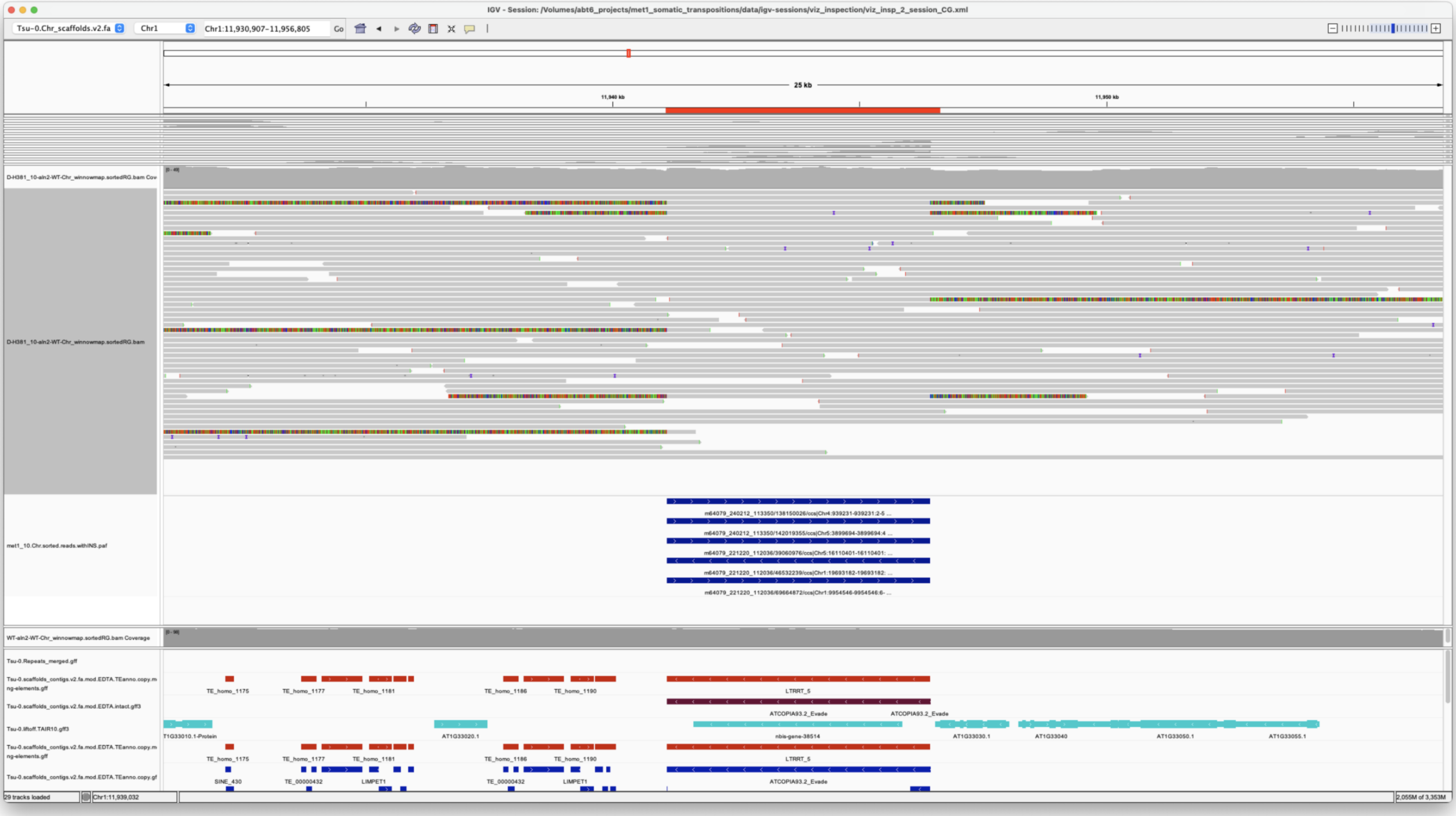

Confirmed

Chr5 16993155 16993155 m64079_221220_112036/66716611/ccs Chr5 875413 876436 Chr5|875414|876433|ID=TE_MANUAL_02;Name=PAC;classification=DNA/DTC;sequence_ontology=MANUAL;identity=MANUAL;method=MANUAL;ID=TE_MANUAL_02;sequence_ontology=MANUAL met1_10

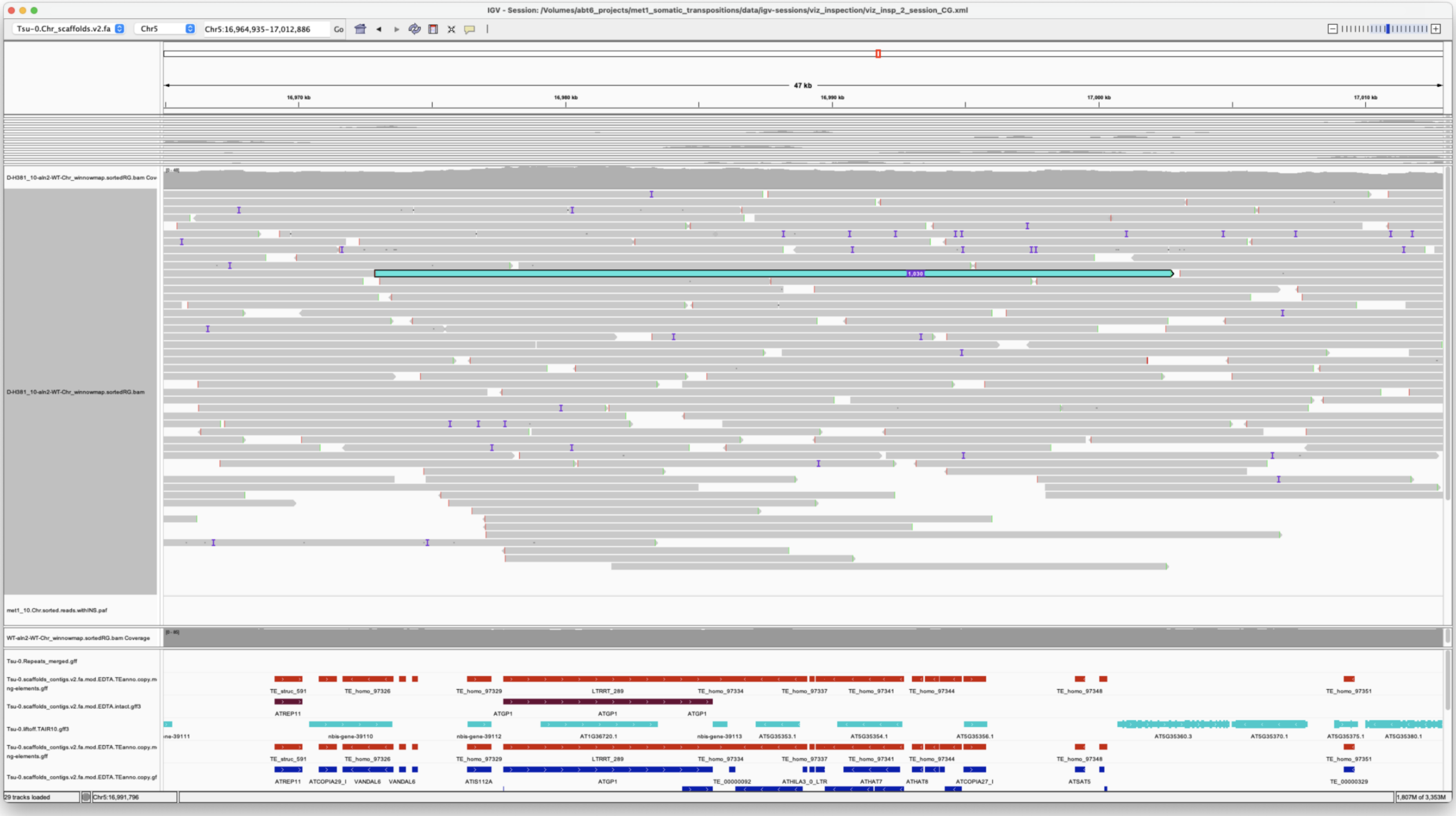
IGV - Session: /Volumes/abt6_projects/met1_somatic_transpositions/data/igv-sessions/viz_inspection/viz_insp_2_session_CG.xml
Tsu-0.Chr_scaffolds.v2.fa
Chr5
Chr5:16,964,935-17,012,886
47 kb
29 tracks loaded

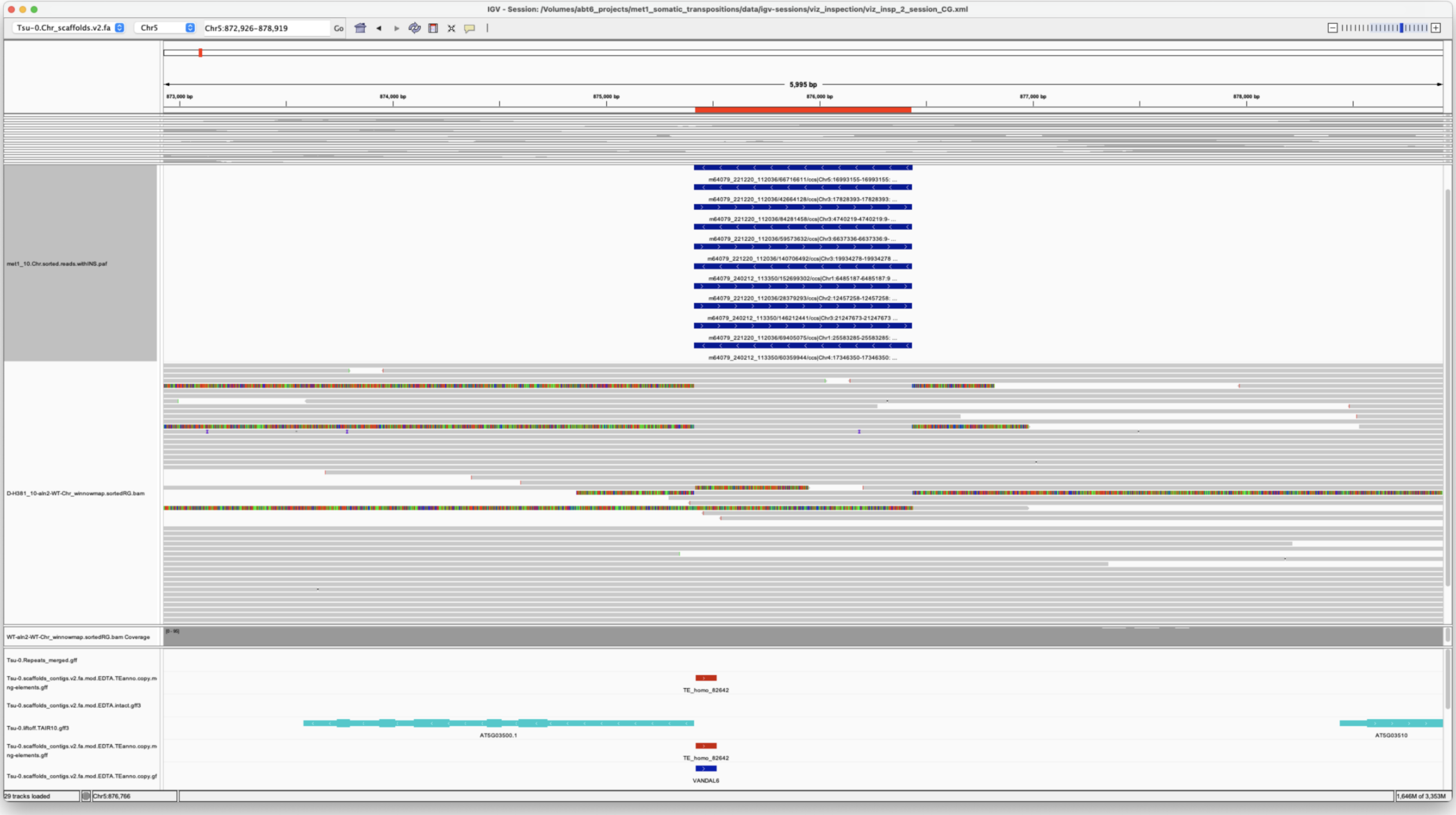
IGV - Session: /Volumes/abt6_projects/met1_somatic_transpositions/data/igv-sessions/viz_inspection/viz_insp_2_session_CG.xml
Tsu-0.Chr_scaffolds.v2.fa
Chr5
Chr5:872,926-878,919
5,995 bp
29 tracks loaded

Confirmed
