# Supplementary figures and images for: Long-read detection of transposable element mobilization in the soma of hypomethylated Arabidopsis thaliana individuals
Source: Genome Biol. 2025 Jul 30;26:231. doi: 10.1186/s13059-025-03691-7 (PMC12312487; doi:10.1186/s13059-025-03691-7)

**Chr1 11941106 11946436 ATCOPIA93\_Evade**

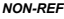

**REF**

**REF**  
with deletion in Col-0

**ATENSPM2 Chr2:8337982**

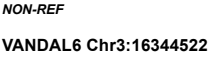

**NON-REF**

**VANDAL6 Chr3:16344522**





### CAC2A Chr5:8256939

Supplement: Supplementary file 2 — Additional file 2. Visual inspection of somatic insertion and excision events, available at https://github.com/aerilli/Somatic-transposition_met1/tree/551df407370c6528225f404ba62a073dced14b08/Supplementary-Files/Visual_inspection. [file 13059_2025_3691_MOESM2_ESM.gz › Split_Supplementary-File4/File_4-5_Synteny-check/File5_Synteny-check_Tsu-0-TEs-in-Col-0.pdf]
