# Supplementary material for: Long-read detection of transposable element mobilization in the soma of hypomethylated Arabidopsis thaliana individuals
Source: Genome Biol. 2025 Jul 30;26:231. doi: 10.1186/s13059-025-03691-7 (PMC12312487; doi:10.1186/s13059-025-03691-7)
Supplement: Supplementary file 5 — Additional file 5. PDF version of the present publication. [file 13059_2025_3691_MOESM5_ESM.pdf]

Long-read detection of transposable element mobilization in the soma of hypomethylated *Arabidopsis thaliana* individuals

Andrea Movilli[1], Svitlana Sushko[1], Fernando A. Rabanal[1], Detlef Weigel[1,2†]

[1]Department of Molecular Biology, Max Planck Institute for Biology Tübingen, 72076 Tübingen, Germany

[2]Institute for Bioinformatics and Medical Informatics, University of Tübingen, 72076 Tübingen, Germany

[†]For correspondence: weigel@tue.mpg.de

Abstract

Background

Because transposable elements (TEs) can cause heritable genetic changes, past work on TE mobility in *Arabidopsis thaliana* has mostly focused on new TE insertions in the germline of hypomethylated plants. It is, however, well-known that TEs can also be active in the soma, although the high-confidence detection of somatic events has been challenging.

Results

Here, we leverage the high accuracy of PacBio HiFi long reads to evaluate the somatic mobility of TEs in individuals of an *A. thaliana* non-reference strain lacking activity of METHYLTRANSFERASE1 (MET1), a major component of the DNA methylation maintenance machinery. Most somatically mobile families coincide with those found in germline studies of hypomethylated genotypes, although the exact TE copies differ. We also discover mobile elements that had been missed by standard TE annotation methods. Somatic TE activity is variable among individual plants, but also within TE families. Finally, our approach points to the possible involvement of alternative transposition as a cause for somatic hypermutability in a region that contains two closely spaced VANDAL21 elements.

Conclusions

Long-read sequencing reveals widespread TE transposition in the soma of *A. thaliana* hypomethylated mutants. Assessing somatic instead of germline mobilization is a fast and reliable method to investigate different aspects of TE mobility at the single plant level.

Background

Within the dynamics of ongoing evolutionary genomic conflict, TEs, being ‘genomic parasites’, should be silent – or at least minimally disruptive – in both somatic and germline cells of the host [1,2]. In animals, the germline is frequently specified early during development, thereby minimizing the number of cell divisions in the germline and also the opportunity for TEs to proliferate. In contrast, the separation between soma and germline is not as clear in plants, and the later and more plastic determination of the plant germline [3] allows for rare mutagenic events in the soma, including TE insertions, having a non-zero chance to be passed on to the next generation [4].

Germline mobilization of TEs in *Arabidopsis thaliana* has been studied both in epigenetic mutants [5–11] and in epigenetic recombinant inbred lines (epiRILs) [12–15], but there has been much less focus on assessing somatic TE mobilization. In addition to direct detection of mobile TEs by whole-genome sequencing, TE activity of long terminal repeat (LTR) transposons can be assessed by linear extrachromosomal DNA (ecDNA) identification [16], isolation of virus-like particles (VLP) [17] or extrachromosomal circular DNA molecules (eccDNAs) [10,18]. The latter technique has also allowed for detection of active non-LTR transposons [10].

A plethora of enzymes belonging to different pathways synergistically and with different degrees of redundancy establish TE repression in plants, through DNA methylation in all cytosine contexts, deposition of histone variants, and post-translational modification of histones [5,15,19–29]. The chromatin remodeller DECREASE IN DNA METHYLATION 1 (DDM1), which defines heterochromatin by depositing the H2A.W histone variant, mediates methyltransferases accessibility, ultimately establishing methylation in all cytosine contexts, CG, CHG and CHH, in *A. thaliana* [21,30,31]. On the other hand, METHYLTRANSFERASE1 (MET1) – a DNA methyltransferase 1 (DNMT1) homolog – is directly responsible for only CG methylation maintenance, and its disruption leads to massive gene expression changes and the stochastic generation of epialleles [32–34]. *MET1* activity can be further modulated by the genetic background and, as a consequence, mutations of *MET1* in different accessions cause phenotypes that range from near-wild-type to dwarves with reduced fertility and compromised survival [34]. Although much of the phenotypic variation due to loss of *MET1* activity is explained by the generation of epialleles [14], germline insertions in a range of genes have also been shown to be a cause for morphological defects in later-generation *met1* mutants and *met1*-derived epiRILs [12,15].

Despite there being hundreds of TE families, in *met1* and *met1*-derived epiRILs only a few TE families of both class I (“copy-and-paste” retrotransposons) and class II (“cut-and-paste” DNA transposons) become active. These are the ATCOPIA93 ÉVADÉ (EVD), CACTA/EnSpm (ATENSPM3) and Pack-TYPE CACTA elements [6,13–15,35]. Compared to inactivation of *MET1*, disruption of *DDM1* triggers much more extensive TE mobilization [5]. In addition the three families that become active in *met1* mutants, several Ty1/Copia families (ATCOPIA93, -13, -51, -63, -21, -31, ATRE1), several Ty3 families (ATGP3-1, -2, ATGP2N), CACTA/EnSpm elements (ATENSPM3), and MuDR elements (VANDAL21, ATMU1/5) transpose in *ddm1* mutants and *ddm1*-derived epiRILs [5,7,8,12,36]. Some of these TEs have also been found to mobilize in mutants defective in the RNA-dependent DNA methylation pathway (RdDM), which

is responsible for *de novo* DNA methylation of TEs [9,10], and in plants lacking all DNA methyltransferases [37].

A limitation of studying only *de novo* TE insertions that have survived selection during the haploid phase of the germline is that not all insertions can be passed on to the next generation. Biases in the detection of TE insertions may in turn result in important aspects of TE biology being overlooked. Moreover, the study of somatic TE mobilization is worthwhile in its own right given that it can have phenotypic consequences, which has been known since McClintock linked variegated pigmentation in the maternal layers of maize kernels to “controlling element” excision [38,39]. More recently, somatic TE activity has been suggested to underlie neuronal heterogeneity in diverse animals including humans, contributing to aging and disease pathophysiology [40–46]. A challenge has been the repetitive nature of TEs, which negatively impacts mappability of short reads [47]. In addition, chimeric artifacts introduced during Illumina library preparation can increase false positives for methods that rely on discordant paired-end reads [48]. In contrast, long-read sequencing technologies inherently mitigate multimapping issues, while enabling the identification of hallmarks of transpositions, *e.g.*, target site duplications (TSDs) [49]. Nevertheless, short-read approaches have been successfully employed to detect somatic transpositions, particularly when paired with targeted enrichment strategies, specialized experimental designs or tailored computational methods [46,50,51].

Here, we investigate whether highly accurate single-molecule long-read sequencing can be used to detect even very rare somatic TE transposition events in *A. thaliana*. Long-read technologies have been increasingly employed to study somatic transposition in other systems [44,46,49,51–53]. Because with this approach each sequence is read multiple times, the per-base error rates are low, potentially allowing for events that are present only on a single chromosome in a single cell of the sample to be detected. While this approach would have limitations for estimating single-base mutations, the longer sequences of TEs make a low per-base error rate acceptable. We demonstrate the potential of our approach in methylation-defective *A. thaliana* mutants by measuring somatic TE insertions and excisions in 10 first-generation *met1* siblings in a non-reference accession. We describe extensive interindividual, within- and between-family variation in TE mobilization and uncover family-specific biases. Finally, we identify a case of sequence hypermutability, probably driven by alternative transposition, in a region where two TEs are spaced closely apart.

Results

Detection of somatic TE mobilization events in *met1* single individuals

To investigate the extent to which TEs mobilize somatically in *A. thaliana*, we made use of *met1* mutants, which are known to have increased TE activity [14]. First, we *de novo* assembled the genome of the non-reference accession Tsu-0 (1001 Genomes Project ID: 7373) with PacBio HiFi reads, followed by annotation of protein-coding genes and TEs (Additional file 1: Table S1a,b, Fig. S1a,b, S2a-d). Next, we sequenced, also with PacBio HiFi reads, 10 first-generation Tsu-0 *met1* individual plants (Line 2, [34]) (Additional file 1: Fig. S3a-d, S4), obtaining on average a genome-wide coverage of 38x (Additional file 1: Fig. S3d).

To detect rare somatic transposition events, we mapped reads with Winnowmap2 [54] from *met1* individuals, and, as a control, from wild type, to the Tsu-0 genome. We identified candidate reads spanning new insertions of annotated TEs because they either (1) were flagged as “chimeric”, *i.e.*, had one or more discontiguous supplementary alignments (SA tag) in addition to a primary alignment, or (2) showed ≥500 bp insertions or deletions relative to the target genome, as reported in their CIGAR strings (Fig. 1a). CIGAR strings have been used before to assess structural variations (SVs) in *A. thaliana* mutants [10]. If either (1) one alignment of a “chimeric” read, or (2) the insertion/deletion section of a read mapped precisely to an annotated TE – *i.e.*, respecting the annotated TE borders –, we considered this *bona fide* evidence for a mobilization event (Fig. 1a, “read 2”, “read 3” and “read 4”). To reduce false positives, we visually inspected the genome alignments of reads describing candidate insertion/deletion events (Additional file 2 – File 1-3). First, we discarded all events at one locus, because an insertion of organellar DNA into the nuclear genome had been erroneously annotated as a TE (Additional file 2 – File 1). After visual inspection of candidate insertion events, we discarded 26 events, of which 15 were apparent rearrangements overlapping with a region that appeared to be highly variable in our *met1* mutants (Additional file 2 – File 1), which we decided to investigate further (see below). Of 23 candidate excisions events, we confirmed nine, while of the other fourteen, seven overlapped with satellite DNA rearrangements, two with the above-mentioned hypermutable region and five were considered deletions internal to TEs, and were therefore excluded (Additional file 2 – File 3).

When both ends of the *de novo* TE insertions could be resolved by (1) “chimeric” reads or (2) could be extracted from CIGAR strings (Fig. 1a, “central configuration”, “read 2” and “read 4”, see also Additional file 2 – File 1,2), which was the case for 42% of our candidate insertion

events (122/293), we could almost always identify apparent target site duplications (TSDs). This was the case for 119 out of 122 such events. While in one case the repetitive context at the insertion site of a read with a "central configuration" likely hindered TSD identification, we acknowledge that in the remaining two cases, the absence of confirmed TSDs may reflect mechanisms unrelated to endonuclease-dependent transpositions, *e.g.*, associated with DNA damage repair [55–57]. A similar uncertainty applies to reads with partial support, where transposition hallmarks could not be detected.

Although some insertion events lacked both-ended read support ("central configuration"), we believe that reads with partial mapping – if the boundary of the potential DNA insertion coincided exactly with an annotated TE border – can still represent *bona fide* somatic transposition events. While artifacts cannot be entirely ruled out, the absence of such reads in the wild-type sample and their consistency with TE boundaries support their validity. Other processes could generate partial mappings at TEs [55–57], however somatically inserted TEs are also expected to give rise to partial mappings and their number should increase as the TE length increases. In line with this, the fraction of insertion events with partial mappings that we detected were in line with the expected partial mapping fraction given their length (Additional file 1: Fig. S5). Therefore, partial mappings should not be automatically dismissed, especially for insertions of longer TEs that are expected to have a higher chance to be partially spanned during sequencing.

Our pipeline strongly relies on the annotation of TEs, and a limitation is that not all TEs might have been properly annotated (see Methods). To reduce false negatives introduced by lack of TE annotation of a mobilizing sequence, we took advantage of the fact that class II TEs are expected not only to transpose, but also to excise (Fig. 1a, "read 1" and "read 5"), as they move by a "cut-and-paste" mechanism [58]. Therefore, we also retained every read with global evidence for overlapping insertions and deletions of a specific sequence (Fig. 1a, "read 4" and "read 5"). In this manner, we recovered somatic mobilization events associated with elements that appear to be class II TEs that had been incorrectly annotated or missed altogether during TE annotation. Because this is a relatively specific approach, we might have missed somatic insertions of un-annotated TEs that are not mobilized by a "cut-and-paste" mechanism.

Overall we identified 362 reads that fulfilled our criteria for somatic transposition, which in total explained 293 independent insertions and nine deletions in the ten *met1* individuals (Fig. 1b, 2a), which, since they overlap with class II TE annotations, might correspond to repaired excision events. By contrast, we could not confirm any somatic mobilization in the wild type (Fig. 2a).

a)

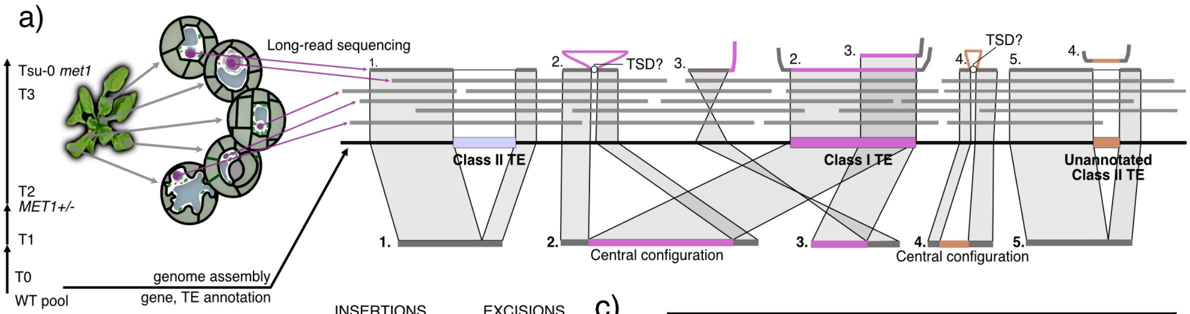

b)

TE COPY NAME (ABBREVIATED)		INSERTIONS Retained reads	Confirmed events	Events with partial config	Events with ≥1 central config	Events with confirmed TSD	EXCISIONS Confirmed events
**ATCOPIA93 ÉVADÉ**	EVD_1	129	79	40	39	39	-
	EVD_2	15	15	7	8	8	-
**Pack-Type MULE**	PAC	33	32	3	29	28	1
**ATENSPM2A**	CAC2A_1	2	1	1	-	-	-
	CAC2A_2	6	4	2	2	2	2
**ATDNAI27T9A**	ATDNAI27T9A	-	-	-	-	-	1
**VANDAL21**	V21_1	1	1	-	1	1	-
	V21_2	99	97	74	23	22	5
	V21_3	3	3	1	2	2	-
	V21_4	-	-	-	-	-	-
	V21_5	-	-	-	-	-	-
**VANDAL6**	V6_1	50	46	33	13	12	-
	V6_2	15	15	10	5	5	-

c)

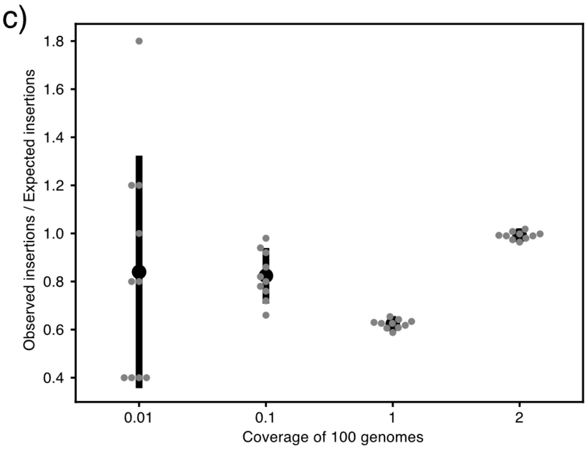

Figure 1. **Single-molecule method for detecting somatic TE insertions, excisions in *met1* mutants. a** Experimental design and diagram that show how different configurations of mapped long reads were used to call somatic mobilization events of TEs. The sequenced *met1* homozygous mutants were three generations away from the plants that were used for *de novo* genome assembly. **b** Numbers of retained candidate reads for insertions and number of non-redundant insertion and excision events. The region around V21_4 and V21_5 had signs of non canonical TE-mediated SV formation (see Fig. 5). **c** Observed over expected mock ÉVADÉ somatic insertions retrieved using simulated HiFi reads at different coverages. TE: transposable element, TSD: target site duplication, SV: structural variation.

Tsu-0 *met1* mutants at 6 weeks after germination have ~15 leaves (Additional file 1: Fig. S4), with a size comparable to a 3-week-old wild-type plant. Assuming that cell size is largely unaffected in *met1* mutants and that each leaf has ~20,000 cells [59], we estimated that we extracted DNA from a total of ~300,000 cells per *met1* individual. With an average of 421,821 mapped reads, each cell would have been roughly sampled once. Assuming in addition that there is an equal chance for a given TE to transpose during development, most of the mobilization events should happen at the end of development, when the plant has the maximum number of cells. As a consequence, most sampled TE mobilization events would occur relatively late during development, meaning that the vast majority of transpositions should only be present in one or a few cells and thus observed only once. This is indeed what we see, with 96% of apparent TE mobilization events being associated with a single read (Fig 1b, 2a).

Transposition events in the heterozygous T1 (or the wild-type) generation are unlikely, as we did not find events that were shared between the ten *met1* T3 individuals. Should TEs have mobilized in T2 gametes, gametophytes or earlier in the shoot apical meristem, we would have expected approximately 50% sequencing coverage in individual T3 plants. Similarly, high coverage could also be indicative of a transposition event very early during development. Only two of the events we found had approximately 50% coverage (23 reads/45 genome-wide coverage in met1_06 and 29/51 in met1_07) (Fig. 2a), suggesting the vast majority of detected events are somatic.

The detection power of our semi-automated method depended on multiple factors, including the length of overlap with the mobile TE, the genomic uniqueness and length of the TE, and the read length. To assess this complexity, we conducted an empirical test to estimate the probability of correctly identifying a TE insertion given a supporting read. In more detail, we randomly inserted 5 ATCOPIA93 ÉVADÉ TE sequences per genome in 100 concatenated *A. thaliana* Tsu-0 genomes, for a total of 500 insertions, and used these modified genomes to simulate PacBio HiFi reads at 2x genome-wide coverage using PBSIM3 software [60]. To evaluate the robustness of our method across different sequencing depths, the simulated reads were either subsampled beforehand or directly mapped to obtain genome-wide coverages of 2x of all 100 genomes (or 200x of a single genome), 1x, 0.1x, and 0.01x, and subsequently used to call somatic insertions (Fig. 1c). At 2x coverage of all 100 genomes, essentially all 500 randomly inserted EVDs were discovered. When coverage was more sparse (0.1x or 0.01x), our pipeline and the specifics of a randomly positioned read overlapping a given insertion together result in an ~80% chance of detecting that insertion. At 1x coverage of all 100 genomes, because of the random distribution of reads, only about two thirds of the theoretically possible maximum of 500 insertions were retrieved.

Our experiment corresponds to the sparse sampling situation, as described above, and we conclude that we were able to discover the vast majority of somatic transposition events for TEs with characteristics similar to those of ÉVADÉs.

High interindividual variability in active TEs of first-generation *met1* plants

We analyzed the annotations of the detected mobilization events in Tsu-0 *met1*s, finding a total of 13 distinct active TE copies (Fig. 1b) that had evidence of insertions, excisions or both.

Specifically, these included two ATCOPIA93 ÉVADÉ (EVD_1 and EVD_2) copies, three VANDAL21 (V21_1 to V21_3) copies, two VANDAL6 copies (V6_1 and V6_2), an uncharacterized non-autonomous element (PAC), two previously uncharacterized ATENSPM family members (CAC2A_1 and CAC2A_2) and a copy of ATDNAI27T9A, a MuDR element

with one somatic excision. Two adjacent VANDAL21 copies (V21_4 and V21_5) showed evidence of having caused many SVs, but no canonical somatic transpositions (see below).

Because we had generated independent PacBio HiFi libraries from 10 individual siblings, we could investigate interindividual variation in TE mobilization. The number of detected mobilization events in our individuals ranged from nine to 48, and there were up to 79 reads in a single individual supporting these events (Fig. 2a). To assess whether TE mobility differed significantly between individuals, we used a bootstrap approach to compare the observed insertion counts with a simulated distribution of expected positive reads, *i.e.*, reads that cover a mobilization event (Fig. 2b). Assuming that the true TE insertion frequency in a *met1* individual is close to the arithmetic mean in our sample of 10 individuals, we combined the reads from all 10 individuals and sampled with replacement 1,000 times reads corresponding to the average number of reads obtained from each individual. The results supported the conclusion that the differences in observed transposition events between *met1* individuals were very unlikely to be due to chance, especially for the individuals with few detected events, as their counts fall into the lower end of the simulation curve.

In addition, different TE copies showed different degrees of somatic mobilization in different sister plants (Fig. 2c). The mobilization of some families, such as VANDAL6 and VANDAL21, was more uniform than that of other TEs such as EVDs and PAC (Fig. 2c).

In some cases, we detected somatic excisions of full-length class II TEs, most likely underlying repaired excisions (Fig. 1b, Additional file 2 – File 3). For any given class II TE, somatic excisions were rarer compared to somatic insertions (Fig. 1b).

Somatic transposition as evidence for annotation of associated TEs

The somatic mobilization events we detected did not always overlap with TE annotations, which we had generated with the EDTA pipeline [61] based on a curated library of TE models of the Col-0 reference accession (https://github.com/oushujun/TAIR12-TE). We exploited this finding to refine our TE annotation, both in terms of correcting mis-annotated copies and annotating non-reference TEs missing from the curated library.

As an example, all VANDAL elements mobilizing in our experiments had been annotated as non-contiguous segments and were therefore merged. In addition, overlapping insertions and excisions helped us to identify two non-reference TEs that we curated using approaches proposed by Goubert and coauthors [62] (Additional file 1: Fig. S6). In one such case, we detected overlapping excisions and insertions of two 8.1 kb fragments that were nearly identical to other two fragments in the Tsu-0 genome (Additional file 1: Fig. S6a,b), all four of

which were predicted to encode an ATENSPM2-like transposase (Additional file 1: Fig. S6c,f). As these sequences did not comply with the 80-80-80 rule (≥80 bp total length, ≥80% identity over ≥80% of element [63,64]) for any of the *consensus* sequences in our Col-0-based library, we concluded that they belong to a new TE family that we call ATENSPM2A (CAC2A), with two actively mobilizing elements (Additional file 1: Fig. S6a-f). Since the CAC2A *consensus* is absent from the Col-0-based library, the EDTA pipeline apparently mis-annotated portions of CAC2As as fragments of different ATENSPM families (Additional file 1: Fig. S6a,g). Looking for matches to the Tsu-0 CAC2A *consensus* in the Col-0 reference genome, we found one copy that was apparently mis-annotated as a series of separate TEs, mirroring the fragmented EDTA annotation of Tsu-0, but lacking one of the terminal inverted repeats (TIRs) (Additional file 1: Fig. S6h), most likely compromising its mobilizability. With the PANTERA software [65], which can make use of polymorphic copies in different *A. thaliana* genomes (see Methods), we could *de novo* annotate four full copies of CAC2A in the Tsu-0 genome (Additional file 1: Fig. S6b).

A second instance of non-reference TE identification was a ~1 kb mobilizing element, for which we found several somatic insertions and one somatic excision (Additional file 1: Fig. S7a). This appears to be a Pack-TYPE element (here abbreviated as "PAC"). Pack-TYPE TEs are often missed during *de novo* TE annotation [66]. Tsu-0 PAC had two direct repeats that could potentially be targeted by VANC6 demethylases [67] (Additional file 1: Fig. S7b), and the mode of the TSD lengths extracted from its somatic insertions coincided with those of VANDAL elements in our dataset (Additional file 1: Fig. S7c). These observations point to the fact that PAC is a non-autonomous VANDAL transposon and possibly a Pack-TYPE MULE [68].

Taken together, these results highlight the detection of somatic mobilization as a source for discovering new elements and assisting TE annotation. In this manner, we could identify new TEs based on the ability of sequences to transpose, independently of sequence similarity to known elements.

Intraspecific variation of the mobilizable mobilome

We compared our inventory of mobile TEs in Tsu-0 *met1* mutants with a collection of TEs that have been reported to be active in the germline of *ddm1, met1* or epiRILs in the Col-0 reference accession [5–8,12,13,36]. Among the mobilizing TE copies in Tsu-0, only four were present at syntenic locations in Col-0, but none of these had been previously reported as mobile in *met1* and *ddm1* mutants and epiRILs (Fig. 2d). Likewise, none of the TEs that are mobile in Col-0 mutants were syntenic to TEs that are mobilizable in Tsu-0.

At the family level, we did not find evidence for mobilization of many Ty1/Copia, CACTA/EnSpm, Ty3 and MuDR elements, which often actively transpose in Col-0 *ddm1* epiRILs [8,10,12] (Fig. 2d). At the same time, we detected the mobilization of families either absent or previously unknown to mobilize in Col-0.

Both copies of intact full-length EVDs, ÉVADÉ and ATTRAPÉ (ATR), are activated in Col-0 epigenetic mutants, with ÉVADÉ arguably being the most active TE in the Col-0 background [12,15,35]. Tsu-0 has five EVDs that are highly similar to each other. Four of these were intact, but only two were mobile in our material (here abbreviated as EVD_1, EVD_2) (Fig. 1b,2a).

We found three copies of VANDAL21 (V21_1, V21_2, V21_3) and two copies of VANDAL6 (V6_1, V6_2) to be somatically mobilizing in Tsu-0. As mentioned before, we did not further consider a region in chromosome 5 with two head-to-head VANDAL21s (V21_4 and V21_5), which showed high somatic variability, but where we could not confidently call somatic transposition events. Of the mobilizing VANDAL21s in Tsu-0, only V21_1 and V6_2 were syntenic in Col-0 (Fig. 1b,2a).

We detected mobilization of two copies of ATENSPM2A, a previously uncharacterized ATENSPM/CACTA family member (Fig. 1b, Additional file 1: Fig. S6). A different ATENSPM/CACTA family member, ATENSPM3, becomes mobile in Col-0 *ddm1* and *met1* epiRILs [12] (Fig. 2a).

We found one mobilizing non-autonomous Pack-TYPE MULE [69], present in a single non-reference copy (Fig. 1b, Additional file 1: Fig. S7). While, to our knowledge, Pack-TYPE MULEs are not active in Col-0, non-autonomous Pack-TYPE CACTA elements have been reported to become mobile in *met1* epiRILs [13].

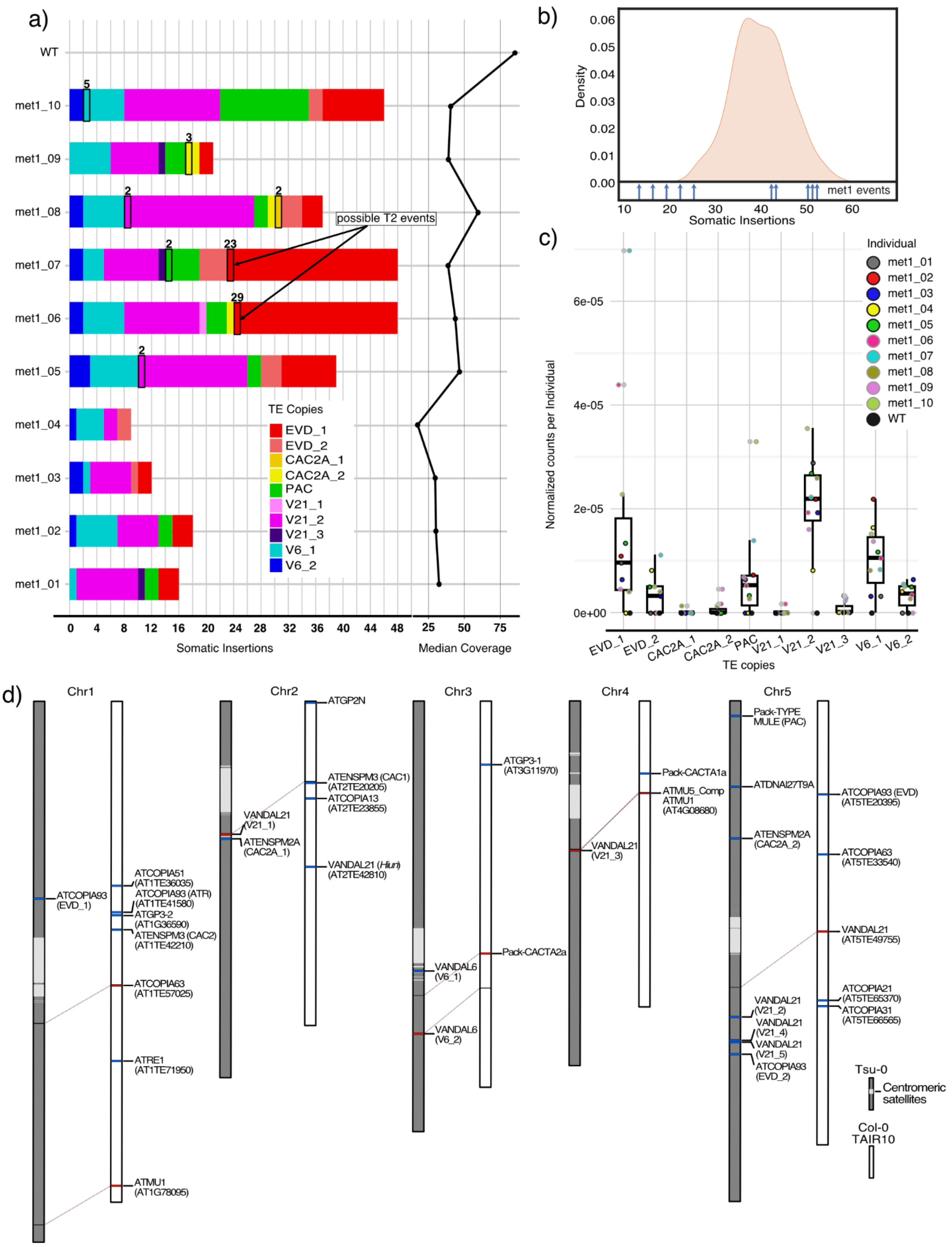
a)
WT
met1_10
met1_09
met1_08
met1_07
met1_06
met1_05
met1_04
met1_03
met1_02
met1_01
possible T2 events
TE Copies
EVD_1
EVD_2
CAC2A_1
CAC2A_2
PAC
V21_1
V21_2
V21_3
V6_1
V6_2
Somatic Insertions
Median Coverage
b)
Density
met1 events
Somatic Insertions
c)
Normalized counts per Individual
Individual
met1_01
met1_02
met1_03
met1_04
met1_05
met1_06
met1_07
met1_08
met1_09
met1_10
WT
TE copies
d)
Chr1
Chr2
Chr3
Chr4
Chr5
ATCOPIA93 (EVD_1)
ATCOPIA51 (AT1TE36035)
ATCOPIA93 (ATR) (AT1TE41580)
ATGP3-2 (AT1G36590)
ATENSPM3 (CAC2) (AT1TE42210)
ATCOPIA63 (AT1TE57025)
ATRE1 (AT1TE71950)
ATMU1 (AT1G78095)
ATGP2N
ATENSPM3 (CAC1) (AT2TE20205)
ATCOPIA13 (AT2TE23855)
VANDAL21 (V21_1)
ATENSPM2A (CAC2A_1)
VANDAL21 (Hiun) (AT2TE42810)
ATGP3-1 (AT3G11970)
Pack-CACTA2a
VANDAL6 (V6_1)
VANDAL6 (V6_2)
Pack-CACTA1a
ATMU5_Comp ATMU1 (AT4G08680)
VANDAL21 (V21_3)
Pack-TYPE MULE (PAC)
ATDNAI27T9A
ATENSPM2A (CAC2A_2)
VANDAL21 (V21_2)
VANDAL21 (V21_4)
VANDAL21 (V21_5)
ATCOPIA93 (EVD_2)
ATCOPIA93 (EVD) (AT5TE20395)
ATCOPIA63 (AT5TE33540)
VANDAL21 (AT5TE49755)
ATCOPIA21 (AT5TE65370)
ATCOPIA31 (AT5TE66565)
Tsu-0
Centromeric satellites
Col-0 TAIR10

Figure 2. **TE mobilization at the individual, family and species level. a** Stacked barplot indicating the raw count of somatic insertions in each *met1* individual and wild types. Events that were supported by multiple reads are framed by black rectangles and the number of reads supporting them are reported above. Median coverage for the *met1* individual sequencing is shown on the right. **b** Density plot of 1,000 counts of "reads explaining insertions" that were sampled with replacement *n* times, where *n* is the average number of reads per individual, from the entirety of reads from all *met1* individuals. The insertion count for the *met1* individuals is reported at the bottom of the plot with blue arrows. **c** Boxplot and jitterplot of individual insertion counts normalized by library size per different TE copy. **d** Double karyoplot showing the difference between somatically mobilizing copies in non-reference Tsu-0 accession (this study) (grey) and published mobile copies from TE mobilization germline studies in *met1*, *ddm1* mutants and epiRILs in the Col-0 reference background (TAIR10) [5–8,12,13] (white). Syntenic TEs are highlighted in red and a line links them to the position in the Tsu-0 genome. Non-syntenic TEs are highlighted in blue.

We also found one excision of ATDNAI27T9A, a MuDR TE for which we did not find any associated insertion. As host-mediated mechanisms could give rise to the same outcome through TE-independent activity, we were not confident in excluding it from being a false positive. Methylation at ATDNAI27T9A is highly sensitive to temperature [70] and it is mobile in Tsu-0 wild-type plants [9].

In conclusion, our approach can be used to investigate intraspecific variation of actively mobilizing TEs.

Potential explanations for different mobilizability of ÉVADÉ copies

The two actively mobilizing EVD copies on chromosomes 1 and 5, EVD_1 and EVD_2, are almost identical but were mobilizable to different extent (Fig. 2c). The third intact EVD copy on chromosome 4, EVD_3, showed no detectable mobilization in our material. Tsu-0 EVDs are highly similar in sequence not only to each other but also to Col-0 EVD and ATR (Fig. 3a), which mobilize to different extent as well [12]. Three additional non-mobile EVD copies on chromosome 3 of Tsu-0 were degenerate, with several frameshift mutations ("3:86", "3:11", "3:19"; Additional file 1: Fig. S8a).

As expected, identity between the two long terminal repeats (LTRs) seemed to be a necessary condition for EVD mobilization, as the LTRs of both mobile copies were 100% identical. The immobile EVD_3 was also intact, without ORF truncating mutations, and had 100% LTR identity (Fig. 3a), indicating that LTR identity and ORF intactness are necessary but not sufficient for mobilization of EVDs.

LTR retrotransposons show recombination patterns during mobilization bursts [71]. To check for recombination between newly inserted EVD_1 and EVD_2 transposons, we identified unique single nucleotide variants (SNVs) by mapping their sequence to Col-0 EVD sequence. We then identified SNVs for all the reads pointing to insertions of both EVD_1 and EVD_2, but

we could not find patterns of mixing of EVD_1 and EVD_2 diagnostic SNVs (Additional file 1: Fig. S9). Given this result, recombination between EVD_1 and EVD_2 prior to insertion could not be substantiated.

Re-mapping of published RNA-seq data [34] indicated a slightly higher expression level of EVD_2 than EVD_1 (Fig. 3b, Additional file 1: Fig. S8b) in Tsu-0 *met1*, but comparable accessibility and residual DNA methylation levels (Additional file 1: Fig. S8c,d). EVD_3 for which we did not find evidence of mobility in our material despite appearing to be intact at the sequence level, had the lowest expression (Fig. 3b), with the proviso that estimating expression levels of elements with closely related copies is inherently difficult.

To look into residual methylation as a driver for interindividual differences in EVD mobility, we extracted CG methylation profiles from HiFi circular consensus sequencing (CCS) reads of the mobile EVDs, EVD_1 and EVD_2. Residual CG methylation on EVDs was generally very low in *met1* compared to the wild type (Additional file 1: Fig. S8e). Across individuals, EVD_2, and less so EVD_1, showed an inverse correlation between residual CG-methylation in the GAG portion and mobilization level (Spearman correlation coefficient = -0.76/-0.51, p-value = 0.01/0.13) (Fig. 3c). This result is in line with EVD's GAG being methylated via translation-dependent RdDM [28] as a first line of defense against its pervasive mobility. EVD_1 as a whole showed a strong inverse correlation between total residual CG-methylation and mobilization level (Spearman correlation coefficient = -0.88, p-value < 0.001) (Fig. 3c).

Taken together, expression, accessibility and residual methylation are not good predictors for differential mobilization of EVDs in *met1* Tsu-0 mutants.

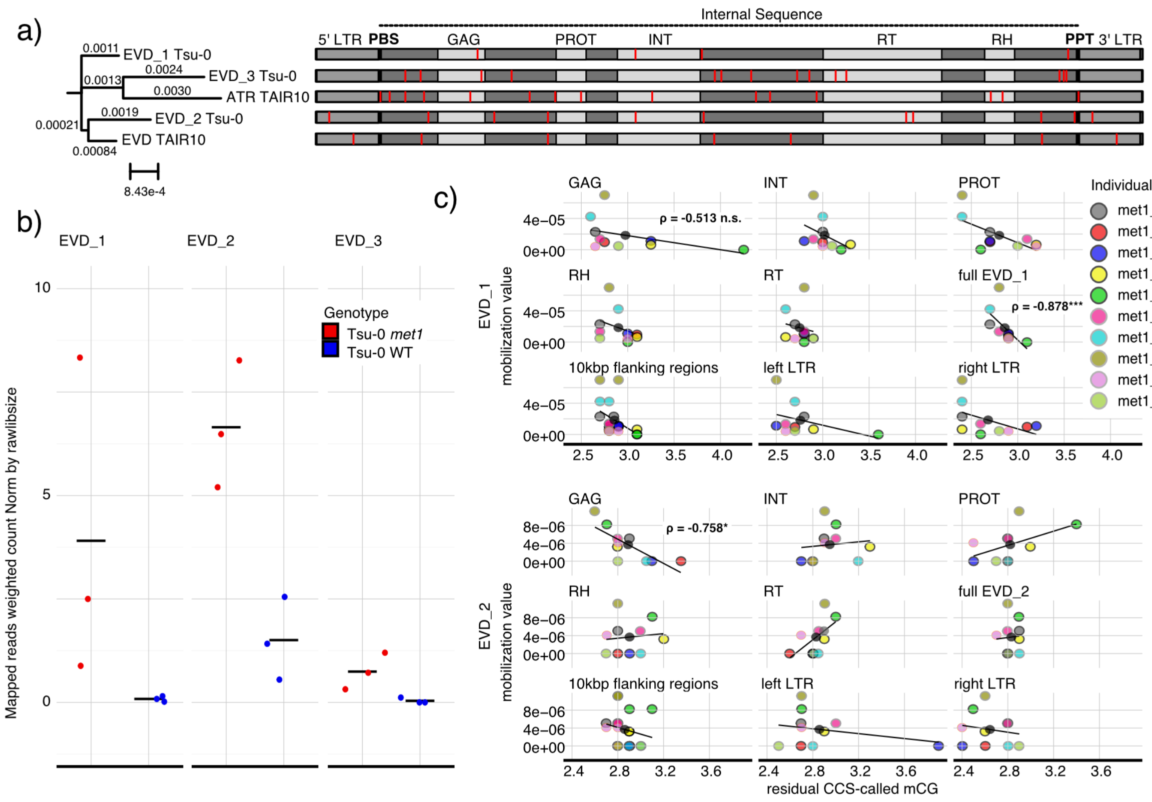

Figure 3. **Differential mobility of ATCOPIA93 ÉVADÉ copies. a** Phylogenetic tree (left) and multiple sequence alignment (right) of intact non-degenerate EVD copies in Tsu-0 and Col-0. LTRs (grey), primer binding site (PBS, black), polypurine tract (PPT) (black), and protein domains (GAG, PROT, INT, RT, RH) (light grey) are indicated. Substitutions are shown in red, small deletions in white. **b** Dotplot of expression levels of intact and non-degenerate Tsu-0 EVDs. Average is indicated as a black solid line. **c** Scatterplot with regression line of mobilization values, *i.e.*, individual counts normalized for library size as in Fig. 2c, as a function of residual PacBio HiFi-called CpG methylation for EVD_1 and EVD_2. Values are plotted for the full copies, LTRs, flanking sequences and sequences encoding for protein domains. Spearman correlation: * p-value<0.05; *** p-value<0.001; n.s., p-value≥0.05.

Somatic insertion preferences generally mirror germline bias

Actively mobilizing transposons did not show any evident bias towards or against specific regions of each chromosome, except for centromeres (Fig. 4a). We detected only five somatic transpositions within centromeric satellite repeats and three within centrophilic Athila elements in all chromosomes but chromosome 3 (Fig. 4a).

Concerning somatic insertion bias towards specific genomic features, we characterized the insertion sites (Fig. 4b) and measured their distance to the nearest gene, TE, and repeat type (rDNA, centromere, telomere, nuclear insertions of organellar DNA) (Additional file 1: Fig. S10a). We compared the insertion preferences with randomly generated insertion sites and with a random distribution of insertion site features assuming the arithmetic mean of all the insertions to be the true mean. Since ATENSPM insertions were too few, we could not assess

biases towards any particular genomic feature. For the other TE categories, we found the insertion preference towards genomic features to be different between EVDs, and VANDALs together with PAC (Fig. 4b; Additional file 1: Fig. S10a,b).

In our material, VANDALs preferentially inserted near genes, especially 5' UTRs and upstream regions, with PAC showing similar insertion preferences. EVDs showed a bias towards exons (Fig. 4b, Additional file 1: Fig. S10c), consistent with previous studies [12,36].

Somatic insertion preference of EVDs also mirrored the germline bias towards genes, especially non-essential genes [12] (Fig. 4c, Additional file 1: Fig. S10b), suggesting that this pattern might not be due to purifying selection at the cellular level.

Reads with single-ended read support – *i.e.*, lacking "Central configuration" – exhibited the expected insertion preferences, lending additional confidence to their validity in identifying insertion events (Additional file 1: Fig. S10d).

Sites disrupted by different TEs show different expression and accessibility

We assessed the expression of genes disrupted by somatic insertions in *met1* with published RNA-Seq data [34] by re-mapping reads of *met1* to our *de novo* assembled Tsu-0 genome. EVDs showed a bias towards genes with a significantly lower expression in Tsu-0 *met1* (Fig. 4d). In contrast, VANDALs and PACs more often transposed within genes with a higher expression level compared to EVDs, but not significantly different from random (Fig. 4d).

To test whether the pattern of somatic TE insertions reflects chromatin accessibility landscape, we made use of ATAC-Seq data generated for Tsu-0 *met1* [34]. We found that EVDs had opposing insertion preferences compared to the majority of TE families, as they were more likely to insert in less accessible regions, whereas the insertion sites of the other families were in general significantly more accessible (Fig. 4e). The similarity in insertion preferences between PACs and VANDALs were reflected in similar accessibility preferences (Fig. 4e). Accessibility of somatic insertion sites was not explained by residual DNA methylation, as deduced from CCS-called and BS-Seq methylation data (Additional file 1: Fig. S11a,b).

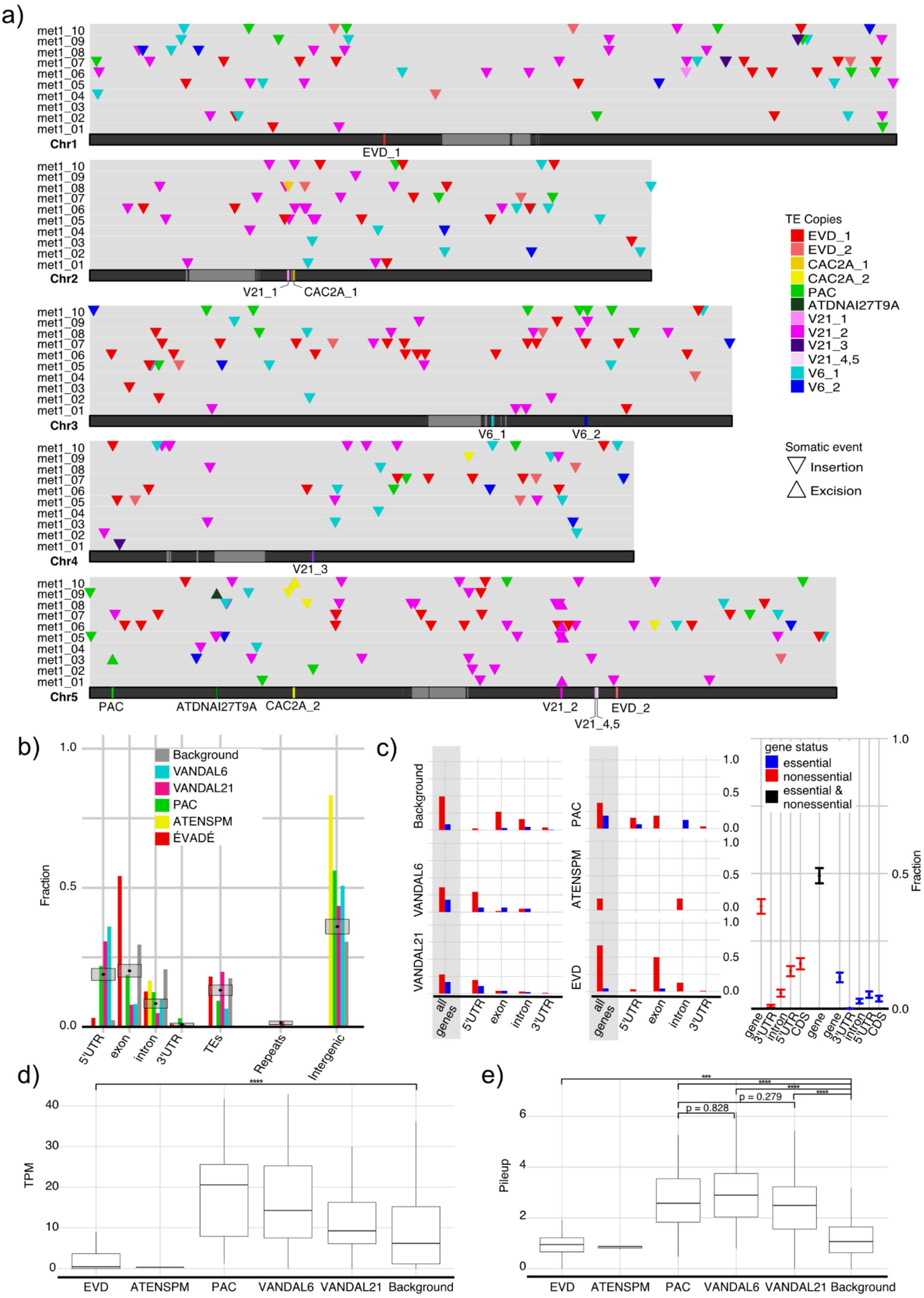
a)
met1_10
met1_09
met1_08
met1_07
met1_06
met1_05
met1_04
met1_03
met1_02
met1_01
Chr1
EVD_1
Chr2
V21_1
CAC2A_1
Chr3
V6_1
V6_2
Chr4
V21_3
Chr5
PAC
ATDNAI27T9A
CAC2A_2
V21_2
EVD_2
V21_4,5
TE Copies
EVD_1
EVD_2
CAC2A_1
CAC2A_2
PAC
ATDNAI27T9A
V21_1
V21_2
V21_3
V21_4,5
V6_1
V6_2
Somatic event
Insertion
Excision
b)
Fraction
Background
VANDAL6
VANDAL21
PAC
ATENSPM
ÉVADÉ
5'UTR
exon
intron
3'UTR
TEs
Repeats
Intergenic
c)
Background
VANDAL6
VANDAL21
PAC
ATENSPM
EVD
all genes
5'UTR
exon
intron
3'UTR
gene status
essential
nonessential
essential & nonessential
Fraction
gene
3'UTR
intron
5'UTR
CDS
d)
TPM
EVD
ATENSPM
PAC
VANDAL6
VANDAL21
Background
****
e)
Pileup
p = 0.828
p = 0.279
***
****

Figure 4. **Insertion preference in Tsu-0 *met1* mutants. a** Karyoplot of genome-wide somatic insertion landscape. Centromeric satellites in lighter grey. Mobile TE copies are highlighted with different colors, with insertions as downward pointing and excisions as upward pointing triangles. **b** Insertion preferences towards genomic features. "Background" describes the fraction of randomly generated sites. Grey boxes represent the random distributions of genomic features at somatic insertions generated by 1,000 iterations of sampling with replacement from the entirety of the *met1* reads. Black dots indicate the means. **c** Barplots of the somatic insertions in genes colored by "essentiality". "Background" describes the fraction of randomly generated insertions. On the right, distributions of "essentiality" of genes disrupted by mobilizing TEs generated by 1,000 iterations of sampling with replacement from the entirety of the *met1* reads. **d** Expression levels of genes disrupted by insertions in *met1*. "Background" represents randomly generated sites. Outliers are not shown. **e** Accessibility of insertion sites in *met1*. "Background" represents randomly generated sites. Outliers are not shown. ***Welch Two-sample t-test p-value<0.001; ****p-value<0.0001. Kolmogorov-Smirnov test was performed beforehand to test for normality. TPM, transcripts per million.

VANDAL21-associated SVs display patterns of alternative transposition

We found evidence of extensive somatic rearrangements in a region in chromosome 5 centered around two head-to-head VANDAL21s, which we abbreviated as V21_4 (left) and V21_5 (right) and which were separated by a ~200 bp spacer (Fig. 5). These two VANDAL21 copies disrupted a region with homology to a pseudogene of the TIR-NLR family in Col-0, AT5G40920 (Additional file 1: Fig. S12). Many reads with supplementary alignments ("chimeric" reads) mapped to this region (Fig. 5a), but visual inspection did not support the presence of *bona fide* transposition events, as there were no reads where both ends simultaneously mapped to the same region and included a complete or partial VANDAL21 element in their center, while respecting the TE boundaries (Additional file 2). The structural rearrangements in this region were diverse, but none could be straightforwardly associated with simple insertion/excision events. These SVs included inversions, deletions, apparent translocations where reads described a VANDAL21 being contiguous with a different region of the genome ("out-region") and "complex" SVs where a duplication and a deletion in addition to the inversion of the spacer co-occurred (Fig. 5b). The most often found SV was the deletion of a median of 185 bp spacer DNA between the two VANDAL21s (Fig. 5b, Additional file 1: Fig. S13a), also evident from a drop in read coverage (Fig. 5a), which did not always coincide with the borders of V21_4 and V21_5 (Additional file 1: Fig. S13b). In addition, there were excisions of V21_4 and V21_5 (Fig. 5b), four longer deletions comprising the left flanking region of V21_4, inversions of either V21_4, V21_5 or the internal spacer, along with other "complex" SVs (Fig. 5b). "Chimeric" reads mapping to this region very often spanned either V21_4 or V21_5 and the end of the mapping was right before or after the spacer sequence respectively (Fig. 5a). The break-end (BND) coordinates of those "chimeric" reads that described "out-region" non-local rearrangements (Fig. 5c) were located almost entirely on

chromosome 5, with more than 75% within a 10 Mb region around the head-to-head VANDAL21s (Fig. 5c), pointing to a spatial constraint. BND coordinates of “out-region” SVs also showed a bias towards 5’ UTRs (Fig. 5d), reminiscent of known VANDAL insertion preferences. In contrast, wild type showed no mapping of “chimeric” reads over the TEs, no spacer deletions and no other signatures of hypermutability (Additional file 1: Fig. S14).

It is plausible that the specific configuration and proximity of the two adjacent VANDAL21s could lead to alternative transposition, a phenomenon that has been extensively studied for Ac/Ds transposons in maize and which can generate deletions, duplications, composite insertions [72–74], inversions [75], as well as interchromosomal translocations [76]. It takes place during DNA replication, after multivalent transpososomes have bound to distinct elements, transposition intermediates are aberrantly resolved, generating different types of SVs in combination with DNA repair mechanisms [73]. As a result, the spacer between the transposons is circularized and lost [77]. Virtually all the SVs that were identified at this head-to-head VANDAL21 region could be explained by alternative transposition, although the reads were not long enough to fully resolve all the SVs including TSDs (Fig. 5b).

That alternative transposition could underlie the observed hypervariability of this region was supported by direct detection of the circular excised spacer using PCR with outward-facing primers (Fig. 5e), including when PCR was preceded by rolling circle amplification (RCA) (Additional file 1: Fig. S15a-c). This pointed to the presence of alternative transposition byproducts.

To search for additional alternative transposition events in our dataset, we looked for similar patterns of “chimeric” reads mapping to regions in which either two annotated class II TEs with the same classification or simply two TIR sequences – extracted from our TE *consensus* library – were arranged in a head-to-head fashion. We did not find any additional instances in either mutants or wild type, which perhaps is not surprising, given the greater activity of VANDAL21s compared to any other TE in our material.

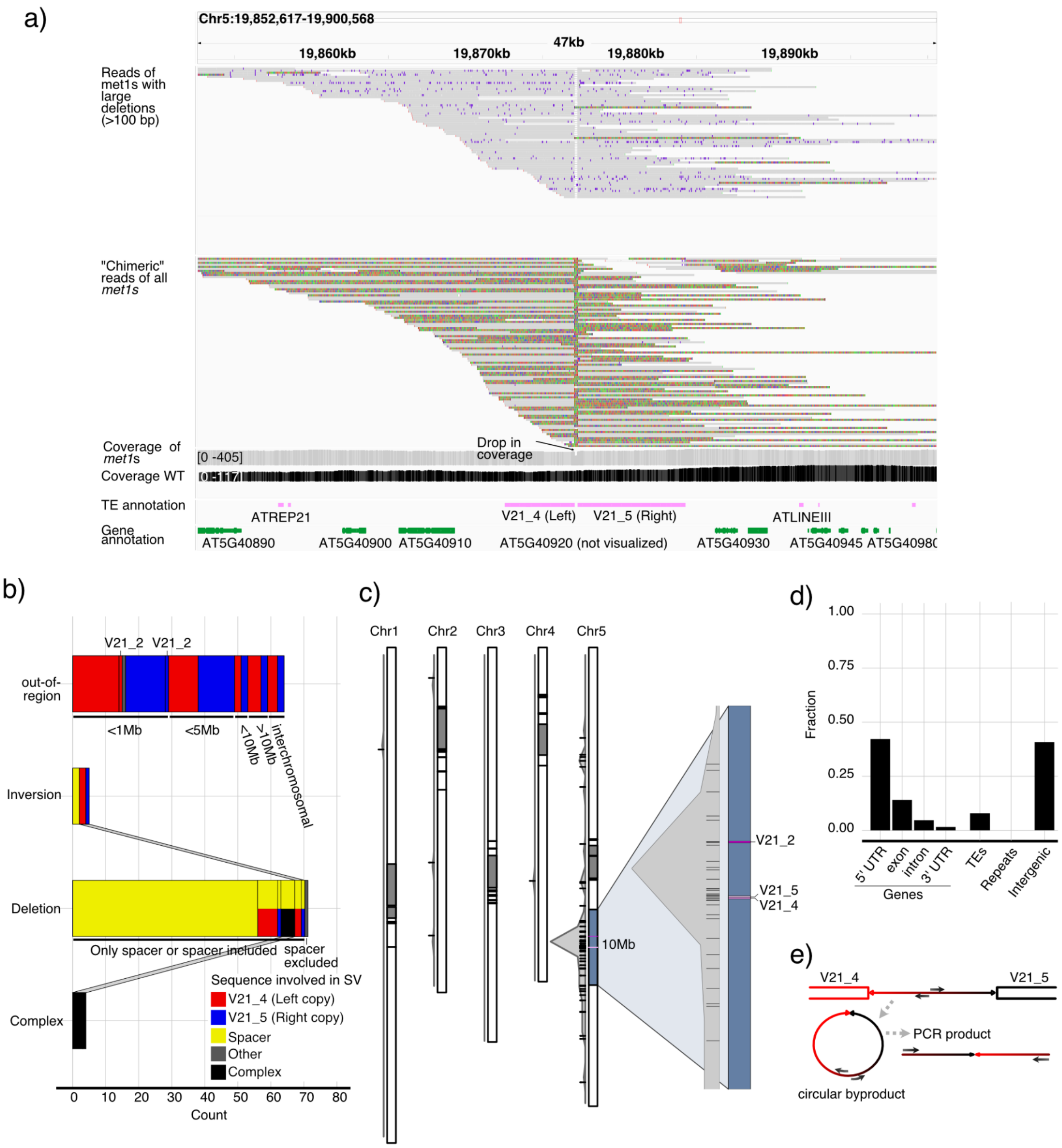

Figure 5. **Local VANDAL21-mediated somatic hypermutability. a** IGV screenshot of reads mapping to the hypermutable region. Tracks, from top: reads with spacer deletion, reads with supplementary alignment (colored portion) indicating putative somatic hypermutability, *met1* reads (unfiltered) coverage in grey, wild-type reads (unfiltered) in black, TE annotation in pink, gene annotation in green. **b** Counts of different classes of SVs overlapping with the region colored by involved sequence. The distance from the region and the mapping to the close V21_2 copy is indicated. It is specified if deletions contain the spacer or not. If a deletion involves two different sequences it is colored with two different colors. SVs that could be classified in two different ways are linked. **c** Karyoplot with density of BND mapping coordinates of "out-region" SVs not overlapping with the hypermutable region. On the right, close-up of the 10 Mb centered around V21_4 and V21_5. **d** Barplot of the fraction of genomic features overlapping with BND coordinates of "out-region" SVs. **e** Schematic representation of spacer circular DNA detection via PCR. SV: structural variation, BND: break-end.

Discussion

By long-read sequencing of genomic DNA from hypomethylated *met1* mutants in *Arabidopsis thaliana*, we detected somatic insertions and excisions of transposable element (TE) copies. The analysis of closely related siblings (Fig. 1a) allowed us to assess interindividual variation in TE mobility (Fig. 2a, 4a), while use of the non-reference Tsu-0 background revealed TEs absent or not mobile in Col-0 reference accession (Fig. 2d). Our approach also helped to generate support for an underappreciated aspect of TE biology, namely the hypermutability linked to alternative transposition, a phenomenon previously studied for Ac/Ds transposon in maize and snapdragon, and P-elements in Drosophila, but not in *A. thaliana* [78,79].

Plants have flexible germline specification, allowing the inheritance of somatic TE variations [4] and in some cases for clonal propagation. Even if somatically variable cells do not contribute to the germline, *i.e.*, becoming 'germ-track' – as per Haig's definition [1] –, they still can significantly impact phenotypes [38], making it important to assess their prevalence. Measuring somatic events in plants bypasses some biases found in germline studies. For instance, the plant life cycle, with its extended gametophytic phase [80], may act as a bottleneck for the transmission of deleterious mutations, limiting the passage of somatic transpositions to future generations. We classified all low-coverage TE mobilizations as "somatic" because it was unlikely that they had become fixed in the L2 layer, which gives rise to the gametes ("germ-track"). Only two insertions featured exceptionally high coverage, suggesting possible mobilization in the parental gametophyte, gamete or shoot apical meristem. Recently mobilized copies of a given TE may also be more likely to transpose again, potentially contributing to interindividual variation in transposition rates. Notably, the two individuals carrying these high-coverage insertions also exhibited the highest number of insertions for that TE.

As Siudeja, van de Beek and coauthors [51] have pointed out, long reads can span complete insertions, thus resolving both the central TE and their flanking sequences, intrinsically increasing confidence in detection of somatic events – even if supported only by single reads. Several aspects of our analyses further boost the confidence we have in our somatic insertion calls: (1) absence of mobilization in wild-type plants; (2) reads having been precisely mapped to full or partial TEs; (3) target site duplication (TSD) being detected in "chimeric" reads; and (4) mobilizing TEs belonging to families known to become active in the germline of epigenetic mutants.

In the soma of Tsu-0 *met1* mutants, we observed more TE copies mobilize compared to what has been reported for the germline of Col-0 *met1* mutants [14,15,32,33]. While this difference

may reflect true biological differences – potentially due to Tsu-0 belonging to a miRNA haplotype that reduces TE silencing efficiency [81] – this interpretation rests on the assumption that somatic and germline mobilization results are directly comparable, both in terms of biology and the methodologies used to evaluate them. In our dataset, the extent of TE mobilization seemed to vary across individuals (Fig. 2a), with only some elements showing high variation in mobilization rates (Fig. 2c). We confirmed previous findings on TE insertion preferences (Fig. 4b,c), such as VANDAL elements targeting 5' UTRs [36] and EVDs preferring non-essential genes as targets [12]. We detected centromeric insertions of ÉVADÉ and VANDAL TEs, probably as a consequence of changes in chromatin architecture change and increased accessibility of centromeres in *met1* mutants [82–84]. The insertion of EVDs into genes with lower expression levels may be associated with transcriptional repression associated with H2A.Z enrichment [85,86].

Beyond TE derepression, hypomethylation impairs genome stability more broadly, generating SVs [10] and TE-driven mechanisms may contribute to region-specific hypermutability [87]. We propose that alternative transposition between closely positioned VANDAL21 transposons (V21_4 and V21_5) may explain local hypermutability, similar to alternative transposition of Ac/Ds TEs in maize [73,79]. While VANDAL transposons are class II TEs, like Ac/Ds, they have not been linked to alternative transposition before. A VANDAL5-mediated paracentric inversion event has been suggested as having generated the polymorphic chromosome 4 heterochromatin knob in *A. thaliana* [88]. The proposed causal mechanism involves the 5' end of one of two proximal VANDAL5s having inserted into a distal gene while its 3' end remained attached to its origin [88]. An alternative transposition event could have been the underlying cause of this paracentric inversion in agreement with directly demonstrated mechanisms in maize [89].

The narrow 10 Mb break-end coordinates interval of V21_4 and V21_5 rearrangements (Fig. 5c) likely reflect positional constraints or reduced fitness of cells with extreme rearrangements, such as translocations or large deletions. These alternative transposition-driven SVs may contribute to the reduced viability of *met1* by destabilizing chromosome structure and accumulating DNA damage, which is prominent in severely dwarfed *met1* plants [90].

With our approach we detected non-autonomous and mis-annotated class II TEs not found in the Col-0 reference-based TE library. While the contribution to the improvement of TE annotations was small here, improvements might be more substantial in other plant species with more TE-rich genomes, especially those with prominent clonal propagation abilities, where somatic variation is intrinsically more impactful. For species where epigenetic mutants and epiRILs are difficult to obtain, this method could provide insights into TE mobility in cases where germline studies are impractical – which is the case even for some *A. thaliana* accessions [34].

Lastly, the lack of synteny in actively mobilizing TEs between Tsu-0 and Col-0 (Fig. 2d) suggests considerable diversity in the mobilizable mobilome of *A. thaliana*. Future efforts to explore this diversity at the species level are warranted.

Conclusions

With the use of long-read DNA sequencing of *A. thaliana met1* mutants we revealed widespread somatic TE transposition, with variable mobilization extents across individuals. Unlike traditional germline-focused approaches, methods such as ours enable the interrogation of the mobilome in mutant individuals already in the first generation, without the need for inherited events, and thus is applicable to infertile, semi-lethal genotypes. Our approach taught us new aspects of TE biology as, for instance, we found evidence for alternative transposition events as the cause for local hypermutability.

Methods

Plant material

Seeds of *Arabidopsis thaliana* Tsu-0 accession (1001 Genomes Project ID 7373) were sterilized with gaseous chlorine and stratified in 0.1% w/v agarose for 7 days at 4°C in the dark. Tsu-0 wild-type seeds from the pool used for transformation with the *MET1* targeted CRISPR-Cas9 guide construct in [34] were directly sown on soil. Because of their delayed development, Tsu-0 *met1* mutants (line 2, [34]) were first grown for 2 weeks on ½ strength MS agar plates supplemented with 1% w/v sucrose and then transferred on soil. Before harvest, plants were placed for 24 h in the dark to reduce chloroplast DNA. The aerial parts were harvested and snap-frozen with liquid nitrogen. Wild-type material was collected from pools of plants four weeks after germination and ten *met1* plants were collected individually three weeks after transfer on soil, at a stage of development comparable with that of the wild types. Mutant plants at harvest are shown in Additional file 1: Fig. S4.

DNA extraction, PacBio HiFi library preparation and sequencing

The plant material was finely ground with mortar and pestle in liquid nitrogen, and high-molecular-weight (HMW) DNA extraction was carried out as published [91].

HMW DNA was sheared with Covaris gTUBEs and was then directly used for PacBio HiFi SMRTbell library preparation, as specifically described for the Col-0 HiFi library in [91]. 5 µg of Tsu-0 wild-type DNA was used as input for HiFi SMRTbell library using SMRTbell Express

Template Prep Kit 2.0 with the barcode “bc1010”, multiplexed with two other libraries and sequenced on a single SMRTcell.

Sheared DNA from individual plants *met1_01* to *met1_10* (as they appear in Additional file 1: Fig. S4) was used as input for PCR amplification of *MET1* exon 7 [34]. Sanger sequencing of the PCR product was used to assay the known mutant sequence. Different amounts of input DNA (1480 - 5000 ng) were used for preparation of individual *met1* mutant HiFi SMRTbell libraries with SMRTbell Express Template Prep Kit 3.0 with 10 adapters “bc1001” to “bc1003” and “bc1008” to “bc1016”, sequenced on a single SMRTcell and resequenced on another SMRTcell multiplexed with other libraries. Prior to sequencing the final pooled libraries were size-selected on a BluePippin (SageScience) with 10 kb cutoff in a 0.75% DF Marker S1 High Pass 6–10 kb v3 gel cassette (Biozym). Sequencing was performed on the Sequel II system using Binding Kit 2.2 for wild-type material and Binding Kit 3.2 for mutants.

Genome assembly

High Fidelity (HiFi) reads for all samples were generated with the DeepConsensus pipeline (https://github.com/google/deepconsensus): generation of draft consensus sequences with pbccs v6.4.0 (`--min-rq=0.88`) (https://ccs.how/), alignment of subreads to the draft consensus sequence with v0.2.0, with DeepConsensus v1.2.0 run in CPU mode [92]. Assembly of the Tsu-0 genome was carried out with hifiasm v.0.19.2 (`-l0 -f0`) [93]. Contigs larger than 50 kb were scaffolded into chromosomes with RagTag v.2.1.0 (`scaffold -q 60 -f 30000 -i 0.5 --remove-small`) [94], using as reference genome a version of TAIR10 in which organellar DNA nuclear insertions, and repetitive sequences had been masked [91].

The contiguity of the genome assembly was assessed using Quast v5.2.0 [95] (https://github.com/ablab/quast). The completeness was checked with BUSCO v5.4.2 [96] (https://busco.ezlab.org) in ‘genome’ mode using ‘viridiplantae_odb10’ lineage gene models.

Annotation

Repeats

Repeats were annotated with a previously described pipeline that uses RepeatMasker [97] (http://www.repeatmasker.org) with a custom library to identify centromere satellite repeats, ribosomal RNA genes, and telomeres, and minimap2 [98] (https://lh3.github.io/minimap2/minimap2.html) to find nuclear insertions of organellar DNA [91].

Genes

Genes were lifted over to the Tsu-0 genome from the TAIR10 annotation (12 July 2019) with liftoff v1.6.3 [99] (https://github.com/agshumate/Liftoff), with `-copies` option and a list of features was provided for lift-over: 'CDS, exon, five_prime_UTR, gene, miRNA, mRNA, ncRNA, snoRNA, snRNA, three_prime_UTR'. Exclusively primary isoforms were included. Gene annotation was translated with gffread [100] (https://github.com/gpertea/gffread) and quality-checked via BUSCO v5.4.2 [96] (https://busco.ezlab.org) in 'protein' mode using 'viridiplantae_odb10' lineage.

Transposable elements

For primary TE annotation, we ran the EDTA v2.2 [101] (https://github.com/oushujun/EDTA) pipeline providing the Col-CC curated library (https://github.com/oushujun/TAIR12-TE). To overcome fragmentation of seemingly intact annotated TEs we increased the `-maxdiv` parameter from 3.5 to 5, as EDTA tries to combine RepeatMasker lines that are likely the same element (https://github.com/oushujun/EDTA/blob/v2.2.0/EDTA.pl#L694).

We applied a custom script to merge all elements in the TE annotation file that had the same name and were at most 1 bp apart (https://github.com/aerilli/Somatic-transposition_met1). TE annotations overlapping repeats (rDNA, centromere, telomere, nuclear insertions of organellar DNA) annotations were excluded.

We made use of LTRpred v1.1.0 [102] (https://github.com/HajkD/LTRpred) to calculate LTR identity and annotate primer binding site (PBS) and polypurine tract (PPT) of EVDs, and DANTE v0.1.9 [103] (https://github.com/kavonrtep/dante) to annotate domains. We used EMBOSS getorf (https://emboss.sourceforge.net/apps/cvs/emboss/apps/getorf.html) to predict orfs of the different EVDs as a measure of their degeneration state.

To determine whether improved TE annotation methods would substantially affect our results, we additionally ran PANTERA v0.2.1 [65] (https://github.com/piosierra/pantera) to detect TEs in a pangenome graph that had been generated from 67 *A. thaliana* genome assemblies from different accessions. TE models detected by PANTERA were classified by TEsorter v1.4.6 [104] (https://github.com/zhangrengang/TEsorter) and only the models with a confident classification were kept. Afterwards, the models were inspected using TEtrimmer v1.4.0 [105] (https://github.com/qjiangzhao/TEtrimmer) and new consensus sequences were built for some TE families. We added 93 non-redundant TE models detected by PANTERA to the Col-CC curated library and used this library to annotate the Tsu-0 genome with EDTA v2.2.1. Also, TE models generated by *de novo* EDTA annotation step that overlapped with a CDS or with more than 50% of a protein-coding gene were removed. By using this improved TE annotation we could detect single insertion events of few new TE copies.

Read simulation

To simulate HiFi reads of somatic TE insertions, we concatenated 100 Tsu-0 genomes and inserted the sequence of the Tsu-0 ÉVADÉ transposon (EVD_1) with seqkit v2.8.2 ‘mutate’ function [106] (https://bioinf.shenwei.me/seqkit/usage/#mutate) at 500 randomly generated positions, *i.e.*, on average 5 per genome. We then used the concatenated, modified genomes to run pbsim3 v3.0.4 software [60] (https://github.com/yukiteruono/pbsim3) as follows: `pbsim --strategy wgs --method errhmm --length-mean 15775.0 --length-sd 7500 --length-min 5000 --difference-ratio 22:45:33 --errhmm ./pbsim3/data/ERRHMM-SEQUEL.model --depth 2 --genome ${concatenated_genome_with_random_insertions} --accuracy-mean 0.9 --pass-num 7`. Length parameters were chosen to coincide with the specific details of our dataset. With the depth parameter set to ‘2’, the final apparent coverage would be around 200x since 100 concatenated genomes were used as input.

We then generated HiFi reads using pbccs v6.4.0 (https://ccs.how/) with ‘--all’ option and extracthifi v1.0.0 (https://github.com/PacificBiosciences/pbtk). We repeated this process ten times.

Somatic TE insertion and excision calling

HiFi reads, either simulated or from PacBio sequencing, were mapped to the Tsu-0 genome using winnowmap v2.03 [54] (https://github.com/marbl/Winnowmap), using `meryl count k=15`, `meryl print greater-than distinct=0.9998` and `winnowmap -Y -L -ax map-pb`. We used the sorted bam files as input for our somatic TE mobilization pipeline (https://github.com/aerilli/Somatic-transposition_met1). Candidate somatic insertions were initially recognized as reads with “Supplementary alignment”, *i.e.*, “chimeric” reads. Because the aligner sometimes does not generate supplementary alignments but describes insertions within CIGAR strings, we also made use of CIGAR strings, using an approach inspired by [10]. We did not attempt to detect somatic insertions that mapped to highly repetitive regions because of the inherent difficulties in read mappability and thus high likelihood of producing erroneous results.

In more detail, we first collected IDs of all reads that mapped to an annotated TE and then retained the IDs of reads that had a “Supplementary alignment” tag. Different sections of “chimeric” reads can be independently aligned to different regions of the genome and the “Supplementary alignment” tag is assigned to one of at least two independent alignments. We retained as reads from candidate insertions only those with at least 99% overlap of the annotated TE. Regions of a read with a mapping quality <50 were removed. In other words, the part of a “chimeric read” that maps to a TE should respect the TE boundaries and be fully

contained in the TE. Additional formatting steps are required to generate a file with TE insertion site, name, and position of the original TE copy and the IDs of the reads supporting the same insertion site. All candidate insertions, including possible TSDs, were visually inspected.

In a second step, we identified reads including newly inserted sequences ≥500 bp, as deduced from their CIGAR strings, and with mapping quality ≥50. We extracted the insertion sequences and re-mapped them to the genome with minimap2 v2.24 [98] (https://lh3.github.io/minimap2/minimap2.html) (`-ax map-pb`). We intersected the mappings with the TE annotation of the genome, followed by additional formatting steps. All candidate insertions except for the simulated ones were visually inspected. To check for TSD, we extracted sequences of the two borders (50bp) of the insertions from the associated read into a fasta file and sequences of a 100bp interval around insertion sites from the reference genome into a fasta file. Then we aligned the two fasta files to generate a dotplot with re-DOT-able software (https://bioinformatics.babraham.ac.uk/projects/redotable) with a 2bp window size. Dotplots were subsequently visually inspected.

From the two steps, we compiled a final curated list of confirmed somatic insertions for downstream analyses. We merged reads pointing to insertions within a 5bp range into the same event.

To retrieve somatic excisions, we retrieved the IDs of all the reads that had a deletion greater or equal than 500 bp, as described in the associated CIGAR string, filtered them by mapping quality (≥50) and intersected the filtered bam file with TE annotation. We extracted the coordinates of the deletions and checked for a 90% overlap with an annotated TE. All instances were visually inspected.

To minimize the annotation bias for un-annotated Class II TEs, we collected all reads from all *met1* plants that had "supplementary alignment" tag and indicated deletions ≥500 bp, and visually inspected them to assess whether we had missed any mobilizing TE.

Expected partial mappings

Across size intervals ranging from 500 bp to 10 kbp in 500 bp steps, we generated 10,000 random segments from the Tsu-0 genome. For each mutant and the wild type, we subsequently quantified the number of reads that mapped to the segments but failed to completely overlap.

Curation of non-reference TE families

The borders of VANDAL21_3 were redefined based on which sequence was subject to *de novo* somatic insertion, and multiple VANDALs with different names, as annotated by EDTA, were merged.

The PAC annotation was manually added based on the sequence that was subject to *de novo* somatic insertion and excision.

TSDs were retrieved using a custom bash script (https://github.com/aerilli/Somatic-transposition_met1) agnostic of the TSD length expected for different TE families. We compared with blastn v2.9.0 with default parameters the sequence of each read with the flanking sequences. As mapping of the sequences immediately flanking a TE can be misleading depending on the sequence context of the insertion site, we collected all putative TSDs shorter than 15 bp and determined the most often found lengths (modes) for each family.

For CAC2A curation, we used steps inspired by [62]. In brief, we used blastn to find additional copies in the genome, we extended the borders of the putative copies. To determine the exact borders, we inspected their multiple sequence alignment from mafft [107] https://github.com/GSLBiotech/mafft) `--adjustdirection --auto`. We produced a *consensus* with cons (EMBOSS), obtained its ORFs via getorf (EMBOSS) and ran pfam_scan.pl to identify its domains. We also ran TE-Aid (https://github.com/clemgoub/TE-Aid) to obtain additional information. Conserved domains were extracted using DANTE v0.1.9 (https://github.com/kavonrtep/dante) and sequences were aligned with mafft `--auto`. Multiple sequence alignments and iqtree v2.3.0 [108] (https://github.com/iqtree/iqtree2) (`-bb 1000`) were used to build trees of the full sequences of CAC2A *consensus* and other CACTA/EnSpm (https://github.com/oushujun/TAIR12-TE) and of their extracted domains.

Synteny of mobilizable TEs in different A. thaliana accessions

To determine whether TEs that are mobilizable in Col-0 are found in syntenic positions in Tsu-0,we collected TAIR10 positions of TEs that have been reported as mobilizable in Col-0 *met1* and *ddm1* mutants and in epiRILs from [5–8,12,13,36]. We manually checked whether the presence of such TEs at the same positions in the Tsu-0 genome was supported in a whole-genome alignment (WGA) of Tsu-0 and Col-0 TAIR10 that we had generated with wfmash v0.12.6-0-g7205bf7 (https://github.com/waveygang/wfmash) (`wfmash -a -s 10000 -n 1 -p 90 -k 19 -H 0.001 --hg-filter-ani-diff 30`) (Additional file 2 – File 4,5). To determine whether Tsu-0 *met1* mobilizable TEs were found in syntenic positions in TAIR10, we extracted the TEs with

500 bp upstream and downstream flanking sequences and mapped these to TAIR10 using blastn with default parameters.

Recombination of mobilizing ÉVADÉ transposons

We mapped EVD_1, EVD_2 and all the reads that explain insertions of either EVD_1 or EVD_2 to Col-0 EVD with minimap2 (`-ax asm5 --eqx`). We called variants bcftools v1.21 (https://samtools.github.io/bcftools/bcftools.html) (`bcftools mpileup`;`bcftools call -vm`). For EVD_1 and EVD_2 variant-calling, we retained unique variants, *i.e.*, not shared between EVD_1 and EVD_2. For each read, we recorded the overlapping of their variants with unique variants of EVD_1 or EVD_2 with bcftools isec (`-n~11`).

Estimated distribution of detected somatic insertions

We used a bootstrap approach to estimate how many detected somatic insertions we should expect from our sample. We drew with replacement a sample of *n* reads, with *n* equaling the average reads per *met1* individual, from the pool of total reads of all the *met1* individuals, and tallied how often we retrieved a read that corresponded to an observed somatic insertion. We repeated this process 1,000 times.

DNA methylation

BS-Seq

BS-Seq reads of the Tsu-0 *met1* line 2 [34] were analyzed as before [34] with minor differences: reads were trimmed using skewer v0.2.2 [109] (https://github.com/relipmoc/skewer) and processed using Bismark suite v0.23.1 [110] (https://github.com/FelixKrueger/Bismark). Reads were aligned to the newly assembled Tsu-0 genome, bam files were deduplicated, methylation calls in all contexts were extracted and the output was converted to BedGraph format for downstream analyses.

PacBio CG methylation calling

PacBio HiFi reads were extracted from the sequencing movies (PacBio raw format) of *met1* individuals via pbccs (`--all --hifi-kinetics`) and extracthifi, demultiplexed with lima v2.6.0 (https://github.com/PacificBiosciences/pbbioconda) (`--same --ccs --min-score 70 --min-scoring-regions 2 --min-ref-span 0.8 --peek-guess --split-bam-named`). We called cytosine methylation for every *met1* individual using jasmine v2.0.0 (https://github.com/PacificBiosciences/pbbioconda), mapped them to the Tsu-0 reference genome using the 'align' option of pbmm2 v1.10.0

(https://github.com/PacificBiosciences/pbbioconda), and generated site methylation probabilities with the ‘aligned_bam_to_cpg_scores’ tool v2.3.2 from pb-CpG-tools suite (https://github.com/PacificBiosciences/pb-CpG-tools) using the provided model.

Chromatin accessibility

ATAC-Seq reads of Tsu-0 *met1* line 2 [34] were analyzed as before [34] with minor differences: reads were trimmed using skewer v0.2.2 and mapped to the newly assembled Tsu-0 genome using bowtie2 v2.3.5.1 [111] (https://github.com/BenLangmead/bowtie2) and duplicates were marked with picard ‘MarkDuplicates’ v2.27.5 (https://broadinstitute.github.io/picard), we then called ATAC-seq peaks independently for the three replicates with total gDNA as control with macs2 ‘callpeak’ v2.2.9.1 [112] (https://github.com/macs3-project/MACS) (`--nomodel --extsize 147 --keep-dup=all -g 1.35e`). “Treatment” pile-up BedGraph output files were used for downstream analyses.

RNA-expression

RNA-Seq data of Tsu-0 *met1* line 2 [34] were mapped to the newly assembled Tsu-0 genome and transcripts per million (TPM) were extracted as before [34].

To estimate expression of highly identical ÉVADÉ (EVD) copies, we mapped RNA-Seq reads of Tsu-0 wild-type and *met1* line 1,2 [34] with bowtie2 `--mp 13 --rdg 8,5 --rfg 8,5 --very-sensitive` to the newly assembled Tsu-0 genome. We estimated the expression of EVD_1,2,3 as the summation of the mapping qualities of each mapping read divided by the maximum mapping quality (60). In other words, a mapped read was counted as 1 only if its mapping quality was 60 and it could be counted as 0.5 if, for instance, its mapping quality was 30. We then normalized the resulting values by raw library size.

Inspection of candidate reads from alternative transposition events

We collected mapped HiFi reads from the *met1* individuals that overlapped the VANDAL21 hypermutable region, and the up- and down- stream 20 kb flanking regions. The subsetting of reads from the merged BAM files ensured that IGV would not crash. We extracted reads with supplementary alignments, *i.e.*, reads that were “chimeric” (SAM flag ‘2048’) and had deletions ≥100 bp according to their CIGAR string. We then inspected the reads in IGV and classified them according to the structural variation that they supported. When one portion of a “chimeric” read mapped outside the hypermutable region or its flanking 20 kb regions, we recorded its break-end (BND) coordinate.

Insertion preferences

Genomic features

We intersected the final curated list of confirmed somatic insertions, VANDAL21 BND coordinates and 10,000 randomly generated insertion sites with the annotations of genes, TEs and repeats, after TEs overlapping repeats and genes overlapping TEs had been removed. Insertions not overlapping with any of these features were labeled as "intergenic". When an insertion occurred within a gene, we determined whether this gene was present in a list of lethal genes [113].

We used a bootstrap approach to randomly resample the features and essentiality of genes overlapping insertion sites to estimate the true distribution of such insertions (https://github.com/aerilli/Somatic-transposition_met1).

We also determined the feature that is the closest downstream and upstream (gene, TE), or just closest (repeat), to any somatic or random insertion site, or to any VANDAL21 BND.

Methylation bias

To evaluate residual methylation of sites in *met1* mutant where TEs somatically inserted, we assessed the distribution of CG, CHG, CHH (BS-seq) or CG (HiFi reads) methylation values of *met1* at confirmed somatic insertion sites, and up- and down- stream 25 bp.

ATAC-Seq analysis

To evaluate the accessibility of sites where TEs somatically inserted, we assessed the distribution of the average of three replicates of "Treatment" pile-up values at confirmed somatic insertion sites, and up- and down- stream 25 bp in *met1*. Outliers were excluded from plotted distributions.

RNA-Seq analysis

We evaluated the expression (TPM) of genes overlapping with the position of the confirmed somatic insertions – *i.e.,* the genes that were somatically disrupted by a TE insertion – and of 10,000 randomly generated insertion sites in *met1*. Outliers were excluded from plotted distributions.

Circular DNA detection

The aerial parts of one Tsu-0 *met1* mutant (line 2, [34]) were finely ground with mortar and pestle in liquid nitrogen. DNA extraction was carried out with a standard CTAB protocol,

including one hour incubation in CTAB at 65°C followed by chloroform:IAA (24:1) precipitation. We performed genotyping via PCR amplification of *MET1* exon 7 [34] followed by digestion with Hpy188I (NEB), which selectively recognizes the substituted “T” of the mutant allele. We used the DNA of the mutant as input for rolling circle amplification (RCA) as published (dx.doi.org/10.17504/protocols.io.bz8np9ve), with EquiPhi29 DNA Polymerase (ThermoFisher) and a reaction temperature of 42°C. We detected circular DNA by PCR amplification with outward facing primers (Additional file 1: Table S2) using either *met1* genomic DNA or the RCA reaction as input. We cloned the PCR bands in p-GEM-T Easy vectors (Promega) and confirmed their identity via Sanger-sequencing.

Softwares, visualization and statistics

For statistical analyses and visualizations we used RStudio Pro 2024.04.2 with R v4.4.0 and jupyterlab v4.0.7 with Python v3.10.13. Read mappings were visualized in IGV v2.16. Multiple sequence alignments were visualized in AliView v1.28.

Supplementary Information

Additional file 1: Fig. S1-S15, Table S1-S2.

Additional file 2. Visual inspection of somatic insertion and excision events, available at https://github.com/aerilli/Somatic-transposition_met1/tree/551df407370c6528225f404ba62a073dced14b08/Supplementary-Files/Visual_inspection.

Additional file 3: Dataset S1. List of curated transposable element insertions and excisions in ten Tsu-0 *met1* mutants.

Additional file 4: Dataset S2. List of curated 'out-region' BND coordinates at Tsu-0 hypermutable VANDAL21 region.

Additional file 5. PDF version of the present publication.

Acknowledgements

We acknowledge Haijun Liu and Magnus Nordborg for contributing to the initial idea of the project. We thank for their valuable feedback Pierre Baduel, Zhigui Bao, Ilja Bezrukov, Michael Borg, Mireia Bueno-Merino, Adrián Contreras-Garrido, Rory J. Craig, Erica Dinatale, Hajk-Georg Drost, Alejandra Duque-Jaramillo, Li He, Miriam Lucke, Arturo Marí-Ordoñez, Kevin D Murray, Gal Ofir, Luisa Pallares, Leandro Quadrana, Sheila Roitman, Rebecca Schwab, Gautam Shirsekar, Thanvi Srikant, Yueqi Tao, Luisa Teasdale, Marja Timmermans, and Wenfei Xian. We thank Christa Lanz and Dafne Ibarra-Morales for helping during PacBio HiFi library preparation.

A.M. is member of the International Max Planck Research School 'From Molecules to Organisms'.

Authors' contributions

A.M. and D.W conceived the project. A.M. performed all the experimental work and bioinformatic analyses, except for HiFi reads generation and genome assembly (F.A.R.), and additional TE annotation (S.S.). A.M. and D.W. interpreted the data and wrote the manuscript. All authors read and approved the final manuscript.

Funding

This study was supported by the Max Planck Society and the Novozymes Prize of the Novo Nordisk Foundation (D.W.).

Availability of data and materials

The PacBio HiFi reads generated for this study are available in the European Nucleotide Archive (ENA, https://www.ebi.ac.uk/ena/browser/home) under project name PRJEB85646 [114]. Additional files and scripts are available in GitHub at https://github.com/aerilli/Somatic-transposition_met1 [115] and are archived in Zenodo [116].

Reads from ATAC-Seq, RNA-Seq and BS-Seq experiments were obtained for *met1* mutant Line 2 (“Tsu-0_P2”) from PRJEB53354 [117], PRJEB54034 [118] and PRJEB54036 [119] respectively.

Ethics approval and consent to participate

Not applicable.

Competing interests

D.W. holds equity in Computomics, which advises plant breeders. D.W. also consults for KWS SE, a globally active plant breeder and seed producer. The other authors declare no competing interests.

Peer review information

Leandro Quadrana and Tim Sands the primary editors of this article and managed its editorial process and peer review in collaboration with the rest of the editorial team. The peer-review history is available in the online version of this article.

References

1. Haig D. Transposable elements: Self-seekers of the germline, team-players of the soma. Bioessays. 2016;38:1158–66.

2. Chang C, Pagano DJ, Lowe DD, Kennedy S. DNA repair strategy sets transposon

mobilization rates in Caenorhabditis elegans. bioRxiv. 2022;2022.08.01.502254.

3. Berger F, Twell D. Germline specification and function in plants. Annu Rev Plant Biol. 2011;62:461–84.

4. Bourque G, Burns KH, Gehring M, Gorbunova V, Seluanov A, Hammell M, et al. Ten things you should know about transposable elements. Genome Biol. 2018;19:199.

5. Tsukahara S, Kobayashi A, Kawabe A, Mathieu O, Miura A, Kakutani T. Bursts of retrotransposition reproduced in Arabidopsis. Nature. 2009;461:423–6.

6. Kato M, Takashima K, Kakutani T. Epigenetic control of CACTA transposon mobility in Arabidopsis thaliana. Genetics. 2004;168:961–9.

7. Miura A, Yonebayashi S, Watanabe K, Toyama T, Shimada H, Kakutani T. Mobilization of transposons by a mutation abolishing full DNA methylation in Arabidopsis. Nature. 2001;411:212–4.

8. Singer T, Yordan C, Martienssen RA. Robertson's Mutator transposons in A. thaliana are regulated by the chromatin-remodeling gene Decrease in DNA Methylation (DDM1). Genes Dev. 2001;15:591–602.

9. Baduel P, Leduque B, Ignace A, Gy I, Gil J Jr, Loudet O, et al. Genetic and environmental modulation of transposition shapes the evolutionary potential of Arabidopsis thaliana. Genome Biol. 2021;22:138.

10. Zhang P, Mbodj A, Soundiramourtty A, Llauro C, Ghesquière A, Ingouff M, et al. Extrachromosomal circular DNA and structural variants highlight genome instability in Arabidopsis epigenetic mutants. Nat Commun. 2023;14:5236.

11. Vendrell-Mir P, Leduque B, Quadrana L. Ultra-sensitive detection of transposon insertions across multiple families by transposable element display sequencing. bioRxiv. 2024;2024.08.21.608910.

12. Quadrana L, Etcheverry M, Gilly A, Caillieux E, Madoui M-A, Guy J, et al. Transposition favors the generation of large effect mutations that may facilitate rapid adaption. Nat Commun. 2019;10:3421.

13. Catoni M, Jonesman T, Cerruti E, Paszkowski J. Mobilization of Pack-CACTA transposons in Arabidopsis suggests the mechanism of gene shuffling. Nucleic Acids Res. 2019;47:1311–20.

14. Reinders J, Wulff BBH, Mirouze M, Marí-Ordóñez A, Dapp M, Rozhon W, et al. Compromised stability of DNA methylation and transposon immobilization in mosaic Arabidopsis epigenomes. Genes Dev. 2009;23:939–50.

15. Mirouze M, Reinders J, Bucher E, Nishimura T, Schneeberger K, Ossowski S, et al. Selective epigenetic control of retrotransposition in Arabidopsis. Nature. 2009;461:427–30.

16. Cho J, Benoit M, Catoni M, Drost H-G, Brestovitsky A, Oosterbeek M, et al. Sensitive detection of pre-integration intermediates of long terminal repeat retrotransposons in crop plants. Nat Plants. 2019;5:26–33.

17. Lee SC, Ernst E, Berube B, Borges F, Parent J-S, Ledon P, et al. Arabidopsis retrotransposon virus-like particles and their regulation by epigenetically activated small RNA. Genome Res. 2020;30:576–88.

18. Merkulov P, Egorova E, Kirov I. Composition and structure of Arabidopsis thaliana extrachromosomal circular DNAs revealed by nanopore sequencing. Plants. 2023;12:2178.

19. Zhang H, Lang Z, Zhu J-K. Dynamics and function of DNA methylation in plants. Nat Rev Mol Cell Biol. 2018;19:489–506.

20. Stroud H, Greenberg MVC, Feng S, Bernatavichute YV, Jacobsen SE. Comprehensive analysis of silencing mutants reveals complex regulation of the Arabidopsis methylome. Cell. 2013;152:352–64.

21. Osakabe A, Jamge B, Axelsson E, Montgomery SA, Akimcheva S, Kuehn AL, et al. The chromatin remodeler DDM1 prevents transposon mobility through deposition of histone variant H2A.W. Nat Cell Biol. 2021;23:391–400.

22. Deleris A, Stroud H, Bernatavichute Y, Johnson E, Klein G, Schubert D, et al. Loss of the DNA methyltransferase MET1 Induces H3K9 hypermethylation at PcG target genes and redistribution of H3K27 trimethylation to transposons in Arabidopsis thaliana. PLoS Genet. 2012;8:e1003062.

23. Lippman Z, May B, Yordan C, Singer T, Martienssen R. Distinct mechanisms determine transposon inheritance and methylation via small interfering RNA and histone modification. PLoS Biol. 2003;1:e67.

24. Sasaki E, Gunis J, Reichardt-Gomez I, Nizhynska V, Nordborg M. Conditional GWAS of non-CG transposon methylation in Arabidopsis thaliana reveals major polymorphisms in five genes. PLoS Genet. 2022;18:e1010345.

25. Creasey KM, Zhai J, Borges F, Van Ex F, Regulski M, Meyers BC, et al. miRNAs trigger widespread epigenetically activated siRNAs from transposons in Arabidopsis. Nature. 2014;508:411–5.

26. To TK, Yamasaki C, Oda S, Tominaga S, Kobayashi A, Tarutani Y, et al. Local and global crosstalk among heterochromatin marks drives DNA methylome patterning in Arabidopsis. Nat Commun. 2022;13:861.

27. Déléris A, Berger F, Duharcourt S. Role of Polycomb in the control of transposable elements. Trends Genet. 2021;37:882–9.

28. Oberlin S, Rajeswaran R, Trasser M, Barragán-Borrero V, Schon MA, Plotnikova A, et al. Innate, translation-dependent silencing of an invasive transposon in Arabidopsis. EMBO Rep. 2022;23:e53400.

29. Hure V, Piron-Prunier F, Yehouessi T, Vitte C, Kornienko AE, Adam G, et al. Alternative silencing states of transposable elements in Arabidopsis associated with H3K27me3. Genome Biol. 2025;26:11.

30. Osakabe A, Takizawa Y, Horikoshi N, Hatazawa S, Negishi L, Sato S, et al. Molecular and structural basis of the chromatin remodeling activity by Arabidopsis DDM1. Nat Commun. 2024;15:5187.

31. Lee SC, Adams DW, Ipsaro JJ, Cahn J, Lynn J, Kim H-S, et al. Chromatin remodeling of histone H3 variants by DDM1 underlies epigenetic inheritance of DNA methylation. Cell. 2023;186:4100–16.e15.

32. Mathieu O, Reinders J, Caikovski M, Smathajitt C, Paszkowski J. Transgenerational

stability of the Arabidopsis epigenome is coordinated by CG methylation. Cell. 2007;130:851–62.

33. Rigal M, Becker C, Pélissier T, Pogorelcnik R, Devos J, Ikeda Y, et al. Epigenome confrontation triggers immediate reprogramming of DNA methylation and transposon silencing in Arabidopsis thaliana F1 epihybrids. Proc Natl Acad Sci U S A. 2016;113:E2083–92.

34. Srikant T, Yuan W, Berendzen KW, Contreras-Garrido A, Drost H-G, Schwab R, et al. Canalization of genome-wide transcriptional activity in Arabidopsis thaliana accessions by MET1-dependent CG methylation. Genome Biol. 2022;23:263.

35. Marí-Ordóñez A, Marchais A, Etcheverry M, Martin A, Colot V, Voinnet O. Reconstructing de novo silencing of an active plant retrotransposon. Nat Genet. 2013;45:1029–39.

36. Fu Y, Kawabe A, Etcheverry M, Ito T, Toyoda A, Fujiyama A, et al. Mobilization of a plant transposon by expression of the transposon-encoded anti-silencing factor. EMBO J. 2013;32:2407–17.

37. He L, Huang H, Bradai M, Zhao C, You Y, Ma J, et al. DNA methylation-free Arabidopsis reveals crucial roles of DNA methylation in regulating gene expression and development. Nat Commun. 2022;13:1335.

38. McClintock B. The origin and behavior of mutable loci in maize. Proc Natl Acad Sci U S A. 1950;36:344–55.

39. Döring H-P, Starlinger P. Barbara McClintock's controlling elements: Now at the DNA level. Cell. 1984;39:253–9.

40. Baillie JK, Barnett MW, Upton KR, Gerhardt DJ, Richmond TA, De Sapio F, et al. Somatic retrotransposition alters the genetic landscape of the human brain. Nature. 2011;479:534–7.

41. Kazazian HH Jr. Mobile DNA transposition in somatic cells. BMC Biol. 2011;9:62.

42. Li W, Prazak L, Chatterjee N, Grüninger S, Krug L, Theodorou D, et al. Activation of transposable elements during aging and neuronal decline in Drosophila. Nat Neurosci. 2013;16:529–31.

43. Muotri AR, Chu VT, Marchetto MCN, Deng W, Moran JV, Gage FH. Somatic mosaicism in neuronal precursor cells mediated by L1 retrotransposition. Nature. 2005;435:903–10.

44. Gerdes P, Lim SM, Ewing AD, Larcombe MR, Chan D, Sanchez-Luque FJ, et al. Retrotransposon instability dominates the acquired mutation landscape of mouse induced pluripotent stem cells. Nat Commun. 2022;13:7470.

45. Faulkner GJ, Garcia-Perez JL. L1 mosaicism in mammals: Extent, effects, and evolution. Trends Genet. 2017;33:802–16.

46. Zhang Y, Guo Y, Jia H, Ma H, Tan S, Zhang YE. Jumping in the human brain: A review on somatic transposition. Fundamental Research [Internet]. 2025 [cited 2025 Apr 17]; Available from: http://dx.doi.org/10.1016/j.fmre.2025.03.001

47. Goerner-Potvin P, Bourque G. Computational tools to unmask transposable elements. Nat Rev Genet. 2018;19:688–704.

48. Treiber CD, Waddell S. Resolving the prevalence of somatic transposition in Drosophila. Elife [Internet]. 2017;6. Available from: http://dx.doi.org/10.7554/elife.28297

49. Smits N, Rasmussen J, Bodea GO, Amarilla AA, Gerdes P, Sanchez-Luque FJ, et al. No evidence of human genome integration of SARS-CoV-2 found by long-read DNA sequencing. Cell Rep. 2021;36:109530.

50. Richardson SR, Gerdes P, Gerhardt DJ, Sanchez-Luque FJ, Bodea G-O, Muñoz-Lopez M, et al. Heritable L1 retrotransposition in the mouse primordial germline and early embryo. Genome Res. 2017;27:1395–405.

51. Siudeja K, van den Beek M, Riddiford N, Boumard B, Wurmser A, Stefanutti M, et al. Unraveling the features of somatic transposition in the *Drosophila* intestine. EMBO J. 2021;40:e106388.

52. Rubanova N, Singh D, Barolle L, Chalvet F, Netter S, Poidevin M, et al. An endogenous retroviral element co-opts an upstream regulatory sequence to achieve somatic expression and mobility [Internet]. bioRxiv. 2025. p. 2025.01.02.631056. Available from: https://www.biorxiv.org/content/10.1101/2025.01.02.631056v1

53. D'Costa AV, Simpson JT. Somrit: The somatic retrotransposon insertion toolkit. bioRxivorg. 2023;

54. Jain C, Rhie A, Hansen NF, Koren S, Phillippy AM. Long-read mapping to repetitive reference sequences using Winnowmap2. Nat Methods. 2022;19:705–10.

55. Morrish TA, Gilbert N, Myers JS, Vincent BJ, Stamato TD, Taccioli GE, et al. DNA repair mediated by endonuclease-independent LINE-1 retrotransposition. Nat Genet. 2002;31:159–65.

56. Lin Y, Waldman AS. Capture of DNA sequences at double-strand breaks in mammalian chromosomes. Genetics. 2001;158:1665–74.

57. Moore JK, Haber JE. Capture of retrotransposon DNA at the sites of chromosomal double-strand breaks. Nature. 1996;383:644–6.

58. Quesneville H. Twenty years of transposable element analysis in the Arabidopsis thaliana genome. Mob DNA. 2020;11:28.

59. Gonzalez N, De Bodt S, Sulpice R, Jikumaru Y, Chae E, Dhondt S, et al. Increased leaf size: different means to an end. Plant Physiol. 2010;153:1261–79.

60. Ono Y, Hamada M, Asai K. PBSIM3: a simulator for all types of PacBio and ONT long reads. NAR Genom Bioinform. 2022;4:lqac092.

61. Ou S, Su W, Liao Y, Chougule K, Agda JRA, Hellinga AJ, et al. Author Correction: Benchmarking transposable element annotation methods for creation of a streamlined, comprehensive pipeline. Genome Biol. 2022;23:76.

62. Goubert C, Craig RJ, Bilat AF, Peona V, Vogan AA, Protasio AV. A beginner's guide to manual curation of transposable elements. Mob DNA. 2022;13:7.

63. Wicker T, Sabot F, Hua-Van A, Bennetzen JL, Capy P, Chalhoub B, et al. A unified classification system for eukaryotic transposable elements. Nat Rev Genet. 2007;8:973–82.

64. Wells JN, Feschotte C. A Field Guide to Eukaryotic Transposable Elements. Annu Rev

Genet. 2020;54:539–61.

65. Sierra P, Durbin R. Identification of transposable element families from pangenome polymorphisms. Mob DNA. 2024;15:13.

66. Gisby JS, Catoni M. The widespread nature of Pack-TYPE transposons reveals their importance for plant genome evolution. PLoS Genet. 2022;18:e1010078.

67. Sasaki T, Ro K, Caillieux E, Manabe R, Bohl-Viallefond G, Baduel P, et al. Fast co-evolution of anti-silencing systems shapes the invasiveness of Mu-like DNA transposons in eudicots. EMBO J. 2022;41:e110070.

68. Jiang N, Bao Z, Zhang X, Eddy SR, Wessler SR. Pack-MULE transposable elements mediate gene evolution in plants. Nature. 2004;431:569–73.

69. Jiang N, Ferguson AA, Slotkin RK, Lisch D. Pack-Mutator-like transposable elements (Pack-MULEs) induce directional modification of genes through biased insertion and DNA acquisition. Proc Natl Acad Sci U S A. 2011;108:1537–42.

70. Dubin MJ, Zhang P, Meng D, Remigereau M-S, Osborne EJ, Paolo Casale F, et al. DNA methylation in Arabidopsis has a genetic basis and shows evidence of local adaptation. Elife. 2015;4:e05255.

71. Sanchez DH, Gaubert H, Drost H-G, Zabet NR, Paszkowski J. High-frequency recombination between members of an LTR retrotransposon family during transposition bursts. Nat Commun. 2017;8:1283.

72. Zhang J, Peterson T. A segmental deletion series generated by sister-chromatid transposition of Ac transposable elements in maize. Genetics. 2005;171:333–44.

73. Zhang J, Zuo T, Wang D, Peterson T. Transposition-mediated DNA re-replication in maize. Elife. 2014;3:e03724.

74. Su W, Zuo T, Peterson T. Ectopic expression of a maize gene is induced by composite insertions generated through alternative transposition. Genetics. 2020;216:1039–49.

75. Sharma SP, Peterson T. Complex chromosomal rearrangements induced by transposons in maize. Genetics. 2023;223:iyac124.

76. Wang D, Yu C, Zuo T, Zhang J, Weber DF, Peterson T. Alternative transposition generates new chimeric genes and segmental duplications at the maize p1 locus. Genetics. 2015;201:925–35.

77. Zhang J, Peterson T. Transposition of reversed Ac element ends generates chromosome rearrangements in maize. Genetics. 2004;167:1929–37.

78. Gray YH. It takes two transposons to tango: transposable-element-mediated chromosomal rearrangements. Trends Genet. 2000;16:461–8.

79. Su W, Sharma SP, Peterson T. Evolutionary Impacts of Alternative Transposition. Origin and Evolution of Biodiversity. Cham: Springer International Publishing; 2018. p. 113–30.

80. Bowman JL, Sakakibara K, Furumizu C, Dierschke T. Evolution in the cycles of life. Annu Rev Genet. 2016;50:133–54.

81. Borges F, Parent J-S, van Ex F, Wolff P, Martínez G, Köhler C, et al. Transposon-derived small RNAs triggered by miR845 mediate genome dosage response in

Arabidopsis. Nat Genet. 2018;50:186–92.

82. Feng S, Cokus SJ, Schubert V, Zhai J, Pellegrini M, Jacobsen SE. Genome-wide Hi-C analyses in wild-type and mutants reveal high-resolution chromatin interactions in Arabidopsis. Mol Cell. 2014;55:694–707.

83. Zhong Z, Feng S, Duttke SH, Potok ME, Zhang Y, Gallego-Bartolomé J, et al. DNA methylation-linked chromatin accessibility affects genomic architecture in Arabidopsis. Proc Natl Acad Sci U S A. 2021;118:e2023347118.

84. Naish M, Alonge M, Wlodzimierz P, Tock AJ, Abramson BW, Schmücker A, et al. The genetic and epigenetic landscape of the Arabidopsis centromeres. Science. 2021;374:eabi7489.

85. Gómez-Zambrano Á, Merini W, Calonje M. The repressive role of Arabidopsis H2A.Z in transcriptional regulation depends on AtBMI1 activity. Nat Commun. 2019;10:2828.

86. Coleman-Derr D, Zilberman D. Deposition of histone variant H2A.Z within gene bodies regulates responsive genes. PLoS Genet. 2012;8:e1002988.

87. López-Cortegano E, Craig RJ, Chebib J, Balogun EJ, Keightley PD. Rates and spectra of de novo structural mutations in Chlamydomonas reinhardtii. Genome Res. 2023;33:45–60.

88. Fransz P, Linc G, Lee C-R, Aflitos SA, Lasky JR, Toomajian C, et al. Molecular, genetic and evolutionary analysis of a paracentric inversion in Arabidopsis thaliana. Plant J. 2016;88:159–78.

89. Zhang J, Yu C, Pulletikurti V, Lamb J, Danilova T, Weber DF, et al. Alternative Ac/Ds transposition induces major chromosomal rearrangements in maize. Genes Dev. 2009;23:755–65.

90. Liang W, Li J, Sun L, Liu Y, Lan Z, Qian W. Deciphering the synergistic and redundant roles of CG and non-CG DNA methylation in plant development and transposable element silencing. New Phytol. 2022;233:722–37.

91. Rabanal FA, Gräff M, Lanz C, Fritschi K, Llaca V, Lang M, et al. Pushing the limits of HiFi assemblies reveals centromere diversity between two Arabidopsis thaliana genomes. Nucleic Acids Res. 2022;50:12309–27.

92. Baid G, Cook DE, Shafin K, Yun T, Llinares-López F, Berthet Q, et al. DeepConsensus improves the accuracy of sequences with a gap-aware sequence transformer. Nat Biotechnol. 2023;41:232–8.

93. Cheng H, Concepcion GT, Feng X, Zhang H, Li H. Haplotype-resolved de novo assembly using phased assembly graphs with hifiasm. Nat Methods. 2021;18:170–5.

94. Alonge M, Lebeigle L, Kirsche M, Jenike K, Ou S, Aganezov S, et al. Automated assembly scaffolding using RagTag elevates a new tomato system for high-throughput genome editing. Genome Biol. 2022;23:258.

95. Mikheenko A, Saveliev V, Hirsch P, Gurevich A. WebQUAST: online evaluation of genome assemblies. Nucleic Acids Res. 2023;51:W601–6.

96. Manni M, Berkeley MR, Seppey M, Simão FA, Zdobnov EM. BUSCO update: Novel and streamlined workflows along with broader and deeper phylogenetic coverage for scoring of eukaryotic, prokaryotic, and viral genomes. Mol Biol Evol. 2021;38:4647–54.

97. Smit AFA, Hubley R, Green P. *RepeatMasker Open-4.0*. http://www.repeatmasker.org. 2013-2015.

98. Li H. Minimap2: pairwise alignment for nucleotide sequences. Bioinformatics. 2018;34:3094–100.

99. Shumate A, Salzberg SL. Liftoff: accurate mapping of gene annotations. Bioinformatics. 2021;37:1639–43.

100. Pertea G, Pertea M. GFF utilities: GffRead and GffCompare. F1000Res. 2020;9:304.

101. Ou S, Su W, Liao Y, Chougule K, Agda JRA, Hellinga AJ, et al. Benchmarking transposable element annotation methods for creation of a streamlined, comprehensive pipeline. Genome Biol. 2019;20:275.

102. Drost H-G, Sanchez DH. Becoming a selfish clan: Recombination associated to reverse-transcription in LTR retrotransposons. Genome Biol Evol. 2019;11:3382–92.

103. Neumann P, Novák P, Hoštáková N, Macas J. Systematic survey of plant LTR-retrotransposons elucidates phylogenetic relationships of their polyprotein domains and provides a reference for element classification. Mob DNA. 2019;10:1.

104. Zhang R-G, Li G-Y, Wang X-L, Dainat J, Wang Z-X, Ou S, et al. TEsorter: an accurate and fast method to classify LTR-retrotransposons in plant genomes. Hortic Res. 2022;9.

105. Qian J, Xue H, Ou S, Storer J, Fürtauer L, Wildermuth MC, et al. TEtrimmer: a novel tool to automate the manual curation of transposable elements. bioRxiv. 2024;2024.06.27.600963.

106. Shen W, Sipos B, Zhao L. SeqKit2: A Swiss army knife for sequence and alignment processing. Imeta. 2024;3:e191.

107. Katoh K, Standley DM. MAFFT multiple sequence alignment software version 7: improvements in performance and usability. Mol Biol Evol. 2013;30:772–80.

108. Minh BQ, Schmidt HA, Chernomor O, Schrempf D, Woodhams MD, von Haeseler A, et al. IQ-TREE 2: New models and efficient methods for phylogenetic inference in the genomic era. Mol Biol Evol. 2020;37:1530–4.

109. Jiang H, Lei R, Ding S-W, Zhu S. Skewer: a fast and accurate adapter trimmer for next-generation sequencing paired-end reads. BMC Bioinformatics. 2014;15:182.

110. Krueger F, Andrews SR. Bismark: a flexible aligner and methylation caller for Bisulfite-Seq applications. Bioinformatics. 2011;27:1571–2.

111. Langmead B, Salzberg SL. Fast gapped-read alignment with Bowtie 2. Nat Methods. 2012;9:357–9.

112. Zhang Y, Liu T, Meyer CA, Eeckhoute J, Johnson DS, Bernstein BE, et al. Model-based analysis of ChIP-Seq (MACS). Genome Biol. 2008;9:R137.

113. Lloyd JP, Seddon AE, Moghe GD, Simenc MC, Shiu S-H. Characteristics of plant essential genes allow for within- and between-species prediction of lethal mutant phenotypes. Plant Cell. 2015;27:2133–47.

114. Movilli A, Sushko S, Rabanal FA, Weigel D. PacBio HiFi data generated for Movilli et al., 2025. Eur Nucleotide Arch. https:/www.ebi.ac.uk/ena/browser/view/PRJEB85646

(2025).

115. Movilli A, Sushko S, Rabanal FA, Weigel D. Code repository for Movilli et al., 2025. GitHub. https://github.com/aerilli/Somatic-transposition_met1 (2025).

116. Movilli A, Sushko S, Rabanal FA, Weigel D. Code repository for Movilli et al., 2025. Zenodo. 10.5281/ZENODO.15824971, (2025).

117. Srikant T, Yuan W, Berendzen KW, Contreras-Garrido A, Drost H, Schwab R, et al. ATAC-Seq data generated for Srikant et al., 2022. Eur Nucleotide Arch. https:/www.ebi.ac.uk/ena/browser/view/PRJEB54034 (2022).

118. Srikant T, Yuan W, Berendzen KW, Contreras-Garrido A, Drost H, Schwab R, et al. RNA-Seq data generated for Srikant et al., 2022. Eur Nucleotide Arch. https:/www.ebi.ac.uk/ena/browser/view/PRJEB53354 (2022).

119. Srikant T, Yuan W, Berendzen KW, Contreras-Garrido A, Drost H, Schwab R, et al. Bisulfite-Seq data generated for Srikant et al., 2022. Eur Nucleotide Arch. https:/www.ebi.ac.uk/ena/browser/view/PRJEB54036 (2022).
